# Exploring Computational Techniques in Preprocessing Neonatal Physiological Signals for Detecting Adverse Outcomes: Scoping Review

**DOI:** 10.2196/46946

**Published:** 2024-08-20

**Authors:** Jessica Rahman, Aida Brankovic, Mark Tracy, Sankalp Khanna

**Affiliations:** 1 Commonwealth Scientific and Industrial Research Organisation (CSIRO) Australian e-Health Research Centre, Australia Sydney Australia; 2 Commonwealth Scientific and Industrial Research Organisation (CSIRO) Australian e-Health Research Centre, Australia Brisbane Australia; 3 Neonatal Intensive Care Unit, Westmead Sydney Australia

**Keywords:** physiological signals, preterm, neonatal intensive care unit, morbidity, signal processing, signal analysis, adverse outcomes, predictive and diagnostic models

## Abstract

**Background:**

Computational signal preprocessing is a prerequisite for developing data-driven predictive models for clinical decision support. Thus, identifying the best practices that adhere to clinical principles is critical to ensure transparency and reproducibility to drive clinical adoption. It further fosters reproducible, ethical, and reliable conduct of studies. This procedure is also crucial for setting up a software quality management system to ensure regulatory compliance in developing software as a medical device aimed at early preclinical detection of clinical deterioration.

**Objective:**

This scoping review focuses on the neonatal intensive care unit setting and summarizes the state-of-the-art computational methods used for preprocessing neonatal clinical physiological signals; these signals are used for the development of machine learning models to predict the risk of adverse outcomes.

**Methods:**

Five databases (PubMed, Web of Science, Scopus, IEEE, and ACM Digital Library) were searched using a combination of keywords and MeSH (Medical Subject Headings) terms. A total of 3585 papers from 2013 to January 2023 were identified based on the defined search terms and inclusion criteria. After removing duplicates, 2994 (83.51%) papers were screened by title and abstract, and 81 (0.03%) were selected for full-text review. Of these, 52 (64%) were eligible for inclusion in the detailed analysis.

**Results:**

Of the 52 articles reviewed, 24 (46%) studies focused on diagnostic models, while the remainder (n=28, 54%) focused on prognostic models. The analysis conducted in these studies involved various physiological signals, with electrocardiograms being the most prevalent. Different programming languages were used, with MATLAB and Python being notable. The monitoring and capturing of physiological data used diverse systems, impacting data quality and introducing study heterogeneity. Outcomes of interest included sepsis, apnea, bradycardia, mortality, necrotizing enterocolitis, and hypoxic-ischemic encephalopathy, with some studies analyzing combinations of adverse outcomes. We found a partial or complete lack of transparency in reporting the setting and the methods used for signal preprocessing. This includes reporting methods to handle missing data, segment size for considered analysis, and details regarding the modification of the state-of-the-art methods for physiological signal processing to align with the clinical principles for neonates. Only 7 (13%) of the 52 reviewed studies reported all the recommended preprocessing steps, which could have impacts on the downstream analysis.

**Conclusions:**

The review found heterogeneity in the techniques used and inconsistent reporting of parameters and procedures used for preprocessing neonatal physiological signals, which is necessary to confirm adherence to clinical and software quality management system practices, usefulness, and choice of best practices. Enhancing transparency in reporting and standardizing procedures will boost study interpretation and reproducibility and expedite clinical adoption, instilling confidence in the research findings and streamlining the translation of research outcomes into clinical practice, ultimately contributing to the advancement of neonatal care and patient outcomes.

## Introduction

### Background

Premature infants are those born at <37 weeks gestational age, ranging from extreme preterm (23 weeks’ gestation) to late preterm (37 weeks’ gestation), and are defined as having very low birth weight of <1500 g. These extremely premature infants have a higher risk of death, and surviving infants are highly prone to physical, cognitive, and emotional impairment [1]. The patients usually have a long length of stay, ranging from <10 to >120 days [2], in the neonatal intensive care unit (NICU), where high-fidelity physiological changes are monitored to observe their health status and signs of deterioration. During this long length of stay, a large amount of data from infants are generated and not typically electronically aggregated for permanent storage [3]. With the advent of electronic health records, relevant patient information is easily available for advanced data analytics that can be used to improve health outcomes. The records contain demographics, etiology, pathology, medication, and physiology information. Physiological changes are regularly monitored in preterm infants, notably, electrocardiogram (ECG), oxygen saturation (SpO_2_), heart rate (HR), respiratory rate, arterial blood pressure, electroencephalography (EEG), and temperature. Some advanced centers around the world have started linking the information derived from the electronic health records data with the continuously monitored physiological information for permanent storage, more frequently in lower resolution, which facilitates various data analytics [4-6]. Compared with intermittent assessment and review, continuous capturing and analysis of the physiological data from the standard bedside monitors allow for a better understanding of trends and have been shown to improve outcomes of infants in the NICU [5].

Clinical decision support systems (CDSSs) can integrate clinical and physiological information to provide automated support in patient care planning to facilitate the diagnostic process and therapy planning, generate critical alerts and reminders, and predict the risk of patient deterioration. CDSSs have the potential for a positive impact in improving clinical and economic measures in the health care system [7-9]. The technological advancement that allowed storing big data, as well as the advancement of artificial intelligence (AI), has given rise to machine learning (ML)– and AI-based CDSSs aiming to build data-driven models to predict adverse outcomes in premature infants ahead of clinical diagnosis time [10-12].

The steps of building the ML pipeline to predict adverse outcomes involve several intermediate computational steps using the physiological data, of which data preprocessing is the first indispensable step. Namely, in the NICU, physiological signals are collected using a diverse range of devices, which introduce a number of artifacts such as *environmental artifacts* (eg, device connection failure, equipment noise, electrosurgical noise, and power line interferences); *experimental or human error* due to patient movement during data acquisition, incorrect or poor contact of the electrodes, and other contact noise; and *artifacts* due to muscle contraction, cardiac signals, and blinking [13,14]. These noises distort signals and may adversely affect model generalization capability and predictive power [10].

Although recently much progress has been made in building ML models using neonatal physiological data, there are limitations in the detailed reporting of the preprocessing techniques of these signals [15], which in turn hinder the reproducibility of the methods and results. In AI-powered software as a medical device (SaMD), this is especially important as the implementation of a software quality management system (QMS) is only possible by following the best practices and adhering to relevant regulatory standards and guidelines for medical devices, such as ISO 13485, IEC 62304, and IEC 82304-1. Beyond market access considerations, the ongoing international discourse on the regulation of medical software is specifically concentrated on AI and ML. This focus is a response to their growing applications, demanding increased attention from regulatory bodies such as the Australian Therapeutic Goods Administration and the US Food and Drug Administration [16]. Thus, it is crucial to adhere to a standardized protocol following clinical principles guided by domain experts and regulatory requirements while preprocessing the signals and reporting these techniques in detail; this ensures the reproducibility of the methods, allowing transparency in their clinical adoption.

### Objectives

As the first step in bridging the gap in their reproducibility for clinical adoption, this review aims to identify studies that used computational methods to analyze premature infants’ physiological signals for detecting adverse outcomes. The review describes different tools and techniques used to preprocess physiological signals and provides recommendations on what aspects need further details for the clinical adoption of the techniques. The remainder of the paper is organized as follows: the Methods section explains the detailed search and screening process, while the Results section begins with an overview of the reviewed studies, followed by a detailed analysis. The Discussion section highlights the key reporting patterns identified in this review along with their shortcomings and provides recommendations for transparent reporting of future studies as it allows for accurate reproduction of the results and makes them usable in the clinical setting [17]. A summary of the work concludes the paper.

## Methods

### Search Strategy

The database searches and study screening were conducted following the recommendations of PRISMA (Preferred Reporting Items for Systematic Reviews and Meta-Analyses) guidelines [18] and the Centre for Reviews and Dissemination guidance for undertaking reviews in health care [19].

### Database and Search Strategies

A systematic database search was conducted on 5 databases: PubMed, IEEE, Web of Science, Scopus, and ACM Digital Library. The keywords were categorized into four concepts, which were then merged using the “AND” operator: concept 1—neonates or preterm infants; concept 2—vital signs or physiological signals; concept 3—computational techniques or signal processing; and concept 4—outcomes relating to neonates. Within each of these concepts, a combination of keywords and MeSH (Medical Subject Headings) terms were used to conduct the search process. The keywords under each concept were combined by the “OR” operator. The searches were limited to only the titles and abstracts. Table 1 shows the list of keywords and Medical Subject Headings terms used to search the database.

The search was done on January 9, 2023, and the publication year of the papers was limited to 2013 to 2023. The reason for choosing the 10-year range was to report on recent techniques and tools, as the devices and computational tools used >10 years ago may be obsolete. Scopus, Wiley Online Library, and Web of Science have an additional filter for choosing the subject area. This was used to restrict the subject areas to multidisciplinary, engineering, computing, and statistics. This was done to identify more papers on multidisciplinary areas through these databases, as PubMed covers all the major medical and health informatics databases. The combination of the 5 databases ensured that all medical, information technology, and multidisciplinary research papers were included in the database search. The search was restricted to English-language articles. Finally, review articles were excluded from the search.

**Table 1:** List of keywords and MeSH (Medical Subject Headings) terms used to conduct the database search.

Concepts			Search strategy
**Concept 1: neonates or preterm babies**			
	MeSH terms	“Infant, Premature”	
	Keywords	“premature” OR “preterm” OR “neonat*” OR “newborn” OR “infant” OR “nicu” OR “neonatal intensive care unit”	
**Concept 2: physiological signals or vital signs**			
	MeSH terms	“Vital Signs” OR “Physiology”	
	Keywords	“physiolog*” OR “ecg” OR “heart rate” OR “electrocardiography” OR “vital sign*” OR “physiomarker” OR “biomarker” OR “hrv”	
**Concept 3: computational techniques or signal processing**			
	MeSH terms	“Signal Processing, Computer-Assisted”	
	Keywords	“signal *” OR “predict*” OR “detect*” OR “comput*”	
**Concept 4: outcomes**			
	MeSH terms	None	
	Keywords	“sepsis” OR “mortality” OR “length of stay” OR “intraventricular hemorrhage” OR “hypoxi*” OR “apnea” OR “necrotising entercolitis” OR “necrotizing entercolitis”	

### Screening and Study Selection

The initial screening of the databases led to 3585 papers. Of these, 590 (16.46%) papers were manually identified as duplicates and excluded from the analysis. One paper was identified as a duplicate by the automation tool and removed. The remaining 2994 (83.51%) papers were subjected to title and abstract screening using the Rayyan Intelligent Systematic Review application (Qatar Computing Research Institute) [20].

Several inclusion criteria were set to select papers for full-text review. The criteria are mentioned in Textbox 1.

After screening the titles and abstracts, 81 articles were selected for full-text review; 29 (36%) papers were excluded during this stage as they did not align with the inclusion criteria, leaving 52 (64%) papers eligible for detailed synthesis and analysis. The title and abstract screening was done by 1 reviewer, while 2 reviewers independently checked for paper eligibility against the inclusion criteria at the full-text review stage. When both reviewers were not in agreement on any papers, a third reviewer assessed them to provide a final decision on the inclusion and exclusion of the papers. Data charting was done using Microsoft Excel, and the following variables were recorded in line with related review papers [10,21]: title, year, journal, authors, digital object identifier, data set, participant number, participant demographic, signals used, data set size, sample rate, other data (if applicable), outcome metric, device software, programming language, preprocessing methods, algorithms, other techniques, features, models, model type, results (quantified), and key findings. Data synthesis was done using a narrative approach by summarizing findings based on the similarities in the data sets and techniques used. The detailed search queries, bibliography files of all databases, all included papers, metadata of all papers and metadata of all papers included for full-text review are provided in Multimedia Appendices 1-5 [22-73].

**Inclusion and exclusion criteria.**

**Inclusion criteria**

- Article type: articles must be peer-reviewed publications in a journal, conference, or workshop
- Data: articles must conduct an analysis on premature human infant data; articles must use physiological responses in some form
- Outcome: articles discuss applications relating to adverse neonatal outcomes such as mortality, length of stay, sepsis, necrotizing enterocolitis, intraventricular hemorrhage, hypoxic-ischemic encephalopathy, apnea, bradycardia, and other poor health outcomes, also known as morbidity. The disease outcomes were chosen based on the commonly researched outcome metric using preterm infant data and the search terms used in McAdams et al [10] that investigated artificial intelligence and machine learning techniques used to predict clinical outcomes in the neonatal intensive care unit
- Analysis: articles reported some form of computational techniques in their analysis
- Language: English

**Exclusion criteria**

- Article type: review papers are excluded
- Data: nonhuman data (eg, piglet infant data would not be considered); videos and images that do not look at the physiological responses and articles solely using demographic data for analysis were excluded
- Outcome: articles not focusing on these specified neonatal adverse outcomes were excluded
- Analysis: articles that only reported responses in their raw format were excluded
- Language: any languages other than English

## Results

### Overview of the Included Studies

**Figure 1:**
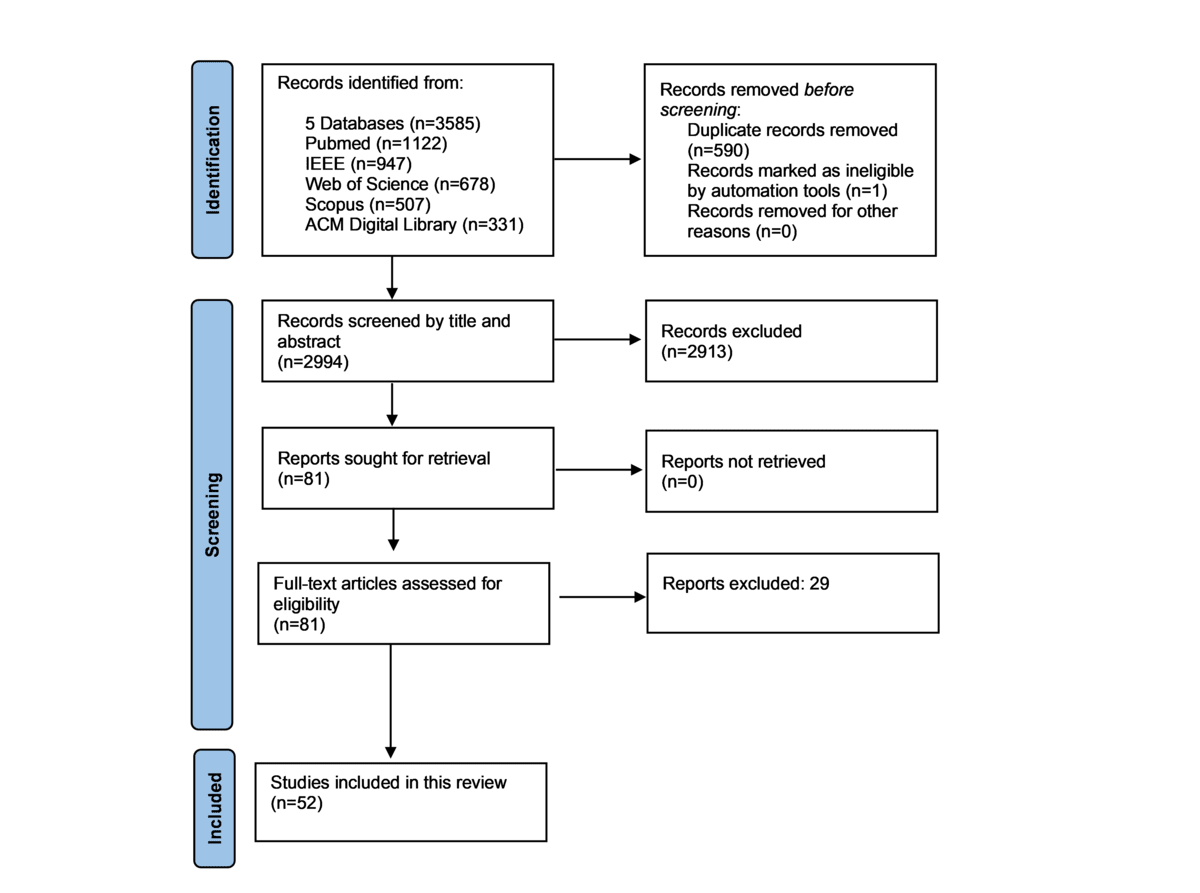
PRISMA (Preferred Reporting Items for Systematic Reviews and Meta-Analyses) flow diagram for the database search and study selection.

Figure 1 shows the full process of database search and study selection using a PRISMA flow diagram.

Of the 52 selected articles, 24 (46%) studies focused on diagnostic models, while the rest (n=28, 54%) focused on prognostic models. These included journal articles (n=34, 65%), conference articles (n=17, 33%) and a workshop article (n=1, 2%). The most prominent physiological signals analyzed were ECG (n=36, 69%), SpO_2_ (n=21, 40%), HR (n=16, 31%), respiration (n=16, 31%), BP (n=6, 12%), EEG (n=4, 8%), and temperature (n=3, 6%). While 8 (15%) studies used a combination of programming languages; others used MATLAB (n=6, 12%), Python (n=6, 12%), and R software (n=1, 2%), while the remaining studies (n=31, 60%) did not report what language was used. Physiological data monitoring and capturing was done using a range of systems, which subsequently impacted the sampling rate and quality of the data, thus leading to heterogeneity of the studies. The most commonly used devices for data capturing were Phillips Intellivue MP20, MP70, MP450, and MX800 machines [74] (n=14, 27%). Some other notable devices and software were BedMaster Ex System [75], NicoletOne EEG system [76], ixTrend, Phillips Data Warehouse connect [77], and Vuelogger patient monitoring system. The most commonly analyzed outcomes of interest were sepsis (n=20, 38%), apnea (n=17, 33%), bradycardia (n=13, 25%), mortality (n=7, 13%), and hypoxic-ischemic encephalopathy (n=5, 10%). It should be noted that 14 (27%) of the reviewed studies analyzed a combination of adverse outcomes.

As the studies were found to be heterogeneous in their study design and analysis techniques, a narrative approach was taken to summarize the studies and their key findings. The studies were grouped according to the homogeneity in terms of the data sets used and sorted by the publication year. This approach was inspired by the review article by Mann et al [78].

One of the noticeable patterns identified through the results reported in Table 2 is that the groups publishing studies using the same data set followed similar preprocessing techniques, although not at every step. For instance, studies using the ECG data from Cork University Maternity Hospital all used the same algorithm for QRS complex detection. However, they were diverse in their selection of filtering techniques and segmentation duration. Furthermore, they systematically failed to report detailed parameter settings for the QRS complex detection. While the approach of using similar preprocessing techniques helps maintain consistency to some extent, they do not confirm adhering to clinical practices identified from domain expert knowledge.

The QRS complex characteristics and RR intervals for neonates are different from those of adults and as such require an appropriate adjustment for QRS detection algorithms. This is a necessary first step for HR variability (HRV) analysis in neonates. However, a review published on neonatal HRV by Latremouille et al [15] revealed that given a lack of clear guidelines on neonatal vital signs and HRV analysis, several studies followed HRV analysis guidelines for adults published by the Task Force of the European Society of Cardiology and the North American Society of Pacing and Electrophysiology [79]. Our review found that 16 (44%) out of the 36 studies analyzing ECG signals used the Pan-Tompkins algorithms for QRS complex detection. The original implementation of the algorithm was based on the ECG characteristics of the adult population and therefore was preprocessed accordingly. Only 4 (25%) of those 16 studies reported adjustment of the original algorithm to adapt to neonates, of which only 2 provided specific modification details. In the absence of detailed reporting on the parameter settings, it is difficult to determine whether the settings adhered to neonatal waveform morphology. Incomplete reporting and lack of transparency hinder the understanding of the strengths and weaknesses of a study and limit its reproducibility and usability. Moreover, transparent and detailed reporting is required to confirm the adherence to regulatory compliance and is crucial for the clinical adoption of these methods.

Similar to the QRS complex in ECG signals, the acceptable ranges of physiological signals for neonates are also different from those of the adult population. This review found that no studies reviewed the acceptable ranges of the analyzed signals against any published guidelines, which could pose several limitations in the clinical adoption of the methods. This is consistent with another review looking into physiological vital sign ranges from 34 weeks gestational age, and it identified that several studies reported the means of vital signs instead of ranges, which makes the interpretation into clinical practice difficult [80]. Here, we recommend clear reporting and the use of physiological signal ranges that are clinically validated through published studies and textbooks [81-83].

**Table 2:** Summary of the articles reviewed in this study, grouped according to the homogeneity in terms of the data sets used and sorted by the publication year.

Data set used	Author, year	Study settings	Physiological signal analyzed	Signal processing and computational techniques	Key findings
National CHIME^a^ database [84]	Cohen and de Chazal [22], 2013	Participants: n=288; data size: NR^b^; model: diagnostic; outcome metric: sleep apnea	ECG^c^ from single channel at 100 Hz, SpO_2_^d^ at 1 Hz	SpO_2_ values <65% and changes in saturation exceeding 4% per second were discarded. ECG QRS complex was detected using the Pan-Tompkins algorithm [85] to generate RR intervals. QRS complexes were filtered using a technique from Chazal et al [86]. Filtered intervals were time aligned with SpO_2_ using 30-second epochs	Eleven features were extracted from the signals. A combination of features from both signals resulted in 88.8% accuracy, 94.3% specificity, and 73.4% sensitivity in detecting sleep apnea
CHIME	Cohen and de Chazal [23], 2014	Participants; n=402; data size: NR; model: diagnostic; outcome metric: sleep apnea	ECG from single channel at 100 Hz, SpO_2_ at 1 Hz, actigraphy signals at 50 Hz	Actigraphy signals artifact rejection was done using the technique described by Lewicke et al [87]. SpO_2_ values <65% and changes in saturation exceeding 4% per second were discarded. ECG data were passed through a QRS detection algorithm (NR) to produce RR intervals, which were filtered using a previously outlined method [86]	Fourteen features were extracted from the signals. A linear discriminant classifier achieved an accuracy of 74.1%, a sensitivity of 82.0%, and a specificity of 60.9% in detecting sleep apnea
CHIME	Cohen and de Chazal [24], 2015	Participants; n=394; data size: NR; model: diagnostic; outcome metric: sleep apnea	ECG from single channel at 100 Hz, SpO_2_ at 1 Hz	SpO_2_ and ECG signals were time aligned to 30-second epochs. SpO_2_ values <65% and changes in saturation exceeding 4% per second were discarded. ECG QRS complex detected using the Pan-Tompkins algorithm [85] to generate RR intervals. QRS complexes were filtered using a technique from Chazal et al [86]	Eleven features were extracted from both signals. A linear discriminant model achieved 66.7% accuracy, 67% specificity, and 58.1% sensitivity using features from both signals
PICS^e^ database [25,88]	Gee et al [26], 2016	Participants; n=10; data size: ~20-70 hours each; model: diagnostic; outcome metric: bradycardia	3-lead ECG at 500 Hz, respiration signal at 50 Hz	RR intervals from ECG were extracted using a modified Pan-Tompkins algorithm (modification details NR). Analysis was done on a 3-minute window before each bradycardia. No processing was reported for respiration signals	Bradycardia severity estimation accuracy was improved by an average of 11% using a point process model of heart rate and respiration
PICS	Gee et al [25], 2017	Participants, 10; data size: ~20-70 hours each; model: prognostic (+116 seconds); outcome metric: bradycardia	3-lead ECG at 500 Hz, respiration signal at 50 Hz	RR intervals from ECG were extracted using a modified Pan-Tompkins algorithm (modification details NR). The artifacts, due to movement, disconnection, or erroneous peaks, were removed by visual inspection. No processing was reported for respiration signals. Additional analysis on the frequency content of the RR time series was done using Morlet wavelet transform [89]	A point process model–based prediction algorithm achieved a mean AUROC^f^ of 0.79 for >440 bradycardic events and was able to predict bradycardic events on an average of 116 seconds before onset (FPR^g^=0.15)
PICS	Das et al [27], 2019	Participants; n=10; data size: ~20-70 hours each; model: prognostic (time NR); outcome metric: bradycardia	3-lead ECG at 500 Hz	Baseline wander was removed using a high-pass filter with a cutoff frequency between 0.5 and 0.6 Hz. Motion and disconnection artifacts were removed by visual inspection. QRS complexes were detected using Pan-Tompkins algorithm [85]. Signals were segmented 5 minutes before and 2 minutes after a bradycardic event	Nonparametric modeling using kernel density estimation achieved a 5% false alarm rate in predicting the onset of bradycardia events
PICS	Mahmud et al [28], 2019	Participants; n=11; data size: ~20-70 hours each for 10 and 10 weeks for 1 participant; model: prognostic (time NR); outcome metric: bradycardia	3-lead ECG at 500 Hz	QRS complex was detected using an algorithm (NR). RR intervals were calculated from the detected peaks	Time and frequency domain features were extracted. An extreme gradient boosting model achieved an average AUROC of 0.867. HRV^h^ results showed a significant variation between a healthy infant and an infant prone to bradycardia
PICS	Gee et al [29], 2019	Participant, n=10; data size: ~20-70 hours each; model: diagnostic; outcome metric: bradycardia, apnea of prematurity	3-lead ECG at 500 Hz, respiration signal at 50 Hz	Respiration signals were clipped into 60-second segments and normalized to 0-mean, unit variance. RR intervals from ECG signals were extracted using a Morlet wavelet transformation. An open-source peak finder (name NR) was applied to the wavelet scale ranging from 0.01 to 0.04, which is related to the QRS complex formation in the spectrogram. ECG signals were segmented to 15 seconds with the event in the middle. The segments were bandpassed filtered from 3 to 45 Hz, scaled to 0-mean unit variance, and scaled to the median QRS complex amplitude. Waveforms were visually inspected to remove segments with no distinguishable QRS complex or respiratory peaks	An autoencoder-prototype model was proposed, which achieves 93.1% (SD 0.4%) accuracy in predicting bradycardia and 82.3% (SD 3.8%) accuracy in classifying apnea
MIMIC-III^i^ database from Beth Israel Deaconess Medical Center [90]	Song et al [30], 2020	Participants: 2819 (21 sepsis, 2798 control); data size: NR; model: prognostic (+48 hours); outcome metric: sepsis	HR^j^, SBP^k^, DBP^l^, MBP^m^, SpO_2_, respiration, temperature, other (sampling rate NR)	Data quality was assessed by missing value filter and 3-sigma rule. The final observation carried forward was applied to vital signs not meeting data quality. Zero imputation was performed if calculation could not be performed (eg, divided by 0)	Several statistical features were extracted at 3-, 6-, 12-, and 24-hour window. Linear model, naive Bayes, decision tree, ensemble method, and neural network models were evaluated. The AUROC of the 48-hour prediction model achieved 0.861 and that of the onset detection model was 0.868
MIMIC-III	Baker et al [31], 2021	Participants; n=179 for 3-day and n=181 for 14-day model; data size, NR; model: prognostic (+3 days); outcome metric: mortality	HR, respiration signal, sampled hourly	Values <0 and flatline cases were eliminated	Several statistical features were extracted from the signals. CNN-LSTM^n^ model using a 3-day scheme achieved AUROC of 0.9336 (SD 0.0337) across 5-fold cross-validation
MIMIC-III	Juraev et al [32], 2022	Participants; n=3133; data size: 24 hours from each; model: prognostic (time NR); outcome metric: mortality and LOS^o^	HR, respiration signal, SpO_2_, BP, temperature (sampling rate NR)	Missing data were filled by forward and backward filling, using the mean value. For participants >24 measurements, they were reduced by taking the average of the nearest records. For <24 measurements, values were generated using filling algorithm	A dynamic ensemble KNN^p^ method reached 0.988 (SD 0.001) *F*_1_-score in mortality classification. Voting of static ensemble regression models achieved an RMSE^q^ of 12.509 (SD 0.079) in LOS prediction
University Hospitals in France	Ghahjaverestan et al [33], 2015	Participants; n=32; data size: 105 segments of ECG with 250 seconds duration; model: diagnostic; outcome metric: apnea- bradycardia	One lead ECG at 400 Hz and respiration signals (sampling rate NR)	Baseline and noise of 50 Hz were removed from ECG signals, QRS complexes were detected using Pan-Tompkins algorithm [85]. RR intervals were further downsampled to 10 Hz for 1 prediction model	A Kalman-filter–based method achieved sensitivity and specificity of 94.74% and 94.17%, respectively, in predicting apnea-bradycardia episodes
University Hospitals in France	Navarro et al [34], 2015	Participants; n=51; data size: testing cohort mean duration—2.4 hours; model: diagnostic; outcome metric: sepsis	Respiration signals at 400 Hz, downsampled to 64 Hz	Frequency content >32 Hz from breathing signals was removed using a seventh-order Butterworth low-pass filter. After rejecting artifacts due to gross movements, a fourth-order Butterworth filter with a cutoff frequency between 0.5 and 20 Hz was applied. Smoothing filtering using an SG^r^ filter [91] was applied. A simple extrema detector is then applied to detect respiratory cycles	14 features, computed in 10-second sliding excerpts, were extracted from the breathing signals. A logistic regression classifier automatically rejects artifacts to 86% sensitivity and specificity, which is used in the proposed framework for neonatal sepsis detection
University Hospitals in France	Ghahjaverestan et al [35], 2016	Participants; n=32; data size: real (236 segments Synthetic) 200 sequences of 400 seconds; model: diagnostic (0.59-second delay); outcome metric: apnea-bradycardia	ECG at 400 Hz. Synthetic signals at 10 Hz	Baseline and noise of 50 Hz were removed from ECG signals using a combination of low-pass and notch filters; QRS complexes were detected using Pan-Tompkins algorithm [85]. Three features were extracted using a wavelet-based beat delineator [92]. Features were transformed to 10 Hz using interpolation (technique NR)	A CHMM^s^ achieved 95.74% sensitivity and 91.88% specificity in detecting apnea-bradycardia episodes, with a detection delay of –0.59 seconds
University Hospitals in France	León et al [36], 2021	Participant, n=49; data size: NR; model: prognostic (+6 hours); outcome metric: sepsis	ECG at 500 Hz	RR intervals were detected using modified Pan-Tompkins algorithms, and filter coefficients were adapted for newborns [93]. A sliding window of 30 minutes, with no overlaps, was applied to extract HRV parameters from the RR time series. 30-minute segments with a maximum RR >1 second or a minimum RR of <0.19 seconds were excluded,	Time, frequency, and nonlinear features were extracted from the HRV parameters. A logistic regression model using visibility graph features achieved 0.877 AUROC in predicting sepsis 6 hours before the start of antibiotics
University Hospitals in France	León et al [37], 2021	Participants; n=259; data size: NR; model: prognostic (+6 hours); outcome metric: sepsis	ECG at 500 Hz	RR intervals were detected using modified Pan-Tompkins algorithms and filter coefficients were adapted for neonates [93]. RR time series were extracted and segmented into 5-minute segments. The 5-minute periods corresponding to 30 continuous minutes were grouped by calculating the median of each corresponding HRV feature	Time, frequency, nonlinear, and visibility graph features were extracted from the HRV parameters. An RNN^t^ model achieved 0.904 AUROC in predicting sepsis 6 hours before the time of infection and >80% accuracy 24 hours before the onset of infection
University Hospitals in France	Doyen et al [38], 2021	Participants; n=52; data size: 8 hours of recording from each; model: diagnostic (+2.9-second delay); outcome metric: bradycardia	3-lead ECG at 300 Hz	QRS complexes were detected using a multifeature probabilistic real-time detector [93]	A high rate of false alarms (64%) was observed in real life. The proposed optimal decentralized fusion of 3 detection methods had a significant detection delay of 2.9 seconds, sensitivity of 97.6% and false alarm rate of 63.7%
University Hospitals in France	Sadoughi et al [39], 2021	Participants; n=32; data size: 233 episodes with a duration of 21.48 (SD 16.07) seconds; model: diagnostic (+5.05-second delay); outcome metric: apnea-bradycardia	One lead ECG at 400 Hz	The same preprocessing techniques as reported in Ghahjaverestan et al [35]. QRS complexes were identified using Pan-Tompkins method [85]. The RR time series were uniformly upsampled to 10 Hz using a linear interpolation technique	A proposed layered HMM^u^ model achieved 97.14% (SD 0.31%) accuracy in detecting apnea-bradycardia episodes, with a detection delay of –5.04 (SD 0.41) seconds
Cork University Maternity Hospital	Ahmed et al [40], 2015	Participants: NR; data size: 54 1-hour recordings; model: diagnostic; outcome metric: HIE	2-lead ECG, EEG^v^ (sampling rate NR)	Artifacts were manually removed. R-peaks from raw ECG signals were extracted using Pan-Tompkins method [85]. The timing of the peaks was adjusted and uniformly sampled to 256 Hz using Hermite spline quadratic interpolation. Then, HRV features were extracted from a 1-minute window with 30-second overlap using the normalized RR interval	Seven time and frequency domain HRV features were extracted. A Gaussian supervector approach with SVM^w^ achieved 0.81 AUROC in classifying HIE^x^
Cork University Maternity Hospital	Temko et al [41], 2015	Participant, n=38; data size: 1 hour of EEG and ECG recordings from each; model: diagnostic; outcome metric: HIE	ECG and video EEG at 256 Hz	The 1-hour EEG segments were downsampled to 32 Hz with an antialiasing filter set to 16 Hz. The filtered EEG was segmented into a 60-second epoch with no overlap. QRS complexes from ECG signals were extracted using the algorithm reported in [94]. The resulting peaks were manually inspected to correct ectopic beats or mark artifacts. Then, signals were segmented into 60-second epochs	An SVM classifier using a subset of 9 EEG, 2 hours, and 1 clinical feature achieved 87% AUROC and 84% accuracy in predicting HIE
Cork University Maternity Hospital	Lloyd et al [42], 2016	Participant, n=43; data size: mean recording duration 41 hours 40 minutes; model: diagnostic; outcome metric: future adverse outcome in infants	EEG at 256 Hz, SpO_2_ and HR at 1 Hz	EEG recordings were visually checked for quality, and poor-quality data were discarded. 1-hour epochs of EEG at 12 and 2 hours of age were then extracted from each recording. 1-hour epochs of HR and SpO_2_ were extracted at 12- and 24-hour time point.	A logistic regression model predicted a 2-year poor outcome with an AUROC of 0.83
Cork University Maternity Hospital	Semenova et al [43], 2018	Participants; n=35 with 23 used; data size: 824 hours; prognostic (time NR); outcome metric: short-term adverse outcome	ECG at 256 or 1024 Hz, BP at 1 Hz	Diastolic and systolic pressures every second were used to calculate MAP^y^. ECG signals were segmented to nonoverlapping 5-minute epochs. QRS complexes were extracted by the Pan-Tompkins method [85]. Abnormal RR intervals were corrected by moving average. Periods of clear movement of artifacts were automatically discarded (method NR)	Fifteen time, frequency, and nonlinear features were extracted from HRV. An XGBoost decision tree using all features achieved an AUROC of 0.97 in predicting short-term outcomes in infants
Cork University Maternity Hospital	Semenova et al [44], 2019	Participants; n=43 with 23 used; data size: total 831 hours; prognostic (time NR); outcome metric: 5 adverse outcomes	ECG at 256 or 1024 Hz, BP at 1 Hz	DBP and SBP every second were used to calculate MAP. Segments with MAP<10 mm Hg were discarded due to disconnection of the pressure transducer or movements. The MAP was segmented into 1-hour windows. Values outside 3 SD were discarded. ECG signals were segmented into nonoverlapping 5-minute epochs. QRS complexes were extracted using the Pan-Tompkins method [85]. ECG signal was bandpass filtered with 4-30-Hz cutoff frequency. Abnormal values of RR intervals were corrected by the moving average filter	Time, frequency, and nonlinear features were extracted from HRV. An XGBoost decision tree using a single HRV feature achieved 0.87 AUROC, while multiple features reached 0.97 AUROC in predicting adverse outcomes
Máxima Medical Center NICU^z^	Joshi et al [45], 2020	Participants; n=49; data size: ~144 hours each; model: prognostic (+0-24 h); outcome metric: sepsis	ECG at 250 Hz, CI^aa^ at 62.5 Hz	Respiration waveforms were bandpass filtered between 0.45 and 1.45 Hz. QRS complexes from ECG were extracted using a DT-CWT^ab^–based method described in [95]. IBIs^ac^ were detected from the CI signal peaks using an algorithm (NR). Features were extracted from every 3-hour data	Twenty-two features were extracted from the signals. A naive bayes classifier reached up to 0.78 AUROC and 3 hours leading up to sepsis
Máxima medical Center NICU	Varisco et al [46], 2021	Participants; n=20; data size: ~570 hours; model: prognostic (+6 hours); outcome metric: central apnea preceding late-onset sepsis	ECG at 240 or 250 Hz, CI at 60 or 62.5 Hz, SpO_2_ at 0.5 or 1 Hz	A filtered respiration signal without cardiac artifacts was generated using algorithms reported in studies by Lee et al [96], Mohr et al [97], and Vergales et al [98]. Steps include Fourier transformation and integer frequencies filtered out, then resampled to 60 Hz and high-pass filtered with a cutoff frequency of 0.4 Hz, and a low-pass filter with a very low cutoff frequency optimized to fit apnea annotations by clinical experts (value NR)	An optimization of the algorithm was proposed to detect central apnea, which achieved 90.5% recall, 19.7% precision, and 30.8% *F*_1_-score
Máxima medical Center NICU	Cabrera-Quiros et al [47], 2021	Participants; n=64; data size: NR; model: prognostic (+3 hours); outcome metric: sepsis	ECG at 250 Hz, CI at 62.5 Hz	QRS complexes from ECG were extracted using a DT-CWT–based method described the same as Joshi et al [45]. CI signal was filtered to remove cardiac artifacts, and peaks were detected using methods similar to those in previous works (NR). Features were extracted from every 1-hour signal	Time domain features were extracted from HRV. Classification using a combination of all features and logistic regression model reached a mean accuracy of 0.79 (SD 0.12) and mean precision of 0.82 (SD 0.18), 3 hours before the onset of sepsis
Máxima Medical Center NICU	Varisco et al [48], 2022	Participants; n=20; data size: 960 hours of data from 20 infants, 7818 event extracted; model: diagnostic; outcome metric: central apnea	ECG at 250 Hz, CI at 62.5 Hz, SpO_2_ at 1 Hz	QRS complexes were detected using the same method as reported in Joshi et al [45] and Cabrera-Quiros et al [47]. From ECG, SII^ad^ was calculated by applying a bandpass filter (0.001-0.40 Hz) using 10-second segments and then computing a kernel density estimate to return patient motion measurement every second. RR intervals were resampled at 250 Hz. CI signal was processed using the method by Redmond et al [99] to calculate RRE^ae^. No preprocessing was done on SpO_2_. Each feature was extracted using 30-second windows. *z* score normalization was applied to the feature matrix	47 features were extracted from the vitals. A logistic regression model achieved 0.9 AUROC in detecting central apnea
Máxima Medical Center NICU	Peng et al [49], 2022	Participants; n=128; data size: ~24 hours each; model: prognostic (+24 hours); outcome metric: sepsis	ECG at 250 Hz	QRS complexes from ECG were extracted using a DT-CWT–based method described by Rooijakkers et al [95]. RR intervals from the complexes were divided into nonoverlapping 1-hour segments. The segments were centered, and missing values in the segments were filled by zero padding on the 2 ends	A ResNet-based neural network, DeepLOS, was proposed, which achieved a 0.72 *F*_1_-score in predicting late-onset sepsis
Máxima Medical Center NICU	Peng et al [50], 2022	Participants; n=127; data size: ~48 hours each; model: prognostic (+6 hours); outcome metric: sepsis	ECG at 250 Hz, CI at 62.5 Hz	QRS complexes from ECG were extracted using a DT-CWT–based method described in [95]. CI signal was filtered to remove cardiac artifacts (method NR). Peaks were detected using the method reported by Lee et al [96]. SII was calculated from ECG and CI waveforms using a CWT-based method, as reported by Zuzarte et al [100]. Signals were divided into 1-hour-long nonoverlapping segments. Features were calculated in both 1-hour segments and 5-minute subsegments	60 Features were extracted from the signals. An XGB model using the features achieved an AUROC of 0.88 in predicting late-onset sepsis 6 hours preceding the onset.
Royal Infirmary of Edinburgh NICU	Stanculescu et al [51], 2014	Participants; n=24; data size: 30 hours each; model: prognostic (+3-6 hours); outcome metric: sepsis	ECG-derived HR, PR^af^ (sampling rate NR)	An extension of the forward-backward algorithm [101] is developed for missing data inference	An autoregressive HMM model achieved up to 0.80 AUROC in predicting sepsis
Royal Infirmary of Edinburgh NICU	Stanculescu et al [52], 2014	Participants; n=24; data size: 540 hours; model: diagnostic; outcome metric: sepsis	ECG-derived HR, PR core and peripheral temperature and SpO_2_ at 1 Hz	An automated oximeter error detection algorithm was applied on the basis of the method described by Stanculescu et al [51]. Rows containing missing data on the observation matrix are set to 0	An HSLDS^ag^ was able to predict sepsis with up to 0.65 *F*_1_-score
Kasturba hospital NICU, Manipal, India	Shirwaikar et al [53], 2016	Participant: NR; data size: 229 examples; model: diagnostic; outcome metric: apnea	HR (sampling rate NR)	Visualization technique was applied to identify issues in data. Missing values were not treated due to low percentage. For categorical features, 0 was added for missing values. Minimum-maximum normalization and *z* score normalization were done	An RF^ah^ model using HR features achieved 0.88 accuracy and 0.72 κ in detecting apnea
Kasturba hospital NICU, Manipal, India	Shirwaikar et al [54], 2019	Participants; n=367 (315 used); data size: NR; model: diagnostic; outcome metric: apnea	ECG (sampling rate NR)	No preprocessing techniques were reported on the raw signals. Observations with missing features were discarded. Other features (continuous values) that had missing values were converted to discrete with the addition of the group name “not known”	Statistical features were extracted from the signals. A Multilayer Perceptron model and a deep autoencoder model reached 0.82 and 0.83 AUROC, respectively, in detecting apnea
University of Massachusetts Memorial Healthcare NICU	Williamson et al [55], 2013	Participants; n=6; data size: ~5-8 hours for each patient; model: prognostic (+5.5 minutes); outcome metric: apnea	ECG, SpO_2_, respirator signal, pulse plethysmogram (sampling rate NR)	IBIs were extracted from abdominal respiratory movements (method NR), and RR intervals were extracted from ECG signals (method NR). Physically implausible IBI and RR interval values were automatically removed (range NR). Values were resampled to 10 Hz using shape-preserving piecewise cubic interpolation. Signals were then log transformed and converted to 0 mean, unit variance	Features were extracted from all signals. A GMM^ai^ model reached 0.8 AUROC in predicting apnea
Jackson Memorial Hospital NICU	Schiavenato et al [56], 2013	Participants; n=20; data size: 1186 minutes; model: diagnostic; outcome metric: periods of high distress or pain	ECG at 1000 Hz	Pan-Tompkins algorithm [85] was modified to detect QRS complexes. ECG was filtered using a bandpass filter with a 16-26-Hz cutoff frequency. A low-pass filter by an order 120 FIR^aj^ filter with a corner frequency of 25 Hz and a high-pass filter by an order 160 FIR filter with a corner frequency of 25 Hz were applied. Then, a polynomial filter of order 21 was applied as the differentiator filter. Finally, a 111-order moving average filter was used, and QRS complex was detected using an adaptive threshold. Lomb-Scargle LMS^ak^ spectral estimation [102] was used for missing and irregular RR intervals	The proposed framework provided real-time analysis and HRV extraction to identify the characteristics correlated to periods of high distress or pain
Montreal Children’s Hospital	Rubles-Rubio et al [57], 2014	Participants; n=24; data size: 9.0 (SD 2.2) hours for each; model: diagnostic; outcome metric: apnea	SpO_2_, RIP^al^ (sampling rate NR)	Signals were low-pass filtered with a cutoff frequency of 10 Hz, with an 8-pole Bessel antialiasing filter digitized and sampled at 50 Hz	A linear Gaussian discriminant classifier detected the episodes with a 0.73 probability of detection and 0.22 probability of false alarm
University of Alabama at Birmingham	Amperayani et al [58], 2017	Participants; n=18; data size: 24 hours each; model: prognostic (+23 hours); outcome metric: bradycardia, hypoxemia	ECG at 500 Hz and HR at 1 Hz	HR data were converted to interbeat RR intervals using RR=60/HR. No processing on ECG signals was reported	A point process model using RR intervals showed a strong correlation with bradycardia events and a modest correlation with hypoxemia events
Monash Children’s Hospital NICU, Australia	Hu et al [59], 2018	Participants; n<80; data size: 407 patient-day; model: prognostic (+24 hours); outcome metric: sepsis	HR, SpO_2_, respiration signal at 1 Hz	Data were scaled down to 1 record per minute. Data blocks with invalid values were deleted. Then, the sliding window was set to 60 minutes to feed to the ML^am^ models	Features were extracted from all signals. A gradient boosting decision tree achieved up to 0.97 AUROC and 0.92 weighted *F*_1_-score in patient-based cross-validation in predicting sepsis
University of Virginia and Columbia University NICU	Sullivan et al [60], 2018	Participants; n=78; data size: NR; model: prognostic (+12 hours); outcome metric: death, sIVH^an^ (severe), BPD^ao^, treated ROP, ^ap^ late-onset sepsis, and NEC^aq^	HR, SpO_2_ at 0.5 Hz	Infants with <6 hours of data within 12 hours of birth were discarded. Cross-correlation of HR and SpO_2_ was calculated over 10-minute windows using the XCORR function of MATLAB with a lag time of –30 to +30 seconds	A POPS^ar^ was developed and fit a multivariate logistic regression model, which performed well in predicting death, sIVH, and BPD, but not tROP, sepsis, and NEC
9 NICUs in the United States	Zimmet et al [61], 2020	Participants; n=2989; data size: 121 data points per infant; model: prognostic (+2 days); outcome metric: mortality, sepsis	HRC^as^ index from ECG	Infants with missing data on either end of the total duration were extrapolated to the window edge by repeating the most proximal HRC index values. Interior missing values were updated using linear interpolation. A fifth-order B-spline with equally spaced knots was used to capture information from independent samples (HRC indexes 12 samples apart)	An unsupervised ensemble of clustering techniques was proposed to cluster infants to different levels of risk
Children’s National Hospital, Washington	Kota et al [103], 2020	Participants; n=95; data size: median recording duration of 75.78 hours; model: diagnostic; outcome metric: HIE	EEG at 200 or 256 Hz	ECG contamination from EEG was detected using the method described by Govindan et al [104]. EEG signals with amplitude>500 μV or SD<0.01 μV were discarded as artifacts. The volume conduction was attenuated by calculating the global average of EEG voltages from all electrodes and subtracting the global average from the EEG value of every electrode in the frequency domain [62]. The values were then transformed to the time domain for spectral analysis. EEG was segmented into 10-minute nonoverlapping artifact and seizure-free epochs. Spectral analysis was done using a Welch periodogram approach [105,106] using 3-second epochs	EEG delta power was identified to be a crucial biomarker for predicting neonates with HIE who died with those who survived
Akbar Abadi Hospital NICU, Iran	Mirnia et al [63], 2021	Participants; n=5; data size: ~24 hours each; model: diagnostic, outcome metric: sepsis	ECG at 200 Hz	RR intervals were calculated from ECG using HeRO^at^ model	Features were extracted from HRV. HeRO model was tested using this data set. HeRO score was able to distinguish between healthy and septic newborns
St Louis Children’s Hospital NICU	Lee et al [64], 2021	Participants; n=275; data size: 4, 01,33,460 data points; model: prognostic (+6 hours); outcome metric: mortality	HR, respiration signal and SpO_2_ at 1 Hz	Missing or out-of-range values were replaced with NaN and then imputed using mean values for that variable across all training and testing data. Data were downsampled to every 10 seconds to extract features. Dynamic variables were calculated as rolling means, SD, and absolute *z* score on 5- and 30-minute windows to reduce the influence of outliers	Thirty-four features were extracted from the signals. An RF model achieved 88% sensitivity and 0.93 AUROC in predicting mortality
University of Virginia Children’s Hospital, Morgan Stanley Children’s Hospital, and St Louis Children’s Hospital	Sullivan et al [65], 2021	Participants; n=408, (266 used); data size: NR; model: diagnostic; outcome metric: sepsis	HR and SpO_2_ at 0.5 Hz	HR and SpO_2_ values of 0 were removed. Eight features were extracted in 10-minute windows and averaged hourly. Cross-correlation between HR and SpO_2_ was calculated in 10-minute windows of signals normalized to have 0 mean and SD of 1. Cross-correlation was done using the XCORR function of MATLAB with a lag time of –30 to +30 seconds	A logistic regression model using clinical and physiological features achieved an AUC^au^ of 0.821 in predicting late-onset sepsis
St Louis Children’s Hospital NICU	Feng et al [66], 2021	Participants; n=285; data size: ~80 hours each; model: prognostic (+6 hours); outcome metric: mortality	HR, respiration, SpO_2_, and ART-M^av^ or NIBP-M^aw^ at 1 Hz	Infants with data >80 hours were truncated, and <80 hours were padded with 0s. Mean, median, mode, and Bayesian ridge data imputation techniques were explored. Bayesian ridge was used to sample 5 data sets by sampling different posteriors each time. Then, the average was reported using 4-fold cross-validation. The rolling mean of each vital sign with a range of 5 minutes was used to reduce noise. Finally, the end of each sample was padded with 1 segment where all features equaled 0. Features were extracted from 5-minute segments	A deep learning model using LSTM named DeepPBSMonitor was developed to predict mortality with 0.888 accuracy, 0.78 recall, and 0.897 AUC
University of Massachusetts Memorial Healthcare	Zuzarte et al [67], 2021	Participants; n=10; data size: 241.34 hours; model: prognostic (+310 seconds); outcome metric: apnea-bradycardia-hypoxia	ECG at 500 Hz, PPG^ax^ at 125 Hz, SpO_2_, HR, respiration signals from pneumogram at 50 Hz	PPG signals were filtered using a wavelet-based algorithm to remove gross body movements. A binary marker sampled at 25 Hz was obtained to indicate the presence or absence of movement. QRS complexes were detected using a modified Pan-Tompkins algorithm (modification NR). IBIs were detected using automated peak detection from LabChart Software RR intervals, and IBI values were then interpolated at 10 Hz	The prediction framework using GMM and logistic regression model achieved 75% accuracy in predicting bradycardia severity during the apnea-bradycardia-hypoxia event
University of Virginia NICU	Niestroy et al [68], 2022	Participants; n=5957; data size: random daily 10 minutes segments from each; model: prognostic (+1-7 days); outcome metric: mortality	HR and SpO_2_ at 0.5 Hz	No preprocessing was reported on the vitals. They were grouped to calculate the average in 10-minute nonoverlapping windows	Features were extracted from all signals. A multivariable logistic regression model using 5 features achieved the AUROC of 0.83 in predicting mortality
University of Virginia Children’s Hospital, Morgan Stanley Children’s Hospital and St Louis Children’s Hospital	Kausch et al [69], 2023	Participants; n=2494; data size: NR; model: prognostic (+24 hours); outcome metric: sepsis	HR and SpO_2_ at 0.5 Hz	HR and SpO_2_ were preprocessed by removing the values containing 0. Features were calculated in 10-minute nonoverlapping windows. Windows with >50% missing data were excluded from subsequent analysis	Several features were extracted from the vitals. An XGB model achieved training AUROC of 0.834 using the data from NICU 1, and 0.792 and 0.807 testing AUROC using data from NICU 2 and NICU 3, respectively
Karolinska University Hospital Solna and Huddinge NICU, Stockholm, Sweden	Honoré et al [70], 2023	Participants; n=325; data size: 2866 hospitalization days; model: prognostic (+24 hours); outcome metric: sepsis	IBI from ECG, respiration from CI, SpO_2_ (sampling rate 1-500 Hz)	All signals were resampled to 1 Hz. Segments with at most 15 seconds missing were linearly interpolated. All signals were filtered with a moving mean filter of width 3. IBI signals were further filtered to remove ectopic beats and strong nonlinearities with a moving median filter of width 3 and Butterworth bandpass filter of order 6 with low-cut and high-cut frequencies of 0.0021 and 0.43 Hz. Signals were divided into 45-minute segments. Features were calculated using a sliding time frame with 50% overlap	A naive bayes classifier achieved an AUROC of 0.82 up to 24 hours before clinical suspicion of sepsis. Adding respiratory signals improved the performance compared with only using heart rate features
Simulated and real data (NICU name NR)	Masoudi et al [71], 2013	Participants; n=32; data size: 233 episodes, ~7 seconds each; model: diagnostic (+2.32-second delay); outcome metric: apnea-bradycardia	2-channel ECG	No preprocessing techniques were reported. Signals were sampled in 7-second intervals	A coupled HMM model achieved 84.92% sensitivity, 94.17% specificity with a time detection delay of 2.32 (SD 4.82) seconds in detection apnea-bradycardia episodes
Simulated and real signals from NICU (NICU name NR)	Altuve et al [72], 2015	Participants; n=32; data size: 148 RR intervals with a mean duration of 26.25 (SD 11.37) minutes; model: diagnostic (+1.73-second delay); Outcome metric: apnea-bradycardia	ECG (sampling rate NR)	Hidden semi-Markov models to represent the temporal evolution of RR intervals. A preprocessing method that includes quantization and a delayed version of the observation vector is proposed. RR time series was resampled at 10 Hz and segmented at a 7-second interval	The proposed model achieved up to 93.84 (SD 0.79) in specificity and 89.66 (SD 0.71) in sensitivity with a detection delay of 1.59 (SD 0.24) seconds
NICU (name NR)	Honoré et al [73], 2020	Participants; n=22; data size: 3501time series, 1200 samples in each; model: prognostic (+72 hours); outcome metric: sepsis	SpO_2_, respiratory frequency, and RR interval from ECG at 1 Hz	Data were segmented into 20-minute time frames. Time frames with missing data were discarded	Features were extracted from all signals. A combined GMM-HMM model achieved 0.74% (SD 0.05%) accuracy in detecting sepsis. The model was compared with HeRO model, which underperformed using this data set

^a^CHIME: Collaborative Home Infant Monitoring Evaluation.

^b^NR: not reported.

^c^ECG: electrocardiogram.

^d^SpO_2_: oxygen saturation.

^e^PICS: Preterm Infant Cardio-Respiratory Signals.

^f^AUROC: area under receiver operating characteristic curve.

^g^FPR: false positive rate.

^h^HRV: heart rate variability.

^i^MIMIC-III: Medical Information Mart for Intensive Care.

^j^HR: heart rate.

^k^SBP: systolic blood pressure.

^l^DBP: diastolic blood pressure.

^m^MBP: mean blood pressure.

^n^LSTM: convolutional neural network-Long Short-Term Memory Network.

^o^LOS: length of stay.

^p^KNN: k-nearest neighbor.

^q^RMSE: root mean square error.

^r^SG: Savitzky-Golay.

^s^CHMM: coupled Hidden Markov Model.

^t^RNN: recurrent neural network.

^u^HMM: Hidden Markov Model.

^v^EEG: electroencephalography.

^w^SVM: support vector machine.

^x^HIE: hypoxic-ischemic encephalopathy.

^y^MAP: mean arterial pressure.

^z^NICU: neonatal intensive care.

^aa^CI: chest impedance.

^ab^DT-CWT: Discrete Time Continuous Wavelet Transform.

^ac^IBI: interbreath variable.

^ad^SII: Signal Instability Index.

^ae^RRE: ribcage respiratory effort.

^af^PR: pulse oximeter.

^ag^HSLDS: Hierarchical Switching Linear Dynamical System.

^ah^RF: random forest.

^ai^GMM: Gaussian Mixture Model.

^aj^FIR: Finite impulse response.

^ak^LMS: least-mean-square.

^al^RIP: respiratory inductive plethysmograph.

^am^ML: machine learning.

^an^IVH: intraventricular hemorrhage.

^ao^BPD: bronchopulmonary dysplasia

^ap^ROP: retinopathy of prematurity.

^aq^NEC: necrotizing enterocolitis

^ar^POPS: pulse oximetry predictive score.

^as^HRC: heart rate characteristics.

^at^HeRO: heart rate observation.

^au^AUC: area under the curve.

^av^ART-M: arterial mean blood pressure.

^aw^NIBP-M: noninvasive blood pressure.

^ax^PPG: photoplethysmography.

### Preprocessing Steps

#### Overview

Preprocessing of physiological data typically involves several steps, including the handling of missing data, filtering, segmentation, and waveform analysis for feature extraction. Here, we define 5 required preprocessing steps (based on the steps outlined in Berkaya et al [13]) and identify the steps reported by each of the studies in this review (Table 3). The definition of each of the steps is given in subsequent sections.

**Table 3:** Required physiological signal preprocessing steps reported by each of the studies in this review.

Author, year	Required preprocessing step reported				
	Handling of missing data	Artifact removal	Resampling, normalization	Waveform feature extraction	Data segmentation
Cohen and de Chazal [22], 2013	✓	✓		✓	✓
Cohen and de Chazal [23], 2014	✓	✓	✓	✓	✓
Cohen and de Chazal [24], 2015	✓	✓		✓	✓
Gee et al [26], 2016				✓	✓
Gee et al [25], 2017		✓		✓	✓
Das et al [27], 2019		✓		✓	✓
Mahmud [28], 2019					
Gee et al [29], 2019		✓	✓	✓	✓
Song et al [30], 2020	✓	✓		N/A^a^	
Baker et al [31], 2021		✓	✓	N/A	✓
Juraev et al [32], 2022	✓		✓	N/A	
Montazeri Ghahjaverestan et al [33], 2015		✓	✓	✓	
Navarro et al [34], 2015		✓	✓	✓	✓
Montazeri Ghahjaverestan et al [35], 2016		✓	✓	✓	✓
León et al [36], 2021				✓	✓
León et al [37], 2021				✓	✓
Doyen et al [38], 2021				✓	✓
Sadoughi et al [39], 2021		✓	✓	✓	
Ahmed et al [40], 2015	✓	✓	✓	✓	✓
Temko et al [41], 2015		✓	✓	✓	✓
Lloyd et al [42], 2016		✓		N/A	✓
Semenova et al [43], 2018		✓		✓	✓
Semenova et al [44], 2019		✓	✓	✓	✓
Joshi et al [45], 2020		✓		✓	✓
Varisco et al [46], 2021		✓	✓	✓	
Cabrera-Quiros et al [47], 2021				✓	✓
Varisco et al [48], 2022		✓	✓	✓	✓
Peng et al [49], 2022				✓	✓
Peng et al [50], 2022		✓		✓	✓
Stanculescu et al [51], 2014	✓	✓		N/A	
Stanculescu et al [52], 2014	✓	✓		N/A	
Shirwaikar et al [53], 2016	✓	✓	✓	N/A	✓
Shirwaikar et al [54], 2019	✓			N/A	✓
Williamson et al [55], 2013			✓		✓
Schiavenato et al [56], 2013	✓	✓		✓	
Robles-Rubio et al [57], 2014		✓	✓	N/A	
Amperayani et al [58], 2017					
Hu et al [59], 2018	✓	✓	✓	N/A	✓
Sullivan et al [60], 2018	✓			N/A	✓
Zimmet et al [61], 2020	✓			N/A	✓
Kota et al [62], 2020		✓		✓	✓
Mirnia et al [63], 2021				N/A	
Lee et al [64], 2021	✓	✓	✓	N/A	✓
Sullivan et al [65], 2021	✓	✓		N/A	✓
Feng et al [66], 2021	✓	✓	✓	N/A	✓
Zuzarte et al [67], 2021		✓	✓	✓	✓
Niestroy et al [68], 2022				N/A	✓
Kausch et al [69], 2023	✓	✓		N/A	✓
Honoré et al [70], 2023	✓	✓	✓	N/A	✓
Masoudi et al [71], 2013					✓
Altuve et al [72], 2015		✓	✓		✓
Honoré et al [73], 2020	✓			N/A	✓

^a^N/A: Not applicable.

#### Handling of Missing Data

During neonatal physiological monitoring, instances of missing data may arise due to sensor disconnection, improper placements, or signal dropouts. To tackle this issue, methodologies like data imputation or interpolation are applied. For example, if gaps exist in a neonate’s HR monitoring data, interpolation methods can estimate the missing values by considering neighboring data points. Widely used interpolation techniques include linear interpolation, spline interpolation, and time-based interpolation. In addition, common data imputation methods involve forward fill, backward fill, and imputation using mean or median values. Methods such as forward fill [30], moving average [44], mean imputation [64,66], and interpolation [67] were used by some studies reviewed in this paper.

#### Artifact Removal

Neonatal signals can be affected by artifacts, such as those from muscle movements or electrical interference. Commonly used techniques, such as bandpass or notch filters, along with moving averages, are used to effectively eliminate these disturbances. For instance, in neonatal EEG signals, adaptive filters prove beneficial in eliminating artifacts caused by muscle movements, resulting in a clearer representation of the baby’s brain activity. Some methods used by the reviewed papers were high-pass filter [27,46] bandpass filter [29,33,44,45,56].

#### Resampling and Normalization

##### Overview

Resampling is a technique that standardizes data intervals, involving either upsampling (increasing data point frequency) or downsampling (decreasing frequency) to create a regular time series. This aligns signals from different devices or physiological sources. Normalization ensures uniformity and reliability across these standardized sampling rates. For instance, if neonatal HR signals from different devices have varied sampling rates, resampling achieves a common rate, while normalization, using techniques such as minimum-maximum, *z* score, or log scale, ensures consistent amplitude scaling for accurate comparative analysis. In the reviewed studies, normalization techniques such as minimum-maximum [53] and 0 mean normalization [29,59] were used. In terms of resampling, both downsampling [33,34,41] and upsampling [39] techniques were used.

##### Waveform Feature Extraction

Extracting relevant features from a signal’s waveform is a fundamental step in signal preprocessing. This involves identifying key characteristics such as peaks, troughs, or other significant points in the signal. In the context of neonatal ECG, feature extraction may involve identifying key points such as R-peaks to analyze HRV, providing valuable insights into the infant’s autonomic nervous system development. The Pan-Tompkins algorithm is a popular method chosen by multiple papers reviewed in this study that conducted R-peak detection from the QRS complex [22,24,27,33,35,39].

##### Data Segmentation

Segmenting data is the process of breaking down a continuous signal into smaller, more manageable sections to enable targeted analysis. This practice is especially beneficial when dealing with lengthy signals. Data segmentation is a common preprocessing step in ML workflows. For instance, in the analysis of neonatal sleep patterns using EEG, data segmentation can involve dividing the continuous EEG signal into epochs, allowing for the identification and study of sleep stages in shorter, more manageable segments. Commonly used segmentation techniques include fixed length, sliding window, and threshold- and feature-based segmentation. Some of the data segmentation sizes used in the reviewed studies were 30-second [22-24,45] and 1-minute [41] epochs and a sliding window of varied sizes [35,40,55,59,64].

In neonatal physiological signal processing, these preprocessing techniques contribute to the accurate interpretation of signals, aiding health care professionals in monitoring and providing appropriate care in the NICU or other clinical settings.

It can be seen from Table 3 that only 7 (13%) out of the 52 reviewed studies reported all the recommended preprocessing steps. This could have several impacts on the downstream analysis. For instance, several papers missed reporting on how they segmented the data for feature extraction and classification, although it is essential for clinical validation in cases where the segment duration is dependent on the adverse outcome prediction performance. In HRV analysis, it is important to indicate whether it is a short-term (~5 minutes) or a long-term (≥24 hours) analysis as they reflect different underlying physiological processes and thus demonstrate different predictive power [107]. Along with the segment duration, additional information such as the sampling rate of the signals will provide a clear reflection of the data set size. Downsampling the data to a low sampling rate (eg, 50 Hz) has also shown a significant impact on HRV analysis [108]. Although all the reviewed studies mentioned the participant number, and majority of them (n=39) reported the sampling rate of the signals, very few provided details on the sample size or data set duration or whether the data set was resampled for subsequent analysis. These elements provide a clearer picture of the computational time and resources required for clinical validation and adoption. Although physiological recordings collected in the NICU environment suffer greatly from missing data due to similar factors that introduce artifacts [109], reporting how missing data are handled is scarce. Different methods for dealing with missing values could cause different results, and not all might be suitable for a particular problem. Therefore, it is important to report all the details related to the adopted approach.

The incomplete or partial reporting found in these studies has significant implications for the implementation of QMS in using these techniques for clinical adoption. A good implementation of QMS requires a comprehensive reporting of each intermediary step involved in constructing an AI and ML pipeline. The International Medical Device Regulators Forum offers guidance on the clinical evaluation required for any product intended for use as a medical device [110]. According to the International Medical Device Regulators Forum guidelines, during clinical evaluation, relevant research articles are reviewed to identify clinical evidence supporting the product [111]. The guideline encourages manufacturers to follow these recognized standards and best practices in the development, validation, and manufacturing processes. Clinical evaluations are required by the European Union medical device regulation, and it is also mentioned in the ISO 13485 (the quality management standard for medical devices). Thus, detailed reporting is crucial as it can be used by regulatory bodies to evaluate future SaMD products clinically. Steps such as the missing data handling procedures are also required by the TRIPOD (Transparent Reporting of a Multivariable Prediction Model for Individual Prognosis or Diagnosis) checklist for model development and validation, which assesses the risk of bias and clinical usefulness of the prediction model [112]. Another example is a questionnaire prepared by the German Notified Body Interest Group, and it was adopted to assess some AI-powered medical products in the European Union. This questionnaire includes inquiries about data management, including data collection, labeling, preprocessing procedures, and relevant documentation. Transparent and detailed reporting of these steps is essential to ensure the safety, efficacy, and reliability of SaMD.

## Discussion

### Principal Findings

This review aimed to summarize the computational methods used for preprocessing preterm infants’ physiological data as a first step in developing data-driven predictive models for adverse outcomes related to clinical decision support. This is an important step, especially from a clinician’s perspective, because it increases the trustworthiness of the developed models by allowing for the verification and reproduction of the results. In addition, it aids in achieving regulatory compliance and ensures the safety, efficacy, and ethical use of AI-based health care devices. Furthermore, it allows us to recognize the shortcomings in the current state-of-the-art studies and recommend guidelines for transparent reporting. The review found that the studies were heterogeneous in terms of their methods and applications. Therefore, a narrative approach to reporting the results was taken instead of a quantitative approach. Through the analysis we identified several key components that were incomplete or partially reported by the included studies, which are summarized in Table 3. To ensure transparent reporting for any future studies in this area, we recommend detailed reporting of all preprocessing steps listed in Table 3, which will allow revealing their strengths and weaknesses and ultimately make them usable and reproducible. Reproducible research allows clinicians to make more informed decisions about patient care and treatment based on the evidence that has been thoroughly assessed.

### Comparison With Prior Work

The reviews published in recent years have highlighted the potential of big data and AI in supporting clinical decision-making in the neonatal health care domain [10,15,21,113,114], particularly in using physiological data for detecting or predicting neonatal health outcomes. However, appropriate preprocessing of these data is a prerequisite for developing clinically deployable models. A systematic review by McAdams et al [10] reported different ML models used to predict different clinical outcomes in neonates. However, their primary focus was on 5 neonatal morbidities, and they did not focus on reporting the preprocessing methods applied before building the ML models. Furthermore, they did not include studies using real-time continuous physiological data; 28 out of their 68 studies were based on physiological data (not continuous), and the rest were based on electronic medical records and imaging data. Latremouille et al [15] performed a review on HRV analysis for neonates. The primary limitation of the work was the lack of reporting in detail about the preprocessing steps of ECG signals before HRV analysis, such as ECG handling and segmentation, R-wave (QRS complex) identification technique, software and parameters, and ranges of all HRV features. They identified these components as incomplete or missing in the studies they reviewed and thus recommended clear reporting of these aspects for future studies in this area. These limitations served as a motivation for our review to focus on the preprocessing techniques of neonatal physiological signals in a broader sense, which serves as the preliminary step for any big data–based approaches.

### Limitations

There are several limitations to this review. Screening of all the included studies was conducted independently by 1 reviewer, which may have introduced bias. In addition, this review did not include a quantitative or comparative analysis of the reviewed studies, as the techniques used to analyze the physiological signals were diverse. Future work could include a quantitative evaluation of the studies that were homogeneous in design.

### Conclusions

This review explores the computational methods used by the current state-of-the-art ML-driven clinical decision support approaches to preprocess physiological signals collected from infants treated in the neonatal setting. A summary of the studies identified heterogeneity in the techniques used for analysis and revealed a lack of consistent and detailed reporting, which is important for building robust, transparent, and clinically deployable prediction models. The availability of powerful hardware and software resources in the NICU environment and growing interest in big data and AI are driving strong demand for clinical decision support applications. We recommend clear reporting of the different steps in the preprocessing of the neonatal physiological signals to ensure transparency in clinical validation and accelerate the adoption of developed models in the clinical setting. This will further enhance the delivery and adoption of reliable, regulatory-compliant, safe, and effective products in health care.

## Appendix

Multimedia Appendix 1 Detailed search queries.

Multimedia Appendix 2 Bibliography files for all databases.

Multimedia Appendix 3 All included papers.

Multimedia Appendix 4 Metadata of all papers.

Multimedia Appendix 5 Metadata of all papers in the full-text review.

Multimedia Appendix 6 PRISMA-ScR checklist.

## Abbreviations

AI: artificial intelligence
CDSS: clinical decision support system
ECG: electrocardiogram
EEG: electroencephalography
HR: heart rate
HRV: heart rate variability
MeSH: Medical Subject Headings
ML: machine learning
NICU: neonatal intensive care unit
PRISMA: Preferred Reporting Items for Systematic Reviews and Meta-Analyses
QMS: quality management system
SaMD: software as a medical device
SpO2: oxygen saturation
TRIPOD: Transparent Reporting of a Multivariable Prediction Model for Individual Prognosis or Diagnosis

## Data Availability

The data sets generated during and analyzed during this study are available from the corresponding author on reasonable request or are available in the Multimedia Appendices.
