# Supplementary material for: Exploring Computational Techniques in Preprocessing Neonatal Physiological Signals for Detecting Adverse Outcomes: Scoping Review
Source: Interact J Med Res. 2024 Aug 20;13:e46946. doi: 10.2196/46946 (PMC11372324; doi:10.2196/46946)
Supplement: Multimedia Appendix 1 [file ijmr_v13i1e46946_app1.docx]

**Database Search Strategy**

This document lists the databases searched for the review paper, the keywords and MeSH terms used, and the full search query for each of the databases searched.

**Databases**

1. Pubmed
2. Scopus
3. ACM Digital Library
4. Web of Science
5. IEEE

**Concepts**

**Concept 1: Neonates/Preterm babies**

**MeSH Term (Pubmed Only):** "Infant, Premature"[Mesh]

**Keywords:** premature OR preterm OR neonat* OR newborn OR infant OR nicu OR “neonatal intensive care unit”

**Concept 2: Physiological Signals/Vital Signs**

**MeSH Term (Pubmed Only):** "Vital Signs"[Mesh]) OR "Physiology"[Mesh]

**Keywords:** physiolog* OR ecg OR "heart rate *" OR electrocardiography OR "vital sign**" OR physiomarker OR biomarker OR hrv

**Concept 3: Signal processing**

**MeSH Term (Pubmed Only):** "Signal Processing, Computer-Assisted"[Mesh]

**Keywords:** “signal ” OR predict OR detect* OR comput*

**Concept 4: Outcomes**

**MeSH Term:** N/A

**Keywords:** sepsis OR mortality OR “length of stay” OR “intraventricular hemorrhage” OR “hypoxi*” OR apnea OR “necrotising entercolitis” OR “necrotizing entercolitis”

**Search Queries**

**Pubmed**

((("premature"[Title/Abstract] OR "preterm"[Title/Abstract] OR "neonat*"[Title/Abstract] OR "newborn"[Title/Abstract] OR "infant"[Title/Abstract] OR "nicu"[Title/Abstract] OR "neonatal intensive care unit"[Title/Abstract]) AND ("physiolog*"[Title/Abstract] OR "ecg"[Title/Abstract] OR "heart rate*"[Title/Abstract] OR "electrocardiography"[Title/Abstract] OR "vital sign*"[Title/Abstract] OR "physiomarker"[Title/Abstract] OR "biomarker"[Title/Abstract] OR "hrv"[Title/Abstract]) AND ("signal*"[Title/Abstract] OR "predict*"[Title/Abstract] OR "detect*"[Title/Abstract] OR "comput*"[Title/Abstract]) AND ("sepsis"[Text Word] OR "mortality"[Text Word] OR "length of stay"[Text Word] OR "intraventricular hemorrhage"[Text Word] OR "hypoxi*"[Text Word] OR "apnea"[Text Word] OR "necrotising entercolitis"[Text Word] OR "necrotising entercolitis"[Text Word])) NOT ("review"[Publication Type] OR "systematic review"[Publication Type])) AND 2013/01/01:2023/01/09[Date - Publication] -

**Scopus**

(TITLE-ABS-KEY(premature OR preterm OR neonat* OR newborn OR infant OR nicu OR "neonatal intensive care unit") AND TITLE-ABS-KEY(physiolog* OR ecg OR "heart rate " OR electrocardiography OR "vital sign" OR physiomarker OR biomarker OR hrv) AND TITLE-ABS-KEY("signal *" OR predict* OR detect* OR comput*) AND TITLE-ABS-KEY(sepsis OR mortality OR "length of stay" OR "intraventricular hemorrhage" OR "hypoxi*" OR apnea OR "necrotising entercolitis" OR "necrotizing entercolitis")) AND ( LIMIT-TO ( DOCTYPE,"ar" ) OR LIMIT-TO ( DOCTYPE,"cp" ) OR LIMIT-TO ( DOCTYPE,"ch" ) ) AND ( LIMIT-TO ( SUBJAREA,"MULT" ) OR LIMIT-TO ( SUBJAREA,"ENGI" ) OR LIMIT-TO ( SUBJAREA,"COMP" ) ) AND (LIMIT-TO ( PUBYEAR,2023) OR LIMIT-TO ( PUBYEAR,2022) OR LIMIT-TO ( PUBYEAR,2021) OR LIMIT-TO ( PUBYEAR,2020) OR LIMIT-TO ( PUBYEAR,2019) OR LIMIT-TO ( PUBYEAR,2018) OR LIMIT-TO ( PUBYEAR,2017) OR LIMIT-TO ( PUBYEAR,2016) OR LIMIT-TO ( PUBYEAR,2015) OR LIMIT-TO ( PUBYEAR,2014) OR LIMIT-TO ( PUBYEAR,2013)) AND ( LIMIT-TO ( LANGUAGE,"English" ) )

**ACM Digital Library**

*[[All: premature] OR [All: preterm] OR [All: neonat*] OR [All: newborn] OR [All: infant] OR [All: nicu] OR [All: "neonatal intensive care unit"]] AND [[All: physiolog*] OR [All: ecg] OR [All: "heart rate "] OR [All: electrocardiography] OR [All: "vital sign*"] OR [All: physiomarker] OR [All: biomarker] OR [All: hrv]] AND [[All: "signal *"] OR [All: predict*] OR [All: detect*] OR [All: comput*]] AND [[All: sepsis] OR [All: mortality] OR [All: "length of stay"] OR [All: "intraventricular hemorrhage"] OR [All: "hypoxi*"] OR [All: apnea] OR [All: "necrotising entercolitis"] OR [All: "necrotizing entercolitis"]] AND [Publication Date: (01/01/2013 TO 09/01/2023)]

**Web of Science**

premature OR preterm OR neonat* OR newborn OR infant OR nicu OR “neonatal intensive care unit” (All Fields) AND physiolog* OR ecg OR "heart rate " OR electrocardiography OR "vital sign*" OR physiomarker OR biomarker OR hrv (All Fields) AND “signal *” OR predict* OR detect* OR comput* (All Fields) AND sepsis OR mortality OR “length of stay” OR “intraventricular hemorrhage” OR “hypoxi*” OR apnea OR “necrotising entercolitis” OR “necrotizing entercolitis” (All Fields) and Article or Proceeding Paper or Book Chapters (Document Types) and 2023 or 2022 or 2021 or 2020 or 2019 or 2018 or 2017 or 2016 or 2015 or 2013 or 2014 or 2012 or 2011 or 2010 or 2009 or 2008 or 2007 or 2006 or 2005 or 2004 or 2003 (Publication Years) and 2022 or 2021 or 2020 or 2019 or 2018 or 2017 or 2016 or 2015 or 2014 or 2013 or 2012 or 2011 or 2010 or 2009 or 2008 or 2007 or 2006 or 2005 or 2004 or 2003 (Publication Years) and Engineering Multidisciplinary or Biochemical Research Methods or Computer Science Artificial Intelligence or Computer Science Theory Methods or Computer Science Information Systems or Medical Informatics or Mathematical Computational Biology or Computer Science Interdisciplinary Applications or Engineering Electrical Electronic or Multidisciplinary Sciences or Engineering Biomedical or Pediatrics (Web of Science Categories)

**IEEE**

(("All Metadata": premature OR "All Metadata": preterm OR "All Metadata": neonat* OR "All Metadata": newborn OR "All Metadata": infant OR "All Metadata": nicu OR "All Metadata": “neonatal intensive care unit”) AND ("All Metadata": physiolog* OR "All Metadata": ecg OR "All Metadata": "heart rate " OR "All Metadata": electrocardiography OR "All Metadata": "vital sign*" OR "All Metadata": physiomarker OR "All Metadata": biomarker OR "All Metadata": hrv) )
