# Supplementary material for: Exploring Computational Techniques in Preprocessing Neonatal Physiological Signals for Detecting Adverse Outcomes: Scoping Review
Source: Interact J Med Res. 2024 Aug 20;13:e46946. doi: 10.2196/46946 (PMC11372324; doi:10.2196/46946)
Supplement: Multimedia Appendix 3 [file ijmr_v13i1e46946_app3.zip › Included Papers - Final/2939/C. León et al. - 2021 - Early Detection of Late Onset Sepsis in Premature .pdf]

Early Detection of Late Onset Sepsis in Premature Infants Using Visibility Graph Analysis of Heart Rate Variability

C. Leon, G Carrault, P. Pladys, A. Beuchee

To cite this version:

C. Leon, G Carrault, P. Pladys, A. Beuchee. Early Detection of Late Onset Sepsis in Premature Infants Using Visibility Graph Analysis of Heart Rate Variability. IEEE Journal of Biomedical and Health Informatics, Institute of Electrical and Electronics Engineers, 2021, 25 (4), pp.1006-1017. 10.1109/JBHI.2020.3021662 . hal-03225182

HAL Id: hal-03225182
https://hal.archives-ouvertes.fr/hal-03225182

Submitted on 19 May 2021

HAL is a multi-disciplinary open access archive for the deposit and dissemination of scientific research documents, whether they are published or not. The documents may come from teaching and research institutions in France or abroad, or from public or private research centers.

L'archive ouverte pluridisciplinaire **HAL**, est destinée au dépôt et à la diffusion de documents scientifiques de niveau recherche, publiés ou non, émanant des établissements d'enseignement et de recherche français ou étrangers, des laboratoires publics ou privés.

This article has been accepted for publication in a future issue of this journal, but has not been fully edited. Content may change prior to final publication. Citation information: DOI 10.1109/JBHI.2020.3021662, IEEE Journal of Biomedical and Health Informatics

Early detection of late onset sepsis in premature infants using visibility graph analysis of heart rate variability

Cristhyne León, Guy Carrault, Patrick Pladys, and Alain Beuchée

Abstract—**Objective: This study was designed to test the diagnostic value of visibility graph features derived from the heart rate time series to predict late onset sepsis (LOS) in preterm infants using machine learning. Methods: The heart rate variability (HRV) data was acquired from 49 premature newborns hospitalized in neonatal intensive care units (NICU). The LOS group consisted of patients who received more than five days of antibiotics, at least 72 hours after birth. The control group consisted of infants who did not receive antibiotics. HRV features in the days prior to the start of antibiotics (LOS group) or in a randomly selected period (control group) were compared against a baseline value calculated during a calibration period. After automatic feature selection, four machine learning algorithms were trained. All the tests were done using two variants of the feature set: one only included traditional HRV features, and the other additionally included visibility graph features. Performance was studied using area under the receiver operating characteristics curve (AUROC). Results: The best performance for detecting LOS was obtained with logistic regression, using the feature set including visibility graph features, with AUROC of 87.7% during the six hours preceding the start of antibiotics, and with predictive potential (AUROC above 70%) as early as 42h before start of antibiotics. Conclusion: These results demonstrate the usefulness of introducing visibility graph indexes in HRV analysis for sepsis prediction in newborns. Significance: The method proposed the possibility of non-invasive, real-time monitoring of risk of LOS in a NICU setting.**

Index Terms—**Heart rate variability, visibility graph, late onset sepsis, machine learning, premature infants, predictive monitoring.**

I. Introduction

Late onset sepsis (LOS) is one of the main causes of morbidity and mortality in neonates [1]. Very preterm infants are especially vulnerable, with 10-25% of them presenting at least one episode of late onset sepsis, which in turn increases the length of hospital stay, morbidity, and mortality [2].

Some studies have found that prompt diagnosis and administration of antibiotics can significantly reduce mortality ([3], [4]). However, indiscriminate administration of antibiotics is also counterproductive, as it can further increase the level of antimicrobial resistance [5], which is already considered a threat to global public health by the World Health Organization [6]. In addition, antibiotics themselves can cause harmful side effects to the patients ([7], [8]). Therefore, prompt and accurate diagnosis, leading to adequate use of antibiotics is the key to decrease sepsis-related morbidity and mortality, while also protecting patients from unnecessary antibiotic treatment. However, blood cultures and other laboratory tests used to diagnose sepsis are invasive, take time, and present variations in their predictive value, especially in the early phases of infection [9]. Changes in heart rate variability (HRV) have been associated with neonatal sepsis [10].

Diagnosis relying on heart rate and HRV have the advantage of being non-invasive and readily and continuously available in the context of neonatal intensive care unit (NICU). HRV analysis typically relies on three different categories of features: time-domain, frequency-domain, and non-linear measurements. Previous studies have shown that machine learning algorithms (MLAs) using HRV, based on these features as input, can be useful in early detection of sepsis both in infants and adults ([11]–[14]). More recently, network-based time series analysis has also been applied to HRV ([15], [16]), and visibility graph features have shown to be of interest for the diagnosis of different conditions known to alter HRV characteristics ([17], [18]).

Studies have found that some of the features derived from the visibility graph analysis of the HRV time series have a weak correlation to the traditional features, both in adults [19] and infants [20], which suggest that these features might add complementary information to HRV analysis. One previous study used network analysis of heart rate and blood pressure as input features, alongside multiscale entropy features and clinical measurements from the patients' electronic medical record, for a machine learning algorithm (MLA) that successfully predicted sepsis in adults, achieving an AUROC of 80% on the test population; an improvement of 7% over the AUROC obtained by their model trained on only the multiscale entropy features and clinical measurements [21].

In this study we aimed to test the diagnostic value of HRV analysis integrating new visibility graph indexes when used in MLAs, in combination with the traditional HRV features, to discriminate between septic and non-septic infants in a selected population of premature infants.

In the following sections, we describe the database used for the study, and the chain of treatment from acquisition of the ECG to computation of the HRV features. We describe the preprocessing of the data and generation of different variants of the feature set, and the MLAs employed to predict sepsis in our population. Finally, we present an evaluation of the machine learning models used on the different variants of the feature set, and then present the results for two sample cases as examples of the differences in HRV between septic and non-

C. León, G. Carrault, P. Pladys, and A. Beuchée are with Univ Rennes, Inserm, LTSI - UMR 1099, F-35000 Rennes, France

ACCEPTED MANUSCRIPT

This article has been accepted for publication in a future issue of this journal, but has not been fully edited. Content may change prior to final publication. Citation information: DOI 10.1109/JBHI.2020.3021662, IEEE Journal of Biomedical and Health Informatics

septic infants, and the predictions made by the best performing MLA. In the last section we discuss these results and compare them with other results reported in the literature.

II. Materials and Methods

A. Population

The data used in this study is part of the database of the Digi-NewB cohort (NCT02863978, EU GA n°689260). The cohort prospectively included preterm infants born before 30 weeks of gestation, hospitalized in the NICU of six university hospitals in the western region of France (University Hospitals of Rennes, Angers, Nantes, Brest, Poitiers, and Tours) in 2017-2019. The collection of the data was carried out after approval by the ethics committee (CPP Ouest 6-598) and informed parental consent. All the patients with available data having received more than five days of antibiotics, beginning at least 72 hours after birth, were included in the LOS group. The control group consisted of infants who did not receive antibiotics after the first three days of life. For this study, we used data coming from 24 infants who developed LOS, and 25 control infants.

The clinical characteristics of the preterm infants studied is presented in Table I. The results are presented as either median and interquartile range (IQR) or as the number of cases and corresponding percentage. Comparisons between the two populations were performed using Mann-Whitney U test or Chi-squared.

	LOS Group (n=24)	Control Group (n=25)	
Gestational age (weeks)	26.5 (25.3-28)	28 (27-28.5)	p <0.01
Birth weight (g)	840 (740-1025)	1107 (925-1260)	p <0.01
Apgar at 1 minute	8 (5-9)	7 (2-8)	NS
Apgar at 5 minutes	9 (8-10)	9 (8-9)	NS
Male/Female	15/9	12/13	NS
Surfactant	17 (71%)	13 (52%)	NS
Twins	5 (21%)	6 (25%)	NS
Premature rupture of membranes >12h	5 (21%)	7 (28%)	NS
Cesarean section	12 (50%)	15 (60%)	NS
Postnatal age at start of antibiotics (days)	8.4 (5.6-10.5)		
Delay between blood culture and start of antibiotics (hours)	1 (1-2.5)		

TABLE I: Characteristics of the study population.

Patients in the LOS group were more premature than in the control group. LOS occurred at 8.4 (5.6-10.5) days after birth, with 17 cases of cocci gram positive bacteria on blood cultures. The LOS group consisted of 17 cases of central line-associated bloodstream infection, five cases of central line-associated infection without positive blood culture, and two clinically suspected infections in patients without central line. The bacteria involved were Staphylococcus Haemolyticus (n=5), Staphylococcus Epidermidis (n=4), Staphylococcus Warnerii (n=3), Staphylococcus Capitis (n=2), Staphylococcus Aureus (n=2) and Enterococcus faecalis (n=1).

B. Proposed approach

The general approach we propose is described in Figure 1. In general terms, we acquired the ECG data from both septic and non-septic patients. This data was processed to detect the R peak and thus obtain the RR time series, which is then segmented in periods of 30 minutes. The HRV features are then extracted from each of these periods.

Afterwards there was a labeling process for each neonate, in which the first hours of measurements were used as a calibration period, to which the measurements from the remaining hours are compared. Then the hours before the beginning of antibiotic therapy, in the case of septic infants, were labeled as infected. For the control population, we randomly selected a time between the third and tenth day of life (corresponding to the time of late onset sepsis diagnosis in the LOS group), and the hours before it were labeled as no-sepsis.

Different methods were then used to select the features to be passed as input to four different MLAs. We used the leave-one-out cross-validation (LOOC) method, leaving one infant out in each iteration, so each infant was at some point the test patient, while the rest formed the training set. We used 8-fold cross validation, grouped by patients, in the training set to optimize the hyperparemeters for each MLA.

The following sections will explain in greater detail each of the steps of our proposed approach.

C. Signal Processing

The ECG from the infants were obtained with a sampling rate of 500Hz. The RR intervals were detected using a modified version of the Pan and Tompkins algorithm, with filter coefficients specifically adapted for newborns, as proposed in [22]. Afterwards, a sliding window of 30 minutes, with no overlap, was applied to calculate the RR series and from it all the HRV parameters that will be described in section II-D.

D. Extraction and Analysis of HRV Parameters

a) Time-Domain Measurements: The time domain parameters calculated for this study were the mean of the RR intervals (meanRR), the standard deviation (sdRR), the root mean square of successive RR intervals (RMSSD), the maximum and minimum value for the RR intervals in the time series (maxRR and minRR, respectively)[23], skewness, kurtosis [24], and AC and DC, which characterize the acceleration and deceleration capacity, respectively, of the heart rate [25].

b) Frequency-Domain Measurements: In the frequency domain we calculated the low frequency power (LF), with limits 0.02-0.2Hz, the high frequency power (HF), with limits 0.2-2Hz. We also calculated these features in normalized units (LFnu and HFnu, respectively), and the LF/HF ratio [23].

c) Non-linear Measurements: The non-linear parameters include the sample entropy (SampEn) and approximate entropy (ApEn), which estimates the level of regularity and predictability of the signal; the coefficients $\alpha 1$ and $\alpha 2$, obtained from the detrended fluctuation analysis of the time series, and which represent, respectively, the short-range and long-range fractal correlations of the signal; and the parameters SD1 and SD2, derived from the Poincaré plot, and which reflect the short and long term variability, respectively [23].

ACCEPTED MANUSCRIPT

This article has been accepted for publication in a future issue of this journal, but has not been fully edited. Content may change prior to final publication. Citation information: DOI 10.1109/JBHI.2020.3021662, IEEE Journal of Biomedical and Health Informatics

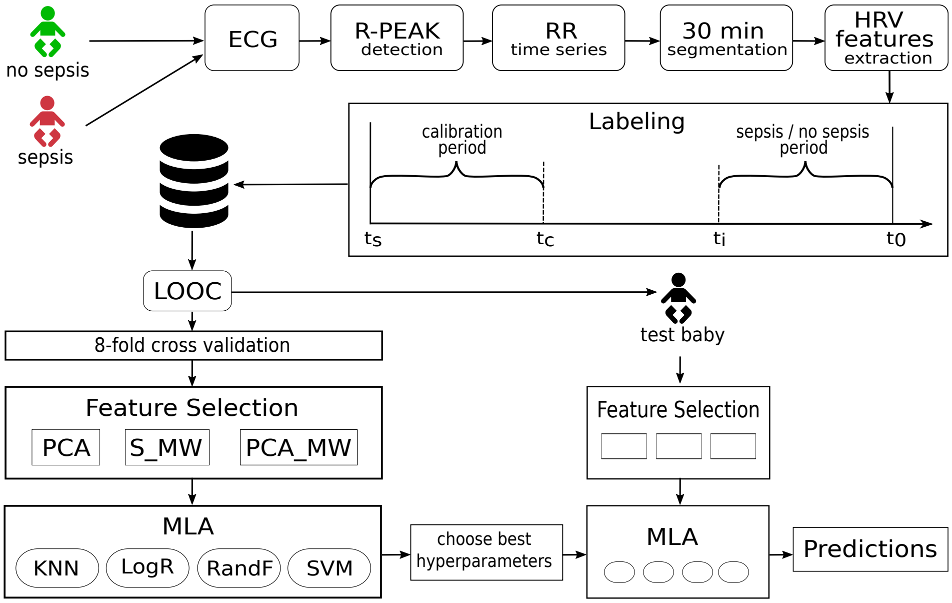

Fig. 1: Proposed approach.

d) Visibility Graph Indexes: The visibility graph (VG) is a network-based time series analysis; it converts the series into a graph that inherits several of its properties, by transforming every data point of the time series into a node. For this we used the visibility graph criterion proposed by Lacasa et al. in [26]. The horizontal visibility graph (HVG) is a subset of the VG, which we calculated using the HVG criterion proposed by Luque et al. in [27].

Several indexes were computed to characterize each graph:

- The mean degree (MD_VG and MD_HVG, respectively) of all the nodes in the graph, where the degree of a node is defined as the number of connected edges of the node. The mean degree is a measure of the complexity of the network [28].
- The cluster coefficient (C_VG and C_HVG, respectively) is the average of the local cluster coefficient of all the nodes in the graph, where the coefficient of a node is a measure of how much its neighbors are also connected to each other, and is defined as the ratio between all triangles involving that node, and the number of connected triples centered on that node [28].
- The transitivity (Tr_VG and Tr_HVG, respectively) is a global version of the cluster coefficient, and is obtained as the ratio between the number of triangles in the graph, and the number of connected triples [28].
- The assortativity (r_VG and r_HVG), which is a correlation between the degrees of all nodes on two opposite ends of an edge, with a graph being assortative if the connected nodes have comparable degree ($r > 0$), and disassortative otherwise ($r < 0$) [29].

The details of how the graphs were constructed and how the indexes were calculated are presented in appendix A.

E. Data Analysis and Machine Learning

To prepare the data for analysis and machine learning, we first excluded all the 30-minute segments with a maxRR greater than one second, or a minRR of less than 0.19 seconds. Afterwards, for the infected infants we selected all the remaining segments prior to the time of administration of antibiotics (t_0); for the infants in the control group we selected a moment at random between the third and tenth day of recording as t_0 (due to the fact that for our LOS group the median value for infection onset is eight days after birth), and selected all the segments prior to that moment. Then, for each infant we calculated the median value of each parameter over a calibration period; we tried three different lengths of calibration period: (i) the first 24 hours of recording, (ii) the first 48 hours of recording, and (iii) the first 72 hours of recording. For the rest of the 30-minute segments of each infant, we subtracted from each feature the median value of that feature obtained over the calibration period. In this way we obtained the calibrated features (Δfeatures). Thus, we generated three variants of the feature set, one for each different length of the calibration period. Additional variants of the feature set were obtained by changing the time we considered as the learning window (t_i): we considered all cases starting from t_i = -72 hours (that is, 72 hours before t_0) [30], until t_i = -6 hours, with six hours increments. For the infected group we labeled the entire duration of the learning window as infected, and for the control group as not infected. Finally, for each variant of the feature set we considered two cases: one including the visibility graph features, and one were they were excluded.

For the statistical analysis and machine learning process we used the LOOC method, leaving one patient out in each iteration. For each variant of the feature set, we used the Mann-Whitney U test to compare the LOS and control population in the training set on each HRV feature, and thus retain only the features that yielded a p-value under 0.1 (MW). Then, we performed principal component analysis (PCA) on the training data with all the features, as well as PCA with only the features with p-value < 0.1 (PCA_MW); in both cases we retained the

ACCEPTED MANUSCRIPT

This article has been accepted for publication in a future issue of this journal, but has not been fully edited. Content may change prior to final publication. Citation information: DOI 10.1109/JBHI.2020.3021662, IEEE Journal of Biomedical and Health Informatics

components for 95% of the variance of the feature set. Thus, we created two different sets of features based on PCA. We created a third set with only the features for which p-value < 0.1, which were standardized (S_MW) before being passed to the MLAs.

We used each of these sets of features to train four different machine learning algorithms: k-nearest neighbors (KNN), logistic regression (LogR), random forest (RandF), and support vector machine (SVM). We used grid search with a per subject 8-fold cross validation split in the training set to find the best hyperparameters specific to each algorithm to maximize recall for each MLA. Finally, each algorithm trained with the best hyperparameters was tested on the patient left out. This process was repeated until every infant in the database had been used as the test subject (the patient left out). The probability curve thus obtained for every patient was then smoothed by calculating for each point in time (equivalent to a 30-minute segment), the probability of infection as the median value between the current predicted probability, and the probability of the two previous segments.

F. Evaluation Method

The evaluation of the performance of each MLA has been done in terms of its area under the receiver operating characteristics curve (AUROC). Our main analysis focused on the performance on the time window comprising the six hours before t_0. However, we were also interested in evaluating the performance during earlier periods of time to determine how early the infection could be detected by the MLAs tested. For this purpose, we evaluated the AUROC on a sliding window with a duration of six hours, starting at the interval between -6h to t_0, and ending at -48h to -42h, sliding with a 50% overlap. For both analyses the AUROC was calculated when combining the predictions made for each patient.

To analyse the value added by the visibility graph indexes to the models, we performed a likelihood ratio test ([31], [32]) on the best performing model, to compare its performance when the visibility graph indexes are included in the feature set to when they are not included. With this test, a p-value under 0.05 means that the information added to the model by the new features causes a statistically significant improvement on the model.

For the purpose of analysis and visualization of the predictions given by a particular MLA, trained on a given variant of the feature set, and for a specific patient, we compared the predicted probability of infection to a fixed threshold of 0.5 for all MLAs, although this might not be the optimal threshold for that MLA. We chose this method to simplify the comparison between the results from different MLAs, by comparing them all based on a set threshold. Thus we considered a false negative as a probability lower than 0.5 for an infected infant, and a false positive as a probability greater than 0.5 for a control patient.

III. Results

In this section we first present the behavior of certain measurements of the HRV analysis in the whole population. Afterwards we report the predictive performance of the MLAs with the different variants of the feature set. For simplicity, we only present the best results obtained. Next, we present the HRV measurements which showed statistically significant differences between LOS and control group, and then we analyze the effect of varying the calibration and learning windows on the predictive performance of the algorithm. Subsequently we consider the effect of including the visibility graph indexes. Finally we present the results for two patients of our population, one from the control group and one from the LOS group, as sample cases and to exemplify how our method could be used for monitoring in the NICU.

A. General Behaviour of Some HRV Parameters

In Figure 2 we present the comparison between the median values of the 24 infected infants (in red) and the 25 control infants (in blue) for some of the calibrated HRV parameters (Δfeatures), for which a difference between both groups was easily observable. The green line represents the time t_0. The features shown in the figure were calculated using a calibration period of 48 hours.

Figure 2a shows the median value of the ΔminRR for the control and sepsis groups, and it can be observed that the value is generally higher for the LOS group, which is consistent with an expected increase of the occurrence of bradycardia in the infected patients. In Figure 2b we observe that the ΔLFnu is generally lower in the infected population in the days around the t_0, as compared to the control group; this could reflect alterations in baroreflex induced changes in HRV during sepsis. The ΔSD2 is also generally lower in the infected group, as observed in Figure 2c, which correlates to the decreased HRV associated with sepsis. Similarly, Figure 2d also shows evidence of a decrease of the ΔMD_VG in the infected infants, which also signals decreased HRV in this group.

B. Predictive Performance of the MLAs

We evaluated the predictive performance of all the MLAs using every variant of the feature set, created as explained in section II-E, for a time window comprising the six hours before t_0. These results are presented in Table II, where we show the variant of the feature set that gave the best result, in terms of greatest AUROC, for each of the four types of MLAs we tested. The left side of the table shows the results when the visibility graph indexes were included in the feature set, while the right side displays the results obtained when these features were excluded. The *Features* columns specify which feature selection technique was used to construct the feature set (PCA, S_MW, or PCA_MW); the *Calib.* column indicates how many hours were used for the *calibration period*; the t_i column shows how many hours before t_0 were used for the training window. Finally, in the *AUROC* column we present the AUROC, and its corresponding 95% confidence interval (CI), achieved by the algorithm using the given variant of the feature set on both the training and testing data.

In Table II we observe that the best performance for detecting whether a test patient is infected or not during the

ACCEPTED MANUSCRIPT

This article has been accepted for publication in a future issue of this journal, but has not been fully edited. Content may change prior to final publication. Citation information: DOI 10.1109/JBHI.2020.3021662, IEEE Journal of Biomedical and Health Informatics

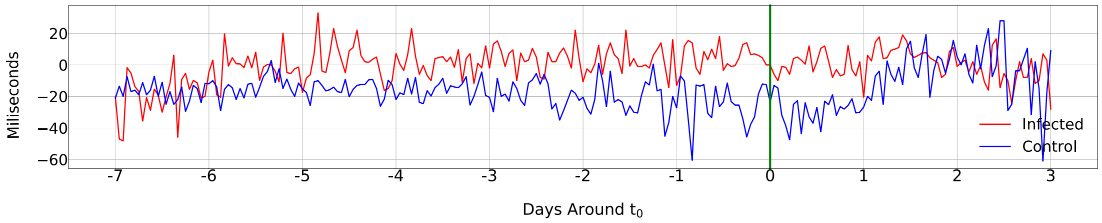

(a) ΔminRR.

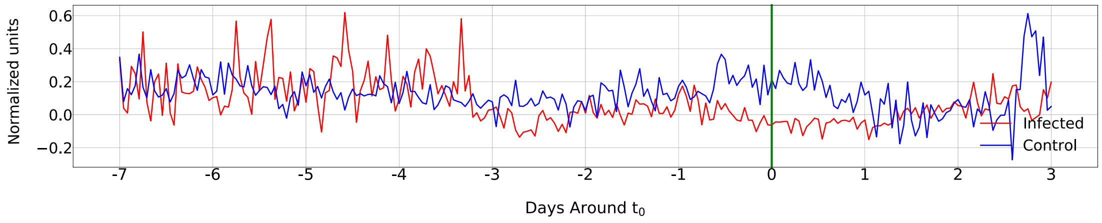

(b) ΔLFnu.

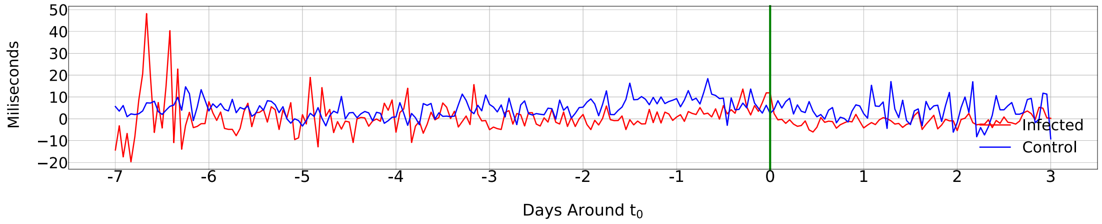

(c) ΔSD2.

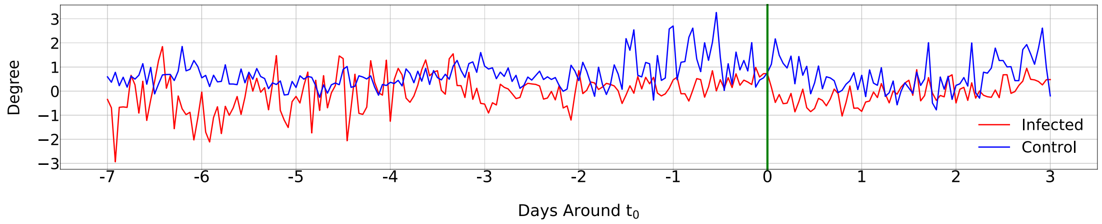

(d) ΔMD_VG.

Fig. 2: Median value of the Δfeatures over several days.

**MLA**	**Including Visibility Graph Features**					**Excluding Visibility Graph Features**				
	Feature Set Variant			AUROC (% [95% CI])		Feature Set Variant			AUROC (% [95% CI])	
	Features	Calib.	t_i	Training	Testing	Features	Calib.	t_i	Training	Testing
KNN	S_MW	48h	-30h	89 [88.7, 89.3]	77.7 [73.1, 82.3]	PCA	48h	-24h	87.9 [87.5, 88.3]	73.2 [66.9, 79.5]
LogR	PCA_MW	48h	-42h	88.4 [87.9, 88.9]	87.7 [83.3, 92.2]	PCA_MW	48h	-42h	82.2 [81.7, 82.7]	80.9 [75.9, 85.9]
RandF	PCA_MW	48h	-6h	99 [98.6, 99.4]	81 [75.7, 86.3]	PCA_MW	48h	-6h	98.4 [97.8, 99]	73 [66.4, 79.6]
SVM	PCA	72h	-12h	91.5 [90.4, 92.6]	78.3 [72.1, 84.5]	PCA	72h	-6h	92.6 [91.7, 93.3]	82.3 [77.1, 87.5]

TABLE II: Best feature set variants for the six hours evaluation window and their respective AUROC. AUROC are presented as median value and 95% confidence interval.

six hours before t_0 was obtained by the LogR algorithm, on the feature set that included the visibility graph indexes, with 87.7% AUROC on the testing data. All the MLAs performed better when the visibility graph indexes were included in the feature set, except the SVM which performed better without the visibility graph indexes. Similarly, all MLAs performed better when the calibration period used was of 48 hours, except the SVM which performed better with a calibration period of 72 hours. This might be due to a more robust calibration when using a 72h period, making it less sensitive to the heart rate variability changes normally associated with the first hours of life in neonates [33]; SVM might benefit from this more than the other MLAs given its sensitivity to any noise or outliers in the training data [34].

Regarding the feature selection, LogR and RandF performed better when trained with the PCA_MW features, while KNN performed better on the set of S_MW features, and SVM had a better performance on the PCA of all the features. Finally, the best training window for KNN was 30 hours before t_0, for LogR it was 42 hours before t_0, while for RandF and SVM the best training window was when t_i = -6 hours.

We also observe that, as expected, the AUROCs obtained from the predictions for the training data are bigger than those obtained from the predictions for the testing data. For the

ACCEPTED MANUSCRIPT

This article has been accepted for publication in a future issue of this journal, but has not been fully edited. Content may change prior to final publication. Citation information: DOI 10.1109/JBHI.2020.3021662, IEEE Journal of Biomedical and Health Informatics

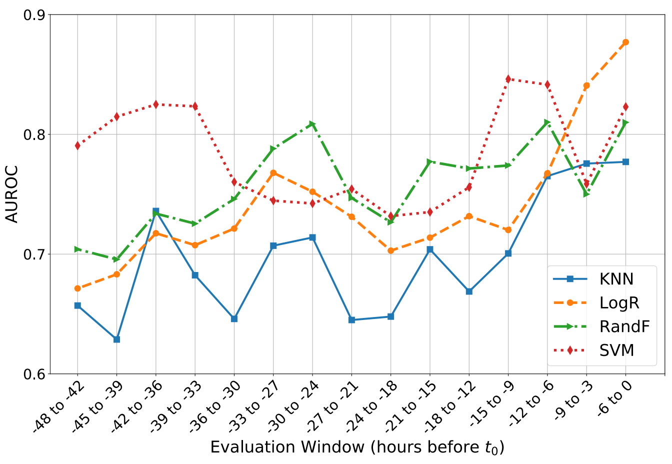

Fig. 3: Progress of the AUROC for the best performing MLAs configurations evaluated on a sliding time window.

KNN, RandF, and SVM the difference between training and testing AUROC ranges from 11.3% to 25.4%. Instead, for the LogR the difference between the training and testing AUROCs is smaller, at 0.7% when visibility graph features are included, and 1.3% when they are not. This suggests that the main reason why the LogR, despite being one of the simplest of the MLAs we tested, outperforms more powerful algorithms such as RandF and SVM, is because it is not over fitting on the training data, while the other algorithms are.

In Figure 3 we show how the AUROC from the testing data changes for each algorithm when evaluated on a sliding window of six hours, with a 3-hour overlap, between t_0 and t_0 = -48 hours. For this, each algorithm was evaluated using its optimal variant of the feature set, as presented in Table II. We observe that all algorithms have an AUROC above 60% for all time windows. Furthermore, LogR, RandF, and SVM have an AUROC above 70% since at least 42 hours before t_0. In general, their AUROCs begin rising at 24 hours before t_0 and until t_0, with LogR, RandF, and SVM ending with AUROCs of over 80%. We observe that the AUROC for all algorithms present some oscillations over time, with the LogR being the most stable of the four. This suggests that the oscillations might be due to the overfitting on the training data which, as observed in Table II, was more marked in the other three MLAs, and overfitting can cause small changes in the test data to translate into significant changes in the predictions made by the models.

Given that, when visibility graph indexes were excluded, the LogR model had a very similar performance to the SVM, which was the best performing MLA in this case, with a difference in AUROC of only 1.4%, and similar confidence intervals, compounded with the fact that LogR is a simpler algorithm, faster to train than the others, less prone to overfitting, and that it outperformed all other algorithms when the visibility graph features were included, from this point on we focus our results and analysis on the results obtained with the LogR algorithm, on the feature set variant as presented in Table II, both when including and excluding the visibility graph features.

HRV Feature	Occurrence	HRV Feature	Occurrence
meanRR	100%	HFnu	100%
sdRR	96.4%	LF/HF	100%
RMSSD	100%	SD1	100%
maxRR	98.2%	SD2	96.4%
minRR	100%	SampEn	100%
Skewness	100%	ApEn	94.6%
Kurtosis	100%	$\alpha 1$	73.2%
AC	98.2%	$\alpha 2$	100%
DC	98.2%	MD_VG	100%
LF	100%	Tr_VG	100%
HF	98.2%	r_VG	100%
LFnu	100%	MD_HVG	100%

TABLE III: HRV measurements with statistically significant differences (p-value < 0.1) between control and infected population. The column Occurrence provides the percentage of LOOC iterations for which the feature is significant.

C. Feature Selection

For every variant of the feature set, we also implemented the Mann-Whitney U test to compare the HRV calibrated features (Δfeatures) of the control group to those of the infected group. We applied this to the training data in every iteration of the LOOC procedure. For simplicity, in this section we present the measurements that had p-value < 0.1, when the calibration period was set at 48h, and the and learning window at -42h, as presented in Table II for the best performing MLA, LogR. In Table III we present the HRV features which had statistically significant differences (p-value < 0.1) between LOS and control group in at least 50% of the LOOC iterations for said configuration. The column *Occurrence* shows the percentage of the LOOC iterations for which the given measurement showed statistically significant differences.

We observe that out of 28 HRV measurements considered, 24 were relevant (p-value < 0.1) in at least 50% of the cases, when using a calibration window of 48h and a learning window of -42h. Particularly, from the visibility graph features, four were relevant in 100% of the iterations: *MD_VG*, *Tr_VG*, *r_VG*, and *MD_HVG*.

D. Optimization of the Calibration Period and Learning Window

To analyze the effect of varying the calibration window and learning hours, we evaluated the AUROC for each possible combination when evaluated in the period of six hours before t_0.

In Figure 4 we present the results for the LogR algorithm, using the PCA_MW method for feature selection for the training and testing data. We observe that the best results were mostly obtained when using the 48 hours calibration period, with the minimum AUROC for that case being of 84.8%. Using 72 hours of calibration also gave good results; the best AUROC for this case was obtained when using -42 hours in the learning window, at 86.7%, only 1% below the best AUROC for this algorithm, which was also obtained with t_i = -42h, but calibration hours set to 48. Using the calibration period of 24 hours resulted in the lowest AUROCs for the LogR. The lowest AUROC for this MLA was of 76.4%, obtained when

ACCEPTED MANUSCRIPT

This article has been accepted for publication in a future issue of this journal, but has not been fully edited. Content may change prior to final publication. Citation information: DOI 10.1109/JBHI.2020.3021662, IEEE Journal of Biomedical and Health Informatics

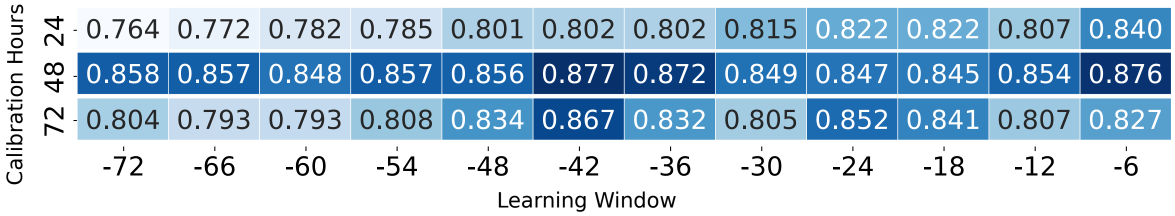

Fig. 4: Variations of the AUROC of the LogR as the calibration hours and learning windows change.

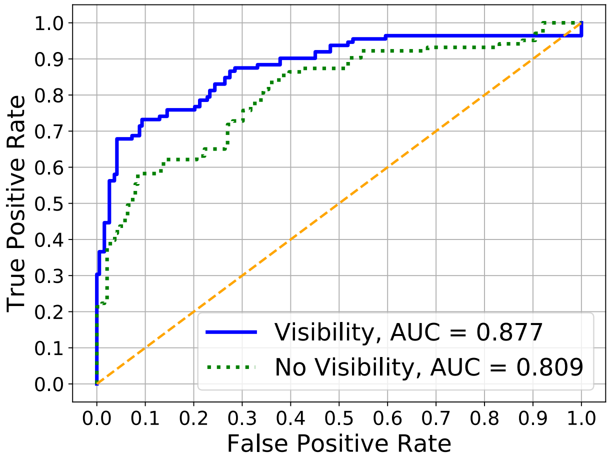

Fig. 5: Best predictive performance with and without visibility graph indexes six hours before administration of antibiotics.

using 24 hours for calibration and -72 hours for the learning window.

E. Effect of Visibility Graph Indexes

As it was shown in Table II, the MLA with the best predictive performance for the six hours before the infection was the LogR, using a calibration period of 48 hours and the time for the onset of the infection set at 42 hours before the administration of antibiotics, using as input features the principal components of the features with p-value < 0.1 (PCA_MW), and including the visibility graph indexes in the feature set. This setting obtained an AUROC of 87.7%, which is presented as the blue solid line (Visibility) in Figure 5.

When excluding the visibility graph features, the LogR model, using the same calibration period, learning window, and feature selection method as before, obtained an AUROC of 80.9%, which is presented as the green dotted line (No Visibility) in Figure 5.

Thus introducing the visibility graph indexes in the feature set increased the performance of the MLA by 6.8%. Furthermore, we performed a likelihood ratio test to determine if the improvement obtained by the inclusion of the the visibility graph indexes to the feature set is statistically significant, obtaining a p-value of $3.5\mathrm{e}{-6}$ in the training set, and $2.9\mathrm{e}{-4}$ in the testing set. This indicates that the information added by the visibility graph leads to an statistically significant improvement in the fit of the model.

F. Sample Cases

The two cases presented in this section illustrate the results obtained for two patients: one from the LOS group and one from the control group. The patient from the control group was a very preterm male, born at 27 weeks gestation, with birth weight of 1220g and Apgar score of 2/8, and received 48 hours of antibiotics at birth for an unconfirmed suspicion of early onset sepsis. He was treated with caffeine and nasal continuous positive ventilation with 23% oxygen. He did not develop any infection during his stay in neonatology.

The patient from the LOS group developed LOS with positive blood culture which identified an Enterococcus Faecalis on the 14th day of life. This patient was also very preterm male, born at 25 weeks of gestation, with birth weight of 730g and Apgar score of 5/7. The patient received 48 hours of antibiotics at birth for an unconfirmed suspicion of early onset sepsis. He was treated with caffeine and nasal intermittent positive ventilation with 28% of oxygen, and fed through a venous central line. At the time of clinical suspicion of LOS an isolated increase in cardio-respiratory events was observed without other clinical signs. The results below show that the proposed method would have been able to diagnose the emerging infection at least 12 hours before the clinical suspicion.

In Figure 6a we observe the RR time series from the LOS patient, corresponding to a period of 30 minutes, three hours before the beginning of administration of antibiotics. In Figure 6b we present the RR time series of a 30-minute segment from the control patient. In this figure a difference we observe that the infected patient displays less variability in its heart rate in comparison with the patient from the control group.

In Figure 7 we present the visibility graph obtained from the RR time series presented in Figure 6. To facilitate the visual interpretation of the graph, for both patients we have zoomed into a window of only 300 beats of the time series, which is shown in the left side of the figures; we have also highlighted some interesting beats and their respective nodes in the visibility graph: in green, red, and magenta we highlight local maxima of the RR time series, and in grey local minima. We observe that the beats that are local minima in the time series, in the visibility graph convert into nodes that have very few connections and that are in the outer part of the clusters. On the other hand, the nodes that are local maxima convert into nodes that connect different clusters. In Figure 7a we observe that the low heart rate variability of the infected baby translates into a visibility graph with less connections within clusters. In comparison, in Figure 7b we observe that for the non-septic patient, the connections within each cluster are denser.

ACCEPTED MANUSCRIPT

This article has been accepted for publication in a future issue of this journal, but has not been fully edited. Content may change prior to final publication. Citation information: DOI 10.1109/JBHI.2020.3021662, IEEE Journal of Biomedical and Health Informatics

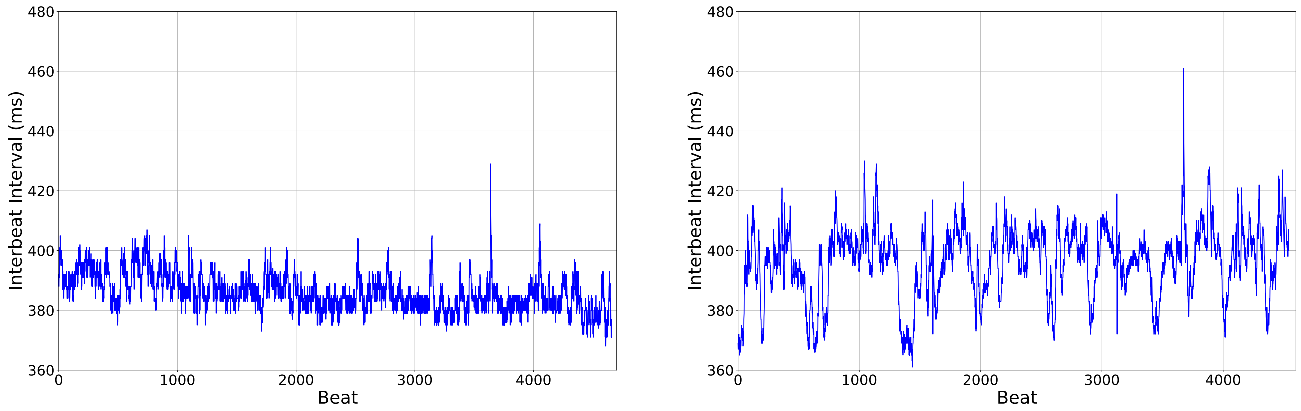

(a) Sepsis patient.

(b) Non-sepsis patient.

Fig. 6: RR time series for 30-minute segments observed three hours before t_0.

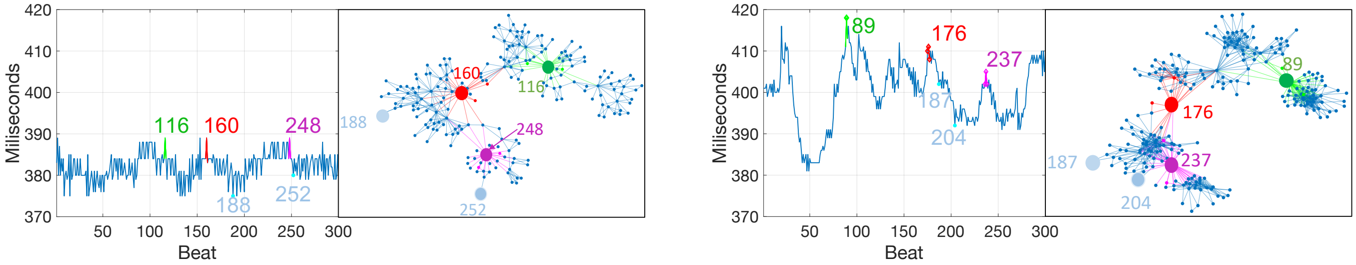

(a) Sepsis patient.

(b) Non-sepsis patient.

Fig. 7: RR time series and its corresponding visibility graph

The HRV measurements of these patients were calculated and calibrated. Thus, we obtained the features that would be used by the MLA, some of which are shown in Figure 8, where we compare the Δfeatures for the same non-septic (blue solid line) and septic (red dashed line) patients shown in Figure 6, during the 12 hours before (t_0). Differences in the HRV of the non-septic and LOS patient can be observed in the four different types of measurements we considered: time-domain (exemplified by minRR in Figure 8a), frequency-domain (LFnu in Figure 8b), non-linear measurements (SD2 in Figure 8c), and visibility graph indexes (MD_VG in Figure 8d). It's important to note that the Δfeatures shown in Figure 8 are the same as those shown in Figure 2, and that the features for these two sample patients follow the same tendency observed when comparing the entire LOS group to the control group.

Finally, the predictions of the probability of infection over time for both sample patients were calculated using the LogR model, with the configuration which yielded the best results in the six hours before t_0, and including and excluding the visibility graph indexes, as presented in section III-B. In Figure 9 we present the predicted probability during the 24 hours before t_0, and highlight in yellow the period corresponding to the six hours before t_0.

The results for the infected infant are presented in Figure 9a, with the top row showing the predicted probability (in blue) when the visibility graph features were included, and the bottom row presenting the predicted probability (in blue) when these features were excluded from the feature set. The black dotted line represents the threshold probability of 0.5. In the case where the visibility graph indexes are included, observe that while the probability gets very close to the threshold in the period between -24h and -22h, it never actually crosses the line, so there are no false negatives. On the other hand, when these features are excluded there is a false negative in the period between -24h and -22h.

In the case of the patient from the control group, presented in Figure 9b, when visibility graph indexes were excluded there were false positives in predicted probability of infection (bottom figure), which was not the case when these features were included in the feature set (top figure).

IV. Discussion

This study proposes a method for estimating the probability of LOS in premature neonates using MLAs, with the aim to aid an earlier and more accurate diagnosis. Our proposed method is based on extracting HRV features from the continuous heart rate (HR) monitoring, and then using those features as input for the MLA. For this we use the traditional HRV features: time-domain, frequency-domain, and non-linear measurements. However, we also propose the inclusion of more novel measurements based on visibility graph indexes.

Previous studies have demonstrated that MLAs can detect sepsis in both adults and infants. Unlike the method we

ACCEPTED MANUSCRIPT

This article has been accepted for publication in a future issue of this journal, but has not been fully edited. Content may change prior to final publication. Citation information: DOI 10.1109/JBHI.2020.3021662, IEEE Journal of Biomedical and Health Informatics

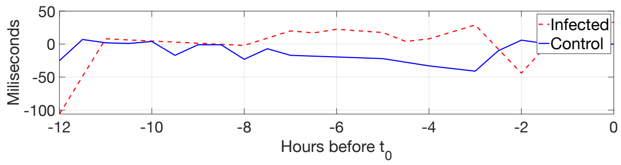

(a) ΔminRR.

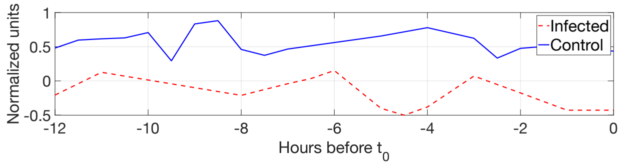

(b) ΔLFnu.

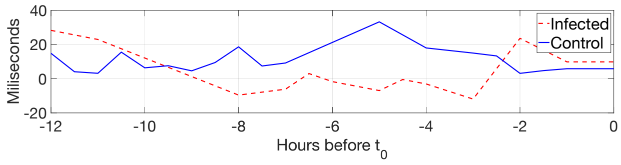

(c) ΔSD2.

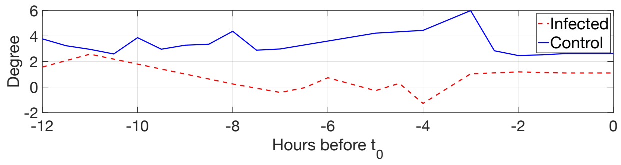

(d) ΔMD_VG.

Fig. 8: Calibrated Features for Sample Cases.

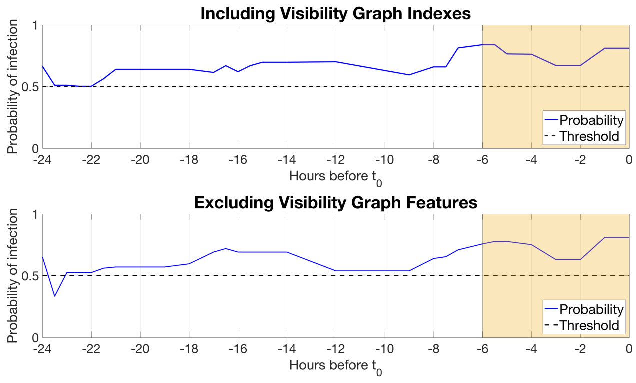

(a) Septic patient.

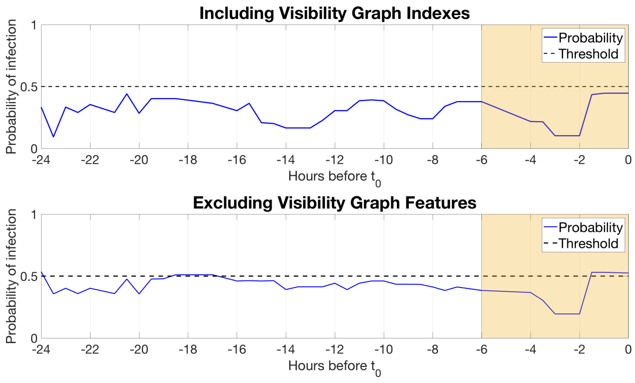

(b) Non-septic patient.

Fig. 9: Predictions six hours before t_0.

have proposed here, most of these studies rely not only on HR measurements, but may also include respiratory rate, blood pressure, motion, clinical signs, and laboratory tests ([30], [35]–[41]). And among those studies that have focused exclusively on HR or HRV measurements ([11]–[13]), we did not find any study that included visibility graph indexes or network-based analysis. In fact, we could find only one previous study that used visibility graph indexes of HR and blood pressure to diagnose sepsis in adults using MLA, which found a improvement of 7% in the AUROC when these measurements were included [21]. But to our knowledge, no previous study has used visibility graph indexes for diagnosis of sepsis on premature infants.

Thus, one of our main findings was the contribution of visibility graph features to the performance of the MLAs. We found that the AUROC of the best performing MLA improved by 6.8% when the visibility graph indexes were included. The likelihood ratio suggests that the improvement introduced by these features is statistically significant, with p-value $< 3e{-4}$.

Another important aspect of our method is that we use the median value of HRV features for each individual patient during a calibration period as a baseline reference for that patient, and it is the difference between this reference value and the value measured for the following segments what is actually passed as input to the MLA. A similar approach has been proposed before, both in adults, where one study reports using the mean value of the HRV metrics over the first 24 hours of recording as reference value [42], and in premature infants, where another study used a calibration period of 72 hours to predict sepsis based on HR, respiratory rate, and clinical signs [30], however this study does not specify how the calibration was performed. But this type of method might be specially useful in the case of evaluating HRV in premature infants, for different studies have shown that differences in gestational age can imply significant differences in the HRV of the infant. In our study the best results were obtained when using the first 48 hours of recording as the calibration period.

In regards to the different MLAs evaluated, we found that LogR had a better performance than the others, even though it was also the simplest one used. This might be due to the fact that we did not have a very large population, and precisely due to its simplicity, LogR is less likely to overfit on the training set. This is supported by that fact that all the other MLAs had a significantly bigger AUROC on the training data than on the testing data, while this difference was very small with the LogR. However, with a larger population better results could possibly be obtained using more complex MLAs.

The fact that best performance was achieved when training

ACCEPTED MANUSCRIPT

This article has been accepted for publication in a future issue of this journal, but has not been fully edited. Content may change prior to final publication. Citation information: DOI 10.1109/JBHI.2020.3021662, IEEE Journal of Biomedical and Health Informatics

the MLA using the 42 hours before t_0, for both infected and control patients, might be due to this yielding a bigger dataset for the training of the MLA.

Concerning the preprocessing for feature selection, we obtained the best results when choosing the measurements for which the comparison between LOS and control population had p-value under 0.1, and then performing a PCA on those, finally passing the components that represented 95% of the variance as input for the MLA. This might be explained by the fact that different relevant HRV metrics might reflect the same underlying information, and PCA helps to reduce this information into fewer features.

The method we propose could be deployed in real time in a NICU setting, updating the probability of late onset sepsis every half hour, based exclusively on the heart rate of the patient. Although this method would require a 48h observation period before the first prediction is made, in order to calibrate the system for the individual infant, this is not an impediment given that late onset sepsis is defined as sepsis occurring after the first 72h of life.

V. Conclusion and Future Work

Based on our findings, we propose a method for LOS diagnosis in premature neonates using MLA based on HRV. Our first recommendation is to include visibility graph indexes, which are a novel method for HRV analysis, alongside the traditional metrics for HRV, to construct the feature set. Likewise, we recommend using a calibration period of 48 hours, proposing the median value over this time as the baseline for each individual infant, and then measuring the variation in regards to this value. For training the MLA we recommend using the period of 42 hours before the beginning antibiotic treatment in the case of the infected population, and continuous periods of equal duration in the control population. For feature selection we recommend performing PCA over the features with p-value under 0.1 when comparing the measurements for sepsis versus non-sepsis population. Finally, for studies with a small population we recommend using logistic regression for making the predictions.

Since this study was performed, more LOS and control infants have been included in the protocol. In the future we would like to carry out a study using some of the new patients to expand our training set, and the others patients exclusively as validation population. Finally, we would also like to evaluate the adaptability of our proposed approach to real-time monitoring in a NICU environment.

Appendix A
Construction of the Visibility Graphs and Calculation of their Indexes

To construct the visibility graph (VG) every data point of the RR time series is transformed into a node, and the connectivity between nodes is defined with the visibility criterion proposed by Lacasa et al. [26]. By this criterion, two data values of the time series (t_a, y_a) and (t_z, y_z) have visibility, and therefore are connected, if any other data point (t_i, y_i) placed between them, so that $t_a < t_i < t_z$ fulfill the following condition:

$$y_i < y_z + (y_a - y_z)\frac{t_z - t_i}{t_z - t_a}$$

The horizontal visibility graph (HVG) is a subset of the VG, in which the connectivity between nodes is defined by the criterion proposed by Luque et al. [27], by which (t_a, y_a) and (t_z, y_z) have visibility, and therefore are connected, if:

$$\forall t_i \in t_a, t_z : y_a > y_i \text{ and } y_z > y_i$$

We then calculated four indices from the VG and HVG thus obtained, in order to give a numerical characterization of their properties.

a) Mean Degree (MD): The degree of a node if the number of connections (or edges) it has. The mean degree of the graph is then calculated as:

$$\text{MD} = \frac{1}{N}\sum_{n=1}^{N} d_n$$

Where N is the total number of nodes in the graph, and d_n is the degree of node n [28].

b) Cluster Coefficient (C): This index quantifies how connected the neighbours of a node are. The local cluster coefficient of node y_n, c_n, is given by:

$$c_n = \frac{\text{number of triangles connected to } y_n}{\text{number of connected triples centered on } y_n}$$

Where a triangle corresponds to three nodes that are connected to each other, and a connected triple is a set of three nodes which can be reached from each other. In other words, a connected triple is equivalent to a path formed by two edges, and in this with node y_n as the central node.

Finally, the cluster coefficient C is calculated as the average of all the local cluster coefficients of all the nodes in the graph [28]:

$$\text{C} = \frac{1}{N}\sum_{n=1}^{N} c_n$$

c) Transitivity (Tr): The transitivity index also measures the density triangles in the graph, and is a global version of the cluster coefficient. It is calculated as:

$$\text{Tr} = \frac{\text{3 x number of triangles in the graph}}{\text{number of connected triples in the graph}}$$

The factor three assures that assures that $0 \leq C \leq 1$, given the fact that each triangle can be seen as three different connected triples, one with each of the data points as the central nodes [28].

d) Assortativity (r): Assortativity is a correlation coefficient between the degrees of the nodes on opposite ends of an edge. It is calculated as:

$$\text{r} = \frac{\frac{1}{N}\sum_{n=1}^{N} j_n k_n - [\frac{1}{N}\sum_{n=1}^{N}\frac{1}{2}(j_n + k_n)]^2}{\frac{1}{N}\sum_{n=1}^{N}\frac{1}{2}(j_n^2 + k_n^2) - [\frac{1}{N}\sum_{n=1}^{N}\frac{1}{2}(j_n + k_n)]^2}$$

ACCEPTED MANUSCRIPT

This article has been accepted for publication in a future issue of this journal, but has not been fully edited. Content may change prior to final publication. Citation information: DOI 10.1109/JBHI.2020.3021662, IEEE Journal of Biomedical and Health Informatics

Where j_n and k_n are the degrees of the nodes at each end of the nth edge, and N is the total number of edges in the graph.

A network is assortative if the connected nodes have comparable degree ($r > 0$), otherwise the network is disassortative ($r < 0$) [29].

Acknowledgment

This study received funding from the European Union's Horizon 2020 research and innovation program under grant agreement No. 689260 (Digi-NewB project).

References

[1] L. Liu, H. L. Johnson, S. Cousens, J. Perin, J. E. L. Susana Scott, I. Rudan, H. Campbell, R. Cibulskis, M. Li, R. E. B. Colin Mathers, for the Child Health Epidemiology Reference Group of WHO, and UNICEF, "Global, regional, and national causes of child mortality: an updated systematic analysis for 2010 with time trends since 2000," *The Lancet*, vol. 379, no. 9832, pp. 2151–2161, Jun. 2012.

[2] B. J. Stoll, N. Hansen, A. A. Fanaroff, L. L. Wright, W. A. Carlo, R. A. Ehrenkranz, J. A. Lemons, E. F. Donovan, J. E. T. Ann R. Stark, W. Oh., C. R. Bauer, S. B. Korones, S. Shankaran, A. R. Laptook, D. K. Stevenson, L.-A. Papile, and W. K. Poole, "Late-Onset Sepsis in Very Low Birth Weight Neonates: The Experience of the NICHD Neonatal Research Network," *Pediatrics*, vol. 110, no. 2, pp. 285–291, Aug. 2002.

[3] A. Kumar, D. Roberts, K. E. D. Wood, B. Light, J. E. Parrillo, S. Sharma, R. Suppes, D. Feinstein, S. Zanotti, L. Taiberg, D. Gurka, A. Kumar, and M. Cheang, "Duration of hypotension before initiation of effective antimicrobial therapy is the critical determinant of survival in human septic shock," *Critical Care Medicine*, vol. 34, no. 6, pp. 1589–1596, Jun. 2006.

[4] H Nguyen, S. Corbett, R. Steele, J. Banta, R. Clark, S. Hayes, J. Edwards, T. Cho, and W. Wittlake, "Implementation of a bundle of quality indicators for the early management of severe sepsis and septic shock is associated with decreased mortality," *Critical Care Medicine*, vol. 35, no. 4, pp. 1105–1112, Apr. 2007.

[5] V. S. Kuppala, J. Meinzen-Derr, A. L. Morrow, and K. R. Schibler, "Prolonged Initial Empirical Antibiotic Treatment is Associated with Adverse Outcomes in Premature Infants," *The Journal of Pediatrics*, vol. 159, no. 5, pp. 720–725, Nov. 2011.

[6] W. H. Organization. (2018). Antimicrobial resistance, [Online]. Available: https://www.who.int/en/news-room/fact-sheets/detail/antimicrobial-resistance (visited on 04/11/2019).

[7] J. Alverdy and M. Krezalek, "Collapse of the Microbiome, Emergence of the Pathobiome, and the Immunopathology of Sepsis," *Critical Care Medicine*, vol. 45, no. 2, 337–347, Feb. 2017.

[8] R. Singh, L. Sripada, and R. Singh, "Side effects of antibiotics during bacterial infection: Mitochondria, the main target in host cell," *Critical Care Medicine*, vol. 16, pp. 50–54, May 2014.

[9] A. Malik, C. P. S. Hui, R. A. Pennie, and H. Kirpalani, "Beyond the Complete Blood Cell Count and C-Reactive Protein," *Archives of Pediatrics & Adolescent Medicine*, vol. 157, no. 6, pp. 511–516, Jun. 2003.

[10] K. D.Fairchild and M. O'Shea, "Heart Rate Characteristics: Physiomarkers for Detection of Late-Onset Neonatal Sepsis," *Clinics in Perinatology*, vol. 37, no. 3, pp. 581–598, Sep. 2010.

[11] C. J. Chiew, N. Liu, T. Tagami, T. H. Wong, Z. X. Koh, and M. E. H. Ong, "Heart rate variability based machine learning models for risk prediction of suspected sepsis patients in the emergency department," *Medicine*, vol. 98, no. 6, e14197, Feb. 2019.

[12] J. Moorman, D. Lake, and M. Griffin, "Heart Rate Characteristics Monitoring for Neonatal Sepsis," *IEEE Transactions on Biomedical Engineering*, vol. 53, no. 1, pp. 126–132, Jan. 2006.

[13] S. Ahmad, A. Tejuja, K. D. Newman, R. Zarychanski, and A. J. Seely, "Clinical review: A review and analysis of heart rate variability and the diagnosis and prognosis of infection," *Critical Care*, vol. 13, no. 6, p. 232, Nov. 2009.

[14] W.-L. Chen, J.-H. Chen, C.-C. Huang, C.-D. Kuo, C.-I. Huang, and L.-S. Lee, "Heart rate variability measures as predictors of in-hospital mortality in ED patients with sepsis," *The American Journal of Emergency Medicine*, vol. 26, no. 4, pp. 395–401, May 2008.

[15] N. Marwan, N. Wessel, H. Stepan, and J. Kurths, "Recurrence based complex network analysis of cardiovascular variability data to predict pre-eclampsia," in *Proceedings of Biosignal 2010*, Jul. 2010.

[16] X. Sun, Y. Zhao, and X. Xue, "Analyzing spatial characters of the ecg signal via complex network method," in *2011 4th International Conference on Biomedical Engineering and Informatics (BMEI)*, vol. 3, Oct. 2011, pp. 1650–1653.

[17] Z.-G. Shao, "Network analysis of human heartbeat dynamics," *Applied Physics Letters*, vol. 96, no. 7, p. 073 703, 2010.

[18] X. Li and Z. Dong, "Detection and prediction of the onset of human ventricular fibrillation: An approach based on complex network theory," *Physical Review E*, vol. 84, p. 062 901, 6 Dec. 2011.

[19] T. Madl, "Network analysis of heart beat intervals using horizontal visibility graphs," *2016 Computing in Cardiology Conference (CinC)*, pp. 733–736, 2016.

[20] T. Nguyen Phuc Thu, A. I. Hernández, N. Costet, H. Patural, V. Pichot, G. Carrault, and A. Beuchée, "Improving methodology in heart rate variability analysis for the premature infants: Impact of the time length," *PLOS ONE*, vol. 14, no. 8, pp. 1–14, Aug. 2019.

[21] S. P. Shashikumar, Q. Li, G. D. Clifford, and S. Nemati, "Multiscale Network Representation of Physiological Time Series for Early Prediction of Sepsis," *Physiologi-*

ACCEPTED MANUSCRIPT

This article has been accepted for publication in a future issue of this journal, but has not been fully edited. Content may change prior to final publication. Citation information: DOI 10.1109/JBHI.2020.3021662, IEEE Journal of Biomedical and Health Informatics

cal Measurement, vol. 38, no. 12, pp. 2235–2248, Nov. 2017.

[22] M. Doyen, D. Ge, A. Beuchée, G. Carrault, and A. I. Hernández, "Robust, real-time generic detector based on a multi-feature probabilistic method," *PLOS ONE*, vol. 14, no. 10, pp. 1–22, Oct. 2019.

[23] F. Shaffer and J. P. Ginsberg, "An Overview of Heart Rate Variability Metrics and Norms," *Frontiers in Public Health*, vol. 5, p. 258, 2017.

[24] M. Bolanos, H. Nazeran, and E. Haltiwanger, "Comparison of heart rate variability signal features derived from electrocardiography and photoplethysmography in healthy individuals," in *2006 International Conference of the IEEE Engineering in Medicine and Biology Society*, Aug. 2006, pp. 4289–4294.

[25] A. Bauer, J. W. Kantelhardt, P. Barthel, R. Schneider, T. Mäkikallio, K. Ulm, K. Hnatkova, A. Schömig, H. Huikuri, A. Bunde, M. Malik, and G. Schmidt, "Deceleration capacity of heart rate as a predictor of mortality after myocardial infarction: cohort study," *The Lancet*, vol. 367, no. 9523, pp. 1674–1681, May 2006.

[26] L. Lacasa, B. Luque, J. Luque, and J. C. Nuño, "The visibility graph: A new method for estimating the Hurst exponent of fractional Brownian motion," *EPL (Europhysics Letters)*, vol. 86, no. 3, p. 30 001, May 2009.

[27] B. Luque, L. Lacasa, F. Ballesteros, and J. Luque, "Horizontal visibility graphs: Exact results for random time series," *Physical Review E*, vol. 80, no. 4, p. 046 103, Oct. 2009.

[28] L. da F. Costa, F. A. Rodrigues, G. Travieso, and P. R. V. Boas, "Characterization of complex networks: A survey of measurements," *Advances in Physics*, vol. 56, no. 1, pp. 167–242, 2007.

[29] M. E. J. Newman, "Assortative Mixing in Networks," *Physical Review Letters*, vol. 89, no. 20, Oct. 2002.

[30] L. B. Mithal, R. Yogev, H. L. Palac, D. Kaminsky, I. Gur, and K. K. Mestan, "Vital signs analysis algorithm detects inflammatory response in premature infants with late onset sepsis and necrotizing enterocolitis," *Early Human Development*, vol. 117, pp. 83 –89, Feb. 2018.

[31] F. Harrell. (2019). Statistically efficient ways to quantify added predictive value of new measurements, [Online]. Available: https://www.fharrell.com/post/addvalue/ (visited on 07/18/2020).

[32] S. Chen, L. Kang, Y. Lu, N. Wang, Y. Lu, B. Lo, and G.-Z. Yang, "Discriminative information added by wearable sensors for early screening - a case study on diabetic peripheral neuropathy," in *2019 IEEE 16th International Conference on Wearable and Implantable Body Sensor Networks (BSN)*, May 2019, pp. 1–4.

[33] V. Oliveira, W. von Rosenberg, P. Montaldo, T. Adjei, J. Mendoza, V. Shivamurthappa, D. Mandic, and S. Thayyil, "Early postnatal heart rate variability in healthy newborn infants.," *Frontiers in Physiology*, vol. 10, p. 922, 2019.

[34] W. An and M. Liang, "Fuzzy support vector machine based on within-class scatter for classification problems with outliers or noises," *Neurocomputing*, vol. 110, pp. 101–110, 2013.

[35] J. S. Calvert, D. A. Price, U. K. Chettipally, C. W. Barton, M. D. Feldman, J. L. Hoffman, M. Jay, and R. Das, "A computational approach to early sepsis detection," *Computers in Biology and Medicine*, vol. 74, pp. 69 –73, 2016.

[36] F. Lamping, T. Jack, N. Rübsamen, M. Sasse, P. Beerbaum, R. T. Mikolajczyk, M. Boehne, and A. Karch, "Development and validation of a diagnostic model for early differentiation of sepsis and non-infectious SIRS in critically ill children - a data-driven approach using machine-learning algorithms.," *BMC Pediatrics*, vol. 18, no. 1, p. 112, Mar. 2018.

[37] S. Mani, A. Ozdas, C. Aliferis, H. A. Varol, Q. Chen, R. Carnevale, Y. Chen, J. Romano-Keeler, H. Nian, and J.-H. Weitkamp, "Medical decision support using machine learning for early detection of late-onset neonatal sepsis.," *Journal of the American Medical Informatics Association*, vol. 21, no. 2, pp. 326–336, Mar. 2014.

[38] A. J. Masino, M. C. Harris, D. Forsyth, S. Ostapenko, L. Srinivasan, C. P. Bonafide, F. Balamuth, M. Schmatz, and R. W. Grundmeier, "Machine learning models for early sepsis recognition in the neonatal intensive care unit using readily available electronic health record data," *PLOS ONE*, vol. 14, no. 2, pp. 1–23, Feb. 2019.

[39] S. Nemati, A. Holder, F. Razmi, M. D. Stanley, G. D. Clifford, and T. G. Buchman, "An Interpretable Machine Learning Model for Accurate Prediction of Sepsis in the ICU.," *Critical Care Medicine*, vol. 46, no. 4, pp. 547–553, Apr. 2018.

[40] D. W. Shimabukuro, C. W. Barton, M. D. Feldman, S. J. Mataraso, and R. Das, "Effect of a machine learning-based severe sepsis prediction algorithm on patient survival and hospital length of stay: a randomised clinical trial," *BMJ Open Respiratory Research*, vol. 4, no. 1, 2017.

[41] R. Joshi, D. Kommers, L. Oosterwijk, L. Feijs, C. van Pul, and P. Andriessen, "Predicting neonatal sepsis using features of heart rate variability, respiratory characteristics, and ecg-derived estimates of infant motion," *IEEE Journal of Biomedical and Health Informatics*, vol. 24, no. 3, pp. 681–692, 2020.

[42] S. Ahmad, T. Ramsay, L. Huebsch, S. Flanagan, S. McDiarmid, I. Batkin, L. McIntyre, S. R. Sundaresan, D. E. Maziak, F. M. Shamji, P. Hebert, D. Fergusson, A. Tinmouth, and A. J. E. Seely, "Continuous Multi-Parameter Heart Rate Variability Analysis Heralds Onset of Sepsis in Adults," *PLOS ONE*, vol. 4, no. 8, pp. 1–10, Aug. 2009.

ACCEPTED MANUSCRIPT
