# Supplementary material for: Exploring Computational Techniques in Preprocessing Neonatal Physiological Signals for Detecting Adverse Outcomes: Scoping Review
Source: Interact J Med Res. 2024 Aug 20;13:e46946. doi: 10.2196/46946 (PMC11372324; doi:10.2196/46946)
Supplement: Multimedia Appendix 3 [file ijmr_v13i1e46946_app3.zip › Included Papers - Final/2964/R. Joshi et al. - 2020 - Predicting Neonatal Sepsis Using Features of Heart.pdf]

EINDHOVEN UNIVERSITY OF TECHNOLOGY

Predicting neonatal sepsis using features of heart rate variability, respiratory characteristics and ECG-derived estimates of infant motion

Citation for published version (APA):
Joshi, R., Kommers, D., Oosterwijk, L., Feijs, L., van Pul, C., & Andriessen, P. (2020). Predicting neonatal sepsis using features of heart rate variability, respiratory characteristics and ECG-derived estimates of infant motion. *IEEE Journal of Biomedical and Health Informatics*, *24*(3), 681-692. [8758174]. https://doi.org/10.1109/JBHI.2019.2927463

DOI:
10.1109/JBHI.2019.2927463

Document status and date:
Published: 01/03/2020

Document Version:
Accepted manuscript including changes made at the peer-review stage

Please check the document version of this publication:

• A submitted manuscript is the version of the article upon submission and before peer-review. There can be important differences between the submitted version and the official published version of record. People interested in the research are advised to contact the author for the final version of the publication, or visit the DOI to the publisher's website.
• The final author version and the galley proof are versions of the publication after peer review.
• The final published version features the final layout of the paper including the volume, issue and page numbers.

Link to publication

General rights
Copyright and moral rights for the publications made accessible in the public portal are retained by the authors and/or other copyright owners and it is a condition of accessing publications that users recognise and abide by the legal requirements associated with these rights.

• Users may download and print one copy of any publication from the public portal for the purpose of private study or research.
• You may not further distribute the material or use it for any profit-making activity or commercial gain
• You may freely distribute the URL identifying the publication in the public portal.

If the publication is distributed under the terms of Article 25fa of the Dutch Copyright Act, indicated by the "Taverne" license above, please follow below link for the End User Agreement:

www.tue.nl/taverne

Take down policy
If you believe that this document breaches copyright please contact us at:

openaccess@tue.nl

providing details and we will investigate your claim.

Download date: 09. Sep. 2022

This article has been accepted for publication in a future issue of this journal, but has not been fully edited. Content may change prior to final publication. Citation information: DOI 10.1109/JBHI.2019.2927463, IEEE Journal of Biomedical and Health Informatics

Predicting Neonatal Sepsis Using Features of Heart Rate Variability, Respiratory Characteristics and ECG-Derived Estimates of Infant Motion

Rohan Joshi*[1,2,3], Deedee Kommers[4,5], Laurien Oosterwijk[4], Loe Feijs[1], Carola van Pul[2,5], Peter Andriessen[4]

***Abstract*— This study in preterm infants was designed to characterize the prognostic potential of several features of heart rate variability (HRV), respiration, and (infant) motion for the predictive monitoring of late-onset sepsis (LOS). In a neonatal intensive care setting, the cardiorespiratory waveforms of infants with blood-culture positive LOS were analyzed to characterize the prognostic potential of 22 features for discriminating control- from sepsis-state, using the Naïve Bayes algorithm. Historical data of the subjects acquired from a period sufficiently before the clinical suspicion of LOS was used as control-state whereas data from the 24 hours preceding the clinical suspicion of LOS were used as sepsis-state (test-data). The overall prognostic potential of all features was quantified at three-hourly intervals for the period corresponding to test data by calculating the area under the receiver operating characteristics curve. For the 49 infants studied, features of HRV, respiration, and movement showed characteristic changes in the hours leading up to the clinical suspicion of sepsis, namely an increased propensity towards pathological heart rate decelerations, increased respiratory instability and a decrease in spontaneous infant activity, i.e., lethargy. While features characterizing HRV and respiration can be used to probe the state of the autonomic nervous system, those characterizing movement probe the state of the motor system – dysregulation of both reflects an increased likelihood of sepsis. By using readily interpretable features derived from cardiorespiratory monitoring, opportunities for preemptively identifying and treating LOS can be developed.**

Index terms—**Body movement, heart rate variability, lethargy, neonatal sepsis, predictive monitoring, respiratory characteristics, respiratory dynamics.**

I. Introduction

Preterm infants are at high risk for developing infections that lead to sepsis – a life-threatening and multi-organ complication triggered by an immunological response to the infection [1]. Sepsis constitutes a global public health concern, substantially contributing to neonatal morbidity and mortality [2], [3]. Sepsis in neonates is characterized into early-onset sepsis (EOS) and late-onset sepsis (LOS), differentiated by the timing of the onset of symptoms, the virulence of the infecting organism(s) and the associated pathogenesis. EOS is defined by the onset of symptoms within 72 hours after birth and is frequently associated with maternal Group B streptococcal colonization as a result of ascending infection from the maternal-genital tract [3]. On the other hand, LOS is characterized by the onset of symptoms consistent with sepsis at greater than 72 hours of life and is frequently associated with nosocomial or catheter-related coagulase-negative staphylococci infection. Notably, the prevalence of LOS is considerably higher than that of EOS, approximately 15% versus 1%, in infants born below 1500g [2], [4].

Timely diagnosis of LOS is important since delayed antibiotic therapy is associated with high mortality and adverse long-term outcomes [2], [5]. Further, as the incidence of sepsis has been steadily rising due to improved survival rates of progressively more preterm infants, correctly diagnosing LOS will continue to remain an important issue in the future [2], [6]. However, diagnosing LOS is challenging because the clinical signs thereof are nonspecific and often inconspicuous [7].

The definitive diagnosis of neonatal sepsis is based on the results of blood culture, even though these typically take 24 hours or longer to obtain and are associated with a significant number of false negatives and positives [8]. Further, once a blood culture is identified as positive, conclusively identifying the offending bacterial strain takes additional time [9], [10]. Consequently, based solely on the clinical suspicion of sepsis, typically, broad-spectrum antibiotic therapy is initiated at the time the blood culture is ordered, potentially followed by targeted antibiotic therapy if the offending pathogenic strains are isolated [2]. However, the liberal and prolonged use of

This research did not receive any specific grant from funding agencies in the public, commercial, or not-for-profit sectors. The authors report no conflict of interest.

*Corresponding Author. Contact: Rohan Joshi, Department of Family Care Solutions, Philips Research, High Tech Campus 34, 5656 AE, Eindhoven, The Netherlands, Tel: 06-17935137, Email: rohan.joshi@philips.com

[1]Department of Industrial Design, Eindhoven University of Technology, The Netherlands. [2]Department of Clinical Physics, Máxima Medical Centre Veldhoven, The Netherlands. [3]Department of Family Care Solutions, Philips Research, Eindhoven, The Netherlands. [4]Department of Neonatology, Máxima Medical Centre, Veldhoven, The Netherlands. [5]Department of Applied Physics, Eindhoven University of Technology, The Netherlands.

2168-2194 (c) 2019 IEEE. Personal use is permitted, but republication/redistribution requires IEEE permission. See http://www.ieee.org/publications_standards/publications/rights/index.html for more information.

This article has been accepted for publication in a future issue of this journal, but has not been fully edited. Content may change prior to final publication. Citation information: DOI 10.1109/JBHI.2019.2927463, IEEE Journal of Biomedical and Health Informatics

broad-spectrum antibiotics at even the slightest suspicion of sepsis, while carried out to reduce the burden of disease in vulnerable preterm infants, has contributed to growing antibiotic resistance in neonatal intensive care units (NICUs), worldwide [11], [12]. Therefore, multiple other approaches, such as those based on blood-based biomarkers as well as algorithms based on exploiting vital signs for the predictive monitoring of LOS have been researched to potentially augment the clinical diagnosis of sepsis at the bedside in the hope of both early and more specific identification of LOS [13].

While certain biomarkers do hold promise for diagnosing sepsis, their inherent limitations include being invasive, having a typical turnaround time of several hours, and offering only discrete snapshots of the infant's risk-status. Herein, taking advantage of the routinely monitored cardiorespiratory waveforms for continuously tracking the pathophysiological changes brought about by LOS is an attractive alternative to using blood-based biomarkers alone. The underlying hypothesis is that, in the hours leading up to the clinical suspicion of sepsis, autonomic regulatory mechanisms change characteristically [14] – the analysis thereof can help in continuously evaluating the risk of sepsis [13], [15]. Indeed, research based on displaying an index developed using heart rate variability (HRV) was found to be a promising method for predicting sepsis, and in a randomized trial of over 3000 preterm infants, continuously displaying this index reduced the overall mortality rate from 10% to 8% [16]. The rationale being that dysregulation, as measured by, both, a lowered HRV and a propensity towards transient heart rate (HR) decelerations, reflects subclinical signatures of sepsis up to even 24h before the clinical suspicion of sepsis and can thus preempt clinical intervention – i.e., initiating antibiotic therapy and ordering a blood culture [17].

However, it is possible that the longitudinal analysis of other physiological data-streams such as respiration, oxygen saturation, temperature, and infant activity, amongst others, can, both, independently provide diagnostic information for the predictive monitoring of sepsis as well as improve upon HRV-based approaches for predicting LOS [10], [13], [18], [19]. For instance, regarding respiration, it is well known that the regulation of respiration is disrupted by sepsis, as evidenced by an increased tendency towards apnea [20]–[23]. Further, a recent meta-analysis has shown that lethargy, i.e., the absence of regular (infant) motion, is one of the strongest indicators of neonatal sepsis [7]. Therefore, in our recent work, we have developed and clinically validated a method for quantitatively estimating infant motion from routinely acquired cardiorespiratory waveforms enabling the possibility of continuously tracking (infant) movement or the pathological lack of motion, which may be a marker for impending sepsis [24].

The current study aimed to characterize the prognostic potential of several features of HRV, features derived from the respiratory waveform, and ECG-derived estimates of infant motion towards the predictive monitoring of sepsis. To the best of our knowledge, while HRV and respiratory rates have been used for the predictive monitoring of LOS, the propensity towards apneas, disruption or dysregulation of respiration as mirrored in the respiratory waveform, as well as lethargy measured by infant motion, remain unexplored in the context of algorithmically tracking LOS.

II. Methods

A. Patient population and data acquisition

We conducted this study in the tertiary care NICU of Máxima Medical Center, the Netherlands from July 2016 – February 2018. Retrospectively, we inducted all infants born in this period when they, (i.) yielded a positive blood culture 72 hours after birth or later, (ii.) were born at less than 32 weeks gestational age (GA), (iii.) presented with clinical signs of generalized infection according to the Vermont Oxford criteria [25], and (iv.) received antibiotic therapy for at least five days, implying that these infants did get septic. Data on demographics, respiratory support and the clinical suspicion of sepsis, based on the CRASH-moment (Cultures, Resuscitation, and Antibiotics Started Here [26]), were collected from patient medical records. Infants with congenital anomalies or syndromes were excluded as were those infants with CRASH-moments that did not have 72 hours of patient monitoring data preceding CRASH. Further, in those infants presenting with multiple CRASH-moments, only the first CRASH-moment was retained for analysis.

As is routine, all infants received continuous cardiorespiratory monitoring (Philips IntelliVue MX 800, Germany). Based on the CRASH-moments, the electrocardiogram (ECG, 250 Hz) and the chest impedance (CI, 62.5 Hz) waveforms corresponding to the 72 hours before and after CRASH were retrospectively acquired from a data warehouse (PIIC iX, Data Warehouse Connect, Philips Medical Systems, Andover, MA). Henceforth, we refer to the 72 hours before and after CRASH as the pre- and post-CRASH periods respectively. As this was a retrospective study in which anonymized data corresponding to routine patient monitoring was used, the medical ethical committee provided a waiver in accordance with the Dutch law on medical research with humans.

B. Signal pre-processing

A peak detection algorithm was used to detect the R-peaks in the ECG-recordings [27], followed by calculating the interbeat or R-R intervals (RRi). The time series of RRi, also known as the tachogram, reflected the HRV, which was characterized using several features described below. Concerning the CI waveform, also derived from the ECG-electrodes, the peaks and troughs of the CI signal were algorithmically detected and were used to calculate the instantaneous respiratory rate, i.e., from breath-to-breath.

2168-2194 (c) 2019 IEEE. Personal use is permitted, but republication/redistribution requires IEEE permission. See http://www.ieee.org/publications_standards/publications/rights/index.html for more information.

This article has been accepted for publication in a future issue of this journal, but has not been fully edited. Content may change prior to final publication. Citation information: DOI 10.1109/JBHI.2019.2927463, IEEE Journal of Biomedical and Health Informatics

C. *Feature generation*

Three types of signals, all derived from the ECG-electrodes (i.e., ECG and CI waveforms) were used to generate a battery of features for further testing. The signals included HRV, the CI-based respiration signal, and the ECG-waveform derived estimates of infant motion. The features acquired from these signals are discussed below and are listed in Table 1 for convenience:

(i.) HRV

8 features of HRV were used for predictive modeling. These features included commonly employed features of HRV, originally used in adults, such as the SDNN, features specifically designed to capture neonatal HRV, such as the pDec and SampAsy, as well as features, such as the AAR, based on phase-rectified signal averaging (PRSA), a method robust to non-stationarities in the signal and noise. Details on feature description and their physiological interpretation are provided in Table 1.

(ii.) CI-based respiration

11 features were generated from the respiration signal – 4 were based on the instantaneous respiratory rate, for instance, the mean and the interdecile range (IDR) of the respiratory rate, while 4 and 3 features respectively were based on measures of respiratory instability and respiratory cessations. Measures of respiratory instability, described below, are original and were developed for this work to capture respiratory dysregulation. An algorithm described in the literature was used for characterizing features of respiratory cessations [28].

Dysregulation of respiration or equivalently dysregulation of the respiratory drive is reflected in the characteristics of the respiration waveform. For instance, during a stable epoch, the respiratory waveform may appear periodic and resemble a sinusoidal waveform. Conversely, during epochs with intermittent gasps, cessations in breathing or unstable breathing with variations in respiratory amplitude from breath-to-breath, the respiratory waveform may appear disorganized – such disorganization of respiration may not be fully captured in the respiratory rate alone. To quantitatively capture respiratory instability, we calculated the entropy of the windowed and band-pass filtered (0.45- 1.45 Hz) respiration waveform. We calculated the approximate entropy (ApEn) [29] using a historical window of 30s and a sequence-length for comparison, also known as the embedded dimension, equal to 1 second of data (empirical choice) with a tolerance value defined as 0.15 times the standard deviation of the windowed data. Since entropy is a measure of system disorganization, a perfectly stable and periodic respiration signal would have low entropy whereas complete cessation of breathing would have high entropy, since then the respiration signal would reflect sensor noise alone. Epochs with respiratory instability, i.e., epochs between perfectly stable breathing and complete cessation of breathing, would have intermediate values of entropy. We calculated the ApEn of the respiratory waveform, as described above, at a frequency of 1 Hz and termed this time-series as respiratory instability – 4 features based on which were used in the predictive monitoring of LOS in this study (Table 1).

Since preterm infants are susceptible to respiratory cessations, some of which may qualify as clinically recognized apneas, we used the algorithm developed by Lee et al. to identify respiratory cessations in the CI signal [28]. The duration of individual respiratory cessations that was considered as valid was based on the rules provided by Lee et al. They ignored cessations shorter than 2s and considered as valid all periods when the infant ceased to breathe for a duration longer than 5s, as well as episodes less than 5s if they were sufficiently close to other cessations (< 3s) [28]. We ignored cessations longer than 120s as potential artifacts. Three features based on respiratory cessations were intended to capture the propensity towards, (i.) short cessations, (ii.) clinically significant cessations (or apnea), and (iii.) the total burden of cessations, measured by calculating the percentage of time the breathing ceased, irrespective of the length of cessation. Further details on these features are provided in Table 1.

(iii.) ECG-derived estimates of infant motion – the signal instability index

As stated in the introduction, lethargy is an important clinical symptom or warning sign of sepsis. However, there have been no attempts to use quantitative, continuous, and automated measures of lethargy in the predictive monitoring of sepsis. Recently, we have developed and tested an algorithm that can be applied on the ECG waveform to yield the signal instability index (SII) – an integrated measure to quantify infant motion by capturing both the extent and duration of movements. The intention is that by reliably capturing movement, one can also detect the pathological lack of movement that is expected to precede sepsis. Briefly, the SII is a non-parametric measure based on the kernel density estimate that can be applied to a band-pass filtered (0.001 – 0.40 Hz) ECG waveform to obtain an estimate of motion (the SII) every second (1 Hz) using the ECG-data of the past 10s, as described elsewhere [15]. Low values of the SII are indicative of the absence of movement, while higher values are a quantitative estimate of body movement [15]. Three features – the IDR, standard deviation (SD) and the skewness of the SII were used to quantify body movement, or the lack thereof. Low values of the IDR and the SD represent a lack of movement (or lethargy), while large positive values of the skewness feature represent lethargy.

D. *Data selection corresponding to control state and sepsis state*

We calculated all features, every three hours, using data of the past three hours. Therefore, for instance, corresponding to the CRASH-moment, denoted by t = 0, the corresponding feature-values would be based on the data from -3 to 0 hours. The same reasoning applies to feature values across the entire time-series,

2168-2194 (c) 2019 IEEE. Personal use is permitted, but republication/redistribution requires IEEE permission. See http://www.ieee.org/publications_standards/publications/rights/index.html for more information.

This article has been accepted for publication in a future issue of this journal, but has not been fully edited. Content may change prior to final publication. Citation information: DOI 10.1109/JBHI.2019.2927463, IEEE Journal of Biomedical and Health Informatics

for the pre- and the post-CRASH period.

Table 1: A list of features, along with a description and interpretation of the same, used for the predictive monitoring of LOS.

Feature description	Abbreviation	Interpretation
**Features of HRV**		
RR-interval	RRi	Average heart rate
The square root of the mean of the squares of successive differences between adjacent NN intervals	RMSSD	Captures dispersion in the duration between consecutive heart beats with increased sensitivity to larger dispersions.
The standard deviation of RRi	SDNN	Captures dispersion in the duration between consecutive heartbeats
Percentage deceleration of RRi [31]	pDec	Captures the percentage of HR decelerations
The standard deviation of RRi corresponding to pDec [31]	SDDec	Captures extent of HR decelerations
Average deceleration response [32], [33][a]	ADR	Average HR response to decelerations using PRSA
Average acceleration response [32], [33][a]	AAR	Average HR response to accelerations using PRSA
Sample asymmetry [34]	SampAsy	Captures asymmetry in the histogram of RRi
**Features characterizing respiration**		
Mean respiratory rate	RespMean	Average respiration rate
Interdecile range of respiratory rate	RespIDR	A measure of the difference between the 90th and 10th percentile of the respiratory rate
The standard deviation of respiratory rate	RespSD	Dispersion of the respiratory rate
The skewness of respiratory rate	RespSkew	A measure of the degree of distortion compared to a Gaussian distribution of the respiratory rate
Mean value of ApEn [29]	ApEnMean	An average measure of respiratory instability
The standard deviation of ApEn	ApEnSD	Dispersion of respiratory instability
Interdecile range of ApEn	ApEnIDR	A measure of the difference between the 90th and 10th percentile estimate of respiratory instability
Skewness of ApEn[b]	ApEnSkew	A measure of the degree of distortion compared to a Gaussian distribution of respiratory instability
Percentage contribution of cessations of up to 5s to the total duration of cessation	percCess-5	The propensity to short cessations
Percentage contribution of cessations of up to 20s to the total duration of cessation	percCess-20	The propensity to cessations that are clinically significant
Percentage cessations throughout the monitored period, irrespective of the duration	percCessTotal	Total percentage of time with no breathing
**Features characterizing ECG-derived estimate of infant motion**		
Interdecile range of the SII	SII-IDR	A measure of the difference between the 90th and 10th percentile estimate of infant motion
The standard deviation of the SII	SII-SD	A measure of the dispersion of estimated infant motion
The skewness of the SII	SII-Skew	A measure of the degree of distortion compared to a Gaussian distribution of estimated infant motion.

[a] All increases and decreases in the RRi served as an anchor point for deceleration and acceleration, respectively [32]. The ADR and AAR were calculated as the difference in the average of the 25 RRi after (including anchor point) and the 25 RRi before the anchor points. [b] Skewness, the third standardized moment, is a measure of the asymmetry of the data around the sample mean. The skewness is 0 for symmetric distributions, positive if the distribution has a longer tail to the right, and negative if the distribution has a longer tail to the left.

Research has shown that the earliest subclinical signatures of LOS may start manifesting up to 24 hours preceding CRASH [26]. Therefore, considering that this study for the predictive monitoring of LOS was of a single-group design, we sought to obtain historical time-series data from a period sufficiently before the CRASH-moment and treated it as *control-state*. While we extracted data from the period 72 hours before CRASH to the 72 hours after CRASH, we included the 10 three-hour intervals from -66 to -39 hours before CRASH as control-state since this period is sufficiently far from the 24 hours preceding CRASH and is expected to be a representative measure of (healthy) baseline variability. Data from the -72 and -69 hours before CRASH were not used as control-state since this period was, for some infants, just after birth and potentially features from this period could still exhibit effects of the birthing process.

While there is evidence that subclinical signatures of LOS manifest in the 24 hours preceding CRASH, the temporal manifestation of their predictive potential is not clear. Therefore, we explored how the predictive potential of the features under consideration changed in the hours leading up to

2168-2194 (c) 2019 IEEE. Personal use is permitted, but republication/redistribution requires IEEE permission. See http://www.ieee.org/publications_standards/publications/rights/index.html for more information.

This article has been accepted for publication in a future issue of this journal, but has not been fully edited. Content may change prior to final publication. Citation information: DOI 10.1109/JBHI.2019.2927463, IEEE Journal of Biomedical and Health Informatics

the CRASH-moment by independently analyzing all three-hour intervals (total 9) between -24 to 0 hours – i.e., the *sepsis-state* – compared to the control-state.

E. Data analysis and evaluation of classification

Since baseline values of the features may vary from one infant to the other, owing to factors such as differences in physiological maturity, the time-series of all features were standardized (mean value subtracted, followed by dividing by the standard deviation) based on data from the control-state. For all 22 features – 8 based on HRV, 11 based on respiration and 3 based on infant motion – the pre- and post-CRASH time series corresponding to the mean values (standard error of the mean, SEM) of the features were plotted to visualize how features changed in the period leading up to CRASH and beyond.

Further, the Naïve Bayes classifier was used to distinguish the control-state from septic-state. The use of the Naïve Bayes classifier was motivated by the fact that it works well, even for a small amount of training data and is highly interpretable. Concerning classifier characteristics, the class priors were defined to be uniform while feature distributions were non-parametrically estimated using multiple kernels of the normal distribution. The predictive strength of the classifier was evaluated by calculating the area under the receiver operating characteristics (AUROC) curve from the left out folds of fivefold cross-validation (CV) stratified by control- and sepsis-states, carried out five times to provide an average estimate of the AUROC and its dispersion (SD). Further, along with the AUROC, we calculated the true positive rate (TPR) of classification while setting the classifier's false positive rate (FPR) at 10%. As described earlier, 10 data points from each infant served as control-state while 9 data points from the 24 hours preceding CRASH were analyzed from each infant, one at a time, serving as data corresponding to the sepsis-state. By individually analyzing each three-hour epoch in the hours leading up to CRASH, the temporal trajectory of the discriminative potential of the classifier, for individual features, was quantified using the AUROC and the TPR.

While the AUROC measures the predictive strength of the classifier, it does not explain how changes in the individual features affect the probability of sepsis. Thus, to make the classification process a white-box model, we calculated the probabilities of sepsis for different values of the individual features. The mean values and standard deviations of these probabilities were calculated over the different runs of the CV process for a chosen model and plotted individually for each feature. All models were developed and evaluated in a MATLAB environment (MATLAB R2017, MathWorks).

III. RESULTS

During the study, based on the clinical suspicion of sepsis, 185 blood cultures were drawn 72 hours after birth or later. Of these blood cultures, 92 were positive. 4 and 3 cultures respectively were excluded from analysis since they were obtained from infants born after 32 weeks of gestation and in infants that did not present signs of generalized infection. One infant presented with a congenital anomaly and therefore, the associated CRASH moment was excluded; 14 CRASH-moments did not have sufficient data in the pre-CRASH period while 21 blood cultures were repeat measures and therefore excluded. The remaining 49 CRASH-moments, including data from their corresponding pre- and post-CRASH periods, were used for analysis – the patient metadata corresponding to these 49 infants are provided in Table 2. It should be noted that these 49 CRASH-moments were obtained from 49 different infants. The most common pathogen isolated from blood cultures was *Staphylococcus epidermidis* (55%), followed by *Staphylococcus capitis* (12%), both coagulase-negative staphylococci that are commonly associated with LOS.

Table 1: Patient metadata, including characteristics of invasive mechanical ventilation (intubation).

Characteristic	Median (IQR)
No. of infants	49
Male gender (%)	57.14
Gestational age (weeks)	27.85 (26.85-29.32)
Birthweight (gram)	1085 (931-1292)
Postnatal age at CRASH (days)	6.5 (4.9-10.1)
Mortality (%)	12.24
Invasive mechanical ventilation in the pre-CRASH period (no.)	2 infants
Pre-CRASH period: Duration between intubation and CRASH (hours)	9 (2-16)
Invasive mechanical ventilation in the post-CRASH period (no.)	16 infants
Post-CRASH period: Duration between CRASH and intubation (hours)	1.5 (0.5-7)

Legend: IQR, Interquartile range

Concerning the changes in the features that manifest in the hours leading up to CRASH and beyond, Figure 1 shows the time-series of 6 features – 2 features each from the signal categories of HRV, respiration, and movement. Regarding the features of HRV, the SampAsy and the AAR exhibited a decreasing trend in the hours leading up to CRASH, implying increasing asymmetry in the histogram of RRi and a reduced ability to accelerate the HR, respectively. Regarding the features based on the respiration waveform, both, RespIDR and ApEnMean increased preceding CRASH. This reflects an increase in the dynamic range of respiratory rate and increasing respiratory instability, respectively. For features based on movement, the SII-IDR, and the SII-SD decreased, reflecting a reduction in spontaneous movement, in the hours leading up to CRASH.

2168-2194 (c) 2019 IEEE. Personal use is permitted, but republication/redistribution requires IEEE permission. See http://www.ieee.org/publications_standards/publications/rights/index.html for more information.

This article has been accepted for publication in a future issue of this journal, but has not been fully edited. Content may change prior to final publication. Citation information: DOI 10.1109/JBHI.2019.2927463, IEEE Journal of Biomedical and Health Informatics

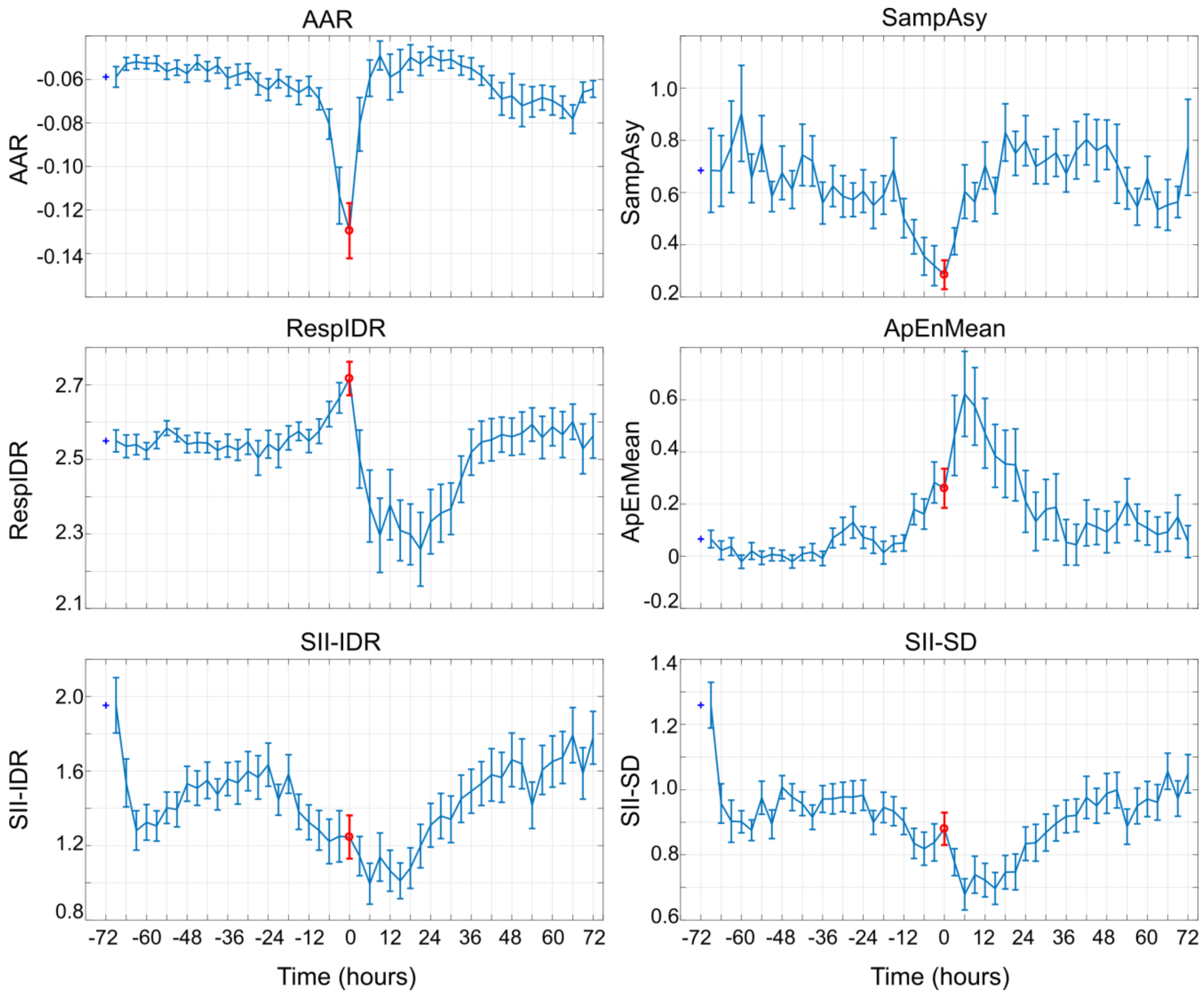

Figure 1: Time series of standardized features values – HRV (top), respiration (middle) and movement (bottom) – in the 72 hours before and after CRASH (t = 0; error bar with circular marker). All features show characteristic changes in the hours leading up to the CRASH-moment and beyond. The error bars represent the SEM.

Figure 2 shows, for the 6 features discussed above, how the performance of the Bayesian classifier changes in the 24 hours leading up to sepsis. Some features (e.g., SampAsy, ApEnMean) showcase potential for discriminating the septic-state from the control-state up to 15 hours before CRASH (AUROC > 0.50), and broadly speaking the overall predictive potential of the classifier, as measured by the AUROC, improves considerably leading up to the CRASH-moment, across all features. Since, from a clinical perspective, false predictions of sepsis are undesirable, alongside the AUROC, the TPR of correctly predicting impending sepsis while tolerating a low FPR of 10% are also provided in Figure 2. Here too, an improving trend can be seen in the hours leading up to CRASH. Table 3 characterizes the overall predictive potential (AUROC) of the Bayesian classifier for all 22 features in the 24 hours leading up to sepsis. Overall, RMSSD is the best-performing feature with an AUROC of 0.84 (0.001) at t = 0.

For features of respiration and respiratory instability, overall, the performance is lower in comparison to features of HRV. Nevertheless, at t = -3 and at t = -6, features such as RespIDR and ApEnIDR were comparable to or even outperformed some features of HRV. Notably, features based on respiration and respiratory instability consistently outperformed features based on respiratory cessations, including cessations constituting clinical apneas. Features based on movement exhibited a trend similar to that of HRV and respiration-based features, i.e., improving performance in the hours leading up to sepsis with SII-SD performing best with an AUROC of 0.67 (0.001) at t = -3.

For the changes in feature-values that determined classification, examples of one feature each from the categories of HRV, respiration, and movement, are shown in Figure 3, along with the corresponding ROC curve, for the model trained using data from the period t = -3, i.e., data from -6 to -3 hours preceding sepsis. This period merely serves as an example; results from models corresponding to other time-periods were comparable in their trend. Regarding interpretation, the graph corresponding to AAR in Figure 3 shows that with decreasing values of AAR the classifier-interpreted probability of sepsis

2168-2194 (c) 2019 IEEE. Personal use is permitted, but republication/redistribution requires IEEE permission. See http://www.ieee.org/publications_standards/publications/rights/index.html for more information.

This article has been accepted for publication in a future issue of this journal, but has not been fully edited. Content may change prior to final publication. Citation information: DOI 10.1109/JBHI.2019.2927463, IEEE Journal of Biomedical and Health Informatics

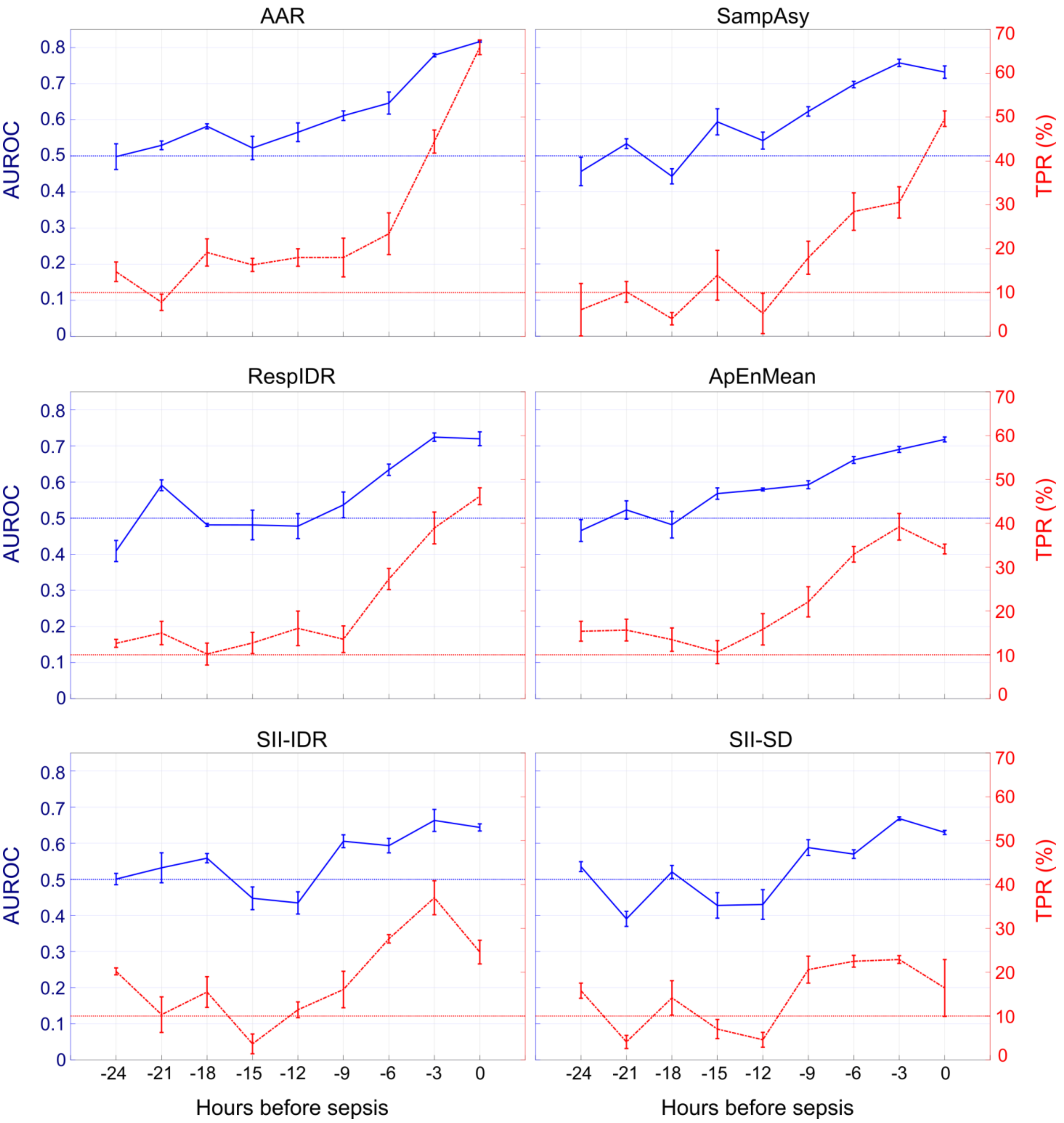

Figure 2: The performance of the Naïve Bayesian model in discriminating sepsis- from control-data is characterized by the AUROC, in the 24 hours preceding CRASH for features of HRV (top), respiration (middle) and movement (bottom). The TPR of correctly identifying sepsis, while accepting an FPR of 10% is shown alongside. With increasing proximity to the CRASH moment, all features exhibit an improved ability to identify sepsis from control.

increases while for increasing values of the IDR of respiration, the probability of sepsis increases. Similarly, relating to movement, a decrease in the IDR of the SII, reflecting decreased movement or lethargy, corresponds to an increased probability of sepsis. Further, the fact that the standard deviations for the classifier-estimated probabilities of sepsis are small suggests that the training was stable with little evidence of overfitting.

2168-2194 (c) 2019 IEEE. Personal use is permitted, but republication/redistribution requires IEEE permission. See http://www.ieee.org/publications_standards/publications/rights/index.html for more information.

This article has been accepted for publication in a future issue of this journal, but has not been fully edited. Content may change prior to final publication. Citation information: DOI 10.1109/JBHI.2019.2927463, IEEE Journal of Biomedical and Health Informatics

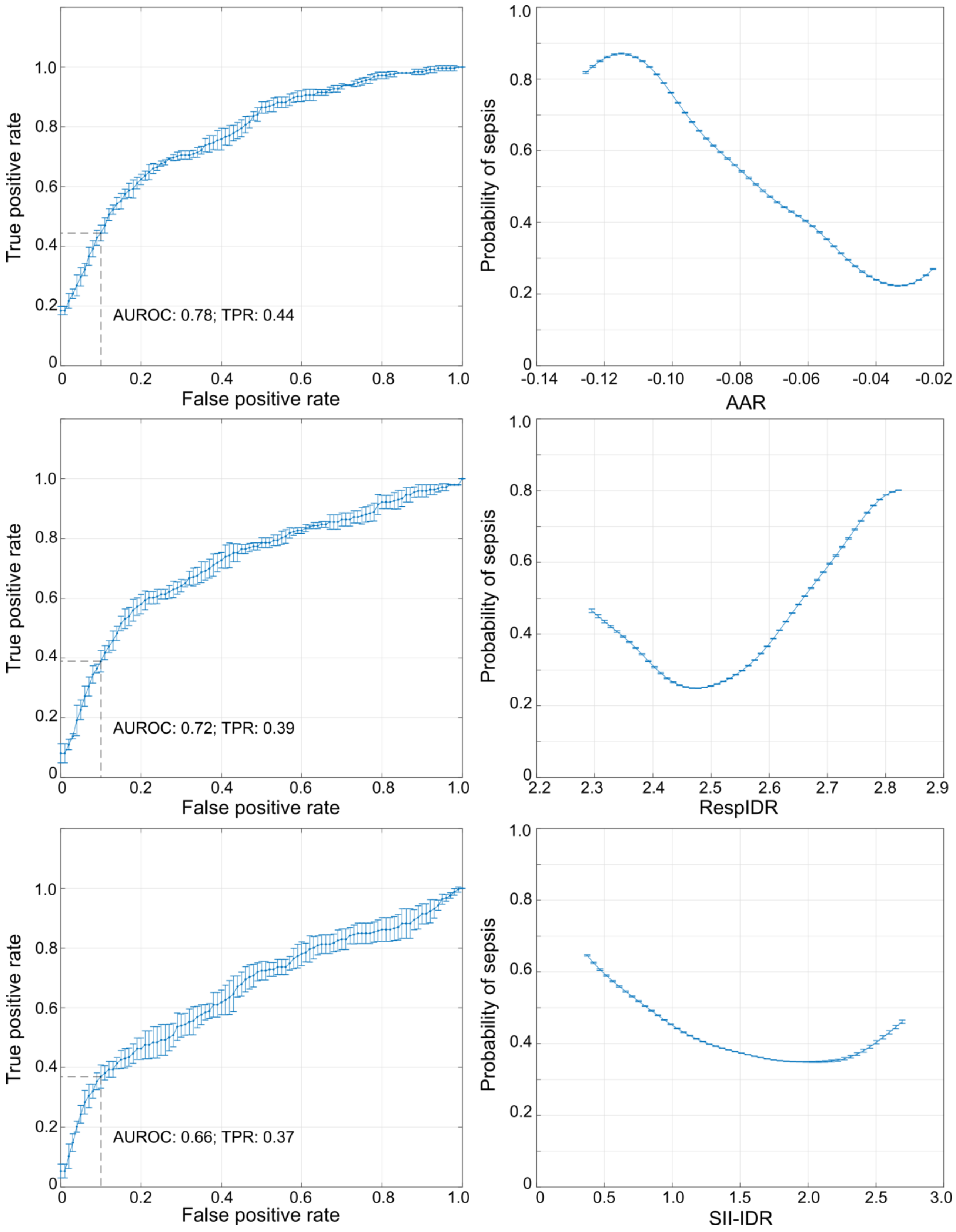

Figure 3: The left column shows the ROC curves for the model trained at t = -3 hours, for a feature each from HRV (top), respiration (middle) and movement (bottom). The TPR of classification, while setting the classifier's FPR at 0.1, is shown in the corresponding legends. The column on the right shows the mean (SD, error bars) values of the classifier estimated probabilities of sepsis for a range of (standardized) feature values. The x-axes were incremented in steps of 1% while the lower and upper bounds of the x-axes were obtained from the 5th and 95th percentile values of the features, estimated from the raw data. The small SD suggests that the training was stable with little evidence of overfitting.

2168-2194 (c) 2019 IEEE. Personal use is permitted, but republication/redistribution requires IEEE permission. See http://www.ieee.org/publications_standards/publications/rights/index.html for more information.

This article has been accepted for publication in a future issue of this journal, but has not been fully edited. Content may change prior to final publication. Citation information: DOI 10.1109/JBHI.2019.2927463, IEEE Journal of Biomedical and Health Informatics

Table 3: The predictive strength of the classifier as measured by the mean (SD) of the AUROC leading up to the CRASH moment. Additionally, for the features in bold, the temporal trajectory of the performance of features is shown in Figure 2.

**Feature**	**t = -24**	**t = -21**	**t = -18**	**t = -15**	**t = -12**	**t = -9**	**t = -6**	**t = -3**	**t = 0**
RRi	0.57 (0.01)	0.60 (0.02)	0.64 (0.02)	0.64 (0.00)	0.67 (0.01)	0.70 (0.01)	0.69 (0.01)	0.67 (0.01)	0.71 (0.01)
RMSSD	0.60 (0.02)	0.65 (0.00)	0.65 (0.00)	0.68 (0.00)	0.68 (0.00)	0.71 (0.01)	0.72 (0.01)	0.75 (0.00)	0.84 (0.00)
SDNN	0.49 (0.01)	0.53 (0.01)	0.62 (0.0)	0.62 (0.01)	0.62 (0.01)	0.57 (0.03)	0.57 (0.02)	0.71 (0.00)	0.78 (0.01)
pDec	0.45 (0.03)	0.54 (0.02)	0.44 (0.03)	0.45 (0.04)	0.55 (0.03)	0.58 (0.02)	0.64 (0.01)	0.74 (0.01)	0.72 (0.01)
SDDec	0.51 (0.01)	0.51 (0.01)	0.53 (0.02)	0.60 (0.01)	0.57 (0.02)	0.44 (0.03)	0.66 (0.01)	0.77 (0.01)	0.81 (0.00)
ADR	0.50 (0.02)	0.50 (0.04)	0.57 (0.0)	0.48 (0.05)	0.59 (0.03)	0.59 (0.02)	0.64 (0.00)	0.75 (0.01)	0.79 (0.01)
**AAR**	**0.49 (0.03)**	**0.53 (0.01)**	**0.58 (0.01)**	**0.52 (0.03)**	**0.57 (0.03)**	**0.61 (0.01)**	**0.65 (0.03)**	**0.78 (0.00)**	**0.82 (0.00)**
**SampAsy**	**0.45 (0.03)**	**0.53 (0.01)**	**0.44 (0.02)**	**0.59 (0.04)**	**0.54 (0.02)**	**0.62 (0.01)**	**0.70 (0.01)**	**0.76 (0.01)**	**0.73 (0.02)**
RespMean	0.51 (0.02)	0.44 (0.03)	0.53 (0.02)	0.65 (0.01)	0.65 (0.01)	0.68 (0.01)	0.60 (0.03)	0.66 (0.01)	0.73 (0.01)
**RespIDR**	**0.40 (0.02)**	**0.59 (0.01)**	**0.48 (0.00)**	**0.48 (0.04)**	**0.48 (0.03)**	**0.54 (0.04)**	**0.63 (0.02)**	**0.72 (0.01)**	**0.72 (0.02)**
RespSD	0.49 (0.01)	0.63 (0.01)	0.59 (0.02)	0.44 (0.04)	0.58 (0.01)	0.61 (0.01)	0.63 (0.01)	0.71 (0.00)	0.73 (0.01)
RespSkew	0.48 (0.02)	0.53 (0.03)	0.51 (0.01)	0.54 (0.03)	0.53 (0.01)	0.56 (0.0)	0.62 (0.01)	0.61 (0.02)	0.62 (0.01)
**ApEnMean**	**0.46 (0.03)**	**0.52 (0.03)**	**0.48 (0.04)**	**0.57 (0.02)**	**0.58 (0.00)**	**0.59 (0.01)**	**0.66 (0.01)**	**0.69 (0.01)**	**0.72 (0.01)**
ApEnSD	0.52 (0.03)	0.46 (0.03)	0.38 (0.02)	0.47 (0.03)	0.50 (0.03)	0.56 (0.02)	0.59 (0.03)	0.65 (0.01)	0.72 (0.01)
ApEnIDR	0.51 (0.01)	0.42 (0.03)	0.49 (0.02)	0.51 (0.02)	0.59 (0.01)	0.61 (0.01)	0.67 (0.01)	0.70 (0.01)	0.70 (0.01)
ApEnSkew	0.51 (0.04)	0.53 (0.01)	0.51 (0.04)	0.52 (0.03)	0.51 (0.03)	0.52 (0.03)	0.58 (0.02)	0.69 (0.01)	0.50 (0.03)
percCess-5	0.51 (0.03)	0.47 (0.02)	0.43 (0.06)	0.52 (0.04)	0.49 (0.03)	0.49 (0.03)	0.57 (0.02)	0.62 (0.02)	0.50 (0.02)
percCess-20	0.44 (0.03)	0.46 (0.04)	0.48 (0.02)	0.52 (0.02)	0.40 (0.04)	0.49 (0.03)	0.50 (0.02)	0.55 (0.03)	0.58 (0.04)
percCessTotal	0.45 (0.04)	0.49 (0.02)	0.47 (0.03)	0.49 (0.01)	0.46 (0.03)	0.42 (0.01)	0.42 (0.04)	0.55 (0.02)	0.61 (0.03)
**SII-IDR**	**0.50 (0.01)**	**0.53 (0.04)**	**0.56 (0.01)**	**0.45 (0.03)**	**0.43 (0.03)**	**0.61 (0.02)**	**0.59 (0.02)**	**0.66 (0.03)**	**0.64 (0.01)**
**SII-SD**	**0.53 (0.01)**	**0.39 (0.02)**	**0.52 (0.02)**	**0.43 (0.04)**	**0.43 (0.04)**	**0.59 (0.02)**	**0.57 (0.01)**	**0.67 (0.00)**	**0.63 (0.01)**
SII-Skew	0.41 (0.04)	0.52 (0.03)	0.51 (0.03)	0.48 (0.03)	0.47 (0.03)	0.51 (0.03)	0.57 (0.02)	0.56 (0.01)	0.59 (0.04)

IV. Discussion

The global burden of neonatal sepsis is large and contributes to high mortality and morbidity rates in preterm infants, including potentially lifelong neurological deficits [35]. Predictive monitoring of neonatal sepsis can help in redirecting the attention of caregivers to infants exhibiting subclinical signatures of deterioration and can thus provide a longer

2168-2194 (c) 2019 IEEE. Personal use is permitted, but republication/redistribution requires IEEE permission. See http://www.ieee.org/publications_standards/publications/rights/index.html for more information.

This article has been accepted for publication in a future issue of this journal, but has not been fully edited. Content may change prior to final publication. Citation information: DOI 10.1109/JBHI.2019.2927463, IEEE Journal of Biomedical and Health Informatics

window of opportunity for preemptive therapeutic action. The premise of such predictive models is that by exploiting the high-resolution, physiological streams of data that are continuously acquired through routine patient monitoring, one can identify subclinical signatures of disease before they manifest to the extent that the deterioration is apparent to clinicians. Such predictive models are reliant on developing robust, interpretable, and computationally efficient features that change characteristically in the hours leading up to sepsis. In this study, we characterized the prognostic potential of multiple features of HRV, as well as novel features based on respiration and ECG-derived estimates of infant motion, towards the predictive monitoring of sepsis.

Regarding HRV, we reaffirm previous findings, largely from a sole research group, that features based on HRV change in the hours leading up to CRASH [10], [26], [36]. For instance, as previously described in the literature, we too identified that the asymmetry of the RRi-histogram increases (SampAsy, Figure 1) in the hours preceding CRASH [34]. A notable finding was that, as measured by the size of the SEM of HRV-values in the time-series around CRASH (Figure 1), the features AAR and ADR were most robust, likely because they were least affected by noise. This can be attributed to the fact that these features were based on the technique of PRSA, a method designed to deal with the issues of noise and non-stationarities, prevalent in biological signals [33]. In general, for the 24 hours preceding CRASH, as measured by the AUROC, RMSSD followed by AAR performed best. Furthermore, the TPR, at t = 0, for identifying sepsis using RMSSD and AAR was equal to 66% and 61%, respectively, at the expense of an FPR of 10%.

By analyzing the breath-to-breath variability and respiratory instability, we could identify characteristic signs of sepsis in the hours leading up to CRASH. Notably, both the dynamic range of the respiratory rate as well as dysregulation reflected in the respiratory waveforms increased in the hours preceding CRASH. These findings can be reconciled with the familiar clinical observation of lowered respiratory drive owing to sepsis. Notably, though, features based on respiratory cessations, including apneas, had only limited prognostic potential and underperformed compared to features based on the respiratory rate and respiratory instability. This might be explained by our limited ability to accurately identify apneas, especially obstructive apnea, based on the NICU-standard for monitoring respiration – impedance plethysmography. As respiratory cessations, including apneas, have only limited prognostic potential in predicting sepsis, it further underlines the motivation of incorporating features based on respiratory instability, as reflective of respiratory drive, into the analysis [28].

Concerning the subclinical signatures of sepsis, both, features based on HRV and respiration used in this work, longitudinally probed the disruption of autonomic regulation. Thus, while the computationally derived features described here reflect an underlying disruption of regulation preceding CRASH, these changes would not be necessarily visible to the naked eye, even those of an experienced clinician. These remarks should be considered in light of the fact that in the 12 hours preceding CRASH there would have been at least 6 nursing-care periods that included enteral feeding – this is a certainty as all infants received two-hourly enteral feeds. However, in this period, despite proximity to and interaction with the infant, clinicians did not suspect sepsis, else, blood cultures would have been ordered earlier.

In a manner similar to which sepsis disrupts the functioning of the autonomic nervous system, sepsis also disturbs the functioning of the motor system, as evidenced by increasing lethargy preceding sepsis [37], [38]. Frequently, changes in motor-state such as changes reflected by the absence of spontaneous movement are also of subclinical nature, in the sense that they are subtle and not apparent during routine observations. Based on our analysis, our most notable finding in this study was that features based on clinically tested estimates of infant motion [24], derived from the continuously acquired ECG waveform could quantitatively assess the pathological lack of movement preceding sepsis. While the performance of these movement-features was poorer than that of HRV- and respiration-based features, this might be attributed to the fact that the ECG is not an ideal motion-sensor, as the ECG-acquisition system it is designed to filter out motion artifacts. Notably, superior sensing modalities for continuously monitoring movement, that is low-cost and unobtrusive, do exist and are described elsewhere [24]. In our current work, we identified lethargy as a useful warning sign of impending sepsis; the continuous and automated monitoring thereof, in addition to routine cardiorespiratory monitoring, opens up exciting possibilities for the predictive monitoring that may be of particular interest to resource-constrained settings where supplementary lab-tests and blood cultures, in response to suspected sepsis, may not be financially viable.

For predictive analytical models to be clinically accepted and incorporated into the clinical workflow, it remains vitally important that the underlying mechanics of the model are transparent and that clinicians can readily appreciate the science behind the approach – in effect, the model needs to be a *white-box* model [39]. Therefore, we demonstrate how changes in feature-values affect the classifier-estimated probability of sepsis, for one feature each from the category of HRV, respiration, and movement (Figure 3). The analysis showed that a decrease in the AAR, i.e., a reduced ability to accelerate the heart rate, was associated with an increased risk of sepsis. Similarly, large fluctuations in the breath-to-breath respiratory rate, interpreted as an increase in the dynamic range of respiration were associated with an increased risk of sepsis. Concerning the SII-IDR, moderate values that represent a routine degree of spontaneous motion were associated with a low risk of sepsis while low values of SII-IDR, indicative of lethargy, increased the likelihood of sepsis. In practice, if the (trained) model were to be deployed in patient monitors, it would simply be a look-up table of feature value(s) versus the

2168-2194 (c) 2019 IEEE. Personal use is permitted, but republication/redistribution requires IEEE permission. See http://www.ieee.org/publications_standards/publications/rights/index.html for more information.

This article has been accepted for publication in a future issue of this journal, but has not been fully edited. Content may change prior to final publication. Citation information: DOI 10.1109/JBHI.2019.2927463, IEEE Journal of Biomedical and Health Informatics

probability of sepsis. Computationally speaking, with regard to calculating features, patient monitors already identify the heart rate and respiratory rate from beat-to-beat and from breath-to-breath. Based on this information, intermittently calculating (a subset of) the features, including features based on movement, is computationally inexpensive. Visually comparing where a feature value falls in its risk for predicting sepsis is straightforward and can be incorporated into patient monitoring systems – we propose this approach as opposed to setting a threshold for classification. Such an approach enables clinicians to incorporate the classifier-proposed risk of sepsis, and in particular *changes* in the longitudinally predicted risk, into their own heuristics of decision-making including past experience, lab-values, observational scoring, and unit policies, amongst others. Of course, automated alarms based on setting a threshold, say for feature-values breaching the 80th percentile value of the training data, can additionally be programmed, as is commonly done for cardiorespiratory alarms.

In this work, we detailed the results of univariable analysis, i.e., the prognostic potential of individual features, using the Naïve Bayesian model. Unfortunately, combining features, even features based on different feature-categories such as HRV, respiration, and movement, did not improve classifier-performance as measured by the AUROC. For instance, corresponding to t = 0, RMSSD was the best-performing feature with a mean AUROC of 0.84 while for the same period the best-performing 2 and 3 feature combinations had mean AUROCs of 0.84 and 0.82, respectively (analysis not shown). This suggests that combining features did not add *new* information to the prediction model, although this might change if, for instance, lethargy was more reliably monitored through dedicated motion sensors. Another aspect of note is that while the AUROC did not increase upon combining features, there was an increase in the TPR of classification without any appreciable decrease in the AUROC, suggesting that multivariable analysis remains a promising area for future investigation. The performance of the Naïve Bayesian model for the two best-performing combination of features in the hours leading up to sepsis is shown in the Appendix.

Concerning features based on respiration, a limitation of this study was that information regarding supplemental oxygen was unavailable. This implies that if an infant exhibited respiratory distress, as is common in NICU infants, and if a clinician were to increase the fraction of supplemental oxygen leading to improved respiratory stability, distress, as measured by respiratory features, may potentially be camouflaged. Herein, information on the (de-) intensification of respiratory support would likely add novel value, not just standalone but also to the features based on respiration. On the contrary, merely monitoring the oxygen saturation, as opposed to also incorporating information on the intensification of respiratory support, while potentially useful, will not suffice. This is due to the S-shaped nature of the oxygen-dissociation curve, where despite large increases in dissolved arterial oxygen, the oxygen saturation levels (O_2 bound to hemoglobin), may change only marginally [40].

Since this study aimed to characterize, in the hours leading up to sepsis, the *dynamic* prognostic potential of several features, the single-group design, based on using historical data from the subjects themselves as control-state was not a major limitation. However, caution should be exercised in interpreting the results of the study – the model predicts the *relative* risk of sepsis in the hours leading up to sepsis and not the *absolute* risk thereof (recall that the priors of the classifier were uniform). Nonetheless, extending the model to predict the *absolute* risk is straightforward. This would require titrating the priors of the Naïve Bayesian model, for each infant, to reflect the propensity towards sepsis, in a manner similar to risk stratification. Assignment of such priors can be based on modeling (regression analysis would be a good choice) the risk of sepsis based on longitudinally collated infant metadata data such as birth weight, GA, postnatal age as well as maternal characteristics, from, for instance, regional or national databases. A limitation of this study based on the single-group nature of the design is that the model developed herein probes the regulatory systems of the infant, both, the autonomic nervous system and the motor system, for subclinical signs of dysregulation. Likely, the model is therefore sensitive not just to sepsis alone but also events such as mechanical intubation and illnesses such as necrotizing enterocolitis and cerebral hemorrhage that affect cardiorespiratory stability – this, however, cannot be ascertained without incorporating an alternative control-arm into the study[41]. As in this study, only two infants were intubated in the pre-CRASH period; the effect of mechanical ventilation on the current findings is minimal and does not constitute a limitation. Future directions for testing the model include analysis for infants whose blood culture was negative as well as model validation for predictive monitoring of LOS in prospective studies.

V. Conclusion

In the hours leading up to the clinical diagnosis of late-onset neonatal sepsis, multiple regulatory systems including the autonomic nervous system and the motor system exhibit subclinical signatures of disease that may not be visibly apparent to caregivers. Compared to baseline, in the hours leading up to the clinical suspicion of blood-culture positive sepsis, HRV exhibited extensive decelerations and an inability to accelerate the heart in response to pathological heart rate decelerations. Concurrently, the dynamic range of respiration increased along with characteristic signatures of dysregulation of the respiratory drive. At the same time, ECG-derived estimates of infant motion were lowered, reflecting a pathological lack of spontaneous body movement and disruption in the functioning of the motor system. Quantitative estimates of respiratory drive and lethargy, in addition to the previously described changes in HRV, are novel and can be used to continuously and non-invasively probe an infant's regulatory state, the disruption of which is an early marker of sepsis.

2168-2194 (c) 2019 IEEE. Personal use is permitted, but republication/redistribution requires IEEE permission. See http://www.ieee.org/publications_standards/publications/rights/index.html for more information.

This article has been accepted for publication in a future issue of this journal, but has not been fully edited. Content may change prior to final publication. Citation information: DOI 10.1109/JBHI.2019.2927463, IEEE Journal of Biomedical and Health Informatics

VI. APPENDIX

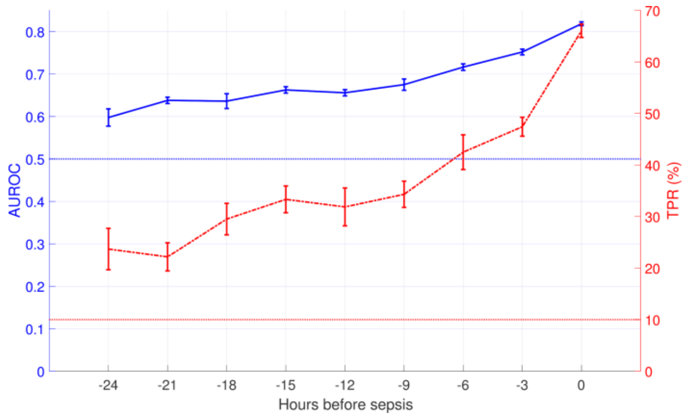

Figure: The performance of the Naïve Bayesian model for identifying sepsis while employing the combination of the two best-performing features. In comparison to the use of a single feature (Table 3 and Figure 2), the TPR of the classifier improves, especially early in the prediction-window, without any appreciable decrease in the AUROC.

REFERENCES

[1] A. L. Shane and B. J. Stoll, "Neonatal sepsis: Progress towards improved outcomes," *J. Infect.*, vol. 68, pp. S24–S32, 2014.

[2] Y. Dong and C. P. Speer, "Late-onset neonatal sepsis:Recent developments," *Arch. Dis. Child. Fetal Neonatal Ed.*, vol. 100, no. 3, pp. F257–F263, 2015.

[3] A. L. Shane, P. J. Sánchez, and B. J. Stoll, "Seminar Neonatal sepsis," *Lancet*, pp. 1–11, 2017.

[4] W. H. Lim *et al.*, "Prevalence and pathogen distribution of neonatal sepsis among very-low-birth-weight infants," *Pediatrics and Neonatology*, vol. 53, no. 4. pp. 228–234, 2012.

[5] A. Gordon and H. E. Jeffery, "Antibiotic regimens for suspected late onset sepsis in newborn infants," in *Cochrane Database of Systematic Reviews*, 2005.

[6] H. Blencowe *et al.*, "Born too soon: the global epidemiology of 15 million preterm births.," *Reprod. Health*, vol. 10 Suppl 1, no. Suppl 1, p. S2, 2013.

[7] E. H. Verstraete, K. Blot, L. Mahieu, D. Vogelaers, and S. Blot, "Prediction Models for Neonatal Health Care-Associated Sepsis: A Meta-analysis," *Pediatrics*, vol. 135, no. 4, pp. e1002–e1014, 2015.

[8] O. Zwang and R. K. Albert, "Analysis of strategies to improve cost effectiveness of blood cultures.," *J. Hosp. Med.*, vol. 1, no. 5, pp. 272–276, 2006.

[9] H. W. Kilbride *et al.*, "Evaluation and Development of Potentially Better Practices to Prevent Neonatal Nosocomial Bacteremia," *Pediatrics*, vol. 111, no. 4, p. e504, 2003.

[10] F. J. Bohanon *et al.*, "Heart rate variability analysis is more sensitive at identifying neonatal sepsis than conventional vital signs," *Am. J. Surg.*, vol. 210, no. 4, pp. 661–667, 2015.

[11] K. Madaras-Kelly, "Optimizing Antibiotic Use in Hospitals: The Role of Population-Based Antibiotic Surveillance in Limiting Antibiotic Resistance: Insights from the Society of Infectious Diseases Pharmacists," *Pharmacotherapy*, vol. 23, no. 12 I. pp. 1627–1633, 2003.

[12] A. Das, S. Shukla, N. Rahman, D. Gunzler, and N. Abughali, "Clinical Indicators of Late-Onset Sepsis Workup in Very Low-Birth-Weight Infants in the Neonatal Intensive Care Unit," *Am J Perinatol*, vol. 33, no. 212, pp. 856–860, 2016.

[13] K. D. Fairchild, "Predictive monitoring for early detection of sepsis in neonatal ICU patients," *Curr. Opin. Pediatr.*, vol. 25, no. 2, pp. 172–179, 2013.

[14] K. D. Fairchild, V. Srinivasan, J. Randall Moorman, R. P. A. Gaykema, and L. E. Goehler, "Pathogen-induced heart rate changes associated with cholinergic nervous system activation," *AJP Regul. Integr. Comp. Physiol.*, vol. 300, no. 2, pp. R330–R339, 2011.

[15] L. B. Mithal, R. Yogev, H. L. Palac, D. Kaminsky, I. Gur, and K. K. Mestan, "Vital signs analysis algorithm detects inflammatory response in premature infants with late onset sepsis and necrotizing enterocolitis," *Early Hum. Dev.*, vol. 117, no. January, pp. 83–89, 2018.

[16] J. R. Moorman *et al.*, "Mortality reduction by heart rate characteristic monitoring in very low birth weight neonates: a randomized trial," *J Pediatr*, vol. 159, no. 6, pp. 900–6 e1, 2011.

[17] M. P. Griffin, D. E. Lake, T. M. O'Shea, and J. R. Moorman, "Heart Rate Characteristics and Clinical Signs in Neonatal Sepsis," *Pediatr. Res.*, vol. 61, no. 2, pp. 222–227, Feb. 2007.

[18] I. Stanculescu, C. K. I. Williams, and Y. Freer, "Autoregressive Hidden Markov Models for the Early Detection of Neonatal Sepsis," *IEEE J. Biomed. Heal. Informatics*, vol. 18, no. 5, pp. 1560–1570, 2014.

[19] B. A. Sullivan and K. D. Fairchild, "Predictive monitoring for sepsis and necrotizing enterocolitis to prevent shock," *Semin. Fetal Neonatal Med.*, vol. 20, no. 4, pp. 255–261, 2015.

[20] S. Mani *et al.*, "Medical decision support using machine learning for early detection of late-onset neonatal sepsis," *J. Am. Med. Informatics Assoc.*, vol. 21, no. 2, pp. 326–336, 2014.

[21] B. A. Sullivan *et al.*, "Early Pulse Oximetry Data Improves Prediction of Death and Adverse Outcomes in a Two-Center Cohort of Very Low Birth Weight Infants," *Am. J. Perinatol.*, vol. 1, no. 212, 2018.

[22] A. Ohlin, M. Björkqvist, S. M. Montgomery, and J. Schollin, "Clinical signs and CRP values associated with blood culture results in neonates evaluated for suspected sepsis," *Acta Paediatr. Int. J. Paediatr.*, vol. 99, no. 11, pp. 1635–1640, 2010.

[23] R. E. Rosenberg *et al.*, "Nosocomial sepsis risk score for preterm infants in low-resource settings," *J. Trop. Pediatr.*, vol. 56, no. 2, pp. 82–89, 2009.

[24] R. Joshi *et al.*, "A Ballistographic Approach for Continuous and Non- Obtrusive Monitoring of Movement in Neonates Journal:," *IEEE J. Transl. Eng. Heal. Med.*, vol. PP, pp. 1–30, 2018.

[25] D. Vermont Oxford Network, "Manual of Operations: Part 2 Data Definitions and Infant Data Forms. [https://public.vtoxford.org/]. Updated 2015 November. Accessed Oct 2018.," *Vermont Oxford Network Database*. 2015.

[26] M. P. Griffin and J. R. Moorman, "Toward the Early Diagnosis of Neonatal Sepsis and Sepsis-Like Illness Using Novel Heart Rate Analysis," *Pediatrics*, vol. 107, no. 1, pp. 97–104, 2001.

[27] M. J. Rooijakkers, C. Rabotti, S. G. Oei, and M. Mischi, "Low-complexity R-peak detection for ambulatory fetal monitoring," *Physiol. Meas.*, vol. 33, no. 7, pp. 1135–1150, 2012.

[28] H. Lee *et al.*, "A new algorithm for detecting central apnea in neonates," *Physiol. Meas.*, vol. 33, no. 1, pp. 1–17, 2012.

[29] S. M. Pincus, "Approximate entropy as a complexity measure," *Chaos*, vol. 5, no. 1, pp. 110–117, 1995.

2168-2194 (c) 2019 IEEE. Personal use is permitted, but republication/redistribution requires IEEE permission. See http://www.ieee.org/publications_standards/publications/rights/index.html for more information.

This article has been accepted for publication in a future issue of this journal, but has not been fully edited. Content may change prior to final publication. Citation information: DOI 10.1109/JBHI.2019.2927463, IEEE Journal of Biomedical and Health Informatics

[30] R. Joshi *et al.*, “A Ballistographic Approach for Continuous and Non-Obtrusive Monitoring of Movement in Neonates,” *IEEE J. Transl. Eng. Heal. Med.*, pp. 1–30, 2018.

[31] D. R. Kommers *et al.*, “Features of Heart Rate Variability Capture Regulatory Changes During Kangaroo Care in Preterm Infants,” *J. Pediatr.*, vol. 182, pp. 92-98.e1, 2017.

[32] R. Joshi *et al.*, “Cardiorespiratory coupling in preterm infants,” *J. Appl. Physiol.*, vol. 126, no. 1, pp. 202–213, 2018.

[33] A. Bauer *et al.*, “Phase-rectified signal averaging detects quasi-periodicities in non-stationary data,” *Phys. A Stat. Mech. its Appl.*, vol. 364, pp. 423–434, 2006.

[34] B. P. Kovatchev, L. S. Farhy, H. Cao, M. P. Griffin, D. E. Lake, and J. R. Moorman, “Sample Asymmetry Analysis of Heart Rate Characteristics with Application to Neonatal Sepsis and Systemic Inflammatory Response Syndrome,” *Pediatr. Res.*, vol. 54, no. 6, pp. 892–898, 2003.

[35] C. Fleischmann-Struzek, D. M. Goldfarb, P. Schlattmann, L. J. Schlapbach, K. Reinhart, and N. Kissoon, “The global burden of paediatric and neonatal sepsis: a systematic review,” *The Lancet Respiratory Medicine*, vol. 6, no. 3. pp. 168–170, 2018.

[36] K. D. Fairchild and T. M. O. Shea, “Heart Rate Characteristics: Physiomarkers for Detection of Late- Onset Neonatal Sepsis,” *Clin Perinatol*, vol. 37, no. 3, pp. 581–598, 2011.

[37] M. Mahallei, M. A. Rezaee, B. Mehramuz, S. Beheshtirooy, and B. Abdinia, “Clinical symptoms, laboratory, and microbial patterns of suspected neonatal sepsis cases in a children’s referral hospital in northwestern Iran.,” *Medicine (Baltimore).*, vol. 97, no. 25, p. e10630, 2018.

[38] M. W. Weber, J. B. Carlin, S. Gatchalian, D. Lehmann, L. Muhe, and E. K. Mulholland, “Predictors of neonatal sepsis in developing countries,” *Pediatr. Infect. Dis. J.*, vol. 22, no. 8, pp. 711–717, 2003.

[39] J. Keim-Malpass *et al.*, “Advancing Continuous Predictive Analytics Monitoring: Moving from Implementation to Clinical Action in a Learning Health System,” *Crit. Care Nurs. Clin. North Am.*, vol. 30, no. 2, pp. 273–287, 2018.

[40] S. Y. P. K. Shiao and C. N. Ou, “Validation of oxygen saturation monitoring in neonates,” *Am. J. Crit. Care*, vol. 16, no. 2, pp. 168–178, 2007.

[41] K. D. Fairchild *et al.*, “Vital signs and their cross-correlation in sepsis and NEC: A study of 1,065 very-low-birth-weight infants in two NICUs,” *Pediatr. Res.*, vol. 81, no. 2, pp. 315–321, 2017.

2168-2194 (c) 2019 IEEE. Personal use is permitted, but republication/redistribution requires IEEE permission. See http://www.ieee.org/publications_standards/publications/rights/index.html for more information.
