# Supplementary material for: Exploring Computational Techniques in Preprocessing Neonatal Physiological Signals for Detecting Adverse Outcomes: Scoping Review
Source: Interact J Med Res. 2024 Aug 20;13:e46946. doi: 10.2196/46946 (PMC11372324; doi:10.2196/46946)
Supplement: Multimedia Appendix 3 [file ijmr_v13i1e46946_app3.zip › Included Papers - Final/2991/G. Varisco et al. - 2021 - Optimized Detection of Central Apneas Preceding La.pdf]

2021 43rd Annual International Conference of the
IEEE Engineering in Medicine & Biology Society (EMBC)
Oct 31 - Nov 4, 2021. Virtual Conference

2021 43rd Annual International Conference of the IEEE Engineering in Medicine & Biology Society (EMBC) | 978-1-7281-1179-7/21/$31.00 ©2021 IEEE | DOI: 10.1109/EMBC46164.2021.9629528

Optimized Detection of Central Apneas Preceding Late-Onset Sepsis in Premature Infants

Gabriele Varisco[1], Deedee Kommers[2], Xi Long[3], Zhuozhao Zhan[4], Marina M. Nano[5], Ward Cottaar[6], Peter Andriessen[2] and Carola van Pul[1]

***Abstract*— In neonatal intensive care units, respiratory traces of premature infants developing late onset sepsis (LOS) may also show episodes of apneas. However, since clinical patient monitors often underdetect apneas, clinical experts are required to investigate patients' traces looking for these events. In this work we present a method to optimize an existing algorithm for central apnea (CA) detection and how we used it together with human annotations to investigate the occurrence of CAs preceding LOS.**

The algorithm was optimized by using a previously-annotated dataset consisting of 90 hours, extracted from 10 premature infants. This allowed to double precision (19.7% vs 9.3%, median values per patient) without affecting recall (90.5% vs 94.5%) compared to the original algorithm. This choice caused the missed identification of just 1 additional CA (4 vs 3) in the whole dataset. The optimized algorithm was then used to annotate a second dataset consisting of 480 hours, extracted from 10 premature infants diagnosed with LOS. Annotations were corrected by two clinical experts.

A significantly higher number of CA annotations was found in the 6 hours prior to sepsis onset (p-value $<$ 0.05). The use of the optimized algorithm followed by human annotations proved to be a suitable, time-efficient method to annotate CAs before sepsis in premature infants, enabling future use in large datasets.

I. INTRODUCTION

Infants born very prematurely are characterized by an immature respiratory system upon delivery. As a consequence, these infants require hospitalization in neonatal intensive care units (NICUs) [1], [2]. In particular, an immature respiratory system may result in apneic events, or so called apnea of prematurity [3]–[6]. Previous studies indicated an increase in the number of apneas preceding sepsis, an infection which is among the leading cause of premature death in NICUs [7]–[10]. Therefore, apnea detection is important to adjust the therapeutic strategy to prevent the occurrence of sepsis.

[1] Gabriele Varisco and Carola van Pul are with the Faculty of Applied Physics, Eindhoven University of Technology, 5612 AZ Eindhoven, The Netherlands and with the department of Clinical Physics, Máxima Medical Center, 5504 DB Veldhoven, The Netherlands, g.varisco@tue.nl

[2] Deedee Kommers and Peter Andriessen are with the Faculty of Applied Physics, Eindhoven University of Technology, 5612 AZ Eindhoven, The Netherlands and with the department of Pediatrics, Máxima Medical Center, 5504 DB Veldhoven, The Netherlands

[3] Xi Long is with the Faculty of Electrical Engineering, Eindhoven University of Technology, 5612 AZ Eindhoven, The Netherlands and with Philips Research, 5656 AE Eindhoven, The Netherlands

[4] Zhuozhao Zhan is with the Faculty of Mathematics and Computer Science, Eindhoven University of Technology, 5612 AZ Eindhoven, The Netherlands

[5] Marina M. Nano is with the Faculty of Electrical Engineering, Eindhoven University of Technology, 5612 AZ Eindhoven, The Netherlands

[6] Ward Cottaar is with the Faculty of Applied Physics, Eindhoven University of Technology, 5612 AZ Eindhoven, The Netherlands

The definition of apnea of prematurity has often been debated in the past due to a lack of consensus among clinical experts [2], [3], [11]. Apneas are currently most often defined as cessations of breathing longer than 20 seconds, or longer than 10 seconds accompanied by a bradycardia (heart rate $\leq$ 100 beats/minute) and/or desaturation (SpO2 $\leq$ 80%) [2], [4]. Apneas can be further classified into different types. These include central apneas (CAs, absence of respiratory effort due to a cessation of output from the central respiratory centers), obstructive apneas (indicated by an obstruction in the upper airway which causes inadequate respiratory efforts to maintain ventilation) and mixed apneas (which include characteristics of both previous groups) [3], [7], [12], [13].

Different algorithms have been developed for automatic detection of apnea (e.g. in patient monitoring systems) [12], [14]–[17]. However, since patient signals show high inter-patient and intra-patient variability, such automated apnea detection often fails, particularly in premature infants [12], [17]. Research to improve algorithms for the detection of apnea of prematurity is thus ongoing [15], [16]. For instance, Lee and colleagues proposed an algorithm focusing primarily on CA detection, with a key feature being the removal of the cardiac signal from the chest impedance (CI) as that may falsely be detected as respiration effort [12], [14], [17].

The aim of our study is to facilitate the time consuming detection of CAs in data tracings of premature infants by optimizing Lee's detection algorithm [12], [14], [17] and investigating its ability to detect CAs preceeding late-onset sepsis (LOS), defined as sepsis with an onset after the first 72 hours of life [9], [10]. This algorithm was selected for the use in this study due to its simplicity in the implementation and since, compared to other algorithms that make use of several additional vital signs to predict sepsis onset [18], [19], it only uses the information included in CI and RR-intervals (the latter is used to filter out the cardiac artifact from CI) for the specific identification of CAs [12].

II. METHODS

A. Study Design

The improvement of CA detection was addressed in the following two steps, as presented in Fig. 1(a). First, Lee's algorithm [12], [14], [17] was optimized using a first dataset (training dataset), by comparing all central apnea-suspected events (CASEs) returned by the algorithm with apnea annotations by two clinical experts. Second, the optimized algorithm was applied in a second dataset (sepsis dataset), containing data from premature infants that developed LOS.

Authorized licensed use limited to: CSIRO Information Technology Services. Downloaded on September 09,2022 at 00:40:50 UTC from IEEE Xplore. Restrictions apply.

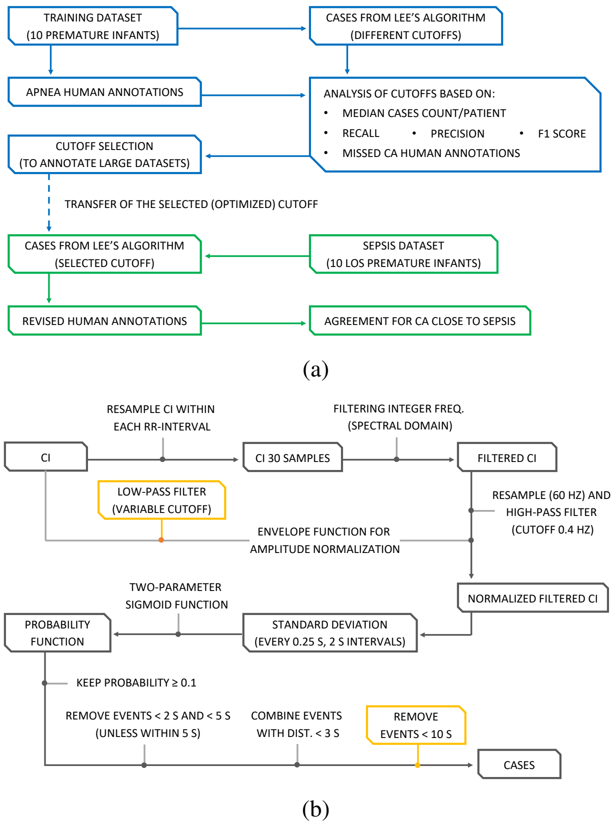

Fig. 1. (a) Workflow of the steps included in the current study and (b) steps included in Lee's algorithm for central apnea detection. Steps that led to the optimized cutoff for the envelope function of Lee's algorithm are represented in blue in (a), whereas steps to evaluate the agreement for CA before sepsis onset are represented in green. Steps that were optimized or added in this work for Lee's algorithm are highlighted in orange in (b). Abbreviations: CI - chest impedance, CASE - central apnea suspected events, CA - central apnea

Two clinical experts examined the vital signs during episodes returned as CASEs by the algorithm, and annotated CAs. CASEs as well as matching CAs were evaluated as a function of the distance from sepsis onset.

As this study had a retrospective nature, a waiver was provided by the medical ethical committee in accordance with the Dutch law on medical research with humans (WMO).

B. Study Datasets

Training dataset: This dataset included 10 premature infants (gestational age 28.5 ± 1.6 weeks) undergoing continuous cardiorespiratory monitoring in the NICU of Máxima Medical Center (MMC, Veldhoven, The Netherlands). Patients' characteristics are presented in Table I. A total of 90 hours of continuous data was extracted from these patients. ECG, CI and oxygen saturation (SpO2) were collected from NICU bedside monitors (GE Healthcare, models Solar 8000M and I), with both ECG and CI waveforms tracked using three ECG leads with electrodes on both sides of the heart. ECG was collected at 240 Hz, whereas the CI was collected at 60 Hz. SpO2 was captured by PPG at 0.5 Hz.

Sepsis dataset: This dataset included 10 premature infants

TABLE I
CHARACTERISTICS FOR ALL PATIENTS INCLUDED IN BOTH DATASETS

Dataset	Training	Sepsis
Gestational Age (wk)	28.5 ± 1.6	27.9 ± 0.9
Postnatal Days	7.0 ± 7.0	10.2 ± 8.1
Birth Weight (g)	1041.5 ± 203.4	998.0 ± 215.9
Sex	7M, 3F	5M, 5F
Hours per Patient	9	48
Total Hours	90	480

(gestational age 27.9 ± 0.9 weeks) diagnosed with LOS. All patients were part of a sepsis cohort consisting of patients admitted to the NICU of MMC [8]. Patients' characteristics are presented in Table I. Their data was collected for all 48 hours prior to the onset of sepsis (when cultures, resuscitation and antibiotics started), which allowed to extract a total of 480 hours of data. ECG, CI and SpO2 were monitored using the Philips IntelliVue MX800 patients' monitors (Philips Medical Systems, Böblingen, Germany), according to clinical standard. ECG and CI were measured using three ECG leads. ECG was collected at 250 Hz, whereas CI was collected at 62.5 Hz. SpO2 was captured by PPG at 1 Hz.

C. Detection of central apnea-suspected events based on Lee's algorithm

Starting point of our work was the central apnea detection algorithm described by Lee [12], [14], [17], that generated a filtered respiration signal without cardiac artifacts. The workflow including all the steps for this algorithm is presented in Fig. 1(b). For each RR-interval, the corresponding CI was resampled to provide 30 equidistant samples. The Fourier transform was computed and the frequency band at the integer frequencies was filtered out to remove the cardiac artifact. The signal resulting from inverse transformation was then resampled at 60 Hz and filtered with a high-pass filter with a cutoff frequency of 0.4 Hz, to remove low-frequency artifacts. The amplitude of the resulting signal was finally adjusted using an envelope function. This was computed by applying a low-pass filter with a very low cutoff frequency at the original CI. This was done to normalize the amplitude of the resulting filtered respiration, so that results for different patients could be compared. The value proposed by Lee et al. for this cutoff was 0.0025 Hz. In this study, however, we optimized its value to fit apnea annotations provided by clinical experts from our group for the training dataset.

The standard deviation of the filtered respiration was then computed every quarter of a second for centered 2 second intervals. Apnea probability was defined by applying a two-parameter sigmoid function, resulting in a continuous probability function. Events starting when apnea probability exceeded 0.1 and ending when it decreased below this value were detected. After removing events shorter than 2 seconds, events shorter than 5 seconds were discarded unless they were within 5 seconds of another event. Finally, all events that were separated by less than 3 seconds were combined into one single event, referred as CASEs in this work. Based

Authorized licensed use limited to: CSIRO Information Technology Services. Downloaded on September 09,2022 at 00:40:50 UTC from IEEE Xplore. Restrictions apply.

on apnea definition from [2], [4], only CASEs longer than 10 seconds were considered for further human annotations for CAs.

D. Central Apnea Annotations by Clinical Experts

Training dataset: This dataset had previously been annotated by two clinical experts from MMC. Annotations were performed according to the definition of apnea of prematurity, irrespectively of its type, provided in [2], [4]. The presence of apnea was evaluated using 3-minute windows from this dataset, visualized by means of an in-house annotation software developed using Matlab (The MathWorks, Natick, USA). The software interface comprised five physiological signals: ECG, SpO2, RR-intervals (derived from ECG), CI and respiration rate (derived from CI). All included physiological signals were presented using 3-minute windows. An exception was the ECG signal, for which just the last 10 seconds of the 3-minute windows were presented. The annotators had the option in each window to generate an apnea annotation by indicating its start and end on the CI.

Sepsis dataset: For this dataset the optimized algorithm was used to annotate CAs from CASEs. We developed a new annotation software using Matlab to resemble the representation returned by a patient monitor and to allow for the identification of CAs. All the characteristics of this software interface are presented in Fig. 2. Two clinical experts provided annotations for apneic events with a central origin using this annotation software. Information extracted from multiple signals (e.g. CI, HR, SpO2) was delivered to the clinical experts to provide accurate annotations.

Different annotation-options were discussed with the clinical experts and the following labels were created: 'central apnea' (a trace of flat CI signal following previously regular fluctuating CI), 'rejection' (flat CI trace, however mixed with large CI fluctuations) and 'error' (missing or corrupt signal).

E. Data analysis and Statistical analysis

Training dataset: All human annotations longer than 10 seconds were identified. Matching apnea annotations were then defined in case the annotations by the different clinical experts presented an overlap for more than 1 second (60 samples). To determine the optimal settings for Lee's algorithm, different cutoffs for the envelope function were evaluated to maximize the level of agreement with matching apnea annotations. Furthermore, the two clinical experts who annotated sepsis dataset were asked to annotate CAs among all matching apnea annotations missed by the algorithm with different cutoffs in the sepsis dataset. The optimal cutoff was defined after clinical consensus considering a trade-off among five parameters: the median counts of CASEs per patient, median recall, median precision, median F1-score (harmonic mean of recall and precision) as well as the total count (sum considering all patients) of missed matching CAs.

Sepsis dataset: The total count of CASEs, matching CAs agreed by both annotators and their median counts per patient were investigated. The count of CASEs, CAs annotated by either annotators (either CAs) and matching CAs was performed using 2-hour windows and a step length of 1 hour. This solution was used to avoid considerations for single hours, since consecutive hours often presented high differences in the count of CASEs.

Then we investigated if the percentage of agreement (ratio between matching CAs and CASEs computed per patient using the same moving window) was influenced by the time-distance from sepsis onset by computing its median value over the different hours. A Cochran-Mantel-Haenszel test was used to evaluate differences between the count of matching CAs found in hours close to sepsis onset and the count found in hours at further time-distance. Differently from a Chi-Square test, this test allows for an analysis of these counts stratified per patient albeit it assumes that the difference in terms of odds ratio is the same across all patients [20].

Data analyses were performed using Matlab for both datasets whereas statistical analyses were performed using R (R Core Team, Vienna, Austria). A p-value < 0.05 was considered significant.

III. RESULTS

Training dataset: A total of 259 matching apnea annotations (i.e. annotations of the two different annotators matching by at least one second) were found (median count of matching apnea annotations per patient 21). When running Lee's algorithm on this dataset, the median count of CASEs was much higher, depending on the cutoff for the envelope function.

Since five parameters related to different cutoffs were analyzed, these will be indicated following the order presented in the method section, for an easier comprehension: median count of CASEs per patient, median recall, median precision, median F1-score and total count of missed matching CAs. First, for the cutoff 0.0025, value suggested by Lee et al. [12] and represented with an orange vertical line in Fig. 3, a median count of 147 CASEs per patient was found (238/259 identified matching apnea annotations), together with a median recall of 94.5%, a median precision of 9.3%, a median F1-score of 16.9% and 3 missed matching CAs in total for all patients. For the cutoff 0.0030, represented with a red vertical line in Fig. 3, the following parameters were extracted: a median count of 144 CASEs per patient (240/259 identified matching apnea annotations), a median recall of 95.5%, a median precision of 9.6%, a median F1-score of 17.4% and 2 missed matching CAs in total. Despite this cutoff showed the maximum median recall, being able to detect most of matching apnea annotations, it also showed a very low precision, structurally detecting a high number of CASEs that were not marked as apneas by the annotators.

To reduce false apnea detection, we aimed for a true to false ratio for CASEs equal to 1:5 (precision = 0.2) and identified 0.0005 as optimal cutoff. For this cutoff, represented with a green vertical line in Fig. 3, the following parameters were extracted: a median count of 75 CASEs per patient (222/259 identified matching apnea annotations),

Authorized licensed use limited to: CSIRO Information Technology Services. Downloaded on September 09,2022 at 00:40:50 UTC from IEEE Xplore. Restrictions apply.

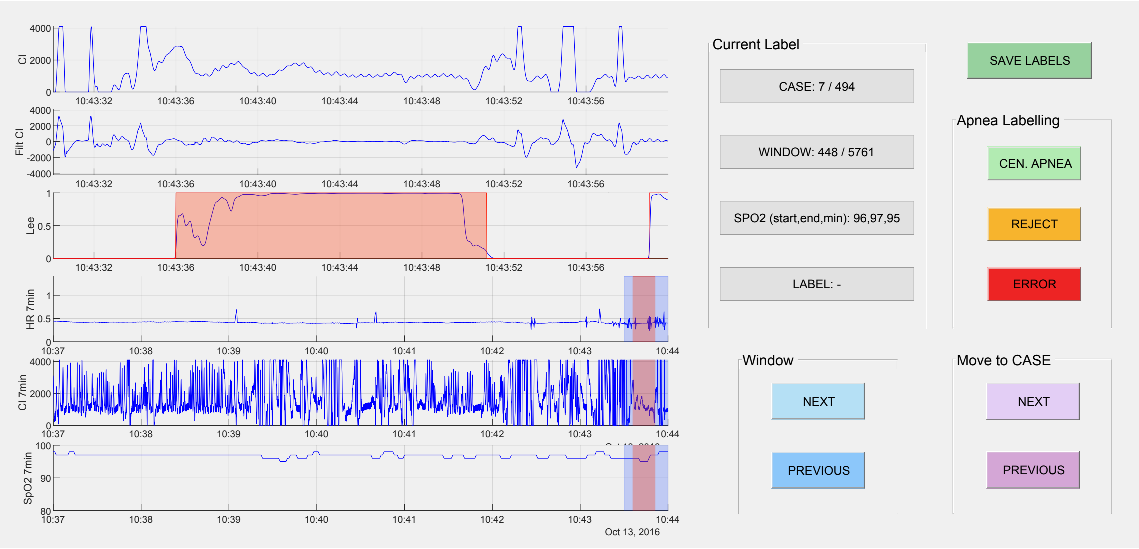

Fig. 2. Representation of the software annotation used to provide clinical experts' annotations for sepsis dataset based on central apnea suspected events (CASEs) provided by Lee's algorithm. This software includes 30-second representations for CI, filtered respiration, apnea probability computed using Lee's algorithm. RR-intervals, SpO2 and also CI are represented using 7-minute windows. The starting and ending SpO2 values registered for each annotation from Lee's algorithm as well as the minimum recorded value found within the annotation are indicated in a grey string.
The current CASE provided by Lee's algorithm is represented in red and is superimposed to all the 7-minute windows. The light blue area is instead used to indicate the position of the 30-second windows in the 7-minute windows. The different annotations options are presented on the far right. These are 'central apnea', 'reject' and 'error'. Whenever a choice is made, this is indicated after the string 'label'.
Information about the number of the current CASE as well as the time reference to the whole 48 hours for the selected patients are indicated in gray. 'Save labels' allow the annotator to save all previous annotations

a median recall of 90.5%, a median precision of 19.7%, a median F1-score of 30.8% and 4 missed matching CAs in total. Considering the significant reduction in the count of CASEs returned by the algorithm (improved precision) at the cost of losing just 1 additional matching CA, this cutoff was found to be suitable to annotate large datasets. Despite the median F1-score computed with a cutoff equal to 0.0005 showed a lower value compared to its maximum, as this metric penalizes precision and recall disagreeing with each other too much [21], [22], this value was found to be almost doubled compared to the F1-score computed using the original cutoff, proving to be a suitable choice for the use with sepsis dataset.

Sepsis dataset: A total of 4773 CASEs were detected by the optimized algorithm (median count per patient 537). A total of 772 matching CAs (i.e. CAs agreed on by both annotators) were detected (median count per patient 47).

Time dependencies for the annotations were evaluated by considering the median count of CASEs, either CAs and matching CAs per 2-hour windows, as shown in Fig. 4. A median count of 15 CASEs per patient per 2-hour windows was found, with higher counts located in proximity to sepsis onset (last 6 hours). The median count of either CAs and matching CAs also showed higher values closer to sepsis. Percentage of agreement (ratio between matching CAs and CASEs computed per patient) showed higher values in the 6 hours close to sepsis onset as well as very low values in the preceding hours (10-7 hours before sepsis onset). The counts of matching CAs for the last 6 hours prior to sepsis onset were found to be statistically higher than all previous hours by using the Cochran-Mantel-Haenszel test ($p\text{-value} < 0.05$).

IV. DISCUSSION

In this study we optimized an automatic algorithm to facilitate human annotations for CAs in a dataset of premature infants and used it to detect CAs and to evaluate agreement for CAs preceeding LOS. This study proposes a method to tune Lee's algorithm for CA detection for the use in diverse and large datasets of premature infants.

Algorithm optimization was possible by establishing an optimal cutoff for the envelope function (0.0005). This choice provided only a slight loss of recall (90.5% vs 94.5%, median values per patient) but resulted in an improved precision (19.7% vs 9.3%) and F1-score (30.8% vs. 16.9%). The optimized algorithm returned therefore less than half CASEs per patient for further clinical annotations compared to when the original cutoff was used, significantly reducing the time needed by clinical experts to annotate a dataset. Since availability of clinical experts is always limited, we considered the increase of precision a valuable improvement for the algorithm. This result was obtained at the price of missing just one additional matching CA in the whole training dataset (4 vs 3).

A software annotation tool was created in close contact with clinicians to allow for the detection of CAs among all CASEs. The solution proposed in this work considers both the need to present information in a similar way to the one commonly shown by patient monitors and to include relevant information for the detection of CAs.

Authorized licensed use limited to: CSIRO Information Technology Services. Downloaded on September 09,2022 at 00:40:50 UTC from IEEE Xplore. Restrictions apply.

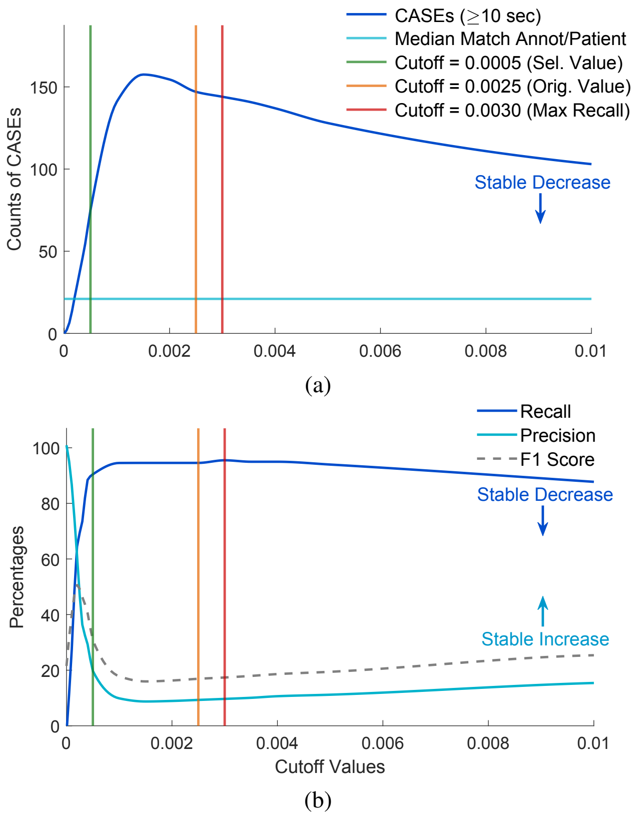

Fig. 3. (a) Median count of central apnea-suspected events (CASEs) and (b) median precision, recall and F1-score per patient provided by comparing CASEs with matching apnea annotations (provided by clinical experts) from the training dataset. Both figures are generated considering different cutoffs for the envelope function in Lee's algorithm. The optimal cutoff (0.0005) selected for the analysis in sespis dataset is indicated with a green line. The original choice selected by Lee indicated is with an orange line whereas the cutoff with the highest recall is indicated is with a red line

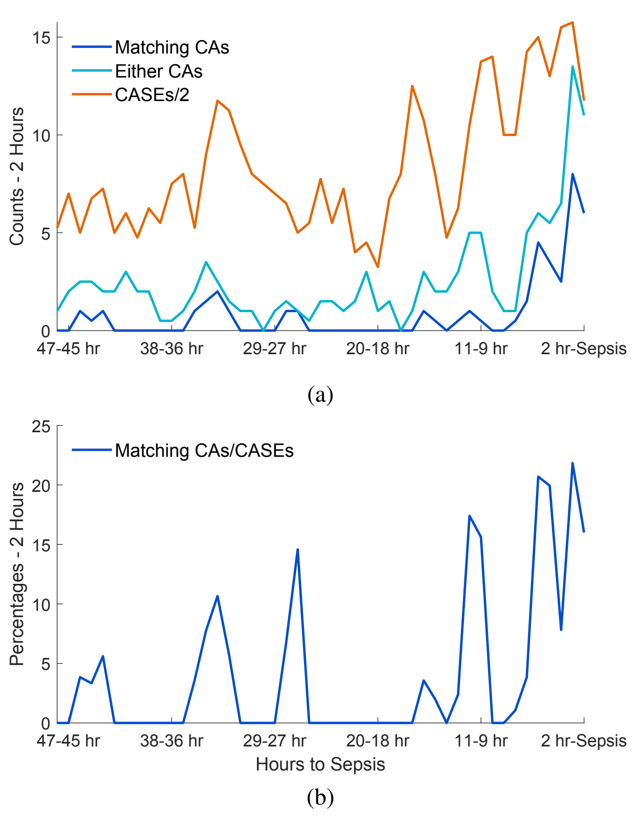

Fig. 4. (a) Median count of central apnea-suspected events (CASEs) (divided by 2, to allow for scale comparison with the following measures) provided by Lee's algorithm, central apneas (CAs) annotated by either annotators (either CAs) and agreed on (matching CAs) and (b) median percentage agreement computed per patient as ratio between matching CAs and CASEs. All measures were computed using 2-hour windows and a step length of 1 hour

The influence of the time to sepsis on CASEs and CAs was then evaluated. The optimized algorithm returned a higher count of CASEs per 2-hour windows in the last 6 hours prior to sepsis onset. The count of matching CAs in the last 6 hours was found to be statistically higher when compared to all previous hours (p-value < 0.05). This difference was also reflected by the percentage of agreement, a result which could indicate that apnea characteristics may be more pronounced close to sepsis. Previous works showed that sepsis is preceded by an increase in the number of apneic periods [7]–[9]. A higher agreement for CAs in these hours can therefore be motivated by the association between sepsis and apneas. Interestingly, the effect of sepsis is already visible 6 hours before its onset: future studies may investigate if the presence of a higher number of CAs may be relevant for the prediction of sepsis onset while using different machine learning algorithms.

This study has limitations. First, only the cutoff parameter of the envelope function of Lee's algorithm was optimized. The possibility of using different functions from the two-parameter sigmoid function to compute apnea probability could be further examined. Second, in this work we only focused on CA annotations but both annotators clearly reported that 'rejected' CASEs often would have been categorized as mixed or obstructive apneas. An evaluation of the precision and recall for this type of events could provide additional value to the optimized algorithm.

V. CONCLUSIONS

Annotation of CAs in a large dataset of premature infants is feasible by using the optimized algorithm, which returns CASEs for further human annotations. This optimized algorithm proved to be reliable for annotating CAs (very few CAs missed) while also reducing the time needed by clinical experts to annotate a dataset. The identification of high counts of CAs seems a promising tool to provide additional useful information to help clinical experts predict the onset of sepsis in premature infants.

ACKNOWLEDGMENT

This work was done within the framework of the Eindhoven MedTech Innovation Center (e/MTIC) which is a collaboration of the Eindhoven University of Technology, Philips Research, and MMC. This work is a result of the ALARM project funded by the Nederlandse Organisatie voor Wetenschappelijk Onderzoek (NWO) grant number 15345.

Authorized licensed use limited to: CSIRO Information Technology Services. Downloaded on September 09,2022 at 00:40:50 UTC from IEEE Xplore. Restrictions apply.

References

[1] G. Schmalisch, “Neonatal monitoring,” Capnography, Second Ed., no. 2012, pp. 80–95, 2011.

[2] N. N. Finer, R. Higgins, J. Kattwinkel, and R. J. Martin, “Summary proceedings from the apnea-of-prematurity group,” Pediatrics, vol. 117, no. 3, pp. S47–S51, 2006.

[3] E. C. Eichenwald and Committee on Fetus and Newborn, “Apnea of Prematurity,” Pediatrics, vol. 137, no. 1, 2016.

[4] J. Zhao, F. Gonzalez, and D. Mu, “Apnea of prematurity: From cause to treatment,” Eur. J. Pediatr., vol. 170, no. 9, pp. 1097–1105, 2011.

[5] J. M. Di Fiore, R. J. Martin, and E. B. Gauda, “Apnea of prematurity - Perfect storm,” Respir. Physiol. Neurobiol., vol. 189, no. 2, pp. 213–222, 2013.

[6] A. Janvier, M. Khairy, A. Kokkotis, C. Cormier, D. Messmer, and K. J. Barrington, “Apnea is associated with neurodevelopmental impairment in very low birth weight infants,” J. Perinatol., vol. 24, no. 12, pp. 763–768, 2004.

[7] K. Fairchild et al., “Clinical associations of immature breathing in preterm infants: Part 1-central apnea,” Pediatr. Res., vol. 80, no. 1, pp. 21–27, 2016.

[8] L. Cabrera-Quiros et al., “Prediction of Late-Onset Sepsis in Preterm Infants Using Monitoring Signals and Machine Learning,” Crit. Care Explor., vol. 3, no. 1, p. e0302, 2021.

[9] M. H. Tsai et al., “Recurrent late-onset sepsis in the neonatal intensive care unit: Incidence, clinical characteristics and risk factors,” Clin. Microbiol. Infect., vol. 20, no. 11, pp. O928–O935, 2014.

[10] B. J. Stoll et al., ”Late-onset sepsis in very low birth weight neonates: the experience of the NICHD Neonatal Research Network”. Pediatrics, vol. 110, no. 2, pp. 285-291, 2002.

[11] R. J. Martin, J. M. Abu-Shaweesh, and T. M. Baird, “Apnoea of prematurity,” Paediatr. Respir. Rev., vol. 5, no. SUPPL. A, 2004.

[12] H. Lee et al., “A new algorithm for detecting central apnea in neonates,” Physiol. Meas., vol. 33, no. 1, pp. 1–17, 2012.

[13] S. Picone, R. Aufieri, and P. Paolillo, “Apnea of prematurity: challenges and solutions,” Res. Reports Neonatol., p. 101, 2014.

[14] M. A. Mohr et al., “Very long apnea events in preterm infants,” J. Appl. Physiol., vol. 118, no. 5, pp. 558–568, 2015.

[15] S. Y. Belal, A. J. Emmerson, and P. C. W. Beatty, “Automatic detection of apnoea of prematurity,” Physiol. Meas., vol. 32, no. 5, pp. 523–542, 2011.

[16] J. R. Williamson, D. W. Bliss, D. W. Browne, P. Indic, E. Bloch-Salisbury, and D. Paydarfar, “Using physiological signals to predict apnea in preterm infants,” Conf. Rec. - Asilomar Conf. Signals, Syst. Comput., pp. 1098–1102, 2011.

[17] B. D. Vergales et al., “Accurate automated apnea analysis in preterm infants,” Am. J. Perinatol., vol. 31, no. 2, pp. 157–162, 2014.

[18] K. D. Fairchild, “Predictive monitoring for early detection of sepsis in neonatal ICU patients,” Curr. Opin. Pediatr., vol. 25, no. 2, pp. 172–9, 2013.

[19] N. Kumar, G. Akangire, B. Sullivan, K. Fairchild, and V. Sampath, “Continuous vital sign analysis for predicting and preventing neonatal diseases in the twenty-first century: big data to the forefront,” Pediatr. Res., vol. 87, no. 2, pp. 210–220, 2020.

[20] A. Agresti, An introduction to categorical data analysis, 2nd ed. John Wiley & Sons, 2007.

[21] Y. Sasaki, “The truth of the F-measure,” no. January 2007, pp. 1–6, 2015.

[22] D. J. Hand, P. Christen, and N. Kirielle, “F*: an interpretable transformation of the F-measure,” Mach. Learn., vol. 110, no. 3, pp. 451–456, 2021.

Authorized licensed use limited to: CSIRO Information Technology Services. Downloaded on September 09,2022 at 00:40:50 UTC from IEEE Xplore. Restrictions apply.
