# Supplementary material for: Exploring Computational Techniques in Preprocessing Neonatal Physiological Signals for Detecting Adverse Outcomes: Scoping Review
Source: Interact J Med Res. 2024 Aug 20;13:e46946. doi: 10.2196/46946 (PMC11372324; doi:10.2196/46946)
Supplement: Multimedia Appendix 3 [file ijmr_v13i1e46946_app3.zip › Included Papers - Final/3032/C. León et al. - 2021 - Recurrent Neural Networks for Early Detection of L.pdf]

Recurrent Neural Networks for Early Detection of Late Onset Sepsis in Premature Infants Using Heart Rate Variability

Cristhyne León[1], Patrick Pladys[1], Alain Beuchée[1], Guy Carrault[1]

[1] Univ Rennes, CHU Rennes, Inserm, LTSI - UMR 1099, F-35000 Rennes, France

Abstract

Early diagnosis of late onset sepsis (LOS) in premature infants can help reduce morbidity and mortality in this particularly vulnerable population. In this work, we propose a machine learning model based on recurrent neural networks for the early detection of late onset sepsis in premature infants. The model combines gated recurrent units and long short-term memory units, and uses heart rate variability features as input data. The population used for this study consisted of 259 premature infants; 193 of them were used for training the model, which was then tested in the remaining 66 infants. Thus, we obtained an area under the receiver operating characteristics curve (AUROC) of more than 80% for the 24 hours before the onset of the infection, and reaching 90.4% (95% CI [88.1%, 92.6%]) six hours before the time of the infection. The proposed method has the potential to be easily implemented as a decision support system for real-time LOS detection in neonatal intensive care units, as it uses only data which is continuously and automatically available in such settings.

1. Introduction

Preterm births, defined as live births before 37 completed weeks of gestation, account for approximately 10% of live births worldwide [1]. Late onset sepsis (LOS) is one of the most important risk factors for this population, affecting 10-25% of premature infants, which increases morbidity, mortality, and length of hospital stay [2]. While early diagnosis and antibiotic treatment significantly reduces mortality [3], the indiscriminate use of antibiotics should be avoided as it can have harmful side effects [4]. Thus, early and accurate diagnosis of LOS, leading to adequate use of antibiotics, is pivotal to decrease LOS-related morbidity and mortality among preterm infants.

However, traditional methods and machine learning approaches to diagnose LOS use results from blood cultures and laboratory tests which are invasive, take time, and present variations in their predictive value [5]. Physiological signs, such as changes in heart rate variability (HRV), have been associated with neonatal LOS [6], and previous studies have shown that machine learning algorithms, using HRV as input, can be useful for early LOS detection in infants [7]. Diagnostic tools which rely solely on HRV have the advantage of being non-invasive and continuously available in neonatal intensive care units (NICU).

In a previous study, carried out on a population of 49 premature infants, we showed that a simple machine learning algorithm, using only HRV features as input data, could detect LOS as early as 42 hours before the start of administration of antibiotics, with an area under the receiver operating characteristics curve (AUROC) of 87.7% for the period of six hours before the start of antibiotics [8]. However, that method required significant feature engineering, including a calibration period of 48 hours for each patient, to account for differences between the starting state of the patients and their end state. This is an important consideration when working with preterm infants, given that their HRV characteristics undergo considerable changes as their autonomic nervous system continues to mature [9].

Therefore, we propose the use of recurrent neural networks (RNN). This is a type of artificial neural network specially well suited to analyse time-series and exploit time-dependant patterns. Thus, it would eliminate the need for intensive feature engineering or calibration periods in order to succeed at the task of early LOS detection in premature infants. For this study we used a population of 259 premature infants, from whom we acquired and processed the ECG signal to extract the HRV features. The population was split into a training set, used to train the RNN model, and the test set, used to evaluate the performance of the model. The evaluation was done using the AUROC as the main metric. Finally, we also evaluate results from sample cases to analyse how the proposed model could be used as a decision support system (DSS).

2. Methods

2.1. Population

The data used in this study is part of the Digi-NewB cohort (NCT02863978, EU GA n°689260). The cohort prospectively included infants born between 25 and 42

 ISSN: 2325-887X DOI: 10.22489/CinC.2021.060

Authorized licensed use limited to: CSIRO Information Technology Services. Downloaded on September 09,2022 at 00:40:40 UTC from IEEE Xplore. Restrictions apply.

Table 1: Population

Group	Control	LOS	Total
Train Set	163	30	193
Test set	55	11	66
Total	218	41	259

weeks of gestation, hospitalized in the NICU of six university hospitals in France (University Hospitals of Rennes, Angers, Nantes, Brest, Poitiers, and Tours) in 2017-2019. Data collection was done with approval by the ethics committee (CPP Ouest 6-598) and informed parental consent.

For this study, we considered only the premature infants in the cohort. Further selection of the patients was done retrospectively by a group of experts in neonatal medicine, who classified the infants into either LOS or control group. This classification was done according to the NEO-KISS protocol for nosocomial infection surveillance [10].

The resulting population consisted on 259 infants, of which 218 were in the control group, and 41 in the LOS group. Both groups were further split by randomly choosing 75% of the population of each group for the training set, and the remaining 25% for the test set. The final number of infants in each group is detailed in Table 1.

2.2. Signal Processing and HRV Features Extraction

The ECGs were obtained with a sampling rate of 500Hz. R-peak detection was done with a modified version of the Pan-Tompkins algorithm, with filter coefficients adapted for neonates [11]. Afterwards, the R-R interval time series were extracted and segmented into five minutes periods.

From each of the five minutes segments, we extracted the HRV features, which are categorized in four different types: time-domain, frequency-domain, non-linear measurements [12], and visibility graph indexes. Further detail about the HRV features used in this study and how they were extracted can be found in [8].

Finally, five minutes periods corresponding to 30 continuous minutes were grouped together by calculating the median value of each of their corresponding HRV features. This was done to minimize any noise in the data that could have resulted from artifacts in the ECG. Thus, the final dataset consists of the time series of 28 features that characterize the HRV, sampled in periods of 30 minutes.

2.3. Data labeling

Given that the objective of early diagnosis systems is to reduce delays in the beginning of treatment, we have defined the time of the LOS onset (denoted as t_0) as the time of beginning of administration of antibiotics.

For the patients in the LOS group we included all available data before t_0. Based on the findings of our previous study [8], for the patients in this group we labeled all the time segments within the period of 42 hours before t_0 as LOS, and any previous segments as not infected. For patients in the control group we included all available data up to the eight day of life, so the average length of the features time series for both groups would match. For the purpose of comparison with the LOS group when evaluating the performance of the machine learning model, we assigned the time of the last segment within this period as the t_0 for each patient in the control population.

2.4. Recurrent Neural Network

RNN are a type of artificial neural networks characterized by having recurrent connections [13]. In this type of architecture, each unit has a hidden recurrent state whose activation at each time step depends on the previous step.

The model we implemented consists of an input layer, two hidden layers, and an output layer. The input layer takes the 28 HRV features from one time-step (equivalent to 30 minutes) at a time. The two hidden layers combine different types of RNN units, using gated recurrent units (GRU) [13] in the first hidden layer, and long short-term memory (LSTM) units [13] in the second. The third layer consists of fully-connected units with a sigmoid activation function that returns the probability of LOS for each time-step as output. The model was optimized using binary cross-entropy as the loss function, and the Adam algorithm as optimizer [14].

Because the data is unbalanced, with more infants in the control than in the LOS group, we assigned different weights to the samples of each class in the train set, so that the weighted sum of all the samples of one class is equal to the weighted sum of the other. This helps avoid bias of the loss function in favor of the majority class during training.

3. Results

3.1. Predictive Performance of the Model

Using the same evaluation methods as in [8], the proposed model achieved an AUROC on the test set of 90.4%, with 95% confidence interval (CI) [88.1%, 92.6%], when evaluated on the six-hour window preceding t_0. In Figure 2 we show how the AUROC evaluated on the test set changes on a sliding window of six hours, with a 3-hour overlap, during the 72 hours preceding t_0. We observe that the RNN model has an AUROC above 60% for all time windows. Furthermore, the AUROC is consistently above 70% since 48 hours before t_0, and above 80% for the 24

Authorized licensed use limited to: CSIRO Information Technology Services. Downloaded on September 09,2022 at 00:40:40 UTC from IEEE Xplore. Restrictions apply.

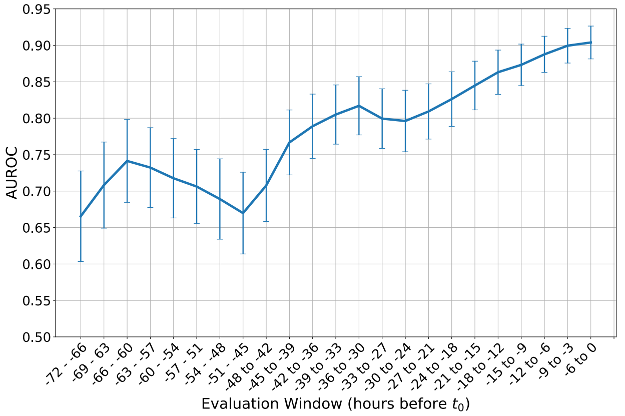

Figure 2: Progress of the AUROC achieved by the RNN model, evaluated on a sliding time window of six hours, with 50% overlap. The error bars represent the 95% CI.

hours before t_0, until it peaks at 90.4% six hours before t_0. We also observe how the range of the CI is smaller for evaluation windows closer to the infection onset, at approximately $\pm 2\%$ for the 12 hours before t_0. It then gets progressively larger for evaluation windows further from t_0, peaking at 6.2% for the earliest evaluation window.

3.2. Sample Cases

To exemplify how the proposed method could be used as a DSS, in Figure 1 we show the performance of the model on three sample cases of patients belonging to the test set. The first, in Figure 1a, is a control patient for whom we observe that the probability of LOS estimated by the model is close to zero for the duration of the entire studied period.

In Figure 1b, we present the results obtained for a patient from the LOS group (LOS group patient 1). As mentioned in Section 2.3, for the patients in this group we labeled the period of the 42 hours before t_0 as infected, and the rest as not infected. However, we observe that the model estimates a very low probability of LOS from the beginning of the studied period and until approximately 20 hours before t_0, at which time it starts detecting a very high probability of LOS for this patient; the probability remains very close to one until the end of the studied period. In contrast, in Figure 1c we observe the results for another patient belonging to the LOS group (LOS group patient 2), for whom the probability of LOS estimated by the model rises at around 80 hours before t_0, and then remains close to one for the reminder of the time.

4. Discussion

The main contribution of this work is the proposal of a machine learning model, based on RNN architecture, that uses only HRV data to produce reliable, early diagnosis of LOS in preterm infants. The model achieves an AUROC of 90.4% for the period of six hours before infection onset. This type of architecture offers the advantage of being able to detect patterns that develop over time, without requiring extensive feature engineering or feature selection processes. While previous studies have suggested the use of RNN for detecting sepsis in adults [15], studies targeted to sepsis diagnosis in neonates using deep learning have mostly focused on convolutional neural networks [16].

Many of the previous studies in early sepsis detection also use input data from multiple physiological signals, clinical signs, or laboratory test results [17]. Therefore, another advantage of the model we propose is that it relies exclusively on HRV data, which can be automatically processed from heart rate monitoring readings that are done continuously and routinely in NICU. This also allows it to produce the LOS detection nearly in real time, being able to measure an updated probability of LOS every 30 minutes. These characteristics make the proposed method easy and practical to potentially implement in a NICU setting as a DSS, complementing other clinical signs.

An interesting aspect of the results we obtained is that the AUROC increases as the evaluation window gets closer

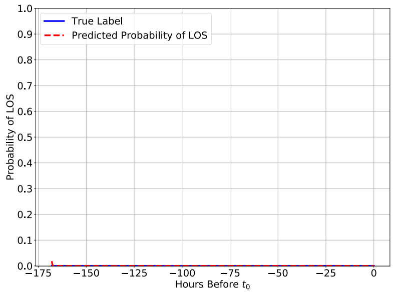

(a) Control Group Patient

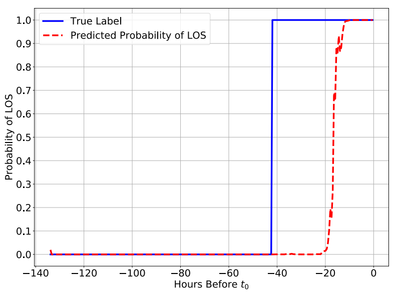

(b) LOS Group Patient 1

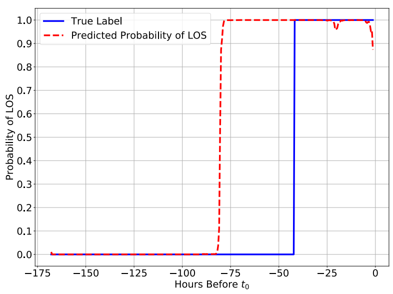

(c) LOS Group Patient 2

Figure 1: Examples of the predicted probabilities of a patient having LOS. The blue solid line represents the label assigned to a time period, and the red dashed line represents the probability of LOS calculated by the RNN model.

Authorized licensed use limited to: CSIRO Information Technology Services. Downloaded on September 09,2022 at 00:40:40 UTC from IEEE Xplore. Restrictions apply.

to t_0, while its 95% CI reduces. This is consistent with the fact that the labeling of the samples, although based on the results of a previous study, is still arbitrary as it is impossible to determine exactly when a particular patient became infected, but as the evaluation gets closer to t_0, the certainty that a patient is infected increases. This is exemplified in the two sample cases of LOS patients we presented, where for patient 1 the detection of LOS by the model occurred approximately 20 hours before t_0, while for patient 2, it occurred approximately 70 hours before t_0. These differences between the results of individual patients might reflect the differences in the time each patient was infected, rather than periods of incorrect detection.

5. Conclusion

In this paper we propose a method for early LOS diagnosis in a preterm infants, based on recurrent neural networks and the use of HRV features. This method achieves an AUROC of more than 80% for the 24 hours before LOS onset, and a maximum AUROC of 90.4% for the period of six hours previous to LOS onset. As infection also affects the respiration rate, future work will focus on developing a similar model but using respiration rate features.

Acknowledgments

This study received funding from the European Union's Horizon 2020 research and innovation program under grant agreement No. 689260 (Digi-NewB project).

References

[1] Chawanpaiboon S, Vogel JP, Moller AB, Lumbiganon P, Petzold M, Hogan D, Landoulsi S, Jampathong N, Kongwattanakul K, Laopaiboon M, Lewis C, Rattanakanokchai S, Teng DN, Thinkhamrop J, Watananirun K, Zhang J, Zhou W, Gülmezoglu AM. Global, regional, and national estimates of levels of preterm birth in 2014: a systematic review and modelling analysis. The Lancet Global Health January 2019;7(1):e37–e46.

[2] Stoll BJ, Hansen N, Fanaroff AA, Wright LL, Carlo WA, Ehrenkranz RA, Lemons JA, Donovan EF, Ann R. Stark JET, Oh. W, Bauer CR, Korones SB, Shankaran S, Laptook AR, Stevenson DK, Papile LA, Poole WK. Late-onset sepsis in very low birth weight neonates: The experience of the nichd neonatal research network. Pediatrics August 2002; 110(2):285–291.

[3] Kumar A, Roberts D, Wood KED, Light B, Parrillo JE, Sharma S, Suppes R, Feinstein D, Zanotti S, Taiberg L, Gurka D, Kumar A, Cheang M. Duration of hypotension before initiation of effective antimicrobial therapy is the critical determinant of survival in human septic shock. Critical Care Medicine June 2006;34(6):1589–1596.

[4] Alverdy J, Krezalek M. Collapse of the microbiome, emergence of the pathobiome, and the immunopathology of sepsis. Critical Care Medicine February 2017;45(2):337–347.

[5] Malik A, Hui CPS, Pennie RA, Kirpalani H. Beyond the complete blood cell count and c-reactive protein. Archives of Pediatrics Adolescent Medicine June 2003;157(6):511–516.

[6] D.Fairchild K, O'Shea M. Heart rate characteristics: Physiomarkers for detection of late-onset neonatal sepsis. Clinics in Perinatology September 2010;37(3):581–598.

[7] Moorman J, Lake D, Griffin M. Heart rate characteristics monitoring for neonatal sepsis. IEEE Transactions on Biomedical Engineering January 2006;53(1):126–132.

[8] León C, Carrault G, Pladys P, Beuchée A. Early detection of late onset sepsis in premature infants using visibility graph analysis of heart rate variability. IEEE Journal of Biomedical and Health Informatics 2021;25(4):1006–1017.

[9] Nakamura T, Horio H, Miyashita S, Chiba Y, Sato S. Identification of development and autonomic nerve activity from heart rate variability in preterm infants. Bio Systems 2005; 79(1-3):117–124.

[10] National Reference Center For Nosocomial Infection Surveillance at the Institute for Hygiene and Environmental Medicine Charité – University Medicine Berlin. NEO-KISS Protocol Nosocomial Infection Surveillance for preterm infants with birthweight < 1500g, February 2010.

[11] Doyen M, Ge D, Beuchée A, Carrault G, I. Hernández A. Robust, real-time generic detector based on a multi-feature probabilistic method. PLOS ONE October 2019;14(10):1–22.

[12] Shaffer F, Ginsberg JP. An overview of heart rate variability metrics and norms. Frontiers in Public Health 2017;5:258.

[13] Chung J, Gulcehre C, Cho K, Bengio Y. Empirical evaluation of gated recurrent neural networks on sequence modeling, December 2014. URL `https://arxiv.org/abs/1412.3555v1`.

[14] Diederik P. Kingma JB. Adam: A method for stochastic optimization, January 2017. URL `https://arxiv.org/abs/1412.6980`.

[15] Kam HJ, Kim HY. Learning representations for the early detection of sepsis with deep neural networks. Computers in Biology and Medicine 2017;89:248–255.

[16] Amiri P, Abbasi H, Derakhshan A, Gharib B, Nooralishahi B, Mirzaaghayan M. Potential prognostic markers in the heart rate variability features for early diagnosis of sepsis in the pediatric intensive care unit using convolutional neural network classifiers. In 2020 42nd Annual International Conference of the IEEE Engineering in Medicine Biology Society (EMBC). 2020; 5627–5630.

[17] He Z, Chen X, Zhen F, Yi W, Wang C, Jiang L, Tong Z, Bai Z, Li Y, Pan Y. Early sepsis prediction using ensemble learning with features extracted from lstm recurrent neural network. 12 2019; .

Address for correspondence:

Cristhyne León

LTSI-Université de Rennes 1. Campus de Beaulieu. Bât 22. 35042. Rennes. France

cristhyne.leon@univ-rennes1.fr

Authorized licensed use limited to: CSIRO Information Technology Services. Downloaded on September 09,2022 at 00:40:40 UTC from IEEE Xplore. Restrictions apply.
