# Supplementary material for: Exploring Computational Techniques in Preprocessing Neonatal Physiological Signals for Detecting Adverse Outcomes: Scoping Review
Source: Interact J Med Res. 2024 Aug 20;13:e46946. doi: 10.2196/46946 (PMC11372324; doi:10.2196/46946)
Supplement: Multimedia Appendix 3 [file ijmr_v13i1e46946_app3.zip › Included Papers - Final/3046/A. H. Gee et al. - 2016 - Improving heart rate estimation in preterm infants.pdf]

Improving Heart Rate Estimation in Preterm Infants with Bivariate Point Process Analysis of Heart Rate and Respiration

Alan H. Gee*, Riccardo Barbieri, *Senior Member*, *IEEE*, David Paydarfar, and Premananda Indic, *Senior Member, IEEE*

Abstract — **Accurate estimation of heart rate dynamics in preterm infants is important for predicting recurrent episodes of severe bradycardia. We hypothesize that estimation of heart rate can be improved by including respiration as a state variable, based on mechanisms that underlie cardio-respiratory coherence. For ten preterm infants, we demonstrate that including respiration as a covariate improves estimation accuracy by an average of 11% across bradycardia severity, and reduces the maximum error by 8%. We also find that cardio-respiratory coherence increases in low frequency content just prior to severe bradycardia. Thus, incorporating respiratory information may improve models of heart rate dynamics and narrow potential features for bradycardia prediction.**

I. Introduction

Respiratory and cardiac signals are monitored closely in the Neonatal Intensive Care Unit (NICU) to avoid life-threatening morbidity associated with episodes of apnea and bradycardia. The prediction of these events is important to prevent hypoxemia and/or ischemia, which result in impaired neurological development and end-organ damage [1-4]. In order to reliably predict these phenomena, improved estimation of both heart rate and respiratory signals is needed. In this paper, we use the correlation between respiration and cardiac rhythms [5-7] to improve estimation of heart rate dynamics in preterm infants, with goals of improving bradycardia prediction.

Patterns and measures of heart rate variability (HRV) hold important information in uncovering physiological mechanism that govern cardiovascular control [8]. Preterm infants, in particular, exhibit a paradoxical relationship between respiration and cardiac output. Preterm infants breathe approximately 60 breaths per minute (1 Hz), while normal adults breathe at a lower rate (0.33 to 0.2 Hz). In addition preterm infants can experience apneas (> 10 s) or repeated pauses in breathing (e.g. periodic breathing) that introduce low frequencies in the respiratory signal [9]. Thus, the possible interplay between respiration and cardiovascular control may provide important indicators of maturation of the autonomic nervous system in the cardiac system [10, 11].

We hypothesize that there exists a transfer of information between heart rate and respiration that can be leveraged to improve estimation of heart rate. Previous work has alluded to unidirectional, causal spectral coherence from respiration to cardiac output at approximately 1 Hz [12]. To investigate the above hypothesis, we examine heart rate variability using point process theory and incorporate a respiratory covariate to account for potential cardio-respiratory interactions. The goal of improving heart rate estimation is to improve prediction of life-threatening events, like bradycardia. Thus, we evaluate the benefit of a respiratory covariate in heart rate estimation in segments preceding bradycardia.

II. Methods

A. Preterm Infant Data Set

Ten preterm infants, who were spontaneously breathing room air, were studied. The post-conceptual ages were 29 3/7 to 34 2/7 weeks (mean: 31 1/7), and the weights were 843 to 2100 grams (mean: 1468). A 3-lead electrocardiogram (ECG) signal (500 Hz) was collected and recorded for ~20-70 hours (mean: 43). Respiratory inductance plethysmography was also used to record a respiration signal (50 Hz) from abdominal inspiration and exhalation movements. The study protocol was approved by the University of Massachusetts Medical School Institutional Review Board for human subjects.

We generate peak-to-peak R-R intervals from the ECG signal, using a modified Pan-Tompkins peak detection algorithm. We investigate segments consisting of normal heart rates (> 100 bpm) and clinical bradycardias: mild (100-80 bpm), moderate (80-60 bpm), and severe (<60 bpm). We randomly choose 30 severe bradycardias, due to data limitation, and 40-60 examples from each other severity. All analysis is on the 3 minute window prior to each bradycardia.

B. Bivariate Modeling of Heart Rate and Respiration

Heartbeats are a serial procedure where action potentials from the autonomic nervous system govern cardiac contractions. We can model a heartbeat at time k with a univariate p-order linear regression:

$$\mu(rr,\theta) = \theta_o + \sum_{j=1}^{p} \theta_j \, rr(t-j) \tag{1}$$

where $\theta=\{\theta_o, \dots, \theta_j, \dots, \theta_p\}$ is the estimation vector of optimized model parameters, and $rr(t-j)$ is the heart beat time series (i.e. time difference between contractions). Respiration has been known to influence cardiac control [10-

This work was supported in part by the Hansjörg Wyss Institute for Biologically Inspired Engineering at Harvard University, by the National Science Foundation (NSF) Smart and Connected Health (SCH) Grant #1401690, and by the National Institutes of Health (NIH) R01 GM104987. *Asterisk represents corresponding author.*

*A. H. Gee is with the Wyss Institute, Harvard University, Boston, MA 02115, USA. (e-mail: Alan.Gee@wyss.harvard.edu).

R. Barbieri is with the Department of Electronics, Information and Bioengineering, Politecnico di Milano, Milano, Italy, and with the Dept. of Anesthesia, Critical Care, and Pain Medicine, Massachusetts General Hospital, Harvard Medical School, Boston, MA 02114 USA.

D. Paydarfar is with the Department of Neurology, University of Massachusetts Medical School, MA 01655, USA and also with Wyss Institute, Harvard University, Boston, MA 02115, USA.

P. Indic is with the Department of Neurology, University of Massachusetts Medical School, MA 01655, USA.

978-1-4577-0220-4/16/$31.00 ©2016 IEEE

Authorized licensed use limited to: CSIRO Information Technology Services. Downloaded on September 09,2022 at 00:27:50 UTC from IEEE Xplore. Restrictions apply.

12], so we include respiration as a covariate term of (1) to create a bivariate linear regression of *(p + q)*-order:

$$\beta(rr,\theta,resp,\gamma) = \mu(rr,\theta) + \sum_{j=1}^{q} \gamma_j \, resp(t-j) \quad (2)$$

where $\gamma = \{\gamma_1, \dots, \gamma_j, \dots, \gamma_q\}$ is the estimation vector for the covariate respiration, and $resp(t-j)$ is the respiration signal for the same time window. The linear regression functions, $\mu(rr,\theta)$ and $\beta(rr,\theta,resp,\gamma)$, represent the mean peak time of the lognormal sample distribution. We assume the collection of heartbeats obeys a lognormal probability distribution [13] and the next heartbeat can be estimated as:

$$f_{k+1}(t|H_k,\theta) = \left[\frac{1}{2\pi\sigma^2(t-u_k)^2}\right]^{\frac{1}{2}} exp\left\{-\frac{1}{2}\frac{(ln(t-u_k)-X)^2}{\sigma^2}\right\} \quad (3)$$

where u_k is the time for a given peak k, f_{k+1} is the waiting time until the next heartbeat time, u_{k+1}, and X is either $\mu(rr,\theta)$ or $\beta(rr,\theta,resp,\gamma)$ with σ^2, respectively. We can then apply the traditional exponential transformation to the lognormal estimates, X and σ^2, from lognormal mean and variance to obtain a Gaussian mean and variance:

$$M(t) = e^{X+\sigma(t)^2/2}$$
$$V(t) = \left(e^{\sigma(t)^2} - 1\right)e^{2X+\sigma(t)^2} \quad (4)$$

M(t) and *V(t)* are Gaussian estimates of the sample mean and variance of the distribution of R-R intervals. These estimates are considered instantaneous since the sample probability distribution parameters, X and σ^2, are updated by overlapping maximum likelihood estimates every Δt, where Δt is less than the next available peak time [14].

C. Validity of the Point Process Model

We use the Kolmogorov-Smirnov (KS) statistic to evaluate the goodness-of-fit of the point process model [15]. We employ the Time-Rescaling Theorem, which states that a point process with an integrable conditional intensity function can be transformed into a Poisson process with unit rate [15]. As a consequence, we can quantify any discrepancy between our model and the R-R time series with a KS plot.

The KS plot depicts the effectiveness of the point process estimation in capturing the statistical properties of the real data, with a 45-degree line representing a perfect estimation [15]. We can compare the maximum deviation distance and the area between our estimation and the perfect estimation (e.g. the 45-degree line) to quantify the differences of models (1) and (2). The model with the lower maximum distance and/or smaller area has the better fit. We calculate 95% confidence bounds and express the improvement of the model as a fraction of the total error area over the 95% confidence interval area.

D. Point Process Features Prior to Bradycardia

We then investigate the behavior of point-process indices (*M(t), V(t)*) using the clustering analysis from [13]. We take the indices corresponding to a 3-minute window prior to bradycardia, and create normalized (*M(t), V(t)*) clusters for each severity class (Fig 4a). We calculate cumulative density curves by summing up the Euclidean distance of each (*M(t), V(t)*) pair to the k-means cluster centroid (Fig 4b).

E. Coherence of R-R Time Series and Respiration

We can determine the linear effect of the cardio-respiratory interaction by evaluating coherence of the R-R and respiration signal. The coherence is calculated using methods and predetermined frequency ranges outlined in [12]. The instantaneous frequencies were determined using un-normalized estimates *M(t)* from the point process framework (4). We then average the coherence within each subject before averaging over all subjects.

III. Results

A. Respiration Covariate in Estimation of R-R

We evaluate the lognormal, point process estimation of heart rate variability by examining heartbeats from clinical bradycardia segments with respiratory information (Fig 1). We compare the maximum KS distances between two different point process models: (1) a univariate 10th-order linear regression on the R-R time series, and (2) a bivariate, linear regression of the R-R time series (10th order) and the respiration signal (8th order).

Figure 1 displays a severe bradycardia (depth 55.3 bpm, duration 27 s). In this example, we observe two different states: heart rate appears stable during pre-bradycardia time

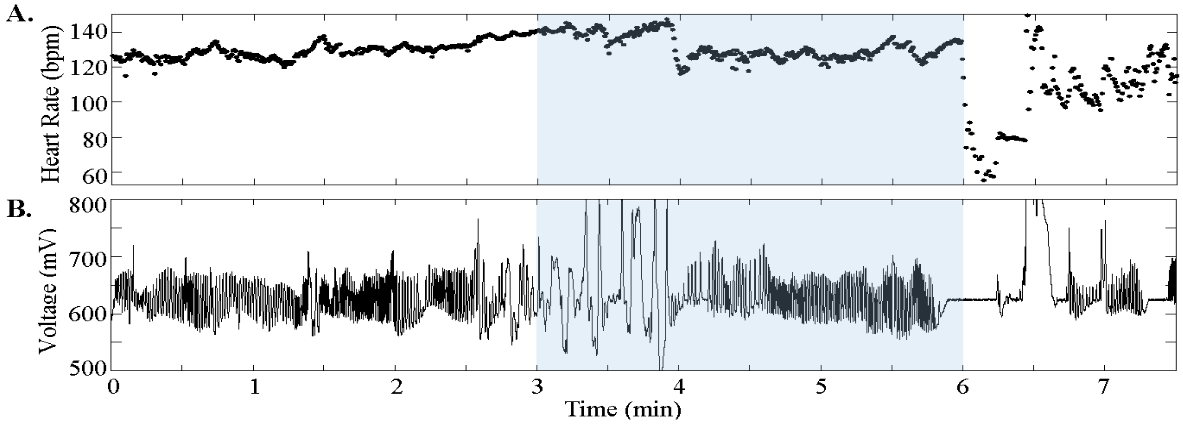

Fig 1. (A) Severe bradycardia from Subject 2 (55.3 bpm and a duration of 27.0 s). The light blue region denotes a 3-minute period prior to bradycardia onset. (B) Corresponding respiration signal measured from inductance plethysmography bands. A 25 s apnea occurs during the severe bradycardia at t = 5.8 min.

Authorized licensed use limited to: CSIRO Information Technology Services. Downloaded on September 09,2022 at 00:27:50 UTC from IEEE Xplore. Restrictions apply.

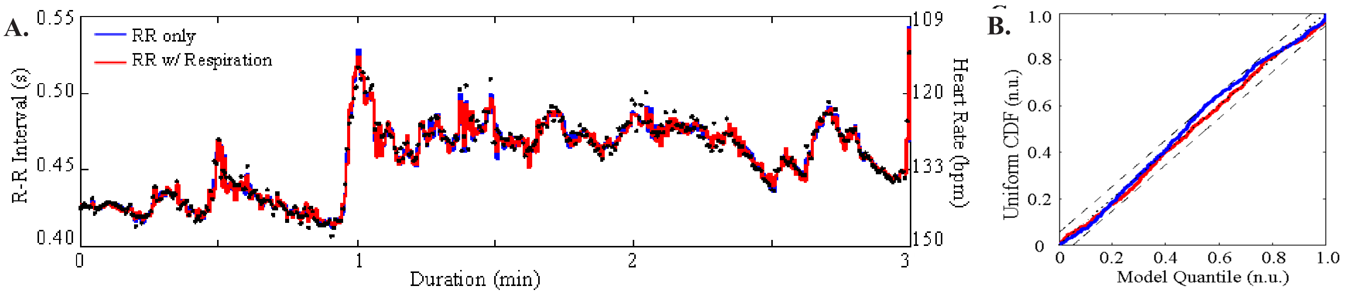

Fig 2 (A) The R-R time series of a 3 minute window prior to severe bradycardia as depicted light blue in Fig 1. The blue line represents the point process estimation (PPE) of the R-R time series using only the R-R signal, while the red line represents the PPE using the R-R signal and a covariate respiration signal. The R-R only estimation over-estimates some regions of the data (e.g. 1.25 to 1.5 min). (B) The KS plot of the estimation presented in (A). The R-R and respiration estimation (red) follows the ideal model (45 degree line), while the R-R only estimation (blue) deviates within the 95 % confidence interval.

and then becomes chaotic post-bradycardia (Fig 1A). Using our two point process models (e.g. with and without respiration), we generate instantaneous estimates of the heartbeat series prior to bradycardia (Fig 2A). We observe that both point process estimates capture the fluctuations in the heartbeat time series and fall within the 95% confidence interval of the empirical data (Fig 2B). To quantify the goodness-of-fit of our models, we use the Kolmogorov-Smirnov (KS) statistic to calculate the maximum deviation

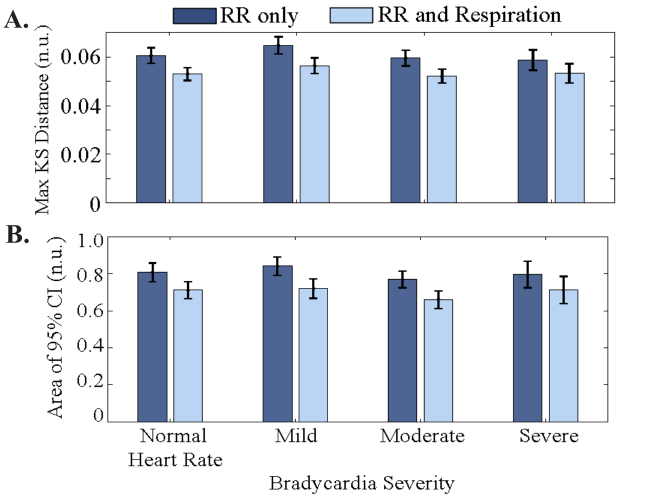

Fig 3. (A) Mean of the maximum KS distance. Each severity class is statistically significant (paired t-test, p-values < 0.02). (B) Mean of the total area between the model estimation and the ideal model estimation. The area is expressed as a fraction of the 95% confidence interval area. Each severity class is statistically significant (paired t-test, p-values < 0.02).

TABLE 1
RELATIVE BENEFIT OF POINT PROCESS ESTIMATION WITH RESPIRATION COVARIATE

Severity	Max KS	KS Area
Normal HR	8.0 %	7.7 %
Mild	11.9 %	14.3 %
Moderate	7.5 %	13.5 %
Severe	5.8 %	9.8 %
Average	8.3 %	11.3 %

from an ideal model (e.g. the 45-degree line) (Fig 2B). For the severe bradycardia example in Fig 2, our results show an improvement in estimation with the bivariate model of heartbeat and respiration. The bivariate model shows a 36% improvement in maximum KS (0.025 n.u. compared to 0.039 n.u.), and a 45% improvement in error area (0.23 n.u. compared to 0.42 n.u.) over the univariate model.

We then examine the deviation in both maximum distance and cumulative area across varying heart rate severity (Fig 3). Across all segments, we observe an improvement in estimation by with the respiration covariate (paired t-test, p-values < 0.02). The average decrease in maximum KS distance was 0.07 n.u., an 8% improvement over the univariate regression model for all bradycardia severity (Table 1). Additionally, the overall error area is reduced by 11% (Table 1). We observe that in all cases of bradycardia severity, the addition of data from respiration improves point process estimation.

B. Impact of Respiration Covariate on Bradycardia Prediction Features

We investigate the effects of the improvement of heartbeat estimation with respiration on the point process indices used to predict bradycardia. We generate (*M(t), V(t)*) clusters and density curves as outlined in [13] (Fig 4). We observe a negligible improvement in the density curves of (*M(t), V(t)*) clusters by incorporating respiration, even though respiration improved estimation of heartbeats overall. This suggests that the normalized clustering feature is largely independent of respiratory effects, and that other point process indices should be investigated to take advantage of respiratory information.

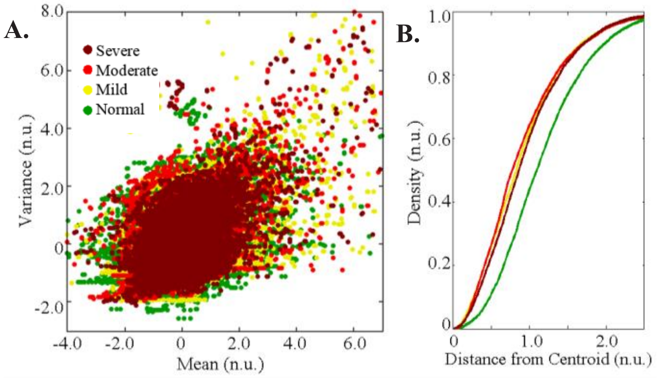

Fig 4. (A) Normalized point process mean and variance cloud for varying bradycardia severity in a 3 minute window prior to bradycardia. (B) Plot of the cumulative distribution of distances from cluster centroids of (A).

Authorized licensed use limited to: CSIRO Information Technology Services. Downloaded on September 09,2022 at 00:27:50 UTC from IEEE Xplore. Restrictions apply.

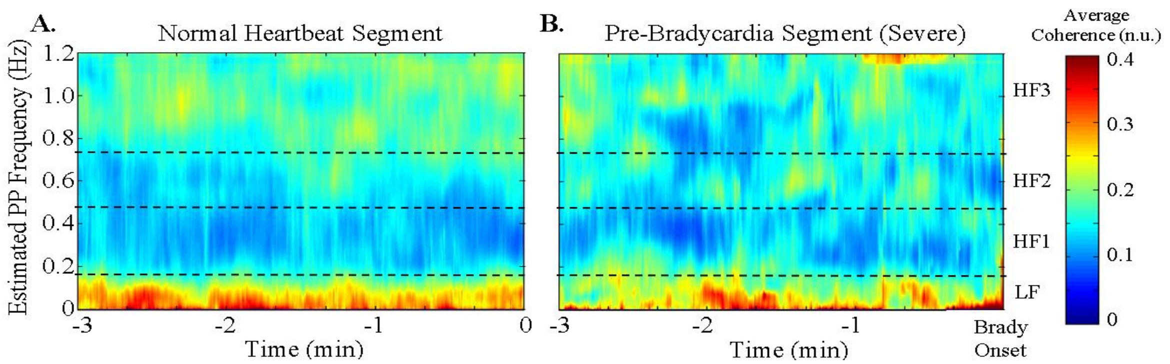

Fig 5. (A) Average coherence between the R-R time series and respiration for normal heartbeat segments of length 3 minutes. Notice the presence of a 1 Hz interaction from respiration. (B) Average coherence for a 3-minute window prior to severe bradycardia. There is an increase in coherence in low frequency content just prior to bradycardia onset (~30 s prior). We also observe a breakdown in the 1 Hz respiration coherence (HF3) present in normal heart rate.

C. *Coherence Change Prior to Bradycardia*

We calculate the average coherence between the R-R time series and respiration for both normal heartbeat segments and a 3-minute window prior to severe bradycardia (Fig 5). We observe an increase in coherence in the low frequency content just prior to bradycardia onset (Fig 5B). This low frequency coherence relates to long pauses in breathing (low frequency respiration) of these infants [9] and can be seen in Fig 1B. We also observe a breakdown in the ~1Hz respiration coherence (HF3) for severe bradycardia that is typically present in normal heart rate for infants [12] (Fig 5).

IV. Conclusion & Discussion

We present an improved point process framework for modeling and characterizing heart rate dynamics in preterm infants. We improve the accuracy of the model by incorporating the respiratory signal as a covariate in the linear regression model of bradycardia (Fig. 2-3, Table 1). By including respiration, we observe a statistical improvement of 11% in the R-R time series estimation and reduced the maximum error in estimation by 8%. We also observe a loss of the 1 Hz respiratory interaction with the R-R signal and an increase in low frequency content during segments of severe bradycardia (Fig 5). The linear interaction between HRV and respiration suggests that fluctuations leading to bradycardia might be related to pathological fluctuations in breathing, particularly long apnea episodes, and could be used as precursory features in bradycardia prediction.

Our results show that there is no significant benefit or improvement by using respiration as a covariate in the context of using normalized *M(t)* and *V(t)* clustering features (Fig 4). However, the improvement of HRV estimation with respiration does not rule out possible benefits from other features utilized in the point process framework (i.e. poles, zeroes, frequency) nor the use of respiration as a viable covariate in another context. Further work is needed to explore features other than *M(t)* and *V(t)* and other methodologies that may benefit from the respiratory covariate. Inclusion of other physiological signals as covariates, like movement and oxygen saturation, should also be explored in future work.

Acknowledgment

The authors thank Courtney Temple and Ian Zuzarte for data collection. We also thank John Osborne and the Wyss Institute for providing the data acquisition system.

References

[1] J. M. Perlman and J. J. Volpe, "Episodes of apnea and bradycardia in the preterm newborn: impact on cerebral circulation," Pediatrics, vol. 76, pp. 333-8, Sep 1985.

[2] A. Janvier, M. Khairy, A. Kokkotis, C. Cormier, D. Messmer, and K. J. Barrington, "Apnea is associated with neurodevelopmental impairment in very low birth weight infants," J Perinatol, vol. 24, pp. 763-8, Dec 2004.

[3] F. Serenius, K. Kallen, M. Blennow, U. Ewald, V. Fellman, G. Holmstrom, E. Lindberg, P. Lundqvist, K. Marsal, M. Norman, E. Olhager, L. Stigson, K. Stjernqvist, B. Vollmer, and B. Stromberg, "Neurodevelopmental outcome in extremely preterm infants at 2.5 years after active perinatal care in Sweden," JAMA, vol. 309, pp. 1810-20, May 1 2013.

[4] G. P. Aylward, "Neurodevelopmental outcomes of infants born prematurely," J Dev Behav Pediatr, vol. 35, pp. 394-407, 2014.

[5] H. Vyas, A. D. Milner, and I. E. Hopkin, "Relationship between apnoea and bradycardia in preterm infants," Acta Paediatr Scand, vol. 70, pp. 785-90, Nov 1981.

[6] D. J. Henderson-Smart, M. C. Butcher-Puech, and D. A. Edwards, "Incidence and mechanism of bradycardia during apnoea in preterm infants," Arch Dis Child, vol. 61, pp. 227-32, Mar 1986.

[7] C. J. Upton, A. D. Milner, and G. M. Stokes, "Episodic bradycardia in preterm infants," Arch Dis Child, vol. 67, pp. 831-4, Jul 1992.

[8] G. G. Berntson, J. T. Bigger, Jr., D. L. Eckberg, P. Grossman, P. G. Kaufmann, M. Malik, H. N. Nagaraja, S. W. Porges, J. P. Saul, P. H. Stone, and M. W. van der Molen, "Heart rate variability: origins, methods, and interpretive caveats," Psychophysiology, vol. 34, pp. 623-48, Nov 1997.

[9] T. B. Waggener, I. D. Frantz, 3rd, A. R. Stark, and R. E. Kronauer, "Oscillatory breathing patterns leading to apneic spells in infants," J Appl Physiol, vol. 52, pp. 1288-95, May 1982.

[10] D. P. Giddens and R. I. Kitney, "Neonatal heart rate variability and its relation to respiration," J Theor Biol, vol. 113, pp. 759-80, Apr 21 1985.

[11] E. G. Rosenstock, Y. Cassuto, and E. Zmora, "Heart rate variability in the neonate and infant: analytical methods, physiological and clinical observations," Acta Paediatr, vol. 88, pp. 477-82, May 1999.

[12] P. Indic, E. Bloch-Salisbury , F. Bednarek, E.N. Brown, D. Paydarfar, R. Barbieri. “Assessment of Cardio-respiratory Interactions in Preterm Infants by Bivariate Autoregressive Modeling and Surrogate Data Analysis,” Early human development. vol 87 (7):477-487, 2011.

[13] A. H. Gee, R. Barbieri, D. Paydarfar, and P. Indic, "Uncovering statistical features of bradycardia severity in premature infants using a point process model," in Engineering in Medicine and Biology Society (EMBC), 2015 37th Annual International Conference of the IEEE, 2015, pp. 5855-5858.

[14] P. Indic, D. Paydarfar, and R. Barbieri, "Point Process Modeling of Interbreath Interval: A New Approach for the Assessment of Instability of Breathing in Neonates," *IEEE* Transactions on Biomedical Engineering, vol. 60, pp. 2858-2866, Oct 2013.

[15] E. N. Brown, R. Barbieri, V. Ventura, R. E. Kass, and L. M. Frank, "The time-rescaling theorem and its application to neural spike train data analysis," Neural Comput, vol. 14, pp. 325-46, Feb 2002.

Authorized licensed use limited to: CSIRO Information Technology Services. Downloaded on September 09,2022 at 00:27:50 UTC from IEEE Xplore. Restrictions apply.
