# Supplementary material for: Exploring Computational Techniques in Preprocessing Neonatal Physiological Signals for Detecting Adverse Outcomes: Scoping Review
Source: Interact J Med Res. 2024 Aug 20;13:e46946. doi: 10.2196/46946 (PMC11372324; doi:10.2196/46946)
Supplement: Multimedia Appendix 3 [file ijmr_v13i1e46946_app3.zip › Included Papers - Final/3066/R. Ahmed et al. - 2015 - Classification of hypoxic-ischemic encephalopathy .pdf]

Classification of Hypoxic-Ischemic Encephalopathy Using Long Term Heart Rate Variability Based Features

Rehan Ahmed, *Student Member, IEEE*, Andrey Temko, *Senior Member, IEEE*, William P. Marnane, *Member, IEEE,* Geraldine Boylan and Gordon Lightbody, *Member, IEEE*

***Abstract*—Hypoxic-ischemic HI injury at the time of birth could lead to severe neurological dysfunction at an older age. Therapeutic hypothermia can be used to treat HI if severity of injury is determined within 6 hours of birth. EEG is generally used to assess the brain injury but it is neither widely recorded after birth nor is the expertise to interpret it commonly available. This study presents a novel system to classify HI injury using heart rate variability. The system makes decisions based on long-term statistical features extracted from the short-term HRV features. The preliminary results show the promising performance and robustness of the proposed method given a poor quality dataset. This tool can serve as decision support system in remote maternity units to help clinical staff to initiate hypothermia.**

I. Introduction

Hypoxic-ischemic encephalopathy (HIE) is one of the most common cause of neonatal deaths and long-term neonatal neurological disorder with reported incidences of 3-5 per 1000 births [1]. HIE injury occurs due to lack of oxygen to the neonatal brain around the time of birth. It is shown that Therapeutic Hypothermia (TH) (body or brain cooling) for the new-born may reduce the effects of progressive encephalopathy and could improve the long term outcomes [2].

HIE is generally graded soon after the birth into mild moderate or severe. An infant is usually treated with TH if it has moderate-severe encephalopathy. Moreover, TH is required to be initiated within 6 hours of birth. Therefore it is at this stage, that clinical staff needs to assess the injury to initiate the treatment. Currently, Electroencephalogram (EEG) is considered to be the gold standard in diagnosing and monitoring neonates with HIE. However, classifying HIE using the EEG requires expertise to acquire the EEG signal and the presence of an expert neurophysiologist to interpret it. This expertise is limited to specialized units only and not readily available in many Neonatal Intensive Care Units (NICU). In addition, EEG is rarely available immediately after birth particularly at remote maternity units where neither the expertise to interpret EEG nor the equipment may be available.

On the other hand, the electrocardiogram (ECG) is a method of monitoring the heart which is widely available after birth and is used to monitor neonates everywhere. It is reported in previous studies on neonates that the heart rate variability (HRV) changes after hypoxia [3], [4] and is shown to be statistically correlated with HIE injury [4].

Fig. 1 shows the comparison of HRV in mild and severe HIE with the related EEG of the similar grade. HRV is inversely related to the HIE grade, the greater the HIE grade lower the HRV. It is reported that this phenomenon is due to injury in the parts of brain which are responsible for the initiation of the HR rhythm [5]. Therefore, there are premises to believe that an early warning brain monitoring system can be designed based on HRV.

To the best of our knowledge there are no previous studies on developing a tool for the classification of HIE injury using HRV. The HRV signal is less complex and does not have similar dynamic nature as EEG. It is shown in [6] that HRV is not very useful for detecting short events like seizures as the changes in HRV are very subtle. Thus, classifying the HRV requires a sequence of data that is large enough to capture the slower time scale components in order to discriminate between HIE grades.

In this work, we present a system based on a cross-disciplinary method of Support Vector Machine (SVM) and Gaussian supervector approach. This system is similar to the one developed by our group for HIE grading using EEG [7]. This technique has shown state of the art results in many pattern recognition areas [8], [9]. This approach enables the extraction of a long-term summary from a sequence of the short-term HRV feature vectors.

The outline of the paper is as follows:, Section 2 describes the dataset and the proposed approach. Section 3 presents results and discusses the performance of the system. Section 4 concludes the work.

II. Method

A. Dataset

The dataset used in this work consists of 54 1-hour recordings of physiological signals collected in the NICU of the Cork University Maternity Hospital, Cork, Ireland. Written informed parental consent and approval from Clinical Ethics Committee of the Cork Teaching Hospitals was obtained for all neonates studied in this work. The EEG recordings were started within 6 hours of birth and continued for 24-72 hours to monitor the evolution of the developing encephalopathy and for seizure surveillance. The neonates in

*This research was supported by a Science Foundation Ireland (SFI) Principal Investigator Award (SFI 10/IN.1/B3036), a SFI Centers Program Award (12/RC/2272).

R. Ahmed, A. Temko, W. Marnane and G. Lightbody are with the Department of Electrical and Electronic Engineering, Neonatal Brain Research Group. Irish Center for Fetal and Neonatal Translational Research (INFANT), University College Cork, Ireland; rehan@eleceng.ucc.ie, {atemko, g.lightbody, l.marnane}@ucc.ie

G. Boylan is with the Department of Pediatrics and Child Health, Neonatal Brain Research Group, INFANT, University College Cork, Ireland: g.boylan@ucc.

978-1-4244-9270-1/15/$31.00 ©2015 IEEE

Authorized licensed use limited to: CSIRO Information Technology Services. Downloaded on September 09,2022 at 00:27:04 UTC from IEEE Xplore. Restrictions apply.

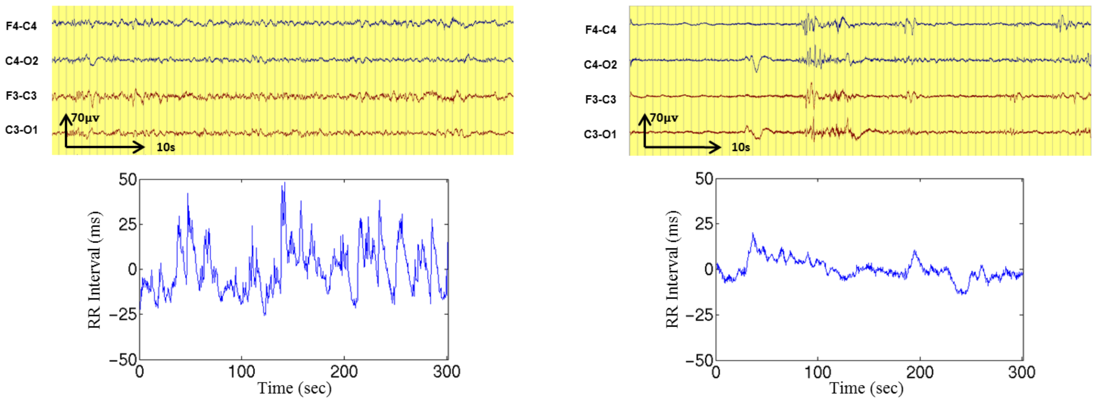

(a) Mild (b) Moderate- Severe

Figure 1: Examples of the Mild and moderate - severe HIE grade on EEG and its related HRV in RR interval. The HRV signals are 5 minute long. The signals are normalized by subtracting the mean HRV in this time duration. The corresponding EEG samples shown are 1minute long.

this cohort were not treated with TH. Two electrodes were placed, one on each shoulder of the neonate for recording the ECG. The ECG recordings were graded based upon the background EEG. Each EEG recording had continuous presence of a specific grade. The EEG grade of the one hour recording was defined with the consensus of two independent EEGers using the guidelines outlined in [10]. The same dataset has earlier been used for grading HIE using EEG [7].

This dataset was collected with the main focus on EEG quality, so the ECG was not monitored and maintained properly during the recordings. Six files from the 54 were dropped due to the extremely bad quality or absence of ECG trace. Major artifacts that were clearly visible, such as movement and electrode displacement were manually removed. Minor artifacts were not removed. Based on the quality of ECG, this dataset can be seen as overly pessimistic if compared to real world situations. The developed system is expected to perform better given the quality of ECG is maintained.

B. *Overall System*

Fig. 2 shows an outline of the complete automated HIE grading system. Features are extracted from the HRV signals. Sequences of these feature vectors are used to create a statistical model. Parameters of this statistical model are then used as input to a discriminant classifier. Details of each individual block are as follows.

1) *Pre-processing and Feature Extraction*

The R-peaks were extracted from the raw ECG signals using the Pan Tompkins method [11].The timing of each peak was adjusted and then was uniformly sampled to 256 Hz using hermite spline quadratic interpolation. Thereafter, 7 HRV features were extracted from a 1 minute window with 30 seconds overlap using the normalized RR interval (NN interval). The features are outlined in the Table 1. They provide a generalized picture of both time and frequency domain representation of the HRV. The performance of these features for HIE grading has been discussed in several recent clinical studies [4], [12].

2) *Gaussian Mixture Model and Supervectors*

The GMM is a probability density function (PDF) represented as a weighted sum of M Gaussian components:

$$p(\mathbf{x}) = \sum_{j=1}^{M} w_j \, g(\mathbf{x}|\mathbf{m}_j, \mathbf{\Sigma}_j). \quad (1)$$

Here $\mathbf{x}$ is a feature vector of n dimensions, $w_j, j = 1, \dots, M$ are the mixture weights, and $g(\mathbf{x}|\mathbf{m}_j, \mathbf{\Sigma}_j)$ are the component densities. During training, the parameters, $w_j, \mathbf{m}_j$ and $\mathbf{\Sigma}_j$ are optimised iteratively using the expectation maximization algorithm in order to maximize the log-likelihood of the model to the input feature space [13].

a) *Universal Background Model (UBM)*

A UBM is a general HRV GMM model that represents all the classes of the HIE and any other background activity. The UBM is created with all the available data of all grades. The benefit of using UBM is that, it compensates for the lack of data for direct training of individual GMMs for each HIE grade. In this work, a UBM is created with 2 Gaussian components. Principle component analysis (PCA) is used to decorrelate the original feature space which allows the use of a diagonal covariance matrix. All the variance of the original space is retained, reducing the original 7 dimensional feature space to an average of 6 dimensions.

b) *Long Term Feature Vector Extraction (Gaussian Supervector)*

Fig. 3 shows the process of extracting long term feature vectors. Once the UBM is trained, a method called maximum a-posteriori (MAP) adaptation is used to adapt the parameters of this UBM using the training data [13] [14]. It can be seen from Fig. 3 that the means of the constituent Gaussian

TABLE 1: HRV FEATURES USED IN THIS STUDY FOR THE CLASSIFICATION OF HIE

Domain	Features
Time	• Mean NN interval<br>• Standard deviation of the NN interval<br>• Triangular interpolation of NN interval histogram<br>• 1st derivative of standard deviation between NN interval<br>• Entropy of the NN interval
Frequency	• Power in frequency band 1 (0.01-0.04 Hz)<br>• Power in frequency band 2 (0.04 – 0.2 Hz)

Authorized licensed use limited to: CSIRO Information Technology Services. Downloaded on September 09,2022 at 00:27:04 UTC from IEEE Xplore. Restrictions apply.

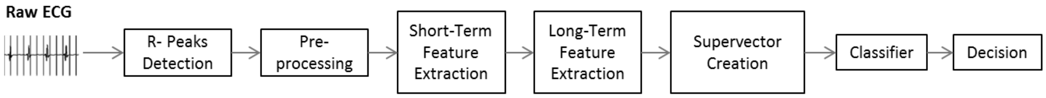

Figure 2: Overview of the complete system

components of the UBM move towards the distribution of the training data. For this information to be useable for the discriminative classifier SVM, a supervector is formed by concatenating the means of each Gaussian component of the adapted model. These supervectors are then fed to the SVM as feature vectors representing the long sequences of the HRV signal.

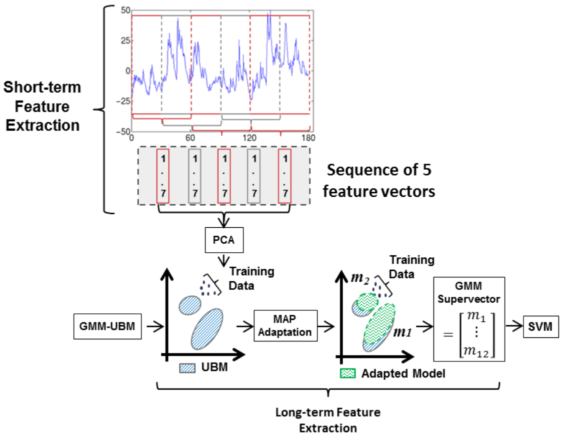

Figure 3: The process of creating long-term feature vector (supervector). The Gaussian components shown here are just for illustration purpose.

B. *Classification*

Classification is a two stage process. In the first stage a classifier is trained on the training data and the best parameters are selected. In the second stage, the developed classifier is tested on the unseen testing data.

The classical SVM is a binary classifier. Consider a two class problem, with a pre-labelled training set $(\mathbf{x}_1 y_1), \ldots, (\mathbf{x}_n y_n)$ where $y_i \in \{-1, +1\}$ and $\mathbf{x}_i \in \Re^n$. In SVM classification a test vector $\mathbf{x}$ is classified by evaluating

$$d(\mathbf{x}) = sign\left(\sum_{i \in I_{sv}} \alpha_i y_i K(\mathbf{x}, \tilde{\mathbf{x}}_i) + b\right) \quad (2)$$

Here, there are n_{sv} retained support vectors from the training data $\tilde{\mathbf{x}}_i$, each with weight α_i and associated target y_i, for $i \in I_{sv}$ (the set of indices of n_{sv} retained support vectors). The kernel $K(.,.)$ of the SVM is used to map the input data into a higher dimensional feature space.

The supervector can be thought of as a mapping between an EEG sequence and a high dimensional vector. The kernel K in the SVM can be used to measure the similarity between two supervectors that represent two HRV sequences. It is shown in [8] that an inner product between two supervectors, $\mathbf{v}$ and $\tilde{\mathbf{v}}_i$, is an upper bound of the Kullback-Leibler divergence between the PDFs of the two sequences modeled by a mixture of Gaussians. Thus, the SVM kernel function which yields a measure of similarity between two supervectors $\mathbf{v}$ and $\tilde{\mathbf{v}}_i$ from the training data is,

$$K(\mathbf{v}, \tilde{\mathbf{v}}_i) = \sum_{j=1}^{M} w_j (\mathbf{m}_j)^T \Sigma_j^{-1} \tilde{\mathbf{m}}_j^i = \sum_{j=1}^{M} \left(\sqrt{w_j} \boldsymbol{\Sigma}_j^{-\frac{1}{2}} \mathbf{m}_j\right)^T \left(\sqrt{w_j} \boldsymbol{\Sigma}_j^{-\frac{1}{2}} \tilde{\mathbf{m}}_j^i\right) \quad (3)$$

Where $\mathbf{v}^T = [\mathbf{m}_1^T \ldots \mathbf{m}_M^T]$ and $\tilde{\mathbf{v}}_i^T = [\tilde{\mathbf{m}}_1^T \ldots \tilde{\mathbf{m}}_M^T]$. Note that the scaling terms, weight w_i and covariance matrix $\boldsymbol{\Sigma}_i$, are the same for all sequences and can be computed beforehand. This allows the use of a simple linear kernel inside the SVM.

During the training phase, supervectors of the two grades are used to create an SVM model. Two-fold cross validation over this data is used to find the regularization parameter C for the linear SVM. In the testing phase, the test supervectors are passed through the trained SVM model to get one decision per sequence of the HRV signal using (2). Output of the SVM is then converted to probabilities using the method outlined in [15].

III. Results

In order to assess the performance of the proposed system, a Leave One Out (LOO) cross validation method was used which is known to produce an almost unbiased performance assessment of the developed system. Here our system was trained using the data from 48 recordings categorized into two classes; mild (grade 1) and moderate-severe (grade 2). The remaining one unseen recording was used to test the system. A separate UBM was created each time which does not contain the data from the unseen recording. The mean probability of grade 2 of all the recordings was calculated from the individual sequence based probabilities. The ROC area was then calculated by putting a threshold on the mean probabilities of all the files. Fig. 4 shows the comparison of the ROC areas of our proposed system compared to their basic mother classification approaches. The best ROC area achieved by our system is 81% as compare to 67% and 70.4% attained by SVM and

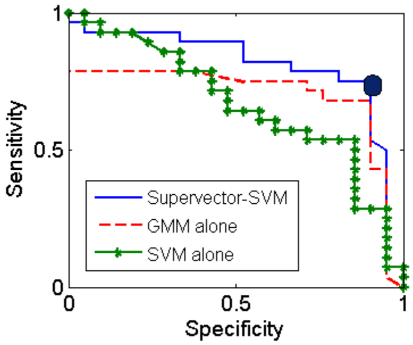

Figure 4: Comparison of ROC area (AUC) obtained by different methods across all neonates. AUC for Supervector-SVM=81%, GMM=70% and SVM =67%

Authorized licensed use limited to: CSIRO Information Technology Services. Downloaded on September 09,2022 at 00:27:04 UTC from IEEE Xplore. Restrictions apply.

TABLE 2 : COMPARISON OF DIFFERENT SEQUENCE LENGTHS ON THE OVERALL PERFORMANCE OF THE SYSTEM

Method	ROC area
**SVM - GMM Supervector**	
(3 epoch sequence)	79.5
(5 epoch sequence)	80.2
(7 epoch sequence)	**81.0**
(9 epoch sequence)	79.8

GMM methods respectively. A radial basis function kernel was used in the SVM and a simple GMM with 2 Gaussians with PCA was used for GMM only results. Clearly the classifier which can make a decision based on the statistics of longer time duration shows the better performance. Moreover, most improvement is obtained in the sensitivity area by the proposed approach meaning it has better performance for classifying the grade 2 recordings. However, individually the supervector based classifier can be thought of as more conservative towards grade 2 as it performs better in the specificity region as compared to sensitivity region.

Table 2 shows the results of our technique with different lengths of sequences to adapt the UBM model. It can be seen that 7 epochs that corresponds to four minutes of ECG, gave the best results. A shift of 1 epoch which corresponds to 30 seconds was used for each of the results for the proposed approach. Sequences longer than 7 epochs are suspected to increase influence of artifacts on the decision. However, clear investigation needs to be carried out in future studies.

Table 3 shows the confusion matrix of the system with the best operating point obtained from the ROC curve of the supervector approach by applying the threshold on the overall probabilities (indicated by circle marker in Fig. 4). Out of 49 recordings, 9 were misclassified by the proposed system. Two misclassifications were in grade 1 whereas 7 errors were made in grade 2. This corresponds to an overall accuracy of 81% achieved by the proposed system. These results are 9% inferior to the 87% obtained by our earlier developed automated HIE grading system based on EEG [7] given only a two class problem.

The last row of Table 3 shows the precision of the system which is defined as the ratio between the number of correctly assigned decisions and the number of total decisions assigned to a specific grade. It can be seen that, although the accuracy of classifying grade 1 is high but its precision is low meaning a lot of neonates that needed to be cooled were not able to get the treatment whereas on the upside, the system was 91% precise in predicting the treatment for the ones who needed it.

TABLE 3: CONTIGENCY TABLE OF THE PERFORMANCE USING PROPOSED APPROACH

		System output		Accuracy%
		**1**	**2**	
**Actual Grade**	**1**	19	*2*	90.4
	**2**	*7*	21	75
**Precision%**		73	91.3	

IV. Conclusion

A novel system of grading HIE injury using HRV in neonates is proposed. This was a proof of concept study to show the importance of HRV for the classification of HIE. The results are promising for grading the HIE severity which could help the clinical staff for making a decision to initiate TH. Furthermore, it may allow the use of this tool as an early warning system for brain injury in neonates in remote areas where the EEG is not readily available. Additionally, It has paved the way towards a system that can provide more reliable classification of HIE by combining EEG and ECG using a multi-stream approach. The dataset for this study was not collected primarily for HRV analysis so the HRV quality was not in good condition. We expect that the results could be further improved with a better quality dataset. The feature set could be further investigated and expanded to get more information from the HRV and decrease the effects of artifacts.

References

[1] E. M. Graham, K. A. Ruis, A. L. Hartman, F. J. Northington, and H. E. Fox, “A systematic review of the role of intrapartum hypoxia-ischemia in the causation of neonatal encephalopathy,” *Am. J. Obstet. Gynecol.*, vol. 199, no. 6, pp. 587–595, Dec. 2008.

[2] D. V. Azzopardi et al., “Moderate hypothermia to treat perinatal asphyxial encephalopathy,” *N. Engl. J. Med.*, vol. 361, no. 14, pp. 1349–1358, 2009.

[3] V. Matić et al., “Heart rate variability in newborns with hypoxic brain injury,” *Adv. Exp. Med. Biol.*, vol. 789, pp. 43–48, 2013.

[4] R. M. Goulding, N. J. Stevenson, D. M. Murray, V. Livingstone, P. M. Filan, and G. B. Boylan, “Heart rate variability in hypoxic ischaemic encephalopathy: correlation with EEG grade and two-year neurodevelopmental outcome,” *Pediatr. Res.*, Feb. 2015.

[5] V. Novak, P. Novak, M. deMarchie, and R. Schondorf, “The effect of severe brainstem injury on heart rate and blood pressure oscillations,” *Clin. Auton. Res.*, vol. 5, no. 1, pp. 24–30, Feb. 1995.

[6] O. M. Doyle, A. Temko, W. Marnane, G. Lightbody, and G. B. Boylan, “Heart rate based automatic seizure detection in the newborn,” *Med. Eng. Phys.*, vol. 32, no. 8, pp. 829–839, Oct. 2010.

[7] R. Ahmed, A. Temko, W. Marnane, G. Boylan, and G. Lightbody, “Grading brain injury in neonatal EEG using SVM and supervector kernel,” in *Proc. IEEE ICASSP*, 2014.

[8] W. M. Campbell, D. E. Sturim, D. Reynolds, and A. Solomonoff, “SVM Based Speaker Verification using a GMM Supervector Kernel and NAP Variability Compensation,” in *Proc. IEEE ICASSP*, 2006.

[9] X. Zhuang, X. Zhou, M. A. Hasegawa-Johnson, and T. S. Huang, “Real-world acoustic event detection,” *Pattern Recognit. Lett.*, vol. 31, no. 12, pp. 1543–1551, Sep. 2010.

[10] D. M. Murray, G. B. Boylan, C. A. Ryan, and S. Connolly, “Early EEG findings in hypoxic-ischemic encephalopathy predict outcomes at 2 years,” *Pediatrics*, vol. 124, no. 3, pp. e459–467, Sep. 2009.

[11] J. Pan and W. J. Tompkins, “A real-time QRS detection algorithm,” *IEEE Trans. Biomed. Eng.*, vol. 32, no. 3, pp. 230–236, Mar. 1985.

[12] D. Aliefendioğlu, T. Doğru, M. Albayrak, E. Dibekmısırlıoğlu, and C. Sanlı, “Heart rate variability in neonates with hypoxic ischemic encephalopathy,” *Indian J. Pediatr.*, vol. 79, no. 11, pp. 1468–1472, Nov. 2012.

[13] D. Reynolds, “Gaussian Mixture Models,” in *Encyclopedia of Biometrics*, S. Z. Li and A. Jain, Eds. Springer US, 2009, pp. 659–663.

[14] J. Gauvain and C.-H. Lee, “Maximum a posteriori estimation for multivariate Gaussian mixture observations of Markov chains,” *IEEE Trans. Speech Audio Process.*, vol. 2, pp. 291–298, Apr. 1994.

[15] J. C. Platt, “Probabilistic Outputs for Support Vector Machines and Comparisons to Regularized Likelihood Methods,” in *Advances in Large Margin Classifiers*, 1999, pp. 61–74.

Authorized licensed use limited to: CSIRO Information Technology Services. Downloaded on September 09,2022 at 00:27:04 UTC from IEEE Xplore. Restrictions apply.
