# Supplementary material for: Exploring Computational Techniques in Preprocessing Neonatal Physiological Signals for Detecting Adverse Outcomes: Scoping Review
Source: Interact J Med Res. 2024 Aug 20;13:e46946. doi: 10.2196/46946 (PMC11372324; doi:10.2196/46946)
Supplement: Multimedia Appendix 3 [file ijmr_v13i1e46946_app3.zip › Included Papers - Final/3086/Y. Hu et al. - 2018 - Prediction of clinicians' treatment in preterm inf.pdf]

Prediction of Clinicians' Treatment in Preterm Infants with Suspected Late-onset Sepsis - An ML Approach

Yifei Hu
Faculty of Information Technology
Monash University
Melbourne, Australia
yifei.hu@monash.edu

Vincent.C.S.Lee
Faculty of Information Technology
Monash University
Melbourne, Australia
vincent.cs.lee@monash.edu

Kenneth Tan
Monash Newborn Clinical Department
Monash Children Hospital
Melbourne, Australia
kenneth.tan@monash.edu

***Abstract*—As a prevalent disease of preterm infants, late-onset neonatal sepsis has taken up a huge proportion of morbidity and mortality of newborn babies. We have been continuously capturing vital signs of preterm infants in NICU, and proposed a non-invasive method based on machine learning techniques to predict the clinicians' treatment on them. Then we provide evaluation of predictive models and prove their feasibility. Our models could help the pediatricians make wiser clinical decision, such as more accurate treatment, avoiding the abuse of antibiotics to some extent.**

***Keywords*—*machine learning, neonatal sepsis, prediction, vital signs* (*key words*)**

I. Introduction

Neonatal sepsis is a very common disease for new born babies especially preterm ones due to their immature immune system. It is classified as early-onset sepsis (EOS) which occurs within 48 hours after birth and late-onset sepsis (LOS) beyond 48 hours [1]. Both EOS and LOS have high mortality rates which are 25.9% and 15.1% respectively[2]. EOS is mostly caused by maternal risk factors such as drugs and illness, and diagnosis standard has been set up for it. LOS is, however, more difficult to be precisely detected as it has no perfect routine to follow. Hence, this research focuses on LOS. In the NICU of Monash Children Hospital, the diagnosis procedure follows a specific diagnosis guideline.

For EOS cases, clinicians need to do some investigations, then check both clinical signs and particular laboratory markers to see if they are beyond the thresholds. For LOS cases, the response of preterm infants to LOS could be subtle. There are certain risk factors that cause LOS and clinical signs could indicate the onset. Normally, 48 hours after birth clinicians will pay more attention to LOS rather than EOS. Signs including apnoea, pale skin, worse tolerance of intragastric feeding and increase lethargy all show the possibility of infection. Besides, temperature instability and septic shock can appear in preterm infants when they are infected. Doctors will do some laboratory tests such as blood count test to help them with the decision making when infants show some suspicious signs. Typically, infected infants get neutropenia and thrombocytopenia due to the consuming of white cells and platelets, and consequently immature neutrophils to total neutrophils (I/T) ratio becomes higher which makes it a predictor in the early stage as well as the C-Reactive Protein. In modern hospitals, paediatricians tend to use blood culture test as the golden standard for the diagnosis of LOS, but blood culture test takes 2-3 days in Melbourne hospitals, they will miss the best time of treatment if clinicians wait until the results come out. Usually doctors give them antibiotics while starting the blood culture test. Unfortunately, in this case, treatment before the test result will cause huge amount of unnecessary use of antibiotics, on average only one out of twenty-four infants who are given antibiotic treatments is blood culture proven sepsis which makes great contribution to drug resistance and increases the cost of healthcare in the future[3]. Another problem using blood culture test as the golden standard is that the blood samples are easily contaminated which will affect the accuracy of the test results[4].

Machine Learning (ML) is a rapidly developing technique in recent years and data science researchers have applied it in healthcare problems. Mani et al[3] proposed an early detection method using ML techniques for late-onset neonatal sepsis. Their retrospective study merged antibiotics, microbiology, laboratory and nursing documentation datasets from off-the-shelf EMR records and fed them into different ML algorithms. Experiments showed that both the sensitivity and specificity of ML algorithms exceeds those of clinicians'. Another similar research by Griffin et al [5] which explains the connection between neonatal sepsis and heart rate (HR). They found that in the course of neonatal sepsis, there exists a transient deceleration in HR while the variability of HR will shrink. Based on those observations they calculate Heart Rate Characteristics (HRC) as the index of risk of being infected, and use this HRC as a marker to predict adverse event such as neonatal infection, urinary tract infection (UTI) and death. However, there exists certain limitations in the HRC method, for example, illness other than sepsis and UTI can cause abnormal HRC as well as surgery effects, which influence the accuracy of prediction. Some other methods are also used in the early detection of neonatal sepsis especially late-onset one. A recent research explored the usage of Autoregressive Hidden Markov Model (AR-HMM)[6]. This paper first defined critical physiological events observed in the patient's monitoring course, then tried to model them with an AR-HMM model with which the onset of the infect was predicted real-time, but the lack of samples makes the accuracy of this method not satisfactory with an f score just over 0.6.

978-1-5386-3758-6/18/$31.00 ©2018 IEEE

Authorized licensed use limited to: CSIRO Information Technology Services. Downloaded on September 09,2022 at 00:45:29 UTC from IEEE Xplore. Restrictions apply.

TABLE I. STRUCTURE OF FEATURE VECTORS

Content	HR	Max, Min, Mean, Medium, Var, PtP	RR	Max…	SpO2	Max…
Size	60	6	60	6	60	6

In this paper, we investigate ML models in early detection of late-onset neonatal sepsis. If we can provide precise prediction of the infection before clinicians' judgement, or in other words before the blood culture test, we can save them valuable time for treatment and on the other hand reduce the unnecessary usage of antibiotics. As in the pilot research stage, our aim is to check the feasibility of predicting the onset of sepsis 24 hours ahead with pure basis vital signs collected from bedside monitoring machines, without any laboratory test data (e.g. blood count). Once the accuracy reaches a satisfactory level, it could provide medical staff with useful advice and help the make wiser decisions.

II. METHODOLOGY

A. Data Source

To collect vital signs of preterm infants for ML models training, we collaborate with NICU of Monash Children Hospital. As no digital health data of infants was stored in the NICU, we set up two independent laptops as data collection servers to collect real-time vital signs of preterm infants. The bedside monitoring machines are manufactured by Drager Medical, and they broadcast all the parameters it is monitoring to the local area network within the same VLAN which makes it possible to capture some basic vital signs of newborns under intensive care in NICU 24/7. We use eDATA-grabber (developed by Drager), which is more like a corresponding client to the bedside machine to collect data.

The data collection task lasted in a period of one month from 23rd October 2017 to 23rd November 2017. We could not capture all the patient data during that time due to a variety of issues such as unexpected laptop shutdown, network issues, NICU duty nurse's intervention and so on. All the physiological data used in our model training were from 32 cots in NICU, and the microbiology test outcome and clinician treatment details are extracted from the EMR system in the hospital. We define one patient-day as the data of one person within one day, and at the end of data collection task, totally we have successfully retrieved 407 patient-day of physiological data. Figure 1 shows the global structure of a neonatal monitoring system.

B. Pre-processing

The bedside monitoring machine has a sampling rate of 200Hz and is able to output one record in csv format every second. Data is accumulated at a very high speed and will bring excessive pressure to data storage, and such frequent data is not necessary for ML tasks. Thus, we scale down the data to one record per minute by sampling, which reduces the size of the dataset to 1/60. The variables used are chosen by experienced paediatricians, and given the limitation of the information bedside monitoring machine broadcasts, we only use heart rate (HR), respiratory rate (RR) and blood oxygen saturation (SpO2) as input vital signs.

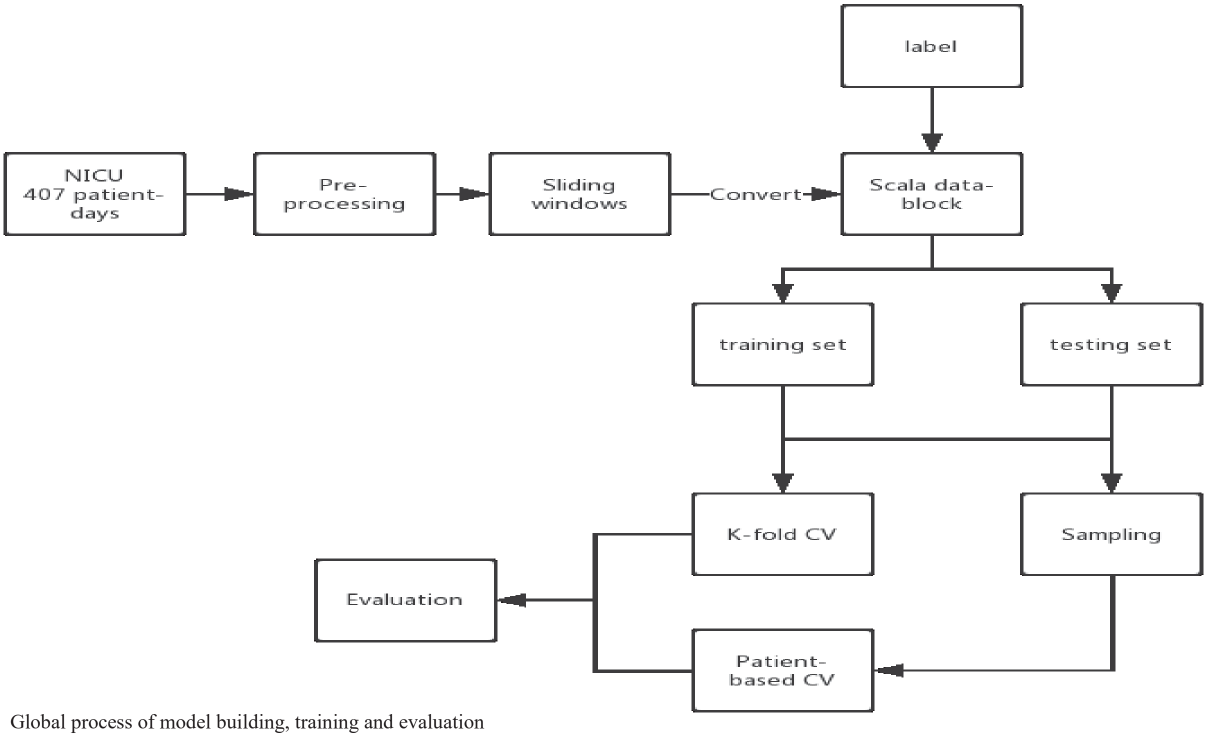

Fig. 1. Global process of model building, training and evaluation

Authorized licensed use limited to: CSIRO Information Technology Services. Downloaded on September 09,2022 at 00:45:29 UTC from IEEE Xplore. Restrictions apply.

Monitored vital signs can only indicate the status of the patients, we need labels to train our models as all the models we used are supervised. Our target is to predict the clinicians' treatment as we are at the preliminary stage of the research. In most cases, clinicians would start using antibiotic while they found an infant suspicious and ordered blood culture test, so we define two types of patients which are "suspicious" and "normal". Any one conducted blood culture test or accepted antibiotic treatment should be labelled "suspicious" and the label "normal" belongs to the rest. If one baby never did blood culture test or accepted antibiotics, he is absolutely classified into normal group, however if there existed suspected sepsis in this baby, the situation becomes subtle. Let the time of blood culture test or antibiotic therapy be denoted as t_0, and the time of ceasing antibiotic as t'. Since we want to detect the sepsis 24 hours ahead, suspicious data should be expanded from t_0 to t_0-24. Note that usually the last 24 hours in the antibiotic treatment patients are almost fully recovered, so we set the end point of suspicious data at t'-24, and all data fall into t_0-24 to t'-24 is labelled "suspicious" while the rest is labelled "normal".

The collected data stream is represented as temporal sequences of a variety of parameters. To feed them into normal ML models, we performed some pre-processing steps before training. We introduced sliding window method to convert temporal data flow to scalar data blocks. Specifically, raw data is cut into data blocks by sliding windows according to time stamps. The width of sliding windows is set to 60 minutes, which means in every data block we have 60 records of each variable since the sampling rate is 1Hz. For each variable, not only do the 60 data points account for input features, we also calculate extra statistical parameters for 60 minutes. The statistical parameters are maximum and minimum value, mean and variance, medium value, and peak-to-peak value. Finally, each variable contributes 60 data points within the sample hour plus another 6 extra statistical parameters which totally are 66 input features. Then the features from three variables (HR, RR, and SpO2) are concatenated to final input feature vectors including 198 features, see table 1. When sliding the window, different step sizes are applied based on labels. The step size is 60 minutes when the label is "normal" and reduces to 10 minutes when the label is "suspicious". The reason we do this is that the number of normal cases and suspicious cases are extremely imbalanced, the former one is much more than the latter one. If we train ML models directly using these imbalanced data, they tend to classify all the data as "normal" because in this way a better accuracy can be achieved. We set the step size smaller than window size in "suspicious" cases, so that there will be certain parts overlapping between two neighbouring windows, and in this case, with the same size of data stream we are able to create more data blocks, in other words training samples with "suspicious" label. Note that the overlapping in our data samples will not increase the chance of overfitting because the relative positions of overlapping data in each data block are different. However, even expanding the "suspicious" data by overlapping the window, there is still a huge gap in terms of the amount between "normal" and "suspicious" data. We sampled "normal" data to make sure the gap between two classes is acceptable.

Since the infants in NICU have moved to other location and sensors may detach from the body of infants, there could be missing or error values among the dataset we have collected. Prior importing data to ML models, we have to filter out these invalid data. Two different strategies of data cleaning were tested, one is to delete the data block with missing or error data directly, the other is to replace them the mean value. It turned out both strategies were led to hardly any differences between the final results, so we decided to choose the simpler one which is just delete the data block with invalid values. With all the pre-processing procedures done, finally we have got totally 4412 data samples of which 3451 are with "normal" label and 961 are with "suspicious".

C. Models fitting

A representative series of ML classification models were chosen for this suspicious patient prediction task. We chose Logistic Regression (LR), Support Vector Machine with Gaussian kernel (SVM), Random Forest (RF), Gradient Boosting Decision Tree (GBDT), and Multi-layer Perceptron Neural Network (NN) as target models, and trained them with pre-processed feature vectors with comparison.

In order to understand the insight of the medical data we have collected and train a model with as good performance as possible, ML models are trained and tested under two different training schemes:

1) K-fold cross validation

In this scheme, all 4421 data samples were input to the algorithm without dividing into training set and testing set. We set k as 4 which means the data were going to be divided into four segments after a random shuffle, and each segment would be taken as testing set for the model trained by the rest three segments.

Note that given the random shuffle before the segmentation, data samples from different patients will be mixed. Our target is to test the prediction performance of chosen models under this scheme, because they could possibly learn from some patients and the data from the same patients occur in the testing set we could check whether our model could predict the label correctly.

2) Patient-based validation

Unlike K-fold Cross Validation, we did not mix data from different patients. We shuffle patients instead of data samples, in order to keep the normal-suspicious ratios of training and testing data are at the same level. "Normal" and "Suspicious" patients are shuffled separately but split into training set or into testing set at the same ratio of 7:3 so we have 2416 "normal" data samples and 673 "suspicious" data samples in the training set, as for the testing set, the numbers are 1035 and 288 respectively. Once a patient is chosen to be in training or testing set, all data samples from him will be included in and only in that set, in other words, data from one patient will not be in training and testing set simultaneously.

The purpose of designing this scheme is to verify the generalization ability of our models. What the models do is to predict the condition of a group of patients by learning from

Authorized licensed use limited to: CSIRO Information Technology Services. Downloaded on September 09,2022 at 00:45:29 UTC from IEEE Xplore. Restrictions apply.

another group of patients, which we believe is closer to the real circumstance in the NICU.

We trained the five models mentioned above with scikit-learn [7], which is a python-based machine learning tool kit. For fine-tuning purpose, grid search method is employed. We have to cross check a variety of values in order to find the optimal one in each model. If a candidate value list of one parameter is provided, the GridSearchCV module is able to train the model with all the values in the list respectively and give a score defined by users, picking up the parameter makes the model have the best performance. For example, we tried different numbers of trees in RF in {10, 20, 50, 100, 150}, and the max depth of each tree is set in {1, 3, 5, 10, 15}. Another point worth mentioning is that although some measurements have been taken in the pre-processing stage, "normal" data samples are still much more than "suspicious" ones, so we use Cost-Sensitive Learning method during the training process, applying a higher weight to "suspicious" samples so that the error is enhanced when they are wrongly classified. In this way, the classifier care more about "suspicious" samples of small amount, and prevent the tendency of the bias to "normal" label which is the majority of the entire dataset.

III. Results

In this section, we report the result of the five models in two training schemes.

1) K-fold Cross Validation

Table 2 shows the performance of five classification models trained by the dataset collected from 23rd Oct to 23rd Nov with 4-fold cross validation. Note that for LR, SVM and NN, we normalized the data samples before training, scaling them down with a mean of 0 and a variance of 1, just to make sure the model wouldn't be biased to the features with larger values. RF and GBDT do not need the normalization though because they are both tree-based model.

TABLE II. Performance of five models in Patient-based CV

Model name	*Precision*	*Recall*	*Weighted_F1*	*AUC*
LR	0.21	0.52	0.53	0.50
SVM	0.20	0.27	0.62	0.47
RF	0.89	0.61	0.90	0.95
GBDT	0.88	0.74	0.92	0.97
NN	0.42	0.20	0.61	0.63

We can see that LR, SVM, and NN did not perform very well compared to two ensemble learning models - RF and GBDT. Since the dataset has to be not linear separable, the linear model LR had a poor precision on the existing data, and SVM seems did not work so well in this case either, both precision and recall were not acceptable. NN is capable of learning from a non-linear dataset, but it may need extra techniques or more complex network structure to reach a better performance. The f1 value in the table is weighted according to the proportion of the data given the certain label, so it may be larger than precision and recall. Two tree-based ensemble learning models had a good performance with both f1 and AUC larger than 0.9, with which we believe that it is possible to separate the infants with suspected sepsis with healthy ones.

2) Patient-based Validation

We also conducted some test in this more realistic situation in which models are trained with data of some patients and predictions are made on others. The parameters of each model are taken from the best model in 4-fold cross validation scheme. To reduce the error, we run each test four times and calculate the mean value as the final results. Table 3 provides the final performance measurements. In this training scheme, the difference among five models becomes small, but still RF and GBDT have the best performance in terms of Area under the receiver operator characteristics curve (AUC). Except f1 score, AUC was used as the general measurement because it provides an efficient measurement regardless of the size of data and is almost not sensitive to the imbalanced data samples [8]. Figure 2 shows the ROC curves of all the five models under two training schemes

TABLE III. Performance of five models in 4-fold CV scheme

Model name	*Precision*	*Recall*	*Weighted_F1*	*AUC*
LR	0.69	0.65	0.67	0.64
SVM	0.65	0.70	0.64	0.61
RF	0.72	0.71	0.61	0.77
GBDT	0.72	0.72	0.64	0.75
NN	0.61	0.69	0.60	0.50

IV. Discussion

The ultimate target of our research is to predict the late-onset neonatal sepsis with physiological data using ML models. As in the first stage, we planned to train the algorithms to have the same performance as the clinicians, once its predictive ability reaches the human level, more complex models and more advanced training methods will be involved.

A. Data review

To have a better understanding of the research, we did a chart review on the data first. Figure 3 shows the average values of HR, RR and SpO2 in each one-hour data sample. From this figure, we can see that there are gaps between normal and suspicious data samples. Heart rate and saturation from normal cases are higher than those from suspicious cases, while the respiratory rate of suspicious patients is higher. Another difference could be noticed is suspicious patients seem to have a periodical saturation, and it is higher than normal patients. All the differences between these two classes of data proved that they are theoretically separable, and that is the reason we add these statistical variables into feature vectors. The implementation of Random Forest in scikit-learn provides a method to investigate the feature importance. Fifteen out of eighteen statistical variables are ranked in top 20 important features, which implies that they play a significant role in identifying data samples from suspicious patients in this study. From the medical point of view, some

Authorized licensed use limited to: CSIRO Information Technology Services. Downloaded on September 09,2022 at 00:45:29 UTC from IEEE Xplore. Restrictions apply.

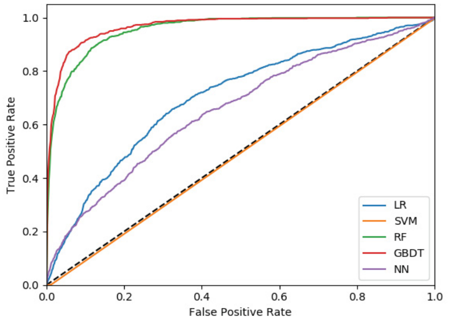

Fig. 2a. ROC curves in two 4-fold CV schemes

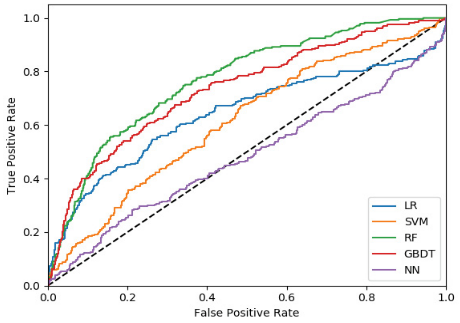

Fig. 2b. ROC curves in patient-based validation schemes

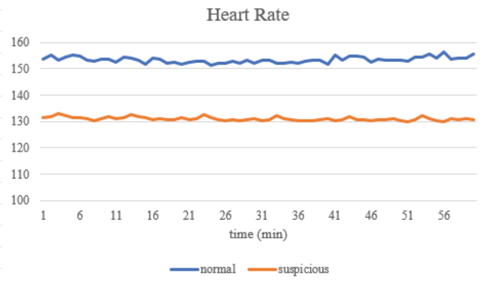

Fig. 3a. Chart review of heart rate

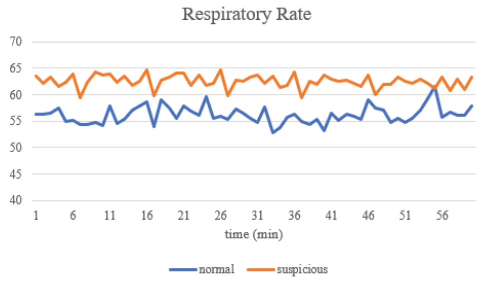

Fig. 3b. Chart review of respiratory rate

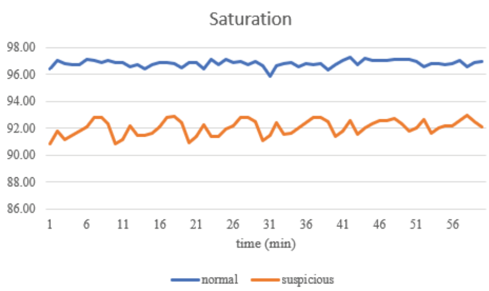

Fig. 3c. Chart review of blood oxygen saturation

measurements in the laboratory test such as CRP and neutrophil count may be more determinant in sepsis detection, we will consider the incorporation of extra clinical variables in the future.

Since the data monitoring is continuously going like a stream, they are really as temporal data sequence or time-series data if the data value are real numbers. Some of the existing temporal data mining methods are not suitable for our case, such as CBS proposed in [9] which extracted subsequences as class specific features and then use them to build a classifier. However, this algorithm works on a premise that the data points in the temporal sequence are discrete and limited in a certain range, which our continuously recorded data are not. The approach used in [10], [11] simply represents a sequence value using its initial value in the same order without any pre-processing. Improved methods in [12], [13] was trying to find a linear approximation of the time-series data. To be precise, time-series data are segmented and the approximation is done segment-wise. It is a good direction to explore, but we assume that this method should be modified a bit before applied in our dataset, because the difference of vital signs in normal and suspicious patients is trivial.

B. Evaluation of the results

In 4-fold cross validation scenario, RF and GBDT performed much better than the other three models, and this should be attributed to their innovative strategies. RF is an enhanced version of decision tree with bagging strategy. The two-step random sampling ensures the model will not easily overfit even without pruning. Every single tree in GBDT tries to fit the residual of its former one's result, when sum them up, the final result will be much better. Ensemble learning strategies make these two algorithms more capable of learning from large size of data with complex feature combination.

In comparison to the first scheme, the AUC of RF and GBDT are lower in patient-based validation scheme. We believe it is because of the nature of the data. Under this training scheme, training data and testing data are from different patients, the patterns models learned from one patient may not be applicable to others. Every individual infant may have his unique reaction to the infection and produces a special pattern in physiological data streams due to different body conditions. Therefore, our models can not learn the pattern of a particular patient from others, unless we have a larger enough cohort of patient which can cover most cases happen to infected infants. However, since the hospital didn't keep backups of vital signs data of patients used to be in NICU, and the data collection task has been doing for just one month, all

Authorized licensed use limited to: CSIRO Information Technology Services. Downloaded on September 09,2022 at 00:45:29 UTC from IEEE Xplore. Restrictions apply.

the data we have were from less than 80 individual infants which obviously can not represent all the neonatal sepsis patients.

C. Limitations

Our study has several limitations. First, the data we used have only three parameters, which are heart rate, respiratory rate and blood oxygen saturation. Limited by the monitoring machine the NICU is using, those are only parameters we can get at the moment. Data from fewer dimensions could reveal less information about the patient, even miss some key points about the respond to certain disease in some patient. We are negotiating with the hospital and trying to have access to more medical systems in order to gather data from more dimensions such laboratory test results, which may improve the predictive performance of our models. Second, it is about the amount of data. We collected data in only one month of patients in only one hospital, and a lot of them are from the same patients. Limited time and number of patients make our data samples not representative, and the data we currently have cannot cover most general patterns which is one of the reason our models are lack of generalization ability. Third, the diversity of features is not rich enough. Except for the raw data points, we only added six basic statistical measurements into the feature vector. For most cases, it is always better to have more features, which can describe the data itself, especially training with ensemble models like Random Forest which is not easily get overfitting. Feature selection would be a very domain knowledge based task, so we will talk to doctors and ask for their advice.

D. Future work

There are some improvements on our current work we could do in the next step of research. First and the most, we are going to keep gathering data from the bedside monitoring machines. As time going, we will accumulate more and more data no matter they are from suspicious patients or normal. By cooperating with the hospital and the manufacturer of the monitoring machine, the dimension of the data should be expanded as well. Once we have accessed to more data, we will explore the possibility of incorporating different forms of data like images and unstructured words, and apply deep learning techniques to deal with more complex and high dimension data. Consider the data points of a patient are the pixels in an image, sepsis detection problem is transformed to an image recognition problem. It has been widely believed that Convolutional Neural Network (CNN) works fine in image recognition so it is worth a try to apply a modified version of CNN in our research.

V. Conclusion

In this paper, we investigated the possibility of predicting the clinicians' treatment towards the preterm infants who are in danger of neonatal sepsis with a series of machine learning models based on purely physiological data. To gather the data, we collaborated with Monash Children Hospital and set up two laptops as the servers in the NICU. Sliding window strategy was employed to transfer the data to the form that normal machine learning models could process. Given the condition that normal and suspicious data samples are extremely imbalanced, we tried to reduce the negative influence in three ways, down-sampling the normal data, overlapping the suspicious data to increase the number of samples in this class and assigning different weight to the two classes during the model training process. Besides, all models are trained under two schemes with different purpose. The k-fold cross validation is to verify the ability of the prediction theoretically, but the patient-based validation is to put models in a more realistic situation and find the weak spots of our study. Our models are able to predict the onset of neonatal sepsis 24 hours ahead which brings clinicians more opportunities to restrain the infection before it actually harms the baby. Although the performance was not perfect, we believe with further optimization, it could provide useful information for paediatricians to make a wiser clinical decisions pertaining to the antibiotic treatment in which way will reduce the possibility of drug resistance in the future.

References

[1] D. of H. & H. Services, "Sepsis in neonates." [Online]. Available: https://www2.health.vic.gov.au:443/hospitals-and-health-services/patient-care/perinatal-reproductive/neonatal-ehandbook/infections/sepsis. [Accessed: 30-Nov-2017].

[2] C. P. Hornik et al., "Early and late onset sepsis in very-low-birth-weight infants from a large group of neonatal intensive care units," Early Hum. Dev., vol. 88, no. SUPPL.2, pp. S69–S74, 2012.

[3] S. Mani et al., "Medical decision support using machine learning for early detection of late-onset neonatal sepsis," J. Am. Med. Inform. Assoc., vol. 21, no. 2, pp. 326–336, 2014.

[4] N. Modi et al., "A case definition for national and international neonatal bloodstream infection surveillance," Arch. Dis. Child. - Fetal Neonatal Ed., vol. 94, no. 1, pp. F8–F12, Oct. 2008.

[5] M. P. Griffin, D. E. Lake, E. A. Bissonette, F. E. Harrell, T. M. O'Shea, and J. R. Moorman, "Heart Rate Characteristics: Novel Physiomarkers to Predict Neonatal Infection and Death," Pediatrics, vol. 116, no. 5, pp. 1070–1074, 2005.

[6] I. Stanculescu, C. K. I. Williams, and Y. Freer, "Autoregressive Hidden Markov Models for the Early Detection of Neonatal Sepsis," IEEE J. Biomed. Health Inform., vol. 18, no. 5, pp. 1560–1570, 2014.

[7] F. Pedregosa et al., "Scikit-learn: Machine learning in Python," J. Mach. Learn. Res., vol. 12, no. Oct, pp. 2825–2830, 2011.

[8] T. Fawcett, "An introduction to ROC analysis," Pattern Recognit. Lett., vol. 27, no. 8, pp. 861–874, Jun. 2006.

[9] V. S. Tseng and C.-H. Lee, "Effective temporal data classification by integrating sequential pattern mining and probabilistic induction," Expert Syst. Appl., vol. 36, no. 5, pp. 9524–9532, Jul. 2009.

[10] L. Lin and T. Risch, "Querying continuous time sequences," in VLDB, 1998, vol. 98, pp. 170–181.

[11] R. A. K. Lin and H. S. S. K. Shim, "Fast similarity search in the presence of noise, scaling, and translation in time-series databases," in Proceeding of the 21th International Conference on Very Large Data Bases, 1995, pp. 490–501.

[12] V. Guralnik and J. Srivastava, "Event detection from time series data," in Proceedings of the fifth ACM SIGKDD international conference on Knowledge discovery and data mining, 1999, pp. 33–42.

[13] G. Das, D. Gunopulos, and H. Mannila, "Finding similar time series," in Principles of Data Mining and Knowledge Discovery, 1997, pp. 88–100.

Authorized licensed use limited to: CSIRO Information Technology Services. Downloaded on September 09,2022 at 00:45:29 UTC from IEEE Xplore. Restrictions apply.
