# Supplementary material for: Exploring Computational Techniques in Preprocessing Neonatal Physiological Signals for Detecting Adverse Outcomes: Scoping Review
Source: Interact J Med Res. 2024 Aug 20;13:e46946. doi: 10.2196/46946 (PMC11372324; doi:10.2196/46946)
Supplement: Multimedia Appendix 3 [file ijmr_v13i1e46946_app3.zip › Included Papers - Final/3112/S. Das et al. - 2019 - Bradycardia Prediction in Preterm Infants Using No.pdf]

Bradycardia Prediction in Preterm Infants Using Nonparametric Kernel Density Estimation

Subhasish Das*, Bahman Moraffah†, Ayan Banerjee*, Sandeep K.S. Gupta* and Antonia Papandreou-Suppappola†
*School of Computing, Informatics, and Decision Systems Engineering
Arizona State University, Tempe, Arizona
Email: {subhasish.das, abanerj3, sandeep.gupta}@asu.edu
†School of Electrical, Computer and Energy Engineering
Arizona State University, Tempe, Arizona
Email: {bahman.moraffah, papandreou}@asu.edu

***Abstract*—In this paper, we propose a statistical method to predict the onset of bradycardia in preterm infants without any prior knowledge. To model information on the QRS complex R wave, we exploit nonparametric methods to estimate the density. Our proposed method takes advantage of the kernel density estimator in order to provide a statistical guarantee of 95% accuracy. We also demonstrate our results through simulations to support our proposed method using preterm infant electrocardiogram (ECG) signals from a database. We show that the method achieves a 5% false alarm rate in predicting the onset of upcoming bradycardia events.**

I. Introduction

Infants who are born prior to completion of 37 weeks of gestation are termed as preterm infants. They are usually kept under strict care and monitoring since their vital organs are still in the process of initial development. Due to their premature birth, these infants are very susceptible to health problems like hypoxemia (low oxygenation of blood), apnea, cerebral problems, gastrointestinal problems, immune system problems and various others that have long and short term effects [1].

A critical problem in preterm infants is bradycardia, which is a slower than normal heart rate that indicates low blood oxygen levels [2]. Infant and preterm infant heart rate, which is the number of times the heart beats per minute, is usually over 100 beats per minute (bpm) [3]. During episodes of bradycardia, their heart rates are lower which leads to the reduction of blood velocity thus impacting the amount of oxygenated blood that can be circulated to the developing organs [4]. Bradycardia has both short and long term effects [5] with the most severe being loss of life. Early detection of bradycardia is thus crucial to avoid negative long term effects. It is also to be noted that since these are preterm infants that are being monitored, their sudden movements often cause motion artifacts which may be wrongly detected as the onset of bradycardia. Along with early detection false alarms must be addressed to ensure that real episodes of bradycardia are addressed in time. A false alarm is caused when normal heartbeats are incorrectly detected as bradycardia events. Various methods have been considered in the literature to predict the onset of bradycardia events. Most of these methods depend on metrics related to the R wave of the QRS complex, such as the peak-to-peak R-R interval (RRI) extracted from ECG signals [6]. In [7], point process theory was used to model instantaneous measures of RRIs between heart beats for use in predicting bradycardia prior to onset with a 15% false alarm rate. Bayesian online change point detection was used in [8] to estimate sequential transitions between RRIs that lead to bradycardia; the RRIs were modeled using a lognormal probability density function (PDF). Multivariate regression predictive modeling was used to identify clinically significant bradycardia parameters with a 5% level of confidence in [9]. Predictive modeling was also used in [10] employing decision trees with time-frequency based ECG features to classify active bradycardia events with 86.7% accuracy. Note that the aforementioned methods require features or prior knowledge of the occurrence of bradycardia.

In the recent years, nonparametric modeling has drawn a great deal of attention in many areas of research [11]–[13]. In this paper we propose a novel method to predict the onset of near-term bradycardia in preterm infants without prior knowledge. Our proposed nonparametric method robustly and accurately estimates a nonspecific probability density function of the continuous non-bradycardia ECG segments and constructs an RRI confidence set that depends on a desirable level of detection accuracy. We then introduce a test to achieve the false alarm rate of 5% by inverting the test. In particular, this method can achieve probability of false alarm, P_{FA}, as low as 5%, which is significantly more accurate compared to the existing methods. Our method does not require any prior features to be extracted from the ECG signal. We note that one can extend the proposed method to construct a confidence interval to predict other physiological transitions like different types of arrhythmias.

The rest of the paper is organized as follows - Section II presents our proposed method. In Section III-A, we discuss the dataset and the required preprocessing methods. We then introduce a metric to measure the prediction error in Section III-B. The experimental setup and results are thoroughly discussed in Section III-C-III-D. We demonstrate through simulation that this method can robustly predict the bradycardia for a given false alarm rate.

Authorized licensed use limited to: CSIRO Information Technology Services. Downloaded on September 09,2022 at 00:45:12 UTC from IEEE Xplore. Restrictions apply.

Preterm Infant	1	2	3	4	5	6	7	8	9	10
Duration (hours)	45.6	43.8	43.7	46.8	48.8	48.6	20.3	24.6	70.3	45.1
Bradycardia Events	77	72	80	66	72	56	34	28	97	40
ECG Segments	77	72	80	66	72	56	34	28	97	40

TABLE I: Duration of ECG, number of Bradycardia Events, number of ECG segments for ten pre-term infants.

II. Nonparametric Modeling to Predict Bradycardia

ECG data obtained from a preterm infant is first preprocessed and segmented (described in Section III-A) such that each segment contains both normal and bradycardia beats. Using continuous normal beats, we extract R-peak information and estimate our nonspecific probability density function. After setting a desired level of false alarm to be tolerated by the system, a threshold region is found using the estimated density and a generated threshold plane. We use R-peaks from further along in time and test against this threshold region to determine the onset of near-term bradycardia. Section III-A describes a statistical guarantee to achieve the desired false alarm rate while Section III-B presents a practical way to construct the prediction set.

A. Nonparametric Prediction Test

We construct a hypothesis test to predict bradycardia in infants' heartbeat. To this end, we employ a nonparametric method for estimating a kernel-based probability density function to model RRI information to use in predicting the onset of bradycardia. We consider ECG segments that are first processed to remove the baseline wander. We then detect the R peaks using Pan-Tompkins algorithm [6]. Assuming that the number of R peaks in an ECG segment is N. We define the R-tuple $x_n = (t_n, R_n)$, $n = 1, \dots, N$ and $x_n \in \mathcal{X}$, to be the time of the peak occurrence and the peak amplitude R_n, respectively. We assume that the R-tuple set $X_N = \{x_1,\ x_2,\ \dots,\ x_N\}$ is identically and independently drawn from an unknown density $p(x)$. We exploit the kernel density estimator to estimate $p(x)$ using the data points X_n as

$$\hat{p}_H(x) = \frac{1}{N}\sum_{n=1}^{N} K_H(x - x_n) \tag{1}$$

for the positive definite bandwidth matrix H and $K_H(x) = |H|^{-1/2}\, K\big(H^{-1/2}x\big)$ where K is smoothing kernel function in $\mathbb{R}$. The estimated density depends directly on the smoothing kernel K_H and the bandwidth H. For the simplicity, we assume that K_H is Gaussian and $H = h^2\, I_2$, where I_2 is the (2×2) identity matrix. We learn the best value of h using leave-one-out cross validation to ensure accurate prediction and avoid overfitting [14].

Given a desirable probability of false alarm, P_{FA}, we design a hypothesis testing that produces $(1 - P_{\text{FA}})$ confidence. We define the null hypothesis $\mathcal{H}_0$ as the hypothesis that the density of the next R-tuple x_{N+1} is the same as that of the previous R-tuples in the set X_N; that is, $\mathcal{H}_0$: $x_{N+1} = x$, for all possible values of $x \in \mathcal{X}$. We aim to construct a confidence set $\mathcal{A}_{\mathcal{X}}$, that consists of all values X_N, such that the probability of the next R-tuple, x_{N+1}, belonging to this set satisfies the following condition

$$\Pr(x_{N+1} \in \mathcal{A}_{\mathcal{X}}) \geq (1 - P_{\text{FA}}).$$

Note that inverting the test produces a prediction set. It is shown that the prediction set $\mathcal{A}_{\mathcal{X}}$ is finite and distribution-free [15]. To this end, we utilize the kernel density estimator $\hat{p}^a_H(x)$ based on the augmented data set $X_N \cup \{x\}$ for a fixed value of $x \in \mathcal{X}$. The rank of $\hat{p}^a_H(x_1), \dots, \hat{p}^a_H(x_{N+1})$ is uniformly distributed under the null hypothesis. Thus, For each value of x, the p-value η_x is given by

$$\eta_x = \frac{1}{N+1}\sum_{n=1}^{N} \mathbb{I}\Big(\hat{p}^a_H(x_n) \leq \hat{p}^a_H(x)\Big) \tag{2}$$

where $\mathbb{I}$ is the indicator function. We thus define the $(1 - P_{\text{FA}})$ confidence set as $\mathcal{A}_{\mathcal{X}} = \{x\ :\ \eta_x\ \geq\ P_{\text{FA}}\}$. As mentioned earlier the prediction set $\mathcal{A}_{\mathcal{X}}$ is distribution-free and is only determined using the finite set X_N [16]. Implementing this method is impractical, thus we introduce a bigger set that contains $\mathcal{A}_{\mathcal{X}}$ and can easily be constructed.

B. Unsupervised Prediction

Given the confidence set $\mathcal{A}_{\mathcal{X}}$, we can successfully predict the next bradycardia event with a 95% accuracy by setting $P_{\text{FA}} = 0.05$. However, as it may not always be possible to compute the set $\mathcal{A}_{\mathcal{X}}$, we construct instead a feasible but larger confidence set $\mathcal{B}_{\mathcal{X}}$ that is easier to compute and preserves the same accuracy. Define $y_n = \hat{p}_H(x_n)$ for $n = 1, \dots, N$. Assume that y_i is sorted in an ascending order, that is, $y_1 \leq \cdots \leq y_N$. We construct the prediction set $\mathcal{B}_{\mathcal{X}}$ as

$$\mathcal{B}_{\mathcal{X}} = \{x\colon \hat{p}_H(x) \geq \mathcal{C}_k\},$$

for the threshold plane is computed as $\mathcal{C}_k = y_k - (K_H(0)/N|H|^{1/2})$ where $k = \lfloor (N+1)P_{\text{FA}} \rfloor$.

Proposition 1 ([15] Theorem 3.4): The set $\mathcal{B}_{\mathcal{X}} \supset \mathcal{A}_{\mathcal{X}}$ satisfies

$$\Pr(x_{N+1} \in \mathcal{A}_{\mathcal{X}}) \geq (1 - P_{\text{FA}}) \implies \Pr(x_{N+1} \in \mathcal{B}_{\mathcal{X}}) \geq (1 - P_{\text{FA}}). \tag{3}$$

In particular, the prediction set $\mathcal{B}_{\mathcal{X}}$ follows from the projection of estimated density which is above the threshold $\mathcal{C}_k$.

III. Experimental Data and Results

A. Dataset and Preprocessing Data

To demonstrate the performance of our proposed method, we make use of the dataset provided by the MIT Preterm Infant

Authorized licensed use limited to: CSIRO Information Technology Services. Downloaded on September 09,2022 at 00:45:12 UTC from IEEE Xplore. Restrictions apply.

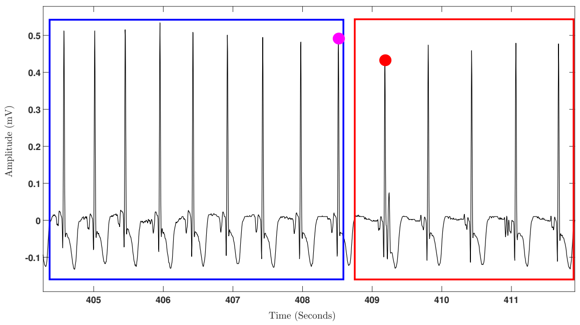

Fig. 1: Preterm infant ECG segments: normal region (blue box) and bradycardia (red box).

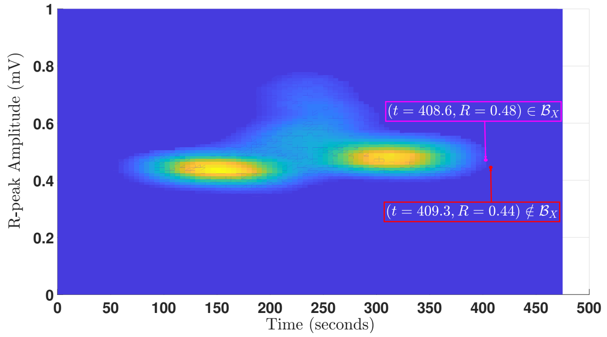

Fig. 3: Region $\mathcal{B}_{\mathcal{X}}$ obtained from the intersection of the density and the threshold region

Cardio-respiratory Signals (PICS) database [7] [17]. ECG data from ten preterm infants with post-conceptional ages ranging between $29\frac{3}{7}$ and $34\frac{2}{7}$ weeks are collected for approximately 20 to 70 hours per infant at a sampling frequency of 500Hz as shown in Table I. ECG data taken from each infant in the database is subject to the removal of baseline wander by using a high-pass filter with cut-off frequency between 0.5-0.6 Hz. We then remove the motion and disconnection artifacts from the signal by visual inspection. Following the aforementioned steps, we obtain the cleaned-up ECG signal for each infant in the database. To locate the R-peaks in the cleaned-up data, we employ the Pan-Tompkins algorithm [6]. Finally, each signal is segmented by using the annotations provided by the database regarding the location of occurrence of bradycardia events. Each signal contains both regular data points (data collected five minutes prior to the first bradycardia event) and bradycardia beats (data collected two minutes after the first bradycardia event).

Table I displays the number of bradycardia events and the number of ECG segments generated for each preterm infant in the dataset. For each infant, R-tuples are generated for each segment and utilized in our proposed model detailed in Section II.

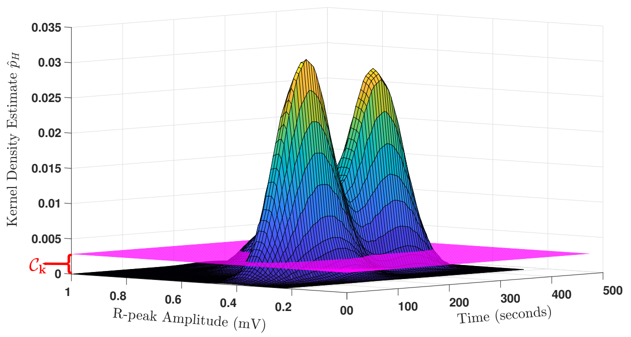

Fig. 2: Kernel density estimator $\hat{p}_H(x)$ and threshold region $\mathcal{C}_k$ for preterm infant 1 ECG segment;

B. Evaluation Metric

To evaluate our proposed method, we define the evaluation metric *estimated predictive error* (EPE) as

$$\text{EPE} = \frac{\text{Number of False Alarms}}{\text{Number of R-tuples tested}} \quad (4)$$

Given an ECG segment containing N peaks, a desired level of P_{FA}, and the prediction set $\mathcal{B}_{\mathcal{X}}$ over the normal heartbeats in that segment, we can compute the estimated predictive error by testing whether R-tuples from a future time belong to $\mathcal{B}_{\mathcal{X}}$. Our proposed method implies that the R-tuple $x_m = (t_m, R_m)$ is predicted, with (1 - P_{FA})% confidence, to be the onset of bradycardia if $x_m \notin \mathcal{B}_{\mathcal{X}}$, $m > N$. If the R-tuple x_m is a bradycardia R-tuple and $x_m \in \mathcal{B}_{\mathcal{X}}$, $m > N$, then it is a false alarm and counts towards the estimated predictive error.

The definition of the EPE indicates that, for a given probability of false alarm P_{FA}, lower value of the estimated predictive error results in the fewer false alarms being raised. Thus, EPE is a measure of performance which is used for different values of P_{FA} to demonstrate how well our proposed method performs.

For a fixed value of P_{FA}, we compute the total estimated predictive error for each preterm infant as the average of the estimated predictive error (EPE) over all ECG segments for that preterm infant. Similarly, lower value of total estimated predictive error indicates better performance of the method.

C. Experimental Setup

Using the preprocessed data from Section III-A, we perform the following procedure for each infant over each segment. For a desired false alarm rate P_{FA}, we choose he bandwidth parameter h through leave-one out cross-validation. Assuming a Gaussian kernel, we estimate the density to be $\hat{p}_H(x)$ for the normal heartbeats in each segment. We then compute the threshold plane $\mathcal{C}_k$ and generate the prediction set $\mathcal{B}_{\mathcal{X}}$ for each segment as the area under the intersection of the density $\hat{p}_H(x)$ and the threshold plane $\mathcal{C}_k$. We compute the estimated predictive error (EPE) by testing whether R-tuples from the near future (during and after the bradycardia event) belong to the prediction set $\mathcal{B}_{\mathcal{X}}$. If a future R-tuple from the bradycardia region belongs to $\mathcal{B}_{\mathcal{X}}$ then it is considered to be an error. The

Authorized licensed use limited to: CSIRO Information Technology Services. Downloaded on September 09,2022 at 00:45:12 UTC from IEEE Xplore. Restrictions apply.

Preterm Infant	1	2	3	4	5	6	7	8	9	10
$P_{\text{FA}} = 0.05$	0.092	0.099	0.092	0.04	0.04	0.19	0.08	0.194	0.091	0.087
$P_{\text{FA}} = 0.04$	0.095	0.284	0.387	0.29	0.31	0.45	0.31	0.295	0.223	0.26
$P_{\text{FA}} = 0.03$	0.35	0.457	0.681	0.321	0.38	0.425	0.498	0.342	0.195	0.46
$P_{\text{FA}} = 0.02$	0.82	0.68	0.943	0.91	0.87	0.9	0.89	0.694	0.83	0.87
$P_{\text{FA}} = 0.01$	0.89	0.901	0.991	0.99	0.99	0.94	0.99	0.82	0.998	0.907

TABLE II: Average estimated predictive error for ten pre-term infants for P_{FA} between 0.05 to 0.01

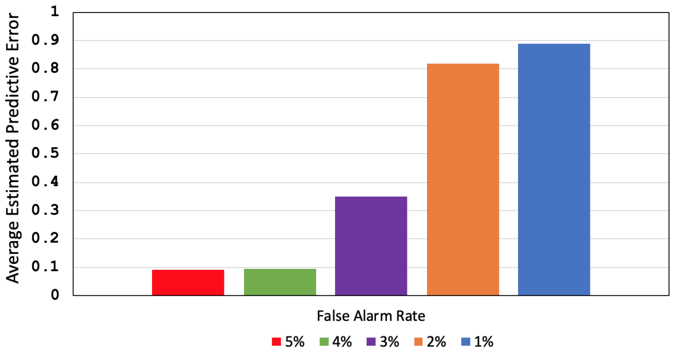

Fig. 4: Region $\mathcal{B}_{\mathcal{X}}$ obtained from the intersection of the density and the threshold region

above process is repeated for each preterm infant for the false alarm rates, P_{FA}, of 5%, 4%, 3%, 2% and 1%.

D. Results

Figure 1 displays a portion of an ECG segment for preterm infant 1 containing both normal and bradycardia heartbeats. Using leave-one-out cross validation, we estimate the bandwidth parameter h for the Gaussian kernel to be 33.2313. Figure 2 provides a pictorial demonstration of our proposed method for fixed value of $P_{\text{FA}} = 0.05$ and the kernel density estimator with the bandwidth $h = 33.2313$. In particular, we estimate the threshold plane $\mathcal{C}_k$ to be 0.0028 and used it to compute the prediction set $\mathcal{B}_{\mathcal{X}}$ for this specific segment. The contour region obtained from the intersection of the estimated density $\hat{p}_H(x)$ and plane $\mathcal{C}_k$ provides the prediction set $\mathcal{B}_{\mathcal{X}}$. The prediction set $\mathcal{B}_{\mathcal{X}}$ is shown in Figure 3. All R-tuples that lie inside this contour region are considered to be normal heartbeats. For the ECG segment shown in Figure 1, we tested two R-tuples that indicate the last normal beat (R-tuple $(408.6, 0.48)$ denoted by a pink marker) and the first bradycardia beat (R-tuple $(409.3, 0.44)$ denoted by a red marker). We observe that the R-tuple for the last normal beat lies just within the prediction set (denoted by the contour region) while the R-tuple for the bradycardia beat lies outside the contour region. Using this threshold region, we can predict the onset of near-term bradycardia by generating R-tuples from future ECG data and locating the first R-tuple that lies outside this region. We use ten random R-tuples from a future time for each segment to compute the estimated predictive error for the corresponding segment. As mentioned in Section III-B, the estimated predictive error value indicates how well our method performs at a desired P_{FA}. Low values of this metric imply that our method is able to predict the onset of bradycardia with the specified level of accuracy and rate of false alarm. This process is repeated for all ECG segments for preterm infant 1 for P_{FA} values ranging between 0.05 to 0.01 (5% to 1%). We compute the total predictive error for each P_{FA} value as shown in Figure 4 for preterm infant 1. Figure 4 demonstrates that our proposed method works best at $P_{\text{FA}} = 0.05$ and is able to predict the onset of bradycardia with 95% accuracy as expected. The total average estimated predictive error is lowest for this value of P_{FA} indicating very few bradycardia R-tuples are mispredicted. We also observe that as we lower the probability of false alarm the total estimated predictive error increases.

Similarly, we compute the total average estimated predictive error for each of the ten preterm infants for P_{FA} values of 5%, 4%, 3%, 2% and 1% shown in Table II. A similar trend can be observed across all ten preterm infants in the database - as the probability of false alarm P_{FA} is reduced, we see a significant increase in the average estimated predictive error (Table II). Hence we demonstrate that our proposed method is able to predict the onset of bradycardia with higher accuracy and low false alarm rate as compared to other existing methods.

E. Discussion

In the previous section, we demonstrated that our proposed method works and is able to predict the onset of near-term bradycardia with an accuracy of $(1 - P_{\text{FA}})$ for a desired probability of false alarm P_{FA}. We also showed that our proposed method achieves the highest prediction accuracy compared to prior work while maintaining the lowest rate of false alarm.

As shown in Figure 4 and Table II, we observe that as the P_{FA} value is lowered from 5% (0.05) to 1% (0.01), the total average estimated predictive error increases. The total average estimated predictive error is lowest at $P_{\text{FA}} = 0.05$ with a slight increase across all infants at $P_{\text{FA}} = 0.04$. The error increases drastically as the P_{FA} value is lowered to 0.03, 0.02 and 0.01. However, this trend lies within our expectation. By lowering the P_{FA} value, the contour region under the intersection of the threshold plane $\mathcal{C}_k$ and density $\hat{p}_H(x)$ increases that is, the prediction set $\mathcal{B}_{\mathcal{X}}$ grows. The enlargement of the prediction set allows for R-tuples that do not belong to the actual set to erroneously be included in the region. This fact results in the fact that bradycardia R-tuples are mistaken as normal beats - false alarms. In practice, it is not feasible to remove false alarms in the entirety. Instead,

Authorized licensed use limited to: CSIRO Information Technology Services. Downloaded on September 09,2022 at 00:45:12 UTC from IEEE Xplore. Restrictions apply.

we strive to minimize the rate of false alarms while trying to maximize the prediction accuracy as much as possible. Our proposed method demonstrates this and shows that it is not feasible to guarantee accurate predictions with an expect level of confidence beyond a certain value of P_{FA}.

IV. Conclusion

In this paper, we proposed a method to predict the onset of near-term bradycardia. Our proposed method provided an accurate prediction set that outperformed the existing methods. Our method exploited the R-peak information and estimated the corresponding density. Using the estimated density, we presented a prediction set that determined the onset of bradycardia in preterm infants for a desired P_{FA}. Our distribution-free method does not require prior knowledge of bradycardia events in the subject. We also demonstrated through simulations that the method can be successfully used to predict the onset of near-term bradycardia with an accuracy of $(1 - P_{\text{FA}})$. We showed that the elimination of false alarms in this situation is not completely possible and lowering P_{FA} may result in the higher total average probability of error.

Acknowledgment

The authors of this paper would like to thank Dr. Premananda Indic and Dr. Alan H. Gee for their valuable input on the PICS database.

References

[1] S. Blackburn, "Problems of preterm infants after discharge," *Journal of Obstetric, Gynecologic, & Neonatal Nursing*, vol. 24, no. 1, pp. 43–49, 1995.

[2] C. J. Upton, A. D. Milner, and G. M. Stokes, "Episodic bradycardia in preterm infants," *Archives of Disease in Childhood*, vol. 67, pp. 831–834, 1992.

[3] J. M. Perlman and J. J. Volpe, "Episodes of apnea and bradycardia in the preterm newborn: impact on cerebral circulation," *Pediatrics*, vol. 76, no. 3, pp. 333–338, 1985.

[4] G. Pichler, B. Urlesberger, and W. Müller, "Impact of bradycardia on cerebral oxygenation and cerebral blood volume during apnoea in preterm infants," *Physiological measurement*, vol. 24, no. 3, p. 671, 2003.

[5] C. F. Poets, R. S. Roberts, B. Schmidt, R. K. Whyte, E. V. Asztalos, D. Bader, A. Bairam, D. Moddemann, A. Peliowski, Y. Rabi *et al.*, "Association between intermittent hypoxemia or bradycardia and late death or disability in extremely preterm infants," *Jama*, vol. 314, no. 6, pp. 595–603, 2015.

[6] J. Pan and W. J. Tompkins, "A real-time QRS detection algorithm," *IEEE Transactions on Biomedical Engineering*, vol. 32, pp. 230–236, 1985.

[7] A. H. Gee, R. Barbieri, D. Paydarfar, and P. Indic, "Predicting bradycardia in preterm infants using point process analysis of heart rate," *IEEE Transactions on Biomedical Engineering*, vol. 64, pp. 2300–2308, 2017.

[8] A. H. Gee, J. Chang, J. Ghosh, and D. Paydarfar, "Bayesian online changepoint detection of physiological transitions," in *IEEE Engineering in Medicine and Biology Society*, 2018, pp. 45–48.

[9] H. Truong, "Predicting adverse outcomes in preterm infants using early bedside monitor data," 2018, thesis for Bachelor of Science in Physics, College of William and Mary, Williamsburg, VA.

[10] S. M. Mahmud, H. Wang, and Y. Kim, "Accelerated prediction of bradycardia in preterm infants using time-frequency analysis," in *Int. Conference on Computing, Networking and Communications*, 2019, pp. 468–472.

[11] A. Mittal and N. Paragios, "Motion-based background subtraction using adaptive kernel density estimation," in *Proceedings of the 2004 IEEE Computer Society Conference on Computer Vision and Pattern Recognition, 2004. CVPR 2004.*, vol. 2. Ieee, 2004, pp. II–II.

[12] B. Moraffah, "Inference for multiple object tracking: A Bayesian nonparametric approach," *arXiv preprint arXiv:1909.06984 cs.LG*, 2019.

[13] B. Moraffah and A. Papandreou-Suppappola, "Random infinite tree and dependent Poisson diffusion process for nonparametric Bayesian modeling in multiple object tracking," in *International Conference on Acoustics, Speech, and Signal Processing*, 2019, pp. 5217–5221.

[14] A. Celisse, "Optimal cross-validation in density estimation with the l^2-loss," *The Annals of Statistics*, vol. 42, pp. 1879–1910, 2014.

[15] J. Lei, J. Robins, and L. Wasserman, "Distribution-free prediction sets," *Journal of the American Statistical Association*, vol. 108, pp. 278–287, 2013.

[16] G. Shafer and V. Vovk, "A tutorial on conformal prediction," *Journal of Machine Learning Research*, vol. 9, pp. 371–421, 2008.

[17] A. L. Goldberger, L. A. N. Amaral, L. Glass, J. M. Hausdorff *et al.*, "PhysioBank, PhysioToolkit, and PhysioNet: Components of a new research resource for complex physiologic signals," *Circulation*, vol. 101, pp. e215–e220, 2000. [Online]. Available: http://physionet.mit.edu/physiobank/database/picsdb

Authorized licensed use limited to: CSIRO Information Technology Services. Downloaded on September 09,2022 at 00:45:12 UTC from IEEE Xplore. Restrictions apply.
