# Supplementary material for: Exploring Computational Techniques in Preprocessing Neonatal Physiological Signals for Detecting Adverse Outcomes: Scoping Review
Source: Interact J Med Res. 2024 Aug 20;13:e46946. doi: 10.2196/46946 (PMC11372324; doi:10.2196/46946)
Supplement: Multimedia Appendix 3 [file ijmr_v13i1e46946_app3.zip › Included Papers - Final/3126/C. A. Robles-Rubio et al. - 2014 - Automated analysis of respiratory behavior for the.pdf]

Automated Analysis of Respiratory Behavior for the Prediction of Apnea in Infants following General Anesthesia

Carlos A. Robles-Rubio, *EMBS Student Member*, Karen A. Brown, Gianluca Bertolizio, and Robert E. Kearney, *EMBS Fellow*

***Abstract*— Infants recovering from general anesthesia are at risk of postoperative apnea (POA), a potentially life threatening event. There is no accurate way to identify which infants will experience POA, and thus all infants with postmenstrual age < 60 weeks are monitored for apnea in hospital postoperatively. Using a comprehensive, automated analysis of the postoperative breathing patterns, we identified the occurrence of respiratory pauses in 24 infants at age risk for POA. We determined the POA category for each infant by using K-medoids to cluster the duration of the longest respiratory pause. Two clusters were identified, corresponding to APNEA and NO-APNEA, with a threshold of 14.6 s, a value consistent with the clinically accepted threshold of 15 s. K-medoids derived POA labels were used to evaluate the predictive ability of demographic and anesthetic management variables. Weight and the intraoperative doses of atropine, propofol, and opioids discriminated between the APNEA and NO-APNEA groups. A linear Gaussian discriminant analysis classifier provided a very good classification with a probability of detection P_D = 0.73 and a probability of false alarm P_{FA} = 0.22. This approach provides a promising tool for the systematic, objective study of infants at risk of POA.**

I. Introduction

Infants recovering from surgery and anesthesia are at increased risk of life threatening postoperative apnea (POA) [1, 2], a clinical entity associated with respiratory pauses in excess of 15 s. Risk factors identified to date include: age, prematurity, associated medical conditions, and the use of some anesthetic medications, including opioids. Although postmenstrual age (PMA) less than 60 weeks is an important risk factor [1, 3, 4], there is as yet no way to predict which of these infants will experience apnea [3]. Consequently, although POA occurs in only approximately 30% of infants [4], clinical practice guidelines recommend postoperative monitoring of all at risk infants [1].

Postoperative apneas are relatively rare events and consequently long postoperative cardiorespiratory records from many infants are required to study POA. Furthermore, the preferred method of analysis is manual scoring [5], which is labor intensive, expensive and suffers from low inter-scorer agreement [6]. These two factors have limited the acquisition and analysis of appropriate data sets.

To overcome this we developed AUREA [7], an Automated Unsupervised Respiratory Event Analysis system. AUREA automatically analyses the respiratory behavior classifying the respiratory state at each point in time into one of four categories: Pause, Movement Artifact, Asynchronous- and Synchronous-Breathing. This is achieved by K-means clustering [8] on metrics that extract the amplitude, frequency and phase information from the ribcage and abdomen signals obtained from respiratory inductive plethysmography (RIP).

The first objective of this study was to establish an automated, standardized way to define POA based on the occurrence of long respiratory pauses identified by AUREA, contrasted to the traditional approach based on clinical judgments at the bedside. The second objective was to combine this objective POA definition with demographic data and variables in the anesthetic management to train a classifier and assess its predictive ability.

The paper is organized as follows: Section II describes the subjects and data; Section III shows the analysis of the postoperative respiratory behavior and the definition of POA categories; Section IV presents the feature selection strategy and describes the classifiers used to predict POA occurrence; Section V gives the prediction results; and Section VI discusses the findings and provides concluding remarks.

II. Materials

A. Subjects

Twenty four infants (19 male, 3.7 ± 1.0 kg, PMA of 43 ± 2 weeks) who underwent elective general anesthesia for inguinal herniorrhaphy, and were at age risk for POA were recruited and studied in the Postanesthesia Care Unit (PACU) of the Montreal Children's Hospital (MCH). Table I gives demographic data and details of the anesthetic management including drug administration and dose.

Inclusion criteria were: (1) PMA < 60 weeks at the time of surgery for preterm infants and < 48 weeks for term infants; and (2) American Society of Anesthesiology physical status 1 or 2. Exclusion Criteria were: (1) emergency surgery; and (2) spinal anesthesia. The study was approved by the Institutional Review Board of McGill University Health Centre/MCH, and informed written parental consent was obtained for each infant. The anesthetic management was not standardized.

Research supported in part by the Natural Sciences and Engineering Research Council of Canada. The work of CARR was supported in part by the Mexican National Council for Science and Technology and in part by the Queen Elizabeth Foundation of the Montreal Chair in Pediatric Anesthesia. KAB was supported by the Queen Elizabeth Foundation of the Montreal Chair in Pediatric Anesthesia.

Carlos A. Robles-Rubio and Robert E. Kearney are with the department of Biomedical Engineering, McGill University, Montreal, QC H3A 2B4, Canada (e-mail: carlos.roblesrubio@mail.mcgill.ca; robert.kearney@mcgill.ca).

Karen A. Brown and Gianluca Bertolizio are with the department of Anesthesia, McGill University Health Center, Montreal, QC H3A 2B4, Canada (e-mail: karen.brown@mcgill.ca; gianluca.bertolizio@mcgill.ca).

978-1-4244-7929-0/14/$26.00 ©2014 IEEE

Authorized licensed use limited to: CSIRO Information Technology Services. Downloaded on September 09,2022 at 00:40:35 UTC from IEEE Xplore. Restrictions apply.

B. Data Acquisition

Upon arrival at the PACU, infant respibands (Ambulatory Monitoring Inc., Inductobands, Ardsley, NY) were placed around the infant's ribcage and abdomen and interfaced with Respiratory Inductive Plethysmograph (Ambulatory Monitoring Inc., Battery Operated Inductotrace, Ardsley, NY). No attempt was made to calibrate the RIP signals in absolute terms. An infant oximeter probe (Nonin 8600 Portable Digital Pulse Oximeter, Plymouth, MN) was taped to a digit. The outputs were low-pass filtered (cut-off frequency 10 Hz) with an 8-pole Bessel anti-aliasing filter (Kemo, Jacksonville, FL), digitized, sampled at 50 Hz, and recorded on a computer using MATLAB™ (The MathWorks Inc., Natick, MA) for off-line analysis. This acquisition system is described in [9]. Data were acquired for 9.0 ± 2.2 hours. Subsets of these data have been reported in other studies [7, 10, 11].

In parallel, infants were independently and continuously monitored clinically with a thoracic impedance respiratory monitor, a pulse oximeter and an electrocardiogram. The thoracic impedance apnea alarm threshold was set to 15 s.

III. Determination of POA Categories

A. Classification based on Clinical Judgment

The acquisition session was continuously attended by at least one of the investigators, who kept a paper record of apneas and the infant's behavior (e.g., sleeping, feeding, diaper change, etc.). At the end of the acquisition session, an investigator (KAB) classified each infant as either Apneic or Non-Apneic by clinically judging the investigators' paper record and annotations in the medical dossier.

B. Classification based on Pause Length Evidence

The rationale for the 15 s pause length clinical definition of POA is unclear; it is based on physicians' personal experience and subjective clinical judgment. Consequently, we developed an objective procedure to determine POA categories. The procedure used unsupervised learning to categorize patients based on the length of respiratory pauses. Thus, the RIP data for each infant were analyzed with AUREA to identify all respiratory pauses. K-medoids [12], an unsupervised learning technique robust to outliers, was used to cluster subjects according to their maximum pause duration into 3 groups: short, medium and long. Infants in the short duration cluster were assigned to the NO-APNEA group. The long duration cluster only contained 3 infants, so it was combined with the medium duration group to form a single APNEA group. Fig. 1 shows the APNEA and NO-APNEA groups separated by the pause duration threshold of 14.6 s indicated by the vertical line.

C. Clinical Judgment vs. Evidence-Based Classification

The results of the K-medoids clustering classification were compared to those based on clinical judgment. It is noteworthy that the evidence-based threshold (14.6 s) corresponded closely to the 15 s pause duration used clinically to define POA [1].

However, Fig. 1 shows that there was substantial disagreement; thus 6 out of 15 (40%) infants who had pauses longer than 14.6 s (i.e., the evidence-based APNEA group) were clinically judged to be Non-Apneic (black-triangle), and conversely four out of 9 (44%) infants with pauses shorter than 14.6 s (i.e., evidence-based NO-APNEA group) were considered Apneic by clinical judgment (red-circle).

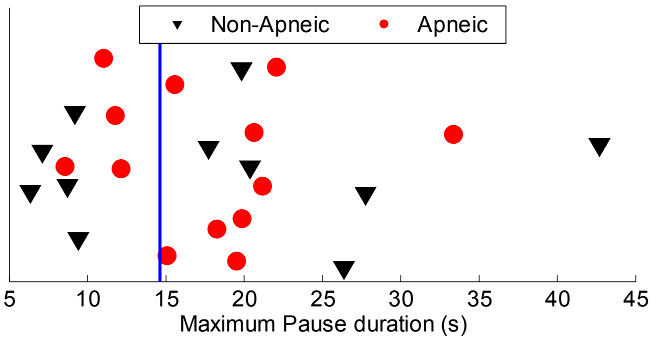

Fig. 1. Classification of subjects according to the occurrence of postoperative apnea (POA). The blue line indicates the threshold obtained with the evidence-based automated analysis; NO-APNEA infants are located on the left side and APNEA infants on the right side. The symbols show the classification based on clinical judgment (Non-Apneic: black triangles, Apneic: red circles). The y-axis is a random value used for visualization.

For this work, we defined the POA categories based on objective measurements of pause length as described above. This because the procedure evaluated in detail the respiratory behavior during the PACU stay, and thus was more precise detecting respiratory pauses and estimating their duration, compared to a combination of nurses' notes based on bedside monitor alarms and acquisition session annotations.

IV. Prediction of POA Occurrence

A. Predictive Ability of Features (Univariate Analysis)

Having established an objective method to determine which infants experienced POA, we analyzed the ability of the demographic and anesthetic management variables listed in Table I to predict the APNEA classification resulting from our automated analysis. To this end we used the Wilcoxon rank sum test [13] for real-valued variables, and the Fisher's exact test [14] for categorical variables, to evaluate for statistically significant differences between POA groups.

B. Feature Selection (Multivariate Analysis)

To evaluate the predictive ability of combinations of variables form Table I, it was necessary to select the combination(s) that would optimize the prediction of POA occurrence using classifiers. To this end, we applied the feature selection strategy described next.

Features considered were the variables listed in Table I and the square of the real-valued variables. Features from this set were selected based on a Most Discriminative and Least Correlated (MDLC) criteria as follows:

(1) Create an empty list of selected features,

(2) Estimate the p-value of each feature,

(3) Add the feature with lowest p-value to the list,

(4) Discard all features with p-value > γ_{pval},

Authorized licensed use limited to: CSIRO Information Technology Services. Downloaded on September 09,2022 at 00:40:35 UTC from IEEE Xplore. Restrictions apply.

TABLE I. PREDICTIVE ABILITY OF DEMOGRAPHIC AND ANESTHETIC MANAGEMENT VARIABLES

Variable	*p*-value	NO-APNEA[a] (n = 9)	APNEA[a] (n = 15)
Demographics			
Weight (kg)	0.06	4.2 [1.4]	3.5 [1.0]
Gender (male %)	0.29	89	73
Gestational Age (weeks)	0.59	34 [6]	31 [7]
Postmenstrual Age (weeks)	0.74	42 [4]	42 [3]
Categorical			
Caudal block (yes %)	0.19	44	67
Rocuronium (yes %)	0.30	44	53
Drug dosage			
Atropine (intraoperative) (μg/kg)	0.01	13.7 [7.6]	24.4 [15.6]
Propofol (mg/kg)	0.04	3.2 [2.8]	4.3 [2.0]
IOME[b] (mg/kg)	0.07	0 [0.1]	0.1 [0.2]
Acetaminophen rectal (mg/kg)	0.13	14.2 [13.4]	20.0 [8.0]
Atropine (reversal) (μg/kg)	0.63	0 [18.9]	17.4 [21.3]

a. Median [interquartile range] or percentage for each group.

b. IOME = Intraoperative Morphine Equivalent.

(5) Estimate the correlation between the features in the list and the remaining input features, and discard all those with an absolute correlation > γ_{corr},

(6) Add the remaining feature with lowest *p*-value to the selection list,

(7) Repeat from (5) until no more features are available.

C. Discriminant Analysis Classifiers

The features selected were used to train and test classifiers based on Gaussian discriminant analysis (DA). These classifiers included the classical Linear and Quadratic DA (cLDA and cQDA respectively), which use the classic estimators of Gaussian parameters, and the robust Linear and Quadratic DA (rLDA and rQDA respectively), which use estimators of Gaussian parameters that are robust to outliers [15]. Classifiers were implemented using the library for robust analysis (LIBRA) toolbox [16].

D. Classifier Performance Evaluation

Classifiers were evaluated with a leave-one-out cross-validation approach. The data set was split into two disjoint subsets: (1) testing, with data from one infant; and (2) training, with data from the remaining infants. The training set was used to estimate the parameters of the DA classifiers, and then the resulting model was used to classify the testing set. This was repeated using a different test set for all subjects. Note that feature selection was performed separately for each iteration, and thus was independent of the testing set. The detection probability (P_D) was estimated as the proportion of subjects correctly identified as APNEA (i.e., they were part of the APNEA group); the probability of false alarm (P_{FA}) was the proportion of subjects incorrectly identified as APNEA (i.e., they were in the NO-APNEA group). The P_D and P_{FA} of each classifier were evaluated for the testing set and for each iteration of the training set.

Each pair of P_{FA} and P_D corresponds to a point on a Receiver Operating Characteristic (ROC) curve, which describes the classifier performance. A perfect classification corresponds to $P_{FA} = 0$ and $P_D = 1$, whereas the performance expected by chance is at the line $P_{FA} = P_D$. The relation

$$d = \frac{(P_D - P_{FA})}{\sqrt{2}} \Big/ \frac{1}{\sqrt{2}} = P_D - P_{FA}, \qquad (1)$$

defines the normalized distance of any point on the ROC curve from the chance line. A $d = 0$ corresponds to a P_D and P_{FA} combination that lies on the chance line, while $d = 1$ indicates perfect classification. A larger distance corresponds to a better combination of P_D and P_{FA}. We used (1) to evaluate overall performance and select the best classifier.

V. RESULTS

A. Anesthetic Management

The median duration of surgery/anesthesia was 90 min with an interquartile range of 32.5 min; differences between POA groups were not statistically significant. Drug regimens differed because anesthetic management was not standardized. At the induction of anesthesia, all infants received atropine, and 21 received propofol. Another infant received a dose of propofol at the end of surgery. One infant received a second dose of atropine at the time of extubation. The total atropine dose administered during anesthesia is defined as intraoperative atropine dose. The maintenance anesthetic agent was either sevoflurane (n = 14) or desflurane (n = 10); the choice of agent was not significantly different between groups. An opioid was administered to 16 infants (fentanyl = 10, sufentanil = 2, remifentanil = 4). To account for the use of different intraoperative opioids, their doses were converted to intraoperative morphine equivalents (IOME) [17] based on: 10 μg fentanyl = 1 mg IOME, 1 μg sufentanil = 1 mg IOME, and 1 μg remifentanil = 0 mg IOME. Acetaminophen was administered to 21 infants. Rocuronium was administered to 12 infants and the muscle relaxant was antagonized with neostigmine and atropine. This atropine dose was referred to as the reversal atropine dose.

B. Predictive Ability of Individual Variables

Table I shows the demographic and clinical variables for the two POA groups and their associated *p*-values. In the APNEA group, the weight was significantly lower ($p = 0.06$), and the intraoperative doses of atropine ($p = 0.01$), propofol ($p = 0.04$), and IOME ($p = 0.07$) were significantly larger.

C. Feature Selection

A *p*-value threshold of $\gamma_{pval} = 0.1$ and a correlation threshold of $\gamma_{corr} = 0.1$ were used for feature selection. Table II shows the feature sets selected and the proportion of cross-validation iterations where each set was selected. It is clear that the intraoperative atropine dose was the most important feature since it was selected in all iterations. In about 2/3 of the cases it was accompanied by the propofol dose squared.

Authorized licensed use limited to: CSIRO Information Technology Services. Downloaded on September 09,2022 at 00:40:35 UTC from IEEE Xplore. Restrictions apply.

TABLE II. SELECTED FEATURES

Feature Set	*Proportion of cross-validation iterations*
Atropine (intraoperative), propofol squared.	62.5 %
Atropine (intraoperative).	37.5 %

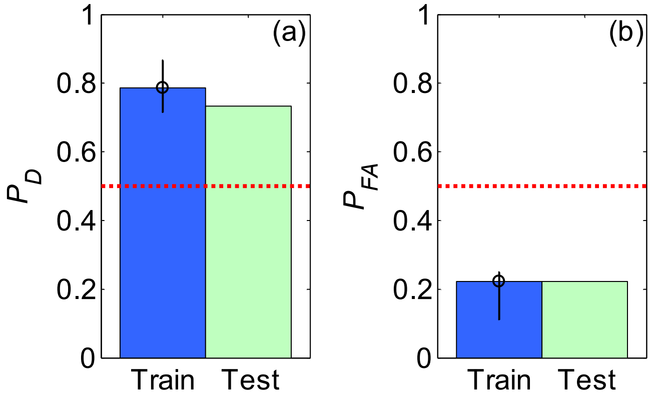

Fig. 2. (a) Probability of detection (P_D), and (b) probability of false alarm (P_{FA}) of the classic Linear Discriminant Analysis classifier when predicting infant POA occurrence. The red dotted lines show the performance expected by chance (e.g., a coin toss). The training results show the median and interquartile range for the training sets during cross-validation.

D. *Classification Performance*

The cLDA classifier had the best overall testing performance (d = 0.51). Fig. 2 shows its training and testing P_D and P_{FA} values, and compares them to those expected by chance. It is clear that the cLDA classification had both higher detection (i.e., identified more infants with POA), and a lower false alarm ratio (i.e., fewer infants without POA were classified as APNEA).

VI. DISCUSSION

This paper presents an objective, standardized approach to identify POA in infants recovering from surgery. Respiratory pauses were identified with AUREA, a comprehensive, automated system for the classification of respiratory behavior. We are very confident in AUREA's classification of pauses, since it agrees very well (Fleiss' kappa [18] > 0.8) with the "gold standard" manual analysis [19]. Furthermore AUREA has perfect repeatability, unlike manual scoring where both intra- and inter-scorer agreement are low [6]. We found that the pause duration threshold that distinguished between APNEA and NO-APNEA was 14.6 s, which is consistent with the 15 s pause duration defining POA [3]. Clinical classification had poor agreement with the automated, evidence-based classification; likely due to the subjective nature of annotation of apneic episodes based on bedside monitors [20] contrasted to the detailed, sample-by-sample, automated evaluation provided by AUREA. These results suggest that a comprehensive, standardized analysis of the respiratory behavior improves POA diagnosis.

We then evaluated the predictive ability of demographic and clinical variables. Four of them reached statistical significance. Thus, the intraoperative doses of atropine, propofol and opioids were higher for the APNEA group, and the weight was lower. Feature selection indicated that the intraoperative dose of atropine together with the propofol dose squared were most important for classification.

We trained DA classifiers to predict each infant's POA occurrence, and found that the cLDA had the best overall performance (d = 0.51), with a P_D = 0.73 and a relatively low P_{FA} = 0.22. These results are promising because they provide a standardized and accurate procedure to stratify infants at risk of POA that is independent of clinical judgment.

REFERENCES

[1] C. D. Kurth, A. R. Spitzer, A. M. Broennle, and J. J. Downes, "Postoperative Apnea in Preterm Infants," *Anesthesiology,* vol. 66, pp. 483-488, 1987.
[2] D. J. Steward, "Preterm Infants are More Prone to Complications Following Minor Surgery than are Term Infants," *Anesthesiology,* vol. 56, pp. 304-306, 1982.
[3] C. J. Cote, A. Zaslavsky, J. J. Downes, C. D. Kurth, L. G. Welborn, L. O. Warner, and S. V. Malviya, "Postoperative Apnea in Former Preterm Infants after Inguinal Herniorrhaphy: A Combined Analysis," *Anesthesiology,* vol. 82, pp. 809-822, 1995.
[4] C. Sims and C. M. Johnson, "Postoperative apnoea in infants," *Anaesth. Intens. Care,* vol. 22, pp. 40-45, 1994.
[5] Anonymous, *The AASM Manual for the Scoring of Sleep and Associated Events: Rules, Terminology and Technical Specifications*. Welchester, Illinois: American Academy of Sleep Medicine, 2007.
[6] N. A. Collop, "Scoring variability between polysomnography technologists in different sleep laboratories," *Sleep Med.,* vol. 3, pp. 43-47, 2002.
[7] C. A. Robles-Rubio, K. A. Brown, and R. E. Kearney, "Automated Unsupervised Respiratory Event Analysis," in *Conf. Proc. 33rd IEEE Eng. Med. Biol. Soc.*, Boston, USA, 2011, pp. 3201-3204.
[8] J. Macqueen, "Some methods for classification and analysis of multivariate observations," in *Proc. Fifth Berkeley Symp. on Math, Statistics, and Probability*, Berkeley, CA, USA, 1967, pp. 281-297.
[9] S. M. Semienchuk, A. L. Motto, H. L. Galiana, K. A. Brown, and R. E. Kearney, "A Portable, PC-Based Monitor for Automated, On-line Cardiorespiratory State Classification," in *Conf. Proc. 27th IEEE Eng. Med. Biol. Soc.*, Shanghai, China, 2005, pp. 4420-4423.
[10] C. A. Robles-Rubio, K. A. Brown, and R. E. Kearney, "Detection of Breathing Segments in Respiratory Signals," in *Conf. Proc. 34th IEEE Eng. Med. Biol. Soc.*, San Diego, USA, 2012, pp. 6333-6336.
[11] C. A. Robles-Rubio, K. A. Brown, and R. E. Kearney, "A New Movement Artifact Detector for Photoplethysmographic Signals," in *Conf. Proc. 35th IEEE Eng. Med. Biol. Soc.*, Osaka, Japan, 2013, pp. 2295 - 2299.
[12] L. Kaufman and P. J. Rousseeuw, "Partitioning Around Medoids (Program PAM)," in *Finding Groups in Data*, ed: John Wiley & Sons, Inc., 2008, pp. 68-125.
[13] F. Wilcoxon, "Individual Comparisons by Ranking Methods," *Biometrics Bull.,* vol. 1, pp. 80-83, 1945.
[14] R. A. Fisher, "On the interpretation of χ2 from contingency tables, and the calculation of P," *J. R. Stat. Soc.,* vol. 85, pp. 87-94, 1922.
[15] M. Hubert and K. Van Driessen, "Fast and robust discriminant analysis," *Comput. Stat. Data An.,* vol. 45, pp. 301-320, 2004.
[16] S. Verboven and M. Hubert, "LIBRA: a MATLAB library for robust analysis," *Chemometr. Intell. Lab.,* vol. 75, pp. 127-136, 2005.
[17] M. Yaster, S. Kost-Byerly, and L. G. Maxwell, "Opioid agonists and antagonists," in *Pain in Infants, Children, and Adolescents*, 2 ed Philadelphia: Lippincott Williams & Wilkins, 2003, pp. 181-224.
[18] J. L. Fleiss, "Measuring nominal scale agreement among many raters," *Psychol. Bulletin,* vol. 76, pp. 378-382, 1971.
[19] C. A. Robles-Rubio, R. E. Kearney, and K. A. Brown, "Automated Classification of Pauses, Breathing and Movement Artifacts in Infant Respiratory Data," presented at the Soc. Anesth. Sleep Med. Annu. Conf., Washington DC, USA, 2012.
[20] B. D. Vergales, A. O. Paget-Brown, H. Lee, L. E. Guin, T. J. Smoot, C. G. Rusin, M. T. Clark, J. B. Delos, K. D. Fairchild, D. E. Lake, R. Moorman, and J. Kattwinkel, "Accurate Automated Apnea Analysis in Preterm Infants," *Amer. J. Perinatol.,* vol. 31, pp. 157-162, 2014.

Authorized licensed use limited to: CSIRO Information Technology Services. Downloaded on September 09,2022 at 00:40:35 UTC from IEEE Xplore. Restrictions apply.
