# Supplementary material for: Exploring Computational Techniques in Preprocessing Neonatal Physiological Signals for Detecting Adverse Outcomes: Scoping Review
Source: Interact J Med Res. 2024 Aug 20;13:e46946. doi: 10.2196/46946 (PMC11372324; doi:10.2196/46946)
Supplement: Multimedia Appendix 3 [file ijmr_v13i1e46946_app3.zip › Included Papers - Final/3147/Doyen et al. - 2021 - Early bradycardia detection and therapeutic interv.pdf]

scientific reports

OPEN

Early bradycardia detection and therapeutic interventions in preterm infant monitoring

Matthieu Doyen[1,7], Alfredo I. Hernández[1,7]✉, Cyril Flamant[2], Antoine Defontaine[6], Géraldine Favrais[3], Miguel Altuve[4], Bruno Laviolle[5], Alain Beuchée[1], Guy Carrault[1] & Patrick Pladys[1]

In very preterm infants, cardio-respiratory events and associated hypoxemia occurring during early postnatal life have been associated with risks of retinopathy, growth alteration and neurodevelopment impairment. These events are commonly detected by continuous cardio-respiratory monitoring in neonatal intensive care units (NICU), through the associated bradycardia. NICU nurse interventions are mainly triggered by these alarms. In this work, we acquired data from 52 preterm infants during NICU monitoring, in order to propose an early bradycardia detector which is based on a decentralized fusion of three detectors. The main objective is to improve automatic detection under real-life conditions without altering performance with respect to that of a monitor commonly used in NICU. We used heart rate lower than 80 bpm during at least 10 sec to define bradycardia. With this definition we observed a high rate of false alarms (64%) in real-life and that 29% of the relevant alarms were not followed by manual interventions. Concerning the proposed detection method, when compared to current monitors, it provided a significant decrease of the detection delay of 2.9 seconds, without alteration of the sensitivity (97.6% vs 95.2%) and false alarm rate (63.7% vs 64.1%). We expect that such an early detection will improve the response of the newborn to the intervention and allow for the development of new automatic therapeutic strategies which could complement manual intervention and decrease the sepsis risk.

Respiration is unstable in the newborn, especially in the preterm infant. In these infants, apnea is a frequent phenomenon, which can be isolated, associated with hypoxemia or with a combination of hypoxemia and bradycardia. The current clinical definition of apnea or cardio-respiratory events in this context is not very precise and remains difficult to use in practice. An apneic spell is defined by the American academy of pediatrics as a cessation of breathing for 20 seconds or longer or a shorter pause accompanied by bradycardia (< 100 beats per minute), cyanosis, or pallor[1]. Those latter combined events may compromise brain oxygenation and tissue perfusion, especially when hypoxemia is followed by bradycardia[2]. The long-term impact of this intermittent hypoxemia during early postnatal life remains to be determined, but cardiorespiratory events have been evoked to potentially impact the risks of retinopathy, growth alteration, sleep-disordered breathing, and neurodevelopmental impairment[3–5].

In Neonatal Intensive Care Units (NICU), preterm infants undergo continuous monitoring through polygraphic recordings, to detect cardiorespiratory events and to initiate quick nursing actions, such as manual stimulation, mask ventilation and sometimes intubation. In those clinical settings, nurse interventions and treatment decisions are mostly based on oxygen saturation (SaO2) and heart rate alarms[6]. The detection of apneas through analysis of chest impedance waveform remains unreliable with reported false alarm rates of more than 60% and lack of detection in more than 25% of apneas lasting more than 10 s[7,8]. The pulse oximeters also exhibit around 20% lack of detection and more than 50% false alarm rates[9]. Bradycardia has been associated with apneas and desaturations in 83% and 86% of the recorded events with a mean delay from the onset of apnea, which is around five seconds[10]. The detection of bradycardias based on simple thresholding techniques

[1]Univ Rennes, CHU Rennes, Inserm, LTSI - UMR 1099, 35000 Rennes, France. [2]Univ-Nantes, CHU Nantes, Inserm, CIC 0004, F-44000 Nantes, France. [3]Univ-Tours, CHU Tours, Inserm, Imagerie et Cerveau UMR930, F-37000 Tours, France. [4]Faculty of Electrical and Electronic Engineering, Pontifical Bolivarian University, Bucaramanga, Colombia. [5]Univ-Rennes, CHU Rennes, Inserm, CIC 1414, F-35000 Rennes, France. [6]Polyclinic Quimper, Dpt Thoracic Surgery, Campus de Beaulieu, Bat 22, F-29000 Quimper, France. [7]These authors contributed equally: Matthieu Doyen and Alfredo I. Hernández. ✉email: alfredo.hernandez@inserm.fr

**Mother**	
Mean [SD] or n (%)	
Age (years)	31.1 [6.7]
Antenatal corticosteroids	48 (92)
Cesarean delivery	41 (79)
**Newborn (n = 52)**	
Mean [SD], median [IQR] or n (%)	
Sex (male/female)	26/26
Gestational age (weeks)	27.7 [1.7]
Birth weight (g)	1078 [329]
Inborn	52 (100)
Singleton	31 (60)
5-min Apgar score	9 [9-10]
Post-natal age (days)	10 [7-14]
**Characteristics at inclusion**	
Respiratory rate > 60 rpm	29 (56)
Abnormal temperature (> 38 °C or <36.5 °C)	5 (10)
Abdominal distension	31 (60)
Hemodynamically significant PDA	7 (13)
Anemia (< 10g/dl)	2 (4)
Abnormal glycemia (< 2.2 or > 8 mmol/l)	1 (2)
Hypokaliemia (< 3.5mmol/l)	4 (8)
Hypo or hyper natremia (< 135 or > 145 mmol/l)	2 (4)
High C-reactive protein (> 5mg/dl)	10 (19)
Sepsis suspicion	16 (31)
Positive hemoculture	10 (19)
Abnormal TF echography	4 (8)
Any use of positive airway pressure	41 (79)
Caffeine	49 (94)
Morphine	3 (6)

Table 1. Characteristics of the population studied.

is more reliable than apnea or desaturation detection but suffers from low specificity and high detection delays from the onset of respiration cessation[6,8,11,12]. From these studies, it mainly appears that the exposure of nurses to non-actionable alarms evokes the phenomenon of alarm fatigue and may yield a longer treatment response time, that may detrimentally affect patient care and safety. Duration and amplitude of bradycardia depend at least in part on the time delay between the onset of the bradycardia and the intervention, which remains often long, around 30 to 60 s[8,13]. Recent works based on system identification approaches have been proposed for the early detection of bradycardia events from ECG signals[14,15]. In both of these works, data from 10 newborns have been processed off-line, in batch mode, following a phase of manual verification for the rejection of artifacts due to movement, disconnection, or erroneous peak detections. A series of papers from our team have been focused on the early detection of apnea bradycardia events, but based on original Markov model architectures[16,17]. These methods have been evaluated using data from 32 preterm newborns, again, using a batch, post-processing mode.

We hypothesize that a systemic view of the problem, integrating a better understanding of the nurse response to NICU alarms together with an improved, early detection of bradycardia episodes, may help decrease the intervention delay and, therefore, reduce the potential consequences of intermittent hypoxemia[18]. Therefore, in this paper, we describe the nurse responses to alarms with regard to the depth of the observed bradycardia and this characterization leads to a detailed annotated database acquired in real-life conditions. We then propose and analyze a new early bradycardia detector, functioning in real-time and on-line mode (as current NICU monitors do), adapted to preterm infants. The proposed detector is compared in terms of detection delay with respect to the cardiac alarms generated by a monitor commonly used in NICU. We design this new detector to be at least equivalent to the existing one in terms of sensitivity and false alarm rate, but minimizing the detection delay.

Results

The observational prospective multicenter clinical study was performed in three French NICU (Rennes, Nantes and Tours). During the study period, 8 h ECG recordings (two periods of 4 h) were obtained from 52 very preterm newborns with a mean gestational age of 27.7 (±1.7) weeks and a birth-weight of 1078 (±329) g. The characteristics of the population and the circumstances associated with cardio-respiratory events at inclusion are presented in Table 1. Cardio-respiratory events were considered as occurring without an associated explanatory factor for 15 preterm newborns (29%).

	False alarm rate (mean [SD], s)	Sensitivity (%)	Detection delay (sec)
**Detection of B_{10s}^{80bpm} (n=84)**			
D_{red}^{mon}	64.1	95.2	12.7 (4.5)
D_{red}^{fusion}	63.7	97.6	9.8 (3.5)*
**Detection of B_{5s}^{100bpm} (n=316)**			
D_{yellow}^{mon}	19.9	89.2	11.5 (3.2)
D_{yellow}^{fusion}	14.1	90.8	8.1 (2.4)*
**Detection of B^{clin} (n=216)**			
D_{red}^{mon}	16	85.7	12.5 (4.4)
D_{red}^{fusion}	16.8	87	9.8 (3.6)*
D_{yellow}^{mon}	40.2	97.2	11.1 (3.1)
D_{yellow}^{fusion}	36.8	97.2	7.9 (2.2)*

Table 2. Comparison of bradycardia detection performances. False alarm rate, sensitivity and detection delay are reported according to the gold-standard annotations. Red alarm and Yellow alarm are respectively denoted D_{red}^{mon}, D_{yellow}^{mon} for the monitors currently used in neonatal units. The proposed fusion method is denoted D_{red}^{fusion}, D_{yellow}^{fusion} for Red and Yellow alarms respectively. *Significant difference ($p < 0.05$) between monitor and fusion delays using the Wilcoxon signed-rank test.

Alarms in the neonatal unit. During the period of observation, 356 monitor alarms (mean = 1 alarm/70 min/patient) were generated by the monitors in the neonatal units, including 225 red alarms. Among these 356 observed monitor alarms, 198 (56%) were manually recorded on nursing sheets during the acquisition period (intra-recording phase) by the attendant nurse. Among these 198 alarms, 77 (39%), were followed by a manual stimulation on the newborn, performed by the nurse. The stimulations were preceded by a rapid hand-wash in 40 cases (53% of the manual interventions).

Characterization of Bradycardia events. As described in the "Methods" section, a post-recording phase of alarm validation and curation was performed, in order to exhaustively classify the bradycardia events into three definitions, in line with recent literature: HR lower than 100 bpm during at least 5 s[1] (B_{5s}^{100bpm}), HR lower than 80 bpm during at least 10 s[5] (B_{10s}^{80bpm}), and "clinically-significant bradycardia" selected by the experts (B^{clin}).

During this curation phase, 316 B_{5s}^{100bpm} events were manually annotated. Among these events, 84 were further classified as B_{10s}^{80bpm}, and 216 as B^{clin} (see first column of Table 2). The other 40 alarms corresponded to artifacts, decrease in HR during less than 5 seconds and respiratory or SpO2 alarms.

Based on this event classification, we have studied the nurse responses during the intra-acquisition phase. Among the 198 alarms recorded on nursing sheets by the attendant nurse, we identified 70 B_{10s}^{80bpm} events. All 77 events that motivated manual stimulation were associated with a B^{clin} annotation. We observed 59 stimulations for B_{10s}^{80bpm} (71% of the total of B_{10s}^{80bpm} annotations) and 18 stimulations for less severe situations. It is to note that none of the preterm infants were orally fed during the recording periods. We did not observe any B_{10s}^{80bpm} induced by external interventions during the whole study period.

Figure 1 shows the main observed profiles, with monophasic bradycardia examples corresponding to B_{5s}^{100bpm} and B_{10s}^{80bpm} definitions in Fig. 1a, b respectively. Typical examples of triphasic (Fig. 1c) and biphasic (Fig. 1d) bradycardia profiles, observed in 34% of the events, are also depicted. The onset and offset instants of each bradycardia were manually annotated and then automatically corrected, as described in the "Methods" section, in order to minimize annotation jitter. The mean shift between manually-annotated onset of bradycardia annotations and the automatically-adjusted time-instant was − 1.94 s (i.e adjusted instants appear earlier than clinician annotations) with a standard deviation of 1.39 s. The mean shift between the offset bradycardia annotation time of the clinician and the adjusted time-instant was 0.11 s with a standard deviation of 1.32 s.

Automatic bradycardia detection performance. Table 2 shows the performance of the proposed bradycardia detection method. The sensitivity and false alarm rate of the proposed detector highly depend on the bradycardia definition used (i.e. B_{5s}^{100bpm}, B_{10s}^{80bpm} or B^{clin}). For the primary endpoint (B_{10s}^{80bpm}), results from the application of the proposed detection fusion approach D_{red}^{fusion} showed a significant decrease of the mean detection delay of 2.9 seconds with respect to the detection performed by the monitor D_{red}^{mon}. This early detection was obtained without altering sensitivity and without increasing the false alarm rate. When compared with the monitor detector, the proposed approach yielded also significantly decreased detection delays for B_{5s}^{100bpm} or B^{clin}, while maintaining comparable performance levels (Table 2). Regarding the fusion step, for D_{yellow}^{fusion}, 40% of the detections were generated by a simultaneaous activation of the 3 methods (fixed-threshold, relative adaptative threshold and abrupt change), 30% by the pair fixed/adaptative, 15% by the pair fixed/abrupt and 14% by the pair adaptative/abrupt. For D_{red}^{fusion}, these values are 69%, 3%, 17% and 11% respectively.

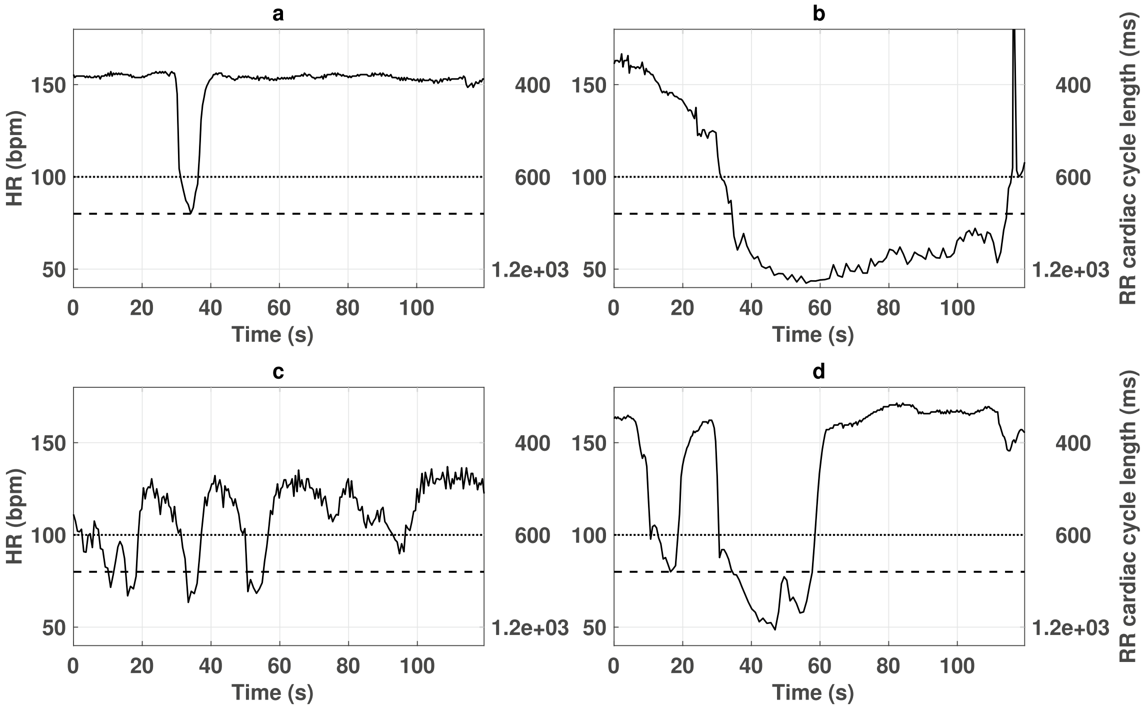

Figure 1. Example profiles of the observed bradycardia events. On each panel, solid curves represent the observed heart rate. Horizontal dotted and dashed lines represent respectively the yellow alarm threshold (100 bpm) and the red alarm threshold (80 bpm). Panel (**a**) shows a bradycardia of less than 100 bpm during at least 5 s (B_{5s}^{100bpm}). Panel (**b**) represents a severe bradycardia of less than 80 bpm during at least 10 s (B_{10s}^{80bpm}). Panel (**c**,**d**) show triphasic and biphasic bradycardias annotated as single B_{10s}^{80bpm} events.

Discussion

In this multicenter observational study performed in very preterm infants we described the nurse response to bedside monitor alarms and we proposed and evaluated a new real-time fusion-based method for the early detection of bradycardia events. Concerning the nurse response to bedside monitor alarms, in this study we observed that: i) A high percentage (29%) of severe bradycardia events with heart rate less than 80 bpm for more than 10 seconds did not result in nurse intervention, ii) the nurse intervention was not preceded by hand-wash before intervening on the newborn in around one half of the situations, iii) there is a high level of false alarm rate for the detection of severe cardio-respiratory events, which is known to favor alarm desensitization.

For all bradycardia definitions (B_{10s}^{80bpm}, B_{5s}^{100bpm}, B^{clin}) and settings (red, yellow), the proposed new fusion-based detector was able to provide an earlier event detection (between 2.7 s earlier than the monitor for B^{clin} on red alarms and 3.4 s earlier than the monitor for B_{5s}^{100bpm} on yellow alarms) without impairing the reliability of the detection, when compared to standard methods currently available on NICU monitors. Taken together, these results suggest that early bradycardia detection is possible without increasing the rate of false alarms, potentially allowing improvement in the management of cardio-respiratory events of the newborn.

Heart rate alarms are currently the most reliable way to detect the cardio-respiratory events associated with prematurity. The choice of the fusion method has the advantage to be easy to implement and embeddable into devices with low computational resources, when compared to other more sophisticated methods[16,17,19–21]. Portet et al also observed a small improvement in the time delay for detection of bradycardia using a decision tree approach[20]. Our group has proposed a set of hidden and coupled semi-Markovian methods that also provide earlier detection delays with increased performance levels[16,17,21]. However, none of these works have evaluated performance sensitivity with respect to the different definitions of bradycardia and under real-life conditions. We have shown here that this performance is particularly sensitive to the bradycardia definition used. This is an important aspect to consider, because the definition of a significant bradycardia remains conflicting with regards to its duration and deepness, due to the absence of established relationships between neonatal bradycardia deepness and duration and later neurodevelopment in very preterm infants[5].

Monitoring of vital signs, and more specifically monitoring of heart rate in neonatal intensive care units, is intended to obtain actionable alarms and rapid responses to clinically-relevant changes in the newborn's conditions. Despite this, as other authors[6,8,11,12], we observed that many relevant alarms were not followed by rapid interventions by the attendant nurses. The probability to respond in time to an alarm not only depends on the duration of the bradycardia, but also on its interpretation by the nurse, which is known to be influenced by many factors. This could also be due to alarm fatigue[6,7,22] or to the fact that alarms are not always directly exploited, but rather used as an additional information for decision support. It seems from the literature that the consequences of cardio-respiratory events in preterm infants could be mainly linked to incidence, duration and severity of

hypoxemic episodes[5]. In this study we have chosen to focus on bradycardia rather than on hypoxemia because of the high false alarm rate associated with pluse oymetry[9]. In the current study we observed nurses intervention in only 36% of the bradycardias associated with a desaturation of at least 10% (B^{clin}) and in only 71% of the events with a bradycardia of less than 80 bpm with duration of more than 10 sec which were always associated with oxygen desaturation. It appears therefore that improving detection and prevention of bradycardia events in preterm infants will result in a decrease of hypoxemic episodes and potentially on the associated morbidity. Moreover, we also observed that hand wash is not always performed in such a context of an emergency intervention. As hand hygiene has a well-known effect on infection reduction, this could have increased the risk of transmission of infectious agents[23]. It seems therefore necessary to test new strategies to limit this risk.

In this study, we have chosen to focus on heart rate alarms as a first step to improve alarm setting for an early detection of cardio-respiratory events. With such an approach, we expect to early detect 85% of the cardio-respiratory events including all the severe episodes[10,24] without increasing the false detection rate. We expect that such an early detection will improve the response of the newborn to the intervention. We also expect that this will allow for the development of new therapeutic strategies, such as automatic intervention systems which could also decrease the sepsis risk associated with manual intervention, as suggested in[18].

One limitation of this work is the lack of combination of the different modes of monitoring (i.e. respiratory rate and SpO2), which would have theoretically been a better way to approach oxygen delivery and therefore to quantify the risk of intermittent hypoxia events. However, the benefit of such a combined approach has not been demonstrated yet in clinical practice for several reasons. Usually, in the presence of artifacts, all the signals are corrupted together[25]. Also, SpO2, as well as chest impedance, are known to be insufficiently reliable[7–9]. Another limitation of heart rate alarms is that it is known that bradycardia, in the absence of concurrent prolonged hypoxemia of more than one minute, may not be of prognostic importance[5]. However, it is known that it can be difficult to reverse the bradycardia when the event is prolonged. Therefore, we think that it is clinically relevant to early detect and intervene on most of the bradycardias, with the expectation to improve neonatal outcome through a decrease in intermittent hypoxemia events. The price to pay for this early detection, using our approach as well as monitor alarms, is that only around one third of the red alarms correspond to severe bradycardias. We think that the proposed approach could be incorporated into new strategies using multi-signal analyses and/or closed-loop, automated stimulation, in order to limit alarm fatigue for healthcare givers and minimize the consequences of repeated cardio-respiratory events for very preterm newborns.

Methods

Clinical study. This observational prospective multicenter study was designed to validate a new method for early detection of severe bradycardia and was performed in the French NICU from Tours, Nantes and Rennes university hospitals. The study was approved by the committee on protection of individuals (Comité de Protection des Personnes Ouest II, 03/05-445) and registered on clinical trials registry (2008-A00898-47, NCT00950287). Informed parental consent was obtained and all methods were carried out in accordance with relevant guidelines and regulations. The responses to the alarms in real-life conditions were observed during two 4-h periods (one following the inclusion and the second one two to seven days later). Eligible infants were preterm born before 33 weeks of gestational age with a post-menstrual age of less than 36 weeks, presenting with at least 2 significant apnea-bradycardia in a 3-hour period. The definition used for a significant apnea-bradycardia at inclusion was a decrease in heart rate of more than 33% with respect to HR baseline during at least 10 s, associated with and oxygen saturation SpO2 less than 80% for at least 4 s[10]. The exclusion criteria were: a postnatal age of less than four days, mechanical ventilation, intracerebral lesion (grade 3 or 4 intracranial hemorrhage, periventricular leukomalacia or ischemic lesion), and treatment with doxapram. Caffeine treatment or nasal continuous positive pressure ventilation were not exclusion criteria.

Data acquisition and intra-recording annotations. Each newborn was recorded twice over 4-hour periods with real-time annotation of the records by a specific research nurse who continuously observed the newborn without intervening in care. Annotations included: characteristics of the cardio-respiratory events, kind of intervention of the nurse in charge of the patient, hand washing before intervention and recording of the bradycardia event by the attendant nurse on the health record. The two recording periods were chosen to get data with different rates of alarms. The first period began as soon as possible after inclusion. The second period was acquired at least 24 h after the first one. The recordings were performed in neonatal units in a real-life situation, with the preterm infant remaining in usual condition with limitations of external stimulations during the study periods.

In order to obtain all raw ECG signals, as well as synchronized annotations, two parallel monitoring systems were applied to each newborn: the standard NICU monitor and a custom-made research ECG monitoring system (INTEM system). The INTEM system integrates (i) an original equipment manufacturer (OEM) CE-marked ECG acquisition board (MCC GmbH & Co. KG, Karlsruhe, Germany) able to acquire 3 ECG leads and to perform analog-to-digital conversion at a sampling rate of 300 Hz; (ii) a Bluetooth communication board for wireless transfer from the acquisition module to a standard computer; (iii) a custom electronic board to control these modules by a specific microcontroller and associated firmware and iv) a computer application with specific GUI for the acquisition, display and storage of the raw signals and to allow the research nurse to annotate the observed events during acquisition. Also, the NICU monitor alarms were recorded by the INTEM system by means of a custom-made automatic detection device connected to the monitors alarm light-emitting diodes (LED). Both systems were connected to the newborn using the same electrodes and a Y-connector.

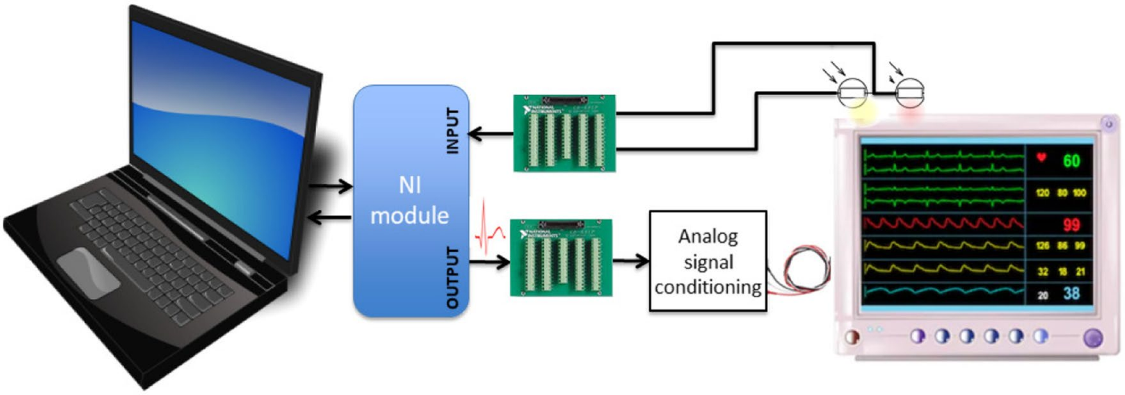

Figure 2. Proposed test-bench. Recorded ECG signals were converted from digital to analog, conditioned, scaled and presented as input to a Philips IntelliVue MP20 monitor. Alarms generated by the monitor were captured using a photoresistor sensor fixed on the alarm activity LED of the monitor.

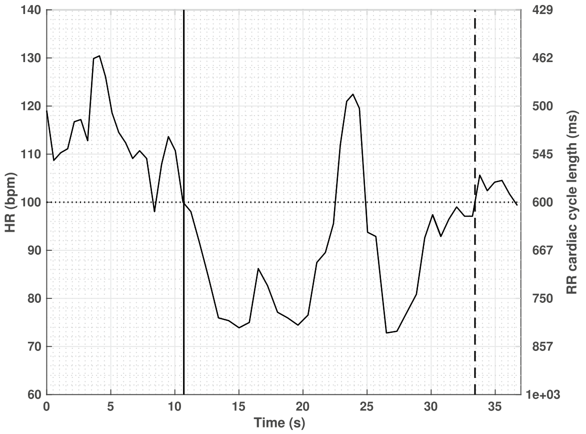

Figure 3. Representation of a biphasic bradycardia event. The solid curve represents the heart rate time series. Horizontal dotted line represents the 100 bpm threshold (Yellow alarm). The vertical solid line represents the beginning of the bradycardia and the vertical dashed line represents the end of the event, as annotated by clinicians. During this bradycardia episode, the heart rate was higher than 100 bpm during a short time period (less than 10 s). We consider this event as one single biphasic bradycardia.

Post-recording annotations and curation of the database. Even if the intra-recording annotations are very valuable, these annotations should be curated and further completed during a post-recording phase in order to cope with four main factors: (i) bradycardia events annotated by the research nurse or automatically recorded by the system can be associated with a false alarm, (ii) miss-detections can occur and the corresponding events are thus not annotated during the intra-recording phase, (iii) LED alarms can be of many types and should be manually curated and, especially, (iv) NICU monitors from each center may be of different models, have different firmware versions or may be configured with different parameters, leading to LED alarms that may not all correspond to the same detection criteria.

In order to cope with these limitations, we decided to develop a test-bench so as to obtain a set of homogeneous HR monitor alarm annotations (Fig. 2). The recorded INTEM ECG signals were converted from digital to analog, conditioned, scaled, and presented as input to a single Philips IntelliVue MP20 monitor (software version F.01.43) with the default settings for NICU monitoring (red alarm set at 80 bpm and yellow alarm at 100 bpm). Alarms generated by the monitor were acquired using a photoresistor sensor fixed on the alarm activity LED, as performed during the intra-recording phase (see Fig. 2). Two National Instruments modules (PCI-6220 for analog input and PCI-6733 for analog output) were linked to an I/O Connector Block to the Philips monitor (CB-68LP) and a computer was used to present all recordings at the appropriate rate.

Once all records have been ran through this test-bench and once all ECG signals were processed for the estimation of heart rate series, a curation and further annotation phase was initiated. Firstly, all bradycardia events were manually annotated by two expert clinicians, by analyzing the original intra-recording annotations, the acquired signals and the derived heart rate series. A given bradycardia was considered as a unique biphasic or multi-phasic event when the interval between two heart rate reductions below 100 bpm lasted less than 10 seconds (Fig. 3).

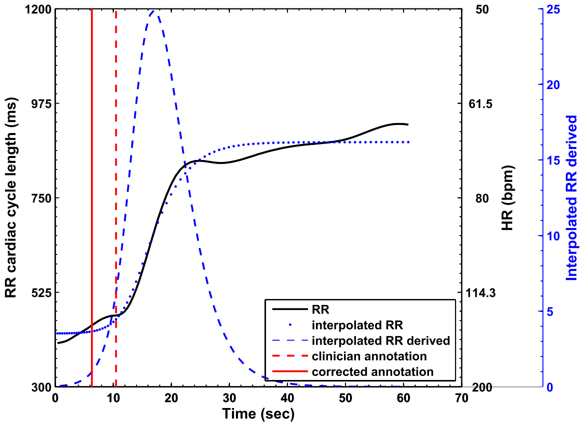

Figure 4. Automatic annotation onset adjustment process. RR time series is represented by the black line. The sigmoid function interpolating the observed RR series is represented by the dotted blue line and its first derivative by the dashed blue line. The vertical red lines represent the onset of the bradycardia proposed by the clinician (dashed line) and the corrected onset (solid line), using the first point where the derivative of the sigmoid adjustment function is greater than 1. In this example, the corrected annotation is located 4.16 s before the one performed by the clinician.

Three definitions of bradycardia events have been used:

- HR lower than 100 bpm during at least 5 s (B_{5s}^{100bpm}): It was chosen to be in line with a recent definition of bradycardia[1] and because it was close to the yellow HR alarms generated by the monitor.
- HR lower than 80 bpm during at least 10 s (B_{10s}^{80bpm}): This globally accepted definition of severe bradycardia was chosen to be in line with a recent publication regarding the outcome after episodes of bradycardia with heart rate below 80 bpm for 10 seconds or longer[5].
- Clinically-significant bradycardia (B^{clin}): These bradycardia were chosen by the experts, taking into account the ECG waveform, SpO2 and real-time clinical annotations of apneas performed by the research nurse during the intra-recording phase. They were considered clinically significant in case of: B_{10s}^{80bpm} or B_{5s}^{100bpm}, associated with apnea and a decrease in SpO2 of at least 10%. Isolated B_{5s}^{100bpm}, which were not associated with a decrease in SpO2 were not considered as clinically significant. Similarly, isolated B_{5s}^{100bpm} observed in case of low basal HR or due to HR oscillations associated with periodic breathing were not considered as clinically significant.

Please note that events classified in both B_{10s}^{80bpm} and B^{clin} are subsets of the events classified as B_{5s}^{100bpm}. For each bradycardia annotation, an automated optimization process initially proposed in[26] was applied in order to obtain precise and reproducible measurements of the instant of bradycardia onset and duration. This optimization method is based on the adjustment of a sigmoid function: the adjusted event corresponds to the first point where the derivative of the interpolated sigmoid function is greater than 1 (see Fig. 4).

In a second step, each annotated bradycardia was compared with the recorded LED alarms from the monitors. If the monitor alarm was caused by another pathophysiological phenomenon, such as tachycardia or by a false positive, the alarm was excluded from the analysis.

Signal processing. ECG processing. Prior to the detection of bradycardia events, the RR series, representing the time intervals between consecutive heart beats (QRS complex) were built using a multi-feature probabilistic real-time detector[27]. Briefly, this detector first derives relevant features (slope, amplitude and correlation on a window length of 50 ms) from the analysis of a single-lead ECG signal and from the estimates of the feature probability distributions. Bayesian probabilities were then computed for each feature before being merged through an adaptive centralized decision node using the Kullback-Leibler divergence measure. Probability distributions were then updated according to the final detection. Let $t(k)$ be the time instant of the $k-th$ detected beat, then the RR series was classically built by computing the difference between two successive QRS complexes $RR(k) = t(k+1) - t(k)$.

Bradycardia detection algorithm. The detection fusion approach used in this paper was initially proposed by our team, in order to perform an early detection of bradycardias without increasing the rate of false alarms[28]. It is based on an optimal decentralized fusion of three detectors: a standard fixed-threshold, an adaptive-threshold and an abrupt change detection method. These three methods are based on an analysis of beat-to-beat intervals (RR intervals) taking into account not only the instantaneous values of the RR series, but also their temporal evolution.

The fixed-threshold method compares each beat-to-beat RR interval with a fixed threshold U_0 (set at 600 ms when B_{5s}^{100bpm} bradycardia are targeted and at 750 ms for B_{10s}^{80bpm}). If successive RR intervals were higher than U_0 during more than 4 s, and if during this 4 s period, 2 RR intervals were higher than a second threshold U_1 (set at 640 ms for B_{5s}^{100bpm} bradycardia and at 800 ms for B_{10s}^{80bpm}), a bradycardia was detected. Thresholds U_0, U_1 and period durations were set in order to minimize detection delay while maximizing detection performance. In summary, a bradycardia detection occurs when the following conditions are satisfied:

$$RR(k) > U_0 \text{ during } \Delta_t > 4\,s \text{ with } 2\ RR(k) \text{ occurrences } > U_1.$$

The relative adaptive threshold method considers the fact that the basal heart rate of a patient can change through time. Thus, the threshold U_0 becomes adaptive and is denoted $U_0'(k) = 1.33 \cdot RR_{mean}(k)$, where $RR_{mean}(k)$ is the mean RR value calculated over all RR values preceding beat k during the last 20 s. As for the precedent method, a second threshold U_1' (set at 640 ms when B_{5s}^{100bpm} bradycardia are targeted and at 800 ms for B_{10s}^{80bpm}) was used. Detection of a bradycardia event occurs when the following conditions are satisfied:

$$RR(k) > U_0^{'}(k) \text{ during } \Delta_t > 4\,s \text{ with } 2\ RR(k) \text{ occurrences } > U_1^{'}.$$

The abrupt change detection method was based on the Cumulative Sum algorithm[28,29]. It models the bradycardia event as a statistically significant change in the mean of the RR series. In this approach, the RR interval is considered as a constant piecewise function perturbed by a noise of known variance. Two composite hypotheses are tested at each k:

$$H_0 : RR(k) = \overline{RR_0}(k) + e(k) \text{ for } 0 \le k \le n \tag{1}$$

$$\begin{aligned} H_1 : RR(k) &= \overline{RR_0}(k) + e(k) \text{ for } 0 \le k \le r-1 \\ RR(k) &= \overline{RR_0}(k) + \nu + e(k) \text{ for r } \le k \le n \end{aligned} \tag{2}$$

where $\overline{RR_0}(k)$ is the mean value of $RR(k)$ before the bradycardia event and ν represents the value of the significant mean change i.e. the cardiac cycle length increase. In order to detect these events, the H_1 hypothesis must be accepted in front of hypothesis H_0. Classically, the decision between the two hypothesis is evaluated through the likelihood ratio between the composite hypotheses. As the time instant of the jump r and the magnitude of the jump ν are unknown, a generalized likelihood test is performed which leads to the famous Page Hinkley algorithm[29] which consists in:

1. computing the cumulative sum $\Lambda_n(t) = \sum_{k=t}^{n} \left(RR(k) - \overline{RR_0}(k) - \frac{\nu}{2}\right)$;
2. recording the current minimum value $m_n(t) = \min_{1 \le k \le n} \Lambda_n(t)$;
3. setting an alarm at a time instant t when : $\Lambda_n(t) - m_n(t) \ge \lambda$.

In this study, the window length of $\overline{RR_0}$ was set to 290 s, the threshold $\lambda = 717$ and $\nu = 415$.

The final step consists in applying a fusion rule to the output of the above-mentioned detectors, in order to obtain the final decision. In this work, we adopted a voting scheme, similar to the ones used in recent ensemble machine learning methods[30,31]. Let $n = 3$, be the number of local detectors and d_i ($i \in 1, .., n$) the individual decision obtained from each detector ($d_i = 1$ if a detection occurs, $d_i = 0$ otherwise), a bradycardia detection occurs when:

$$\sum_{i=1}^{n} d_i > \frac{n}{2} \tag{3}$$

The bradycardia detector described above is characterized by a number of parameters that have to be correctly defined (window sizes, thresholds,...). In order to optimize these parameters, we used data from a previous different study by Beuchée et al, which studied the changes in HR variability associated with neonatal late onset sepsis (local ethics committee 03/05-445)[32]. Briefly, this database was composed of 148 1-hour ECG recordings acquired from 32 very preterm infants, included after parental consent. In this database, 116 bradycardia events with HR less than 100 bpm during more than 5 seconds were identified and validated by two expert neonatologists. The parameter optimization was performed using an evolutionary algorithm, as described in[33]. Once this optimization process was concluded, all the parameters were fixed, in order to apply the method prospectively on the data acquired for this study.

Performance evaluation and statistical analysis. Bradycardia detection performance was evaluated in terms of true positive (TP), false negative (FN), false positive (FP), sensitivity (TP/(TP+FN)), false alarm rate (FP/(FP+TP)) and detection delay. The following rules were applied to define the status of each detection:

- Detections located 5 s before or within the 30 s following the annotation of the onset of a bradycardia event were considered as TP, all other cases were considered as FP;
- If two detections were included in the window [-5:+30 s], corresponding to the same annotation, only the first one was considered as TP and used to calculate the delay (the second one was ignored);

- If two annotations corresponded to the same detection, only one TP was considered, the second annotation was ignored.

The detection delay was computed as the time elapsed between the adjusted bradycardia onset annotation time and the detection instant. Delays induced by the dedicated electronics and real-time QRS detection (1512 ms) were taken into account for bradycardia detection algorithm.

Three different gold-standard annotations, corresponding to the three bradycardia definitions given above (B_{10s}^{80bpm}, B_{10s}^{100bpm}, B^{clin}), were used in this study and compared with the output of the proposed detector, as well as the output of the monitor alarms. In order to compare the performance of the proposed detector versus the monitor alarms, the following notation was used:

- Annotations B_{10s}^{80bpm} were compared to the output of red alarms of the monitors, denoted D_{red}^{mon}, and with the output of proposed detector, denoted here D_{red}^{fusion}, with parameters optimized for B_{10s}^{80bpm} events.
- Annotations B_{5s}^{100bpm} were compared to the yellow alarm output of the monitors denoted here $D_{yelllow}^{mon}$ and with the proposed detector, denoted here D_{yellow}^{fusion}, with parameters optimized for B_{5s}^{100bpm} events.
- B^{clin} annotations are not related to a particular alarm category (red or yellow). Therefore, they were compared to both D_{red}^{type} and D_{yellow}^{type} where *type* stands for the monitor alarms (*mon*) or the proposed fusion method (*fusion*).

Global population results are reported as mean ± standard deviation. Statistical analysis was performed using the Wilcoxon signed-rank test due to the non-normality distribution of the data. For all tests, a significance level of 0.05 was used. No exclusion of outliers was performed prior statistical analysis. The sensitivity and false alarm rate standard deviation values were not reported because they were calulated on the whole database.

Data availability

The datasets analyzed during the current study are available from the corresponding author on reasonable request.

Received: 7 November 2020; Accepted: 13 April 2021
Published online: 18 May 2021

References

1. Eichenwald, E. C. Apnea of prematurity. *Pediatrics* **137**, e20153757. https://doi.org/10.1542/peds.2015-3757 (2016).
2. Schmid, M., Hopfner, R., Lenhof, S., Hummler, H. & Fuchs, H. Cardiorespiratory events in preterm infants etiology and monitoring technologies. *Neonatology* **107**, 137–146 (2015).
3. Di Fiore, J., Poets, C., Gauda, E., Martin, R. & MacFarlane, P. Cerebral oxygenation during intermittent hypoxemia and bradycardia in preterm infants. *J. Perinatol.* **36**, 165–171 (2016).
4. Janvier, A. *et al.* Apnea is associated with neurodevelopmental impairment in very low birth weight infants. *J. Perinatol.* **24**, 763–768. https://doi.org/10.1038/sj.jp.7211182 (2004).
5. Poets, C. *et al.* Association between intermittent hypoxemia or bradycardia and late death or disability in extremely preterm infants. *JAMA* **314**, 595–603 (2015).
6. Brockmann, P. *et al.* Under-recognition of alarms in a neonatal intensive care unit. *Arch. Dis. Child Fetal Neonatal Ed.* **98**, 3524–527 (2013) (**201**).
7. Vergales, B. *et al.* Accurate automated apnea analysis in preterm infants. *Am. J. Perinatol.* **31**, 157–162 (2014).
8. Mohr, M. *et al.* Very long apnea events in preterm infants. *J. Appl. Physiol.* **118**, 558–568 (2015).
9. Nizami, S., Greenwood, K., Barrowman, N. & Harrold, J. Performance evaluation of new-generation pulse oximeters in the nicu, observational study. *Cardiovasc. Eng. Technol.* **6**, 383–391 (2015).
10. Poets, C., Stebbens, V., Samuels, M. & Southall, D. The relationship between bradycardia, apnea, and hypoxemia in preterm infants. *Pediatr. Res.* **34**, 144–147 (1993).
11. Ahlborn, V., Bohnhorst, B., Peter, C. & Poets, C. False alarms in very low birthweight infants, comparison between three intensive care monitoring systems. *Acta Paediatr.* **89**, 571–576 (2000).
12. Belal, S., Emmerson, A. & Beatty, P. Automatic detection of apnea of prematurity. *Physiol. Meas.* **32**, 523–542 (2011).
13. Pichardo, R., Adam, J., Rosow, E., Bronzino, J. & Eisenfeld, L. Vibrotactile stimulation system to treat apnea of prematurity. *Biomed. Instrum. Technol.* **37**, 34–40 (2003).
14. Gee, A., Barbieri, R., Paydarfar, D. & Indic, P. Predicting bradycardia in preterm infants using point process analysis of heart rate. *IEEE Trans. Biomed. Eng.* **64**, 2300–2308. https://doi.org/10.1109/TBME.2016.2632746 (2017).
15. Das, S., Moraffah, B., Banerjee, A., Sandeep, K. & Papandreou-Suppappola, A. Bradycardia prediction in preterm infants using nonparametric kernel density estimation. in *53rd Asilomar Conference on Signals, Systems, and Computers* 1309–1313. https://doi.org/10.1109/IEEECONF44664.2019.9049007 (2019).
16. Montazeri Ghahjaverestan, N. *et al.* Coupled hidden Markov model-based method for apnea bradycardia detection. *IEEE J. Biomed. Health Inform.* **20**, 527–38. https://doi.org/10.1109/JBHI.2015.2405075 (2016).
17. Ghahjaverestan, N. M., Shamsollahi, M. B., Ge, A., Beuchée, D. & Hernández, A. Apnea bradycardia detection based on new coupled hidden semi Markov model. *Med. Biol. Eng. Comput.* **590**, 1–11. https://doi.org/10.1007/s11517-020-02277-8 (2021).
18. Beuchée, A., Hernandez, A., Pladys, P. & Carrault, G. Stimulateur kinesthésique automatisé asservi à la détection d'apnées-bradycardies chez le nouveau-né prématuré. *IRBM* **28**, 124–130. https://doi.org/10.1016/j.rbmret.2007.09.003 (2007).
19. Quinn, J. A. & Williams, C. K. I. Known unknowns: Novelty detection in condition monitoring. In *Pattern Recognition and Image Analysis* (eds Martí, J. *et al.*) 1–6 (Springer, Berlin, Heidelberg, 2007).
20. Portet, F., Gao, F., Hunter, J. & Sripada, S. Evaluation of on-line bradycardia boundary detectors from neonatal clinical data. in *2007 29th Annual International Conference of the IEEE Engineering in Medicine and Biology Society* 3288–3291. https://doi.org/10.1109/IEMBS.2007.4353032 (2007).
21. Altuve, M., Carrault, G., Beuchee, A., Pladys, P. & Hernandez, A. I. Online apnea-bradycardia detection based on hidden semi-Markov models. *Med. Biol. Eng. Comput.* **53**, 1–13. https://doi.org/10.1007/s11517-014-1207-1 (2015).

22. Bonafide, C. *et al.* Association between exposure to nonactionable physiologic monitor alarms and response time in a children's hospital. *J. Hosp. Med.* **10**, 345–351 (2015).
23. Helder, O., Brug, J., Looman, C., van Goudoever, J. & Kornelisse, R. The impact of an education program on hand hygiene compliance and nosocomial infection incidence in an urban neonatal intensive care unit, an intervention study with before and after comparison. *Int. J. Nurs. Stud.* **47**, 1245–1252 (2010).
24. Poets, C. Apnea of prematurity, what can observational studies tell us about pathophysiology. *Sleep Med.* **11**, 701–707 (2010).
25. Joshi, R. *et al.* Pattern discovery in critical alarms originating from neonates under intensive care. *Physiological Measurement* **37**, 564–579. https://doi.org/10.1088/0967-3334/37/4/564 (2016).
26. Altuve, M. *Détection multivariée des épisodes d'apnée-bradycardie chez le prématuré par modéles semi-Markovien cachés*. Ph.D. thesis, Rennes 1 University (2011).
27. Doyen, M., Ge, D., Beuchée, A., Carrault, G. & Hernández, A. I. Robust, real-time generic detector based on a multi-feature probabilistic method. *PlOS ONE* **14**, 1–22. https://doi.org/10.1371/journal.pone.0223785 (2019).
28. Cruz, J., Hernandez, A. I., Wong, S., Carrault, G. & Beuchee, A. Algorithm fusion for the early detection of apnea-bradycardia in preterm infants. in *2006 Computers in Cardiology* 473–476 (2006).
29. Basseville, M. & Nikiforov, I. V. *Detection of Abrupt Changes: Theory and Application* (Prentice-Hall Inc, Upper Saddle River, 1993).
30. Gomes, H. M., Barddal, J. P., Enembreck, F. & Bifet, A. A survey on ensemble learning for data stream classification. *ACM Comput. Surv.* **50**, 23:1-23:36. https://doi.org/10.1145/3054925 (2017).
31. Raza, K. Chapter 8—Improving the prediction accuracy of heart disease with ensemble learning and majority voting rule. In *U-Healthcare Monitoring Systems, Advances in Ubiquitous Sensing Applications for Healthcare* (eds Dey, N. *et al.*) 179–196 (Academic Press, London, 2019). https://doi.org/10.1016/B978-0-12-815370-3.00008-6.
32. Beuchée, A. *et al.* Uncorrelated randomness of the heart rate is associated with sepsis in sick premature infants. *Neonatology* **96**, 109–114. https://doi.org/10.1159/000208792 (2009).
33. Dumont, J., Hernández, A. & Carrault, G. Improving ECG beats delineation with an evolutionary optimization process. *IEEE Trans. Biomed. Eng.* **57**, 607–615 (2010).

Acknowledgements
The authors thank all patients who agreed to participate to the study.

Author contributions
M.D., A.H., C.F., A.D., G.F., M.A., B.L., A.B., G.C., P.P contributed substantially to the study design, data collection, and/or data analysis and interpretation. M.D., A.H., G.C. and P.P. wrote the manuscript and C.F., A.D., G.F., M.A., A.B., G.C. critically revised the manuscript. All authors reviewed the manuscript. A.H. and P.P. had full access to all of the data in the study and take responsibility for the integrity of the data and the accuracy of the data analysis.

Funding
The study was funded by a research grant from the French National Clinical Research Programme (PHRC 0805).

Competing interests
The authors declare no competing interests.

Additional information
Correspondence and requests for materials should be addressed to A.I.H.

Reprints and permissions information is available at www.nature.com/reprints.

Publisher's note Springer Nature remains neutral with regard to jurisdictional claims in published maps and institutional affiliations.

Open Access This article is licensed under a Creative Commons Attribution 4.0 International License, which permits use, sharing, adaptation, distribution and reproduction in any medium or format, as long as you give appropriate credit to the original author(s) and the source, provide a link to the Creative Commons licence, and indicate if changes were made. The images or other third party material in this article are included in the article's Creative Commons licence, unless indicated otherwise in a credit line to the material. If material is not included in the article's Creative Commons licence and your intended use is not permitted by statutory regulation or exceeds the permitted use, you will need to obtain permission directly from the copyright holder. To view a copy of this licence, visit http://creativecommons.org/licenses/by/4.0/.

© The Author(s) 2021
