# Supplementary material for: Exploring Computational Techniques in Preprocessing Neonatal Physiological Signals for Detecting Adverse Outcomes: Scoping Review
Source: Interact J Med Res. 2024 Aug 20;13:e46946. doi: 10.2196/46946 (PMC11372324; doi:10.2196/46946)
Supplement: Multimedia Appendix 3 [file ijmr_v13i1e46946_app3.zip › Included Papers - Final/3165/Mirnia et al. - 2021 - Signal Processing of Heart Rate for Predicting Sep.pdf]

Short Communication

Signal Processing of Heart Rate for Predicting Sepsis in Premature Neonates

Abstract

The heart rate characteristic (HeRO score) is a figure derived from the analysis of premature neonate's electrocardiogram signals, and can be used to detect infection before the onset of clinical symptoms. The United States and Europe accept this diagnostic technique, but we require more tests to prove its efficacy. This method is not accepted in other developed countries so far. The present study aimed to investigate changes in the heart characteristics of two neonates in Akbar Abadi Hospital in Tehran. Experts chose one newborn as a sepsis case, and the other neonate was healthy. The results were analyzed and compared with previous studies. In this research, a group of five neonates was selected randomly from the neonatal intensive care unit, and cardiac leads were attached to them for recording heart rates. We selected two neonates from the five cases, as a case (proven sepsis) and control, to analyze heart rate variability (HRV). Then, we compared the differences in the heart rate of both neonates. Analysis of HRV of these two neonates showed that the pattern of HRV is compatible with reports from US studies. Considering the results of this study, heart rates and their analysis can provide useful indicators for mathematical modeling before the onset of clinical symptoms in newborns.

Keywords: *Heart rate, HeRO, neonates, sepsis, signal processing*

Submitted: 11-May-2020 **Revised:** 27-Sep-2020 **Accepted:** 04-Apr-2021 **Published:** 21-Jul-2021

Kayvan Mirnia[1], Mohammad Heidarzadeh[2], Seyyed Abolfazl Afjeh[3], Parinaz Alizadeh[4], Abbas Abaei Kashan[5], Arash Bordbar[6], Amid Maghsoudi[7]

[1]Children's Medical Center, Tehran University of Medical Science, [3]Department of Pediatrics, Faculty of Medicine, Shahid Beheshti University of Medical Sciences, [4]Department of Pediatrics, Mofid Children Hospital, Shahid Beheshti University of Medical Science, [5]Department of Mechanical Engineering School, Iran University of Science and Technology, [6]Department of Neonatology, Akbar Abadi Hospital, Iran University of Medical Sciences, [7]Department of Mechanical Engineering, Mechanical Engineering School, Iran University of Science and Technology, Tehran University of Medical Science, Tehran, [2]Department of Pediatrics, Pediatric Health Research Center, Tabriz University of Medical Science, Tabriz, Iran

Address for correspondence: *Dr. Amid Maghsoudi, Children's Medical Center, 62 Qarib Street, Keshavarz Blvd., Tehran 14194, Iran. E-mail: maghsoudi.amid@gmail.com*

Access this article online

Website: www.jmssjournal.net

DOI: 10.4103/jmss.JMSS_30_20

Quick Response Code:

Introduction

A reduced rate of neonate mortality is an indicator of the development of a country. Zeinalzadeh *et al.* reported three major causes of death of premature neonates, including congenital abnormalities, asphyxia, and infection, and compared their rates throughout Iran. We cannot control the first two factors in the intensive care unit (ICU), but we can decrease infection with early detection.[1] Proven sepsis, by definition, is the time when an organism grows from the blood culture.

Prognosis of newborns' infection based on electrocardiogram (ECG) signals in the neonatal intensive care unit (NICU) has become a hot topic in recent years.[2-4] By analyzing ECG signals we obtain significant heart rate variability (HRV). To detect sepsis, Europe, and many states in the USA, implemented the HeRO in 2012.[2,5] Many developed countries, including Japan and Canada, and most Asian countries, have not yet investigated the heart characteristic for the prognosis of infection; therefore, the present study aimed to examine changes in HRV according to the ECG signal to predict infection. Developing and generalizing engineering prognosis is important because it warns the physician about infection before the emergence of symptoms. This prognosis results in an earlier performance of necessary medical treatment and tests, and ultimately saves many neonates from infection.[6] By engineering processing in this study, the effect of sepsis on the heart characteristics of neonates is investigated.[7-10]

Methods

We performed this study from September 14, 2019, to February 14, 2020, on five neonates admitted to the NICU of Akbar Abadi Hospital in Tehran. We aim to compare heart rate signals 24 h before proven sepsis with healthy neonates by signal processing to predict sepsis in neonates. The day that the consultant announced the neonate had proven sepsis, we processed the day before, heart rate signals stored in the computer center.

This is an open access journal, and articles are distributed under the terms of the Creative Commons Attribution-NonCommercial-ShareAlike 4.0 License, which allows others to remix, tweak, and build upon the work non-commercially, as long as appropriate credit is given and the new creations are licensed under the identical terms.

For reprints contact: WKHLRPMedknow_reprints@wolterskluwer.com

How to cite this article: Mirnia K, Heidarzadeh M, Afjeh SA, Alizadeh P, Kashan AA, Bordbar A, *et al.* Signal processing of heart rate for predicting sepsis in premature neonates. J Med Sign Sens 2021;11:222-6.

Including criteria were gestational age below 32 weeks or weight <1500 g, healthy, or sepsis. Excluding criteria were congenital malformations, metabolic disorders, congenital heart disease, intraventricular hemorrhage (IVH) grade >2, hemodynamically significant patent ductus arteriosus, intubation, and asphyxia. From forty neonates admitted to NICU, only five neonates had eligible criteria for the study. Finally, we studied two neonates, and the rest were excluded from the study due to technical problems. We connected the chest leads to the neonates, and data of heart rate signals were obtained online every 24 h till the time the neonate was discharged. Data are stored in the computer center of the hospital. Monitors belonged to Pooyandegan Rah Saadat Company with a rate of 200 sample/s. The physicians examined the neonates daily and recorded their data in a minimal data set. We analyzed heart rate characteristics 24 h before sepsis.

To minimize the confounding effects of drugs on the heart rate, we connected chest leads after the peak effect of the drugs.

The characteristics of the ECG signal analysis for the prognosis of infection are as follows:

- RR interval: In this diagram, the horizontal axis shows the heartbeat, and the vertical axis indicates the time interval between each R. An irregular final diagram indicates that the neonate is healthy. A regular diagram may arise from a specific illness[11,12]
- RR interval time distribution: As the heart rate of a premature is between 120-160 beat/min demonstrating the Data within 1 hour is difficult; therefore, a distribution time diagram can help to determine the neonate's heart condition
- Sample asymmetry (SampAs) or R_2/R_1 ratio: R_1 and R_2 represent changes in the upper and lower middle of the ECG signal in the RR interval, respectively. These numbers are expressed as Eq. 1 and 2. Suppose, there are n-point ($x_1, x_2, \ldots, x_i, \ldots, x_n$) in each RR interval and their median is m; therefore:[13]

$$R_1 = \frac{1}{n}\sum_{i=1}^{n}\min\left(x_i - m, 0\right)^2 \quad (1)$$

$$R_2 = \frac{1}{n}\sum_{i=1}^{n}max(x_i - m, 0)^2 \quad (2)$$

R_2/R_1 ratio is referred to as the SampAs and represents the number of signal irregularities in the RR interval (between each R peak). The statistical distribution of this number for half an hour (about 5000 RR intervals) should be reasonable for a healthy neonate; an abnormal one indicates sepsis or IVH.[14,15]

Ethics

The project was found to be under the ethical principles and the national norms and standards for conducting medical research in Iran – Approval ID: IR.TUMS.CHMC.REC.1398.039.

Results

In this research, we studied two neonates with and without sepsis. Tables 1 and 2 show the clinical data of these two newborns.

MATLAB 2018 was used to analyze the selected neonates' signals mentioned in the methodology. We gathered the ECG signal of the neonates without sepsis (A) and with sepsis (B). Then, we analyzed HRV after the identification of R peaks.

Figure 1 shows about 10 min of the event between the R peaks of heartbeats. As seen in the figure, Figure 1a shows a healthy sample (with irregularities). The time between R peaks is between 350 and 450 ms. However, in Figure 1b, the duration of the event tends to be about 380 ms. The results of these tests are consistent with previous studies and indicate an infection in the second neonate. Therefore, about 10 min of this signal is displayed to demonstrate the generality addressed in the discussion and to see the time fluctuations between R peaks.

In Figure 2, the statistical distribution of the RR interval of both neonates is plotted for about 45 min. As shown in Figure 2a, the diagram of the uninfected neonate has a normal distribution, but the infected neonate diagram is abnormal. Although the time in Figure 2b tends to be a particular number, it is changed at different times and has a nonnormal distribution.

Figure 3 depicts the frequency data of the SampAs value for the signals of the previous figure for 45 min. In these diagrams, the SampAs in the first case is <0.003 and has a relatively normal distribution, but the SampAs in the second case is <0.02 and has no normal distribution.

Discussion

In a review article in 2011, Moorman *et al.* showed that heart rate characteristics are affected after infection of the neonate's body and before the onset of clinical symptoms. In this study, standard deviation and SampAs were investigated before and after infection of the neonates

Table 1: Characteristics of the newborn with and without sepsis at birth

	Without sepsis	With sepsis
City	Tehran	Tehran
Gender	Male	Male
First-minute Apgar	8	0
Fifth-minute Apgar	10	5
Delivery method	Cesarean section	Cesarean section
Gestational age	30 weeks 1 day	28 weeks 1 day
Birth weight (g)	1400	900
Receiving surfactant	One 7-mL dose of Survanta	Two doses
Resuscitation	No	Advance CPR
Gestation number	G3P1AB2	G2P2AB0

CPR – Cardiopulmonary resuscitation

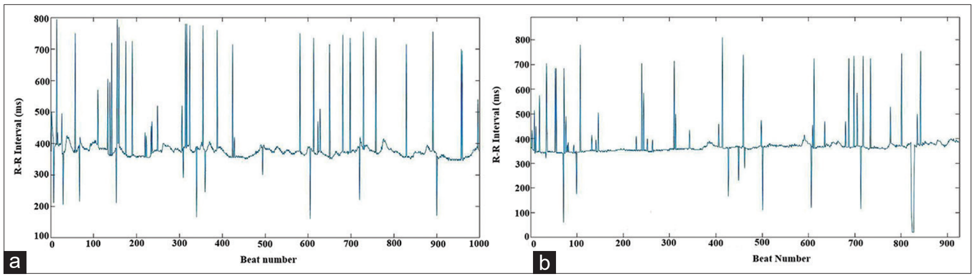

Figure 1: Diagram of the beat number and RR interval of (a) the first neonate. Diagram of the beat number and RR interval of (b) the second neonate

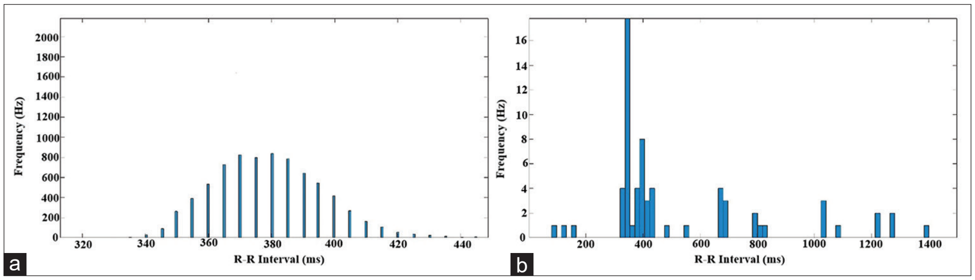

Figure 2: Frequency diagrams of 45-min RR interval of neonates (a) without infection. Frequency diagrams of 45-min RR interval of neonates (b) with infection

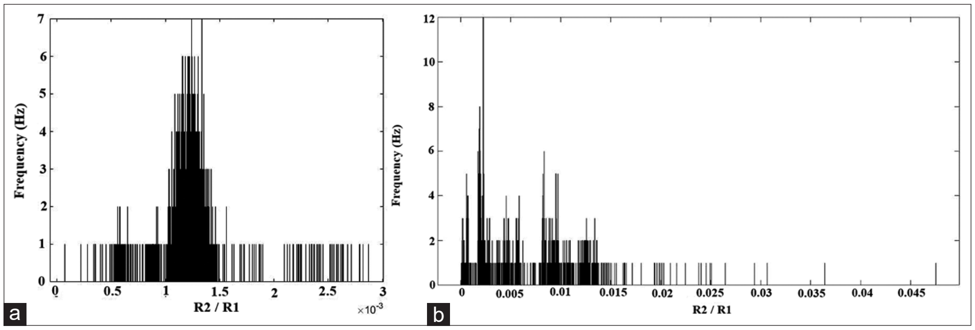

Figure 3: Frequency distribution diagrams of R2/R1 in (a) the first case. Frequency distribution diagrams of R2/R1 in (b) second case

and indicated significant changes in the number and the shape of normal distribution in line with other studies.[16] Based on changes in HRV, an increase in the HeRO score is associated with an increase in neonates' infection. In an article in 2013, this prognosis method was investigated on 3003 premature neonates, after which most NICUs in the United States are using this method.[17] European experts accepted HeRO in 2012, and several studies showed the effectiveness of this prognosis method.[2,5]

A study in Texas (USA) in 2015 showed that the signal analysis of HRV is more sensitive than neonates' vital signs to the presence of lethal diseases such as sepsis. However, further research is required to prove or disprove this method.[18] Sullivan and Fairchild indicated that the use of the HeRO score led to acceptable results in the United States and some centers in Europe selected randomly, and hence, it was confirmed in the USA and Europe in terms of distribution.[2] Ghasemi and Raoufy reported a relationship between mortality prediction and HRV analysis in adults with infections in ICUs of Iran (2016).[19]

Nguyen *et al.* showed that with HRV simple analysis in newborns, they could predict sepsis before the clinical symptoms are appeared.[20] In addition to infection, the accuracy of prognosis through HRV analysis is implemented for other diseases in 2018.[21] In the present study, a newborn with proven sepsis and a premature healthy newborn were investigated in terms of infection through ECG signal analysis. Comparison of the results of Figures 1-3 and their comparison with previous articles showed similar results, indicating the consistency of the HRV pattern in premature neonates with infection and the control neonate, similar to those reported in other parts of the world.

According to what was mentioned earlier, RR interval irregularities indicate the neonate's health. However, this research revealed that this irregularity had a specific regularity because RR intervals had a normal distribution. Nonnormality of this signal characteristic can indicate the neonates' illness. Investigating this subject can help us in the process of engineering prognosis through heart signals of premature neonates to diagnose their diseases. Besides, changes in the SampAs dispersion diagram also showed a considerable change and can help us with the engineering prognosis of infection.

Table 2: Laboratory results of the neonate without and with sepsis

	Date	Time	Weight (on data collection)	Temperature (on data collection)	CRP	Plt	Hb	WBC	WBC		Blood collection	Blood culture	Mechanical ventilation	Bicarbonate	pH
									Neut	Lymph					
Without Sepsis	11/11/2019	10:00 AM	2400 g	37.3	Neg	295000	16.5	106000	37%	60%	No	Neg	Intubation for one day (11/12/2019)	23	7.4
	11/18/2019	-	-	-	Neg	-	-	-	-	-	No	Neg	-	-	-
	Date	Time	Weight (on data collection)	Temperature (on data collection)	CRP	Plt	Hb	WBC	WBC		Blood collection	Blood culture	Mechanical ventilation	Bicarbonate	pH
									Neut	Lymph					
With Sepsis	11/13/2019	10:00 AM	1000 g	37.3	39.6	75000	10.3	8800	70%	27%	Packed cell	Neg	Intubation	24	7.4
	11/20/2019	10:00 AM	1070 g	37.4	49.5	87000	13.9	9100	60%	35%	No	Neg	Intubation	30	7.5
	11/29/2019	10:00 AM	1190 g	37.5	33.8	88000	11.5	16400	57%	38%	Packed cell on 11/26	Neg	NIV	28	7.38

WBC – White blood cell; CPR – Cardiopulmonary resuscitation

Conclusion

In this study, we analyzed the heart characteristics of a premature neonate with and without sepsis. The statistical distribution of the RR interval duration and the SampAs value in the neonate with proven sepsis were abnormal. Concerning the drugs administrated to the neonates, we selected the duration of heart signals to minimize the effects of drugs on the heart signals, and the results of the research have the least uncertainty. The HeRO score implements the engineering processing of heart rates to predict clinical sepsis symptoms. In addition, this study shows that RR interval time has a normal distribution in healthy newborns.

Acknowledgment

We would like to thank from NICU nurses of Akbar Abadi hospital.

Financial support and sponsorship

This study has been funded and supported by Tehran University of Medical Science: Grant no. 98-01-30-41731.

Conflicts of interest

There are no conflicts of interest.

References

1. Zeinalzadeh AH, Khodaei R, Heidarzadeh M. Causes of neonatal mortality in the neonatal intensive care unit of Taleghani Hospital. Iran J Neonatol IJN 2017;8:58-61.
2. Sullivan BA, Fairchild KD. Predictive monitoring for sepsis and necrotizing enterocolitis to prevent shock. Semin Fetal Neonatal Med 2015;20:255-61.
3. Sinha M, Jupe J, Mack H, Coleman TP, Lawrence SM, Fraley SI. Emerging technologies for molecular diagnosis of sepsis. Clin Microbiol Rev 2018;31:1-26.
4. Gupta N, Richter R, Robert S, Kong M. Viral Sepsis in Children. Front Pediatr 2018;6:252.
5. Sullivan BA, Grice SM, Lake DE, Moorman JR, Fairchild KD. Infection and other clinical correlates of abnormal heart rate characteristics in preterm infants. J Pediatr 2014;164:775-80.
6. Fairchild KD, Aschner JL. HeRO monitoring to reduce mortality in NICU patients. Res Rep Neonatol 2012;2:65-76.
7. Honoré A. Machine Learning for Neonatal Early Warning Signs; KTH, School of Electrical Engineering (EES), Information Science and Engineering 2017;43:1-29.
8. Te Pas AB. Improving neonatal care with technology. Front Pediatr 2017;5:110.
9. Fairchild KD, Lake DE, Kattwinkel J, Moorman JR, Bateman DA, Grieve PG, *et al*. Vital signs and their cross-correlation in sepsis and NEC: A study of 1,065 very-low-birth-weight infants in two NICUs. Pediatr Res 2017;81:315-21.
10. Sullivan BA, McClure C, Hicks J, Lake DE, Moorman JR, Fairchild KD. Early heart rate characteristics predict death and morbidities in preterm infants. J Pediatr 2016;174:57-62.
11. Stone ML, Tatum PM, Weitkamp JH, Mukherjee AB, Attridge J, McGahren ED, *et al*. Abnormal heart rate characteristics before clinical diagnosis of necrotizing enterocolitis. J Perinatol 2013;33:847-50.
12. Flower AA. An Investigation of Decelerations in the Neonatal Heart Rate. ProQuest; 2008.

13. Cao H, Lake DE, Griffin MP, Moorman JR. Increased nonstationarity of neonatal heart rate before the clinical diagnosis of sepsis. Ann Biomed Eng 2004;32:233-44.
14. Kovatchev BP, Farhy LS, Cao H, Griffin MP, Lake DE, Moorman JR. Sample asymmetry analysis of heart rate characteristics with application to neonatal sepsis and systemic inflammatory response syndrome. Pediatr Res 2003;54:892-8.
15. Moorman JR, Delos JB, Flower AA, Cao H, Kovatchev BP, Richman JS, *et al*. Cardiovascular oscillations at the bedside: Early diagnosis of neonatal sepsis using heart rate characteristics monitoring. Physiol Meas 2011;32:1821-32.
16. Moorman JR, Carlo WA, Kattwinkel J, Schelonka RL, Porcelli PJ, Navarrete CT, *et al*. Mortality reduction by heart rate characteristic monitoring in very low birth weight neonates: A randomized trial. J Pediatr 2011;159:900-6.e1.
17. Hicks JF, Fairchild K. HeRO monitoring in the NICU: Sepsis detection and beyond. Infant Journal 2013;9:187-91.
18. Bohanon FJ, Mrazek AA, Shabana MT, Mims S, Radhakrishnan GL, Kramer GC, *et al*. Heart rate variability analysis is more sensitive at identifying neonatal sepsis than conventional vital signs. Am J Surg 2015;210:661-7.
19. Ghasemi P, Raoufy MR, editors. Prediction of mortality in patients with sepsis using detrended fluctuation analysis of heart rate variability. 2016 23rd Iranian Conference on Biomedical Engineering and 2016 1st International Iranian Conference on Biomedical Engineering (ICBME); 2016: IEEE.
20. Nguyen N, Vandenbroucke L, Hernández A, Pham T, Beuchée A, Pladys P. Early-onset neonatal sepsis is associated with a high heart rate during automatically selected stationary periods. Acta Paediatrica 2017;106:749-54.
21. Oliveira V, Martins R, Liow N, Teiserskas J, von Rosenberg W, Adjei T, *et al*. Prognostic accuracy of heart rate variability analysis in neonatal encephalopathy: A systematic review. Neonatology 2019;115:59-67.
