# Supplementary material for: Exploring Computational Techniques in Preprocessing Neonatal Physiological Signals for Detecting Adverse Outcomes: Scoping Review
Source: Interact J Med Res. 2024 Aug 20;13:e46946. doi: 10.2196/46946 (PMC11372324; doi:10.2196/46946)
Supplement: Multimedia Appendix 3 [file ijmr_v13i1e46946_app3.zip › Included Papers - Final/3199/Zimmet et al. - 2020 - Trajectories of the heart rate characteristics ind.pdf]

Check for updates

Research Paper

JRSM Cardiovascular Disease
Volume 9: 1–10
© The Author(s) 2020
Article reuse guidelines:
sagepub.com/journals-permissions
DOI: 10.1177/2048004020945142
journals.sagepub.com/home/cvd
SAGE

Trajectories of the heart rate characteristics index, a physiomarker of sepsis in premature infants, predict Neonatal ICU mortality

Amanda M Zimmet[1], Brynne A Sullivan[2], J Randall Moorman[1], Douglas E Lake[1] and Sarah J Ratcliffe[3]

Abstract

Objective: Trajectories of physiomarkers over time can be useful to define phenotypes of disease progression and as predictors of clinical outcomes. The aim of this study was to identify phenotypes of the time course of late-onset sepsis in premature infants in Neonatal Intensive Care Units.

Methods: We examined the trajectories of a validated continuous physiomarker, abnormal heart rate characteristics, using functional data analysis and clustering techniques.

Participants: We analyzed continuous heart rate characteristics data from 2989 very low birth weight infants (<1500 grams) from nine NICUs from 2004–2010.

Result: Despite the relative homogeneity of the patients, we found extreme variability in the physiomarker trajectories. We identified phenotypes that were indicative of seven and 30 day mortality beyond that predicted by individual heart rate characteristics values or baseline demographic information.

Conclusion: Time courses of a heart rate characteristics physiomarker reveal snapshots of illness patterns, some of which were more deadly than others.

Keywords

Predictive Modeling, Continuous Monitoring, Functional Data Analysis, Clinical Phenotypes

Date received: 25 November 2019; revised: 25 June 2020; accepted: 2 July 2020

Introduction

Clinical features of neonatal sepsis can be non-specific, hampering the ability to intervene early in the course of disease. Continuous monitoring can identify subtler signs of infection and facilitate earlier detection of sepsis.[1,2] Neonates have a unique physiological response to infection, manifested as reduced heart rate variability and transient decelerations in heart rate.[3] These heart rate characteristics (HRCs) can be calculated from routine bedside monitoring data, and have been developed into a predictive monitoring score as the HRC index, allowing real-time computation of fold-risk for sepsis in the next 24 hours.[4] A previous randomized controlled trial (RCT) demonstrated that display of the HRC index at the bedside alongside its five-day historic trend reduces infant mortality.[5] The mean HRC index trajectory from this multicenter trial plots as a flat line with a rise in the day before a blood culture,[6] but not all individual patients followed this trajectory before sepsis diagnosis.

While the instantaneous HRC index is well calibrated to provide the fold-risk of sepsis at a given time, it is not known whether the trajectory of the index could provide additional clinical utility. Clinical intuition

[1]University of Virginia, Medicine, Charlottesville, VA
[2]University of Virginia, Pediatrics, Charlottesville, VA
[3]University of Virginia, Public Health Sciences, Charlottesville, VA

Corresponding author:
Sarah J Ratcliffe, University of Virginia, PO Box 800717, Charlottesville, VA 22908, United States.
Email: sjr7pc@virginia.edu

Creative Commons Non Commercial CC BY-NC: This article is distributed under the terms of the Creative Commons Attribution-NonCommercial 4.0 License (https://creativecommons.org/licenses/by-nc/4.0/) which permits non-commercial use, reproduction and distribution of the work without further permission provided the original work is attributed as specified on the SAGE and Open Access pages (https://us.sagepub.com/en-us/nam/open-access-at-sage).

suggests an upward spike in the index should lead the physician to consider obtaining a blood culture, and this has borne out to be a high yield strategy.[7] However, identification of other common trajectories has not been explored and might add further information to the clinical picture.

Functional data analysis is a body of statistical tools which allow for the identification and analysis of common trajectory shapes seen in a dataset. These methods were developed to leverage the information contained in a patient's entire time series as a "function" variable without the loss of information inherent in creating summarized variables at a given time point. We hypothesized that utilizing functional data analysis alongside unsupervised clustering to identify and classify common trajectories in the HRC index would add value in predicting mortality, sepsis, or culture result beyond the raw HRC index and basic demographic information. This may further assist in identifying neonates at risk of impending clinical deterioration.

Methods

The HRC Index

The HRC index combines mathematical algorithms to detect reduced heart rate variability and transient decelerations in heart rate into a single fold-risk score for sepsis. It is computed via a normalized logistic regression algorithm that combines the standard deviation (indicator of reduced variability), sample asymmetry (indicator of transient decelerations),[8] and sample entropy (a measure of pattern regularity that reflects both variability and decelerations)[9] of the RR intervals from the bedside monitor.[4] The HRC index has been mapped to clinical events and shown to support decisions of neonatologists and nurses in saving lives.[2–5,10–12]

The commercial HRC index monitor used in this study was the HeRO (Heart Rate Observation) monitoring system (Medical Predictive Science Corporation, Charlottesville, VA). It calculates the HRC index components from bedside monitor data and displays the current fold-risk alongside a time series plot of the hourly HRC index from the past five days.

Because the HRC index is a fold-risk score, the population mean of the index over all time is set at one. Consequently, a HRC index of two should be understood as a twofold risk of sepsis compared to baseline in the next 24 hours. The HRC index is truncated at a maximum value of seven and waxes and wanes throughout the NICU stay. Infants with persistently low HRC indices tend to have smooth clinical courses, while infants with severe chronic lung disease or brain injury can have persistently high HRC indices.[10,13,14] We studied the HRC trajectory shapes in the five day period leading up to blood culture orders. The five day window was chosen to match the five day historic HRC index trend shown to clinicians on the display.

Dataset

HRC index data from very low birth weight infants (<1500 grams) from nine NICUs were collected from 2004–2010 in a randomized control trial (RCT) where half of the infants had their HRC indices displayed and the other did not.[5] Parents provided written informed consent when necessary to participate in the trial. This study re-analyzes the data recorded in the trial.

Since most of the survival benefit occurred in infants who had sepsis, the benefit of the monitoring display was attributed to early warning of impending illness, leading to earlier intervention (Fairchild, 2013).[12] Importantly, the display group might exhibit a different time course leading to blood culture than the non-display group due to this early warning. To investigate whether the HRC index trajectory added valuable information to the current HRC index, we developed a model based on the five days of data from the display group. Then, we applied a version of that model (described below in the *Comparison to Non-Display Group Infants* section) to the non-display group as an out-of-group comparison.

The analytic sample consisted of HRC index data five days prior to every bacterial, yeast, and/or fungal culture with at least 50% of the measurements available. Data leading to both positive and negative cultures were analyzed. All blood cultures taken in the seven days after a positive culture were excluded except for new positive cultures of a different organism. Cultures obtained on the day of admission, when the infant was less than three days old, and those which returned an unknown or questionable result were also excluded.

Identification of Characteristic Trajectory Phenotypes

We used functional data analysis[15] to model the HRC index trajectories of display group infants in the five days leading up to a blood culture. Functional data analysis refers to a branch of statistical techniques that can identify and analyze the unique shapes seen in a dataset of trajectories. We used functional principal component analysis[16,17] to reduce the dimensionality of the five days of data from each patient while maintaining the essential features of the curvature of the trajectory. This works by finding a set of principal component curves which represent most of the variability in the dataset. Each individual trajectory can then

be represented by the sum of these principal component curves multiplied by a set of corresponding coefficients. This reduction in the dimensionality of the dataset allows for the use of clustering (see the *Clustering* subsection) to find the coefficients that are most commonly seen together. Then, we multiplied the principle component curves by these sets of characteristic coefficients and summed the curves to generate visualizations of the most common curves seen in the dataset (see *Characteristic Curves* subsection).

Functional Data Analysis. In functional data analysis, each trajectory is represented by a linear combination of basis functions that captures the time-varying components. A variety of basis functions can be selected based on the characteristics of the original trajectories, the most familiar being the Fourier series, which is useful for fitting periodic data. Here we chose a B-spline basis because we sought to capture the subtleties of non-periodic trajectories.

Hourly HRC indices over the five day period were captured, yielding up to 121 data points per infant. Infants with missing data at either end of the five day window were extrapolated to the window edge by repeating the most proximal HRC index value. Interior missing values were filled with linear interpolation.

The HRC index monitor algorithm uses the past 12 hours of patient data to generate a single score per hour. Thus, HRC indices 12 samples apart are independent. In order to capture enough information about the curvature of these independent samples we chose to fit one B-spline per six samples (plus one due to the odd number of input data points), resulting in a choice of 21 B-splines. In order to be able to look at the acceleration function, which requires trajectories to be smooth to the fourth derivative, we used fifth order B-splines with equally spaced knots.[18]

Functional principal component analysis[16,17] was used to determine the dominant variability in the HRC index trajectories. The top four principal components were selected since they explained 89% of the data's variability. The functional principal components were then used to represent each infant's data by a linear combination of the four component curves. This generated a smoothed version of the infant's original curve, and reduced the dimensionality of each infant's data from 121 hourly HRC indices to four principal component coefficients. These above steps were completed using modified code from J.O. Ramsay.[19,20]

Clustering. To find the common characteristics of these sets of principal component coefficients, we used an unsupervised ensemble clustering technique. This method uses a k-means cluster generation mechanism with different values of k; the ensemble members were refined using selective clustering by cluster entropy; and the consensus function was locally weighted evidence accumulation (LWEA), a version of hierarchical agglomerative clustering.[21,22]

Characteristic Curves. To generate a visualization of the characteristic curve from each cluster, we found the coordinates of the centroid of each cluster. The linear combination of these coordinates (which are coefficients of the four principal components) with the corresponding principal component curves yielded the characteristic curve. We recognize that, given this is ensemble clustering, the clusters are not necessarily spherical, so the centroid has less meaning than it would in a k-means clustering and is not necessarily the ideal representative curve for the cluster. However, our hope was that the curve would allow us to pair statistical outcomes with a rough visualization of a clinical trajectory.

Model Comparison

In premature infants, baseline risk of late onset neonatal sepsis is correlated with birthweight and days of age. The HRC index at time of blood culture adds further information.[6] We investigated the additional information that cluster (trajectory phenotype) membership added to the risk of a variety of outcomes. Outcomes investigated were all binary in nature: positive vs negative blood culture, mortality within 30 days vs survival for 30 days, mortality within seven days vs survival for seven days, culture-proven sepsis vs clinical sepsis or sepsis ruled out, positive blood culture (excluding coagulase negative staphylococcus species (CONS)) vs negative culture or CONS, and gram-negative organism cultured vs gram positive organism cultured or negative culture. These outcome labels were determined based on detailed data collected by research personnel and manual neonatologist corrections as part of the randomized clinical trial.[12]

Baseline risk of each outcome for each infant was calculated as the discrete prior probability of the outcome given birthweight and days of age. Bins were created for birthweight in 200 g intervals from 200 to 1600 g (since the max birthweight was 1500 g, the last bin contained infants only between 1400 and 1500 g) and for age in four day intervals from zero to 224 days. Baseline risk was calculated as the rate of the outcome in that infant's bin excluding the infant of interest.

Logistic regression was used to determine whether the cluster category provided additional information about any of the outcomes. The inputs to the logistic regression model were the prior probability of the

outcome given birthweight and days of age, the HRC index at the time of blood culture, and a binary variable indicating the cluster to which the infant was assigned. We repeated this process with each of the different outcomes, with the number of clusters ranging from two to 12. The number of clusters included in each model is denoted by "+ [number] Clusters" in the model name.

Re-seeded Clustering

To rule out the possibility of the seed skewing our modeling results, we re-ran the clustering algorithm with 100 different seed values for every model. These cluster results were used for the logistic regression procedure and an average Akaike information criterion (AIC), Bayesian information criterion (BIC), and area under the receiver operating characteristic curve (AUC) (across seeds) were computed for each model.

Comparison to Non-Display Group Infants

Validity of the results was investigated by applying the display group infants' functional principal components and clustering to the non-display group infants. While non-display group infants might not have the same decision point for cultures as the display group infants, changes in cluster membership percentages and outcomes risks by trajectory phenotypes might help quantify the utility of HRC index display. Because the clustering method we used for the display group infants was a hierarchical agglomerative clustering method, new samples could not be incorporated into the clustering algorithm without altering the model itself. Therefore, we used the principal component coefficients and cluster labels from the display group infants in Matlab's bagged trees classifier to create a classification algorithm. This algorithm took in the four principal component coefficients for each infant and output a cluster label.

Results

The original RCT data set included 4379 blood cultures on 1500 infants randomized to HRC index monitoring display that met the inclusion criteria. Of these, 3800 (80.2%) had 50% or more data availability in the five days prior to the culture and were included in the analysis. 1489 infants were randomized to the non-display group. In the non-display group, there were 3827 blood cultures. Of these, 3211 had 50% or more data availability, and thus were included in the analysis. There was a great deal of variability among HRC index trajectories (Figure 1a). Figures 1b-d show the transformation of this high dimensional dataset into a four principal component representation.

With six clusters we achieved the best tradeoff between adding information and variable parsimony. Additionally, the most consistent performer in the model fitting across all outcomes was the model with *Birthweight/Days of Age + Last HRC index + 6 Clusters* when varying the number of B-splines, the number of basis functions, the starting seed, and the outcome parameter. Mortality prediction (both seven day and 30 day) was bolstered by the cluster assignment information. The improvement in prediction using six trajectory phenotypes is shown in Table 1 (for a table showing all models tested, including comparisons to alternative simple models such as maximum HRC Index, see Supplemental Table 1). AUC and AIC improved for both display and non-display groups for both seven and 30 day mortality with addition of the 6 Clusters to the model. Characteristic curves are shown in Figure 2 along with the associated mortality risk. Figure 3 shows the Receiver Operating Characteristic (ROC) Curves for each of the models in Table 1, along with the baseline ROC curve for birthweight/days of age alone (an augmented version of this figure including more alternative models is included as Supplemental Figure 1). Clusters 3 and 6 have higher seven day mortality risk, while Clusters 5 and 6 have higher 30 day mortality risk. The regression results for the non-mortality outcomes are shown in Table 2.

Re-running each of the models with 100 different cluster seeds supported our selection of six clusters. The *Birthweight/Days of Age + Last HRC Index + 6 Clusters* model had the lowest mean AIC of all the models for seven day mortality, and the second lowest mean AIC for 30 day mortality – superseded only by the *Birthweight/Days of Age + Last HRC Index + 5 Clusters* model. For all of the models that excluded the Last HRC Index, the *Birthweight/Days of Age + 6 Clusters* model had the lowest AIC for both seven and 30 day mortality. The mean AIC, BIC, and AUC values are shown in Supplemental Tables 2 and 3. Across the 100 cluster seeds, the addition of the six clusters maintained its improvement in AUC. The *Birthweight/Days of Age + Last HRC Index* model and the *Birthweight/Days of Age + Last HRC Index + 6 Clusters* model returned 7 day mortality AUC values of 0.735 and 0.761 ± 0.008, respectively (an improvement of 0.026 ± 0.008) with the addition of the six clusters. 30 day mortality AUC values improved as well with the addition of the six clusters, increasing from 0.714 to 0.735 ± 0.004 (an improvement of 0.021 ± 0.004).

Display group infants had a higher overall culture count (Table 3), which might be attributable to clinicians reacting to high scores by obtaining cultures. These additional cultures were especially prevalent in infants belonging to Clusters 2 through 4. Only the

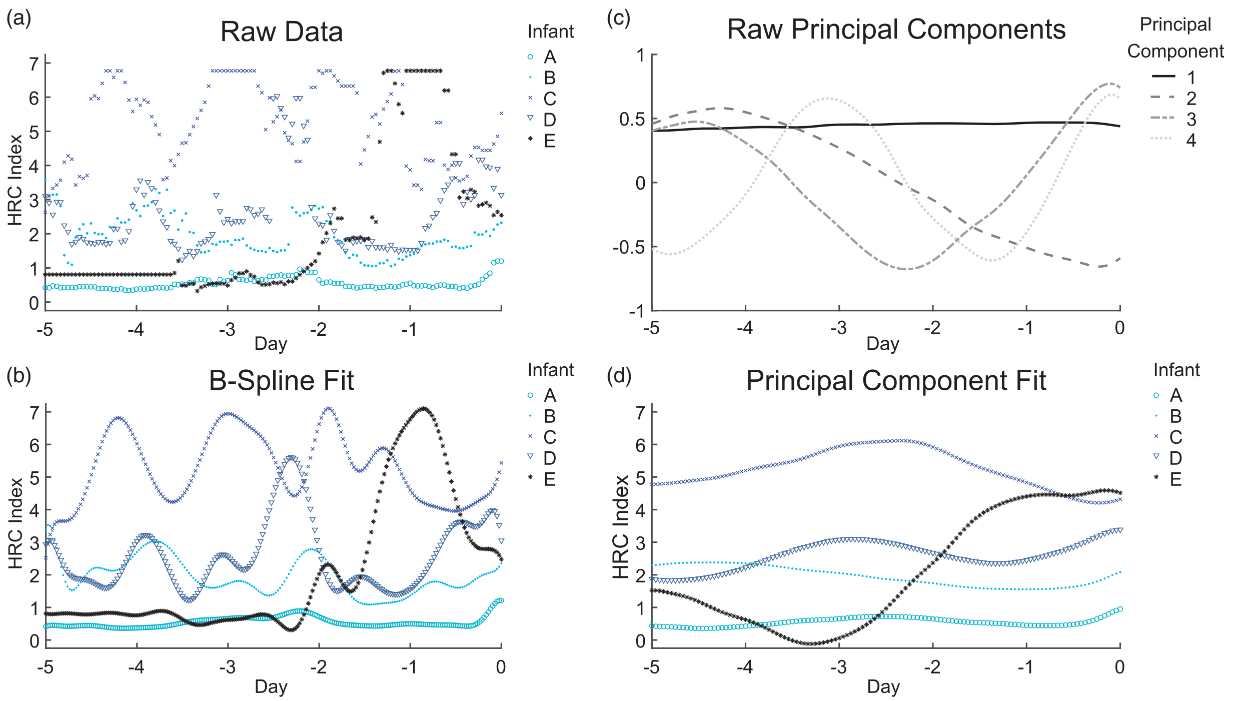

Figure 1. Examples of the Dimension Reduction Procedure
(a) Raw data from five infants show the wide variety of trajectories leading up to blood culture. (b) The tight B-spline fit to the raw data for each of the five infants in (a). (c) The top four principal components that were generated from the full dataset. (d) For the five example infants we show the low-dimensional representation of each infant. We multiply each infant's four principal component coefficients by the principal component curves in (c), sum them together, then add the result to the mean curve. This yields the smooth curves shown here.

Table 1. The cluster assignments added predictive value to both seven and 30 day mortality outcomes. The *Birthweight/Days of Age + Last HRC Index + 6 Clusters* model shows an improvement in AUC and AIC over the baseline *Birthweight/Days of Age + Last HRC Index* model for both seven and 30 day mortality outcomes for both the display and non-display groups. DF = degrees of freedom. AUC = area under the receiver operating characteristic curve. AIC = Akaike information criterion. BIC = Bayesian information criterion.

			Display Group			Non-Display Group		
Outcome	Model	DF	AUC	AIC	BIC	AUC	AIC	BIC
7 Day Mortality	Birthweight/Days of Age + Last HRC Index	2	0.74	892.0	904.5	0.73	962.3	974.4
	Birthweight/Days of Age + 6 Clusters	6	0.76	869.3	906.7	0.71	982.2	1018.7
	Birthweight/Days of Age + Last HRC Index + 6 Clusters	7	0.77	870.3	914.0	0.74	966.6	1009.1
30 Day Mortality	Birthweight/Days of Age + Last HRC Index	2	0.71	1646.9	1659.4	0.75	1745.9	1758.1
	Birthweight/Days of Age + 6 Clusters	6	0.74	1601.1	1638.6	0.75	1744.0	1780.4
	Birthweight/Days of Age + Last HRC Index + 6 Clusters	7	0.74	1597.9	1641.6	0.76	1728.9	1771.4

display group infants in Cluster 6 had a lower number of cultures.

Discussion

We studied trajectories of the heart rate characteristics index, the fold-increase in risk of sepsis in the next 24 hours, in a large cohort of infants from a multicenter randomized trial. The findings expand considerably on our prior work to examine how risk trajectories are related to clinical events. Sullivan et al. previously reported that a rise from a stable and low risk five-day baseline to a high risk (more than threefold increase in HRC index) had a more than 50% positive

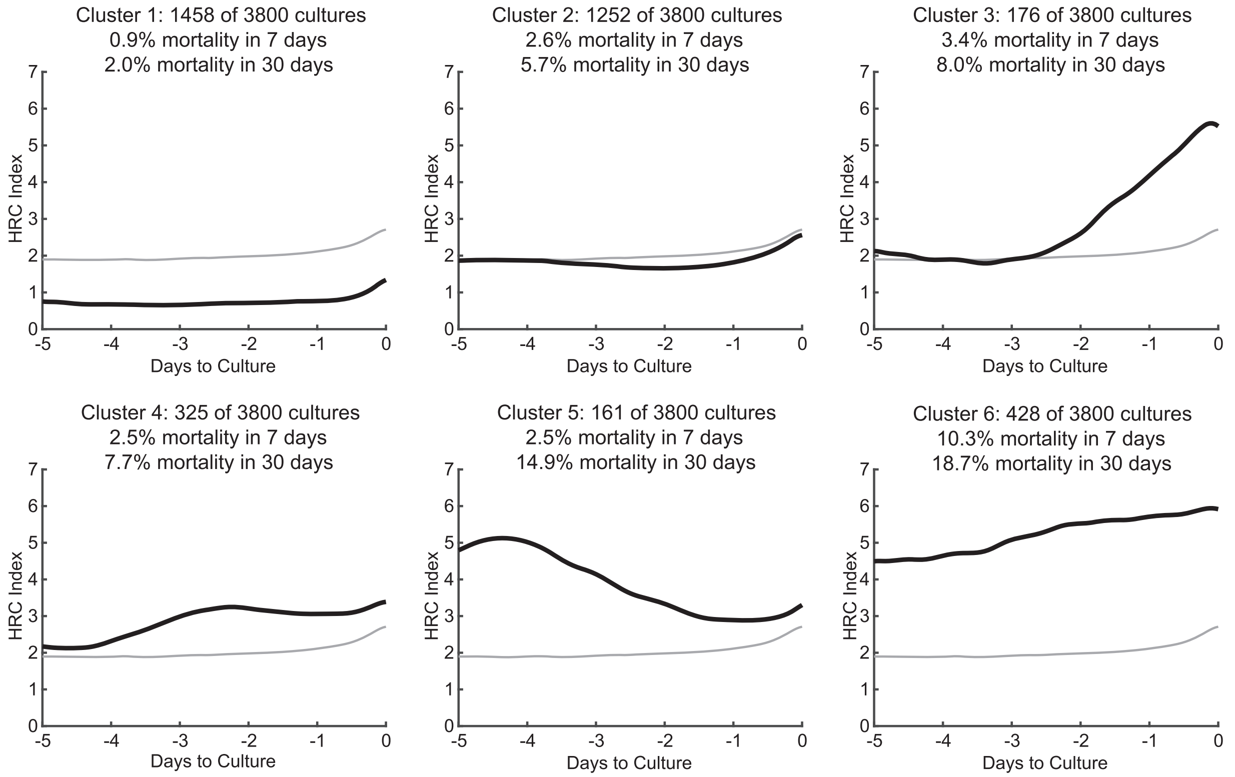

Figure 2. Characteristic HRC Index Trajectories Leading to Blood Culture in Display Group Infants.
The centroids of the six clusters provide an approximation of the trajectory common in each cluster. Gray line = mean trajectory across all infants; black lines = cluster-specific characteristic trajectory. The infants from Figure 1 were assigned to the following clusters: Infant A: Cluster 1, Infant B: Cluster 2, Infant C: Cluster 6, Infant D: Cluster 4, Infant E: Cluster 3.

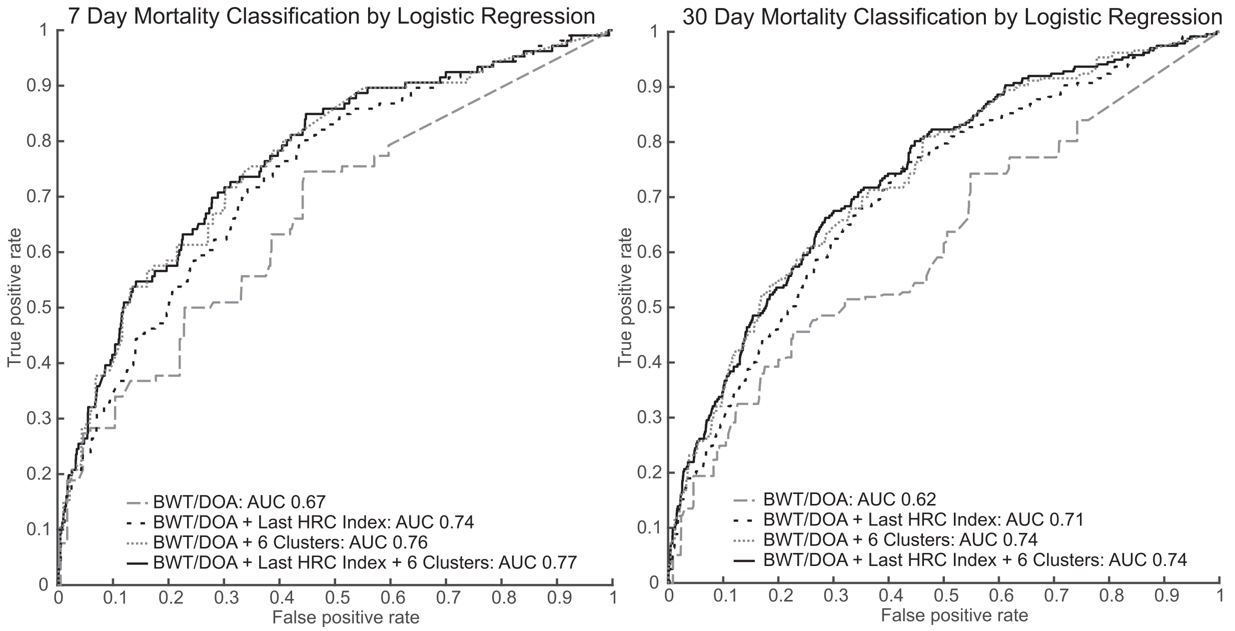

Figure 3. ROC Curves for 7 and 30 Day Mortality
Adding the cluster assignments improves both seven and 30 day mortality prediction. Only display infants are shown here. BWT = Birthweight. DOA = Days of Age.

Table 2. AUCs for non-mortality outcomes show that the clusters did not add predictive value to identifying any of the other outcomes tested other than mortality. DF = degrees of freedom. CONS = coagulase negative staphylococcus.

	Birthweight/ Days of Age+ Last HRC Index (DF = 2)		Birthweight/ Days of Age + 6 Clusters (DF = 6)		Birthweight/ Days of Age + Last HRC Index + 6 Clusters (DF = 7)	
	Display	Non-Display	Display	Non-Display	Display	Non-Display
[Positive] vs [Negative] Blood Culture	0.54	0.58	0.54	0.57	0.55	0.59
[Sepsis Ruled Out] vs [Clinical Sepsis or Sepsis]	0.56	0.58	0.55	0.57	0.56	0.59
[Non-CONS Bacteria] vs. [Negative Culture or CONS]	0.55	0.58	0.56	0.56	0.58	0.58
[Gram Negative] vs [Everything Else]	0.56	0.57	0.57	0.58	0.61	0.58

Table 3. Cluster-Specific Event Distribution Reviewing both the event counts and rates is essential because an overall increase in the number of cultures in a HRC index display cluster can lead to an increased event count but also a decreased event rate. A targeted increase in culture rate could be warranted in a particular cluster because of the increased event rate, but the justification for such an increase could be washed out if only reviewing the percentage.

Cluster	1	2	3	4	5	6	Total
Culture Count							
Non-Display	1329	932	147	206	160	437	3211
Display	1458	1252	176	325	161	428	3800
7 Day Mortality Rate (% of cultures in cluster which preceded mortality within 7 days)							
Non-Display	1.5%	3.1%	7.5%	3.9%	7.5%	9.8%	
Display	0.9%	2.6%	3.4%	2.5%	2.5%	10.3%	
7 Day Mortality Count (number of infants whose cultures in this cluster preceded mortality within seven days)							
Non-Display	15	22	10	7	8	30	92
Display	11	28	6	7	4	30	86
30 Day Mortality Rate (% of cultures in cluster which preceded mortality within 30 days)							
Non-Display	2.9%	7.7%	18.4%	13.1%	12.5%	25.9%	
Display	2.0%	5.7%	8.0%	7.7%	14.9%	18.7%	
30 Day Mortality Count (number of infants whose cultures in this cluster preceded mortality within 30 days)							
Non-Display	24	42	21	20	13	48	168
Display	20	43	14	21	19	41	158
Clinical Sepsis Rate (% of cultures leading to 5+ days of antibiotics with negative blood culture)							
Non-Display	27.8%	37.9%	34.7%	36.9%	32.5%	37.1%	
Display	30.0%	36.7%	40.9%	34.2%	42.2%	38.8%	
Clinical Sepsis Count (number of infants whose cultures in this cluster prompted 5+ days antibiotics with negative blood culture)							
Non-Display	235	207	45	62	36	98	683
Display	258	262	62	78	55	103	818
Sepsis Rate (% of cultures positive and treated with 5+ days antibiotics)							
Non-Display	11.3%	12.4%	15.6%	16.0%	9.4%	14.4%	
Display	11.3%	10.3%	15.3%	9.2%	13.7%	12.9%	
Sepsis Count (number of infants with positive blood cultures treated with 5+ days antibiotics)							
Non-Display	136	105	22	31	15	58	367
Display	138	111	26	29	22	49	375

predictive value for suspected or clinical infection.[7] Here, we have used sophisticated mathematical methods of functional data analysis and unsupervised clustering to discover relevant trajectories beyond the simplified approach of analyzing large spikes.

We found six trajectory clusters that are consistent with clinical scenarios ranging from small increases from a low baseline score to large decreases from a high baseline score. Non-display group infants showed similar trajectory clusters distributions as the display group infants. These trajectories added predictive value for mortality risk beyond that provided by the instantaneous HRC index, birthweight, and days of age. This finding underscores the utility of considering

trajectories rather than spot checks of risk estimates in the field of predictive analytics monitoring.

Moorman et al. did not directly investigate the mechanism of mortality reduction in the display group infants in the randomized controlled trial, but hypothesized that it was due to early detection of sepsis or other systemic inflammation.[5] This hypothesis was supported by the Fairchild et al.[12] finding that the mortality reduction was greatest in septic infants. Here we look at the changes in the distribution of cluster assignments for both the display and non-display infants in Table 3 to help elucidate places where the HRC index monitor may have led to changes in clinical practice, resulting in mortality reduction.

The six distinct trajectories in Figure 2 each tell a story when paired with our clinical experience working alongside these monitors and the outcomes computed in Table 3. The clinical vignettes we deduce from these sources provide a first glimpse toward explaining how clinicians interacted with the HRC index monitor system to reduce mortality.

Cluster 1 - Stable Clinical Course - Cluster 1, the most common trajectory, represents infants with a low baseline HRC index and likely stable clinical course and a blood culture drawn just as the HRC index began to rise, prompted by a clinical change from baseline. Mortality rate in this group was low and not significantly lower in display group infants. Infants in this cluster had the lowest percentage of cultures preceded by another within 5 days (21%).

Clusters 2 and 4 –Increased Risk - Clusters 2 and 4 represent a similar trajectory to Cluster 1 though with a higher average 5-day baseline preceding culture and a slightly higher mortality rate, likely indicating an overall moderately sicker group of infants compared to Cluster 1. Infants in Cluster 4 had the highest percentage of cultures preceded by another within 5 days (49%).

Cluster 3 – Rapidly Rising Risk - Cluster 3 shows the HRC index trajectory following a course from low to rapidly rising approximately two days prior to blood culture. This is the expected signature for the onset of illness, and what would be expected to provide the greatest clinical utility. The mortality outcomes support this: the display group infants in this group have a lower 7 and 30 day mortality count (6 vs 10 for 7 day mortality and 14 vs 21 for 30 day mortality) and rate (3.4% vs 7.5% for 7 day mortality and 8.0% vs 18.4% for 30 day mortality) than non-display infants.

Additionally, the clinical sepsis rate and counts were higher for the display than the non-display infants in Cluster 3. This points to the possibility that the HRC index trajectory may have been used as an early warning or tiebreaker in the face of early, equivocal clinical signs of sepsis, possibly influencing clinicians' decisions to continue antibiotics despite negative blood cultures. While it is also important to avoid antibiotic overuse for unproven sepsis, there may have been a mortality benefit from empiric treatment for display group infants with this trajectory, given that display group infants showed a decrease in both the mortality rate and count.

Cluster 5 - Not Out of the Woods Yet - We can conjecture that Cluster 5 represents infants whose initial peak was missed clinically, and now they peak again. In our earliest studies, we found that the HRC index has a waxing and waning quality prior to clinical recognition in some cases of sepsis[4]. Moreover, the periodic and recurrent nature of bacteremia has been documented in other clinical settings[23]. The large mortality difference seen here (between 2.5% mortality in seven days vs 14.9% mortality in 30 days) indicates that though the infant's trajectory is improving in the days leading to culture, he or she is still at increased risk for death in the subsequent 30 days.

It is noteworthy that this is the only cluster where the 30 day mortality rate increased in comparison to the non-display group. This points to a potential danger that a high score (5) dropping down to a lower score (3), could unintentionally promote a false sense of security, despite the score of 3 still indicating a threefold risk in comparison to baseline.

Cluster 6 – High Risk - Cluster 6 had the highest seven and 30 day mortality rate and count. Both culture-positive and clinical sepsis were common in this group. This cluster likely represents infants who were sick for days before the blood culture was drawn. Comparing cultures from display group infants in this cluster to non-display, fewer blood cultures were categorized into this cluster (428 vs 437), fewer resulted in a diagnosis of sepsis (12.9% vs 14.4%), fewer were followed by death within 30 days (18.7% vs 25.9%), and more cultures (44% vs 40%) were preceded by another culture during the prior five days. Moreover, fewer infants with HRC index displayed were diagnosed with sepsis in Cluster 6, while sepsis counts were generally higher in the display group than the non-display group for the other trajectory clusters. These data suggest that HRC index display may have resulted in earlier diagnosis or treatment of sepsis and fewer sepsis-related deaths. Overall, Table 3 provides evidence supporting the hypothesis that the HRC index monitoring system is allowing for earlier recognition of illness in VLBW infants.

Conclusion

The results of our analysis inform use of HRC index monitoring for clinical care in several ways. First, we found that the most notable mortality advantage in

patients randomized to HRC index display occurred in infants with a rise in HRC index from a low baseline that preceded blood culture by almost two days (Cluster 3) and therefore may have encouraged antibiotic administration in a situation with equivocal clinical signs of illness. Second, different HRC index trajectories are associated with different patterns in practice, measured by rates of blood cultures and clinical sepsis diagnoses, or the decision to extend antibiotics in the face of a negative blood culture. Furthermore, mortality and sepsis rates associated with each of six distinct HRC index trajectories provide information on mortality risk associated with different patterns in the manifestation of abnormal physiology. Finally, our analysis shows that knowing the trajectory cluster adds information on mortality risk over that predicted by birthweight, days of age and last HRC index.

Acknowledgements

We would like to thank the original HeRO trial clinical investigators: Waldemar A. Carlo, MD, John Kattwinkel, MD, Robert L. Schelonka, MD, Peter J. Porcelli, MD, Christina T. Navarrete, MD, Eduardo Bancalari, MD, Judy L. Aschner, MD, Marshall Whit Walker, MD, Jose A. Perez, MD, Charles Palmer, MD, George J. Stukenborg, PhD, and Thomas Michael O'Shea, MD. We are grateful to the original HeRO trial study coordinators as well: Stella Parolisi, Cathy Horton, Terri Smoot, Patty Brown, Monica Collins, Shirley Cosby, Daniela Castano, Steven Steele, Amy (Law) Beller, Nikki Barrett, Charlene Wells, Catherine Wilson, Paige Wilson, Judy Vallati, and Amy Blackman.

Contributorship

AZ, SR, and DL conceived the study. DL provided the data and support for using it. AZ analyzed the data. SR and DL provided guidance in the data analysis process. AZ and RM wrote the first draft of the paper. BS provided clinical expertise regarding interpretation of the data. All authors reviewed and edited the manuscript and approved the final version of the manuscript.

Data

Data and code are posted to the University of Virginia Dataverse: https://dataverse.lib.virginia.edu/dataset.xhtml?persistentId=doi:10.18130/V3/2XENWC.[24]

Declaration of Conflicting Interests

The author(s) declared the following potential conflicts of interest with respect to the research, authorship, and/or publication of this article: RM and DL have equity shares in Medical Predictive Science Corporation, Charlottesville, VA. RM is an officer and owns equity in Advanced Medical Predictive Devices, Diagnostics, and Displays. The other authors declare no conflicts of interest.

Ethical approval

This was approved by the University of Virginia Institutional Review Board - Health Science Research #21985.

Funding

The author(s) disclosed receipt of the following financial support for the research, authorship, and/or publication of this article: Translational Health Research Institute of Virginia. National Institutes of Health R01- HD48562. Medical Predictive Science Corporation, Charlottesville, VA.

Guarantor

SR

ORCID iDs

Amanda M Zimmet https://orcid.org/0000-0003-1457-3072
Sarah J Ratcliffe https://orcid.org/0000-0002-6644-8284

References

1. Griffin MP, Lake DE, Bissonette EA, et al. Heart rate characteristics: novel physiomarkers to predict neonatal infection and death. *Pediatrics* 2005; 116: 1070–4.
2. Griffin MP, Lake DE, O'Shea TM, et al. Heart Rate Characteristics and Clinical Signs in Neonatal Sepsis. *Pediatr Res* 2007; 61: 222–227.
3. Griffin MP, Moorman JR. Toward the Early Diagnosis of Neonatal Sepsis and Sepsis-Like Illness Using Novel Heart Rate Analysis. *Pediatrics* 2001; 107: 97–104.
4. Griffin MP, O'Shea TM, Bissonette EA, et al. Abnormal Heart Rate Characteristics Preceding Neonatal Sepsis and Sepsis-Like Illness. *Pediatr Res* 2003; 53: 920–926.
5. Moorman JR, Carlo WA, Kattwinkel J, et al. Mortality Reduction by Heart Rate Characteristic Monitoring in Very Low Birth Weight Neonates: A Randomized Trial. *J Pediatr* 2011; 159: 900–906.e1.
6. Lake DE, Fairchild KD, Moorman JR. Complex signals bioinformatics: evaluation of heart rate characteristics monitoring as a novel risk marker for neonatal sepsis. *J Clin Monit Comput* 2013; 28: 329–339.
7. Sullivan BA, Grice SM, Lake DE, et al. Infection and Other Clinical Correlates of Abnormal Heart Rate Characteristics in Preterm Infants. *J Pediatr* 2014; 164: 775–780.
8. Kovatchev BP, Farhy LS, Cao H, et al. Sample Asymmetry Analysis of Heart Rate Characteristics with Application to Neonatal Sepsis and Systemic Inflammatory Response Syndrome. *Pediatr Res* 2003; 54: 892–898.
9. Lake DE, Richman JS, Griffin MP, et al. Sample entropy analysis of neonatal heart rate variability. *Am J Physiol Integr Comp Physiol* 2002; 283: R789–R797.
10. Griffin MP, O'Shea TM, Bissonette EA, et al. Abnormal Heart Rate Characteristics Are Associated with Neonatal Mortality. *Pediatr Res* 2004; 55: 782–788.

11. Griffin MP, Lake DE, Moorman JR. Heart rate characteristics and laboratory tests in neonatal sepsis. *Pediatrics* 2005; 115: 937–41.
12. Fairchild KD, Schelonka RL, Kaufman DA, et al. Septicemia mortality reduction in neonates in a heart rate characteristics monitoring trial. *Pediatr Res* 2013; 74: 570–575.
13. Sullivan BA, McClure C, Hicks J, et al. Early Heart Rate Characteristics Predict Death and Morbidities in Preterm Infants. *J Pediatr* 2016; 174: 57–62.
14. Fairchild KD, Sinkin RA, Davalian F, et al. Abnormal heart rate characteristics are associated with abnormal neuroimaging and outcomes in extremely low birth weight infants. *J Perinatol* 2014; 34: 375–379.
15. Ramsay JO, Silverman BW. *Functional Data Analysis*. 1st ed. New York: Springer-Verlag, 1997. DOI: 10.1007/978-1-4757-7107-7.
16. Jones MC, Rice JA. Displaying the Important Features of Large Collections of Similar Curves. *Am Stat* 1992; 46: 140–145.
17. Rice JA, Silverman BW. Estimating the Mean and Covariance Structure Nonparametrically When the Data are Curves. *J R Stat Soc Ser B* 1991; 53: 233–243.
18. de Boor C. *A Practical Guide to Splines*. New York, NY: Springer New York. Epub ahead of print 1978. DOI: 10.1007/978-1-4612-6333-3.
19. Ramsay JO, Hooker G, Graves S. *Functional Data Analysis with R and MATLAB*. Springer, 2009. Epub ahead of print 2009. DOI: 10.1007/978-0-387-98185-7.
20. Ramsay JO. Functional Data Analysis, http://www.psych.mcgill.ca/misc/fda/index.html (2013, accessed 19 August 2019).
21. Huang D, Wang C, Lai J. Locally Weighted Ensemble Clustering. *IEEE Trans Cybern* 2018; 48: 1460–1473.
22. Huang D, Wang C, Lai J. *MATLAB Source Code for Locally Weighted Ensemble Clustering*. 2018.
23. Mellinkoff SM, Higgins JR. The heart rate in malaria; a review of ninety cases. *Ann Intern Med* 1947; 27: 433.
24. Zimmet AM, Sullivan BA, Moorman JR, Lake DE and Ratcliffe SJ. *Trajectories of the heart rate characteristics index , a physiomarker of sepsis in premature infants, predict Neonatal ICU mortality*. University of Virginia Dataverse, V1 2020. DOI:10.18130/V3/2XENWC
