# Supplementary material for: Exploring Computational Techniques in Preprocessing Neonatal Physiological Signals for Detecting Adverse Outcomes: Scoping Review
Source: Interact J Med Res. 2024 Aug 20;13:e46946. doi: 10.2196/46946 (PMC11372324; doi:10.2196/46946)
Supplement: Multimedia Appendix 3 [file ijmr_v13i1e46946_app3.zip › Included Papers - Final/3266/Sullivan et al. - 2021 - Clinical and vital sign changes associated with la.pdf]

HHS Public Access
Author manuscript
J Neonatal Perinatal Med. Author manuscript; available in PMC 2021 November 17.

Published in final edited form as:
J Neonatal Perinatal Med. 2021 ; 14(4): 553–561. doi:10.3233/NPM-200578.

Clinical and vital sign changes associated with late-onset sepsis in very low birth weight infants at 3 NICUs

B.A. Sullivan[a,i,*], **V.P. Nagraj**[g,h], **K.L. Berry**[c,d], **N. Fleiss**[e], **A. Rambhia**[f], **R. Kumar**[a], **A. Wallman-Stokes**[e], **Z.A. Vesoulis**[f], **R. Sahni**[e], **S. Ratcliffe**[d,i], **D.E. Lake**[b,i], **J.R. Moorman**[b,i], **K.D. Fairchild**[a,i]

[a]Department of Pediatrics, Division of Neonatology, University of Virginia School of Medicine, Charlottesville, VA, USA

[b]Department of Medicine, Division of Cardiology, University of Virginia School of Medicine, Charlottesville, VA, USA

[c]University of Virginia School of Medicine, Charlottesville, VA, USA

[d]University of Virginia School of Public Health Sciences, Charlottesville, VA, USA

[e]Department of Pediatrics, Division of Neonatology, Columbia University, New York, NY, USA

[f]Department of Pediatrics, Division of Newborn Medicine, Washington University School of Medicine, St. Louis, MO, USA

[g]Department of Research Computing, University of Virginia School of Medicine, Charlottesville, VA, USA

[h]Signature Science, LLC, Charlottesville, Virginia, USA

[i]University of Virginia Center for Advanced Medical Analytics, Charlottesville, VA, USA

Abstract

BACKGROUND: In premature infants, clinical changes frequently occur due to sepsis or non-infectious conditions, and distinguishing between these is challenging. Baseline risk factors, vital signs, and clinical signs guide decisions to culture and start antibiotics. We sought to compare heart rate (HR) and oxygenation (SpO_2) patterns as well as baseline variables and clinical signs prompting sepsis work-ups ultimately determined to be late-onset sepsis (LOS) and sepsis ruled out (SRO).

*Address for correspondence: Brynne A. Sullivan, MD, University of Virginia, Pediatrics, PO Box 800386, Charlottesville, VA, 22908 0386, USA. Tel.: +1 434 924 5428; Fax: +1 434 924 2816; Bsa4m@virginia.edu.

Conflict of interest
D.E. Lake and J.R. Moorman own stock in Medical Predictive Sciences Corporation. J. R. Moorman is an officer and owns stock in Advanced Medical Predictive Devices, Diagnostics, and Displays.

Human Research Statement
We affirm that this research involving human subjects was conducted in accordance with the ethical standards of all applicable national and institutional committees and the World Medical Association's Helsinki Declaration.

Supplementary material
The supplementary table is available in the electronic version of this article: https://dx.doi.org/10.3233/NPM-200578.

Author Manuscript Author Manuscript Author Manuscript Author Manuscript

Author Manuscript Author Manuscript Author Manuscript Author Manuscript

METHODS: At three NICUs, we reviewed records of very low birth weight (VLBW) infants around their first sepsis work-up diagnosed as LOS or SRO. Clinical signs prompting the evaluation were determined from clinician documentation. HR-SpO_2 data, when available, were analyzed for mean, standard deviation, skewness, kurtosis, and cross-correlation. We used LASSO and logistic regression to assess variable importance and associations with LOS compared to SRO.

RESULTS: We analyzed sepsis work-ups in 408 infants (173 LOS, 235 SRO). Compared to infants with SRO, those with LOS were of lower GA and BW, and more likely to have a central catheter and mechanical ventilation. Clinical signs cited more often in LOS included hypotension, acidosis, abdominal distension, lethargy, oliguria, and abnormal CBC or CRP ($p <$ 0.05). HR-SpO_2 data were available in 266 events. Cross-correlation HR-SpO_2 before the event was associated with LOS after adjusting for GA, BW, and postnatal age. A model combining baseline, clinical and HR-SpO_2 variables had AUC 0.821.

CONCLUSION: In VLBW infants at 3-NICUs, we describe the baseline, clinical, and HR-SpO_2 variables associated with LOS versus SRO.

Keywords

Neonatal sepsis; infection; bacteremia; prematurity; vital sign analytics; predictive modeling

1. Introduction

Late-onset sepsis (LOS) continues to cause significant morbidity and mortality in very low birth weight (VLBW, < 1500 g) infants [1, 2]. Although LOS incidence has decreased with improvements in central line care, hand hygiene, and breast milk feeding [3-5], sepsis remains a major threat, and earlier detection and treatment are likely to improve outcomes. Diagnosis is complicated because the most common early signs of LOS overlap with normal premature infant physiology, such as apnea and feeding intolerance. More obvious signs of sepsis occur late in the course, at which point the systemic inflammatory response may irreversibly damage multiple organ systems [6, 7].

The risk of catastrophic outcomes with delay in sepsis treatment prompts conservative management, resulting in frequent negative work-ups diagnosed as “sepsis ruled out” (SRO) and exposure to antibiotics, which have adverse effects if overused [8-10]. Two important yet challenging goals are thus to improve the timeliness of antibiotics, with treatment starting early for true infection, and the precision of antibiotics, where treatment is avoided or quickly discontinued when there is no infection [11]. Our group previously developed a continuous heart rate characteristics (HRC) index (HeRO score) to alert clinicians of abnormal HR patterns of low variability and super-imposed decelerations, which may occur before overt clinical signs of sepsis [12-14]. Since most infants with abnormal HRC are not septic, clinicians must consider multiple other variables when making decisions about starting antibiotics versus taking a “wait and watch” approach [15, 16].

In this retrospective study of a large, multicenter cohort of VLBW infants, we sought to describe baseline risk factors and clinical signs as well as heart rate (HR) and oxygenation (SpO_2) patterns surrounding sepsis work-ups diagnosed as sepsis ruled in or sepsis ruled out.

Author Manuscript

We hypothesized that these three categories of data have additive value in distinguishing LOS from SRO. Our ultimate goal is not to develop a “calculator” for withholding antibiotics but instead to systematically evaluate a framework of variables for clinicians to consider evaluating VLBW NICU patients with non-specific changes in their clinical status. Such a framework might be useful when advanced physiologic monitoring brings the attention of the medical team to an infant, at which point multiple variables associated with sepsis risk must be considered.

2. Methods

2.1. Patient population and sepsis definitions

We reviewed electronic health records for demographic and clinical data for VLBW infants at three level-IV NICUs over a 3–5 year period during which continuous vital sign data from the bedside monitors were being stored: University of Virginia Children’s Hospital (UVA 2012–2016), Morgan Stanley Children’s Hospital of New York, Columbia University (CU 2013–2015) and St. Louis Children’s Hospital, Washington University School of Medicine (SLCH 2015–2017). The Institutional Review Boards of each institution approved the study. VLBW infants were included if they had no major congenital or chromosomal anomalies and had at least one blood culture drawn after three days of age for suspected sepsis. The first sepsis work-up with a positive blood culture and at least five days of antibiotics administered was designated as LOS. For infants with no LOS, the first sepsis work-up with negative cultures and less than five days of antibiotics given was designated as SRO. We did not include infants without LOS or SRO events who had only other infectious diagnoses, such as clinical sepsis (a negative blood culture treated with at least five days of antibiotics) or focal infection (pneumonia, necrotizing enterocolitis, urinary tract infections, other).

2.2. Clinical data collection

We collected demographic and perinatal variables for all infants from the medical record and NICU databases. Data were recorded in a REDCap database (Vanderbilt University, Nashville, TN) designed for this study. We recorded baseline risk factors from the day before the work-up, including the level of respiratory support, presence of a central venous catheter, and enteral feeding. We reviewed progress notes and recorded the clinical changes or signs that led to the decision to evaluate for sepsis. Clinical signs were listed in REDCap, and any number of signs could be checked off or added for each work-up. No objective values were required for a laboratory value, or vital sign change, or clinical sign to be recorded as abnormal. We grouped similar clinical signs for analysis: apnea, bradycardia, and desaturation spells were grouped as ABDs, low platelets, high or low white blood cells, or increased bands were grouped as abnormal complete blood count (CBC), and findings noted on chest or abdominal plain films to prompt a sepsis work-up, such as intestinal ileus or lung infiltrates, were grouped as abnormal radiographic findings.

2.3. HR and SpO_2 data collection and analysis

Infants at all three NICUs routinely have continuous heart rate (HR) from electrocardiogram and SpO_2 from pulse oximetry collected at 0.5 Hz from standard bedside monitors and archived, using the BedMaster system (Hillrom’s Medical Device Integration Solution,

Author Manuscript Author Manuscript Author Manuscript Author Manuscript

Chicago, Il; formerly Excel Medical, Jupiter, FL). SpO_2 was measured with the default averaging time of 8 seconds, using Masimo technology at UVA and CU and Nellcor technology at WU. HR and SpO_2 values of zero were removed.

The mean, standard deviation, skewness, and kurtosis of HR and SpO_2 were calculated in 10-minute windows and averaged hourly. The maximum cross-correlation of HR-SpO_2 was calculated in 10 min windows of signals normalized to have a mean of zero and standard deviation of one using the Matlab function XCORR with a lag window of 30 seconds and averaged hourly.

For bradycardia and desaturation event quantitation, we employed a previously published automated algorithm [18]. Bradycardia was defined as HR< 100 bpm for ≥four seconds, and desaturation as SpO_2 < 80% for ≥ 10 seconds. We joined events if the value rose above then fell back below the threshold in <4 seconds for bradycardia and <10 seconds for desaturation events. We quantified the number and duration of events, normalized to the amount of available data, and the means of the numbers of events per hour of data before and after the time of blood culture. The hourly averages were smoothed in a 12-hour moving average for visualizations of the trends for the five days before and two days after the time of blood culture. For hypothesis testing and regression modeling, HR-SpO_2 features were summarized as their averages in the 24 hours leading up to the culture.

2.4. Statistical analysis

The distributions of demographic and baseline characteristics, clinical signs, and HR-SpO_2 features were compared for infants with LOS to those with SRO using Fisher's exact test for categorical variables and the Wilcoxon rank-sum test for continuous variables. We used logistic regression to answer two questions. First, is an individual feature associated with the outcome on its own? Here, we used multivariable logistic regression to adjust for the influence of GA, BW, and postnatal age at the time of blood culture. Second, is an individual feature associated with the outcome when all other features are taken into account? For this, anticipating multicollinearity, we used logistic regression with the Least Absolute Shrinkage and Selection Operator (LASSO) procedure to shrink the coefficients of correlated or unimportant variables to zero [9, 20]. We built four models using LASSO logistic regression: one with only baseline risk variables, another with only clinical signs, another with only HR-SpO_2 features, and another with all variables combined. Only the 266 infants with HR-SpO_2 data available were included for this analysis. To assay the performances of the models, we calculated the AUC, with 95% CI estimated from bootstrapping. The percent correctly classified, sensitivity, and specificity were calculated based on thresholds chosen to maximize the Youden index [21]. AUCs were compared using DeLong's tests[22].

Statistical analyses were performed using R version 3.5.2 (R Foundation for Statistical Computing, Vienna, Austria).

Author Manuscript
Author Manuscript
Author Manuscript
Author Manuscript

3. Results

3.1. Characteristics and blood culture evaluations

We studied 882 VLBW infants admitted to the three centers who survived beyond three days of age, had no major chromosomal or congenital anomalies, and had recordings of sufficient duration and quality for analysis (426 UVA, 283 CU, 173 SLCH). Of these, 173 (20%) had at least one LOS event. The infective organisms were similar at the three centers, with coagulase-negative staphylococcus (CONS) species as the most common organism (55% of positive blood cultures) (Table 2).

Of the remaining 709 infants who never had LOS, 275 had one or more work-ups for suspected sepsis beyond day three, and we reviewed the first work-up diagnosed as SRO in 235 infants.

3.2. Baseline variables in LOS versus SRO

Table 1 shows the baseline demographics and clinical variables at the time of the work-up for the full cohort of 408 infants (173 LOS, 235 SRO) and for the subgroup of 266 infants with HR and SpO_2 data available for analysis. Infants with LOS were of lower GA and BW, and more likely to be on a ventilator and have a central catheter in place at the time of the work-up compared to those with SRO ($p < 0.05$). Clinical characteristics (Table 1) and clinical signs (Table S4) were similar for the entire cohort and for the sub-cohort with HR-SpO_2 data available.

3.3. Clinical signs prompting work-ups diagnosed as LOS versus SRO

Figure 1A shows the distribution of the eighteen most common clinical signs cited as prompting the blood culture in ≥5% of all work-ups, grouped by whether the diagnosis was LOS or SRO. Table S4 provides a list of all clinical signs. They are ordered top to bottom from most to least common in LOS cases. The most common clinical sign, cited in nearly half of all work-ups, was an increase in spells of apnea, bradycardia, or desaturation (ABD). ABD events were associated with positive and negative work-ups with approximately the same frequency (47% vs 48%). Likewise, the requirement for increased respiratory support was commonly cited as a reason for the sepsis work-up but just as likely to be associated with a confirmed infection compared to an SRO outcome (39% vs 34%).

An abnormal CBC value, elevated CRP, abdominal distention, acidosis, and hypotension were cited more often in work-ups diagnosed as LOS than those diagnosed as SRO ($p < 0.05$ by Fisher's test). These signs were also significant predictors in a multivariable analysis adjusted for BW and postnatal age. A subgroup analysis of LOS with CONS bacteremia cases excluded showed a similar distribution of clinical signs compared to all LOS cases, with slightly higher proportions of abnormal CBC, acidosis, and hypotension in cases with predominantly gram negative organisms (Fig. 1B).

3.4. HR and SpO_2 patterns in LOS versus SRO

Figure 2 shows HR and SpO_2 features plotted as the rolling 12-hour mean of the hourly averages for the five days before and the two days after blood cultures The HR and SpO_2

Author Manuscript Author Manuscript Author Manuscript Author Manuscript

trends were similar for LOS and SRO, and several features changed near the time of blood culture for both outcomes. While the mean cross-correlation HR-SpO_2, SD HR, and mean SpO_2 in the 24-hours leading up to culture differed individually between the groups, only the mean cross-correlation of HR-SpO_2 distinguished the groups in multivariable analysis including GA, BW, and postnatal age as predictors.

3.5. Variable importance and modeling

We developed and compared four multiple logistic regression models and used the LASSO procedure to identify important variables and deal with multicollinearity within each group of predictors. Separate models were built for baseline risk variables, clinical signs, HR-SpO_2 features, and all variables combined.

Table 3 lists the LASSO feature selection step results in order of variable importance, alongside summaries of the performance of logistic regression models. Baseline variables with non-zero coefficients included GA, BW, and the presence of a central catheter. In the model using the 18 clinical signs cited in at least 5% of all work-ups, eight variables had non-zero coefficients. Of the 11 HR-SpO_2 features modeled, four had non-zero coefficients. The HR-SpO_2 model had the lowest AUC (0.642, 95% CI 0.574–0.710). The model combining baseline, clinical signs, and HR-SpO_2 features had the highest point estimate for AUC (0.821). However, the 95% CI for AUC, sensitivity, specificity, and percent correctly classified were comparable to the clinical signs model (Table 3).

4. Discussion

In a three-NICU cohort, we studied the baseline risk factors and clinical signs prompting sepsis work-ups in VLBW infants, together with the HR and SpO_2 changes near the work-up to test the idea that infants with the diagnosis of sepsis could be distinguished from those for whom sepsis was ruled out. This is an important problem because clinical deterioration from suspected infection occurs often, but the empiric treatment of every event leads to unnecessary antibiotic exposure. Therefore, understanding how these clinical changes prompting work-ups fit in with baseline risk variables, physiologic data, and diagnosis might help guide decisions on when to obtain cultures and start antibiotics.

We found overlap in the clinical and vital sign presentations of infants with sepsis and those with sepsis ruled out. The most common signs prompting sepsis work-ups in our retrospective study, as well as the baseline variables associated with LOS, were similar across the three NICUs and generally consistent with those previously reported [23-26]. Baseline risk factors for sepsis of mechanical ventilation, central vascular catheter, and delayed enteral feeding have been described by others [23, 27, 28] and highlight that infants requiring higher-level support are more susceptible to developing a systemic infection. Clinical changes more often documented in sepsis included hypotension, acidosis, abnormal CBC and CRP values, lethargy, oliguria, abnormal radiographic findings, and abdominal distention. We did not analyze the severity of abnormalities nor use thresholds or cutoffs to classify signs as abnormal. Instead, we analyzed the documented changes prompting a work-up. This approach may be viewed as a strength and a weakness of the analysis, since

Author Manuscript Author Manuscript Author Manuscript Author Manuscript

not all thoughts are documented in the medical record and not all changes from baseline meet standard definitions of “abnormal”.

The most common sign overall, spells of apnea, bradycardia, or desaturation, was equally frequently cited in both groups. We emphasize this finding not to discourage using apnea as a reason to evaluate an infant for sepsis, but instead to describe its sensitive yet non-specific nature. Similarly, clinician documentation of an acute increase in respiratory or oxygen support as a reason for performing a sepsis evaluation was noted equally in cases of sepsis ruled in or out. This finding confirmed our initial expectations that these respiratory events are common in both septic and non-septic VLBW infants and often represent immature control of breathing and lung disease rather than infection [16, 29, 30].

Analysis of HR and SpO_2 patterns near the time of blood culture paralleled the clinical findings, in that most patterns of cardiorespiratory instability were similar for events diagnosed as sepsis and not sepsis. The cross-correlation of HR-SpO_2 increased near the time of blood culture in both groups of events, but more so for sepsis than sepsis ruled-out. Additional features might help identify late-onset sepsis, including HR kurtosis, HR SD, and mean SpO_2. These results add to our previous two-NICU report of an increase in HR-SpO_2 cross-correlation in the hours before overt clinical deterioration [31, 32]. Cross-correlation HR-SpO_2 reflects concurrent decelerations and desaturations that occur with apnea, which is a leading sign of sepsis in preterm infants due to prostaglandin and inflammatory cytokine effects on respiratory drive [31-34].

A previous study showed that the heart rate characteristics algorithm adds information on sepsis risk to a score based on clinical signs. [35] The clinical signs used for risk modeling in the cited study were chosen based on expert opinion and included feeding intolerance, apnea, increased respiratory support, an elevated immature to total neutrophil ratio, lethargy or hypotonia, temperature instability, and hyperglycemia. Not surprisingly, the important and common clinical signs identified by our chart reviews in the current study are found among those identified by clinical experience. While individual HR and SpO_2 features did not distinguish infants with and without sepsis at the time of blood culture in our analysis, modeling multiple features again added information to the clinical signs.

The retrospective design of our analysis limits its application. A review of the medical record does not capture the clinicians’ entire thought process in deciding to perform a sepsis work-up. A prospective survey of experienced clinicians might find a different array of clinical signs associated with sepsis, as would including every episode of evaluation for sepsis. We did not consider other common results of sepsis work-ups, such as clinical (culture-negative) sepsis and focal infections. The clinical signs prompting the work-up in these events often define the diagnosis. We excluded these events to compare and contrast events generally considered to be true positive and true negative events, but acknowledge that they contribute to the uncertainty about antibiotic therapy duration in NICU patients. Gestational age and birthweight were slightly lower for infants with LOS analyzed than for those with SRO analyzed. Our modeling approach adjusts for multiple possible confounders, but it is likely that more exist that we were not able to account for, such as concurrent medications, the fraction of inspired oxygen, or the severity of illness. Analysis of heart rate

characteristics shows that heart rate standard deviation increases with increasing gestational and postmenstrual age, but that infants at any age and maturity with sepsis have abnormal HRC compared to those without [12].

Our 3-center sepsis collaborative aims to develop, implement, and test early warning systems for sepsis in the NICU. Waiting for overt clinical signs of sepsis to emerge increases the risk of progression to shock, organ damage, and death. Continuous monitoring for abnormal cardiorespiratory vital sign patterns and displaying predictive analytics with or without an alarm might alert clinicians of the need for a careful clinical assessment. This assessment should include consideration of baseline variables, clinical changes, and vital sign changes known to be associated with sepsis. We advocate using this three-pronged approach to guide important decisions about starting and stopping antibiotics in VLBW infants who are susceptible both to sepsis and to adverse effects of antibiotic overuse.

5 Conclusion

In a large cohort of VLBW infants at three NICUs, we describe baseline risk factors, clinical signs prompting sepsis work-ups, and changes in HR and SpO_2 features in the days surrounding the blood culture. Clinical and cardiorespiratory changes in late-onset sepsis and sepsis ruled out were similar, high-lighting the difficulty in developing a clinical decision support tool for withholding antibiotics. Nonetheless, we speculate that careful consideration of variables associated with sepsis might decrease the excessive use of antibiotics in the absence of culture-proven infection.

Supplementary Material

Refer to Web version on PubMed Central for supplementary material.

Acknowledgments

Financial support

K23 HD097254-01 (PI: B. Sullivan), Translational Health Research Institute of Virginia (THRIV) (PI: B. Sullivan); R01 HD072071-05 (Co-PIs: K. Fairchild and J.R. Moorman); K23 NS111086 (PI: Z. Vesoulis).

References

[1]. Greenberg RG, Kandefer S, Do BT, Smith PB, Stoll BJ, Bell EF, et al. Late-onset sepsis in extremely premature infants: 2000-2011. Pediatr Infect Dis J. 2017;36(8):774–9. [PubMed: 28709162]

[2]. Stoll BJ, Hansen N. Infections in VLBW infants: Studies from the NICHD Neonatal Research Network. Semin Perinatol. 2003;27(4):293–301. [PubMed: 14510320]

[3]. Schulman J, Stricof R, Stevens TP, Horgan M, Gase K, Holzman IR, et al. Statewide NICU central-line-associated bloodstream infection rates decline after bundles and check-lists. Pediatrics. 2011;127(3):436–44. [PubMed: 21339265]

[4]. Shah J, Jefferies AL, Yoon EW, Lee SK, Shah PS, Canadian Neonatal Network. Risk factors and outcomes of late-onset bacterial sepsis in preterm neonates born at < 32 weeks' gestation. Am J Perinatol. 2015;32(7):675–82. [PubMed: 25486288]

[5]. Shane AL, Stoll BJ. Neonatal sepsis: Progress towards improved outcomes. J Infect. 2014;68(Suppl 1):S24–32. [PubMed: 24140138]

Author Manuscript Author Manuscript Author Manuscript Author Manuscript

Author Manuscript Author Manuscript Author Manuscript Author Manuscript

[6]. Dong Y, Speer CP. Late-onset neonatal sepsis: Recent developments. Arch Dis Child Fetal Neonatal Ed. 2015;100(3):F257–263. [PubMed: 25425653]

[7]. Goldberg O, Amitai N, Chodick G, Bromiker R, Scheuerman O, Ben-Zvi H, et al. Can we improve early identification of neonatal late-onset sepsis? A validated prediction model. J Perinatol. 2020;40(9):1315–22. [PubMed: 32203177]

[8]. Ting JY, Synnes A, Roberts A, Deshpandey A, Dow K, Yoon EW, et al. Association between antibiotic use and neonatal mortality and morbidities in very low-birth-weight infants without culture-proven sepsis or necrotizing enterocolitis. JAMA Pediatr. 2016;170(12):1181–7. [PubMed: 27775765]

[9]. Ting JY, Roberts A, Sherlock R, Ojah C, Cieslak Z, Dunn M, et al. Duration of initial empirical antibiotic therapy and outcomes in very low birth weight infants. Pediatrics. 2019;143(3):e20182286. [PubMed: 30819968]

[10]. Mukhopadhyay S, Puopolo KM. Antibiotic use and mortality among premature infants without confirmed infection-perpetrator or innocent bystander? JAMA Pediatr. 2016;170(12):1144–6. [PubMed: 27775753]

[11]. Lavoie PM, Popescu CR, Molyneux EM, Wynn JL, Chiume M, Keitel K, et al. Rethinking management of neonates at risk of sepsis. Lancet. 2019;394(10195):279–81. [PubMed: 31354128]

[12]. Griffin MP, O'Shea TM, Bissonette EA, Harrell FE, Lake DE, Moorman JR. Abnormal heart rate characteristics preceding neonatal sepsis and sepsis-like illness. Pediatr Res. 2003;53(6):920–6. [PubMed: 12646726]

[13]. Moorman JR, Carlo WA, Kattwinkel J, Schelonka RL, Porcelli PJ, Navarrete CT, et al. Mortality reduction by heart rate characteristic monitoring in very low birth weight neonates: a randomized trial. J Pediatr. 2011;159(6):900–906.e1. [PubMed: 21864846]

[14]. Fairchild KD, Schelonka RL, Kaufman DA, Carlo WA, Kattwinkel J, Porcelli PJ, et al. Septicemia mortality reduction in neonates in a heart rate characteristics monitoring trial. Pediatr Res. 2013;74(5):570–5. [PubMed: 23942558]

[15]. Griffin MP, Moorman JR. Toward the early diagnosis of neonatal sepsis and sepsis-like illness using novel heart rate analysis. Pediatrics. 2001;107(1):97–104. [PubMed: 11134441]

[16]. Sullivan BA, Grice SM, Lake DE, Moorman JR, Fairchild KD. Infection and other clinical correlates of abnormal heart rate characteristics in preterm infants. J Pediatr. 2014;164(4):775–80. [PubMed: 24412138]

[17]. Fairchild KD, Nagraj VP, Sullivan BA, Moorman JR, Lake DE. Oxygen desaturations in the early neonatal period predict development of bronchopulmonary dysplasia. Pediatr Res. 2019;85(7):987–93. [PubMed: 30374050]

[18]. Nagraj VP, Sinkin RA, Lake DE, Moorman JR, Fairchild KD. Recovery from bradycardia and desaturation events at 32 weeks corrected age and NICU length of stay: An indicator of physiologic resilience? Pediatr Res. 2019;86(5):622–7. [PubMed: 31272102]

[19]. Leisman DE, Harhay MO, Lederer DJ, Abramson M, Adjei AA, Bakker J, et al. Development and reporting of prediction models: Guidance for authors from editors of respiratory, sleep, and critical care journals. Crit Care Med. 2020;48(5):623–33. [PubMed: 32141923]

[20]. Tibshirani R Regression shrinkage and selection via the lasso. Journal of the Royal Statistical Society: Series B (Methodological). 1996;58(1):267–88.

[21]. Shapiro DE. The interpretation of diagnostic tests. Stat Methods Med Res. 1999;8(2):113–34. [PubMed: 10501649]

[22]. DeLong ER, DeLong DM, Clarke-Pearson DL. Comparing the areas under two or more correlated receiver operating characteristic curves: A nonparametric approach. Biometrics. 1988;44(3):837–45. [PubMed: 3203132]

[23]. Fanaroff AA, Korones SB, Wright LL, Verter J, Poland RL, Bauer CR, et al. Incidence, presenting features, risk factors and significance of late onset septicemia in very low birth weight infants. The National Institute of Child Health and Human Development Neonatal Research Network. Pediatr Infect Dis J. 1998;17(7):593–8. [PubMed: 9686724]

Author Manuscript Author Manuscript Author Manuscript Author Manuscript

[24]. Masino AJ, Harris MC, Forsyth D, Ostapenko S, Srinivasan L, Bonafide CP, et al. Machine learning models for early sepsis recognition in the neonatal intensive care unit using readily available electronic health record data. PLoS ONE. 2019;14(2):e0212665. [PubMed: 30794638]

[25]. Das A, Shukla S, Rahman N, Gunzler D, Abughali N. Clinical indicators of late-onset sepsis workup in very low-birth-weight infants in the neonatal intensive care unit. Am J Perinatol. 2016;33(9):856–60. [PubMed: 26960700]

[26]. Gonzalez BE, Mercado CK, Johnson L, Brodsky NL, Bhandari V. Early markers of late-onset sepsis in premature neonates: Clinical, hematological and cytokine profile. J Perinat Med. 2003;31(1):60–8. [PubMed: 12661146]

[27]. Stoll BJ, Hansen N, Fanaroff AA, Wright LL, Carlo WA, Ehrenkranz RA, et al. Late-onset sepsis in very low birth weight neonates: The experience of the NICHD Neonatal Research Network. Pediatrics. 2002;110(2 Pt 1):285–91. [PubMed: 12165580]

[28]. Jiang S, Yang C, Yang C, Yan W, Shah V, Shah PS, et al. Epidemiology and microbiology of late-onset sepsis among preterm infants in China, 2015-2018: A cohort study. Int J Infect Dis. 2020;96:1–9. [PubMed: 32209419]

[29]. Dennery PA, Di Fiore JM, Ambalavanan N, Bancalari E, Carroll JL, Claure N, et al. Pre-Vent: the prematurity-related ventilatory control study. Pediatr Res. 2019;85(6):769–76. [PubMed: 30733614]

[30]. Fairchild K, Mohr M, Paget-Brown A, Tabacaru C, Lake D, Delos J, et al. Clinical associations of immature breathing in preterm infants: part 1-central apnea. Pediatr Res. 2016;80(1):21–7. [PubMed: 26959485]

[31]. Fairchild KD, Lake DE, Kattwinkel J, Moorman JR, Bateman DA, Grieve PG, et al. Vital signs and their cross-correlation in sepsis and NEC: a study of 1,065 very-low-birth-weight infants in two NICUs. Pediatr Res. 2017;81(2):315–21. [PubMed: 28001143]

[32]. Fairchild KD, Lake DE. Cross-correlation of heart rate and oxygen saturation in very low birthweight infants: Association with apnea and adverse events. Am J Perinatol. 2018;35(5):463–9. [PubMed: 29141263]

[33]. Siljehav V, Hofstetter AM, Leifsdottir K, Herlenius E. Prostaglandin E2 Mediates Cardiorespiratory Disturbances during Infection in Neonates. J Pediatr. 2015;167(6):1207–1213.e3. [PubMed: 26434370]

[34]. Balan KV, Kc P, Hoxha Z, Mayer CA, Wilson CG, Martin RJ. Vagal afferents modulate cytokine-mediated respiratory control at the neonatal medulla oblongata. Respir Physiol Neurobiol. 2011;178(3):458–64. [PubMed: 21397055]

[35]. Griffin MP, Lake DE, O'Shea TM, Moorman JR. Heart rate characteristics and clinical signs in neonatal sepsis. Pediatr Res. 2007;61(2):222–7. [PubMed: 17237726]

Author Manuscript
Author Manuscript
Author Manuscript
Author Manuscript

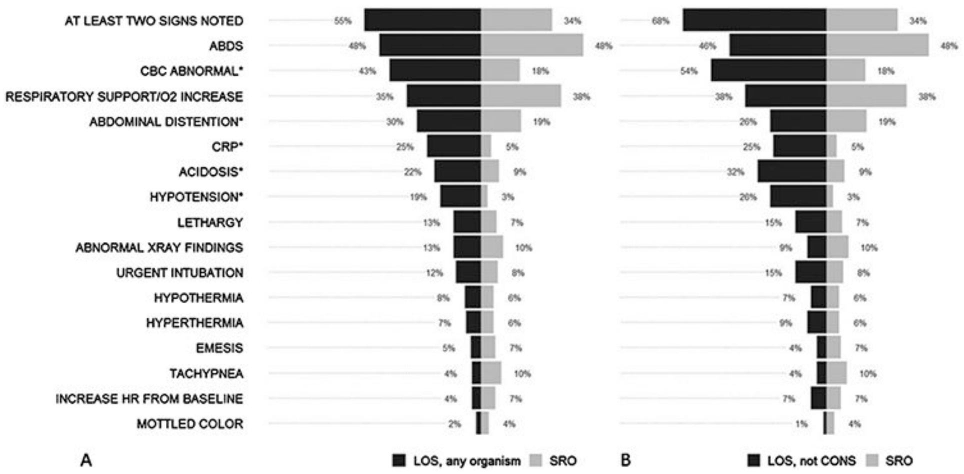

Fig. 1.

A. Frequency of clinical signs prompting sepsis work-ups that were ultimately diagnosed as late-onset sepsis (LOS, black bars) due to any organism compared to sepsis ruled-out (SRO, gray bars). Signs listed in order of decreasing frequency in LOS events. Asterisks indicate signs cited more often in LOS or SRO by Fisher's test (*$p < 0.05$). B. Clinical signs prompting sepsis work-ups diagnosed as LOS, excluding cases due to CONS, compared to SRO. Statistical comparisons are not shown but differ by one clinical sign; abdominal distention was no longer significant.

Author Manuscript Author Manuscript Author Manuscript Author Manuscript

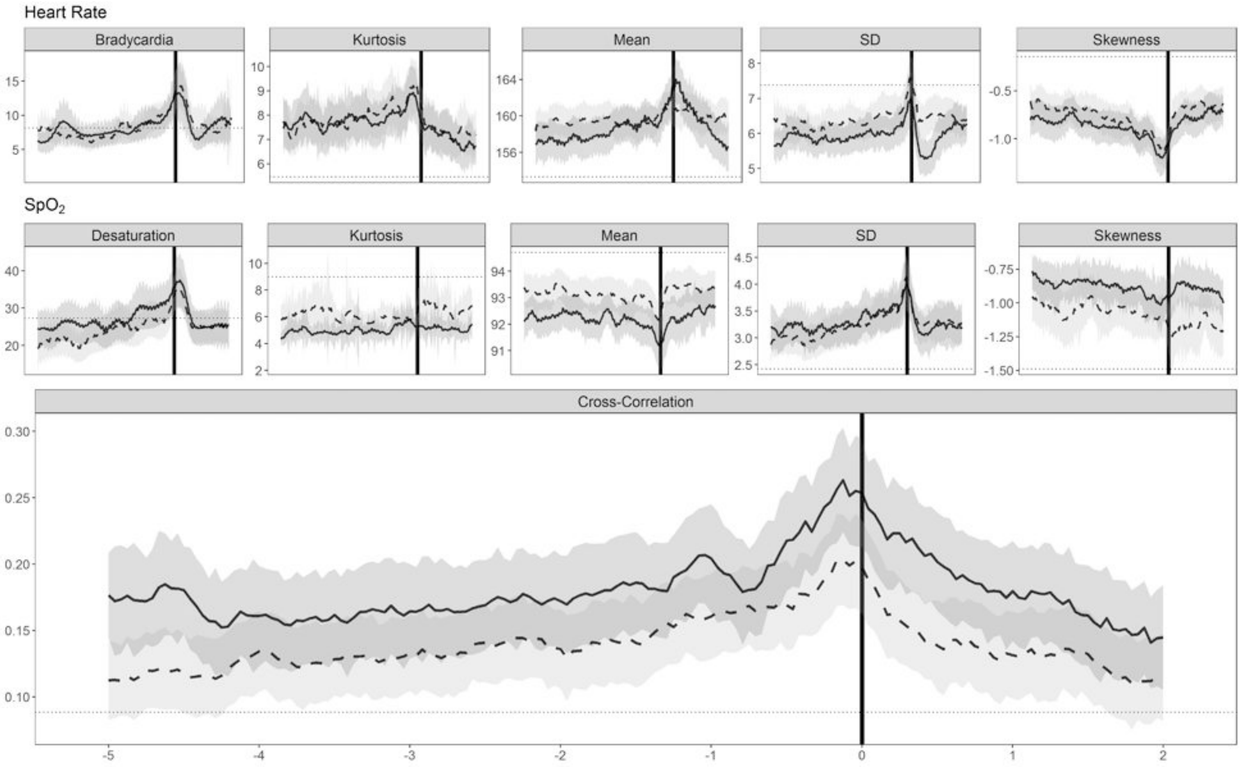

Fig. 2.

HR and SpO_2 features plotted as the 12hr moving average from five days before to two days after the time of blood culture, grouped by diagnosis (LOS, solid line; SRO, dashed line). Gray shaded areas represent the 95% confidence intervals and a horizontal dotted line is drawn on each panel at the mean for all UVA VLBW infants at all available times for that feature.

Author Manuscript Author Manuscript Author Manuscript Author Manuscript

Table 1

Characteristics of infants with LOS and SRO for the full cohort and for those with HR and SpO_2 data available

	**Clinical signs analysis**		**HR, SpO_2 analysis**	
**Baseline Clinical Variables**	**LOS (*n* = 173)**	**SRO (*n* = 235)**	**LOS (*n* = 118)**	**SRO (*n* = 148)**
Gestational age (weeks), median (IQR)	25 (24–27)	27 (25–28)	25 (24–27)	26 (25–28)
Birthweight (grams), median (IQR)	720 (610–895)	900 (715–1090)	760 (630–903)	875 (690–1080)
Male, n (%)	82 (47%)	114 (49%)	59 (50%)	61 (41%)
Age at workup	19 (10–32)	19 (9–38)	18 (9–32)	16 (9–27)
Race/ethnicity n (%)				
White or Caucasian	94 (54%)	130 (55%)	68 (58%)	87 (59%)
Black or African American	51 (29%)	71 (30%)	28 (24%)	40 (27%)
Hispanic or Latino	25 (14%)	27 (11%)	20 (17%)	16 (11%)
Asian	2 (1%)	2 (1%)	1 (1%)	1 (1%)
Unknown or not Reported	1 (<1%)	5 (2%)	1 (1%)	4 (2%)
5 min Apgar, Median (IQR)	6 (4 – 8)	6 (4 – 8)	6 (4 – 8)	7 (4 – 8)
Delivered by C-section, n (%)	115 (67%)	163 (69%)	73 (62%)	97 (66%)
Outborn, n (%)	32 (19%)	51 (22%)	20 (17%)	35 (24%)
**Clinical variables at time of workup**				
Central line present, n (%)	126 (73%)	110 (47%)	81 (69%)	77 (52%)
Mechanical ventilation, n (%)	87 (50%)	72 (31%)	50 (42%)	44 (30%)
Full enteral feeds, n (%)	30 (17%)	77 (33%)	22 (18%)	55 (37%)
Any breast milk feeds, n (%)	96 (56%)	140 (60%)	61 (57%)	98 (66%)

Author Manuscript

Author Manuscript

Author Manuscript

Author Manuscript

Table 2

Frequency and percent of organisms isolated in positive blood cultures

**Blood Culture Organism**	**N (%)**
S. epi or CONS	95(55)
S. aureus	24 (14)
E. coli	16 (9)
Klebsiella sp.	10 (6)
Enterococcus sp.	9 (5)
Group B Streptococcus	7 (4)
Serratia sp.	4 (2)
Enterobacter sp.	3 (2)
Other organism	4 (2)
Pseudomonas sp.	2 (1)

Author Manuscript
Author Manuscript
Author Manuscript
Author Manuscript

Table 3

Performance and variable importance of models including variables from one category of data and all categories combined using LASSO logistic regression

Model	Variables with non-zero coefficients	AUC	% Correctly classified	Sensitivity	Specificity
Baseline risk factors	Postnatal age<br>Gestational age<br>Sex<br>Central catheter<br>Outborn<br>Feeding status<br>Birthweight	0.710 (0.648 –0.773)	68%	75%	59%
Clinical signs	CRP elevated<br>Hypotension<br>Abdominal Distension<br>Acidosis<br>CBC abnormal<br>Dyspnea<br>Abnormal X-ray	0.751 (0.692 –0.810)	74%	90%	53%
HR-SpO_2 features	XCORR HR-SpO_2<br>SD HR<br>Mean SpO_2 Kurtosis HR	0.642 (0.574 –0.710)	64%	83%	42%
Combined[1]	Baseline+Clinical+[2]HR-SpO_2	0.821 (0.769 – 0.872)	76%	82%	70%

Abbreviations: AUC= area under the receiver operator characteristic curves, CBC = complete blood count, CRP = C-reactive protein, XCORR= cross-correlation, HR= heart rate, SpO_2 = oxygen saturation, SD = standard deviation.

[1]AUC of combined model is significantly different than clinical signs model ($p = 0.002$)

[2]Includes all component model features except BW, feed vol, Mean SpO_2.
