# Supplementary material for: Exploring Computational Techniques in Preprocessing Neonatal Physiological Signals for Detecting Adverse Outcomes: Scoping Review
Source: Interact J Med Res. 2024 Aug 20;13:e46946. doi: 10.2196/46946 (PMC11372324; doi:10.2196/46946)
Supplement: Multimedia Appendix 3 [file ijmr_v13i1e46946_app3.zip › Included Papers - Final/3311/Zuzarte et al. - 2021 - Predicting apneic events in preterm infants using .pdf]

Computer Methods and Programs in Biomedicine 209 (2021) 106321

Contents lists available at ScienceDirect

Computer Methods and Programs in Biomedicine

journal homepage: www.elsevier.com/locate/cmpb

Predicting apneic events in preterm infants using cardio-respiratory and movement features

Ian Zuzarte[a], Dagmar Sternad[b], David Paydarfar[c,d,*]

[a] Department of Bioengineering, Northeastern University, Boston, MA 02115, United States
[b] Departments of Biology, Electrical and Computer Engineering & Physics, Northeastern University, Boston, MA 02115, United States
[c] Department of Neurology, Dell Medical School, Austin, TX 78712, United States
[d] Oden Institute for Computational Sciences and Engineering, The University of Texas at Austin, Austin, TX 78712, United States

ARTICLE INFO

Article history:
Received 12 January 2021
Accepted 25 July 2021

ABSTRACT

Background and objective: Preterm neonates are prone to episodes of apnea, bradycardia and hypoxia (ABH) that can lead to neurological morbidities or even death. There is broad interest in developing methods for real-time prediction of ABH events to inform interventions that prevent or reduce their incidence and severity. Using advances in machine learning methods, this study develops an algorithm to predict ABH events.

Methods: Following previous studies showing that respiratory instabilities are closely associated with bouts of movement, we present a modeling framework that can predict ABH events using both movement and cardio-respiratory features derived from routine clinical recordings. In 10 preterm infants, movement onsets and durations were estimated with a wavelet-based algorithm that quantified artifactual distortions of the photoplethysmogram signal. For prediction, cardio-respiratory features were created from time-delayed correlations of inter-beat and inter-breath intervals with past values; movement features were derived from time-delayed correlations with inter-breath intervals. Gaussian Mixture Models and Logistic Regression were used to develop predictive models of apneic events. Performance of the models was evaluated with ROC curves.

Results: Performance of the prediction framework (mean AUC) was 0.77 $\pm$ 0.04 for 66 ABH events on training data from 7 infants. When grouped by the severity of the associated bradycardia during the ABH event, the framework was able to predict 83% and 75% of the most severe episodes in the 7-infant training set and 3-infant test set, respectively. Notably, inclusion of movement features significantly improved the predictions compared with modeling with only cardio-respiratory signals.

Conclusions: Our findings suggest that recordings of movement provide important information for predicting ABH events in preterm infants, and can inform preemptive interventions designed to reduce the incidence and severity of ABH events.

© 2021 Elsevier B.V. All rights reserved.

1. Introduction

Apnea of prematurity is defined as a respiratory pause for at least 20s or a pause shorter than 20s when accompanied by bradycardia (<100 bpm), cyanosis, pallor and and/or marked hypotonia [1]. In practice, apneic events that fall short of these criteria may also put the premature infant at risk. Preterm infants, born at a gestational age of <37 weeks, are prone to apneic episodes due to numerous factors. Amongst them is their low birth weight [2] and the immaturity of their organs, including lungs, the respiratory controller and other structures in the brain [3]. This results in pathophysiological mechanisms such as increased inhibitory reflexes of breathing and hypoxia depressed ventilation [4,5]. Maturation with increasing post-menstrual age is associated with a reduction in the incidence of apneic events [6].

Apneas are classified into central, obstructive and mixed types based on respiratory effort and airflow [7]. Central apneas arise due to damage or immaturity of the brainstem which is responsible for respiratory rhythm generation. This results in cessation of breathing due to lack of movement of the respiratory muscles in chest and abdomen. Obstructive apneas occur due to airflow obstruction at the pharyngeal level [8,9]. The cause of obstruction can be due to a primary mechanical event, e.g., head and neck positional changes, or neural event, e.g., loss of neural drive

* Corresponding author at: 1601 Trinity Street Building B 5.704, Austin, Texas, 78712, United States.
E-mail address: david.paydarfar@austin.utexas.edu (D. Paydarfar).

https://doi.org/10.1016/j.cmpb.2021.106321
0169-2607/© 2021 Elsevier B.V. All rights reserved.

to the pharyngeal and laryngeal dilator muscles. In mixed apneas the obstruction of the airflow results in a central apneic pause, or vice versa. Mixed apneas are the most prevalent type in preterm infants constituting more than 50% of all apneas [3,10]. Apneic episodes and the associated bradycardias and hypoxias (termed “ABH events”) cause significant reduction in cerebral oxygenation, blood flow velocity, and tissue perfusion when bradycardias fall below 80 bpm [11,12]. Such events put these infants at an increased risk for brain injury and poor neurodevelopmental outcomes. Regardless of the specific type of apnea, prolonged and repetitive apneas are associated with neurological morbidity and mortality.

ABH events are treated with methylxanthine drugs, such as caffeine, theophylline and aminophylline that stimulate the central nervous system [13,14]. Premature infants are continuously monitored in neonatal intensive care units (NICU). Nurses and physicians respond to apneic, bradycardic and hypoxic alarms by manual stimulation of the infant or, in severe cases, by placing the infant on a ventilator. Although manual stimulation is effective in reducing ABH [15], its implementation is limited by risk of infection due to increased manual contact by staff. NICU staff are often faced with simultaneous apnea alarms across the unit, and there is concern that manual stimulation can transmit infections by contamination across infants due to inadequate time for staff to implement sanitation procedures.

Non-pharmacological treatment based on using a mattress that applies stochastic vibrotactile stimulation (SVS) has been shown to reduce the incidences of ABH events [16,17]. However, the mattress needs to be actuated prior to onset of the event. Hence, this method is only effective in preventing, but not attenuating or aborting the episodes. Mattress SVS can be automated in a closed-loop system, in which SVS is actuated during periods of high risk for apneas [18]. Therefore, it would be of high clinical significance if apneas could be predicted before they occurred, in order to actuate the SVS intervention. Another important benefit of predicting ABH events would be to provide nursing staff more time to perform required sanitation procedures such as hand washing and wearing isolation gowns, to prevent cross-contamination between infants.

Many studies have developed models to detect apnea [19-23], but relatively little has been done in the prediction of apneic events. Several methods used the prior knowledge of the distribution of the RR-intervals of the ECG to predict bradycardia based on hierarchical classification methods [24], point-process theory [25] and decision trees [26]. Prediction based on nonparametric kernel-based probability density function has also been reported [27]. Williamson and colleagues have proposed prediction of apnea based on cardio-respiratory signals using Quadratic Classifiers [28]. A different study predicted apneic episodes using physiological, demographical and medical information via support vector machines and random forests [29] and deep neural networks [30]. All these studies only used cardio-respiratory events for the prediction of bradycardias and apneas. However, there is evidence that bodily movements are correlated with apnea occurrences [31-33]. Our previous work has provided quantitative evidence that respiratory variability and apneas are closely associated with movements [34]. The aim of this study extends from this result and uses features from movement along with cardio-respiratory intervals to predict ABH events.

A previous preliminary study of our group used movement features inferred from Fourier transform of the PPG signal to predict apneas in 6 subjects, using a Gaussian Mixture Model (GMM) [35]. In the study reported here, we extend elements of this previous approach by: 1) including a larger number of subjects; 2) analysing the onsets and durations of movement with greater temporal resolution using a wavelet-based algorithm of the PPG signal [36]; 3) demonstrating the benefits of including movement-based features in a machine learning framework for prediction of ABH events. Briefly, cardiorespiratory features for predictions were created based on time-delayed correlations of log-transformed interbeat and interbreath intervals with past values. Movement features were obtained by considering its influence on inter-breath intervals using time-delayed correlations. The classification approach combined unsupervised (GMMs) and supervised (Logistic Regression) machine learning methods. Our study also demonstrated the use of stratified k-fold cross-validation to resolve the imbalance between different classes representing physiological states relative to ABH events. Performance evaluation based on ROC curves was used for evaluating model performance. This framework was able to predict 75% of the most severe episodes. Notably, inclusion of movement features significantly improved the predictions compared with modeling only cardio-respiratory signals. Our findings suggest that recordings of movement provide important predictive information for ABH events in preterm infants, and can inform preemptive interventions designed to reduce the incidence and severity of ABH events.

2. Methods

2.1. Subjects and ethical approval

The study protocol was approved by the University of Massachusetts’s Medical School Institutional Review Board for Human Subjects. Ten preterm infants were studied at the University of Massachusetts Memorial Healthcare NICU. All infants were spontaneously breathing room air. Infants with hydrocephalus, intraventricular hemorrhage higher than grade 2, congenital defects or bronchopulmonary disease were excluded. Eligible infants were identified to the investigators by the attending neonatologist. Written informed consent was obtained from the infant’s mother or legal guardian prior to enrollment in the study.

2.2. Data acquisition and pre-processing

All subjects were studied in their NICU incubator with a bedside monitor (IntelliVue MP70, Philips Medical Systems, Andover, MA), which displayed electrocardiogram (ECG) and photoplethysmogram (PPG) waveforms along with numeric vitals such as blood oxygen saturation ($Sp0_2$%), heart rate and respiration rate. ECG was recorded using electrodes placed on the infant’s chest in a 3-lead configuration. The PPG signal was acquired from the infant’s hand or foot using a pulse oximeter probe (Masimo SET LNCS Neo, Masimo, Irvine, CA). Abdominal respiratory movements (pneumogram) were recorded using respiratory inductance plethysmography (Somnostar PT, Viasys Healthcare, Yorbalinda, CA) via bands placed around the abdomen. A video camera, placed in one corner of the incubator, continuously monitored the infant (resolution: 320 × 240 pixels, Edmund Optics, Barrington, NJ). Data were recorded using the Vuelogger^{TM} Patient Monitoring System (Wyss Institute, Boston, MA), which retrieved physiological information from the bedside monitors and time-synced them with signals from the pneumography system and the video. The signals were sampled at the following rates: ECG: 500Hz; pneumogram: 50Hz; PPG: 125Hz; video: 10fps. The data were streamed to the Vuelogger’s hard disk at the bedside and were subsequently exported for analysis.

During movement the PPG signal showed distortions, i.e., artifactual events in the PPG signal, that are distinct from the pulsatile signal related to perfusion. A wavelet-based algorithm developed previously was used to estimate gross body movements by identifying the onset and offset of these disruptions [36]. A binary marker sampled at 25Hz was thus obtained indicating the presence or absence of movement. The movement marker together

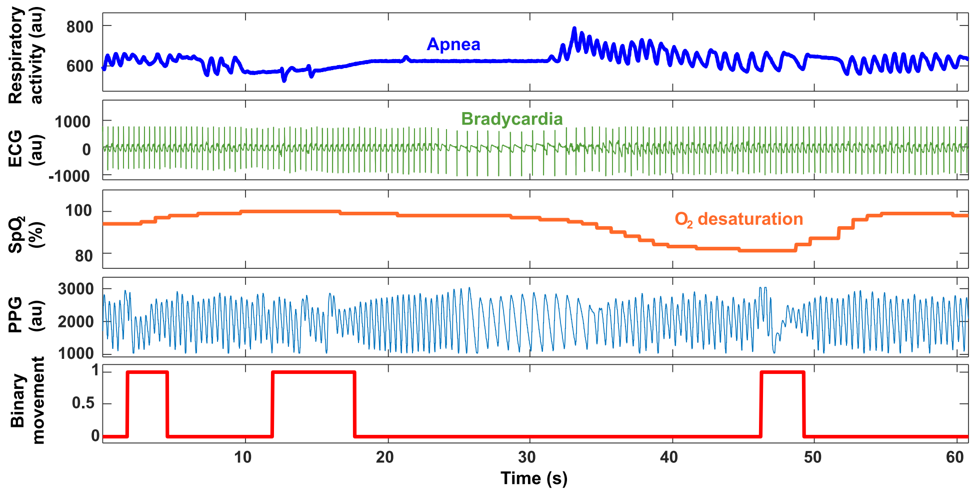

Fig. 1. Raw signals of respiratory activity, ECG, SPO_2 and PPG. Binary markers of movements were derived from the PPG signal. Apneas and the associated bradycardias and oxygen desaturation are highlighted.

Table 1
Subject and study characteristics. GA: Gestional age of the infant at birth, PMA: Post-menstrual age of the infant at the time of study.

Sub ID	GA (weeks)	PMA (weeks)	Study weight (g)	Total study time (hr)	Excluded time(hr)	Total analyzable time(hr)	Inter-apnea frames	Pre-apnea frames
1	30.57	30.71	1755	43.54	22.16	21.38	161	10
2	29.43	30.14	843	44.15	8.15	36.00	283	5
3	31.29	32.29	1674	46.78	19.15	27.63	209	12
4	28.86	30.14	1140	48.00	16.63	31.38	245	6
5	28.57	30.14	1110	19.40	6.52	12.88	99	4
6	30.29	30.57	1230	70.16	26.79	43.38	328	19
7	29.57	31.57	1480	23.87	12.75	11.13	79	10
8	27.29	29.43	1220	43.24	25.54	17.69	167	26
9	30.14	30.71	1710	40.92	22.13	18.79	193	12
10	33.86	34.29	1900	41.42	20.33	21.08	227	3

with raw cardio-respiratory signals is illustrated in Fig. 1. R-R intervals (RRI) were extracted from the ECG signals using a modified Pan–Tompkins algorithm. Inter-breath intervals (IBIs) were extracted from the pneumogram using automated peak-detection software (LabChart 7, ADI Instruments, Colorado Springs, CO).

Data during periods of nurse/caregiver interventions and intervals that were contaminated by technical interruptions, such as disconnection of sensors, were excluded from analyses. The total excluded times for each subject and the study durations are listed in Table 1. The remaining RRI and IBI values were first interpolated at 10Hz. The feature engineering methods described in Section 2.4 are based on correlations between the interval time series; normality of their distribution was assumed. Since the IBI and RRI signals followed a log-normal distribution, they were log-transformed to make the intervals approximately normal (Fig. 2).

2.3. Problem definition

Apnea of prematurity has been defined as a pause in breathing for at least 20s or between 10-20s, if accompanied by bradycardia, oxygen desaturation or cyanosis in infants [1]. Bradycardia was defined when the heart rate dropped below 100bpm and persisted for at least 3 heart beats. Oxygen desaturation was defined as $SpO_2 < 88\%$. This definition was used to automatically label apneic/ABH events in our datasets. The onset of apneic pauses $>$10s was considered as the last respiratory peak before the pause. For events where bradycardia and desaturation occurred simultaneously, the onset of apnea was defined at 20s prior to their first point of co-occurrence, or the onset of the associated apneic pause, whichever occurred earlier. Apneic offsets were defined as the end of the apneic pause or the time point where both heart rate and SpO_2 were above their thresholds, whichever occurred later. Fig. 1 illustrates an example of an apnea along with the associated bradycardia and oxygen desaturation.

The goal of apnea prediction was motivated by preliminary work by our group [28,35], based on an analytical framework that segmented data into apnea, pre-apnea and inter-apnea blocks. Apneas could be predicted by an algorithm that distinguished between pre-apnea (segments of data before an apneic onset) and inter-apnea periods (segments of data before the pre-apnea period). Detection of a pre-apnea period would therefore constitute the prediction of an upcoming apnea. Pre-apnea periods were chosen to be of length 7.5min immediately preceding an apneic onset. Post-apnea periods were chosen as the 7.5min-segment from the end of the all apnea labels. Since apneas tend to be clustered in time, predicting the first apnea in a cluster is of great clinical importance. This is critical since features obtained from the RRI and IBI time series change significantly after an apneic onset. If another apnea occurred within 7.5min of the first apnea onset, they were assigned to the same cluster without any pre-apnea segments. Inter-apnea periods were considered as all the remaining time points in the dataset. They were further divided into smaller non-overlapping segments of 7.5min to match the length of the pre-apnea segments. Apnea and post-apnea periods were not used in generating the prediction models.

A schematic representation of the apnea, pre-apnea, inter-apnea and apnea blocks obtained from 6 hours of data is shown in Fig. 3. Single apneic events are in black, whereas the yellow blocks constitute an apneic block which included the first apnea and the 7.5min following its offset. The 3rd and 5th apneic blocks are longer in duration since they had new apneic episodes occurring within 7.5min of the offset of their preceding apneic events. The

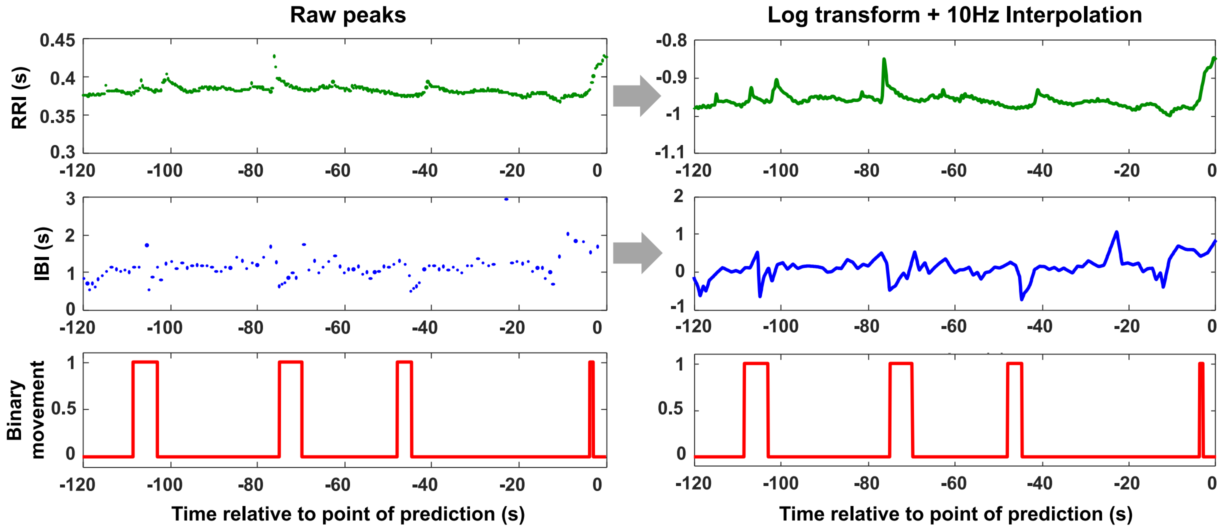

Fig. 2. Signals used for feature engineering. Left: Time-series of RR intervals, inter-breath intervals (IBI) and movement. Right: Log-transformed and interpolated versions of RR intervals and IBIs.

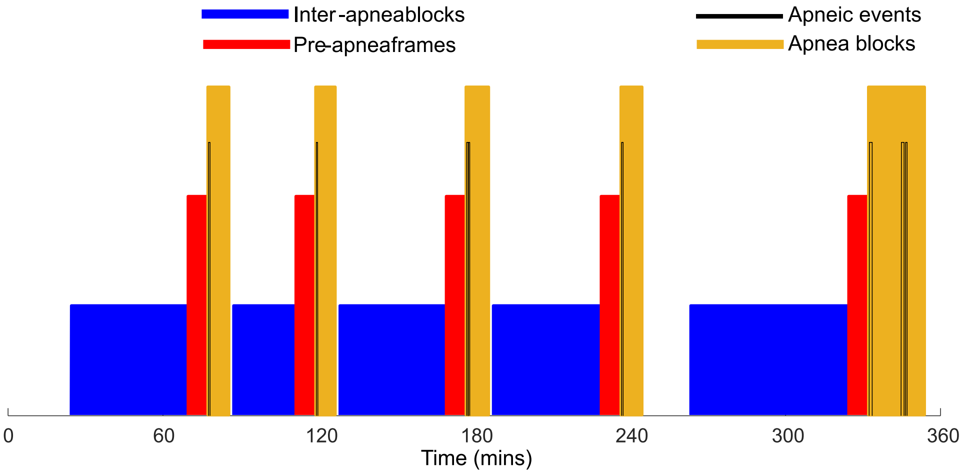

Fig. 3. Labeling of apnea, pre-apnea and inter-apnea blocks.

7.5min-period immediately preceding the apneic blocks are pre-apnea blocks (or pre-apnea frames). The blocks in blue are inter-apnea blocks of varying duration which were further divided into smaller non-overlapping frames of 7.5min to match with the pre-apnea frames. All other sections of unlabeled data were excluded as they were confounded by feedings or nursing assessments. The total number of inter-apnea and pre-apnea frames from each subject is listed in Table 1.

2.4. Feature engineering

Many studies have suggested the existence of a bidirectional cardio-respiratory coupling in preterm infants where the cardiovascular and the respiratory rhythms are regulated synergistically to ensure adequate ventilation [37,38]. The physiological basis includes delays from a host of latencies between output variables and feedback signals to sensors. An example is the time delayed effect on respiratory and heart rate due to a brief change in ventilation. These delays reflect a series of complex signaling processes, for example circulatory transport and chemoreceptor signaling of carbon dioxide and hydrogen ion. Higher coordination between two time-series has been demonstrated when small delays between the signals were considered [39].

In order to exploit this physiological finding, a time-delayed correlation analysis was performed to capture effects from within as well as across a time-delayed channel. This approach is motivated by the observation that auto- and cross-correlations of time-delayed signals can reveal hidden parameters in the stochastic dynamical system that generates the signals. These features may differ between the inter-apnea and pre-apnea segments. The time-delayed correlation procedure is illustrated in Fig. 4. The length of a data block and the time delay are parameters to generate the features that should be predefined. Since these were unknown, 6 different block lengths were used (10s, 20s, 30s, 40s, 50s and 60s). This implies that at every time point, 2min of past data were used for the computation of features, 60s from the length of current block, and 60s from the time-delayed block used for cross-correlation. This effectively translated each 7.5 min pre-apnea segment into a 5.5min feature matrix. For each block length, the zero-lag correlation of the current block with past data was obtained. The time delay between current and past blocks was varied between 0s and 59s at 1s increments, thus generating 330 time-observations from the 5.5min (330 s) feature matrix . For the RRI time series, correlation with past values within the channel resulted in a total of 360 features (60 features obtained from each of the 6 block lengths). The IBI time-series yielded another 360

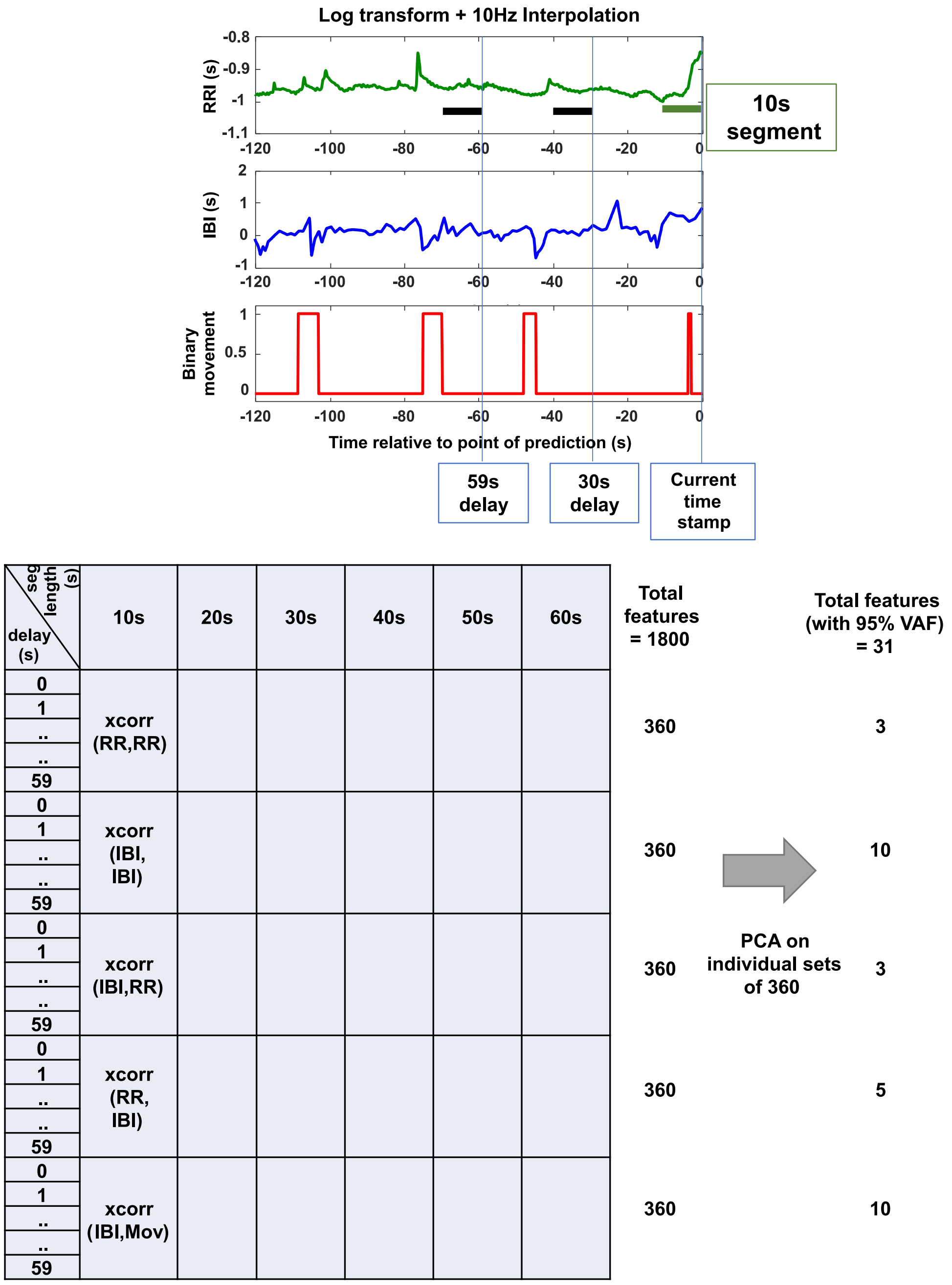

Fig. 4. Schematic representation for feature engineering.

features. In addition, 360 features were obtained from the cross-channel correlations from previous RRI values to IBI and 360 features from previous IBI values to RRI values.

In previous work, we showed that apneic events were associated with longer movement durations occurring before the onset compared to after the onset of apneas. Additionally, movement significantly increased respiratory variability [34]. This information was used by taking zero-lag correlations between IBI and time-delayed previous movement, thus providing another 360 features. Presence or absence of movement was captured in a binary time-series. Therefore, at each of the 330 observations, a total of 1800 features were obtained, which were highly correlated within each set of 360 that they were obtained from. Principal component analysis (PCA) was then used on each set of 360 features to convert them into statistically independent, uncorrelated and orthogonal features (principal components). The number of principal components accounted for at least 95% of the dataset variance. This resulted in a total of 31 features: 3 from the within RRI correlations; 10 from the within IBI correlations; 3 from cross-correlations between previous RRI and IBI; 5 cross-correlations between previous IBI and RRI; and 10 from cross-correlations between past movement and IBI. The features were standardized to have zero mean and unit variance before conducting the PCA. Features were generated from past data at 10s intervals.

2.5. Training and testing data

The subjects' data were divided into two groups: a training data set (comprising 7 subjects) and a testing data set (comprising 3 subjects). Each subject in the training data set was sep-

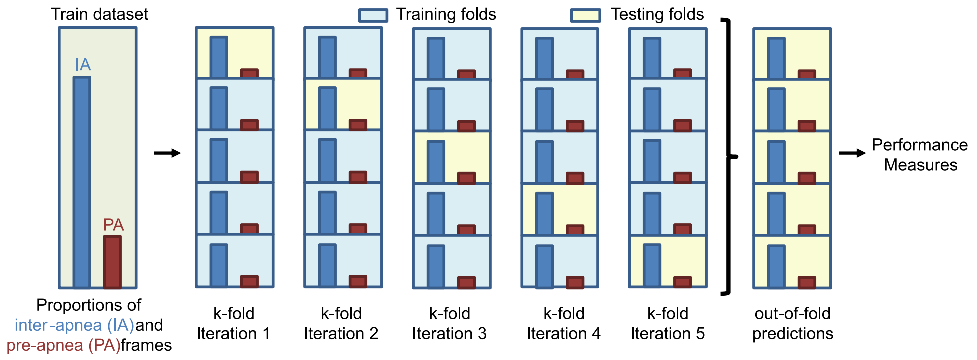

Fig. 5. Example of stratified k-fold cross-validation.

arately used for cross-validation to evaluate the model's performance. Due to the imbalance between the number of pre-apnea and inter-apnea segments, stratified k-fold cross-validation was used [40] (Fig. 5). This maintained the proportion of pre-apnea and inter-apnea frames in all folds. The number of folds was selected as the total number of pre-apnea frames in a subject but was capped at 10. This ensured that for subjects with a small number of pre-apnea frames (<10), each pre-apnea frame would be used only once in the out-of-fold testing. The model is then trained k times, with the performance measures computed on predictions of the out-of-fold dataset. The cross-validation approach identified the best model parameters used for the testing datasets. The testing dataset was not involved in the training procedure but used to report the model's performance on unseen data.

2.6. Model development

The objective of the model was to predict an apneic event. This could be achieved by classifying the data into inter-apnea and pre-apnea frames. We used a combination of unsupervised and supervised learning methods to build this model. The unsupervised method consisted of Gaussian Mixture Models, whereas the supervised method was a Logistic Regression as a final stage of the classification.

A Gaussian mixture model (GMM) is a parametric probability density function represented as a weighted sum of Gaussian component densities [41]. In this model, data from the N-observation, D-feature space ($\mathbf{x}_1, \mathbf{x}_2, \ldots \mathbf{x}_d$) were assumed to arise from a random vector with density given by,

$$f(\boldsymbol{x}) = \sum_{i=1}^{C} W_i \; g(x|\mu_i, \Sigma_i) \qquad (1)$$

where, C is the number of Gaussian mixture components, W_i is the mixture weight, i.e., the prior probability of a certain component and $g(\boldsymbol{x}|\mu, \Sigma)$ are the component Gaussian densities. Each component density is a D-variate Gaussian function of the form,

$$g(x\mu_i, \Sigma_i) = \frac{1}{(2\pi)^{\frac{D}{2}} |\Sigma_i|^{\frac{1}{2}}} e^{-\frac{1}{2}(x-\mu_i)' \Sigma_i^{-1} (x-\mu_i)} \qquad (2)$$

with mean vector $\boldsymbol{\mu}$ and covariance matrix $\boldsymbol{\Sigma}$. Generally, the mixing parameters $\theta = (W_1, \ldots W_k, \mu_1, \ldots \mu_k, \Sigma_1, \ldots \Sigma_k)$ are estimated from training data by maximizing the log-likelihood,

$$L(\boldsymbol{\theta}|\boldsymbol{x}) = \sum_{j=1}^{N} ln \left[\sum_{i=1}^{K} W_i \; g(x|\mu_i, \Sigma_i) \right] \qquad (3)$$

A generally used approach for a maximum likelihood solution is the Expectation Maximization (EM) algorithm. EM is an iterative algorithm that starts with an initial estimate for θ and iteratively modifies θ to increase the likelihood of the observed data.

GMMs have been extensively used in speaker and language identification [41-43]. The premise of the method is to build separate GMMs from the inter-apnea and pre-apnea datasets. Each of these GMMs encode the distribution of the corresponding class features. This technique has been used before in biomedical applications such as identification of epileptic seizures [44] and ECG signal classification [45,46]. It was also used in the preliminary study by [35] to predict apneas that performed a coupling approach using Bayesian adaptation which assisted in the separation of the Gaussian representing pre-apneas from the Gaussian representing inter-apneas. The Bayesian adaptation was essential since the individual Gaussians were sensitive to the random initial values in the EM algorithm.

We applied a different approach to counter this problem. First, for each (k-1)-fold of the cross-validation, 10 individual models were created separately for the inter-apnea and pre-apnea segments of the training data. The training data were then parsed through each of the 20 models and negative loglikelihood (nlogL) values for each observation were obtained. The average nlogL was then computed from 10 inter-apnea and 10 pre-apnea model outputs. The two new columns along with their class labels were then used to train a logistic regression classifier. The model is illustrated in Fig. 6.

The logistic regression is a binary classification model in which the conditional probability of one of the two possible realizations of the output variable was assumed to be equal to a linear combination of the input variables, transformed by the logistic function. It is expressed as,

$$f = \frac{1}{1 + e^{-p^T \Phi}} \qquad (4)$$

where f is the output of the logistic expression having values in the interval [0,1]; $\boldsymbol{\phi}$ has two column vectors corresponding to the average nlogL obtained from the inter-apnea and pre-apnea models for each observation and $\mathbf{p}$ is a column vector containing the weights of the linear combination. T is the transpose operator.

The out-of-fold test data were subjected to the same procedure to make predictions, where the features were inputs to the 20 GMM's, their nlogL averaged to produce two columns, which were then inputted to the trained logistic regression classifier. Predictions from all the test folds were collected and compared with their expected outcome to evaluate performance across the entire training dataset. Also, to account for the randomness in the k-fold splits and the initialization in the EM algorithm, the above procedure was repeated 50 times and the average (±SD) performance measures were obtained. For each subject, the number of Gaussian components in each GMM was chosen as the one that maximized the Area Under the Curve (AUC), described in the next section. A higher number of Gaussian components resulted in overfitting of

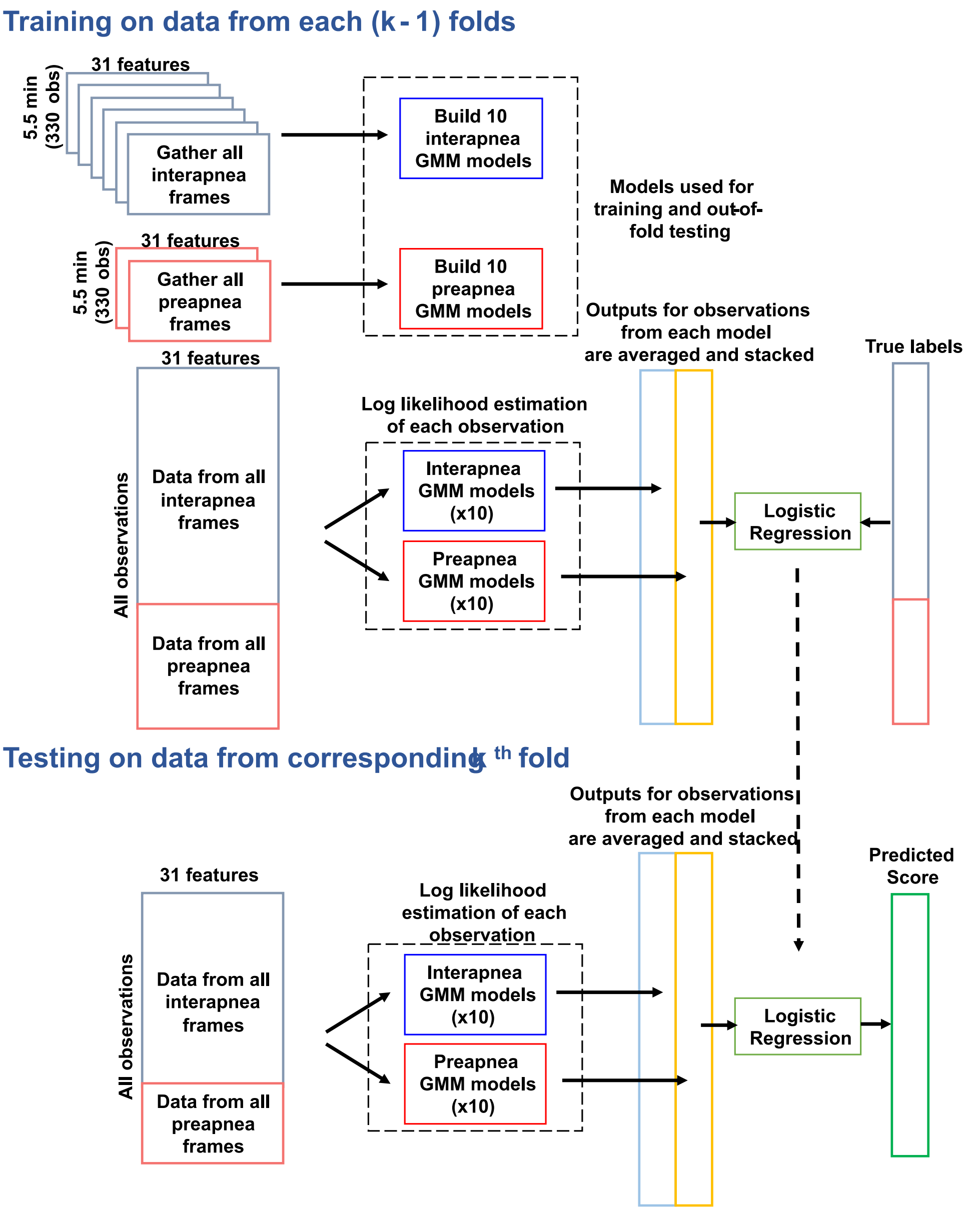

Fig. 6. Schematic Diagram of the classifier based on Gaussian Mixture Models (GMM) and Logistic Regression.

the training data. Therefore, the best performance measures were reported when 2 or 3 Gaussian components were used.

Training on (k-1)th fold: GMM development: For each inter-apnea and pre-apnea frame, a feature matrix of dimension (330-by-31) was obtained, where 31 indicates the number of features from PCA at each time step; 330 indicates the total duration of 5.5 min (330s). All inter-apnea frames were used to create 10 different inter-apnea GMM models. A similar procedure was conducted on the pre-apnea frames.

Training the Logistic Regression Classifier: The training data were parsed through each of the 20 models and negative loglikelihood (nlogL) values for each observation were obtained. The average nlogL was then computed from 10 inter-apnea and 10 pre-apnea model outputs. The two new columns along with their class labels were then used to train a logistic regression classifier.

Testing on kth fold: Data from the kth fold were also parsed through each of the 20 models and nlogL values for each observation were obtained. The trained logistic regression classifier was then used to classify the observations as belonging to either a pre-apnea or an inter-apnea segment.

2.7. Performance evaluation

The performance evaluation criteria were based on the AUC (area under the curve) with reference to the previously published work [35]. The AUC was used to evaluate the prediction performance of the classification model based on the Receiver Operating characteristic Curve (ROC): a plot of the sensitivity versus 1-specificity that was obtained by varying the classification threshold from output (f) of the trained logistic regression model. The AUC scores range between 0 and 1, with 0 defining perfect classification to the wrong class, and 1 defining perfect classification to the correct class. An AUC score of 0.5 corresponds to prediction by chance. Higher scores correspond to better classification performance.

Sensitivity or True Positive Rate (TPR) was defined as the fraction of pre-apnea frames that were predicted correctly. Specificity or True Negative Rate was defined as the fraction of inter-apnea frames that were predicted correctly. Therefore, for the AUC we computed (1-Specificity) = False Positive Rate (FPR), or the fraction of inter-apnea frames incorrectly classified as pre-apnea

frames. Hence, FPR represented the probability of false alarms in the model. The optimal values of TPR and FPR were reported at the threshold that minimized the distance between the ROC curve and its top left corner (perfect classification). Since the proportion of pre-apnea and inter-apnea frames was highly imbalanced, accuracy of the classifier was not computed as its interpretation would be misleading.

A permutation test was performed with two objectives: 1) To test the null hypothesis that there was no signal in the data. If the data did not have any predictive information about apneas, its performance would be statistically similar to what would be expected by chance. 2) To quantify the degree of overfitting when sampling under the null hypothesis. If the mean AUC from the permutations was not 0.5, then the analysis protocol was biased by an amount equal to the distance from 0.5. Permutation testing was performed by randomly shuffling the pre-apnea and inter-apnea labels for all frames and re-running all steps of the predictive analytic framework including k-fold cross-validation, model development and performance evaluation. The shuffling procedure was repeated 1000 times and an AUC was obtained for each profile. The mean AUC and the distribution of AUCs from the 1000 profiles was compared with the AUC of the original unshuffled data (one-sided p-value) to test that the original AUC was significantly higher than chance.

In order to compute features at any time point, data from the past 2 minutes were used. Thus, the predication of the positive class (pre-apnea) could only occur in the last 5.5min of the 7.5min-frame. Further, to reduce number of false positives, a time point was considered as pre-apnea if at least one of the last two points had a positive outcome. Since features were only generated every 10s, this further reduced the prediction time from 5.5min (330s) to 310s. The cumulative distribution of the time of earliest prediction was obtained, showing the efficacy of the model in predicting apneic events prior to their onset. Total false alarm durations (FA_{time}) were reported as total time in minutes normalized per hour of inter-apnea duration.

2.8. Probability of prediction based on severity of events

The clinical importance of the model would arise from successful prediction of severe ABH events. The severity of an apnea is related to the degree of bradycardia since bradycardias are closely associated with apneic pauses. In preterm infants, bradycardias below 100bpm result in a 10-50% reduction in cerebral blood flow velocity. The reduction is more than 50% when the heart rate drops below 60bpm [12]. This can further lead to reduced clearance of metabolic by-products and to desaturation of cerebral oxygen. Based on these findings, apneic events were classified as mild (100–80bpm), moderate (80–60bpm), and severe (<60bpm). For the ABH events in our study, median bradycardia values were used within each range of severity.

2.9. Importance of including movement-derived features

The predictive analytic framework was repeated for all data, first with exclusion of any movement features, then by including movement features. Excluding movement features meant that only 21 of the 31 features from the PCA output were used in the training of the models. The objective of this analysis was to test if excluding movement features improves or worsens the prediction performance of apneas. For this test, AUC obtained by using 31 features and 21 non-movement related features were compared with a paired t-test.

Table 2
Performance measures of the model for all subjects that were used for cross-validation. For the original AUC, mean ± standard deviation are presented. For the label shuffling, the means and the 95% confidence intervals are presented. The predictive performance was significantly different from chance for all subjects at $p < 0.05$.

Sub ID	AUC	p-value	AUC(permutated labels)
1	0.73 ± 0.03	*0.01*	0.49 (0.28 - 0.70)
2	0.87 ± 0.03	*0.01*	0.51 (0.22 - 0.79)
3	0.81 ± 0.02	*0.00*	0.47 (0.28 - 0.66)
4	0.84 ± 0.06	*0.00*	0.47 (0.20 - 0.73)
5	0.77 ± 0.08	*0.03*	0.50 (0.21 - 0.78)
6	0.67 ± 0.02	*0.01*	0.50 (0.35 - 0.64)
7	0.71 ± 0.04	*0.02*	0.52 (0.33 - 0.71)
Mean	**0.77 ± 0.04**		

2.10. Real-time prediction on unseen data

The test data consisted of 3 subjects that were not used in the training procedure. To apply the model to these subjects, labeled data including a sufficient number of pre-apnea segments would be needed. This could cause delay in deploying the model in real time. In some situations, labeling data may not even be practical. To avoid this problem, we completed the training procedure using data from train datasets (7 subjects) and predictions were made on the test subjects. Subjects were matched based on their average heart rates. Once a matched train dataset was identified, their normalization values and PCA coefficients were applied to the corresponding test subject data. Predictions were obtained from 50 models comprising of the GMM-Logistic Regression combination, and final prediction was based on a majority vote from the 50 models. The prior proportion of inter-apnea and pre-apnea frames in the training set was used in determining the prediction threshold with each of the 50 models.

In a real-time application, physiological data would be recorded as new predictions are made. A positive prediction would sound an alarm or turn on a stochastic vibratory mattress. If an undetected apnea was encountered, a new pre-apnea frame would be created from 7.5min of data prior to the apneic onset. New inter-apnea frames would also be formed from data that the model labeled as true negative. These new frames would replace data from the train subject and new models would be generated to make predictions on future data. If there were no undetected apneas for 4 hours (or to the respective feeding interval), only the new inter-apnea frames would be used to create new models upon replacing the train subject's data (Fig. 7).

We tested this framework on the 3 test subjects and evaluated the performance measures of TPR and FA_{time}. Note that in real time, if an action is taken to respond to an alarm (whether true or false), it may mitigate an upcoming apnea before it occurs and both TPR and FA_{time} will not be available. However, for the retrospective analysis of our test data we were able to report these performance measures. The distribution of the earliest detection times and the probability of detection based on severity is also reported.

3. Results

3.1. Classification performance of model

Performance evaluation measures were obtained from the area under the ROC curve (AUC). Fig. 8 shows ROC curves from 2 subjects. The red dashed line signifies random classification. The shaded areas around the ROC curve represent the standard deviation achieved by k-fold cross-validation of AUC estimates over 50 repeated runs of cross-validation. Table 2 shows that the AUC values for all subjects were above 0.5. Statistical significance for each

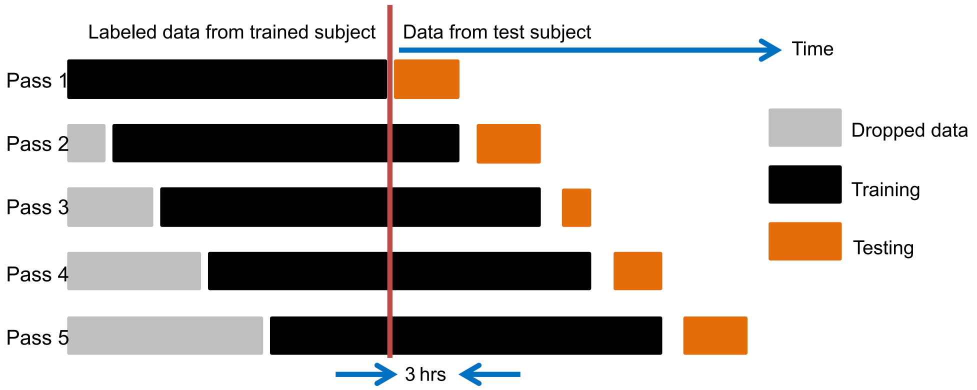

Fig. 7. Real-time prediction scheme.

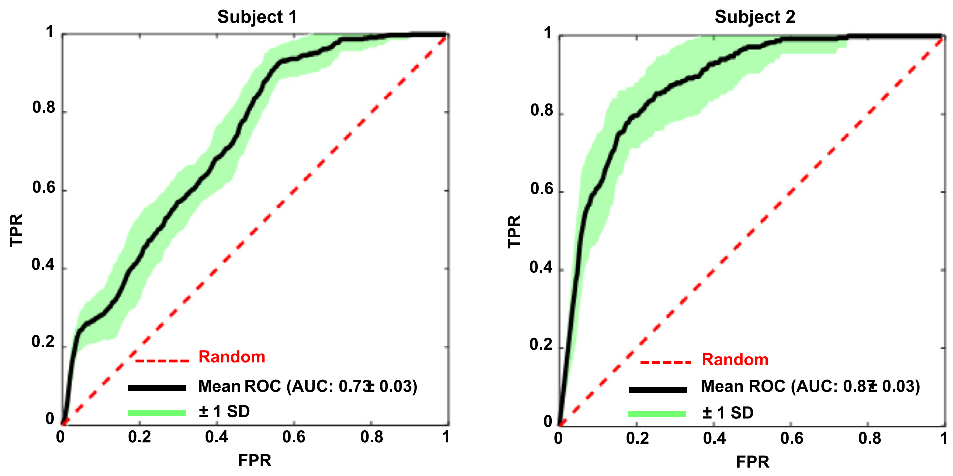

Fig. 8. ROC curves from prediction scores for Subjects 1 and 2. The dashed red line signifies random classification. The shaded green area represents the standard deviation of the ROC curve obtained from 50 repeated runs of the model.

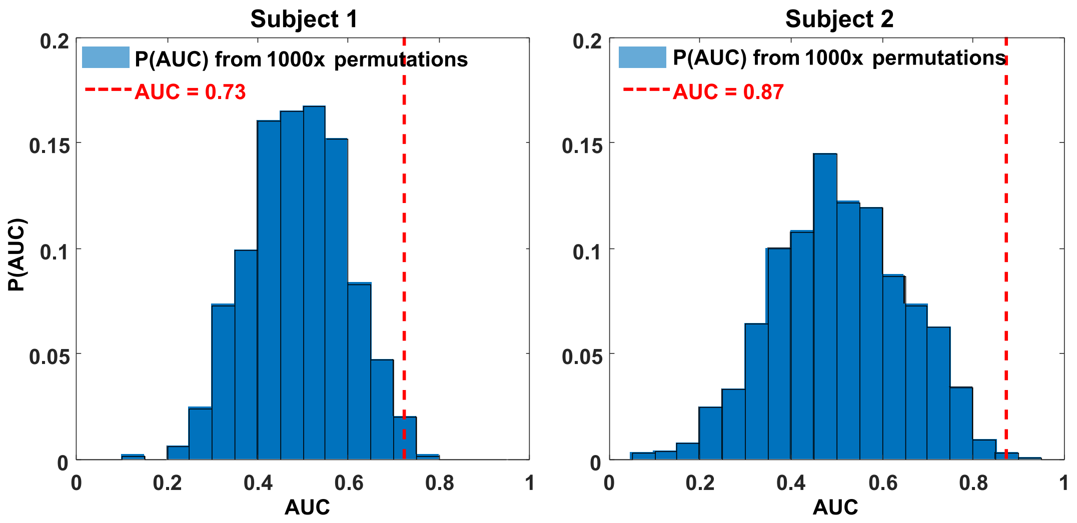

Fig. 9. Probability distribution of the AUC from 1000 shuffled runs for Subjects 1 and 2. The original AUC is indicated by the dashed red lines.

subject's AUC was tested by randomly shuffling the labels 1000 times. For 2 subjects, Fig. 9 shows the distribution of the AUC from the 1000 permutations and the AUC of the original labels is indicated by the dashed red line. The table shows the p-values for all subjects. These results suggest that the AUC was significantly different from chance, and that the data indeed had predictive information about apneas. Table 1 also reveals that the mean AUC from permutation testing was 0.5 and that then the analysis protocol was not biased.

3.2. Apnea predications and false-alarm duration on cross-validated subjects

For each subject, the TPR and FPR were achieved from the ROC curve at the prediction score threshold that minimized the dis-

Table 3
True Positive Rate and false positive duration for each subject.

Sub ID	True Positive Rate	False Positive Rate	Total inter-apnea duration(hr)	False positive duration(min/hr)
1	0.70 ± 0.10	0.34 ± 0.09	14.76	2.82 ± 0.96
2	0.86 ± 0.09	0.15 ± 0.06	25.94	1.11 ± 0.49
3	0.88 ± 0.08	0.31 ± 0.04	19.16	8.59 ± 1.80
4	0.85 ± 0.11	0.23 ± 0.07	22.46	1.34 ± 0.55
5	0.74 ± 0.19	0.15 ± 0.05	9.08	1.02 ± 0.47
6	0.67 ± 0.05	0.28 ± 0.05	30.07	3.35 ± 0.92
7	0.71 ± 0.08	0.32 ± 0.08	7.24	1.88 ± 0.70
Mean	**0.77 ± 0.10**	**0.25 ± 0.06**		**2.87 ± 0.84**

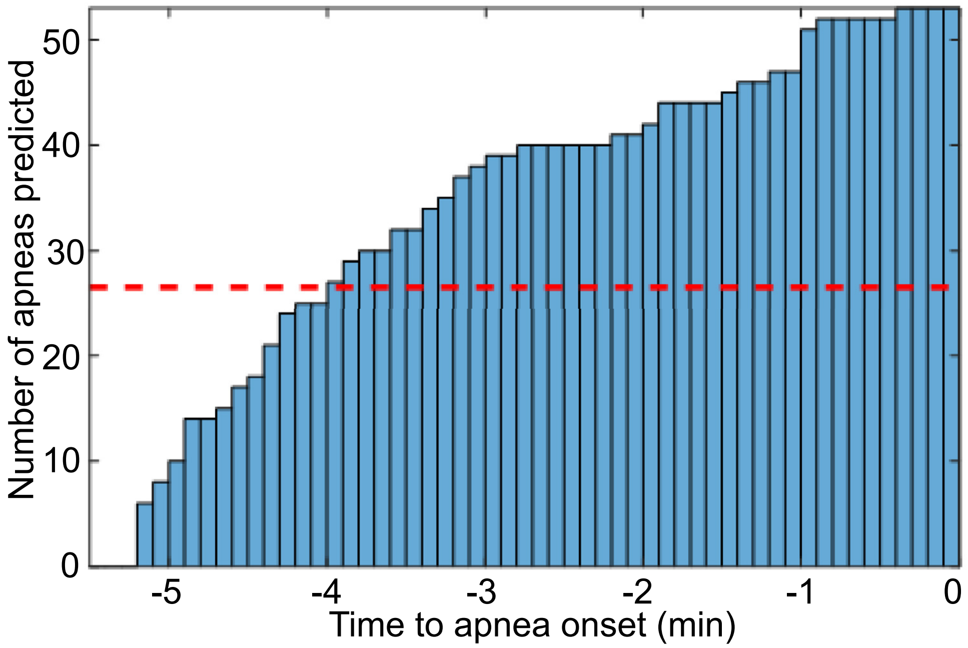

Fig. 10. Cumulative distribution of prediction time relative to the onset of apnea (training data set).

tance between the ROC curve and the perfect classification point at the top left corner. Since FPR reported a proportion of inter-apnea frames that were misclassified as pre-apnea frames, FPR was further converted to represent the total false alarm time. The false alarm time was reported in minutes per hour of the total predictable inter-apnea duration as each subject had different recording durations. Table 3 shows the TPR for each subject along with the total inter-apnea duration and the false positive time in min/hr.

Regarding the accurately classified pre-apnea frames, their prediction times relative to the apnea onsets were computed. Fig. 10 shows the cumulative distribution of these prediction times: 53 of all 66 pre-apnea frames were correctly classified. The red dashed line indicates that 50% of the correct predictions occurred 4min before their apnea onset.

The probability of predicting an apneic event was further plotted against their severity based on the bradycardia associated with the apnea as shown in Fig. 11. The different shades of red indicate severity of an apneic event based on bradycardia categorized into mild (80-100bpm), moderate (60-80bpm) and severe (<60bpm). Darker red shades represent more severe events. Dots in these ranges represent an apneic event, with its x-coordinate indicating the median bradycardia in the category and y-coordinate indicating the probability of prediction of that event obtained from 50 repeated runs of the model. The dashed blue line represents the threshold for final classification of an event as predicted or missed. The color of the dot encodes information about the oxygen saturation levels associated with the event. Note that the range >100bpm by definition does not indicate a bradycardia. The dots in this range represent apneas (non-bradycardic) that were greater than 20s, but were not accompanied by a bradycardia. The x-coordinate of these dots are the minimum heart rate values. The model was able to successfully predict events in all 4 ranges (probability of predicted events: severe: 0.83, mild: 0.74, moderate: 0.83, non-bradycardic: 0.83).

3.3. Performance measures of models with and without movement-derived features

The next step was to build models after movement-derived features were excluded to test the importance of movement in prediction of apnea. Only 21 features from the PCA were used to build these models. Fig. 12 compares the AUC, TPR and false positive time between the models that include and exclude movements. The AUC from the models without movement features was significantly smaller than the ones that included movement features (p=0.02). Moreover, the AUC from 5 of the 7 subjects (Subject #1, 4, 5, 6, 7) were not significantly different from chance, implying that including movement-derived features improved the prediction of apneas. TPR and the total duration of false positive time was also improved when movement-derived features were included.

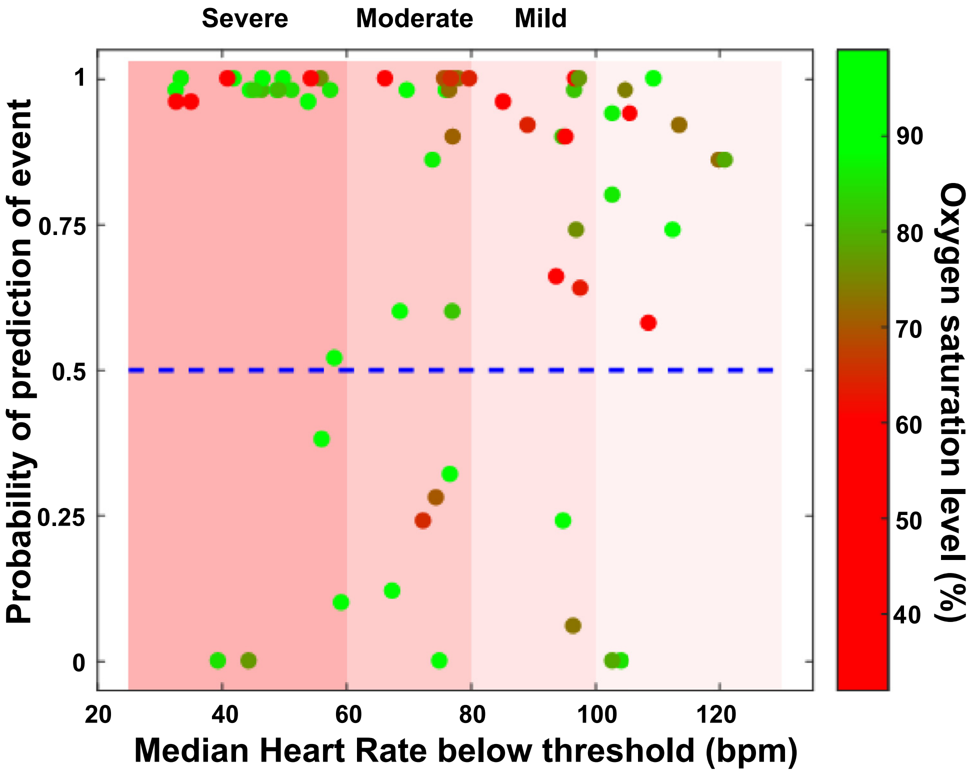

Fig. 11. Cross-validated prediction based on severity of apneas (training data set).

3.4. Apnea predictions and false-alarm durations for new test subjects

The models were then applied on the data from test subjects. Due to the randomness in the initializations of the EM algorithm to fit GMMs, 50 such models were used, and predictions were made at every time point. Fig. 13 shows the cumulative distribution of these prediction times: 31 of 41 ABH events were correctly predicted. The red dashed line indicated that 50% of the correct predictions occurred 3.2min before their apneic onset. As for the cross-validated dataset, prediction based on severity is plotted in Fig. 14. The model yielded better predictions in the 3 clinically significant ranges (probability of predicted events: severe: 0.75, mild: 0.75, moderate: 0.78).

4. Discussion

This study proposed that cardiorespiratory features coupled with movement features are better predictors of apneas than cardiorespiratory signals alone. To test this hypothesis the study used a combination of unsupervised and supervised machine learning methods. These features are readily available from signals that are continuously monitored in the NICU as a part of routine care of preterm infants. Furthermore, we proposed a framework for the implementation of the model that will be able to make real-time predictions on new subjects.

4.1. Comparison with previous studies and novelty of this study

Only few studies have investigated the prediction of apneic and bradycardic events in preterm infants and reported the model performance. For example, Shirwaikar et al. used support vector machines and random forests [29] and deep neural networks [30] to predict the total occurrences of apneic episodes one week after NICU admission. While successful, their studies were not designed to predict the timing of individual apneic events. A number of studies focused on the prediction of bradycardic events. For example, Pravisani et al. [24] used hierarchical classification methods and were able to predict 10 out of 13 severe bradycardic events 5 premature infants, Gee et al. [25] examined heart rate intervals in 10 preterm infants with a total of 444 bradycardic events. Their prediction algorithm was based on point process analysis and achieved an AUC of 0.79. The same dataset was used by Mahmud et al. [26] who achieved a slightly higher AUC of 0.86 with boosted decision trees. These studies [24-26], however, only looked at bradycardic events without taking apnea and hypoxias (ABH events) into account. As these ABH events are of similar clinical importance and are subject to surveillance and alarms, Williamson et al. [28] examined these ABH events using quadratic classifiers on cardiorespiratory based features. On 6 subjects, the AUC of their model was 0.73, but their results were statistically different from chance in only 3 infants. Their AUC improved to 0.80 and became statistically different from chance in 5 of 6 infants when they used Gaussian mixture models and when they included movement features [35]. However, each of the 6 subjects were recorded for only a short time between 4 and 7 hours.

Our study expanded on these previous approaches by adding several enhancements. Firstly, our study increased the number of subjects to 10, each with a total recording duration between 19 and 72 hours. Secondly, movement was estimated from the PPG signal at a greater temporal resolution using a wavelet-based algorithm [36] in contrast to Fourier-based analysis used in [35]. We also demonstrated how the inclusion of movement features statistically improved the AUC of the models. Thirdly, we reported the efficacy of the model in predicting individual apneic events based on the severity of the associated bradycardia and hypoxia. Finally, we also included the distribution of their prediction times. Training models on datasets of longer durations as well as obtaining features at a higher temporal resolution significantly improved the prediction of ABH events, thus yielding an AUC of 0.77.

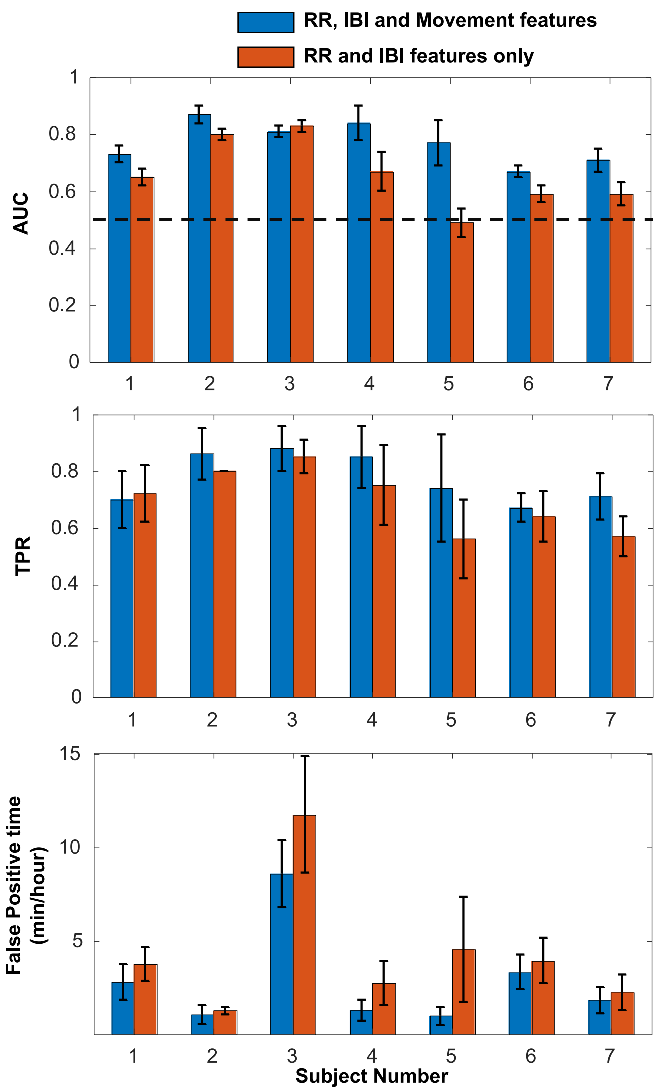

Fig. 12. Comparison of performance methods from models including (blue) and excluding (red) movement-derived features. The black dashed line in the AUC Fig. indicates classification by chance. When movement features were excluded to train the models, classification within the subjects #1, 4, 5, 6 and 7 were not significantly different from chance. All other models were significant.

4.2. Improvement in model performance with inclusion of movement features

Our results showed that the AUC of the model was better when features from IBI, RR and movement were included, in contrast to when only features from IBI and RR were used. Moreover, in all 7 subjects, the models including movement had an AUC value significantly greater than 0.5, indicating that the predictions were significantly different from those made by random chance. Before movement was included, the AUC in 5 of the 7 subjects were not different from 0.5. From a data analysis point of view, IBI's from respiratory signals that are acquired through respiratory inductance plethysmography are usually distorted during movement. Hence, it may have been that adding movement information improved the model since it also considered IBI variability during movement. However, from a physiological perspective, movement and apneas have been shown to be associated with each other [31-34]. The respiratory controller in the brainstem that regulates the rate of breathing, receives inputs through several feedback and feedforward (central command) mechanisms [47]. One of these feedback mechanisms is movement which sends signals through muscle afferents to the respiratory controller or even fine-tune the feedforward commands [48]. Although the exact mechanism of why some movements in preterm infants inhibit their breathing is unknown, one could speculate that immature control of the respiratory center and reduced chemoreceptor sensitivity account for some of the causes [49,50].

The true positive rate (TPR) or the probability of prediction of an apneic event was 77% on average. This estimate was further sub-classified based on the severity on the bradycardia that was associated with the apneic event. Severe bradycardias (<60bpm) have been shown to cause more than 50% reduction in cerebral blood velocity [12]. Our results show that 83% of these severe events could be predicted. Of the ones that were not predicted, the minimum SpO_2 levels were still above 70%. Similar results were obtained on apneas from the test datasets. Detection of these severe bradycardic events are of utmost clinical importance as they can enable early interventions that circumvent the upcoming event.

4.3. Prediction time

Pre-apnea segments were considered as the 7.5min segments prior of the onset of an apnea. Clark et al. modeled apneas of prematurity as a Markov process where apneas were the result of transitions through 4 breathing states [51]. These 4 states had average lifetimes, categorized in term of decreasing stability from 12hr, 2hr, 10min and 10s, with transitions to an apneic state possible from all states, but more likely from the lesser stable states of 10min and 10s. They speculated that the 12-hr state represented wakefulness and the 2-hr state represented various stages of sleep. The remaining two states of 10min and 10s represent transition states to apnea due to disproportionate feedback mechanisms to the respiratory controller. A prediction window of 7.5min that encompassed these two states was a good compromise between being close enough to an apneic onset and not being too far away that would model inter-apnea distributions. On both training and test data sets, half of the ABH events were predicted between 3 to 4min before their onset. In future work, other prediction windows between 5 and 10min might be evaluated for comparison.

4.4. Real-time prediction

The prediction on test subjects was demonstrated as a basis for the real-time implementation, where labeled data would not be readily available. Labeled data from trained subjects were used for initial prediction, but they were replaced by auto-labeled data from the test subjects as they became available. For this study, a matching training subject with a comparable average heart rate was selected. In practice, once data from a large number of subjects are available, additional demographic information, such as gestation age, post-menstrual age, birth and study weight could be used to find appropriately matching training subjects. Data on the training subjects could easily be auto-labelled into inter-apnea and pre-apnea segments using the defined threshold of apnea, bradycardia and oxygen desaturation. However, sections of feeds and other interventions would have to be manually annotated in order to be excluded from training the models. Data during these conditions can have a different physiological basis when compared to the inter-apnea and pre-apnea frames.

In a real-time prediction scenario, a positive detection of a pre-apnea frame could be a prediction for apnea or a false alarm. If an intervention is performed in response to the positive detection, the outcome will be unknown. Interventions could be automated where a stochastic vibratory mattress would turn on. Stochastic vibrotactile stimulation has been demonstrated to reduce the inci-

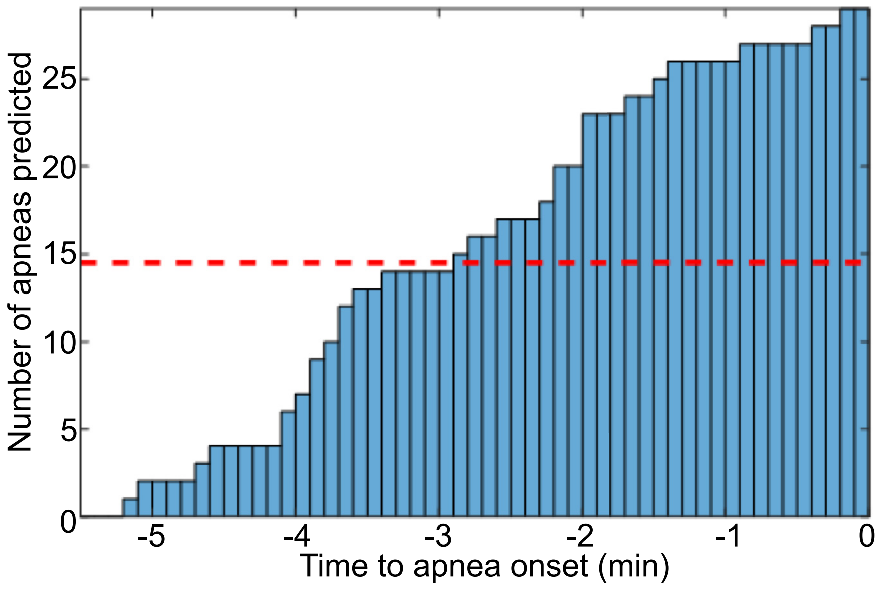

Fig. 13. Cumulative distribution of prediction time relative to onset of apnea (test data set).

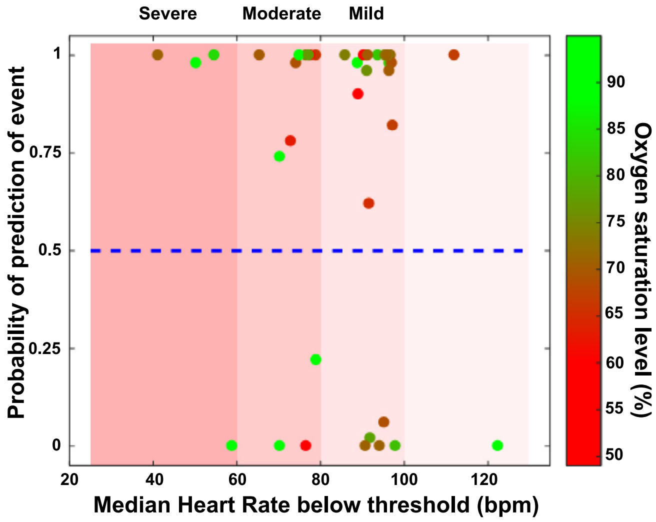

Fig. 14. Prediction based on severity of apneas (test data set).

dences of apneas, bradycardias and desaturations in preterm infants [16,17]. This effect is based on the hypothesis that stochastic inputs to the respiratory controller would stabilize its rhythmic activity through a stochastic resonance-type mechanism [16,52]. False alarms of apneas would result in intermittent periods of vibrotactile stimulation for a preset duration. However, this does not pose any clinical risk to the infant, since the subtle stimulation does not disrupt sleep. On the other hand, one might suggest that keeping the mattress 'on' at all times or for a longer period of time would not require the need for prediction. However, this could result in habituation to the stimulation to an extent that it may eventually attenuate the effect of reducing apneas [53]. If the response to a positive prediction is intervention by a nurse, additional thresholds would need to be coded in the algorithm where the first few alarms are warning tones, leading to apnea alarms for persistent positive predictions.

An undetected apnea would prompt the algorithm to retrain the model based on recently tested data. Labels for the tested data would be automatically labeled from the ECG, respiration and PPG signals and the corresponding features would be obtained. New prediction would resume at least 7.5min from the end of the last apnea in the cluster of the undetected apnea, providing enough time to retrain the new models. During the re-training, cross-validation to find the best number of clusters was not performed since it is a time-consuming process that may not be practical for real-time prediction. However, depending on availability of computational resources, this could be performed in parallel to the prediction algorithm.

4.5. Alternative modelling techniques

Our feature engineering procedure utilized correlations between past data and current data to get a total of 1800 features which were reduced to 31. Other feature selection techniques based on maximum relevance minimum redundancy and neighborhood component analysis were also tested. But since the features within each set of 360 features were highly correlated, PCA seemed to present the appropriate technique for feature reduction. Feature selection could also have been performed on the set of 31 features to further generate a small set of important features. As this exhaustive search is usually impractical it was therefore not performed.

Previous work with GMMs have used a background GMM for all data from subject and then tuned this background GMM using Bayesian adaptation on data for each class to further create separate GMMs for each class [35]. We used the concept of bootstrapped aggregation on the GMM-Logistic Regression models to account for the random initializations in the EM algorithm of the GMMs. This method was still able to classify the two labels from the underlying distributions of the GMMs, therefore Bayesian adaptation was not performed.

Our dataset has an imbalance between the number of inter-apnea and pre-apnea frames. One of the techniques that deal with imbalanced datasets focuses on rebalancing them, usually performed by undersampling the majority class or oversampling the minority class [54]. However, depending on the domain, rebalancing has shown to yield marginal improvements at best. The logistic regression model handles this imbalance since the probabilities achieved are based on the proportion of the class labels, and an appropriate threshold can be used for the class assignment.

We attempted several other machine learning algorithms including linear discriminant analysis, support vector machines (SVM) and ensembles of boosted decision trees. Linear discriminant analysis and linear SVMs yielded poor performance measures since they were unable to find an optimum linear separable boundary between the two classes. Non-linear SVMs and boosted ensemble models have high complexity due to the number of hyperparameters that required tuning to train the model. Furthermore, the best set of hyperparameters varied between subjects, making it difficult to use in a real-time implementation. This was likely due to the heterogeneity between subjects at different stages of prematurity. However, the GMMs were still able to encode the distibutions of the features in the inter-apnea and pre-apnea frames within each subject. The only hyperparameter that required tuning in our proposed model was the number of mixtures in the GMM that was usually two or three. Higher number of mixtures tended to overfit to the training data set.

4.6. Future work

This study proposed a framework consisting of Gaussian Mixtures and Logistic Regression Models to predict ABH events in preterm infants monitored in the NICU. Importantly, this analysis could be implemented in real-time. Our results showed that including features of movement into our framework significantly improved its predictive ability. However, it is important to note that the incidences and severities of ABH events in preterm infants depend on several factors, such as gestational and post-menstrual age of the infant and complications due to prematurity. The latter subjects the infant to interventions, procedures, and a variety of different drugs. Regarding the use of movement features in the prediction framework, our study used recordings for a duration of only 1 to 3 days. However, in a previous longitudinal study, where movement was continuously recorded over 3 months, we observed that these features changed as an infant matured [55]. Therefore, to generalize our work to all preterm infants with a wide range of ages and comorbidities, the training of long-term recordings over the period of maturation and from a larger cohort of preterm infants is needed. Ideally, multi-site longitudinal recordings would assist in understanding how the framework evolves and how important features at different stages of prematurity can be isolated.

5. Conclusions

This study proposed a model to predict ABH events using cardiorespiratory and movement features. Our findings provide evidence that movement is a valuable physiological signal for improving the predictive ability of such events. The prediction of apneas and the associated bradycardias and hypoxias in preterm infants can enable a timely intervention by the nursing staff. It can also be combined with using an automated therapeutic mattress that can attenuate or prevent the onset of apneas. Hence, the algorithm has the potential to reduce morbidity, mortality and time of hospital stay of preterm infants. A study on a larger cohort of infants with varying complications due to prematurity will help corroborate and extend our present findings.

Declaration of Competing Interest

The authors declare that they have no conflict of interest.

Acknowledgment

The authors thank Courtney Temple and Alan Gee for their help in data collection, the NICU staff for subject recruitment, and the infants and families for participating in this study.

Funding

This study received funding from the NIH grants R01-GM104987, R01-HD081346, R21-HD089731, and U01-HL133536; NSF SCH grant #1664815; SIMONS FOUNDATION grant SFARI-602705, and the Wyss Institute at Harvard University.

References

[1] W.J.R. Daily, M. Klaus, H. Belton, P. Meyer, Apnea in premature infants: monitoring, incidence, heart rate changes, and an effect of environmental temperature, Pediatrics 43 (1969) 510–518.
[2] D.A. Paul, K.H. Leef, R.G. Locke, L. Bartoshesky, J. Walrath, J.L. Stefano, Increasing illness severity in very low birth weight infants over a 9-year period, BMC Pediatr. 6 (2006) 2.
[3] R.J. Martin, C.G. Wilson, Apnea of prematurity, Comprehensive Physiol. 2 (2012) 2923–2931.
[4] O.P. Mathew, Apnea of prematurity: pathogenesis and management strategies, J. Perinatol. 31 (2011) 302–310.
[5] D.C. Shannon, Pathophysiologic mechanisms causing sleep apnea and hypoventilation in infants, Sleep 3 (1980) 343–349.
[6] R.J. Martin, J.M. Abu-Shaweesh, T.M. Baird, Apnoea of prematurity, Paediatr. Respir. Rev. 5 (2004) S377–S382.
[7] L.A. Stokowski, A primer on apnea of prematurity, Adv. Neonatal Care 5 (2005) 155–170.
[8] N. Idiong, R.P. Lemke, Y.-J. Lin, K. Kwiatkowski, D.B. Cates, H. Rigatto, Airway closure during mixed apneas in preterm infants: Is respiratory effort necessary? J. Pediatr. 133 (1998) 509–512.
[9] A.D. Milner, A.W. Boon, R.A. Saunders, I.E. Hopkin, Upper airways obstruction and apnoea in preterm babies, Arch. Dis. Child. 55 (1980) 22–25.
[10] T.B. Waggener, I.D. Frantz, B.A. Cohlan, A.R. Stark, Mixed and obstructive apneas are related to ventilatory oscillations in premature infants, J. Appl. Physiol. 66 (1989) 2818–2826.
[11] A. Yamamoto, N. Yokoyama, M. Yonetani, Y. Uetani, H. Nakamura, H. Nakao, Evaluation of change of cerebral circulation by SpO2 in preterm infants with apneic episodes using near infrared spectroscopy, Pediatr. Int. 45 (2003) 661–664.
[12] J.M. Perlman, J.J. Volpe, Episodes of apnea and bradycardia in the preterm newborn: impact on cerebral circulation, Pediatrics 76 (1985) 333–338.
[13] S. Supcun, P. Kutz, W. Pielemeier, C. Roll, Caffeine increases cerebral cortical activity in preterm infants, J. Pediatr. 156 (2010) 490–491.
[14] D.J. Henderson-Smart, A.G.D. Paoli, Methylxanthine treatment for apnoea in preterm infants, Cochrane Database of Systematic Reviews, 2010.
[15] J. Kattwinkel, H.S. Nearman, A.A. Fanaroff, P.G. Katona, M.H. Klaus, Apnea of prematurity: comparative therapeutic effects of cutaneous stimulation and nasal continuous positive airway pressure, J. Pediatr. 86 (1975) 588–592.
[16] E. Bloch-Salisbury, P. Indic, F. Bednarek, D. Paydarfar, Stabilizing immature breathing patterns of preterm infants using stochastic mechanosensory stimulation, J. Appl. Physiol. 107 (2009) 1017–1027.
[17] V.C. Smith, D. Kelty-Stephen, M.Q. Ahmad, W. Mao, K. Cakert, J. Osborne, D. Paydarfar, Stochastic resonance effects on apnea, bradycardia, and oxygenation: a randomized controlled trial, Pediatrics 136 (2015) e1561–e1568.
[18] D. Paydarfar, J. Niemi, I. Zuzarte, P. Indic, C. Knodel, J. Osborne, Systems and methods for inhibiting apneic and hypoxic events, U.S. Patent Application 14/889,486 (2016).
[19] J. Cruz, A.I. Hernandez, S. Wong, G. Carrault, A. Beuchee, Algorithm fusion for the early detection of apnea-bradycardia in preterm infants, in: 2006 Computers in Cardiology, 2006, pp. 473–476.
[20] M. Altuve, G. Carrault, A. Beuchée, P. Pladys, A.I. Hernández, On-line apnea-bradycardia detection using hidden semi-Markov models, in: 2011 Annual International Conference of the IEEE Engineering in Medicine and Biology Society, 2011, pp. 4374–4377.
[21] H. Lee, C.G. Rusin, D.E. Lake, M.T. Clark, L. Guin, T.J. Smoot, A.O. Paget-Brown, B.D. Vergales, J. Kattwinkel, J.R. Moorman, J.B. Delos, A new algorithm for detecting central apnea in neonates, Physiol. Meas. 33 (2011) 1–17.
[22] B.D. Vergales, A.O. Paget-Brown, H. Lee, L.E. Guin, T.J. Smoot, C.G. Rusin, M.T. Clark, J.B. Delos, K.D. Fairchild, D.E. Lake, R. Moorman, J. Kattwinkel, Accurate automated apnea analysis in preterm infants, Am. J. Perinatol. 31 (2014) 157–162.
[23] L. Cattani, D. Alinovi, G. Ferrari, R. Raheli, E. Pavlidis, C. Spagnoli, F. Pisani, Monitoring infants by automatic video processing: a unified approach to motion analysis, Comput. Biol. Med. 80 (2017) 158–165.
[24] G. Pravisani, A. Beuchee, L. Mainardi, G. Carrault, Short term prediction of severe bradycardia in premature newborns, Comput. Cardiol. 2003 (2003) 725–728.
[25] A.H. Gee, R. Barbieri, D. Paydarfar, P. Indic, Predicting bradycardia in preterm infants using point process analysis of heart rate, IEEE Trans. Biomed. Eng. 64 (2017) 2300–2308.
[26] M.S. Mahmud, H. Wang, Y. Kim, Accelerated prediction of bradycardia in preterm infants using time-frequency analysis, in: 2019 International Conference on Computing, Networking and Communications (ICNC), 2019, pp. 468–472.

[27] S. Das, B. Moraffah, A. Banerjee, S.K.S. Gupta, A. Papandreou-Suppappola, Bradycardia prediction in preterm infants using nonparametric kernel density estimation, in: 2019 53rd Asilomar Conference on Signals, Systems, and Computers, 2019, pp. 1309–1313.
[28] J.R. Williamson, D.W. Bliss, D.W. Browne, P. Indic, E. Bloch-Salisbury, D. Paydarfar, Using physiological signals to predict apnea in preterm infants, in: 2011 Conference Record of the Forty Fifth Asilomar Conference on Signals, Systems and Computers (ASILOMAR), 2011, pp. 1098–1102.
[29] R.D. Shirwaikar, D.U. Acharya, K. Makkithaya, M. Surulivelrajan, L.E.S. Lewis, Machine learning techniques for neonatal apnea prediction, J. Artific. Intell. 9 (2016).
[30] R.D. Shirwaikar, D. Acharya U, K. Makkithaya, M S., S. Srivastava, L.E.S. Lewis, Optimizing neural networks for medical data sets: a case study on neonatal apnea prediction, Artific. Intellig. Med. 98 (2019) 59–76.
[31] Y.K. Abu-Osba, R.T. Brouillette, S.L. Wilson, B.T. Thach, Breathing pattern and transcutaneous oxygen tension during motor activity in preterm infants, Am. Rev. Respir. Dis. 125 (1982) 382–387.
[32] L. Curzi-Dascalova, E. Christova-Guéorguiéva, Respiratory pauses in normal prematurely born infants. A comparison with full-term newborns, Biol. Neonate 44 (1983) 325–332.
[33] O.P. Mathew, C.K. Thoppil, M. Belan, Motor activity and apnea in preterm infants. Is there a causal relationship? Am. Rev. Respir. Dis. 144 (1991) 842–844.
[34] I. Zuzarte, D. Paydarfar, D. Sternad, Effect of spontaneous movement on respiration in preterm infants, Exp. Physiol. 106 (2021) 1285–1302.
[35] J.R. Williamson, D.W. Bliss, D.W. Browne, P. Indic, E. Bloch-Salisbury, D. Paydarfar, Individualized apnea prediction in preterm infants using cardio-respiratory and movement signals, in: 2013 IEEE International Conference on Body Sensor Networks, 2013, pp. 1–6.
[36] I. Zuzarte, P. Indic, D. Sternad, D. Paydarfar, Quantifying movement in preterm infants using photoplethysmography, Ann. Biomed. Eng. 47 (2019) 646–658.
[37] P. Indic, E.B. Salisbury, D. Paydarfar, E.N. Brown, R. Barbieri, Interaction between heart rate variability and respiration in preterm infants, in: 2008 Computers in Cardiology, 2008, pp. 57–60.
[38] S. Reulecke, S. Schulz, A. Voss, Autonomic regulation during quiet and active sleep states in very preterm neonates, Front. Physiol. 3 (2012).
[39] M.M. Kabir, D.A. Saint, E. Nalivaiko, D. Abbott, A. Voss, M. Baumert, Quantification of cardiorespiratory interactions based on joint symbolic dynamics, Ann. Biomed. Eng. 39 (2011) 2604.
[40] M.S. Santos, J.P. Soares, P.H. Abreu, H. Araujo, J. Santos, Cross-validation for imbalanced datasets: avoiding overoptimistic and overfitting approaches [research frontier], IEEE Comput. Intell. Mag. 13 (2018) 59–76.
[41] D. Reynolds, Gaussian mixture models, in: S.Z. Li, A. Jain (Eds.), Encyclopedia of Biometrics, Springer US, Boston, MA, 2009, pp. 659–663.
[42] D.A. Reynolds, T.F. Quatieri, R.B. Dunn, Speaker verification using adapted gaussian mixture models, Digit. Signal Process. 10 (2000) 19–41.
[43] P.A. Torres-Carrasquillo, D.A. Reynolds, J.R. Deller, Language identification using Gaussian mixture model tokenization, in: 2002 IEEE International Conference on Acoustics, Speech, and Signal Processing, 1, 2002, pp. I-757–I-760.
[44] S. Kusmakar, R. Muthuganapathy, B. Yan, T.J. O'Brien, M. Palaniswami, Gaussian mixture model for the identification of psychogenic non-epileptic seizures using a wearable accelerometer sensor, in: 2016 38th Annual International Conference of the IEEE Engineering in Medicine and Biology Society (EMBC), 2016, pp. 1006–1009.
[45] R.J. Martis, C. Chakraborty, A.K. Ray, A two-stage mechanism for registration and classification of ECG using Gaussian mixture model, Pattern Recognit. 42 (2009) 2979–2988.
[46] R. Ghorbani Afkhami, G. Azarnia, M.A. Tinati, Cardiac arrhythmia classification using statistical and mixture modeling features of ECG signals, Pattern Recognit. Lett. 70 (2016) 45–51.
[47] N.A. Shevtsova, V. Marchenko, T. Bezdudnaya, Modulation of respiratory system by limb muscle afferents in intact and injured spinal cord, Front. Neurosci. 13 (2019).
[48] M. Amann, G.M. Blain, L.T. Proctor, J.J. Sebranek, D.F. Pegelow, J.A. Dempsey, Group III and IV muscle afferents contribute to ventilatory and cardiovascular response to rhythmic exercise in humans, J. Appl. Physiol. 109 (2010) 966–976.
[49] J.M. Di Fiore, R.J. Martin, E.B. Gauda, Apnea of prematurity–perfect storm, Respir. Physiol. Neurobiol. 189 (2013) 213–222.
[50] E.B. Gauda, M. Shirahata, A. Mason, L.E. Pichard, E.W. Kostuk, R. Chavez-Valdez, Inflammation in the carotid body during development and its contribution to apnea of prematurity, Respir. Physiol. Neurobiol. 185 (2013) 120–131.
[51] M.T. Clark, J.B. Delos, D.E. Lake, H. Lee, K.D. Fairchild, J. Kattwinkel, J.R. Moorman, Stochastic modeling of central apnea events in preterm infants, Physiol. Meas. 37 (2016) 463–484.
[52] D. Paydarfar, D.M. Buerkel, Dysrhythmias of the respiratory oscillator, Chaos: An Interdisciplinary, J. Nonlinear Sci. 5 (1995) 18–29.
[53] V. Dumont, J. Bulla, N. Bessot, J. Gonidec, M. Zabalia, B. Guillois, N. Roche-Labarbe, The manual orienting response habituation to repeated tactile stimuli in preterm neonates: Discrimination of stimulus locations and interstimulus intervals, Dev. Psychobiol. 59 (2017) 590–602.
[54] A. Estabrooks, T. Jo, N. Japkowicz, A multiple resampling method for learning from imbalanced data sets, Comput. Intell. 20 (2004) 18–36.
[55] I. Zuzarte, A.H. Gee, D. Sternad, D. Paydarfar, Automated movement detection reveals features of maturation in preterm infants, in: 2020 42nd Annual International Conference of the IEEE Engineering in Medicine & Biology Society (EMBC), 2020, pp. 600–603.
