# Supplementary material for: Exploring Computational Techniques in Preprocessing Neonatal Physiological Signals for Detecting Adverse Outcomes: Scoping Review
Source: Interact J Med Res. 2024 Aug 20;13:e46946. doi: 10.2196/46946 (PMC11372324; doi:10.2196/46946)
Supplement: Multimedia Appendix 3 [file ijmr_v13i1e46946_app3.zip › Included Papers - Final/3330/Feng et al. - 2021 - Predicting mortality risk for preterm infants usin.pdf]

npj | Digital Medicine

www.nature.com/npjdigitalmed

ARTICLE OPEN

Check for updates

Predicting mortality risk for preterm infants using deep learning models with time-series vital sign data

Jiarui Feng[1,2], Jennifer Lee[3], Zachary A. Vesoulis[4]✉ and Fuhai Li[1,4]✉

Mortality remains an exceptional burden of extremely preterm birth. Current clinical mortality prediction scores are calculated using a few static variable measurements, such as gestational age, birth weight, temperature, and blood pressure at admission. While these models do provide some insight, numerical and time-series vital sign data are also available for preterm babies admitted to the NICU and may provide greater insight into outcomes. Computational models that predict the mortality risk of preterm birth in the NICU by integrating vital sign data and static clinical variables in real time may be clinically helpful and potentially superior to static prediction models. However, there is a lack of established computational models for this specific task. In this study, we developed a novel deep learning model, *DeepPBSMonitor* (Deep Preterm Birth Survival Risk Monitor), to predict the mortality risk of preterm infants during initial NICU hospitalization. The proposed deep learning model can effectively integrate time-series vital sign data and fixed variables while resolving the influence of noise and imbalanced data. The proposed model was evaluated and compared with other approaches using data from 285 infants. Results showed that the *DeepPBSMonitor* model outperforms other approaches, with an accuracy, recall, and AUC score of 0.888, 0.780, and 0.897, respectively. In conclusion, the proposed model has demonstrated efficacy in predicting the real-time mortality risk of preterm infants in initial NICU hospitalization.

npj Digital Medicine (2021)4:108 ; https://doi.org/10.1038/s41746-021-00479-4

INTRODUCTION

One in ten babies are born prematurely (defined as birth before 37 completed weeks of pregnancy) in the United States[1], and the complications of preterm birth are the leading cause of infant death[2,3]. Mortality is concentrated primarily among very low birth weight (VLBW) preterm infants (those weighing <1500 g and born before 32 weeks gestational age (GA)). Mortality rate is inversely proportional to GA and rapidly decreases from nearly 100% at 22 weeks to <1% at 32 weeks[4–7]. Moreover, preterm infants who survive often suffer from long-term health effects, including neurodevelopmental impairment and chronic lung disease. According to the 2019 Global Burden of Disease Study, neonatal disorders were the leading cause of disability-adjusted life-years (DALYs) worldwide (7% of all DALYs)[8].

Accurate estimation of mortality is an important component of antenatal counseling and assists healthcare providers in the allocation of resources. Using large cohorts of preterm infants, several different mortality prediction tools have been developed. The Clinical Risk for Infants and Babies (CRIB-II)[9,10] score uses sex, birth weight, GA, temperature at admission, and base excess to assess the mortality risk of babies upon neonatal intensive care unit (NICU) admission. The Score for Neonatal Acute Physiology-Perinatal Extension-II[11,12] includes mean blood pressure, lowest temperature, PO_2/FiO_2 ratio, lowest serum pH, multiple seizures, and urine output as important factors for estimating the mortality risk. More recently, the Transport Risk Index of Physiologic Stability, Version II (TRIPS-II)[13] uses temperature, blood pressure, respiratory status, and response to noxious stimuli as predictor variables—this measure was validated in 17,075 infants admitted between 2006 and 2008. Furthermore, the TRIPS-II score can be used to measure the change in mortality risk in the first 24 h. Finally, the NMR-2000 score[14] is a multivariate model with reverse stepwise selection, validated in >100,000 cases between 2010 and 2017.

While these tools are useful and have gained widespread use, the mortality estimates they provided have several limitations. For example, while overall mortality risk can be quantified, these models cannot assess the timing of highest mortality risk, thus confounding efforts to provide timely therapies. Second, additional information collected after birth may provide meaningful modification of the initial assessment of mortality risk (in either direction) permitting real-time prediction and the opportunity for intervention. Moreover, early detection of a change in mortality risk, particularly if the identified changes are subclinical, is critical to detect and prevent acute complications of prematurity, as such events are often acute and catastrophic (e.g., respiratory failure, sepsis, or intraventricular hemorrhage[2,15]).

Machine learning and deep learning approaches have been developed for prediction of mortality following preterm birth. Deep learning models have a growing presence in the healthcare field and often outperform traditional machine learning models[16–18]. For example, the Preterm Infants Survival Assessment (PISA) predictor was developed to predict preterm birth mortality but used only a few fixed variables[19]. In a recent study[20], the addition of time-series sensor data (e.g., systolic, diastolic, and mean blood pressure; oxygen saturation; and heart rate for temporal variables) achieved better results than the PISA predictor. However, even the newer model does not function in a real-time prediction manner. Moreover, the data are noisy and imbalanced because there are only a few risk signals in most time periods of preterm babies. The down-sampling, up-sampling, and weighting of samples does not improve the performance of such models. As a result, the application of standard deep learning models, like the general deep belief network[21] and long short-term memory (LSTM)

[1]Institute for Informatics, Washington University School of Medicine, St. Louis, MO, USA. [2]Department of Electrical and Systems Engineering, Washington University, St. Louis, MO, USA. [3]Washington University School of Medicine, St. Louis, MO, USA. [4]Department of Pediatrics, Division of Newborn Medicine, Washington University School of Medicine, St. Louis, MO, USA. ✉email: vesoulis_z@wustl.edu; Fuhai.Li@wustl.edu

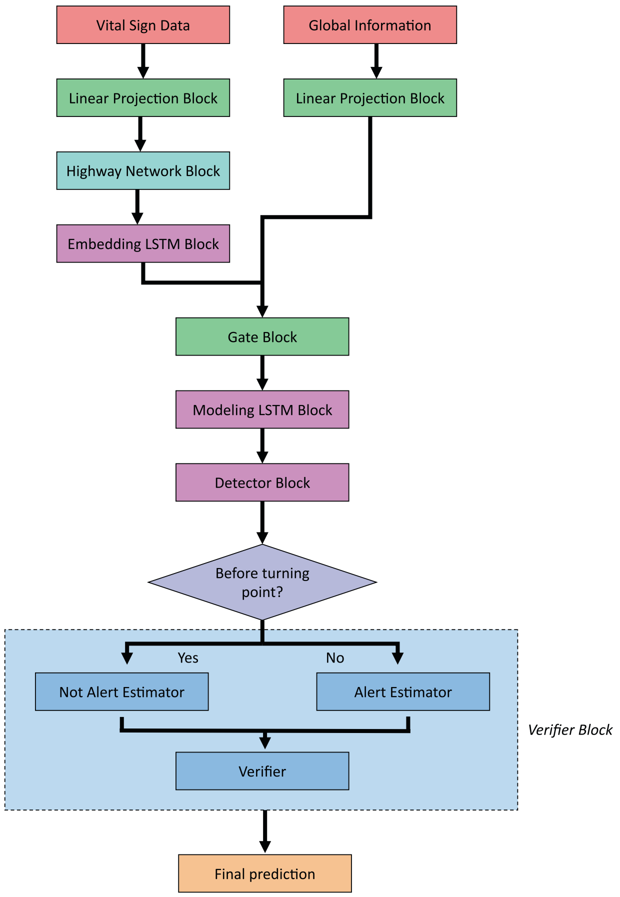

Fig. 1 Architecture overview of *DeepPBSMonitor*. The vital sign and global data were integrated via linear projection, highway network, LSTM, gate,detector and verifier blocks.

models[22], cannot achieve reliable and accurate predictions. We hypothesized that augmenting these basic deep learning approaches in an informed and goal-oriented manner would lead to significant improvements in performance.

In this study, we developed a novel deep learning model, *DeepPBSMonitor* (Deep Preterm Birth Survival Risk Monitor) (see Fig. 1), to predict the mortality of preterm births in a real-time manner by integrating time-series sensor data and fixed factors. These deep learning modules take the fixed variables and time-series signals as input and map the input signals into informative features for outcome prediction. The parameters of the mapping functions are the parameters of the deep learning model, which are initialized randomly and updated iteratively during the model training process. Specifically, a highway block was combined with an LSTM model to extract informative signals from time-series vital sign data. In addition, the fixed variables (e.g., birth weight, GA) were integrated using a gate block, which can compute weights for fixed variables and the time-series vital sign values in a single hidden dimension granularity. For this specific prediction task, we used detection–verification models and turning point detection to identify the state transition from "not alert" to "alert" in the course of a preterm infant's NICU stay. The model first identifies a possible turning point (from "not alert"/infant is OK to "alert"/infant should be checked on) in the signal sequence. Then, time-series data before and after the turning point is used to verify the prediction. The proposed model was evaluated and compared with other approaches using data from 285 infants. The comparison results showed that the proposed model out-performed other traditional models.

Table 1. Validation result of different imputation techniques.

	Accuracy	Recall	AUC	Accuracy*Recall
Bayesian ridge	0.898	0.756	0.901	0.679
Mean	0.894	0.704	0.859	0.629
Median	0.871	0.673	0.846	0.586
Mode	0.910	0.688	0.857	0.626
Decision tree	0.884	0.770	0.890	0.681
Multiple imputation	0.909	0.732	0.897	0.665

RESULTS

Data cohort

During the study period, a total of 6271 infants were admitted to the NICU, 1465 of which met GA and weight criteria to be considered a VLBW infant. Vital sign recording data were available for 525 of those infants. After examination of the recordings, 221 infants were excluded due to unreadable or corrupted recordings and 19 infants were excluded for truncated recordings (<6 h).

After exclusion, the final cohort was 285 infants, 65 of whom died. The mean GA was 26.7 ± 2.3 weeks, mean birth weight was 929 ± 281 g, and the cohort was 51% male. The median age at death was 10 days (range 0–387 days) and the median CRIB-II score was 10 (range 2–18). As would be expected, infants who died were significantly more premature (24.8 vs 27.2 weeks, $p < 0.01$) and were of lower birth weight (687 vs. 1000 g, $p < 0.01$). Detailed cohort characteristics can be found in Supplementary Table 1 and a diagram of inclusion/exclusion in Supplementary Fig. 1.

Missing data imputation

Missing data imputation is an important consideration in any data analytics project. Inappropriate imputation will introduce bias into the dataset. In this study, we tested and validated various missing data imputation techniques. Specifically, both single data imputation and multiple imputation[23] were evaluated. The single data imputation methods included mean, median, mode, decision tree, and Bayesian ridge imputation. For mean, median, and mode imputation, we used the mean, median, and mode of a given feature in each sample to replace the missing value. For decision tree and Bayesian ridge imputation, we used the corresponding model to predict the missing value. For multiple imputation, we used Bayesian ridge to sample five different datasets by sampling different posteriors each time. Next, we used fourfold cross-validation and reported the average metrics. For multiple imputation, the average of all five datasets is reported. We fixed the model hyperparameters as $n_h = 128$, $l_{highway} = 1$, $l_{cnn} = 1$, $p_d = 0.1$, $\beta = 1$. The validation metric was chosen as *accuracy*recall*, as we were balancing total accuracy with the performance in "not alert" time steps. During training, we evaluated the model with a validation dataset at regular intervals (every 50 training steps). The result is shown in Table 1.

We can see that our model performs well with all six different imputation techniques, which means that our model is robust to potential bias introduced by missing data imputation. Among these six techniques, Bayesian ridge and decision tree achieved the best results. We therefore selected Bayesian ridge as our imputation method.

Model validation

To find the best model parameters, we fine-tuned the following hyperparameters: n_h, $l_{highway}$, l_{cnn}, p_d, and β using fourfold cross-validation. The validation metric was chosen as *accuracy*recall*. During training, we evaluated the model with a validation dataset at regular intervals (every 50 training steps). The model

Table 2. Validation results for hidden size.

Hyperparameter	Accuracy	Recall	Accuracy*Recall
$n_h = 64$	0.888	0.780	0.6926
$n_h = 128$	0.898	0.756	0.6788
$n_h = 256$	0.918	0.724	0.6646
$n_h = 512$	0.932	0.684	0.6374

Table 3. Validation results for highway layers.

Hyperparameter	Accuracy	Recall	Accuracy*Recall
$l_{highway} = 1$	0.888	0.780	0.6926
$l_{highway} = 2$	0.929	0.656	0.6092
$l_{highway} = 3$	0.905	0.725	0.6568

parameters that result in the best validation metrics were saved. First, we fine-tuned the n_h. We set $l_{highway} = 1$, $l_{cnn} = 1$, $p_d = 0.1$, $\beta = 1$. The result is shown in Table 2. For hidden size, $n_h = 64$ achieved the best performance.

Then, we fine-tuned the $l_{highway}$. We set $n_h = 64$, $l_{cnn} = 1$, $p_d = 0.1$, $\beta = 1$. The result is shown in Table 3. We can see that $l_{highway} = 1$ achieved the best performance. Next, we fine-tuned the l_{cnn}. We set $n_h = 64$, $l_{highway} = 1$, $p_d = 0.1$, $\beta = 1$. The result is shown in Table 4. The parameter $l_{cnn} = 1$ had better performance than $l_{cnn} = 2$. Next, we fine-tuned p_d. We set $l_{cnn} = 1$, $l_{highway} = 1$, $n_h = 64$, $\beta = 1$. The result is shown in Table 5. The parameter $p_d = 0.10$ achieved the best performance. Finally, we adjusted the β. The result is shown in Table 6. We find that $\beta = 1$ is the best parameter. The detailed cross-validation results can be found in Supplementary Table 2.

Model prediction result

Based on our validation results, the hyperparameters of our final model were set as $n_h = 64$, $l_{cnn} = 1$, $l_{highway} = 1$, $p_d = 0.1$, $\beta = 1$. The prediction results per fold are provided in Tables 7–10. The detailed predictions for the validation set per fold can be found in Supplementary Figs. 1–4.

Performance comparison

To further evaluate the performance of the proposed model, we compared our model with the existing CRIB-II score and a simple deep neural network (DNN).

Fourfold cross-validation was applied to both the proposed model and the DNN. Results are in Table 11. As shown below, our proposed model achieved the best prediction performance in terms of accuracy, recall, and area under the characteristic curve (AUC) metrics.

DISCUSSION

In this manuscript, we proposed a novel deep learning model, Deep Preterm Birth Survival Risk Monitor or *DeepPBSMonitor*. This model utilizes an LTSM deep learning approach to examine continuous vital sign data and identify "alert" periods where the model detects underlying changes in the vital signs concerning for an increased risk of mortality. Another novel innovation of this model is the addition of a module to detect turning points, where infants transition from a low- to a high-risk state. When compared to an existing mortality prediction model (CRIB-II) and a simple DNN model, *DeepPBSMonitor* provides superior accuracy (88.8%) with the greatest AUC (0.897) (Fig. 2).

Table 4. Validation results for CNN layers.

Hyperparameter	Accuracy	Recall	Accuracy*Recall
$l_{cnn} = 1$	0.888	0.780	0.6926
$l_{cnn} = 2$	0.877	0.770	0.6748

Table 5. Validation results for dropout rates.

Hyperparameter	Accuracy	Recall	Accuracy*Recall
$p_d = 0.05$	0.877	0.710	0.6226
$p_d = 0.1$	0.888	0.780	0.6926

Table 6. Validation results for loss function constants.

Hyperparameter	Accuracy	Recall	Accuracy*Recall
$\beta = 1$	0.888	0.780	0.6926
$\beta = 2$	0.898	0.746	0.6672

Table 7. Confusion matrix of final model on first fold validation set.

	PREDICT		
		Alert	Not alert
	Alert	TP:903	FN:249
TRUE	Not alert	FP:6305	TN:56471

Table 8. Confusion matrix of final model on second fold validation set.

	PREDICT		
		Alert	Not alert
	Alert	TP:737	FN:127
TRUE	Not alert	FP:9583	TN:50090

Table 9. Confusion matrix of final model on third fold validation set.

	PREDICT		
		Alert	Not alert
	Alert	TP:813	FN:267
TRUE	Not alert	FP:3853	TN:57694

Table 10. Confusion matrix of final model on fourth fold validation set.

	PREDICT		
		Alert	Not alert
	Alert	TP:1154	FN:430
TRUE	Not alert	FP:6347	TN:49821

Mortality prediction is of great value to providers and families, as this information can guide counseling and decision-making during NICU care. Existing approaches utilize fixed clinical factors deriving from the immediate perinatal period. While these

Table 11. Performance comparison of CRIB-II, DNN, and proposed model.

	CRIB-II (per Infant)	DNN (independent time point with fourfold cross-validation)	Proposed model (time sequence prediction with fourfold cross-validation
Accuracy	0.696	0.758	0.888
Recall	0.754	0.723	0.780
AUC	0.751	0.791	0.897

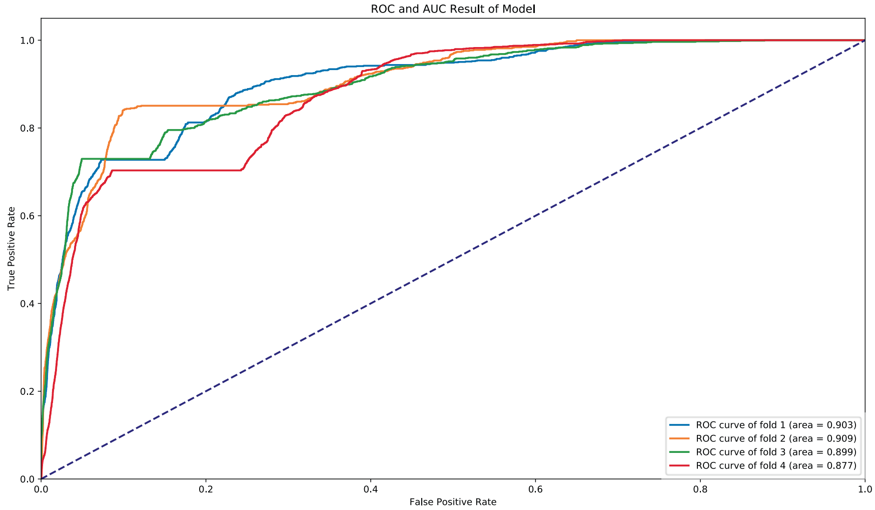

Fig. 2 The ROC curve and AUC of our final model on the four validation sets. The mean AUC of the model is 0.897. The plot of predictions for each infant in four validation sets are shown in Supplemental Figs. 1–4.

calculations are simple and fast, they provide an incomplete picture. For example, although an infant born at 23 weeks of gestation has a high risk of mortality, the instantaneous risk of mortality is not constant throughout hospitalization but instead is heavily concentrated in the first 1–2 weeks of life. Furthermore, when all other factors are held constant (gestation age, birth weight, severity of metabolic acidosis, etc.), it is impossible for providers to identify which of these high-risk infants are at the greatest risk. The addition of continuous vital sign data is enormously valuable in this endeavor, as changes in physiologic state are often the first manifestation of illness.

There are several other mortality prediction models, namely, NMR-2000 and TRIPS-II. While these models take dynamic factors into account, have comparable performance, and have been externally validated, they have limitations addressed by the model proposed in this manuscript. Both NMR-2000 and TRIPS-II utilize factors measured within the first 24 h after birth to predict in-hospital (total NICU) mortality. This is in contrast to our proposed model, which provides a continuous calculation of mortality risk that is updated throughout an infant's NICU stay. Second, this new proposed model was developed specifically for use in the VLBW population, a subgroup with the greatest proportional risk of death in the NICU. Based on provided and estimated data from the NMR-2000 and TRIPS-II studies, many VLBW and extremely LBW infants were included in those studies; however, they comprised only 20% of the total samples. While this makes these tools more generalizable for a total NICU population, it may impact performance in this specific high-risk subgroup. Given the focus on developing a tool for exclusive use in the VLBW and ELWB population, an enriched cohort of these infants is necessary.

Routine vital sign monitoring, however, results in a veritable forest of false alarms with very few true pathologic events. These repeated false alarms quickly lead to alarm fatigue, further damaging the signal-to-noise ratio of continuous monitoring. Deep learning can be employed to identify subtle patterns in vital signs that are readily lost in human interpretation. *DeepPBSMonitor* builds on previous vital sign analytic methodology by focusing on the transition between low- and high-risk states. This inflection point could be of great potential value to providers. Rather than alarming with movement artifact or brief excursion of values outside of programmed alarm limits, this tool identifies significant paradigm shifts in the trajectory of the patient based on the composite evaluation of multiple sources of information.

There are a number of potential limitations for this project. First, as with all machine learning applications, a larger sample size would improve the accuracy and reliability of the model and reduce potential bias from inherent characteristics of the chosen sample. We made effective use of fourfold cross-validation to reduce this concern, but larger samples (on the order of the several thousand VLBW infants included in TRIPS-II and NMR-2000 models) would provide for a greater degree of confidence in prediction accuracy. Similarly, this model was developed from the patient population at a single institution. Although patients come from a variety of locations (urban, suburban, rural), the sample is limited to the hospital catchment area and all patients receive care by the same set of providers. Future research should include a more geographically diverse sample, ideally multinational, to account for all sources of variability.

Meanwhile, missing data is another source of bias that may compromise model validity. To mitigate this issue, we utilized multiple missing data imputation methods on the model, which considerably reduces the risk of bias. Second, *DeepPBSMonitor* does not identify the underlying mechanism of increased mortality risk. Alerting providers to this concern is an important first step but contextualizing the source of increased risk will be a key part of moving this approach to practical clinical use. Third, a small number of infants were transferred to other hospitals before their initial NICU discharge. Although it is possible that some of

these infants died before discharge, such transfer occurs almost exclusively to a lower level of care for completion of convalescence. Mortality in this clinically stable population is exceedingly unlikely.

In conclusion, the proposed deep learning model has demonstrated efficacy in predicting the mortality risk of preterm infants in the NICU and is superior to existing clinical models of mortality risk prediction and simple deep learning models. The proposed model effectively integrates time-series vital sign data and fixed variables while resolving the influence of noise and imbalanced data.

METHODS

Cohort selection and clinical data

All infants admitted to the NICU at St. Louis Children's Hospital, a level IV NICU serving urban, suburban, and rural populations have vital sign data prospectively archived into a research database (BedMaster EX, Excel Medical, Jupiter, FL). For this convenience sample of infants admitted between 2012 and 2018, we included all infants who were born prior to 32 completed weeks of gestation and had at least 6 h of recorded vital sign data. Given the novel nature of this study, a priori sample size calculation was not performed. Only inborn and outborn preterm infants in their initial NICU hospitalization were included; infants with cyanotic heart disease and those readmitted after hospital discharge are initially admitted to other units of the hospital and did not have any collected vital sign data (thus were not included). Comprehensive clinical factors including sex, race, GA, weight, length of stay, age at admission, and outcome were collected. The study protocol was reviewed and approved by the Washington University Human Research Protection Office. Given the retrospective nature of the study, waiver of consent was granted.

Raw vital sign data were recorded using Philips IntelliVue MP70 or MX800 patient monitors (Philips Medical Andover, MA) as the time-integrated mean with recorded rate of 1 Hz. All infants had vital signal data for heart rate (HR), respiratory rate (RR), and oxygen saturation (SPO_2). In addition, all infants had either non-invasive systolic (NIBP-S), non-invasive mean (NIBP-M), and non-invasive diastolic (NIBP-D) blood pressure measurements or invasive arterial lines, which provided arterial systolic (ART-S), arterial mean (ART-M), and arterial diastolic (ART-D) blood pressure measurements.

Labeling of samples

For model training and evaluation, the time-series data were labeled as "alert" and "not alert." For infants who died, each time point in the final 6 h prior to death was labeled as "alert" and the remaining time points were labeled as "not alert." For the babies who survived, all time points were labeled as "not alert." The final objective of model was to predict the correct label for each time point.

Model architecture of *DeepPBSMonitor*

The model architecture of *DeepPBSMonitor* is shown in Fig. 1. Specifically, the model consists of the following blocks: linear projection block, highway network block, embedding LSTM block, gate block, modeling LSTM block, detector block, and verifier block.

The innovation of the *DeepPBSMonitor* model is driven by the integration of time-series vital sign data with fixed global variables, and a reduction in the false prediction rate by introducing the state turning point detection (especially useful with imbalanced data, as is the case with this specific preterm birth survival and mortality risk prediction). Specifically, the highway network and LSTM blocks were used to capture the most informative signal patterns from the time-series vital sign data. The global variables and identified hidden signal patterns were then combined using a gate block. More importantly, instead of directly predicting the mortality risk over time, we designed the novel state turning point detection and verification model for this specific task. This is based on the hypothesis that, at the turning point, the stable (not alert) state of preterm births transitions to a non-stable (alert) state. The model first checks every 5 min if a turning point has appeared. If there is a turning point, the time-series data are divided into two segments, i.e., before and after the turning point. The verifiers then use these two segments of signals to verify the detection. If there is no turning point in the time sequence, the model should report the final point (padded segment) as the turning point prediction. Moreover, by introducing detection loss, the model can be more effective in avoiding false positive predictions caused by the imbalanced data. Model parameters were defined as follows: n_h is the hidden size of model, $l_{highway}$ is the number of highway layer in the modeling LSTM block, l_{cnn} is the number of convolutional neural network (CNN) layer in the detector block, p_d is the dropout probability, and β is the weight in loss function.

Prediction features and data preprocessing

For raw vital sign data, we selected HR, RR, SPO_2, and ART-M or NIBP-M as predictive features. The recording for all infants was processed at a consistent length. For preterm babies with data spanning >80 h, the data were truncated, and only the last 80 h were used in the predictive models. For patients with <80 h, the data were padded with zeros in the missing portion of the recording, and that section was masked during model training and prediction.

The valid ranges of each vital signal were defined as follows: HR (0, 250], RR (0, 120], SPO_2 (0, 100], ART-M [10, 90], NIBP-M (10, 90]. To better handle missing data in the time sequence, we applied various missing data imputation techniques and compared the performance of each technique. The details regarding missing value imputation and evaluation are further discussed in the "Results" section.

To reduce noise, the rolling mean of each vital sign data was used with a range of 5 min. For each sample (with 80 h data), the vital sign data was divided into 959 segments ((80 h × 60 min/5 min) − 1 = 959). The reason for subtracting 1 from the segments is that the first 5 min were used to compute the first rolling mean value. For each segment, 1500 vital sign signals were acquired (5 features × 300 s (5 min) = 1500). Finally, we padded the end of each sample with one segment where all features equaled zero. The final length of samples was therefore 960.

In addition to the time-series data, the sex, race, GA, and weight were used in the prediction model. GA and weight have a complex non-linear relationship with mortality[7]; thus, both variables were considered in the model. For modeling purposes, sex and race were converted into dummy variables. To mitigate the effect of truncating, we also created an additional feature to indicate the length of the infant's stay in the NICU prior to the start of the model evaluation period (which consisted of the final 80 h of a given stay). Finally, there were nine variables to characterize the fixed/global information of preterm infants. The prediction features are listed in Table 12.

The *DeepPBSMonitor* model

Let $X \in R^{S \times n_v}$ represent the vital signal data, where S is the length of sequence and n_v is the number of signals in each time step. Here $S = 960$ and $n_v = 1500$. Let $G \in R^{n_g}$ denote the fixed global information, where $n_g = 9$ is the number of global variables.

Linear projection block. The model will first project both vital signal data and global data to hidden size n_h by linear projection block. Let $W_x \in R^{n_v \times n_h}$, $b_x \in R^{n_h}$, $W_g \in R^{n_g \times n_h}$, and $b_g \in R^{n_h}$ be trainable parameters; the linear projection block processes the data with:

$$H_X = \mathrm{ReLU}(XW_x + b_x) \quad (1)$$

$$h_g = \mathrm{ReLU}\left(GW_g + b_g\right) \quad (2)$$

where $H_X \in R^{S \times n_h}$ and $h_g \in R^{n_h}$ are vital signal features and global information, and ReLU is the activation function. Finally, in order to integrate the global information into each time step, we tile the h_g by S times to get $H_g \in R^{S \times n_h}$:

$$H_g = \mathrm{Tile}\left(h_g\right) \quad (3)$$

Highway network block. Next, the vital signal features outputted from the linear projection block will be further processed by the highway network block. The highway network block contains $l_{highway}$ highway layers, where $l_{highway}$ is a hyperparameter. Let the input of each highway layer be H_X; the computation in each layer is then as follows:

$$g = \sigma\left(H_X W_g + b_g\right) \quad (4)$$

$$t = \mathrm{ReLU}(H_X W_t + b_t) \quad (5)$$

$$\overline{H}_X = g^* t + (1 - g)^* H_X \quad (6)$$

Table 12. Prediction features.

	Description	Type
HR	Heart rate	Vital sign data
RR	Respiratory rate	Vital sign data
SPO2	Oxygen saturation	Vital sign data
ART-M	Arterial blood pressure—mean	Vital sign data
NIBP-M	Non-invasive blood pressure—mean	Vital sign data
Sex	The sex of babies	Fixed variable
GA	Gestational age of babies	Fixed variable
Birth weight	The weight when babies were born	Fixed variable
Time	Length of the infant's stay in the NICU prior to the start of the model evaluation period	Fixed variable
Is Asian?	Whether the baby is Asian	Fixed variable
Is Black?	Whether the baby is Black or African American	Fixed variable
Is Hispanic?	Whether the baby is Hispanic	Fixed variable
Is White?	Whether the baby is White or Caucasian	Fixed variable
Is Other race?	Whether the race of baby is unknown	Fixed variable

where $W_g \in R^{n_h \times n_h}$, $W_t \in R^{n_h \times n_h}$, $b_g \in R^{n_h}$, and $b_t \in R^{n_h}$ are trainable parameters, * is Hadamard product, and σ is sigmoid function. $\overline{H}_X \in R^{S \times n_h}$ is the output of the highway layer. Highway layers are useful to capture relative information from each time step.

Embedding LSTM block. To further capture information among different time steps, we design an embedding LSTM block. The embedding LSTM block contains three LSTM layers with residual connections. Let the input and the hidden state in the LSTM block at time step t be $\overline{h}_{xt} \in R^{n_h}$ and $\overline{h}_{ht} \in R^{n_h}$, respectively. The computation in each LSTM layer is then as follows:

$$\overline{h}_{ht+1} = \text{LSTM}(\overline{h}_{xt}, \overline{h}_{ht}) \tag{7}$$

where $\overline{h}_{ht+1} \in R^{n_h}$ is the hidden state in time step $t+1$. LSTM layers are used to extract information from whole sequences. Meanwhile, we introduce residual connections for each LSTM layer. The output for each residual LSTM layer is:

$$\overline{h}^{i+1}_{xt\quad ht} = \text{LSTM}\left(\overline{h}^{i}_{xt}, \overline{h}^{i}_{ht}\right) + \overline{h}^{i}_{xt} \tag{8}$$

where $\overline{h}^{i+1}_{xt} \in R^{S \times n_h}$ is the output in the ith residual LSTM layer. Finally, we integrate information from all residual LSTM layers via:

$$L_X = \text{Concat}\left(\overline{h}^{i}_{x} \text{ for } i = 1, 2, 3\right) \tag{9}$$

$$P_l = \text{Softmax}\,(L_X W_l + b_l) \tag{10}$$

$$\overline{L}_X = \sum_{i=1}^{3} P_{li} \overline{h}^{i}_{x} \tag{11}$$

where $W_l \in R^{3^* n_h \times 3}$ and $b_l \in R^{l_{\text{LSTM}}}$ are trainable parameters, $L_X \in R^{S \times 3^* n_h}$ is the concatenation of the output from three residual LSTM layers, $P_l \in R^{S \times 3}$ is an indication of how much information from each residual LSTM layer should be integrated into the final information representation, and $\overline{L}_X \in R^{S \times n_h}$ is the output of embedding LSTM block.

Gate block. To integrate vital sign information and global information, a gate block is designed. Let $\overline{L}_X \in R^{S \times n_h}$ be the vital sign embedding information outputted by the embedding LSTM block. The point-wise gate state p is computed by:

$$p = \sigma\left(\text{Concat}\left(\overline{L}_X, H_g, \overline{L}^{*}_X H_g, \overline{L}_X - H_g\right) W_m + b_m\right) \tag{12}$$

where $W_m \in R^{4n_h \times n_h}, b_m \in R^{n_h}$ are trainable parameters, Concat is a concatenate function, and $p \in R^{S \times n_h}$. In this step, we want to capture how much information should be retained in vital sign information and global information, respectively, among each hidden dimension. Next, we use p to integrate vital signal information and global information.

$$M = p^{*}\overline{L}_X + (1 - p)^{*} H_g \tag{13}$$

where $M \in R^{S \times n_h}$ is the output of the gate block.

Modeling LSTM block. Next, we apply a modeling LSTM block to generate final state information for each time step. The modeling LSTM block contains one LSTM layer, similar to the embedding LSTM layer. This final state information captures the total risk of infant in time step t.

Detector block. Instead of directly predicting the state distribution for each time step, we designed a detection–verification mechanism using detector and verifier blocks. The responsibility of the detector block is to discover whether there is a turning point of an infant's state from "not alert" to "alert," and if so, determine where it is. Since the state of the current time step is more related to the previous time steps near this step than to those far earlier, we apply depthwise separable convolution to focus on local information. The depthwise separable convolution is more memory efficient and has better generalization power. In detail, the detector block contains l_{cnn} depthwise separable convolution layers. Let the output of modeling LSTM block be $\overline{M}$. The computation in the convolution layer is then as follows:

$$F = \text{ReLU}\left(\text{Batch normalization}\left(\text{CNN}\left(\overline{M}\right)\right)\right) \tag{14}$$

where $F \in R^{S \times n_h}$ captures the local information in each time step. In our model, the output channel in CNN is n_h and kernel size is 7. Next, the turning point is predicted with:

$$\text{TP} = \text{Softmax}\left(FW_{\text{tp}} + b_{\text{tp}}\right) \tag{15}$$

where $W_{\text{tp}} \in R^{n_h \times 1}$ and $b_{\text{tp}} \in R$. $\text{TP} \in R^S$ is the distribution of the turning point among all time steps. If the sample has no turning point, the distribution should be maximized in the last time step (padded segment). During training, the turning point (tp) is chosen as the time step with the maximum probability. During evaluation, to avoid noise detection, we designed a turning point selection rule. Specifically, if the probability of time step with maximum probability is >0.5, we directly choose this time step as our turning point. Otherwise, several candidates with maximum probability are selected. In this case, the number of candidates is computed by the length of sequence: Round(length of time sequence/960). Then the candidate who is the last time step among all candidates is chosen as the turning point, as long as the probability of that point is greater than 4/length of time sequence. If not, we assume that the baby does not have a turning point.

Verifier block. After the turning point in the sequence is detected, a verifier block is used to verify the detection result in the detection block. To be more specific, we divide the data into two parts: time steps before a given turning point, and time steps after. We assume that these two parts will have different distributions of mortality risk. Then we estimate each distribution and derive the risk prediction. However, the model will sometimes give a false turning point and therefore trigger an unwarranted jump in risk. To mitigate the effect of false turning points, the verifier block is designed to generate a final prediction based on the results of two separate neural network layers. The computation in the verifier block is as

Published in partnership with Seoul National University Bundang Hospital

follows:

$$P_b = \overline{M}W_{nw} + b_{nw} \quad (16)$$

$$P_a = \overline{M}W_w + b_w \quad (17)$$

$$P = \text{Softmax}(\text{concat}(P_b[:\text{tp}], P_a[\text{tp}:])) \quad (18)$$

where $W_{nw} \in R^{n_h \times 2}$, $b_{nw} \in R^2$, and $W_w \in R^{n_h \times 2}$ and $b_w \in R^2$ are trainable parameters, $P_b \in R^{S \times 2}$ and $P_a \in R^{S \times 2}$ are the risk prediction for time steps based on the distributions before and after the turning point. Next, we concatenate P_b (before turning point) and P_a (after turning point) to get our final risk prediction. Then this prediction will be verified as follows:

$$P_{\text{verified}} = \text{Softmax}(\text{concat}(P_b[:\text{tp}], P_a[\text{tp}:]) - \text{LSTM}(P_b) + \text{LSTM}(P_a)) \quad (19)$$

The model will use P_{verified} as the final risk prediction.

Loss function. To support the detection–verification mechanism, we designed a unique loss function. The loss is divided into three parts—the detection loss, the prediction loss, and the verification loss. The detection loss is used to measure how accurately the detector predicts the turning point. To mitigate false detection, we apply focal loss[24] with greater weight on the last padded time step:

$$L_{\text{detection}} = weighted\_focal\_loss(PTP, real\_PTP) \quad (20)$$

where *real_PTP* is the real turning point and *PTP* is the predicted turning point. The parameter γ and α of focal loss are set to be 2 and 1, respectively. The weight of each time step is 1 if it is not the last padded step, or 1.3 if it is. The prediction loss measures the difference between the prediction and true risk:

$$L_{\text{prediction}} = weighted\_nll\_loss(P, real\_P) \quad (21)$$

where *weighted_nll_loss* is the weighted negative log-likelihood loss, and *real_P* is the real final state for each time step. The verification loss measures the difference between the verified prediction and true risk:

$$L_{\text{verification}} = weighted\_nll\_loss(P_{verified}, real\_P) \quad (22)$$

The final loss is designed as:

$$L = \beta L_{\text{detection}} + L_{\text{prediction}} + 2L_{\text{verification}} \quad (23)$$

where β is a hyperparameter.

Model training

To train the *DeepPBSMonitor*, we use Adadelta as our optimizer, with a learning rate set at 0.5. To address overfitting, L2 weight decay was applied with parameter $\lambda = 3 \times 10^{-7}$. A dropout layer was applied after each block with drop probability p_d. Batch size was set as 6 and epoch was set as 60.

During training, to deal with the unbalanced sequence problem, we used *weighted_nll_loss* in prediction loss and verification loss. To be more specific, we assigned different weights for each "not alert" and "alert" time step of each infant. The weight is varied in each fold based on the number of "not alert" and "alert" time steps in the training data. The weight of a "not alert" time step was set as 1 and the weight of an "alert" time step was set as:

$$\text{Weight}_{\text{alert}} = \frac{\text{number of not alert time steps}}{\text{number of alert time steps}} * 0.1153$$

In each training epoch, we reconstructed the training set. The reconstructed training set contains all infants who eventually died and $n/6$ randomly selected infants who eventually survived, where n is the number of infants who eventually died in the training dataset.

To smooth the training and validation procedure, we clipped the gradient with threshold 5.0 before each back-propagation step. Meanwhile, exponential moving average (EMA) was applied on all trainable variables with a decay rate $\mu = 0.999$. To be more specific, model weights after back-propagation step t were W_t and model weights after EMA in step t were E_t. After back-propagation step $t+1$, we derived model weights W_{t+1} from W_t. The new EMA model weights were updated with the function $E_{t+1} = (1-\mu)E_t + W_{t+1}$. If we reached the validation procedure at step t, model weights E_t were used instead of W_t. Finally, we implemented our model in Python using Pytorch[25] and carried out cross-validation on an MSI GeForce RTX 2070 GPU Super (Micro-Star International, Zhonghe, New Taipei, Taiwan) on a local machine with 8 GB memory.

Data imputation

The decision tree and Bayesian ridge imputation are implemented using the scikit-learn package in Python. For multiple imputation, the parameter sample_posterior is set to be true and different random states are applied for each imputed dataset.

Model comparison

CRIB-II Score. The CRIB-II score is widely used tool for evaluating initial mortality risk in preterm infants. It considers the birth weight, GA, admission body temperature, base excess, and sex of the baby and results in a numerical score. CRIB-II scores ≥11 have been associated with a significantly increased risk of mortality[10,26]. To evaluate the performance of our model, predictions using CRIB-II score were made by labeling each baby with the "alert" label if the infant had a CRIB-II score ≥11; otherwise, we labeled the baby as "not alert." For CRIB-II scores, we only consider the risk in the scalar of per infant, not per time step. This approach is consistent with real-world usage of the CRIB-II score, where it is calculated once at the time of NICU admission but is not updated over time (as the factors do not change). Then we compared the result with the true information and computed accuracy, recall, and AUC. A small number of infants do not have CRIB-II score due to missing components (generally admission temperature); for these, we assign the infant a random value in the range of normal admission temperatures (35–38 °C).

Simple DNN. We also compared our proposed model with a simple DNN. The DNN contains four linear projection layers, the hidden size of each layer is 512, 128, 32, and 2, respectively. The activation function was ReLU except for the final layer, which used the Softmax function to convert scores to probability distributions. Similarly, a dropout layer and L2 weight decay were applied to deal with overfitting. The optimizer was Adadelta with the learning rate set as 0.5. Additionally, to deal with the imbalanced data, we also evaluated different weights to "alert" and "not alert" points. Specifically, the weights of "alert" samples were set as number_of_not_alert_time_steps/number_of_alert_time_steps. However, DNNs do not have the ability to make predictions based on whole time series or sequences. Therefore, we considered data as independent data points; for each data point, corresponding global features were concatenated to construct final features. The feature dimension of each data segment was thus 1509. During the training process, the batch size was set as 128 and epoch time was 10. The model parameters with the best Accuracy*Recall were used for comparison.

Reporting summary

Further information on research design is available in the Nature Research Reporting Summary linked to this article.

DATA AVAILABILITY

Although privacy restrictions prevent publication of non-aggregated or raw clinical data, aggregated and de-identified data are available upon reasonable request to the corresponding author.

CODE AVAILABILITY

The source code for the *DeepPBSMonitor* model is available at: https://github.com/fuhailab/PreTermMortalityPrediction.

Received: 10 November 2020; Accepted: 21 June 2021;
Published online: 14 July 2021

REFERENCES

1. Kochanek, K. D., Murphy, S. L., Xu, J. & Arias, E. Deaths: final data for 2017. *Natl Vital Stat. Rep.* **68**, 1–77 (2019).

2. Muhe, L. et al. Major causes of death in preterm infants in selected hospitals in Ethiopia (SIP): a prospective, cross-sectional, observational study. *Lancet Glob. Health* **7**, E1130–E1138 (2019).
3. Liu, L. et al. Global, regional, and national causes of under-5 mortality in 2000-15: an updated systematic analysis with implications for the Sustainable Development Goals. *Lancet* **388**, 3027–3035 (2016).
4. Manuck, T. A. et al. Preterm neonatal morbidity and mortality by gestational age: a contemporary cohort. *Am. J. Obstet. Gynecol.* **215**, 103.e1–103.e14 (2016).
5. Horbar, J. D. et al. Trends in mortality and morbidity for very low birth weight infants, 1991-1999. *Pediatrics* **110**, 143–151 (2002).
6. Wilcox, A. J. & Skjaerven, R. Birth weight and perinatal mortality: the effect of gestational age. *Am. J. Public Health* **82**, 378–382 (1992).
7. Boghossian, N. S., Geraci, M., Edwards, E. M. & Horbar, J. D. Morbidity and mortality in small for gestational age infants at 22 to 29 weeks' gestation. *Pediatrics* **141**, e20172533 (2018).
8. Vos, T. et al. Global burden of 369 diseases and injuries in 204 countries and territories, 1990–2019: a systematic analysis for the Global Burden of Disease Study 2019. *Lancet* **396**, 1204–1222 (2020).
9. Parry, G., Tucker, J. & Tarnow-Mordi, W. CRIB II: an update of the clinical risk index for babies score. *Lancet* **361**, 1789–1791 (2003).
10. Ezz-Eldin, Z. M., Hamid, T. A. A., Youssef, M. R. L. & Nabil, H. E.-D. Clinical Risk Index for Babies (CRIB II) scoring system in prediction of mortality in premature babies. *J. Clin. Diagn. Res.* **9**, SC08–SC11 (2015).
11. Richardson, D. K., Corcoran, J. D., Escobar, G. J. & Lee, S. K. SNAP-II and SNAPPE-II: simplified newborn illness severity and mortality risk scores. *J. Pediatr.* **138**, 92–100 (2001).
12. Harsha, S. S. & Archana, B. R. SNAPPE-II (Score for Neonatal Acute Physiology with Perinatal Extension-II) in predicting mortality and morbidity in NICU. *J. Clin. Diagn. Res.* **9**, SC10–SC12 (2015).
13. Lee, S. K. et al. Transport Risk Index of Physiologic Stability, version II (TRIPS-II): a simple and practical neonatal illness severity score. *Am. J. Perinatol.* **30**, 395–400 (2013).
14. Medvedev, M. M. et al. Development and validation of a simplified score to predict neonatal mortality risk among neonates weighing 2000 g or less (NMR-2000): an analysis using data from the UK and The Gambia. *Lancet Child Adolesc. Health* **4**, 299–311 (2020).
15. Schindler, T. et al. Causes of death in very preterm infants cared for in neonatal intensive care units: a population-based retrospective cohort study. *BMC Pediatr.* **17**, 59 (2017).
16. Rajkomar, A. et al. Scalable and accurate deep learning with electronic health records. *npj Digit. Med.* **1**, 18 (2018).
17. Shickel, B., Tighe, P. J., Bihorac, A. & Rashidi, P. Deep EHR: a survey of recent advances in deep learning techniques for electronic health record (EHR) analysis. *IEEE J. Biomed. Health Inform.* https://doi.org/10.1109/JBHI.2017.2767063 (2018).
18. Miotto, R., Li, L., Kidd, B. A. & Dudley, J. T. Deep patient: an unsupervised representation to predict the future of patients from the electronic health records. *Sci. Rep.* https://doi.org/10.1038/srep26094 (2016).
19. Podda, M. et al. A machine learning approach to estimating preterm infants survival: development of the Preterm Infants Survival Assessment (PISA) predictor. *Sci. Rep.* **8**, 13743 (2018).
20. Jaskari, J. et al. Machine learning methods for neonatal mortality and morbidity classification. *IEEE Access* **8**, 123347–123358 (2020).
21. Hinton, G. E., Osindero, S. & Teh, Y.-W. A fast learning algorithm for deep belief nets. *Neural Comput.* **18**, 1527–1554 (2006).
22. Hochreiter, S. & Schmidhuber, J. Long short-term memory. *Neural Comput.* **9**, 1735–1780 (1997).
23. Hayati Rezvan, P., Lee, K. J. & Simpson, J. A. The rise of multiple imputation: a review of the reporting and implementation of the method in medical research. *BMC Med. Res. Methodol.* **15**, 30 (2015).
24. Lin, T., Goyal, P., Girshick, R., He, K. & Dollár, P. Focal loss for dense object detection. In *2017 IEEE International Conference on Computer Vision (ICCV)* 2999–3007 (IEEE, 2017).
25. Paszke, A. et al. PyTorch: an imperative style, high-performance deep learning library. In *Advances in Neural Information Processing Systems 32* 8024–8035 (Curran Associates, 2019).
26. de Felice, C., del Vecchio, A. & Latini, G. Evaluating illness severity for very low birth weight infants: CRIB or CRIB-II? *J. Matern. Neonatal Med.* **17**, 257–260 (2005).

ACKNOWLEDGEMENTS

Support for this project was provided by NIH/NINDS though grant number K23 NS111086 (Z.A.V.).

AUTHOR CONTRIBUTIONS

Z.A.V. collected the data. Z.A.V. and F.L. conceived the study and model design. J.F. implemented the model and conducted the data analysis. J.F., J.L., Z.A.V., and F.L. analyzed the results and prepared and revised the manuscript.

COMPETING INTERESTS

The authors declare no competing interests.

ADDITIONAL INFORMATION

Supplementary information The online version contains supplementary material available at https://doi.org/10.1038/s41746-021-00479-4.

Correspondence and requests for materials should be addressed to Z.A.V. or F.L.

Reprints and permission information is available at http://www.nature.com/reprints

Publisher's note Springer Nature remains neutral with regard to jurisdictional claims in published maps and institutional affiliations.

Open Access This article is licensed under a Creative Commons Attribution 4.0 International License, which permits use, sharing, adaptation, distribution and reproduction in any medium or format, as long as you give appropriate credit to the original author(s) and the source, provide a link to the Creative Commons license, and indicate if changes were made. The images or other third party material in this article are included in the article's Creative Commons license, unless indicated otherwise in a credit line to the material. If material is not included in the article's Creative Commons license and your intended use is not permitted by statutory regulation or exceeds the permitted use, you will need to obtain permission directly from the copyright holder. To view a copy of this license, visit http://creativecommons.org/licenses/by/4.0/.

© The Author(s) 2021
