# Supplementary material for: Exploring Computational Techniques in Preprocessing Neonatal Physiological Signals for Detecting Adverse Outcomes: Scoping Review
Source: Interact J Med Res. 2024 Aug 20;13:e46946. doi: 10.2196/46946 (PMC11372324; doi:10.2196/46946)
Supplement: Multimedia Appendix 3 [file ijmr_v13i1e46946_app3.zip › Included Papers - Final/3359/Shirwaikar et al. - 2019 - Optimizing neural networks for medical data sets .pdf]

Artificial Intelligence In Medicine 98 (2019) 59–76

Contents lists available at ScienceDirect

Artificial Intelligence In Medicine

journal homepage: www.elsevier.com/locate/artmed

Optimizing neural networks for medical data sets: A case study on neonatal apnea prediction

Rudresh Deepak Shirwaikar[a], Dinesh Acharya U[a,*], Krishnamoorthi Makkithaya[a], Surulivelrajan M[b], Shikhar Srivastava[a], Leslie Edward S Lewis U[c]

[a] Department of Computer Science and Engineering, Manipal Institute of Technology, Manipal Academy of Higher Education, Manipal, India
[b] Department of Pharmacy Practice, Manipal College of Pharmaceutical Sciences, Manipal Academy of Higher Education, Manipal, India
[c] Department of Paediatrics, Kasturba Medical College, Manipal Academy of Higher Education, Manipal, India

ARTICLE INFO

Keywords:
Deep network architectures
Multi-layer perceptron
Optimizing neural network
Deep belief networks
Deep autoencoders

ABSTRACT

Objective: The neonatal period of a child is considered the most crucial phase of its physical development and future health. As per the World Health Organization, India has the highest number of pre-term births [1], with over 3.5 million babies born prematurely, and up to 40% of them are babies with low birth weights, highly prone to a multitude of diseases such as Jaundice, Sepsis, Apnea, and other Metabolic disorders. Apnea is the primary concern for caretakers of neonates in intensive care units. The real-time medical data is known to be noisy and nonlinear and to address the resultant complexity in classification and prediction of diseases; there is a need for optimizing learning models to maximize predictive performance. Our study attempts to optimize neural network architectures to predict the occurrence of apneic episodes in neonates, after the first week of admission to Neonatal Intensive Care Unit (NICU). The primary contribution of this study is the formulation and description of a set of generic steps involved in selecting various model-specific, training and hyper-parametric optimization algorithms, as well as model architectures for optimal predictive performance on complex and noisy medical datasets.
Methods: The data used for the study being inherently complex and noisy, Kernel Principal Component Analysis (PCA) is used to reduce dataset dimensionality for the analysis such as interpretations and visualization of the dataset. Hyper-parametric and parametric optimization, in different categories, are considered, including learning rate updater algorithms, regularization methods, activation functions, gradient descent algorithms and depth of the network, based on their performance on the validation set, to obtain a holistically optimized neural network, that best model the given complex medical dataset. Deep Neural Network Architectures such as Deep Multilayer Perceptron's, Stacked Auto-encoders and Deep Belief Networks are employed to model the dataset, and their performance is compared to the optimized neural network obtained from the parametric exploration. Further, the results are compared with Support Vector Machine (SVM), K Nearest Neighbor, Decision Tree (DT) and Random Forest (RF) algorithms.
Results: The results indicate that the optimized eight layer Multilayer Perceptron (MLP) model, with Adam Decay and Stochastic Gradient Descent (AUC 0.82) can outperform the conventional machine learning models, and perform comparably to the Deep Auto-encoder model (AUC 0.83) in predicting the presence of apnea in neonates.
Conclusion: The study shows that an MLP model can undergo significant improvements in predictive performance, by the proposed step-wise optimization. The optimized MLP is proved to be as accurate as deep neural network models such as Deep Belief Networks and Deep Auto-encoders for noisy and nonlinear data sets, and outperform all conventional models like Support Vector Machine (SVM), Decision Tree (DT), K Nearest Neighbor and Random Forest (RF) algorithms. The generic nature of the proposed step-wise optimization provides a framework to optimize neural networks on such complex nonlinear datasets. The investigated models can help neonatologists as a diagnostic tool.

* Corresponding author.
E-mail address: dinesh.acharya@manipal.edu (D. Acharya U).

https://doi.org/10.1016/j.artmed.2019.07.008
Received 16 January 2018; Received in revised form 9 February 2019; Accepted 24 July 2019
0933-3657/ © 2019 Elsevier B.V. All rights reserved.

1. Introduction

Machine Learning over the last decade, has come leaps and bounds, with various advancements in solving numerous problems once considered intuitive. It is practiced in multiple fields such as Face Recognition [2], Disease prediction [3], Speech Recognition [4], Anomaly detection [5], along with a myriad of others. As this field has matured, new opportunities have emerged for various applications in the medical domain. Neural networks have proved to be highly powerful models in this domain, is used to solve multiple classifications and regression problems, especially medical diagnosis. Artificial Neural Network (ANN) a black box method and a versatile learner, can be applied to nearly any learning tasks such as classification and numeric prediction. Neural networks are a form of supervised learners trained by back-propagating the error between the required output and that of the network output, to the layers between the input and the output layers, called the hidden layers. A neural network with multiple layers is called a universal function approximator, as it can learn a diverse set of functions without explicit structure. It also handles complex interactions as well as highly nonlinear relationships between input and output [6].

The last decade has seen exponential growth in the field of Machine Learning and Artificial Intelligence, primarily due to the introduction of Deep Neural Networks [7] that have drastically improved the ability of machines to solve problems which rely on intuitive decisions learned from experience rather than pre-determined rules. The shallow models such as Support vector machines (SVM), Decision Trees (DT) are incapable of capturing underlying concepts and corresponding relationships [8]. The essential requirement of shallow models is that they require well designed feature representation. Therefore, a model with automatic feature learning and classification or regression will be a practical solution. Deep architectures can simulate and express complex problems at different concept levels that are difficult to be expressed in a problem domain. Multilayer neural network architecture is capable of learning the exact underlying features and feature logic and therefore generalizes very well, but weight learning algorithms did not work efficiently on multilayer architectures, paving the way for Deep learning.

Deep Learning is a subfield of machine learning which attempts to learn high-level abstractions in data by utilizing hierarchical architecture [9,10]. The concept used for deep learning is to train the layers in the sequence where each of the non-output layers is trained to be an auto-encoder with weight adjustment algorithm. In essence, it is forced to learn abstractions of the salient features that describe what comes from the previous layers. Lastly, final layers are trained to predict class based on the output from previous layers.

The basic neural networks are unable to handle selectivity invariance dilemma and also suffer from vanishing gradients which are addressed by deep neural network architectures [11]. Deep learning techniques by reinforcing the discriminative power of simple neural networks help in solving selectivity invariance dilemma [12]. Further, they work by automatically extracting essential features from raw data, making them robust concerning variations in input. Higher classification accuracy even with lesser data, better accuracy with many hidden layers by using nonlinearity, optimally weighted and lower dimensional production mechanism are the merits of deep learning over shallow networks [13]. In medical science, real-time data is known to be noisy and nonlinear in nature [14], and therefore there is a need to optimize learning models to maximize predictive performance based on hypermetric optimization.

A neonate is a newborn baby in the age group of 1–30 days [15]. Among the neonatal diseases, Apnea is predominant in premature neonates. Apnea is a breathing disorder for more than 10–15 seconds often associated with bradycardia, cyanosis or both [16]. Apnea of prematurity (AOP) is the major concern for caretakers of neonates in intensive care units. In premature babies, apnea can fail cerebral blood flow, causing ischemia and eventually even leukomalacia. Further, as per the National Institutes of Child Health and Human Development (NICHD), critical conditions that are responsible for the occurrence of AOP are poorly understood and aren't integrated into the care facilities [17]. Early identification of neonatal apnea in infants and providing better treatment for those who are diagnosed with apnea is important in NICUs. Researchers had proposed algorithms for neonatal apnea prediction based on the cardio respiratory and the movement signals with statistical classifier such as the Gaussian Mixture Model (GMM) and the equal prior Quadratic Classifier on a limited set of examples [16,18]. These studies dealt with the prediction of apnea at birth, but did not deal with the predicting persistence of apnea episodes in neonates who are already diagnosed with apnea. Prediction of neonatal apnea at the earliest is a challenging task in clinical practice. Therefore, there is need for optimal machine learning model for predicting the presence of apnea episodes after the first week of admission at Neonatal Intensive Care Unit.

In our study, we formulate a set of generic steps involved in selecting various model-specific, training and hyper-parametric optimization algorithms, as well as model architectures for optimal predictive performance of neural network. The specific problem of binary classification, the presence or absence of neonatal apnea, is used as a case study for this purpose. Deep Neural Network Architectures such as Stacked Auto-encoder and Deep Belief Network are employed to model the dataset and their performance in predicting the presence or absence of neonatal apnea is compared to the optimized Multi Layer Perceptron (MLP) obtained from our parametric exploration. The models are evaluated with K (10)-Fold Cross Validation using (AUROC) Area Under the Receiver Operating Characteristic curve as the performance evaluating criteria. Further, the results are also compared with Support Vector Machine (SVM), K Nearest Neighbors (KNN), Decision Tree (DT) and Random Forest (RF) algorithms.

The contribution of this paper is as follows:

- A novel framework is proposed for step-wise empirical tuning of neural networks, selection of algorithms with their hyper parametric optimization. The proposed steps could be used to reproduce the experiment on other data sets in medical domain where the data set is noisy and nonlinear.
- The comparative evaluation of the optimal MLP model with Deep Belief Networks, Stacked Auto Encoders, Support Vector Machine (SVM), Random Forest (RF) and K Nearest Neighbor (KNN) are also presented in this paper.
- The developed machine learning models are used to predict the presence of apnea after first week of NICU admission on the study population at Kasturba Medical College, Manipal, India.

The remainder of the paper is organized as follows. Section 2 describes the background study dealing with deep learning approaches, neural network architectures and applications used in the medical domain. Section 3 focuses on the methodology adopted to build deep network architectures and other machine learning models. Section 4 presents thorough experimental results and discussion and section 5 concludes the study with suggestions for future work.

2. Background

2.1. Deep learning approaches

A neural network is composed of the following elements such as learning process, set of neurons or weights and connectivity functions. This set of elements enables us to build a broad range of neural networks, ranging from a directed acyclic graph such as Multi-Layer Perceptron (MLP) with creative alternatives of deep networks to specialized deep learning methods such as Restricted Boltzmann Machine (RBM), Stacked Denoising Autoencoders, and Convolution Neural Networks (CNN). Learning methods in deep networks use unsupervised

as well as supervised approach. The supervised approach requires class labels to modify weights and also a mechanism to communicate error at lower layers [11]. MLP does not get adequately trained due to diffusion of gradient and slow training. Unsupervised training between layers can decompose the problem into distributed sub problems with a higher level of abstraction to be further decomposed at subsequent layers.

Table A1 in Appendix A describes the different deep learning models with learning approach and architecture [11–13]. Most of the deep learning models use greedy layer-wise training approach with unsupervised learning followed by supervised learning approach.

2.2. Neural network architectures and applications in medical domain

Various machine learning algorithms have been used in the domain of healthcare, and neural networks have found use in multiple medical applications. Shanthi et al. [12] proposed a Thrombo-embolic stroke disease predictor system that used feed-forward MLP architecture with three layers. From the 25 physiological parameters considered as features for the prediction of heart stroke, 20 features were selected using a backward stepwise method based on their correlation importance with the provided class label. The trained artificial neural network model provided an accuracy of 89% overall for heart stroke prediction. In another study, a decision-reference system was proposed by Vanisree et al. [19] for diagnosis of Congenital Heart Diseases. The method used a Multilayer feed forward neural network that was trained on a benchmarked dataset. Various physiological features of a patient were considered in this work, including signs, symptoms and medical test parameters. This system managed to achieve an accuracy of 90% in providing an accurate measure of the patient's condition. In another work by Dangare et al. [20], a Heart disease prediction system was developed based on neural networks. The model predicts the probability of a patient developing a heart disease using 13 continuous medical parameters like blood pressure and cholesterol, discrete features such as obesity and smoking habits, as features. The study manages to diagnose heart disease with 99.25% accuracy by training a comparatively simple neural network with one hidden layer and 570 training observations. This study is a good example of how efficiently neural networks even in their nascent form, assisted in the diagnosis of medical diseases. Daphne et al. [21] proposed maximum likelihood and single layer Gradient Descent Multi-Layer for neonatal risk prediction which proved to be inconsistent. Further, Kolmogorov's superposition theorem is proposed to justify the number of neurons in the network. Choudhury et al. [22] proposed a single hidden MLP for neonatal disease diagnosis with a Genetic method for feature selection. The accuracy was found to be 75% which could be increased with different hyper parametric variations and the use of deep networks. More recently, a Natural Language processing technique was proposed to automate medical transcription and translation i.e., Automated Speech Recognition (ASR) system developed by Wołk et al. [23]. In this work, neural network is used to maximize translation performance. The majority of the studies have used simple ANNs with single hidden layer architecture, back propagation algorithm using gradient descent for optimization, without optimized networks to prevent over-fitting of their models on possibly skewed medical datasets.

Deep learning network architectures can perform better than the shallow architectures on nonlinear datasets [24,25]. For complex, noisy and nonlinear data sets there is a need to optimize the MLP model, based on hyperparametric and parametric optimization to better "fit" the latent distributions and performs better predictions. Deep Learning architectures can attain higher classification performance on highly complex distributions that simple shallow models cannot "fit," as their VC (Vapnik–Chervonenkis) dimensions and inherent capacity for learning higher-order functions are significantly higher. Besides the models provide good accuracy, even when the data set size is small [12]. Neural networks, since 2006, under the aegis of Deep Learning, have been applied to various other medical domains such as biomedical imaging, medical transcription, medical history analysis amongst numerous others, with great success. Some of the major work in Deep learning are summarized in Table B1 of Appendix A [8,10,13,26–29]. From the literature, it can be inferred that deep neural networks architectures aided at a medical diagnosis where the data is non-linear and noisy.

Our study attempts to specify a generic procedure for model optimization to improve neural network model performance and justifies its efficacy by a comparison of the optimized network model and other existing deep learning architectures and shallow models. It is important to note that the study doesn't attempt to formulate invariable and fixed rules to improve network optimization; instead it attempts to outline a flexible yet effective methodology of approaching the various parametric optimizations that are regularly carried out in an effective training process.

3. Methodology

The study attempts to build the best performing Multi Layer Perceptron (MLP) by altering and tuning hyperparameters to predict neonatal apnea. Furthermore, based on evaluation criteria the best performing MLP was compared with the Deep Belief Networks (DBN), the Deep Auto-Encoders, the Support Vector Machine (SVM), the Random Forest (RF) and the K Nearest Neighbors (KNN). The overall methodology consists of following steps: a) Data exploration and Preprocessing, b) Classification using K Nearest Neighbours, Support Vector Machines and Random Forest c) Classification using Multi Layer Perceptron and d) Training Deep Belief Networks (DBN) and Deep Autoencoder. The various machine learning models and visualizations had been implemented using Python packages such as Tensorflow, Sklearn, Keras, and Matplotlib. To build the deep neural network models, the Java library DeepLearning4j was used.

3.1. Data exploration and preprocessing

A data set containing 367 cases of neonates of over two years were used for the study by obtaining ethical approval from Institutional Ethics Committee of Maniapl University. The data collected comprised 30 predictor variables belonging to: a) demographics such as birth weight, birth cry, gestation age, apgar score, etc; b) maternal covariates included mode of delivery, steroids, and surfactant, etc; and c) physiological parameters such as heart rate, respiration rate, desaturation, etc. The outcome variable was defined as the total number of occurrences of apnea episodes, at the end of the first week of NICU admission. The occurrence of apnea episode was defined as a pause of respiration for more than 10 s followed by a drop in heart rate (< 100) and oxygen saturation ($\leq$ 80% for $\geq$ 4 s) in neonates born less than 37 weeks of gestation. The outcome variable signifying the number of occurrences of apnea episodes were chosen to be the final class label after encoding. The variable was assigned binary codes, where 0 was assigned to those observations where the number of apnea episodes are 0, and 1 was assigned to those observations where the number of apneic episodes were at least one. This resulted in a binary classification problem, between the presence or absence of apnea occurrence in neonates. Thus, at the end of the first week, if the apneic episodes were present, it was coded as "1″ otherwise as "0″.

The features which were entirely textual, descriptive or deemed medically irrelevant by the expert were removed. For example, features such as length of NICU stay, mode of ventilation support, duration of ventilation, development of Broncho Pulmonary Dysplasia (BPD), Patent Ductus Arteriosus (PDA), Necrotizing Entero Colitis (NEC), Retinopathy of Prematurity (ROP), etc., followed by features which were biased and statistically irrelevant were removed. Of these 30 features, 20 were deemed as medically and statistically relevant after careful critiquing by the experts. These variables were analyzed using scatter plots for numeric variables, and with bar charts for categorical

variables. The relationship between each of the predictor variables and outcome variable was assessed based on how these variables were correlated. This analysis confirmed that the chosen features were good predictors of the presence of the apnea. The final dataset consisted 20 predictor variables or features and 1 binary class label.

The combination of summary statistics mean, median, variance, and visualization techniques such as t-SNE and Principal Component Analysis (PCA) were employed to explore the data. On exploration, the data was found to be noisy, and several observations with missing values were discovered. Techniques to approximate these missing features, such as nearest neighbor model approximations, functions of mean and mode, proved to be unreliable and inconsistent due to the inherent noise in the data. As a result, some of the observations of missing features were discarded from the dataset. This was done to ensure that any additional noise in the dataset due to approximated feature values was not introduced. Also, other features (continuous values) with missing values which were medically relevant were converted to discrete with addition of group (not known). The not know category in medically field are technical errors or human error. Therefore, this initial preprocessing of the dataset resulted in a reduction of 14% of the dataset, from 367 to 315 observations. The source dataset after initial pre-processing had 146 observations of apnea being positively diagnosed (class label as 1) and the remaining 169 were observations of absence of apnea episodes (class label 0). The classes were partially skewed by a 47:53 ratio where 53% of the observations had an outcome 0 and 47% of the observations had an outcome.

The frequency count of input features which are discrete or categorical with the outcome variable apnea (blue color indicating presence of apnea episodes and red indicating absence) are represented in Fig. 1.

The features such as birth weight (BWT), head circumference (HC_birth), heart rate (HR day 1, 48 h, 78 h) and gestation age, which were continuous (numeric) in nature were plotted with distribution in Fig. 2. The detail description of all the features is presented in Appendix C (Table C1)

Forest of trees, a feature selection method [30], was used to select statistically relevant features from the set of 20 features. Random Forest (RF) model was considered for feature selection due to its ability to generalize well in distributions with partial class imbalance. It was proven to be a highly accurate feature selection method on noisy datasets, as it generated unbiased estimate of the generalization error [31]. It created randomness to the given dataset by creating shadow features. Furthermore, to analyze the importance of the various features in the dataset, a RF model was trained on the whole dataset. The variable importance was measured with accuracy estimate using Area Under Curve Receiver Operating Characteristics (AUCROC). The resultant trained model was used to provide a measure of the importance of the various features by building a classification model. Table 1 represented the 20 features with their decreasing importance. The feature importance of all the 20 different features had a cumulative sum of 100%, as expected. The cumulative sum of the importance of the features selected provided a measure of the confidence of the model in its classification process. With the aim to remove noise and select the most correlated features, only the features with importance greater than 1.5% were retained for the final dataset. The 1.5% threshold is chosen approximately based on the degree of Gini impurity and information gain introduced by each feature. Therefore, four features, namely birth cry, surfactant, Apparent Life Threatening Event (ALTE) and bradycardia were removed from the original dataset, bringing the cumulative sum of the feature-importance of the remaining selected features to 95.8%.

To visualize and understand the complexity and nature of the data the Principal Component Analysis (PCA) algorithm was used. PCA is a great tool to analyze multi-dimensional distributions in lower dimensions. It discovered the eigen vectors of distribution along the axis of maximal variances and used them to identify multiple uncorrelated independent features called the principal components with the highest corresponding eigen values [32]. These principal components enabled PCA to provide a more useful visualization of high-dimensional data, into lower dimensional feature spaces. However, the PCA was ineffective in reducing the non-linear distributions, as it was unable to linearly separate the data points reduced from a complex multivariate K-dimensional feature space to dimensions lower than K [33]. Kernel principal component analysis [34], an extension of linear PCA algorithm, is capable of inferring a representation of non-linear manifolds using kernels and addresses the sparsity problems that medical datasets are ailed by [35]. From Fig. 3 (a and b) it was be observed that the initially noisy uncorrelated linear PCA representation of the data now found a linear correlation, with reduced noise in the Kernel PCA representation of the data as shown in Fig. 3 (b). It was thus inferred that the data was correlated albeit non-linearly, and hence the dataset was observed as noisy, as was with most medical datasets. Therfore, it may be classifiable reliably on hyper-plane using a high-dimensional non-linear classifier.

Deep neural network architectures were especially successful on modelling such complex non-linear distributions, due to their ability to develop higher abstracted representations of the data by warping the plane of high-dimensional space to facilitate better classification [25]. Deep neural networks were known to outperform conventional models such as Support Vector Machines (SVN), Random Forests (RF), Decision Trees and K Nearest Neighbour (KNN), on such complex noisy distributions [24].

3.2. Classification using K nearest neighbors, support vector machines and random forest

KNN used similarity measures to classify new cases based on training data. It used distance functions such as Euclidean or Manhattan to compute the similarity [36]. The K stand for neighbors and number of K determines how well the model would predict the future values. Overfitting and underfitting were the measure problems associated with KNN and to overcome the bias variance trade off, the data sets needed to be validated with n number of K values. The methodology adopted for predicting apnea using KNN used normalized data set for building training model. The square root of number of training data sets (315 cases of neonates) = 17.74 i.e. 17 was used as the K value to validate the model. Alternate K values (1, 5, and 11) were also used to validate and compare the accuracy of the models.

SVM was found to be very powerful at separating highly complex and non-linear data [37]. It used kernel functions such as polynomial, gaussian radial bias and sigmoid for non-linear classification. The advantage of using a kernel function was to avoid over fitting, if the data was noisy [38]. The performance of the SVM depends on the choice of the kernel function sigma (ξ) and the margin factor (C) as shown in Eqs. (1) and (2):

$$\min \frac{1}{2} \, || \hat{w} ||^2 + C \sum_{i=1}^{n} \xi_i \tag{1}$$

$$y_i(\hat{w}.\hat{x}_i + b) \geq 1 - \xi_i \,, \forall \; \hat{x}_i, \xi_i \geq 0 \tag{2}$$

Where, a cost value is denoted as (C), $\hat{w}$ is a normal vector decision hyper plane, $\hat{x}_i$ denotes data points and b is a constant. To find the optimal solution, the error function in Eq. (1) had to be minimized.

SVM as a classifier was used to build a classification model with a hyper plane separating data for two classes. The hyper plane building procedure was a challenge while building the SVM model. The proposed steps used to build optimal SVM model are presented in Fig. 4. The SVM model was trained with linear and non-linear kernel (radial bias) for the data set with 16 input features and one output. The model was tuned for maximum performance based on kernel function sigma (ξ) and the margin factor (C). The comparative evaluation of all SVM models with various accuracy parameters are described in the result section.

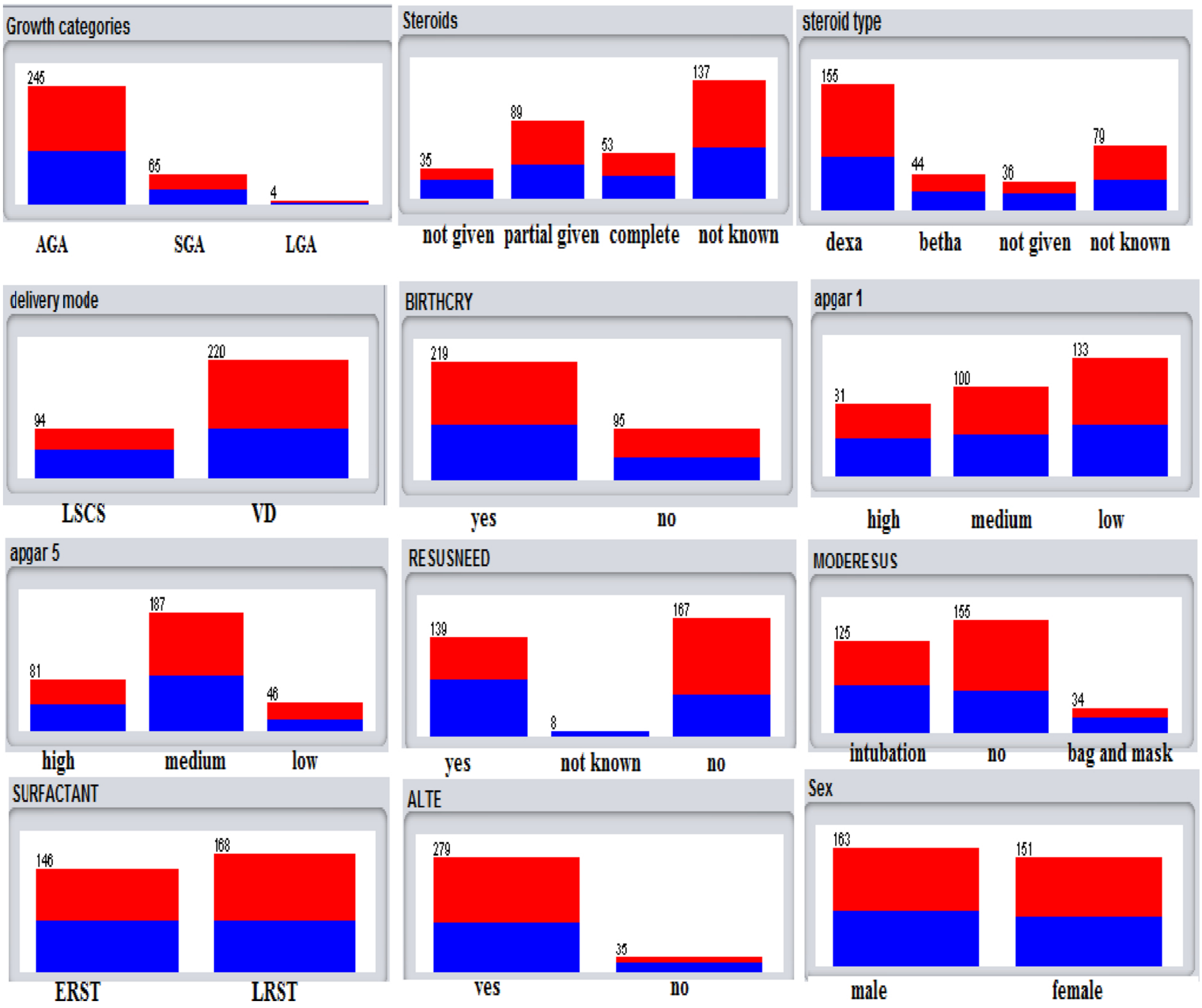

Fig. 1. Categorization of nominal data indicating presence or absence of apnea.

Random Forest or Decision Tree Forest is a classifier which can handle noisy or missing data, categorical or continuous variables [36]. It combines the base principles of Bagging with random variables section to add distinction to the Decision Tree. The cross validated RF was optimized with the tuning grid, which defined how many features were randomly selected at each split (i.e., the square root of the total number of input features). The grid of trials was formed with square root of features, half of square root of features, double square root of features, and full sets of input features (i.e., the default) with an ensemble of 500 trees. The advantage of using a large number of trees is that every feature can appear in multiple models. The grid of 2, 4, 8, and 16 was trained with the model function, and the Kappa metric was used to select the most accurate model.

3.3. Classification using Multi layer perceptron

Multi Layer Perceptron (MLP) is a feed-forward neural network, with a linear or non-linear activated neuron as its core unit, having one or more hidden layers. The network consists of an input layer of source neurons, at least one or more hidden layer of computational neurons, and an output layer. The input signal is propagated in a forward direction on a layer by layer basis. The network is then trained using backpropagation algorithm, altering the weights according to the gradient of the error propagated, with the aim to achieve the optimal parametric settings. In our study, the MLP network was tuned to be optimized, i.e., the performance of the network was empirically maximized by selecting, fine-tuning tuning various parameters and hyperparameters of the network, as shown in Fig. 5. Initial network training showed that the 4 hidden layer model performed better than the simple MLP with 1, 2 or 3 layers. Therefore, an MLP with 4 hidden layers, with Stochastic Gradient Descent as the gradient descent algorithm, was considered as the initial model at the start of the process. The various learning algorithms, parameters, and hyperparameters of the resultant network were altered gradually by selecting the highest performing model at each step. For all further model training, the dataset was normalized to a standard normal distribution, with zero mean and unit variance with the aim to ensure that no feature was weighted arbitrarily more than the other. The initial weights in all networks were sampled from a gaussian distribution with zero mean and variance of 1/N, with N being the average of the number of inputs and outputs in the network [39]. All the models had been trained for 5000 epochs, with an initial learning rate of 0.01, the momentum of 0.9 and iterations of 5 for each data points. The models were cross-validated at every 25 epochs, and the subsequent evaluations were logged and plotted as graphs for comparison. The training parameters were fixed for all further steps involving the training of an MLP model, from activation function to the updater algorithm comparisons. The validation scores were logged for every 25 epochs during every training process. The negative log likelihood errors, along with the Area under the Receiver Operating Characteristic Curve (AUCROC) scores for the validation sets, were compared at every instance, and corresponding inferences were made. The various learning algorithms, parameters, and hyper parameters of

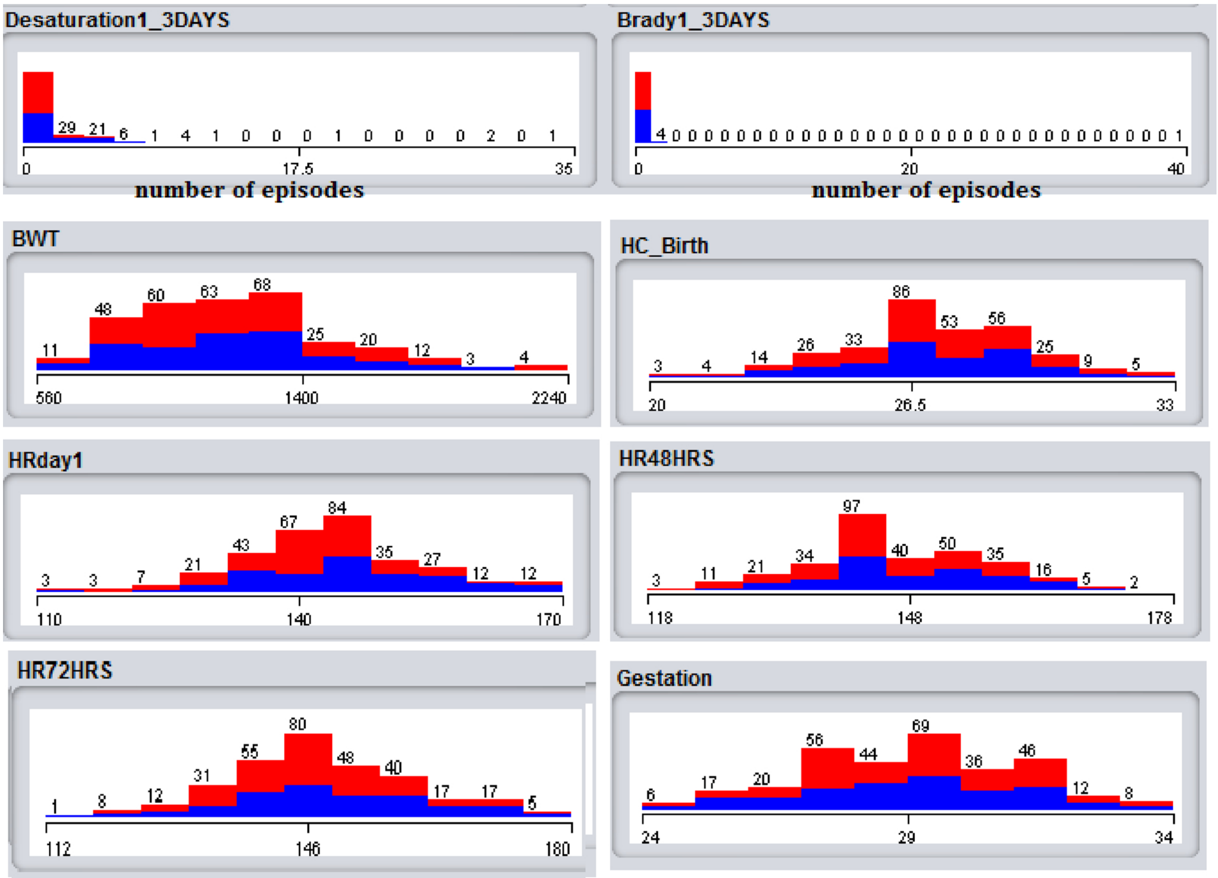

Fig. 2. Distribution of numeric data indicating presence or absence of apnea.

Table 1
Selected features in decreasing order of importance.

Number	Features	Feature importance in %(Mean decrease accuracy)
1	Birth weight	14.1
2	Heart rate Day 3	12.0
3	Heart rate Day 1	11.3
4	Heart rate Day 2	10.0
5	Desaturation	7.1
6	Gestation age	7.1
7	Head circumference Birth	6.4
8	Dexa / Betha	4.8
9	Mode of Resuscitation	4.8
10	Resuscitation	3.5
11	Steroids	3.5
12	Delivery mode	3.8
13	Apgar 1 minute	2.5
14	Apgar 5 minute	2.3
15	Sex	2.2
16	AGA / SGA	1.9
17	Surfactant	1.4
18	Birth cry	1.2
19	Bradycardia	0.7
20	ALTE	0.7

the resultant network were altered gradually by selecting the highest performing model at each step.

3.3.1. *Activation functions*

Activation functions are responsible for deciding the operation performed by each neuron, i.e., the output of every neuron in the neural network for a group of inputs thereby dramatically influencing the behavior of the network. Activation functions directly affect the capacity and the hypothesis space accessible by the neural network model. Non linear activation functions like Tanh, Sigmoid allow propagation of non-linear representations of the inputs through the network over a wider range of values. At the same time, the non-linear activation functions also contribute to the inherent variance of the model with an increase in capacity and algorithmic complexity. Linear activations functions are comparatively simpler; maintain an inherently higher bias and lower variance, while allowing easier training, and therefore facilitating training at a lower computational cost [40]. The popular activation functions Rectified Linear Unit (ReLU), Exponential Linear Unit (ELU), Tanh, Sigmoid, Hard Tanh, Leaky ReLU were considered in this work for comparison. The further detail description of the activation functions are mentioned in Appendix B.

For training, the initial design of the MLP model with four hidden layers, and hyperparameters initialized as per Table 2, were used. The above activation functions were individually applied to train the model, with all the other hyper-parameters initialization kept constant. The activation functions corresponding to the highest performing model were retained for all future steps in the optimization process.

3.3.2. *Gradient descent algorithms*

The MLP network was trained on three popular gradient descent algorithms [41] namely: Stochastic Gradient Descent (SGD), Line Gradient Descent (LGD), and Conjugate Gradient Descent (CGD) algorithms, the details of which are mentioned in Appendix B [42,43]. The 4 hidden layer MLP model with the activation function led with the highest performance, obtained from the previous step, was selected and initialized with the default hyper parametric values. The above gradient descent algorithms were individually used to train an instance of the selected model. Finally, the algorithm lending the maximal performance was retained, as a part of the updated model with the obtained

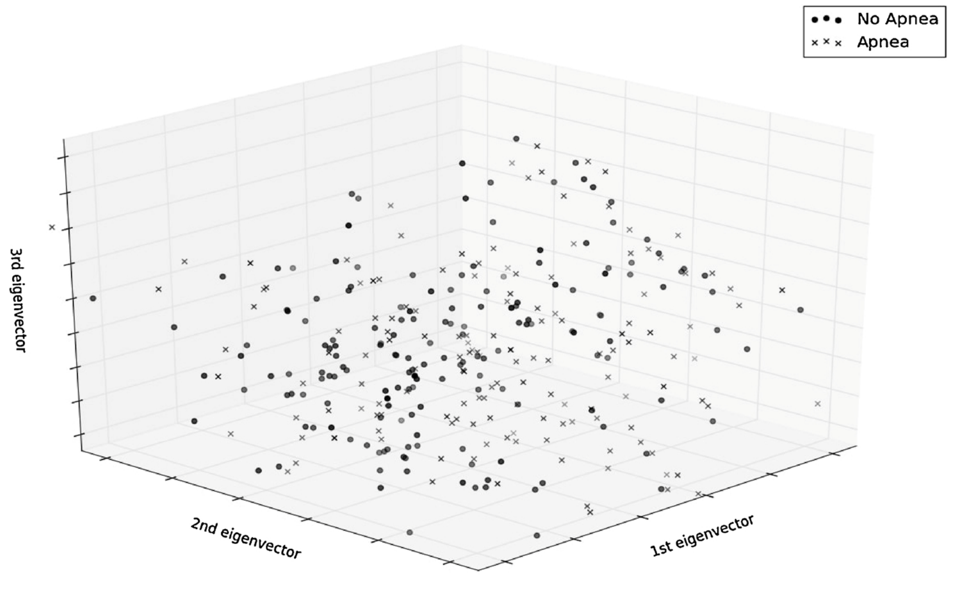

(a) Data representation with linear PCA

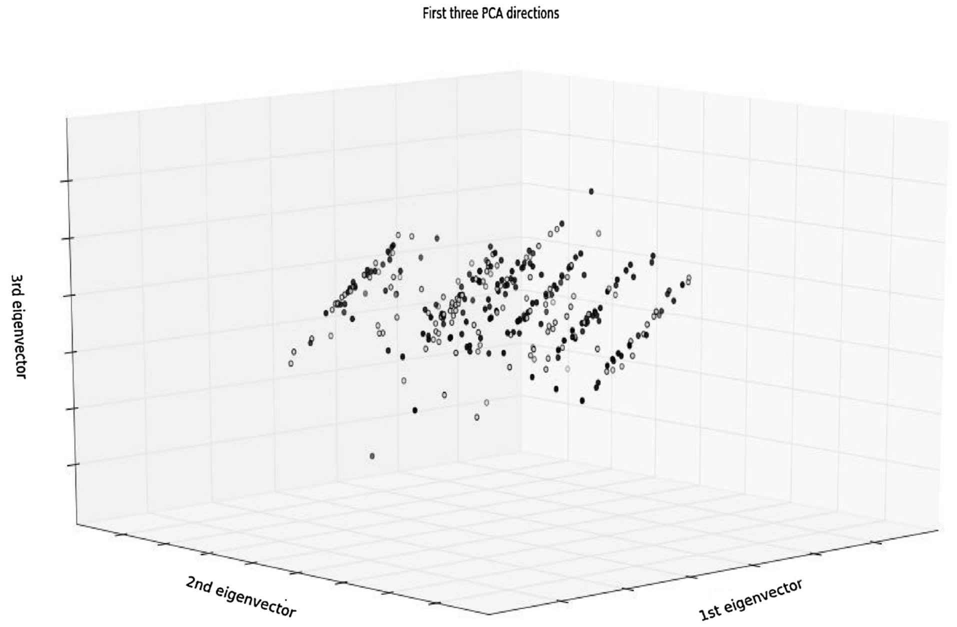

(b) Data representation with kernel PCA

Fig. 3. (a) Data representation with linear PCA. (b) Data representation with kernel PCA.

highest AUCROC score.

3.3.3. *Depth of the neural network*

The depth of a neural network is defined in terms of the number of hidden layers in the architecture and as it increases, so did its complexity and ability to learn complex data and abstractions. The number of layers in a network critically determines the degree of bias and variance inherent in the model. With deeper networks, it is expected that the complexity and variance of the model will increase significantly, along with its tendency to overfit [44]. Inferring from the MLP model, obtained after retaining the optimal activation function and gradient descent algorithm from the previous steps, the MLP models varied from 1 to 8 hidden layers as shown in Table 3, were created.

The selected optimization algorithm and activation function were used to train the model with the initial hyperparametric settings kept constant. Furthermore, L1 regularization was introduced into the network to take the emphasis off the number of neurons in the network as L1 severely down weights inactive neurons. This allowed the variations in the layers, and the subsequent learning of higher abstractions to

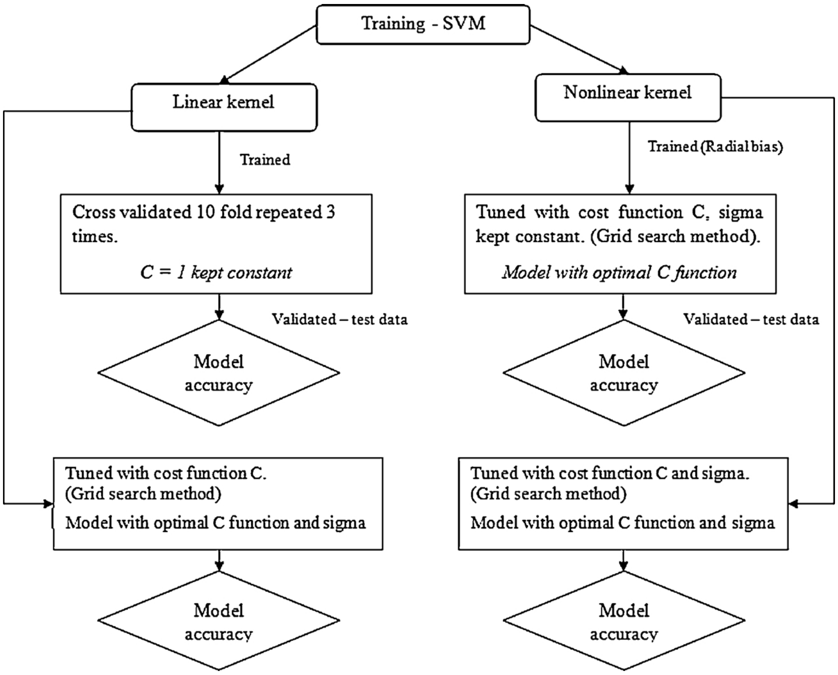

Fig. 4. Proposed methodology to build SVM model.

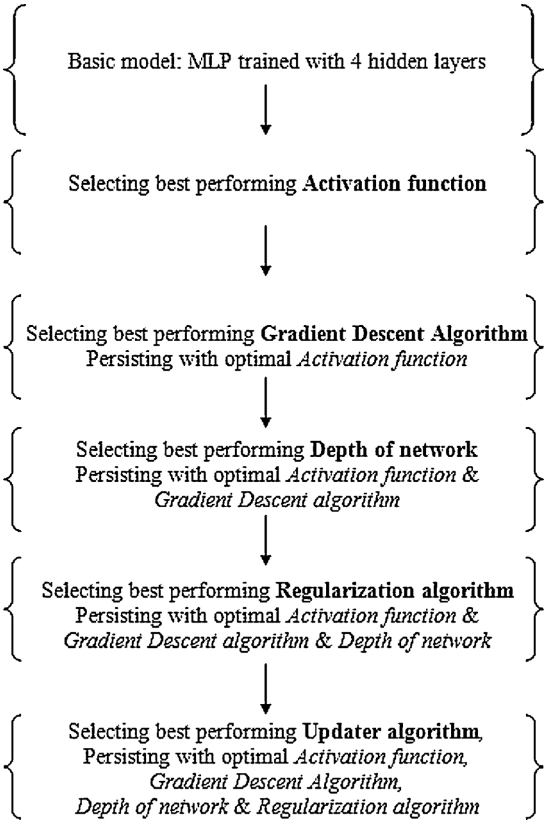

Fig. 5. Optimization of Multi-layer perceptron (MLP).

Table 2
Defined hyper parameters for Multi Layer Perceptron.

Learning Rate	Number of Epochs	Iterations (For each data point)	Momentum (Nesterov)
0.01	5000	5	0.9

influence the experiment, rather than the exact number of neurons used [45]. The regularization function would eliminate the increase in variance. The number of layers that corresponded to the highest performing model was noted, and the particular model was selected as the updated MLP model, for further optimization.

3.3.4. Regularization

Regularization algorithms were used to optimize the bias variance trade off in machine learning models and to facilitate a better ‘fit' of the models on a given dataset. Regularization is widely used in practice to limit and manage the inherent bias in neural networks with the aim to prevent over fitting during the training of the model. It allows the network to emphasize a more uniform distribution of weights across the architecture as the training proceeds [45]. The literature [46] mentioned several regularization techniques such as L1 and L2 regularization as well as combinations of the two. L1 and L2 norm regularization were derived from the Lasso and Ridge regression methods used to optimize the bias variance trade off. L2 regularization can be implemented by augmenting the error function with the squared magnitude of all the weights in the neural network. In the case of L1, λ a regularization-strength hyper parameter value was added to reduce overfitting.

To facilitate the comparison between the performance benefit provided by regularization techniques, the updated MLP model obtained from the comparison of the MLP models with varying layers was selected, modified with the corresponding regularization settings and trained with the retained initial hyper-parameters. The model was trained using a varying L1 and L2 regularization methods and combining of the two and with drop off, with each as a separate model. The drop out also helped in spreading out the weights at all layers as the system will be reluctant to put more weight on some specific node. So it helps in shrinking weights and had an adaptive effect on weights.

3.3.5. Learning rate optimization

The Stochastic Gradient Optimization algorithm has several variations to its learning rate decay scheduling method, which were used to improve its performance and efficiency significantly [47]. The learning rate in a neural network model is crucial to its performance as it directly influences the rate of weight change in the model and the rate at which

Table 3
Parameters defining structure of neural network.

No. Hidden Layers	Number of Layers (L)									
	L1	L2	L3	L4	L5	L6	L7	L8	L9	L10
1	Input (16)	16*4	Output (1)							
2	Input (16)	16*4	16*2	Output (1)						
3	Input (16)	16*4	16*2	16	Output (1)					
4	Input (16)	16*4	16*2	16	16/2	Output (1)				
5	Input (16)	16*4	16*2	16	16/2	16/4	Output (1)			
6	Input (16)	16*8	16*4	16*2	16	16/2	16/4	Output (1)		
7	Input (16)	16*16	16*8	16*4	16*2	16	16/2	16/4	Output (1)	
8	Input (16)	16*32	16*16	16*8	16*4	16*2	16	16/2	16/4	Output (1)

Note: [Input(16), input neurons; Output (1), output neuron].

it learns the features in the high dimensional functional space. By the use of learning rate updater algorithms, the rate at which the network learns can be dynamically changed to facilitate optimized learning of the network and increase network performance on noisy and non-linear data. The several updater algorithms used to allow dynamic change of the learning rate in a neural network model were as follows: Momentum, Nesterov momentum, Adam, RMS prop, AdaDelta and SGD. The details of the algorithms are mentioned in Appendix B.

The updated model obtained from the comparison of various regularization techniques in the previous step was trained using the gradient descent algorithm with each specific updater algorithm individually, while all the other remaining hyperparameters were initialized as shown in Table 2.

3.4. Training deep belief networks and autoencoders

This section deals with the implementations of Deep Belief Networks (DBN), an Restricted Boltzmamn Machine (RBM) based methods and Stacked Autoencoders. The DBN, a RBM based method and Stacked autoencoders had been trained to predict neonatal apnea.

Deep Belief Network (DBN) is a probabilistic generative model with a joint probability distribution and an efficient layer by layer greedy learning strategy. It is a fully connected architecture with partial bi-directional connections with unsupervised training which removed the necessity of labelled data for training [48].

In this study, the DBN, initialized with architectural parameters as tabulated in Table 4, has 6 hidden layers with three encoding and three decoding layers. It used a linear gradient descent algorithm for optimization and trained for 5000 epochs with 5 iterations for each training example. The model was initially trained for 1000 epochs, before using a supervised training with back propagation algorithm. Furthermore, the model was cross-validated at every 25 epochs, and the resulting evaluations were logged for observations and analysis.

Stacked Autoencoders were generative multiple hidden layers, fully connected networks with bidirectional connections which used an unsupervised learning for pre training, applying back propagation by setting the target values equal to inputs [49,50].

The architecture used in this study had 6-hidden layers, with three encoding and three decoding layers, as shown in Table 4. The model was initially layer wise per-tained for 1000 epochs, after which it was fine-tuned by supervised and trained by back propagation for 1000 epochs. The model was trained with an initial learning rate of 0.01, Nesterov's Momentum of 0.5, and SGD algorithm, AdaGrad optimization, and ReLU activation function for all neurons in the hidden layer, with 5 iterations for every training batch/example.

The initial hyper parametric settings used in these deep models, were obtained as a result of the classical method of grid parameter search as well as trial and error heuristics. The proposed steps of optimization were not followed in training these deep models, to allow these deep models to serve as a viable comparison between the two training methodologies and their respective efficacy.

4. Results and discussion

The results obtained from experimental evaluation of machine learning techniques were discussed in this section. Also, the section described the results obtained by the proposed step wise approach used to tune hyper parameters of MLP. Furthermore, the comparative result analysis of the optimal MLP model with deep belief networks, stacked auto encoders, SVM, RF and KNN were also presented in our study.

4.1. Evaluation of K nearest neighbors, support vector machines and Radom Forest

The KNN model was built with k = 17 neighbors, and the results obtained on the test data is presented in Table 5. The true negative results of 40 of 84 values indicated cases with absence of apnea episodes, as correctly identified by KNN. True positive rate of 9 values indicated the correct classification of presence of apnea. The 28 examples indicated false negatives in this case, the predicted values were absence of apnea but the neonates were actually suffering from apnea. The model had 9 cases where the model incorrectly classified as the class of interest. Compared to all the K values (17, 1, 5 and 11), the model build with 17 nearest neighbor was found to be accurate with accuracy of 0.58. All the models are able to predict absence of apnea better than presence of apnea indicated by higher specificity and lower sensitivity.

SVM models were developed with proposed methodology as described in Fig. 4. SVM with linear kernel tuned with cost function (C = 0.01) was found to be more accurate compared to other models (Table 6).

Random Forest (RF) model tuned with (mtry), number of variables randomly sampled at each split was build. Initially mtry = square root of number of variables was considered and compared with other values

Table 4
Deep network model configuration.

Deep architectures	Layer 1	Layer 2 Neurons	Layer 3 Neurons	Layer 4 Neurons	Layer 5 Neurons	Layer 6 Neurons	Layer 7 Neurons	Layer 8 Neurons
Stacked Autoencoder	16 Input	16/2 Encoding	16/4Encoding	16/8 Encoding	16/4 Decoding	16/2 Decoding	16 Decoding	16/8 Softmax Classifier Output
Deep Belief Network	16 Input	16/4 Encoding	16/8 Encoding	16/4 Encoding	16/2 Decoding	16 Decoding	32 Decoding	16/8Softmax Output

Table 5
Comparative evaluation results of K Nearest Neighbor.

Model description	Sensitivity	Specificity	F Score	Accuracy	AUC
K = 17	0.24	0.85	0.33	0.58	0.55
K = 1	0.35	0.55	0.36	0.46	0.40
K=5	0.16	0.78	0.22	0.51	0.50
K = 11	0.13	0.89	0.21	0.55	0.51

Table 6
Comparative evaluation results of Support Vector Machine.

Model description	Sensitivity	Specificity	F Score	Accuracy	AUC
SVM Linear	0.51	0.72	0.55	0.62	0.61
SVM Linear optimized	0.58	0.78	0.63	0.68	0.68
SVM non-linear	0.44	0.76	0.51	0.61	0.60
SVM non-linear optimized	0.41	0.78	0.50	0.61	0.59

(2, 4, 8, 16). Also, the results obtained were compared with Decision Tree (DT) model on the study population (Table 7). The model with the highest accuracy was RF (mtry = 8), with 0.59 AUCROC score. The results also proved the importance of Ensembles and Random Forest over Decision Tree. Decision tree (DT) classifier is found to be with lowest accuracy of 0.51.

4.2. Hyperparametric optimization of multi layer perceptron

The Multi Layer Perceptron (MLP) with different hyper parametric and parametric settings were evaluated based on evaluation criteria such as Area under the Receiver Operating Characteristic curve (AUCROC) and F1 score. The final optimal model was built with the best performing activation function, gradient descent, depth of network, regularization and learning rate for optimal predictive performance on the dataset.

4.2.1. Activation functions to train multi layer perceptron

Graph comparing AUC Scores for models trained with different activation functions at various iterations is shown in Fig. 6. It can be seen that the ReLU and its variant Leaky-ReLU clearly outperformed all the other activation function models with a much higher and stable performance on the validation data set. Furthermore, the rate of performance growth in the models with ReLU and Leaky ReLU was strikingly higher in the initial iterations of the training procedure. This higher performance of the ReLU activation functions could be attributed to the reduced computational complexity of linear activation. Also, Hard Tanh, a linear approximation of the non-linear Tanh function, actually resulted in a model with higher performance, and served as another instance of the benefits of linear activation functions in training deep networks.

4.2.2. Multi layer perceptron trained with gradient descent algorithms

From Fig. 7 depicting cross entropy vs. iteration graph of the models, it was observed that the Line Gradient Descent (LGD) algorithm converged to lower Cross Entropy values faster than both the Conjugate Gradient Descent (CGD) and Stochastic Gradient Descent (SGD)

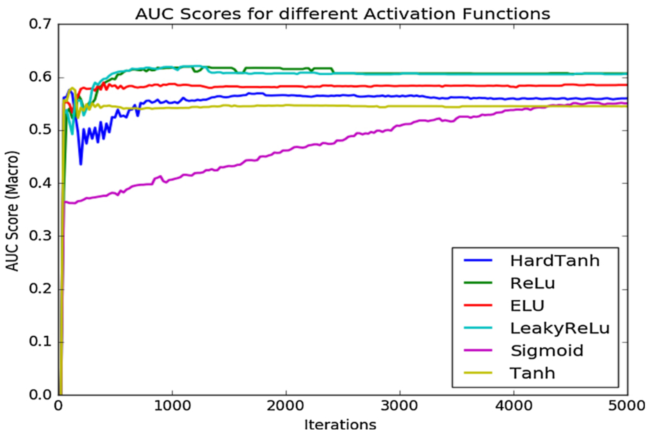

Fig. 6. AUC scores for different activation functions.

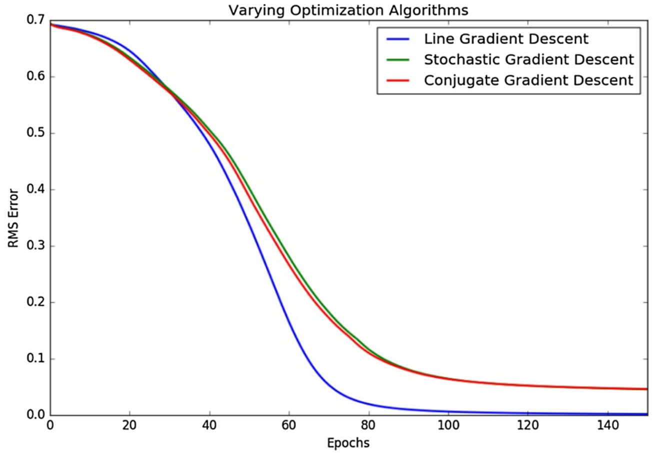

Fig. 7. Negative Log Likelihood Error for different gradient descent algorithms.

algorithms. The results could be attributed to the iterative averaging of the gradient in batches from both the SGD and CGD algorithms that causes the error rate to reduce gradually as compared to the LGD algorithm. The LGD algorithm alters the weights of the network based on its efficient Bayesian optimization algorithm, automating both the selection of a good learning rate and its decay scheduling. Furthermore, Fig. 8 compared the graphs with AUC scores on the validation sets for the three algorithms. It could be seen that the CGD algorithm consistently provided the highest performance. The LGD algorithm settled to non-optimal local minima, after the 700th iteration as could be witnessed by the corresponding flat gradient, while the other two iterative batch algorithms vary drastically as the training proceeds with the performance of the SGD algorithm dropping eventually. It could be inferred that the CGD proved the most effective for the existing complex noisy dataset. This might be attributed to the CGD algorithm's ability to optimize the gradient by finding the conjugate directions, ensuring a more direct convergence to the local minima. Conjugate Gradient Descent, was guaranteed to approach a local minimum, in at most the number of mutually conjugate directions in the basis [41].

Table 7
Comparative evaluation results of Randon Forest ensemble with Decision Tree.

Model description	Sensitivity	Specificity	Precision	AUC ROC	F Score	Accuracy
RF (mtry = 2)	0.52	0.63	0.59	0.58	0.55	0.57
RF (mtry = 4)	0.50	0.65	0.60	0.58	0.54	0.57
RF (mtry = 8)	*0.52*	*0.65*	*0.60*	*0.59*	*0.56*	*0.58*
RF (mtry = 16)	0.50	0.63	0.58	0.57	0.53	0.56
DT	0.47	0.55	0.52	0.51	0.50	0.51

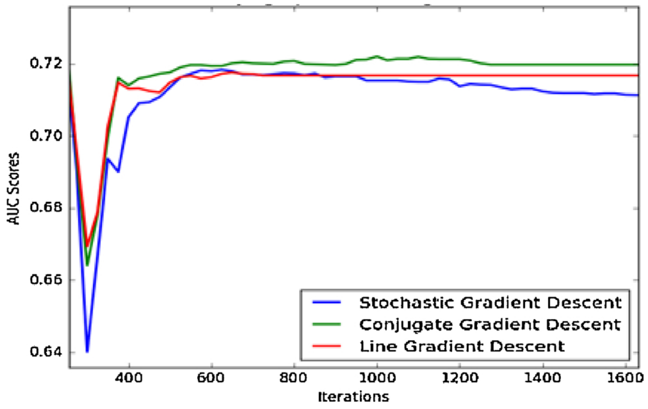

Fig. 8. AUC Scores for different gradient descent algorithms.

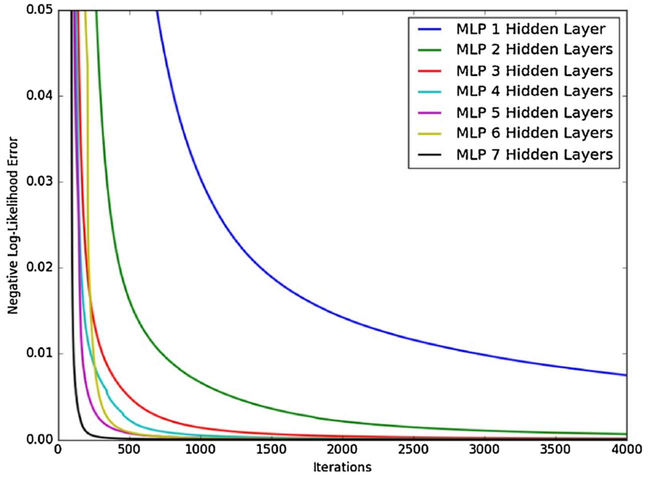

Fig. 9. Negative Log – Likelihood Error for varying hidden layers.

4.2.3. *Depth of the neural network*

From the Root mean Square RMS Error vs. Iterations graph shown in Fig. 9, for the models with varying network depth. It was observed that deeper networks i.e. with more hidden layers converge to lower error rates exponentially faster than their shallower counterparts. Correspondingly from the validation set, AUC Score vs. Iteration graph for the models, shown in Fig. 10, it could be inferred that the model with the greatest number of hidden layers, provided the highest performance model, which clearly indicated the ability to "fit" on complex datasets. The model capacity, increased with the depth of the model. Although the models with higher hidden layers followed the higher performance, their tendency to overfit on the dataset also increased as expected. For the models with hidden layers from 2 to 4, in the AUC score vs. Iteration graph, it could be seen that performance, in fact, droped as the training proceeds that could be attributed to a possible local-minima or a resultant over-fit as the models gradually settled to a zero-gradient slope. It was also noted that the seven layer MLP overfits due to high variance and did not generalize very well with a drop in performance. The MLP model with 6 hidden layers had the highest AUC score amongst all the models under comparison.

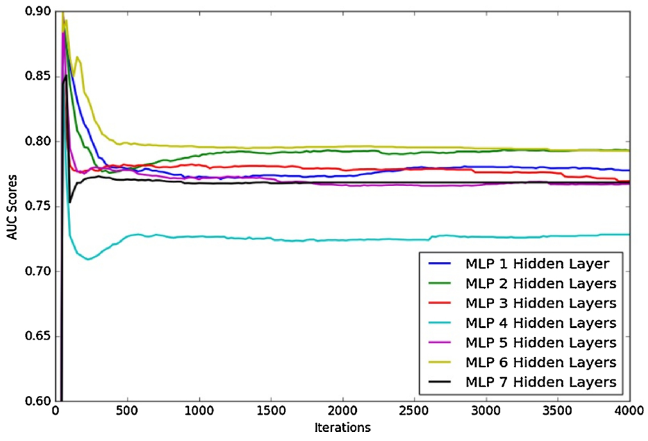

Fig. 10. AUC scores for varying hidden layers.

4.2.4. *Regularization for a neural network*

From the obtained results in the comparison of models with varying regularization settings given in Fig. 11, it was observed that regularization, as both L1 and L2, individually or as a combination, did not provide any significant advantage over the model with no regularization. The models with L1 regularization consistently resulted in lower performance. It could be inferred that penalizing large weights in the network, did not always result in better represented feature space, and for complex datasets. Also, the L2 regularization did not impact the model performance, with little impact on the scarcity of model weights.

4.2.5. *Learning rate optimization*

From the training RMS Error vs. Iteration graph based on different updater algorithms for gradient descent algorithm, shown in Fig. 12, it could be clearly observed that updating the learning rate through either a second-order or first-order algorithm made a drastic difference in the model's ability to converge to a local-minima during training. Furthermore, from the Fig. 13, it could be observed that algorithms that utilized the second order moment like Adagrad and Adam, consistently performed higher than the other learning rate updater algorithms. Although the SGD algorithm (without momentum) during the early iterations gave a higher performance due to the zig-zag nature of descent of the algorithm, the performance was not stable, and as the model slowly converged to the minima, it gradually shifted towards a non-optimal local optimum. On the other hand, the adam, AdaDelta consistently provide higher performances as the training proceeded, and gradually descent to local-minima with a much higher performance than that of the SGD algorithm. It could, therefore, be inferred, that a combination of second and first order moment in updating the learning rate was beneficial in complex feature spaces with multiple local-minima's, as could be expected from a noisy complex medical data, to obtain a more stable consistently higher performing model.

From Fig. 14, it could be seen that the optimized 6 Hidden Layer MLP network, trained using the SGD with Adam Updater, in fact outperformed the 6 Hidden Layer MLP network trained using the CGD.

4.3. *Comparative evaluation of machine learning models*

After the formulated optimization process, the 6-hidden layer (total 8 layers including input and output layer) MLP with Adam decay and SGD proved to be the most effective model with an AUC Score of 0.82 (Table 8). Table 8 shows comparison of the optimized MLP models with their AUC scores for diagnosing neonatal apnea i.e. classifying each neonate as either having apnea or not after the first week of diagnosis.

The best performing SVM model with AUCROC of 0.68, Random Forest (AUC = 0.59) and K Nearest Neighbor with AUC 0.55 (Table 9) were found to be less efficient compared to deep neural network models as shown in Table 10.

The observed performance metrics of the optimal MLP model with Deep Belief Network and Stacked denoising auto encoder with various evaluation parameters had been logged in Table 10. The models performance with AUCROC curve is shown in Fig. 15. The evaluation results seemed to favor deep neural network architectures such as auto-encoders with AUC of 0.83 followed by an optimal MLP obtained from a heuristics optimization process with 0.82.

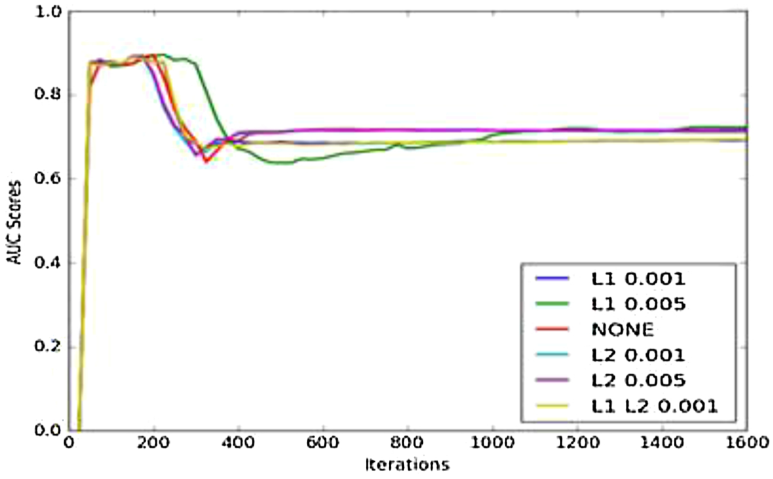

Fig. 11. AUC scores for varying regularization parameters.

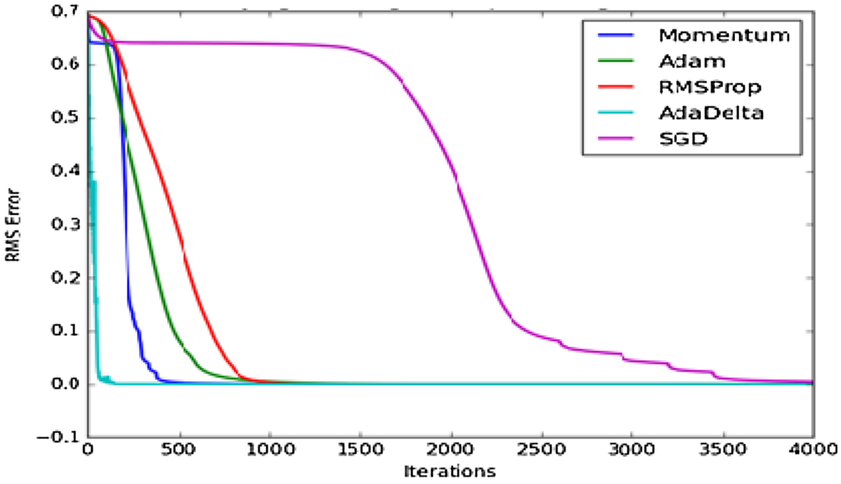

Fig. 12. RMS errors for different updater algorithms.

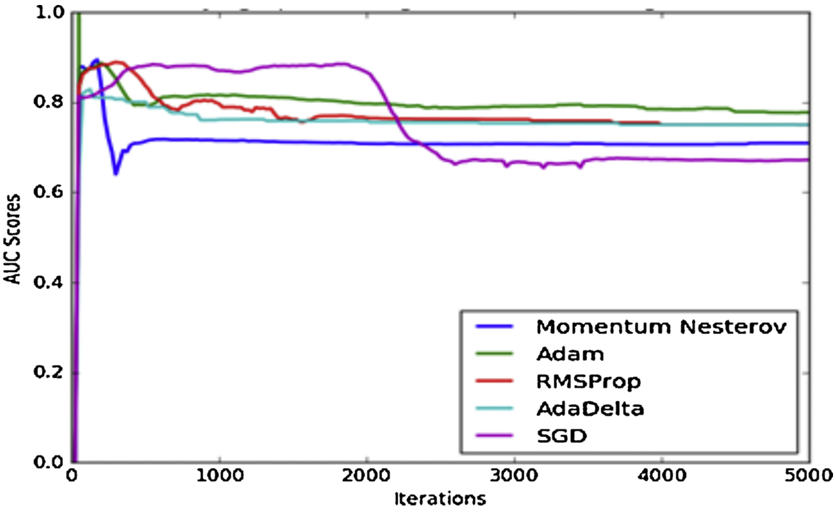

Fig. 13. AUCROC score for different updater algorithm.

5. Conclusion

The study presents a step-wise approach to optimizing neural network architectures, selecting the appropriate algorithms and fine-tuning hyper-parameters, for a complex, nonlinear medical data set. The 8 Layer MLP model, with Adam Decay and Stochastic Gradient Descent, gave an AUC Score of 0.82 at predicting neonatal apnea. While the Deep Autoencoders with an AUC score of 0.83 proved to be the most effective model, the Deep Belief Network had a lower AUC of 0.78. Since the MLP model performs comparably to the deep learning models, it could be

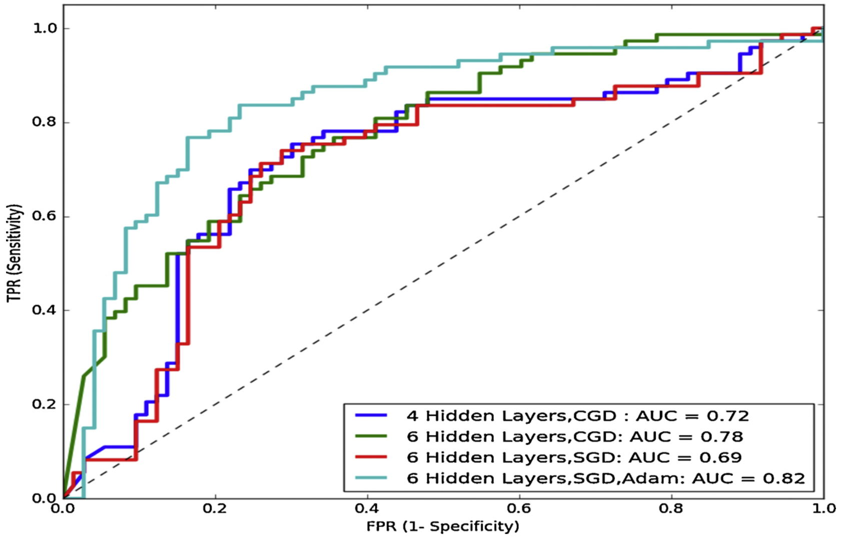

Fig. 14. Comparison between various stages of optimization in MLP models.

Table 8
Comparing different stages of optimization for the MLP model.

Neural Network Model Specification	AUC
MLP with 6 Hidden Layers, Stochastic Gradient Descent (SGD), ReLU	0.69
MLP with 4 Hidden Layers, Conjugate Gradient Descent (CGD), ReLU	0.72
MLP with 6 Hidden Layers, Conjugate Gradient Descent (CGD), ReLU	0.78
MLP with 6 Hidden Layers, ReLU, SGD with Adam Updater	0.82

Table 9
Comparison of performance of machine learning techniques.

Machine Learning Techniques	AUC
K nearest neighbor K = 17	0.55
Random Forest – mtry = 8	0.59
SVM linear – tuned with cost function	*0.68*

inferred that a carefully optimized MLP model was as good at learning inferences from noisy, highly non-linear datasets as deep learning models such as Deep Belief Networks and Autoencoders for predicting neonatal apnea. With the tuning and use of suitable hyperparameters in Deep MLP models, the results showed that it was possible to extract significant improvements in performance, for the case study. Our study also revealed that, compared to shallow models such as Support Vector Machines, K Nearest Neighbour, Decision Trees and Random forest algorithms, the optimized MLP, Deep Autoencoders significantly outperform the others in both, the classification accuracy and F1 measure, in predicting neonatal apnea. The steps involved in designing optimized MLP were generic and could be applied to any predictive problem. The methods used in this work present a generic framework for step-wise empirical optimization of neural networks, selection of optimization algorithms with their hyper parametric optimization. This study provides several empirical inferences that can be used to intuit the factors contributing to the improved/reduced performance of the network, and which hyperparameters are beneficial in the process of model optimization. Further, the study can be extended to optimizing Deep Belief Network, Deep auto-encoders and use of heterogeneous ensemble approach in improving the predictive accuracy.

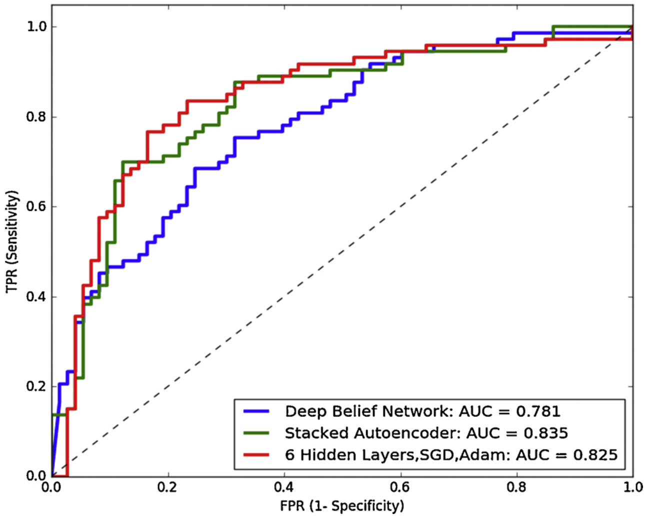

Fig. 15. The area under an ROC curve for deep neural network architectures.

Declaration of Competing Interest

None.

Acknowledgements

The authors are indebted to Kasturba Hospital, and Manipal Academy of Higher Education for providing an opportunity to work on the research problem. This research received no specific grant from any funding agency in the public, commercial, or not-for profit sectors.

Table 10
Comparing final optimized MLP Model with other Deep Neural Network Models.

Neural Network Model Specification	AUC	Recall	F1 Score	Precision
Deep Belief Network (DBN)	0.78	0.70	0.62	0.62
MLP with 6 Hidden Layers, SGD, ReLU activation function, with Adam Updater	0.82	0.72	0.68	0.66
Deep auto encoder	*0.83*	*0.73*	*0.72*	*0.71*

Appendix A

Table A1
Overview of Deep learning approaches.

Method	Characteristics	Advantages / Disadvantages	Architecture / Learning
Restricted Boltzmann Machines (RBMs)	- No lateral connections between hidden (h) and visible (x) nodes.<br>- Symmetric weights<br>- Not seeking global minima, but rather an incremental transformation of feature space.<br>- Uses probabilistic logistic node.<br>- Works with small training sets<br>- Theoretical justification possible	- Hidden and visible layers are conditionally independent allowing sample of one layer using activation of other.<br>- Formation of bipartite graph allows training using gradient based contrastive divergence algorithm	- Deep Belief Networks<br>- Deep Boltzmann machines<br>- Deep energy models
Deep Belief Networks (DBN)	- Constructed using multiple stacked RBMs.<br>- Greedy layer wise training with each layer being RBM.<br>- Directed connections at lower layers and undirected connections at top two layers.	- Type: generative<br>- Initialization of networks weights preventing poor local optima.<br>- Unsupervised training which removes necessity of labelled data.<br>- Computationally expensive due to initialization process.	- Fully connected architecture with multiple hidden layers and partial bidirectional connections.<br>- Unsupervised training (pre / fine tuning)
Deep Boltzmann machines (DBM)	- Multiple hidden layers, fully connected architecture with bidirectional connections	- Type: generative<br>- Ambiguous inputs makes it robust by incorporating top down feedback.	- Pretraining: unsupervised with large amount of unlabeled data<br>- Fine tuning: supervised
Deep Auto-encoder	- Learning efficient encodings with ANN.<br>- Trained to reconstruct its own input, therefore having output vectors same dimensionality as input.<br>- Unsupervised learning which tries to discover generic features of the data.	- Type: generative<br>- Easier to train than RBMs with contrastive divergence and are thus preferred in contexts where RBMs train less effectively.<br>- Effectively decreases if errors are present in first few layers.<br>- Pretraining the network with initial weight can solve this problem.	- Architecture: multiple hidden layers, fully connected architecture with bidirectional connections<br>- Pretraining: unsupervised, fine tuning: supervised<br>- Conjugate gradient method a variant of back propagation is used to train the network.
Sparse Auto- encoder	- Extract sparse features from raw data.	- Making the complex data more meaningful by making categories more separable.<br>- Simple interpretation of complex input data<br>- More robust to noise.	- Achieved by penalizing the hidden unit biases or by directly penalizing the output of hidden unit activations
Stacked Auto- encoders	- Stack many (sparse) encoders in succession and then train them using greedy layer wise training.<br>- In place of fine tuning, a concatenation approach with both hidden features and original features in final or other layers is adopted.	- Type: Generative<br>- Not as accurate as Deep Belief networks<br>- With denoising encoders they are as competitive as Deep belief networks	- Architecture: multiple hidden layers, fully connected architecture with bidirectional connections<br>- Learning:<br>- Pretraining: unsupervised<br>- Fine-tunning: supervised
Denoising Auto encoder	- Stochastically corrupt training instance each time, but still train auto encoder to decode the uncorrupted instances, forcing it to learn conditional dependencies within the instance.	- Robust to noise.<br>- Not as generative as DBNs.	——
Convolutional Neural Networks (CNN)	- Point wise multiplication of two functions to produce a third function is called convolution. In CNN it is used to extract features from input image. The matrix is formed by sliding the filter over the image and computing the dot product is called convolved feature or activation map or feature map.	- Curse of dimensionality for high resolution images is avoided.<br>- Preprocessing is avoided as network learns the filters before doing the actual classification.<br>- Filters are used to exploit spatial locality.<br>- Used when images are input to classification algorithm.	- Algorithm consists of image as a input. Kernel as a convolution filter to be learned and response is the feature map.<br>- Mobile Net 2017, Inception v3 2016, Res Net 2015 are some of the well known CNN models.

Appendix B

Table B1
Literature review based on Deep learning models.

Authors	Objectives	Methodology	Result / Discussion
Zhaohui et al. [8]	- Conventional Deep Belief Network as an effective training method	- Unsupervised feature extraction with DBN followed by supervised learning by standard SVM.	- Variants of deep learning methods can be implemented for medical data analysis.<br>- Knowledge extraction based on extracted features.
Maryam et al. [10]	- Utility of Deep learning concept in Big data analytics	- Deep learning methods for complex pattern extraction, semantic indexing, data tagging, information retrieval and for simplifying descriptive tasks.	- Study deals with applications of deep learning algorithms and architectures for Big data analytics and challenges of adaptation of deep learning algorithms.
Premaladha et al. [13]	- Efficient algorithms to predict Melanoma based on Computer Aided Diagnosis (CAD) system	- Hybrid approach is adopted with Deep learning based neural network and Adaboost SVM.	- High classification accuracy with of 93% slightly better than simple ANN.
Nicholas et al. [26]	- Compare the performance of logistic regression and ordinary regression with ANN based predictive methods for disease risk prediction.	- ANN: varying network architecture structure.<br>- Back propagation: training the network.	- Results suggest a potential for applying deep learning methods to improve disease risk prediction.
Nhathai et al. [27]	- To study human behavior prediction for overweight and obese people through online social networks.	- Social Restricted Boltzmann machine (SRBM) is proposed.	- Model predicts future activity levels of users more accurately and more stably then conventional models.
Hinton et al. [28]	- Describes a nonlinear generalization of PCA that uses an adaptive, multilayer “encoder” network to transform the high dimensional data into a low dimensional code and a similar “decoder” network to recover the data from the code.	- To optimize weights in nonlinear encoders with multiple hidden layers a “pretraining” procedure using RBM is proposed.	- Layer by layer pretraining can also be used for classification and regression.<br>- Pretraining helps in generalization as very little information in the labels are used to slightly adjust the weights found by pretraining.
Kang et al. [29]	- Proposed a deep learning based real time video analyzing module inside the CCTV device to detect and analyze disasters.	- Deep neural network architectures.	Evaluation showed that the average detection time and accuracy for situations involving “fire” demonstrated a higher detection rate than those involving “car accidents”.

a) Activation Functions

Some of the activation functions which are used are defined below:

Rectified Linear Unit (ReLU): An activation function that produces an activation linearly proportional to all positive inputs. It is preferred for larger networks due to its reduced computational complexity and for its ability to produce unsaturated activations even for large inputs. Mathematically, ReLU activation function can be represented as:

$$f(x) = \max(1, x)$$

Exponential Linear Unit (ELU): ELU’s improve over other activation functions like the ReLU function, by allowing negative values that allow the mean activations to be closer to zero. They also overcome the problem of vanishing gradient.

$$f(x) = \left\{\begin{array}{l} x:\ x > 0 \\ \alpha\,(\exp(x) - 1) : x \leq 0 \end{array}\right\}$$

Tanh: The output of a neuron with the Tanh activation function is a non-linear function of the input, represented by the Tanh function as:

$$f(x) = \frac{2}{(1 + e^{(-2x)}) - 1}$$

Sigmoid: Non-linear activation function which is an alternative to the Tanh function that results in an output that is a non-linear function of the inputs to the neuron. Mathematically, the Sigmoid function is represented as:

$$f(x) = \frac{1}{1 + e^{x}}$$

It has been empirically found that Tanh function converges faster than the Sigmoid function.

HardTanh: Activation function that is a linear approximation of the tanh function with the following mathematical representation as:

$$f(x) = \left\{\begin{array}{l} -1 : x < -1 \\ x : -1 \leq x \leq 1 \\ 1:\ x > 1 \end{array}\right\}$$

The function HardTanh may be preferred over the Tanh function due to its reduced computational cost.

LeakyReLU: Rectified Linear Unit (ReLU) activated neurons don’t propagate any output when the input summand is negative. LeakyReLU allow a small constant negative error to be propagated for negative valued inputs, while still activate linearly for positive inputs. Mathematically expressed as:

$$f(x) = \left\{\begin{array}{l} x : x > 0 \\ \alpha x : otherwise \end{array}\right\}$$

where is α a small constant

b) Gradient Descent Algorithms

The Standard Gradient Descent is a first-order optimization algorithm also known as the method of steepest descent, which guarantees convergence to a local minimum for any convex function. The algorithm works by updating negatively the parameters which are to be optimized proportional to the differential or "gradient" of the value function, where *w* is the parameter to be optimized, *G(w)* is the loss/value function, and α is the proportionality constant or learning rate.

$$w := w + \alpha(\nabla G(w))$$

Stochastic Gradient Descent: SGD is a stochastic approximation of the gradient descent optimization algorithm that iteratively minimizes the error or objective function by considering the error for each data point. Defined as G (w), where G(w) is the estimated loss function obtained as a summand of G_i (w), associated with each loss/value function at each iteration i.

SGD is not as direct as the standard gradient descent but is shown to converge significantly faster than the Standard Gradient descent algorithm. SGD can further be optimized by using various learning rate updater algorithms like Momentum, AdaGrad, Adam, to ensure smoother weight updates and a faster, more steady descent to the minima.

Conjugate Gradient Descent: CGD algorithm is another iterative variation of the standard gradient descent optimization algorithm that calculates a sequence of mutually orthogonal vectors, to compute the representation of the parameters using these resultant basis vectors. CGD allows approximation of the value function for large and sparse datasets at reduced computational complexity, albeit restricted to symmetric matrices. CGD algorithm guarantees convergence to minima in at most n iterative steps, equal to the number of mutually orthogonal vectors in the basis.

Line Gradient Descent: Stochastic Gradient Descent with Probabilistic Line Search or Line Gradient Descent (LGD). Probabilistic Line Search is constructed by combining the structure of deterministic methods with notions from Bayesian optimization.

c) Updater Algorithms

Momentum: Algorithm uses a hyperparameter as part of first order equation to change the value of the learning rate, depending on the previous gradient of the loss function. Further allowing a smoothed descent with steps of varying sizes, encourages the gradient descent algorithm to reach the local minima faster, and more reliably.

Nesterov's Momentum: Algorithm operates similarly to the standard Momentum algorithm, albeit adding a correction factor to allow anticipatory gradient updates that provide both stronger convergence and consistency, preventing the descent from straying into a non-optimal path.

*Adagrad:*Algorithm provides another first-order equation for scaling the learning rate according to the history of the gradient. It ensures that for larger gradients, the learning rate is reduced, and similarly, for smaller gradients the learning rate is enhanced.

*RMSProp:*It provides a second-order moment equation to change the learning rate by exponentially decaying the average and not the sum of gradients.

AdaDelta: AdaDelta provides two second-order moment equation to change the learning rate.

Adam: Algorithm uses both second-order and first-order moment equations to update the learning rate, but the moments decrease with time.

Appendix C

Table C1
Description of Features used in Neonatal Apnea Prediction.

Number	Features	Coding	Description
1	Growth categorization	AGA (1)<br>SGA (2)<br>LGA (3)	AGA- appropriate for gestation age<br>SGA- small for gestation age<br>LGA- large for gestation age
2	Steroids	Not given (1)<br>Partial given (2)<br>Complete given (3)	Maternal details
3	Dexa 2	Dexa metha (1)<br>Betha metha(2)<br>Not given (3)	Maternal details – type of steroid given
4	Delivery mode	LSCS (1)<br>VD (2)	Maternal details - caesarean section or vaginal delivery
5	Birth cry	Yes (1)<br>No (2)	–
6	Resusneed	Yes(1)<br>No(2)<br>Not known (3)	Artificial respiration required
7	Mode of Resusneed	Intubation (1)<br>BMV(2) No (3)	BMV – Bag and Mask ventilation

(*continued on next page*)

Table C1 (*continued*)

Number	Features	Coding	Description
8	Surfactant	ERST (1)<br>LRST (2)<br>Provide advice surfactant therapy (3)<br>Not given (4)	Early surfactant therapy<br>Late surfactant therapy
9	Apgar	Apgar score (first minute)<br>Apgar score (five minute)	Risk identification score
10	Birth Weight	Grams (continuous)	–
11	HC_Birth	Head circumference (centimetres)	–
12	HR_day 1	Heart rate (24 hours)	average
13	HR day 2	Heart rate (48 hours)	average
14	HR day 3	Heart rate (78 hours)	average
15	Desaturation episodes(1-3 days)	Number of episodes	continuous
16	Apnea (first week)	Presence of apnea episodes are coded as 1<br>Absence of apnea episodes are coded as 0	Persistence of apnea

References

[1] Rastogi DrAruna. Preterm health Internet: https://www.nhp.gov.in/disease/reproductive-system/female-gynaecological-diseases-/preterm-birth. May 23 2016 March 31 Ministry of Health and Family Welfare, National Health Portal (India); 2017.

[2] Parkhi OM, Vedaldi A, Zisserman A. Deep face recognition. Proceedings of British Machine Vision Conference, 2015 2015;Vol.1. no.3.

[3] Ferreira D, Oliveira A, Freitas A. Applying data mining techniques to improve diagnosis in neonatal jaundice. BMC Med Inform Decis Mak 2012;12(December 143).

[4] Sak H, Senior A, Rao K, Beaufays F. Fast and accurate recurrent neural network acoustic models for speech recognition. arXiv preprint arXiv 2015;1507(06947).

[5] Guha S, Mishra N, Roy G, Schrijvers O. Robust random cut Forest based anomaly detection on streams. Proceedings of the 33rdInternational Conference on Machine Learning. 2016. p. 2712–21.

[6] Bose NK, Liang P. Neural network fundamentals with graphs, algorithms and applications. McGraw-Hill Series in Electrical and Computer Engineering. 1996.

[7] Schmidhuber J. Deep learning in neural networks: an overview. Neural Networks Elsevier 2015;61(January):85–117.

[8] Zhaohui L, Gang Z, Jimmy HX, Qinminh HV. Deep learning for healthcare decision making with EMRs. IEEE Bioinformatics Biomedicine (BIBM) 2014(November):556–9.

[9] Yanming G, Yu L, Ard O, Songyang L, Song W, Michael Liu LS. Deep learning for visual understanding: A review, Elsevier. Neurovcomputing 2016;187(September):27–48.

[10] Maryan NM, Flavio V, Taghi KM, Naeem S, Randall W, Edin M. Deep learning applications and challenges in big data analytics, Springer. J Big Data 2015;2(1).

[11] Soniya SPaul, Singh L. A review on advances in deep learning. Proceedings of the Computational Intelligence: Theories, Applications and Future Directions (WCI) 2016:1–6.

[12] Shanthi D, Sahoo G, Saravanan N. Designing an artificial neural network model for the prediction of thrombo-embolic stroke. Int J Biometric Bioinformatics (IJBB) 2009;3(1):10–8.

[13] Premaladha J, Ravichandran KS. Novel approaches for diagnosing melanoma skin lesions through supervised and deep learning algorithms. Springer. J Med Syst 2016;40(February 4):1–12.

[14] Bellazzi R, Riccardo, Zupan B. Predictive data mining in clinical medicine: current issues and guidelines. Int J Med Inform 2008;77.2:81–97.

[15] Goodwin L, Maher S. Data mining for preterm birth prediction. Proc ACM symposium Appl Comp 2000;1:46–51.

[16] Williamson JR, Bliss DW, Browne DW, Indic P, Bloch-Salisbury E, et al. Individualized apnea prediction in preterm infants using cardio- respiratory and movement signals. Proceedings of IEEE Conference on Body Sensor Networks (BSN) 2013. May.

[17] Nimavat D. Apnea of prematurity Internet:http://emedicine.Medspace.com/article/974971-Overview. Nov. 06 2016 [March 27 2017.

[18] Williamson JR, Bliss DW, Browne DW, Indic P, Bloch-Salisbury E, Paydarfar D. Using physiological signals to predict apnea in preterm infants. Proceedings of 45thAsilomar Conference on Signals, Systems and Computers (ASILOMAR) 2011. Nov.

[19] Vanisree K, Jyothi S. Decision support system for congenital heart disease diagnosis based on signs and symptoms using neural networks. Int J Comput Appl 2011;19(April 6).

[20] Dangare SC, Apte SS. A data mining approach for prediction of heart disease using neural networks. Int J Comp Eng Tech (IJCET) 2012;3(3):30–40.

[21] Townsend D, Monique F. Complimentary artificial neural network approaches for prediction of events in the neonatal intensive care unit. 30th Annual International Conference of the IEEE Engineering in Medicine and Biology Society 2008:4605–8.

[22] Chowdhury DR, Chattergee M, Samanta RK. An artificial neural network model for neonatal disease diagnosis. Int J Artificial Inte Expert Systems (IJAE) 2011;2(August 3):96–106.

[23] Wołk K, Marasek K. Neural-based machine translation for medical text domain. Based on European medicines agency leaflet texts Vol. 64. Procedia Computer Science, Elsevier; 2015. p. 2–9.

[24] Koutsoukas A, Monaghan K, Xiaoli Li, Huam Jun. Deep-learning: investigating deep neural networks hyper-parameters and comparison of performance to shallow methods for modeling bioactivity data. J Cheminform 2017;9(42).

[25] Mhaskar H, Pereverzyev S, Walt M. A deep learning approach to diabetic blood glucose prediction. Front Appl Math Stat 2017;3(4).

[26] Nicholas A, Babak A, David HA. Deep learning and the prediction of human disease risk. 2015. Internet: https://github.com/Lasagne/Lasagne, [June 2018].

[27] Nhathai P, Dejing D, Piniewski B, David K. A deep learning approach for human behavior prediction with explanations in health social networks: social restricted Boltzmann machine (SRBM), Springer. Soc Netw Anal Min 2016;6(September 13):79.

[28] Hinton GE, Salakhutdinov RR. Reducing the dimensionality of data with neural networks. Science 2006;313(5786):504–7.

[29] Byungseok K, Hyunseung C. A deep-learning-based emergency alert system," Elsevier. Ict Express 2016;2(May- June 21 2016):67–70.

[30] Guyon I, Elisseeff A. An introduction to variable and feature selection. J Mach Learn Res 2003(March):1157–82.

[31] Rodriguez-Galiano VF, Ghimire B, Rogan J, Chica-Olma M, Rigil -Sanchez JP. An assessment of the effectiveness of a random forest classifier for land-cover classification. Isprs J Photogramm Remote Sens 2012;67:93–104.

[32] Jolliffee IT. Principal component analysis and factor analysis. Principal component analysis Vol.7. New York: Springer; 1986. p. 115–28.

[33] Tipping M, Bishop C. Probabilistic principal component analysis. J Royal Statis Soc, Series B, (Statis Res) 1999;61(3):611–22.

[34] Schölkopf B, Smola A, Müller KR. Kernel principal component analysis. Proceedings of International Conference on Artificial Neural Networks 1997:583–8.

[35] Mika S, Schölkopf B, Smola AJ, Müller KR, Scholz M, Rätsch G. Kernel PCA and denoising in feature spaces. NIPS 1998;11:536–42.

[36] Han J, Kamber M. Data mining concepts and Techniques. 3rd edition San Fransisco: Elsevier; 1998.

[37] Shirwaikar RD, Mago N, Acharya D, Makkithaya K, Hegde G. Supervised leaning technique for analysis of neonatal data. Proceedings of the 2nd International Conference on Applied and Theoretical Computing and Communication Technology (iCATccT) 2017.

[38] Yang SY, Huang Q, Li LL, Ma CY, Zhang H, Bai R, Teng QZ, Xiang ML, Wei YQ. An integrated scheme for feature selection and parameter setting in the support vector machine modeling and its application to the prediction of pharmacokinetic properties of drugs. Artif Intell Med 2009;46(June 2):155–63.

[39] Xavier G, Bengio Y. Understanding the difficulty of training deep feedforward neural networks. Proceedings of the 13th International Conference on Artificial Intelligence and Statistics (AISTATS) 2010;Vol. 9:249–56.

[40] Shenouda E, Andrews AM. A quantitative comparison of different MLP activation functions in classification. Proceedings of International Symposium on Neural Networks 2006:849–57.
[41] Fletcher R, Reeves CM. Function minimization by conjugate gradients. Comput J 1964;7(2):149–54.
[42] R. Sebastian. An optimization of Gradient descent optimization algorithm, Cornell University Library, Internet: https://arxiv.org/abs/1609.04747.
[43] Mahsereci M, Hennig P. Probabilistic line searches for stochastic optimization. J Mach Learn Res 2017;18:1–59.
[44] Sun S, Chen W, Wang L, Liu X, Liu T. On the depth of deep neural networks: a theoretical View. Proceedings of 30th AAAI Conference on Artificial Intelligence. 2016. p. 2006–72.
[45] Andrew Y. Feature selection, L 1 vs. L 2 regularization, and rotational invariance. Proceedings of the 21stInternational Conference on Machine Learning 2004:78.
[46] Srivastava N, Hinton GE, Krizhevsky A, Sutskever I, Salakhutdinov R. Dropout: a simple way to prevent neural networks from overfitting. J Mach Learn Res 2014;15(1):1929–58.
[47] Sutskever I. Training recurrent neural networks. Diss. University of Toronto; 2013.
[48] Salakhutdinov R, Murray I. On the quantitative analysis of deep belief networks. Proceedings of the 25thInternational Conference on Machine Learning 2008:78.
[49] Erhan D, Bengio Y, Courville A, Manzagol PA, Vincent P, Bengio S. Why does unsupervised pre-training help deep learning? J Mach Learn Res 2010:625–60.
[50] Vincent P, Larochelle H, Lajoie I, Bengio Y, Manzagol PA. Stacked denoising autoencoders: learning useful representations in a deep network with a local denoising criterion. J Mach Learn Res 2011:3371–408.
