# Supplementary material for: Exploring Computational Techniques in Preprocessing Neonatal Physiological Signals for Detecting Adverse Outcomes: Scoping Review
Source: Interact J Med Res. 2024 Aug 20;13:e46946. doi: 10.2196/46946 (PMC11372324; doi:10.2196/46946)
Supplement: Multimedia Appendix 3 [file ijmr_v13i1e46946_app3.zip › Included Papers - Final/3400/Md Mahmud et al. - 2019 - Accelerated Prediction of Bradycardia in Preterm I.pdf]

2019 International Conference on Computing, Networking and Communications (ICNC): Internet Services and Applications

Accelerated Prediction of Bradycardia in Preterm Infants Using Time-Frequency Analysis

Md Shaad Mahmud[1]
Electrical and Computer Engineering
University of New Hampshire
Dover, NH, USA
mdshaad.mahmud@unh.edu

Dr. Honggang Wang[2]
Electrical and Computer Engineering
University of Massachusetts Dartmouth
Dartmouth, MA, USA
hwang1@umassmed.edu

Dr. Yong Kim[3]
Biomedical Engineering
University of Massachusetts Dartmouth
Dartmouth, MA, USA
ykim@umassmed.edu

***Abstract*—The rapid growth of the high-performance data processing algorithms and fusion of wearable technologies has enabled us to continuously monitor the health status of infants. However, monitoring preterm infants is still a challenge due to their frightfully tiny size and their undeveloped skin. In this paper, we have demonstrated a framework of complete monitoring of the preterm infants and real-time accelerated prediction of bradycardia. Real-time prediction of bradycardia episodes in the NICU has the potential to provide quality care to these neonates. In the proposed system we incorporated a multi-GPU gradient boosting algorithm that was able to outperform the traditional CPU's performance. This can overcome the manually maintained progress reports by nurses, which is a major hurdle in the NICU. The system maps the workflow in a Java based responsive application to provide statistical information along with growth charts and reports.The system extracts data with a specific interval to feed the GPU based extreme gradient boosting model. The feature extraction was performed on the time and frequency domain of the heart rate of infants to predict an episode of bradycardia. With an average accuracy of 86% and shortest detection time when compared to the models of other similar products, the proposed system showed that it can improve care time, minimize the skill gap, analyze early disease perdition, and reduce preterm infant's morbidity and mortality.**

***Index Terms*—GPU, Bradycardia, Preterm Infants, Real-time, IoT**

I. Introduction

According to the statistics, millions of infants all around the world have died due to bradycardia. Critical health conditions like this can be predicted and prevented using medical sensors, actuators, and artificial intelligence. Infants born before 37 weeks are considered premature and these premature births occur at a rate of 10% worldwide [1]. Only in the USA, more than 1 in 10 pregnancies will end in a preterm birth that also involve critical heart conditions (CHD). About 25% of the preterm babies are affected by critical CHDs. That is about 40000 births per year [2]. The general definition of Apnea is a pause in the regular breathing of a baby lasting longer than 15-20 seconds. Normal breathing will vary but does not stop for any length of time. During sleep apnea, the infant's heart rate will decrease and that is defined as Bradycardia [2].

Heart rate variability (HRV) is an important indicator of the cardiovascular health of a subject. The autonomic nervous system activity regulates it, thus accurate quantiïňĄcation of heart rate variability provides vital information on the subject's autonomic modulation as well as their cardiovascular health. Furthermore, respiratory sinus arrhythmia is characterized when one's heart rate oscillations occur simultaneously with their respiratory cycle [3]. Though named as an arrhythmia, respiratory sinus arrhythmia is a normal physiological process which characterizes the variations in the heart rate during inspiratory and expiratory phases of the respiratory cycle [3, 4]. It serves as an important role in providing synchronization in between the respiratory and cardiovascular systems. Accurate quantification of respiratory sinus arrhythmia provides critical insights into the mechanisms involved in short-term and long-term cardiorespiratory coupling [2]. Several algorithms have been presented in the literature for characterizing heart rate variability and respiratory sinus arrhythmia in time and frequency domains.

If the heart rate of preterm infants goes below 100 beats per minute (bpm) then it reduces their blood velocity by 10%-50% [4]. In the case of bradycardia, where an infant's heart rate is below 60 bpm, their blood velocity will likely decrease by more than 50%. This results in reduced oxygenated hemoglobin and metabolic byproducts [5]. Hence, preterm infants may go through different degrees of complications, for example, delays in development, cognitive deficits, and retinal disease due to diseases that drastically reduce their heart rates. Our proposed method can aid hospital members for early therapeutic intervention with these diseases [6].

Additionally, Mobile Cloud Computing has appeared in several researches in different medical subdomains. Dinh et al. developed and applied MCC to different medical applications to reduce the limitations of current medical systems, including security, privacy, storage, and data errors [7-10]. These articles will be summarized along with our contributions to previous work in this section. With the advancement of technologies, we can introduce new types of applications to take advantage of their ubiquitous sensing. With an increasing number of sensors, the amount of the data collected also increases, which brings in the concept of big data [8]. To analyze big data, especially to clean all

Authorized licensed use limited to: [illegible] Information Technology Services. Downloaded on September 09,2022 at 00:44:44 UTC from IEEE Xplore. Restrictions apply.

these data quickly, we need a novel approach to utilize any kind of machine learning algorithms. Moreover, to store huge amounts of data is a daunting task, particularly when it is for complex applications such as monitoring preterm infants. This work aims to take advantage of the GPU based platform to develop a medical application for preterm infants. We focus mainly on developing a computational heterogeneous model which can predict a bradycardia event of preterm infants utilizing GPU. Figure 1 depicts the block diagram of the complete system. The advantage of the proposed framework is flexibility of sharing the workload and resources with minimum latency and maximum accuracy.

II. Technical Approach

In our experiment, the bradycardia event was referred to as the heart rate decreasing to below 100 bpm (for t >1.5Sec). In the dataset there are 622 events bradycardia events were detected. And the modified features of each class were randomly divided into the training and test data sets in an estimated ratio of 70% and 30% respectively. The correlation analysis has also been performed on healthy infants and infants with bradycardia. The results show a strong correlation between both high and low frequency components in comparison with standard deviation. In effort to visualize complete statistical informations we have also developed a GUI, which is presented in figure 2.

A. Feature Collection

Time-frequency distributions have been used extensively in analyzing non-stationary signals in joint the time-frequency domain. Thus, they could be used for tracking the dominant fibrillatory frequency in time. There are many time-frequency distributions with different characteristics, thus a systematic way of generating a smaller set of distributions and finding a simplifying relationship between those members becomes relevant [9]. We address this problem to generate novel time-frequency distributions with desirable distributional properties. Furthermore, as the ECG signal is dominated by the ventricular activity of the infant, the extracted arterial waveform usually consists of corrupted data segments. Thus, it is not straightforward to perform time-frequency analysis directly on a signal with missing or corrupted data segments.

This section describes the analysis method for HRV. First, we will discuss the techniques to process IBI time series before we consider HRV handling. Later within the section, the different categories of HRV analysis are discussed. These major characteristics include: time series analysis, frequency-based analysis, nonlinear based metrics which estimate the complexity and similarity; and time-frequency metrics that evaluate frequency components over time.

Temporal locations of the beats are chosen to be R-peaks as it is one of the easiest waves to detect [10]. Compared to other peaks such as P, Q, S and T, typically the R wave has the highest amplitude. Hence, the IBI can be referred to as the time difference in between consecutive R peaks, also termed RR-intervals. These intervals are also known as NN-intervals (normal-to-normal). The statistical or geometrical method can be derived from the RR/IBI series. This method includes, calculating the mean of the IBI, standard deviation of the NN series (SDNN), root mean square of successive differences of RR (RMSSD), the number of successive differences that are greater than n time referred to as non (in this paper, we have used NN50), and the percentage of the intervals that successively differ by more n milliseconds (in our case it is pNN50).

To quantify the variability, power spectrum density (PSD) can be used. The PSD defines the density of the power spectrum at a given time as a function of frequency. This helps to identify the amount of power IBI time series have for a given frequency. In HRV four rhythms/ frequency bands can be distinguished for preterm infants, these are-

Ultra-low frequency (ULF): 0.0001Hz < ULF< 0.003Hz
Very low frequency (VLF): 0.003Hz < VLF < 0.04Hz
Low frequency (LF): 0.04Hz < LF < 0.15Hz
High frequency (HF): 0.15Hz < HF < 0.4Hz

The commonly used linear and cubic spline resampling were shown to overestimate the LFHF ratio with an error that is greater than the error between population differences. But methods like Lomb-Scaller do not require resampling, interpolation, and wavelet transformation, hence do not require the signal to be stationary. Equation 1 is used to calculate frequency component [10].

$$P_{LS}(f) = \frac{1}{\sigma^2} \left(\frac{\left[\sum_{n=1}^{N} \left(X(t_n) - \bar{X}\right) \cos\left(2\pi f (t_n - \tau)\right)\right]^2}{\sum_{n=1}^{N} \cos^2\left(2\pi f (t_n - \tau)\right)} + \frac{\left[\sum_{n=1}^{N} \left(X\left(t_n - \bar{X}\right) \sin\left(2\pi f (t_n - \tau)\right)\right)\right]^2}{\sum_{n=1}^{N} \sin^2\left(2\pi f (t_n - \tau)\right)} \right. \tag{1}$$

Where X and σ^2 are the mean and variance of the time series. As we have mentioned above, it does not require the time series signal to be resampled [11]. It uses only data available to the timing window. Unlike Welch's periodogram, weighted windowing functions do not apply to data in LSP because standard weighting methods cannot be applied to unevenly sampled data. Only frequency analysis will provide the information about how RR time series power has been distributed in the frequency domain. It does not give a full insight of the temporal distribution of the spectrum. To measure or monitor time and frequency information is also known as Time-Frequency analysis. There are two major types of methods to quantify time-frequency analysis 1) windowed Fourier transform and 2) Continuous wavelet transform [11,

Authorized licensed use limited to: CSIRO Information Technology Services. Downloaded on September 09,2022 at 00:44:44 UTC from IEEE Xplore. Restrictions apply.

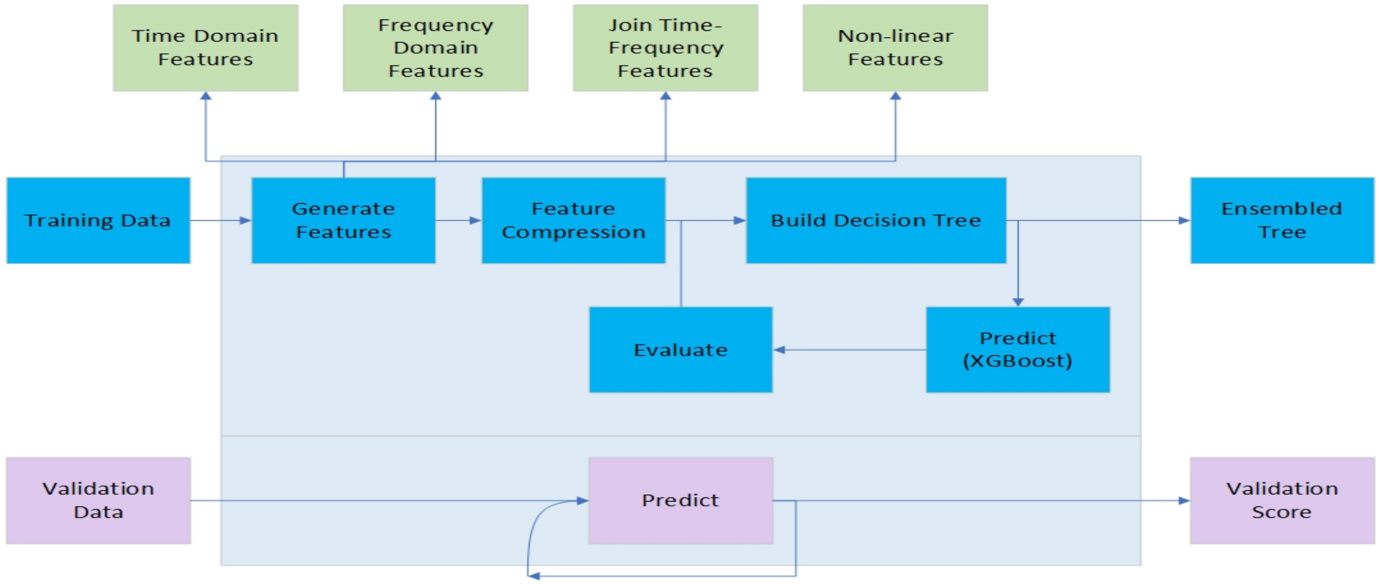

Fig. 1. Block diagram of the proposed system architecture.

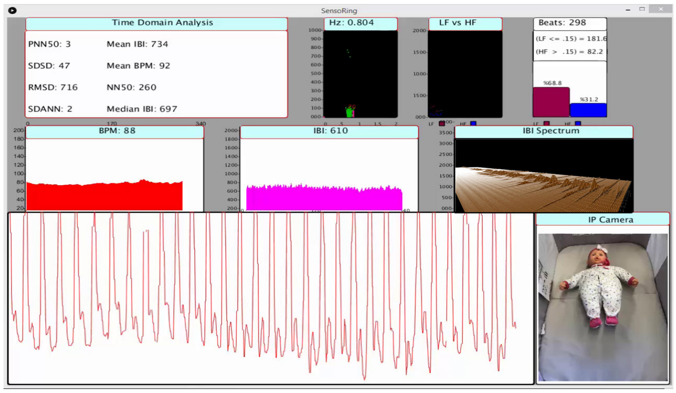

Fig. 2. Graphical user interface for the proposed system.

18]. In this paper, we will be using windowed periodogram which is also known as short-time Fourier transform. Non-linear components are also included in the HRV calculation, such as poincare analysis, entropy measures, and fractal-based measures. Figure 3 shows a poincare plot of one of the subjects prone to bradycarida.

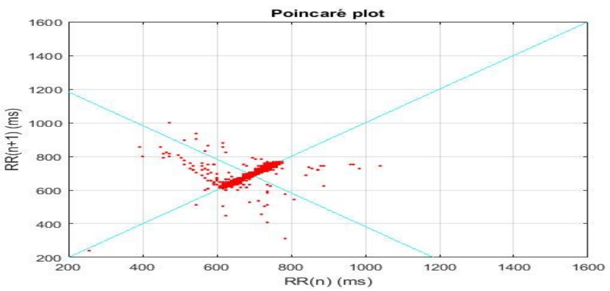

Fig. 3. Poincare plot of the preterm infant 3.

B. Classification and Prediction

To classify the event, we have utilized customized extreme gradient boosting. Before this type of classification is discussed, information regarding its predecessor, gradient boosting, is included. Gradient boosting is a technique which can be applied to both in regression and classification problems and generates a predictive model. The gradient boosting focuses on minimizing the loss of the model by adding weak learners utilizing gradient decent procedures. It is similar to a stage-wise additive model meaning that when one new learner is added at a time, other learners are fixed and unmodified. However, the problem with gradient boosting is it can be parallelized easily, because each tree can be trained simultaneously on various subsets. To overcome this problem, extreme gradient boosting, also known as XGBoost has been introduced. XGBoost can train trees on hundreds of features in seconds, making it one of the fastest and most robust machine learning algorithms available today. XGBoost divides the objective function in two parts; training loss and regularization. The objective function determines how good our model fit the training data. Training loss can help to calculate what is the prediction of our model based on the training data. And loss function is mostly ignored in other boosting tree methods, but it still considered an important function within this research. This function in particular, measures the complexity of the model and helps the model avoid overfitting. This is a different trait than that which can be found in gradient boosting. XGBoost determines the number of steps for solving the equation 2 [12]-

$$\frac{\partial L(y, f^{m-1}(x) f_m(x))}{\partial f_m(x)} = 0 \qquad (2)$$

There are multiple hyperparameters which can be tuned even taking into consideration that these parameters differ from learner to learner. In the proposed method, the learning

Authorized licensed use limited to: CSIRO Information Technology Services. Downloaded on September 09,2022 at 00:44:44 UTC from IEEE Xplore. Restrictions apply.

rate has been set to 0.1, indicating how quickly the model fits the data. If the learning rate is very small then it takes more boosting, hence more time to train the model. Gamma, alpha and lambda are part of the regularization hyperparameter. Gamma is the minimum loss reduction to create a new tree, which is set to 0.3 and the rest is unchanged [12].

$$L(f_m) \infty \sum_{j=1}^{T_m} [G_{jm} w_{jm}] + \frac{1}{2} H_{jm} w_{jm}^2] + \gamma T_m + \frac{1}{2} \lambda \sum_{j=1}^{T_m} w_{jm}^2 + \alpha \sum_{j=1}^{T_m} |w_{jm}|$$
$$= \sum_{j=1}^{T_m} [G_{jm} w_{jm} + \frac{1}{2} (H_{jm} + \lambda) w_{jm}^2 + \alpha |w_{jm}|] + \gamma T_m \quad (3)$$

In the loss function we included a regularization factor in our calculations, as shown in Equation 3. G_{jm} is the gradient sum and H_{jm} is the hessian sum in the j region. Also, γ represents the penalization term and α and λ are the two regularization terms which we have discussed previously in this paper. The feature extraction and pruning can reduce the tree construction problem and thereby speeding up the execution time. Quantization of the input features can be time consuming, if we can map this into GPU it makes the preprocessing take dramatically less time. Compression of the input matrix is applied to take additional advantage of the features' space. Features are compressed down to log2 (maxValue) bits, where maxValue is the maximum number of preprocessed matrix elements. Compressing the data reduces the GPU load by a factor of four or more compared to standard methods. Multiple GPU are used to construct trees in the proposed algorithm. Single GPU is responsible for processing a training subset and splitting these in child nodes. At the same time, it computes the partial gradient histogram of each instance. After merging the histograms, it calculates the split gain of each feature. XGBoost makes prediction on both training and test datasets in order to validate the evaluation process. Each training instance is then mapped on each GPU thread and then iterated throughout all of the trees. Although, this method is not best for the GPU architecture, it still outperforms standard methods that utilize CPU due to high memory bandwidth.

As this is a real-time detection, the feature selection is made based on a simpler algorithm, and at the same time avoiding overfitting. Over fitting causes bad quality output in generalization with new features, although it fits well in the training dataset.

III. Results and Discussion

The data has been collected from our collaborators at the University of Massachusetts Memorial Hospital. Experimental procedure has been discussed before in one of their articles [13]. The study was done on 10 preterm infants in the NICU at the University of Massachusetts Memorial Hospital. The age of the preterm infants was between 29 to 34 weeks with a mean of 31 weeks, and their weights ranged from 843 to 2100g with a mean of 1468g. To measure the infants' vital signs, a three lead ECG monitoring system was attached to the infants for approximately 20-70 hours. This work is implemented with the help of piSpark and the configuration of the machine as follows: Windows 7 platform with Intel(R) Core(TM) i7-3520M 2.9 GHz CPU and 8 G memory.

TABLE I
Comparison of Time Domain Analysis for Both Cases

Measure	Significance	Healthy infant	Bradycardia	Unit
		Mean ±SD	Mean ±SD	
MeanBPM	S	147.3±43.47	126.8±50.9	BPM
MeanRR	S	792.53 ±76.53	686.45 ±85.13	ms
SDNN	S	29.56 ±8.46	21.12 ±11.56	ms
pNN50	S	5.22 ±5.12	2.98 ±2.75	%
RMSSD	NS	26.71 ±7.98	23.54 ±11.54	ms
SDANN	NS	26.45 ±8.45	16.78 ±8.21	ms
HRVti	S	3.45 ±0.98	1.95 ±0.65	n.u.
TINN	S	82.65 ±37.46	90.46 ±64.78	ns

TABLE II
Comparison of Frequency Domain Analysis for Both Cases

Measure	Significance	Healthy infant	Bradycardia	Unit
		Mean ±SD	Mean ±SD	
aLF	NS	0.03 ±0.01	0.01 ±0.012	ms2
aHF	MS	0.0053 ±0.006	0.012 ±0.0086	ms2
aTotal	NS	0.04 ±0.01	0.022 ±0.013	ms2
nLF	MS	0.75 ±0.13	0.65 ±0.18	n.u.
nHF	S	0.26 ±0.13	0.45 ±0.16	n.u.
LFHF	S	0.36 ±0.22	0.28 ±0.17	%

TABLE III
Comparison of Joint Time-Frequency Domain Analysis for Both Cases

Measure	Significance	Healthy infant	Bradycardia	Unit
		Mean ±SD	Mean ±SD	
aLF	S	3.13 ±2.82	2.74 ±2.08	ms2
aHF	NS	14.67 ±8.45	18.92 ±7.79	ms2
aTotal	MS	17.8 ±11.27	21.66 ±9.87	ms2
nLF	MS	0.74 ±0.12	0.54 ±0.13	n.u.
nHF	S	0.26 ±0.11	0.46 ±0.13	n.u.
LFHF	S	0.37 ±0.185	0.168 ±0.092	%
rLFHF	S	0.04 ±0.055	0.01 ±0.01	%

It would be possible to use all the features that were included in the classification of the datasets, however, this can reduce the performance of the output information given by the model. To evaluate the performance of the proposed model, we have shown the receiver operating characteristics (ROC) curve in Figure 4. The ROC curve represents the likelihood of an event as a function of the likelihood of the false alarm. The sensitivity is calculated as true positive (TP) over the summation of TP and false negative (FN). Specificity, this relationship can be referred to as the ratio in

Authorized licensed use limited to: CSIRO Information Technology Services. Downloaded on September 09,2022 at 00:44:44 UTC from IEEE Xplore. Restrictions apply.

between true negative (TN) and the summation of TN and false positive (FP). The predictor was validated using the measurement of the area under the curve (AUC) of the ROC. AUC signifies the probability of a bradycardia event detected by the model. For example, if AUC is 0.5 that means there is a 50 % chance of detecting a TP event, and 100% means it's a perfect model. In our case the average AUC is 86.7%, which is a significant improvement when comparing to other models.

The HRV results show a significant variation in between a healthy infant and an infant prone to bradycardia. Table 1-3 show various features in different domains to compare the results of both cases. To feed the XGBoost model, we have taken only the features which are significant to the label [15, 16]. It has a major impact on reducing the dimensionality which results in a faster output [14]. In the time domain features, all except for RMSSD and SDANN are considered significant. Also, in the frequency domain nHF and LFHF ratios are significant. However, aHF and aLF are considered moderately significant. For joint time-frequency domain, four features are significant and two of them are moderately significant and only one is non-significant. This shows that features in this domain are relatively important compared to other features. Non-linear analyses have also been taken into consideration such entropy and standard deviation of Poincare calculations.

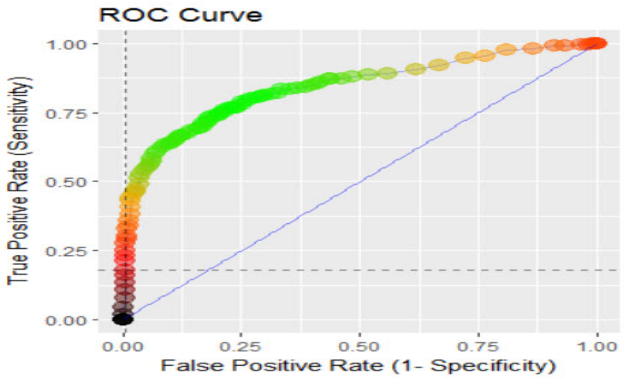

Fig. 4. AUC ROC evaluation metrics.

IV. Conclusion

The main findings of this experiment fit the description of an accelerated prediction of bradycardia on preterm infants. The prediction relies on two factors, how quickly one can extract the statistical information and where all of the data can be processed. Accurate prediction of bradycardia allows caregivers to observe the severity of preterm infants and perform early intervention. The algorithm is consistent among other subjects and capable of identifying events reliably. The proposed method can also allow increased granularity of neurological disease data, thereby improving the risk assessment and predictive analysis of cardiovascular diseases. In the next phase of our research, we plan to extend this work to not only predict but also for preventing bradycardia events. This study was performed on a limited number of patients. Therefore, in the future we expect to add more subjects to assess additional bradycardia and sleep apnea events.

Acknowledgment

The authors thank Premananda Indic and Alan H. Gee for data collection. This research was supported by NSF CCSS1407882, IIS1401711 and 1429120 to Dr. Wang.

References

[1] Preterm birth, World Health Organization, Available at: http://www.who.int/mediacentre/factsheets/fs363/en/. [Accessed on 05th Jun 2017].

[2] World Health Organization. (2018). Preterm birth. [online] Available at: http://www.who.int/news-room/fact-sheets/detail/preterm-birth [Accessed 18 Aug. 2018].

[3] A. Janvier *et al.* "Apnea is associated with neurodevelopmental impairment in very low birth weight infants," *J. Perinatol*, vol. 24, pp.763-8, 2004.

[4] S. Cardoso, M. Silva, and H. Guimaraes, "Autonomic nervous system in newborns: a review based on heart rate variability," *Child's Nervous System*, 33(7), pp.1053-1063.

[5] C. Krueger, J. H. V. Oostrom, and J. Shuster, "A Longitudinal Description of Heart Rate Variability in 28–34-Week-Old Preterm Infants," *Biological Research For Nursing*, vol. 11, no. 3, pp. 261âĂŞ268, 2009.

[6] S. Mahmud, H.Wang, Y. Kim, "Real time non-contact remote cardiac monitoring," Presented at the *2016 IEEE International Conference on Communications (ICC),* Kuala Lumpur, Malaysia, 2016.

[7] H. T. Dinh, C. Lee, D. Niyato, and P. Wang, "A survey of mobile cloud computing: architecture, applications, and approaches," *Wireless Communications and Mobile Computing*, vol. 13, no. 18, pp. 1587-1611, Nov. 2011.

[8] S. Mahmud *et al.* "SensoRing: An Integrated Wearable System for Continuous Measurement of Physiological Biomarkers", Presented at the *2018 IEEE International Conference on Communications (ICC),* MO, USA, 2018.

[9] A. I. Hernandez, J. Dumont, M. Altuve, A. BeuchÃľe, and G. Carrault, "Evolutionary Optimization of ECG Feature Extraction Methods: Applications to the Monitoring of Adult Myocardial Ischemia and Neonatal Apnea Bradycardia Events," *ECG Signal Processing, Classification and Interpretation*, pp. 237-273, Nov. 2011.

[10] G. Blain, O. Meste, A. Blain, and S. Bermon, "Time-frequency analysis of heart rate variability reveals cardiolocomotor coupling during dynamic cycling exercise in humans," *American Journal of Physiology-Heart and Circulatory Physiology*, vol. 296, no. 5, 2009.

[11] S. Mahmud and H. Wang, "An Integrated Wearable Sensor for Unobtrusive, Continuous Measurement of Autonomic Nervous System", *IEEE Internet of Things Journal*, pp.1-1, 2018.

[12] T. Chen and C. Guestrin, "XGBoos: A Scalable Tree Boosting System," *Proceedings of the 22nd ACM SIGKDD International Conference on Knowledge Discovery and Data Mining- KDD 16*, 2016.

[13] A. H. Gee, R. Barbieri, D. Paydarfar, and P. Indic, "Predicting Bradycardia in Preterm Infants Using Point Process Analysis of Heart Rate," *IEEE Transactions on Biomedical Engineering*, vol. 64, no. 9, pp. 2300-2308, 2017.

[14] L. L. C. Kasun, Y. Yang, G.-B. Huang, and Z. Zhang, "Dimension Reduction With Extreme Learning Machine," *IEEE Transactions on Image Processing*, vol. 25, no. 8, pp. 3906-3918, 2016.

[15] S. Mahmud, H. Fang, H. Wang, S. Carreiro, E. Boyer, "Automatic Detection of Opioid Intake Using Wearable Biosensor", *IEEE International Conference on Computing, Networking and Communications (ICNC)*, Maui, Hawaii, 2018.

[16] R. Mitchell and E. Frank, "Accelerating the XGBoost algorithm using GPU computing," *PeerJ Computer Science*, vol. 3, 2017.

[17] N. Khan, P. Jonsson, and M. Sandsten, "Performance Comparison of Time-Frequency Distributions for Estimation of Instantaneous Frequency of Heart Rate Variability Signals," *Applied Sciences*, vol. 7, no. 3, p. 221, 2017.

Authorized licensed use limited to: CSIRO Information Technology Services. Downloaded on September 09,2022 at 00:44:44 UTC from IEEE Xplore. Restrictions apply.
