# Supplementary material for: Exploring Computational Techniques in Preprocessing Neonatal Physiological Signals for Detecting Adverse Outcomes: Scoping Review
Source: Interact J Med Res. 2024 Aug 20;13:e46946. doi: 10.2196/46946 (PMC11372324; doi:10.2196/46946)
Supplement: Multimedia Appendix 3 [file ijmr_v13i1e46946_app3.zip › Included Papers - Final/3416/Gee et al. - 2017 - Predicting Bradycardia in Preterm Infants Using Po.pdf]

Predicting Bradycardia in Preterm Infants Using Point Process Analysis of Heart Rate

Alan H. Gee*, *Student Member, IEEE*, Riccardo Barbieri*, Senior Member, IEEE*, David Paydarfar, and Premananda Indic*, Senior Member, IEEE*

***Abstract*—*Objective:* Episodes of bradycardia are common and recur sporadically in preterm infants, posing a threat to the developing brain and other vital organs. We hypothesize that bradycardias are a result of transient temporal destabilization of the cardiac autonomic control system and that fluctuations in the heart rate signal might contain information that precedes bradycardia. We investigate infant heart rate fluctuations with a novel application of point process theory. *Methods:* In ten preterm infants, we estimate instantaneous linear measures of the heart rate signal, use these measures to extract statistical features of bradycardia, and propose a simplistic framework for prediction of bradycardia. *Results:* We present the performance of a prediction algorithm using instantaneous linear measures (mean area under the curve = 0.79 ± 0.018) for over 440 bradycardia events. The algorithm achieves an average forecast time of 116 s prior to bradycardia onset (FPR = 0.15). Our analysis reveals that increased variance in the heart rate signal is a precursor of severe bradycardia. This increase in variance is associated with an increase in power from low content dynamics in the LF band (0.04–0.2 Hz) and lower multiscale entropy values prior to bradycardia. *Conclusion:* Point process analysis of the heartbeat time series reveals instantaneous measures that can be used to predict infant bradycardia prior to onset. *Significance:* Our findings are relevant to risk stratification, predictive monitoring, and implementation of preventative strategies for reducing morbidity and mortality associated with bradycardia in neonatal intensive care units.**

***Index Terms*—Bradycardia, heart rate variability, interbeat intervals, point process, prediction, preterm infants.**

Manuscript received July 15, 2016; revised October 31, 2016; accepted November 12, 2016. Date of publication June 7, 2017; date of current version August 18, 2017. This work was supported in part by the Hansjörg Wyss Institute for Biologically Inspired Engineering, Harvard University, in part by the National Science Foundation Smart and Connected Health under Grant #1401690, and in part by the National Institutes of Health under Grant R01 GM104987. *Asterisk represents corresponding author.*

*A. H. Gee was with the Wyss Institute at Harvard University, Boston, MA 02115 USA. He is now with the Department of Electrical and Computer Engineering, and Department of Neurology, Dell Medical School, University of Texas at Austin, Austin, TX 78712 USA (e-mail: alangee@utexas.edu).

R. Barbieri is with the Department of Electronics, Information and Bioengineering, Politecnico di Milano, and also with the Department of Anesthesia, Critical Care, and Pain Medicine, Massachusetts General Hospital, Harvard Medical School.

D. Paydarfar was with the Department of Neurology, University of Massachusetts Medical School and the Wyss Institute. He is now with the Department of Neurology, Dell Medical School, University of Texas at Austin.

P. Indic was with the Department of Neurology, University of Massachusetts Medical School. He is now with the Department of Electrical Engineering, University of Texas at Tyler.

Digital Object Identifier 10.1109/TBME.2016.2632746

I. Introduction

INFANT prematurity, defined as <37 weeks gestational age, occurs at a rate of 10% worldwide. These infants experience developmental disorders that can lead to impaired health outcomes [1]–[3]. A common disorder observed in majority of preterm infants is recurrent episodes of apnea and bradycardia, which may cause end organ damage related to hypoxemia (low oxygenation of blood) and ischemia (reduced blood flow) [4]. Although apnea often precedes onsets of bradycardia [5]–[7], apnea and bradycardia can be uncorrelated [8].

In preterm infants, heart rates below 100 bpm result in decreased cerebral blood velocities of ∼10%–50% from baseline, whereas more severe bradycardias (<60 bpm) cause >50% blood velocity reduction [9]. These changes result in reduced cerebral blood velocity and delivery of oxygenated hemoglobin, as well as reduced clearance of metabolic byproducts [10]–[12]. The aggregate result of cardiorespiratory events is hypoxic-ischemic injury in tissue with high-metabolic demands. Intermittent hypoxia in preterm infants is associated with a range of complications including retinopathy, developmental delays, and neuropsychiatric disorders [13]–[15]. To aid clinicians and medical staff, therapeutic interventions, for example as presented in [16], [17], might be most effective if intervention is initiated early in high-risk infants. In particular, implementation of algorithms for detection of apnea–bradycardia [18] and their limited success in prediction [13], [19], [20] might help risk-stratify infants for long-term outcomes, alert clinicians for short-term intervention, and ultimately provide automated therapeutic care that reduce the hypoxic-ischemic complications of preterm cardiorespiratory control.

Heart rate is regulated by a neural feedback control system [21]–[25]. Blood pressure fluctuations are sensed by carotid sinus baroreceptors that send afferent impulses to brainstem and suprabulbar circuits. The circuits' output regulates heart rate through vagal-sympathetic efferent nerves that affect cardiac pacemakers. In pathological circumstances, the heart rate control system can be dysregulated, resulting in episodes of vagally-mediated bradycardia [26]. In theory, pathological instabilities in heart rate should be evident prior to overt bradycardia, which is supported by [27] and [28]. We hypothesize that the immature cardiovascular control system in preterm infants exhibits transient temporal instabilities in heart rate that can be detected as a precursor signal of bradycardia.

We explore this hypothesis by extracting statistical features in heartbeat signals prior to bradycardia and evaluating the

0018-9294 © 2016 IEEE. Personal use is permitted, but republication/redistribution requires IEEE permission.
See http://www.ieee.org/publications_standards/publications/rights/index.html for more information.

Authorized licensed use limited to: CSIRO Information Technology Services. Downloaded on September 09,2022 at 00:28:51 UTC from IEEE Xplore. Restrictions apply.

TABLE I
PCA, WEIGHT, HEART RATE AND NUMBER OF BRADYCARDIAS* IN TEN SUBJECTS

Subject	1	2	3	4	5	6	7	8	9	10
PCA (wks)	$29\frac{3}{7}$	$30\frac{5}{7}$	$30\frac{5}{7}$	$30\frac{1}{7}$	$32\frac{2}{7}$	$30\frac{1}{7}$	$30\frac{1}{7}$	$32\frac{3}{7}$	$30\frac{4}{7}$	$34\frac{2}{7}$
Weight (kg)	1.20	1.76	1.71	0.84	1.67	1.14	1.11	2.10	1.23	1.90
Duration (h)	45.6	43.8	43.7	46.8	48.8	48.6	20.3	24.6	70.3	45.1
Mean [SD] HR (bpm)	155 [10]	131 [14]	131 [13]	167 [9]	143 [16]	137 [8]	162 [13]	141 [13]	150 [13]	156 [16]
*Mild**	65	37	40	15	35	34	12	19	28	29
*Moderate**	11	27	30	20	33	11	15	5	25	11
*Severe**	1	8	10	31	4	11	7	4	44	0
Total	77	72	80	66	72	56	34	28	97	40

utility of these features for prediction. Infant heart rate is innately nonstationary, and conventional analysis methods may not fully capture the idiosyncratic fluctuations of heartbeat signals. Point process analysis can be used to generate real-time, stochastic measures from discrete observables of continuous biological mechanisms [29]–[34]. Introducing a stochastic estimation of heart rate can capture the fleeting instabilities (e.g., instantaneous variance and poles) beyond sampling rate limited measures. In a preliminary study [33], we introduced a point process model of infant heart rate dynamics and showed that a lognormal probability distribution (PD) of interbeat intervals (R–R intervals) provided instantaneous mean and variance estimates of heart rate that exhibited increased clustering preceding severe bradycardias. This statistical feature suggests a possible discrimination that can be used for building a predictive tool.

In this paper, we use instantaneous mean and variance estimates from electrocardiogram (ECG) alone to develop a novel algorithm for near-term prediction of bradycardia in preterm infants. Our goal is to create a real-time, prospective system for clinical practice. We also investigate a selection of other dynamical features to help elucidate the properties of cardiovascular control that can be used for future investigation of bradycardia.

II. Methods

A. Preterm Infant Dataset

We collected data in the neonatal intensive care unit (NICU) at the University of Massachusetts Memorial Healthcare (available at http://physionet.org). Ten preterm infants were studied, with postconceptional age of $29\frac{3}{7}$ to $34\frac{2}{7}$ weeks (mean: $31\frac{1}{7}$ weeks) and study weights from 843 to 2100 g (mean: 1468 g). The infants were spontaneously breathing room air and lacked any congenital or perinatal infection of the central nervous system, intraventricular hemorrhage of grade II or higher, and hypoxic-ischemic encephalopathy. A three-lead ECG signal was recorded (500 Hz) from bedside patient monitors (Intellivue MP70, Philips Medical Systems) for ~20–70 h per infant (see Table I). Respiratory signals, using external inductance bands placed around the chest wall and abdomen, were also recorded (50 Hz) and synchronized using VueLogger, a data acquisition system developed at the Wyss Institute, Harvard University. The study protocol was approved by the University of Massachusetts Medical School Institutional Review Board for human subjects.

TABLE II
PARAMETERS FOR PREDICTION ALGORITHM

Symbol	Quantity	Description	Value
ε	Length of evaluation window	Window of point process indices	3 min
δ	Window increment	Time increment for sliding ε	5 s
τ_r	Refractory period	No predictions made	[δ 5 min]
POS	Positive bradycardia region	Region around bradycardia	$[x-3\ x+6]$ min
NEG	Negative bradycardia region	Region with no bradycardia	All time not POS

We process the ECG signals by calculating peak-to-peak R–R intervals using a modified Pan–Tompkins algorithm and visually remove artifacts due to movement, disconnection, or erroneous peak detections. Based on cerebral oxygenation deficiencies from [9] and clinical practice for bradycardia, we investigate normal heart rates (>100 bpm) and clinical bradycardias: mild (100–80 bpm), moderate (80–60 bpm), and severe (<60 bpm) (see Table I). We define a bradycardia event as an event with a heart rate less than 100 bpm for at least two beats (>1.2 s) in duration. In total there are 622 bradycardia events (178 in training set, 444 in prediction set).

B. Point Process Modeling of Infant Heart Rate

Point process theory provides a stochastic method to estimate instantaneous processes of continuous systems from discrete observables. The heartbeat generation mechanism can be modeled as a point process. The timing of heart contractions are modulated by neural signals from the sympathetic and parasympathetic branches of the autonomic nervous system. These efferent nerves project to cardiac pacemaker cells that control the timing of depolarization and hyperpolarization of each cardiac cell, which macroscopically couple to produce the sequential contraction of the heart. Hence, the occurrence of a heartbeat (i.e., R peak on the ECG) is highly dependent on previous instances, and the heartbeat time series can be highly dynamic due to variable neuron and pacemaker firing.

If we consider a data collection interval $[T_a, T_b]$, the time of each R peak is given by: $T_a \le u_1 < u_2 < \ldots < u_k \le T_b$, where each u_i is the time of the ith R peak. Thus, the corresponding R–R time interval at time k is given by the set $H_k = \{w_k, w_{k-1}, , \ldots, w_{k-p+1}\}$, where $w_k = u_k - u_{k-1}$ and $0 \le p \le k$. Because heart rate is a serial procedure, we can estimate a heartbeat at time k with a p-order linear regression [34]

$$\mu\left(H_k, \theta\left(\mathrm{t}\right)\right) = \theta_o + \sum_{j=1}^{p} \theta_j w_{k-j+1} \qquad (1)$$

where $\theta(t) = \{\theta_o, \ \ldots, \ \theta_j, \ \ldots, \ \theta_k\}$ is the estimation vector of optimized model parameters.

We can then use the estimation in (1) to generate future estimations of heartbeats from an appropriate PD. From [33], we showed that the lognormal PD captures the statistical distribution of R–R intervals associated with bradycardia in preterm infants. Hence, we propose that a collection of infant R–R

Authorized licensed use limited to: CSIRO Information Technology Services. Downloaded on September 09,2022 at 00:28:51 UTC from IEEE Xplore. Restrictions apply.

intervals follow a lognormal PD, and that future R–R values can be estimated from this PD with sample $\mu(H_k, \theta(t)) = \mu(t)$ from the linear regression.

At any given peak u_k, we assume the time until the next heartbeat u_{k+1} obeys a lognormal probability density [31], [33]

$$f_{k+1}(t|H_k, \theta) = \left[\frac{1}{2\pi\sigma(t)^2(t-u_k)^2}\right]^{\frac{1}{2}} \exp\left\{-\frac{1}{2}\frac{(\ln(t-u_k)-\mu(t))^2}{\sigma(t)^2}\right\}. \quad (2)$$

$\mu(t)$ and $\sigma(t)$ are state variables and are estimates over time t, using a local maximum-likelihood optimization to create a continuous estimation of the heartbeat signal (described in Section II-C). We estimate instantaneous mean *M(t)* and variance *V(t)* of the heartbeat signal by applying the traditional transformation from a lognormal to a normal distribution

$$M(t) = e^{\mu(t)+\sigma(t)^2/2}$$
$$V(t) = \left(e^{\sigma(t)^2} - 1\right) e^{2\mu(t)+\sigma(t)^2}. \quad (3)$$

These estimates are instantaneous because for any time t, a new PD is computed at Δt with new state variables $\mu(t)$ and $\sigma(t)$ before the next heartbeat data (see Section II-C). We later use the descriptors *M(t)* and *V(t)* as features to predict onsets of bradycardia.

C. Local Maximum Likelihood Estimation of Parameters

For the ECG interval $[T_a, T_b]$, let l be the length of the local likelihood observation window for $t \in [T_a + l, T_b]$, and let Δt be the incremental time to update the parameters. To compute optimal estimates of $\theta(t)$ and $\sigma(t)$, we define a local joint probability density of $u_{t-l:t}$, with $u_{t-l:t}$ being the collection of R-wave peaks on the interval (*t*–*l*, *t*] that are generated with the previous p R–R intervals [31], [34]. We define the maximum likelihood estimate (MLE) of $\theta(t)$ and $\sigma(t)$ on $(t-l, t]$ to be $\hat{\theta}$ and $\hat{\sigma}$, respectively. The local log likelihood is

$$\log f(u_{t-l:t}|\theta(t)) = \sum_{i=2}^{n_t} w(t-u_i) \log f(u_i - u_{i-1}|H_{u_{i-1}}, \theta(t)) + w(t-u_{n_t}) \log \int_{t-u_{n_t}}^{\infty} f(\vartheta|H_{u_{n_t}}, \theta(t))\, d\vartheta \quad (4)$$

where $w(t-u) = \alpha^{(t-u)}$ is the weighting function for the local likelihood estimation, and α is a weight that assigns the influence of previous observations. We chose $\alpha = 0.98$.

For a given time t, we maximize $\log f(u_{t-l:t}|\theta(t))$ with the previous local MLE of time $t - \Delta t$. The overlap between adjacent local likelihood intervals is large. The state variables $\theta(t)$, $\mu(t)$, and $\sigma(t)$ are updated through this MLE even though new peak data from the ECG are not available. We can obtain estimates of all the descriptors defined in Section II-A. at a resolution of Δt, which if small enough, can be considered instantaneous. We chose $\Delta t = 0.005$ s.

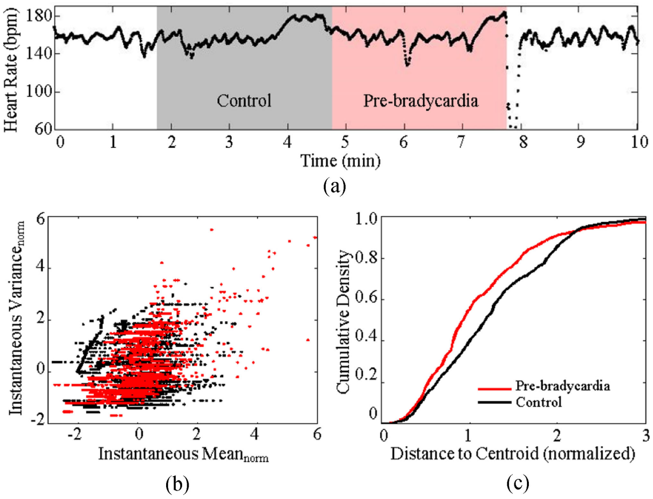

Fig. 1. (a) Example of severe bradycardia for infant 7. The gray region represents a three-minute CW, and the red region represents a three-minute prebradycardia window. Statistical fluctuations of the point process estimation of R–R intervals from these two regions are used to evaluate the likelihood of an impending bradycardia. (b) Normalized mean and normalized variance of the point process indices from seven events of infant 7. The PBW indices cluster distinctly from the CW. (c) The resulting average cumulative density of the indices from the cluster map.

D. Bradycardia Prediction Algorithm

We restrict the training data to the first third of the data (mean of 12 ± 6 h) for each infant, and evaluate our model on the remaining signal. This design was chosen as a first step to simulate prospective, clinical monitoring. To investigate the behavior of varying bradycardia severity, we choose a sample of 7–10 isolated bradycardias [exhibiting normal heart rate for at least 8 min prior to onset and at least one second in duration, see Fig. 1(a)]. Bradycardias in the training set that did not meet this criteria were excluded. We then generate point process estimates of the heartbeat signal with an eighth-order linear regression using methods described in Section II-B.

We denote $\boldsymbol{B}_n$ as the collection of bradycardia examples for infant *n*, where $n = 1, 2, \ldots, 10$. For infant *n*, let *j* be a particular bradycardia event in $\boldsymbol{B}_n$, and $\boldsymbol{M}_j$ and $\boldsymbol{V}_j$ be the set of all *M(t)* and *V (t)*, respectively, in a window of duration $\varepsilon = 3$ min prior to event *j* [see Fig. 1(a)]. A second set of $\boldsymbol{M}_j$ and $\boldsymbol{V}_j$ is also collected for a control region. The choice of ε is influenced by [27] and early work on the behavior of bradycardia features [33]. We normalize both $\boldsymbol{M}_j$ and $\boldsymbol{V}_j$ to have zero mean and unit variance (5). This normalization allows for comparisons across different time frames while preserving oscillations inherent in the signal.

$$\varphi(\boldsymbol{M_j}) = \frac{M_j - \text{mean}(M_j)}{\text{var}(M_j)} \qquad \varphi(\boldsymbol{V_j}) = \frac{V_j - \text{mean}(V_j)}{\text{var}(V_j)}. \quad (5)$$

We take the normalized point process means $\varphi(\boldsymbol{M_j})$ and variances $\varphi(\boldsymbol{V_j})$ and create a $(\varphi(\boldsymbol{M_j}), \varphi(\boldsymbol{V_j}))$ point cloud of all *j* bradycardias in $\boldsymbol{B}_n$ [see Fig 1(b)]. We calculate a cumulative density curve (CDC) by summing the number of $(\varphi(M_j), \varphi(V_j))$ pairs as a function of distance from a

Authorized licensed use limited to: CSIRO Information Technology Services. Downloaded on September 09,2022 at 00:28:51 UTC from IEEE Xplore. Restrictions apply.

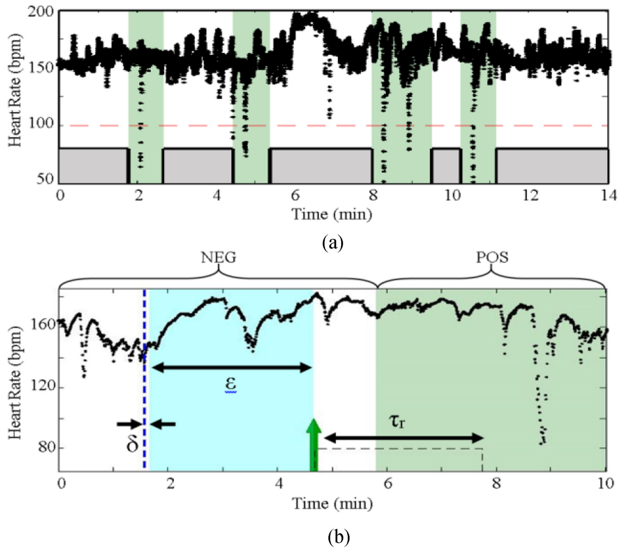

Fig. 2. (a) Green areas denote regions that encompass a bradycardia. Notice that secondary bradycardias (8–9 min) are classified as one event. The other regions (gray) represent regions absent bradycardia. (b) Detailed diagram of one particular bradycardia segment. The parameters used for the algorithm are outlined in the Table II. A prediction is triggered with a time stamp of the leading edge of the EW (green arrow).

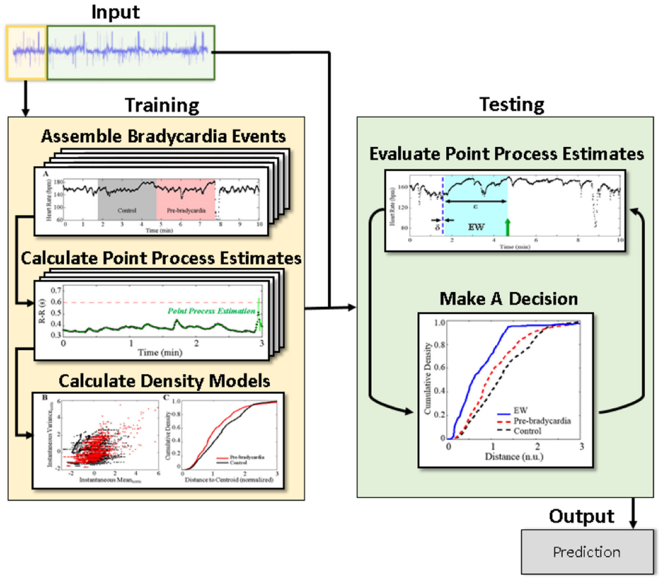

Fig. 3. Schematic of the prediction algorithm. For each subject, a portion of the ECG signal (beige) is used to create the classification models, whereas the remaining signal (green) is used for prediction.

k-means cluster centroid [see Fig. 1(c)]. Steep inclinations in the CDC indicate a dense collection of $(\varphi(\boldsymbol{M_j}, \varphi(V_j))$ pairs. We choose this method as it is sensitive to large densities of $(\varphi(\boldsymbol{M_j}), \varphi(\boldsymbol{V_j}))$ pairs.

We then use the density curves of the prebradycardia and control regions from the training data to evaluate the remainder of the ECG signal. We start the evaluation from the last time stamp of the last bradycardia in the training set. We incrementally slide an evaluation window (EW) by δ s [see Fig. 2(b)]. To determine whether a new input is associated with an impending bradycardia, we define two segments to collect statistical information from: 1) the prebradycardia window (PBW), which marks the window of length ε just prior to the onset of bradycardia, and 2) the control window (CW), which marks the window of length ε just prior to the PBW [see Fig. 1(a)]. The underlying hypothesis is that the heart rate in the CW behaves distinctly from the heart rate just prior to bradycardia onset For the results presented, we choose $\delta = 5$ s.

We calculate the point process estimates using (3) and generate a cumulative curve for the EW and compare this curve with our classifiers by calculating an element-wise Euclidean distance array between the EW curve $(\boldsymbol{EW}_{\rm CDC})$ and to each of the classifiers, $\boldsymbol{PBW}_{\rm CDC}$ and $\boldsymbol{CW}_{\rm CDC}$ (6). The resultant distance arrays are then compared element-wise with each other. A decision statistic is generated by finding the fractional total of points where $\boldsymbol{EW}_{\rm CDC}$ is more similar to the PBW curve (i.e., magnitude of $\Delta\boldsymbol{PBW}_{\rm CDC} < \Delta\boldsymbol{CW}_{\rm CDC}$) (7).

$$\Delta\boldsymbol{PBW}_{\rm CDC} = (\boldsymbol{PBW}_{\rm CDC} - \boldsymbol{EW}_{\rm CDC})^2$$
$$\Delta\boldsymbol{CW}_{\rm CDC} = (\boldsymbol{CW}_{\rm CDC} - \boldsymbol{EW}_{\rm CDC})^2 \quad (6)$$
$$\frac{|\Delta\boldsymbol{PBW}_{\rm CDC} < \Delta\boldsymbol{CW}_{\rm CDC}|}{|\Delta\boldsymbol{PBW}_{\rm CDC}|} \geq \text{threshold}. \quad (7)$$

By design, the threshold is bound from 0 to 1. Any window that satisfies criteria (7) will generate a prediction with a time stamp of the leading window edge [see Fig. 2(b)]. If a prediction is within one EW of a bradycardia (e.g., $\varepsilon = 3$ min), the result will be reported as a true prediction. Any other prediction will be a false alarm. This criterion is driven by our choice of ε in the construction of $\boldsymbol{PBW}_{\rm CDC}$ and $\boldsymbol{CW}_{\rm CDC}$.

Once a prediction is triggered, we can employ a refractory period, τ_r, where no further predictions are made. Clinically, this refractory period can be seen as an intervention period for the infant where clinical staff can start procedures to resolve an impending bradycardia (e.g., sterilize hands and apply physical arousal). For the results presented in Section III-A, we choose $\tau_r = \delta = 5$ s, the minimum refractory time. Although this value may not be clinically relevant, this choice is used to evaluate the frame-by-frame performance of the algorithm. As intervention methods arise, we will be able to tune τ_r accordingly. An overview of the algorithm is provided in Fig. 3.

E. Evaluation Metric for Algorithm

To evaluate the performance of our analysis, we calculate the receiver operating characteristic (ROC) curve. These curves depict the probability of detection (sensitivity = TP/P) as a function of the probability of false alarm (1-specificity = 1–TN/NEG). We define sensitivity as the number of bradycardias that are predicted (TP) over the total number of bradycardias (P). Any prediction within one ε prior to a bradycardia is predicted. For a given bradycardia event x the $[x - \epsilon, x + 6]$ min interval region is classified as the positive bradycardia region (POS) [see Fig. 2(a)]. This criterion ensures that the EW has enough time to pass any peripheral effects of the previous bradycardia. As a consequence, the results achieved by the algorithm are not influenced by bradycardia clustering since we ignore the

Authorized licensed use limited to: CSIRO Information Technology Services. Downloaded on September 09,2022 at 00:28:51 UTC from IEEE Xplore. Restrictions apply.

six-minute regions postbradycardia. The negative-bradycardia regions (NEG) are defined as all regions outside of the POS region [see Fig. 2(a)]. Hence, specificity is defined as the rate of not predicting bradycardias (TN) within NEG.

To evaluate the performance of the predictor, we use the area under the curve (AUC) of the ROC curve to determine the classification power of the algorithm. AUC scores of 0.5 signify an algorithm that performs by chance (i.e., 50% probability of detection and 50% probability of false alarm), and scores of 1.0 signify a perfect predictor.

F. Other Physiological Features of Bradycardia

In our framework, we focus on instantaneous mean *M(t)* and variance *V(t)* as two features for bradycardia prediction. To further understand the physiological effect and implications of instantaneous *M(t)* and *V(t)* on heart rate variability (HRV), we investigate two physiological features known to be influenced by mean and variance in a dynamic system: frequency and complexity. These analyses are evaluated on isolated segments prior to bradycardia and segments within normal heart rate regions.

We evaluate the frequency content of the R–R time series by calculating a traditional Morlet wavelet transform [35] with parameters $\Delta t = 1/3$, $s_o = 2\Delta t$, $\delta j = 1/256$, and $J = 9/\ \delta j$. We also calculate the multiscale entropy (MSE) of a 20-min epoch leading up to a bradycardia to investigate the complexity of heart rate during a diseased state. MSE is a time-varying calculation of sample entropy (SEn) across different time scales [36], and commands a long-time interval for processing.

III. Results

A. Prediction of Infant Bradycardia

After generating a prebradycardia model for each subject, we evaluate the remainder of the ECG signals with the following algorithm parameters: $\varepsilon = 3$ min, $\delta = 5$ s, $\tau_r = \delta$. Fig. 4(a) shows a sample evaluation of a 28-minute segment of discrete heart rates from infant 7. We observe a series of moderate bradycardias at time 10 and 25 min, and a collection of false and true prediction outcomes, red and green, respectively. Within the EW [the blue frame in Fig. 4(a)], instantaneous *M(t)* and *V(t)* estimates are computed (using Section II-B.) from the raw R–R intervals [see Fig. 4(b)]. The resulting CDC is then compared with an *a priori* infant-dependent model of prebradycardia events [see Fig. 4(c)]. For the example in Fig. 4(a) (threshold is set to 0.11 so that FPR is 0.15), the algorithm triggers predictions in multiple locations before the onset of moderate bradycardias with depths of 73 and 74 bpm. In this example, the algorithm successfully predicts the bradycardias in a three minute window prior to the onset.

To evaluate the algorithm, we investigate the ROC curve by varying the detection threshold in (8) by increments of 0.01. We evaluate the predictive capability for all bradycardia, as clinical practice does not differentiate between severities. Fig. 5 depicts the ROC curve for infant 7. Using sensitivity and specificity defined in Section II-E, we observe an AUC of 0.85 for infant 7. Collectively, we achieve a mean AUC of 0.79 ± 0.018 a.u. for a total of 444 bradycardia events with a range of AUC values from 0.72 to 0.93. We also observe mean AUCs for mild bradycardia of 0.81 ± 0.09 a.u., for moderate of 0.80 ± 0.04 a.u., and for severe of 0.76 ± 0.07 a.u. This suggests that the usage of two instantaneous measures *M(t)* and *V(t)*, for advanced prediction of bradycardias, is consistent across infants. Since AUC values of 0.5 depict a random classifier, we observe utility in predicting

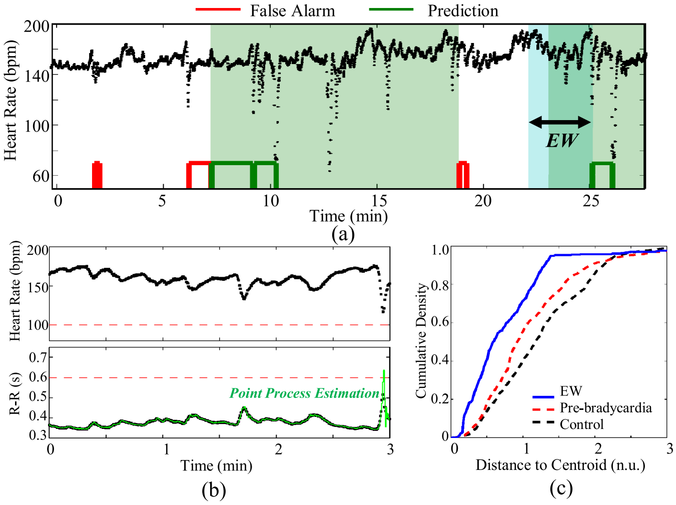

Fig. 4. (a) Prediction outcome for infant 7 ($\varepsilon = 3$ min, $\delta = 5$ s, $\tau_r = \delta$). The instantaneous *M(t)* and *V(t)* indices from the EW (blue region) are calculated and used to predict bradycardia. The red and green blocks (bottom) denote false and positive predictions, respectively, at a FPR of 0.15. (b) The heart rate and interbeat interval data are depicted as black, and the point process estimation is depicted as green. (c) A CDC for the EW (blue curve) is compared with the training models. In this instance, the evaluation curve satisfies a threshold to trigger a prediction.

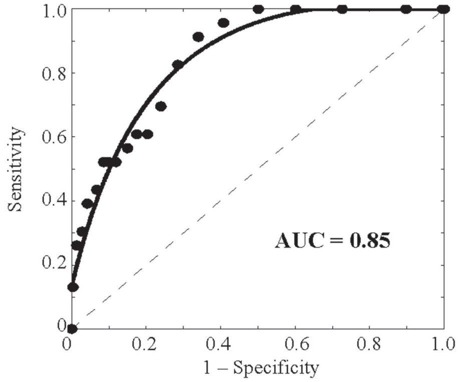

Subject	1	2	3	4	5	6	7	8	9	10
Total AUC	0.79	0.75	0.75	0.79	0.76	0.76	0.85	0.72	0.79	0.93
Events	58	46	61	54	58	37	23	16*	65	26
Mild AUC	0.80	0.70	0.75	0.84	0.76	0.74	0.96	0.72	0.88	0.95
Mod. AUC	0.73	0.77	0.75	0.85	0.77	0.80	0.82	0.83	0.82	0.85
Sev. AUC	--	0.92	0.78	0.72	0.72	0.74	0.71	0.75	0.76	--

Fig. 5. ROC curve for infant 7. The dashed line represents an algorithm preforming by chance (AUC of 0.5). We observe a mean AUC of 0.79 ± 0.018 for 444 bradycardia events. The severity performance is also given. Note "—" denotes no events. *Infant 8 exhibited frequent single skipped-beat episodes that led to instantaneous bradycardia.

Authorized licensed use limited to: CSIRO Information Technology Services. Downloaded on September 09,2022 at 00:28:51 UTC from IEEE Xplore. Restrictions apply.

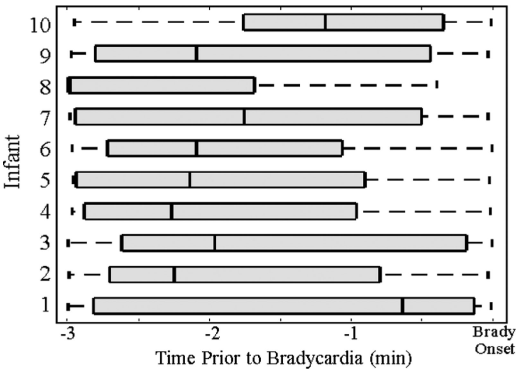

Fig. 6. Quantile representation of the earliest prediction time in a three-minute window preceding bradycardias. We observe a mean forecast time of 116 s across ten infants.

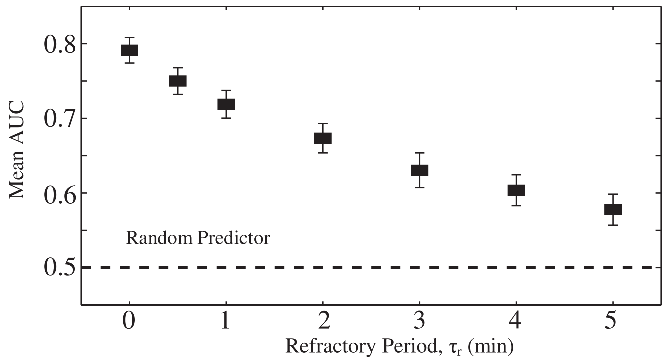

Fig. 7. Mean AUC of prediction algorithm with a varying refractory period. As the refractory period increases, the AUC decreases. There is a tradeoff between the performance of the algorithm and the waiting time after predictions.

bradycardia with only two linear, instantaneous features in the proposed framework.

B. Analysis of Prediction Algorithm

As one common mechanism in humans is cardiorespiratory coupling, we investigate whether predicted bradycardias are associated with preceding apnea. For a baseline comparison, we looked at a statistical approach of using apneas, gained from the respiration signal, as predictor for bradycardia. Using the same parameters (i.e., a 3-min observation window for bradycardia), we obtain a mean AUC of 0.67 for apneas greater than 5 s, and an AUC of 0.34 for apneas greater than 10 s. Additionally, at a FPR of 0.15, we find that, of the predicted bradycardias (215/444), 35% are associated with an apnea greater than 10 s, and 74% are associated with an apnea of at least 5 s. We observe that the prediction of bradycardia is independent of the presence of an apnea. Adding respiratory information as a covariate may improve heart rate estimation [37], and the effect of covariates can be explored in future prediction algorithm development.

We next evaluate the first prediction time within a 3-minute window prior to bradycardia. This advanced warning time allows clinicians and medical staff to initiate intervention protocol before bradycardia onset. Looking at the quantile distribution of forecast times for a false positive rate of 0.15, we observe a mean prediction time of 116.2 s, with a range of median prediction times from 39 to 179 s (see Fig. 6).

For clinical applications, we explore a refractory period after predictions to include a time lag for intervention or to account for the intervention itself. During these periods, subsequent predictions may not be useful. If a bradycardia occurs during the refractory period, the refractory period will still be classified as a true positive prediction. Alternatively, if no bradycardia occurs during the refractor period, the entire refractory time period will be attributed as false positive time. By instituting this refractory period, we observe a decrease in the mean AUC score. For example, if the duration of τ_r is 3 min, we see a decrease in mean AUC to 0.63 (see Fig. 7). This suggests that instantaneous measurements of mean and variance may not be sufficient in predicting bradycardias with long-refractory time frames (e.g., >3 min). This all-or-nothing criteria may not be representative

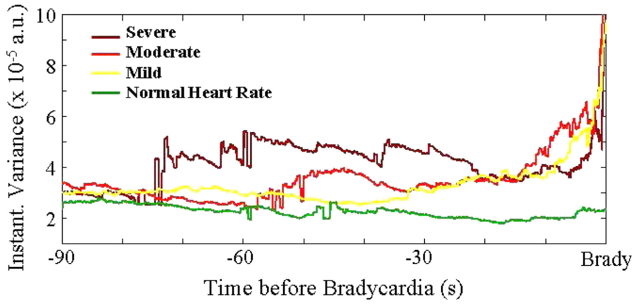

PCA ANALYSIS OF POINT PROCESS PARAMETERS

$M(t)$	Norm	Mild	Mod	Severe
Avg. λ	1.00 ± 0.06 x10^{-6}	1.00 ± 0.17 x10^{-6}	1.00 ± 0.05 x10^{-6}	1.00 ± 0.29 x10^{-6}
p-value	--	0.24	0.57	0.06

$V(t)$	Norm	Mild	Mod	Severe
Avg. λ	(0.33 ± 0.08)x10^{-3}	(0.52 ± 0.11)x10^{-3}	(0.50 ± 0.07)x10^{-3}	(0.71 ± 0.17)x10^{-3}
p-value	--	0.15	0.09	0.03*

** denotes significance compared to respective normal with an unpaired t-test*

Fig. 8. We observe an increase in the average instantaneous variance measure just prior to bradycardia onset. The table details the average eigenvalues (λ) from postconceptional age (PCA) analysis of the *(M(t), V(t))* clusters prior to bradycardias. We observe statistical significance between variance of normal and severe segments.

of the capability of the algorithm. To fully understand the implementation of a refractory period beyond a data-driven approach, a more robust understanding of the physiological mechanism driving bradycardia is needed.

C. Increased Variance as a Feature of Bradycardia

We investigate the influence of $M(t)$ and $V(t)$ on the predictive capability of the algorithm. In a 90-s window prior to bradycardia, we observe a temporal elevation of variance as bradycardia severity increases. The average instantaneous variance increases 80 s prior to severe bradycardia, whereas average variance prior to a mild bradycardia increases at 33 s (see Fig. 8). To determine which parameter, $M(t)$ or $V(t)$, is important to the clustering feature used in the algorithm, we investigate the principal component of the $M(t)$ and $V(t)$ clusters. We observe that as severity

Authorized licensed use limited to: CSIRO Information Technology Services. Downloaded on September 09,2022 at 00:28:51 UTC from IEEE Xplore. Restrictions apply.

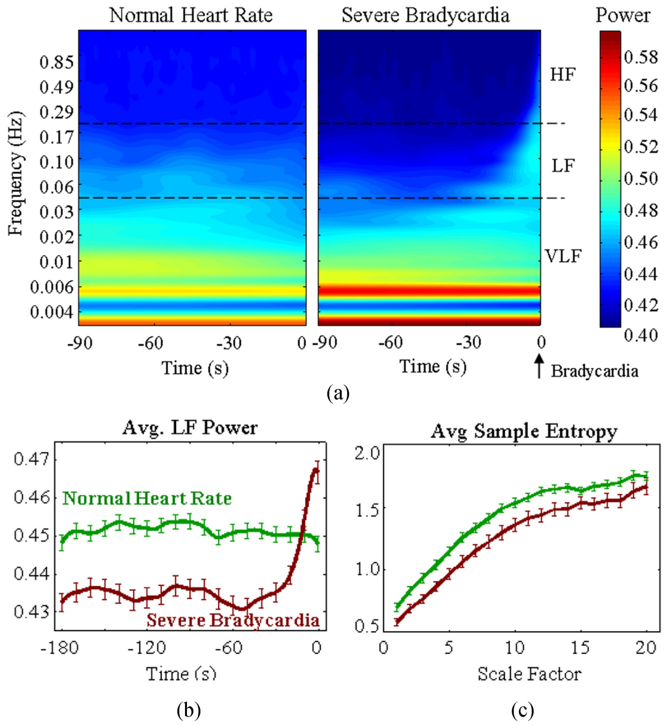

Fig. 9. (a) Temporal evolution of frequency content of R–R time series prior to bradycardia, with a Morlet wavelet transform. (b) Severe bradycardias exhibit decreased power in the LF content compared with normal heart rate segments. (c) We observe decreased SEn across all time scales.

increases, the scaling (i.e., eigenvalue λ) of the $V(t)$ basis vectors becomes greater, where the scaling of the basis vector for $M(t)$ remains the same (see Fig. 8). We perform an unpaired t-test between all the eigenvalues with respect to their parameter. That is, we compare $V(t)$ severe bradycardia λ with $V(t)$ normal bradycardia λ, $M(t)$ severe bradycardia λ with $M(t)$ normal bradycardia λ, $V(t)$ moderate λ with $V(t)$ normal λ, *etc*. We observe statistical significance between $V(t)$ of normal and severe segments (p value $= 0.03$). This observation suggests that the clustering across bradycardia severity is due to the $V(t)$ parameter.

D. Physiological Impact of Increased Variance

We observe a statistical significance in variance between normal heart rate segments and segments prior to severe bradycardia. To understand the physiological implications of increased variance, we explore other linear and nonlinear features in severe bradycardia. The goal is to discover and implement other features into our instantaneous framework.

Using a parametric wavelet transform, we observe an average reduction of 3.3% in power for a region 90 s prior to severe bradycardia onset, when compared with normal segments in the LF spectrum (0.04–0.2 Hz) [see Fig. 9(a)]. The decrease in LF content and increase in very low frequency content [see Fig. 9(a)] equates to slowing of the heartbeat time series and an elongation of the heart rate waveform just prior to bradycardia onset (see Fig. 1). Severe bradycardias then exhibit a mean increase in LF power content 20 s prior to onset, while normal segments exhibit a dispersion of power across the same LF range (see Fig. 9(b)). In the same segments, SEn at the first time scale (raw data) and across all time scales (time-averaged data) are lower in segments prior to severe bradycardia [see Fig. 9(c)]. Thus, we observe decreased complexity in segments prior to bradycardia.

Our findings of decreased entropy are consistent with previous studies on pathological neonatal heart conditions [27], [38]. The finding of decreased complexity aligns with the notion that pathological diseased conditions (e.g., congestive heart failure) exhibit apparent loss in multifractal complexity inherent in normal physiological systems [39]. We propose that bradycardia brings heart rate dynamics in the low frequency range, with increased variance, as generated by a nonlinear system with decreased complexity.

IV. Discussion

This study uses single-channel ECG data to predict bradycardia events with a novel application of point process statistics that can be implemented in future prospective, real-time monitoring studies. Our key result shows that the heartbeat signal exhibits a rise in variance prior to episodes of severe bradycardia. This increase in variance is associated with low, complex power dynamics in LF content and low MSE values prior to bradycardia. Our findings suggest that point process analysis of ECG data is a powerful method for predicting bradycardia in individual infants. However, further research is needed to determine clinical utility in a larger population of preterm infants and to determine if the analytic framework is valid across a wider range of infant ages, weights, and comorbidities.

One study [27] showed a progressive decrease of power in LF (0–0.2 Hz) just prior to bradycardia onset (<80 bpm) in one example. Although we observe lower power content in LF for severe bradycardia when compared with normal heart rate [see Fig. 9(b)], we observe a sharp increase in LF content 20 s prior to bradycardia from an average of 29 severe events. This increase in power is consistent with our observation of increased variance (see Fig. 8). In the future, we can incorporate instantaneous frequency, as well as HRV measures from pole analysis [31] and complexity (e.g., the instantaneous Lyapunov exponents [40] and entropy [41]) into the prediction algorithm.

So far we have considered only two instantaneous point process measures, $M(t)$ and $V(t)$, from ECG to demonstrate a framework for bradycardia prediction. To improve the accuracy, other known precursors of bradycardia should be considered, e.g., apnea-based features. In our study, apnea from the respiratory signal is a relatively poor predictor of bradycardia (see Section III-B). Although respiratory monitoring using chest wall impedance is routinely performed in the NICU, this signal fails to detect apnea during gross movement or airway obstruction, and apneas are often unaccompanied by bradycardia. For future work, other methods to detect apnea events [42] might be considered, including ECG-derived respiration [43], [44]. More analysis is needed to determine whether inclusion of other physiological signals would improve predictive utility, even though respiration improves point process estimation of heart rate [37].

Authorized licensed use limited to: CSIRO Information Technology Services. Downloaded on September 09,2022 at 00:28:51 UTC from IEEE Xplore. Restrictions apply.

Additionally, we can investigate ways to improve our implementation. The cumulative density metric used in the study is a simplistic approach to characterize high densities of normalized *M(t)* and *V(t)* pairs as a function of distance from the overall cluster. We can explore other characterizations of point process patterns, like computing spike-time distances and evaluating the temporal changes in the point process estimates [45], [46], and use other frameworks like machine learning [13], [19], [20] for predicting cardiorespiratory events.

V. Conclusion

We present a novel framework for near-term prediction of bradycardia in preterm infants. We apply point process theory to heart rate and generate linear, instantaneous estimates for ten preterm infants. The point process dynamics just prior to bradycardia onset indicate an increase in variance (see Fig. 8). Across our data set of ten infants, we achieve prediction capability (AUC of 0.79 $\pm$ 0.018) for 444 bradycardia events (see Fig. 5). These result demonstrate the ability to predict the majority of bradycardias with an average of 116 s by using an ECG signal alone.

Improved prediction outcomes can eventually lead to automated, therapeutic intervention to reduce morbidity and mortality associated with bradycardia and prematurity, like apnea and hypoxia [16], [17], and help direct medical attention toward high-risk infants. The current nursing protocol is to initiate manual stimulation after bradycardia has already started. This protocol leaves little time for full antiseptic procedure (e.g., hand wash and gown change), and could promote risk of transmission of infectious agents (e.g., Methicillin-resistant Staphylococcus aureus (MRSA)) due to insufficient time to properly decontaminate. Advanced warning would allow sufficient time to implement the nursing protocol. In the future, an automated advanced warning system could provide a signal for closed-loop systems that triggers a preventive intervention, such as a subarousal stochastic vibration via the infant's mattress [16], [17]. The framework proposed in this manuscript provides a prospective method for automated monitoring of infants and risk stratification. Thus, incorporating other instantaneous features in the proposed framework is important for devising a robust real-time warning system to improve quality of life for infants in the NICU.

Acknowledgment

The authors thank Courtney Temple and Ian Zuzarte for data collection, John Osborne and the Wyss Institute for providing VueLogger, and the reviewers for critical review. A. H. Gee has no financial interests to disclose. R. Barbieri, D. Paydarfar, and P. Indic are coinventors of the Wyss Institute's neonatal stochastic resonance delivery system, patent WO 2013033433. The methods for monitoring physiological signals, which include using point process to model heartbeat intervals, are also included in the patent. This patent was licensed in 2014 by Harvard University to SR-Bio, Inc., a Delaware Corporation. All authors have no financial stake (equity, stock, or income) in SR-Bio, Inc.

References

[1] A. Janvier *et al.*, "Apnea is associated with neurodevelopmental impairment in very low birth weight infants," *J. Perinatology*, vol. 24, pp. 763–768, 2004.

[2] F. Serenius *et al.*, "Neurodevelopmental outcome in extremely preterm infants at 2.5 years after active perinatal care in Sweden," *JAMA*, vol. 309, pp. 1810–1820, 2013.

[3] G. P. Aylward, "Neurodevelopmental outcomes of infants born prematurely," *J. Dev. Behav. Pediatrics*, vol. 35, pp. 394–407, Jul./Aug. 2014.

[4] R. J. Martin and C. G. Wilson, "Apnea of prematurity," *Comprehensive Physiol.*, vol. 2, pp. 2923–2931, 2012.

[5] H. Vyas *et al.*, "Relationship between apnoea and bradycardia in preterm infants," *Acta Paediatrica Scand.*, vol. 70, pp. 785–790, Nov. 1981.

[6] D. J. Henderson-Smart *et al.*, "Incidence and mechanism of bradycardia during apnoea in preterm infants," *Arch. Dis. Child*, vol. 61, pp. 227–232, 1986.

[7] C. J. Upton *et al.*, "Episodic bradycardia in preterm infants," *Arch. Dis. Child*, vol. 67, pp. 831–834, 1992.

[8] D. P. Southall *et al.*, "Undetected episodes of prolonged apnea and severe bradycardia in preterm infants," *Pediatrics*, vol. 72, pp. 541–551, Oct. 1983.

[9] J. M. Perlman and J. J. Volpe, "Episodes of apnea and bradycardia in the preterm newborn: Impact on cerebral circulation," *Pediatrics*, vol. 76, pp. 333–338, 1985.

[10] L. N. Livera *et al.*, "Effects of hypoxaemia and bradycardia on neonatal cerebral haemodynamics," *Arch. Dis. Child*, vol. 66, pp. 376–380, Apr. 1991.

[11] G. Pichler *et al.*, "Impact of bradycardia on cerebral oxygenation and cerebral blood volume during apnoea in preterm infants," *Physiol. Meas.*, vol. 24, pp. 671–680, 2003.

[12] M. B. Schmid *et al.*, "Cerebral oxygenation during intermittent hypoxemia and bradycardia in preterm infants," *Neonatology*, vol. 107, pp. 137–146, 2015.

[13] J. R. Williamson *et al.*, "Forecasting respiratory collapse: Theory and practice for averting life-threatening infant apneas," *Respiratory Physiol. Neurobiology*, vol. 189, pp. 223–231, 2013.

[14] C. F. Poets *et al.*, "Association between intermittent hypoxemia or bradycardia and late death or disability in extremely preterm infants," *JAMA*, vol. 314, pp. 595–603, 2015.

[15] J. M. Di Fiore *et al.*, "Cardiorespiratory events in preterm infants: Interventions and consequences," *J. Perinatology*, vol. 36, pp. 251–258, 2016.

[16] E. Bloch-Salisbury *et al.*, "Stabilizing immature breathing patterns of preterm infants using stochastic mechanosensory stimulation," *J. Appl. Physiol.*, vol. 107, pp. 1017–1027, 2009.

[17] V. C. Smith *et al.*, "Stochastic resonance effects on apnea, bradycardia, and oxygenation: a randomized controlled trial," *Pediatrics*, vol. 136, pp. e1561–e1568, 2015.

[18] A. Thommandram *et al.*, "A rule-based temporal analysis method for online health analytics and its application for real-time detection of neonatal spells," in *Proc. IEEE Int. Congr. Big Data*, 2014, pp. 470–477.

[19] R. D. Shirwaikar *et al.*, "Machine learning techniques for neonatal apnea prediction," *J. Artif. Intell.*, vol. 9, no. 1/3, pp. 33–38, 2016.

[20] J. R. Williamson *et al.*, "Individualized apnea prediction in preterm infants using cardio-respiratory and movement signals," in *Proc. IEEE Body Sensor Netw.*, Cambridge, MA, USA, 2013, pp. 1–6.

[21] D. Paydarfar and D. M. Buerkel, "Collapse of homeostasis during sleep," in *Sleep Science: Integrating Basic Research and Clinical Practice*, Basel, Switzerland: Karger, 1997, pp. 60–85.

[22] L. Glass, "Synchronization and rhythmic processes in physiology," *Nature*, vol. 410, pp. 277–284, 2001.

[23] J. M. Karemaker and K. H. Wesseling, "Variability in cardiovascular control: The baroreflex reconsidered," *Cardiovascular Eng.*, vol. 8, pp. 23–29, 2008.

[24] G. Landesberg *et al.*, "Step baroreflex response in awake patients undergoing carotid surgery: Time-and frequency-domain analysis," *Amer. J. Physiol. Heart Circulatory Physiol.*, vol. 274, pp. H1590–H1597, 1998.

[25] J. M. Legramante *et al.*, "Investigating feed-forward neural regulation of circulation from analysis of spontaneous arterial pressure and heart rate fluctuations," *Circulation*, vol. 99, pp. 1760–1766, 1999.

[26] T. Ketch *et al.*, "Four faces of baroreflex failure hypertensive crisis, volatile hypertension, orthostatic tachycardia, and malignant vagotonia," *Circulation*, vol. 105, pp. 2518–2523, 2002.

[27] G. Pravisani *et al.*, "Short term prediction of severe bradycardia in premature newborns," in *Proc. Comput. Cardiol.*, 2003, pp. 725–728.

Authorized licensed use limited to: CSIRO Information Technology Services. Downloaded on September 09,2022 at 00:28:51 UTC from IEEE Xplore. Restrictions apply.

[28] R. G. Meny *et al.*, "Cardiorespiratory recordings from infants dying suddenly and unexpectedly at home," *Pediatrics*, vol. 93, pp. 44–49, 1994.
[29] E. N. Brown *et al.*, "The time-rescaling theorem and its application to neural spike train data analysis," *Neural Comput.*, vol. 14, pp. 325–346, 2002.
[30] R. Barbieri *et al.*, "A point-process model of human heartbeat intervals: New definitions of heart rate and heart rate variability," *Amer. J. Physiol. Heart Circulatory Physiol.*, vol. 288, pp. H424–H435, 2005.
[31] P. Indic *et al.*, "Point process modeling of interbreath interval: A new approach for the assessment of instability of breathing in neonates," *IEEE Trans. Biomed. Eng.*, vol. 60, no. 10, pp. 2858–2866, Oct. 2013.
[32] G. Valenza *et al.*, "Inhomogeneous point-process entropy: An instantaneous measure of complexity in discrete systems," *Phys. Rev. E*, vol. 89, 2014, Art. no. 052803.
[33] A. H. Gee *et al.*, "Uncovering statistical features of bradycardia severity in premature infants using a point process model," in *Proc. IEEE Eng. Med. Biol.*, Milan, Italy, 2015, pp. 5855–5858.
[34] R. Barbieri and E. N. Brown, "Application of dynamic point process models to cardiovascular control," *Biosystems*, vol. 93, pp. 120–125, 2008.
[35] C. Torrence and G. P. Compo, "A practical guide to wavelet analysis," *Bull. Amer. Meteorological. Soc.*, vol. 79, pp. 61–78, 1998.
[36] M. Costa *et al.*, "Multiscale entropy analysis of biological signals," *Phys. Rev. E Stat. Nonliner Soft Matter Phys.*, vol. 71, 2005, Art. no. 021906.
[37] A. H. Gee *et al.*, "Improving heart rate estimation in preterm infants with bivariate point process analysis of heart rate and respiration,," in *Proc. IEEE Eng. Med. Biol.*, Orlando, FL, USA, 2016, pp. 920–923.
[38] D. E. Lake *et al.*, "Sample entropy analysis of neonatal heart rate variability," *Amer. J. Physiol. Regulatory Integrtive Comparative Physiol.*, vol. 283, pp. R789–R797, 2002.
[39] A. L. Goldberger *et al.*, "Fractal dynamics in physiology: Alterations with disease and aging," *Proc. Nat. Acad. Sci. USA*, vol. 99, Suppl. 1, pp. 2466–2472, 2002.
[40] G. Valenza *et al.*, "Estimation of instantaneous complex dynamics through Lyapunov exponents: a study on heartbeat dynamics," *PLoS One*, vol. 9, 2014, Art. no. e105622.
[41] G. Valenza *et al.*, "Instantaneous monitoring of heart beat dynamics during anesthesia and sedation," *J. Comput. Surg.*, vol. 3, pp. 1–18, 2014.
[42] W. Daw *et al.*, "Medical devices for measuring respiratory rate in children: A Review," *J Adv. Biomed. Eng. Technol.*, vol. 3, pp. 21–27, 2016.
[43] G. Moody *et al.*, "Derivation of respiratory signals from multi-lead ECGs," *Comput. Cardiology*, vol. 12, pp. 113–116, 1985.
[44] E. Helfenbein *et al.*, "Development of three methods for extracting respiration from the surface ECG: A review," *J. Electrocardiology*, vol. 47, no. 6, pp. 819–825, 2014.
[45] D. M. Diez *et al.*, "Algorithms for computing spike time distance and point process prototypes with application to feline neuronal responses to acoustic stimuli," *J. Neurosci. Methods*, vol. 203, pp. 186–192, 2012.
[46] J. D. Victor and K. P. Purpura, "Metric-space analysis of spike trains: Theory, algorithms and application," *Netw, Comput. Neural Syst.*, vol. 8, pp. 127–164, 1997.

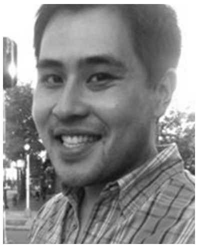

Alan H. Gee (S'16) received the B.S. degree in applied and computational mathematics from the California Institute of Technology, Pasadena, CA, USA, in 2010. He is currently working toward the M.S. and Ph.D. degrees in the Department of Electrical and Computer Engineering, University of Texas at Austin, Austin, TX, USA.

He was a Research Assistant in the Systems and Analysis Group at MIT Lincoln Laboratory, Lexington, MA, USA, and a Postgraduate Research Fellow at the Wyss Institute for Biologically Inspired Engineering at Harvard University, Boston, MA, USA. His research interests include developing intelligent algorithms for real-time prediction of adverse events and translating these systems for mobile, at-home healthcare.

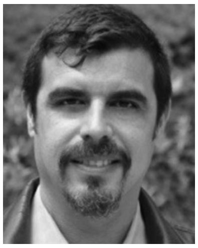

Riccardo Barbieri (M'00–SM'08) received the M.S. degree in electrical engineering from the University of Rome "La Sapienza", Rome, Italy, in 1992, and the Ph.D. in biomedical engineering from Boston University, Boston, MA, USA, in 1998.

He is currently an Associate Professor in the Department of Electronics, Information and Bioengineering, Politecnico di Milano, Milano, Italy. He is also affiliated with the Massachusetts General Hospital, the Massachusetts Institute of Technology, and was with the Wyss Institute for Biologically Inspired Engineering, Harvard University, Boston, MA, USA. His research interests include the development of signal processing algorithms for the analysis of biological systems, with focuses on computational modeling of neural information encoding, and on the application of nonlinear and multivariate statistical models to characterize heart rate variability and cardiovascular control dynamics.

Dr. Barbieri is a member of the American Association for the Advancement of Science, the European Society of Hypertension, the Society for Neuroscience, and the Engineering in Medicine and Biology Society.

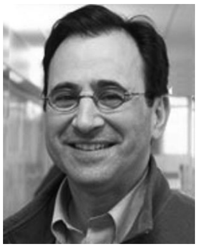

David Paydarfar received the B.S. degree in physics from Duke University, Durham, NC, USA, in 1980, the M.D. degree from the University of North Carolina at Chapel Hill, Chapel Hill, NC, USA, in 1985.

He received the postgraduate training in neurology at the Massachusetts General Hospital and Harvard Medical School, Boston, MA, USA.

He is currently the inaugural Chair of Neurology at the Dell Medical School, The University of Texas at Austin, Austin, TX, USA. He was a Professor and the Executive Vice Chair of Neurology at the University of Massachusetts Medical School, and an Associate Faculty Member of the Wyss Institute for Biologically Inspired Engineering, Harvard University. His research interests include developing new approaches for quantifying and controlling oscillatory behavior at the cellular, tissue and organism levels, and applying these insights to treat pathological conditions, for example preventing apnea of prematurity related to pathological pauses in the central respiratory oscillator.

Dr. Paydarfar is a Fellow of the American Neurological Association.

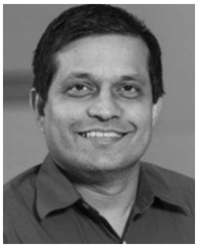

Premananda Indic (M'10–SM'15) received the B.Tech. degree in electrical and electronics engineering, the M.Tech. degree in electrical engineering from the University of Calicut, Kozhikode, Kerala India, and the Ph.D. degree from the Cochin University of Science and Technology, Thrikkakara, Kerala, India, in 2001.

He is currently an Assistant Professor in the Department of Electrical Engineering, University of Texas at Tyler, Tyler, TX, USA. He received his postdoctoral training from Harvard Medical School, Boston, MA, USA, and was a Research Faculty in the Department of Neurology, University of Massachusetts Medical School, Worcester, MA, USA. His research goals include developing signal processing algorithms and wearable sensors to detect and predict abnormal behaviors of physiological signals, and creating appropriate countermeasures to control the dynamics of these systems. His research interests include understanding apnea of prematurity in infants, circadian dysrhythmias in humans, and suicidal ideation in military veterans.

Authorized licensed use limited to: CSIRO Information Technology Services. Downloaded on September 09,2022 at 00:28:51 UTC from IEEE Xplore. Restrictions apply.
