# Supplementary material for: Exploring Computational Techniques in Preprocessing Neonatal Physiological Signals for Detecting Adverse Outcomes: Scoping Review
Source: Interact J Med Res. 2024 Aug 20;13:e46946. doi: 10.2196/46946 (PMC11372324; doi:10.2196/46946)
Supplement: Multimedia Appendix 3 [file ijmr_v13i1e46946_app3.zip › Included Papers - Final/3433/Apurupa Amperayani et al. - 2017 - An algorithm for risk stratification of preterm in.pdf]

An Algorithm for Risk Stratification of Preterm Infants

VenkataNagaSai Apurupa Amperayani[1], Premananda Indic[1], Colm P Travers[2], Riccardo Barbieri[3], David Paydarfar[4], Namasivayam Ambalavanan[2]

[1]Department of Electrical Engineering, University of Texas at Tyler, Tyler, USA
[2]Department of Pediatrics, University of Alabama at Birmingham, Birmingham, USA
[3]Politecnico di Milano, Milano, Italy
[4]Dell Medical School, University of Texas at Austin, Austin, USA

Abstract

Preterm infants have a higher incidence of life-threatening events including apnea (cessation of breathing), bradycardia (slowing of heart rate) and hypoxemia (oxygen de-saturation). In Neonatal Intensive Care Units, clinicians face a demanding task of assessing the risk of these infants based on their physiological signals. In this study, we propose an algorithm of heart rate dynamics that could potentially be employed for risk stratification of preterm infants.

We collected and analysed heart rate (HR) measures in beats per minute (bpm) in 18 preterm infants for 24 hours during oxygen therapy. We investigated whether the HR fluctuations in the first one hour could predict the number of bradycardia events N (i.e. HR below 100 bpm) in the subsequent 23 hours.

Since RR intervals estimated from HR (i.e. RR = 60/HR) in seconds follow a lognormal distribution, we employed an algorithm based on a point process modelling framework to capture HR fluctuations.

We found that the instantaneous variance $\sigma^2(t)$ *calculated by the point process model for the first 1-hour correlates significantly with N. We also found that* $\sigma^2(t)$ *correlates with number of hypoxemia in the subsequent 23 hours. Thus, we conclude that the fluctuations in the HR data captured using a point process model can be used to predict life threatening events.*

1. Introduction

Infants who are born very prematurely with a gestational age less than 32 weeks are at high risk of life threatening events such as apnea (cessation of breathing), bradycardia (slowing of heart rate) and hypoxemia (oxygen de-saturation). The frequency of these cardio-respiratory events has been associated with long-term neurodevelopmental impairment and death [1-6].

The average heart rate of a preterm infant ranges from 120- 180 beats per minute (bpm). A heart rate less than 100 bpm would result in a 10-50% reduction of cerebral blood velocities from baseline [7], which may have adverse effects.

There are no validated indices available to assess the risk for life threatening events or to predict the future neurodevelopmental impairment in these infants. This study explored whether the risk stratification in premature infants can be achieved using heart rate (HR) data obtained from patient monitors.

We hypothesized that HR fluctuations can predict future life threatening events such as bradycardia and hypoxemia. To test this hypothesis, we employed an algorithm using the point process model and studied whether indices that capture RR interval fluctuations can provide information about life threatening events. We evaluated 18 preterm infants managed with servo-controlled oxygen environment for a 24-hour time period and studied whether the indices estimated in the first one hour correlated with the bradycardia as well as hypoxemia events in the following 23 hours.

2. Methods

2.1. Infant Data Set

This study includes the data collected from preterm infants at the Level 4 Regional Neonatal Intensive Care Unit (NICU) at the University of Alabama at Birmingham using the ixTrend and Philips MP70 systems. The electrocardiogram (ECG) data were obtained using a sampling rate of 500 Hz and the HR using a sampling rate of 1Hz. The obtained HR data was converted to inter beat intervals (RR in seconds) using the relation $RR = 60/HR$.

 ISSN: 2325-887X DOI:10.22489/CinC.2017.333-127

Eighteen preterm infants whose parents/legal guardian provided informed consent at the time of enrollment were studied at a gestational age of 27 to 37 weeks (Mean: 33, SD = 3) and weights of 920 to 2380 grams (Mean: 1313; SD = 327) recorded for 24 hours per infant in a servo-controlled oxygen environment. The Institutional Review Board at the University of Alabama at Birmingham approved the study protocol and the study from which the data was obtained is registered with www.clinicaltrials.gov (NCT02794662).

2.2. Point Process Modeling of RR

It has been shown that RR interval of preterm infant follows a lognormal distribution [8-9]. Hence, we employed an algorithm by representing RR as a lognormal distribution and estimated the instantaneous mean $\mu(t)$ as well as instantaneous variance $\sigma^2(t)$. Thus at the kth interval given $RR_k = u_k - u_{k-1}$ and for a time $t > k$ before the next beat occurs, the probability distribution can be represented as

$$f_{k+1}(t \mid HD_k, \beta) = \left[\frac{1}{2\pi\sigma(t)^2(t-u_k)}\right]^{\frac{1}{2}} \exp\left\{-\frac{1}{2}\frac{(\ln(t-u_k)-\mu(t)^2)}{\sigma^2(t)}\right\}$$

where $f_{k+1}(t \mid HD_k, \beta)$ represents lognormal probability distribution and u_k time of kth estimated R-wave peak. HD_k is the set $\{RR_k, RR_{k-1}, \dots\dots, RR_{k-n+1}\}$. The instantaneous mean is represented as a nth order linear regression process as

$$\mu(RR_k, \beta(t)) = \beta_0 + \sum_{i=1}^{n} \beta_i RR_{k-n+1}$$

whose estimation vector $\beta(t)$ is set $\{\beta_0, \dots, \beta_i, \dots, \beta_n\}$.

$\mu\ (t)$ and $\sigma\ (t)$ are the indices estimated using a local maximum-likelihood optimization to obtain a continuous estimation of mean as well as variance of the RR signal by using a history-dependent window of 120 seconds and 4th order linear regression process [10].

We calculated the average of $\mu(t)$ and $\sigma^2(t)$ as $\bar{\mu}$ and $\overline{\sigma^2}$ respectively for the first one hour. For comparison, we also calculated the standard mean m and variance v of the logarithm of the original RR data for the first one hour.

2.3. Estimation of Life Threatening Events

We estimated the number bradycardia events *N*, defined as a heart rate below 100 bpm. For example, since the HR is sampled at 1 Hz, a bradycardia of 10 seconds duration was estimated as $N = 10$. Similarly, we estimated the number hypoxemia events *H*, defined as oxygen level below 85%. These events were estimated for the remaining 23 hour recordings.

We considered all events below predefined thresholds (HR < 100 bpm and hypoxemia as < 85%) to define life threatening events because there is insufficient evidence in the literature to indicate that a specific threshold for a certain duration has greater association with worse outcome. The total number of events experienced by infants is predictive of worse outcomes [11].

Table 1 provides the growth characteristics of the infants along with the life threatening events at the time of study.

Table 1

Gestational Age (GA), Weight at enrolment, Bradycardia events *N* and Hypoxia events *H* of 18 preterm infants

Infant ID	GA at Enrollment (weeks)	Weight at Enrollment (grams)	Events *N*	Events *H*
E2	34.42	1310	112	2396
E4	30.00	1250	84	1449
E5	31.28	1330	33	1905
E7	31.85	1130	148	4027
E9	31.57	1170	3	1372
E10	32.42	920	58	8749
E12	37.57	2380	23	52
E13	29.00	1080	108	1708
E14	31.28	1150	61	6063
E17	33.85	1270	194	6364
E18	35.42	1600	9	3250
E20	27.42	1010	129	2282
E22	32.57	1240	399	5506
E23	31.14	1190	246	8201
E24	31.57	1310	299	8662
E25	31.57	1220	93	5746
E26	37.57	1350	68	6341
E27	36.00	1730	72	4275

3. Results

The results from our analysis are presented below as divided into three main sections. First, we investigated whether the life threatening events were associated with growth characteristics of the infants. Second, we compared the point process indices with the traditional statistical measures. Finally, we studied the correlation between the point process indices (as well as the traditional statistical measures) with the life threatening events.

3.1. Estimation of Point Process Indices

Figure 1 shows the heart rate of infant E25 along with the point process indices of RR estimated for the first one-hour. As the heart rate goes below 100 bpm, the $\mu(t)$ as well as $\sigma^2(t)$ shows a significant increase. While $\mu(t)$ follows the RR interval, $\sigma^2(t)$ captures the variability in the fluctuations of RR during bradycardia events.

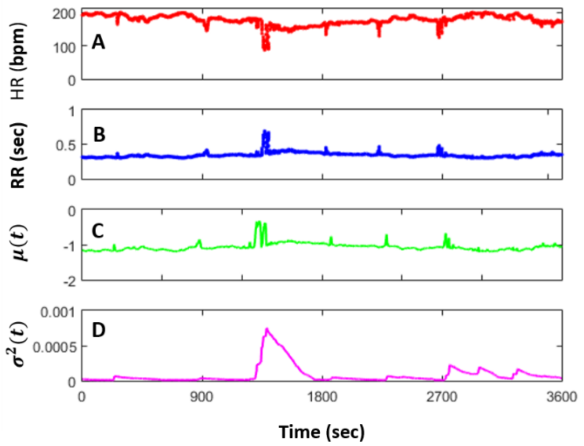

Figure 1. Point Process Indices from Infant E25 in the first one-hour data (A) HR in bpm, (B) RR in seconds, (C) instantaneous mean $\mu(t)$, and (D) instantaneous variance $\sigma^2(t)$

Table 2

$\bar{\mu}$, $\overline{\sigma^2}$, m and v of 18 preterm infants

Infant ID	$\bar{\mu}$	$\overline{\sigma^2}$ (x10^{-3})	*m*	*v* (x10^{-3})
E2	-1.01	0.059	-1.01	4.29
E4	-0.98	0.033	-0.98	1.69
E5	-1.02	0.030	-1.02	2.53
E7	-0.98	0.025	-0.98	2.71
E9	-1.05	0.023	-1.06	3.72
E10	-1.11	0.013	-1.11	1.07
E12	-0.90	0.041	-0.91	3.17
E13	-1.05	0.023	-1.05	0.73
E14	-1.05	0.028	-1.05	0.86
E17	-0.93	0.106	-0.93	5.31
E18	-1.02	0.030	-1.02	3.68
E20	-1.06	0.031	-1.06	1.69
E22	-0.89	0.136	-0.89	2.84
E23	-1.01	0.091	-1.02	2.16
E24	-1.01	0.173	-1.02	7.03
E25	-1.06	0.081	-1.07	9.66
E26	-0.73	0.021	-0.74	11.31
E27	-1.12	0.039	-1.12	1.88

Table 2 presents the averages of $\mu(t)$ and $\sigma^2(t)$ obtained using point process algorithm along with the traditional statistical mean *m* and variance *v* of logarithm of the original RR data.

3.2. Relationship of Life Threatening Events with Growth Characteristics

We investigated whether infant growth characteristics were associated with life threatening events (Table 3). This was undertaken to ensure that the infant vulnerability to events was independent of age and weight, and that each infant requires special attention regardless of growth characteristics. We found that both GA and Weight at the time of enrolment do not correlate with either *N* or *H*.

Table 3

Growth Characteristics vs. Life Threatening Events

Growth	*N*		*H*	
	r	***p***	***r***	***p***
GA	-0.22	0.38	0.03	0.9
Weight	-0.25	0.31	-0.38	0.11

3.3. Relationship of Point Process Indices with Traditional Statistical Measures

We investigated whether the point process indices $\bar{\mu}$ and $\overline{\sigma^2}$ correlate with the standard statistical estimates mean *m* and variance *v*. We found that the $\bar{\mu}$ correlates strongly with *m* (***r*** = 0.99 and ***p*** = < 0.0001), suggesting that no additional information is gained by employing the point process framework for estimating the first order statistics. On the other hand, $\overline{\sigma^2}$ shows no correlation with *v* (***r*** = 0.32 and ***p*** = 0.2), suggesting that the point process model captures the fluctuations in RR differently than the traditional statistical variance. This lack of correlation would point at a possible improvement in risk stratification by using the point process algorithm.

3.4. Relationship of Life Threatening Events with Point Process Indices

We found that the $\overline{\sigma^2}$ correlates strongly with the bradycardia events *N* but correlates modestly with hypoxemia events *H*. None of the standard statistical measures as well as $\bar{\mu}$ correlated with life threatening events (Table 4).

Table 4
Point Process Indices vs. Statistical Measures

**Measures**	***N***		***H***	
	r	***p***	***r***	***p***
$\bar{\mu}$	0.22	0.37	0.01	0.95
$\overline{\sigma^2}$	0.84*	<0.001	0.49*	0.04
m	0.22	0.37	0.002	0.99
v	0.07	0.80	0.29	0.23

*represents significance

4. Conclusion

In this work, we employed an algorithm using point process modelling to risk stratify very preterm infants in terms of life threatening events during their stay in NICU. We found that instantaneous variance obtained from point process algorithm is an important predictor of life threatening events.

Premature infants are at high risk of Sudden Infant Death Syndrome (SIDS) during their post-discharge period. In addition, many of these infants develop neurodevelopmental impairment later in life. The vast amount of physiological signals routinely collected in the NICU may allow risk stratification of infants in terms of not only short-term outcomes but also long-term neuro-developmental outcomes.

A technological system that can assist clinicians to assess these risks in preterm infants is not available currently. The indices obtained from the point process model along with other features derived from physiological signals might be useful to develop a machine-learning framework to assess these risks. Such framework embedded in the current physiological recording system might be useful to clinicians for effective management and treatment of preterm infants in the NICU.

Acknowledgements

This work is supported by National Institute of Health grant U01 HL133536, National Science Foundation, Smart and Connected Health grant IIS 166481, the Agency for Healthcare Research and Quality grant 5T32HS013852-14, the Dixon Fellowship of the University of Alabama at Birmingham and Children's of Alabama, and the European Commission LINK project H2020-692023.

References

[1] Williamson JR, Bliss DW, Paydarfar D. Forecasting respiratory collapse: theory and practice for averting life-threatening infant apneas. Respir Physiol Neurobiol. 2013;189(2):223-31.

[2] Poets CF, Roberts RS, Schmidt B, Whyte RK, Asztalos EV, Bader D, Bairam A, Moddemann D, Peliowski A, Rabi Y, Solimano A. Association Between Intermittent Hypoxemia or Bradycardia and Late Death or Disability in Extremely Preterm Infants. JAMA. 2015 Aug 11;314(6):595-603.

[3] Di Fiore JM, Poets CF, Gauda E, Martin RJ, MacFarlane P. Cardiorespiratory events in preterm infants: interventions and consequences. Journal of Perinatology. 2016 Apr 1;36(4):251-9.

[4] Janvier A, May K, Kokkotis A, Cormier C, Messmer D, Barrington KJ. Apnea is associated with neurodevelopmental impairment in very low birth weight infants. Journal of perinatology. 2004 Dec 1;24(12):763.

[5] Serenius F, Källén K, Blennow M, Ewald U, Fellman V, Holmström G, Lindberg E, Lundqvist P, Maršál K, Norman M, Olhager E. Neurodevelopmental outcome in extremely preterm infants at 2.5 years after active perinatal care in Sweden. Jama. 2013 May 1;309(17):1810-20.

[6] Aylward GP. Neurodevelopmental outcomes of infants born prematurely. Journal of Developmental & Behavioral Pediatrics. 2005 Dec 1;26(6):427-40.

[7] Perlman JM, Volpe JJ. Episodes of apnea and bradycardia in the preterm newborn: impact on cerebral circulation. Pediatrics. 1985 Sep 1;76(3):333-8.

[8] Gee AH, Barbieri R, Paydarfar D, Indic P. Uncovering statistical features of bradycardia severity in premature infants using a point process model. InEngineering in Medicine and Biology Society (EMBC), 2015 37th Annual International Conference of the IEEE 2015 Aug 25 (pp. 5855-5858). IEEE.

[9] Gee AH, Barbieri R, Paydarfar D, Indic P. Predicting Bradycardia in Preterm Infants Using Point Process Analysis of Heart Rate. IEEE Transactions on Biomedical Engineering. 2017 Sep;64(9):2300-8.

[10] Indic P, Paydarfar D, Barbieri R. Point process modeling of interbreath interval: a new approach for the assessment of instability of breathing in neonates. IEEE Transactions on Biomedical Engineering. 2013 Oct;60(10):2858-66.

[11] Di Fiore JM, Walsh M, Wrage L, Rich W, Finer N, Carlo WA, Martin RJ, SUPPORT Study Group of the Eunice Kennedy Shriver National Institute of Child Health, Human Development Neonatal Research Network. Low oxygen saturation target range is associated with increased incidence of intermittent hypoxemia. The Journal of pediatrics. 2012 Dec 31;161(6):1047-52.

Address for correspondence:

Venkata Naga Sai Apurupa Amperayani
Department of Electrical Engineering
The University of Texas at Tyler
3900 University Blvd., Tyler, Texas 75799 - USA.
vamperayani@patriots.uttyler.edu
