# Supplementary material for: Exploring Computational Techniques in Preprocessing Neonatal Physiological Signals for Detecting Adverse Outcomes: Scoping Review
Source: Interact J Med Res. 2024 Aug 20;13:e46946. doi: 10.2196/46946 (PMC11372324; doi:10.2196/46946)
Supplement: Multimedia Appendix 3 [file ijmr_v13i1e46946_app3.zip › Included Papers - Final/3449/Altuve et al - 2015 - Online apnea–bradycardia detection based on hidden.pdf]

Med Biol Eng Comput (2015) 53:1–13
DOI 10.1007/s11517-014-1207-1

ORIGINAL ARTICLE

Online apnea–bradycardia detection based on hidden semi-Markov models

Miguel Altuve · Guy Carrault · Alain Beuchée · Patrick Pladys · Alfredo I. Hernández

Received: 18 July 2013 / Accepted: 27 September 2014 / Published online: 10 October 2014
© International Federation for Medical and Biological Engineering 2014

Abstract In this paper, we propose a new online apnea–bradycardia detection scheme that takes into account not only the instantaneous values of time series, but also their temporal evolution. The detector is based on a set of hidden semi-Markov models, representing the temporal evolution of beat-to-beat interval (RR interval) time series. A preprocessing step, including quantization and delayed version of the observation vector, is also proposed to maximize detection performance. This approach is quantitatively evaluated through simulated and real signals, the latter being acquired in neonatal intensive care units (NICU). Compared to two conventional detectors used in NICU, our best detector shows an improvement on average of around 15 % in sensitivity and 7 % in specificity. Furthermore, a reduced detection delay of approximately 2 s is also observed with respect to conventional detectors.

Keywords Time series analysis · Hidden semi-Markov models · Data mining · Electrocardiogram · Apnea–bradycardia

M. Altuve (✉)
Grupo de Bioingeniería y Biofísica Aplicada, Universidad Simón Bolívar, Caracas, Venezuela
e-mail: maltuve@usb.ve

M. Altuve
Departamento de Tecnología Industrial, Universidad Simón Bolívar, Camurí Grande, Caracas, Venezuela

G. Carrault · A. Beuchée · P. Pladys · A. I. Hernández
Université de Rennes 1, LTSI, Rennes, France
e-mail: guy.carrault@univ-rennes1.fr

A. Beuchée
e-mail: alain.beuchee@chu-rennes.fr

P. Pladys
e-mail: patrick.pladys@chu-rennes.fr

A. I. Hernández
e-mail: alfredo.hernandez@univ-rennes1.fr

G. Carrault · A. Beuchée · P. Pladys · A. I. Hernández
INSERM, U1099, Rennes, France

A. Beuchée · P. Pladys
Pôle Médico-Chirurgical de Pédiatrie et de Génétique Clinique, Néonatologie, CHU Rennes, Rennes, France

1 Introduction

Apnea–bradycardia (AB) episodes are common in preterm infants. AB events are defined as a respiratory pause accompanied with a significant fall in heart rate. The repetition of AB episodes has been related to short-term morbi-mortality and neurological impairment during childhood [27, 35]. As a consequence, preterm infants are continuously monitored by a monitoring system installed in a neonatal intensive care unit (NICU), where several signals (electrocardiogram, respiration, oxygen saturation, …) are acquired and analyzed in real time.

In current NICU monitors, AB is detected by processing the cardiac cycle length (RR interval) extracted from the electrocardiogram (ECG) and by comparing its instantaneous values to a fixed or relative threshold [12, 36, 37]. Even if these threshold-based methods are widely spread, they should be improved for two main reasons: (1) they still generate a relatively high rate of false alarms [1, 9, 10, 23, 26, 30, 31] and (2) they show significant detection delays and therefore induce a delayed application of the therapy [6–8, 13, 33]. To minimize these delays, which is of crucial importance, and to improve detection performance, we propose to take into account not only the instantaneous values of the RR series, but also their temporal evolution

(e.g., their dynamics). We hypothesize that the information contained in the dynamics of these signals could be used to develop a new online AB detector.

Figure 1 illustrates three examples of RR interval time series, acquired from preterm infants, during different pathophysiological states: In a stable electrophysiological state, showing a typical heart rate variability of preterm newborns (blue dashed line), in the presence of a small, but not significant, bradycardia (red dotted line) and in a profound bradycardia state (continuous green line) which has to be detected as soon as possible. It appears clearly that conventional analysis (based only on the amplitude analysis of time series) would not be effective to differentiate the two latter cases. We have also observed that the dynamics of these time series show significant intra- and inter-patient differences.

Several methods have been proposed in the literature for modeling the dynamics of time series: phase space reconstruction models [24, 29, 41], Kalman filters [19, 25, 28], dynamic bayesian networks [32, 45], neural networks [5, 39], hidden Markov models (HMM) [38] or hidden semi-Markov models (HSMM) [20, 44]. In our previous works, we have proposed specific HMM and HSMM architectures and compared them to some of these methods in the context of the analysis of biomedical time series [2, 15]. Results of these works have shown that HMM and HSMM present a number of properties that are particularly suitable for modeling the dynamics of time series estimated from the ECG, such as the RR or QT intervals, or the QRS duration. This paper represents the continuation of these previous works, but is focused on the proposition of new ways

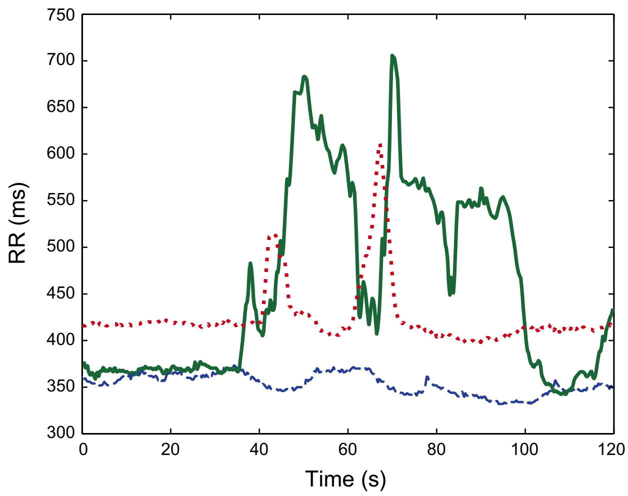

Fig. 1 Examples of RR time series extracted from the ECG of preterm newborns: in a rest state (*blue dashed line*), with slight (nonsignificant) bradycardia episode (*red dotted line*) and in the presence of an apnea–bradycardia episode (*continuous green line*) (color figure online)

to preprocess the observation vector so as to improve the detection performance of AB episodes. Two preprocessing methods are proposed and evaluated: (1) the quantization of the observation vector at different resolutions and (2) the use of a delayed version of the RR time series as an additional dimension of the observation vector.

After the description of the proposed methodology, the online detection scheme is presented in Sect. 2. Then, a quantitative evaluation methodology of the proposed detector on simulated and real signals is described in Sect. 3. Results on simulated and real signals are reported and discussed. Finally, the conclusions and future works are outlined in the last section.

2 A new online apnea–bradycardia detector

This section is divided into two parts. The first one presents briefly the theory of HMM and HSMM and how they can be used for time series modeling. The second one describes how HMM and HSMM can be used efficiently for early AB detection and proposed an adaptation of HMM and HSMM to this problem.

2.1 Architecture of HMM and HSMM

An HMM is a statistical model with unobserved states that produces a sequence of observations, in which the stochastic process to be modeled is Markovian, implying that the change to a future state depends on the current state. An HSMM is similar to a classic HMM, but the unobserved process is semi-Markovian, i.e., a change to a future hidden state depends on both the current state and the time spent on this state.

HMM and HSMM are characterized by a number of M states and the set of parameters $\lambda \triangleq \{a_{ij}, b_i, \pi_i\}$ for HMM and $\lambda \triangleq \{a_{ij}, b_i, \pi_i, p_i\}$ for HSMM where a_{ij} is the transition probability between states i and j ($a_{ii} \neq 0$ for HMM and $a_{ii} = 0$ for HSMM), b_i is the probability of emission of observations, π_i is the probability of the initial state and p_i is the probability of duration for state i.

The time spent on a given HSMM state (sojourn time) is of crucial importance when modeling the dynamics of time series extracted from the ECG. It is possible that some particular states are characterized by a shorter sojourn time (faster dynamics) than others. In HMM, the probability of remaining in one state is not explicit and it follows a geometric law $p_i = a_{ii}^d(1 - a_{ii})$, which is an unrealistic representation of the duration of the observation sequences. In other words, it assumes that a short occupation of a state is more likely than long ones. HSMM define explicitly the sojourn time in each state by using parametric distributions. This representation is most suitable for our application, where Gaussian laws are often observed.

As described in [16, 18], we proposed to represent b_i and p_i as Gaussian distributions: $b_i(\boldsymbol{\mu}, \Sigma)$ and $p_i(\mu_d, \sigma_d)$, where $\boldsymbol{\mu}$ and Σ correspond to the mean and covariance matrix of observations and μ_d and σ_d are the mean and the standard deviation of the sojourn time. In order to avoid negative durations, Gaussian distributions for $p_i(\mu_d, \sigma_d)$ are always positive (truncated in zero).

2.2 Apnea–bradycardia detector

We remind here that the problem is to detect as soon as possible a significant drop in heart rate and, if possible, before the heart rate reaches a given value (often 400 ms in practice). The detection principle can be viewed as an attempt to distinguish between K hypotheses, each one modeled by an HMM or HSMM. In other words, K models (HMM or HSMM) are used to represent K different observation dynamics that may be observed on a time series, each dynamics being associated with distinct physiopathological states.

A learning phase is firstly applied, in order to estimate each λ^k, $\forall k \in \{1, 2, \ldots, K\}$, for each HMM or HSMM, using a training observation dataset and applying an expectation-maximization algorithm. During this phase, the number of states M^k of each model is estimated using the Bayesian information criterion (BIC) [40], and a_{ij}^k and π_i^k are initialized with uniform probabilities, where $b_i^k(\boldsymbol{\mu}, \Sigma)$ is set, as mentioned above, by a Gaussian mixture model. The mean of each Gaussian distribution corresponds to the barycenter of each state in the observation space and is initialized by a K-means algorithm. Figure 2 illustrates this point for a particular observation sequence. In this figure, and as an example, hidden states S1 and S3 represent a portion of the dynamic range of the observation. The hidden state S1 represents the sequence of observations with a steep slope and has a shorter sojourn time (in cyan) than the hidden state S3, which represents the sequence of observations with a reduced slope and larger magnitude (in blue) and a longer sojourn time. Additionally, λ^k for HSMM is

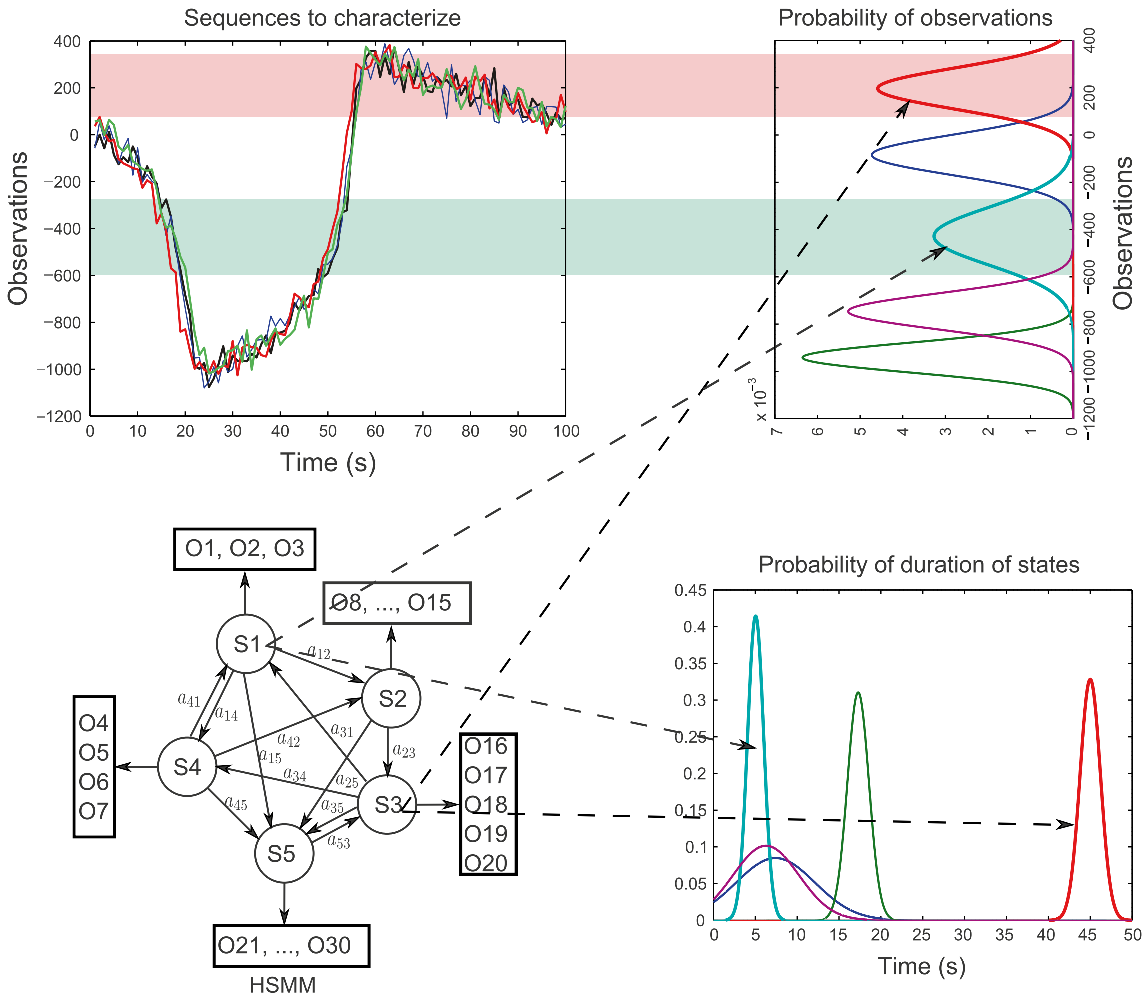

Fig. 2 Initialization of the observation and duration distributions for an HSMM for a specific dynamic sequence (simplified representation). See text for further details (color figure online)

initialized from the parameters of the equivalent HMM, in which the Viterbi algorithm is used to estimate $p_i^k(\mu_d, \sigma_d)$. The Viterbi algorithm and its extension to HSMM are subsequently applied to obtain the final value of λ^k for HMM and HSMM, respectively, through an expectation-maximization step [22]. Appendices 1 and 2 detail the Viterbi algorithm and the extended Viterbi algorithm to HSMM.

Training is achieved when the log-likelihood

$$\mathcal{L}^k = \log P(O_{1:T}|\lambda^k) \tag{1}$$

converges to a maximum value, where $P(O_{1:T}|\lambda^k)$ is the probability that the observation sequence $O_{1:T} = O_1, O_2, \ldots, O_T$ is generated by model k, with parameters λ^k. In this work, we propose to stop the training procedure when:

$$\frac{\left(\mathcal{L}^k\right)^{it} - \left(\mathcal{L}^k\right)^{it-1}}{\left(\mathcal{L}^k\right)^{it}} < 0.01 \tag{2}$$

where *it* is the current iteration.

Once the training phase is completed, the K HMM and the K HSMM are initialized to perform event detection. The detection algorithm computes the log-likelihood, in a sliding window of length T (see Fig. 3), for instant t and model k such as:

$$\mathcal{L}_t^k = \log P(O_{t-T+1:t}|\lambda^k) \tag{3}$$

Online detection of event $\alpha \in \{1, 2, \ldots, K\}$ is finally decided when:

$$\mathcal{L}_t^\alpha - \mathcal{L}_t^k > \delta^{\alpha,k} \quad \forall k \in \{1, 2, \ldots, K\},\ k \neq \alpha \tag{4}$$

where $\delta^{\alpha,k}$ are thresholds chosen experimentally, using the training dataset, to maximize the probability of detection and minimize the probability of error. This decision rule was proposed in order to clearly discriminate different dynamics.

An example of this detection approach is presented in the right side of Fig. 3, which shows the evolution of the log-likelihood of producing the observation vector $O_{t-T+1:t}$, using each model $k(\mathcal{L}_t^k)$. Note that these $(\mathcal{L}_t^k)$ intersect at different points. It can be seen from this example that the application of Eq. 4 with appropriate thresholds can be useful to detect the event of interest (near $t = 120$ s).

2.3 Optimizing the distribution of states within the dynamic range of the observed series

As proposed in [16, 18], the observation vectors used as inputs to the proposed HSMM detector are the result of an automatic ECG detection and segmentation phase that may produce errors. Moreover, the representation of these data into the HMM or HSMM is limited to a finite number of states (M^k). In addition, the AB inference is based on the dynamics (temporal evolution) of the signal, and not on its variability or dispersion. These aspects justify the interest in analyzing the effect of a preprocessing methodology, including different quantization approaches of the HSMM inputs. This quantizer may: (1) reduce the dispersion of the signal, (2) increase the signal to noise ratio (SNR), (3) keep only the most significant dynamics. Moreover, the small dispersion of the observation sequence obtained after the quantization procedure may facilitate the classification of the observation to the states in the recursion step of the Viterbi algorithm.

Discretization presents several advantages, among other things, it allows to: (1) simplify the description of the data, (2) improve the understanding of the data and the results of learning algorithms, (3) reduce the execution time of the algorithms, (4) eliminate the repetition of attributes test in decision trees, (5) treat data by any algorithm, and (6) eliminate some irrelevant attributes.

Two quantizers are applied and tested:

- a uniform quantizer, which is characterized by a constant quantization step Δ_{UQ},
- a non-uniform quantizer, where the quantization edge $\boldsymbol{\Delta}_{\mathrm{NUQ}}$, depends on the distribution of the signal.

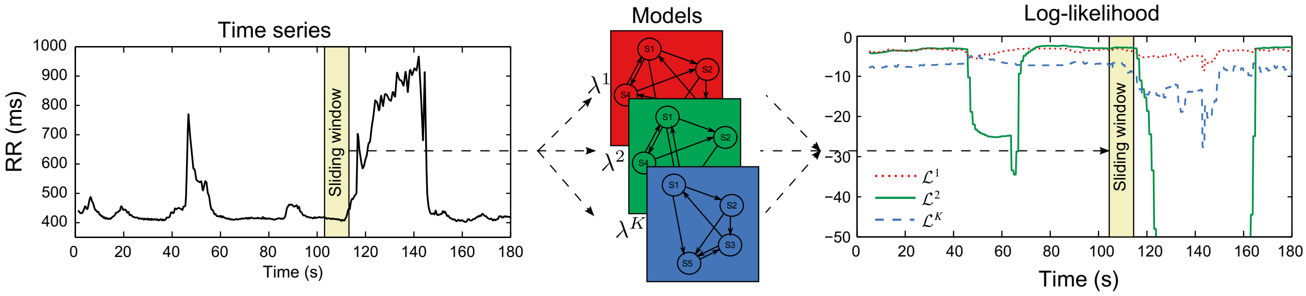

Fig. 3 Methodology for event detection using HMM or HSMM. A sliding window is used to select a portion of the RR time series ($O_{t-T+1:t}$) to be passed through the K models in order to obtain the log-likelihood of producing $O_{t-T+1:t}$ using each model $k(\mathcal{L}_t^k)$ (color figure online)

The optimal value of Δ_{UQ} is estimated by using the rules detailed in Algorithm 1. Vector $\mathbf{\Delta}_{NUQ}$ is found by comparing the cumulative sum of the values of the normalized histogram with respect to a threshold δ_{NUQ}, as reported in Algorithm 2. K classes of the variable to quantify are concatenated using the same number of samples per class. Algorithm presents the computation of the optimal $\mathbf{\Delta}_{NUQ}$.

Algorithm 1 Pseudo-code to obtain the optimal Δ_{UQ}.

1: Initialize Δ_{QU} based on an *a priori*: the median of the abolute value of the derivative of the observation sequence $\mu_{1/2}(|\frac{dx}{dt}|)$
2: Estimate M^k using the BIC
3: Estimate λ^k
4: Perform the detection
5: Determine D according to Eq 11
6: Modify the value of Δ_{QU}
7: Repeat 2-6 until obtain the minimum value of D

Algorithm 2 Pseudo-code of the proposed method for non uniform quantization

```
Construct the vector x as the concatenation of the variable to quantify
using the same number of samples per class
Determine y = hist(x) the normalized histogram of the variable x
Define the threshold δ_NUQ
k = 1
j = 1
for i = 1 to length(y) do
  if Σ_{m=k}^{i} y_m > δ_NUQ then
    Δ_NUQ_j = y_i − y_k
    k = i
  end if
end for
```

In the following, RR_{UQ} and RR_{NUQ} indicate the outputs from the uniform and non-uniform quantizers, respectively.

Algorithm 3 Pseudo-code to obtain the optimal $\mathbf{\Delta}_{NUQ}$

1: Initialize δ_{NUQ}
2: Estimate M^k using the BIC
3: Estimate λ^k
4: Perform the detection
5: Determine D, according to Eq 11
6: Modify the value of δ_{NUQ}
7: Repeat 2-6 until obtain the minimum value of D

2.4 Exploitation of physiological delays in the observed time series

Sometimes, the observation vector has a limited number of observable values that cannot be extended. In this work, the observation vector is composed by only one observable value: the RR interval time series. We propose in this paper to increase the observability of the system, while keeping the same number of observable values, by using physiological knowledge of the observed time series. In the case of the RR time series, which is used to detect the bradycardia, the application of a time delay is of interest since it is known that the RR interval is modulated by the autonomic nervous system, with distinct dynamics for the sympathetic (slower) and parasympathetic (faster) systems.

Here, we proposed to construct a new observation vector with two observable values: (1) the original version of the observed time series at time t and (2) its delayed version at time $t - \tau$, where τ is a predefined time delay to optimize (see Sect. 4), taking into account the physiology and knowing that the RR at a time instant t is also related to their past values [42]. So the observation vector becomes, an observation matrix:

$$\mathbf{O} = \begin{bmatrix} O_{t-T+1:t} \\ O_{t-\tau-T+1:t-\tau} \end{bmatrix} \quad (5)$$

where for clarity, observation matrices will be represented in bold in this paper, such as **RR**.

3 Evaluation procedure of the online apnea–bradycardia detector

The proposed detector was firstly evaluated with simulated signals in order to optimize its structure and evaluate the impact of the different preprocessing methods in a controlled way. It was then evaluated with real signals acquired in NICU, in which AB episodes have been manually annotated by an expert clinician.

3.1 Evaluation on simulated signals

Synthetic time series were generated with the FitzHugh–Nagumo model, described by the following differential equations [21]:

$$\begin{aligned} \frac{dv}{dt} &= 3\left(v - \frac{1}{3}v^3 + r + I\right) \\ \frac{dr}{dt} &= -\frac{1}{3}(v - a + 0.8r) \end{aligned} \quad (6)$$

Variables v and r are in a "rest" value until a perturbation is introduced into variable I. This induces an excursion in the phase space on variables v and r, before returning to their resting values. This model is particularly suited to our problem because its two state variables have heterogeneous dynamics (slow and fast state variables) and these dynamics can be modified by adjusting a parameter.

The simulated series have a size of 400 s and a perturbation $I = -0.2$ is introduced during the interval [300, 305] s. Parameter a was modified according to a uniform distribution in the interval $a1 \sim \mathcal{U}(0.58, 0.62)$ and $a2 \sim \mathcal{U}(0.78, 0.82)$ to generate series presenting two different dynamics. Moreover, the simulated series were divided by its maximum value, in order to generate signals with similar magnitudes. The SNR was set to 5 dB and an additive Gaussian white noise was added to the observed series. Responses generated with $a1$ and $a2$ are thus difficult to differentiate by only analyzing their instantaneous values (such as with a threshold-based detector).

We simulated 80 synthetic signals of 400 s length: 40 of them were generated with $a1$ and 40 others with $a2$. Using these signals, three synthetic datasets were used for the training phase:

- Dataset named LSFN1 comprising 80 segments randomly taken during the "rest" state (before the perturbation) generated with $a1$ (40 segments) and $a2$ (40 segments), with duration $T = 10$ s each segment. This means that from the 80 original synthetic signals (400 s), we have taken only one segment of 10 s per signal.
- Dataset LSFN2 including 40 segments from the beginning of the perturbation and generated with $a1$, with duration $T = 10$ s each segment.
- Dataset LSFN3 enclosing 40 segments from the beginning of the perturbation and generated with $a2$, with duration $T = 10$ s each segment.

- In the case of synthetic signals, $K = 3$ models have been used, to represent and differentiate three distinct dynamics: rest state, disturbance state from signals generated with parameter $a1$, and disturbance state from signals generated with parameter $a2$. The model with set of parameters λ^1 is trained with the signals found in LSFN1, the model with set of parameters λ^2 is trained with the signals found in LSFN2, and the model with set of parameters λ^3 is trained with the signals found in LSFN3.

Once the models have been trained, they are applied to a test dataset consisting of 400 synthetic signals of 400 s each: 200 of these signals were generated with parameter $a1$ and 200 with parameter $a2$. Equations 3 and 4 where applied as the online detection approach, with a sliding window of size $T = 10$ s.

3.2 Evaluation on real signals

RR interval time series were extracted from the ECG of 32 preterm infants with frequent AB episodes as described in [3, 17]. This procedure comprises: (1) beat detection from ECG signals and (2) the construction of the RR time series (successive differences of R-wave time instants) for each individual of the dataset. These time series were uniformly resampled at 10 Hz to have sufficient temporal resolution for the application of the models.

Rest episodes and bradycardia events were manually annotated by an expert from 148 time series. In order to precisely locate the instant of bradycardia onset and avoid the variability related to manual annotations, an annotation correction method was used. A sigmoid function (Eq. 7)

$$f(t) = A + \frac{B}{1 + e^{\frac{t-C}{D}}} \qquad (7)$$

was used to approximate the RR time series around the annotated time instant of the bradycardia onset. The bradycardia onset corresponds to the first point where the derivative of the sigmoid function is >1 such as:

$$t_{\text{onset-bradycardia}} = \arg \frac{\mathrm{d}f(t)}{\mathrm{d}t} > 1. \qquad (8)$$

This procedure is illustrated in Fig. 4a.

In total, 148 RR interval time series (series duration = 26.25 ± 11.37 min) from the database of 32 preterm infants were used and 233 bradycardia episodes were annotated with a mean duration of 21.48 ± 16.07 s. From these time series, 48 RR interval time series were randomly drawn to train the models (training set) and the remaining (100) were taken for test (test set). To avoid overtraining, a cross-validation was performed by repeating 10 times the training/test procedure and by using different partitions of the data in each round of the cross-validation.

From the training set, two datasets were constructed for the training phase:

- Dataset LS1 including the segments enclosing at least one AB event, taken at the beginning of the bradycardia with duration of $T = 7$ s. During each round of the cross-validation procedure, the number of bradycardia in each segment in LS1 is variable because each RR time series can be composed of one or several AB events.
- Dataset LS2 consisting of 300 segments at rest, drawn randomly from the series, with duration of $T = 7$ s.

The length of the segments (7 s) corresponds to the time measured from the beginning of the bradycardia to the peak RR value measured on mean bradycardia episode estimated on several (233) episodes, as depicted in Fig. 4b.

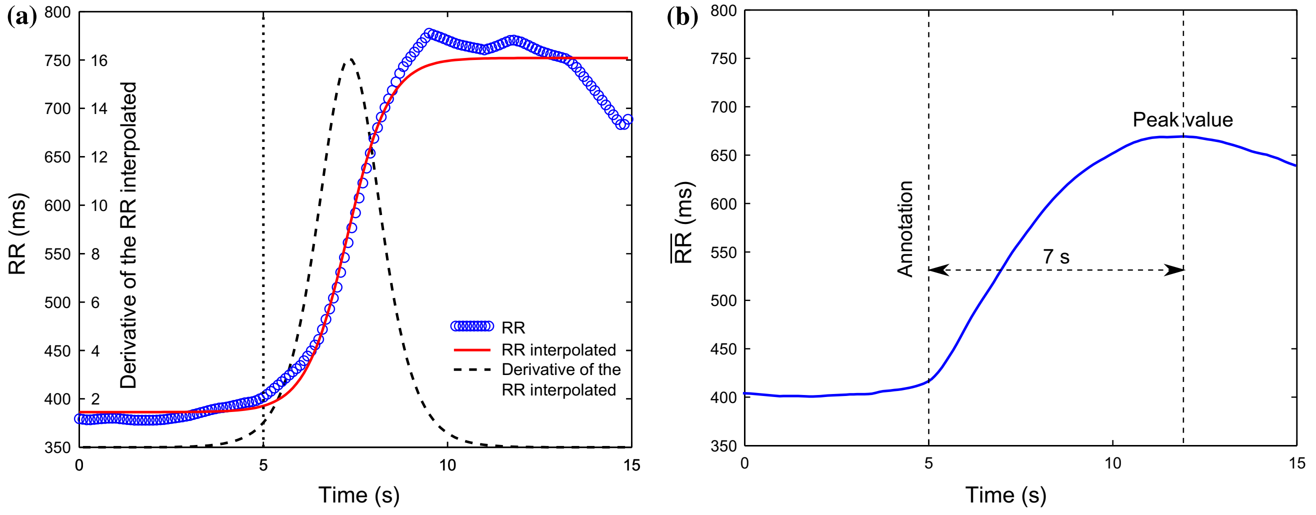

Fig. 4 In (**a**), approximation of the RR time series (represented by *blue circles*) during an episode of bradycardia by a sigmoid function (*continuous red line*). The *dashed black curve* is the derivative of the sigmoid function and the *vertical dotted line* corresponds to the corrected annotation of the onset of the bradycardia episode. In (**b**), average of the 233 blocks of RR time series with AB episodes (color figure online)

In order to reduce the variability of the first sample of the time series, the mean, determined within 5 s before the start of each segment, was removed for all segments and for all time series. This is particularly important for the estimation of the first state (π_i) of the models.

In the case of real signals, $K = 2$ models have been used, in order to represent and differentiate two different dynamics: the rest state and the bradycardia state. The model used to represent the bradycardia state was trained with the signals found in dataset LS1, in which only segments of 7 s length enclosing a bradycardia episode are found, and the model used to represent the rest state was trained with the signals found in dataset LS2, in which only segments of 7 s length without bradycardia episodes are found.

The evaluation of the models was performed on the test set. As explained before, the number of AB episodes in the test set is variable during each round of the cross-validation procedure. The online HSMM or HMM detector is applied to these series as described in the previous section, with a sliding window of size $T = 7$ s.

3.3 Performance evaluation

For both simulated and real signals, true positives (TP), true negatives (TN), false positives (FP) and false negatives (FN) were determined for each sample by comparing the obtained detections with the available annotations. TP occur when a detection falls within a 20 s window, centered at a given annotation. Even if this window seems particularly large, this choice is justified by the fact that the detection delay is also an important feature that will be analyzed for the selection of the optimal detection method.

Detection performance was evaluated classically by comparing receiver operating characteristics (ROC) and then by estimating the sensitivity (Eq. 9) and specificity (Eq. 10) of each detector, for different detection thresholds $\delta^{\alpha,k}$. The sensitivity and specificity were evaluated classically by:

$$\text{Se} = \frac{\text{TP}}{\text{TP} + \text{FN}} \tag{9}$$

$$\text{Sp} = \frac{\text{TN}}{\text{TN} + \text{FP}} \tag{10}$$

Optimal detection performances were evaluated using the shortest distance to perfect detection (D) according to Eq. 11.

$$D(\delta^{\alpha,k}) = \min_{\delta^{\alpha,k}} \sqrt{(1 - \text{Se}(\delta^{\alpha,k}))^2 + (1 - \text{Sp}(\delta^{\alpha,k}))^2} \tag{11}$$

The detection delay (dd) was also determined, and defined as the time elapsed between the annotation instant (beginning of the bradycardia) and the detection time. Only one detection delay is measured for each AB episode, so only the nearest TP is used, the following TP inside the same AB episode are discarded. For a round i of the cross-validation procedure, we compute the mean ($\overline{\text{dd}}^i$) and the standard deviation of the detection delay ($\sigma(\text{dd})^i$) of the vector formed by all the detection delays, according to the following equations:

$$\overline{\text{dd}}^i = \frac{1}{N} \cdot \sum_{n=1}^{N} \text{dd}_n^i \tag{12}$$

$$\sigma(\mathrm{dd})^i = \sqrt{\frac{1}{N-1} \cdot \sum_{n=1}^{N} \left(\mathrm{dd}_n^i - \overline{\mathrm{dd}}^i\right)^2} \quad (13)$$

where, N is the number of AB events detected.

The best $\mathrm{Se}^i, \mathrm{Sp}^i$ and $\overline{\mathrm{dd}}^i \pm \sigma(\mathrm{dd})^i$ at the optimal detection point D were then estimated for each round of the cross-validation procedure. The mean and the standard deviation were computed for the sensitivity, the specificity, the detection delay and the standard deviation of the detection delay.

4 Results

Detection results obtained using synthetic signals are firstly presented. Then, the detection performance obtained on real signals are detailed.

4.1 Simulated signals

This experience is particularly interesting, since, as described earlier, a simple threshold-based detection would be unable to discriminate the series produced with $a1$ from those produced with $a2$ (see Fig. 5a, c). Only the case of the detection of signals generated with $a1$ is analyzed, since the detection of signals generated with $a2$ do not provide any additional information for our purpose of validation. Thus, the proposed online detector acts here as a sequential classifier. To validate our detector based upon dynamics, only results obtained by HSMM based detector are reported. Since the training and test sets are independent, no cross-validation procedure was performed using the simulated signals.

$K = 3$ HSMM were employed to model each training dataset: HSMM with set λ^k to model LSFNk, $k \in \{1, 2, 3\}$ (see Sect. 3.1 for further details). The BIC was applied in each case to estimate the number of states per model. An optimized sliding window of size $T = 10$ s was used to obtain the observation sequence and the log-likelihoods $\mathcal{L}^k$ are computed for each sample. The parameters were set empirically to $\tau = 5$ s, $\Delta_{\mathrm{UQ}} = 25$ and $\delta_{\mathrm{NUQ}} = 0.003$ after several experiences. The values of these parameters (τ, Δ_{UQ}, and δ_{NUQ}) are relevant in the detection performance. They correspond to the optimal values deduced from the ROC curves maximizing both the sensibility and the specificity.

Figure 5a shows an example of v series generated with $a1$ and $a2$. Figure 5c show the respective log-likelihood curves. We observe how the log-likelihood curves change to indicate the state (“rest” or “disturbance”) at time t. Two thresholds ($\delta^{2,1}$ and $\delta^{2,3}$) were used to detect the perturbation generated with $a1$, as proposed in Eq. 4. Detection results for simulated signals are shown in Table 1.

4.2 Real signals

We propose here to retain $K = 2$ HMM and $K = 2$ HSMM. One model will be used to learn the characteristics of the stable state, while the second one is focussed on the early detection of bradycardia events. Each parameter set λ^k was estimated to reproduce the dynamics of dataset LSk, $k \in \{1, 2\}$. Only one threshold is thus used, $\delta^{1,2}$. The number of states M^k was, as previously, estimated using the BIC. A $T = 7$ s sliding window was used to construct $O_{t-T+1:t}$ and to determine $\mathcal{L}_t^k$.

For RR time series, the optimal value of τ was estimated by maximizing the detection performance using the set of values $\tau = \{0.34, 0.45, 0.56, 0.67, 0.78\}$. These values were chosen to be centered around 0.556 s, which corresponds to a half period of the difference between peaks at the low-frequency and high-frequency components (0.9 Hz), retrieved by spectral analysis of the heart rate variability from experimental studies [4, 11].

Figure 5b, d show an example of AB detection, based on the likelihood calculation. Two episodes of bradycardia are reported (Fig. 5b). The curves of log-likelihoods ($\mathcal{L}^1$ and $\mathcal{L}^2$) change over time and, at the time instant of bradycardia, the log-likelihoods $\mathcal{L}^1$ (resp. $\mathcal{L}^2$) increases (resp. decreases) significantly (Fig. 5d). The beginning and end annotations of each AB episode are also shown (vertical dotted lines).

ROC curves (sensibility vs 1-specificity) are shown in Fig. 6a and their respective detection delays in Fig. 6b. Conventional detection methods (fixed and relative thresholds) used in NICU are also reported as a reference. In these figures, the optimal points (maximizing both sensibility and 1-specificity) are shown with character “x”. In synthesis, Table 2 lists the mean and the standard deviation of the sensibility Se, specificity Sp, detection delay $\overline{\mathrm{dd}}$ and standard deviation of the detection delay $\sigma(\mathrm{dd})$ at the optimal detection points of AB episodes, after the cross-validation procedure. These values were obtained using the optimal parameters $\Delta_{\mathrm{QU}} = 1.3$, $\delta_{\mathrm{NUQ}} = 0.01$ and $\tau = 0.67$ s when applied.

5 Discussion

Results on simulated signals clearly show that the detection performance is improved when the quantization step is applied (see Table 1). The best detection performance and the minimum mean detection delay were obtained when a uniform quantization is applied (v_{UQ}), followed by the non-uniform quantization version (v_{NUQ}). The interest of integrating a time-delayed version of the signal was also underlined. In this case, the synchronization of different effects which are not necessarily in phase is of benefit to the performance. These results confirm the interest of the

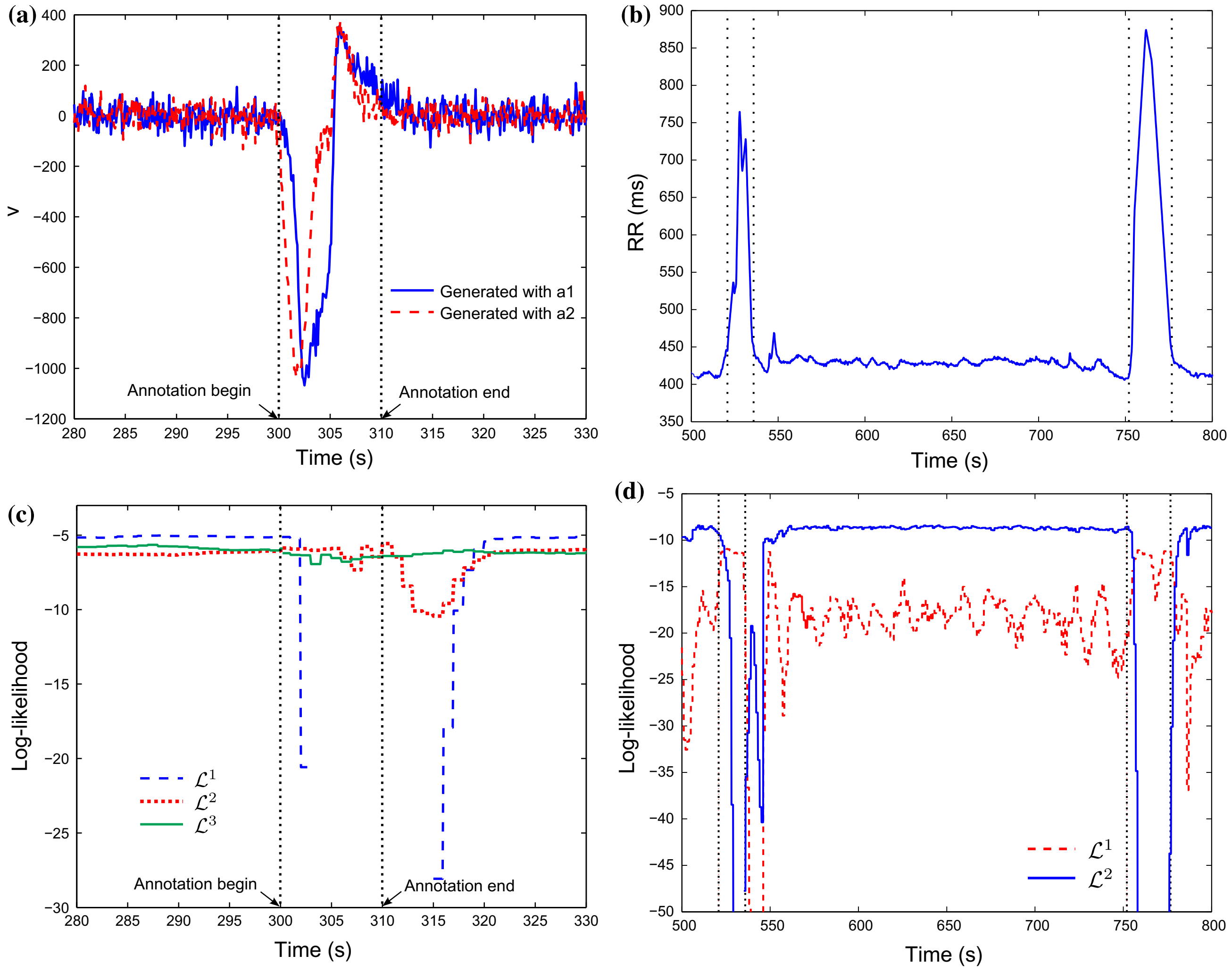

Fig. 5 Representative examples of the processed time series and the corresponding log-likelihood curves, obtained from the application of the proposed HSMM approach: **a** synthetic time series v, generated with parameters $a1$ (*continuous blue line*) and $a2$ (*red dashed line*); **b** RR time series with two episodes of bradycardia; **c** log-likelihood curves obtained from the application of three HSMM models to synthetic signals in **a**, where $\mathcal{L}^1$ (*blue dashed line*) represents the rest state, $\mathcal{L}^2$ (*red dotted line*) represents the disturbance state for signals generated with parameter $a1$ and $\mathcal{L}^3$ (*continuous green line*) represents the disturbance state for signals generated with parameter $a2$; **d** log-likelihood curves obtained when applying the proposed HSMM approach to real signals in (**b**), where $\mathcal{L}^1$ (*red dashed line*) represents the bradycardia state and $\mathcal{L}^2$ (*continuous blue line*) represents the rest state. *Vertical dotted lines* correspond to the annotations of the beginning and end of the episodes of interest: perturbation at the left and AB at the right. It is worth to notice that the AB duration is more than 20 s (color figure online)

Table 1 Sensitivity, specificity and detection delay by evaluating D (Eq. 11) for simulated signals

Variable	Se (%)	Sp (%)	dd (s)
v	89.29	97.98	2.09 ± 0.07
v_{UQ}	93.26	97.85	1.41 ± 0.24
v_{NUQ}	92.14	96.19	0.16 ± 3.12
$\mathbf{v}$	90.25	97.61	1.73 ± 1.40

proposed online HSMM detector combining the quantization step and the integration of a delayed version of the observation.

Detection results from real signals (Fig. 6; Table 2) show that modeling the dynamics of features extracted from the ECG by HSMM or HMM provides an improved detection performance, when compared to conventional detection methods used in NICU (fixed or relative threshold detections). In addition, the application of the proposed preprocessing methods further improves this performance and reduces the mean detection delay.

Detection results were similar when using the approaches based on HMM or HSMM. In terms of sensitivity and specificity, on average, the best detection performance is achieved using the HSMM based detector when

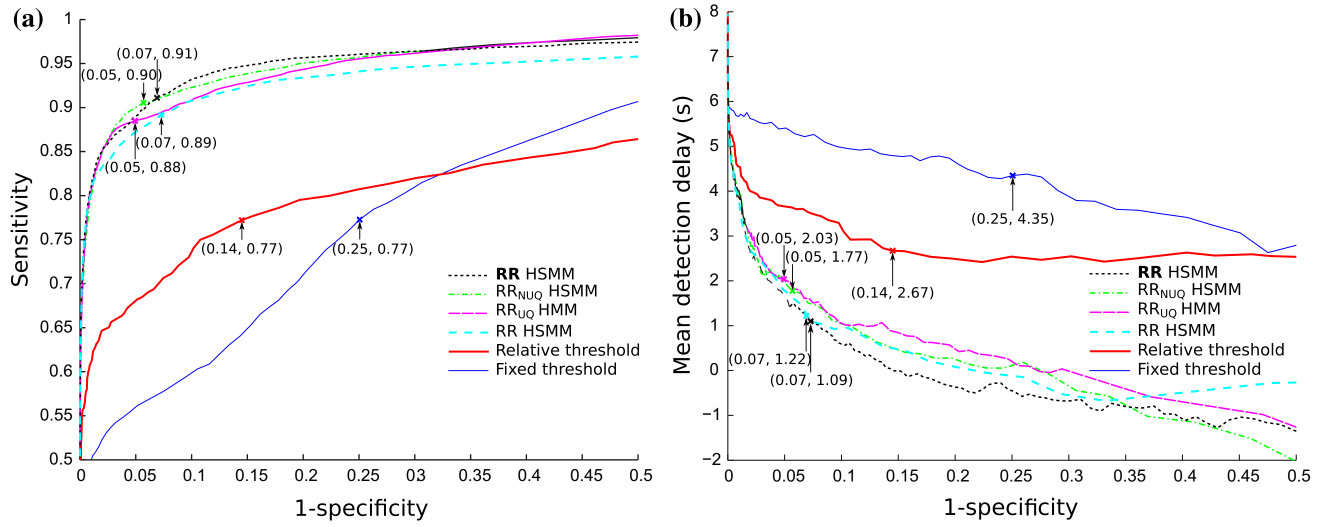

Fig. 6 In (**a**), ROC curves for AB event detection and in (**b**), mean detection delay of AB episodes (color figure online)

Table 2 AB detection, in terms of sensitivity, specificity and detection delay by evaluating D (Eq. 11)

Variable	Method	Se (%)	Sp (%)	$\overline{\text{dd}}$ (s)	σ (dd) (s)
RR	HSMM	86.88 ± 2.10	92.23 ± 1.15	1.61 ± 0.36	4.04 ± 0.22
RR_{UQ}	HSMM	88.30 ± 0.98	92.81 ± 1.34	1.71 ± 0.24	3.71 ± 0.36
RR_{NUQ}	HSMM	**89.66** ± 0.71	93.15 ± 1.32	1.84 ± 0.34	3.46 ± 0.51
**RR**	HSMM	88.56 ± 1.72	92.87 ± 0.86	**1.59** ± 0.24	3.61 ± 0.30
RR	HMM	89.08 ± 1.19	91.61 ± 0.83	1.59 ± 0.33	3.64 ± 0.29
RR_{UQ}	HMM	88.28 ± 1.62	**93.84** ± 0.79	2.09 ± 0.28	3.53 ± 0.38
RR_{NUQ}	HMM	89.66 ± 0.85	93.10 ± 1.16	1.89 ± 0.25	3.69 ± 0.47
**RR**	HMM	86.52 ± 3.96	92.27 ± 1.77	1.61 ± 0.43	3.74 ± 0.32

Bold numbers correspond to the best value of Se, Sp and $\overline{\text{dd}}$

the RR time series are quantized non-uniformly, and it is slightly higher than that obtained by the equivalent HMM when the RR time series are quantized non-uniformly. In terms of detection delay, on average, the HSMM based detector with time-delayed versions of the RR series provides the best results. Results obtained indicate that our approach detects, on average, more than 1.5 s earlier than conventional detectors.

Another parameter that can be optimized in the proposed approach is the number of models K. In this work, the number of models has been fixed to the number of different classes that were expected to be differentiated. As in other classification approaches, increasing the number of models may lead to a better performance. For instance, in the case of real signals, $K = 5$ models could have been defined, to represent: λ^1 the rest state, λ^2 the tachycardia state, λ^3 the reduction of the heart rate (but not a significant bradycardia) state, λ^4 the short-duration bradycardia state and λ^5 the long-duration bradycardia state. However, increasing the value of K leads to a higher complexity for constituting the training dataset and increased computational costs both for training and test.

6 Conclusions and future work

In this paper, the characterization of time series and the difficult problem of exploiting the dynamics of biomedical time series, for the early detection of cardiorespiratory events, were addressed. A sequential detector, based on HSMM or HMM and combining different preprocessing methods (uniform and non-uniform quantization steps and delayed observation vector), was proposed and evaluated using simulated and real signals. Synthetic time series were generated with the FitzHugh–Nagumo model and real signals were obtained from RR interval time series, extracted from the ECG of 32 preterm infants monitored in NICU, with frequent apnea–bradycardia episodes.

The feasibility of the proposed approach was evaluated on synthetic signals and we have shown that the proposed online method is able to take into account the dynamics of the observed signals in order to generate the detection decision. The performance of the proposed approach for AB episode detection was evaluated using real data from NICU, and it has shown to be higher than classical methods, while minimizing the detection delay. The usefulness of the quantization phase and of the integration of delayed versions of the observation into the models were also evaluated and showed to improve the detection performance. This is an important result, since the minimization of the delay from the beginning of the AB episode to the therapy applied by the nurse is one of the major issues in this clinical field. Another main advantage of the proposed approach is its simplicity, opening the possibility of an embedded implementation into a monitoring device.

Future works are directed toward the inclusion of others features extracted from the ECG, such as the R-wave amplitude, QRS complex duration, PR interval, P-wave morphology and QT interval, but also data from other sensors, such as respiration. This will probably require the adaptation of the model architecture in order to reproduce the dynamics of each one of these variables independently, while keeping a multivariate representation by coupling the states of each model [14, 34].

Acknowledgments This work was partly funded by the ECOS NORD project V09S04.

Appendix 1: The viterbi algorithm

The Viterbi algorithm has first appeared in the literature of coding in 1967 [43]. This algorithm selects the states individually most likely, in order to find the states sequence most likely associated with the observed sequence. The algorithm includes a step of recursion which travels the signal and records the states that maximize $\mathcal{L}(O_{1:T}|\lambda)$, and a step of back propagation that travels the signal in the opposite direction, starting from the state for which the likelihood at time T is maximum, and taking into account the previous states stored in the recursion step.

To find the best state sequence $S_{1:T} = S_1, \ldots, S_T$ for the sequence of observation $O_T \triangleq O_1, \ldots, O_T$,

$$\delta_t(i) \triangleq \max_{s_{1:t-1}} \big(P(s_1, s_2, \ldots, s_t = i, o_{1:t}|\lambda)\big) \tag{14}$$

represents the maximum likelihood of a single path until time t, which takes into account the first t observations and stops in state i. By induction we have

$$\delta_{t+1}(j) = \max_{i\in\mathcal{S}} \big(\delta_t(i)a_{ij}\big) b_j(o_{t+1}). \tag{15}$$

The argument that maximizes Eq. 15 for each t and j, is recorded through the array $\Psi_t(j)$. The full procedure to find the best state sequence is:

- Initialization:

$$\begin{aligned} &\delta_1(i) = \pi(i)b_i(o_1), \quad i \in \mathcal{S} \\ &\Psi_1(i) = 0. \end{aligned} \tag{16}$$

- Recursion:

$$\begin{aligned} &\delta_t(j) = \max_{i\in\mathcal{S}} \big(\delta_{t-1}(i)a_{ij}\big) b_j(o_t), \quad 2 \le t \le T, j \in \mathcal{S} \\ &\Psi_t(j) = \arg\max_{i\in\mathcal{S}} \big(\delta_{t-1}(i)a_{ij}\big), \quad 2 \le t \le T, j \in \mathcal{S} \end{aligned} \tag{17}$$

- Termination:

$$\begin{aligned} &P^* = \max_{i\in\mathcal{S}} \big(\delta_T(i)\big) \\ &i_T^* = \arg\max_{i\in\mathcal{S}} \big(\delta_T(i)\big). \end{aligned} \tag{18}$$

- backpropagation (path obtained):

$$i_t^* = \Psi_{t+1}(i_{t+1}^*), \quad t = T-1, T-2, \ldots,, 1. \tag{19}$$

Appendix 2: The viterbi algorithm extended to HSMM

The quantity

$$\delta_t(i,d) \triangleq \max_{s_{1:t-d}} \big(P(s_{1:t-d}, S_{t-d+1:t} = i, o_{1:t}|\lambda)\big) \tag{20}$$

represents the maximum likelihood of a single path until time t, which takes into account the first t observations and stops at the state i of length d. By induction we have:

$$\delta_{t+d}(j,d) = \max_{i\in\mathcal{S}\backslash\{j\}, d'\in\mathcal{D}} \big(\delta_t(i,d')a_{ij}\big) p_j(d) b_j(o_{t+1:t+d}). \tag{21}$$

The array $\Psi_t(j,d)$ is used to record the states sequence and times that maximize 21. The complete procedure is as follows:

- Initialization:

$$\begin{aligned} &\delta_1(i) = \pi(i)b_i(o_1), \quad i \in \mathcal{S} \\ &\Psi_1(i) = 0. \end{aligned} \tag{22}$$

- Recursion:

$$\begin{aligned} \delta_t(j,d) = &\max_{i\in\mathcal{S}\backslash\{j\}, d'\in\mathcal{D}} \big(\delta_{t-d}(i,d')a_{ij}\big) p_j(d) b_j(o_{t-d+1:t}), \\ &2 \le t \le T, j \in \mathcal{S}, d \in \mathcal{D} \\ \Psi_t(j,d) = &\arg\max_{i\in\mathcal{S}\backslash\{j\}, d'\in\mathcal{D}} \big(\delta_{t-d}(i,d')a_{ij}\big), \\ &2 \le t \le T, j \in \mathcal{S}, d \in \mathcal{D}. \end{aligned} \tag{23}$$

- Termination:

$$P^* = \max_{i \in \mathcal{S}, d \in \mathcal{D}} \big(\delta_T(i,d)\big)$$
$$(i_T^*, d_T^*) = \arg\max_{i \in \mathcal{S}, d \in \mathcal{D}} \big(\delta_T(i,d)\big). \quad (24)$$

- backpropagation (path obtained):

$$(i_t^*, d_t^*) = \Psi_{t+1}(i_{t+1}^*, d_{t+1}^*), \quad t = T - d_t^*, \ldots, 1. \quad (25)$$

References

1. Ahlborn V, Bohnhorst B, Peter C, Poets C (2000) False alarms in very low birthweight infants: comparison between three intensive care monitoring systems. Acta Paediatr 89(5):571–576
2. Altuve M, Carrault G, Beuchee A, Flamand C, Pladys P, Hernandez AI (2012) Comparing hidden markov model and hidden semi-Markov model based detectors of apnea–bradycardia episodes in preterm infants. In: Computing in cardiology (CinC), Krakow, pp 389–392.
3. Altuve M, Carrault G, Cruz J, Beuchee A, Pladys P, Hernandez A (2011) Multivariate ecg analysis for apnoea-bradycardia detection and characterisation in preterm infants. Int J Biomed Eng Technol 5(2):247–265
4. Beuchée A (2005) Intérêt de l'analyse de la variabilité du rythme cardiaque en néonatalogie. Comportement des systèmes de régulation cardiovasculaire dans le syndrome apnée/bradycardie du nouveau-né. Ph.D. thesis, Université de Rennes 1
5. Bishop C (1995) Neural networks for pattern recognition. Oxford university press, Oxford
6. Bitan Y, Meyer J, Shinar D, Zmora E (2000) Staff actions and alarms in a neonatal intensive care unit. In: Proceedings of the human factors and ergonomics society annual meeting, vol 44. SAGE Publications, pp 17–20
7. Bitan Y, Meyer J, Shinar D, Zmora E (2004) Nurses' reactions to alarms in a neonatal intensive care unit. Cognit Technol Work 6(4):239–246
8. Bohnhorst B, Peter CS, Poets CF (2000) Pulse oximeters' reliability in detecting hypoxemia and bradycardia: comparison between a conventional and two new generation oximeters. Crit Care Med 28(5):1565–1568
9. Brockmann PE, Wiechers C, Pantalitschka T, Diebold J, Vagedes J, Poets CF (2013) Under-recognition of alarms in a neonatal intensive care unit. Arch Dis Child Ed 98(6):F524–F527. doi:10.1136/archdischild-2012-303369
10. Chambrin MC et al (2001) Alarms in the intensive care unit: how can the number of false alarms be reduced? Crit Care 5(4):184–188
11. Chatow U, Davidson S, Reichman B, Akselrod S (1995) Development and maturation of the autonomic nervous system in premature and full-term infants using spectral analysis of heart rate fluctuations. Pediatr Res 37(3):294
12. Cruz J, Hernández A, Wong S, Carrault G, Beuchee A (2006) Algorithm fusion for the early detection of apnea–bradycardia in preterm infants. In: Computers in Cardiology, Valencia, pp 473–476.
13. Cvach M (2012) Monitor alarm fatigue: an integrative review. Biomed Instrum Technol 46(4):268–277
14. Derrode S, Pieczynski W (2004) Signal and image segmentation using pairwise Markov chains. In: IEEE Trans Signal Process 52(9):2477–2489
15. Dumont J (2008) Fouille de dynamiques multivariées, application à des données temporelles en cardiologie. Ph.D. thesis, Université de Rennes 1
16. Dumont J, Carrault G, Gomis P, Wagner G, Hernández A (2009) Detection of myocardial ischemia with hidden semi-Markovian models. Comput Cardiol 2009:121–124
17. Dumont J, Hernández AI, Carrault G (2010) Improving ecg beats delineation with an evolutionary optimization process. IEEE Trans Biomed Eng 57(3):607–15
18. Dumont J, Hernandez AI, Fleureau J, Carrault G (2008) Modelling temporal evolution of cardiac electrophysiological features using hidden semi-Markov models. In: Engineering in medicine and biology society, 2008. EMBS 2008. 30th annual international conference of the IEEE. IEEE, pp 165–168
19. Fahrmeir L (1992) Posterior mode estimation by extended Kalman filtering for multivariate dynamic generalized linear models. J Am Stat Assoc 87(418):501–509
20. Ferguson J (1980) Variable duration models for speech. In: Proceedings of the symposium on the application of hidden Markov models to text and speech, vol 1, pp 143–179
21. Fitzhugh R (1961) Impulses and physiological states in theoretical models of nerve membrane. Biophys J 1(6):445–466
22. Forney G Jr (1973) The viterbi algorithm. Proc IEEE 61(3):268–278
23. Freudenthal A, Van Stuijvenberg M, Van Goudoever JB (2013) A quiet NICU for improved infants' health, development and well-being: a systems approach to reducing noise and auditory alarms. Cogn Technol Work 15(3):329–345
24. Garcia SP, Almeida JS (2006) Multivariate phase space reconstruction by nearest neighbor embedding with different time delays. Phys Rev E 72(2):027205. doi:10.1103/PhysRevE.72.027205
25. Houtekamer P, Mitchell H (2001) A sequential ensemble Kalman filter for atmospheric data assimilation. Mon Weather Rev 129(2):123–137
26. Imhoff M, Fried R (2009) The crying wolf: still crying? Anesth Analg 108(5):1382–1383
27. Janvier A, Khairy M, Kokkotis A, Cormier C, Messmer D, Barrington K (2004) Apnea is associated with neurodevelopmental impairment in very low birth weight infants. J Perinatol 24(12):763–768
28. Kalman R et al (1960) A new approach to linear filtering and prediction problems. J Basic Eng 82(1):35–45
29. Kong L, Yang C, Wang Y, Gui W (2009) Generic phase space reconstruction method of multivariate time series. In: Control and decision conference, 2009. CCDC'09. Chinese, pp 3752–3755
30. Lawless ST (1994) Crying wolf: false alarms in a pediatric intensive care unit. Crit Care Med 22(6):981–985
31. Lee HJ, Choi JH, Min SJ, Kim DH, Kim HS (2010) Comparison of the clinical performance between two pulse oximeters in nicu: Nellcor n-595 versus masimo set. J Korean Soc Neonatol 17(2):245–249
32. Murphy K (2002) Dynamic bayesian networks: representation, inference and learning. Ph.D. thesis, Citeseer
33. Pichardo R, Adam JS, Rosow E, Bronzino J, Eisenfeld L (2003) Vibrotactile stimulation system to treat apnea of prematurity. Biomed Instrum Technol 37(1):34–40
34. Pieczynski W (2003) Pairwise markov chains. IEEE Trans Pattern Anal Mach Intell 25(5):634–639. doi:10.1109/TPAMI.2003.1195998
35. Pillekamp F, Hermann C, Keller T, von Gontard A, Kribs A, Roth B (2007) Factors influencing apnea and bradycardia of prematurity-implications for neurodevelopment. Neonatology 91(3):155
36. Poets C, Stebbens V, Samuels M, Southall D (1993) The relationship between bradycardia, apnea, and hypoxemia in preterm infants. Pediatr Res 34(2):144
37. Portet F, Gao F, Hunter J, Sripada S (2007) Evaluation of on-line bradycardia boundary detectors from neonatal clinical data. In: Engineering in medicine and biology society, 2007. EMBS 2007. 29th annual international conference of the IEEE, pp 3288–3291

38. Rabiner LR (1989) A tutorial on hidden Markov models and selected applications in speech recognition. Proc IEEE 77(2):257–286
39. Raman H, Sunilkumar N (1995) Multivariate modelling of water resources time series using artificial neural networks. Hydrol Sci J 40(2):145–164
40. Schwarz G (1978) Estimating the dimension of a model. Ann Stat 6(2):461–464
41. Suzuki T, Ikeguchi T, Suzuki M (2003) Multivariable nonlinear analysis of foreign exchange rates. Phys A Stat Mech Appl 323:591–600
42. Task Force (1996) Heart rate variability: standards of measurement, physiological interpretation and clinical use. Task force of the European society of cardiology and the north American society of pacing and electrophysiology. Circulation 93(5):1043–65
43. Viterbi A (1967) Error bounds for convolutional codes and an asymptotically optimum decoding algorithm. IEEE Trans Inf Theory 13(2):260–269
44. Yu SZ (2010) Hidden semi-Markov models. Artif Intell 174(2):215–243
45. Zweig G, Russell S (1998) Speech recognition with dynamic Bayesian networks. In: Proceedings of the national conference on artificial intelligence, pp 173–180
