# Supplementary material for: Exploring Computational Techniques in Preprocessing Neonatal Physiological Signals for Detecting Adverse Outcomes: Scoping Review
Source: Interact J Med Res. 2024 Aug 20;13:e46946. doi: 10.2196/46946 (PMC11372324; doi:10.2196/46946)
Supplement: Multimedia Appendix 3 [file ijmr_v13i1e46946_app3.zip › Included Papers - Final/3461/Stanculescu et al. - 2014 - Autoregressive Hidden Markov Models for the Early .pdf]

Autoregressive Hidden Markov Models for the Early Detection of Neonatal Sepsis

Ioan Stanculescu, Christopher K. I. Williams, and Yvonne Freer

***Abstract*—Late onset neonatal sepsis is one of the major clinical concerns when premature babies receive intensive care. Current practice relies on slow laboratory testing of blood cultures for diagnosis. A valuable research question is whether sepsis can be reliably detected before the blood sample is taken. This paper investigates the extent to which physiological events observed in the patient's monitoring traces could be used for the early detection of neonatal sepsis. We model the distribution of these events with an autoregressive hidden Markov model (AR-HMM). Both learning and inference carefully use domain knowledge to extract the baby's true physiology from the monitoring data. Our model can produce real-time predictions about the onset of the infection and also handles missing data. We evaluate the effectiveness of the AR-HMM for sepsis detection on a dataset collected from the Neonatal Intensive Care Unit at the Royal Infirmary of Edinburgh.**

***Index Terms*—Autoregressive hidden Markov model (AR-HMM), intensive care, neonatal sepsis, real-time inference.**

I. Introduction

LATE onset neonatal sepsis is a bloodstream infection, usually bacterial, generally occurring after the third day of life. Its onset is a major cause of mortality, lifelong neurodisability, and increased health care costs [1]. Estimates show that 10% of all neonates and 25% of very low birth weight babies (VLBW, $<$1500 g birth weight) are affected [2], [3]. This number rises to 50% for extremely preterm infants [1].

The major challenge in successfully treating septic babies is making the diagnosis of infection in the first place. Early signs are subtle and yet it is at this stage that treatment will be effective. A deterioration of the baby's condition over the course of a few hours is a strong symptom for neonatal sepsis, and prompts clinicians to take a blood sample for laboratory testing. However, laboratory culture results can take up to a day before becoming available. Because of the dangers of delaying treatment, antibiotic therapy is usually started at the same time as taking the blood sample. However, applying low thresholds in suspecting sepsis results in a high number of patients being treated unnecessarily for each true case [4]. Thus, if achievable, a reliable early detection of sepsis based on monitoring data would be of great value.

In modern Neonatal Intensive Care Units (NICUs), the patient's vital signs are continuously monitored and often recorded. In this paper, we exclusively rely on the information contained in these traces for building a sepsis detection system. Clinical events informative of the baby's health condition, such as bradycardias or oxygen desaturations, can be associated with patterns in the monitoring data (see Fig. 1 and Table I). However, accurately detecting these events is a nontrivial task and the problem of high false alarm rates is well known [5]. A solution proposed by Quinn *et al.* [6] is the factorial switching linear dynamical system (FSLDS), which is closely related to other work generalizing state-space models [7], [8]. The FSLDS is shown to produce accurate real-time inferences about clinical events affecting NICU data.

In this paper, we propose a probabilistic approach for monitoring the evolution of baby-generated clinical events. An increased incidence of such events is a symptom of sepsis [1]. Starting from this hypothesis, we study the amount of predictive information about neonatal sepsis that can be extracted from the distribution of clinical events. First, using domain knowledge, we define and annotate a set of clinical events. Our main contribution is the formulation of sepsis detection as inference and learning in an autoregressive hidden Markov model (AR-HMM). In addition, we show how exact inference can be obtained in the presence of missing data. The effectiveness of the method is tested both on prediction of sepsis/normality on a second-by-second basis, and in terms of detected sepsis episodes. We also study the relevance of individual clinical event streams.

Griffin, Moorman *et al.* [9], [10] have previously proposed using heart rate data to discriminate sepsis (positive culture) and sepsis-like (negative culture) babies pooled together, from a control group (no culture). They observed a positive skew in the interbeat (RR) interval histograms in the hours before the clinical suspicion of sepsis, and an absence of skew during normal periods. This finding was quantified by a set of summary statistics referred to as the heart rate characteristics (HRC). The HRC are then fed to a logistic regression classifier. A larger dataset was employed for demonstrating that HRC add predictive information to a classifier using only demographic features to discriminate sepsis and sepsis-like illness patients from controls [4]. More precisely, they showed an increase in area under ROC curve (AUC) from 0.72 to 0.77 on a test set. In recent work [11], they conducted a clinical trial which showed that HRC monitoring can decrease mortality. However, this

Manuscript received July 7, 2013; revised October 23, 2013; accepted November 30, 2013. Date of publication December 11, 2013; date of current version September 2, 2014. The work of Ioan Stanculescu was supported by the Scottish Informatics and Computer Science Alliance and the Engineering and Physical Sciences Research Council.

I. Stanculescu and C. K. I. Williams are with the School of Informatics, University of Edinburgh, Edinburgh, EH8 9AB, U.K. (e-mail: i.a.stanculescu@sms.ed.ac.uk; c.k.i.williams@ed.ac.uk).

Y. Freer is with the Neonatal Intensive Care Unit, Simpson Centre for Reproductive Health, The Royal Infirmary of Edinburgh, Edinburgh, EH16 4SA, U.K. (e-mail: Yvonne.Freer@nhslothian.scot.nhs.uk).

Color versions of one or more of the figures in this paper are available online at http://ieeexplore.ieee.org.

Digital Object Identifier 10.1109/JBHI.2013.2294692

2168-2194 © 2013 IEEE. Personal use is permitted, but republication/redistribution requires IEEE permission.
See http://www.ieee.org/publications_standards/publications/rights/index.html for more information.

Authorized licensed use limited to: CSIRO Information Technology Services. Downloaded on September 09,2022 at 00:24:00 UTC from IEEE Xplore. Restrictions apply.

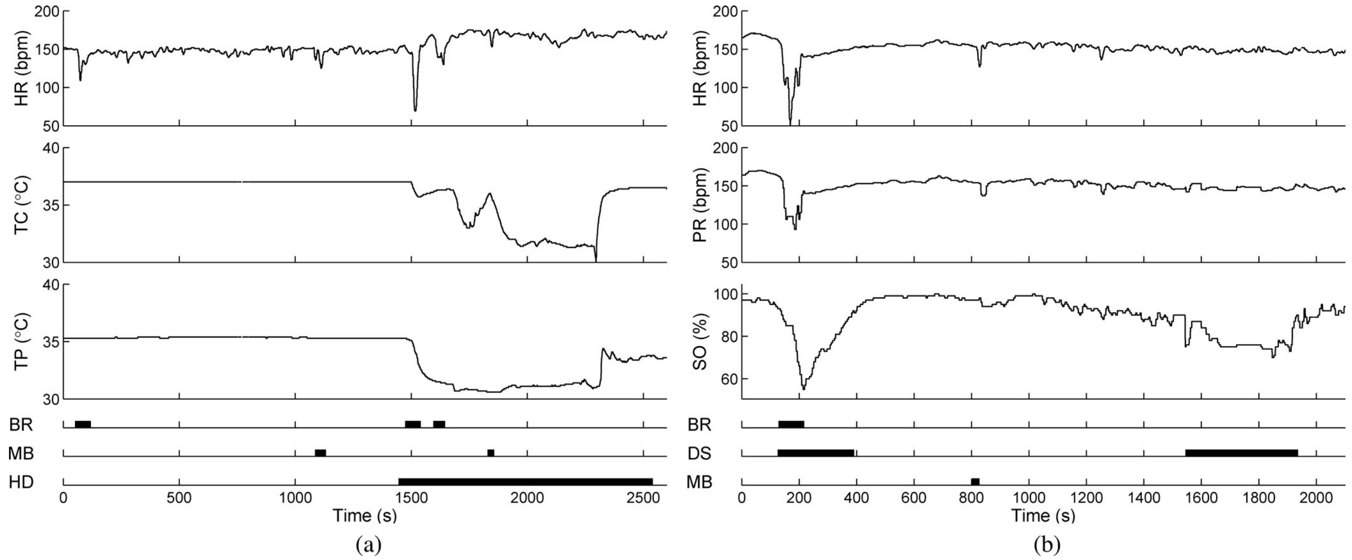

Fig. 1. Examples of clinical events affecting neonatal monitoring data. In panel (a), several instances of bradycardia ("BR") and mini-bradycardia ("MB") can be seen on the ECG heart rate trace ("HR"). Around time $t = 1450$, a sudden fall in both the core and peripheral temperatures ("TC" and "TP") signals the start of a handling ("HD") event. Note that physiological events occurring during handling episodes are not used for sepsis detection. Panel (b) shows several instances of physiological events. Since the pulse oximeter heart rate trace ("PR") agrees with the HR trace, the two instances of desaturation ("DS") annotated on the oxygen saturation channel ("SO") are genuine events.

TABLE I
EXHAUSTIVE LIST OF CLINICAL EVENTS MONITORED FOR DETECTING NEONATAL SEPSIS

Event	Type	Brief Description
Probe dropout	artifactual	lack of monitoring data due to temporary removal or malfunctioning of the monitoring devices
Handling	physiological	some clinical procedure is performed (e.g changing nappies); the incubator's door are thus open; the presentation is a decay in 'TC' and/or 'TP' together with increased variability or dropouts on the other physiological channels
Bradycardia	physiological	sharp fall in 'HR' ('PR') of at least 30 beats per minute (bpm) from a reference level followed by a sharp recovery
Oximeter error	artifactual	disagreement between the oximeter ('PR') and EEG ('HR') heart rates; the disagreement is associated with temporary malfunctioning of the oximeter; this translates into the unreliability of the 'SO' trace
Desaturation	physiological	sudden fall in 'SO' followed by recovery; desaturations are commonly associated with 'SO' falling below 85%
X-factor	any	non-normal pattern occurring on at least one physiological channel that cannot be explained by ANY of the events above

approach does not fully exploit the sequential nature of the monitoring data, nor does it explore the use of other physiological channels for sepsis detection.

A novel case definition for neonatal bloodstream infection is introduced in Modi *et al.* [1]. This study is motivated by statistics saying that around two-thirds of the positive cultures are skin commensals or mixed growth. First, they identify ten binary clinical signs predictive of a positive blood culture. Then, the number of present clinical signs is used to predict a positive blood culture. Based on the classification results, a new case definition is proposed: a baby is infected if either a recognized pathogen is found or if the test yields mixed growth or skin commensal and ≥ 3 clinical signs are present. Note that this study still relies on laboratory results and thus is not being directed toward an earlier detection of the infection.

The Artemis system [12], a stream computing project for neonatal intensive care sets the detection of sepsis as one of its primary objectives. Their method introduces patient agents able to perform multidimensional temporal abstraction on monitoring data [13]. In [14], they propose the use of both heart rate and respiratory rate variabilities for real-time sepsis detection. The latter is intended to help discriminate sepsis from confounding factors such as surgery or narcotics. A performance evaluation of this approach is yet to be published.

The time-series model we use for the early detection of neonatal sepsis is described in Section II. Inference in the presence of missing data is discussed and we also explain how durations can be explicitly modeled. Section III begins by discussing the diagnosis of neonatal sepsis. We then describe the data we have collected and the set of clinical events that affects it. We follow by showing how the model introduced in the previous section is trained. The results are presented and analyzed in Section IV. We conclude and highlight the directions of future research in Section V.

II. Hidden State Models

The family of hidden Markov models (HMMs) is a flexible tool for generative probabilistic modeling of sequential data. It is often employed solving for sequential classification tasks. Its applicability has been long proven in areas such as speech recognition [15], natural language processing [16], biological sequence analysis [17], or electrocardiography [18], [19]. For

Authorized licensed use limited to: CSIRO Information Technology Services. Downloaded on September 09,2022 at 00:24:00 UTC from IEEE Xplore. Restrictions apply.

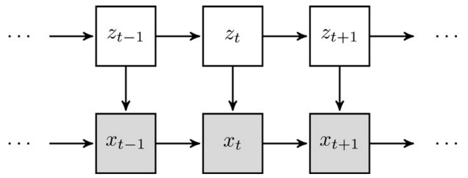

Fig. 2. DAG of the AR-HMM for sepsis detection. Squares indicate discrete variables and shaded nodes are observed.

sepsis modeling, we restrict the discussion to a particular type of HMM: the autoregressive HMM.

A. Autoregressive HMM

An AR-HMM enhances the HMM architecture by introducing a direct stochastic dependence between observations [20], [21]. It is designed to explicitly model the (possibly long range) correlations in sequential data. We first give a brief description of the model and then explain how it can be applied for neonatal sepsis detection.

Like the HMM, the AR-HMM models two types of variables: hidden discrete states z_t and observations x_t. In an HMM, the current observation is independent of all the other observations given the current state. Consequently, there is no explicit constraint on HMM samples to be smooth. The AR-HMM encourages correlation among observations by adding direct dependencies between them. Samples drawn from an AR-HMM are thus smoother than samples from an HMM, usually making the former a better generative model in time-series problems.

The hidden states of an AR-HMM can take one of J values and are organized as a first-order Markov chain with parameters $\theta_{j|i} = p(z_t = j|z_{t-1} = i)$ and $\pi_j = p(z_1 = j)$. Observations in the general AR-HMM can be continuous, but for our purposes, we restrict the discussion to the discrete case. Furthermore, we introduce direct dependencies only between consecutive observations. The corresponding directed acyclic graph (DAG) is shown in Fig. 2. Conditioned on the state z_t, the emission process is again a first-order Markov chain parameterized by $\phi_{l|kj} = p(x_t = l|x_{t-1} = k, z_t = j)$ and $\pi_{l|j} = p(x_1 = l|z_1 = j)$. The joint probability distribution for a sequence of length T is

$$p(z_{1:T}, x_{1:T}) = \pi_{z_1} \pi_{x_1|z_1} \prod_{t=2}^{T} \theta_{z_t|z_{t-1}} \phi_{x_t|x_{t-1} z_t}, \qquad (1)$$

where we have employed the notational convention $a_{t_0:t_1} \triangleq a_{t_0}, a_{t_0+1}, \ldots, a_{t_1}$.

For neonatal condition monitoring, the hidden state variables are modeling the state of the infection. At each time step t, we are observing a set of S clinical events $x_t^{(1)}, x_t^{(2)}, \ldots, x_t^{(S)}$. For each of them, $x_t^{(s)}$ denotes which of its possible $L^{(s)}$ settings clinical event s takes on at time t. Thus, AR-HMM observations are given by the cross product

$$x_t = x_t^{(1)} \otimes x_t^{(2)} \otimes \cdots \otimes x_t^{(S)}$$

and can take one of $L = \prod_{s=1}^{S} L^{(s)}$ settings. The events are assumed to be conditionally independent given the state

$$p(x_t \mid x_{t-1}, z_t) = \prod_{s=1}^{S} p\Big(x_t^{(s)} \Big| x_{t-1}^{(s)}, z_t\Big).$$

Each of the events is modeled as a Markov chain with parameters $\{\phi_{l|kj}^{(s)}, \pi_{l|j}^{(s)}\}$. Importantly, clinical event streams tend to have long runs in the same setting. This motivates our preference for an AR-HMM over a standard HMM, where observations are correlated only through the hidden variables. Notice that, in general, clinical events are not marginally independent.

B. Inference

For real-time prediction, we are interested in inferring the presence of neonatal sepsis from the patient's historical data up to a query time. Technically, this corresponds to computing the filtering distribution $p(z_t|x_{1:t})$. It is also useful to study if observing future data improves the filtering prediction. This means computing the smoothing distribution $p(z_t|x_{1:T})$. The latter is also useful for unsupervised parameter estimation. We first show how the forward–backward algorithm [15] is applied for AR-HMM inference. Then, we explain how we extend it to address the problem of missing data.

The forward–backward algorithm is a message passing routine which exploits conditional independence relationships for doing exact inference in HMMs. In the AR-HMM, the past observations are independent of the future observations given both the current state and the current observation: $x_{t_0} \perp\!\!\!\perp x_{t_1}|z_t, x_t, \quad \forall t_0, t_1 \quad t_0 < t < t_1$. Using this, we can write

$$\begin{aligned} p(z_t, x_{1:T}) &= p(z_t, x_{1:t}) p(x_{t+1:T}|z_t, x_{1:t}) \\ &= p(z_t, x_{1:t}) p(x_{t+1:T}|z_t, x_t) \\ &= \alpha(z_t)\beta(z_t), \end{aligned} \qquad (2)$$

where we have defined the forward message $\alpha(z_t) \triangleq p(z_t, x_{1:t})$ and the backward message $\beta(z_t) \triangleq p(x_{t+1:T}|z_t, x_t)$. The messages can be computed recursively in a forward pass for α and in a backward pass for β [20], [21]. When the likelihoods are precomputed, the total computational cost is $O(TJ^2)$.

If is often the case that we do not have access to observations at all-time steps. We make a missing at random (MAR) assumption [22], which means there is no need to explicitly model the missing data mechanism. For sepsis modeling, missing data issues mainly occur when the patient is being handled by clinical staff. This will be detailed in Section III-C.

One advantage of generative probabilistic models is that they can handle missing data in a principled way by marginalization. For a sequence of length T, let $\mathcal{V}$ be the set of time steps for which we have observations. We define $x_{t_0:t_1}^{v} = \{x_t | t_0 \le t \le t_1, t \in \mathcal{V}\}$ as the set of observed variables between t_0 and t_1. Using this notation, $x_{1:T}^{v}$ is the set of observed variables for the given sequence. Similarly, let $\mathcal{M} = \{1:T\} \setminus \mathcal{V}$ and $x_{1:T}^{m}$ be the set of missing observations. The goal of filtering becomes

Authorized licensed use limited to: CSIRO Information Technology Services. Downloaded on September 09,2022 at 00:24:00 UTC from IEEE Xplore. Restrictions apply.

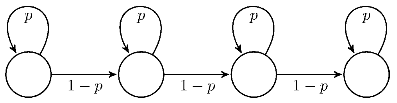

Fig. 3. Topology giving the transition matrix for the new hidden state variables in an explicit duration model with $\tau_0 = 4$.

computing

$$p(z_t|x^v_{1:t}) = \sum_{x^m_{1:t}} p(z_t, x^m_{1:t}|x^v_{1:t}),$$

while for smoothing we want

$$p(z_t|x^v_{1:T}) = \sum_{x^m_{1:T}} p(z_t, x^m_{1:T}|x^v_{1:T}).$$

In the AR-HMM, such marginalizations need to consider the direct dependencies between consecutive observations. For instance, if $t-1 \in \mathcal{M}$, then the forward message at time t must take into account the uncertainty about the unobserved quantity x^m_{t-1}. Our solution is a simple extension of AR-HMM inference. For $t \in \mathcal{M}$ only, we now compute $\alpha(z_t, x^m_t) \triangleq p(z_t, x^m_t, x^v_{1:t})$ and $\beta(z_t, x^m_t) = p(x^v_{t+1:T}|z_t, x^m_t)$. A full explanation is given in the Appendix. After recursively obtaining these messages the desired inference results for $t \in \mathcal{M}$ are obtained by marginalization (e.g., $p(z_t, x^v_{1:t}) = \sum_{x^m_t} \alpha(z_t, x^m_t)$). If $|\mathcal{V}| = T_v$ and $|\mathcal{M}| = T_m$, then the computational expense increases to $O(T_v J^2 + T_m J^2 L^3)$. Since we expect the amount of missing data to be relatively small compared to the size of the dataset, the increase will be modest.

For neonatal condition monitoring the observations are a cross-product of discrete variables (see Section II-A). Missing data can independently occur for each of the monitored events. This means that at certain time steps only some dimensions of x_t are observed. We only need to marginalize over the remaining ones. Extending the missing data inference routine for this case was straightforward.

In practice, forward and backward messages defined as above exponentially decay to zero. For preventing this, we have derived a scaled version of the recursions [23]. It follows the same reasoning as shown in [24, Sec. 13.2.4] for the HMM.

C. Explicit Duration Modeling

HMM-like models make the implicit assumption that the time spent in each hidden state follows a geometric distribution. For sepsis monitoring, we expect episodes of infection to last for at least a few hours. Thus, assuming a geometric distribution for their duration is likely to be a performance limiting factor. There is a large body of work on methods that explicitly model the time spent in each regime (e.g., [15] and [25]).

One solution discussed in [25] and [26] is to replace each state variable with τ_0 copies of itself. Each copy shares the same emission distribution as the original variable. Transitioning between the new states is given by the topology exemplified in Fig. 3. The distribution of staying times becomes

$$p(\tau|p, \tau_0) = \binom{\tau - 1}{\tau_0 - 1} p^{\tau - \tau_0} (1-p)^{\tau_0}. \quad (3)$$

It is defined for $\tau \geq \tau_0$ and is equivalent to the negative binomial distribution [26]. Its mean and variance are $E[\tau] = \tau_0/1 - p$ and $Var[\tau] = p\tau_0/(1-p)^2$, respectively. Inference in the explicit duration AR-HMM shares the same routines with a standard AR-HMM. Taking advantage of the state topology constraints explained earlier, the cost of the forward–backward algorithm becomes $O(TJ(J + 2(\tau_0 - 1)))$.

III. Methods

A. Neonatal Sepsis Diagnosis

The result of the blood culture is widely regarded as the "gold standard" for diagnosing neonatal sepsis. Nevertheless, it is acknowledged that the test can have a poor accuracy [1], [4], [9]. First, small sample volumes and antibiotic therapy can give false-negative results [1]. Estimates show that 30% to 40% of sepsis cases have negative blood tests [9]. Second, positive blood cultures do not always imply infection. The reason is that blood samples often contain contaminants [1].

In the NICU at the Royal Infirmary of Edinburgh, the diagnosis method follows the work of Modi *et al.* [1]. Positive cultures are classified as either recognized pathogens, mixed growth, or skin commensal. For cultures in the first category, clinicians are certain that the patient is infected and the diagnosis is "proven sepsis". The latter two categories cannot distinguish true infection from sample contamination and, if corroborated with the presence of ≥ 3 clinical signs, the diagnosis is recorded as "suspected" sepsis.

B. Neonatal Monitoring Data

We have collected anonymized data from VLBW babies admitted at the NICU in the Royal Infirmary of Edinburgh between 2008 and 2011. All the analyzed patients were intrinsically unstable, and thus nursed in incubators. The data consists exclusively of physiological monitoring channels sampled once per second. These are: heart rate, core and peripheral temperatures ("TC" and "TP") and oxygen saturation ("SO"). Heart rate measurements are available from two sources: ECG leads ("HR") and pulse oximeter ("PR"). Our samples are monitoring windows with a duration of 30 h and fall into one of the following two categories: the sepsis group or the control group. Sepsis samples have been selected such that the time the positive blood sample was taken occurs precisely 24 h after the start of the window.

For the sepsis group, we first considered monitoring all babies who had at least one blood sample taken for culture analysis. The group was refined to include only samples where the culture grew organisms ordinarily considered as pathogenic leading to a diagnosis of "proven sepsis." This was 10% of the original group, as 65% of the samples were negative, and the remaining 25% were allocated to either the mixed growth or skin commensal categories. For the control group, there was no suspicion of sepsis in a consecutive three-day period around the selected intervals and no blood sample had been analyzed.

In order to investigate the utility of multichannel data for sepsis detection, we selected babies for which all the channels

Authorized licensed use limited to: CSIRO Information Technology Services. Downloaded on September 09,2022 at 00:24:00 UTC from IEEE Xplore. Restrictions apply.

above were present. These are needed for defining the events given in Table I. Since there was no systematic reason for the absence of any of these five channels, this is an unbiased selection criterion. During this step, 20% of the sepsis samples were removed. Finally, in some cases, the bedside devices consistently failed to record measurements (or probes were displaced) for extended periods of time. We placed a data availability threshold of 50% for all channels. This resulted in a reduction from 26 to 18 sepsis samples.

Under the same data availability criteria, we selected sufficient control samples to provide an equal amount of data to the sepsis group. In summary, we are studying 36 samples divided as follows:

1) the sepsis group: 18 samples obtained from 18 different patients, mean gestation 27.2 weeks (SD = 1.5), mean birth weight 873 g (SD = 256), and mean age 14.5 days (SD = 8.5);
2) the control group: 18 samples obtained by taking two samples from each of nine different patients,[1] mean gestation 26.7 weeks (SD = 1.7), mean birth weight 837 g (SD = 139), and mean age 15.2 days (SD = 14.0).

Three patients have samples in both sepsis and control groups, which means we are analyzing a total of 24 different neonates.

C. Clinical Events for Sepsis Detection

This study is centered around the idea that the onset of neonatal sepsis is associated with an increase in clinically significant events. In the following, we summarize the knowledge about NICU monitoring data used for defining and annotating these events. We then discuss some summary results of the annotation process.

It is useful to classify clinical events into physiological events and artifactual events. During physiological events, the monitoring traces reflect the true values of the baby's vital signs. Artifactual events occur when the traces are corrupted by faults with the monitoring equipment and do not reflect the true state of the patient. Table I gives the list of clinical events we use, together with their brief descriptions. This list is adapted from the one proposed in [6] for the purposes of this study. Here, we chose not to monitor blood sampling episodes, because of the small amount of blood pressure data available. Several examples of clinical events are shown in Fig. 1. Note that the inclusion of the X-factor [6] makes the list exhaustively cover all the patterns appearing in the monitoring data. Since the X-factor can be either physiological or artifactual, it cannot be directly used for inferring the patient's state of health.

For all the monitoring data in our study, expert annotations were initially obtained for bradycardia, desaturation, handling, and for the X-factor. In subsequent data exploration, the X-factor annotations were inspected for any recurring patterns potentially predictive of sepsis. Annotators found low-amplitude bradycardia-like patterns to display a higher incidence in the hours before the positive test [see Fig. 4(c)]. These would often appear in clusters and close to significant drops in the heart rate. They were not initially annotated as bradycardias because they did not fall into our standard working definition (see Table I). We chose to separately define these events as mini-bradycardias, bradycardias with a drop of 15 to 30 bpm. Thus, annotations for mini-bradycardias were a later addition.

[1]One control sample initially selected has been discarded due to an atypical oxygen saturation trace. This was most likely caused by a fault with the monitoring equipment. The sample was readily classified as an outlier.

TABLE II
CLINICAL EVENT INCIDENCE (NUMBER OF EVENTS), TOTAL AND MEDIAN DURATIONS FOR THE SEPSIS/CONTROL GROUPS

Event	Group	Incidence	Total (hrs)	Median (sec)
Bradycardia	Sepsis	1128	13.9	38.5
	Control	773	8.4	37
Desaturation	Sepsis	742	32.3	101
	Control	231	10.5	124
Mini-Bradycardia	Sepsis	598	10.7	42
	Control	374	4.1	34
Handling	Sepsis	201	41.7	510
	Control	205	53.7	592
X	Sepsis	227	10.3	94
	Control	175	7.0	114
Oximeter error	Sepsis	4051	44.6	16
	Control	3395	36.4	18

Only baby-generated physiological events have been considered. The total amount of data for each group is 18 × 30 = 540 h.

Less clinical expertise is required for annotating the remaining two events, probe dropouts, and oximeter errors. Thus, these artifactual events were handled automatically. The monitoring equipment already marks probe dropouts by recording the value 0. Dropout statistics depend on the channels affected by each clinical event, but on average we lack monitoring data for 2% of the time. Oximeter errors are characterized by a disagreement between the two heart rate channels (see Table I). We first aligned "HR" traces with respect to the "PR" ones by maximizing their cross-correlation. An HMM finding periods of oximeter error was then applied to the difference between the aligned "HR" and "PR." We tested this procedure by comparing it against expert annotations obtained for a subset of our data. An AUC of 0.96 obtained by cross-validation encouraged us to apply the method on the whole dataset.

We frequently observe instances of the other physiological events during handling episodes. An example is shown in Fig. 1(a). In such cases, we cannot distinguish whether the events are caused by the baby's true state of health or because an extremely fragile patient is being handled by staff. Our solution is to not use these instances for sepsis detection. Consequently, we rely exclusively on physiological events happening outside handling episodes. Such instances can be confidently classified as being baby generated.

Table II summarizes the output of the data annotation process. Baby-generated physiological events display a higher incidence in the sepsis group. Also, the amount of patient handling does not differ much between the two groups. The same conclusion can be drawn about the numbers of both X episodes and oximeter errors.

A visualization of the time evolution of the number of baby-generated physiological events is shown in Fig. 4. The samples in the sepsis group are naturally aligned using the time of the positive blood test. Importantly, labels for baby-generated physiological events cannot be provided for all the data. First,

Authorized licensed use limited to: CSIRO Information Technology Services. Downloaded on September 09,2022 at 00:24:00 UTC from IEEE Xplore. Restrictions apply.

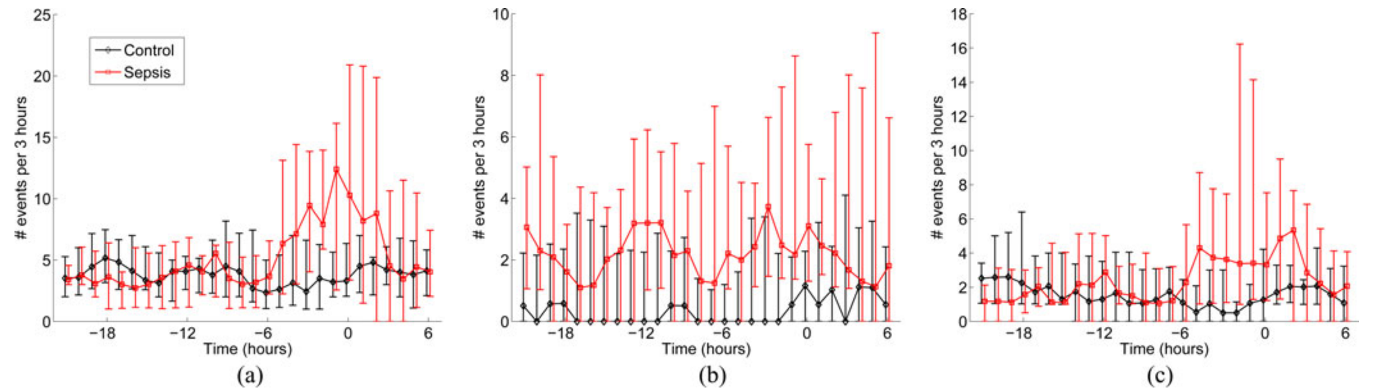

Fig. 4. Time evolution of the median weighted number of baby-generated physiological events for both sepsis and control groups. The data have been aligned such that for babies in the sepsis group 0 denotes the time the positive blood sample was taken. The counts are computed hourly and summarize the preceding 3-h period. The error bars mark the first and third quartiles. We have used a small offset between the two patient groups to improve readability. (a) Bradycardia. (b) Desaturation. (c) Mini-Bradycardia.

as already explained, handling periods were discarded. Second, during probe dropouts, there is no access to the true values of the baby's vital signs and consequently it is impossible to provide annotations. Similarly, during oximeter error events one cannot annotate desaturations. The counts shown have been weighted according to this information. More precisely, if $p\%$ of a monitoring interval could be annotated, the count for that interval was multiplied by $100/p$. For the sepsis group, there is an increase in baby-generated bradycardias and mini-bradycardias in the 9 h before the positive test. Desaturations are generally more present in the sepsis group, but seem to be less informative about the onset of the infection.

These findings can be associated with the work of Griffin, Moorman *et al.* [9], [10]. We do not have access to RR data, but a positive skew in the RR histograms translates into a negative skew of "HR" data, due to the inverse relationship between intervals and frequencies. By computing the sample skewness of the "HR" channel, we found that indeed lower values of skewness often characterize the hours before the positive blood test. However, by removing the bradycardias and mini-bradycardias from the analysis most of the skewness is eliminated. Thus, the distribution of heart rate events and the skew of RR histograms can be interpreted as different observations of the same phenomenon. Our findings about mini-bradycardia events preceding the neonatal sepsis diagnosis provide quantitative evidence with respect to the claims about patterns in heart-rate decelerations made in [27].

The periods of time for which annotations of baby-generated events could not be provided will be treated as missing data. The sources of missing data identified in this section are handling episodes, probe dropouts, and oximeter errors. As discussed previously, the summary statistics of these events do not substantially differ between the two patient groups, justifying the MAR assumption made in Section II-B.

D. Model Fitting

We fit an AR-HMM model to observations of $S = 3$ baby-generated physiological events: bradycardias, desaturations, and mini-bradycardias. The hidden state is chosen to be a binary variable which can take on values $z_t = normal$ or $z_t = sepsis$. In the following, we explain how we label the presence of sepsis in the training data. These labels are then used for supervised learning of the AR-HMM parameters.

Labeling the sepsis variable is different for the two patient groups. For the sepsis group, we know the exact time of the positive blood test. Following consultation with clinicians, it was agreed that labeling the period of 6 h before this moment as sepsis would be reasonable. The onset of the infection cannot be assumed to be an instantaneous event. Thus, we define a transition period in which the patient progresses from being in the $normal$ state to being in the $sepsis$ state. We take this to be 12 h between 18 and 6 h before the positive test. This period is left unlabeled and will not be used for either training or testing. All monitoring data before the transition period (i.e., the first 6 h of a sample in the sepsis group) is labeled as $normal$. We do not assign a label to the data after the positive test, as this is likely to be affected by the patient's response to treatment and has less relevance for the task of real-time sepsis detection. All the data in the control group are labeled as $normal$.

The use of the annotations simplifies parameter estimation. Our optimization goal is maximizing the joint probability of the labeled hidden states and the corresponding observations. Maximum *a posteriori* (MAP) estimates of the state transition probabilities are given by

$$\hat{\theta}_{j|i} = \frac{n_{j|i} + \xi}{\sum_{j'} (n_{j'|i} + \xi)}, \tag{4}$$

where $n_{j|i}$ is the number of times, we transit from hidden state i to hidden state j. Here, we use a symmetric Dirichlet prior with parameter $\xi = 1$, in order to prevent estimates from being too small when data counts are low. Similarly, we learn the emission probability parameters using

$$\hat{\phi}^{(s)}_{l|kj} = \frac{n^{(s)}_{l|kj} + \xi}{\sum_{l'} (n^{(s)}_{l'|kj} + \xi)}, \tag{5}$$

where $n^{(s)}_{l|kj}$ is the number of times event s transitions from setting k to setting l when the hidden state takes on value j. In

Authorized licensed use limited to: CSIRO Information Technology Services. Downloaded on September 09,2022 at 00:24:00 UTC from IEEE Xplore. Restrictions apply.

TABLE III
SUMMARY STATISTICS OBTAINED BY CROSS-VALIDATION IN A SECOND-BY-SECOND ANALYSIS

	Filtering		Smoothing	
	AUC	EER	AUC	EER
AR-HMM md	0.74	0.33	0.75	0.29
AR-HMM wmd	0.72	0.34	0.73	0.32
HMM	0.50	0.46	0.53	0.40
AR-HMM ed	0.80	0.30	0.79	0.27

Section III-C, we explained why it was not always possible to annotate baby-generated events. We could use an expectation-maximization (EM) procedure to account for missing data when estimating parameters. However, the total amount of annotated data is much larger than the amount of missing data. Thus, we would expect the benefits to be minimal.

Note that, in the absence of any sepsis labels, the AR-HMM can be trained unsupervised. Maximum likelihood parameters are usually determined by optimizing the probability of the observations with EM. The inference procedure described in Section II-B can be used in the expectation step.

IV. RESULTS

In this section, we describe experimental results for detecting neonatal sepsis on the data introduced in Section III-B. We show the models' performance and discuss learnt parameters in Section IV-A. The relevance of individual physiological event streams is examined in Section IV-B. An alternative episode-based analysis is presented in Section IV-C.

The following results have been obtained using leave-one-out cross-validation. The quality of the second-by-second inferences is measured against the sepsis labeling defined in Section III-D. We draw ROC curves showing the dependence between the false positive rate (FPR) and the true positive rate (TPR). We report the AUC and the equal error rate (EER).[2] These evaluation criteria are preferred because they account for the class imbalance in our dataset. If misclassification costs had been available, we could have visualized the expected cost in ROC space as explained in [28]. Posterior distributions are given as gray-scale horizontal bars, with white meaning 0 sepsis probability and black corresponding to probability 1. For the episode-based analysis, we use precision-recall (PR) curves[3] [29]. We report the average precision (AP) [30] and the maximum F-score.[4] Both the second-by-second and the episode-based analyses are projections of the inferences onto different metrics and can reveal different performance aspects.

A. Model Evaluation With a Second-by-Second Analysis

ROC curves for several sepsis models are given in Fig. 5, and the corresponding summary statistics are presented in Table III. We are mainly interested in real-time prediction. Smoothing results can be interpreted as an upper bound for the predictive power of the selected physiological events.

[2]EER is the error rate computed at the threshold for which the FPR equals the false negative rate (FNR). Note that FNR = 1 − TPR.

[3]Precision is defined as TP/(TP+FP) and recall equals the TPR.

[4]The F-score is the harmonic mean of precision and recall.

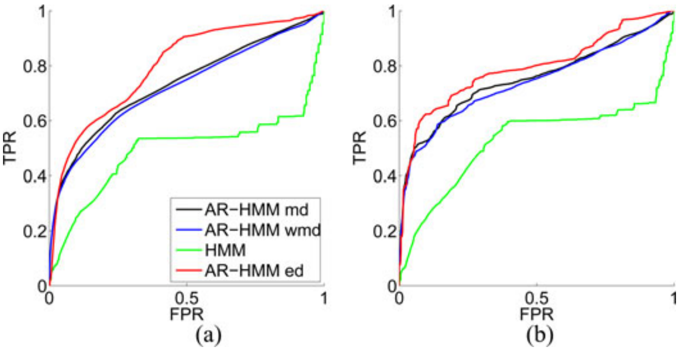

Fig. 5. ROC curves corresponding to different models for sepsis detection. (a) Filtering. (b) Smoothing.

TABLE IV
MONTE CARLO ESTIMATES OF THE EXPECTED NUMBER OF BABY-GENERATED EVENTS OVER A $T = 3$-h PERIOD

State	Bradycardia	Desaturation	Mini-Bradycardia
Normal	4.29	1.63	2.10
Sepsis	11.01	5.95	6.65

"AR-HMM md" is the standard AR-HMM model which handles missing data. If we do label the missing data assuming no baby-generated physiological event was happening, we obtain a model without any missing data, "AR-HMM wmd." This approach performs worse, mostly due to long handling events happening during sepsis episodes wrongly classified as normal. The marginalization performed in the missing data approach helps to correctly classify these periods as sepsis. The benefits of explicitly modeling events as Markov chains with AR-HMMs are clear when compared to an "HMM," whose performance is close to that of a random classifier. "AR-HMM ed" explicitly models staying times in the hidden states. For each hidden state, the parameters of the corresponding event duration distribution (3) can be learnt from the sepsis labeling. However, due to the lack of diversity in the length of labeled sepsis episodes, we treat τ_0 as a hyper-parameter, and consider values in $\{5, 10, 15, 25, 50, 100\}$. To avoid bias, we determine the performance of the explicit duration model using nested cross-validation (see, e.g., [31]). In the inner cross-validation steps, selection for τ_0 was performed using the filtering AUC. Table III shows that the explicit duration model delivers the best performance for both filtering and smoothing.

The fitted emission distributions can be used to characterize the $sepsis$ and $normal$ regimes. Since the learnt ϕ's are hard to interpret directly, we show an alternative representation which can be easily associated with the information in Fig. 4. More precisely, we used a Monte Carlo approach to estimate the expected number of physiological events over a $T = 3$-h period. For each event–regime pair, we separately sampled the corresponding Markov chain. In all cases, $N = 5000$ samples of length $T = 3$ h have been empirically found to suffice for convergence. Table IV shows these estimates. In the $sepsis$ state, we see an approximately threefold increase in the expected incidence of the monitored events compared to periods when the patients are not infected.

Authorized licensed use limited to: CSIRO Information Technology Services. Downloaded on September 09,2022 at 00:24:00 UTC from IEEE Xplore. Restrictions apply.

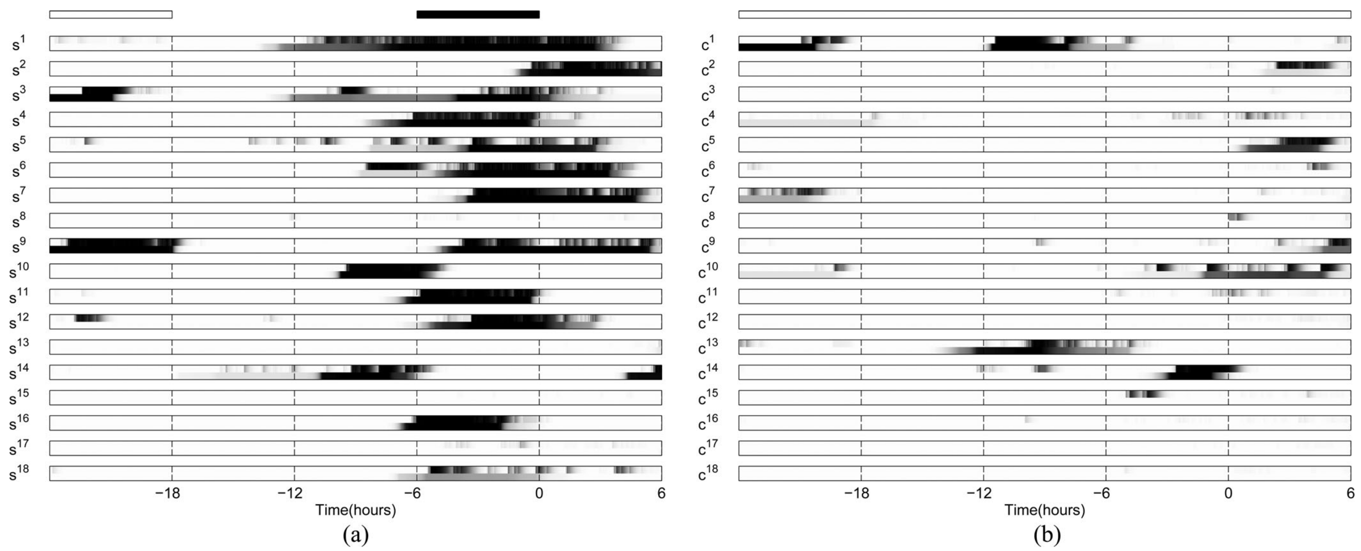

Fig. 6. Cross-validation inference for both patient groups using the explicit-duration AR-HMM. The top row of each figure represents the sepsis labeling. Normal periods are white, sepsis periods are black. Transitioning and treatment periods are not assigned any label. For each sepsis sample s^k or control sample c^k, the top row of the corresponding image represents the filtering distribution and the bottom row represents the smoothing distribution. (a) Sepsis group. (b) Control group.

TABLE V
SUMMARY STATISTICS FOR THE SECOND-BY-SECOND ANALYSIS OF EXPLICIT DURATION AR-HMMS MODELING SEVERAL SETS OF PHYSIOLOGICAL EVENTS

		ALL	BR+MB	DS
Filtering	AUC	0.80	0.80	0.78
	EER	0.30	0.30	0.30
Smoothing	AUC	0.79	0.79	0.76
	EER	0.27	0.27	0.32

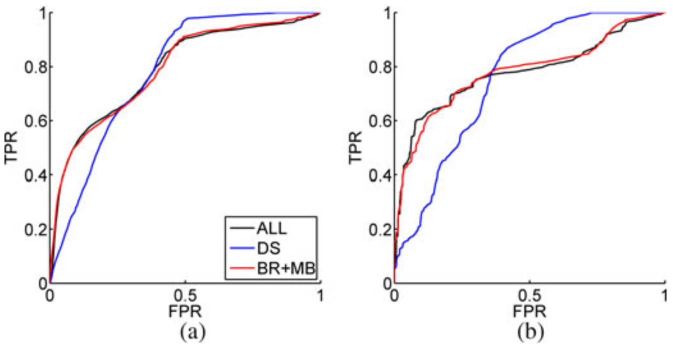

Fig. 7. ROC curves corresponding to different sets of physiological events modeled with an explicit duration AR-HMM for sepsis detection. (a) Filtering. (b) Smoothing.

Fig. 6 shows inference results for the model delivering the best performance, the explicit duration AR-HMM. For 12 samples in the sepsis group (s^{1-7}, s^9, s^{11}, s^{12}, s^{16}, and s^{18}), a significantly long sepsis episode is identified during 6 hours before the positive blood test. In all but two samples (s^2 and s^3), the sepsis episode is detected at least 3 h before the positive test. For two cases (s^{10} and s^{14}), sepsis episodes are flagged mostly during the transition period rather than the sepsis one. In the remaining four samples (s^8, s^{13}, s^{15}, and s^{17}), no clear sepsis episode has been identified. The sepsis periods flagged in the control group are usually short. We believe that many of them can be explained by handling events which do not display corresponding falls in either "TC" or "TP" channels.

B. Physiological Event Evaluation

It is useful to understand which types of physiological events contribute most for detecting sepsis. Since bradycardias and mini-bradycardias are intimately related, we phrase this question as asking whether monitoring desaturations brings additional information about sepsis compared to only monitoring the heart rate. In Fig. 7 and Table V, we compare an explicit duration AR-HMM monitoring all events ("ALL") with one monitoring only heart rate channel events ("BR+MB") and one looking only at desaturations ("DS"). For all event types, ROC curves have been obtained using nested cross-validation. This analysis shows that monitoring desaturations on top of monitoring the heart rate channel does not give better performance. Also, due to better TPR values at high FPRs, monitoring only desaturations delivers a surprisingly good performance. However, when choosing an operating point from these ROC curves, we are more interested in the performance at low FPRs.

The event-type analysis is continued in the next section when looking at episode-based analysis.

C. Episode-Based Analysis

We have also evaluated our models from the perspective of detecting episodes of infection. This analysis is intended to be closer to clinical practice than the second-by-second evaluation. Similar procedures have been used in applications such as object detection [30] or keyword spotting [32, Sec. 17.19].

First, the posteriors are thresholded to give a binary output. Strings of 1's are predicted sepsis episodes. Since true episodes last for at least a few hours, we keep only instances longer than 1 h. An inferred episode overlapping with the *sepsis* period but not with any *normal* period is a true positive (TP). If multiple TPs are detected for a sepsis patient, then only the first is recorded. Episodes exclusively contained in either the transition

Authorized licensed use limited to: CSIRO Information Technology Services. Downloaded on September 09,2022 at 00:24:00 UTC from IEEE Xplore. Restrictions apply.

TABLE VI
SUMMARY STATISTICS OBTAINED BY CROSS-VALIDATION IN AN EPISODE-BASED ANALYSIS

	Filtering		Smoothing	
	AP	F-score	AP	F-score
AR-HMM md	0.59	0.61	0.60	0.65
AR-HMM wmd	0.56	0.59	0.57	0.63
HMM	0.10	0.29	0.19	0.32
AR-HMM ed	0.59	0.65	0.63	0.69

TABLE VII
SUMMARY STATISTICS FOR THE EPISODE-BASED ANALYSIS OF EXPLICIT DURATION AR-HMMS MODELING SEVERAL SETS OF PHYSIOLOGICAL EVENTS

		ALL	BR+MB	DS
Filtering	AP	0.59	0.53	0.33
	F-score	0.65	0.59	0.46
Smoothing	AP	0.63	0.61	0.25
	F-score	0.69	0.65	0.42

or treatment periods are not labeled. All remaining episodes are false positives (FP). The number of positive examples is the number of infected patients. In this setting, evaluation via a PR curve is a better choice than via a ROC one, as true negative episodes are hard to define, and are not necessary for a PR curve.

Table VI shows the AP and maximum F-score for the same set of models discussed in Section IV-A. The performance of the explicit duration model is again computed using nested cross-validation. However, we now optimize the filtering AP in the inner cross-validation. Most of the findings in Table III are confirmed in PR space as well. Again, the explicit duration model dominates the other models, although its filtering AP score equals that of the standard AR-HMM model.

It was interesting to perform the evaluation of Section IV-B using the episode-based analysis. The summary statistics are given in Table VII. The episode-based analysis reveals bigger performance differences between the models. Monitoring desaturations on top of monitoring the heart rate channels improves both filtering and smoothing performance. Monitoring only desaturations does much worse when we assess the detected infection episodes.

V. DISCUSSION

This paper introduces a hidden variable probabilistic model capable of making early predictions about the onset of neonatal sepsis. Our approach extensively uses domain knowledge to facilitate learning and inference. We have explained how missing data can be treated and experimented with explicit duration modeling. The results show that by monitoring the incidence of baby-generated physiological events, we can often detect sepsis well in advance of the time when a positive blood test was taken. In the remainder, we discuss a number of ways in which this study can be extended.

The primary direction is to directly use the raw physiological data to infer sepsis. A hierarchical model can be obtained assuming that the clinical events are unobserved and placing the hidden state variables of the AR-HMM on top of the FSLDS of Quinn *et al.* [6]. Inference in the resulting model can be performed using the same methods as for the FSLDS [33]. A direct comparison against the results presented in Section IV is possible, partly because the AR-HMM can be obtained by conditioning the hierarchical model on the clinical events. We can interpret the AR-HMM results as an upper bound for the performance of a fully automated sepsis detection system relying on the distribution of clinical events. The bound is justified by the fact that any automated system for inferring clinical events from monitoring data cannot outperform an expert annotator. Automatically detecting sepsis from monitoring data also opens up the opportunity to study much larger datasets.

In Section III-C, we have explained how the distribution of physiological events is related to previous work on using the loss of variability in the vital signs for sepsis prediction [9], [14]. It would be interesting to define such events and see what predictive effect they have in our framework, either on their own or combined with the other events. However, assessment of variability requires comparison of data over a given time frame, and there are many possible ways to come up with measures of variability. It could be interesting to look at this question in the FSLDS framework [6], where a new factor and dynamical model for low variability could be introduced.

In Section III-D, we have explained a method for labeling periods of infection. The main difficulty was that while the time of the positive test is known, the time of the sepsis onset cannot be exactly determined. An interesting alternative is to construct a probabilistic labeling of sepsis. At the time of the positive test, the probability of sepsis should be 1 and going back in time it should monotonically decay to 0. In this case, the goal of learning is to minimize the Kullback–Leibler divergence between the labeling and the posterior distribution of the AR-HMM. This objective can be optimized by a gradient descent method using inference as a subroutine. The procedure is closely related to discriminative training of HMMs [34], [35]. A difficulty of this approach is that some parametric form of the labeling distribution should be fixed *a priori*.

APPENDIX
AR-HMM INFERENCE WITH MISSING DATA

When there is no missing data, the messages in (2) can be recursively computed as follows:

$$\alpha(z_t) = p(x_t|z_t, x_{t-1}) \sum_{z_{t-1}} p(z_t|z_{t-1})\alpha(z_{t-1}) \quad (6)$$

$$\beta(z_t) = \sum_{z_{t+1}} p(z_{t+1}|z_t)p(x_{t+1}|z_{t+1}, x_t)\beta(z_{t+1}). \quad (7)$$

In the presence of missing data, $\mathcal{V}$ is the set of time steps for which we have observations. We would like to treat both $t \in \mathcal{V}$ and $t \notin \mathcal{V}$ in a unified framework. Thus, we introduce a function $V(x_t) : \{1, \ldots, L\} \rightarrow \{0, 1\}$

$$V(x_t) = \begin{cases} \delta_{x_t x_t^v}, & \text{if } t \in \mathcal{V} \\ 1, & \text{if } t \notin \mathcal{V} \end{cases}$$

where δ_{ij} is the Kronecker delta. For any $t_0 < t < t_1$, the following holds:

Authorized licensed use limited to: CSIRO Information Technology Services. Downloaded on September 09,2022 at 00:24:00 UTC from IEEE Xplore. Restrictions apply.

$$p(z_t, x^v_{t_0:t_1}) = \sum_{x_t} p(z_t, x^v_{t_0:t-1}, x_t, x^v_{t+1:t_1}) V(x_t). \quad (8)$$

If x_t is not observed, the summation in (8) represents the marginalization of the hidden variable x_t from $p(z_t, x^v_{t_0:t_1}, x_t)$. If x_t is observed, then the summation only selects the term $p(z_t, x^v_{t_0:t-1}, x^v_{t+1:t_1}, x^v_t)$.

Applying (8) together with (2) we get

$$\begin{aligned} p(z_t, x^v_{1:T}) &= \sum_{x_t} p(z_t, x^v_{1:t-1}, x_t, x^v_{t+1:T}) V(x_t) \\ &= \sum_{x_t} p(z_t, x^v_{1:t-1}, x_t) p(x^v_{t+1:T} | z_t, x_t, x^v_{1:t-1}) V(x_t) \\ &= \sum_{x_t} p(z_t, x^v_{1:t-1}, x_t) p(x^v_{t+1:T} | z_t, x_t) V(x_t) \\ &= \sum_{x_t} \alpha(z_t, x_t) \beta(z_t, x_t) \end{aligned}$$

where we have defined the messages

$$\alpha(z_t, x_t) \triangleq p(z_t, x^v_{1:t-1}, x_t) V(x_t)$$

$$\beta(z_t, x_t) \triangleq p(x^v_{t+1:T} | z_t, x_t) V(x_t)$$

and we have used the fact that $V^2(x_t) = V(x_t)$. Similar to (6) and (7), the following recursions can be written

$$\begin{aligned} \alpha(z_t, x_t) &= V(x_t) \sum_{x_{t-1}} p(z_t, x^v_{1:t-2}, x_{t-1}, x_t) V(x_{t-1}) \\ &= V(x_t) \sum_{x_{t-1}} \sum_{z_{t-1}} p(z_{t-1}, z_t, x^v_{1:t-2}, x_{t-1}, x_t) V(x_{t-1}) \\ &= V(x_t) \sum_{x_{t-1}} p(x_t | z_t, x_{t-1}) \\ &\quad \times \sum_{z_{t-1}} p(z_{t-1}, z_t, x^v_{1:t-2}, x_{t-1}) V(x_{t-1}) \\ &= V(x_t) \sum_{x_{t-1}} p(x_t | z_t, x_{t-1}) \sum_{z_{t-1}} p(z_t | z_{t-1}) \alpha(z_{t-1}, x_{t-1}) \end{aligned}$$

$$\begin{aligned} \beta(z_t, x_t) &= V(x_t) \sum_{x_{t+1}} p(x_{t+1}, x^v_{t+2:T} | z_t, x_t) V(x_{t+1}) \\ &= V(x_t) \sum_{x_{t+1}} \sum_{z_{t+1}} p(z_{t+1}, x_{t+1}, x^v_{t+2:T} | z_t, x_t) V(x_{t+1}) \\ &= V(x_t) \sum_{x_{t+1}} \sum_{z_{t+1}} p(z_{t+1} | z_t) p(x_{t+1}, x^v_{t+2:T} | z_{t+1}, x_t) V(x_{t+1}) \\ &= V(x_t) \sum_{z_{t+1}} p(z_{t+1} | z_t) \sum_{x_{t+1}} p(x_{t+1} | z_{t+1}, x_t) \beta(z_{t+1}, x_{t+1}). \end{aligned}$$

When training an AR-HMM with missing data via EM, the following quantities are needed in the M-step

$$\begin{aligned} p(z_t, z_{t-1}, x^v_{1:T}) &= \sum_{x_t, x_{t-1}} p(z_t, z_{t-1}, x^v_{1:t-2}, x_{t-1}, x_t, x^v_{t+1:T}) V(x_t) V(x_{t-1}) \\ &= \sum_{x_t, x_{t-1}} \alpha(z_{t-1}, x_{t-1}) p(z_t | z_{t-1}) p(x_t | z_t, x_{t-1}) \beta(z_t, x_t) \end{aligned}$$

$$\begin{aligned} p(z_t, x^v_{1:t-2}, x_{t-1}, x_t, x^v_{t+1:T}) V(x_t) V(x_{t-1}) &= \sum_{z_{t-1}} p(z_t, z_{t-1}, x^v_{1:t-2}, x_{t-1}, x_t, x^v_{t+1:T}) V(x_t) V(x_{t-1}) \\ &= \sum_{z_{t-1}} \alpha(z_{t-1}, x_{t-1}) p(z_t | z_{t-1}) p(x_t | z_t, x_{t-1}) \beta(z_t, x_t). \end{aligned}$$

ACKNOWLEDGMENT

We would like to thank Prof. N. McIntosh for providing expert insight and supervising with data annotation.

REFERENCES

[1] N. Modi, C. J. Doré, A. Saraswatula, M. Richards, K. B. Bamford, R. Coello, and A. Holmes. (2009, Jan.). A case definition for national and international neonatal bloodstream infection surveillance. *Arch. Disease Childhood—Fetal Neonatal Edition* [Online]. *94(1)*, pp. F8–F12. Available: http://dx.doi.org/10.1136/adc.2007.126458

[2] B. J. Stoll, N. Hansen, A. A. Fanaroff, L. L. Wright, W. A. Carlo, R. A. Ehrenkranz, J. A. Lemons, E. F. Donovan, A. R. Stark, J. E. Tyson *et al.* (2002). Late-onset sepsis in very low birth weight neonates: The experience of the NICHD Neonatal Research Network. *Pediatrics* [Online]. *110(2 Pt 1)*, pp. 285–291. Available: http://www.ncbi.nlm.nih.gov/pubmed/12165580

[3] C. M. Beck-Sague, P. Azimi, S. N. Fonseca, R. S. Baltimore, D. Powell, L. A. Bland, M. J. Arduino, S. McAllister, R. S. Huberman, and R. L. Sinkowitz, "Bloodstream infections in neonatal intensive care unit patients: Results of a multicenter study," *Pediatr. Infect. Disease J.*, vol. 13, no. 12, pp. 1110–1116, 1994.

[4] M. P. Griffin, T. M. O'Shea, E. A. Bissonette, F. E. Harrell, D. E. Lake, and J. R. Moorman. (2003). Abnormal heart rate characteristics preceding neonatal sepsis and sepsis-like illness. *Pediatr Res* [Online]. *53(6)*, pp. 920–926. Available: http://www.biomedsearch.com/nih/Abnormal-heart-rate-characteristics-pre ceding/12646726.html

[5] J. Quinn. (2007). "Bayesian Condition Monitoring in Neonatal Intensive Care," Ph.D. dissertation, Univ. Edinburgh, [Online]. Available: http://hdl.handle.net/1842/2144

[6] J. A. Quinn, C. K. I. Williams, and N. McIntosh, "Factorial switching linear dynamical systems applied to physiological condition monitoring," *IEEE Trans. Pattern Anal. Mach. Intell.*, vol. 31, no. 9, pp. 1537–1551, Sep. 2009.

[7] R. Shumway and D. Stoffer, "Dynamic linear models with switching," *J. Amer. Statist. Assoc.*, vol. 86, pp. 763–769, 1991.

[8] Z. Ghahramani and M. I. Jordan, "Factorial hidden Markov models," *Mach. Learn.*, vol. 29, pp. 245–273, 1997.

[9] M. P. Griffin and J. R. Moorman, "Toward the early diagnosis of neonatal sepsis and sepsis-like illness using novel heart rate analysis," *Pediatrics*, vol. 107, pp. 97–104, 2001.

[10] J. Moorman, D. Lake, and M. Griffin, "Heart rate characteristics monitoring for neonatal sepsis," *IEEE Trans. Biomed. Eng.*, vol. 53, no. 1, pp. 126–132, Jan. 2006.

[11] J. R. Moorman, W. A. Carlo, J. Kattwinkel, R. L. Schelonka, P. J. Porcelli, C. T. Navarrete, E. Bancalari, J. L. Aschner, M. W. Walker, J. A. Perez, C. Palmer, G. J. Stukenborg, D. E. Lake, and T. M. OShea. (2011). Mortality reduction by heart rate characteristic monitoring in very low birth weight neonates: A randomized trial. *J. Pediatr.* [Online]. *159(6)*, pp. 900–906.e1. [Online]. Available: http://www.sciencedirect.com/science/article/pii/S0022347611006718

[12] M. Blount, M. Ebling, J. Eklund, A. James, C. McGregor, N. Percival, K. Smith, and D. Sow, "Real-time analysis for intensive care: Development and deployment of the artemis analytic system," *IEEE Eng. Med. Biol. Mag.*, vol. 29, no. 2, pp. 110–118, Mar./Apr. 2010.

[13] M. Stacey, C. McGregor, and M. Tracy, "An architecture for multi-dimensional temporal abstraction and its application to support neonatal intensive care," in *Proc. IEEE 29th Annu. Int. Conf. Eng. Med. Biol. Soc.*, Aug. 2007, pp. 3752–3756.

[14] C. McGregor, C. Catley, and A. James, "Variability analysis with analytics applied to physiological data streams from the neonatal intensive care unit," in *Proc. 25th Int. Symp. Comput.-Based Med. Syst.*, 2012, pp. 1–5.

Authorized licensed use limited to: CSIRO Information Technology Services. Downloaded on September 09,2022 at 00:24:00 UTC from IEEE Xplore. Restrictions apply.

[15] L. Rabiner, "A tutorial on Hidden Markov Models and selected applications in speech recognition," *Proc. IEEE*, vol. 77, no. 2, pp. 257–286, Feb. 1989.

[16] C. D. Manning and H. Schütze, *Foundations of Statistical Natural Language Processing*. Cambridge, MA, USA: MIT Press, 1999.

[17] A. Krogh, M. Brown, I. S. Mian, K. Sjlander, and D. Haussler, "Hidden Markov models in computational biology: Applications to protein modeling," *J. Molecul. Biol.*, vol. 235, pp. 1501–1531, 1994.

[18] D. Coast, R. Stern, G. Cano, and S. Briller, "An approach to cardiac arrhythmia analysis using hidden Markov models," *IEEE Trans. Biomed. Eng.*, vol. 37, no. 9, pp. 826–836, Sep. 1990.

[19] R. Andreao, B. Dorizzi, and J. Boudy, "ECG signal analysis through hidden Markov models," *IEEE Trans. Biomed. Eng.*, vol. 53, no. 8, pp. 1541–1549, Aug. 2006.

[20] Y. Ephraim, D. Malah, and B.-H. Juang, "On the application of hidden Markov models for enhancing noisy speech," *IEEE Trans. Acoust., Speech Signal Process.*, vol. 37, no. 12, pp. 1846–1856, Dec. 1989.

[21] P. Woodland, "Hidden Markov models using vector linear prediction and discriminative output distributions," in *Proc. IEEE Int. Conf. Acoust., Speech, Signal Process.*, 1992, vol. 1, pp. 509–512.

[22] R. Little and D. Rubin, *Statistical Analysis With Missing Data*. New York, NY, USA: Wiley, 1987.

[23] I. Stanculescu, "Dynamical models for neonatal intensive care," Forthcoming Ph.D. thesis, School of Informatics, Univ. Edinburgh, Edinburgh, U.K., 2014.

[24] C. M. Bishop, *Pattern Recognition and Machine Learning*. New York, NY, USA: Springer-Verlag, 2007.

[25] M. Johnson, "Capacity and complexity of HMM duration modeling techniques," *IEEE Signal Process. Lett.*, vol. 12, no. 5, pp. 407–410, May 2005.

[26] K. Murphy, *Machine Learning: A Probabilistic Perspective*, (Adaptive Computation and Machine Learning Series). Cambridge, MA, USA: MIT Press, 2012.

[27] A. Flower, R. J. Moorman, D. Lake, and J. Delos, "Periodic heart rate decelerations in premature infants," *Exp. Biol. Med.*, vol. 235, no. 4, pp. 531–538, 2010.

[28] F. Provost and T. Fawcett. (2001, Mar.). Robust classification for imprecise environments. *Mach. Learn.* [Online]. *42(3)*, pp. 203–231. Available: http://dx.doi.org/10.1023/A:1007601015854

[29] V. Raghavan, P. Bollmann, and G. S. Jung. (1989, Jul.). A critical investigation of recall and precision as measures of retrieval system performance. *ACM Trans. Inf. Syst.* [Online]. *7(3)*, pp. 205–229. Available: http://doi.acm.org/10.1145/65943.65945

[30] M. Everingham and J. Winn. (2012). "The PASCAL Visual Object Classes Challenge 2012 (VOC2012) Development Kit," [Online]. Available: http://www.pascal-network.org/challenges/VOC/voc2012

[31] S. Varma and R. Simon. (2006, Feb.). Bias in error estimation when using cross-validation for model selection. *BMC Bioinformat.* [Online]. *7(1)*, pp. 91+. Available: http://dx.doi.org/10.1186/1471-2105-7-91

[32] S. J. Young, G. Evermann, M. J. F. Gales, T. Hain, D. Kershaw, G. Moore, J. Odell, D. Ollason, D. Povey, V. Valtchev, and P. C. Woodland, *The HT Book, version 3.4*. Cambridge, U.K.: Cambridge Univ. Engineering Department, 2006.

[33] D. Barber and B. Mesot, "A novel Gaussian sum smoother for approximate inference in switching linear dynamical systems," in *Advances in Neural Information Processing Systems 19*, B. Schölkopf, J. Platt, and T. Hoffman, Eds. Cambridge, MA, USA: MIT Press, 2007, pp. 89–96.

[34] L. Bahl, P. Brown, P. De Souza, and R. Mercer, "Maximum mutual information estimation of hidden Markov model parameters for speech recognition," in *Proc. IEEE Int. Conf. Acoust., Speech, Signal Process.*, 1986, vol. 11, pp. 49–52.

[35] A. Krogh and S. K. Riis, "Hidden neural networks," *Neural Comput.*, vol. 11, pp. 541–563, 1999.

Ioan Stanculescu received the B.Sc. degree in electronics, telecommunications, and information technology from the Politehnica University of Bucharest, Bucharest, Romania, in 2009, and the M.Sc. degree in artificial intelligence from the University of Edinburgh, Edinburgh, U.K., in 2010, where he is currently working toward the Ph.D. degree in the Institute for Adaptive and Neural Computation, School of Informatics.

His research is centred on developing probabilistic models for explaining time series data and has deep interests in machine learning and Bayesian inference.

Christopher K. I. Williams received the M.Sc. and Ph.D. degrees from the University of Toronto, Toronto, ON, Canada, in 1990 and 1994, respectively, under the supervision of Geoff Hinton.

He is currently a Professor of machine learning in the School of Informatics, University of Edinburgh, Edinburgh, U.K. He was a member of the Neural Computing Research Group at Aston University from 1994 to 1998, and has been at the University of Edinburgh since 1998. He is interested in a wide range of theoretical and practical issues in machine learning, statistical pattern recognition, probabilistic graphical models and computer vision. This includes theoretical foundations, the development of new models and algorithms, and applications. His main areas of research are in visual object recognition and image understanding, models for understanding time-series, unsupervised learning, and Gaussian processes.

Dr. Williams was the Program Cochair of NIPS in 2009, and is on the editorial boards of the *Journal of Machine Learning Research*, the *International Journal of Computer Vision*, and Proceedings of the Royal Society A.

Yvonne Freer received the Ph.D. degree in nursing from the University of Edinburgh, Edinburgh, U.K., in 1997.

She is currently a Clinical Academic Nurse at the Royal Infirmary of Edinburgh, Edinburgh, U.K. Her research has been on clinically focused issues within the neonatal intensive care unit and she has published papers in peer reviewed journals on aspects of information and communication technologies. One of her primary research interests is providing support for parents of babies admitted to the neonatal unit, through collaboration and information sharing.

Authorized licensed use limited to: CSIRO Information Technology Services. Downloaded on September 09,2022 at 00:24:00 UTC from IEEE Xplore. Restrictions apply.
