# Supplementary material for: Exploring Computational Techniques in Preprocessing Neonatal Physiological Signals for Detecting Adverse Outcomes: Scoping Review
Source: Interact J Med Res. 2024 Aug 20;13:e46946. doi: 10.2196/46946 (PMC11372324; doi:10.2196/46946)
Supplement: Multimedia Appendix 3 [file ijmr_v13i1e46946_app3.zip › Included Papers - Final/3493/Williamson et al. - 2013 - Individualized apnea prediction in preterm infants.pdf]

Individualized Apnea Prediction in Preterm Infants using Cardio-Respiratory and Movement Signals

James R. Williamson, Daniel W. Bliss, David W. Browne,
Premananda Indic, Elisabeth Bloch-Salisbury, and David Paydarfar

Abstract—**Apnea of prematurity is a common developmental disorder in preterm infants that is implicated in a number of acute and long-term complications. Therapeutic stochastic resonance (TSR) is a noninvasive preventative intervention for stabilizing breathing patterns and reducing the incidence of apnea and hypoxia. Because the stabilizing effect of TSR lags its initiation, it can be used most effectively if it is linked to a system for apnea prediction. We present a real-time algorithm for generating apnea predictions based on cardio-respiratory and movement features extracted from multiple physiological sensors. The features are used to create patient-specific statistical models of apnea precursors. The state parameters generated by these models are evaluated over time to form apnea predictions. The algorithms predictions are evaluated using a short, 5.5 minute prediction horizon. The algorithm obtains highly accurate predictions, with statistical significance obtained on five out of the six patients that it is evaluated on.**

Index Terms—**prematurity, hypoxia, bradycardia, monitoring, algorithm, feature vector, machine learning**

I. Introduction

ONE in eight live births in the United States is preterm (< 37 weeks post conception) [1] and these high risk births require specialized monitoring and treatment in neonatal intensive care units (NICU). Apneic pauses causing transient hypoxia and associated bradycardia - often referred to as cardio-respiratory events - are common in preterm infants [2]-[6], with severity ranging from presumably benign periodic apnea with mild oxygen desaturations and cardiac decelerations to severe life-threatening apnea that requires mechanical ventilation. Prospective studies have linked intermittent hypoxia with a number of acute and long-term complications [2]-[6], including multiorgan dysfunction, retinopathy [7], developmental delays, and neuropsychiatric disorders. There remains uncertainty regarding how immaturity of respiratory control leads to poor outcomes. However, it is clear that apnea of prematurity (AOP) is a major factor in prolonging hospitalization as well as raising concerns for subsequent risk of apparent life-threatening events and sudden infant death syndrome (SIDS) at home [2]-[6]. Despite the existence of interventions for apnea of prematurity [4], [5], [8], [9], [10], there remains strong endorsement by neonatologists [2]–[6], [11] for developing new approaches to stabilize breathing patterns and to prevent intermittent hypoxia and bradycardia episodes in preterm infants.

One new approach that has shown significant efficacy in stabilizing infant breathing is therapeutic stochastic resonance (TSR) using small stochastic displacements in mattresses from embedded actuators [12]. In a study of 10 preterm infants, TSR induced a $\approx 50\%$ reduction ($P = 0.003$) in the variance of interbreath intervals and a $\approx 50\%$ reduction in the incidence of apneic pauses of all durations > 5 sec ($P = 0.002$). The improved stability of breathing was associated with a $\approx 65\%$ reduction in the duration of O_2 desaturation ($P = 0.04$). The physiological effects of TSR appears to exhibit a time lag with onset and offset half times of about $1-2$ minutes [12].

TSR would therefore benefit from predictive knowledge of when a patient is at high risk for apnea. We have developed an algorithm for providing this predictive knowledge by detecting apnea precursors that are observed in multimodal clinical data minutes before the onset of significant apnea and hypoxia events. Motivated by our success in predicting epileptic seizures from electroencephalographic signals [13], [14], we have adopted a prediction framework that comprises three significant components: 1. feature-vector construction from multimodal time series data, 2. machine learning of patient-specific statistical models, and 3. evaluation of state parameters. A previous version of our algorithm has shown promising results in apnea prediction based on features extracted solely from cardio-respiratory signals [15]. In our current work, we improve upon these results by complementing the cardio-respiratory features with infant movement features, and by employing a more sophisticated statistical modeling approach.

II. Methods

A. Patient data and preprocessing

Our study was approved by the Committee on the Use of Humans as Experimental Subjects at the Massachusetts Institute of Technology and the Committee for the Protection of Human Subjects in Research at the University of Massachusetts Medical School. Physiological recordings of six preterm infants were obtained from the University of Massachusetts Memorial Neonatal Intensive Care Unit. These

J.R. Williamson and D.W. Browne are with the Massachusetts Institute of Technology, Lincoln Laboratory, Lexington, MA.
E-mail: jrw@ll.mit.edu

D.W. Bliss is with the School of Electrical, Computer and Energy Engineering at Arizona State University, Glendale, AZ.

P. Indic, E. Bloch-Salisbury, and D. Paydarfar are with the Department of Neurology, University of Massachusetts Medical School, Worcester, MA. D. Paydarfar is also with the Wyss Institute for Biologically Inspired Engineering, Harvard University, Boston, MA.

This work is sponsored by Assistant Secretary of Defense for Research and Engineering under Air Force Contract FA8721-05-C-0002. Opinions, interpretations, conclusions, and recommendations are those of the author and are not necessarily endorsed by the United States Government.

978-1-4799-0330-6/13/$31.00 ©2013 IEEE

Authorized licensed use limited to: CSIRO Information Technology Services. Downloaded on September 09,2022 at 00:23:03 UTC from IEEE Xplore. Restrictions apply.

TABLE I
PATIENT INFORMATION.

Pat. no.	Total record min.	Eligible apnea no.	Birth age (wks)	Study age (wks)	Birth weight (kg)	Study weight (kg)
1	387	5	27.3	32.0	1.07	1.48
2	313	4	31.0	32.1	1.50	1.30
3	395	10	29.6	32.1	1.42	1.47
4	376	6	29.4	35.0	1.30	1.71
5	289	6	25.4	32.3	.78	1.27
6	255	3	29.0	33.1	1.39	1.77

recordings were made as part of a larger study examining interventions to help reduce apnea in premature infants. Multiple channels of physiological measurements were recorded over a time period of 5-8 hours for each patient. Table I lists many pertinent details, including the total amount of data analyzed per patient, as well as the total number of apneas that were eligible for prediction. The apnea labeling criteria are described in Section IIB.

Respiratory signals were obtained using abdominal inductance plethysmography (Somonstar PT, Viasys healthcare, Yorbalinda, CA), and cardiac signals were obtained using an electrocardiograph (ECG). Blood oxygen saturation (SpO_2), as well as a pulse plethysmogram signal, were obtained using a pulse oximeter attached to the infants foot or wrist. These measurements were acquired using the Embla N7000 recording system (Embla, Denver, CO). Interbreath intervals (IBIs) were extracted from abdominal respiratory movements, and heartbeat intervals (RRIs) were extracted from the ECG signal. The quality of the signals, particularly the respiratory and pulse plethysmogram signals, is adversely affected by gross body movements, which are typically present in about one quarter of recording times [12]. Despite these movement effects, the respiratory and ECG signals were always used when they were both available. Out of a total of 2,030 minutes of recorded data across all 6 patients, only 15 minutes were discarded due to the unavailability of either abdominal or ECG sensor data. Physically implausible IBI and RRI values were automatically removed, and the remaining values were then resampled at 10 Hz using shape-preserving piecewise cubic interpolation. The signals were then log-transformed and converted to standard units (zero mean, unit variance) for each patient. The log transformation makes the IBI and RRI signals approximately normally distributed, and thus well described by second order statistics. This forms an appropriate basis for our cardiorespiratory feature extraction approach, which is described in Section IIC.

B. Problem definition

Severe apneas are typically classified as breathing pauses of 10s or more, followed shortly by bradycardia and hypoxemia. The automatic labeling of apneas in this study was based on co-occurrences of bradycardia and hypoxemia, which are the conditions of primary clinical concern due to their possible effects on infant development; significant bradycardia and hypoxemia episodes could result either from a single long breathing pause or a sequence of several short breathing pauses. Thresholds for detecting bradycardia and hypoxemia (HR$<$0.7 of a time-varying baseline, and $SpO_2 < 88\%$) were adopted from [16]. If both of these conditions were met, then an apnea period was defined as the time interval beginning 20s before the first threshold crossing of HR or SpO_2, and lasting until both signals went back above threshold. The presence of apneic breathing pauses prior to the labeled bradycardia and hypoxemia events was confirmed using visual inspection of the respiratory signal. An example from Patient 6, in which a single apnea event is labeled within a 20-minute segment, is shown in Figure 1 (top).

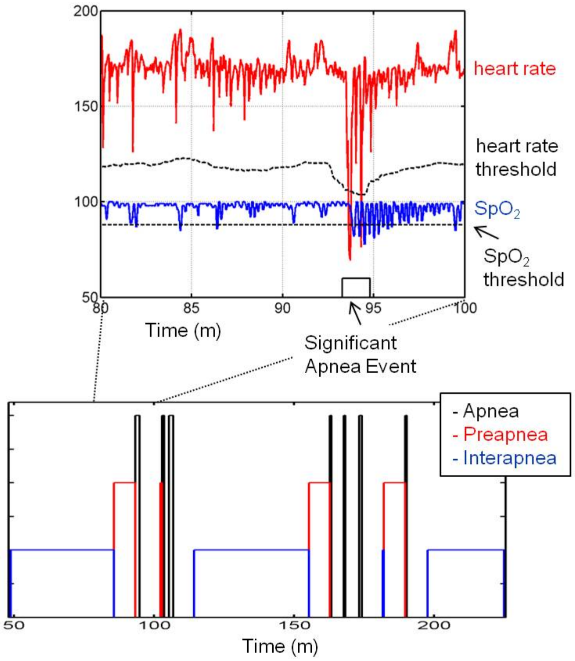

Fig. 1. Annotation of significant apneas based on co-occurence of bradycardia and hypoxemia (top). Labeling of apnea, preapnea, and interapnea periods (bottom).

The labeled apneas are often clustered in time. Within each cluster, subsequent apneas after the first one should be easier to predict based on the context of previously detected apneas. As a result, the primary clinical benefit would derive from accurate prediction of the first apnea in each cluster. This motivates our formulation of the problem as one of detecting *preapnea* periods, defined as those periods preceding apneas that are themselves apnea-free. The periods immediately following apneas, *postapnea* periods, were also excluded from prediction analysis due to our finding that the IBI and RRI values during these periods were more highly variable than normal. All the remaining time periods are labeled as *interapnea* periods. The apnea prediction algorithm is evaluated based on its ability to distinguish preapnea from interapnea periods.

Figure 1 (bottom) illustrates how the periods are defined over a 176 minute data segment that contains multiple apneas, including the single apnea that is shown above. The apnea

Authorized licensed use limited to: CSIRO Information Technology Services. Downloaded on September 09,2022 at 00:23:03 UTC from IEEE Xplore. Restrictions apply.

periods are shown in black, the preapnea periods in red, and the interapnea periods in blue. The specific criteria for labeling these periods are as follows. Preapnea periods are the 7.5 minutes immediately preceding an apnea, unless precluded by a postapnea period. A postapnea period is the 7.5 minutes immediately succeeding an apnea, unless precluded by a different apnea. Interapnea periods are all the remaining times in the data record. Only three of the seven apneas shown are preceded by a sufficiently long preapnea period (about 3 minutes) to be eligible for prediction (see Section IIE). The ranges of maximum IBIs that occurred during the eligible apnea periods were as follows: $6s-11s$ for patient 1, $8s-28s$ for patient 2, $6s-22s$ for patient 3, $8s-15s$ for patient 4, $5s-15s$ for patient 5, and $5s-16s$ for patient 6.

C. Cardio-respiratory features

Feature vectors used in this analysis are constructed from interbreath intervals (IBIs) and heartbeat intervals (RRIs), due to their known correlation with AOP [17],[18],[19]. A multivariate feature construction approach is used, which characterizes the structure of correlations both within and between the IBI and RRI signals. This feature construction approach is also applied to the RRI signals alone. This was done to obtain accurate estimates of cardiac correlation structure while avoiding the effects of movement-based contamination of the respiratory signals. The multivariate feature construction approach was first developed for analysis of EEG signals for seizure prediction [13], [14], and was extended to the analysis of cardio-respiratory signals for apnea prediction in [15].

In this approach, *channel-delay* correlation and covariance matrices are computed from the different modality time series. These are matrices containing correlation or covariance coefficients between time series from multiple measurement modalities at multiple relative time delays. The approach is motivated by the observation that auto- and cross-correlations of measured signals can reveal hidden parameters in the stochastic-dynamical systems that generate the signals. Changes over time in the eigenvalue spectra of these channel-delay matrices register changes in coupling strengths among the measurement modalities.

For example, Figure 2 (top) shows the IBI and RRI signals within a single 60s frame of interapnea data. Figure 2 (bottom left) shows a channel-delay correlation matrix extracted in this data frame at the smallest temporal scale. Each 30×30 block along the main diagonal contains the within-channel correlations for 30 time delays, whereas the off-diagonal blocks contain the cross-channel correlations. Figure 2 (bottom right) plots the eigenvalue distribution of the channel-delay matrix, with the eigenvalues rank-ordered from largest to smallest. The eigenvalue distribution encodes the shape of the covariance distribution; that is, the magnitude of covariance in each dimension under the change of basis that decorrelates the dimensions. Figure 3 shows the features extracted in a later data frame from the same patient, which occurs during a preapnea period. The preapnea and interapnea eigenvalue distributions are plotted in red and blue respectively (Figure 3, lower right). The differences between these eigenvalue distributions suggests that they may provide an effective basis for discrimination.

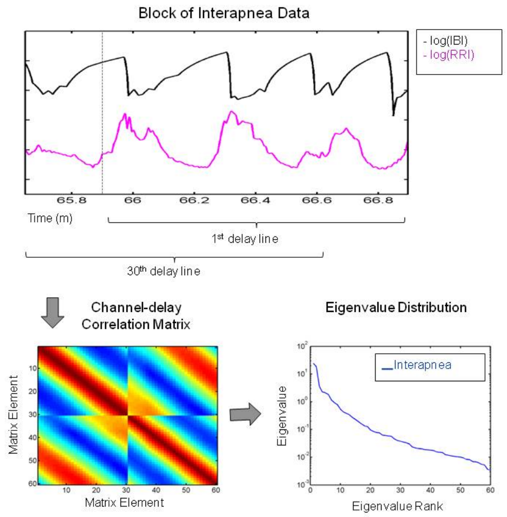

Fig. 2. Single 60s frame of IBI and RRI signals during interapnea period (top). Channel-delay correlation matrix and resulting eigenvalue distribution (bottom).

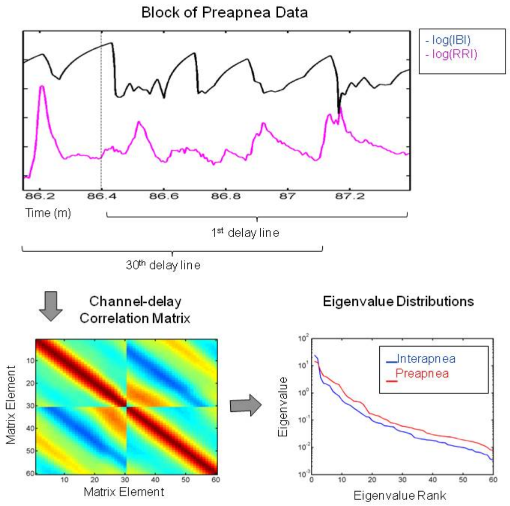

Fig. 3. Single 60s frame of IBI and RRI signals during preapnea period (top). Channel-delay correlation matrix and resulting eigenvalue distribution, showing comparison with eigenvalue distribution from interapnea period (bottom).

Across the five delay scales, a total of 125 single-channel (RRI) or 240 multichannel (IBI & RRI) features are extracted from the jth data frame. These features are highly correlated. The final stage of feature extraction is dimensionality reduction using principal component analysis into a smaller set of uncorrelated features containing the greatest amount of variance. A critical step is to first normalize each of the 125 or 240 features into standard units (zero mean unit variance), which allows the variation of each feature to be considered relative to its baseline variation. The top n_p principal components are

Authorized licensed use limited to: CSIRO Information Technology Services. Downloaded on September 09,2022 at 00:23:03 UTC from IEEE Xplore. Restrictions apply.

used as the cardiorespiratory features for machine learning and apnea prediction, as described in Section IIE. The number of components, $n_p = 6$, was empirically determined.

D. Movement features

Features derived from infant movement patterns are also used as apnea predictors. Clinical studies have reported a link between infant apnea and movement [20],[21],[22]. Infant movements could be predictive either for causal reasons – for example, movements could trigger hyperventilation that in turn leads to hypocapnia – or for symptomatic reasons. For example, movements could occur in response to a change in the underlying physiological state that is itself the causal factor leading to apnea. Movement estimation is also useful in building statistical models of the joint feature distributions because it helps to explain the existence of movement artifacts in other measurement modalities. For example, one of the complications in interpreting our previous prediction results on the same data set [15] are the presence of movement artifacts in the IBI estimates. The conflation of movement with breathing signals makes it unclear if the respiratory channel's predictive value is primarily due to the information it contains about breathing dynamics or about movement patterns.

In order to better understand the predictive value of movement signals, and to improve our apnea prediction capability, we therefore add an independent movement feature channel based on readily available physiological sensor measurements. The movement channel uses the pulse plethysmogram (PPG) signal, which is provided by a pulse oximeter attached to each infant's foot or wrist. In addition to a pulse waveform, the PPG signal also contains power in the low frequency bands during infant movement. We make use of these movement artifacts to produce a PPG-derived movement signal, based on the power in a low frequency band relative to the total power in the PPG signal. A ratio-based calculation is warranted due to the high variability of total power in the PPG waveform. An example PPG signal is shown in Figure 4 (blue), with the corresponding PPG-derived movement signal also shown (red), over a 120 second time interval. The low-frequency movement components in the PPG signal, which are visually apparent at about 40s, are registered by increases in the movement signal.

Three statistical features describing the distribution of the movement values, m, are used as input to the machine learning algorithm. These features are the local mean (μ_m) and standard deviation (σ_m) of the movement signal, as well as the ratio of these quantities (coefficient of variation), with an additional denominator term that attenuates the ratio when the mean is small, ($\frac{\sigma_m}{.001+\mu_m}$). These features are computed based on eight movement values, which are obtained at 7.5-second intervals within each 60-second data frame.

E. Machine learning

The feature vectors used for machine learning and apnea prediction are 9-dimensional, consisting of the six principal component cardio-respiratory features described in Section IIC and the three movement features described in Section IID.

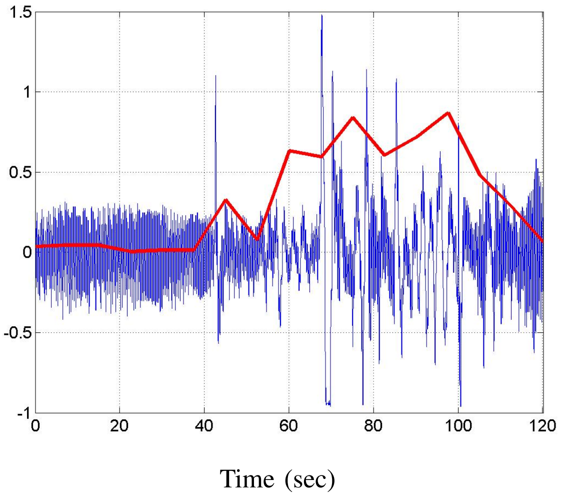

Fig. 4. PPG signal over a 120 second interval (blue), and PPG-derived movement amplitude signal (red). PPG signal is in standard units (zero mean, unit variance). Movement signal ranges between zero and one.

These feature vectors are computed from 60-second data frames at 10-second intervals.

In our previously published study, the patient-specific preapnea and interapnea feature distributions were each modeled with a Gaussian distribution [15]. We have obtained improved prediction performance by adapting a machine learning approach that has been widely used in automatic speaker recognition. In that problem domain, the approach is to form a statistical background model from all the speakers in a database, and then to form a model tuned to a particular speaker using Bayesian adaptation from the background model [23]. The statistical models in question are Gaussian mixture models (GMMs), which are weighted combinations of multiple Gaussian densities. In our adaptation of this approach, we train a separate background GMM that is individualized to each patient. This GMM encodes the feature densities arising from all of the patient's encountered physiological states. Bayesian adaptation is used to form both a preapnea GMM and an interapnea GMM from this background model, using training data from the patient's preapnea and interapnea periods [23].

The adapted GMM models are evaluated on each patient using 40-fold cross-validation, in which the nearest training data to each test segment is separated by at least two minutes. To obtain robustness to small data sets, the likelihoods from ten independently trained GMMs are combined. Specifically, ten different background GMMs are obtained using independent random initializations, resulting in the adaptation of ten preapnea and interapnea GMMs. The single-frame prediction score is then the 2-class log-likelihood ratio obtained from the log of the sum of the ten preapnea GMM likelihoods minus the log of the sum of the ten interapnea GMM likelihoods. Multi-frame prediction scores are obtained by adding these preapnea/interapnea log-likelihood ratios over time. This is done using the maximum cumulative sum statistic [13],[15] over a time interval of 2 minutes and 50 seconds. Figure 5

Authorized licensed use limited to: CSIRO Information Technology Services. Downloaded on September 09,2022 at 00:23:03 UTC from IEEE Xplore. Restrictions apply.

(top) shows the multiframe prediction score computed in the first 180 minutes of the data record from Patient 1, plotted with a solid blue line during interapnea periods and with a solid red line during preapnea periods.

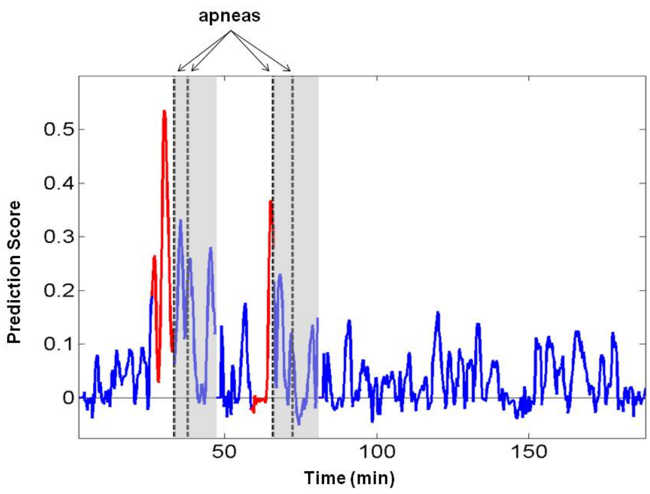

Fig. 5. The multiframe prediction score is plotted for the first 180 minutes of the data record from Patient 1. The score is plotted in blue during the interapnea periods and in red during the preapnea periods. The labeled apneas are shown with vertical dotted lines, and the postapnea periods are shaded in grey.

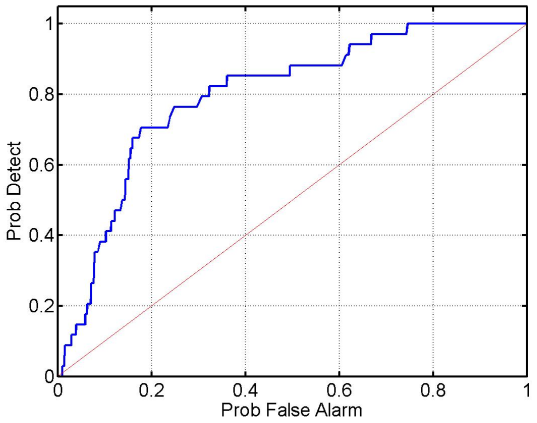

Fig. 6. The ROC curve is shown from the prediction scores of all six patients together. The area under the ROC curve is .80

III. Results

Apnea predictions are admissable for evaluation only after 12 data frames are observed, which is the maximum number of single-frame scores that are combined to create a multiframe prediction score. Therefore, an apnea is eligible for prediction only if it occurs 2 minutes and 50 seconds after the start of a data record, or 10 minutes and 20 seconds after the end of a previous apnea.

With this caveat in mind, the effectiveness of the apnea prediction scores is computed as follows. The probability of detection, P_D, is the fraction of eligible apneas that are predicted within the prediction time window, given a prediction threshold. The probability of false alarm, P_{FA}, is the number of interapnea frames in which the prediction window is triggered divided by the total number of interapnea frames. Therefore, increasing (decreasing) the duration of the prediction window tends to increase (decrease) both P_D and P_{FA}. In this study the prediction time window was kept fixed at 5.5 minutes and a receiver operating characteristic (ROC) curve was obtained for each patient by varying the prediction threshold. The area under the ROC curve (AUC) serves as the evaluation metric. Figure 6 shows an ROC curve computed from all six patients, which results in AUC=.80.

To test for statistical signficance, the algorithm's AUC values are compared to the AUC values that are obtained using random surrogates [24],[15], in which the single-frame 9-dimensional feature vectors are kept the same, but the apnea clusters are shuffled in time, subject to the following constraints. All successive apneas that are separated by less than 9.5 minutes are assigned to the same apnea cluster; otherwise they are assigned to different clusters. In surrogate apnea profiles, the within-cluster inter-apnea time distances are kept constant. The between-cluster distances are randomly varied, provided that all clusters remain at least 9.5 minutes apart. 500 surrogate apnea profiles are generated for each patient, with machine learning, temporal integration, and prediction evaluation done independently for each of the surrogate profiles. One-sided p values are computed by comparing the algorithm's AUC scores obtained on the real data with the distribution of AUC scores that are obtained using random surrogates.

Table II summarizes the apnea prediction results obtained on all six patients, either individually (rows one through six), or as a group (bottom row). Columns one and two show the results obtained using the two feature combinations that were tried: RRI features plus Movement features (left column), and joint RRI-IBI features plus Movement features (middle column). The right column shows the results when the GMM likelihoods from these two feature combinations are summed prior to computing the single-frame log-likelihood ratio. Combining both classifiers in this way produces the best results overall, with significance ($p < .05$) obtained on five out of six patients, and the highest net AUC value (AUC=.80; see Figure 6). Additional evaluations on larger data sets are needed to confirm the reliability of this improvement from combining classifiers.

These results are greatly improved over a previous version of our algorithm [15]. These improvements are attributable in roughly equal measure to two factors: the addition of a PPG-derived movement signal, and the replacement of a Gaussian classifier with a GMM classifier. It is notable that, with the current algorithm, the respiratory channel provides only marginal benefits. It appears likely that a large portion of the respiratory channel's contribution to apnea prediction in the previous study was due to the information it provided about infant movement, since the IBI estimates were impacted by movement artifacts.

Authorized licensed use limited to: CSIRO Information Technology Services. Downloaded on September 09,2022 at 00:23:03 UTC from IEEE Xplore. Restrictions apply.

TABLE II
APNEA PREDICTION PERFORMANCE.

Pat no.	RRI, Mvmnt AUC (p)	RRI-IBI,Mvmnt AUC (p)	Combined AUC (p)
1	.90 (.02)	.89 (.00)	.95 (.00)
2	.78 (.02)	.50 (.48)	.82 (.01)
3	.80 (.02)	.80 (.01)	.80 (.01)
4	.62 (.27)	.67 (.17)	.65 (.19)
5	.88 (.01)	.85 (.01)	.88 (.01)
6	.69 (.39)	.91 (.08)	.94 (.04)
1-6	.77 (.00)	.75 (.00)	.80 (.00)

IV. CONCLUSION

The ability to predict severe apneas in preterm infants may be clinically beneficial if used in conjunction with preventative interventions, such as Therapeutic Stochastic Resonance (TSR). The current study explored the predictive value of features characterizing changes in the correlation patterns of breathing rate and heart rate, along with changes in movement patterns. The key technical innovations introduced in this paper require a more detailed exposition. Complete algorithmic details will be provided in a forthcoming journal article.

Strong prediction accuracy was obtained, with statistical significance found on five out of six patients individually ($p < .05$), and with strong prediction results on all six patients collectively, as summarized in Table II and Figure 6. We will next evaluate the apnea prediction algorithm on larger data sets, containing long-term continuous data recordings from each patient. This evaluation will be useful for validating the current algorithm, and for developing possible improvements to it. Our subsequent goal is to implement the prediction algorithm in a closed-loop clinical system in the neonatal intensive care unit, in which the algorithm's apnea predictions are used to trigger TSR stimulation using an instrumented mattress.

REFERENCES

[1] (U.S.) NC for HS. Public Use Data Tapes from the National Center for Health Statistics Set: 1992-2002. U.S. Department of Health and Human Services, Public Health Service, Centers for Disease Control, National Center for Health Statistics; 2005.

[2] C.F. Poets. Interventions for apnoea of prematurity: a personal view. *Actal Paediatrica*, 2010; 99(2):172-177. PMID 19958303.

[3] C.F. Poets. Apnea of prematurity: What can observational studies tell us about pathophysiology? *Sleep Med.*, 2010 Aug;11(7):701707. PMID: 20621558.

[4] J. Zhao, F. Gonzalez, D. Mu. Apnea of prematurity: from cause to treatment. *Eur J Pediatr*, 2011 Sep;170(9):10971105. PMID: 21301866.

[5] O.P. Mathew. Apnea of prematurity: pathogenesis and management strategies. *J Perinatol*, 2011 May;31(5):302310. PMID: 21127467.

[6] R.J. Martin, K. Wang, . Krolu, J. Di Fiore, P. Kc. Intermittent Hypoxic Episodes in Preterm Infants: Do They Matter? *Neonatology* 2011;100(3):303310.

[7] J.M. Di Fiore, J.N. Bloom, F. Orge, A. Schutt, M. Schluchter, V.K. Cheruvu, M. Walsh, N. Finer, R.J. Martin. A higher incidence of intermittent hypoxemic episodes is associated with severe retinopathy of prematurity. *J Pediatr*, 2010 Jul;157(1):6973. PMID: 20304417.

[8] J. Kattwinkel, H.S. Nearman, A.A. Fanaroff, P.G. Katona, M.H. Klaus. Apnea of prematurity. Comparative therapeutic effects of cutaneous stimulation and nasal continuous positive airway pressure. *J. Pediatr.*, 1975 Apr;86(4):588592. PMID: 1092821.

[9] B. Schmidt, R.S. Roberts, P. Davis, L.W. Doyle, K.J. Barrington, A. Ohlsson, A. Solimano, W. Tin. Long-term effects of caffeine therapy for apnea of prematurity. *New England Journal of Medicine*, 2007;357(19):18931902.

[10] B. Schmidt, P.J. Anderson, L.W. Doyle, D. Dewey, R.E. Grunau, E.V. Asztalos, P.G. Davis, W. Tin, D. Moddemann, A. Solimano, A. Ohlsson, K.J. Barrington, R.S. Roberts. Survival without disability to age 5 years after neonatal caffeine therapy for apnea of prematurity. *JAMA*, 2012 Jan;307(3):275282. PMID: 22253394.

[11] R.J. Martin, C.G. Wilson. What to do about apnea of prematurity? *J Appl Physiol*, 2009 Oct;107(4):1015 1016.

[12] E. Bloch-Salisbury, P. Indic, F. Bednarek, D. Paydarfar. Stabilizing immature breathing patterns of preterm infants using stochastic mechanosensory stimulation. *J Appl Physiol*, 2009 Oct;107(4):10171027.
Jan;59(1):412. PMID: 16287079.

[13] J.R. Williamson, D.W. Bliss, D.W. Browne. Epileptic seizure prediction using the spatiotemporal correlation structure of intracranial EEG. *IEEE International Conference on Acoustics, Speech and Signal Processing*, 2011:665-668.

[14] J.R. Williamson, D.W. Bliss, D.W. Browne, J.T. Narayanan. Seizure prediction using EEG spatiotemporal correlation structure. *Epilepsy & Behavior*, 2012; 25:230-238.

[15] J.R. Williamson, D.W. Bliss, D.W. Browne, P. Indic, E. Bloch-Salisbury, D. Paydarfar. Using physiological signals to predict apnea in preterm infants. *In: Signals, Systems and Computers (ASILOMAR), 2011 Conference Record of the Forty Fifth Asilomar Conference.* 2011 p. 1098 1102.

[16] C. Sreenan, R.P. Lemke, A. Hudson-Mason, and H. Osiovich. High-flow nasal cannulae in the management of apnea of prematurity: a comparison with conventional nasal continuous positive airway pressure. *Pediatrics*, 2001, 1081-1083.

[17] U. Frey, M. Silverman, A.L. Barabasi, B. Suki. Irregularities and power law distributions in the breathing pattern in preterm and term infants. *Journal of Applied Physiology*, 1998; 85:789-797.

[18] T.B. Waggener, I.D. Frantz III, A.R. Stark, R.E. Kronauer. Oscillatory breathing patterns leading to apneic spells in infants. *Journal of Applied Physiology*, 1982; 52:1288-1295.

[19] J.A. Henslee, V.L. Schechtman, R.M. Harper. Developmental patterns of heart rate and variability in prematurely-born infants with apnea of prematurity. *Early Human Development*, 1997; 47:35-50.

[20] Y.K. Abu-Osba, R.T. Brouillette, S.L. Wilson, B.T. Thach. Breathing pattern and transcutaneous oxygen tension during motor activity in preterm infants. *Am Rev Respir Dis*, 1982;125:382387.

[21] O.P. Mathew, C-K. Thoppil, M. Belan. Motor activity and apnea in preterm infants. Is there a causal relationship? *Am Rev Respir Dis*, 1991;144:842844.

[22] M. Fukumizu, J. Kohyama. Central respiratory pauses, sighs, and gross body movements during sleep in children. *Physiol Behav*, 2004;82:721726.

[23] D.A. Reynolds, T.F. Quatieri, R.B. Dunn. Speaker verification using adapted Gaussian mixture models. *Digital Signal Processing*, 2000 Jan;10(13):1941.

[24] R.G. Andrzejak, F. Mormann, T. Kreuz, C. Ricke, A. Kraskov, C.E. Elger, and K. Lehnertz. Testing the null hypothesis of the nonexistence of a preseizure state. *Physical Review E*, 2003, 67:010901.

Authorized licensed use limited to: CSIRO Information Technology Services. Downloaded on September 09,2022 at 00:23:03 UTC from IEEE Xplore. Restrictions apply.
