# Supplementary material for: Exploring Computational Techniques in Preprocessing Neonatal Physiological Signals for Detecting Adverse Outcomes: Scoping Review
Source: Interact J Med Res. 2024 Aug 20;13:e46946. doi: 10.2196/46946 (PMC11372324; doi:10.2196/46946)
Supplement: Multimedia Appendix 3 [file ijmr_v13i1e46946_app3.zip › Included Papers - Final/3520/Sullivan et al. - 2018 - Early Pulse Oximetry Data Improves Prediction of D.pdf]

HHS Public Access
Author manuscript
Am J Perinatol. Author manuscript; available in PMC 2019 November 01.

Published in final edited form as:
Am J Perinatol. 2018 November ; 35(13): 1331–1338. doi:10.1055/s-0038-1654712.

Early pulse oximetry data improves prediction of death and adverse outcomes in a two-center cohort of very low birth weight infants

B.A. Sullivan[1], **A. Wallman-Stokes**[2], **J. Isler**[2], **R Sahni**[2], **J.R. Moorman**[3], **K.D. Fairchild**[1], and **D.E. Lake**[3]

[1.]Department of Pediatrics, University of Virginia, Charlottesville, VA

[2.]Department of Pediatrics, Columbia University, New York, NY

[3.]Department of Medicine, University of Virginia, Charlottesville, VA

Abstract

Background: We previously showed, in a single-center study, that early heart rate (HR) characteristics predicted later adverse outcomes in very low birthweight (VLBW) infants. We sought to improve predictive models by adding oxygenation data and testing in a second NICU.

Methods: HR and SpO_2 from the first 12 hours and first 7 days after birth were analyzed for 778 VLBW infants at two NICUs. Using multivariate logistic regression, clinical predictive scores were developed for death, severe intraventricular hemorrhage (sIVH), bronchopulmonary dysplasia (BPD), treated retinopathy of prematurity (tROP), late-onset septicemia (LOS) and necrotizing enterocolitis (NEC). Ten HR-SpO_2 measures were analyzed, with first 12-hour data used for predicting death or sIVH and first 7 days for the other outcomes. HR-SpO_2 models were combined with clinical models to develop a Pulse Oximetry Predictive Score (POPS). Net reclassification improvement (NRI) compared performance of POPS to the clinical predictive score.

Results: Models using clinical or pulse oximetry variables alone performed well for each outcome. POPS performed better than clinical variables for predicting death, sIVH and BPD (NRI>0.5, p<0.01), but not tROP, LOS or NEC.

Conclusion: Analysis of early HR-SpO_2 characteristics adds to clinical risk factors to predict later adverse outcomes in VLBW infants.

Introduction

Identifying very low birth weight (VLBW) infants at increased risk of death and adverse outcomes is important for clinical research, resource allocation, and benchmarking across NICUs. While demographic and perinatal clinical variables are often used to predict adverse

Corresponding Author: Brynne A. Sullivan, MD, Assistant Professor, Department of Pediatrics, University of Virginia, PO Box 800386, Charlottesville, VA 22908-0386, bsa4m@virginia.edu, Phone: (434) 924-5428, Fax: (434) 924-2816.

Conflicts of Interest: The authors declare no conflicts of interest.

Author Manuscript Author Manuscript Author Manuscript Author Manuscript

Author Manuscript Author Manuscript Author Manuscript Author Manuscript

outcomes, physiologic measures of illness severity are likely to improve risk stratification.[1,23]

Previously, we developed a risk score in a single-center study using heart rate characteristics (HRC) in the first day and first week after birth to predict death and multiple short-term adverse outcomes related to prematurity.[1] While the HRC index was developed to predict risk of imminent sepsis,[4] early HRC added information to gestational age about risk of later sepsis as well as death, severe IVH and BPD at 36 weeks post-menstrual age (PMA). We then showed, in a two-center study, that adding oxygen saturation (SpO_2) analysis to HRC improved algorithms for predicting imminent sepsis and NEC.[5] The study analyzed multiple vital signs and their continuous interaction in over 1,000 VLBW infants, the cross-correlation of HR and SpO_2 was the best single predictor in 186 cases of imminent LOS and NEC. The model with the best predictive performance combined multiple measures of HR and SpO_2 (mean SpO_2, standard deviation of HR and cross-correlation of HR-SpO_2).

For the current study, we hypothesized that analysis of HR and SpO_2 using pulse oximetry data from the first week after birth could improve prediction of later adverse outcomes when compared to analysis of clinical variables alone. This is a fundamentally different concept than using such data for early detection of deterioration. Rather, the goal is to identify infants at high risk of morbidity later in their course in order to direct care towards mitigating or preventing disease. Thus, the objectives of this study were to develop and validate multivariable predictive models that combine pulse oximetry data and clinical data to predict risk of death and other adverse neonatal outcomes in a two-center cohort of VLBW infants.

Methods

Patient population

The Institutional Review Boards at the University of Virginia (UVA) and Columbia University (CU) approved this study with waiver of consent. We included all VLBW (< 1500g at birth) infants admitted to the UVA NICU from 2009–2015 and to the CU NICU from 2012–2015 with at least one hour of pulse oximetry data available within 12 hours of birth. Infants with cyanotic congenital heart defects and infants provided comfort care only from birth were excluded.

Medical records were reviewed to determine the occurrence of death prior to NICU discharge, sIVH (grades III-IV), LOS (clinical sepsis after 3 days of age with a positive blood culture and at least 5 days antibiotics), NEC (Bell's stage 2–3), BPD (receiving supplemental oxygen at 36 weeks' postmenstrual age) and severe retinopathy of prematurity treated with laser or bevacizumab therapy (tROP).

Pulse oximetry monitoring

Heart rate (HR) and oxygen saturation (SpO_2) data were collected every 2 seconds (0.5 Hz) from all infants using Masimo pulse oximeters (Masimo Corporation, Irvine, CA) with 8 second SpO2 averaging time. These devices use signal extraction technology for high accuracy during patient movement and low perfusion by separating the pulsatile arterial

signal from other signals created by motion.[6] Data at both centers are continuously stored for review using BedMasterEx (Excel Medical, Jupiter, FL) data transfer system.

Clinical predictive score

Baseline risk of adverse outcomes was analyzed by developing a Clinical Predictive Score from variables available at the time of birth that are known to impact risk of adverse outcomes. Clinical data included in the score were birth weight (BW), gestational age (GA), sex, antenatal steroid exposure (at least 1 dose) and 1 and 5 minute Apgar scores. Differences among sites were also considered by including a binary variable for NICU 1 versus NICU 2. Each outcome was used to fit a multivariate logistic regression model including these variables.

All available pulse oximetry data from both the first 12 hours and first 7 days after birth were used to calculate the mean, standard deviation, kurtosis and skewness of HR and SpO_2 and the maximum and minimum cross correlation of HR and SpO_2 over each of these time periods. At least 6 hours of data within 12 hours of birth were required to be included in the analysis.

Pulse oximetry measures used in the models were chosen as descriptive summary statistics for a time series data set. Mean, standard deviation, skewness and kurtosis all describe the shape of the distribution of HR and SpO_2 values, sampled every 2 seconds, during the defined time periods. Skewness is a measure of symmetry of a data set and kurtosis is a measure of the shape of a data distribution compared to a normal distribution. The kurtosis of a normal distribution is 3, and variables with kurtosis higher than this will have more extreme outliers.

The cross-correlation of two variables is a measure of the degree to which they trend together, either in the same or opposite directions, within a set lag time of each other. For this study, cross-correlation of HR and SpO_2 was calculated over 10-min windows using the Matlab function XCORR with a lag time of −30 to +30 seconds.[5] A high cross-correlation indicates the two signals are in positive synchrony and a negative or low cross-correlation indicates the two signals travel in synchrony in opposite directions. We used the maximum and minimum cross-correlation HR- SpO_2 values in the first 12 hours or 7 days after birth in the multivariate models for each outcome.

A predictive score using pulse oximetry measures alone was developed for each outcome to fit a multivariate logistic regression model. Measures from the first 12 hours after birth were used in the models to predict risk of death and sIVH while data from the first 7 days after birth were used in the models for risk of LOS, NEC, BPD and tROP. These time frames were chosen based on pathophysiology of the targeted outcomes and testing of multiple time periods to optimize the predictive model. Other time periods tested were 3 and 24 hours and 3 days after birth.

Pulse oximetry predictive score (POPS)

POPS was calculated for each outcome for each infant using a bivariate logistic regression model to combine the model using pulse oximetry measures alone with the clinical

Author Manuscript Author Manuscript Author Manuscript Author Manuscript

Author Manuscript Author Manuscript Author Manuscript Author Manuscript

predictive score. The individual models were converted to units of log-odds with the logit function prior to training the model. To ensure high quality results, we used the Transparent Reporting of a multivariable prediction model for Individual Prognosis or Diagnosis (TRIPOD) statement checklist for our approach to analyzing and reporting this study.[7]

Validation

Bootstrapping was used to calculate confidence intervals and p-values of the area under the receiver operating characteristic curve (AUC) for all predictive models (TRIPOD type 1b prediction model study). In addition, models were validated by training models on each NICU and externally validating on the other NICU (TRIPOD type 3 prediction model study). Bootstrapping was used here as well to calculate standard errors and confidence intervals for AUC estimates and calculate p-values for test of whether performance differed between sites.

Statistical analysis

The AUC of each model was calculated to determine the predictive performance of the clinical predictive score, pulse oximetry score and POPS for each outcome. The p-values on the coefficient were used to test the hypothesis of significance of each model when corrected for the other and to quantify added value.

The added value of POPS over the predictive model using clinical variables alone was also quantified by calculating a net reclassification improvement (NRI),[8] a measure of the sum of proportions of patients reclassified into the correct risk group by one risk marker compared to another. NRI serves to quantify the relative accuracy of a new test or marker in comparison with an established test or marker. For this study we used a continuous NRI calculation method since the predictive scores compared are not categorical.[9] Statistical analyses were performed in MATLAB (MathWorks, INC, Natick, MA) and in GraphPad Prism 7 (GraphPad Software, Inc., San Diego, CA) with two-tailed p-values <0.05 considered significant.

Results

Patient Population

Of 1,023 inborn VLBW infants, 778 met inclusion criteria (Table 1). Of those excluded, 44 had congenital anomalies or received comfort care only and 201 had insufficient pulse oximetry data available. The rates of each outcome were compared between the two NICUs using Fisher's exact test and were significantly different for BPD, tROP and NEC but not for death, sIVH and LOS (Table 1). The average age at death was 31 ± 19 days and age at diagnosis of LOS or NEC was 28 ± 14 days. Mean gestational age was significantly higher by 0.5 weeks for infants at NICU 2, but there was no significant difference in birthweight. Twenty percent of infants in the combined cohort were SGA (24% at NICU 2 and 18% at NICU 1) as determined using the tenth percentile of Fenton growth charts for premature male and female infants. Maternal race was predominantly Caucasian in the NICU 1 cohort (65%) while NICU 2 mothers were more ethnically and racially diverse. The mode of

Author Manuscript Author Manuscript Author Manuscript Author Manuscript

delivery was by caesarean section for 65% of infants in the combined cohort with no significant difference between the two sites.

Predictive Models

For the first 12h after birth, the mean number of hours of pulse oximetry data available for analysis per infant was 10 hours, and for the first 7 days, 6 days. Pulse oximetry measures were averaged and compared for infants with or without each outcome (Table 2). The significance and relative rank of variable coefficients in the multivariate logistic regression models used to calculate the clinical and physiologic risk scores are shown in Table 3. In the model using pulse oximetry measures alone, the pulse rate standard deviation was the overall highest ranking variable of all outcomes combined, followed by HR skewness. Gestational age was highest ranking overall for the clinical variables predictive score, followed by site.

Predictive model performance

Both clinical and pulse oximetry scores modeled using the two-center cohort demonstrated high predictive performance by AUC for all outcomes (Table 4). The AUCs were highest when clinical and pulse oximetry models were combined to calculate a POPS for each outcome. POPS predicted all adverse outcomes ($p \leq 0.05$), but the predictive performance was most significant for the outcomes of death and BPD. Analysis using NRI showed that pulse oximetry data improved risk prediction over clinical data alone for death, sIVH and BPD, but not for tROP, LOS or NEC. Plotting POPS vs. the clinical predictive score (Figure 1) in cases and controls illustrates the net proportion of infants correctly reclassified as high risk or low risk by adding pulse oximetry data to the predictive model. Visually, this is represented by the proportion of cases in the left upper quadrant and of controls in the right lower quadrant of each graph. Quadrants are divided by dotted lines drawn at the score on the x and y axes above which the number of patients equals the incidence of that outcome.

Cross-validation

We validated POPS using NICU 1 as a training data set and NICU 2 as the test data set and then reversed the process. Performance was similar between test and training site when using NICU 1 as the development cohort and NICU 2 as the validation cohort. However, performance varied for several outcomes with NICU 2 set as the development cohort and NICU 1 as the validation cohort (Table 5).

Discussion

In this two-center study of VLBW infants, we found that analysis of early cardiorespiratory pulse oximetry data adds information to clinical data for prediction of death and adverse outcomes, particularly severe IVH and BPD. We developed a Pulse Oximetry Predictive Score (POPS) that uses analysis of HR and SpO_2 in addition to several clinical variables known to be associated with adverse outcomes and available at the time of birth. While POPS performed well for predicting late-onset sepsis, necrotizing enterocolitis and severe retinopathy of prematurity, our analysis suggests the most important variables in the first week for predicting these outcomes are demographic. We found that site affected model

Author Manuscript Author Manuscript Author Manuscript Author Manuscript

performance, but to a relatively small degree, even though the sites had very different rates of the outcome events.

One major premise for our approach is that abnormal cardiorespiratory dynamics early after VLBW birth denote vulnerability to subsequent adverse outcomes. Established scores such as the Apgar score, PhysiScore [2] and Score for Neonatal Acute Physiology (SNAP)[3], which are measured in the first 10 minutes, 3 and 24 hours, respectively, after birth, identify infants at increased risk of death and morbidity. All take into account, with different degrees of mathematical sophistication, cardiorespiratory status. A different kind of predictive measure, the heart rate characteristics (HRC) index, was developed by our group and is a continuously updated risk of sepsis based on the finding of decreased heart rate variability and transient heart rate decelerations. The average first-day HRC index also reports on risk of death and IVH and average first-week value on risk of later sepsis or BPD diagnosis but not NEC or severe ROP[1].

A second premise is that evaluation of the interaction of the heart and lungs holds information about cardiorespiratory control that isolated analysis of each system lacks. This is affirmed by recent findings about changes in the cross-correlation of vital signs preceding sepsis and NEC. Fairchild, et al. recently published a study of cross-correlation of heart rate, SpO_2 and respiratory rate in >1000 VLBW infants and found that cross-correlation of HR and SpO_2 increases during the 24 hour period prior to clinical diagnosis of sepsis or NEC.[10] Some increase in cross-correlation of HR and SpO_2 is attributable to bradycardia and desaturation occurring with apnea, but it is seen even when infants are mechanically ventilated.[11]

In the current analysis, we combined these ideas; first, that cardiorespiratory analysis soon after birth can help identify patients at risk for later morbidity and mortality; and, second, that cardiorespiratory analysis should include the degree of interaction of the heart and lungs. Variables selected for POPS include measures that describe the shape of the histograms for HR and SpO_2 as well as the interaction between these two important vital signs that can both be measured by pulse oximetry alone. Our results suggest that different early HR and SpO_2 histogram phenotypes may exist for different conditions and that early cross-correlation does not add as much for distant outcomes such as BPD as it may for imminent sepsis and NEC. While maximum cross-correlation HR-SpO_2 was associated with late onset sepsis in unadjusted univariate analysis, this measure was not significant in the multivariate analysis. Standard deviation of HR had the highest ranked coefficient in the vital sign risk models for both LOS and NEC, suggesting early decreased heart rate variability is potentially an important risk marker of later acute inflammatory illness. Others have shown that early decreased heart rate variability is predictive of NEC, possibly as a marker of abnormal vagal tone, which may have a role in risk or pathogenesis of NEC.[12]

In multivariate analysis, mean SpO_2, a measure of overall oxygenation, was highly associated with death when calculated over the first 12 hours and with BPD when calculated over the first 7 days. For BPD, this relationship likely reflects more severe respiratory distress syndrome, but also warrants study as a target for interventions to improve oxygen saturation targeting during this period. While data on FiO_2 were not available for this

Author Manuscript
Author Manuscript
Author Manuscript
Author Manuscript

analysis, we assume they would not have changed the results given similar prescribed oxygen saturation targets in the two units. Still, future studies should evaluate this assumption as more units acquire capability to collect and analyze FiO_2 data. High SpO_2 variability as measured by SD SpO_2 was also associated with risk of BPD and ranked highly in the multivariate vital sign risk model, which likely indicates that swings from hypoxemia to hyperoxemia are important as well. Our analysis did not consider position of the pulse oximetry sensor relative to the ductus arteriosus or its patency. We assumed that variation in sensor position was not related to outcome and if anything would decrease the strength of the association between SpO_2 patterns and outcomes. Measures of oxygenation did not add information to severe ROP prediction, despite their implication in the pathophysiology of this condition. In this and our previous study using early HRC to predict later outcomes, gestational age and birthweight were so highly correlated with severe ROP that models using these variables alone left little room for improvement from physiologic data.

There were several differences between patient populations at the institutions involved in this study. Most notably, there were large differences in rates of BPD and treated severe ROP. These differences likely reflect dissimilarities in population demographics, variations in practice and illness severity. However, the performance of POPS developed at either center and tested at the other was not dramatically different. At both centers, pulse oximetry data analysis added significantly for BPD prediction and added little above clinical risk factors for severe ROP prediction. This suggests that these findings are likely to hold true at other NICUs. A study conducted at multiple NICUs in the NICHD Neonatal Network developed a model for BPD using demographic and clinical variables together with FiO_2 at multiple points in the first 28 days after birth, and showed that the weight of individual model components changed over time.[13] The model was developed and externally validated in cohorts that differed somewhat in demographics and BPD rates and performance was lower in the validation cohort. Further optimization and validation in prospective and additional NICU cohorts are needed to determine whether POPS can be applied broadly for risk adjustment between centers.

Methods other than logistic regression might improve model performance. Several recent publications use detrended fluctuation analysis of vital signs to predict IVH.[14,15,16] It is important to note that IVH prediction has its limitations in retrospective studies such as ours due to the fact that the exact timing of the event is unknown. Abnormal HR and SpO_2 patterns in the first 12 hours after birth can represent disturbances caused by the hemorrhage rather than a warning of the event occurring later. Future work should test machine learning techniques in addition to more sophisticated regression analyses to predict both imminent and remote outcomes. Nonetheless, logistic regression is an appropriate first step for this type of analysis and performed well within and between centers.

Partnership with bioengineering or software developers could allow an algorithm such as POPS to be built-in to pulse oximetry monitoring and thus available for use in clinical care. At the bedside, POPS could potentially improve outcomes through targeted therapies or preventative strategies for infants marked high risk by abnormal physiologic patterns in addition to standard clinical risk factors. POPS could also help to risk stratify patients within populations for research.

Author Manuscript Author Manuscript Author Manuscript Author Manuscript

Conclusion

While demographic and clinical variables can be used to predict adverse outcomes in preterm infants, physiologic data improves outcome prediction by adding information on illness severity. In this study we developed POPS, a tool that uses pulse oximetry and clinical data to predict adverse outcomes in VLBW infants in the first 12 hours or first week after birth. We also showed that adding HR and SpO_2 analysis to clinical data improved prediction of death, severe intraventricular hemorrhage and bronchopulmonary dysplasia over clinical factors alone. Early identification of high risk infants gives clinicians an opportunity to target specific populations or individual patients for preventative interventions or new treatments.

Acknowledgments

Funding support: This research was supported by research grants from the NICHD (NIH R01 HD072071) and the UVA Translational Health Research Institute of Virginia (THRIV).

References

1. Sullivan BA, McClure C, Hicks J, Lake DE, Moorman JR, Fairchild KD. Early Heart Rate Characteristics Predict Death and Morbidities in Preterm Infants. J Pediatr 2016;174:57–62. [PubMed: 27113378]
2. Saria S, Rajani AK, Gould J, Koller D, Penn AA. Integration of early physiological responses predicts later illness severity in preterm infants. Sci Transl Med 2010;2(48):48ra65.
3. Richardson D, Gray J. Score for Neonatal Acute Physiology: a physiologic severity index for neonatal intensive care. Pediatrics. 1993.
4. Griffin MP, O'Shea TM, Bissonette EA, Harrell FE, Lake DE, Moorman JR. Abnormal heart rate characteristics preceding neonatal sepsis and sepsis-like illness. Pediatr Res 2003;53(6):920–926. [PubMed: 12646726]
5. Fairchild KD, Lake DE, Kattwinkel J, et al. Vital signs and their cross-correlation in sepsis and NEC: a study of 1,065 very-low-birth-weight infants in two NICUs. Pediatr Res 2016. doi: 10.1038/pr.2016.215.
6. Hay WW, Rodden DJ, Collins SM, Melara DL, Hale KA, Fashaw LM. Reliability of conventional and new pulse oximetry in neonatal patients. J Perinatol 2002;22(5):360–366. doi:10.1038/sj.jp.7210740. [PubMed: 12082469]
7. Collins GS, Reitsma JB, Altman DG, Moons K. Transparent Reporting of a multivariable prediction model for Individual Prognosis Or Diagnosis (TRIPOD): The TRIPOD Statement. Ann Intern Med 2015;162(8):600. doi:10.7326/L15-0078-4.
8. Pencina MJ, D'Agostino RB, D'Agostino RB, Vasan RS. Evaluating the added predictive ability of a new marker: From area under the ROC curve to reclassification and beyond. Stat Med 2008;27(2): 157–172. doi:10.1002/sim.2929. [PubMed: 17569110]
9. Pencina M, D'Agostino R, Steyerberg E. Extensions of net reclassification improvement calculations to measure usefulness of new biomarkers. Stat Med 2011;30(1):11–21. [PubMed: 21204120]
10. Fairchild KD, Lake DE, Kattwinkel J, et al. Vital signs and their cross-correlation in sepsis and NEC: a study of 1,065 very-low-birth-weight infants in two NICUs. Pediatr Res 2016. doi: 10.1038/pr.2016.215.
11. Fairchild KD, Lake DE. Cross-Correlation of Heart Rate and Oxygen Saturation in Very Low Birthweight Infants: Association with Apnea and Adverse Events. Am J Perinatol 11 2017. doi: 10.1055/s-0037-1608709.

Author Manuscript Author Manuscript Author Manuscript Author Manuscript

12. Doheny KK, Palmer C, Browning KN, et al. Diminished vagal tone is a predictive biomarker of necrotizing enterocolitis-risk in preterm infants. Neurogastroenterol Motil 2014;26(6):832–840. [PubMed: 24720579]
13. Laughon MM, Langer JC, Bose CL, et al. Prediction of bronchopulmonary dysplasia by postnatal age in extremely premature infants. Am J Respir Crit Care Med 2011;183(12):1715–1722. doi: 10.1164/rccm.201101-0055OC. [PubMed: 21471086]
14. Tuzcu V, Nas S, Ulusar U, Ugur A, Kaiser JR. Altered heart rhythm dynamics in very low birth weight infants with impending intraventricular hemorrhage. Pediatrics. 2009;123(3):810–815. doi: 10.1542/peds.2008-0253. [PubMed: 19255007]
15. Zhang Y, Chan GSH, Tracy MB, Hinder M, Savkin A V., Lovell NH. Detrended fluctuation analysis of blood pressure in preterm infants with intraventricular hemorrhage. Med Biol Eng Comput 2013;51(9):1051–1057. doi:10.1007/s11517-013-1083-0. [PubMed: 23716182]
16. Huvanandana J, Nguyen C, Thamrin C, Tracy M, Hinder M, McEwan AL. Prediction of intraventricular haemorrhage in preterm infants using time series analysis of blood pressure and respiratory signals. Sci Rep 2017;7:46538. doi:10.1038/srep46538. [PubMed: 28436467]

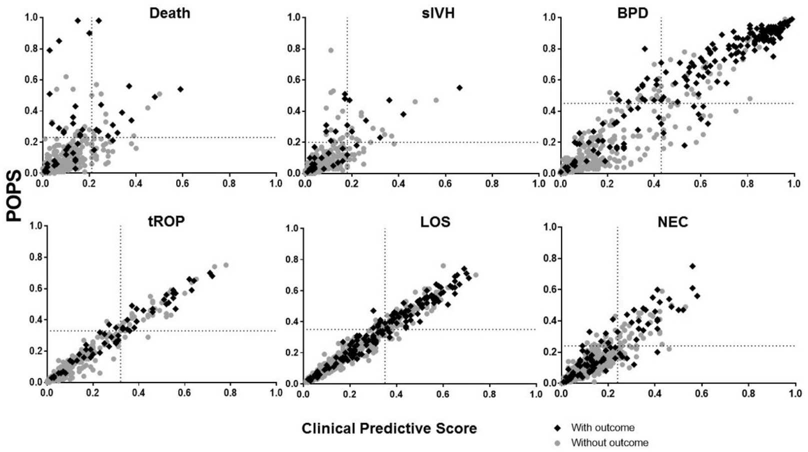

Figure 1.
Pulse Oximetry Predictive Score (POPS, y-axis) versus Clinical Predictive Score (x-axis) plotted for VLBW infants with (cases, black diamonds) and without (controls, gray dots) each outcome. Vertical and horizontal lines indicate the score at which the number of infants with higher scores equals the incidence of the outcome. The Clinical Predictive Score uses gestational age, birth weight, 1 and 5 minute Apgar scores, antenatal steroid exposure, and sex in a multivariate logistic regression model for each outcome, adjusted for site. POPS includes analysis of HR and SpO_2 measures in addition to clinical variables. Plotting POPS vs Clinical Risk Score in cases and controls illustrates the net proportion of infants correctly reclassified as high risk or low risk by adding pulse oximetry data to the risk model. Visually, this is represented by the cases in the left upper quadrant and controls in the right lower quadrant of each graph.

Author Manuscript Author Manuscript Author Manuscript Author Manuscript

Author Manuscript Author Manuscript Author Manuscript Author Manuscript

Table 1.

Patient characteristics and outcomes. Demographics, perinatal variables and adverse outcomes are shown for infants in a combined, two-center cohort and individual NICU cohorts. All clinical variables shown were used for modelling risk of each outcome in a clinical predictive score.

	Combined	NICU 1	NICU 2
**N**	778	443	335
**Clinical variables**			
GA (mean ± SD, weeks)	28.0 ± 2.8	27.7 ± 2.9	28.3 ± 2.8 *
BW (mean ± SD, grams)	1028.7 ± 298	1031.4 ± 295	1025.0 ± 304
Sex (% male)	47	50	44
5 min Apgar score (median)	8	8	8
Antenatal steroids (≥ 1 dose, n, %)	733 (94)	411 (93)	314 (94)
**Outcomes**	N (%)		
Death prior to NICU discharge	48 (6)	27 (6)	21 (6)
Severe intraventricular hemorrhage	34 (4)	24 (5)	10 (4)
Bronchopulmonary dysplasia	186 (26)	159 (39)	27 (9) *
Treated retinopathy of prematurity	58 (8)	46 (11)	12 (4) *
Late-onset septicemia	139 (18)	82 (19)	57 (17)
Necrotizing enterocolitis	81 (11)	33 (8)	48 (15) *

*(p<0.01 for NICU 2 vs NICU 1)

Author Manuscript Author Manuscript Author Manuscript Author Manuscript

Table 2.

Relationship of heart rate (HR) and oxygen saturation (SpO_2) measures (higher ↑, lower ↓ with $p < 0.05$, or not significantly different, ns) for infants with each outcome relative to those without the outcome based on univariate analysis

	First 12 hours										First 7 days									
	Mean SpO_2	Mean HR	SD SPO_2	SD HR	Max XC HR - SPO_2	Min XC HR - SPO_2	Skewness SPO_2	Skewness HR	Kurtosis SPO_2	Kurtosis HR	Mean SPO_2	Mean HR	SD SPO_2	SD HR	Max XC HR - SPO_2	Min XC HR - SPO_2	Skewness SPO_2	Skewness HR	Kurtosis SPO_2	Kurtosis HR
Died	↓	↑	↑	ns	ns	ns	ns	ns	ns	ns										
sIVH	↓	↑	↑	ns	ns	ns	↑	↓	↓	ns										
BPD											↓	↑	↑	↓	↑	↑	↑	↓	↓	↑
tROP											↓	ns	↑	↓	↑	↑	↑	ns	↓	ns
LOS											↓	ns	↑	↓	↑	↑	↑	↓	↓	ns
NEC											↓	ns	↑	↓	↑	↑	↑	ns	↓	ns

Author Manuscript Author Manuscript Author Manuscript Author Manuscript

Table 3.

Coefficient rank in multivariate logistic regression models to predict each outcome using pulse oximetry measures (A) or clinical variables (B).

Outcome	Mean SPO_2	SD SPO_2	Mean HR	SD HR	Max XC HR-SpO_2	Min XC HR-SpO_2	Skewness SPO_2	Skewness HR	Kurtosis SPO_2	Kurtosis HR
**Coefficient Rank A**										
Died	1*	6	2*	4	7	8	10	5	3	9
sIVH	10	2	1*	3	5	6	8	4	9	7
BPD	3*	2*	10	9	8	7	5*	4*	6*	1*
tROP	2*	10	8	6	4	5	9	3*	7	1*
LOS	8	4	10	1*	2	9	6	3	7	5
NEC	6	7	5	1*	10	2	4	8	3	9

Outcome	GA	BW	Sex	Antenatal Steroids	Apgar 1m	Apgar 5m	Site
**Coefficient Rank B**							
Died	5	1*	3	7	4	2*	6
sIVH	2*	7	4	1*	5	6	3*
BPD	3*	2*	4*	7	5	6	1*
tROP	1*	3*	7	6	4	5	2*
LOS	2*	1*	7	4	6	3	5
NEC	2*	6	3*	4	5	7	1*

* $p \leq 0.05$

Author Manuscript Author Manuscript Author Manuscript Author Manuscript

Table 4.

Performance and comparison of predictive models. The table shows results of calculating the area under the receiver operating characteristic curve (AUC) with confidence intervals determined by bootstrap for each predictive model. Significance of pulse oximetry scores in bivariate POPS model are indicated next to AUC confidence interval. All clinical predictive scores are highly significant. The net reclassification improvement (NRI) represents the performance of patients reclassified into the correct risk group by POPS compared to the model using clinical variables alone.

	Clinical Predictive Score	Pulse Oximetry Score	POPS	POPS NRI (95% CI)
	AUC (95% CI)			
Died	0.788 (0.725-0.849)	0.793 (0.721-0.860)	0.864 (0.796-0.916) ***	0.76 (0.47-1.05) **
sIVH	0.840 (0.763-0.889)	0.790 (0.727-0.867)	0.879 (0.829-0.936) **	0.53 (0.19-0.87) **
BPD	0.921 (0.897-0.945)	0.886 (0.854-0.913)	0.935 (0.920-0.951) ***	0.52 (0.35-0.68) **
tROP	0.910 (0.874-0.933)	0.845 (0.811-0.884)	0.914 (0.885-0.941) *	0.22 (−0.04-0.47)
LOS	0.802 (0.765-0.840)	0.749 (0.703-0.782)	0.808 (0.766-0.840) *	0.20 (0.02-0.38)
NEC	0.760 (0.700-0.811)	0.710 (0.659-0.775)	0.782 (0.729-0.832) **	0.36 (0.13-0.59)

* p-value ≤ 0.05,

** p-value < 0.01,

*** p-value < 0.00001

Author Manuscript Author Manuscript Author Manuscript Author Manuscript

Table 5.

Cross-validation of the Pulse Oximetry Predictive Score (POPS). AUCs with 95% confidence intervals using bootstrapping for model development and validation using individual NICU cohorts as both train and test cohorts.

	NICU 1 Train	NICU 2 Test	NICU 2 Train	NICU 1 Test
Died	0.865 (0.786-0.934)	0.839 (0.748-0.918)	0.880 (0.795-0.935)	0.825 (0.725-0.901)
sIVH	0.869 (0.797-0.920)	0.850 (0.707-0.967)	0.932 (0.870-0.982)	**0.815 (0.733-0.889)***
BPD	0.931 (0.900-.0952)	0.875 (0.802-0.937)	0.897 (0.830-0.943)	0.914 (0.891-0.940)
tROP	0.919 (0.875-0.944)	0.844 (0.766-0.915)	0.951 (0.928-0.971)	**0.862 (0.817-0.891)***
LOS	0.772 (0.718-0.829)	0.845 (0.786-0.891)	0.886 (0.830-0.934)	**0.729 (0.677-0.790)***
NEC	0.756 (0.682- 0.829)	0.724 (0.646-0.785)	0.842 (0.792-0.901)	**0.654 (0.556-0.756)***

*p-value <0.05 comparing test with train model
