# Supplementary material for: Exploring Computational Techniques in Preprocessing Neonatal Physiological Signals for Detecting Adverse Outcomes: Scoping Review
Source: Interact J Med Res. 2024 Aug 20;13:e46946. doi: 10.2196/46946 (PMC11372324; doi:10.2196/46946)
Supplement: Multimedia Appendix 3 [file ijmr_v13i1e46946_app3.zip › Included Papers - Final/3556/Temko et al. - 2015 - Multimodal predictor of neurodevelopmental outcome.pdf]

Computers in Biology and Medicine 63 (2015) 169–177

Contents lists available at ScienceDirect

Computers in Biology and Medicine

journal homepage: www.elsevier.com/locate/cbm

Multimodal predictor of neurodevelopmental outcome in newborns with hypoxic-ischaemic encephalopathy

Andriy Temko [a,c,*], Orla Doyle [d], Deirdre Murray [b,c], Gordon Lightbody [a,c], Geraldine Boylan [b,c], William Marnane [a,c]

[a] Department of Electrical and Electronic Engineering, University College Cork, Ireland
[b] Department of Pediatrics and Child Health, University College Cork, Ireland
[c] Neonatal Brain Research Group, INFANT Research Centre, University College Cork, Ireland
[d] Department of Neuroimaging, Institute of Psychiatry, King's College London, London, UK

ARTICLE INFO

Article history:
Received 11 February 2015
Accepted 23 May 2015

Keywords:
Neonatal
Multimodal
EEG
ECG
Neurodevelopmental
Outcome
Decision support system

ABSTRACT

Automated multimodal prediction of outcome in newborns with hypoxic-ischaemic encephalopathy is investigated in this work. Routine clinical measures and 1 h EEG and ECG recordings 24 h after birth were obtained from 38 newborns with different grades of HIE. Each newborn was reassessed at 24 months to establish their neurodevelopmental outcome. A set of multimodal features is extracted from the clinical, heart rate and EEG measures and is fed into a support vector machine classifier. The performance is reported with the statistically most unbiased leave-one-patient-out performance assessment routine. A subset of informative features, whose rankings are consistent across all patients, is identified. The best performance is obtained using a subset of 9 EEG, 2 h and 1 clinical feature, leading to an area under the ROC curve of 87% and accuracy of 84% which compares favourably to the EEG-based clinical outcome prediction, previously reported on the same data. The work presents a promising step towards the use of multimodal data in building an objective decision support tool for clinical prediction of neurodevelopmental outcome in newborns with hypoxic-ischaemic encephalopathy.
© 2015 Elsevier Ltd. All rights reserved.

1. Introduction

Perinatal hypoxic-ischaemic injury remains a major cause of neurodevelopmental disability. It is thought to affect between 3 and 5 per 1000 live births [1] and accounts for 23% of all neonatal deaths worldwide. With the advent of potential neuroprotective therapies in the form of induced hypothermia, early and accurate methods of diagnosis of hypoxic-ischaemic encephalopathy (HIE) have become increasingly important [38,39]. At the same time, reliable prognostic information is vital in order to counsel parents and caregivers. It is often a major challenge facing those caring for infants with HIE [2].

To date, several different types of monitoring have been studied for outcome prediction. The prognostic value of the EEG in the prediction of long-term outcome is well documented [2–5]. A meta-analysis by Sinclair et al. [3] concluded that burst suppression, slow activity, low voltage and an isoelectric pattern are associated with a markedly increased risk of death or neurodevelopmental handicap. Ramaswamy et al. [6] reviewed biomarkers in full-term newborns with encephalopathy to determine if current biomarkers were strong enough for clinical implementation as predictors of outcome. The review concluded that no biomarker had yet been studied extensively enough to warrant routine clinical use. Laptook et al. [7] reported that Apgar scores assigned at 10 min provided useful prognostic information. However, both the American Academy of Pediatrics and the American College of Obstetrics and Gynaecology recommended that the Apgar score alone should not be used as a predictor of neurodevelopmental outcome [8]. Lingwood et al. [9] hypothesized that cerebral impedance as measured by bioimpedance spectroscopy would be increased in newborns who have suffered a hypoxic/ischaemic insult and who subsequently have a poor neurological outcome. However, on examining a set of 24 newborns it was concluded that this attribute was not suitable for discrimination of outcome. Jyoti et al. [10] developed simplified magnetic resonance grades and found these grades to be highly predictive of neurodevelopmental outcome. However, the optimal timing of an MRI examination for prognosis in newborns with HIE is the second week of life and therefore its use for early prognostication may be limited [11].

The development of automated decision support systems for monitoring in the newborn is a rapidly expanding area [44]. Both

* Corresponding author at: Room 2.12, Dept. Electrical and Electronic Engineering, College Road, University College Cork, Ireland. Tel.: +353214903662.
E-mail addresses: atemko@ucc.ie (A. Temko), orla.doyle@kcl.ac.uk (O. Doyle), d.murray@ucc.ie (D. Murray), g.lightbody@ucc.ie (G. Lightbody), g.boylan@ucc.ie (G. Boylan), l.marnane@ucc.ie (W. Marnane).

http://dx.doi.org/10.1016/j.compbiomed.2015.05.017
0010-4825/© 2015 Elsevier Ltd. All rights reserved.

EEG and heart rate variability (HRV) have been incorporated in the automated detection of neonatal seizures [12,13]. Neonatal HR monitoring has also been used in the prediction of sepsis and systematic inflammatory response syndrome [14]. HRV is thought to provide information on the autonomic balance of the infant, which may be disturbed post hypoxic injury [15]. More recently, depressed HRV in neonates has been associated with moderate-to-severe abnormalities on EEG and MRI [40]. Vergales et al. [40] have also found that low HRV remained significantly associated with adverse short-term outcomes (day 4–7).

The messages from the clinical literature, both with positive and negative conclusions, show that there is no single measure that provides reliable long term prognostication. Most of the cited works though were limited to linear methods that consider a single feature at a time. A nonlinear complex relationship between these predictors has not been explored, and in fact it may improve accuracy, with each parameter providing complementary information. In this study, a multimodal combination of routine clinical markers, EEG and HR parameters is investigated together with non-linear support vector machines (SVMs) employed for neurodevelopmental outcome prediction at 24 months in newborn infants with HIE.

The paper is organized as follows: Section 2 details the clinical dataset used in the experiments, introduces the investigated features, and describes the outcome prediction system developed in the group, along with the feature selection routine. Section 3 presents and discusses obtained results. Conclusions are drawn in Section 4.

2. Methods

2.1. Database

Newborns were prospectively recruited into this study if they fulfilled two or more of the following criteria: initial capillary or arterial pH <7.1, Apgar score <5 at 5 min, initial capillary or arterial lactate >7 mmol/l (normal newborn values <4 mmol/l) or abnormal neurology/clinical seizures. Infants who met the initial criteria were examined using a standardized method of neonatal neurological assessment, the Amiel–Tison method [16]. Initial pH and base deficit (BD) were analysed on admission to the neonatal unit (usually within 30 min of birth) on a unit-based ABL300 blood gas analyser (Radiometer, Copenhagen, Denmark).

Video-EEG and ECG data were recorded synchronously for each patient using the Viasys NicOne EEG system, with a sampling rate of 256 Hz. The 10–20 system of electrode placement, modified for newborns was used with the following montage: F4-C4, C4-O2, F3-C3, C3-O1, T4-C4, C4-Cz, Cz-C3 and C3-T3. Recordings were commenced as soon as possible after birth. All recordings took place in the neonatal intensive care unit of Cork University Maternity Hospital between May 2003 and May 2005, and the study had full ethical approval from the Clinical Research Ethics Committee of the Cork Teaching Hospitals. Newborns were not treated with therapeutic hypothermia. From these recordings, 1-hour segments of EEG and ECG that were mostly free from visual artifacts were selected for analysis at 24 h of age for each infant. Developmental follow-up was assessed using the Griffiths Scales of Mental Development at 24 months [17]. A neurological assessment of motor function was performed at the same time. An abnormal outcome was defined as a general quotient less than 87, significant motor dysfunction, or death.

In total, 38 term infants fit the criteria for this study with 21/17 found to have abnormal/normal outcomes at 24 months, respectively. Fig. 1(top) shows an example of multi-channel EEG and ECG recordings for a newborn who subsequently had a normal neurodevelopmental outcome. Fig. 1 (bottom) presents an example from a newborn with an abnormal outcome. In contrast to the continuous EEG activity in Fig. 1(top), the EEG for this patient is in a state of low voltage burst-suppression with clear asymmetry between hemispheres.

2.2. Features

Limited prior knowledge was available on what features would perform well for the considered task. A large set of features was extracted from the three modalities, EEG, ECG and clinical. It is our

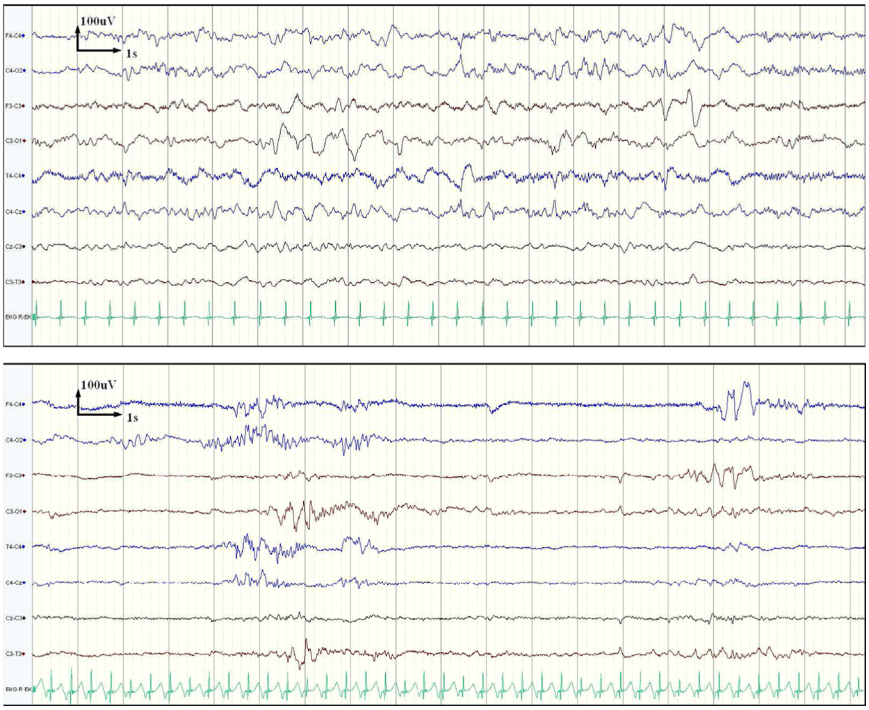

Fig. 1. Multi-channel EEG and ECG recordings from newborns who had a normal (top) and abnormal (bottom) neurodevelopmental outcome at 24 months.

intention to describe the signals from as many perspectives as possible.

2.2.1. EEG

The 1-hour EEG per patient was down-sampled from 256 Hz to 32 Hz with an anti-aliasing filter set at 16 Hz. The filtered EEG was then segmented into 60 s epochs with no overlap.

A set of 57 EEG features was investigated in this work (Table 1). This long feature set carries frequency, energetic and structural information (information theory) and can be seen as a general multi-resolution descriptor of EEG activity.

In fact, 55 of these 57 features have successfully been exploited for detection of seizures in newborns [12,18,33], and epilepsy in adults [19]. These features have been used for automated background EEG classification [20,41]. Previous work on prognosis of newborn outcome using EEG [3,5] has shown that quantification of brain asymmetry can be indicative of brain damaging processes. Thus, brain symmetry index [21] was added to the set of 55 features to quantify the difference between spectral characteristics of the left and right hemispheres. Similarly, normalized delta band power which measures the percentage of power in the 0.5 to 4 Hz band has been related to outcome in [22] and was also added to the features set.

As HIE is assumed to be a global injury, the information across all EEG electrodes was combined by taking the feature mean. The median has resulted in a similar performance and its results are not included here.

2.2.2. Heart rate

The HR was estimated using the time intervals between the QRS complexes of the ECG signal. The algorithm reported in [23] was used to extract the R-waves. The resulting R-points were manually inspected to correct any ectopic beats or mark artifacts. The instantaneous HR was calculated in beats per minute (BPM). The 1-hour HR signal was first segmented into 60 s epochs. A window length of 2 to 5 min was recommended to calculate short-term HRV features in adults [24]. However, the resting HR of a newborn infant is on average twice that for a typical adult; 100–200 BPM for newborns in comparison to 60–100 BPM for adults. Thus, the window length can be set to 60 s in newborn analysis.

A set of 60 features was then extracted from each 60 s epoch of ECG (Table 1). These features have partially been implemented in apnoea studies [25], automated ECG-based neonatal seizure detection [15], sleep monitoring [26], sepsis monitoring [27], central nervous system innervations in adults [28], and detection of food allergy from paediatric ECG [29]. Similarly to EEG, these features serve as a general multi-resolution descriptor of ECG and are used to quantify the baseline and variability of the HR. Feature formulas and detailed feature descriptions can be found in [15,29].

2.2.3. Clinical

The Apgar score provides a clinical evaluation of the newborn by combining 5 signs such as respiratory effort, reflex irritability, muscle tone, HR, and colour. The score is assigned 5 min after birth, ranging from 0 to 10. In addition, the initial pH and base deficit were also analysed, to give a total of three clinical features.

2.3. Multimodal predictor of neurodevelopmental outcome

The diagram of the developed multimodal outcome predictor is shown in Fig. 2. After the features are extracted, every newborn is then represented by approximately 60 vectors of 120 features (over the whole period). All feature vectors from newborns with normal and abnormal outcomes are labelled 1 and -1, respectively. To assure commensurability of the various features the training data were normalized to zero mean and unit variance. The obtained normalization template is saved and then applied to the testing data.

The EEG and HR features were synchronized as they were extracted from the same 60 s epochs across 1 h of data. In contrast, the clinical features represent one value per newborn, i.e. a one-off measurement. To account for this, the clinical features are replicated for each epoch and appended to each feature vector prior to classification. The normalized features extracted from each epoch were then fed to train a single SVM classifier with a Gaussian kernel. Model selection on the training data was performed to choose suitable SVM model parameters. The outputs of the SVM were converted to pseudo probabilistic values using Platt's method

Table 1
Features extracted for each epoch.

Modality	Features
EEG (57)	– Total power (0–12 Hz), - Peak frequency of spectrum, - Spectral edge frequency (SEF80%, SEF90%, SEF95%), - Power in 2 Hz width subbands (0–2 Hz, 1–3 Hz, . 10–12 Hz), - Normalised power in same subbands, - Wavelet energy (Db4 wavelet coefficient corresponding to 1–2 Hz), - Normalised delta band power,<br>– Curve length, - Number of maxima and minima, - Root mean square (RMS) amplitude, - Hjorth parameters (inactivity, mobility and complexity), - Zero crossing rate (ZCR), - ZCR of the Δ and the $\Delta\Delta$, - Variance of Δ and $\Delta\Delta$, - Autoregressive modelling error (AR model order 1–9), - Skewness, - Kurtosis, - Nonlinear energy<br>– Shannon entropy, - Spectral entropy, - Brain symmetry index; - SVD entropy, - Fisher information
HR (60)	– Total power, - Power in very low frequency (VLF), Power in LF (0.04–0.2 Hz), - Power in high frequency (HF) (freq. $>$ 0.2 Hz), - VLF/HF, - LF/HF, - Spectral entropy, - Power in 0.03125 Hz subbands (from 0 to 0.8 Hz)<br>– Mean NN, - Standard deviation (std) of NN (SDNN), - RMS of SDNN, - Coefficient of variation, - Percentage of consecutive NNs that vary by more than 5, 10, 15, 20, 25 ms (pNNx), - Std of the successive NN differences, - Mean of the absolute value of first derivative of NN, - Max change in NN, - Poincare plot measures (SD1, SD2, CSI, CVI), - Sequential trend analysis measures (del plus, del minus), - Allan Factor at 5, 10, 15, 20, 25 s scales, - Line length, - Nonlinear energy, - ZRC<br>– Shannon entropy
Clinical (3)	– Apgar score, - Initial pH, - Base deficit

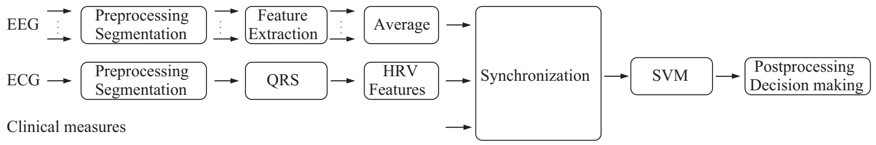

Fig. 2. Architecture of the SVM-based multimodal outcome prediction system.

[30] to provide a probability of having a normal outcome given a feature vector observation. In fact, the system outputs a trend of probabilistic values, one per each 60 s epoch within the 1 h of data. An example of the probabilistic trend for a patient with normal and a patient with abnormal outcomes is shown in Fig. 3. These ~60 probabilistic measures were averaged for each specific patient to give a single value representing a probability that the patient will have a normal outcome given a set of 1-hour EEG, 1-hour ECG, and one-off clinical observations.

2.4. Feature selection routine

A large set of features is extracted from EEG and HR in order to accompany the clinical measures. Due to the novelty of research area, no features have yet been conclusively established as being useful for this application. One of the aims of the study is to identify a feature subset which is informative for the prediction of neurodevelopmental outcome at 24 months. Here, the Recursive Feature Elimination (RFE) method [31] was employed to provide ranking for the multimodal feature set. It has been initially proposed for selecting genes that are relevant for a cancer classification problem. An advantage of this routine for our application is that the SVM optimization criterion is used in feature selection. Thus, there is a match between the feature selection and classification algorithms. In this routine, an SVM classifier is first trained. The deviation in the cost function is then calculated for the removal of each one of the currently active features, while preserving the same set of support vectors. The cost deviation DJ_i for the ith feature is computed as

$$DJ_i = \left|\frac{1}{2}\alpha^T H\alpha - \frac{1}{2}\alpha^T H_i \alpha\right|, \quad (1)$$

where H is the matrix with elements, $H(p, q)=y_p y_q K(x_p, x_q)$, and here K is Gaussian kernel function, x_p, x_q are support vectors and y_p, y_q are their respective labels. The feature corresponding to the smallest difference in DJ_i is removed. The set of support vectors with corresponding Lagrange multipliers (α) is assumed to remain unchanged for the H_i matrix which is re-computed without feature i. To relax this assumption, only a single feature is eliminated per iteration. The SVM model is then retrained to balance the space of remaining features and support vectors. By sequentially eliminating each least useful feature the RFE feature selection results in the nested subsets of features. The experiments section explains how the RFE can be used to rank features. It is worth noting that the RFE feature selection routine does not require testing the SVM model. Therefore, feature selection can be performed on the training data.

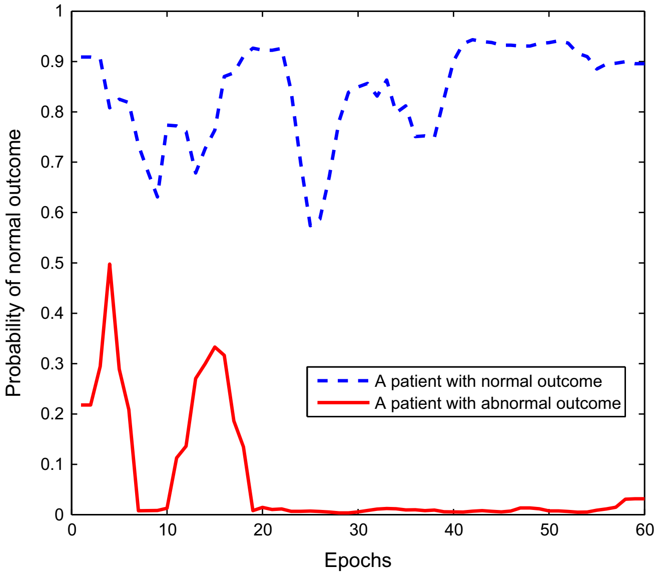

Fig. 3. An example of the system output as a continuous probabilistic trend for 60 1-minute epochs for 1 patient with normal (dashed line) and 1 patient with abnormal outcomes (solid line).

2.5. Performance assessment and metrics

The leave-one-patient-out (LOO) method was used to assess the performance of the developed system. For each iteration of the LOO all but one patients' data were used for training and the remaining patient's data were used for testing. This procedure was repeated until each patient had been a test subject. The LOO is known to be an almost unbiased estimation of the true error [32] – the error obtainable by testing on a separate dataset of infinite size. Importantly, the performance assessment routine is independent of the model selection routine, so that the testing patient is not seen or used for training the classifier or tuning other system parameters at any time. The detailed explanation of the performance assessment and model selection routines are indicated in Fig. 4.

The metric used in this work is the area under Receiver Operating Characteristic (ROC) curve. The ROC curve plots sensitivity and specificity values which are defined as the accuracy of each class (abnormal and normal) separately. By varying the threshold from zero to one, 38 individual sensitivity and specificity pairs were produced to construct the ROC curve. Essentially, this curve displays the performance of the system across all possible operating points. The range of the ROC area is from 50% for no apparent distributional difference between normal and abnormal classes, to 100% for perfect separation between classes [42]. Furthermore, the ROC area is equivalent to the Wilcoxon rank sum statistical test [43]. This can be interpreted as such: For an outcome predictor giving a continuous probabilistic value, the ROC area quantifies the probability that a randomly sampled patient with normal outcome will have a higher value than a randomly sampled patient with abnormal outcome.

3. Experiments and discussion

3.1. Performance of the single modalities

The ROC areas for each modality system (EEG, HR and clinical) can be seen in Fig. 5. Using clinical features only, an ROC area of 61.3% was achieved. The ROC areas of 66.1% and 75.1% were achieved using HR and EEG features, respectively. Each modality separately provides discrimination which is higher than that of the random choice (ROC=50%), but far from being a robust predictor of outcome in a clinical environment. As expected, the EEG modality achieves the highest performance, followed by the HR and clinical features. In effect, these results can serve as a quantified indication of discriminative capacity contained within each modality, which confirms what has been reported in the clinical literature. However, to date there has been no reported research investigating all the modalities simultaneously or exploiting the nonlinear relations within a multidimensional feature set.

3.2. Multimodal and feature selection results

The system which was trained on all 120 features resulted in an ROC performance of 72.6% which actually was worse than the ROC area observed using EEG features alone (Fig. 5). In order to gain insight into the role of the each modality, we examined the order in which the features were eliminated by the RFE routine. Three of

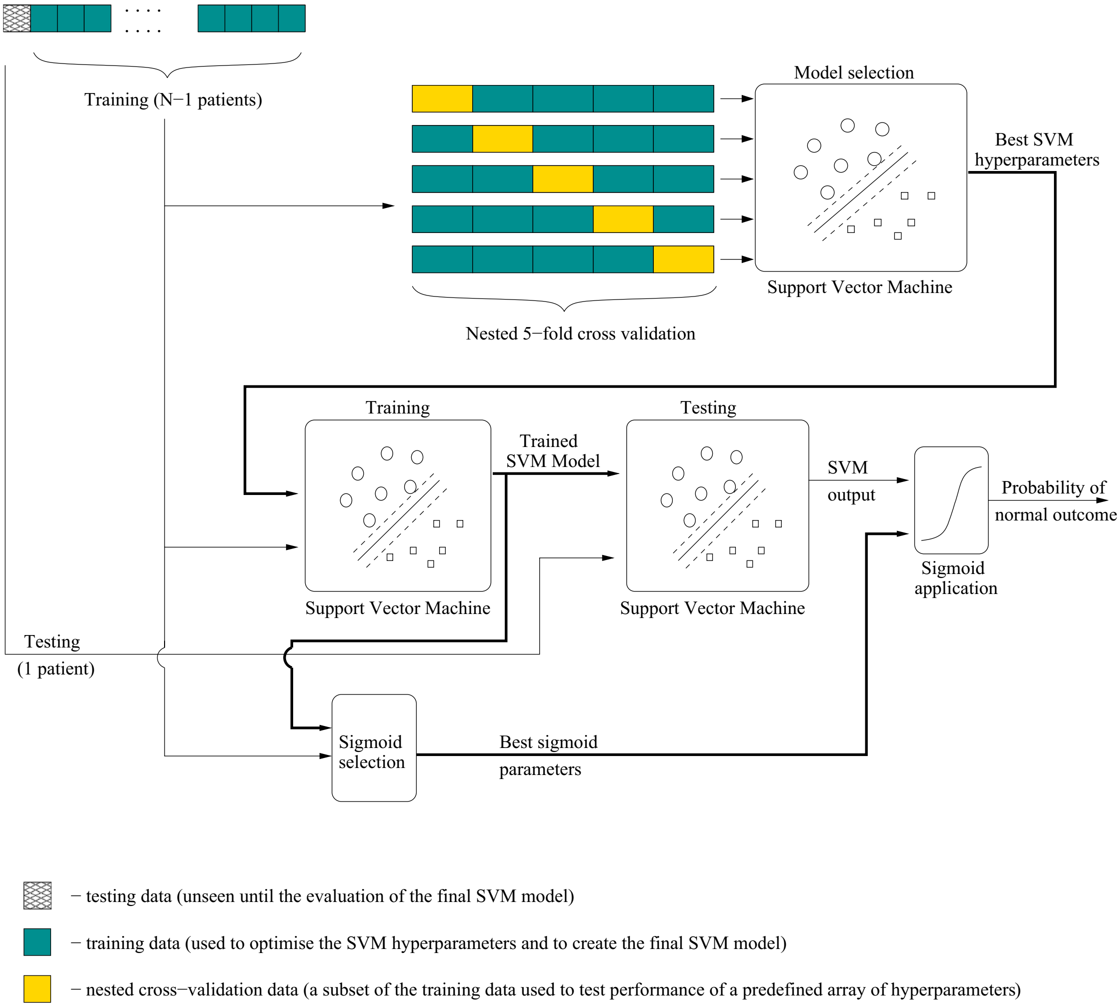

Fig. 4. A diagram of dataflow in the leave-one-out performance assessment and 5-fold cross-validation model selection routines.

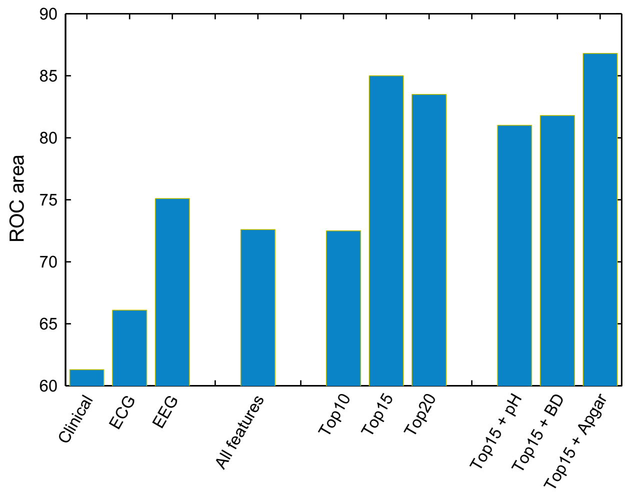

Fig. 5. Performance of outcome prediction in terms of the ROC area. From left to right: single modalities, all features (no feature selection), selected EEG/ECG features, and selected EEG/ECG features in combination with clinical measurements.

the 120 features investigated are clinical features – the Apgar score, pH of the umbilical cord and the base deficit. Numerically, they represent one value per newborn and have zero intra-patient variability. These measures are quantified with low resolution within a restricted range and can be thought of as ‘quasi-categorical’ features. For this reason, the 3 clinical features were not used along with non-categorical variables in the RFE feature selection. Instead, the RFE routine was first applied to the EEG and HR features which were pooled together, i.e. 117 features, and then the contribution of each of the clinical features to the resultant feature set was investigated.

The RFE routine was applied to every training set in the LOO performance assessment. Thus, the order in which features were eliminated is different for iteration. For instance, considering a subset of 20 remaining features these features may be different for each training dataset. In order to identify features that appear to be consistently useful in all LOO iterations, the top 10, 15 and 20 features, after the RFE routine has been applied, were examined and only the features that appear among the top N features for all patients were retained.

The results with feature selection are shown in Fig. 5. Using these criteria, the resultant feature subsets contain 4, 11 and 17 features. The system based on the 11 features selected from the Top 15 features resulted in the highest performance with an ROC area of 85%. These 11 features appeared in each patient's Top 15 ranking which highlights the consistency of the RFE routine for feature selection across all patients.

Next, the contribution of every clinical feature to the 11 features selected from EEG and HR modalities is assessed. It can be seen that only the addition of the Apgar score results in an increase of the ROC area, reaching 86.8%. As can be seen from Fig. 5

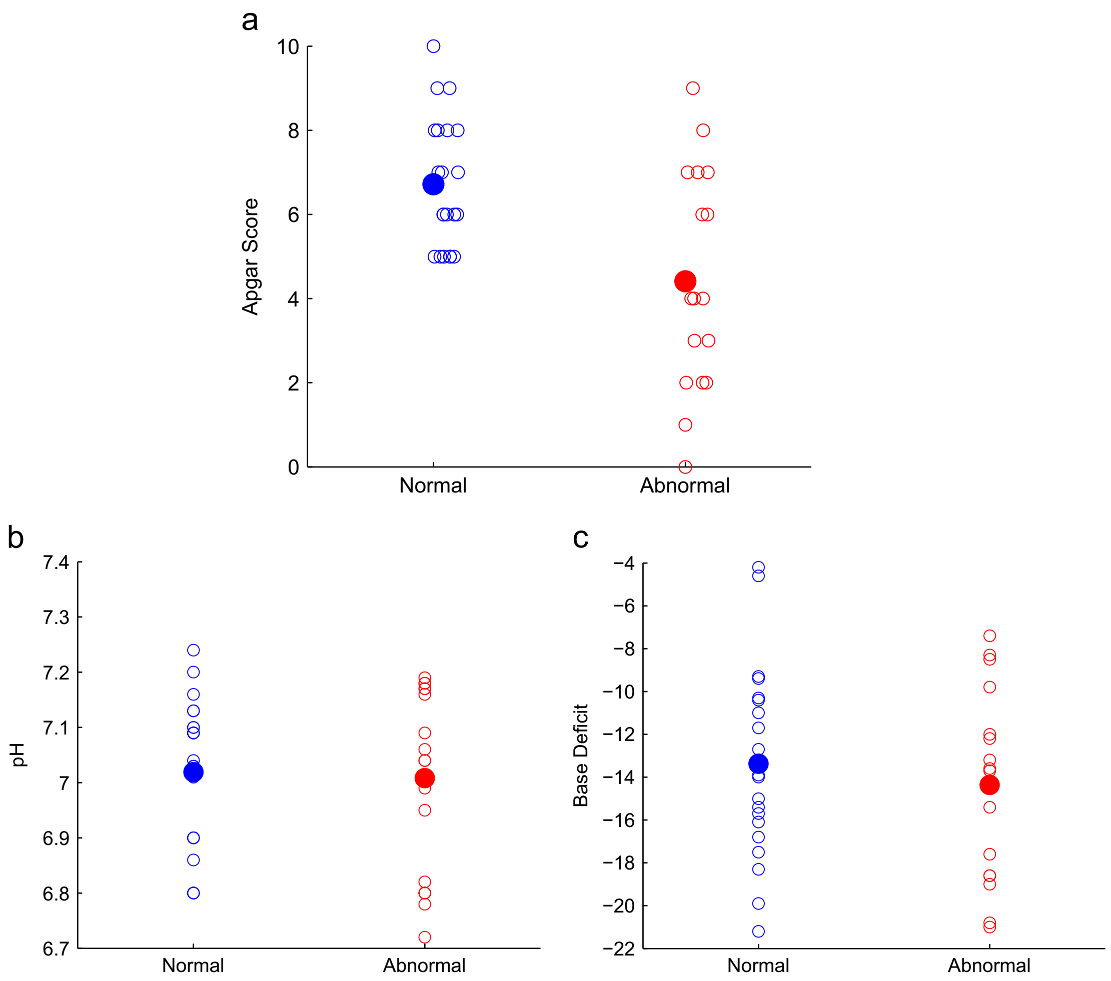

Fig. 6. Clinical features. (a) Apgar score, , $p < 0.05$ (b) pH , $p > 0.05$ and (c) base deficit > 0.05 versus outcome. The filled data point represents the mean feature of the group. The statistical significance of the separation between the groups is assessed using the Wilcoxon rank-sum test.

the ROC area for the system which uses all 120 features is worse than that of the system that uses the 12 features selected from the EEG, HR and clinical measures. Actually, feature selection resulted in a substantial improvement over the system which uses all investigated features. It also indicates that 90% of features used for generic description of EEG and HR signals are not informative for the purpose of the considered task.

3.3. Feature analysis

The discriminative power of the three clinical features for the outcome prediction is illustrated in Fig. 6. It can be seen that while some discriminatory information is contained in these features and especially the Apgar score, the individual features and their combination through visual interpretation would be insufficient for accurate outcome prediction.

The feature ranking procedure allows generalization of the importance of a particular feature. A given feature is unlikely to serve as a robust descriptor for the task if it is important for one patient and useless for another. The 12 features that resulted in the highest performance are shown in Table 2. It can be seen that the final feature set consists of 9 EEG features, 2 h features, and 1 clinical feature.

Looking at the selected EEG features from Table 2, it can be seen that a number of parameters aim at quantifying the spectral content of the EEG signal. In particular, the selection of the normalised powers in EEG sub-bands implies that certain frequencies may

Table 2
A set of 12 selected features.

EEG	Normalised energy in the 2–4 Hz subband
	Normalised energy in the 3–5 Hz subband
	Normalised energy in the 9–11 Hz subband
	Peak frequency
	Spectral entropy
	Inactivity
	Number of zero crossings
	Number of zero crossings in the first derivative
	Number of zero crossings in the second derivative
HR	Shannon entropy
	Power in the HF band of the HR power
Clinical	Apgar

contain more important information than others. Similarly, the peak frequency and the number of zero crossings aim at quantifying the dominant frequency of the EEG, suggesting that these measures are highly dichotomous in outcome prediction. The spectral entropy measures the distribution of the frequency components. The inactivity of the EEG which is calculated by subtraction of the adjacent samples of the EEG and counting the number which fall below 0.01 μV is useful for quantifying periods of suppression in the EEG. In contrast to energy-based features (EEG *amplitude analysis*), the number of zero crossings of the first and second derivatives of EEG are related to EEG *interval analysis* [34] and measures the distribution of intervals between the extremes (1st derivative) as well as saddle points (2nd derivative).

The Shannon entropy of the HR measures the complexity of the HR signal. The power in the HF band is influenced by respiration and governed exclusively by the parasympathetic nervous system. Clinical studies have reported that the effect of encephalopathy in newborns on autonomic function is related to its clinical stage [15].

Although it is possible to provide reasonable rationale for the chosen features, it is worth noting that the selected features are only important if considered together. It is known in machine learning that two features may be completely useless when considered separately and provide a perfect separation when considered together [35]. The statistical analysis of each feature alone, which is common in clinical literature, could lead to different conclusions with regards to the importance of these features, as neither the context (the presence of other features) nor the nonlinear relations are considered.

For illustration purposes, the projection of neurodevelopmental outcome prediction onto the multimodal 3-D space is shown in Fig. 7. The single best feature is taken from each modality – the number of zero crossings of the 2nd derivative of the EEG, the Shannon entropy of the HR and Apgar score. Certain separation is visually perceivable between the normal and abnormal outcome groups.

3.4. Comparison with clinical accuracy

The point on the ROC curve with the highest accuracy results in a correct detection of abnormal outcome of 71% (5 errors) and correct detection of normal outcome of 95% (1 error), 6 errors in total. Murray et al. [2] performed a clinical analysis of EEG patterns over one hour periods by focusing on burst suppression and sleep-wake cycling using the same dataset as employed in this work. After EEG grades were assigned, they were examined relative to neurodevelopmental outcome. The EEG grading was found to correctly predict the outcome in 32 out of 38 newborns, with 5 errors in the normal and 1 error in the abnormal outcome group. The performance of the automated system developed here is comparable to that achieved using clinical methods which require the time-consuming visual analysis of the EEG by an expert. It is worth noting that although the number of errors is the same the errors themselves are different, being from groups with different outcome. It indicates that the developed system can complement diagnosis-based decisions, and the two approaches together have a potential to minimise the final prediction error.

In this study, the results have been reported using the ROC area which quantifies discriminability independent of an operating point. The clinical, financial (cost of unnecessary treatment) and legal cost (malpractice) of a false negative or false-positive results as well as the knowledge of the probability of outcome for the population can aid the choice of a desirable operating point [36]. One of the major benefits of the developed system is the availability of a continuous probabilistic value that a patient will have a normal neurodevelopmental outcome. This probability can be interpreted as a continuous HIE grading, which ranges from 0 to 1, and might be as informative as a binary label for a clinician. Fig. 8 shows the distribution of the raw (unsmoothed) continuous system probabilities for each HIE grade as boxplots with the central mark being the median, the edges of the box being the 25th and 75th percentiles, the whiskers extending to the most extreme datapoints. Despite the fact that the system was trained to match the neurodevelopmental outcome as a target and the final system decision is made by taking the average probability over the whole hour of observations, it is possible to see that even a raw probability over a one-minute epoch can be correlated with an HIE grade. Murray et al. [2] concluded that the normal/mild HIE grade had 100% positive predictive value for the normal outcome. Here, looking at the problem from the opposite direction, it is possible to indicate that a very low probability of normal outcome is mostly associated with severe brain injury. A higher probability of normal outcome is more specific to the mild and moderate injuries as shown in Fig. 8. Usually diagnosis drives treatment and is used to prognosticate outcome as it has been done in [2]. It is interesting to observe that similarly the other way around has a potential to provide complementary information.

3.5. Limitation of the study

In this study, multimodal data were combined using feature-level fusion. The aim of the work is to show that the combination of multimodal physiological data can lead to more robust classification. The work does not intend to contribute to the general area

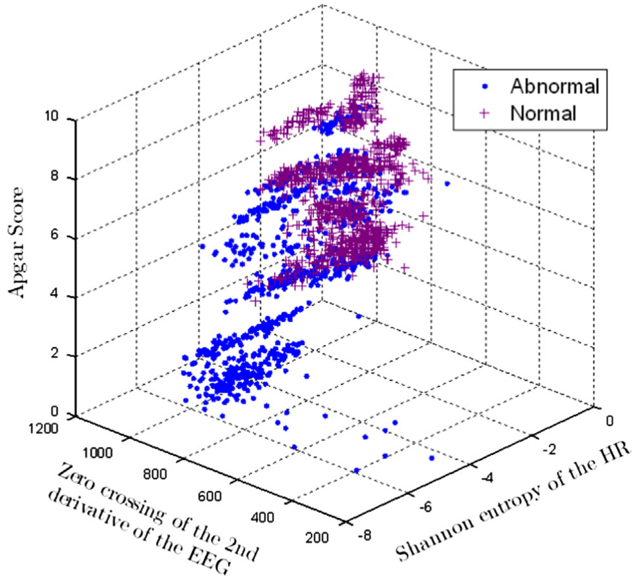

Fig. 7. A 3-D scatter plot of the best performing features from each modality – the number of zero crossings of the 2nd derivative of the EEG, the Shannon entropy of the HR, and Apgar score. Marker type indicates which outcome group the data belong to and each marker represents an individual 60 s epoch.

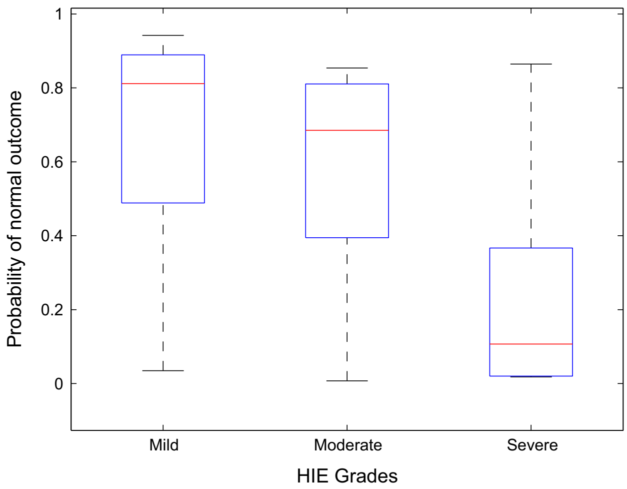

Fig. 8. Boxplots of the 1 m-epoch probabilistic output from the outcome prediction classifier across different HIE grades. The figure shows how prognosis information can be mapped to diagnosis information, whereas the opposite direction is usually followed – diagnosis drives treatment and is used to prognosticate outcome.

of pattern recognition by comparing various fusion methods or investigating various levels on which information from different modalities can be combined.

Although LOO performance assessment has been shown to be an accurate predictor of the error obtainable in practice [37], validation of the developed model on a separate dataset would be very valuable to assess the level of robustness of the system, e.g. to different recording equipment. The proposed methodology will be validated on a separate cohort of newborns that were treated with therapeutic hypothermia. It is known that 'cooling' affects both EEG and ECG signals and it is reasonable to assume that a different model for cooled newborns has to be built following the same methodology. Similarly, pharmacology is not taken into account in the proposed study.

A clinical analysis of patterns that resulted in prediction errors is not reported in this study. Neither did we investigate sensitivity analysis of the chosen time points where the recordings and clinical measurements were obtained. The influence of the location of brain pathologies on the developed system architecture is left outside of the scope of this study.

It is reasonable to assume that a more accurate identification of the outcome of newborns with HIE could be achieved by combining the developed model with automated EEG grading [38], or automated EEG background classification [20]. This work intends to show that using a long set of simple features and no prior information on relevance of those features, an accurate multimodal prediction system can be built. It is achieved using a rigorous performance assessment and model selection routines but without the help of higher level features such as quantified measures of temporal and/or cyclic patterns like burst suppression [39] or sleep-wake cycling. Non-linear combination of multiple features taken from different modalities provides less insight into the internal system functionality to healthcare professional than e.g. a quantified amount of bursts and suppression though the former may provide better final results. Often in the area of biomedical engineering one has to find a trade-off between the need for intuitive interpretation of the designed model and the requested application accuracy.

4. Conclusions

A novel framework has been presented that combines clinical information with HR and EEG measures to predict the neurodevelopmental outcome of newborns with HIE at 24 months. Signal processing techniques were used to create a large set of signal descriptors. It has been shown that 12 multimodal features provide promising prediction results across 38 newborns reaching ROC are of 86.8% and accuracy of 84%. The developed system represents a positive step towards the automated decision support tools for prediction of outcome in newborns.

Conflict of interest

None declared.

Acknowledgements

This work was supported by a Science Foundation Ireland Principal Investigator (10/IN.1/B3036) and Research Centres (12/RC/2272) Awards.

Appendix A. Supporting information

Supplementary data associated with this article can be found in the online version at http://dx.doi.org/10.1016/j.compbiomed.2015.05.017.

References

[1] J. Volpe, Neurology of the Newborn, Saunders, Philadelphia, USA, 2001.
[2] D. Murray, G. Boylan, C. Ryan, S. Connolly, Early EEG findings in hypoxic-ischemic encephalopathy predict outcome at 2 years, Pediatrics 124 (2009) 459–467.
[3] D. Sinclair, M. Campbell, P. Byrne, W. Prasertsom, C. Robertson, EEG and long-term outcome of term infants with neonatal hypoxic-iischemic encephalopathy, Clin. Neurophys. 110 (1999) 655–659.
[4] H. van Lieshout, J. Jacobs, J. Rotteveel, W. Geven, M. Hof, The prognostic value of the EEG in asphyxiated newborns, Acta Neurol. Scand. 91 (1995) 203–207.
[5] R. Pressler, G. Boylan, M. Morton, C. Binnie, J. Rennie, Early serial EEG in hypoxic ischaemic encephalopathy, Clin. Neurophysiol. 112 (2001) 31–37.
[6] V. Ramaswamy, J. Horton, B. Vandermeer, N. Buscemi, S. Miller, J. Yager, Systematic review of biomarkers of brain injury in term neonatal encephalopathy, Pediatr Neurol. 40 (2009) 215–226.
[7] A. Laptook, et al., Outcome of term infants using apgar scores at 10 minutes following hypoxic-iscemic encephalopathy, Pediatrics 124 (2009) 1619–1626.
[8] American Academy of Pediatrics, Committee on Fetus and Newborn, American College of Obstetricians & Gynecologists, Committee on Obstetric Practice, The Apgar score, Pediatrics 117 (2006) 1444–1447.
[9] B. Lingwood, G. Healy, Z. Kecskes, K. Dunster, P. Gray, L. Ward, P. Colditz, Prediction of outcome following hypoxic/ischaemia in the human infant using cerebral impedance, Clin. Neurophysiol. 120 (2009) 225–230.
[10] R. Jyoti, R. O'Neill, Predicting outcome in term neonates with hypoxic-ischaemic encephalopathy using simplified MR criteria, Pediatr. Radiol. 36 (2006) 38–42.
[11] J. Rennie, C. Hagmann, N. Robertson, Neonatal Cerebral Investigation, Cambridge University Press, New York, USA (2008) 130–172.
[12] A. Temko, G. Boylan, W. Marnane, G. Lightbody, Robust neonatal EEG Seizure detection through adaptive background modelling, Int. J. Neural Syst. 23 (4) (2013).
[13] O. Doyle, A. Temko, G. Lightbody, W. Marnane, G. Boylan, Heart rate based automatic seizure detection in the newborn, Med. Eng. Phys. 32 (2010) 829–839.
[14] J. Moorman, D. Lake, M. Griffin, Heart rate characteristics monitoring for neonatal sepsis, IEEE Trans. Biomed. Eng. 53 (2006) 26–32.
[15] H. Sarnat, M. Sarnat, Neonatal encephalopathy following fetal distress: a clinical and electroencephalographic study, Arch. Neurol. 33 (1976) 696–705.
[16] C. Amiel-Tison, Update of the Amiel-Tison neurological assessment for the term neonate or at 40 weeks corrected age, Pediatr. Neurol. 27 (2002) 196–212.
[17] R. Griffths, The Abilities of Babies, University of London Press, 1954.
[18] E. Thomas, A. Temko, W. Marnane, G. Boylan, G. Lightbody, Discriminative and generative classification techniques applied to automated neonatal seizure detection, IEEE J. Biomed. Health Inform. 17 (2) (2013) 297–304.
[19] S. Faul, A. Temko, W. Marnane, Age-independent seizure detection, Proc. IEEE Eng. Med. Biol. Soc. (2009) 6612–6615.
[20] J. Lofhede, M. Thordstein, N. Lofgren, A. Flisberg, M. Rosa-Zurera, I. Kjellmer, K. Lindecrantz, Automated classification of background EEG activity in healthy and sick neonates, J. Neural Eng. 7 (2010).
[21] M. van Putten, The revised brain symmetry index, Clin. Neurophysiol. 118 (2007) 2362–2367.
[22] A. Bell, B. McClure, E. Hicks, Power spectral analysis of the EEG of term infants following birth asphyxia, Dev. Med. Child Neurol. 32 (1990) 990–998.
[23] P. Hamilton, W. Tompkins, Quantitative investigation of QRS detection rules using the MIT/BIH arrhythmis database, IEEE Trans. Biomed. Eng. 33 (1986) 1157–1165.
[24] M. Malik, J. Bigger, A. Camm, R. Kleiger, A. Malliani, A. Moss, P. Schwartz, Heart rate variabiltiy: standards of measurement, physiological interpretation and clinical use. Task force of the european society of cardiology and the north american society of pacing and electrophysiology, Eur. Heart J. 17 (1996) 354–381.
[25] P. De Chazal, C. Heneghan, E. Sheridan, R. Reilly, P. Nolan, M. O'Malley, Automated processing of the single-lead electrocardiogram for the detection of obstructive sleep apnoea, IEEE Trans. Biomed. Eng. 50 (2003) 686–696.
[26] O. Doyle, I. Korotchikova, G. Lightbody, W. Marnane, D. Kerins, G. Boylan, Heart rate variability during sleep in healthy term newborns in the early postnatal period, Physiol. Meas. 30 (2009).
[27] B. Kovatchev, L. Farhy, H. Cao, M. Griffin, D. Lake, J. Moorman, Sample asymmetry analysis of heart rate characteristics with application to neonatal sepsis and systemic inflammatory response syndrome, Pediatr. Res. 54 (2003) 892–898.
[28] M. Toichi, T. Sugiura, T. Murai, A. Sengoku, A new method of assessing caridac autonomic function and its comparison with spectral analysis and coefficient of variation of RR interval, J. Auton. Nerv. Syst. 62 (1997) 79–84.

[29] N. Twomey, A. Temko, J. Hourihane, W. Marnane, Fully-automated allergy detection from paediatric ECG, IEEE J. Biomed. Health Inform. 18 (2014) 1051–1057.
[30] J. Platt, Probabilistic outputs from SVM and comparison to regularized likelihood methods in: Alexander J. Smola, Peter Bartlett, Bernhard Schoelkopf, Dale Schuurmans (Eds.), Advances in Large Margin Classifiers, pp. 61–74, MIT Press, Massachusetts, USA, 1999.
[31] I. Guyon, J. Weston, S Barnhill, Gene selection for cancer classification using support vector machines, Mach. Learn. 46 (2002) 389–422.
[32] V. Vapnik, Estimation of Dependences Based on Empirical Data, Springer-Verlag, New York, 1982.
[33] A. Temko, C. Nadeu, W. Marnane, G. Boylan, G. Lightbody, EEG signal description with spectral-envelope-based speech recognition features for detection of neonatal seizures, IEEE Trans. Inf. Technol. Biomed. 15 (2011) 839–847.
[34] E. Niedermeyer, F. Lopes Da Silva, Electroencephalography: Basic Principles, Lippincott Clinical Applications and Related Fields, Philadelphia, USA, 2004.
[35] I. Guyon, C. Aliferis, G. Cooper, A. Elisseeff, J. Pellet, P. Spirites, A. Statnikov, Design and analysis of the causation and prediction challenge, J. Mach. Learn. Res. 3 (2008) 1–33.
[36] B. McNeil, E. Keeler, S. Adelstein, Primer on certain elements of medical decision making, N. Engl. J. Med. 293 (1975) 211–215.
[37] A. Temko, G. Boylan, W. Marnane, G. Lightbody, Robust neonatal EEG classification through adaptive background modelling, Int. J. Neural Syst. v. 23 (4) (2013).
[38] N. Stevenson, I. Korotchikova, A. Temko, G. Lightbody, W. Marnane, G. Boylan, An automated system for grading EEG abnormality in term neonates with hypoxic-ischaemic encephalopathy, Ann. Biomed. Eng. 41 (2013) 775–785.
[39] J. Lofhede, N. Lofgren, M. Thordstein, A. Flisberg, I. Kjellmer, K. Lindecrantz, Classification of burst and suppression in the neonatal electroencephalogram, J. Neural Eng. 5 (2008) 402–410.
[40] B. Vergales, S. Zanelli, J. Matsumoto, H. Goodkin, D. Lake, J. Moorman, K. Fairchild, "Depressed heart rate variability is associated with abnormal EEG, MRI, and death in neonates with hypoxic ischemic encephalopathy," Am. J. Perinatol., 2014.
[41] R. Ahmed, A. Temko, W. Marnane, G. Boylan, G. Lightbody, Grading Brain Injury in Neonatal EEG Using SVM and Supervector Kernel, in Proceedings of the IEEE International Conference on Acoustics, Speech, and Signal Processing, ICASSP'14, Florence, Italy, May 2014.
[42] M. Zweig, G. Campbell, Receiver-operating characteristics (ROC) plots: a fundamental evaluation tool in clinical medicine, Clin. Chem. 39 (4) (1993) 561–577.
[43] J. Hanley, B. McNeil, The meaning of use of the area under a receiver operating characteristic (ROC) curve, Radiology 143 (1) (1982) 29–36.
[44] A. Temko, W. Marnane, G. Boylan, G. Lightbody, Clinical implementation of a neonatal seizure detection algorithm, Decis. Support Syst. 70 (2015) 86–96.

Andriy Temko received the Engineering degree in Informatics in 2002 from Dniepropetrovsk National University, Dniepropetrovsk, Ukraine and the PhD degree in Telecommunication in 2008 from Universitat Politècnica de Catalunya (UPC), Barcelona, Spain. His main research interests include kernel methods, signal processing, and multimodal interfaces. During 2006–2007 he was a task leader in detection and classification of acoustic events within the EU-funded international evaluation campaigns on detection of events, activities, and their relationships (CLEAR 2006/CLEAR 2007). Since late 2008 he has been with the Neonatal Brain Research Group, University College Cork, Ireland, working on algorithms for EEG and ECG based detection of seizures in newborns and adults. He has been involved in several EU and national governments funded projects on speech and biomedical signal processing. He is a senior member of IEEE.

Orla Doyle received a BE (Hons) 1st class in electrical and electronic engineering from University College Cork, Ireland in 2006 and her Ph.D. from University College Cork in 2010 on biomedical signal processing of neonatal physiological data. Her thesis investigated the usage of statistical pattern recognition of physiological signals for automated monitoring in newborns. Since 2010 she has been with the Centre for Neuroimaging Sciences, Institute of Psychiatry, King's College London, where she is currently a research fellow specialising in the development of machine learning methods for neuroimaging data. Her main research interests include machine learning for medical data and signal processing.

Deirdre Murray is a Consultant Paediatrician and Senior Lecturer in the Department of Paediatrics and Child Health, University College, Cork. Deirdre qualified from University College Cork in 1995, and completed subspecialist paediatric training in Paediatric Intensive Care Medicine in Bristol Royal Hospital for Sick Children, and as a Paediatric Intensive care Fellow in the Royal Children's Hospital, Melbourne from 2003–2004. She completed her PhD thesis on neurodevelopmental outcome in hypoxic-ischaemic encephalopathy in 2008. For the last 5 years, Dr Murray's research has focused on early brain injury, and the development of new ways to predict and assess neurocognitive outcome in children.

Gordon Lightbody graduated with the MEng degree (distinction) (1989), and then PhD (1993) both in Electrical and Electronic Engineering from Queen's University Belfast. After completing a one year Post-Doctoral position funded by Du Pont, he was appointed by Queen's University as a Lecturer in Modern Control Systems. In 1997 he was appointed as a Lecturer in Control Engineering at University College Cork, and subsequently promoted to Senior Lecturer in 2008. His current research interests include artificial intelligence techniques for intelligent control and signal-processing, focusing on biomedical and energy/power applications. He is a member of the IET, and is currently an associate editor with the Elsevier journal, "Control Engineering Practice"

Geraldine Boylan received the M.Sc. degree in physiology and the Ph.D. degree in clinical medicine from University College London, London, U.K. She worked as a Clinical Scientist in Neonatal Medicine in Kings College Hospital London from 1996–2001. She is currently a Professor in the Department of Paediatrics & Child health, University College Cork, Cork, Ireland. Her research interests concentrate on accurately diagnosing seizures or "fits" in newborn babies by monitoring electrical brain activity and studies of blood flow regulation during neonatal seizures. Much of her more recent work is of an interdisciplinary nature and aims to create a synergy between medicine and engineering by using the skills and techniques of engineering signal processing research to address important medical problems such as seizure detection in the neonate.

William Marnane received the B.E. degree in electrical engineering from the National University of Ireland, Cork, in 1984, and the Ph.D. degree from the University of Oxford, Oxford, U.K., in 1989. He was a lecturer at the School of Electronic Engineering Science, University of Wales, Bangor from 1989 to 1993. In 1992 he was a Visiting Researcher and Marie Cure Fellow at the Institute de Recherche en Informatique et Systemes Aleatoires, at the University of Rennes, France. In 1993 he was appointed as a Lecturer in Digital Signal Processing in the Department of Electrical & Electronic Engineering at University College Cork and as a Senior Lecturer in 1999. In 1999 he was a visiting researcher to the Electronic Devices Research Group, Department of Physics, University of Linköping. His research interests include Biomedical Signal Processing and digital design for DSP, coding and cryptography.
