# Supplementary material for: Exploring Computational Techniques in Preprocessing Neonatal Physiological Signals for Detecting Adverse Outcomes: Scoping Review
Source: Interact J Med Res. 2024 Aug 20;13:e46946. doi: 10.2196/46946 (PMC11372324; doi:10.2196/46946)
Supplement: Multimedia Appendix 3 [file ijmr_v13i1e46946_app3.zip › Included Papers - Final/3610/Schiavenato et al. - 2013 - Development of a System for the Assessment of Hear.pdf]

Development of a System for the Assessment of Heart Rate Variability in the Neonatal Intensive Care Unit (NICU)

Martin Schiavenato, Carlos Oliu, Edward Bello, Jorge Bohórquez and Nelson Claure

Abstract— **Heart rate variability (HRV) analysis is used to examine morbidity and mortality in premature neonates. We developed a system to acquire and analyze full frequency spectrum HRV based on ECG signal to identify frequency ranges associated with responses to "normal care" of the NICU neonate. The system will allow real-time monitoring of specific frequency ranges at the bedside. Twenty NICU newborns were enrolled under University of Miami IRB approved protocol. Infants were recorded before, during and after procedures associated with normal NICU care. ECG signals were sampled from analog output from a standard bedside monitor using a custom interface based in an Arduino system. Interface was connected to a computer running a LabView program for real-time process. Pam-Tompkins QRS detection algorithm was tuned to spectral characteristics of the preterm ECG signal; HRV computation was done using the Lomb-Scargle algorithm. ECG acquisition was safe, reliable and appropriate for HRV computation. 1186 minutes of ECG were sampled at 1000SPS and processed. The QRS detection algorithm was reliable, requiring minor intervention to eliminate artifacts. HRV computation using non-uniformly spaced samples was appropriate for ECG analysis. HRV can be used in real time to monitor neonatal response to normal care.**

I. Introduction

The physiologic vulnerability of prematurity coupled with the distress associated with life-saving/life-sustaining procedures underscore the need for valid pain assessment in infants in the newborn intensive care unit (NICU). Current tools to assess pain in this population include standardized observational scales applied clinically based on individualized judgment and clinician expertise [1]; however, variability associated with clinician subjectivity may introduce bias. Heart rate variability (HRV) has recently been examined as an objective means to quantify various morbidities in this population [2, 3]. We set out to develop a system to record ECG and calculate HRV in the frequency domain to monitor infant response to routine NICU care, including painful procedures, in real-time.

II. Methods

A. Subjects

A convenience sample of NICU infants 28-31 weeks of gestational age (GA), purposefully sampled by sex (half female) were enrolled under University of Miami IRB approved protocol. Infants with major congenital anomalies, severe brain injury, documented seizures, septic, or infants exposed to maternal illicit drug use during pregnancy were excluded. Each "procedural epoch" per infant consisted of ECG records of procedures considered "normal care" of the NICU infant including physical assessment, diaper change, feeding, heel-stick, etc. We aimed to capture baseline readings (at least 3 minutes prior to initiation of any procedure), recording through procedures, and up to at least 5 minutes after conclusion of last procedure.

B. Electrocardiogram (ECG) Acquisition

To avoid interfering with the newborn care setting, a custom board, based on Arduino architecture, was designed to interface the newborn monitor (GE Dash 3000) and a computer running the signal analysis and recording software. The monitor has available an amplified (1000 times) analog replica of the patient ECG. The interface board samples the signal at 1KSPS, 10 bits and transmits it using USB connectivity.

C. Real Time Monitoring System Design

The monitoring software is a Virtual Instrument implemented in LabView (Fig. 1). The instrument receives the ECG, detects the QRS and updates the HRV approximately every minute (since a fixed number of beats is used in the HRV computation, the HRV update time can vary). Before the procedure, when the patient is presumably at lower distress levels, HRV spectrum is stored as baseline. The deviation of the HRV from the baseline controls the color of a visual output that is used as a distress indicator. The software saves the complete raw ECG data, the low frequency HRV (LF-HRV) and allows the operators to store time stamped events for offline analysis. The system is designed to keep a patient database updated that will allow the periodic releases in improved algorithms.

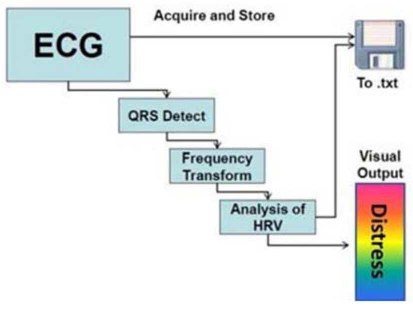

Figure 1. HRV based distress monitoring system

Research supported by the Robert Wood Johnson Foundation, Nurse Faculty Scholars Program.
M. Schiavenato*[1,2] , C.Oliu[2], E.Bello[2], J.Bohórquez[2] ,N. Claure[3]
*Corresponding author: Tel. 305-284-6379; fax 305-284-4221; email:m.schiavenato@miami.edu.;[1]Univ. of Miami School of Nursing and Health Studies, [2]Univ. of Miami College of Engineering, Dept. Biomedical Eng., Coral Gables, FL, 33146 USA.[3]Univ. of Miami Miller School of Medicine, Division of Neonatology, Miami, FL 33136 USA.

978-0-7695-5032-9/13 $26.00 © 2013 IEEE
DOI 10.1109/SBEC.2013.21

IEEE computer society

Authorized licensed use limited to: CSIRO Information Technology Services. Downloaded on September 09,2022 at 00:23:08 UTC from IEEE Xplore. Restrictions apply.

D. Detailed ECG Signal Processing

The original Pam-Tompkins QRS detection algorithm, [4] optimized for computational efficiency and high signal to noise ratio (SNR) of adult QRS complex was customized to account the high frequencies of the neonatal ECG (Fig. 2).

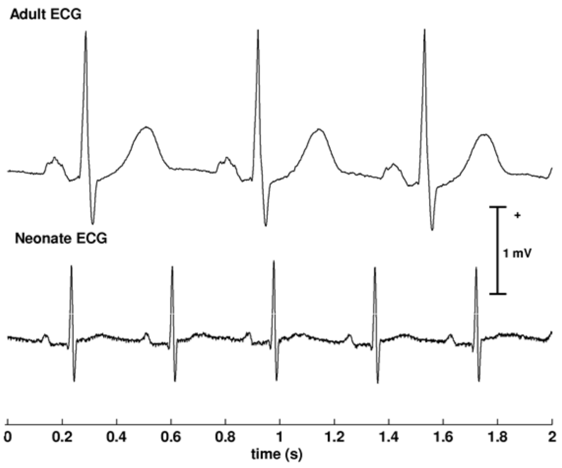

Figure 2. Example of 2 seconds of ECG acquired on an adult and a neonate. Note the high frequency components in the newborn QRS ECG, compared with the adult one.

The Pam-Tompkins algorithm uses a cascade of low-pass (LPF), high-pass (HPF) and differentiator filters to produce an equivalent band-pass filter (BPF) with bandwidth 5-15Hz. The signal is then squared and filtered with a moving average filter. The ECG digital filter design was modified to produce a BPF of 16-26 Hz which is significantly higher than the one original algorithm, tuned for adults. The LPFwas implemented by an order 120 FIR filter with corner frequency of 25 Hz; the HPF is a FIR filter, order 160 with a corner frequency of 15 Hz; finally, the differentiator filter is polynomial filter of order 21. A 111 order moving average filter was used after the square operation. The QRS was detected using an adaptive threshold on the processed ECG.

Each detected QRS produce a couple of numbers: the time stamp and the difference with the previous QRS (RR time). If there are missing detections (amplifier saturation or excessive noise, for example) it is necessary to wait two consecutive QRS detections to produce a valid RR sample. The time series of valid RR intervals was used as input to the HRV spectrum computation. Because of the possibility of missing samples and irregular sampling, a conventional Fourier Transform approach was not used. Instead, a matlab implementation by C. Saragiotis of the Lomb-Scargle LMS spectral estimation method was adapted to this study [5]. This method allows for irregular sampling periods; it is very resilient to missing data and produces a statistical significance value for each spectral peak. Windows with 128 RR samples, 64 samples overlap, were used for the estimation of the LF-HRV (0.05-0.25Hz). A new LF-HRV was then obtained every 30 seconds, approximately, depending on the infant's HR.

III. Methods

Twenty infants were enrolled (10 female). Race/ethnicity distribution was: 11 White Hispanic; 4 Black American; 4 Black Caribbean; and 1 non-White Hispanic. Mean GA was 30 weeks. Mean post-natal age was 4 days. A total of 1186 minutes of ECG data were recorded and processed in real time. The data are now being re-analyzed a-posteriori to improve the performance of the programs. Fig. 3 shows an example of the analysis of 30 minutes of ECG. We can differentiate three main periods: a baseline with LF-variability followed by two apneic events resulting in bradycardia around 17 and 25 min. The period between the two bradycardic events presents of very low LF-HRV.

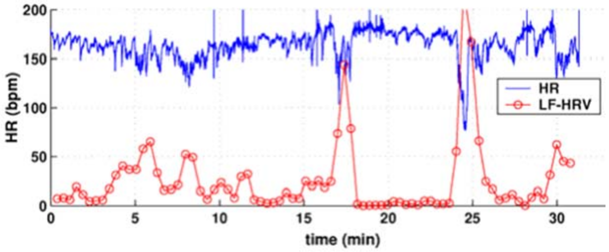

Figure 3. HRV example on a newborn LF-HRV analysis.

IV. Discussion

This work presents the implementation of a system that enables researchers to investigate the correlation of HRV with usual care of the NICU infant. Despite the difficult recording conditions, a reliable, non intrusive system was developed. The system performs a real-time analysis of the LF-HRV and saves the complete set of data for off-line analysis. The data base generated in the study is being currently used to optimize the detection methods and to investigate what are the most appropriated ECG characteristics correlated with periods of high distress/pain.

Acknowledgment

We thank the nurses (clinicians, educators and managers) at Jackson Memorial Hospital NICU for their patience and collaboration. We also thank Johanna Kelly, Carmen D'Ugard and Drs. Shahnaz Duara and Eduardo Bancalari for their contribution and support.

References

[1] T. Blauer and D. Gerstmann, "A simultaneous comparison of three neonatal pain scales during common NICU procedures," *Clin. J. Pain*, vol. 14, pp. 39-47, Mar, 1998.

[2] M. P. Griffin, D. E. Lake, E. A. Bissonette, F. E. Harrell Jr, T. M. O'Shea and J. R. Moorman, "Heart rate characteristics: novel physiomarkers to predict neonatal infection and death," *Pediatrics,* vol. 116, pp. 1070-1074, Nov, 2005.

[3] P. M. Faye, J. De Jonckheere, R. Logier, E. Kuissi, M. Jeanne, T. Rakza and L. Storme, "Newborn infant pain assessment using heart rate variability analysis," *Clin. J. Pain*, vol. 26, pp. 777-782, Nov-Dec, 2010.

[4] J. Pan and W. Tompkins, "A real-time QRS detection algorithm," IEEE Trans. Biomed. Eng., vol. BME-32, pp. 230–236, Mar. 1985.

[5] Lomb, N. R. (1976). Least-squares frequency analysis of unequally spaced data. Astrophysics and Space Science, 39, 447–462.

Authorized licensed use limited to: CSIRO Information Technology Services. Downloaded on September 09,2022 at 00:23:08 UTC from IEEE Xplore. Restrictions apply.
