# Supplementary material for: Exploring Computational Techniques in Preprocessing Neonatal Physiological Signals for Detecting Adverse Outcomes: Scoping Review
Source: Interact J Med Res. 2024 Aug 20;13:e46946. doi: 10.2196/46946 (PMC11372324; doi:10.2196/46946)
Supplement: Multimedia Appendix 3 [file ijmr_v13i1e46946_app3.zip › Included Papers - Final/3630/Sadoughi et al. - 2021 - Detection of Apnea Bradycardia from ECG Signals of.pdf]

Annals of Biomedical Engineering, Vol. 49, No. 9, September 2021 (© 2021) pp. 2159–2169
https://doi.org/10.1007/s10439-021-02732-z

Original Article

Detection of Apnea Bradycardia from ECG Signals of Preterm Infants Using Layered Hidden Markov Model

AZADEH SADOUGHI,[1] MOHAMMAD BAGHER SHAMSOLLAHI,[2] EMAD FATEMIZADEH,[2] ALAIN BEUCHÉE,[3] ALFREDO I. HERNÁNDEZ,[3] and NASIM MONTAZERI GHAHJAVERESTAN[4,5]

[1]Faculty of Medical Sciences and Technologies, Science and Research Branch, Islamic Azad University, Tehran, Iran; [2]Biomedical Signal and Image Processing Laboratory (BiSIPL), School of Electrical Engineering, Sharif University of Technology, Tehran, Iran; [3]Univ Rennes, CHU Rennes, Inserm, LTSI - UMR 1099, 35000 Rennes, France; [4]KITE - Toronto Rehabilitation Institute, University Health Network, Toronto, ON, Canada; and [5]Institute of Biomaterials and Biomedical Engineering, University of Toronto, Toronto, ON, Canada

(Received 10 July 2020; accepted 13 January 2021; published online 26 February 2021)

Associate Editor Ka-Wai Kwok oversaw the review of this article.

Abstract—Apnea-bradycardia (AB) is a common complication in prematurely born infants, which is associated with reduced survival and neurodevelopmental outcomes. Thus, early detection or predication of AB episodes is critical for initiating preventive interventions. To develop automatic real-time operating systems for early detection of AB, recent advances in signal processing can be employed. Hidden Markov Models (HMM) are probabilistic models with the ability of learning different dynamics of the real time-series such as clinical recordings. In this study, a hierarchy of HMMs named as layered HMM was presented to detect AB episodes from pre-processed single-channel Electrocardiography (ECG). For training the hierarchical structure, RR interval, and width of QRS complex were extracted from ECG as observations. The recordings of 32 premature infants with median 31.2 (29.7, 31.9) weeks of gestation were used for this study. The performance of the proposed layered HMM was evaluated in detecting AB. The best average accuracy of $97.14 \pm 0.31\%$ with detection delay of -5.05 ± 0.41 s was achieved. The results show that layered structure can improve the performance of the detection system in early detecting of AB episodes. Such system can be incorporated for more robust long-term monitoring of preterm infants.

Keywords—Hidden Markov Model, Apnea-Bradycardia, Electrocardiography, Machine learning, Early detection.

Address correspondence to Mohammad Bagher Shamsollahi, Biomedical Signal and Image Processing Laboratory (BiSIPL), School of Electrical Engineering, Sharif University of Technology, Tehran, Iran. Electronic mail: mbshams@sharif.edu

ABBREVIATIONS

AB	Apnea-bradycardia
NO	Normal
NICU	Neonatal intensive care unit
ECG	Electrocardiography
HMM	Hidden Markov Model
LHMM	Layered Hidden Markov Model
CHMM	Coupled Hidden Markov Model
HsMM	Hidden semi-Markov Model
CHSMM	Coupled Hidden semi-Markov Model
ACC	Accuracy
SEN	Sensitivity
SPC	Specificity
ROC	Receiver operating characteristic
AUC	Area under the ROC curve
PD	Perfect detection
DPD	Distance to PD

INTRODUCTION

Apnea of prematurity is a common disorder in preterm infants (born before 37 weeks of gestation) and is defined as a respiratory pause (apnea) for more than 15–20 s. In severe cases, apnea of prematurity is followed by a significant reduction in heart rate. These episodes are commonly denoted apnea-bradycardia (AB). In preterm infants, the occurrence of AB causes

0090-6964/21/0900-2159/0 © 2021 Biomedical Engineering Society

hypoxia (low oxygenation of blood), which can be detrimental in the long run, leading to developmental problems.[21,25] Thus, early detection of AB helps with early clinical intervention and lowering the adverse consequences.

In preterm infants, AB is commonly detected through monitoring of respiration and oxygen saturation in blood.[3,13] However, the related sensors for recording these signals can be irritating and may affect the natural breathing in neonates. In contrast, the electrical activity of the heart, Electrocardiography (ECG) signal, can be recorded comfortably using weight less chest leads. Thus, a number of studies have analyzed ECG for detection of AB. In studies, by Poets and Portet, AB episodes were detected by extracting RR interval, representing cardiac period, from ECG signals and comparing their instantaneous values with a fixed or relative threshold.[21,22] Although threshold based algorithms provide high specificities (few false alarms), they suffer from low sensitivities, 34.57%, (few true alarms) and high delays, 6.35 s.[8]

In addition to RR interval, previous studies have reported significant changes in the amplitude and width of QRS complex, representing the depolarization of the ventricles, at the onset of AB.[16] By incorporating these features, McNames *et al.* were able to improve the accuracy of AB detection to 92.6%. In this regard, to analyze the dynamic and pattern of the signals rather than just their amplitude, more complex approaches have been developed such as: Point process analysis,[9] Machine learning techniques,[26,29] Hidden Markov Model (HMM) and its generalizations.[4,5,7,10,15,17]

HMM is a statistical model with finite number of unobservable or hidden states that produces a sequence of observations as Markov process (a change in the next state depends on the current state).[23]

For early detection of AB, Altuve *et al.*[4,5,7] proposed two different methods based on HMM and Hidden semi-Markov Model (HsMM) using RR interval series of normal (NO) and AB episodes. Coupled Hidden Markov Model (CHMM) and Coupled Hidden semi-Markov Model (CHSMM) have been also used to detect AB using RR intervals, QRS duration and amplitude series.[10,15,17] These studies successfully improved the average sensitivity and specificity of detection to 95.99 and 93.84%, respectively with time delay of − 1.11 s. These results are proof of concept that AB episodes can be early detected.

In this study, to detect AB episodes, we proposed a hierarchical model based on HMM, called Layered Hidden Markov Model (LHMM). LHMM was proposed by Oliver *et al.*[18] to decompose the parameter space in a way to enhance the model robustness, while reducing training expenses. LHMM has been used in different applications such as human activity recognition,[11,14,20] intension recognition,[1,2,12,24] event detection and prediction.[27,28] Instead of using a huge HMM, LHMM creates a hierarchy of HMMs in different layers. At each layer of this hierarchy, there is a set of HMMs each of which is related to a dynamic. The input of each layer is the output of the previous one. At different layers, the observations are analyzed in different time scales defined as "time granularity". The time granularity indicates the length of a sliding window that segments the sequence of observations. One of the advantages of LHMM is that each layer can be trained and evaluated separately, which decreases the risk of overfitting.[18] The inputs of the first layer are the observations, while the inputs of the following layers are the inferential outputs of the previous layer.

In this study, a model based on LHMM was constructed, trained and evaluated to detect AB episodes. This paper is organized as follows: in "Materials and Methods" section, an overview of the data used in this study and the details of the proposed method as well as the optimization and evaluation methodology are presented. The results obtained are exposed in "Results" section. Finally, the discussion and conclusion are outlined in "Discussion" section.

MATERIALS AND METHODS

Database

Thirty-two premature infants hospitalized in the neonatal intensive care unit (NICU) who presented more than one bradycardia per hour or the need for bag-and-mask resuscitation were included for this study. The exclusion criteria were the usage of drugs known to influence the autonomic nervous system (ANS) except for caffeine, being on intra-tracheal respiratory aid, and diagnosed intra-cerebral lesion or malformation. At the time of recording, the median birth weight was 1235 (1065-1360) gr, the median age was 31.2 (29.7-31.9) weeks, and the postnatal age was 12.1 (6.7-19.5) days. The infants were placed in incubators, positioned on their side, wrapped in a single blanket. The parents who accepted to participate their infant in this study signed a consent form. The same database was used in previous works.[4–7,10,15,17]

The same preprocessing and feature extraction were performed as in previous studies.[4,5,7,10,15,17] The data contained 236 one-lead ECG recordings, for each of which, QRS complexes were identified using Pan and Tompkins's algorithm.[19] Then, the distance between two consecutive R peaks (RR) and the width of the QRS complex (QRSd) were extracted. Every instant was

marked as bradycardia or non-bradycardia by a clinical expert in the field. After clinical annotation, for more precise annotation of the AB onset, a sigmoid function with the best reproducibility was fitted to the rising segment of the RR time series around the annotated time instants of the AB episode. The AB onset corresponded to the first point at which the derivation of the sigmoid function is more than 1. Using the linear interpolation technique, the obtained time series for RR and QRSd were uniformly upsampled to 10 Hz to obtain sufficient time resolution for further analysis. Out of 236 recordings, 148 of them, with length of 26.25 ± 11.37 min, were selected due to their sufficient length, less noise, and less interruptions in data acquisition. The selected data includes 233 AB episodes with duration of 21.48 ± 16.07 s. Among the selected time series, 53 of them were void of any AB episode, while 41 and 54 time series had one and more than one episode, respectively. For more information about the database see.[6] Authors have agreed to make data and materials supporting the results or analyses presented in this paper available upon reasonable request.

Methodology

All the analyses were performed in Matlab (2018b, The MathWorks Inc., Natick, MA, USA) software.

LHMM Structure

In order to detect AB episodes using LHMM, a two-layered hierarchy of HMMs was proposed as shown in Fig. 1. The first layer was designed to include two HMM banks to separately analyze RR (B_{RR}) and QRSd (B_{QRSd}). Each HMM bank itself consisted of two continuous density HMMs,[23] one to be trained by AB segments and another one by NO segments. Each segment of data had $T_1 = 7$ s duration extracted by a window that moves sample by sample. The 7-second was selected for the training of the models in the first layer based on the analysis in previous studies,[5,7] in which the length of the analysis segment was defined as the average of the elapsing time between the onset of AB episodes and the corresponding peak in RR signal over the 233 AB episodes.

For each segment of observation, $O_{t-T_1+1:t}$, the HMM of class k, $k \in \{\mathrm{AB}, \mathrm{NO}\}$, in bank B, $B = B_{\mathrm{RR}}, B_{\mathrm{QRSd}}$, characterized by model parameters set λ_k^{B}, generates a likelihood value as its output:

$$ll_k^{\mathrm{B}}(t) = \log P\left(O_{t-T_1+1:t} | \lambda_k^{\mathrm{B}}\right). \quad (1)$$

The likelihood of a dynamic (class) represents the probability of the segment being generated by that dynamic, for which model λ_k^{B} is trained. For comparing the likelihoods generated by the two models inside a bank B, the difference log-likelihood was calculated as:

$$ll_{\mathrm{diff}}^{\mathrm{B}}(t) = ll_{\mathrm{AB}}^{\mathrm{B}}(t) - ll_{\mathrm{NO}}^{\mathrm{B}}(t). \quad (2)$$

To detect the AB episodes, a threshold can be applied to each sample of $ll_{\mathrm{diff}}^{\mathrm{B}}(t)$, as:

$$ll_{\mathrm{diff}}^{\mathrm{B}}(t) \geq \delta_{\mathrm{B}}. \quad (3)$$

Two different thresholds were used for the banks related to RR and QRSd: $\delta_{\mathrm{B_{RR}}}, \delta_{\mathrm{B_{QRSd}}}$,respectively. For the samples lower than the threshold, it was concluded that they were not as part of AB episodes. Through applying the thresholds to the output of each bank of the first layer ($ll_{\mathrm{diff}}^{\mathrm{B}}(t)$), two binary sequences were generated and used as the inputs to the second layer.

The purpose of the second layer is to make the final decision of detecting the AB episodes by integrating and analyzing the two outputs of the first layer with higher time granularity. For this purpose, the outputs of each bank in the first layer were synchronously segmented with sliding windows with $T_2 = 14$ s length and stride of 1 sample. The length of the sliding window in the second layer was chosen by greedy search. To construct the second layer, one bank (B_{II}) including two discrete HMMs (one for class AB and another for class NO) was used.

Similar to the first layer, the log-likelihood of each sliding window ($O_{t-T_2+1:t}$) for each HMM at time t was calculated, and the difference of the two log-likelihoods generated was compared to a threshold($\delta_{B_{\mathrm{II}}}$). A window was labeled as AB episode if the following condition was met:

$$ll_{\mathrm{diff}}^{B_{\mathrm{II}}}(t) \geq \delta_{B_{\mathrm{II}}}, \quad (4)$$

otherwise, it was labeled as NO recording.

The Optimization and Evaluation

The parameters ($\lambda_k^{B_{\mathrm{RR}}}, \lambda_k^{B_{\mathrm{QRSd}}}, \lambda_k^{B_{\mathrm{II}}}, k \in \{\mathrm{AB}, \mathrm{NO}\}$) required for development of the proposed LHMM structure and the detection thresholds ($\delta_{B_{\mathrm{RR}}}, \delta_{B_{\mathrm{QRSd}}}, \delta_{B_{\mathrm{II}}}$) were optimally determined. To optimize the parameters and to evaluate the performance of the proposed method in AB detection, the extracted time series ($N = 148$) were randomly grouped into two sets: 48 and 100 time series for optimization and evaluation datasets, respectively. The flow diagram of the data division in this study is shown in Fig. 2.

Out of 48 time series in the optimization set, 24 time series were randomly selected and divided into two subsets 15 and 9 time series for training and validation in the first layer, respectively. A training phase was

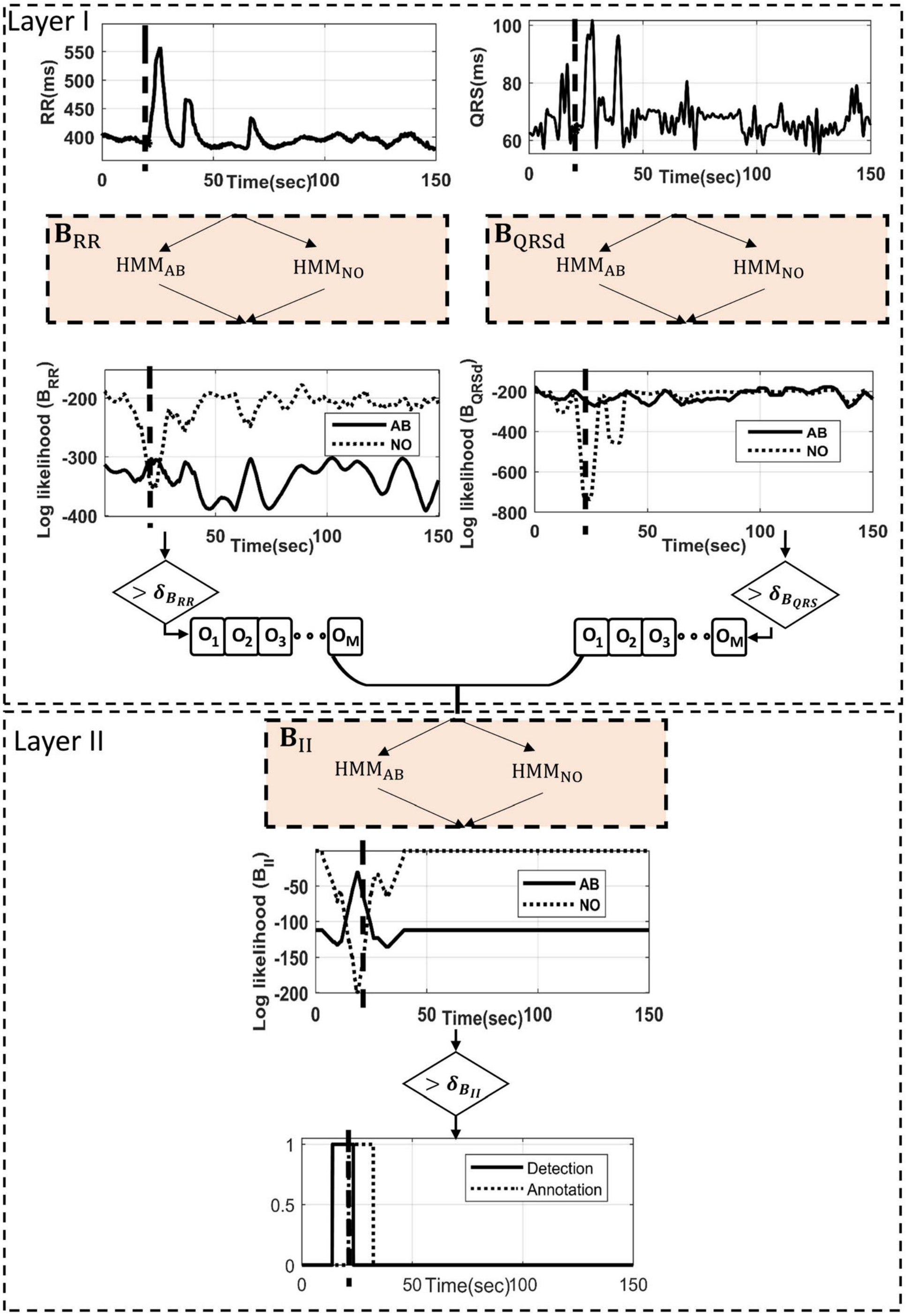

FIGURE 1. The proposed LHMM structure for AB detection.

applied to estimate parameters of each HMM using training observation dataset.

In the first layer, two datasets were constructed for the training phase; depending on the number of AB episodes in the 15 time series, segments were synchronously selected from the RR and QRSd time series starting from the onset of AB episodes with a duration of $T_1 = 7$ s for each AB model in the two banks (B_{RR}, B_{QRSd}). To construct training data for NO models at both banks (B_{RR}, B_{QRSd}), 300 segments of NO parts void of AB with a duration of $T_1 = 7$ s were randomly chosen from the 15 time series.

To determine the optimal states of each HMM, a set of number of states, {3, 4, …, 9}, were investigated. Then, the selected segments were used to train AB and NO models in relevant banks with different combina-

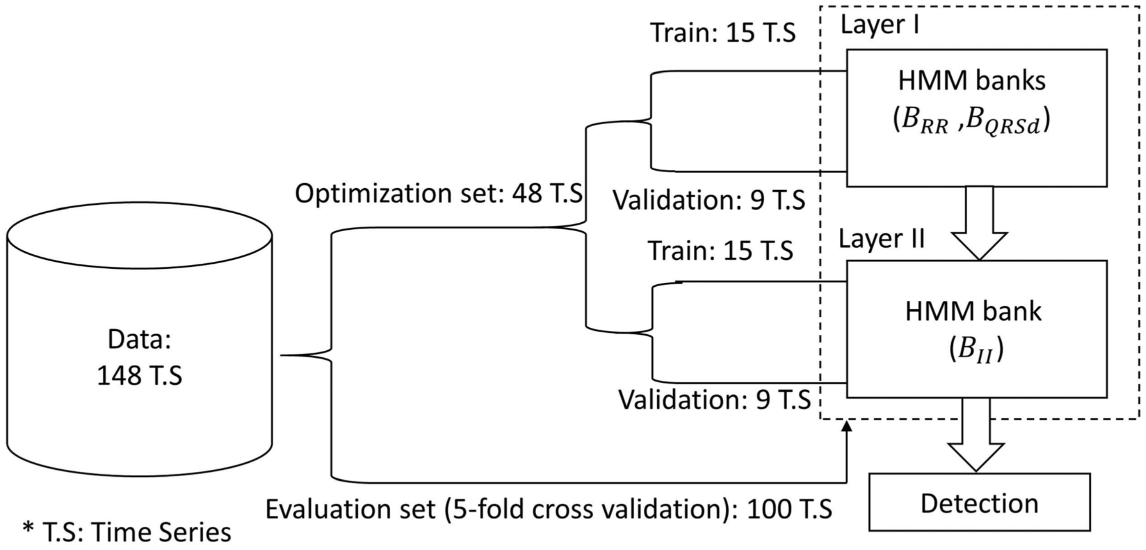

FIGURE 2. Flow diagram of data division.

tion of states. To optimize the log-likelihood thresholds ($\delta_{B_{RR}}, \delta_{B_{QRSd}}$), a range of 110 values from -100 to 1000 with interval 10 was investigated. In each bank, the difference of two log-likelihoods generated by the models was compared to each threshold (Eq. (3)), and metrics such as accuracy (ACC, Eq. (5)), sensitivity (SEN, Eq. (6)) and specificity (SPC, Eq. (7)) were calculated for each value of the thresholds:

$$\mathrm{ACC} = (\mathrm{TP} + \mathrm{TN})/(\mathrm{TP} + \mathrm{TN} + \mathrm{FN} + \mathrm{FP}), \quad (5)$$

$$\mathrm{SEN} = \mathrm{TP}/(\mathrm{TP} + \mathrm{FN}), \quad (6)$$

$$\mathrm{SPC} = \mathrm{TN}/(\mathrm{TN} + \mathrm{FP}), \quad (7)$$

where TP, FP, TN and FN denote the number of true positives, false positives, true negatives and false negatives, respectively. Using SEN and error (1-SPC) for different thresholds, Receiver Operating Characteristic (ROC) curves were used to find optimal threshold value and states at each bank. The ROC curves were created for models with different combinations of states. Using a criterion called Perfect Detection (PD) defined as:

$$\mathrm{PD} = \arg\max\{\mathrm{SEN} \times \mathrm{SPC}\}, \quad (8)$$

the point with optimum detection performance and its related threshold were chosen for each model.[10,17] Finally, states and threshold of the model with the maximum perfect detection among the models were chosen as the optimum parameters. For more comparison, the area under the ROC curve (AUC) and the distance to PD (DPD) were reported. DPD was defined as:

$$\mathrm{DPD} = \sqrt{(1 - \mathrm{SEN})^2 + (1 - \mathrm{SPC})^2}. \quad (9)$$

The remaining 24 time series of the optimization set were used to determine the parameters of HMMs in the second layer and were divided into two subsets 15 and 9 time series for training and validation, respectively. At the training phase, two approaches were considered to train HMMs (shown in Fig. 3):

1. Prior-segment approach, where the models were trained using segments selected before the onset of the desired event while their last sample fell inside the event of interest.
2. Onset-segment approach, which is to train models with segments that were selected within the onset and termination of the event.

According to each approach, two datasets were constructed from optimization observation dataset. Depending on the number of AB episodes in the 15 time series, the 14-second segments for AB model of the second layer were synchronously selected from the outputs of the first layer banks and training data were constructed. For the NO model of the second layer, 14-second segments of the first layer outputs were randomly selected. The number of selected segments for NO model was chosen equal to the number of data in AB related model. To determine the sliding window length in the second layer by greedy search, values from 9 to 21 s (maximum length of AB episode) with variable steps were investigated.

The algorithm to obtain the optimal parameters in the second layer was similar to the first one except sliding windows were $T_2 = 14$ s. In the second layer, in

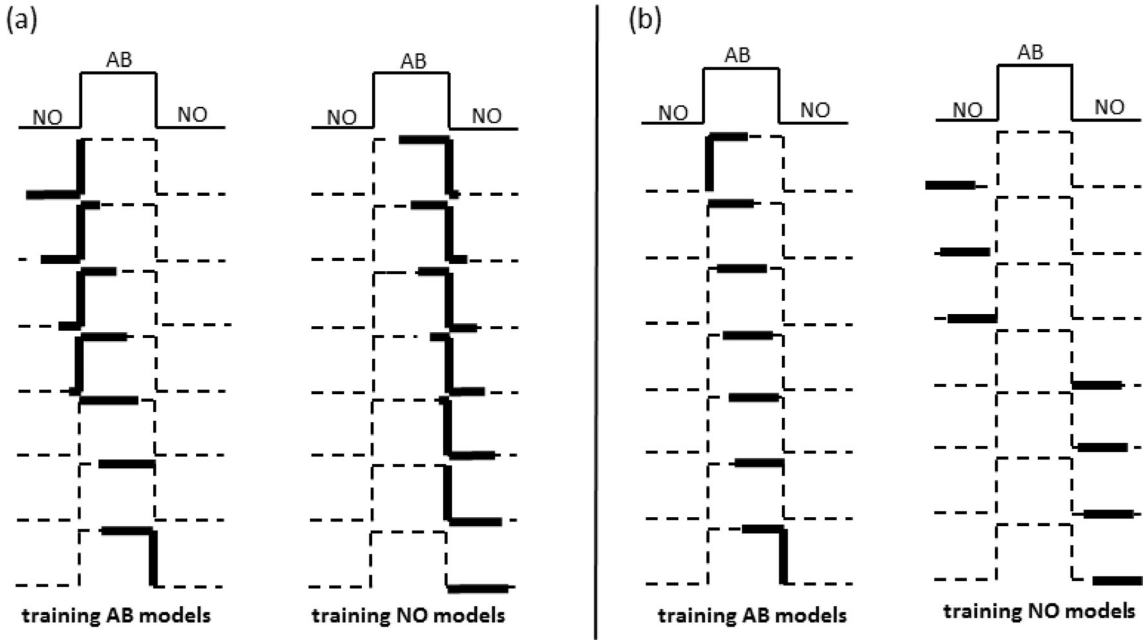

FIGURE 3. Training approaches of the second layer HMMs. (a) The prior-segment approach: training HMMs with segments in which the last sample of them were included in the desired events, (b) The onset-segment approach: training HMMs with segments were considered during the desired events.

addition to the aforementioned metrics, time detection delay was calculated and reported. The detection delay is defined as the time elapsed between the onset of AB and detection. With this definition, for those onsets detected before the annotated AB onsets, a negative time detection delay was calculated. The detection delay was only calculated for AB episode and not for normal segments.

A cross-validation was performed on the evaluation set by repeating 5 folds, each of which involved the calculation of metrics. In each fold, 20% of the data was used for training and the rest were remained for test data.

Statistical Analysis

R statistical software (version 3.6.2) was used to compare the performance of the two detection approaches, prior-segment approach and onset-segment approach. Based on the normality of the data examined by Shapiro-Wilk test, t-test or Mann-Whitney test was applied. The p-value < 0.05 was considered as significant.

RESULTS

Figure 4 shows an example trace of AB detection in the proposed method according to the prior-segment approach. An episode of AB can be recognized by increases in RR (Fig. 4a) and fluctuations for QRSd (Fig. 4b). The log-likelihood curves of the banks related to RR and QRSd change over time (Figs. 4c and 4d). At the time instant of AB, the log-likelihood of AB model increases (NO model decreases) in bank related to RR but the increase in the log-likelihood of AB model (decrease in the log-likelihood of NO model) in bank related to QRSd occurs before the onset of AB. After performing detection in the first layer, the outputs of the HMM banks form two-dimensional observation inputs of the second layer (Figs. 4e and 4f). The log-likelihoods in the second layer change at the onset of AB and the log-likelihood of AB model increases (NO model decreases) (Fig. 4g). The result of detection is shown in (Fig. 4h).

Figures 5 and 6 show the ROC curves analyzed to determine the optimal parameters for the two approaches, prior-segment and onset-segment. In each figure, the model with the maximum PD is shown as the best model. The optimal values of the parameters in the first and the second layer, as well as calculated metrics in these values, are summarized in Tables 1 and 2, respectively.

Table 3 shows the metrics measured in the second layer by varying the sliding window length on the training set of the prior-segment approach. The maximum result of the PD metric was achieved by a 14-second sliding window, for which we obtained SEN and SPC 98.5 and 94.39%, respectively with time delay of − 5.7 s.

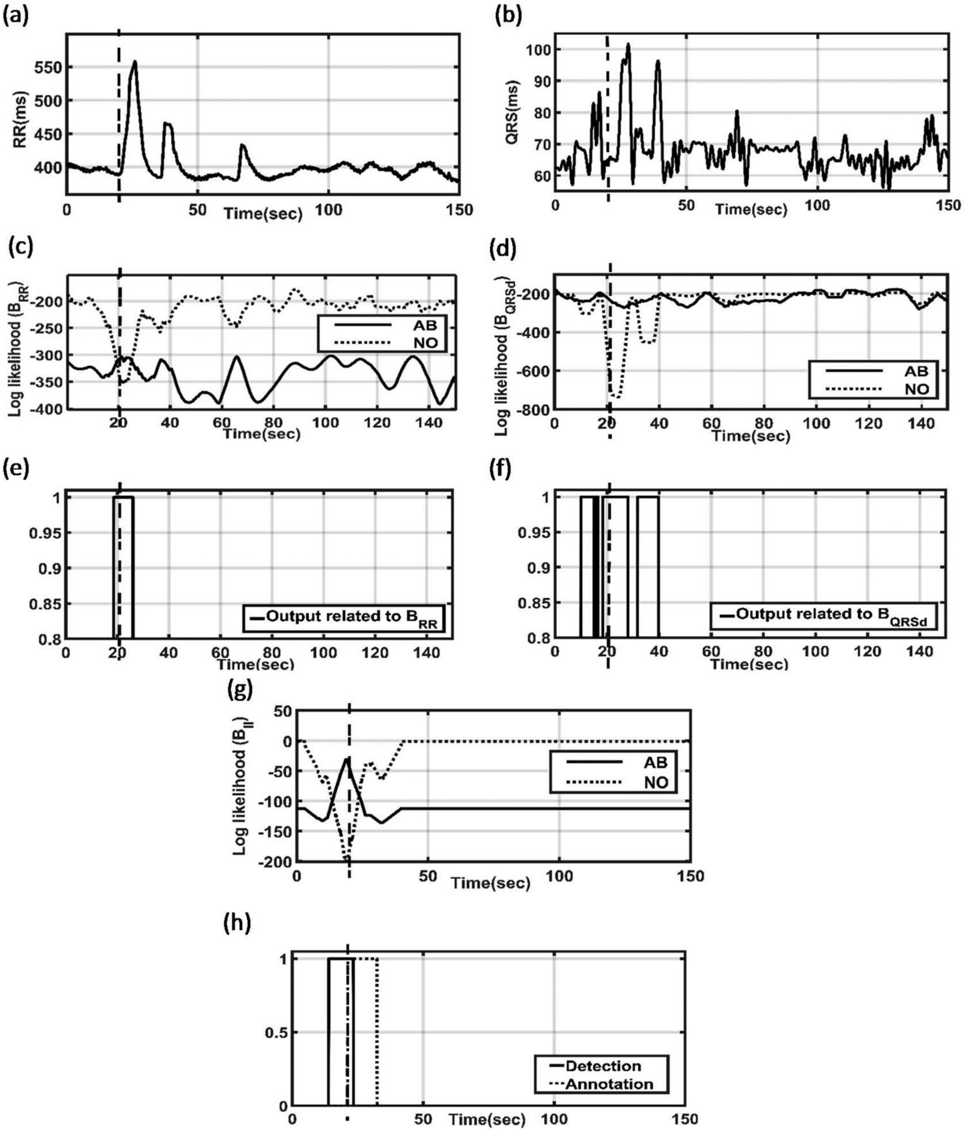

FIGURE 4. Example of the AB detection from time series (RR and QRSd) by the proposed method. The annotated onset of the AB episode is shown by vertical dash lines. (a), (b), (c) and (d): the input observations and log-likelihood curves of banks related to RR and QRSd, respectively. (e), (f): the outputs of the first layer after thresholding that constitute input observations of the second layer. (g) log-likelihood curves of bank related to the second layer. (h) the output of the second layer after thresholding (final detection).

The performance metrics of each bank for the two approaches on test data were calculated through cross-validation and reported in Tables 4 and 5. According to these tables, the best results for AB detection were achieved by the second layer in both approaches with the average SEN and SPC of 98.15 ± 0.67 and $97.11 \pm 0.31\%$ for the prior-segment approach and 93.76 ± 4.02 and $98.41 \pm 0.43\%$, respectively for the second one. In addition, the average time delays of -5.05 ± 0.41 s and -3.73 ± 0.62 s were achieved by the prior-segment and onset-segment approaches, respectively. It should be noted that, the reported value of time delay for each approach is the average of all obtained values consisting of the prediction with negative time delays and the detection cases with positive time delays.

Table 6 presents the statistical comparison results of the metrics between the two detection approaches. No significant difference was found between the accuracy of the two approaches ($p = 0.12$). In contrast, the time delay was significantly higher in the prior-segment approach compares to the onset-segment approach (-5.05 ± 0.41 s vs. -3.73 ± 0.62 s, $p = 4.6760E-04$).

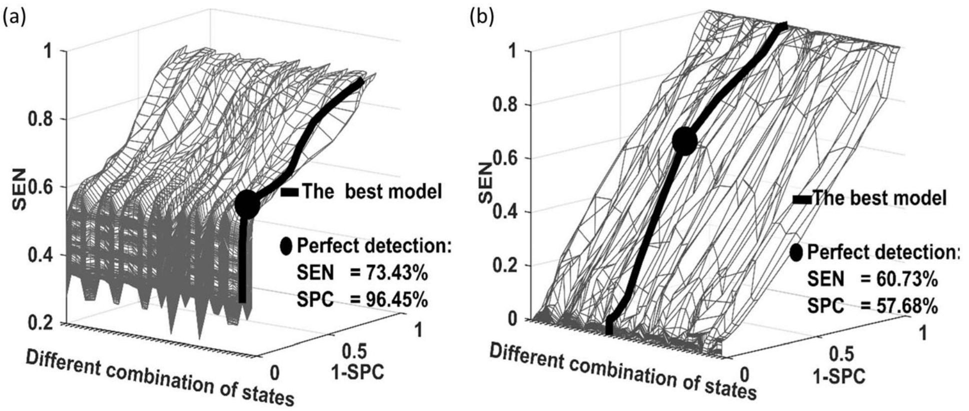

FIGURE 5. ROC curves to determine optimal parameters in the first layer. (a): HMM bank related to RR (B_{RR}), (b): HMM bank related to QRSd (B_{QRSd}).

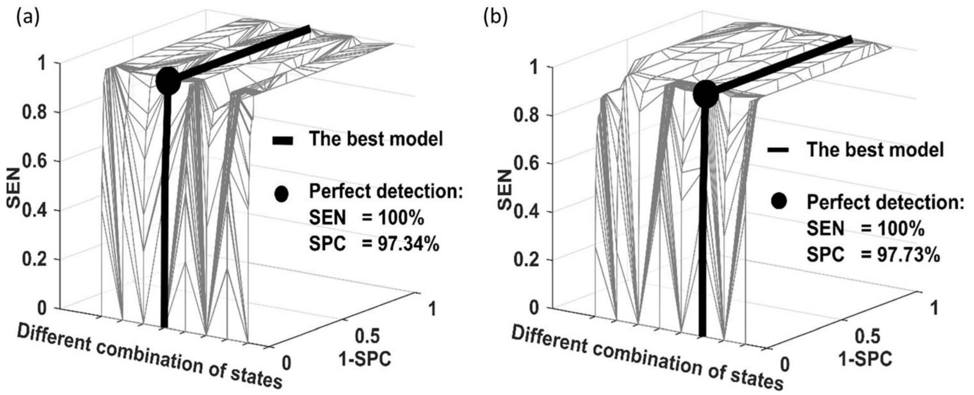

FIGURE 6. ROC curves to determine optimal parameters in the second layer. (a) the prior-segment approach, (b) the onset-segment approach.

TABLE 1. The optimal values of the parameters in the first layer and related metrics.

Performance metrics	B_{RR}	B_{QRSd}
Training ACC (%)	95.82	57.83
Optimal states [AB,NO]	[3,5]	[6,8]
SEN (%)	73.43	60.73
SPC (%)	96.45	57.68
AUC (%)	63.79	62.27
PD (%)	70.82	35.03
DPD	0.26	0.57

DISCUSSION

In this study, an early detection algorithm based on LHMM was presented to detect the occurrence of AB in preterm infants. We were able to successfully: 1- implement the structure of LHMM for analyzing RR distance and QRS duration, 2- validating the performance of each layer separately, and 3- detect AB episodes with average ACC of 97.14 ± 0.31% and time delay of -5.05 ± 0.41 s for the prior-segment approach and average ACC of 98.27 ± 0.42% and time delay -3.73 ± 0.62 s for the onset-segment approach.

LHMM is a hierarchical model based on HMM that enables: 1- independently training and evaluating each layer, 2- analyzing of the observations by different time granularity and temporal resolution, 3- interpreting the effect of each layer separately. Additionally, as in LHMM, the inputs of the second layer are processed by the previous layer, it is less sensitive to noise and baseline fluctuations of the observations. Finally, using LHMM, the detection can be implemented by using smaller HMMs in the hierarchy instead of defining a single huge HMM.

Our proposed model based on LHMM has been trained appropriately without any overfitting as indicated by high training and test accuracy of 97.38 and 97.14 ± 0.31% for the prior-segment approach and 97.77 and 98.27 ± 0.42% for the onset-segment approach, respectively. Such results indicate the robustness of the proposed model to be used as a monitoring system for preterm infants. An optimal monitoring system not only detects the AB episodes

TABLE 2. The optimal values of the parameters in the second layer for both approaches and related metrics.

Performance metrics	The prior-segment approach	The onset-segment approach
Training ACC (%)	97.38	97.77
Optimal states [AB,NO]	[4,4]	[3,5]
SEN (%)	100	100
SPC (%)	97.34	97.73
AUC (%)	94.95	99.49
PD (%)	97.34	97.73
DPD	0.02	0.02
Time delay (s)	− 5.24	− 3.86
STD delay (s)	1.05	0.69

TABLE 3. The results of calculated metrics by varying the sliding window in the second layer (the prior-segment approach), the row of the maximum value of PD is marked as bold.

Sliding window (s)	ACC (%)	SEN (%)	SPC (%)	Time delay (s)	STD delay (s)	PD (%)
21	91.33	99.94	90.85	− 11.97	2.11	90.79
19	91.17	99.6	90.73	− 10.1	2	90.36
16	90.7	98.99	90.32	− 7.55	1.81	89.40
**14**	**94.56**	**98.50**	**94.39**	**− 5.7**	**1.68**	**92.97**
11	97.48	87.25	97.84	− 3.3	0.8	85.36
9	97.22	0	1	NaN	NaN	0

TABLE 4. The cross-validation results of proposed LHMM at optimal parameters (the prior-segment approach).

Bank	ACC (%)	SEN (%)	SPC (%)	Time delay (s)	STD delay (s)
B_{RR}	95.29 ± 1.25	77.91 ± 0.99	95.65 ± 1.28	–	–
B_{QRSd}	55.21 ± 3.72	50.58 ± 3.39	55.31 ± 3.87	–	–
B_{II}	97.14 ± 0.31	98.15 ± 0.67	97.11 ± 0.31	− 5.05 ± 0.41	2.21 ± 0.21

Values are reported as mean ± standard deviation.

TABLE 5. The cross-validation results of proposed LHMM at optimal parameters (the onset-segment approach).

Bank	ACC (%)	SEN (%)	SPC (%)	Time delay (s)	STD delay (s)
B_{RR}	95.82 ± 1.51	77.36 ± 1.12	96.20 ± 1.55	–	–
B_{QRSd}	55.02 ± 4.64	48.48 ± 3.36	55.16 ± 4.81	–	–
B_{II}	98.27 ± 0.42	93.76 ± 4.02	98.41 ± 0.43	− 3.73 ± 0.62	3.76 ± 2.05

Values are reported as mean ± standard deviation.

TABLE 6. Comparison statistical analyses of two approaches.

Performance metrics of B_{II}	The prior-segment approach	The onset-segment approach	*p*-value (Cut off value = 0.05)
ACC (%)	97.14 ± 0.31	98.27 ± 0.42	0.1222
SEN (%)	98.15 ± 0.67	93.76 ± 4.02	8.44E−05
SPC (%)	97.11 ± 0.31	98.41 ± 0.43	0.02679
Time delay (s)	− 5.05 ± 0.41	− 3.73 ± 0.62	4.6760E−04

Values are reported as mean ± standard deviation.

accurately (high sensitivity) but demonstrates minimum false alarms (high specificity). To find high sensitivity and specificity, we optimize the detection threshold ($\delta_{B_{RR}}, \delta_{B_{QRSd}}, \delta_{B_{II}}$) using ROC curves based on PD. This metric was previously used in Ref. 17 with DPD 0.06, however our results have improved DPD to 0.02 in both approaches.

For the proposed LHMM, higher accuracies were obtained in the second layer (97.14 ± 0.31% for the prior-segment approach and 98.27 ± 0.42% for the onset-segment approach) compared to RR (95.29 ± 1.25% for the prior-segment approach and 95.82 ± 1.51% for the onset-segment approach) and QRSd (55.21 ± 3.72% for the prior-segment approach and 55.02 ± 4.64% for the onset-segment approach) banks in the first layer. This result indicates that combining the outputs of the first layer and analyzing them through a longer course of time in the second layer can improve the detection accuracy.

The database analyzed in this study was used in previous studies with the same preprocessing and feature extraction. In these studies, different detection models/algorithms were suggested for AB detection.[4,5,7,10,15,17] The main contribution of this work was to propose a detection model based on LHMM. This model enabled to analyze the signals with different temporal resolution in different layers, and combining the analysis of each feature with lower computation costs and less complexity compared to coupling approaches.[10,17] The complexity of the standard HMM algorithm with N state and observation length T is $O(TN^2)$. For a LHMM with two layers, the computational cost can be calculated as $O(TM_{L1} + DTM_{L2})$, in which D and T are the dimension and length of the observations, and M_{L1}, M_{L2} are the sum of squares of the number of states in the first and the second layer, respectively. The computational complexity of LHMM is the considerably lower compared to the more complex HMM generalizations, such as CHMM $O(TN_s^C)$ (N_s = state space, C = number of channels)[10] that were previously used for AB episode detection. Furthermore, in comparison to the previous studies (Table 7), LHMM showed more accurate and earlier detection of AB episodes, especially using the prior-segment approach.

No significant differences were observed in the accuracy of the two detection approaches. However, the prior-segment approach provided more negative time delay than the other approach presumably due to incorporating the dynamics of the observations prior to the onset of AB episodes.

The AB detection algorithm based on LHMM had the ability to early detect AB episodes, which was reflected by the negative values of the detection time delay. The negative average value for time delay indicated the ability of detecting the changes in the dynamic of the observations eventually ending up to AB occurrence. Therefore, the proposed algorithm can be used for early detection and risk prediction of AB upon further validation on the ECG signals recorded in real world.

One of the limitations of our work was that the study was performed in a single institution and the algorithm was validated on low sample size. These limitations can be addressed by recording more data from in different institutions and incorporating the proposed model for experimental monitoring preterm infants in future. Another limitation of our study was related to the usage of rather clean data without any major disturbance caused by movements or clinical interventions performed on the infants. Although, the algorithm is required to detect AB, while the infant is sleep with minimal level of interventions and disturbances, further improvement of the pre-processing aspects of the proposed method are warranted in future developments to evaluate the robustness of the method to very low signal to noise ratios.

In this study, we proposed more accurate algorithm for detecting AB with less time delay and less computation costs from single channel ECG. For future

TABLE 7. Comparison the results with other studies for AB detection.

	Method	SEN (%)	SPC (%)	Time delay (s)	STD delay (s)
Masoudi *et al.*[15]	CHMM	84.92 ± 0.26	94.17 ± 0.51	2.32 ± 0.01	4.82 ± 0.03
Altuve *et al.*[7]	HsMM	88.56 ± 1.72	92.87 ± 0.86	1.59 ± 0.24	3.61 ± 0.30
	HMM	86.52 ± 3.96	92.27 ± 1.77	1.61 ± 0.43	3.74 ± 0.32
Montazeri Ghahjavarestan *et al.*[10,17]	CHMM	95.74 ± 0.82	91.88 ± 0.31	− 0.59 ± 0.21	2.79 ± 0.06
	CHSMM	95.99 ± 0.31	93.84 ± 0.24	− 1.11 ± 0.04	2.56 ± 0.03
This study	LHMM[a]	98.15 ± 0.67	97.11 ± 0.31	− 5.05 ± 0.41	2.21 ± 0.21
	LHMM[b]	93.76 ± 4.02	98.41 ± 0.43	− 3.73 ± 0.62	3.76 ± 2.05

Values are reported as mean ± standard deviation.
[a]The prior-segment approach, [b]the onset-segment approach.

work, the LHMM model can include the integration of other clinical signals such as respirations and oxygen saturation in the blood into for even earlier detection of apnea of prematurity.[3,13]

CONFLICT OF INTEREST

The authors have no conflict of interest to declare.

REFERENCES

1. Aarno, D., and D. Kragic. Evaluation of layered HMM for motion intention recognition. In: *IEEE International Conference on Advanced Robotics*, 2007.
2. Aarno, D., and D. Kragic. Layered HMM for motion intention recognition. In: *IEEE/RSJ International Conference on Intelligent Robots and Systems*, 2006.
3. Aguirre, L. A., V. C. C. Barros, and Á. V. Souza. Nonlinear multivariable modeling and analysis of sleep apnea time series. *Comput. Biol. Med.* 29(3):207–228, 1999.
4. Altuve, M., et al. Comparing hidden Markov model and hidden semi-Markov model based detectors of apnea-bradycardia episodes in preterm infants. In: *Computing in Cardiology,* 2012.
5. Altuve, M., et al. On-line apnea-bradycardia detection using hidden semi-Markov models. In *Annual International Conference of the IEEE Engineering in Medicine and Biology Society*, 2011.
6. Altuve, M., *et al.* Multivariate ECG analysis for apnoea–bradycardia detection and characterisation in preterm infants. *Int. J. Biomed. Eng. Technol.* 5(2–3):247–265, 2011.
7. Altuve, M., *et al.* Online apnea–bradycardia detection based on hidden semi-markov models. *Medical & Biological Engineering & Computing* 53(1):1–13, 2015.
8. Cruz, J., et al. Algorithm fusion for the early detection of apnea-bradycardia in preterm infants. In: *Computers in Cardiology*, 2006.
9. Gee, A. H., *et al.* Predicting Bradycardia in preterm infants using point process analysis of heart rate. *IEEE Trans. Biomed. Eng.* 64(9):2300–2308, 2017.
10. Ghahjaverestan, N. M., *et al.* Coupled Hidden Markov model-based method for apnea bradycardia detection. *IEEE J. Biomed. Health Inf.* 20(2):527–538, 2016.
11. Glodek, M., et al. Recognizing human activities using a layered Markov architecture. In: *International Conference on Artificial Neural Networks*, 2012. Berlin: Springer.
12. He, L., C.-F. Zong, and C. Wang. Driving intention recognition and behaviour prediction based on a double-layer hidden Markov model. *J. Zhejiang Univ. Sci. C* 13(3):208–217, 2012.
13. Hornero, R., *et al.* Utility of approximate entropy from overnight pulse oximetry data in the diagnosis of the obstructive sleep apnea syndrome. *IEEE Trans. Biomed. Eng.* 54(1):107–113, 2006.
14. Kabir, M. H., *et al.* Two-layer hidden Markov model for human activity recognition in home environments. *Int. J. Distrib. Sensor Netw.* 2016:1–12, 2016.
15. Masoudi, S., et al. Early detection of apnea-bradycardia episodes in preterm infants based on coupled hidden Markov model. In: *IEEE International Symposium on Signal Processing and Information Technology (ISSPIT),* 2013.
16. McNames, J. and A. Fraser. Obstructive sleep apnea classification based on spectrogram patterns in the electrocardiogram. In: *Computers in Cardiology 2000, Vol. 27 (Cat. 00CH37163)*, 2000, IEEE.
17. Montazeri Ghahjaverestan, N., *et al.* Apnea bradycardia detection based on new coupled hidden semi Markov model. *Med. Biol. Eng. Comput.* 59:1–11, 2020.
18. Oliver, N., A. Garg, and E. Horvitz. Layered representations for learning and inferring office activity from multiple sensory channels. *Comput. Vis. Image Understand.* 96(2):163–180, 2004.
19. Pan, J., and W. J. Tompkins. A real-time QRS detection algorithm. *IEEE Trans. Biomed. Eng.* 3:230–236, 1985.
20. Perdikis, S., T. Dimitrios, and M.G. Strintzis. Recognition of humans activities using layered hidden Markov models. In: *Cognitive Information Processing Workshop*, 2008.
21. Poets, C. F., *et al.* The relationship between bradycardia, apnea, and hypoxemia in preterm infants. *Pediatr. Res* 34(2):144–147, 1993.
22. Portet, F., et al. Evaluation of on-line bradycardia boundary detectors from neonatal clinical data. In: 29th Annual International Conference of the IEEE Engineering in Medicine and Biology Society, 2007. IEEE.
23. Rabiner, L. R. A tutorial on hidden Markov models and selected applications in speech recognition. *Proc. IEEE* 77(2):257–286, 1989.
24. Razin, Y. S., *et al.* Predicting task intent from surface electromyography using layered hidden Markov models. *IEEE Robot. Autom. Lett.* 2(2):1180–1185, 2017.
25. Richard J. Martin, C.G.W., *Apnea of Prematurity*, in *Comprehensive Physiology* 2012. pp. 2923–2931.
26. Shirwaikar, R.D., et al. Machine learning techniques for neonatal apnea prediction. *J. Artific. Intell.* 9(1–3), 2016.
27. Solaimanpour, S., and P. Doshi. A layered HMM for predicting motion of a leader in multi-robot settings. In: *IEEE International Conference on Robotics and Automation (ICRA)*, 2017.
28. Thome, N., S. Miguet, and S. Ambellouis. A real-time, multiview fall detection system: a LHMM-based approach. *IEEE Trans. Circ. Syst. Video Technol.* 18(11):1522–1532, 2008.
29. Williamson, J.R., et al. Individualized apnea prediction in preterm infants using cardio-respiratory and movement signals. In: *IEEE International Conference on Body Sensor Networks*, 2013.

Publisher's Note Springer Nature remains neutral with regard to jurisdictional claims in published maps and institutional affiliations.
