# Supplementary material for: Exploring Computational Techniques in Preprocessing Neonatal Physiological Signals for Detecting Adverse Outcomes: Scoping Review
Source: Interact J Med Res. 2024 Aug 20;13:e46946. doi: 10.2196/46946 (PMC11372324; doi:10.2196/46946)
Supplement: Multimedia Appendix 3 [file ijmr_v13i1e46946_app3.zip › Included Papers - Final/3647/Semenova et al. - 2019 - Prediction of short-term health outcomes in preter.pdf]

Computer Methods and Programs in Biomedicine 180 (2019) 104996

Contents lists available at ScienceDirect

Computer Methods and Programs in Biomedicine

journal homepage: www.elsevier.com/locate/cmpb

Prediction of short-term health outcomes in preterm neonates from heart-rate variability and blood pressure using boosted decision trees

Oksana Semenova[a,c,*], Giorgia Carra[a,c,1], Gordon Lightbody[a,c], Geraldine Boylan[b,c], Eugene Dempsey[b,c], Andriy Temko[a,c]

[a] Department of Electrical and Electronic Engineering, University College Cork, 60 College Rd, Cork, Ireland
[b] Department of Pediatrics and Child Health, University College Cork, Cork, Ireland
[c] Irish Center for Fetal and Neonatal Translational Research (INFANT), University College Cork, Cork, Ireland

ARTICLE INFO

Article history:
Received 1 April 2019
Revised 11 July 2019
Accepted 25 July 2019

Keywords:
Hypotension
HRV
Boosted decision tree
Outcome prediction

ABSTRACT

Background and Objective: Efficient management of low blood pressure (BP) in preterm neonates remains challenging with considerable variability in clinical practice. There is currently no clear consensus on what constitutes a limit for low BP that is a risk to the preterm brain. It is argued that a personalised approach rather than a population based threshold is more appropriate. This work aims to assist health-care professionals in assessing preterm wellbeing during episodes of low BP in order to decide when and whether hypotension treatment should be initiated. In particular, the study investigates the relationship between heart rate variability (HRV) and BP in preterm infants and its relevance to a short-term health outcome.

Methods: The study is performed on a large clinically collected dataset of 831 h from 23 preterm infants of less than 32 weeks gestational age. The statistical predictive power of common HRV features is first assessed with respect to the outcome. A decision support system, based on boosted decision trees (XGboost), was developed to continuously estimate the probability of neonatal morbidity based on the feature vector of HRV characteristics and the mean arterial blood pressure.

Results: It is shown that the predictive power of the extracted features improves when observed during episodes of hypotension. A single best HRV feature achieves an AUC of 0.87. Combining multiple HRV features extracted during hypotensive episodes with the classifier achieves an AUC of 0.97, using a leave-one-patient-out performance assessment. Finally it is shown that good performance can even be achieved using continuous HRV recordings, rather than only focusing on hypotensive events – this had the benefit of not requiring invasive BP monitoring.

Conclusions: The work presents a promising step towards the use of multimodal data in providing objective decision support for the prediction of short-term outcome in preterm infants with hypotensive episodes.

© 2019 Published by Elsevier B.V.

1. Introduction

Every year, more than one in ten babies are born preterm and this number is rising [1]. Premature babies are at a higher risk of complications, which may lead to both short-term and long-term adverse health outcomes, including neuromotor, cognitive, hearing, and visual problems. Low blood pressure (BP) or hypotension is a recognised problem in preterm infants particularly during the first 72 h of life. This may result in inadequate blood flow to the brain and other vital organs. The definition of hypotension is still uncertain and the decision to intervene in the preterm infant with low BP is still unresolved; this has resulted in considerable variability in the practice of hypotension management [2–4]. When the level of mean arterial pressure (MAP) falls below the gestational age (GA) in weeks, an intervention in the form of volume expansion is commonly initiated. This approach, however, lacks supporting evidence. At the same time, hypotension treatment has been associated with adverse outcomes [5,6], including brain injury [7]. For

* Corresponding author at: Department of Electrical and Electronic Engineering, University College Cork, 60 College Rd, Cork, Ireland.
E-mail address: o.semenova@umail.ucc.ie (O. Semenova).

[1] G. Carra is with the Laboratory of Intensive Care Medicine, KU Leuven, Belgium, giorgia.carra@kuleuven.be. The work was performed during her stay at the INFANT Research Centre at University College Cork, Cork, Ireland.

https://doi.org/10.1016/j.cmpb.2019.104996
0169-2607/© 2019 Published by Elsevier B.V.

babies with no clinical or biochemical signs of shock, no intervention may be the most appropriate management [8]. The “Hypotension in Preterm Infants” trial was aimed at determining whether a standard approach to the management of hypotension with volume expanders and dopamine, versus a more observational approach with placebo, would result in improved short-term and long-term outcomes for preterm infants [8].

The ability to objectively assess preterm wellbeing during episodes of low BP may improve the efficacy of hypotension management. ECG is routinely recorded in preterm infants from which the time variation between successive heartbeats (heart rate variability, HRV) can be estimated. HRV provides a non-invasive assessment of both the sympathetic and parasympathetic control of the heart rate [9]. HRV analysis is uniquely suitable to explore the influence of the immature autonomic system on cardio-respiratory control in preterm neonates.

ECG has been used extensively for assessing aspects of newborn health. Promising results have been obtained for automated computer-based outcome prediction in full term neonates using a combination of multimodal features including HRV and EEG [10]. In term neonates, a significant association between HRV, severity of hypoxic ischemic brain injury and long-term neurodevelopmental outcome at two years of age was reported for 61 full-term neonates [11].

For the preterm population, different physiological modalities have also shown promise in predicting neurodevelopmental outcome [12,13]. A quantitative analysis of EEG, heart rate, peripheral oxygen saturation and other clinical features from forty three preterm infants was conducted. Logistic regression of all combined measures showed the potential for the prediction of both mortality and 2-year outcome [12].

HRV features together with the quantification of general physical movements were also found to be useful for 2-year outcome prediction in preterms [14]. The high frequency component of preterm HRV was shown to be a good biomarker of necrotizing enterocolitis, an acute neonatal inflammatory disease which may lead to death [15]. Time-domain HRV measurements have shown significant differences between septic and non-septic newborns [16]. Lower HRV was observed in children with low birth weight, including preterm babies [17]. HRV has been assessed as a predictor for successful removal of mechanical ventilation [18]; it was demonstrated that babies who failed their first extubation had decreased HRV. A correlation between low frequency oscillation of HRV and BP for preterm neonates has also been reported [19]. Another study on 92 preterm neonates revealed a significant association between neonatal HRV and respiratory distress syndrome, and proposed the use of HRV as an indicator of morbidity and mortality in preterm infants [20]. HRV was also shown to be effective for the monitoring of preterm neonates in the NICU. The heart rate observation monitor (HeRO) [21] which allows for the detection of sepsis in preterm infants, based on three (SDNN, sample asymmetry and sample entropy) HRV characteristics is now used in many NICUs. Results of a large randomised clinical trial have reported a reduction in mortality (from 10.2% to 8.1%) when providing a sepsis score to the medical staff in real time [22].

While several studies have identified an association between HRV and neonatal health outcomes in term and preterm infants, there is still a lack of understanding about this relationship in the context of low BP episodes in preterm infants. This study aims to investigate the relationship between HRV and BP and short-term health outcome in preterm infants with a GA less than 32 weeks. In particular, the usefulness of various HRV features for the estimation of short-term health outcome is assessed and the predictive power of the combination of HRV features in a classifier during the episodes of low BP is studied.

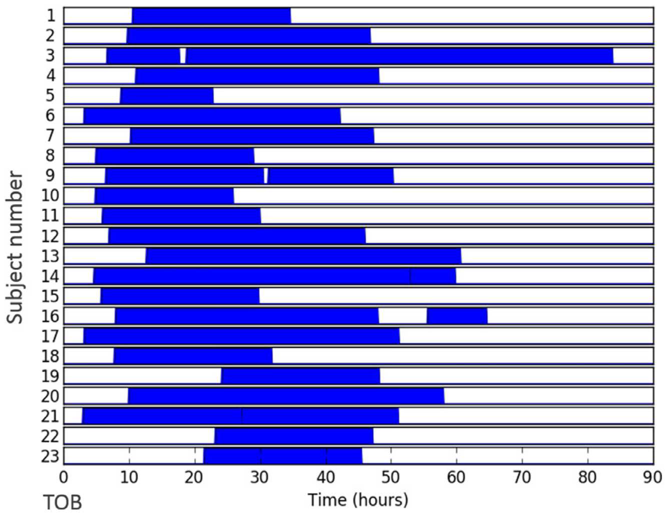

Fig. 1. Schematic representation of the duration and temporal location of recordings. Each recording is represented with respect to the time of birth (TOB) for each neonate.

2. Materials and methods

2.1. Dataset

The data used in this work is a subsample of a larger dataset reported by Lloyd et al. [12]. Out of a total of 43 preterm neonates, 23 had continuous blood pressure monitoring and were used in this study. The clinical dataset was recorded at the neonatal intensive care unit of Cork University Maternity Hospital, Ireland. The study had full ethical approval from the Clinical Research Ethics Committee of the Cork Teaching Hospitals. The dataset includes continuous synchronously recorded ECG and BP signals with a total duration of 831 h (median = 38 h, IQR: 24 to 48 h). Clinical characteristics of the dataset are provided in Table 1. The temporal location of each recording with respect to the time of birth for each infant is shown in Fig. 1.

One-channel ECG was recorded on a Natus NicOne video EEG machine (CareFusion Co., San Diego, USA). ECG data were sampled at 256 Hz (20 subjects) or 1024 Hz (3 subjects). Continuous invasive arterial BP monitoring was simultaneously recorded via an umbilical arterial catheter using the Philips Intellivue MP70 machine (with direct output to the Natus NicOne), which provides BP data sampled at 1 Hz. All infants were nursed supine. The positioning of the tip of the umbilical catheter in the descending aorta was confirmed by chest radiograph. An example of a data segment is presented in Fig. 2.

The well-being of the newborn infant in the NICU can be quantified by a number of measures. Illness scores are widely used for neonates as they allow for standardised comparisons. Fig. 3 schematically represents the timing of the various illness scores including Apgar score, clinical risk index for babies (CRIB II) and clinical course score (CCS). All scores summarise various criteria of the well-being of the neonate on the different stages. This study concentrates on the usage of CCS which represents the short-term health outcome of the preterm neonate.

The CCS is a binary score assigned independently to every infant by two consultant neonatologists at discharge [12]. When grades differed between reviewers, a consensus was reached by discussion. The CCS was based on information from the discharge summary and medical notes summarising the presence or absence of at least one out of five major neonatal complications: 1) grade III/IV intraventricular haemorrhage or cystic periventricular leukomalacia (CNS, central nervous system complications);

Table 1
Clinical information for preterms in the dataset. CCS, clinical course score; ROP, retinopathy of prematurity; NEC, necrotizing enterocolitis; BPD, bronchopulmonary dysplasia, CNS, central nervous system complications (grade III/IV intraventricular haemorrhage or cystic periventricular leukomalacia).

Subject #	GA (weeks)	Birth weight (g)	Gender	Apgar score 5 min	Sepsis	CNS	NEC	BPD	ROP	CCS
1	30	1540	F	8	0	0	0	0	0	0
2	28	980	F	10	1	0	0	0	0	1
3	30	1450	M	5	1	1	0	0	0	1
4	29	1230	M	9	0	0	0	0	0	0
5	26	840	M	8	0	0	0	0	0	0
6	25	640	F	6	1	–	–	–	0	1
7	31	960	F	9	0	0	0	0	0	0
8	28	980	F	8	0	0	0	0	0	0
9	28	650	F	7	0	0	0	0	0	0
10	28	530	F	6	0	0	0	0	0	0
11	26	860	M	8	1	0	0	0	0	1
12	26	980	M	8	1	0	0	0	0	1
13	24	740	F	9	0	0	1	0	0	1
14	24	670	M	6	0	0	1	0	0	1
15	28	1040	F	9	0	0	0	0	0	0
16	23	540	F	7	1	0	1	1	0	1
17	26	660	F	3	1	0	1	1	0	1
18	28	1330	F	4	0	0	0	0	0	0
19	30	730	M	10	0	0	0	0	0	0
20	23	580	F	6	0	0	0	1	0	1
21	28	680	M	8	1	0	0	1	0	1
22	28	1130	F	9	0	0	0	0	0	0
23	31	1900	M	8	0	0	0	0	0	0
**Median**	**28**	**860**	**64%**	**8**	**35%**	**0.04**	**17%**	**17%**	**0%**	**52%**
**(IQR)**	**(26 to 28.5)**	**(665 to 1085)**	**(F)**	**(6 to 9)**	**(1 - sepsis)**	**(1 - CNS)**	**(1 - NEC)**	**(1 - BPD)**	**(1 - ROP)**	**(0 - healthy)**

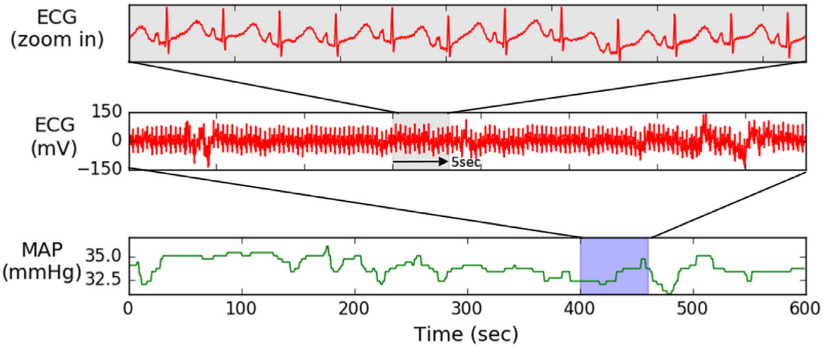

Fig. 2. One minute of raw ECG and ten minutes of mean arterial pressure (MAP) recordings (GA = 26 weeks).

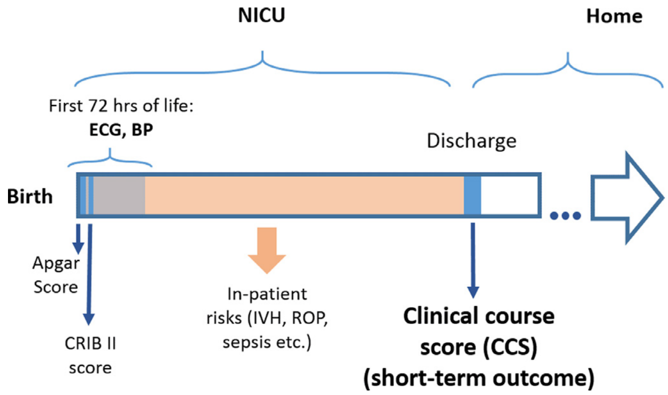

Fig. 3. The timing of illness scores assigned to an infant during the course in the neonatal intensive care unit (NICU). In-patient risks include major neonatal complications: IVH (intraventricular haemorrhage), cystic periventricular leukomalacia, necrotizing enterocolitis, infection (sepsis), retinopathy of prematurity (ROP). The diagram is adapted from Lloyd et al. [12].

2) bronchopulmonary dysplasia defined by oxygen dependency at 36 weeks postmenstrual age (BPD); 3) necrotizing enterocolitis Bells stage 2b or greater (NEC); 4) infection defined as positive blood culture with abnormal inflammatory markers (sepsis); and 5) retinopathy of prematurity of stage 2 or greater (ROP). In the cohort used in this study 12 out of 23 neonates had a good short-term outcome.

While both EEG and ECG are used for assessing newborn health [10], this study incorporates only HRV recording. HRV characterizes physiological stability within the first 72 h of life and therefore is considered as a more suitable method for the prediction of short-term health status associated with the development of different morbidities that constitute the CCS.

A recent survey has confirmed that the most common criterion used by neonatologists to direct intervention for hypotension management is when the level of mean arterial pressure (MAP) falls below GA [23]. In this study, we aim to investigate the effect of hypotension on preterm wellbeing. The term ‘hypotension’ in this work does not confer a clinical diagnosis, but simply refers to episodes when the BP (MAP) falls below some specified age-related (GA) threshold. Three thresholds were considered in this work: $MAP \leq GA$, $MAP \leq GA + 2$ mmHg and $MAP \leq GA + 4$ mmHg. Each threshold results in a subset of preterm neonates who had at least one hypotensive episode of at least 5 min duration. For example, only 15 preterms (8 healthy) out of 23 will satisfy the threshold of $MAP \leq GA$ – that is; only 15 subjects will have at least one episode of hypotension as defined by the threshold. Sim-

Table 2
Frequency- and time-domain features extracted from ECG, and BP.

Domain	HR features
**Time**	MeanRR, SDNN, skewness, kurtosis, TINN, RMSSD, SDNN/RMSSD, ApEn, Allan Factor
**Frequency**	Power in VLF (0.008 – 0.04 Hz), LF (0.04 – 0.2 Hz) and HF (0.2 - 1 Hz) bands, ratio LF/HF
	**BP features**
**Time**	MAP, MAP–GA, MAP/GA

ilarly, allowing the threshold to be larger, more preterms will be included – for MAP ≤ GA + 2 mmHg the resultant dataset contains 19 preterms (10 healthy) and for MAP ≤ GA + 4 mmHg the resultant dataset contains all 23 subjects (12 healthy). The episodes under the chosen threshold were marked as “hypotensive” episodes in each recording. The influence of the threshold on the definition of hypotensive episodes will be studied with respect to the ability of HRV features to predict outcome.

2.2. Signal preprocessing and feature extraction

Segments which were highly corrupted by artefacts were excluded from the study.

2.2.1. MAP

The diastolic (DP) and systolic (SP) pressures were recorded every second and used to calculate the MAP as $MAP = DP + 1/3\,(SP - DP)$. The segments with MAP < 10 mmHg were excluded to remove artefacts due to brief disconnection of the pressure transducer or movements. The MAP was then segmented into 1-hour windows. Values beyond ±3 SD were marked and then discarded. This allowed for the removal of sharp and non-physiological changes in MAP and this was confirmed by neonatologist (ED). The values of MAP can also be affected by interventions such an infusions given through the umbilical line. The ten minutes of the MAP signal before and after any marked interventions were also eliminated.

2.2.2. ECG features

The ECG signal was segmented into non-overlapping 5-minute epochs. The R-peaks were identified using the Pan-Tompkins method [24]. The normal range of the heart rate changes with maturation. In order to account for this difference and adjust the algorithm for preterm neonates, the preterm ECG signal was band-pass filtered using 4–30 Hz cut-off frequencies instead of the originally proposed 5–15 Hz. Increasing the low-pass cut-off frequency to 30 Hz emphasises the R-peaks to better distinguish between the R-peak and the P-wave. No manual inspection of the detected R-peaks was conducted. Abnormal values of time intervals between R-peaks (RR intervals) caused by artefacts were corrected by the moving average filter or discarded if the epoch was too corrupted. The corrected RR intervals, NN intervals, were used to estimate the instantaneous heart rate signal.

The behaviour of HR was quantified using time-domain and frequency-domain analysis. Thirteen HRV characteristics were extracted from the RR intervals (Table 2).

The time domain features were derived using simple statistics of RR interval distribution and include the mean of the RR interval (MeanRR), the standard deviation of NN interval (SDNN), which reflects all the periodic components responsible for the variability in given epoch; skewness and kurtosis of NN distribution; triangular interpolation of the NN histogram (TINN), defined as the width of the baseline of an imaginary triangle that best approximates the histogram of the NN signal [25]. Other features include the temporal information, such as root mean square of successive RR intervals (RMSSD) and the ratio between SDNN and RMSSD.

Nonlinear methods of HRV analysis can provide additional information about the autonomic control of heart rate. Nonlinear HRV features include approximate entropy (ApEn) to quantify regularity and complexity of stationary signal [26] and the Allan Factor (AF), a scale-dependent measure which quantifies the variability of successive counts [27]. AF is defined as the ratio of the variance of successive counts for a specific counting number t divided by twice the mean of events in the counting time. In this study, AF is calculated for every 5-minute epoch of NN intervals, with a time scale $t = 1$ min.

Frequency domain features were obtained from the uniformly resampled NN intervals, at 256 Hz. HRV was then quantified by power in the various bands: very low frequency (0.008–0.04 Hz) band (VLF); low frequency (0.04–0.2 Hz) band (LF), high frequency (0.2–1 Hz) band (HF) as well as the ratio between the LF and HF power (LF/HF). The power in these three bands and the ratio LF/HF is a reflection of both sympathetic and parasympathetic response. For some HRV features (VLF, LF, HF, LF/HF, SDNN, TINN, skewness, kurtosis and ApEn) the NN intervals were normalized with respect to the average (over epoch) NN interval [28]. These features have been previously used for various purposes to quantify the HRV in term and preterm infants [10, 29]. To the best of our knowledge, these features have not been previously researched with respect to BP. For this reason, a diverse set of HRV features was retained to examine which features are useful for the task.

2.3. Statistical modelling

2.3.1. Predictive power of HRV characteristics

The area under the receiver operating characteristic curve (AUC) is used to quantify the discriminative (predictive) power of each HRV feature with respect to the health status of the preterm, which is represented by CCS. The receiver operating curve is constructed with a set of sensitivity and specificity values which are obtained by thresholding each feature. The AUC is directly connected to the Mann-Whitney U-statistic (AUC is the U-statistic normalised by the product of positive and negative sample sizes). U-statistic is a robust non-parametric alternative to the student's t-test to assess the difference between two distributions [30].

2.3.2. Boosted decision trees

In order to check the discriminative capability of a combination of features, a boosted decision trees classifier was used. Boosting is a method for the creation of an accurate and strong classifier from a set of weak classifiers [31]. Gradient boosted decision trees is a machine learning technique for both regression and classification problems, which produces a prediction model in the form of an ensemble of decision trees which are built in a stage-wise fashion by optimizing an arbitrary differentiable loss function [32]. The extreme gradient boosting classifier, XGBoost [33] is a well-known implementation of regularised boosted decision trees which is available as open source software. The package has been widely recognized in a number of machine learning and data mining challenges, (e.g. in Kaggle competitions), where state-of-the-art results on a wide range of problems have been reported. A detailed explanation of the algorithm if provided in the Appendix.

We have also investigated the interaction of various HRV features [34]. The interaction between two features is defined as a path of length 2 within a tree and is calculated by summing the node gains (Eq. A.5) along the corresponding decision path. The process is repeated for every pair of features in each decision tree from the tree ensemble.

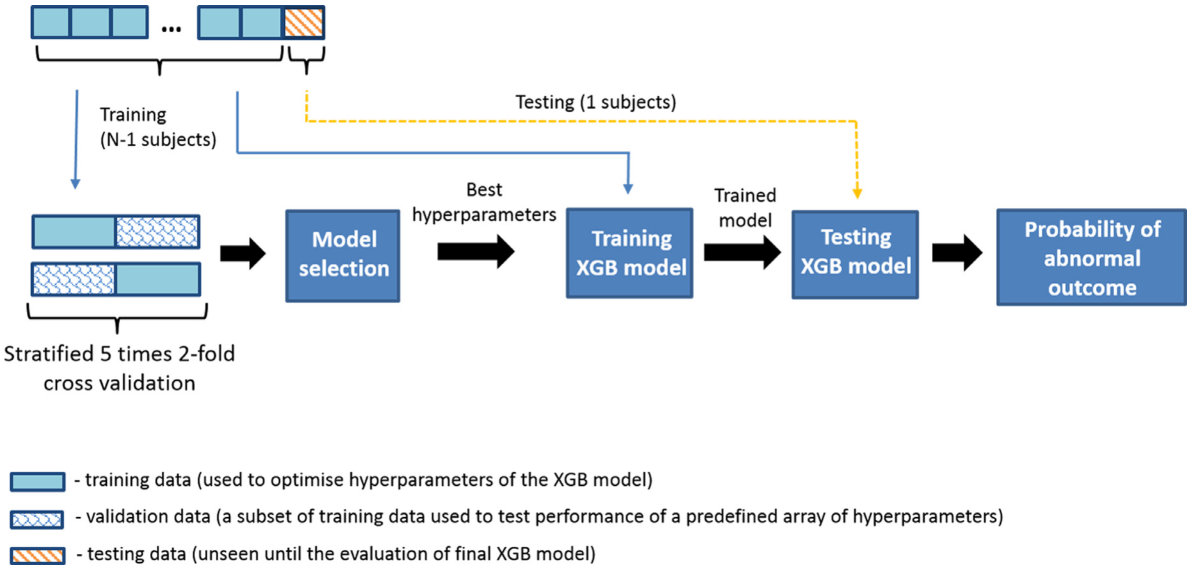

Fig. 4. A diagram of LOO subject independent performance assessment and 5 times 2-fold CV model selection routines.

The XGBoost [33] package implements two additional techniques for regularization. The first one is shrinkage, which scales newly added weights by a factor, η, after each step of tree boosting. This reduces the influence of each individual tree and leaves space for future trees to improve the model. The second is the column (feature) subsampling which is used in Random Forests [35], where only a random subset of features is considered while constructing a given tree. The most important user-tuneable parameters of an XGBoost classifier are thus the depth of the tree, the feature and data subsampling ratios, as well as the number of iterations which is a function of the learning rate.

2.4. Model selection and performance assessment

Whereas using the statistical methods the predictive power of each feature is computed on the whole dataset, the application of machine learning tools requires a clearly defined performance assessment routine – the split of data used for model training and for model testing. The leave-one-patient-out (LOO) subject independent performance assessment is used in this work to estimate the generalisation error. All but one subject's data are used for training and the remaining subject's data are used for testing. The procedure is repeated until each patient has been a test subject (Fig. 4). The LOO method is known to be an almost unbiased estimation of the true generalization error [36], i.e. the error reported with this routine is the most correct estimation of the error this system would get by testing on a separate unseen dataset of infinite size once it is trained on all available data.

The XGBoost classifier has a number of user-tuneable parameters (hyperparameters). In order to balance between underfitting and overfitting, an optimisation of hyperparameters is performed using an exhaustive search through manually specified range for each hyperparameter. The routine used to tune the parameters is called model selection. The hyperparameters were tuned using the stratified 5 times 2-fold cross validation (CV) [37], performed on the training data, once the split of the whole data to train and test has been executed (Fig. 4). The CV folds were designed in a way that preserves the subject integrity – that is no subject data appear in two different folds. This allows for the model selection routine to have the optimization criteria that matches the one of the LOO performance assessment routine. In other words, the model selection routine searches for hyperparameters that maximise the performance on unseen patients in the internal CV, and these parameters are then used to train the model which is assessed on a single unseen patient in the external LOO loop. The performance assessment routine is thus independent of the model selection routine, and the testing patient is not seen or used for training the classifier or tuning of other system parameters at any time. The best performing parameter set is saved for each LOO iteration. The analysis of stability of the classifier is then performed by examining the most frequent set of hyperparameters used to train the classifier.

The performance of the classifier was similarly measured by the AUC metric. When an epoch of the physiological data (a vector of HRV features) is fed into the XGB classifier, the probability of abnormal outcome is returned for this epoch. These probabilities are then compared to the ground truth (outcome scores) and the AUC is computed across all epochs. The ground truth for each epoch is taken as the ground truth for the whole patient. The mean probability across all epochs for a given patient is also calculated, resulting in a single probabilistic value per subject which represents the level of the algorithmic support for the abnormal outcome decision. The subject level AUC is then obtained by contrasting the averaged probabilities with the outcome labels. The two ways to compute the AUC are designed to assess the instantaneous accuracy of the classifier (epoch-based AUC) as well as the accuracy when the whole recording is available (subject-based AUC).

3. Results

The statistical predictive power of each HRV feature is presented in Table 3. The “healthy” and “unhealthy” outcome labels correspond to neonates with normal and poor CCS respectively. The AUC of each HRV feature is computed on the subsection of the data for each of the pre-defined thresholds **(Set 1, Set 2, Set 3)** as discussed in Section 2.1. For a given threshold, the AUC is computed on all available epochs from the qualifying babies (**All epochs)** and on epochs that belong to episodes of hypotension (**Hypotensive events**) as defined by the threshold; the statistically significant differences (p-value<0.001) are indicated in bold. Table 3 compares the predictive power of each particular feature when MAP is taken into consideration across different definitions of hypotension.

Table 3
Predictive power of the HRV features measured using AUC. 'All epochs' represents complete recordings. 'Hypotensive events' represents only epochs under the specific MAP threshold (GA, GA+2 or GA+4) for the same set of babies. The comparison between the AUC values obtained for 'All epochs' and 'Hypotensive events' was performed using the non-parametric DeLong method for AUC comparison [38]. The significantly different AUC (p-value<0.001) are indicated in bold. "Healthy" and "unhealthy" labels correspond to neonates with normal and poor CCS outcomes.

HRV features	Set 1 MAP ≤ GA, 15 subj(8 healthy)		Set 2 MAP ≤ GA+2, 19 subj(10 healthy)		Set 3 MAP ≤ GA+4, 23 subj(12 healthy)	
	All epochs (3968)	Hypotensive events (316)	All epochs (5282)	Hypotensive events (779)	All epochs (6217)	Hypotensive events (1488)
**VLF**	**0.66**	**0.86**	**0.70**	**0.77**	0.67	0.70
**LF**	**0.74**	**0.89**	0.78	0.81	0.76	0.78
**HF**	**0.65**	**0.90**	**0.68**	**0.84**	**0.64**	**0.76**
**LF/HF**	0.69	0.73	0.72	0.68	0.73	0.69
**MeanRR**	0.82	0.84	0.78	0.83	0.82	0.85
**SDNN**	**0.65**	**0.85**	**0.68**	**0.80**	**0.65**	**0.73**
**TINN**	**0.7**	**0.89**	**0.73**	**0.83**	**0.70**	**0.77**
**Skewness**	0.52	0.60	0.56	0.51	0.58	0.52
**Kurtosis**	0.57	0.58	0.57	0.61	0.56	0.61
**ApEn**	**0.70**	**0.92**	**0.69**	**0.85**	**0.67**	**0.78**
**RMSSD**	**0.77**	**0.97**	**0.77**	**0.93**	**0.76**	**0.87**
**SDNN/RMSSD**	0.61	0.71	0.66	0.68	0.64	0.65
**AllanFactor**	**0.52**	**0.66**	**0.52**	**0.62**	0.51	0.55

Examining HRV during the episodes of low BP improves the predictive power of every relevant HRV feature in all three **Sets**. For example, in Set 3 RMSSD improved from AUC of 0.76 to 0.87, in Set 2 from AUC of 0.77 to 0.93 and in Set 1 from AUC of 0.77 to 0.97. The improvement increases with tighter definitions of **Hypotensive events** through the GA-based threshold.

Fig. 5 shows the probability density functions of the best performing RMSSD feature for the whole dataset, the subset with normal BP and the subset of **Hypotensive events** (MAP ≤ GA+4) for the cohort of preterms with both healthy and unhealthy outcomes. These PDFs are demonstrated for 1) babies of all ages (All GA, 23 subjects), 2) babies with GA > 28 subset (6 subjects, 1 poor outcome), and 3) babies with GA ≤ 28 subset (17 subjects, 10 poor outcomes). All distributions for hypotensive events demonstrate a shift in the distributions towards the right in preterm infants with a good outcome.

The separation between distributions increases when considering only hypotensive episodes, Fig. 5(c). Comparing the Fig. 5 (b, all GA) and Fig. 5 (c, all GA) the HRV of healthy newborns (as measured by RMSSD) reacts to episodes of low BP by increasing its median value from *4.21 (IQR: 3.9 to 4.6)* under normal BP to *4.96 (IQR: 4.2 to 5.5)* under **Hypotensive events**. At the same time, for unhealthy newborns no such increase is observed, with the median RMSSD value of *3.89 (IQR: 3.6 to 4.2)* under normal BP and *3.75 (IQR: 3.5 to 4.1)* under **Hypotensive events**.

Fig. 6 shows a comparison between healthy and unhealthy neonates for several relevant HRV features. The obtained results indicate that preterms with abnormal outcome have significantly lower values of HRV (p-value<0.001) even for **All epochs** dataset. This separation increases for **Hypotensive events** as indicated in Table 3 by the increased AUCs.

Fig. 7 (along with the animation in the supplementary material) shows the 3D projection of the first 3 principal components obtained using principal component analysis (PCA) applied on the **All epochs** dataset and **Hypotensive events** subset. This allows for the visualisation of the discriminative power of a linear transformation (PCA) of the 13 HRV characteristics. It can be seen that the combination of multiple features improves the separation in both cases, with very little overlap observable for **Hypotensive events**.

The aim of any decision support system should be to provide high sensitivity, correctly detecting as many (ideally all) of the 'unhealthy' preterm babies in the NICU. So there is a trade-off here – the choice of a tighter threshold (e.g. *MAP ≤ GA*) will provide a better performance. This performance, however, will come at the cost that some unhealthy babies, who do not exhibit any such deep dips in BP across their recording, will be excluded totally from the analysis. This would lead to the risk that a substantial number of unhealthy neonates (in our case 4) would not be processed and correctly detected by the proposed decision support system.

Table 4
AUC for short-term outcome prediction using various combination of HRV and BP features (*MAP≤GA+4*).

	Predictions on Hypotensive event		Predictions on All epochs	
	HRV	HRV & MAP	HRV	HRV & MAP
	23 subjects, 1488 epochs		23 subjects, 6217 epochs	
**Epoch AUC**	0.91	0.90	0.83	0.88
**Subject AUC**	0.97	0.96	0.94	0.95

The combination of features with boosted decision trees was investigated for **Set 3** (*MAP ≤ GA+4*) since for this threshold the separation between healthy and unhealthy newborns is more challenging while all the data can be used. The results of the performance of classifier-based systems are presented in Table 4. The prediction systems were designed to run on **All epochs** and **Hypotensive events** and were scored with respect to their epoch or subject level accuracies.

The HRV-based system which is designed to operate on **All epochs** results in an AUC of 0.83 and 0.94, for the epoch and subject level metrics. It can be seen from Table 4 that the subject-level performance is higher than the epoch-level. The system improves from an AUC of 0.83 to an AUC of 0.91 when observing only **Hypotensive events.** An additional experiment was conducted where MAP information was provided to the classifiers in the form of two additional features: *MAP–GA* and *MAP/GA*. This additional information has improved results on **All epochs**. More specifically, for the model trained on the HRV & MAP features, the AUC increased from 0.83 to 0.88. No consistent benefit was observed for **Hypotensive events** where the dataset has been already pre-selected based on the MAP thresholds and thus MAP features do not bring any new information. The overall best results both for the epoch and subject-level metrics are obtained with the HRV-based system on **Hypotensive events** with an AUC of 0.91 and 0.97, respectively.

The importance of the various HRV features was derived from the model trained to use HRV system for **Hypotensive events**. The importance is averaged across 23 models from the patient independent LOO procedure. Fig. 8 shows that RMSSD, MeanRR, LF/HF, ApEn and LF are the top five most important HRV features. Fig. 9 illustrates the top ten two-feature interactions which have

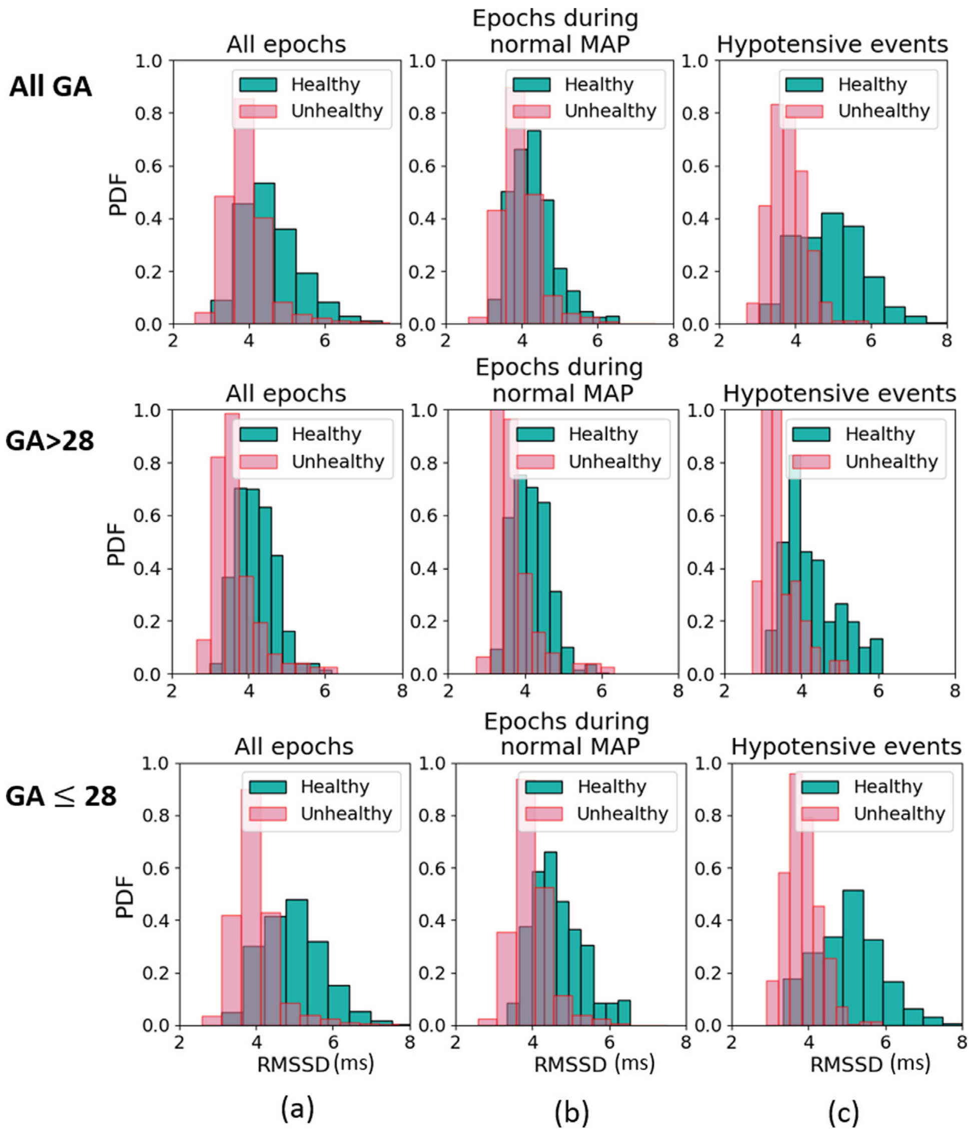

Fig. 5. PDF for the RMSSD feature. Original subset (a) contains RMSSD feature values from the complete recordings. Normal (b) and hypotensive (c) subsets represent RMSSD feature extracted during episodes of normal BP (MAP > GA+4) and during hypotensive events (MAP ≤ GA+4). PDFs are given for all 23 subjects (All GA), babies with GA > 28 (6 subjects) and babies with GA ≤ 28 (17 subjects).

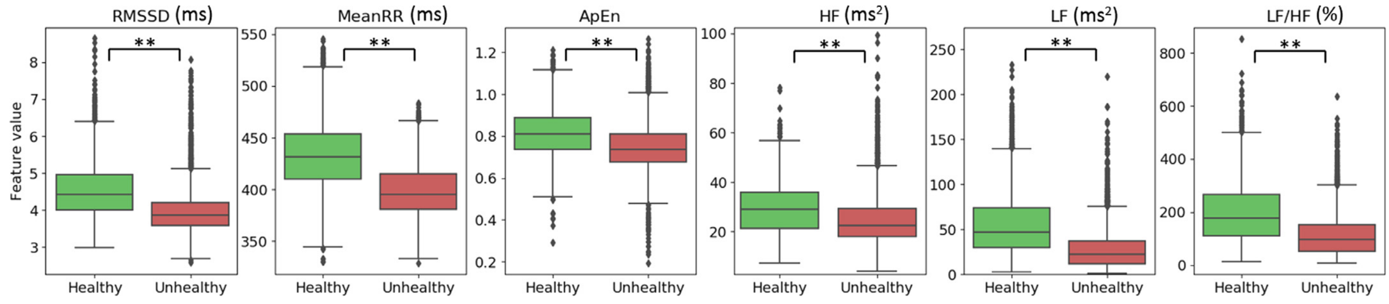

Fig. 6. Values of HRV features extracted from **All epochs** for healthy and unhealthy preterm neonates. Boxplot analyses show the median, 25th and 75th percentiles, and the outliers. '**' represent statistically significant differences between groups with *p*-value<0.001 using Mann-Whitney U test. The predictive power of the features quantified by AUC is presented in Table 3 (**All epochs**, *Set 3*).

the greatest contribution to the preterm outcome prediction. It can be seen that the RMSSD feature is involved in many of the important interactions. This result is consistent with the single feature statistics, where RMSSD showed the highest predictive power (AUC = 0.87, Table 3).

Fig. 10 illustrates the most frequently selected sets of hyperparameter during the internal CV for each of the iterations of the LOO routine, for the HRV-based system for **Hypotensive events**. The radius of each sphere indicates the frequency with which a particular set of hyperparameters is selected.

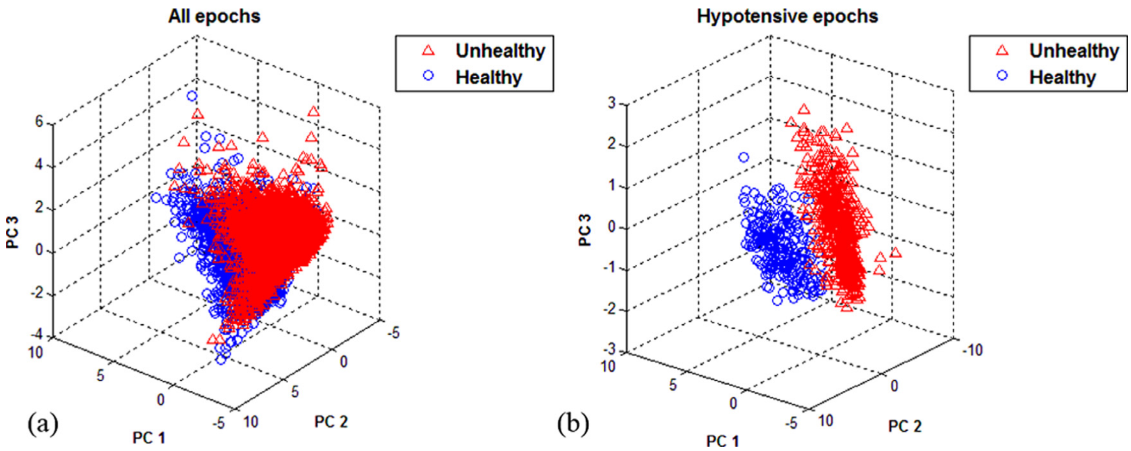

Fig. 7. Principal component analysis (PCA) of the dataset comprised of all epochs (a) and epochs during hypotensive episodes ($MAP \leq GA+2$) (b). In this study PCA is used as a tool for exploratory feature analysis which is aimed at checking the discriminative power of the HRV feature set with respect to the short-term outcome of the preterm neonate.

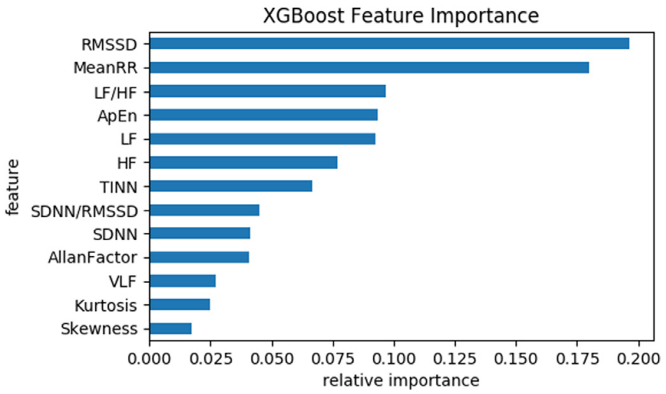

Fig. 8. Mean of the feature importance (gain) reported by the boosted decision tree classifier trained on the HRV features extracted during episodes of low BP ($MAP \leq GA+4$).

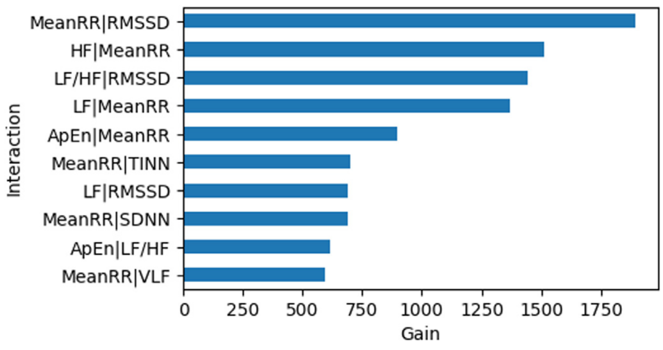

Fig. 9. Representation of the importance (gain) of the top ten two-feature interactions for the short-term outcome prediction. The system is trained on all HRV features extracted during the episodes of low BP ($MAP \leq GA+4$).

The designed systems allow for the continuous observation of the probabilities of morbidity. Fig. 11 depicts the probabilistic traces for two subjects observed for 10 h, one with a healthy and one with an unhealthy outcome.

Fig. 12 presents the probabilistic output for the same unhealthy subject as in Fig. 11 across longer recording time, where behavior of probability during the **Hypotensive events** is highlighted. The thresholded trace of MAP illustrates the definition of hypotensive events.

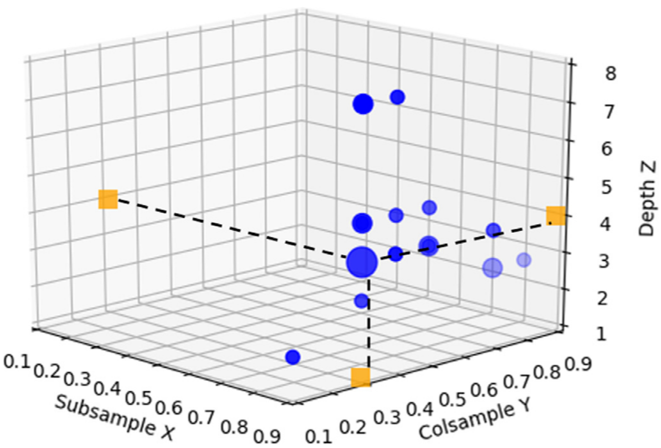

Fig. 10. The density of selected tensors of three main hyperparameters obtained during the LOO routine for the HRV-based systems for **Hypotensive events** ($MAP \leq GA+4$). The most frequently selected parameters are Subsample=0.9, Colsample=0.3 and Depth=4. The projections of the parameters are represented with dash lines.

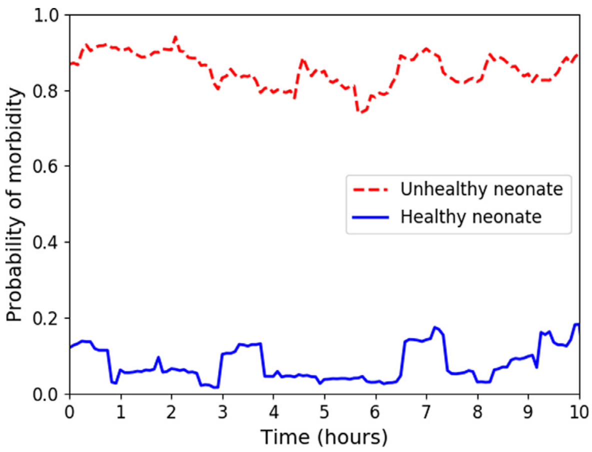

Fig. 11. An example of the system output as a continuous probabilistic trace obtained during 10 h for one healthy (GA=28 weeks, blue solid line) and one unhealthy (GA=23 weeks, red dashed line) patients. The system is trained and evaluated on the **All epochs** dataset.

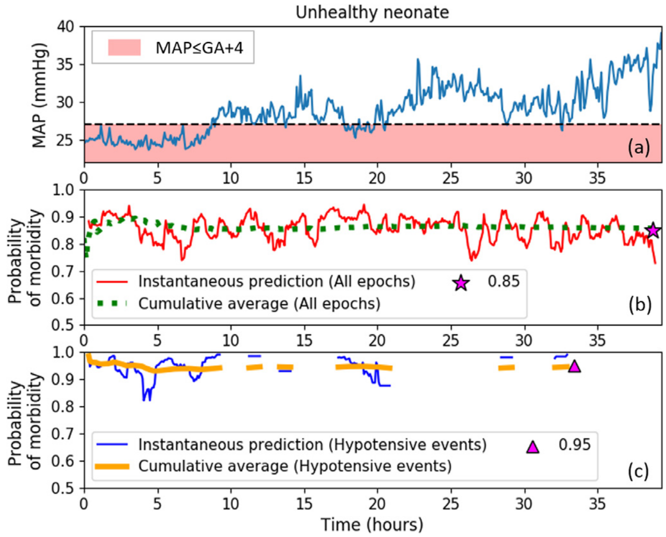

Fig. 12. Comparison of the probabilistic traces for an unhealthy neonate (GA=23 weeks) obtained from the two models trained on HRV features extracted from either the **All epochs** (b) or **Hypotensive events** (MAP ≤ GA+4) (c) datasets. The model trained on the **All epochs** (b) is represented by instantaneous (red solid thin line) and cumulative (green dashed bold line) probabilistic values. The **Hypotensive events** model (c) is represented by the instantaneous probabilistic values (solid blue thin line) and the cumulative average of prediction (solid orange bold line). An average of the morbidity prediction for each model is 0.85 and 0.95 respectively.

4. Discussion

4.1. Statistical analysis of HRV features

This is the first study which investigates an association between HRV and neonatal health outcome for preterms in the context of hypotensive episodes. The results from Table 3 indicate that several HRV features are relevant for the chosen task and have predictive power with respect to the outcome. These features are RMSSD, ApEn, HF, SDNN, TINN. Moreover, HRV features are shown to be sensitive to BP and the features extracted during the episodes of low MAP show better separation between good and poor outcomes. Interestingly, the tighter the overall threshold on the definition of the hypotension, the larger the improvement in AUC that is observed when comparing **All epochs** with **Hypotensive events**. It is worth noting that each **Set** was restricted to include only those newborns with the presence of hypotensive events as defined by the corresponding GA-based thresholds. For each **Set, Hypotensive events** come from the same subjects and represent a subset of **All epochs**. Thus, the obtained improvement is purely attributable to the effect that BP has on HRV rather than an effect which could have been caused by including extra subjects in the category of **All epochs**.

From Fig. 5(a) the RMSDD feature as a measure of the energy of the successive differences between neighbouring RR intervals is generally higher for the healthy cohort than unhealthy cohort with a substantial overlap between the two distributions. This indicates that the HRV of healthy preterms react to the drop in BP. In [39] the increased level of interaction between EEG and BP in preterms was shown to be associated with lower risks of illness severity. Similarly here as shown in Fig. 5, a strong interaction between HRV and BP is associated with a good outcome, whereas the lack of interaction is associated with a poor outcome.

The most important HRV feature, RMSSD, estimates the short-term component of HRV and represents the parasympathetic activity of the heart. In [14] RMSSD was shown to improve 2-year outcome prediction for preterm neonates, with lower RMSSD values corresponding to minor neurologic dysfunction and cerebral palsy. Other studies have reported that the RMSSD feature is a good early predictor of a septic shock for adults [40] and sudden unexplained death in epilepsy [41]. Decreased HRV was previously associated with hypoxic brain injury for newborns [42] and with the failure of the first extubation for the preterm infants [18]. This study has also shown that RMSSD values are lower for unhealthy preterm neonates.

Other HRV characteristics which are shown to be relevant to the short-term outcome of the preterm neonate (Table 3) were previously reported to be indicative of the neonatal health status. For instance, the ApEn measure was proposed by Pincus [26] who previously reported reduced ApEn in distressed foetuses [43] and sick newborns [44]. This can be interpreted as an increased regularity of cardiac rhythm. Further application of entropy estimators to neonatal HRV revealed an association with neonatal sepsis [45]. In this study, lower ApEn values were obtained for unhealthy preterms. Another examples is the TINN characteristic which was previously shown [11] to be significantly associated with severity of the hypoxic ischemic encephalopathy for full-term neonates, with lower TINN values corresponding to sick neonates. Similarly, lower TINN values in this study are shown to be associated with an increased risk of morbidity of the preterm.

Neonatal HRV evaluated by spectral analysis is usually characterised by the dominant activity in the LF band. LF is mediated predominantly by the sympathetic component of the autonomic nervous system. LF of HRV is also considered to represent Mayer waves of BP changes [46]. Mayer waves are cyclic changes in arterial BP at a frequency of about 0.1 Hz resulting from oscillation of sympathetic vasomotor tone [47]. Calculation of the LF/HF ratio is a method to establish the ratio between the components of the autonomic nervous system – sympathetic/parasympathetic balance [25]. Reduced LF and HF features were reported for neonates with hypoxic ischemic encephalopathy, implying the reduction of autonomic function [11]. Similar findings were observed in the current study with a decreased power in the LF and HF bands (Fig. 6). In general, both human and animal studies have supported the finding that reduced spectral power in the low-frequency component is indicative of an impaired function of the autonomic nervous system [48,49].

Preterm neonates are at high risk of developing sepsis, which is known to be an important risk factor for a prolonged stay at the hospital and even death [45, 50]. The heart rate observation monitor (HeRO) which allows for the detection of sepsis in preterm infants is based on three HRV characteristics (SDNN, sample asymmetry and sample entropy) combined using logistic regression. This algorithm generates a clinical score that quantifies the risk of sepsis in the next 24 h. In comparison, the system developed in this study utilises a more powerful tree-based model to generate a final decision, which is based on 5 major neonatal complications (including sepsis). At the same time, a feature set of 13 HRV characteristics was used in this study which interestingly only shares one common feature with the HeRO monitor – SDNN.

From Table 1 it can be seen that 3 out of a total of 11 infants with poor outcome were contributed solely by sepsis, 3 poor outcomes resulted from complications other than sepsis (NEC and BPD); 4 poor outcomes were contributed by the combination of sepsis and other complications; for 1 remaining neonate with sepsis, the information about other complications was not available. None of the preterms in the dataset exhibited ROP (stage 2 or greater). The system can predict the correct outcome for 2 out of 3 infants without sepsis and for 8 out of 8 infants with sepsis. For this reason, the system applicability goes beyond sepsis-affected babies.

4.2. Machine learning

The statistical predictive power measured by the AUC represents statistical descriptive linear inference of a single signal characteristic. This measure of separation is computed from all the data and small intra-subject data variability may impact the conclusions regarding whether the observed phenomenon generalizes across various subjects. To assess the out-of-sample predictive power of a feature or a combination of features then a subject independent performance assessment must be used. With machine learning methods, the LOO performance provides estimates of 1) whether individual features bear predictive power that generalizes across patients, 2) the predictive power of non-linear correlation between the feature and the label, 3) the above for a combination of features. Fig. 7 indicates that a combination of features can indeed increase the separation between good and poor outcomes.

4.2.1. Combination of features with boosted decision trees

From Table 4, it can be seen that the performance achieved with machine learning has improved when compared with the AUCs of single features (Table 3, Set 3). It is important to stress that the performance is assessed using the out-of-sample patient predictions. Observing the results it can be concluded that MAP plays an important role in assessing the wellbeing of a preterm newborn. When MAP is used to drive the definition of hypotensive events, similar to descriptive statistics the performance using machine learning is better, when tested on **Hypotensive events** as compared to **All epochs**. Interestingly, when MAP is not used to identify hypotensive events (All epochs), the inclusion of MAP-based features similarly improved the performance (HRV vs HRV & MAP).

Comparing the epoch-based and subject-based scores, we can see that the accumulation of probabilistic information across the whole recording increases the discriminatory capacity of the classifier, in all considered cases. The performance reported at the subject level assumes the availability of the whole recording before decision making. This means that the system achieves this performance for prognostication purposes rather than for on-the-fly monitoring and decision making.

Overall, the use of machine learning allowed us to obtain an AUC of 0.97 for Set 3, which is similar to the result obtained with descriptive statistics for a single best feature for Set 1 in Table 3. However, it is important to note that Set 3 includes 8 more babies, 4 with poor outcome, who could benefit from the continuous physiological data analysis.

4.2.2. Feature importance & interaction

The ranking of HRV features for the best system based on information gain shown in Fig. 8, is consistent with statistical descriptive analysis (Table 3). The most important feature appeared to be RMSSD which is followed by MeanRR. Additionally, according to the level of feature interaction (Fig. 9), MeanRR is the feature which is involved in the majority (7 out of 10) of the top ten two-way interactions. In other words, when considered in a combination with other HRV characteristics, MeanRR becomes even more important. This indicates that the absolute heart rate information is complementary to many HRV features for the chosen task and HRV should be considered in the context of mean HR. This particular result emphasises the power of multi-feature classification.

4.2.3. Stability of the models

It can be seen from Fig. 10 that while an exhaustive search was performed on the wide range of hyperparameters, the choice of the best hyperparameter vector is stable as represented by the densely located sets of best hyperparameters. According to the CV performance metric, the best set of hyperparameters for the classifier trained on HRV features from **Hypotensive events** dataset is located at (0.9, 0.3, 4), where x, y, and z are the fraction of the data, features, and depth of the tree, respectively. The tree depth of 4 corresponds to a tree, which is capable of creating complex decision boundaries and benefiting from the interaction between features. Every tree is built on a randomly selected set of 90% of data and 30% of all features. The fact that 30% of features are selected supports the conclusions from the analysis of feature importance and the statistical analysis which indicate the presence of many irrelevant features. If we were to generate a single model on all available training data to be used in clinical practice, then these hyperparameters should be used to train the final classifier.

4.2.4. Decision support tools

From Table 4, various decision support tools can be potentially designed depending on the information available and the purpose. Non-invasive techniques for BP measurement are known to be unreliable in small and sick infants. An excessive intervention in sick preterm infants is undesirable and a gold standard invasive BP recordings at times may be difficult to obtain [51]. On the other hand, non-invasive ECG is routinely recorded in preterms. In this context, depending on the physiological data available we have provided 3 possible decision support tools for the clinical prediction of short-term outcome: 1) a tool based only on HRV, that assumes that MAP is unavailable and which targets instantaneous diagnosis (All epochs, HRV, Epoch AUC) which will provide an AUC of 0.83; 2) a tool which uses HRV and MAP and which also targets instantaneous diagnosis running on All epochs, not only on those that meet the threshold MAP $\leq$ GA+4, will improve the performance to an AUC of 0.88 (All epochs, HRV & MAP, Epoch AUC); 3) if MAP is available and episodes of hypotension can be identified, then a decision support tool with the same purpose can achieve an AUC of 0.91 (Hypotensive events, HRV, Epoch AUC). If the purpose is switched to prognostication, then an AUC of 0.94 can be obtained without MAP and 0.97 with MAP focusing on hypotensive events.

The GA-based rule of the definition of hypotension is widely used in clinical practice for the treatment of low BP in the preterm to decide whether the current low BP is of any risk to the wellbeing of the neonate. In this study, the HRV-based systems, **All epoch,** and **Hypotensive events** were scored with respect to their epoch and subject level accuracies. Each system continuously outputs a probability of morbidity for every five-minute window in real-time as shown in Fig. 12. Such systems can be used in clinical practice as a decision support tool for the monitoring of preterm neonates, who may have low BP. The MAP recording along with the probability traces which are shown in Fig. 12, of the two systems, **All epochs** and **Hypotensive events**, give an additional insight into the interrelation between HRV, BP and neonatal health outcome in the context of episodes of low MAP. It can be seen that whereas the system trained on **All epochs** (Fig. 12(b)) outputs probabilities for every segment of data, the system trained on **Hypotensive events** (Fig. 12(c)) outputs probabilities only on the segments where MAP falls below the predefined threshold. The system designed to operate on **Hypotensive events** outperforms the one designed on **All epochs** as shown in Table 4. Similarly, in Fig. 12, the continuous and cumulative average probability of morbidity for a given unhealthy neonate is higher using **Hypotensive events**, 0.95, as compared to 0.85 for **All epochs**.

It can be seen from Fig. 12 that the cumulative average of probability stabilises quickly and thus can approximate the final average probability which is used in the subject-level assessment of accuracy. This implies that with increasing monitoring time, tools

designed for diagnosis can approach the performance of those designed for prognostication. This is represented in Fig. 12(b) as a red solid thin line for instantaneous predictions and green dashed bold line for cumulative average probabilistic values, for the HRV only decision support system. A solid blue thin line for instantaneous predictions and solid orange bold line for cumulative average probabilistic values in Fig. 12(c) are indicative of the same effect on the HRV system for hypotensive events.

4.3. Limitations

While the thresholds used to identify low BP are arbitrary, the first definition was based on the MAP≤ GA rule, which is one of the most commonly utilized definitions worldwide. The other two definitions /thresholds used were arbitrary and are not supported by any clinical use. The natural rise in BP over the first day is approximately 5 mmHg. These two definitions of +2 and +4 would remain less than the normal rise noted in healthy preterm infants.

Other limitations include the potential confounding effect of iatrogenic interventions, such as medications, intubation or mechanical ventilation on BP, HRV and the outcome variable (CCS). The availability of additional clinical information which accounts for inadequate oxygen delivery to the tissue (e.g. anemia) can potentially help to better define the criteria for hypotension definition. These data, however, was not consistently available for the purpose of this retrospective study.

The results obtained in this study demonstrate the sensitivity of HRV to changes in BP. If a lower GA-based threshold is selected, then any event now highlighted as “hypotensive” is, therefore, more severe (lower BP) which results here in an improvement in discriminatory power of the features and an increase in the AUC. However, this comes at the cost of a reduced number of subjects (fewer babies will have hypotensive events, with this lower threshold) and thus a reduced confidence in the observed changes for this lower threshold. It is also possible to show that with a low GA-based threshold 4 infants with a poor outcome would not be identified and not subjected to AI-assisted monitoring.

The population of preterm neonates is extremely vulnerable, and it is usually difficult to get the permission for any kind of intervention from neonatologists. This, therefore, may introduce a bias in the clinical data, as most of the time only sick babies are monitored. In order to better represent the general neonatal population, a larger cohort of preterm infants should be used to test the generalisability of the developed system. In this work only preterm infants that had continuous simultaneous ECG and MAP recordings that were of good signal quality were studied.

5. Conclusion

HRV features extracted during episodes of low BP in preterm infants were shown to correlate with short-term health outcomes as represented by a clinical course score, with a single best feature (RMSSD) reaching an AUC of 0.87. Combining multimodal data of HRV and BP with a machine learning algorithm, a decision support tool for clinical prediction of short-term outcome in preterms with low BP was constructed. ECG with or without BP records are usually available soon after birth and this work presents a promising step towards the use of physiological data in building an objective decision support tool for the clinical prediction of short-term outcome in preterms. Future research will concentrate on the incorporation of other modalities, such as clinical data, and testing on larger cohorts, for the prediction of both long-term and short-term outcome in preterms. In addition, the algorithmic calculation of newborn HRV features used in currently available sepsis monitors such as the HeRO monitor should be investigated to assess the level of complementarity of this work.

Declaration of competing interest

The author declares no conflict of interest.

Acknowledgment

This research was supported by a Science Foundation Ireland Research Centers Award (SFI 12/RC/2272).

Supplementary materials

Supplementary material associated with this article can be found, in the online version, at doi:10.1016/j.cmpb.2019.104996.

Appendix

Given a set of N training examples of the form $\{(x_1,y_1),\ldots,(x_N,y_N)\}$ such that $x_i \in R^d$ is a D-dimensional feature vector of the i th example and y_i is its label, the goal is to create a model, f, to predict values in the form, $\hat{y} = \sum_k f_k(x)$. At each stage of gradient boosting, $1 \le k \le K$, a model, f_k, is constructed. A new improved model is then constructed, $f_{k+1}(x)$, that adds an estimator, h, as $f_{k+1}(x) = f_k(x) + h(x)$, by fitting h to the residuals, $(y - f_k(x))$. At each iteration, the algorithm identifies the new function f_k that optimizes the following objective function:

$$Obj = \sum_i l(y_i, \hat{y}_i) + \sum_k \Omega(f_k), \tag{A.1}$$

where

$$\Omega(f) = \gamma T + \frac{1}{2}\lambda \sum_{j=1}^{T} w_j^2 \tag{A.2}$$

Here l is a differentiable convex loss function that measures the difference between the prediction $\hat{y}_i$ and the target y_i. Each f_k corresponds to an independent tree structure with a vector of leaf weights w; γ and λ are regularization parameters. The second term, Ω, penalizes the complexity of the regression tree functions in terms of the number of leaves in the tree T. The regularization term Ω also helps to smooth the learnt weights to avoid overfitting.

The final objective function optimized using a second-order Taylor approximation is defined as follows:

$$Obj^{(t)} = \sum_{i=1}^{N} l\left(y_i,\ \hat{y}_i^{(t-1)} + f_t(x_i)\right) + \sum_{i=1}^{t} \Omega(f_i)$$
$$\approx \sum_{i=1}^{N} \left[g_i f_t(x_i) + \frac{1}{2} h_i f_t^2(x_i) \right] + \Omega(f_t), \tag{A.3}$$

where $g_i = \partial_{\hat{y}_i^{(t-1)}} l(\hat{y}_i^{(t-1)},\ y_i)$ and $h_i = \partial^2_{\hat{y}_i^{(t-1)}} l(\hat{y}_i^{(t-1)},\ y_i)$ are the first and the second order derivatives of the loss function at iteration t. This results in the following optimal weight w_j^* on the jth leaf:

$$w_j^* = -\frac{\sum_{i \in I_j} g_i}{\sum_{i \in I_j} h_i + \lambda} \tag{A.4}$$

where I_j defines a set of indices of data points that are assigned to the j-th leaf. Learning the structure of the tree f_t implies deciding on how to split the data using features. After each split, the leaf is converted to an internal node. The gain measures the improvement brought by each split and is defined as:

$$Gain = \frac{1}{2}\left[\frac{\left(\sum_{i \in I_L} g_i\right)^2}{\sum_{i \in I_L} h_i + \lambda} + \frac{\left(\sum_{i \in I_R} g_i\right)^2}{\sum_{i \in I_R} h_i + \lambda} - \frac{\left(\sum_{i \in I} g_i\right)^2}{\sum_{i \in I} h_i + \lambda} \right] - \gamma \tag{A.5}$$

The first and second terms (Eq. A.5) represent scores for the left and right leaves added; the third term is a score of the original leaf prior to the split. The tree is built to the predefined maximum depth. The nodes with negative gain are then pruned in a bottom-up order. If the gain is smaller than the regularization parameter γ, the split is not produced. Gain is also used as a parameter which quantifies the feature importance, by averaging the gain of each feature for all splits and all trees.

References

[1] WHO | Preterm birth, WHO. (n.d.). http://www.who.int/mediacentre/factsheets/fs363/en/ (accessed August 23, 2016).
[2] E.M. Dempsey, K.J. Barrington, Diagnostic criteria and therapeutic interventions for the hypotensive very low birth weight infant, J. Perinatol. 26 (2006) 677–681, doi:10.1038/sj.jp.7211579.
[3] M. Laughon, C. Bose, E. Allred, T.M. O'Shea, L.J. Van Marter, F. Bednarek, A. Leviton, Factors associated with treatment for hypotension in extremely low gestational age newborns during the first postnatal week, Pediatrics 119 (2007) 273–280, doi:10.1542/peds.2006-1138.
[4] Development of audit measures and guidelines for good practice in the management of neonatal respiratory distress syndrome. Report of a joint working group of the british association of perinatal medicine and the research unit of the royal college of physicians, Arch. Dis. Child. 67 (1992) 1221–1227.
[5] A. Heuchan, N. Evans, S. Henderson, J. Simpson, Perinatal risk factors for major intraventricular Haemorrhage in the Australian and New Zealand neonatal Network, 1995–97, Arch. Dis. Child. Fetal Neonatal Ed. 86 (2002) F86–F90, doi:10.1136/fn.86.2.F86.
[6] F. De Zegher, G. Van Den Berghe, H. Devlieger, E. Eggermont, J.D. Veldhuis, Dopamine inhibits growth hormone and prolactin secretion in the human newborn, Pediatr. Res. 34 (1993) 642–645, doi:10.1203/00006450-199311000-00016.
[7] A.R. Synnes, L.-Y. Chien, A. Peliowski, R. Baboolal, S.K. Lee, Variations in intraventricular hemorrhage incidence rates among Canadian neonatal intensive care units, J. Pediatr. 138 (2001) 525–531, doi:10.1067/mpd.2001.111822.
[8] E.M. Dempsey, K.J. Barrington, N. Marlow, C.P. O'Donnell, J. Miletin, G. Naulaers, P.-Y. Cheung, D. Corcoran, G. Pons, Z. Stranak, D.V. Laere, on behalf of the H, Consortium, management of hypotension in preterm infants (The HIP Trial): a randomised controlled trial of hypotension management in extremely low gestational age newborns, Neonatology 105 (2014) 275–281, doi:10.1159/000357553.
[9] S. Akselrod, D. Gordon, F.A. Ubel, D.C. Shannon, A.C. Berger, R.J. Cohen, Power spectrum analysis of heart rate fluctuation: a quantitative probe of beat-to-beat cardiovascular control, Science 213 (1981) 220–222.
[10] A. Temko, O. Doyle, D. Murray, G. Lightbody, G. Boylan, W. Marnane, Multimodal predictor of neurodevelopmental outcome in newborns with hypoxic-ischaemic encephalopathy, Comput. Biol. Med. 63 (2015) 169–177, doi:10.1016/j.compbiomed.2015.05.017.
[11] R.M. Goulding, N.J. Stevenson, D.M. Murray, V. Livingstone, P.M. Filan, G.B. Boylan, Heart rate variability in hypoxic ischemic encephalopathy: correlation with EEG grade and 2-y neurodevelopmental outcome, Pediatr. Res. 77 (2015) 681–687, doi:10.1038/pr.2015.28.
[12] R.O. Lloyd, J.M. O'Toole, V. Livingstone, W.D. Hutch, E. Pavlidis, A.-M. Cronin, E.M. Dempsey, P.M. Filan, G.B. Boylan, Predicting 2-y outcome in preterm infants using early multimodal physiological monitoring, Pediatr. Res. 80 (2016) 382–388, doi:10.1038/pr.2016.92.
[13] M. Périvier, J.-C. Rozé, G. Gascoin, M. Hanf, B. Branger, V. Rouger, I. Berlie, Y. Montcho, Y. Péréon, C. Flamant, S.N.T. Tich, Neonatal EEG and neurodevelopmental outcome in preterm infants born before 32 weeks, Arch. Dis. Child. - Fetal Neonatal Ed. 101 (2016) F253–F259, doi:10.1136/archdischild-2015-308664.
[14] L. Dimitrijević, B. Bjelaković, H. Čolović, A. Mikov, V. Živković, M. Kocić, S. Lukić, Assessment of general movements and heart rate variability in prediction of neurodevelopmental outcome in preterm infants, Early Hum. Dev. 99 (2016) 7–12, doi:10.1016/j.earlhumdev.2016.05.014.
[15] K.K. Doheny, C. Palmer, K.N. Browning, P. Jairath, D. Liao, F. He, R.A. Travagli, Diminished vagal tone is a predictive biomarker of necrotizing enterocolitis-risk in preterm infants, Neurogastroenterol. Motil. 26 (2014) 832–840, doi:10.1111/nmo.12337.
[16] F.J. Bohanon, A.A. Mrazek, M.T. Shabana, S. Mims, G.L. Radhakrishnan, G.C. Kramer, R.S. Radhakrishnan, Heart rate variability analysis is more sensitive at identifying neonatal sepsis than conventional vital signs, Am. J. Surg. 210 (2015) 661–667, doi:10.1016/j.amjsurg.2015.06.002.
[17] A. Rakow, M. Katz-Salamon, M. Ericson, A. Edner, M. Vanpée, Decreased heart rate variability in children born with low birth weight, Pediatr. Res. 74 (2013) 339–343, doi:10.1038/pr.2013.97.
[18] J. Kaczmarek, S. Chawla, C. Marchica, M. Dwaihy, L. Grundy, G.M. Sant'Anna, Heart rate variability and extubation readiness in extremely preterm infants, Neonatology 104 (2013) 42–48, doi:10.1159/000347101.
[19] D. Rassi, A. Mishin, Y.E. Zhuravlev, J. Matthes, Time domain correlation analysis of heart rate variability in preterm neonates, Early Hum. Dev. 81 (2005) 341–350, doi:10.1016/j.earlhumdev.2004.09.002.
[20] L.A. Cabal, B. Siassi, B. Zanini, J.E. Hodgman, E.E. Hon, Factors affecting heart rate variability in preterm infants, Pediatrics 65 (1980) 50–56.
[21] K.D. Fairchild, J.L. Aschner, HeRO monitoring to reduce mortality in NICU patients, Res. Rep. Neonatol. (2012), doi:10.2147/RRN.S32570.
[22] J.R. Moorman, W.A. Carlo, J. Kattwinkel, R.L. Schelonka, P.J. Porcelli, C.T. Navarrete, E. Bancalari, J.L. Aschner, M. Whit Walker, J.A. Perez, C. Palmer, G.J. Stukenborg, D.E. Lake, T. Michael O'Shea, Mortality reduction by heart rate characteristic monitoring in very low birth weight neonates: a randomized trial, J. Pediatr 159 (2011) 900–906, doi:10.1016/j.jpeds.2011.06.044.
[23] Z. Stranak, J. Semberova, K. Barrington, C. O'Donnell, N. Marlow, G. Naulaers, E. Dempsey, HIP consortium, international survey on diagnosis and management of hypotension in extremely preterm babies, Eur. J. Pediatr. 173 (2014) 793–798, doi:10.1007/s00431-013-2251-9.
[24] J. Pan, W.J. Tompkins, A real-time QRS detection algorithm, IEEE Trans. Biomed. Eng. 32 (1985) 230–236, doi:10.1109/TBME.1985.325532.
[25] T.F. of the E.S. of C. the N.A.S. of P, Electrophysiology, heart rate variability: standards of measurement, physiological Interpretation, and clinical use, Circulation 93 (1996) 1043–1065, doi:10.1161/01.CIR.93.5.1043.
[26] S.M. Pincus, Approximate entropy as a measure of system complexity, Proc. Natl. Acad. Sci. U. S. A. 88 (1991) 2297–2301.
[27] S.B. Lowen, M.C. Teich, The periodogram and allan variance reveal fractal exponents greater than unity in auditory-nerve spike trains, Acoust. Soc. Am. J. 99 (1996) 3585–3591, doi:10.1121/1.414979.
[28] J. Sacha, Why should one normalize heart rate variability with respect to average heart rate, Front. Physiol. 4 (2013), doi:10.3389/fphys.2013.00306.
[29] F.A. Selig, E.R. Tonolli, E.V.C.M. da Silva, M.F. de Godoy, Heart rate variability in preterm and term neonates, Arq. Bras. Cardiol. 96 (2011) 443–449.
[30] S.J. Mason, N.E. Graham, Areas beneath the relative operating characteristics (ROC) and relative operating levels (ROL) curves: statistical significance and interpretation, Q. J. R. Meteorol. Soc. 128 (2002) 2145–2166, doi:10.1256/003590002320603584.
[31] R.E. Schapire, The boosting approach to machine learning: an overview, in: D.D. Denison, M.H. Hansen, C.C. Holmes, B. Mallick, B. Yu (Eds.), Nonlinear Estim. Classif., Springer, New York, 2003, pp. 149–171, doi:10.1007/978-0-387-21579-2_9.
[32] J.H. Friedman, Greedy function approximation: A a gradient boosting machine., Ann. Stat. 29 (2001) 1189–1232. doi:10.1214/aos/1013203451.
[33] T. Chen, C. Guestrin, XGBoost: A a scalable tree boosting system, ArXiv160302754 Cs. (2016) 785–794. doi:10.1145/2939672.2939785.
[34] Far0n, xgbfi: XGBoost Feature Interactions & Importance, 2018. https://github.com/Far0n/xgbfi.
[35] L. Breiman, Random forests, Mach. Learn. 45 (2001) 5–32, doi:10.1023/A:1010933404324.
[36] V. Vapnik, Estimation of Dependences Based On Empirical Data, Springer-Verlag, New York, 2006 www.springer.com/gp/book/9780387308654 (accessed May 8, 2018).
[37] T.G. Dietterich, Approximate statistical tests for comparing supervised classification learning algorithms, Neural Comput 10 (1998) 1895–1923, doi:10.1162/089976698300017197.
[38] X. Robin, N. Turck, A. Hainard, N. Tiberti, F. Lisacek, J.-C. Sanchez, M. Müller, pROC: an open-source package for r and S+ to analyze and compare ROC curves, BMC Bioinf. 12 (2011) 77, doi:10.1186/1471-2105-12-77.
[39] O. Semenova, G. Lightbody, J.M. O'Toole, G. Boylan, E. Dempsey, A. Temko, Coupling between mean blood pressure and EEG in preterm neonates is associated with reduced illness severity scores, PLOS ONE 13 (2018) e0199587, doi:10.1371/journal.pone.0199587.
[40] W.-L. Chen, C.-D. Kuo, Characteristics of heart rate variability can predict impending septic shock in emergency department patients with sepsis, Acad. Emerg. Med. 14 (2007) 392–397, doi:10.1111/j.1553-2712.2007.tb01796.x.
[41] C.M. DeGiorgio, P. Miller, S. Meymandi, A. Chin, J. Epps, S. Gordon, J. Gornbein, R.M. Harper, RMSSD, a measure of heart rate variability, is associated with risk factors for SUDEP: the SUDEP-7 inventory, Epilepsy Behav. EB. 19 (2010) 78–81, doi:10.1016/j.yebeh.2010.06.011.
[42] V. Matić, P.J. Cherian, D. Widjaja, K. Jansen, G. Naulaers, S. Van Huffel, M. De Vos, Heart rate variability in newborns with hypoxic brain injury, Adv. Exp. Med. Biol. 789 (2013) 43–48, doi:10.1007/978-1-4614-7411-1_7.
[43] S.M. Pincus, R.R. Viscarello, Approximate entropy: a regularity measure for fetal heart rate analysis, Obstet. Gynecol. 79 (1992) 249–255.
[44] S.M. Pincus, I.M. Gladstone, R.A. Ehrenkranz, A regularity statistic for medical data analysis, J. Clin. Monit. 7 (1991) 335–345, doi:10.1007/BF01619355.
[45] D.E. Lake, J.S. Richman, M.P. Griffin, J.R. Moorman, Sample entropy analysis of neonatal heart rate variability, Am. J. Physiol.-Regul. Integr. Comp. Physiol. 283 (2002) R789–R797, doi:10.1152/ajpregu.00069.2002.
[46] A.E. Draghici, J.A. Taylor, The physiological basis and measurement of heart rate variability in humans, J. Physiol. Anthropol. (2016) 35, doi:10.1186/s40101-016-0113-7.
[47] M. Pagani, D. Lucini, O. Rimoldi, R. Furlan, S. Piazza, A. Porta, A. Malliani, Low and high frequency components of blood pressure variability, Ann. N. Y. Acad. Sci. 783 (1996) 10–23.

[48] G. Piccirillo, M. Ogawa, J. Song, V.J. Chong, B. Joung, S. Han, D. Magrì, L.S. Chen, S.-.F. Lin, P.-.S. Chen, Power spectral analysis of heart rate variability and autonomic nervous system activity measured directly in healthy dogs and dogs with tachycardia-induced heart failure, Heart Rhythm 6 (2009) 546–552, doi:10.1016/j.hrthm.2009.01.006.

[49] A.J. Shah, R. Lampert, J. Goldberg, E. Veledar, J.D. Bremner, V. Vaccarino, Post-traumatic stress disorder and impaired autonomic modulation in male twins, Biol. Psychiatry. 73 (2013) 1103–1110, doi:10.1016/j.biopsych.2013.01.019.

[50] B.J. Stoll, N. Hansen, A.A. Fanaroff, L.L. Wright, W.A. Carlo, R.A. Ehrenkranz, J.A. Lemons, E.F. Donovan, A.R. Stark, J.E. Tyson, W. Oh, C.R. Bauer, S.B. Korones, S. Shankaran, A.R. Laptook, D.K. Stevenson, L.-.A. Papile, W.K. Poole, Late-onset sepsis in very low birth weight neonates: the experience of the NICHD neonatal research network, Pediatrics 110 (2002) 285–291.

[51] A.M. Weindling, Blood pressure monitoring in the newborn, Arch. Dis. Child. 64 (1989) 444–447.
