# Supplementary material for: Exploring Computational Techniques in Preprocessing Neonatal Physiological Signals for Detecting Adverse Outcomes: Scoping Review
Source: Interact J Med Res. 2024 Aug 20;13:e46946. doi: 10.2196/46946 (PMC11372324; doi:10.2196/46946)
Supplement: Multimedia Appendix 3 [file ijmr_v13i1e46946_app3.zip › Included Papers - Final/3674/Cohen and de Chazal - 2015 - Automated detection of sleep apnea in infants A m.pdf]

Computers in Biology and Medicine 63 (2015) 118–123

Contents lists available at ScienceDirect

Computers in Biology and Medicine

journal homepage: www.elsevier.com/locate/cbm

Automated detection of sleep apnea in infants: A multi-modal approach

Gregory Cohen [a,*], Philip de Chazal [a,b]

[a] MARCS Institute, University of Western Sydney, Australia
[b] School of Electrical and Information Engineering, University of Sydney, Australia

ARTICLE INFO

Article history:
Received 24 December 2014
Accepted 10 May 2015

Keywords:
Infant sleep apnea
Oximetry
ECG
Minimally invasive sensors
CHIME

ABSTRACT

This study explores the use and applicability of two minimally invasive sensors, electrocardiogram (ECG) and pulse oximetry, in addressing the high costs and difficulty associated with the early detection of sleep apnea hypopnea syndrome in infants. An existing dataset of 396 scored overnight polysomnography recordings were used to train and test a linear discriminants classifier. The dataset contained data from healthy infants, infants diagnosed with sleep apnea, infants with siblings who had died from sudden infant death syndrome (SIDS) and pre-term infants. Features were extracted from the ECG and pulse-oximetry data and used to train the classifier. The performance of the classifier was evaluated using a leave-one-out cross-validation scheme and an accuracy of 66.7% was achieved, with a specificity of 67.0% and a sensitivity of 58.1%. Although the performance of the system is not yet at the level required for clinical use, this work forms an important step in demonstrating the validity and potential for such low-cost and minimally invasive diagnostic systems.

© 2015 Elsevier Ltd. All rights reserved.

1. Introduction

Sleep apnea hypopnea syndrome (SAHS) is a widely underdiagnosed condition in which the cessation of breathing occurs repeatedly during sleep, leading to an oxygen desaturation and cortical arousal. Sleep apnea events are classified according to whether the patient exhibits respiratory effort. An apnea involving such effort is commonly caused by an obstruction of the upper airway and is referred to as an obstructive sleep apnea (OSA). An apnea event lacking any respiratory effort is generally the result of a neurological condition and is termed a central apnea (CSA). Apnea events can also result from a combination of these two factors and is then referred to as a mixed apnea event. Arousals from apnea events, although an involuntary protective mechanism, serve to fragment sleep and have a strong negative impact on sleep quality.

Estimations of the prevalence of SAHS are varied, but obstructive sleep apnea is estimated to affect up to 5% of the adult population in Western countries [1]. Although the majority of studies have focused on developed countries, preliminary data from developing countries shows an unexpectedly high prevalence of OSA in adults. A study carried out in Abuja, Nigeria reported that 19% of the 370 participants were classified as having a high risk of OSA against the Berlin questionnaire criteria [2]. Similar results were obtained from a smaller study carried out in the south-west of Nigeria [3].

SAHS is also prevalent in very young children and infants, although the occurrence of central apneas is quite common in both infants and young children [4] and durations up to 25 s are considered normal [5]. Obstructive apnea events, on the other hand, are extremely rare in healthy young children and recent studies have estimated that obstructive sleep apnea is estimated to affect between 1% and 4% of infants [6]. Whereas the most severe effects of SAHS in adults are linked to hypertension [7], cardiovascular disease [8] and the dangers arising from vehicular accidents due to daytime sleepiness [9], sleep-related breathing disorders in infants and young children have been linked to several negative developmental effects, such as cognitive impairment [10], depression [11], and attention deficit/hyperactivity disorder [12].

SAHS has also been tentatively linked to sudden infant death syndrome (SIDS) [13]. To exacerbate the problem, studies have shown that young children and infants tend to suffer more severe episodes of sleep apnea [14]. It is estimated that over 80% of individuals with sleep-related breathing disorders go undiagnosed [15], due partly to low public awareness of the issue, but primarily due to the limited availability and high costs of appropriate

* Corresponding author.
E-mail addresses: g.cohen@uws.edu.au (G. Cohen), philip.dechazal@sydney.edu.au (P. de Chazal).

http://dx.doi.org/10.1016/j.compbiomed.2015.05.007
0010-4825/© 2015 Elsevier Ltd. All rights reserved.

recording and diagnostic equipment. Overnight polysomnograms are considered to be the gold-standard diagnostic test for SAHS [16] but the costs involved in performing such tests, which require expensive equipment and specially trained staff, makes it prohibitively expensive for widespread diagnostic use and completely unavailable in many countries.

Whilst it is possible to perform overnight polysomnograms on infants and young children, the intrusive nature of the multitude of sensors limits the practicality and effectiveness of the technology and can lead to lower quality results in infants and young children [17]. As a result, there is a clear need for the development of a less invasive, lower cost means for the detection and diagnosis of apnea events using easily accessible and low-cost sensors.

This study explores the efficiency and potential of a multimodal approach to the automated detection of sleep apnea in infants using oximetry data from a minimally invasive finger sensor, and a single-lead ECG. Oximeter features, R–R interval features extracted from the ECG signal and the combined feature set from both sensing modalities are evaluated and compared.

1.1. *Minimally invasive sensor modalities*

A joint study group consisting of the American Thoracic Society, the American Academy of Sleep Medicine and the American College of Chest Physicians investigated the applicability, reliability and accuracy of potential portable monitoring devices for use in the detection of SAHS [18]. Three categories of devices were examined: devices with a minimum of seven channels (Type 2), devices with a minimum of four channels (Type 3) and devices with only one or two channels (Type 4). These were assessed against the current standard diagnostic test – a technician-assisted in-laboratory overnight polysomnogram (Type 1).

The findings of the study were cautious, especially in regards to Type 4 devices, but highlighted the need for continued research in the field and the further exploration of different sensing modalities. Oximetry data is one such modality and has often been studied as a potential means of detecting the desaturations caused by sleep apnea events, as cyclic desaturations are a potential indicator of SAHS. Direct measurements of oxyhemoglobin saturation (SaO_2) are difficult to obtain, but pulse oximetry data (SpO_2) provides an indirect and rapid approximation of SaO_2 saturation levels [19] and due to the low-cost and minimal invasiveness of the technology, unattended, overnight ambulatory oximetry recordings has been widely investigated. Unfortunately, the use of oximetry data is limited by its negative predictive value (NPV) [20], as not all apnea events lead to discernible desaturations [21]. To further complicate matters, the averaging time for commercial pulse oximeters can vary considerably, and it has been shown that sensitivity is lower and specificity higher when longer averaging times are used [22].

Oximetry sensors are peripherally attached to the body and are therefore subject to additional noise resulting from motion and poor perfusion, which often leads to artifacts that can render the data unusable [23]. It is also not possible to determine whether the desaturation was caused by an obstructive or central apnea based on oximetry data alone [24]. This is particularly relevant in the detection of apnea events in young children and infants. Despite the issues associated of oximetry, it has proven to hold some diagnostic value especially in health children with suspected obstructive sleep apnea [25] as it has a potentially high positive predictive value (PPV). Thus, oximetry alone can be used as a potential means of detecting sleep apnea episodes, but not as a reliable means of diagnostically excluding them.

Electrocardiogram (ECG) recordings have also been the focus of many efforts to develop alternative mechanisms for sleep apnea detection. Physiologically, many apnea events (but not all) are associated with a bradycardia and are followed by a recovery breath and an abrupt tachycardia [26], creating cyclic variations in heart rate. These patterns are even more evident in the R–R interval spectrum, and obstructive apnea events can be determined through visual inspection alone [27]. Examining the low frequency, and very low frequency variations in heart rate have also been shown to contain diagnostically relevant information for the detection of OSA [28] with CPAP treatment in OSA-sufferers being shown to reduce this effect on heart rate variability [29]. The ECG data has also been shown to be modulated by respiratory effort [30], and an ECG-derived respiration (EDR) signal can be extrapolated [31]. This respiratory signal holds significant importance as it provides a means to distinguish whether respiratory effort was present during an apnea event, allowing the differentiation between central and obstructive apneas.

Utilizing a multi-modal approach to the detection of sleep apnea, in which carefully selected features are extracted from the ECG and oximetry data and combined, should increase the separability of the classification classes by leveraging the different underlying modalities and providing a mechanism for noise reduction and removal.

1.2. *Database*

The physiological and annotation data used in this study was obtained from the National Collaborative Home Infant Monitoring Evaluation (CHIME) dataset, which was collated by the National Institute of Health (NIH), to study the effectiveness of home monitoring for apnea and bradycardia in infants, especially with reference to sudden infant death syndrome (SIDS) [32].

The database contains overnight physiological data from 1079 infants, obtained between May 1994 and February 2008 and included infants ranging in age from newborns to 27 weeks. The subjects were drawn from four groups: children suspected of suffering from apnea events, healthy term infants, pre-term infants and infants with a history of SIDS in their immediate family. A breakdown of the genders and screening conditions for the subjects used in the study is presented in Table 1.

Primarily, the data consists of overnight home recordings obtained using the CHIME home monitoring device. The device contained seven sensors, including an Aequitron ECG/Impedance device and an Ohmeda pulse-oximeter. In addition, approximately 700 of the infants in the study also underwent full in-laboratory polysomnograms. These polysomnograms were recorded using a Healthdyne ALICE3 system, consisting of 17 sensors (including ECG, EEG, pulse oximetry, respiratory effort and a nasal thermistor).

The database also contains sleep state and arousal scoring information. It is important to note that the CHIME monitor was used in conjunction with the ALICE3 equipment during the overnight polysomnograms as the technicians used the data from the CHIME monitor to do the arousal scoring. Of the 700 recordings available, only 328 subjects were found to contain complete recordings, annotations and sleep state data. Due to a number of factors such as low signal quality, damaged headers and excessively

Table 1
Gender and selection criteria breakdown.

Screening criteria	Male	Female	Total
Apnoea of infancy	42	45	87
Healthy term	44	37	81
Premature	36	30	66
Sibling of SIDS	79	81	160
**Total**	**193**	**201**	**394**

noisy signals, only 288 of the records from the CHIME database could be used to train the classification system.

2. Method

The system presented in this paper is comprised of three main components: a data extraction and pre-processing stage, a feature extraction stage and a linear discriminants classifier. The structure of the system is shown in Fig. 1.

2.1. Pre-processing

The SpO_2 and ECG signals were extracted from each polysomnogram study and time-aligned to 30-s epochs defined by the scoring of the sleep state information extracted for each patient. The sleep state data was also used to identify the first and last periods of active sleep for each subject in order to minimize the introduction of artifacts whilst the subject was awake.

The pulse oximeter used in the ALICE3 system produced a 1 Hz oxygen saturation signal representing a rolling 3 s average. As pulse-oximetry sensors are sensitive to movement and therefore prone to noise, a comprehensive artifact removal step was implemented. Oximetry values below a lower threshold of 65% saturation were discarded, along with any change in saturation exceeding 4% per second. An exclusionary window of ten samples, centered on each artifact, was applied to further suppress artifact errors.

The ECG data was extracted in the form of a single channel 100 Hz signal. A QRS detection algorithm based on the Pan and Tompkins algorithm [33] was used to generate R–R intervals. Due to noise and inaccuracies in the ECG signal and the QRS detector, an R–R interval correction step was performed in which spurious QRS detections were removed and missing QRS intervals approximated using the method outlined in [34]. The filtered R–R intervals were then time-aligned to the same 30-s epochs as used for the SpO_2 signal.

Respiratory and apnea annotations were obtained from the scored CHIME monitor recordings which were recorded alongside the polysomnogram data. These annotations were referenced against the internal clock on the CHIME monitor, and not the ALICE3 system used for the polysomnogram recordings and additional calibration data linking the two datasets had to be extracted from the CHIME database and used to correctly adjust the time reference for the annotations before aligning them to the 30-s epoch basis.

2.2. SpO_2 feature extraction

There has been extensive research done on the subject of feature generation from pulse oximetry sensors. Most of these methods employ time-based statistical properties and are affected by limitations arising from physiological effects, variations in sensor location, and sensor specificity [35]. Standard time-based features include statistical properties such as mean, median value, minimum value, threshold values and various inter-measurement interval calculations [36]. Many of these features suffer from a lack of standardized limits, which make comparisons with other results and studies difficult.

A number of methods have been developed that explore approaches that break away from the standard time-based approach. These include frequency-based features [37], non-linear features [38] and features based on multivariate regression [39]. The bulk majority of the work has been performed on adult subjects and as a result, this study limits itself to well-established, time-based features. Seven different time-based features were calculated for each epoch using both the pre-processed SpO_2 values and the associated baseline SpO_2 value. The features calculated for each epoch were as follows:

1. Mean SpO_2 value over the epoch.
2. Minimum SpO_2 value in the epoch.
3. Number of instances below 92% saturation.
4. Average absolute rate of change per second in the epoch.
5. The 3rd and 57th value in sorted SpO_2 values (corresponding to a 5–95% spread).
6. The number of times the baseline value exceeded the SpO_2 value by at least 3%.
7. The number of times the SpO_2 value exceeded the baseline SpO_2 by at least 3%.

The SpO_2 baseline captures the rolling average of the SpO_2 value during non-apnea periods. Features 6 and 7 capture the instantaneous deviations which exceed 3% from the SpO_2 baseline.

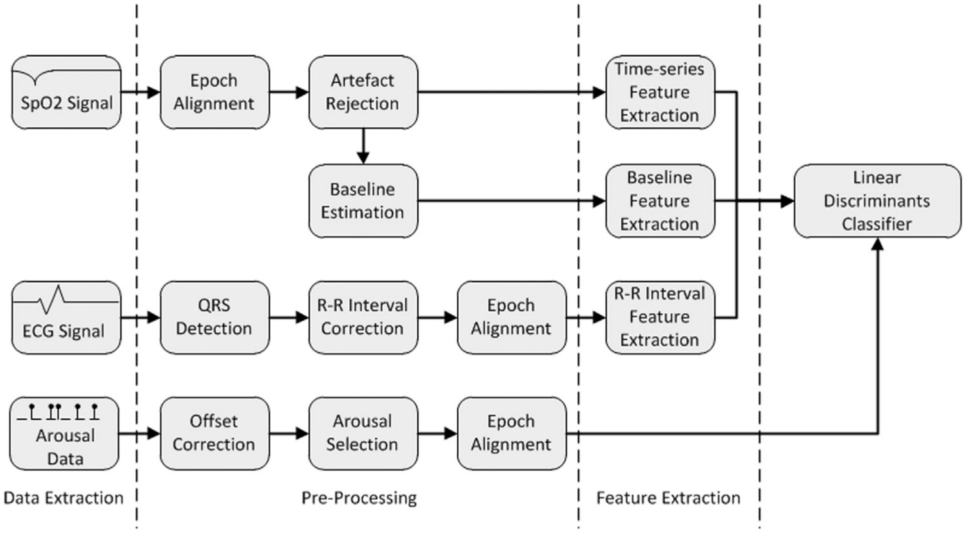

Fig. 1. Overview of the classification system.

2.3. ECG feature extraction

Four features were calculated from the R–R interval derived from the ECG signal. For each epoch, the following features were calculated:

1. The average R–R interval.
2. The standard deviation of the R–R interval.
3. The square root of the mean of the sum of the differences between adjacent R–R intervals.
4. The power spectral density (PSD) of the R–R intervals (32 features).

The PSD features calculated for each epoch were derived using the method outlined in [30]. For each epoch, the R–R interval values were normalized, padded with zeros to a length of 256 and transformed to the frequency domain by a fast Fourier Transform (FFT).

The coefficients of the magnitude of the FFT were then squared and down-sampled with an averaging filter to 64 values representing the PSD for the epoch. As the PSD is symmetrical, only the first 32 samples of the PSD are required as features.

2.4. Classifier

A supervised training technique was implemented as a means for automatic classification [40]. A linear discriminant (LD) model was chosen and was optimized using the maximum likelihood method. The linear discriminant method provides a posterior probability estimate of each class, and the class with the highest probability estimate is chosen as the final classification.

Training the LD classifier uses “plug-in” maximum likelihood estimates and proceeds as follows: let x be a column vector containing d feature values. Assume that we wish to assign x to one of the c possible classes and that a total of N feature vectors are available for training the classifier. The number of feature vectors available for training for class k is N_k and therefore

$$N = \sum_{k=1}^{c} N_k \quad (1)$$

The nth feature vector used for training in class k is designated x_{nk}. In order to train the models, the class-conditional mean vector μ_k needs to be calculated as follows:

$$u_k = \frac{1}{N} \sum_{n-1}^{N_k} x_{nk} \quad (2)$$

The common covariance matrix Σ is calculated using

$$\Sigma = \frac{1}{N-c} \sum_{k=1}^{c} \sum_{n=1}^{N_k} (x_{nk} - \mu_k)(x_{nk} - \mu_k)^T \quad (3)$$

When a feature vector x needs to be classified, values are assumed for the prior probabilities π_k, and, using this, the discriminant value y_k is calculated as follows:

$$y_k = -\tfrac{1}{2}\mu_k^T \Sigma^{-1} \mu_k + \mu_k^T \Sigma^{-1} x + \log(\pi_k) \quad (4)$$

The posterior probabilities, $P(k|x)$, are used in this study and can readily be calculated from the discriminant values as follows:

$$P(k|x) = \frac{e^{y_k}}{\sum_{i=1}^{c} e^{y_l}} \quad (5)$$

The final class is the class with the highest posterior probability or, equivalently, the highest discriminant value.

2.5. Performance measures

The linear discriminant classifier was trained to discriminate between normal and any type of sleep disordered breathing (SDB). Each epoch was either labeled Normal or SDB by the system and the corresponding expert value determined from the arousal data.

Each epoch label by the system was compared to the expert data derived from the arousals information and the outcome determined as one of the following:

- *True positive* (*TP*): an epoch is labeled as SDB by the arousal data and labeled as SDB by the system.
- *True negative* (*TN*): an epoch is labeled as Normal by the arousal data and labeled as Normal by the system.
- *False positive* (*FP*): an epoch is labeled as Normal by the arousal data and labeled as SDB by the system.
- *False negative* (*FN*): an epoch is labeled as SDB by the arousal data and labeled as Normal by the system.

The number of outcomes over all the epochs was calculated and used to form the two way confusion matrix shown in Table 2.

Using Table 2 the following performance measures were then calculated:

- Specificity=TN /(TN + FP).
- Sensitivity=TP / (TP + FN).
- Accuracy=(TN + TP) / (TP + TN + FN + FP).

A leave-one-out cross-validation scheme was used to assess the ability of the classifier in the face of independent data. In this scheme, one record is left out of the training regime and used to assess the performance of the trained classifier. The record is then cycled with another from the training corpus and the process is repeated until each record has been evaluated in this manner. The results are then combined to produce the overall results.

2.6. Implementation

The data handling, pre-processing and feature extraction methods were written in python and executed on an 80-node Rocks cluster running pyMPI. The classification and performance evaluation code was written in Matlab and run separately.

3. Results and discussion

The results for the epoch classifier using the SpO_2 features, the ECG features and the full montage of features are presented in Table 3. These results show the performance of the classifier classifier labelling epochs as either containing any type of sleep disordered breathing event or as a normal epoch.

On the basis of accuracy the best performing system was the ECG-only system as it resulted in a classification accuracy of 74.7% which was approximately 8% higher than the SpO_2 system or the combined system. An immediately identified issue with this system was the poor sensitivity (39.6%) meaning than more than 60% of apnea epochs were incorrectly labelled as normal. A

Table 2
Two-way confusion matrix.

		Actual	
		Normal	SDB
Predicted	Normal	TN	FN
	SDB	FP	TP

Table 3
Epoch-based classification results for oximetry, ECG and combined feature sets.

	**Specificity** (%)	**Sensitivity** (%)	**Accuracy** (%)
7 SpO_2 features	66.7	58.9	66.0
35 ECG features	76.4	39.6	74.7
42 SpO_2 and ECG features	67.0	58.1	66.7

possible explanation for this is that, due to the predominance of central apnea events in infants, the heart rate series may not have shown the same characteristic bradycardia/tachycardia pattern that is frequently seen in adults.

The SpO_2 system resulted in an accuracy of 66.0% with a specificity of 66.7% and a sensitivity of 58.9%. The combined system slightly outperformed the SpO_2 system with an accuracy of 66.7%, a specificity of 67.0% and a sensitivity of 58.1%. Both of these systems had much more balanced performance than the ECG only system. The principal advantage of the combined system was its robustness. In the event that the SpO_2 probe became detached the system could fall back on the ECG signal and still continue to make epoch predictions.

4. Discussion

The results of this study show that it is possible to detect apnea events using a montage of minimally invasive sensors consisting of ECG and SpO_2 devices. Despite the performance of the system falling short of those required for clinical acceptance, this work forms an important step in demonstrating the validity and potential for such low-cost and minimally invasive diagnostic systems.

It is important to note that this study included every available and valid subject from the CHIME study. Only records where there was either missing data or missing annotations were excluded from the classifier. This is particularly important as the subject pool contains a large population of healthy infants. This is important as many studies are performed in sleep clinics or laboratories, where a selection bias may exist towards patients with sleep-related breathing disorders [41]. As all available records were used, and at least 21% of the infants were healthy term, these results are free from any such confounding factors.

Studies involving Holter-oximeters for apnea detection have been reported in adults and children. In our own study [42], a Holter oximeter system was used to identify epochs of sleep disordered breathing (SDB) in 59 sequentially admitted adult patients (11 normal patients, 48 apnea patients). The resulting sensitivity and specificity were 87% and 51% respectively. Xie and Minn [43] achieved a similar sensitivity (85%) and higher specificity (80%) for identifying epochs of SDB on 25 adult patients (2 normal patients, 23 apneas patients). Jeoti [44] reported an accuracy of over 99% in detecting SDB epochs in adults. Unfortunately, no sensitivity, specificity or details on the database are provided, rendering it difficult to put this result into context.

The study by Weese-Mayer et al. [45] in infants provides a benchmark with which to interpret and compare our results. The study focused on detecting apneas lasting longer than 16 s within the CHIME database and their classifier successfully identified 120 out of 233 apnea events, yielding an accuracy of 51%. Incorporating End-Tidal CO_2 and Thermister sensors did not improve the accuracy. Our results, with an accuracy of 67.0%, demonstrate an improvement over these results.

One potential factor that may have contributed to the lower sensitivity and specificity than has been achieved in the adult population is the manner in which the apnea scoring was performed in the CHIME system. During the full polysomnogram recordings, the CHIME monitor was configured in parallel with the polysomnogram equipment, and it was the data captured by the CHIME monitor that was used for apnea scoring. It has been noted by others that the unusual and often low number of apnea events for certain subjects in the study [45], which may be an indicator that the full polysomnogram recordings, might contain apnea events that were not detected using the CHIME equipment. In this case, this would adversely affect the specificity result for our system.

There are a number of future directions which we believe will result in a performance boost to our system, Firstly, using the raw photoplethysmogram signal (PPG) that forms the oximetry signal, to extract breathing effort may lead to a way of separating the reduced breathing effort during a central apnea from the increased effort during an obstructive apnea [46]. Secondly, using the ECG and oximetry to identifying periods of sleep and wake [47] and using this information to focus the apnea detection on periods of sleep would enhance our apnea detection algorithm. Thirdly, introducing an actimetry signal would facilitate identification of sleep stages as well as identify arousals following apnea.

5. Conclusions

It is clear from the results that a multi-modal approach to the detection of sleep apnea in infants produces a superior result and addresses some of the problems encountered when only a single modality is used. The physiological differences between adults and infants may also affect the outcome of the classification technique as certain standard assumptions, such as epoch duration and threshold values, may need to be adjusted and tuned for use in infant apnea detection.

The results of this study demonstrate that there is potential for a multi-modal system consisting of ECG and pulse-oximetry to form the basis of a low-cost means of screening for sleep apnea in areas where laboratory polysomnograms are not available. Further research using data acquired with higher-resolution sensors will be instrumental in assessing the reliability and the diagnostic power of such a system.

Conflict of interest statement

None declared.

Acknowledgement

This research was supported by Australian Research Council Grant number FT110101098.

References

[1] R.J. Davies, J.R. Stradling, The epidemiology of sleep apnoea, Thorax 51 (Suppl. 2) (1996) S65–S70. http://dx.doi.org/10.1136/thx.51.Suppl_2.S65.
[2] O.O. Adewole, A. Hakeem, A. Fola, E. Anteyi, Z. Ajuwon, G. Erhabor, Obstructive sleep apnea among adults in Nigeria, J. Natl. Med. Assoc. 101 (7) (2009) 720–725.
[3] A. Akintunde, O. Okunola, R. Oluyombo, Y. Oladosu, O. Opadijo, Snoring and obstructive sleep apnoea syndrome among hypertensive Nigerians: prevalence and clinical correlates (2012), http://dx.doi.org/10.4314/pamj.v11i1.
[4] E.S. Katz, R.B. Mitchell, C.M. D'Ambrosio, Obstructive sleep apnea in infants, Am. J. Respir. Crit. Care Med. 185 (8) (2012) 805–816. http://dx.doi.org/10.1164/rccm.201108-1455CI.
[5] M.A. Carskadon, K. Harvey, W.C. Dement, C. Guilleminault, F.B. Simmons, T. F. Anders, Respiration during sleep in children, West. J. Med. 128 (6) (1978) 477–481.

[6] K. Bonuck, K. Freeman, R.D. Chervin, L. Xu, Sleep-disordered breathing in a population-based cohort: behavioral outcomes at 4 and 7 years, Pediatrics 129 (4) (2012) e857–65. http://dx.doi.org/10.1542/peds.2011-1402.
[7] W. Pankow, A. Lies, F.W. Lohmann, Sleep-disordered breathing and hypertension, N. Engl. J. Med. 343 (13) (2000) 966, author reply 967, http://dx.doi.org/10.1056/NEJM200009283431310.
[8] J. Rich, A. Raviv, N. Raviv, S.E. Brietzke, All-cause mortality and obstructive sleep apnea severity revisited, Otolaryngol.-Head and Neck Surg.: Off. J. Am. Acad. Otolaryngol.-Head and Neck Surg. 147 (3) (2012) 583–587. http://dx.doi.org/10.1177/0194599812450256.
[9] K.P. Strohl, D.B. Brown, N. Collop, C. George, R. Grunstein, F. Han, L. Kline, A. Malhotra, A. Pack, B. Phillips, D. Rodenstein, R. Schwab, T. Weaver, K. Wilson, An official American thoracic society clinical practice guideline: sleep, apnea sleepiness, and driving risk in noncommercial drivers. An update of a 1994 statement, Am. J. Respir. Crit. Care Med. 187 (11) (2013) 1259–1266. http://dx.doi.org/10.1164/rccm.201304-0726ST.
[10] D. Gozal, Sleep-disordered breathing and school performance in children, Pediatrics 102 (3 Pt 1) (1998) 616–620. http://dx.doi.org/10.1542/peds.102.3.616.
[11] C. VM, V. JW, G. D, Health-related quality of life and depressive symptoms in children with suspected sleep-disordered breathing, Sleep 27 (6) (2004) 1131–1138.
[12] L.M. O'Brien, C.R. Holbrook, C.B. Mervis, C.J. Klaus, J.L. Bruner, T.J. Raffield, J. Rutherford, R.C. Mehl, M. Wang, A. Tuell, B.C. Hume, D. Gozal, Sleep and neurobehavioral characteristics of 5- to 7-year-old children with parentally reported symptoms of attention-deficit/hyperactivity disorder, Pediatrics 111 (3) (2003) 554–563. http://dx.doi.org/10.1542/peds.111.3.554.
[13] F. McNamara, C.E. Sullivan, Obstructive sleep apnea in infants: relation to family history of sudden infant death syndrome, apparent life-threatening events, and obstructive sleep apnea, J. Pediatr. 136 (3) (2000) 318–323.
[14] R.T. Brouillette, S.K. Fernbach, C.E. Hunt, Obstructive sleep apnea in infants and children, J. Pediatr. 100 (1) (1982) 31–40.
[15] V. Kapur, D.K. Blough, R.E. Sandblom, R. Hert, J.B. de Maine, S.D. Sullivan, B.M. Psaty, The medical cost of undiagnosed sleep apnea, Sleep 22 (6) (1999) 749–755.
[16] C.A. Kushida, M.R. Littner, T. Morgenthaler, C.A. Alessi, D. Bailey, J. Coleman, L. Friedman, M. Hirshkowitz, S. Kapen, M. Kramer, T. Lee-Chiong, D.L. Loube, J. Owens, J.P. Pancer, M. Wise, Practice parameters for the indications for polysomnography and related procedures: an update for 2005, Sleep 28 (4) (2005) 499–521.
[17] C.L. Marcus, Sleep-disordered breathing in children, Am. J. Respir. Crit. Care Med. 164 (1) (2001) 16–30. http://dx.doi.org/10.1164/ajrccm.164.1.2008171.
[18] A.L. Chesson, R.B. Berry, A. Pack, Practice parameters for the use of portable monitoring devices in the investigation of suspected obstructive sleep apnea in adults, Sleep 26 (7) (2003) 907–913.
[19] D.S. Morillo, N. Gross, Probabilistic neural network approach for the detection of SAHS from overnight pulse oximetry, Med. Biol. Eng. Comput. 51 (3) (2013) 305–315. http://dx.doi.org/10.1007/s11517-012-0995-4.
[20] E. Chiner, J. Signes-Costa, J.M. Arriero, J. Marco, I. Fuentes, A. Sergado, Nocturnal oximetry for the diagnosis of the sleep apnoea hypopnoea syndrome: a method to reduce the number of polysomnographies, Thorax 54 (11) (1999) 968–971. http://dx.doi.org/10.1136/thx.54.11.968.
[21] F. Series, Interpretation of home oximetry tracings, CHEST J. 121 (3) (2002) 1006. http://dx.doi.org/10.1378/chest.121.3.1006-a.
[22] D.G. Davila, Oximeter performance, CHEST J. 122 (5) (2002) 1654. http://dx.doi.org/10.1378/chest.122.5.1654.
[23] Y. Yamashiro, M.H. Kryger, Nocturnal oximetry: is it a screening tool for sleep disorders, Sleep 18 (3) (1995) 167–171.
[24] F. Sériès, R.J. Kimoff, D. Morrison, M.H. Leblanc, M. Smilovitch, J. Howlett, A.G. Logan, J.S. Floras, T.D. Bradley, Prospective evaluation of nocturnal oximetry for detection of sleep-related breathing disturbances in patients with chronic heart failure, CHEST J. 127 (5) (2005) 1507–1514. http://dx.doi.org/10.1378/chest.127.5.1507.
[25] R.T. Brouillette, A. Morielli, A. Leimanis, K.A. Waters, R. Luciano, F.M. Ducharme, Nocturnal pulse oximetry as an abbreviated testing modality for pediatric obstructive sleep apnea, Pediatrics 105 (2) (2000) 405–412. http://dx.doi.org/10.1542/peds.105.2.405.
[26] C. Guilleminault, R. Winkle, S. Connolly, K. Melvin, A Tilkian, Cyclic variation of the heart rate in sleep apnoea syndrome, Lancet 323 (8369) (1984) 126–131.
[27] P.K. Stein, S.P. Duntley, P.P. Domitrovich, P. Nishith, R.M. Carney, A simple method to identify sleep apnea using Holter recordings, J. Cardiovasc. Electrophysiol. 14 (5) (2003) 467–473. http://dx.doi.org/10.1046/j.1540-8167.2003.02441.x.
[28] T. Shiomi, C. Guilleminault, R. Sasanabe, I. Hirota, M. Maekawa, T. Kobayashi, Augmented very low frequency component of heart rate variability during obstructive sleep apnea, Sleep 19 (1996) 370–377.
[29] F. Roche, J.-M. Gaspoz, I. Court-Fortune, P. Minini, V. Pichot, D. Duverney, F. Costes, J.-R. Lacour, J.-C. Barthelemy, Screening of obstructive sleep apnea syndrome by heart rate variability analysis, Circulation 100 (13) (1999) 1411–1415. http://dx.doi.org/10.1161/01.CIR.100.13.1411.
[30] R.B. Shouldice, L.M. O'Brien, C. O'Brien, P. de Chazal, D. Gozal, C. Heneghan, Detection of obstructive sleep apnea in pediatric subjects using surface lead electrocardiogram features, Sleep 27 (4) (2004) 784–792.
[31] G.B. Moody, R.G. Mark, M.A. Bump, J.S. Weinstein, A.D. Berman, J.E. Mietus, A. L. Goldberger, Clinical validation of the ECG-derived respiration (EDR) technique, Comput. Cardiol. 13 (1986) 507–510.
[32] D.H. Crowell, L.J. Brooks, T. Colton, M.J. Corwin, T.T. Hoppenbrouwers, C. E. Hunt, L.E. Kapuniai, G. Lister, M.R. Neuman, M. Peucker, S.L. Ward, D. E. Weese-Mayer, M. Willinger, Infant polysomnography: reliability. Collaborative home infant monitoring evaluation (CHIME) steering committee, Sleep 20 (7) (1997) 553–560.
[33] J. Pan, W.J. Tompkins, A real-time QRS detection algorithm, IEEE Trans. Bio-Med. Eng. 32 (3) (1985) 230–236. http://dx.doi.org/10.1109/TBME.1985.325532.
[34] P. de Chazal, C. Heneghan, E. Sheridan, R. Reilly, P. Nolan, M. O'Malley, Automated processing of the single-lead electrocardiogram for the detection of obstructive sleep apnoea, IEEE Trans. Bio-Med. Eng. 50 (6) (2003) 686–696. http://dx.doi.org/10.1109/TBME.2003.812203.
[35] D.S. Morillo, N. Gross, A. León, L.F. Crespo, Automated frequency domain analysis of oxygen saturation as a screening tool for SAHS, Med. Eng. Phys. 34 (7) (2012) 946–953.
[36] B. Herer, Value of clinical, functional, and oximetric data for the prediction of obstructive sleep apnea in obese patients, Chest 116 (6) (1999) 1537–1544. http://dx.doi.org/10.1378/chest.116.6.1537.
[37] C. Zamarrón, P.V. Romero, J.R. Rodriguez, F. Gude, Oximetry spectral analysis in the diagnosis of obstructive sleep apnoea, Clin. Sci. 97 (4) (1999) 467–473. http://dx.doi.org/10.1378/chest.121.3.1006-a.
[38] D.S. Morillo, J.L. Rojas, L.F. Crespo, A. León, N. Gross, Poincaré analysis of an overnight arterial oxygen saturation signal applied to the diagnosis of sleep apnea hypopnea syndrome, Physiol. Meas. 30 (4) (2009) 405–420. http://dx.doi.org/10.1088/0967-3334/30/4/005.
[39] U.J. Magalang, Prediction of the apnea–hypopnea index from overnight pulse oximetry, Chest 124 (5) (2003) 1694–1701. http://dx.doi.org/10.1378/chest.124.5.1694.
[40] B.D. Ripley, Pattern Recognition and Neural Networks, Cambridge University Press, Cambridge, UK, 1996.
[41] V. Certal, M. Camacho, J.a.C. Winck, R. Capasso, I. Azevedo, A. Costa-Pereira, Unattended sleep studies in pediatric OSA: a systematic review and meta-analysis, Laryngoscope 125 (January 2015) 255–262. http://dx.doi.org/10.1002/lary.24662.
[42] C. Heneghan, C.-P. Chua, J.F. Garvey, P. de Chazal, R. Shouldice, P. Boyle, W. T. McNicholas, A portable automated assessment tool for sleep apnea using a combined Holter-oximeter, Sleep 31 (10) (2008) 1432–1439.
[43] B. Xie, H. Minn, Real-time sleep apnea detection by classifier combination, IEEE Trans. Inf. Technol. Biomed.: Publ. IEEE Eng. Med. Biol. Soc. 16 (3) (2012) 469–477. http://dx.doi.org/10.1109/TITB.2012.2188299.
[44] V. Jeoti, ECG and blood oxygen level based sleep apnea study and detection, in: 2010 IEEE EMBS Conference on Biomedical Engineering and Sciences (IECBES), Kuala Lumpur, IEEE, 2010, pp. 285–290, http://dx.doi.org/10.1109/IECBES.2010.5742245.
[45] D.E. Weese-Mayer, M.J. Corwin, M.R. Peucker, J.M. di Fiore, D.R. Hufford, L. R. Tinsley, M.R. Neuman, R.J. Martin, L.J. Brooks, S.L.D. Ward, G. Lister, M. Willinger, T.M. Baird, L.J. Brooks, R. O'Bell, C.E. Hunt, D.R. Hufford, M. a. Oess, J.M. Silvestri, S.M. Smok-Pearsall, D.H. Crowell, L.E. Kapuniai, T. T. Hoppenbrouwers, R. Ramanathan, P. Palmer, T.G. Keens, D.B. Bolduc, R. S. Mendenhall, J.T. Zoldak, J.M. di Fiore, T. Colton, S.M. Bak, H. Golub, S. C. Schafer, Comparison of apnea identified by respiratory inductance plethysmography with that detected by end-tidal CO_2 or thermistor, Am. J. Respir. Crit. Care Med. 162 (2 I) (2000) 471–480. http://dx.doi.org/10.1164/ajrccm.162.2.9904029.
[46] E. Gil, M. Mendez, J.M. Vergara, S. Cerutti, A.M. Bianchi, P. Laguna, Discrimination of sleep-apnea-related decreases in the amplitude fluctuations of ppg signal in children by HRV analysis, IEEE Trans. Biomed. Eng. 56 (4) (2009) 1005–1014. http://dx.doi.org/10.1109/TBME.2008.2009340.
[47] G. Cohen, P. de Chazal, A Multi-modal Approach to Sleep-Wake Classification in Infants using Minimally Invasive Sensors, Computers in Cardiology (2014) 149–152.

Gregory Cohen received a BSc (Eng) Electrical and Computer engineering, an MSc (Eng) and a BCom (Hons) in Finance and Portfolio Management from the University of Cape Town, Cape Town, South Africa in 2007, 2008, 2010 respectively.

He worked as a consulting engineer in the field of large-scale HVAC from 2007 to 2009, as a design engineer for an online diagnostics and monitoring company from 2009 to 2011, as an expert consultant for Kaiser Economic Development Practice in 2012 and is currently a PhD student in the Bioelectronics and Neuroscience Group in the MARCS Institute at the University of Western Sydney. His interests include biomedical data analysis, neuroscience and applying neuromorphic principles to control and data analysis problems.

He is a student member of the IEEE, a member of the IEEE Computer Society, a student member of ASHRAE. He received the Bioelectronics and Neuroscience Postgraduate Research Award from the University of Western Sydney in 2012.

Philip de Chazal received the B.E. degree in electronic engineering, and the M. Biomed.E and Ph.D. degrees in biomedical engineering from University of New South Wales, Sydney, Australia in 1989, 1995 and 1999 respectively. He is currently the ResMed Professor Of Biomedical Engineering at University of Sydney. He was a Research Professor at Uinversity of Western Sydney from 2011-2014. He was cofounder, director and Chief Technical Officer of BiancaMed, Dublin from 2003 to 2011. He has authored over 100 articles and 10 inventions. His research interests include signal processing and pattern recognition for biomedical applications.
