# Supplementary material for: Exploring Computational Techniques in Preprocessing Neonatal Physiological Signals for Detecting Adverse Outcomes: Scoping Review
Source: Interact J Med Res. 2024 Aug 20;13:e46946. doi: 10.2196/46946 (PMC11372324; doi:10.2196/46946)
Supplement: Multimedia Appendix 3 [file ijmr_v13i1e46946_app3.zip › Included Papers - Final/3696/Navarro et al. - 2015 - Artifact rejection and cycle detection in immature.pdf]

Biomedical Signal Processing and Control 16 (2015) 9–16

Contents lists available at ScienceDirect

Biomedical Signal Processing and Control

journal homepage: www.elsevier.com/locate/bspc

Artifact rejection and cycle detection in immature breathing: Application to the early detection of neonatal sepsis

X. Navarro [a,b,*], F. Porée [a,b], A. Beuchée [a,b,c], G. Carrault [a,b]

[a] INSERM, U1099, Rennes, F-3500, France
[b] Université de Rennes 1, LTSI, Rennes, F-3500, France
[c] CHU Rennes, Pôle Médico-Chirurgical de Pédiatrie et de Génétique Clinique, Rennes, F-3500, France

ARTICLE INFO

Article history:
Received 3 April 2014
Received in revised form 18 July 2014
Accepted 16 October 2014
Available online 8 November 2014

Keywords:
Respiratory variability signal
Premature infant
Artifact removal
Automated detection
Logistic regression

ABSTRACT

This paper proposes a new framework to obtain quality respiratory variability signals from the raw breathing recorded in neonatal intensive care units (NICUs). It combines three consecutive blocks: an automatic rejection of artifacts, implemented by a logistic regression classifier, a two-step filtering process, and the identification of respiratory cycles, implemented by a peak detection algorithm. By means of a gold standard built from a preterm infants database, the performances of the first and third blocks have been evaluated. While the former obtains a 86% of specificity and sensitivity, the latter attains a respective 97%. The interest of our proposal in the clinical domain is illustrated by a promising application to detect promptly and non-invasively the presence of neonatal sepsis in the NICU.

© 2014 Elsevier Ltd. All rights reserved.

1. Introduction

Preterm infants – born before 37 weeks of gestation – exhibit a very unstable breathing, typified by apneas or pauses in ventilation that may be accompanied by bradycardia, a decrease of the heart beat rhythm [1,2]. This phenomenon, known as apnea of prematurity (AOP), is a consequence of the still underdeveloped brain and lungs, and is inversely related to the gestational age at birth [3,4]. AOP may appear spontaneously, but it can also be provoked or become more severe when other pathologies – specially sepsis, i.e. a generalized infection – are present [5]. Regardless of their origin, sighs and respiratory pauses are the mechanism responsible for the variable manner in which the infants breathe during sleep. Typically, three different patterns can be identified: (1) Regular: quiet breathing with low variability in both amplitude and frequency. (2) Erratic: irregular breathing with high variability in both amplitude and frequency including several episodes of AOP. (3) Periodic: the alternation of pauses lasting a few seconds followed by several rapid and shallow breaths [6].

The continuous monitoring of breathing and cardiac frequencies are of crucial importance to an early intervention and avoid or palliate the associated risks with recurrent apnea-bradycardia [7,8]. A large effort has also been done to predict bradycardia [9] and to detect sepsis from the analysis of heart rate series [10,11], but the respiratory signal has retained less attention. In the present work, breathing signals acquired in neonatal intensive care units (NICUs) are properly processed so that they can be further analyzed to add more insights about the pathological state of the premature infant. As raw signals, provided by abdominal strain gauges, are uncalibrated and cannot be used to study the air flow, they are converted to respiratory variability series (RVS). This data describes the respiratory rhythm by simply sequencing the time duration of breaths and holds interesting properties, such as long-range dependence [12].

This paper is organized as follows: Section 2 presents the database, composed by the breathing traces and clinicians' manual marks (or 'old standard') and the framework to obtain clean signals. Section 3 describes the evaluation methodology as well as the performance of the detection methods. In Section 4, a demonstrative example to support the interest of the here-proposed framework is reported. Finally, a conclusion is drawn in the last section.

2. Materials and methods

2.1. Data selection

The breathing signals employed in this work have been selected from a larger database, collected at the University Hospital of

* Corresponding author: LTSI, Campus de Beaulieu, Université de Rennes 1, 263 Avenue du Général Leclerc – CS 74205 – 35042 Rennes Cedex, France.

http://dx.doi.org/10.1016/j.bspc.2014.10.007
1746-8094/© 2014 Elsevier Ltd. All rights reserved.

Table 1
Description of the testing cohort (mean ± std. dev.). There are no significant differences between the age and weight of infants and duration of records.

	Sepsis	*No-sepsis*
Infants	16	16
PMA (weeks)	30.5 ± 1.73	30.4 ± 1.64
Postnatal age (days)	15.6 ± 12.2	15.8 ± 10.7
Weight (kg)	1.11 ± 0.27	1.12 ± 0.23
Recording time (h)	2.14 ± 1.07	2.61 ± 0.64

Rennes (France), already involved in previous studies [11]. Therefore, the ensemble of the 51 preterm infants served to derive two cohorts with different purposes. The first one has been employed to examine the performances of the artifact rejection and cycle detection (validation cohort), hence it needed to be labeled manually by an experienced clinician in order to establish the references to the automated processes. The remaining group (testing cohort) provides data to illustrate the clinical example.

Breathing was recorded by abdominal strain gauges (Pneumotrace©, Morro Bay, USA), piezoelectric transducers responding linearly to changes in the circumference during respiration. Signals, originally sampled at $Fs = 400$ Hz were subsequently down-sampled to 64 Hz (Fr) after eliminating the frequency content above $Fr/2$ by a low-pass filter (7th order Butterworth) to avoid aliasing.

2.1.1. Validation cohort

This group is constituted by five preterm infants 31.0 ± 1.6 weeks post-menstrual age (PMA) and 1.06 ± 0.29 kg of weight. The selection was performed visually by clinicians to ensure the inclusion of different breathing patterns. A Matlab program was purposely designed to facilitate the labeling procedure to the clinician. It consisted on marking intervals of 10 s (W = 10 s) as clean (class 0) or artifacted (class 1) in a 30-s sliding window. The ECG signal was also displayed to help the observer to make the decision. With this program, a total of 5167 marks (14.35 h) were obtained, the 11.8% of them classified as artifacts.

In a second instance, artifact-free marked periods were used to build the references for the automatic detection of breathing cycles. Thirty minutes of clean breathing were randomly selected per each infant and displayed by another custom-made Matlab tool, that allowed the clinician to visually annotate inspiration and exhalation time intervals. This procedure provided 7234 correctly identified cycles, equivalent to almost 2h30 of breathing.

2.1.2. Testing cohort

The second dataset is composed by a selection of sixteen infected (*Sepsis*) and sixteen non-infected infants (*No-sepsis*) paired by age, gender and weight criteria (see Table 1). The diagnostic of sepsis included the combination of an inflammatory response, i.e. C-reactive protein (CRP) > 5 mg/l 24 h after the recording and positive blood cultures. In non-infected infants, no inflammatory response was observed, i.e. a CRP < 5 mg/l 24 h after the recording and resulted in negative blood cultures.

Given the well-known dependence on maturation, comparing age-equivalent sick and healthy infants is mandatory in the investigation of septicemic processes [13].

2.2. Methods

The processing framework to obtain RVS is composed by three blocks (see Fig. 1): (i) rejection of artifacted epochs involving gross body movements in raw signals by an automatic classifier based on logistic regression, (ii) two-step filtering process, including band-pass and smoothing filters and (iii) detection of the breath intervals in the clean data.

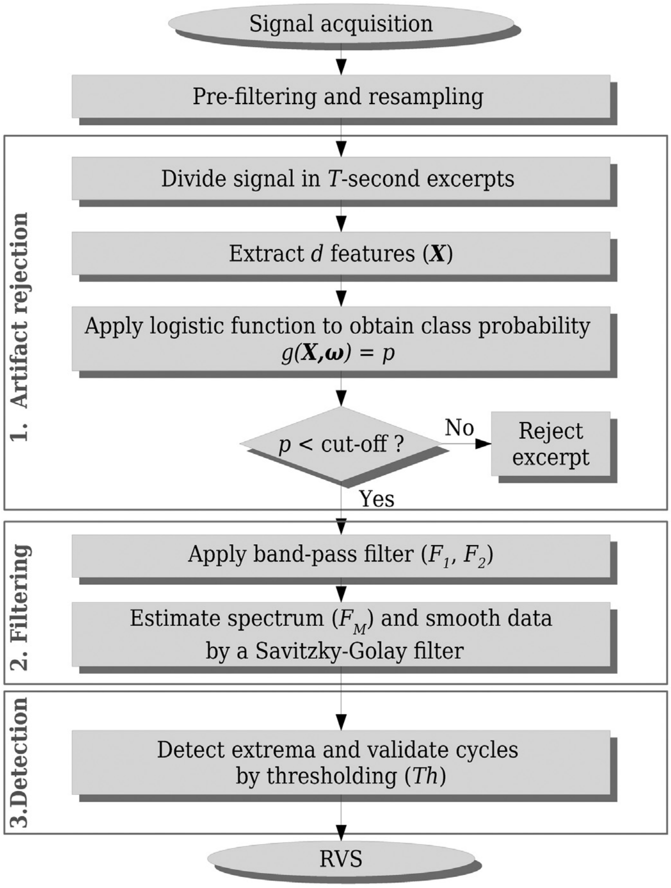

Fig. 1. Flowchart of the proposed method to obtain clean respiratory variability series, identifying the three main blocks. (1) Artifact rejection: After segmenting the pre-processed raw signals, a set of d features (**X**) are obtained. A logistic function is then computed using **X** and a vector of regression coefficients ($\boldsymbol{w}$), learned from the gold standard. The class probability p serves to reject the breathing excerpt if this exceeds the cut-off value, chosen according to a sensitivity/specificity pair. (2) Filtering: Artifact-free data is next filtered by a band-pass filtered and its power spectrum is estimated to find the main frequency, (F_m), necessary to find the parameters of the smoothing (Savitzky-Golay) filter. (3) Cycle detection: A peak detector governed by a threshold Th finds minima and maxima in the trace, i.e. the time instants of breaths that determine the RVS.

2.2.1. Artifact rejection

The study of the statistical distribution of the energy or root-mean square (RMS) in breathing signals is a common artifact detection criterion because in general, gross body movements induce higher amplitudes on the strain gauges. For instance, Motto et al. [14] applied this feature in breathing traces (both abdominal and thoracic) from 45 weeks PMA full term infants, employing a thresholding detector optimized by a Neyman–Pearson approach [15] that attained 89% for sensitivity and 88% for specificity. However, an energy-based threshold could be in some cases too restrictive due to the effect of deep breaths and impedance changes in the amplitudes.

On the other hand, an artifacted component could account for an unexpected transient event or for a background activity, like muscle activity or noise. Thus, in view of the noisy environments our breathing signals come from, an alternative criterion to detect the artifacts could be to measure the randomness of the traces by means of the entropy, as Mammone et al. [16] did in EEG signals by means of ICA and Renyi's entropy. Nevertheless, the erratic breathing patterns typical in preterm infants (see Fig. 2) could be an inconvenient in entropy measures and lead the classifier to false positive detections.

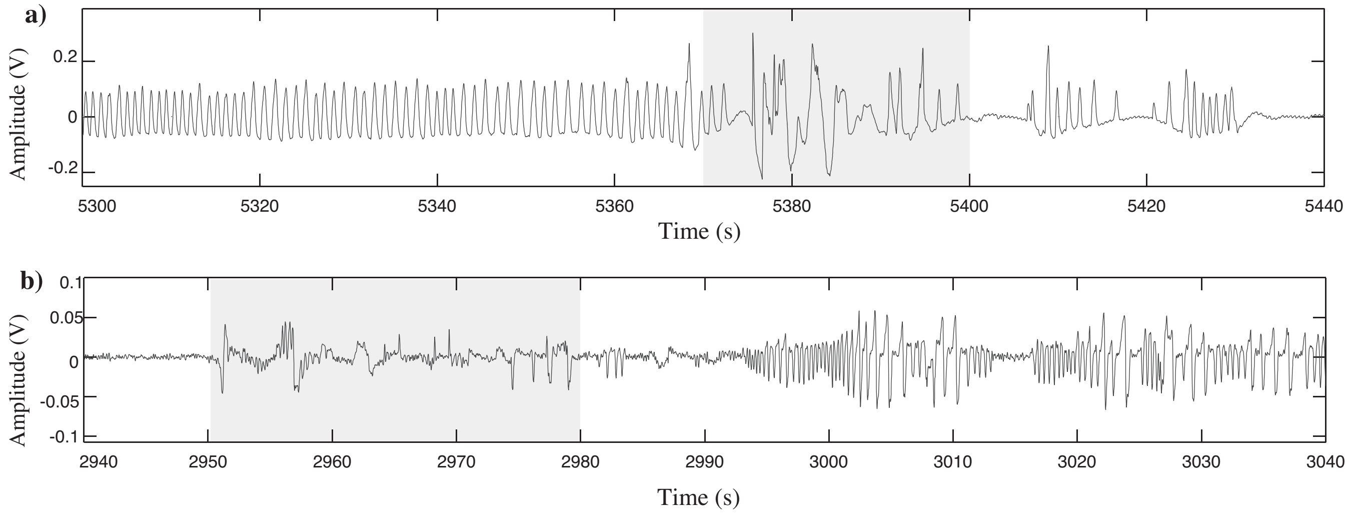

Fig. 2. Unprocessed respiratory signals with artifacts (shadow zones) marked by a clinician. (a) Example of a regular pattern turning into periodic after the artifacts. (b) Example of a heavily contaminated signal exhibiting erratic pattern. In view of the similarities between artifacts in regular patterns and erratic patterns, the main challenge of our detector is to discriminate correctly the artifacts.

Therefore, a binary multi-variable model dealing with several breathing features is necessary. Among the numerous existing solutions, such as support vector machines or neural networks, we propose to employ a classifier based on logistic regression (LR) to discriminate between clean and noisy excerpts. LR is a straightforward solution (see Appendix A) providing good results in many biomedical data classification tasks [17].

From the raw breathing signal, LR exploits a set of $d = 14$ features $\mathbf{X} \in \mathbb{R}^d$, based both in time and frequency domains. These features are computed on an excerpt of duration T, that we subsequently call *resp* and correspond to:

- Time-domain: The absolute difference between the maximum and minimum value (*Mm*), the mean of *resp* divided by its maximum value ($\overline{resp}/M$), the standard deviation (*Sd*), the kurtosis (*Kt*), the maximum value of the first derivate of the excerpt (*D*1), its kurtosis (*KD*1) and the root mean squared value (*RMS*).
- Frequency-domain: the kurtosis of the power spectral density (*KPSD*) and the power computed in four different frequency bandwidths P_{B0}, P_{B1}, P_{B2} and P_{B3}. We also computed the main frequency (*Mf*) over a period T as:

$$Mf = \frac{1}{T}\sum_{k=1}^{T} |resp(k) - resp(k-1)|. \tag{1}$$

- Entropy: The Shannon entropy (*SEnt*) in each excerpt computed as:

$$SEnt = -\sum_{k=1}^{T} resp^2 \log(resp^2). \tag{2}$$

Other features were included to the model in a preliminary test (second derivative properties, number of zero-crossing, different frequency bands, etc.) but they did not improve significantly the model fit.

2.2.2. Band-pass and smoothing filters

As introduced before, respiratory signals in preterm infants breathing spontaneously have several peculiarities. Moreover, the notable influence of the technical artifacts present in the NICU, changes in body position and physiological events as apnea, sighs and swallows, increase the difficulty of determining appropriate filters and performing effective breath detections.

Hence, after rejecting the artifacts due to gross movements, the next step is to remove the baseline and the high frequency noise. This can be performed by a simple band-pass filter, a fourth-order Butterworth whose lower (F_1) and higher (F_2) cut-off frequencies were set, respectively, to 0.5 and 20 Hz. The latter was chosen to be high enough to preserve the shape of the breathing trace but not insomuch to avoid the presence of high frequency noise.

The second filter is used to smooth without distortion the signal so that cycle detection can be performed effectively. Smoothing is necessary to reduce small ripple due, for instance, to cardiac artifacts and other noise unfiltered in the previous step. This can be achieved by Savitzky–Golay (SG) filters [18], specially recommended because they preserve the width and height of peaks of the original signal, which are usually flattened by classical moving average or FIR filters.

2.2.3. Respiratory cycle detection

Once the breathing signal has been cleaned and smoothed, the respiratory cycles (breaths) are recognized by an automatic detector. The duration of each breath is then expressed as an univariate, cycle dependent signal, i.e. the respiratory variability series.

The proposed breath-recognition algorithm takes advantage of the sinusoidal shape of the ventilatory profile to detect the beginning and end of individual breaths. Since a minimum corresponds to the minimal wall distention, it is directly related to the end of exhalation. Then, it is considered as the start-point of a respiratory cycle. Likewise, a maximum in the respiratory trace is reached when the lungs contain the tidal volume, so the time elapsed between minima and maxima defines the inspiration time (t_i), and conversely, the time between maxima and minima yields the exhalation time, t_e (see Fig. 3).

Detection algorithms exploiting large slopes or high frequent content in signals (for example, the QRS complex in ECG) employ transformations such as the signal derivative or the dyadic wavelet transform, producing a feature in which peaks can be easily detected by thresholding [19,20]. However, breathing signals are sinusoidal-like, non-impulsive signals where most of the spectral power is located in a lower frequency range, typically from 0.5 to 2 Hz in newborns. Consequently, a simple extrema detector seems to be a better solution that the mentioned strategies.

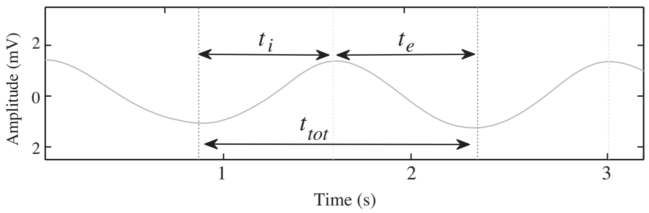

Fig. 3. Illustration of t_i, t_e and t_{tot} times in a respiratory trace. Vertical lines constitute detected minima and maxima.

Thus, the proposed detection algorithm finds all extrema, that will be validated only if the relative difference between surrounding peaks exceeds a predefined threshold, *th*. The pseudo-code is described in the following lines:

```
Inputs
x  := Breathing signal
th := Detection threshold
Outputs
m := Detected minima
M := Detected maxima
Begin
   i_M := Find the indexes of x such that:
    x[i-1] <= x[i] >= x[i+1]
   M := Find x[i_M] such that x[i_M]-th > 0
   i_m := Find the indexes of x such that:
    x[i-1] >= x[i] <= x[i+1]
   m := Find x[i_m] such that x[i_m]+th > 0
End.
```

The detection threshold *th* is obtained by the product of a descriptive statistic of the respiratory signal and a constant κ. The employed statistic was the inter-quartile range (IQR) of the breathing amplitudes because it constitutes a measure of dispersion excluding the 25% of extrema, so deep and shallow breaths are not taken into account in the detection.

3. Results

In this section, the artifact rejection and cycle detection are validated employing the two gold standards (5161 10-s excerpts classified as clean/artifacted and 7234 correctly identified cycles) introduced in Section 2.1.1. Their performances, as well as the parameter tuning of the filtering module, are also described.

3.1. Automatic artifact rejection

Before computing the LR model, we first identified experimentally the bandwidths B_0, B_1, B_2 and B_3 where the powers P_{B0}, P_{B1}, P_{B2} and P_{B3} were maximum. The following frequency intervals were found: $B_0 = [0,\ 0.25]$, $B_1 = [0.25,\ 0.7]$, $B_2 = [0.7,\ 1.2]$ and $B_3 = [1.2, 2]$ Hz. Next, employing the 14 features – computed in sliding excerpts of $T = 10$ s – on the five infants, a logistic function was found by a maximum likelihood estimate (MLE) using the *glmfit* function in the Statistical Toolbox of Matlab. Finally, a Wald test (see Appendix A) was performed to retain the most contributive features. This resulted in reducing the initial 14 variables to seven (detailed in Table 2). The remaining features did not contribute significantly to the model since they failed to reject the null hypothesis ($p > 0.05$).

To see the efficacy of the artifact classifiers, receiver operating characteristic (ROC) curves (see Fig. 4a) are plotted using leave-one out cross-validation (LOOCV). In Fig. 4b) sensibilities, sensibilities and areas under curve are obtained using a logit cut-off value of 0.5, but to find a cost-effective point, we represent the mean of both measures as a function of c (Fig. 4c) and select the intersection point ($c = 0.165$), that corresponds to a 86% sensitivity (*Sn*) and 86% specificity (*Sp*).

The less optimal values obtained with this detector ($Sn = 86\% - Sp = 86\,\%$ against $Sn = 89\% - Sp = 89\,\%$ in full-terms [14]) are probably due to the complexity of the classification problem in more immature breathing patterns. Indeed, the less performing ROC curve corresponds to a patient having patterns predominantly erratic (a sample can be seen in Fig. 2b) whereas the best one contains more regular patterns.

3.2. Band-pass and smoothing filter

The resulting signal, ignoring the artifact period, is then filtered by the band-pass and Savitzky–Golay filters. It is also used to estimate the main frequency F_M in the power spectrum. This information, necessary to tune the second filter, was estimated by means of a 30th order Burg autoregressive model.

In SG filters, data is smoothed by applying local least-squares polynomial approximation. Applied to oversampled signals (all signals in the NICU are sampled at 400 Hz whereas respiratory oscillations are mainly about 1 Hz) corrupted by noise, SG filters match appropriately the original waveform with fitted polynomial slopes reducing high-frequency noise [21].

Two parameters need to be chosen to apply this filter: the polynomial order (n) and the length of frame (m). To perform an optimal

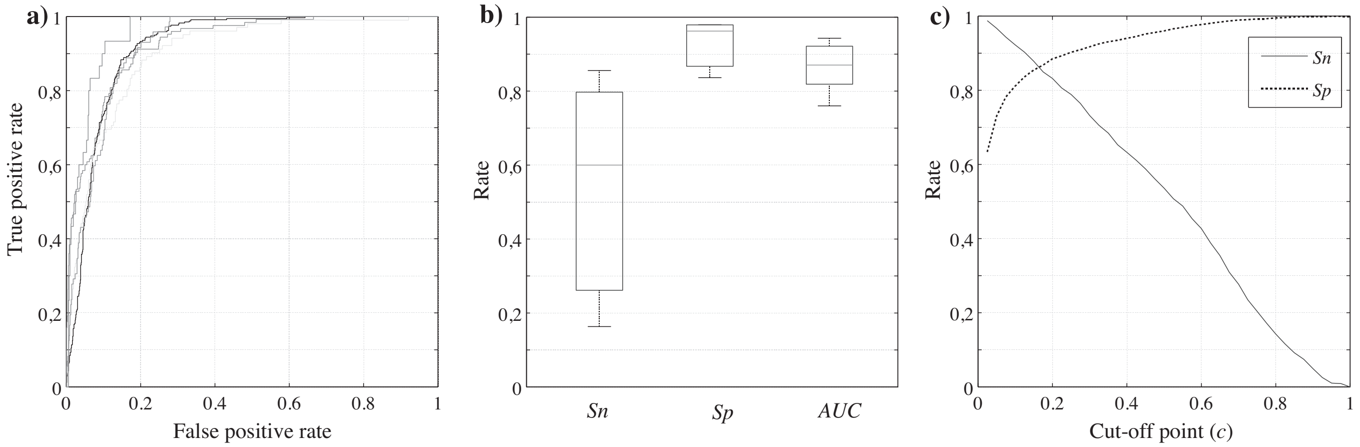

Fig. 4. (a) ROC curves from LOOCV. (b) Sensitivity (*Sn*) and specificity (*Sp*) based on the default cut-off value ($c = 0.5$). Areas under curve (*AUC*) from ROC curves are also shown as a measure of performance. (c) Mean of the sensitivity/specificity pair at several cut-off values.

Table 2
Results from fitting the LR model to the artifact detector. The estimated coefficients ($\widehat{w}$) corresponding to the seven selected features plus the intercept, and the standard errors (*SE*) of the MLE are detailed. We also provide the *z*-statistics and *p*-values of the Wald test.

Dep var →	Intercept	*Mm*	*RMS*	*MF*	*KPSD*	P_{B0}	P_{B3}	*SEnt*
$\widehat{w}$	1.865	−0.743	0.152	−0.009	0.968	0.030	−5.863	−7.176
SE	0.579	0.144	0.069	0.001	0.262	0.008	0.652	0.762
z	3.220	5.184	2.192	5.856	3.687	3.683	8.987	9.407
p	<0.01	<0.001	0.028	<0.001	<0.001	<0.001	<0.001	<0.001

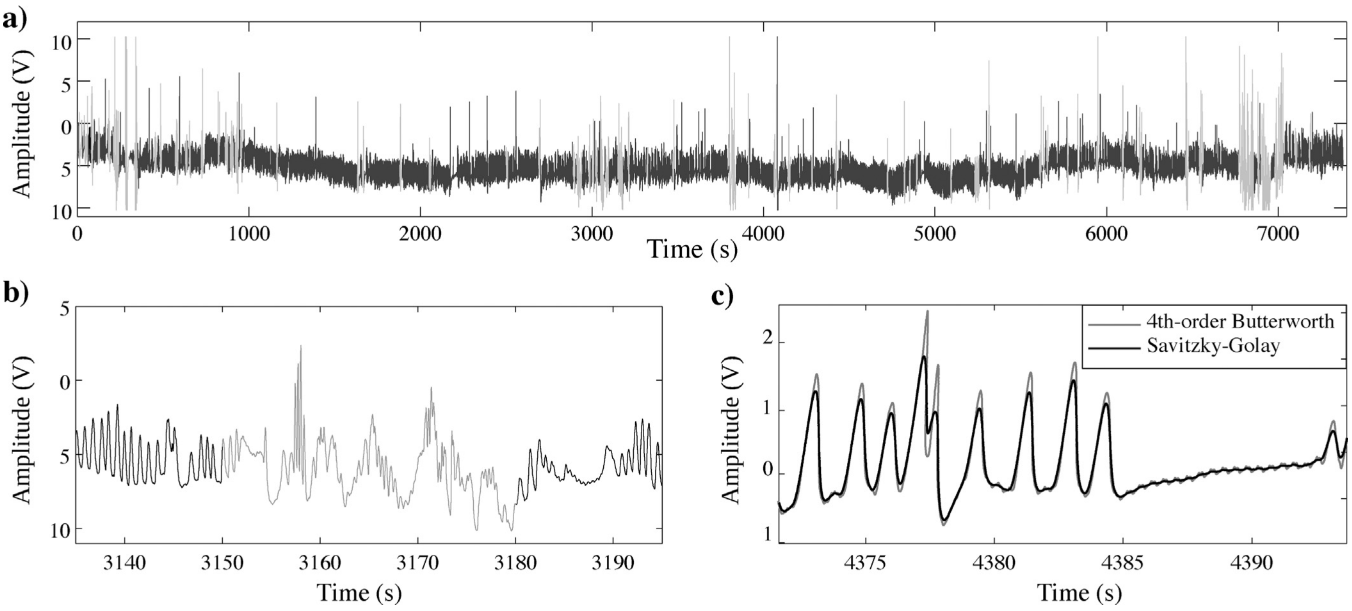

Fig. 5. (a) Example of artifact detection (excerpts in gray) in a patient not used in the training set. As the baseline evidences, no filtering is still performed at this step. (b) Detail of detected artifacts. (c) Detail of the signal, after being processed by the two filters. Note that the small ripple disappears after the SG filter.

smoothing, it should be considered that $n = 2$ takes into account the curvature of the signals and $n = 3$ the inflection points. Larger values are unnecessary and $n = 0$ produces the same effect than a moving average filter. Generally, m needs to be larger than n. If $m \simeq n$ the interpolation uses almost as many points as those contained in the original signal and no smoothing is produced.

After some tests, we found experimentally that an optimal filtering was obtained by fixing $n = 2$ and $m = Fs/2F_M$.

An example of a raw signal, first processed by the artifact detector and then by the filters, is shown in Fig. 5.

3.3. Performance of the cycle detector

Following the guidelines proposed by the Association for the Advancement of Medical Instrumentation [24], the parameters used to evaluate the performance of the breath detection algorithm were based on the traditionally employed measures for heart beat detectors. These were sensitivity and positive predictive value, +*P*, based upon the measures of true positives (*TP*) and false positives (*FP*) by the expression $+P = TP/(TP + FP)$.

The automatic detector was applied to the dataset having the cycles identified by the clinician, varying the threshold *th* multiplied by a coefficient κ from 0.05 to 1 in steps of 0.05. Since the measures of performance considered the number of correctly detected extrema, a peak was classified as valid if there was a match within the surrounding 10% of the cycle time with a manual label. Otherwise, it was counted as false positive. Fig. 6 shows the results of the performances for thresholds based on the IQR, attaining $Sn = Sp = 97\%$ or $Sp = +P = 97.7\,\%$ depending on the cut-off point.

4. Clinical application: detection of infection

As the diagnosis of sepsis still needs blood culture – invasive, slow and low predictive in the earliest phases of infection [23] – in last years it has been an increasing interest on alternative tools based on the analysis of biomedical signals. In this section we propose a linear analysis, employing standard statistical measures and

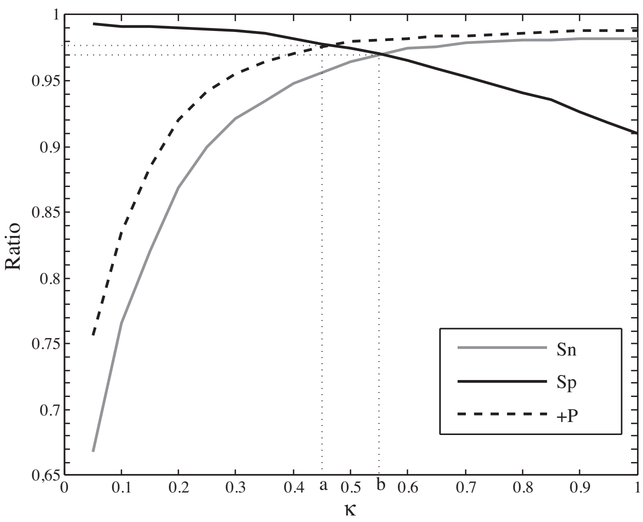

Fig. 6. Performance plot of the cycle detector, applying the κ coefficient to the IQR of the breathing amplitudes. Two possible optimal operating points are indicated: (a) the crossing of specificity (*Sp*) and positive predictive value (+*P*) in κ=0.46 and (b) the crossing of specificity and sensitivity in κ=0.55.

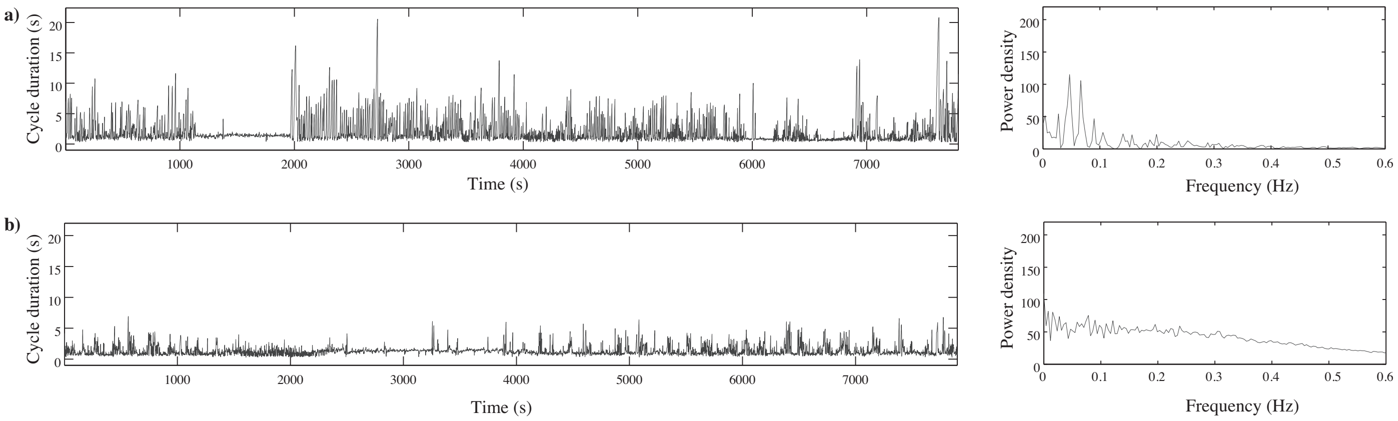

Fig. 7. (a) RVS having periodic breathing and its power density. Periodicities can be found in the spectrum between 0.05 and 0.1 Hz. (b) RVS predominantly regular, characterized by a more flat spectrum.

a basic spectrum analysis, to assess the potential of RVS to detect sepsis.

4.1. Selection of variables

The testing cohort was firstly preprocessed by the noise rejection algorithm, the filters and the cycle detection module. Clean respiratory variability time series t_{tot}, t_i, t_e were then built to find differences between *Sepsis* and *No-sepsis* groups. A first comparison of the general descriptors of breathing could be done between groups. These descriptors reflect the temporal distribution and irregularity of breathing:

- Concerning cycles: The mean duration of cycle times ($\bar{t_{tot}}$) in seconds and their standard deviations (σt_{tot}) to describe breathing variability were estimated. To describe the shape of breathing cycles, we computed the mean ratio of inspiration and exhalation times $\overline{t_i/t_e}$.
- Concerning apnea: The average number of apneas (it is defined as a cessation of the ventilatory activity during at least 15 s), normalized in 1 h (N_{ap}), their mean duration (D_{ap}) and their standard deviation (σ_{ap}) in seconds were computed.

Inspired by the spectral analysis of the heart beat ratio, we hypothesize that some system's control information may be obtained by investigating periodic components in the respiratory rhythm. In RVS spectrum, only data about the duration of cycles is processed and bias due to amplitude variations in breaths can be avoided. Indeed, temporal signals are directly measured from abdominal movements – uncalibrated and noisy – and a spectral analysis would not provide reliable information.

Therefore, we computed the power spectra using the Fast Fourier Transform (FFT) on RVS (see Fig. 7). To this end, the series were previously resampled-interpolated to obtain equidistantly time sampled signals at 2 Hz, as done traditionally with heart rate variability series [22], Dividing the range of frequencies into four components expressed as the percentage relative to the total spectral power:

- VLF, or very low frequency: defined from 0 to 0.05 Hz.
- LF, or low frequency: from 0.05 to 0.15 Hz.
- MF, or medium frequency: from 0.15 to 0.25 Hz.
- HF, or high frequency: from 0.25 to 0.60 Hz.

Note that the above frequencies have been chosen arbitrarily and the components are not equivalent to those typically studied for heart rate variability because of the different dynamics of breathing.

Table 3
Results from the comparative study of *Sepsis* and *No-sepsis* populations. The variables are expressed as mean ± std. dev. Asterisks denote statistically significant differences in a Mann–Whitney *U* test ($p < 0.05$).

	Sepsis	*No-sepsis*
t_{tot} (s)	1.49 ± 0.45	1.37 ± 0.26
σt_{tot}	1.27 ± 0.59	0.84 ± 0.24*
t_i/t_e	1.17 ± 0.10	1.12 ± 0.07
N_{ap}	50.6 ± 33.7	41.1 ± 34.0
D_{ap} (s)	8.09 ± 2.84	5.91 ± 1.20*
σ_{ap}	3.48 ± 2.06	2.01 ± 1.01*
VLF (%)	66.30 ± 19.93	56.87 ± 19.86
LF (%)	29.98 ± 17.20	39.15 ± 17.52
HF (%)	3.32 ± 3.43	3.77 ± 2.85

4.2. Results and discussion

The results comparing the respiratory variables between healthy and infected patients are summarized in Table 3. They show that *Sepsis* patients have in average slightly longer t_{tot}, a higher number of apneas N_{ap}, higher VLF and lower LF contents, although statistical tests did not find significance.

On the other hand, the standard deviation of the cycle duration (σt_{tot}) was significantly higher, a fact that reveals more unstable breathing patterns in sick infants. Moreover, the mean duration of apneas, D_{ap}, as its standard deviation, σ_{ap}, are substantially longer.

Therefore, it can be stated that breathing in infected infants is, in general, more variable concerning the duration of breaths and characterized by longer episodes of apnea. Considering the relationships between apnea and bradycardia, these results are complementary to the findings related to cardiac variability, in which severe, unusual and recurrent bradycardias are documented in infected populations [25,11].

5. Conclusion

This paper proposes a new framework to process the raw breathing signals acquired in the NICU to obtain clean respiratory traces and their respective respiratory variability series. Despite the probability of including corrupted segments (or excluding good segments) is non-negligible, the detector can be adjusted by modifying the cut-off point c in the logistic regression classifier. If the subsequent application does not tolerate noise, such as certain non-linear analyses of the temporal signal, c should maximize sensitivity at the cost of loosing useful information. Contrarily, applications

more robust to noise and needing long records, could deal with lower sensitivities.

Because the normal development of respiratory controlling systems is associated with characteristic changes in linear and non-linear measures, we proposed here the statistical characteristics of the breathing cycles, apneas and the spectra of RVS in relation with the infection. As expected, most of the measures obtained from our data (duration of breaths, apneas) confirmed their evolution in accordance to infection, evidencing functional and anatomical changes in the respiratory system. Indeed, linear parameters evidenced the existence of more irregular breathing cycles in sick infants, as well as longer apneas.

In conclusion, on our data sets, results were consistent to other reference works and should be useful to quantify preterm infants' maturity and the proposed detector becomes a powerful tools to obtain respiratory variability series, that can be analyzed by linear tools or nonlinear methods such as we already reported in [12].

Acknowledgments

The authors would like to thank the clinicians from the Pôle Médico-Chirurgical de Pédiatrie et de Génétique Clinique of the CHU of Rennes for their large involvement in this study.

Appendix A. Logistic regression-based classifiers

Logistic regression (LR) is a type of regression analysis derived from the linear case. In linear regression, a function $g : \mathbb{R}^d \to \mathbb{R}$ related to the probabilities π_i, is expressed as a linear combination of $\mathbf{X_i}$:

$$g(\mathbf{X_i}) = w_0 + w_1 X_{1,i}, .., w_d X_{d,i} = w_0 + \boldsymbol{w} \cdot \mathbf{X_i}, \quad (3)$$

where $\boldsymbol{w} = [w_1, .., w_d]$ is the vector of regression coefficients and w_0 is the intercept coefficient. In logistic regression, π_i is expressed through a function called logit, of the form:

$$logit(\pi_i) = \ln\left(\frac{\pi_i}{1-\pi_i}\right) = w_0 + \boldsymbol{w} \cdot \mathbf{X_i}. \quad (4)$$

The inverse of the above expression, called logistic function, is expressed as:

$$logit^{-1}(\pi_i) = \frac{1}{1 + e^{-(w_0 + \boldsymbol{w}\mathbf{X_i})}} = g(\mathbf{X_i}, \boldsymbol{w}). \quad (5)$$

An important characteristic of the logistic function is that it is bounded between 0 and 1, and thus, it can be used directly to model the probabilities of the possible outcomes:

$$P(Y = 1 \mid \boldsymbol{w}, \mathbf{X_i}) = g(\mathbf{X_i}, \boldsymbol{w}). \quad (6)$$

Given the set of N examples $\{\mathbf{X_i}, Y_i\}$, the learning process aims at finding the best $\boldsymbol{w}$, which is to maximize the conditional probabilities $P(Y_i \mid \mathbf{X_i}, \boldsymbol{w})$. This can be achieved by maximizing the log likelihood function:

$$L(\boldsymbol{w}) = \sum_{i=1}^{N} Y_i \ln P(Y_i = 1 \mid \mathrm{X_i}, \boldsymbol{w}) + (1 - Y_i) \ln P(Y_i = 0 \mid \mathrm{X_i}, \boldsymbol{w}). \quad (7)$$

The maximum likelihood estimate (MLE) gives the optimal coefficients, $\widehat{\boldsymbol{w}}$, employed to estimate the probabilities, $\widehat{\pi}_i$. It is found by a numerical optimization, a process that starts with a set of initial values and iterate according to a hill-climbing algorithm such as Newton-Raphson's method.

Several approaches exist to assess the goodness of the fitted model. The principle of some of them is to estimate two models and compare the fits removing predictor variables from one of the two models. By doing this, the model having less variables (the most restrictive) fits less well, showing a lower log likelihood. The likelihood ratio test, for instance, compares the log likelihoods of the two models and if this difference is statistically significant, then the less restrictive model is chosen as it fits the data significantly better.

The Wald test approximates the likelihood ratio test but only requires estimating one model. Essentially, it tests the null hypothesis that a set of parameters is equal to some value, zero if some variable is removed. If the test fails to reject the null hypothesis, then removing the variables from the model does not harm the fit of that model significantly, because a predictor with a coefficient that is very small relative to its standard error does not generally contribute substantially predict the dependent variable. The contribution of a given coefficient $\widehat{w}_j$ to the model can be quantified by the z-statistic:

$$z = \frac{\widehat{w}_j^{\,2}}{SE^2}, \quad (8)$$

where SE is the standard error (an estimation of the standard deviation) of the estimated parameter.

The decision of class membership given by the activation function is bounded by a cut-off value c, such that $f(\widehat{\pi}_i) > c$ assigns the predictive output value, $\widehat{y}$, to class 1, and $f(\widehat{\pi}_i) \le c$ assigns $\widehat{y}$ to class 0. By default $c = 0.5$, but it can be adjusted to certain specificity or sensitivity specifications.

References

[1] H.C. Miller, F.C. Behrle, N.W. Smull, Severe apnea and irregular respiratory rhythms among premature infants: a clinical and laboratory study, Pediatrics 23 (April (4)) (1959) 676–685.

[2] E.R. Alden, T. Mandelkorn, D.E. Woodrum, R.P. Wennberg, C.R. Parks, W.A. Hodson, Morbidity and mortality of infants weighing less than 1.000 grams in an intensive care nursery, Pediatrics 50 (July (1)) (1972) 40–49.

[3] M. Durand, L.A. Cabal, F. Gonzalez, S. Georgie, C. Barberis, T. Hoppenbrouwers, J.E. Hodgman, Ventilatory control and carbon dioxide response in preterm infants with idiopathic apnea, Am. J. Dis. Child. 139 (July (7)) (1985) 717–720.

[4] R.J. Martin, M.J. Miller, W.A. Carlo, Pathogenesis of apnea in preterm infants, J. Pediatr. 109 (November (5)) (1986) 733–741.

[5] C.F. Poets, V.A. Stebbens, M.P. Samuels, D.P. Southall, The relationship between bradycardia, apnea, and hypoxemia in preterm infants, Pediatr. Res. 34 (August (2)) (1993) 144–147.

[6] H. Rigatto, J.P. Brady, Periodic breathing and apnea in preterm infants: I. Evidence for hypoventilation possibly due to central respiratory depression, Pediatrics 50 (August (2)) (1972) 202–218.

[7] G. Pichler, B. Urlesberger, W.M. ller, Impact of bradycardia on cerebral oxygenation and cerebral blood volume during apnea in preterm infants, Physiol. Meas. 24 (August (3)) (2003) 671–680.

[8] A. Janvier, M. Khairy, A. Kokkotis, C. Cormier, D. Messmer, K.J. Barrington, Apnea is associated with neurodevelopmental impairment in very low birth weight infants, J. Perinatol. 24 (December (12)) (2004) 763–768.

[9] M. Altuve, G. Carrault, J. Cruz, A. Beuchee, P. Pladys, A. Hernandez, Analysis of the QRS complex for apnea-bradycardia characterization in preterm infants, in: Annual International Conference of the IEEE Engineering in Medicine and Biology Society, 2009 (EMBC 2009), IEEE, September, 2009, pp. 946–949.

[10] H. Cao, D.E. Lake, M.P. Griffin, J.R. Moorman, Increased nonstationarity of neonatal heart rate before the clinical diagnosis of sepsis, Ann. Biomed. Eng. 32 (February (2)) (2004) 233–244.

[11] A. Beuchée, G. Carrault, J.Y. Bansard, E. Boutaric, P. Bétrémieux, P. Pladys, Uncorrelated randomness of the heart rate is associated with sepsis in sick premature infants, Neonatology 96 (2) (2009) 109–114.

[12] X. Navarro, F. Porée, A. Beuchée, G. Carrault, Performance analysis of Hurst exponent estimators using surrogate-data and fractional lognormal noise models: application to breathing signals from preterm infants, Digit. Signal Process. 23 (5) (2013) 1610–1619.

[13] R.A. Polin, The ins and outs of neonatal sepsis, J. Pediatr. 143 (1) (2003) 3–4.

[14] A.L. Motto, H.L. Galiana, K.A. Brown, R.E. Kearney, Detection of movement artifacts in respiratory inductance plethysmography performance analysis of a Neyman Pearson energy based detector, in: 26th Annual International Conference of the IEEE Engineering in Medicine and Biology Society, 2004 (IEMBS'04), vol. 1, September, 2004, pp. 49–52.

[15] S. Kay, Fundamentals of Statistical Signal Processing: Detection Theory, vol. II, 1st ed., Prentice Hall, February, 1998.

[16] N. Mammone, F.C. Morabito, Enhanced automatic artifact detection based on independent component analysis and Renyi's entropy, Neural Netw. 21 (September (7)) (2008) 1029–1040.

[17] S. Dreiseitl, L. Ohno-Machado, Logistic regression and artificial neural network classification models: a methodology review, J. Biomed. Inform. 35 (5) (2002) 352–359.

[18] A. Savitzky, M.J.E. Golay, Smoothing and differentiation of data by simplified least squares procedures, Anal. Chem. 36 (1964) 1627–1639.
[19] W.P. Holsinger, K.M. Kempner, M.H. Miller, A QRS preprocessor based on digital differentiation, IEEE Trans. Biomed. Eng. BME-18 (May (3)) (1971) 212–217.
[20] J. Pan, W.J. Tompkins, A real-time QRS detection algorithm, IEEE Trans. Biomed. Eng. 32 (March (3)) (1985) 230–236.
[21] R. Schafer, What is a Savitzky–Golay filter? (lecture notes), IEEE Signal Process. Mag. 28 (July (4)) (2011) 111–117.
[22] M. Malik, J.T. Bigger, A.J. Camm, R.E. Kleiger, A. Malliani, A.J. Moss, P.J. Schwartz, Heart rate variability standards of measurement, physiological interpretation, and clinical use, Eur. Heart J. 17 (3) (1996) 354–381.
[23] A. Malik, C.P.S. Hui, R.A. Pennie, H. Kirpalani, Beyond the complete blood cell count and c-reactive protein: a systematic review of modern diagnostic tests for neonatal sepsis, Arch. Pediatr. Adolesc. Med. 157 (June (6)) (2003) 511–516.
[24] ANSI/AAMI CE57, Testing and Reporting Performance Results of Cardiac Rhythm and ST Segment Measurement Algorithms. Recommended Practice, American National Standard, 1998 http://www.aami.org
[25] M.P. Griffin, T.M. O'Shea, E.A. Bissonette, F.E. Harrell, D.E. Lake, J.R. Moorman, Abnormal heart rate characteristics preceding neonatal sepsis and sepsis-like illness, Pediatr. Res. 53 (6) (2003) 920–926.
