# Supplementary material for: Exploring Computational Techniques in Preprocessing Neonatal Physiological Signals for Detecting Adverse Outcomes: Scoping Review
Source: Interact J Med Res. 2024 Aug 20;13:e46946. doi: 10.2196/46946 (PMC11372324; doi:10.2196/46946)
Supplement: Multimedia Appendix 3 [file ijmr_v13i1e46946_app3.zip › Included Papers - Final/3725/Lloyd et al. - 2016 - Predicting 2-y outcome in preterm infants using ea.pdf]

nature publishing group

# Predicting 2-y outcome in preterm infants using early multimodal physiological monitoring

Rhodri O. Lloyd[1,2], John M. O'Toole[1], Vicki Livingstone[1], William D. Hutch[1], Elena Pavlidis[1,2], Anne-Marie Cronin[3], Eugene M. Dempsey[1,2], Peter M. Filan[1,2] and Geraldine B. Boylan[1,2]

**BACKGROUND:** Preterm infants are at risk of adverse outcome. The aim of this study is to develop a multimodal model, including physiological signals from the first days of life, to predict 2-y outcome in preterm infants.

**METHODS:** Infants <32 wk gestation had simultaneous multichannel electroencephalography (EEG), peripheral oxygen saturation ($SpO_2$), and heart rate (HR) monitoring. EEG grades were combined with gestational age (GA) and quantitative features of HR and $SpO_2$ in a logistic regression model to predict outcome. Bayley Scales of Infant Development-III assessed 2-y neurodevelopmental outcome. A clinical course score, grading infants at discharge as high or low morbidity risk, was used to compare performance with the model.

**RESULTS:** Forty-three infants were included: 27 had good outcomes, 16 had poor outcomes or died. While performance of the model was similar to the clinical course score graded at discharge, with an area under the receiver operator characteristic (AUC) of 0.83 (95% confidence intervals (CI): 0.69–0.95) vs. 0.79 (0.66–0.90) ($P = 0.633$), the model was able to predict 2-y outcome days after birth.

**CONCLUSION:** Quantitative analysis of physiological signals, combined with GA and graded EEG, shows potential for predicting mortality or delayed neurodevelopment at 2 y of age.

Of the 15 million premature births worldwide each year, one to three million infants will die, approximately 10–12% will develop cerebral palsy and a further 19% will develop motor or cognitive problems (1,2). Accurate and early prediction of neurodevelopmental outcome in the preterm infant provides important clinical information that can be used to guide early intervention, assist clinical management, and ensure appropriate long-term needs are identified. Predicting outcome at 2 y or more, in the first few days after birth is ambitious however, as preterm infants are vulnerable to brain injury during their entire stay in the neonatal intensive care unit (NICU) (3).

Many studies have attempted to predict short-term outcome, within the NICU period. Early clinical information, including Apgar scores, gender, birth weight (BW), gestational age (GA) (4–7), and illness severity scores, such as SNAP-II and SNAPPE-II have been used to predict short-term outcome (8). Quantitative analysis of multiple risk factors combined in a multivariate model can improve outcome prediction (7). Saria *et al.* (9) showed that a combination of quantitative features of early physiological measurements, including heart rate (HR), respiratory rate (RR), and peripheral oxygen saturation ($SpO_2$), could predict short-term outcome with a high level of accuracy (sensitivity of 86% and specificity of 96%). The absence of a reliable measure of neurological function, however, may limit the ability of these approaches to predict neurodevelopment in the longer term, beyond the early intensive care stage. Multivariate models including clinical risk factors such as GA, BW, and gender, have shown promise for predicting long-term outcome (10,11).

Previous studies have shown that early measurements of EEG can predict long-term neurodevelopmental outcome, with specificity and sensitivity ranging from 88 to 96% and 25 to 83%, respectively (12–14). Other studies have shown that the amplitude integrated EEG (aEEG) can predict long-term outcome, with specificity ranging from 73 to 89% and sensitivity ranging from 56 to 87% (15–17). To date, however, no standardized method for the accurate prediction of long-term outcome in very preterm infants has been successfully translated into clinical practice.

The aim of this study was to determine if multimodal physiological monitoring including EEG, recorded in the first day of life, combined with demographic risk factors such as BW and GA, can predict outcome status at 2 y of age in very preterm infants. The multimodal model combines EEG grading with quantitative features of routinely-available physiological signals, namely $SpO_2$ and HR (9). A clinical course score, which represents a best estimate of long-term outcome from clinical history of the intensive care period, is used to compare performance of this multimodal approach.

## RESULTS

### Subjects

During the study period, 152 preterm infants were born at the Cork University Maternity Hospital (CUMH) below 32 wk,

[1]Neonatal Brain Research Group, Irish Centre for Fetal and Neonatal Translational Research (INFANT) and the Department of Paediatrics & Child Health, University College Cork, Cork, Ireland; [2]Department of Neonatology, Cork University Maternity Hospital, Cork, Ireland; [3]Department of Physiotherapy, Cork University Maternity Hospital, Cork, Ireland. Correspondence: Geraldine B. Boylan (g.boylan@ucc.ie)

Received 22 September 2015; accepted 16 March 2016; advance online publication 18 May 2016. doi:10.1038/pr.2016.92



Copyright © 2016 International Pediatric Research Foundation, Inc.

of which 81 were enrolled, while the others were missed or refused to consent. From the 81 enrolled, 43 preterm infants met the inclusion criteria for this study. Recording of simultaneous multimodal physiological data commenced within 24 h (mean = 8 h 37 min, SD = 5 h 56 min) of birth and continued for up to 72 h in many cases and longer if clinically warranted. The mean recording duration was 41 h 40 min (SD = 13 h 19 min). Data at both the 12- and 24-h time-points was collected from 33 infants, only the 12-h time-point was collected from 3 infants and only the 24-h time-point was collected from 7 infants. Clinical and demographic characteristics and their relationship with outcome are detailed in **Table 1**. GA ranged from 23.42 to 31.86 wk, with a median (interquartile range) of 28.71 (26.21 to 29.93) weeks. Morphine or phenobarbitone was given to six infants.

### Clinical Course Score

Twenty-two (51.2%) infants were classified as high risk of morbidity and 21 (48.8%) infants as low risk of morbidity based on our clinical grading system.

### EEG Analysis

Thirty-two infants had a normal EEG (74.4%) and 11 (25.6%) had an abnormal EEG; of these 11, three had seizures. An inter-rate agreement was found for the EEG grading, with a Cohen's $\kappa$-coefficient of 0.97.

**Table 1.** Clinical characteristics of the infants, comparing infants with a good and poor outcome

| | Good outcome ($n$ = 27) Median (IQR) | Poor outcome ($n$ = 16) Median (IQR) | *P* value[a] |
|---|---|---|---|
| Gestational age (weeks) | 28.87 (28.29 to 30.14) | 26.29 (24.86 to 29.57) | 0.022 |
| Weight (g) | 1,040 (885 to 1,327) | 800 (675 to 1,315) | 0.122 |
| Apgar score 5 min | 9.0 (8.0 to 9.0) | 8.0 (5.3 to 8.0) | 0.001 |
| Initial pH | 7.2 (7.1 to 7.3) | 7.2 (7.1 to 7.3) | 0.88 |
| | *n* (%) | *n* (%) | *P* value[b] |
| Gender | | | |
| Male | 7 (26) | 9 (56) | 0.059 |
| Illness | | | |
| Grade III/IV IVH or cystic PVL | 2 (7) | 5 (31) | 0.082 |
| Sepsis | 6 (22) | 7 (44) | 0.178 |
| Necrotizing enterocolitis | 0 (0) | 4 (25) | 0.015 |
| Chronic lung disease | 0 (0) | 5 (31) | 0.005 |
| Retinopathy of prematurity | 0 (0) | 0 (0) | 1 |
| Mortality | 0 (0) | 4 (25) | 0.015 |
| EEG | | | |
| Seizures | 0 (0) | 3 (19) | 0.045 |
| Normal | 24 (89) | 8 (50) | 0.010 |

Outcome was defined as neurodevelopmental delay at 2 y of age or death.
[a]Mann-Whitney *U*-test. [b]Fisher's exact test.
EEG, electroencephalography; IVH, intraventricular hemorrhage; IQR, interquartile range; PVL, periventricular leukomalacia.

### Outcome Assessment

Four infants died in the neonatal period. Using the Bayley III Scales, 27 (69.2%) surviving infants had a good neurodevelopmental outcome, and 12 (30.8%) had a poor outcome. Infants with a poor outcome had lower GA ($P$ = 0.022) and were more likely to have NEC ($P$ = 0.015) or chronic lung disease ($P$ = 0.005).

### Data Analysis

The area under the receiver operator characteristic (AUC) was used to rank features and the highest-ranking feature from each modality (heart rate, $SpO_2$, and GA–BW) were HR skew, mean $SpO_2$, and GA. These three features were combined with EEG grades for use in the regression model. Their unadjusted and adjusted odds ratios are given in **Table 2**, indicating that all four features are statistically significant in the multivariate logistic regression model. Although some of the features were significantly correlated, the correlation values were small (<0.5) thus making it unlikely that multi-collinearity would affect the regression model. **Table 3** presents the univariate analysis of these four features as well as the clinical course score, and the regression model.

Lower GA, lower mean $SpO_2$, lower HR skew, and abnormal EEG grade were predictive of an abnormal outcome. AUC for the regression model is similar to the clinical course score: AUC (95% CI) for the regression model is 0.83 (0.69–0.95) vs. clinical course score 0.79 (0.66–0.90), $P$ = 0.633. Although the regression model has a higher AUC than the AUC of the EEG grade alone 0.69 (0.55–0.83), we find no statistical improvement, $P$ = 0.124.

## DISCUSSION

A combination of GA and multimodal physiological signal analysis, recorded within the first 72 h after birth, has the potential to predict death or neurodevelopmental delay at 2 y of age. The adjusted odds ratios (ORs) show that every feature uniquely contributes to the evaluation of outcome and should therefore be included. Although the multimodal model had a larger AUC (0.83) compared to HR skew (0.78), mean $SpO_2$ (0.78), EEG (0.69), and clinical course score (0.79), the

**Table 2.** OR for four features individually (unadjusted OR) and combined within the logistic regression model (adjusted OR)

| Features in regression model | Unadjusted OR (95% CI) | Adjusted OR (95% CI) |
|---|---|---|
| Gestational age (days) | 0.94 (0.90–0.99) | 0.94 (0.92–0.94) |
| Mean SpO2 (%) | 0.71 (0.55–0.91) | 0.82 (0.73–0.86) |
| Heart rate skew | 0.54 (0.33–0.87) | 0.63 (0.50–0.73) |
| EEG grade | 8.0 (1.7–37.7) | 2.9 (2.3–4.8) |

Features are considered statistically significant if the 95% CI excludes 1. The reference EEG grade is the abnormal grade.
CI, confidence interval; EEG, electroencephalography; OR, odds ratio; SpO2, oxygen saturation.

Copyright © 2016 International Pediatric Research Foundation, Inc.

**Table 3.** Univariate analysis and multivariate analysis for prediction of neurodevelopmental outcome

| | AUC (95% CI) | *P* value | Sensitivity (95% CI) | Specificity (95% CI) | PPV (95% CI) | NPV (95% CI) |
|---|---|---|---|---|---|---|
| Physiological features | | | | | | |
| Heart rate skew | 0.78 (0.63–0.92) | 0.002[a] | 70 (56–87) | 69 (54–88) | 79 (62–93) | 58 (38–82) |
| Mean SpO2 | 0.78 (0.62–0.90) | 0.003[a] | 78 (58–88) | 75 (57–90) | 84 (67–96) | 67 (38–85) |
| EEG grade | 0.69 (0.55–0.83) | 0.010[b] | 50 (25–73) | 89 (75–100) | 73 (42–100) | 75 (60–91) |
| Patient demographics | | | | | | |
| Gestational age | 0.71 (0.55–0.87) | 0.022[a] | 67 (43–89) | 69 (45–88) | 78 (55–93) | 55 (30–82) |
| Clinical assessments | | | | | | |
| Clinical course score | 0.79 (0.66–0.90) | <0.001[b] | 88 (69–100) | 70 (53–85) | 64 (43–83) | 90 (75–100) |
| Regression model | 0.83 (0.69–0.95) | | 75 (60–93) | 74 (60–92) | 63 (40–87) | 83 (67–96) |

Univariate analysis (physiological features, patient demographics and clinical assessments) and multivariate analysis (regression model) for prediction of good ($n = 27$) and poor ($n = 16$) neurodevelopmental outcome (including death). Comparison of the regression model with features of the physiological signals, basic patient demographics and later (clinical course score) clinical assessments. Multivariate analysis for the logistic regression model uses cross-validation.
[a]Mann-Whitney U-test. [b]Fischer's exact test.
AUC, area under the receiver operator characteristic; EEG, electroencephalography; NPV, negative predictive value; PPV, positive predictive value; SpO2, oxygen saturation.

differences failed to reach statistical significance. Lack of statistical significance may be due to the small sample size and hence the low power of the tests. Further studies with larger numbers are required to confirm the results observed in this study. The clinical course score included all relevant clinical information for the entire NICU duration, whereas the multimodal model was developed from information obtained in the early transitional period and thus has the advantage of being available in the first few days after birth. This finding highlights the potential value of multimodal monitoring during the transitional period and its possible role in outcome prediction, which could provide useful information for neonatologists in the NICU when guiding early treatment strategies.

Early EEG grade alone demonstrated low sensitivity (50%) and high specificity (89%), highlighting the possible limitation of the EEG grades for the prediction of death or long term neurodevelopmental delay. These results are consistent with other studies which demonstrated sensitivities of 25–61% (12,16). Although many studies have shown EEG grading to be predictive of long term outcome, none have shown that simple quantitative features of the readily-available $SpO_2$ and HR have similar—if not better—performance at predicting 2-y outcome. We find sensitivity and specificity values of 70 and 69% respectively, using quantitative analysis of an HR feature and values of 78 and 75% respectively, using $SpO_2$ quantitative analysis alone. Abnormal HR variability is associated with fetal and neonatal distress (18). A correlation between abnormal HR variability and clinical signs of sepsis has been reported (19). Sepsis is the main cause of preterm infant death during the first week of life and can also increase vulnerability of the brain due to inflammation and white matter damage (20). Low SpO2 to the point of hypoxia, can cause tissue damage of the brain which may result in neurological compromise and neurodevelopmental delay (21). The clinical course score had a higher sensitivity (88%) and similar specificity (70%) to the HR feature. The five risk factors included in our clinical course score were chosen *a priori* as they are associated with long-term morbidity. Intraventricular hemorrhage and cystic periventricular leukomalacia are direct injuries to the brain which increase the risk of developing cerebral palsy and cognitive impairment (22). Developing chronic lung disease is another common condition in preterm infants, which can also impact on neurodevelopment (23). Neurodevelopment dysfunction is also increased in preterm infants who require surgery for necrotizing enterocolitis (24), who are exposed to sepsis (25), or suffer from severe retinopathy of prematurity (26). The clinical course scores were collected at discharge, following diagnosis of any of these major complications, therefore more information, comparative to the early physiological analysis, was available to accurately predict outcome. Yet the multimodal model does provide a more balanced sensitivity–specificity result (75–74%) compared to the clinical score.

Medlock *et al.* (7) found that multivariate models of early clinical information predicted mortality in preterm infants better than BW or GA alone. Studies implementing the commonly used SNAP-II and SNAPPE-II scores showed a range of AUC values for the prediction of neonatal mortality, from 0.66 to 0.78 in SNAP-II studies and 0.60 to 0.91 in SNAPPE-II studies (8). These studies concentrated on predicting mortality only, whereas we were also interested in predicting outcome in survivors. Broitman *et al.* (10) found that a model based on clinical variables performed better than a model using head ultrasound for predicting outcome at both 28 d and 36 wk. Some clinical variables included in this early assessment (by postnatal day 28) were GA and BW. Tyson *et al.* (11) demonstrated that a five-factor model which consists of GA, BW, gender, exposure to antenatal corticosteroids, and singleton vs. twin birth, performed better than GA alone for the prediction of outcome in a cohort of preterm infants between 22–25 wk GA. Our AUC results showed an improvement from both these two predictive models (10,11). Also for our study, the sensitivity, specificity, and OR values showed similar values or improvements to previous studies in which EEG or aEEG was evaluated as one predictor or the only predictor (13,15,16). However, studies that examined serial EEG recordings or used


Copyright © 2016 International Pediatric Research Foundation, Inc.

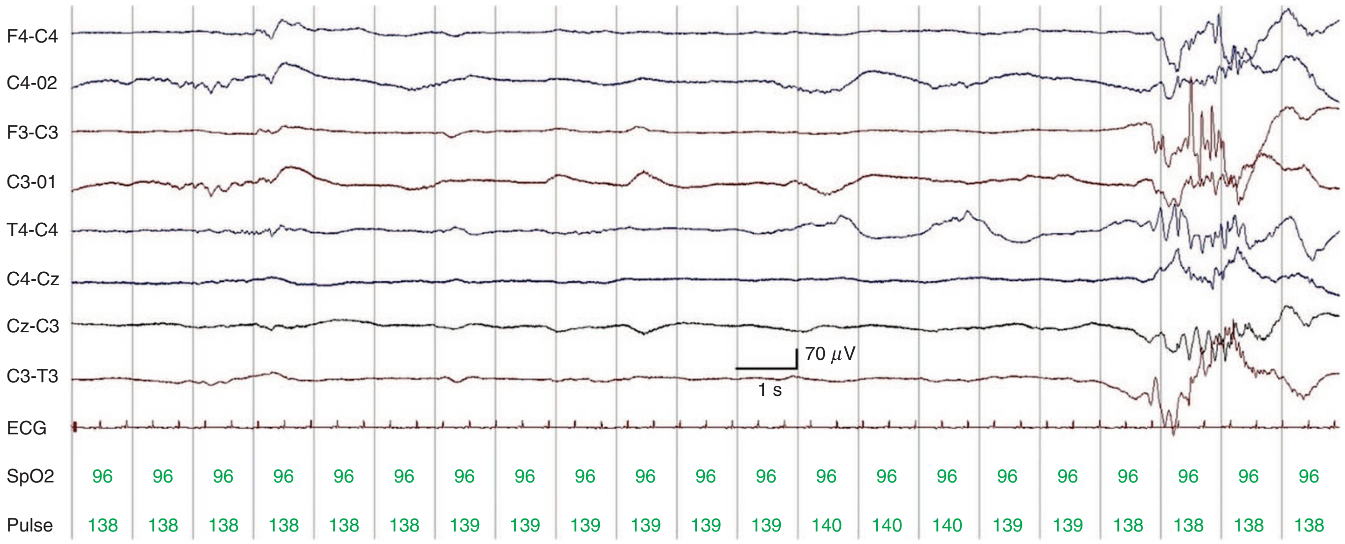


**Figure 1.** Multimodal signals—electroencephalography (EEG) recording of male 26+0 wk GA at 9 h of age. The recording displays the raw EEG, $SpO_2$, and heart rate channels.

a larger cohort size had better sensitivity or specificity values (12,14).

The main limitation of this study is the small sample size and the consequent low statistical power. Although data were collected over a 2-y period in a large maternity hospital, this was a retrospective study and some records had limited EEG data, missing physiological data, or missing outcome data. With low power, only large improvements will reach statistical significance. For example, comparing AUCs between the multivariate model and EEG, we found a difference of 14% but this lacked statistical significance ($P = 0.12$). Another consequence of small numbers is the limit on the number of explanatory variables that the multivariate model can accommodate without over fitting. Because of this limitation, we chose to include only physiological signals in the model in addition to GA, as GA is readily available. Clinical assessments such as Apgar scores were not considered in this study mainly because of this limitation on the number of explanatory variables; but also because of the subjective nature of the score and the fact that this score does not necessarily account for intervention performed in the delivery suite (27). With larger sample sizes, other clinical factors such as respiration, blood pressure, initial pH, lactate, and Apgar, could be explored for inclusion in the multivariate model. Another noticeable limitation is that the multivariate model is not an automated system, as specialist interpretation of the EEG is required. An automated grading system could be developed for preterm EEG similar to available systems for hypoxic–ischemic encephalopathy in the EEG of term infants (28). In addition, missing data may have had a negative impact on the multimodal model: some infants did not have both 12- and 24-h data epochs available for analysis, due to either later EEG application or premature discontinuation of monitoring. Although EEG was graded with knowledge of medication history, we did not consider the effects of medication on heart rate and oxygen saturation and thus medication remains a possible confounder in this study. A potential disadvantage of monitoring at such an early stage is that other complications can occur beyond the monitoring period; early monitoring, however, can provide immediate results at the beginning of critical care in the NICU. Serial EEGs and physiological measurements over the infant's stay in the NICU could add additional predictive information (14).

The main strength of this paper is that we are using large amounts of continuous data from different sources. The EEG recordings were reviewed by experienced clinical physiologists that were not involved in the clinical care of the baby and were blinded to the clinical data. This confirmed that the recordings remained anonymous during review. Using EEG instead of the aEEG was a major asset as it provides more valuable second by second data. Although EEG is not readily available, all of the other features (HR, $SpO_2$, and GA) were objective, quantifiable, and readily available. This leads to a model which consists of multiple different features. Another strength of this paper was that the Bayley Scale of Infant Development III was used to assess all surviving infants, and performed by an experienced physiotherapist.

In conclusion, quantitative analysis of readily available physiological signals, combined with EEG and GA, shows potential for improving our ability to predict death or delayed neurodevelopment at 2 y of age. Early assessment of potential neurological impairment can aid clinical management of the infant. Future studies could consider serial multimodal analysis, including EEG, to monitor maturation and development of EEG features over the first weeks and months of life and their relation to neurodevelopmental outcome.

## METHODS

### Participants

This was a retrospective, observational study performed in the NICU of Cork University Maternity Hospital. Eligible infants were all preterm (<32 wk gestation) born between April 2009 and March 2011.

Preterm infants were included in the study if they had continuous multichannel EEG monitoring with simultaneous registration

Copyright © 2016 International Pediatric Research Foundation, Inc.

of $SpO_2$ and HR, and neurodevelopmental assessment at 2 y. Ethical approval was granted by the Clinical Research Ethics Committee of the Cork Teaching Hospitals, Ireland. Written informed parental consent was obtained.

### Physiological Recordings: EEG, $SpO_2$, and HR

The NicoletOne EEG system (CareFusion, San Diego, CA) was used to record continuous video-EEG. All EEG recordings were initiated within 24 h of birth. EEG application was performed after consultation with the medical and nursing staff and when the infant was stable. Silver-silver chloride electrodes were applied to the scalp, using a modified neonatal version of the international 10/20 system. The active electrodes were applied at positions F4, F3, C4, Cz, C3, T4, T3, O2, and O1 (29). Reference electrodes were placed at Fz and ground electrodes were behind the left ear. A Philips IntelliVue MP70 monitor (Philips Medical System, BG Eindhoven, The Netherlands) was connected to the NicoletOne EEG system, which consequently synchronized the $SpO_2$ and HR with the EEG waveforms. Preterm infants can show physiological instabilities, such as low $SpO_2$ levels and decreased variability in heart rate. Arterial $SpO_2$ measures the amount of oxygenated hemoglobin in the blood. Oxygen desaturation relates to a decrease amount of oxygen in the blood. A systematic review reported that $SpO_2$ values of approximately 85–95% should be targeted for preterm infants (30). Heart rate variability is the variation over time in the interval between heartbeats, providing assessment of the functional state of the autonomic nervous system. Monitoring continued for up to 72 h after birth, depending on the stability of the infants.

### EEG Data Collection

Eight channels of EEG were collected along with simultaneous $SpO_2$ and HR recordings (**Figure 1**). The EEG signal was sampled at 256 Hz, and the $SpO_2$ and HR were sampled at 1 Hz. The EEG recordings were visually analyzed for quality and, if this was poor, the infants were excluded.

The entire EEG recording in each infant was assessed for seizure activity, state change, and maturational features such as delta brushes, occipital delta waves, and temporal sharp waves. The EEGs were graded by two clinical physiologists (R.L. and G.B.) who were blinded to all clinical information except for GA, administration of morphine or phenobarbitone, and time of EEG recording postdelivery. The EEG recordings were scored based on the grading system described by Watanabe *et al.* (31), which differentiated acute abnormalities (ASA) from those of the chronic stage (CSA). ASA were defined as suppressed background activity, decreased continuity, low amplitude, and attenuated fast-wave background. CSA included dysmature patterns and disorganized patterns, such as abnormal delta waveforms, sharp waves, and abnormal delta brushes. ASA and CSA can be classed as mild, moderate or severe (31,32). Therefore, we graded our EEG epochs as follows: normal = normal; mild = mild ASA/CSA; moderate = moderate ASA/CSA; severe = severe ASA/CSA. EEGs were reviewed and consensus was achieved for each recording. Inter-rater agreement was assessed using Cohen's $\kappa$-coefficient.

One hour epochs of EEG at 12 and 24 h of age, were then extracted from each recording for multimodal analysis. These specific time-points were selected for analysis due to the fact that they represented the most consistent time points when multimodal data was available for the entire cohort. Most recordings included both time-points, but some were missing due to late application, instability of the infant or poor quality of the EEG recording at that time period. When both time-points were available, the EEG grades were combined and the most abnormal grade was selected.

### Additional Data Collection

One hour epochs of HR and $SpO_2$ were extracted at the two time-points, 12 and 24 h. Two features were used to summarize $SpO_2$ for the 1 h segments: mean $SpO_2$ and percentage of time <85%, which quantifies hypoxia (21,33). Four features summarized the HR signal over the 1 h segments: mean, SD, skewness, and kurtosis (34). The SD represents the variability of the HR segment; skewness represents the tendency of the HR signal to include large-amplitude transients in either the positive or negative directions, which relate to accelerations and decelerations of the HR (19); and kurtosis quantifies the deviation of the HR signal from a Gaussian process, often the result of high-amplitude transients. These higher-order statistics were included as previous studies relate short-term outcome to changes in the skewness and kurtosis (35). When available, the mean values of the features over both time points were used for subsequent analysis. Clinical and demographic characteristics were also collected.

### Assessment of Clinical Course

Infant demographics and clinical details were collected from the electronic database discharge summary document and the medical notes. Blinded to infant identity and physiological data, two consultant neonatologists (P.F. and E.D.) reviewed the discharge summary documents and medical notes for all infants. Each infant was classified as either at high or low risk of later morbidity based on their clinical course score. Infants were allocated as high risk of morbidity if they suffered from any of the five major complications during their time in the NICU (**Table 4**). When grades differed between reviewers, a consensus was reached by discussion. **Figure 2** illustrates the infant's course in the NICU.

### Two-Year Outcome Assessment

Neurodevelopmental outcome was assessed at 2 y corrected age in all surviving infants using the Bayley Scales of Infant Development III, performed by a single specialist neonatal physiotherapist (A.M.C.). This assessment measures the child's motor, cognitive and language development and provides three subscale scores. An abnormal outcome was defined as any of the three subscales being below 1 SD from the mean; thus for the standardized scores, a value of less than 85 in any of the three subscales was deemed abnormal (36). Conversely, a normal outcome was defined as every subscale being 85 or above. Infants who died were also allocated to the abnormal outcome group.

### Statistical Analysis

For statistical analysis, EEG grades were grouped into two categories: 1 = normal or mildly abnormal and 2= moderately or severe abnormal (37).

Continuous variables were described using mean (SD) and median (interquartile range) where appropriate and categorical variables described using number (percentage). The ability of each physiological feature to predict either normal or abnormal outcome was assessed using the Mann-Whitney *U*-test (continuous data) and the Fisher exact test (binary data). The AUC, sensitivity and specificity, and positive predictive values and negative predictive values were used as performance metrics. Confidence intervals (CI) of the AUC

**Table 4.** Definitions for major neonatal complications

| Five major complications |
|---|
| • Grade III/IV intraventricular hemorrhage or cystic periventricular leukomalacia |
| • Bronchopulmonary dysplasia as defined by oxygen dependency at 36 wk postmenstrual age |
| • Necrotizing enterocolitis Bells stage 2b or greater |
| • Infection – positive blood culture with abnormal inflammatory markers, white cell count or C-reactive protein levels |
| • Retinopathy of prematurity stage 2 or greater |

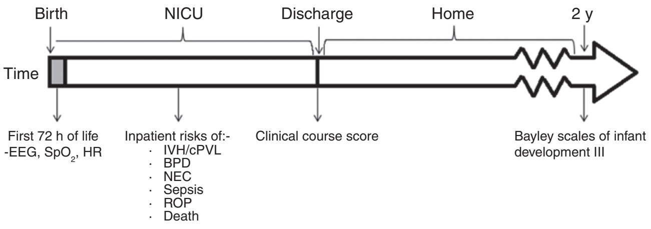


**Figure 2.** Timeline of infant's stay in the neonatal intensive care unit through to the neurodevelopmental follow-up at 2 y of age. IVH, intraventricular hemorrhage; cPVL, cystic periventricular leukomalacia; BPD, bronchopulmonary dysplasia; NEC, necrotizing enterocolitis; ROP, retinopathy of prematurity


Copyright © 2016 International Pediatric Research Foundation, Inc.

were computed using the bootstrap approach with 1,000 iterations. A multivariate logistic regression model was used to combine all features. Only one feature from each of the four modalities (EEG, $SpO_2$, HR, and GA–BW) was included in the regression model, as limiting the number of features eliminates overtraining for the model; AUC rankings determined which feature from each modality to include.

Performance of the regression model was assessed using leave-one-infant-out cross validation. This method trains the regression model by fitting parameters from all infants minus one. Performance is then tested on this single left-out infant, and this process is iterated through all infants (38). To eliminate stratification bias caused by unbalanced class proportions in each training iteration, the training set was modified to retrain constant proportions over all iterations. This modification removes, at random and at most, one infant's data per training iteration (39). The cross-validation procedure provides a better estimate of the generalization performance (the performance on the entire population) compared to the training and testing on the same sample (38).

OR was calculated for each of the four features within the multivariate model and a 95% CI was calculated from the distribution of OR values over all iterations of the cross-validation. A feature significantly contributed to the model if the 95% CI excluded 1. And lastly, the AUC for the multivariate model was compared to the AUC for the clinical course score and EEG grade alone using the bootstrap method in (40). All analyses were performed in MATLAB (version R2013a, The Mathworks, Natick, MA). All tests were two-sided and a $P$ value <0.05 was considered to be statistically significant.

## ACKNOWLEDGMENTS

The authors acknowledge the assistance of Evonne Low, Research Fellow, in collecting and recording data in neonates, and staff from the NICU of Cork University Maternity Hospital for their assistance and support throughout the recruitment period.

## STATEMENT OF FINANCIAL SUPPORT

This research was funded by a Science Foundation Research Centre Award (INFANT-12/RC/2272) and the European Commission within the 7th Framework Programme (EU FP7/2007–2013) under grant agreement no. 260777 (The HIP Trial). JMOT'. received financial support from the Irish Research Council (GOIPD/2014/396). The funders had no role in study design, data collection and analysis, decision to publish, or preparation of the manuscript.

Disclosure: All authors have indicated that they have no financial relationships relevant to this article to disclose. All authors have no conflicts of interest to disclose.

## REFERENCES

1. Howson CP, Kinney MV, McDougall L, Lawn JE; Born Too Soon Preterm Birth Action Group. Born too soon: preterm birth matters. Reprod Health 2013;10 Suppl 1:S1.
2. Platt MJ. Outcomes in preterm infants. Public Health 2014;128:399–403.
3. Benjamin DK Jr, Stoll BJ. Infection in late preterm infants. Clin Perinatol 2006;33:871–82.
4. Forsblad K, Källén K, Marsál K, Hellström-Westas L. Short-term outcome predictors in infants born at 23-24 gestational weeks. Acta Paediatr 2008;97:551–6.
5. Casey BM, McIntire DD, Leveno KJ. The continuing value of the Apgar score for the assessment of newborn infants. N Engl J Med 2001;344: 467–71.
6. Evans N, Hutchinson J, Simpson JM, Donoghue D, Darlow B, Henderson-Smart D. Prenatal predictors of mortality in very preterm infants cared for in the Australian and New Zealand Neonatal Network. Arch Dis Child Fetal Neonatal Ed 2007;92:F34–40.
7. Medlock S, Ravelli AC, Tamminga P, Mol BW, Abu-Hanna A. Prediction of mortality in very premature infants: a systematic review of prediction models. PLoS One 2011;6:e23441.
8. Richardson DK, Corcoran JD, Escobar GJ, Lee SK. SNAP-II and SNAPPE-II: Simplified newborn illness severity and mortality risk scores. J Pediatr 2001;138:92–100.
9. Saria S, Rajani AK, Gould J, Koller D, Penn AA. Integration of early physiological responses predicts later illness severity in preterm infants. Sci Transl Med 2010;2:48ra65.
10. Broitman E, Ambalavanan N, Higgins RD, et al.; National Institute of Child Health and Human Development Neonatal Research Network. Clinical data predict neurodevelopmental outcome better than head ultrasound in extremely low birth weight infants. J Pediatr 2007;151: 500–5, 505.e1–2.
11. Tyson JE, Parikh NA, Langer J, Green C, Higgins RD; National Institute of Child Health and Human Development Neonatal Research Network. Intensive care for extreme prematurity–moving beyond gestational age. N Engl J Med 2008;358:1672–81.
12. Hayashi-Kurahashi N, Kidokoro H, Kubota T, et al. EEG for predicting early neurodevelopment in preterm infants: an observational cohort study. Pediatrics 2012;130:e891–7.
13. Pisani F, Barilli AL, Sisti L, Bevilacqua G, Seri S. Preterm infants with video-EEG confirmed seizures: outcome at 30 months of age. Brain Dev 2008;30:20–30.
14. Le Bihannic A, Beauvais K, Busnel A, de Barace C, Furby A. Prognostic value of EEG in very premature newborns. Arch Dis Child Fetal Neonatal Ed 2012;97:F106–9.
15. Klebermass K, Olischar M, Waldhoer T, Fuiko R, Pollak A, Weninger M. Amplitude-integrated EEG pattern predicts further outcome in preterm infants. Pediatr Res 2011;70:102–8.
16. Wikström S, Pupp IH, Rosén I, et al. Early single-channel aEEG/EEG predicts outcome in very preterm infants. Acta Paediatr 2012;101:719–26.
17. Welch C, Helderman J, Williamson E, O'Shea TM. Brain wave maturation and neurodevelopmental outcome in extremely low gestational age neonates. J Perinatol 2013;33:867–71.
18. Logier R, De Jonckheere J, Jeanne M, Matis R. Fetal distress diagnosis using heart rate variability analysis: design of a high frequency variability index. Conf Proc IEEE Eng Med Biol Soc 2008;2008:4728–31.
19. Griffin MP, Lake DE, O'Shea TM, Moorman JR. Heart rate characteristics and clinical signs in neonatal sepsis. Pediatr Res 2007;61:222–7.
20. Mallard C, Wang X. Infection-induced vulnerability of perinatal brain injury. Neurol Res Int 2012;2012:102153.
21. Lee DS, Zahari M, Russell G, Darlow BA, Scarrott CJ, Reale M. An exploratory investigation of some statistical summaries of oximeter oxygen saturation data from preterm babies. ISRN Pediatr 2011;2011:296418.
22. Tsai AJ, Lasky RE, John SD, Evans PW, Kennedy KA. Predictors of neurodevelopmental outcomes in preterm infants with intraparenchymal hemorrhage. J Perinatol 2014;34:399–404.
23. Li Y, Cui Y, Wang C, Liu X, Han J. A risk factor analysis on disease severity in 47 premature infants with bronchopulmonary dysplasia. Intractable Rare Dis Res 2015;4:82–6.
24. Martin CR, Dammann O, Allred EN, et al. Neurodevelopment of extremely preterm infants who had necrotizing enterocolitis with or without late bacteremia. J Pediatr 2010;157:751–6.e1.
25. Alshaikh B, Yee W, Lodha A, Henderson E, Yusuf K, Sauve R. Coagulase-negative staphylococcus sepsis in preterm infants and long-term neurodevelopmental outcome. J Perinatol 2014;34:125–9.
26. Molloy CS, Anderson PJ, Anderson VA, Doyle LW. The long-term outcome of extremely preterm (<28 weeks' gestational age) infants with and without severe retinopathy of prematurity. J Neuropsychol 2015; e-pub ahead of print 24 March 2015. DOI:10.1111/jnp.12069.
27. Rüdiger M, Braun N, Aranda J, et al.; TEST-Apgar Study-Group. Neonatal assessment in the delivery room–Trial to Evaluate a Specified Type of Apgar (TEST-Apgar). BMC Pediatr 2015;15:18.
28. Ahmed R, Temko A, Marnane W, Lightbody G, Boylan G. Grading hypoxic-ischemic encephalopathy severity in neonatal EEG using GMM supervectors and the support vector machine. Clin Neurophysiol 2016;127:297–309.
29. Lloyd R, Goulding R, Filan P, Boylan G. Overcoming the practical challenges of electroencephalography for very preterm infants in the neonatal intensive care unit. Acta Paediatr 2015;104:152–7.

Copyright © 2016 International Pediatric Research Foundation, Inc.

30. Manja V, Lakshminrusimha S, Cook DJ. Oxygen saturation target range for extremely preterm infants: a systematic review and meta-analysis. JAMA Pediatr 2015;169:332–40.
31. Watanabe K, Hayakawa F, Okumura A. Neonatal EEG: a powerful tool in the assessment of brain damage in preterm infants. Brain Dev 1999;21:361–72.
32. Kidokoro H, Okumura A, Hayakawa F, et al. Chronologic changes in neonatal EEG findings in periventricular leukomalacia. Pediatrics 2009;124:e468–75.
33. Finer N, Leone T. Oxygen saturation monitoring for the preterm infant: the evidence basis for current practice. Pediatr Res 2009;65:375–80.
34. Griffin MP, Moorman JR. Toward the early diagnosis of neonatal sepsis and sepsis-like illness using novel heart rate analysis. Pediatrics 2001;107:97–104.
35. Moorman JR, Lake DE, Griffin MP. Heart rate characteristics monitoring for neonatal sepsis. IEEE Trans Biomed Eng 2006;53:126–32.
36. Schumacher EM, Larsson PG, Sinding-Larsen C, et al. Automated spectral EEG analyses of premature infants during the first three days of life correlated with developmental outcomes at 24 months. Neonatology 2013;103:205–12.
37. Pisani F, Facini C, Pelosi A, Mazzotta S, Spagnoli C, Pavlidis E. Neonatal seizures in preterm newborns: A predictive model for outcome. Eur J Paediatr Neurol 2016;20:243–51.
38. Sahiner B, Chan HP, Hadjiiski L. Classifier performance estimation under the constraint of a finite sample size: resampling schemes applied to neural network classifiers. Neural Netw 2008;21:476–83.
39. Parker BJ, Günter S, Bedo J. Stratification bias in low signal microarray studies. BMC Bioinformatics 2007;8:326.
40. Robin X, Turck N, Hainard A, et al. pROC: an open-source package for R and S+ to analyze and compare ROC curves. BMC Bioinformatics 2011;12:77.




Copyright © 2016 International Pediatric Research Foundation, Inc.
