# Supplementary material for: Exploring Computational Techniques in Preprocessing Neonatal Physiological Signals for Detecting Adverse Outcomes: Scoping Review
Source: Interact J Med Res. 2024 Aug 20;13:e46946. doi: 10.2196/46946 (PMC11372324; doi:10.2196/46946)
Supplement: Multimedia Appendix 3 [file ijmr_v13i1e46946_app3.zip › Included Papers - Final/3761/Ghahjaverestan et al. - 2016 - Coupled Hidden Markov Model-Based Method for Apnea.pdf]

Coupled Hidden Markov Model-Based Method for Apnea Bradycardia Detection

N. Montazeri Ghahjaverestan, *Student Member, IEEE*, S. Masoudi, *Student Member, IEEE*, M. B. Shamsollahi, *Senior Member, IEEE*, A. Beuchée, P. Pladys, D. Ge, *Member, IEEE*, and A. I. Hernández, *Member, IEEE*

***Abstract*—In this paper, we present a novel framework for the coupled hidden Markov model (CHMM), based on the forward and backward recursions and conditional probabilities, given a multidimensional observation. In the proposed framework, the interdependencies of states networks are modeled with Markovian-like transition laws that influence the evolution of hidden states in all channels. Moreover, an offline inference approach by maximum likelihood estimation is proposed for the learning procedure of model parameters. To evaluate its performance, we first apply the CHMM model to classify and detect disturbances using synthetic data generated by the FitzHugh–Nagumo model. The average sensitivity and specificity of the classification are above 93.98% and 95.38% and those of the detection reach 94.49% and 99.34%, respectively. The method is also evaluated using a clinical database composed of annotated physiological signal recordings of neonates suffering from apnea-bradycardia. Different combinations of beat-to-beat features extracted from electrocardiographic signals constitute the multidimensional observations for which the proposed CHMM model is applied, to detect each apnea bradycardia episode. The proposed approach is finally compared to other previously proposed HMM-based detection methods. Our CHMM provides the best performance on this clinical database, presenting an average sensitivity of 95.74% and specificity of 91.88% while it reduces the detection delay by -0.59 s.**

***Index Terms*—Apnea-bradycardia (AB), coupled hidden Markov model (CHMM), electrocardiography (ECG), forward-backward (FB) algorithm, hidden Markov model (HMM).**

I. Introduction

EPISODES of apnea-bradycardia (AB), consisting of a respiratory pause (apnea) accompanied by oxygen desaturation and a significant drop in heart rate (bradycardia), are commonly observed during the first weeks of life of preterm newborns. These episodes compromise tissue perfusion, affecting the normal development of the neurological and cardiovascular systems of the preterm infant and may even be lethal [1], [2]. The detection and characterization of AB episodes during continuous cardio-respiratory monitoring in neonatal intensive care units is thus of primary importance.

Although many methods have been presented in the literature for the detection of adult apnea events [3]–[5], the detection of AB episodes on preterm infants requires specific processing, integrating an analysis of the dynamics of heart rate through the event. The most common approaches for heart rate characterization in this context are simply based on the detection of bradycardia, by applying a fixed or an adaptive threshold on heart rate time-series [6], [7]. In our previous works, we have proposed different methods to improve the characterization of heart rate dynamics of AB episodes using abrupt-change detection methods [8], and different kinds of unidimensional hidden Markov models (HMM) [9]–[11]. In this paper, we present a significant improvement of our previous methods, by proposing a new methodological framework for the characterization of multivariate time-series dynamics, based on a particular kind of Bayesian network (BN), called the coupled hidden Markov model (CHMM). We also present a novel AB detection method for preterm infants, integrating a phase of multivariate feature extraction from the ECG, and a phase of time-series characterization through the proposed CHMM.

HMM is a particular type of BN, in which it is assumed that a sequence of discrete hidden states generates a sequence of observations of the same length. The topology of the state network and their probabilistic pattern rule the transition from one state to another. The state transition and initial state distribution govern the evolution of the unobserved hidden states, whereas the observation probability controls the observation data, for a given state sequence [12]. Although HMMs are, by definition, single-process models, different approaches have been proposed to use them for the analysis of multivariate data. One common approach is to consider the observation data as a multidimensional random variable. Although these approaches provide interesting results in some applications [13], [14] and [10], the single-process nature of HMM can not be adapted easily to other cases, where observations are indeed generated by distinct underlying processes, such as in vision, speech recognition [15] or forensic analysis [16].

Manuscript received September 22, 2014; revised January 9, 2015 and February 11, 2015; accepted February 11, 2015. Date of publication February 20, 2015; date of current version March 3, 2016.

N. Montazeri Ghahjaverestan is with the Biomedical Signal and Image Processing Laboratory, School of Electrical Engineering, Sharif University of Technology, Tehran 11356-9363, Iran and also with LTSI, University of Rennes 1 and also with INSERM, Rennes F-35000, France (e-mail: montazeri.nasim@gmail.com).

S. Masoudi and M. B. Shamsollahi are with the Biomedical Signal and Image Processing Laboratory, School of Electrical Engineering, Sharif University of Technology, Tehran 11356-9363, Iran (e-mail: masoudi@ee.sharif.eedu; mbshams@sharif.edu).

A. Beuchée and P. Pladys are with CHU Rennes, Rennes F-35000, France, and also with the INSERM, Rennes F-35000, France, and also with the LTSI, University of Rennes 1, Rennes F-35000, France (e-mail: alain.beuchee@chu-rennes.fr; patrick.pladys@universal.fr).

D. Ge and A. I. Hernández are with the INSERM, Rennes F-35000, France and also with the LTSI, University of Rennes 1, Rennes F-35000, France (e-mail: di.ge@univ-rennes1.fr; alfredo.hernandez@univ-rennes1.fr).

Color versions of one or more of the figures in this paper are available online at http://ieeexplore.ieee.org.

Digital Object Identifier 10.1109/JBHI.2015.2405075

2168-2194 © 2015 IEEE. Personal use is permitted, but republication/redistribution requires IEEE permission.
See http://www.ieee.org/publications_standards/publications/rights/index.html for more information.

Authorized licensed use limited to: CSIRO Information Technology Services. Downloaded on September 09,2022 at 00:27:34 UTC from IEEE Xplore. Restrictions apply.

In order to overcome these limitations, CHMM has been proposed by Brand in [15], [17] as a generalization of HMM. In a CHMM, each channel (Markov chain) is associated with an univariate observation to represent its underlying generation process, and transition probabilities depend on the current state of all channels. However, this structure implies that the state space grows exponentially with respect to the number of channels [17]. In order to cope with this complexity, Brand proposed a simplification, considering a factorization of the transition matrix

$$P(S_t^c | S_{t-1}^1, S_{t-1}^2, \dots, S_{t-1}^C) = \prod_{c=1}^{C} P(S_t^c | S_{t-1}^c) \quad (1)$$

where S_t^c denotes the state of channel c at time t. According to Brand's assumption, the state conditional probability in the left side of (1) is substituted by the product of all marginal conditional probabilities. This model has been successfully applied in the field of sensor fusion, such as in forensic electronics [16], genetics [18], audio-visual speech recognition systems [19] and target tracking [20].

In another study, Rezek *et al.* computed the forward-backward (FB) parameters without considering Brand's assumption in (1) and derived the maximum likelihood (ML) estimators for the CHMM parameters using the expectation maximization (EM) algorithm [21]. They considered the CHMM as a one-channel HMM with an ordered C-fold state formed by $(S^1, S^2, \dots, S^C)$, with a state space of $N = \prod_{c=1}^{C} n_c$ and a transition matrix of dimension $N \times N$. The algorithm's complexity reaches $O(TM^{2C})$ with $M = n_c$. Zhong *et al.* proposed another approach in [22], [23], in which the transition probability is expressed under the form of a weighted sum of marginal conditional probabilities, associated with a rather complex estimation method for the model parameters considering the normalization of weights.

All the previous methods are based on the recursive relations of FB variables, for which the learning procedure can be complicated and time consuming, such as in [22]. In this paper, we propose an original framework for CHMM with a considerable reduction in the complexity of the FB algorithm. Section II presents the methodological contributions regarding the proposed CHMM framework, addressing the problems of likelihood evaluation, state sequence optimization and model parameter learning. Section III presents the strategy of applying the proposed framework for detection of AB. In Section IV, the proposed method is quantitatively compared with Rezek's approach [21], as well as with classical HMM and its generalization, hidden semi Markov model (HSMM) [24], using simulated and a real database. Finally, Section V presents the conclusions of this study.

II. Proposed CHMM Framework

Let's note $\{S_1^c, S_2^c, \dots, S_{M(c)}^c\}$ to be the state space of channel c in a CHMM and let q_t^c and o_t^c, $t = 1, 2, \dots, T$ be the state and the observation of channel c at time t, respectively. C is the total number of channels in the CHMM model. Also, let $a_{nm}^{c'c} = P(q_t^c = S_m^c | q_{t-1}^{c'} = S_n^{c'})$ denotes the probability of transition to state m in channel c at time t, subjected to being in state n in channel c' at time $t-1$. The probability of the observation is written as $b_m(o_t^c) = P(o_t^c | q_t^c = S_m^c)$, where o_t^c may be either discrete or continuous. We also define $o_t = \{o_t^1, o_t^2, \dots, o_t^C\}$. In this paper, $b_m(o_t^c)$ is assumed to be normally distributed, and characterized by its mean (μ_m^c) and standard deviation (σ_m^c). For simplicity, we also note $v_t^c(m) \equiv \{q_t^c = S_m^c\}$, so that $a_{nm}^{c'c} = P(v_t^c(m) | v_{t-1}^{c'}(n))$. The structure of the interchannel coupling is depicted in Fig. 1 for the two-channel case.

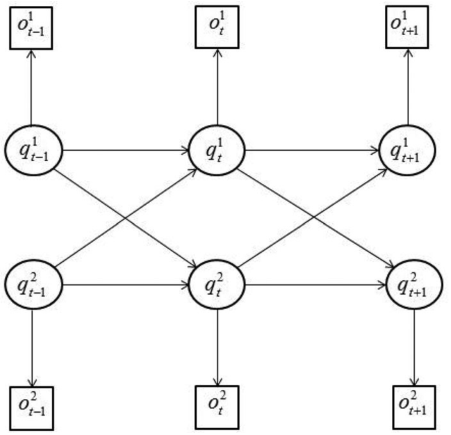

Fig. 1. BN representation for the proposed CHMM showing the probabilistic relations among the states and observations for the particular case of a two-channel CHMM.

In order to define the FB parameters, first consider the probability of observation of each channel at time t given all the previous observations

$$\begin{aligned} P(o_t^c | o_{1:t-1}) &= \sum_{m=1}^{M(c)} P(v_t^c(m), o_t^c | o_{1:t-1}) \\ &= \sum_{m=1}^{M(c)} b_m(o_t^c) P(v_t^c(m) | o_{1:t-1}). \end{aligned} \quad (2)$$

Following [24], we define the forward parameter as

$$\alpha_{t|x}^c(m) = P(v_t^c(m) | o_{1:x}) \quad (3)$$

where for $x = t-1, t, T$, the above quantity is termed as predicted, filtered and smoothed probability, respectively. The forward recursion based on the predicted probability is determined by

$$\begin{aligned} \alpha_{t|t-1}^c(m) =\ & P(v_t^c(m) | o_{1:t-1}) \\ =& \sum_{n_1=1}^{M(1)} \sum_{n_2=1}^{M(2)} \dots \sum_{n_C=1}^{M(C)} P(v_t^c(m), v_{t-1}^1(n_1), v_{t-1}^2(n_2) \\ & , \dots, v_{t-1}^C(n_C) | o_{1:t-1}) \\ =& \sum_{n_1=1}^{M(1)} \sum_{n_2=1}^{M(2)} \dots \sum_{n_C=1}^{M(C)} P(v_t^c(m) | v_{t-1}^1(n_1), v_{t-1}^2(n_2) \\ & , \dots, v_{t-1}^C(n_C), o_{1:t-1}) \\ & \times P(v_{t-1}^1(n_1), \dots, v_{t-1}^C(n_C) | o_{1:t-1}) \end{aligned} \quad (4)$$

Authorized licensed use limited to: CSIRO Information Technology Services. Downloaded on September 09,2022 at 00:27:34 UTC from IEEE Xplore. Restrictions apply.

where the first term can be simply calculated by Brand's assumption in (1). $o_{1:t-1}$ can be omitted since knowing all the previous states, it does not add any information to estimate $v_t^{\varsigma}(m)$

$$P(v_t^{\varsigma}(m)|v_{t-1}^1(n_1),\ldots,v_{t-1}^C(n_C),o_{1:t-1}) = \prod_{c=1}^{C} P(v_t^{\varsigma}(m)|v_{t-1}^c(n_c)). \quad (5)$$

For the second term we show in Appendix B that the states of channels given the observations $o_{1:t-1}$ are independent

$$P(v_{t-1}^1(n_1),\ldots,v_{t-1}^C(n_C)|o_{1:t-1}) = \prod_{c=1}^{C} P(v_{t-1}^c(n_c)|o_{1:t-1}). \quad (6)$$

Substituting (1), (5) and (6) in (4), the following recursion can be obtained:

$$\alpha_{t|t-1}^{\varsigma}(m) = \sum_{n_1=1}^{M(1)}\sum_{n_2=1}^{M(2)}\cdots\sum_{n_C=1}^{M(C)}\prod_{c=1}^{C} P(v_t^{\varsigma}(m)|v_{t-1}^c(n_c)) \times P(v_{t-1}^c(n_c)|o_{1:t-1}). \quad (7)$$

Note that $P(v_t^{\varsigma}(m)|v_{t-1}^c(n_c)) \neq P(v_t^{\varsigma}(m)|v_{t-1}^c(n_c),o_{1:t-1})$. Hence, the summations can be exchanged with the product.

$$\begin{aligned}\alpha_{t|t-1}^{\varsigma}(m) &= \prod_{c=1}^{C}\sum_{n_c=1}^{M(c)} P(v_t^{\varsigma}(m)|v_{t-1}^c(n_c))P(v_{t-1}^c(n_c)|o_{1:t-1})\\ &= \prod_{c=1}^{C}\sum_{n_c=1}^{M(c)} a_{n_c m}^{c\varsigma}\alpha_{t-1|t-1}^c(n_c)\\ &= \prod_{c=1}^{C}\sum_{n_c=1}^{M(c)} a_{n_c m}^{c\varsigma}\alpha_{t-1|t-2}^c(n_c)\tilde{b}_{n_c}^c(o_{t-1}) \end{aligned} \quad (8)$$

where $\tilde{b}_m^{\varsigma}(o_t)$ is defined as the ratio of the filtered probability, $\alpha_{t|t}^{\varsigma}(m)$, over the predicted probability $\alpha_{t|t-1}^{\varsigma}(m)$.

$$\tilde{b}_m^{\varsigma}(o_t) \triangleq \frac{\alpha_{t|t}^{\varsigma}(m)}{\alpha_{t|t-1}^{\varsigma}(m)} = \frac{P(o_t|v_t^{\varsigma}(m),o_{1:t-1})}{P(o_t|o_{1:t-1})} = \frac{P(o_t|v_t^{\varsigma}(m),o_{1:t-1})}{\prod_{c=1}^{C} P(o_t^c|o_{1:t-1})}. \quad (9)$$

The last equality is based on the observation decomposition (cf., Appendix A)

$$P(o_t|o_{1:t-1}) = \prod_{c=1}^{C} P(o_t^c|o_{1:t-1}). \quad (10)$$

Assuming the effects of other channels, the nominator of (9) is summarized as the product of distinct channel effects as follows:

$$P(o_t|v_t^{\varsigma}(m),o_{1:t-1}) = \underbrace{\sum_{n_1=1}^{M(1)}\cdots\sum_{n_C=1}^{M(C)}}_{C-1\text{ (except channel }\varsigma)} P(o_t,v_t^1(n_1),\ldots,v_t^C(n_C)|v_t^{\varsigma}(m),o_{1:t-1}) \quad (11)$$

$$= \underbrace{\sum_{n_1=1}^{M(1)}\cdots\sum_{n_C=1}^{M(C)}}_{C-1\text{ (except channel }\varsigma)} P(o_t^1|v_t^1(n_1))P(o_t^2|v_t^2(n_2))\ldots P(o_t^C|v_t^C(n_C)) \times P(v_t^1(n_1),\ldots,v_t^C(n_C)|v_t^{\varsigma}(m),o_{1:t-1}). \quad (12)$$

Since the probability calculation of o_t^c only needs the corresponding state, i.e., $v_t^c(n_c)$, we can establish the following equation:

$$P(o_t|v_t^{\varsigma}(m),o_{1:t-1}) = b_m(o_t^{\varsigma})\underbrace{\sum_{n_1=1}^{M(1)}\cdots\sum_{n_C=1}^{M(C)}}_{C-1\text{ (except channel }\varsigma)} b_{n_1}(o_t^1)\ldots b_{n_C}(o_t^C) \times P(v_t^1(n_1),\ldots,v_t^C(n_C)|v_t^{\varsigma}(m),o_{1:t-1}). \quad (13)$$

For achieving a recursion, we utilize the following simplification [also necessary to prove (6)]:

$$P(v_t^1(n_1),\ldots,v_t^C(n_C)|o_{1:t-1}) = \prod_{c=1}^{C} P(v_t^c(n_c)|o_{1:t-1}). \quad (14)$$

Then we have

$$P(v_t^1(n_1),\ldots,v_t^C(n_C)|v_t^{\varsigma}(m),o_{1:t-1}) = \frac{\prod_{c=1}^{C} P(v_t^c(n_c)|o_{1:t-1})}{P(v_t^{\varsigma}(m)|o_{1:t-1})} = \prod_{c=1,c\neq\varsigma}^{C} \alpha_{t|t-1}^c(n_c). \quad (15)$$

Hence, using (2), we have

$$\begin{aligned}P(o_t|v_t^{\varsigma}(m),o_{1:t-1}) &= b_m(o_t^{\varsigma})\prod_{c=1,c\neq\varsigma}^{C}\sum_{n_c=1}^{M(c)} b_{n_c}(o_t^c)\alpha_{t|t-1}^c(n_c)\\ &= b_m(o_t^{\varsigma})\prod_{c=1,c\neq\varsigma}^{C} P(o_t^c|o_{1:t-1}).\end{aligned} \quad (16)$$

Accordingly, $\tilde{b}_m^{\varsigma}(o_t)$ can be calculated by

$$\tilde{b}_m^{\varsigma}(o_t) = \frac{b_m(o_t^{\varsigma})}{P(o_t^{\varsigma}|o_{1:t-1})}. \quad (17)$$

Backward parameter is defined in a way that the probability of being in state m of channel c given all the samples of observations, i.e., smoothed probability, could be easily calculated by the product of forward and backward parameters.

$$\alpha_{t|T}^{\varsigma}(m) = \alpha_{t|t-1}^{\varsigma}(m) \times \beta_t^{\varsigma}(m) \quad (18)$$

which leads to

$$\beta_t^{\varsigma}(m) \triangleq \frac{P(v_t^{\varsigma}(m)|o_{1:T})}{P(v_t^{\varsigma}(m)|o_{1:t-1})} = \tilde{b}_m^{\varsigma}(o_t)\frac{P(o_{t+1:T}|v_t^{\varsigma}(m),o_{1:t})}{P(o_{t+1:T}|o_{1:t})}. \quad (19)$$

Authorized licensed use limited to: CSIRO Information Technology Services. Downloaded on September 09,2022 at 00:27:34 UTC from IEEE Xplore. Restrictions apply.

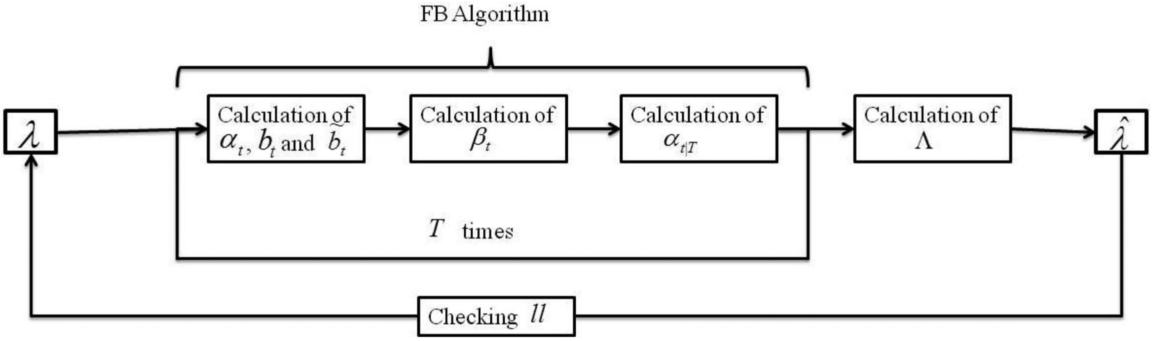

Fig. 2. Block diagram of training procedure. E step: assuming the set of model parameter λ, $\Lambda_t^{c'c}(m', m)$ and $\alpha_{t|T(m)}$ are computed for $m, m' = 1, 2, \ldots, M$ and $t = 1, 2, \ldots, T$ using FB algorithm. M step: calculation of the model parameters using (35) and (37)–(39).

The backward recursion is derived based on the next transition in channel ς as follows:

$$\begin{aligned}\beta_t^{\varsigma}(m) &= \tilde{b}_m^{\varsigma}(o_t)\sum_{n=1}^{M(\varsigma)}\frac{P(o_{t+1:T}, v_{t+1}^{\varsigma}(n)|v_t^{\varsigma}(m), o_{1:t})}{P(o_{t+1:T}|o_{1:t})}\\ &= \tilde{b}_m^{\varsigma}(o_t)\sum_{n=1}^{M(\varsigma)}\frac{P(o_{t+1:T}|v_{t+1}^{\varsigma}(n), v_t^{\varsigma}(m), o_{1:t})}{P(o_{t+1:T}|o_{1:t})}\\ &\quad\times P(v_{t+1}^{\varsigma}(n)|v_t^{\varsigma}(m), o_{1:t}). \end{aligned} \quad (20)$$

Considering (19) and $P(o_{t+1:T}|v_{t+1}^{\varsigma}(n), v_t^{\varsigma}(m), o_{1:t}) = P(o_{t+1:T}|v_{t+1}^{\varsigma}(n), o_{1:t})$, the first term in the summation can be identified as $\beta_{t+1}^{\varsigma}(n)$. The second term can be written as

$$\begin{aligned}&P(v_{t+1}^{\varsigma}(n)|v_t^{\varsigma}(m), o_{1:t})\\ &= \underbrace{\sum_{n_1=1}^{M(1)}..\sum_{n_C=1}^{M(C)}}_{\text{except}\varsigma} P(v_{t+1}^{\varsigma}(n), v_t^1(n_1), \ldots, v_t^C(n_C)|v_t^{\varsigma}(m), o_{1:t})\\ &= \underbrace{\sum_{n_1=1}^{M(1)}..\sum_{n_C=1}^{M(C)}}_{\text{except }\varsigma} P(v_{t+1}^{\varsigma}(n)|v_t^1(n_1), \ldots, v_t^C(n_C), v_t^{\varsigma}(m), o_{1:t})\\ &\quad\times P(v_t^c(n_c), \ldots, v_t^C(n_C)|v_t^{\varsigma}(m), o_{1:t})\\ &= a_{mn}^{\varsigma\varsigma}\prod_{c\neq\varsigma}^{C}\sum_{n_c=1}^{M(c)} a_{n_c n}^{c\varsigma}\alpha_{t|t-1}^c(n_c)\tilde{b}_{n_c}^c(o_t). \end{aligned} \quad (21)$$

Thus, the backward recursion is given by

$$\begin{aligned}\beta_t^{\varsigma}(m) = \tilde{b}_m^{\varsigma}(o_t)\sum_{n=1}^{M(\varsigma)}\{\beta_{t+1}^{\varsigma}(n)a_{mn}^{\varsigma\varsigma}\\ \times\prod_{c=1,c\neq\varsigma}^{C}\sum_{n_c=1}^{M(c)} a_{n_c n}^{c\varsigma}\alpha_{t|t-1}^c(n_c)\tilde{b}_{n_c}^c(o_t)\}. \end{aligned} \quad (22)$$

The initial condition of parameters are $\beta_T^c(m) = \tilde{b}_m^c(o_T)$ and $\alpha_{1|0}^c = \pi_m^c b_m^c(o_1)$.

We resume hereby the three main problems treated in the context of HMM.

Problem 1: Evaluation of the likelihood of an observation sequence, given the model parameters

$$P(o_{1:T}) = P(o_1)\prod_{t=2}^{T} P(o_t|o_{1:t-1}) \quad (23)$$

where

$$P(o_t|o_{1:t-1}) = \prod_{c=1}^{C} P(o_t^c|o_{1:t-1}) \quad (24)$$

the latter can be calculated by (2).

Problem 2: Finding the optimal state m_c^* that generates the observations. We can use the maximum *a posteriori* estimation

$$m_c^*(t) = arg\,\underset{m}{max}\,P(v_t^c(m)|o_{1:T}) = arg\,\underset{m}{max}\,\alpha_{t|T}^c \quad (25)$$

to construct the optimal sequence of channel c: $Q_c^* = \{m_c^*(1), m_c^*(2), \ldots, m_c^*(T)\}$.

Problem 3: Learning of model parameters $\lambda = \{a_{m'm}^{c'c}, \mu_m^c, \sigma_m^c, \pi_m^c\}$ that best fit the observation $\{o_{1:T}\}$. The Baum–Welch algorithm [25] can be applied, using the maximum likelihood (ML) criterion. As a special case of the estimation-maximization (EM) algorithm, the likelihood is estimated using the FB parameters in the E-step while the M-step uses expected counts of transitions and observations to reestimate the parameters λ (see Fig. 2). This procedure is performed iteratively until convergence.

Liporace generalizes the Baum–Welch algorithm to a larger class of distributions of observation probability than just Gaussian distribution [26]. In the following, we present a generalization of the reestimation method inspired from the Liporace paper. The likelihood probability of the observations $\{o_{1:T}\}$ given the model parameters λ can be written as

$$P_\lambda(o_{1:T}) = \sum_{\mathcal{S}} P_\lambda(o_{1:T}, \mathcal{S}) \quad (26)$$

while $P_\lambda(o_{1:T}, \mathcal{S})$ can be written as

$$P_\lambda(o_{1:T}, \mathcal{S}) = \prod_{c=1}^{C}\{\pi_1^c b_1(o_1^c) \times \prod_{t=2}^{T}\prod_{c'=1}^{C} a_{m_{t-1}m_t}^{c'c} b_{m_t}(o_t^c)\} \quad (27)$$

where $\mathcal{S}$ is the set of the state sequences of all channels. The objective is to maximize $P_\lambda(o_{1:T})$ over all parameters λ. The

Authorized licensed use limited to: CSIRO Information Technology Services. Downloaded on September 09,2022 at 00:27:34 UTC from IEEE Xplore. Restrictions apply.

algorithm starts with an initial guess of λ_0, and then updates it to ensure that $P_{\lambda_{k+1}}(o_{1:T}) \geq P_{\lambda_k}(o_{1:T})$ in each iteration. As in the EM algorithm structure, an *auxiliary function* $Q(\lambda, \bar{\lambda})$ is used, defined by (see [26])

$$Q(\lambda, \bar{\lambda}) = \sum_{\mathcal{S}} P_{\lambda}(o_{1:T}, \mathcal{S}) \log(P_{\bar{\lambda}}(o_{1:T}, \mathcal{S})). \tag{28}$$

Extra normalization conditions include

$$\sum_{m=1}^{M(c)} \bar{a}^{c'c}_{m'm} = 1, \text{ and } \sum_{m=1}^{M(c)} \bar{\pi}^c_m = 1 \tag{29}$$

for all channels (c) and states of channel (m'). These constraints are integrated using the Lagrange multiplier method to yield the maximization problem

$$\lambda_{\text{new}} = \underset{\bar{\lambda}}{arg\,max}\, Q(\lambda, \bar{\lambda}) + \sum_{c=1}^{C} \theta_c \left(\sum_{m=1}^{M(c)} \bar{a}^{c'c}_{m'm} - 1 \right) + \sum_{c=1}^{C} \varepsilon_c \left(\sum_{m=1}^{M(c)} \bar{\pi}^c_m - 1 \right) \tag{30}$$

where θ_c, ε_c are the Lagrange parameters.

A description of the reestimation procedure is detailed as follows [26].

A. Reestimation of Transition Matrices

Differentiate (30) with respect to each $\bar{a}^{c'c}_{m'm}$ to obtain

$$\sum_{\mathcal{S}} P_{\lambda}(o_{1:T}, \mathcal{S}) \sum_{t \in \mathfrak{T}^{c'c}_{m'm}} \frac{1}{\bar{a}^{c'c}_{m'm}} - \theta_c = 0 \tag{31}$$

where $\mathfrak{T}^{c'c}_{m'm} = \{t : q^{c'}_{t-1} = S_{m'}, q^c_t = S_m\}$. Interchange the order of summations as in [26] to get

$$\begin{aligned} \bar{a}^{c'c}_{m'm} \theta_c &= \sum_{t=1}^{T} \sum_{\mathcal{S} \in \mathfrak{S}^{c'c}_{m'm}(t)} P(o_{1:T}, \mathcal{S}) \\ &= \sum_{t=1}^{T} P(o_{1:T}, q^{c'}_{t-1} = S_{m'}, q^c_t = S_m) \end{aligned} \tag{32}$$

where $\mathfrak{S}^{c'c}_{m'm}(t) = \{\mathcal{S} : q^{c'}_{t-1} = S_{m'}, q^c_t = S_m\}$ denotes the set of state sequence having state m' at time $t-1$ and state m at time t. Summing over all states of channel c, we obtain θ_c

$$\theta_c = \sum_{k=1}^{T} \sum_{m=1}^{M(c)} P(o_{1:T}, q^{c'}_{t-1} = S_{m'}, q^c_t = S_m). \tag{33}$$

Furthermore, we define $\Lambda^{c'c}_t(m', m) = P(q^{c'}_{t-1} = S_{m'}, q^c_t = S_m | o_{1:T})$ as the conditional smoothed transition probability

$$\begin{aligned} \Lambda^{c'c}_t(m', m) &= P(v^{c'}_{t-1}(m'), v^c_t(m) | o_{1:T}) \\ &= \frac{P(v^{c'}_{t-1}(m'), v^c_t(m), o_{t:T} | o_{1:t-1})}{P(o_{t:T} | o_{1:t-1})} \\ &= \beta^c_t(m) a^{c'c}_{m'm} \alpha^{c'}_{t-1|t-2}(m') \tilde{b}_{m'}(o^{c'}_{t-1}). \end{aligned} \tag{34}$$

It is then straightforward to achieve the reestimation for the transition probability

$$\bar{a}^{c'c}_{m'm} = \frac{\sum_{t=1}^{T} \Lambda^{c'c}_t(m', m)}{\sum_{t=1}^{T} \sum_{m''=1}^{M(c)} \Lambda^{c'c}_t(m', m'')}. \tag{35}$$

B. Reestimation of $\bar{\pi}^c_m$

Adding the appropriate constraint for initial probability of being in state m to the auxiliary function, we have

$$\frac{\partial}{\partial \bar{\pi}^c_m} \{Q(\lambda, \bar{\lambda}) - \varepsilon_c (\Sigma_{m=1}^{M(c)} \bar{\pi}^c_m - 1)\} = 0 \tag{36}$$

$$\sum_{\mathcal{S}} P_{\lambda}(o_{1:T}, \mathcal{S}) \frac{1}{\bar{\pi}^c_m} - \varepsilon_c = 0$$

$$\bar{\pi}^c_m \varepsilon_c = P(o_{1:T}, q^c_1 = S_m)$$

$$\varepsilon_c = \sum_{m=1}^{M(c)} P(o_{1:T}, q^c_1 = S_m).$$

Thus, using (18), the reestimation of initial probability is straightforward

$$\bar{\pi}^c_m = \frac{\alpha^c_{1|T}(m)}{\sum_{m'=1}^{M(c)} \alpha^c_{1|T}(m')}. \tag{37}$$

C. Reestimation of $\bar{\mu}^c_m$ and $\bar{\sigma}^c_m$

As previously mentioned, the density functions of observations $b_m(o^c_t)$ are assumed to be Gaussian functions. Applying the derivation on auxiliary function with respect to the components of $\bar{\mu}^c_m$, we can obtain

$$\bar{\mu}^c_m = \frac{\sum_{t=1}^{T} \alpha^c_{t|T}(m) o^c_t}{\sum_{t=1}^{T} \sum_{m'=1}^{M(c)} \alpha^c_{t|T}(m')} \tag{38}$$

$$\bar{\sigma}^c_m = \frac{\sum_{t=1}^{T} \alpha^c_{t|T}(m)(o^c_t - \bar{\mu}^c_m)^2}{\sum_{t=1}^{T} \sum_{m'=1}^{M(c)} \alpha^c_{t|T}(m')}. \tag{39}$$

III. Evaluation Method

In this section, we present the method applied in this study to evaluate the performance of the proposed CHMM framework, in the contexts of classification and online detection, using both simulated and real datasets. Quantitative performance evaluation is based on common performance metrics and accomplished in two steps: 1) training the CHMM models using a train dataset and 2) performing classification or online detection on the test dataset. Each channel represents a 1-D observation. To initialize states parameters, the k-means clustering method is first applied to the range of observations amplitudes in training data. Each cluster is represented by a Gaussian distribution to characterize the observation probability $b_m(o^c_t)$ while the number of clusters is equal to the number of states.

In the following, we describe the proposed approach for applying the CHMM for the classification of simulated time-series. Then, we explain how the proposed framework can be applied

Authorized licensed use limited to: CSIRO Information Technology Services. Downloaded on September 09,2022 at 00:27:34 UTC from IEEE Xplore. Restrictions apply.

for online detection. Note that the optimum number of states in Markovian models are found by Bayesian information criterion method.

A. Classification

In previous works [13], the problem of classifying a time-series into one of K classes has been addressed by defining a set of K competing models $(M_1, \ldots, M_K)$, for which a learning dataset corresponding to each class $(L_1, \ldots, L_K)$ is used to estimate the model parameters of each model $(\lambda_1, \ldots, \lambda_K)$. Then, in the test phase, each time-series of the test dataset (O) is analyzed by calculating the log-likelihood using each model: $\log P(O \mid Q^*, \lambda_k), j \in \{1, 2, \ldots, K\}$, where Q^* represents the optimal state sequence. The classification result is obtained by choosing the class corresponding to the model presenting the maximum log-likelihood.

A similar approach is applied in this paper for the proposed CHMM. However, in order to cope with the multichannel nature of the CHMM, the overall log-likelihood for a CHMM corresponding to class k will be obtained by summing the log-likelihoods in all channels with their optimal state sequence Q_c^*

$$ll_k = \sum_{c=1}^{C} \log\{P(o^c|Q_c^*, \lambda_k)\}. \quad (40)$$

B. Online Detection

The classification application can also be extended to online detection, as in [13]. The data can be divided into overlapping moving windows, and the classification procedure is applied to each window. One of the K classes (i.e., class k) is defined to represent the event of interest. The difference of log-likelihood of class k from other classes can be obtained

$$ll_{\text{total}}^{kj}(t) = ll_k(t) - ll_j(t) \quad (41)$$

where $j \in \{1, 2, \ldots, K\} - \{k\}$. In case of multiple channels as in our proposed CHMM, the above equation can be rewritten as

$$ll_{\text{total}}^{kj}(t) = \sum_{c=1}^{C} (ll_k^c(t) - ll_j^c(t)). \quad (42)$$

An event corresponding to class k takes place in a window containing time t if the following condition is satisfied:

$$ll_{\text{total}}^{kj}(t) > \delta_{kj} \quad (43)$$

where δ_{kj} is a constant threshold that should be optimized.

C. Performance Evaluation

We evaluate the performance of the classifiers and the detectors using the most common metrics found in the literature: the receiver operating characteristic (ROC) curve and its area under curve (AUC) defined by sensitivity, and specificity of classification. To express how successfully a detector recognizes events without missing them, sensitivity $[\text{SEN} = \text{TP}/(\text{TP} + \text{FN})]$ is used. Likewise, specificity $[\text{SPC} = \text{TN}/(\text{TN} + \text{FP})]$ measures how exclusively it does not detect a wrong event. TP, FP, TN and FN denote the number of true positives, false positives, true negatives and false negatives, respectively. False positive rate error can also be defined as $1 - \text{SPC}$. In a classification task, $AC = (\text{TP} + \text{TN})/(N + P)$, where $N = \text{TN} + \text{FN}$ and $P = \text{TP} + \text{FP}$, is also calculated to evaluate the accuracy of the method. Moreover, time delay is another crucial metric for the overall system performance evaluation in the case of early detection of a desired event, and is defined as the difference between the detected onset and the annotated onset of an event.

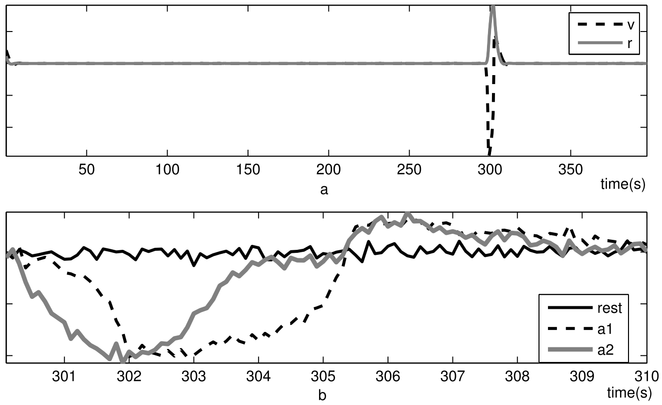

Fig. 3. (a) Simulated signals obtained from the FitzHugh–Nagumo model (state variables v and r) with a disturbance applied in 300–305 (s) without noise. (b) Focusing on v dimension with 5-dB additive noise, in the rest condition and during activations with different dynamics (a_1 and a_2). Note that different dynamics are obtained when using parameters a_1 and a_2, while the amplitude of the responses are similar.

An ROC is traced using different threshold values in (43). The point with optimum detection performance and related threshold are chosen using a criterion called perfect detection (PD) defined as the maximum product of sensitivity and specificity.

All the methods are executed five times with different records in the training and test sets, in order to cross-validate the results. Therefore, the mean and variance over all executions are reported.

D. Evaluation Datasets

1) Simulated Data: The simulated data is generated with the FitzHugh–Nagumo model, defined by the following differential equations:

$$\frac{dv}{dt} = 3(v - \frac{1}{3}v^3 + r + I)$$
$$\frac{dr}{dt} = -\frac{1}{3}(v - a + 0.8r) \quad (44)$$

where the variables r and v are set to their "rest" values (fixed point) and disturbances are injected to the system by changing the value of I from 0 to 1. The dynamics of the system depend on the value of parameter a, which is assumed in this test to be a random variable with a uniform probability density function $a_1 \sim \mathcal{U}(0.58, 0.62)$ for the first class and $a_2 \sim \mathcal{U}(0.78, 0.82)$ for the second class. The impact of changing parameter a in the simulated data is illustrated in Fig. 3. The resemblance between the time series in class 1 and class 2 makes the classification a difficult issue. An appropriate classifier for such problem needs to correctly differentiate the dynamics of these time series and not only their instantaneous amplitudes. In order to evaluate

Authorized licensed use limited to: CSIRO Information Technology Services. Downloaded on September 09,2022 at 00:27:34 UTC from IEEE Xplore. Restrictions apply.

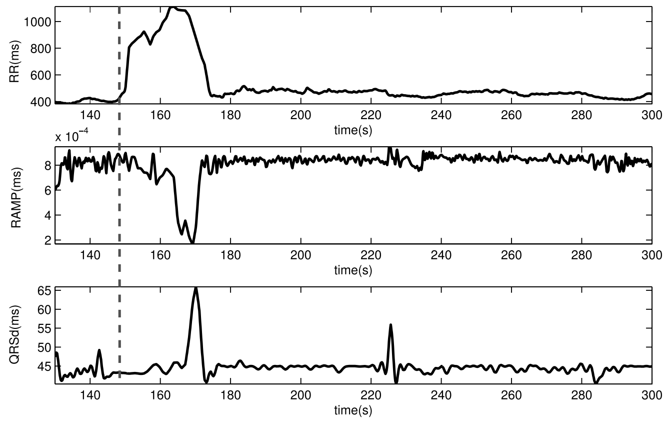

Fig. 4. Example of time series extracted from test real data. The annotated onset of the AB event is shown by dashed grey line.

the proposed approach, 200 sequences of 400 s duration at a sampling frequency of 10 Hz are generated with a disturbance introduced during 300–305 s [see Fig. 3(a)]. 40 segments of 300–310 s are used for training [cf., Fig. 3(b)] and the rest of them for evaluation.

Concerning online detection, train data is similar to that used for the classification case. However, the test data contain the whole 400-s-duration signals and are processed with 10-s sample by sample sliding window. Finally, a white Gaussian noise was added to achieve SNR value of 5 dB to all the database. Moreover, since the range of signal amplitude variations in the rest condition is lower than in dynamic conditions, the number of states of the rest class is less.

2) Real Data: For real data analysis, we use a database acquired during our previous works and consisting of 236, one-lead ECG segments from 32 preterm infants, hospitalized in the NICU at the University Hospital of Rennes, France [14]. ECG signals were acquired at 400 Hz. This observational study was approved by the ethical committee of the University Hospital of Rennes and a written consent was obtained from the parents of each infant. All data were anonymized at acquisition.

Each acquired ECG segment was denoised by a combination of low-pass and notch filters for discarding the baseline and the noise of 50 Hz, respectively. Individual beats for each segment were detected using the Pan and Tompkins algorithm [27]. Finally, three ECG features were extracted in a beat-to-beat basis, by applying a wavelet-based beat delineator described in detail in [13]. The first obvious feature is the time interval between two consecutive beats (RR interval), representing the cardiac period, or the inverse of the heart rate, since a bradycardia event is usually observed as a marked increase of cardiac period. The time series representing the evolution of these cardiac periods are called the RR series. Furthermore, the amplitude of R-wave on the ECG (RAMP) has been considered since it is modulated by the respiratory activity [28]. Some works have also reported an increase in the duration of the QRS complex (QRSd) preceding episodes of AB, so this feature has also been extracted [29]. The total set of features that will be used as observations to evaluate the CHMM framework are thus: the RR interval, R-wave amplitude (RAMP) and QRS duration (QRSd). The

TABLE I
COMPARISON OF THE CROSS-VALIDATION RESULTS CORRESPONDING TO MARKOVIAN METHODS FOR OPTIMAL STATE NUMBER FOR DYNAMICS CLASSIFICATION

Method	Class	♯ States	SEN(%)	SPC(%)	AC(%)
	a_1	7	55.63 ± 37.30	91.56 ± 18.69	79.58 ± 10.13
HMM	a_2	7	83.13 ± 37.39	77.50 ± 18.63	79.38 ± 9.96
(v)	$rest$	3	99.38 ± 0.77	100 ± 0	99.79 ± 0.26
	a_1	3	86.25 ± 5.23	90.63 ± 2.30	89.17 ± 1.59
HMM	a_2	3	81.25 ± 4.59	92.94 ± 2.52	89.04 ± 1.55
(v/r)	$rest$	2	99.63 ± 0.34	100 ± 0	99.88 ± 0.11
	a_1	7	67.00 ± 12.67	84.50 ± 3.81	78.67 ± 4.74
HSMM	a_2	7	69.00 ± 7.62	80.50 ± 6.82	76.67 ± 4.64
(v)	$rest$	3	94.00 ± 3.79	100 ± 0	98.00 ± 1.26
	a_1	3	100 ± 0	74.44 ± 1.26	82.96 ± 0.84
HSMM	a_2	3	48.88 ± 2.52	100 ± 0	82.96 ± 0.84
(v/r)	$rest$	2	100 ± 0	100 ± 0	100 ± 0
	a_1	5	89.56 ± 31.49	96.75 ± 8.65	94.35 ± 11.26
CHMM	a_2	5	99.69 ± 0.53	93.25 ± 17.80	95.40 ± 11.85
(v/r)	$rest$	3	90.75 ± 16.99	100 ± 0	96.92 ± 5.66
our	a_1	5	96.04 ± 9.69	97.19 ± 4.04	96.81 ± 3.43
CHMM	a_2	5	93.98 ± 6.26	95.38 ± 4.49	95.58 ± 4.00
(v/r)	$rest$	3	95.13 ± 2.98	100 ± 0	98.38 ± 0.99

sampling frequency of these extracted signals is transformed to 10 Hz, using well-known interpolation techniques. Fig. 4 depicts an example of these features before, during and after an AB episode.

The objective here is to detect, with the maximum detection performance and the lowest detection delay, all events of AB of the database. Two datasets are constructed for the learning phase, consisting of segments of 7 s duration: LS1: composed of 30 segments taken randomly and beginning with a bradycardia event. LS2: consisting of 30 segments taken randomly from the normal parts of the series (without any AB event). The length of these segments (7 s) corresponds to the average time measured from the beginning of the bradycardia to the peak RR value within the bradycardia episodes [9]. Two models are trained, corresponding respectively to bradycardia and normal segments. Then, the evaluation of the trained models for detecting the onset of bradycardia is applied with a sliding window of size $T = 7$ s. The test dataset includes 40 sets of RR-RAMP-QRSd time-series including mostly normal activity and one or more AB events.

IV. RESULTS

In this section, we report both the classification results on simulated data and the results of detection of disturbance in simulated data and bradycardia arrhythmia in preterm infants. Comparisons are made with respect to existing Markovian-based models such as HMM [12], HSMM [24] and the CHMM proposed by Rezek *et al.* [21].

A. Classification of Dynamics in Simulated Data

A classification is performed on simulated data, by defining three classes: a_1, a_2 and a rest condition. We further assume that the number of states of the two competing models is equal and

Authorized licensed use limited to: CSIRO Information Technology Services. Downloaded on September 09,2022 at 00:27:34 UTC from IEEE Xplore. Restrictions apply.

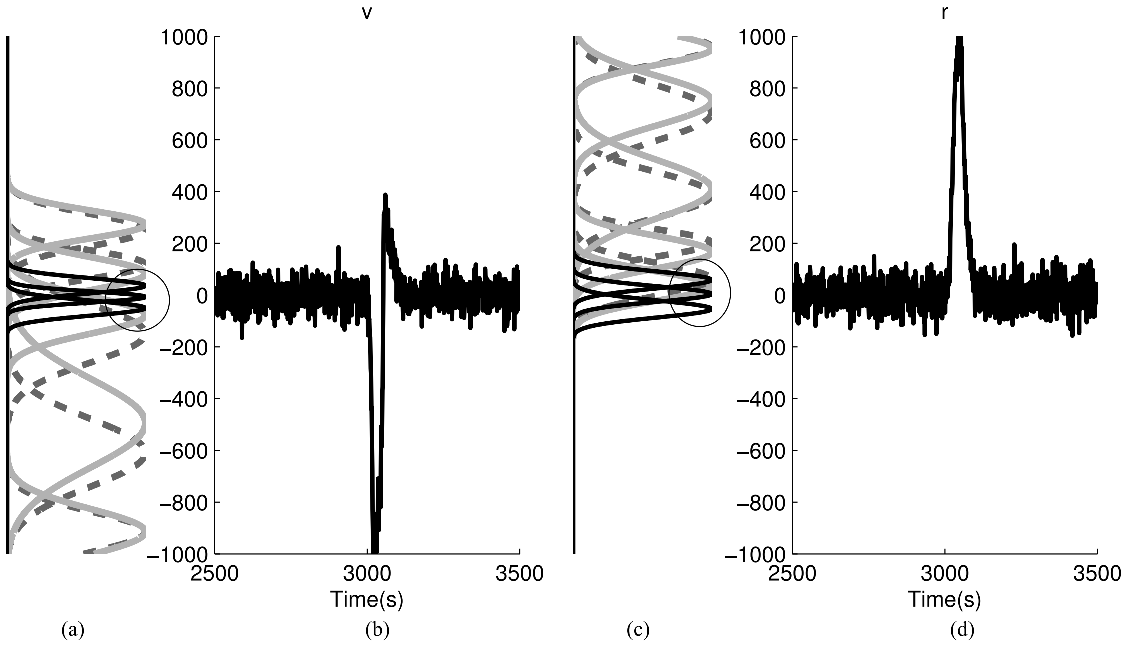

Fig. 5. Examples of simulated responses for state variables of the FitzHugh–Nagumo model (observations) (b) v and (d) r, and the fitted Gaussian pdf $b_m(o_t^c)$ obtained for each state [(a) and (c), respectively] after the training phase. m different Gaussians can be observed in each case, each one characterizing one state of the corresponding model. In (a) and (c) black, light grey and dashed dark grey kernels belong to states of rest, a_1 and a_2 models, respectively. Circles show the source of error around rest conditions.

is more than that of the model for learning the rest condition. The SEN, SPC and AC metrics are calculated for various numbers of states to find its optimum. Classification performance is presented in Table I, including the results of univariate observation of HMM using just dimension v. All results are reported with optimal state numbers. According to this table, coupling approaches are more efficient in distinguishing the two dynamics from each other compared with HMMs. Although our proposed CHMM apparently shows superior performance in terms of SEN and AC to separate dynamics related to a_1 and a_2, it fails to detect dynamics from rest condition accurately. Fig. 5 illustrates the source of this problem. This figure depicts two dimensions of an observation, as well as their fitted Gaussian probability density functions (pdf), each of which corresponds to a state m of channel c of a model ($b_m(o_t^c)$). Focusing on dark circles in the figure, we can observe that for a few samples of the rest condition, the probability of observation generated by one of the dynamic models is more than its probability in rest model. This problem can be suppressed by increasing the number of states in rest model, which leads to slow execution rate. So we accept some errors in classifying rest class in order to separate dynamics from each other accurately and rapidly.

B. Detection of Dynamics in Simulated Data

The detection of dynamic a_1 is accomplished with first detecting the disturbance from rest condition using a threshold on $ll_{\text{total}}^{\{a_1\}\{\text{rest}\}}$. If a disturbance is detected, then using second threshold on $ll_{\text{total}}^{\{a_1\}\{a2\}}$, we are able to determine which dynamic (a_1 or a_2) has generated the observation. The disturbance which is generated with a_2 is not analyzed since it does not provide any additional information. Similar to classification, we assume the same number of states for dynamic models and the model of rest condition has less states. The results corresponding to the optimal number of states are reported in Table II where all the metrics are related to the PD of the corresponding ROC. An interesting result happens while using more than one dimension of observation, since multivariate HMM achieves better SEN and SPC than univariate HMM. Moreover, coupling the dimensions leads to better results than even multivariate HMM because in CHMM, each dimension is processed by a Markov chain and the coupling between them can extract the information more efficiently than using these dimensions in multivariate way in a single HMM. The SEN and SPC of our proposed model reached 94.32% and 99.34%, respectively, and the time delay is 0.98 ± 0.16 s which are superior than the other methods.

C. Detection of AB in Real Data

For real data analysis, we construct LS1 and LS2 training data for obtaining the parameters of the models corresponding to normal and bradycardia episodes and calculate the metrics on 40 test data. This process is repeated five times for cross-validation with different, randomly chosen records, on the training and evaluation datasets. However, prior to this procedure, since the RAMP data are modulated by respiration activities [29] and the effect of apnea will first appear in the RAMP feature, we apply a synchronization time delay (τ), whose possible values are observed around 4.5 s. The exact values of τ are also optimized and reported in Table III and in Fig. 6. This study is performed using RR and RAMP features while considering five states for both models (bradycardia and normal). The best results are achieved with $\tau = 4$ s which corresponds to typical values described in clinical studies [2].

Authorized licensed use limited to: CSIRO Information Technology Services. Downloaded on September 09,2022 at 00:27:34 UTC from IEEE Xplore. Restrictions apply.

TABLE II
COMPARISON OF THE CROSS-VALIDATION RESULTS CORRESPONDING TO MARKOVIAN METHODS FOR OPTIMAL STATE NUMBER FOR DYNAMIC a_1 DETECTION

Method	♯ States	SEN(%)	SPC(%)	Mean Delay(s)	Std Delay(s)
HMM (v)	2-5-5	61.38 ± 2.65	89.73 ± 2.41	0.40 ± 0.02	1.01 ± 0.41
HMM (v/r)	2-4-4	86.61 ± 6.56	85.31 ± 3.78	0.24 ± 0.21	1.05 ± 0.19
HSMM (v)	3-5-5	90.78 ± 2.39	98.81 ± 0.36	2.84 ± 0.71	0.11 ± 0.04
HSMM (v/r)	3-5-5	94.99 ± 0.02	99.45 ± 0.01	1.59 ± 0.01	0.07 ± 0.01
CHMM (v/r)	2-4-4	94.38 ± 0.09	99.42 ± 0.01	1.78 ± 0.03	0.07 ± 0.04
Our CHMM (v/r)	2-5-5	94.49 ± 0.50	99.34 ± 0.12	0.98 ± 0.21	0.16 ± 0.15

TABLE III
SUMMARY OF THE RESULTS OF PROPOSED CHMM USING VARIOUS SYNCHRONIZATION TIME DELAYS FOR RAMP

Synchronization Delay(s)	SEN(%)	SPC(%)	Mean Delay(s)
0	86.44	96.38	2.63 ± 2.50
0.5	80.53	89.89	4.52 ± 4.57
1	86.69	86.99	1.21 ± 3.62
1.5	76.66	65.29	− 4.67 ± 2.45
2	84.71	96.55	1.84 ± 2.38
2.5	92.41	92.60	0.25 ± 2.85
3	92.82	91.67	0.95 ± 2.99
3.5	89.34	91.25	1.12 ± 3.55
4	93.45	97.25	− 0.15 ± 2.09
4.5	92.38	85.84	− 0.59 ± 5.39

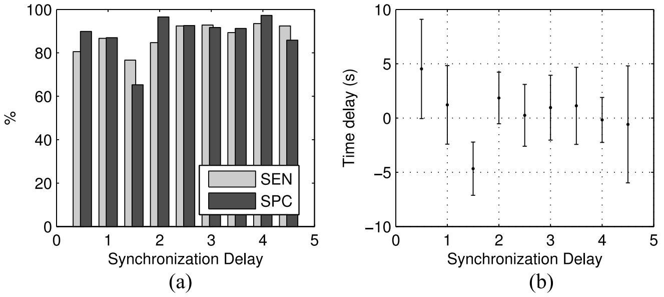

Fig. 6. (a) SEN and SPC obtained by various synchronization time delay. (b) Corresponding mean and variance of the delayed time to detect the bradycardia.

The performance for the bradycardia detection and the detection delays are detailed in Table IV. The comparison of the proposed CHMM with the other methods is reported in various combinations of the three features: (RR-QRSd), (RR-RAMP) and (RR-RAMP-QRSd). In the cross-validation approach, the reported metrics of this table are related to the PD point of the ROC curves obtained by averaging the SENs and SPCs achieved by an identical threshold. Furthermore, three other metrics are also calculated: 1) the distance to PD defined as $\sqrt{(1-\mathrm{SEN})^2+(1-\mathrm{SP}C)^2}$, 2) AUC of the averaged ROC curve and 3) positive windows (PW) which is defined as the ratio of the number of detections occurring after the annotated onset, over the number of detected AB episodes, for all executions. PW illustrates the ability of an algorithm in AB prediction. Lower values of PW (below 50%) is an indication of the detection performance that can be reached by a given detection method, when configured optimally (optimal threshold). Similarly, lower values of this metric indicates a better performance.

The best results of our proposed CHMM in terms of AUC and time delay criteria are achieved by (RR-RAMP-QRSd) combination, for which the average SEN and SPC reach 95.74% and 91.88%, respectively. Our method has an average time delay of -0.59 s with average standard deviation of 2.79 s. Its PW shows that more than the half of the detections occur earlier than the clinically-annotated onset of bradycardia which implies that AB events are better predicted with the proposed method. As illustrated in Fig. 7, the coupling of all the available features provides the higher detection rates. Moreover, our proposed method obtains nearly better results in the three-channel configuration than the two-channels, since we can observe the improvement in all metrics except SPC. The optimum number of states per model is also reported in Table IV.

V. DISCUSSION AND CONCLUSION

A novel Bayesian framework is proposed for modeling interdependences of temporal signal features as a generalization of the HMM in order to AB detection. The model attributes a channel to each 1-D signal feature and the state transitions in each channel are controlled by the current states in all channels. We proposed solutions to the three common problems of HMM based on the recursions of the novel forward and backward variables which are reproduced for each channel using the conditional probabilities. Accordingly, the reestimation equations are modified based on the proposed FB algorithm as well as the ML estimator for the model parameters. To train the model parameters, the variables $\Lambda_t^{c'c}(m',m)$ and $\alpha_{t|T}^c$ are defined using smoothed probabilities and are also useful in finding the optimal state sequences.

Moreover, the evaluation is also performed on the online detection of AB episodes in preterm infants. The results of the proposed CHMM are promising and provide accurate bradycardia detection, with an average sensitivity of 95.74% and specificity of 91.88% using three features extracted from the observed ECG (RR-RAMP-QRSd). This approach also provides the best performance in terms of detection delays with -0.59 s, i.e., our CHMM predicts the occurrence of AB events, with respect to the clinicians' annotations. Note also that these performances are achieved without employing large numbers of states and complex state transition networks.

The proposed CHMM framework is affected by the initial values of the state vector and the density function of observations. Hence, an automated k-means clustering procedure for reliable initialization is proposed. The recursive structure of the frame-

Authorized licensed use limited to: CSIRO Information Technology Services. Downloaded on September 09,2022 at 00:27:34 UTC from IEEE Xplore. Restrictions apply.

TABLE IV
SUMMARY OF THE CROSS-VALIDATION RESULTS OF PROPOSED CHMM COMPARED WITH OTHER FRAMEWORKS USING DIFFERENT CONFIGURATIONS OF FEATURES

Features	Method	♯ States	SEN(%)	SPC(%)	Mean Delay(s)	Std Delay(s)	PW(%)	AUC	Distance to PD
RR	HMM	6	85.82 ± 6.81	88.34 ± 2.21	0.77 ± 0.97	2.14 ± 0.98	64.30	0.94	0.18
RAMP	HSMM	3	93.74 ± 0.40	91.86 ± 0.23	0.56 ± 0.07	1.49 ± 0.03	60.00	0.95	0.10
	CHMM	4	87.59 ± 0.18	88.05 ± 0.24	1.10 ± 0.03	1.67 ± 0.03	69.23	0.94	0.18
	Our CHMM	5	87.64 ± 0.94	92.13 ± 6.44	0.59 ± 0.01	3.11 ± 1.94	47.29	0.95	0.15
RR	HMM	2	82.28 ± 16.87	85.32 ± 3.78	1.81 ± 0.84	4.10 ± 4.09	73.72	0.89	0.23
QRSd	HSMM	5	84.74 ± 0.36	91.33 ± 0.18	2.93 ± 0.03	3.08 ± 0.03	87.50	0.93	0.18
	CHMM	2	84.99 ± 0.26	94.80 ± 0.51	3.43 ± 0.01	2.89 ± 0.03	90.00	0.95	0.17
	Our CHMM	5	87.04 ± 3.78	95.31 ± 1.36	2.51 ± 0.93	3.02 ± 0.18	83.22	0.93	0.14
RR	HMM	2	91.02 ± 0.64	93.47 ± 0.08	6.55 ± 0.69	2.29 ± 0.79	63.13	0.97	0.11
RAMP	HSMM	7	86.83 ± 25.62	93.77 ± 4.44	1.10 ± 2.74	1.64 ± 1.18	44.10	0.96	0.16
QRSd	CHMM	3	84.42 ± 0.27	92.21 ± 0.32	7.27 ± 0.02	4.26 ± 0.02	60.53	0.94	0.18
	Our CHMM	2	95.74 ± 0.82	91.88 ± 0.31	-0.59 ± 0.21	2.79 ± 0.06	44.23	0.98	0.09

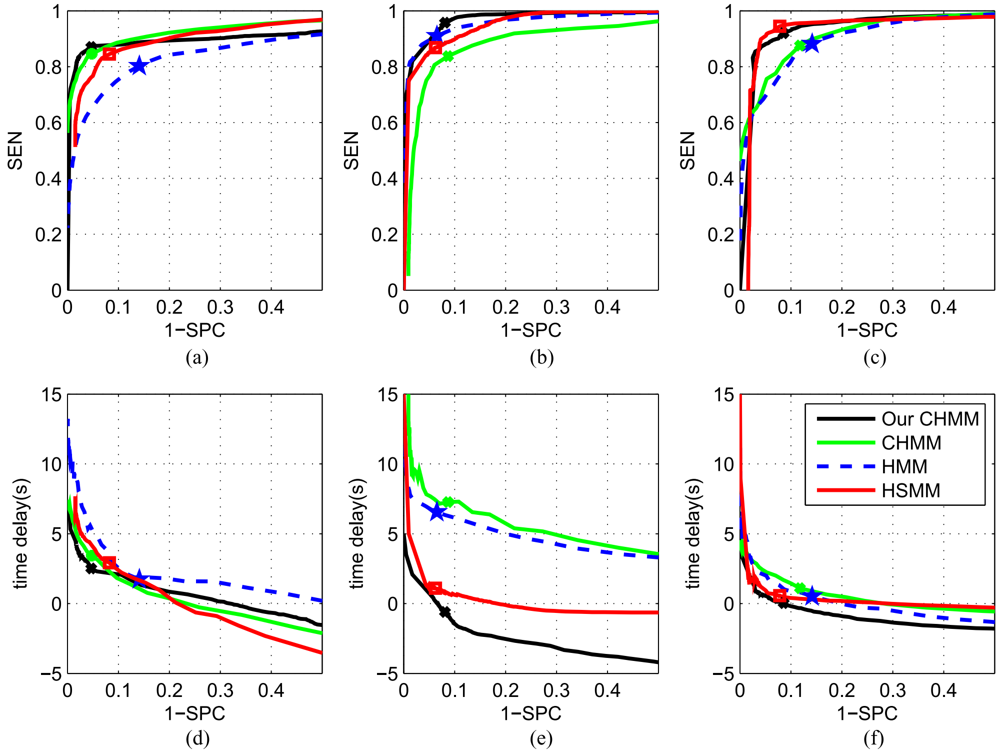

Fig. 7. Comparing the performance of our CHMM for AB detection with other frameworks. (a)–(c) ROC curves. (d)–(f) Time delay curves versus error. (a), (d): RR and QRSd. (b), (e): RR, QRSd and RAMP. (c), (f): RR and RAMP.

work coming from the nature of standard HMM and sample by sample windowing process makes the algorithm computationally tractable, and hence, suitable for real-time applications. Moreover, the computational cost of our proposed FB algorithm is $O(TM^C)$ to be compared with that of the CHMM proposed by Rezek ($O(TM^{2C})$).

The main contributions of this paper can be summarized as: 1) the introduction of a novel framework for CHMM as a generalization of HMM to address the analysis of N-dimensional coupled signal features, 2) the derivation of the FB variables and solutions to the standard problems of the Markovian models according to the proposed framework, and 3) the application of the proposed framework to AB detection, based on the log-likelihoods of competing models.

Future works include incorporating a resting time for the states into the coupled model to provide a more accurate representation of the dynamics of the observed time-series. Since the proposed CHMM approach is completely generic, we plan to apply it to other clinical applications such as adult apnea event detection, using specific datasets coupling different cardiorespiratory signals.

APPENDIX

For better understanding, consider Fig. 1, which depicts a minor loop of the two coupled chains at times $t-1$, t and $t+1$. We simplify the predicted joint probability of states $P(v_t^1(m_1), v_t^2(m_2)|o_{1:t-1})$ by $P(v_t^1(m_1)|o_{1:t-1}) \times P(v_t^2(m_2)$

Authorized licensed use limited to: CSIRO Information Technology Services. Downloaded on September 09,2022 at 00:27:34 UTC from IEEE Xplore. Restrictions apply.

$|o_{1:t-1})$, i.e., $v_t^1(m_1)$ and $v_t^2(m_2)$ are conditionally independent. We generalize it to more than two channels as follows:

$$P(v_t^1(m_1), \ldots, v_t^C(m_C)|o_{1:t-1}) = \prod_{c=1}^{C} P(v_t^c(m_c)|o_{1:t-1}). \tag{45}$$

This simplification helps us to prove other statements like (6) and (10). The following proofs are presented in two channels for simplicity.

Appendix A
Joint Probability Estimation of Channels Observations

During the parameters definition and for obtaining more compact and simpler relation for forward and backward parameters, we need to estimate the joint probability of observations $P(o_t^1, o_t^2|o_{1:t-1})$ [see (10)]. One estimation is to use mean-field assumption of joint probability of the two observations, i.e., the joint probability is calculated by the product of the probability of each variable, which means they are conditionally independent [30].

$$P(o_t^1, o_t^2|o_{1:t-1}) = P(o_t^1|o_{1:t-1}) \times P(o_t^2|o_{1:t-1}). \tag{46}$$

Other estimations are uniform and weighted. In uniform case, the joint probability $P(o_t^1, o_t^2|o_{1:t-1})$ is calculated by the summation of the conditional probability of each observation, $P(o_t^1|o_{1:t-1}) + P(o_t^2|o_{1:t-1})$, which seems to be incorrect from the weighted point of view as follows.

Weighted case is a more general form. The joint probability of the observations of channels can be expressed as follows [30]:

$$P(o_t^1, o_t^2|o_{1:t-1}) = P(o_t^1|o_t^2, o_{1:t-1})P(o_t^1|o_{1:t-1})$$
$$P(o_t^1, o_t^2|o_{1:t-1}) = P(o_t^2|o_t^1, o_{1:t-1})P(o_t^2|o_{1:t-1}).$$

So, it is correct to have

$$\begin{aligned} P(o_t^1, o_t^2|o_{1:t-1}) = \; & [0.5P(o_t^1|o_t^2, o_{1:t-1})]P(o_t^1|o_{1:t-1}) \\ & +[0.5P(o_t^2|o_t^1, o_{1:t-1})]P(o_t^2|o_{1:t-1}) \\ = \; & w_1 P(o_t^1|o_{1:t-1}) + w_2 P(o_t^2|o_{1:t-1}). \end{aligned}$$

As we know, it is difficult to estimate $P(o_t^1|o_t^2, o_{1:t-1})$ or $P(o_t^2|o_t^1, o_{1:t-1})$. Also, mean-field assumption for the observations seems to be correct, since although they are correlated, they give us no information about each other given the previous observations and it is widely used in other works (see [17] and [22]). This is what we have used in definition of $\tilde{b}$.

Moreover, using (14), we can prove the mean-field assumption as follows:

$$\begin{aligned} P(o_t|o_{1:t-1}) = \; & \sum_{m_1}\sum_{m_2} P(o_t, v_t^1(m_1), v_t^2(m_2)|o_{1:t-1}) \\ = \; & \sum_{m_1}\sum_{m_2} P(o_t|v_t^1(m_1), v_t^2(m_2), o_{1:t-1}) \\ & \times P(v_t^1(m_1), v_t^2(m_2)|o_{1:t-1}). \end{aligned} \tag{47}$$

Then we have

$$\begin{aligned} P(o_t|o_{1:t-1}) = \; & \sum_{m_1}\sum_{m_2}\prod_{c=1}^{2} P(o_t^c|v_t^c(m_c), o_{1:t-1}) \\ & \times \prod_{c=1}^{2} P(v_t^c(m_c)|o_{1:t-1}) \\ = \; & \prod_{c=1}^{2}\sum_{m_c} P(o_t^c|v_t^c(m_c), o_{1:t-1}) \\ & \times P(v_t^c(m_c)|o_{1:t-1}) \\ = \; & \prod_{c=1}^{2}\sum_{m_c} P(o_t^c, v_t^c(m_c)|o_{1:t-1}) \\ = \; & \prod_{c=1}^{2} P(o_t^c|o_{1:t-1}) \end{aligned} \tag{48}$$

where it can be generalized for $C > 2$.

Appendix B
Filtered Joint Probability of Forward Parameters

In this part a prove for (6), the filtered joint probability of states given the observations, is presented by using (45) and (46). Therefore, we start with adding the observations at time t to the states

$$\begin{aligned} P(v_t^1, v_t^2|o_{1:t}) &= \frac{P(v_t^1, v_t^2, o_t|o_{1:t-1})}{P(o_t|o_{1:t-1})} \\ &= \frac{P(o_t^1|v_t^1)P(o_t^2|v_t^2)P(v_t^1, v_t^2|o_{1:t-1})}{P(o_t|o_{1:t-1})}. \end{aligned} \tag{49}$$

On the other hand, we can write

$$\begin{aligned} P(v_t^1|o_{1:t})P(v_t^2|o_{1:t}) &= \frac{P(v_t^1, o_t|o_{1:t-1})P(v_t^2, o_t|o_{1:t-1})}{P(o_t|o_{1:t-1})^2} \\ &= \frac{P(o_t^1|v_t^1)P(v_t^1|o_{1:t-1})\sum_{v_t^2} P(o_t^2|v_t^2)P(v_t^2|o_{1:t-1})}{P(o_t|o_{1:t-1})} \\ &\times \frac{P(o_t^2|v_t^2)P(v_t^2|o_{1:t-1})\sum_{v_t^1} P(o_t^1|v_t^1)P(v_t^1|o_{1:t-1})}{P(o_t|o_{1:t-1})} \end{aligned} \tag{50}$$

with some simple manipulation, we can derive the term $P(v_t^1, v_t^2|o_{1:t})$ and simplify the statement above as follows:

$$\begin{aligned} P(v_t^1|o_{1:t})P(v_t^2|o_{1:t}) &= P(v_t^1, v_t^2|o_{1:t}) \\ &\times \frac{\sum_{v_t^1}\sum_{v_t^2} P(o_t^1, v_t^1|o_{1:t-1})P(o_t^2, v_t^2|o_{1:t-1})}{P(o_t|o_{1:t-1})} \\ &= P(v_t^1, v_t^2|o_{1:t}) \times \frac{P(o_t^1|o_{1:t-1})P(o_t^2|o_{1:t-1})}{P(o_t|o_{1:t-1})} \end{aligned} \tag{51}$$

Authorized licensed use limited to: CSIRO Information Technology Services. Downloaded on September 09,2022 at 00:27:34 UTC from IEEE Xplore. Restrictions apply.

where the second term is equal to 1 based on (46). Thus, we have

$$P(v_t^1|o_{1:t})P(v_t^2|o_{1:t}) = P(v_t^1, v_t^2|o_{1:t}). \quad (52)$$

References

[1] F. Pillekamp, C. Hermann, T. Keller, A. von Gontard, A. Kribs, and B. Roth, "Factors influencing apnea and bradycardia of prematurity—Implications for neurodevelopment," *Neonatology*, vol. 91, no. 3, pp. 155–161, 2006.

[2] C. F. Poets, "Apnea of prematurity: What can observational studies tell us about pathophysiology?," *Sleep Med.*, vol. 11, no. 7, pp. 701–707, 2010.

[3] M. O. Mendez, D. D. Ruini, O. P. Villantieri, M. Matteucci, T. Penzel, S. Cerutti, and A. M. Bianchi, "Detection of sleep apnea from surface ecg based on features extracted by an autoregressive model," in *Proc. 29th Annu. Int. Conf. IEEE Eng. Med. Biol. Soc.*, 2007, pp. 6105–6108.

[4] T. Penzel, J. McNames, P. De Chazal, B. Raymond, A. Murray, and G. Moody, "Systematic comparison of different algorithms for apnoea detection based on electrocardiogram recordings," *Med. Biol. Eng. Comput.*, vol. 40, no. 4, pp. 402–407, 2002.

[5] J. McNames and A. Fraser, "Obstructive sleep apnea classification based on spectrogram patterns in the electrocardiogram," in *Proc. Comput. Cardiology*, 2000, pp. 749–752.

[6] C. F. Poets, V. A. Stebbens, M. P. Samuels, and D. P. Southall, "The relationship between bradycardia, apnea, and hypoxemia in preterm infants," *Pediatric Res.*, vol. 34, no. 2, pp. 144–147, 1993.

[7] F. Portet, F. Gao, J. Hunter, and S. Sripada, "Evaluation of on-line bradycardia boundary detectors from neonatal clinical data," in *Proc. 29th Annu. Int. Conf. IEEE Eng. Med. Biol. Soc.*, 2007, pp. 3288–3291.

[8] J. HernáCruz, A. ndez, S. Wong, G. Carrault, and A. Beuchee, "Algorithm fusion for the early detection of apnea-bradycardia in preterm infants," in *Proc. Comput. Cardiology*, 2006, pp. 473–476.

[9] M. Altuve, G. Carrault, A. Beuchée, P. Pladys, and A. I. Hernández, "On-line apnea-bradycardia detection using hidden semi-markov models," in *Proc. Annu. Int. Conf. IEEE Eng. Med. Biol. Soc.*, 2011, pp. 4374–4377.

[10] M. Altuve, G. Carrault, A. Beuchée, P. Pladys, and A. I. Hernández, "On-line apnea–bradycardia detection based on hidden semi-markov models," in *Proc. Eng. Med. Biol. Soc.*, 2014, pp. 1–13.

[11] S. Masoudi, N. Montazeri, M. Shamsollahi, D. Ge, A. Beuchee, P. Pladys, and A. Hernandez, "Early detection of apnea-bradycardia episodes in preterm infants based on coupled hidden markov model," in *Proc. IEEE Int. Symp. Signal Process. Inform. Technol.*, 2013, pp. 000243–000248.

[12] L. Rabiner, "A tutorial on hidden markov models and selected applications in speech recognition," *Proc. IEEE*, vol. 77, no. 2, pp. 257–286, Feb. 1989.

[13] J. Dumont, A. I. Hernández, J. Fleureau, and G. Carrault, "Modeling temporal evolution of cardiac electrophysiological features using hidden semi-markov models," in *Proc. 30th Annu. Int. Conf. IEEE Eng. Med. Biol. Soc.*, 2008, pp. 165–168.

[14] M. Altuve, G. Carrault, J. Cruz, A. Beuchée, P. Pladys, and A. Hernandez, "Multivariate ecg analysis for apnoea? Bradycardia detection and characterisation in preterm infants," *Int. J. Biomed. Eng. Technol.*, vol. 5, no. 2, pp. 247–265, 2011.

[15] M. Brand, N. Oliver, and A. Pentland, "Coupled hidden markov models for complex action recognition," in *Proc. IEEE Comput. Soc. Conf. Comput. Vision Pattern Recog.*, 1997, pp. 994–999.

[16] N. Brewer, N. Liu, O. De Vel, and T. Caelli, "Using coupled hidden markov models to model suspect interactions in digital forensic analysis," in *Proc. Int. Workshop Integrating AI Data Mining,* 2006, pp. 58–64.

[17] M. Brand, "Coupled hidden markov models for modeling interacting processes," MIT Lab for Perceptual Computing, Tech. Rep.-405, 1997.

[18] J. Zhao, F. Gonzalez, and D. Mu, "Apnea of prematurity: From cause to treatment," *Eur. J. Pediatrics*, vol. 170, no. 9, pp. 1097–1105, 2011.

[19] A. V. Nefian, L. Liang, X. Pi, L. Xiaoxiang, C. Mao, and K. Murphy, "A coupled hmm for audio-visual speech recognition," in *Proc. IEEE Int. Conf. Acoust. Speech Signal Process.*, 2002, pp. II-2013–II-2016.

[20] J. Gai, Y. Li, and R. L. Stevenson, "Coupled hidden markov models for robust eo/ir target tracking," in *Proc. IEEE Int. Conf. Image Process.*, 2007, pp. I-41–I-44.

[21] I. Rezek, P. Sykacek, and S. J. Roberts, "Learning interaction dynamics with coupled hidden markov models," *IEE Proc. Sci. Meas. Technol.*, vol. 147, no. 6, pp. 345–350, Nov. 2000.

[22] S. Zhong and J. Ghosh, "A new formulation of coupled hidden markov models," Dept. Elect. Comput. Eng., Univ. Austin, Austin, TX, USA, 2001.

[23] T. Fu, X. X. Liu, L. H. Liang, X. Pi, and A. V. Nefian, "Audio-visual speaker identification using coupled hidden markov models," in *Proc. Int. Conf. Image Process.*, 2003, pp. III-29–32.

[24] S.-Z. Yu and H. Kobayashi, "Practical implementation of an efficient forward-backward algorithm for an explicit-duration hidden markov model," *IEEE Trans. Signal Process.*, vol. 54, no. 5, pp. 1947–1951, May 2006.

[25] L. E. Baum, T. Petrie, G. Soules, and N. Weiss, "A maximization technique occurring in the statistical analysis of probabilistic functions of markov chains," *Ann. Math. Stat.*, vol. 41, no. 1, pp. 164–171, 1970.

[26] L. Liporace, "Maximum likelihood estimation for multivariate observations of markov sources," *IEEE Trans. Inform. Theory*, vol. 28, no. 5, pp. 729–734, Sep. 1982.

[27] J. Pan and W. J. Tompkins, "A real-time qrs detection algorithm," *IEEE Trans. Biomed. Eng.*, vol. 32, no. 3, pp. 230–236, Mar. 1985.

[28] G. B. Moody, R. G. Mark, A. Zoccola, and S. Mantero, "Derivation of respiratory signals from multi-lead ecgs," *Comput. Cardiology*, vol. 12, pp. 113–116, 1985.

[29] K. Haskova, K. Javorka, M. Javorka, K. Matasova, and M. Zibolen, "Apnea in preterm newborns: Determinants, pathophysiology, effects on cardiovascular parameters and treatment," *Acta Medica Martiniana*, vol. 13, no. 3, pp. 5–17, 2013.

[30] F. Ye, N. Yi, and Y. Wang, "Em algorithm for training high-order hidden markov model with multiple observation sequences," *J. Inform. Comput. Sci.*, vol. 8, no. 10, pp. 1761–1777, 2011.

Authors' photographs and biographies not available at the time of publication.

Authorized licensed use limited to: CSIRO Information Technology Services. Downloaded on September 09,2022 at 00:27:34 UTC from IEEE Xplore. Restrictions apply.
