# Supplementary material for: Exploring Computational Techniques in Preprocessing Neonatal Physiological Signals for Detecting Adverse Outcomes: Scoping Review
Source: Interact J Med Res. 2024 Aug 20;13:e46946. doi: 10.2196/46946 (PMC11372324; doi:10.2196/46946)
Supplement: Multimedia Appendix 3 [file ijmr_v13i1e46946_app3.zip › Included Papers - Final/3777/Kota et al. - 2020 - Prognostic Value of Continuous Electroencephalogra.pdf]

Author Manuscript
Author Manuscript
Author Manuscript
Author Manuscript

HHS Public Access
Author manuscript
J Child Neurol. Author manuscript; available in PMC 2020 July 01.

Published in final edited form as:
J Child Neurol. 2020 July ; 35(8): 517–525. doi:10.1177/0883073820915323.

Prognostic Value of Continuous Electroencephalogram Delta Power in Neonates with Hypoxic Ischemic Encephalopathy

Srinivas Kota[1,2], **An N. Massaro**[1,3,4], **Taeun Chang**[4,5], **Tareq Al-Shargabi**[1], **Caitlin Cristante**[1], **Gilbert Vezina**[4,6], **Adre du Plessis**[1,4], **Rathinaswamy B. Govindan**[1,4]

[1]Division of Fetal and Transitional Medicine, Children's National Hospital, Washington, DC, USA

[2]Department of Neurosurgery, University of Texas Southwestern Medical Center, Dallas, TX, USA

[3]Division of Neonatology, Children's National Hospital, Washington, DC, USA

[4]The George Washington University School of Medicine, Washington, DC, USA

[5]Division of Neurology, Children's National Hospital, Washington, DC, USA

[6]Division of Diagnostic Radiology, Children's National Hospital, Washington, DC, USA

Abstract

The objective was to examine the discriminatory ability of electroencephalogram (EEG) delta power (DP) in neonates with hypoxic ischemic encephalopathy (HIE) with well-defined outcomes. Prolonged continuous EEG recordings from term neonates with HIE during therapeutic hypothermia enrolled in a prospective observational study were examined. Adverse outcome was defined as death or severe brain injury by MRI; favorable outcome was defined as normal or mild injury by MRI. Neonates were stratified by Sarnat grade of encephalopathy at admission. EEG was partitioned into 10-minute non-overlapping artifact- and seizure-free epochs. DP was calculated and compared between the groups using receiver operating characteristic (ROC) analyses and Wilcoxon rank-sum tests. An area under the ROC curve > 0.7 with $P < 0.05$ was considered a significant separation between groups. The favorable outcome group (n=67) had higher DP than the adverse outcome group (n=28) across the majority of time periods from 9 to 90 hours of life. DP discriminated outcome groups for neonates with moderate encephalopathy (63 favorable & 14 adverse outcome) earlier in cooling (9 to 42 hours of life) than neonates with severe encephalopathy (21 to 42 hours of life). Outcome groups were differentiated after 81 hours of life in neonates with moderate and severe encephalopathy. DP can distinguish cooled HIE neonates with adverse outcome independently of the encephalopathy grade at presentation. DP may be a real-time continuous biomarker of evolving encephalopathy and brain injury/death in neonates with HIE.

Address for Correspondence: Rathinaswamy B. Govindan, Director, Advanced Physiological Signal Processing Laboratory, Division of Fetal and Transitional Medicine, Children's National Health System, 111 Michigan Ave, NW, Washington, DC 20010, Phone: +1 202 476 5309, rgovinda@childrensnational.org.

Conflict of Interest: None to declare.

Author Manuscript Author Manuscript Author Manuscript Author Manuscript

Keywords

electroencephalogram; hypoxic ischemic encephalopathy; power spectral analysis; neonates

1. Introduction

Neonates with hypoxic-ischemic encephalopathy (HIE) continue to have high rates of death and disability despite the advent of therapeutic hypothermia as standard of care[1,2]. An important clinical gap is a reliable functional assessment that can identify neonates with unresponsive or worsening encephalopathy during hypothermia and can serve as an early predictor of neurological outcome.

Electroencephalogram (EEG) is a functional bedside tool used to assess and monitor severity of encephalopathy and identify seizures in neonates with acute HIE. Continuous video EEG (cEEG) recording is recommended in neonates with acute encephalopathy[3] and is now common in many tertiary neonatal intensive care units (NICU) providing hypothermia. Continuous or serial EEG can have high prognostic value in neonates with HIE.[4,5] Yet the utilization of EEG is limited by the need for trained neurophysiologists with expertise in neonatal EEG recordings to provide clinical interpretation at most centers.

We propose that quantitative characterization of the EEG background in neonates with HIE can overcome this challenge. A basic qualitative feature of EEG background is the predominant rhythms, in particular delta and theta activities for neonates[6]. We previously reported the potential utility of delta power as a quantitative EEG metric in HIE[7]. Recent studies have evaluated various other quantitative EEG metrics in neonates with HIE[8–11], but they examined only a few discrete time periods (e.g. in the first 24 hours of life or during rewarming) and/or used EEG data from a limited number of electrodes. As encephalopathy evolves acutely and location of brain injury is variable, only a comprehensive examination of all surface cortical regions throughout therapeutic hypothermia can identify which time point(s) after injury and which EEG lead location(s) is most predictive of the course and severity of encephalopathy. The aims of this study are to examine the evolution of EEG delta power in neonates with HIE during therapeutic hypothermia and determine its ability to distinguish neonates with moderate and severe HIE with favorable versus adverse outcomes as classified by death or evidence of significant brain injury on magnetic resonance imaging (MRI).

2. Materials and Methods

2.1 Study Population

Term neonates with HIE referred to our level IV NICU from June 2008 to July 2013 were enrolled in a prospective study evaluating biomarkers of brain injury. Following the National Institute of Child Health and Human Development Protocol[1], whole-body therapeutic hypothermia was initiated within six hours of life to maintain a core temperature of 33.5°C for 72 hours followed by rewarming at 0.5°C per 1–2 hours using a servo-controlled blanket (Blanketrol II, Cincinnati SubZero, OH, USA). Continuous video EEG recording

Author Manuscript
Author Manuscript
Author Manuscript
Author Manuscript

(NicoletOneTM, Viasys Healthcare, San Diego, CA, USA or Nihon Kohden, America, Inc., CA, USA) was initiated as soon as possible after admission and continued through a minimum of 6 hours post-rewarming. EEG electrode placement followed the International 10–20 system modified for newborns[3,12] and the EEG signals were sampled at a rate of 200 Hz using Nihon Kohden or 256 Hz using Nicolet. The EEG recordings were archived in Persyst data format (Persyst, AZ, USA). Clinical variables such as sex, gestational age at birth, birth weight, cord blood pH and base deficit, Apgar scores, and degree of encephalopathy at presentation were ascribed from medical records. The clinical grade of encephalopathy at admission (moderate =2 or severe =3) was scored according to Sarnat[13]. The study was approved by the Children's National's Institutional Review Board and informed written consent was obtained from the parent of each neonate.

2.2 Magnetic Resonance Imaging

MRI was performed per clinical protocol post-cooling on a 1.5 Tesla scanner (Signa, GE Healthcare, WI, USA, for patients enrolled between 2008–2010) or a 3 Tesla scanner (Discovery MR750, GE Healthcare, WI, USA, for patients enrolled between 2011–2013). Standard anatomical sequences included sagittal and coronal T1-weighted images, axial spin echo proton density and T2-weighted images (with axial T2 propeller images used in cases of patient motion), and axial diffusion weighted imaging. Details of the imaging protocols have been reported previously[4,15]. The images were scored by an experienced neuroradiologist (G. V.) who was blinded to the clinical outcome and EEG characteristics, using an established scoring system[16]. Adverse outcome was defined as death or severe brain injury by MRI (Basal Ganglia score ≥3 or Watershed ≥4). A normal or mild injury by MRI was defined as a favorable outcome (Basal Ganglia score < 3 or Watershed < 4).

2.3 EEG Preprocessing and Spectral Characterization

EEG recordings were reviewed by a neonatal neurologist (T. C.) to identify and mark seizure onset and end. EEG segments with seizure activity (including one hour of data on either side of every ictal period) were excluded from the analysis. EKG contamination was detected and attenuated using an automated approach without altering the EEG signal content as previously described[17]. EEG data with amplitude >500 μV or standard deviation <0.01 μV was disregarded as artifacts. The volume conduction (spatial spreading of electrical potential activity to nearby electrodes) was attenuated by calculating a global average of EEG voltages from all the electrodes for every sample recording and subtracting that global average from the EEG data of every electrode in the frequency domain[18]. The volume conduction attenuated EEG data was transformed back to time domain for spectral analysis. EEG data from every electrode were partitioned into 10-minute non-overlapping windows to calculate spectral power.

For each 10-minute window, the spectral analysis was performed using a Welch periodogram approach[7,19]. EEG data were divided into 3-second epochs to estimate the spectrum in a frequency resolution of 0.33 Hz (1/3 second). The periodogram of the EEG in each epoch was calculated as the square of the absolute of the Fourier transform of the data. To this end, the estimation of the spectrum was calculated as the average of the periodogram over all the windows. Following our earlier study, the spectral power in the delta frequency

Author Manuscript Author Manuscript Author Manuscript Author Manuscript

band was defined as the median of the logarithm of the spectral power in the frequency band of 0.5–4 Hz[7]. This procedure was repeated for every electrode.

2.4 Statistical Analysis

For each continuous 3-hour window, median delta power was compared between favorable and adverse outcome groups using receiver operating characteristic (ROC) analyses and Wilcoxon rank-sum tests. Since EEG power decays in power law fashion, we used median power as opposed to mean power[20]. These analyses were performed for all 11 electrodes (Fp1, Fp2, C3, C4, P3, P4, O1, O2, T3, T4, and Cz; Figure 2) and the relative magnitude of the area under the ROC curve[21] is depicted as a contour (iso-AUC) map. Analyses were repeated after stratifying neonates by encephalopathy grade to evaluate whether the predictive ability of delta power differed between moderately versus severely encephalopathic neonates.

A value of AUC>0.7 and a P<0.05 (from Wilcoxon rank-sum test) were considered to be significant separation between the delta powers of the two outcome groups. Of note, the P-values were adjusted for multiple comparisons according to the Bonferroni method to deflate Type I error[22]

3. Results

Table 1 displays the demographics of the study population. The distribution of outcome in our cohort is shown in Figure 1. A total of 95 neonates were enrolled in the study; 28 had adverse outcome (n=9 died; n=19 severe brain injury by MRI). Cooling was initiated at a median age of 4.6 hours of life (range 0.42 – 6.07 hours of life).

The median duration of the EEG recording was 75.78 hours (range 4.74 – 96.89 hours). The iso-AUC maps obtained for each continuous 3-hour period is shown in Figure 2. Starting at 9 hours of life, the delta power measured in at least one electrode location distinguished the favorable and adverse outcome groups. This was consistent until 90 hours of life except for four 3-hour periods (54–60 hours, 66–69 hours, 75–78 hours, and 78–81 hours). In general, the central-parietal regions (C3, C4, P3, or P4) demonstrated the highest discriminatory power at majority of time periods, and this is in agreement with an early study[23].

The delta power in favorable versus adverse outcome groups as stratified by encephalopathy grade at presentation are shown in Figure 3 (moderate) and Figure 4 (severe). For neonates with moderate encephalopathy, results were similar to those for the overall cohort, with significant differences from 9 to 90 hours of life except during five 3-hour periods (45–48 hours, 57–60 hours, 66–69 hours, 75–78 hours, and 78–81 hours). For the severe encephalopathy group, the difference between 21–42 hours was similar in topographic distribution observed in the moderate group, but at later hours (81–105 hours) the difference was mainly in the frontal regions overlaying Fp1 and Fp2 electrodes (Figure 4).

The median and interquartile delta power values from P3 are shown in Figure 5 for the overall cohort, as well as the groups stratified by encephalopathy grade. The median ROC thresholds for individual electrodes are shown in Table 2 for the overall cohort.

Author Manuscript
Author Manuscript
Author Manuscript
Author Manuscript

4. Discussion

EEG delta power is significantly lower in cooled neonates with HIE who die or have significant brain injury on MRI compared to neonates with normal or mild brain injury. Delta power can distinguish neonates at risk for significant neurologic injury as early as 9 hours of life. The ability to distinguish outcome groups was observed in neonates with both moderate and severe encephalopathy, although there may be differences in the topography of delta power changes based on severity of encephalopathy at presentation. In particular, delta power measured in the frontal polar, central or parietal region demonstrates consistent discriminative ability over the course of hypothermia and rewarming. This quantitative EEG metric can easily be calculated in real-time to offer an automated alternative or supplement to standard visual interpretation of complex EEG data to the bedside clinician in the NICU.

Several attempts have been made to identify bedside biomarkers of encephalopathy in HIE including peripherally circulating blood-based brain injury markers[14,24,25], amplitude-integrated EEG (aEEG)[26,27], heart rate variability[28–30], and cerebral autoregulation monitoring[31,32]. However, each one of them has certain limitations. Blood-based biomarkers provide only intermittent 'snapshots' of a dynamic rise and fall of each circulating factor after the insult. Some markers, such as pro-inflammatory cytokines, are expressed only transiently[21,30], limiting their utility as ongoing biomarkers. While aEEG allows simplified characterization of EEG background patterns[33], it still requires visual inspection and pattern recognition that may suffer from subjectivity and experience. Heart rate variability offers objective quantitative metrics that can be used to monitor disease progression and have been reported to have good correlation to qualitative EEG ratings in HIE[30]. However, heart rate variability may be affected by other comorbidities of critical illness and their treatments, rather than indicating neurological injury. Cerebral autoregulation monitoring has shown promise in several studies. However, application of this technique is feasible only when continuous blood pressure data from an indwelling arterial line is available. Continuous EEG recording allows for real-time bedside assessment of neuronal activity in critically ill neonates and is widely used in many tertiary care NICUs. Moreover, simple quantitative analysis of EEG has the potential to overcome the limitation of EEG expertise.

EEG studies have shown that delta power is dominant in newborn cortical activity compared to high-frequency waveforms such as theta, alpha, and beta[6,34,35]. Hence, we decided to use power in this band to characterize the EEG activity of neonates with HIE. Total power has been used in other studies as a prognostic indicator in HIE[9,23]. However, total power can be affected by electrical and ventilator artifacts common in the ICU setting. Specifically, a subharmonic of a powerline artifact may appear at 20 Hz or below and affect the characterization of total power. To best capture the predominant neuronal activity in the low-frequency band of neonates, as well as to avoid interference from external sources, we focused on delta power in this work. Use of relative spectral power (power in the delta frequency band divided by the total power) has been recommended in HIE monitoring[35,36]. We have shown in our earlier studies that absolute spectral power tracks evolving encephalopathy better than the relative power[7,37].

Author Manuscript Author Manuscript Author Manuscript Author Manuscript

Clinical grade of encephalopathy at presentation remains the mainstay of early prognostication in HIE. Prior studies have correlated qualitative and quantitative EEG metrics to Sarnat stage of encephalopathy[38,39]. Contrary to these findings, using a small cohort of 10 HIE neonates (5 moderate, 5 severe), Burnsed et al found no association between the EEG total power and HIE severity assessed at the initiation of hypothermia, however, the total power was negatively associated with length of hospital stay[37]. Our results partially support the findings of Burnsed et al. More important than correlating quantitative EEG metrics to initial encephalopathy grade is to assess whether delta power can provide additive prognostic value within clinically discernable groups. Our results suggest that the discriminatory value of EEG delta power is independent of clinical encephalopathy grade and can be used over time to offer additive prognostic information over the course of hypothermia for HIE.

Other signal processing methods such as detrended fluctuation analysis, and multifractal analysis stemming from statistical physics have also been explored to characterize EEG in neonates with HIE[20]. The multifractal spectral width has been shown to be correlated with interburst intervals observed in EEG tracings[20]. A combination of the amplitude of the EEG and interburst intervals measured using a computer-aided approach has been shown to improve the prognostic power of the EEG in neonates with HIE[11]. However, for band-limited signals such as EEG, the time scales (i.e. a region that shows power-law pattern in the fluctuation function) needed to estimate the fluctuation exponent or multifractal spectrum should be chosen with care. The power spectral analysis approach used in this study and elsewhere does not suffer from this limitation[8,9,23,35]. Future work is needed to compare the reliability of alternative quantitative EEG metrics as prognostic indicators in HIE.

Neonatal seizures are associated with worse neurological prognosis in HIE[40,41]. A seizure is a synchronous discharge of neuronal activity, which can increase the variability in the EEG data. Thus, a seizure is a potential confounder in quantitative EEG analysis since this abnormal EEG activity will manifest as an increase in spectral power. We disregarded the visually scored seizure epochs from our analysis for this reason. Delta power quantification will require incorporation of computational tools to distinguish ictal periods from EEG background activity (e.g. automated seizure detection algorithms)[42,43] but this is beyond the scope of this work.

Our study has several strengths such as the use of 1) well-archived prolong continuous full neonatal montage EEG from 95 neonates with well-characterized MRI outcomes, 2) visually scored ictal periods to optimize the characterization of delta power, 3) novel frequency-based techniques to attenuate the EKG contamination and volume conduction, and 4) advanced signals processing techniques to characterize EEG. Our study also has certain limitations; several studies have highlighted the importance of ultraslow brain waves (<0.5 Hz)[35,36,44,45] in neonates to follow the clinical course and it was of interest to the authors[8]. But unfortunately our clinical EEG recordings were collected at a high frequency (high pass cutoff at 0.3 Hz) which prevented us from examining spectral power below 0.5 Hz[35]. The incorporation of ultraslow activities might have improved the ability of delta power to distinguish outcome groups. All moderate-severe HIE neonates are transferred to our center

Author Manuscript Author Manuscript Author Manuscript Author Manuscript

within six hours of life for therapeutic hypothermia. EEG monitoring is initiated as soon as possible after admission (on average approximately 10 hours of life) and continued through rewarming (approximately 90–96 hours of life). Therefore, we did not have enough neonates to make reliable comparisons before 9 hours of life and after 105 hours of life. Hence, we cannot determine whether delta power has utility earlier after birth or later post rewarming. Sedation and seizure medications might have dampened the EEG variability[23]. We did not have detailed information on the dose and timing of these medications in order to evaluate their effects on delta power. Taking these aspects into consideration would strengthen future studies. We used MRI as our primary outcome measure as this is the gold standard for subacute assessment of brain injury after HIE[46]. Correlation with long-term neurodevelopmental outcomes is needed and ongoing.

5. Conclusions

Spectral power in the delta frequency band distinguishes neonates with HIE who die or have significant brain injury by MRI from survivors with favorable outcomes. Measures in the central-parietal or frontal polar regions have the highest discriminatory power. Although further studies are needed to relate delta power to long-term developmental outcomes, this quantitative EEG metric has promise as a bedside prognostic tool in HIE.

Acknowledgments

This work was supported by internal special purpose funds available in the Division of Fetal and Transitional Medicine as well as by the Award Number P30 HD040677, UL1RR031988, and KL2 RR031987 from the National Center for Research Resources.

References

1. Shankaran S, Laptook AR, Ehrenkranz RA, et al. Whole-body hypothermia for neonates with hypoxic-ischemic encephalopathy. N Engl J Med. 2005;353(15):1574–1584. [PubMed: 16221780]
2. Azzopardi DV, Strohm B, Edwards AD, et al. Moderate hypothermia to treat perinatal asphyxial encephalopathy. N Engl J Med. 2009;361(14):1349–1358. [PubMed: 19797281]
3. Shellhaas RA, Chang T, Tsuchida T, et al. The American Clinical Neurophysiology Society's Guideline on Continuous Electroencephalography Monitoring in Neonates. J Clin Neurophysiol. 2011;28(6):611–617. [PubMed: 22146359]
4. Abend NS, Dlugos DJ, Clancy RR. A review of long-term EEG monitoring in critically ill children with hypoxic-ischemic encephalopathy, congenital heart disease, ECMO, and stroke. J Clin Neurophysiol. 2013;30(2):134–142. [PubMed: 23545764]
5. Holmes G, Rowe J, Hafford J, Schmidt R, Testa M, Zimmerman A. Prognostic value of the electroencephalogram in neonatal asphyxia. Electroencephalogr Clin Neurophysiol. 1982;53(1):60–72. [PubMed: 6173201]
6. Tsuchida TN, Wusthoff CJ, Shellhaas RA, et al. American clinical neurophysiology society standardized EEG terminology and categorization for the description of continuous EEG monitoring in neonates: report of the American Clinical Neurophysiology Society critical care monitoring committee. J Clin Neurophysiol. 2013;30(2):161–173. [PubMed: 23545767]
7. Govindan RB, Massaro A, Vezina G, Tsuchida T, Cristante C, du Plessis A. Does relative or absolute EEG power have prognostic value in HIE setting? Clinical Neurophysiology. 2017;128:14–15. [PubMed: 27866114]
8. Ahmed R, Temko A, Marnane W, Lightbody G, Boylan G. Grading hypoxic-ischemic encephalopathy severity in neonatal EEG using GMM supervectors and the support vector machine. Clin Neurophysiol. 2016;127(1):297–309. [PubMed: 26093932]

Author Manuscript Author Manuscript Author Manuscript Author Manuscript

9. Birca A, Lortie A, Birca V, et al. Rewarming affects EEG background in term newborns with hypoxic-ischemic encephalopathy undergoing therapeutic hypothermia. Clin Neurophysiol. 2016;127(4):2087–2094. [PubMed: 26749567]
10. Jain SV, Mathur A, Srinivasakumar P, Wallendorf M, Culver JP, Zempel JM. Prediction of Neonatal Seizures in Hypoxic-Ischemic Encephalopathy Using Electroencephalograph Power Analyses. Pediatr Neurol. 2017;67:64–70 e62. [PubMed: 28062149]
11. Dereymaeker A, Matic V, Vervisch J, et al. Automated EEG background analysis to identify neonates with hypoxic-ischemic encephalopathy treated with hypothermia at risk for adverse outcome: A pilot study. Pediatrics & Neonatology. 2018.
12. Epstein CM. Digital EEG: trouble in paradise? J Clin Neurophysiol. 2006;23(3):190–193. [PubMed: 16751719]
13. Sarnat H, Sarnat M. Neonatal encephalopathy following fetal distress. Arch Neurol. 1976;33:696–705. [PubMed: 987769]
14. Massaro AN, Chang T, Kadom N, et al. Biomarkers of brain injury in neonatal encephalopathy treated with hypothermia. J Pediatr. 2012;161(3):434–440. [PubMed: 22494878]
15. Massaro AN, Bouyssi-Kobar M, Chang T, Vezina LG, du Plessis AJ, Limperopoulos C. Brain perfusion in encephalopathic newborns after therapeutic hypothermia. AJNR Am J Neuroradiol. 2013;34(8):1649–1655. [PubMed: 23493898]
16. Barkovich AJ, Hajnal BL, Vigneron D, et al. Prediction of neuromotor outcome in perinatal asphyxia: evaluation of MR scoring systems. AJNR Am J Neuroradiol. 1998;19(1):143–149. [PubMed: 9432172]
17. Govindan RB, Kota S, Al-Shargabi T, Massaro AN, Chang T, du Plessis A. Effect of electrocardiogram interference on cortico-cortical connectivity analysis and a possible solution. J Neurosci Methods. 2016;270:76–84. [PubMed: 27291356]
18. Kota S, du Plessis A, Massaro A, Chang T, Al-Shargabi T, Govindan RB. A frequency based spatial filter to mitigate volume conduction in electroencephalogram signals. Paper presented at: IEEE EMBC2016; Orlando, Florida, USA.
19. Halliday DM, Rosenberg JR, Amjad AM, Breeze P, Conway BA, Farmer SF. A framework for the analysis of mixed time series/point process data--theory and application to the study of physiological tremor, single motor unit discharges and electromyograms. Prog Biophys Mol Biol. 1995;64(2–3):237–278. [PubMed: 8987386]
20. Matic V, Cherian PJ, Koolen N, et al. Objective differentiation of neonatal EEG background grades using detrended fluctuation analysis. Front Hum Neurosci. 2015;9:189. [PubMed: 25954174]
21. Fairchild KD, Saucerman JJ, Raynor LL, et al. Endotoxin depresses heart rate variability in mice: cytokine and steroid effects. Am J Physiol Regul Integr Comp Physiol. 2009;297(4):R1019–1027. [PubMed: 19657103]
22. Armstrong RA. When to use the Bonferroni correction. Ophthalmic Physiol Opt. 2014;34(5):502–508. [PubMed: 24697967]
23. Jain SV, Zempel JM, Srinivasakumar P, Wallendorf M, Mathur A. Early EEG power predicts MRI injury in infants with hypoxic-ischemic encephalopathy. J Perinatol. 2017;37(5):541–546. [PubMed: 28206999]
24. Chalak LF, Sanchez PJ, Adams-Huet B, Laptook AR, Heyne RJ, Rosenfeld CR. Biomarkers for severity of neonatal hypoxic-ischemic encephalopathy and outcomes in newborns receiving hypothermia therapy. J Pediatr. 2014;164(3):468–474 e461. [PubMed: 24332821]
25. Ennen CS, Huisman TA, Savage WJ, et al. Glial fibrillary acidic protein as a biomarker for neonatal hypoxic-ischemic encephalopathy treated with whole-body cooling. Am J Obstet Gynecol. 2011;205(3):251 e251–257. [PubMed: 21784396]
26. Hellstrom-Westas L. Monitoring brain function with aEEG in term asphyxiated infants before and during cooling. Acta Paediatr. 2015;102(7):678–679.
27. Massaro AN, Tsuchida T, Kadom N, et al. aEEG evolution during therapeutic hypothermia and prediction of NICU outcome in encephalopathic neonates. Neonatology. 2012;102(3):197–202. [PubMed: 22796967]

Author Manuscript

Author Manuscript

Author Manuscript

Author Manuscript

28. Govindan RB, Massaro AN, Al-Shargabi T, et al. Detrended fluctuation analysis of non-stationary cardiac beat-to-beat interval of sick infants. EPL (Europhysics Letters). 2014;108:40005–p40001–p40006.
29. Massaro AN, Govindan RB, Al-Shargabi T, et al. Heart rate variability in encephalopathic newborns during and after therapeutic hypothermia. J Perinatol. 2013;34(11):836–841.
30. Vergales BD, Zanelli SA, Matsumoto JA, et al. Depressed heart rate variability is associated with abnormal EEG, MRI, and death in neonates with hypoxic ischemic encephalopathy. Am J Perinatol. 2013;31(10):855–862. [PubMed: 24347263]
31. Howlett JA, Northington FJ, Gilmore MM, et al. Cerebrovascular autoregulation and neurologic injury in neonatal hypoxic-ischemic encephalopathy. Pediatr Res. 2013;74(5):525–535. [PubMed: 23942555]
32. Massaro AN, Govindan RB, Vezina G, et al. Impaired cerebral autoregulation and brain injury in newborns with hypoxic-ischemic encephalopathy treated with hypothermia. J Neurophysiol. 2015;114(2):818–824. [PubMed: 26063779]
33. Hellstrom-Westas L, Rosen I, Svenningsen NW. Predictive value of early continuous amplitude integrated EEG recordings on outcome after severe birth asphyxia in full term infants. Arch Dis Child Fetal Neonatal Ed. 1995;72(1):F34–38. [PubMed: 7743282]
34. Scher MS, Sun M, Steppe DA, Guthrie RD, Sclabassi RJ. Comparisons of EEG spectral and correlation measures between healthy term and preterm infants. Pediatr Neurol. 1994;10(2):104–108. [PubMed: 8024658]
35. Doyle OM, Greene BR, Murray DM, Marnane L, Lightbody G, Boylan GB. The effect of frequency band on quantitative EEG measures in neonates with hypoxic-ischaemic encephalopathy. Conf Proc IEEE Eng Med Biol Soc. 2007;2007:717–721. [PubMed: 18002057]
36. Temko A, Doyle O, Murray D, Lightbody G, Boylan G, Marnane W. Multimodal predictor of neurodevelopmental outcome in newborns with hypoxic-ischaemic encephalopathy. Comput Biol Med. 2015;63:169–177. [PubMed: 26093065]
37. Burnsed J, Quigg M, Zanelli S, Goodkin HP. Clinical severity, rather than body temperature, during the rewarming phase of therapeutic hypothermia affect quantitative EEG in neonates with hypoxic ischemic encephalopathy. J Clin Neurophysiol. 2011;28(1):10–14. [PubMed: 21221006]
38. Hathi M, Sherman DL, Inder T, et al. Quantitative EEG in babies at risk for hypoxic ischemic encephalopathy after perinatal asphyxia. J Perinatol. 2010;30(2):122–126. [PubMed: 19741652]
39. Murray DM, Boylan GB, Ryan CA, Connolly S. Early EEG findings in hypoxic-ischemic encephalopathy predict outcomes at 2 years. Pediatrics. 2009;124(3):e459–467. [PubMed: 19706569]
40. Glass HC, Shellhaas RA, Wusthoff CJ, et al. Contemporary Profile of Seizures in Neonates: A Prospective Cohort Study. J Pediatr. 2016;174:98–103 e101. [PubMed: 27106855]
41. Srinivasakumar P, Zempel J, Trivedi S, et al. Treating EEG Seizures in Hypoxic Ischemic Encephalopathy: A Randomized Controlled Trial. Pediatrics. 2015;136(5):e1302–1309. [PubMed: 26482675]
42. Temko A, Sarkar AK, Boylan GB, Mathieson S, Marnane WP, Lightbody G. Toward a Personalized Real-Time Diagnosis in Neonatal Seizure Detection. IEEE J Transl Eng Health Med. 2017;5:2800414. [PubMed: 29021923]
43. Temko A, Thomas E, Marnane W, Lightbody G, Boylan G. EEG-based neonatal seizure detection with Support Vector Machines. Clin Neurophysiol. 2011;122(3):464–473. [PubMed: 20713314]
44. Miller JW, Kim W, Holmes MD, Vanhatalo S. Ictal localization by source analysis of infraslow activity in DC-coupled scalp EEG recordings. Neuroimage. 2007;35(2):583–597. [PubMed: 17275335]
45. Thordstein M, Lofgren N, Flisberg A, Bagenholm R, Lindecrantz K, Kjellmer I. Infraslow EEG activity in burst periods from post asphyctic full term neonates. Clin Neurophysiol. 2005;116(7):1501–1506. [PubMed: 15953555]
46. D'Alton ME, Hankins GDV, Berkowitz RL, et al. Executive summary: Neonatal encephalopathy and neurologic outcome, second edition. Report of the American College of Obstetricians and Gynecologists' Task Force on Neonatal Encephalopathy. Obstet Gynecol. 2014;123(4):896–901. [PubMed: 24785633]

47. Variane GFT, Magalhaes M, Gasperine R, et al. Early amplitude-integrated electroencephalography for monitoring neonates at high risk for brain injury. J Pediatr (Rio J). 2017;93(5):460–466. [PubMed: 28238681]

Author Manuscript Author Manuscript Author Manuscript Author Manuscript

Author Manuscript
Author Manuscript
Author Manuscript
Author Manuscript

Highlights:

- Neonates with adverse outcome (brain injury or death) after HIE had reduced EEG delta power.
- The regions overlaying central, parietal or frontal polar electrodes best distinguished the delta power consistently over time.
- EEG delta power may serve as a reliable biomarker to track evolving encephalopathy during cooling.

Author Manuscript
Author Manuscript
Author Manuscript
Author Manuscript

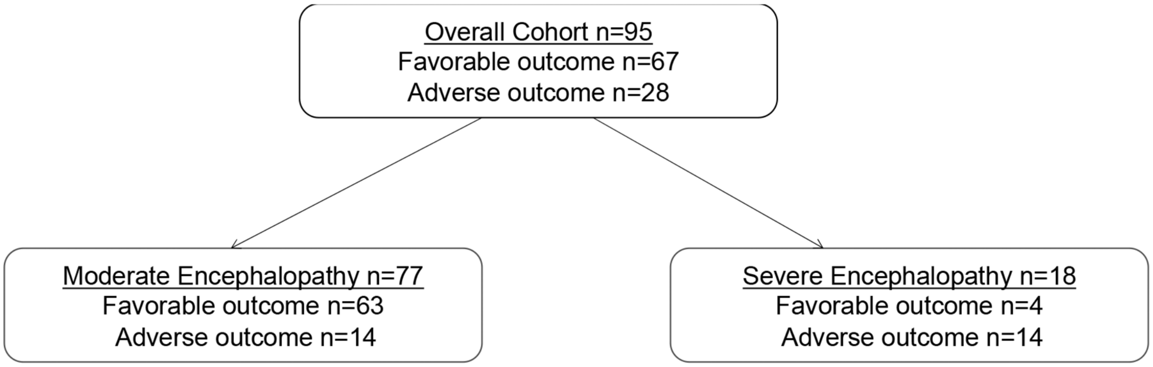

Figure 1.
Study population cohort diagram

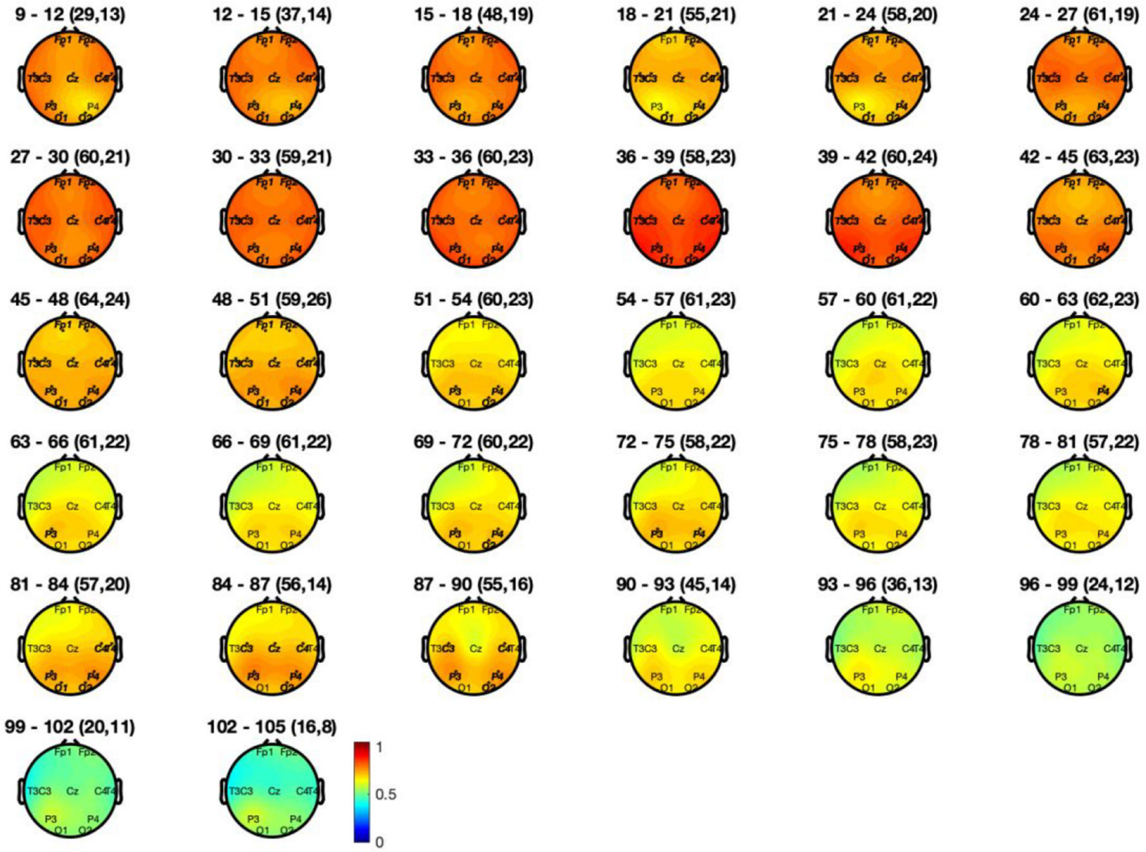

Figure 2.

The iso-AUC maps for every three hours from 9 to 105 hours of life for overall cohort (n=95). Higher AUC (increased ability to discriminate favorable versus adverse outcome groups) is depicted by warmer colors whereas poor discriminatory power is represented by cooler colors. The postnatal age in hours is shown in the title above each map followed by the number of neonates from the favorable (n1) and adverse (n2) outcome groups in that 3-hour window (n1, n2). An asterisk next to the electrode name (font bold and italic) indicates AUC > 0.7 and P < 0.05.

Author Manuscript
Author Manuscript
Author Manuscript
Author Manuscript

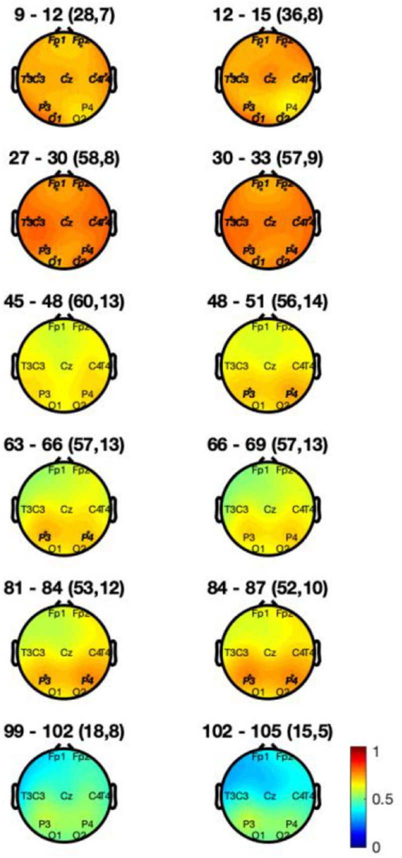

Figure 3.

The iso-AUC maps for every three hours from 9 to 105 hours of life for moderate encephalopathy group (n=77). Higher AUC (increased ability to discriminate favorable versus adverse outcome groups) is depicted by warmer colors whereas poor discriminatory power is represented by cooler colors. The postnatal age in hours is shown in the title above each map followed by the number of neonates from the favorable (n1) and adverse (n2) outcome groups in that 3-hour window (n1, n2). An asterisk next to the electrode name (font bold and italic) indicates AUC > 0.7 and P < 0.05.

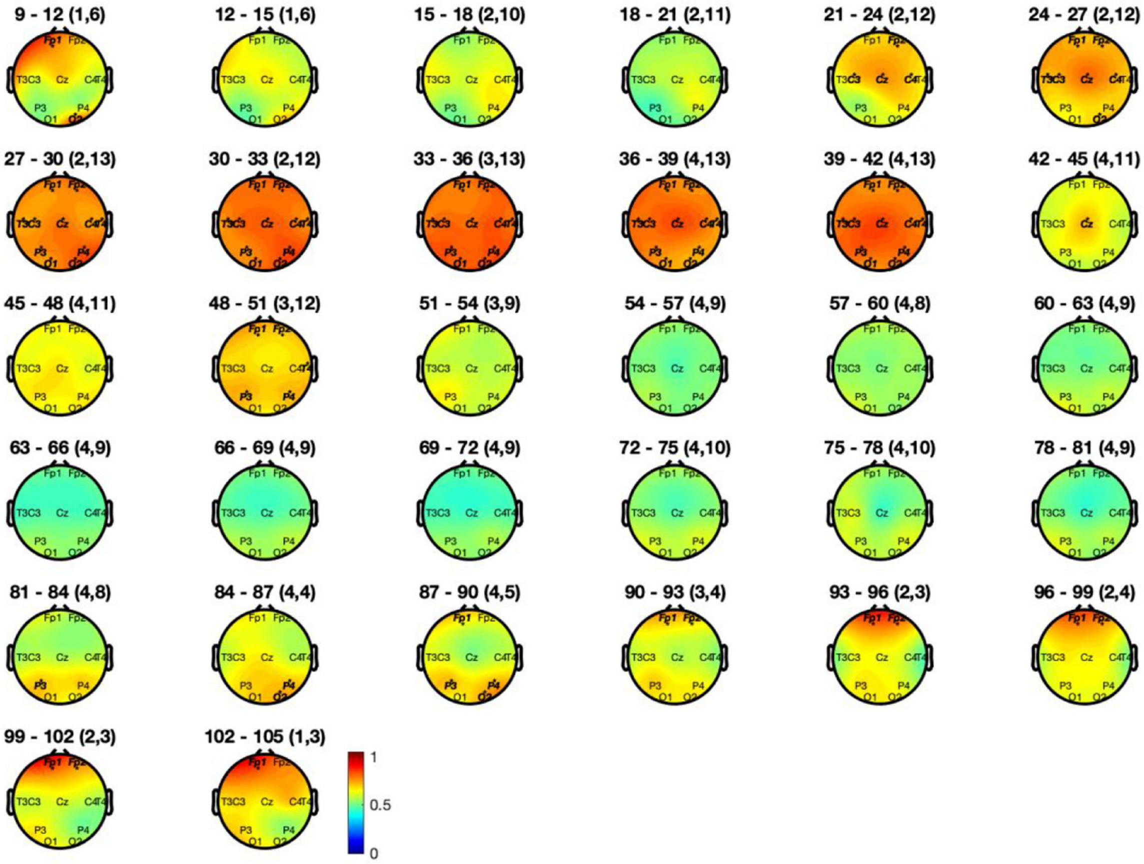

Figure 4.

The iso-AUC maps for every three hours from 9 to 105 hours of life for severe encephalopathy group (n=18). Higher AUC (increased ability to discriminate favorable versus adverse outcome groups) is depicted by warmer colors whereas poor discriminatory power is represented by cooler colors. The postnatal age in hours is shown in the title above each map followed by the number of neonates from the favorable (n1) and adverse (n2) outcome groups in that 3-hour window (n1, n2). An asterisk next to the electrode name (font bold and italic) indicates AUC > 0.7 and P < 0.05.

Author Manuscript Author Manuscript Author Manuscript Author Manuscript

Author Manuscript Author Manuscript Author Manuscript Author Manuscript

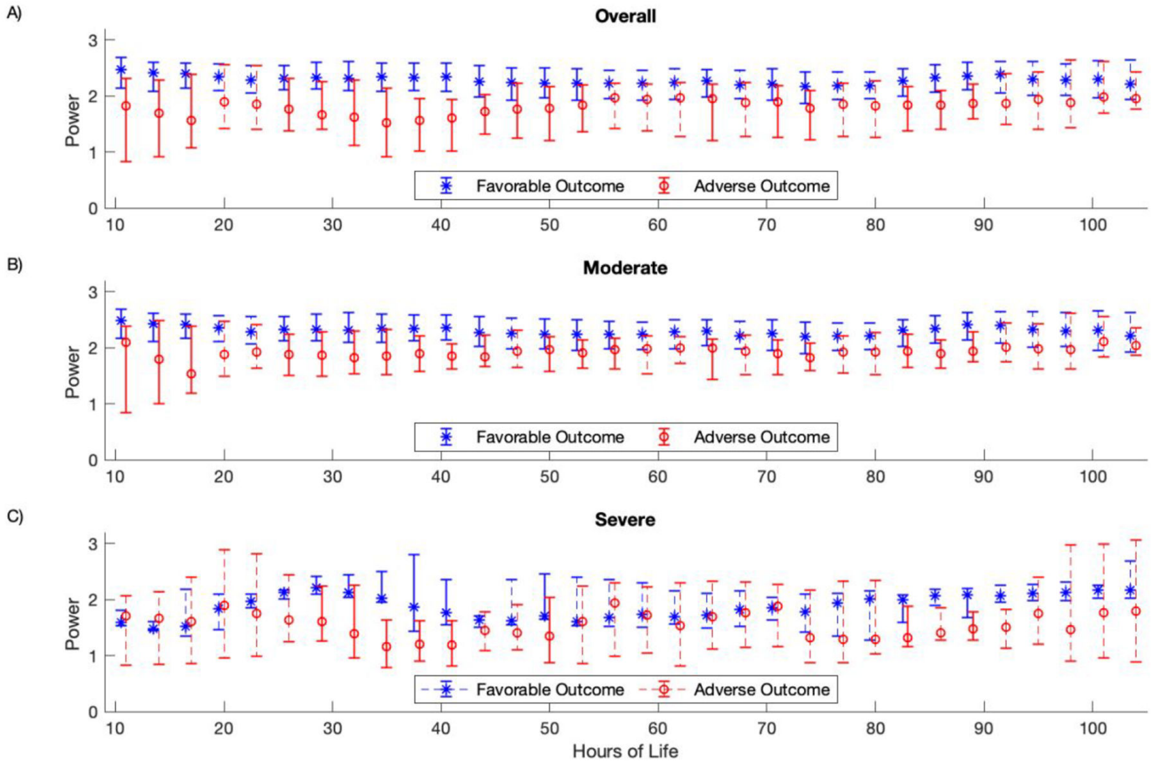

Figure 5.

The delta power values calculated for EEG from P3 are presented as median and interquartile range for favorable (blue) and adverse (red) outcome groups for the A) overall (n=95), B) moderate (n=77), and C) severe (n=18) groups, as a function of hours of life. The solid bars indicate significant difference (AUC > 0.7; $P < 0.05$) while dashed bars indicate $P > 0.05$.

Author Manuscript
Author Manuscript
Author Manuscript
Author Manuscript

Table 1.

Demographics and clinical characteristic at birth (n=95). The data are presented as median [minimum maximum], mean (± standard deviation) or n (%).

**Demographics and Clinical Characteristic**	**Favorable Outcome**	**Adverse Outcome**	**P-Value**
	**n=67**	**n=28**	
Male	40 (59.70%)	16 (57.14%)	0.8172
Gestational Age at Birth in weeks	39 [34 42]	39 [35 43]	0.1293
Birth Weight in kilogram	3.29 (± 0.68)	3.31 (± 0.64)	0.8630
Cord Arterial pH	7 [6.44 7.35][a]	6.89 [6.5 7.3][b]	0.0989
Base Deficit	17 [8 36][c]	20 [7 32][d]	0.1425
Apgar at 1 min	2 [0 6]	1 [0 8]	0.0526
Apgar at 5 min	4 [0 9]	3 [0 9]	**0.0011**
Severe Encephalopathy (Grade = 3)	4 (5.97%)	14 (50%)	< **0.0001**

Data available for

[a]66/67,

[b]26/28,

[c]61/67, and

[d]21/26 neonates

Author Manuscript
Author Manuscript
Author Manuscript
Author Manuscript

Table 2:

The ROC threshold for overall cohort at 9–12, 24–27, 48–51 and 72–25 hours of life.

	Hours of Life			
**Electrode**	**09–12**	**24–27**	**48–51**	**72–75**
Fp1	2.51	2.29	2.43	NS
Fp2	2.11	2.52	2.39	NS
C3	1.58	1.83	1.65	NS
C4	1.56	1.75	1.65	NS
P3	1.97	1.97	1.86	1.94
P4	NS	1.86	2.11	1.99
O1	2.35	2.10	2.41	NS
O2	1.88	2.02	2.30	NS
T3	2.47	2.12	2.36	NS
T4	2.52	2.17	2.25	NS
Cz	2.25	1.97	1.91	NS
