# Supplementary material for: Exploring Computational Techniques in Preprocessing Neonatal Physiological Signals for Detecting Adverse Outcomes: Scoping Review
Source: Interact J Med Res. 2024 Aug 20;13:e46946. doi: 10.2196/46946 (PMC11372324; doi:10.2196/46946)
Supplement: Multimedia Appendix 3 [file ijmr_v13i1e46946_app3.zip › Included Papers - Final/3794/S. Masoudi et al. - 2013 - Early detection of apnea-bradycardia episodes in p.pdf]

Early Detection of Apnea-Bradycardia Episodes in Preterm Infants based on Coupled Hidden Markov Model

S. Masoudi[1], N. Montazeri[1,2], M.B. Shamsollahi[1], D. Ge[2,3], A. Beuchée[2,3,4], P. Pladys[2,3,4], A.I. Hernandez[2,3]

[1] *BiSIPL, School of Electrical Engineering, Sharif University of Technology, Tehran, Iran,*
[2] *LTSI, Université de Rennes 1, Rennes, France*
[3] *INSERM: U1099, Rennes, France*
[4] *CHU Rennes, Pôle Médico-Chirurgical de Pédiatrie et de Génétique Clinique, Néonatologie, Rennes, France*

{masoudi, nmontazeri}@ee.sharif.edu, mbshams@sharif.edu, di.ge@univ-rennes1.fr, {alain.beuchee, patrick.pladys}@chu-rennes.fr, alfredo.hernandez@univ-rennes1.fr

Abstract—**The incidence of apnea-bradycardia episodes in preterm infants may lead to neurological disorders. Prediction and detection of these episodes are an important task in healthcare systems. In this paper, a coupled hidden Markov model (CHMM) based method is applied to detect apnea-bradycardia episodes. This model is evaluated and compared with two other methods based on hidden Markov model (HMM) and hidden semi-Markov model (HSMM). Evaluation and comparison are performed on a dataset of 233 apnea-bradycardia episodes which have been manually annotated. Observations are composed of RR-interval time series and QRS duration time series. The performance of each method was evaluated in terms of sensitivity, specificity and time detection delay. Results show that CHMM has the sensitivity of 84.92%, specificity of 94.17% and time detection delay of 2.32±4.82 seconds, which are better than the reference methods.**

I. Introduction

Apnea-bradycardia episode is defined as a respiratory pause accompanied with a heart rate reduction which is a common event in preterm infants [1]. The repetition of these episodes compromises oxygenation and tissue perfusion and may lead to neurological impairment or even short-term morbi-mortality. Since apnea-bradycardia event consists of an apnea episode followed by a significant fall in heart rate, earlier detection of bradycardia means better detection of apnea. However, most of the works in this area try to provide an acceptable solution using only cardiac signals, limiting the use of the more noisy respiratory signals. Recent researches in detection of apnea-bradycardia episodes, mostly concentrate on processing of a single variable extracted from the electrocardiogram (ECG): the cardiac cycle length (RR interval) [4].

Premature infants' cardiac signals are continuously monitored in neonatal intensive care units (NICU), in order to detect apnea-bradycardia events and to initiate quick nursing actions such as manual or vibrotactile stimulation, oxygenation and ventilation through a mask or intubation [2,3]. Typically, when an infant presents a bradycardia event, an alarm is generated by a monitoring device, an available nurse or physician goes to the appropriate NICU room, and applies a manual stimulation to the infant in distress.

Conventional detectors used in NICU detect bradycardia through fixed or relative-threshold methods. In these methods high specificities (few false alarms) and low sensitivities (missed apnea-bradycardia episodes) are achieved. They also produce long detection delays that prolong the intervention delay. As an evidence for this conclusion the mean intervention delay measured from the activation of the alarm to the application of the therapy based on these approaches, has been estimated to be around 33 seconds with mean manual stimulation duration of 13 seconds [4].

Literature on prognostic methods is extremely limited but the concept has been gaining importance in recent years. Unlike numerous methods available for diagnostics, prognostics is still in its infancy and researchers are yet to present a working model for effective prognostics. In our recent works, hidden Markov model (HMM) and hidden semi-Markov model (HSMM) have been proposed as useful tools for early detection of apnea-bradycardia [5, 6]. HMM, as a probabilistic approach, has been quite effective in some applications such as speech and biomedical signal processing. Although the statistical methods of hidden Markov modelling were initially introduced and studied in the late 1960s and early 1970s, they have become increasingly popular recently. There are two major reasons for this. First, the models have very rich mathematical structure and can form a solid theoretical foundation. Second, the models have many successful applications in practice. The fact that HMM-based models could be applied in the area of prognostics was first pointed out by Bunks et al [7] and Baruah and Chinnam [8] in machining processes field. HMM is a statistical model with unobserved states that produce a sequence of observations. Stochastic processes which can be modelled in HMMs are Markovian. In this network, change for a future state depends

978-1-4799-4796-6/13/$31.00 © 2013 IEEE

Authorized licensed use limited to: CSIRO Information Technology Services. Downloaded on September 09,2022 at 00:23:12 UTC from IEEE Xplore. Restrictions apply.

solely on the current state. An inherent limitation of the HMM approach is that it does not provide adequate representation of systems in which the stage-phase in a given state cannot be represented by a geometrical law. One way to overcome this limitation is to apply Hidden Semi-Markov Models. HSMM's structure is similar to a classic HMM but the main difference is that the unobserved process is semi-Markov in the sense that a change for a future hidden state depends on both the current state and the time spent on this state. Unlike the standard HMM in which one state produces one single observation, a state in a semi-Markov model is defined as consecutive repetitions of that state which generates a segment of observations.

Subsequently, in a simple HMM there might be several observation sequences. This is the case when a number of observation sequences are extracted from one single signal, i.e. the RR series and the QRS durations from the same ECG signal in the current application. For this purpose, it is essential to study the coupling effects between the channel dynamics behind the observation sequences. In such a coupled hidden Markov model (CHMM), states in multiple channels evolve with mutual dependences while each observation sequence follows an emission distribution conditionally to the channel state only. This implies a Cartesian product form of states in multiple channels for the Markovian transition law, to potentially increase the amount of information extracted from data.

The objective of this paper is to apply the CHMM framework for apnea-bradycardia detection. Also, in order to provide a better insight about the added value of a CHMM approach, a quantitative performance comparison with respect to our previous works on HMM and HSMM-based apnea-bradycardia detectors is presented. These three methods are evaluated on the same database. The implementation of CHMM in this work is based on the framework proposed by Rezek et al. [9].

This paper is organised as follows: Section II will introduce basic concepts of CHMM. Application of this method and the comparison with the others in detection of apnea-bradycardia will be discussed in section III. Concluding remarks will be presented in section IV.

II. CHMM

CHMM is a statistical signal model with a coupled multi-channel structure. These channels are parallel to each other, each of them acts like a Markov chain and represents a random sequence of observations. These observations are based on probabilistic functions given the unobserved states [10]. A sequence will be generated as a result of a random walk in transition networks of the states. The transitions are reflected in the corresponded chain and this causes the production of an observation at each visit of a state [11, 12]. A general CHMM model with C channels is depicted in Fig.1 having the following elements, for c=1,..,C :

1. A Set of states: $S^c=\{s_1^c, s_2^c, s_3^c, \ldots, s_{I_c}^c\}$, (Number of states: I_c)

2. A Set of individual observations: $V^c=\{v_1^c, v_2^c, v_3^c, \ldots, v_{K_c}^c\}$, (Number of observations: K_c)

3. The state transition probability distribution $A^c = \{a^c_{i_1 i_2 \ldots i_C j_c}\}$ which is a C+1 ordered tensor needed to encode the state transition law in each channel c and each tensor has $I_c \times \prod_{c'=1}^{C} I_{c'}$ elements.

$$a^c_{i_1 i_2 \ldots i_C j_c} = P(q^c_{t+1} = s^c_{j_c} \mid q^1_t = s^1_{i_1}, \ldots, q^C_t = s^C_{i_C})$$

$$\sum_{j_c=1}^{I_c} a^c_{i_1 i_2 \ldots i_C j_c} = 1 \qquad (1)$$

4. The observation probability distribution in state s_i^c , $B^c = \{b_i^c[k]\}$, where

$$b_i^c[k] = P(O_t^c = v_k^c | q_t^c = s_i^c)$$

$$\sum_{k=1}^{K_c} b_i^c[k] = 1 \qquad (2)$$

5. The initial state distribution $\pi^c = \{\pi_i^c\}$ where

$$\pi_i^c = P(q_1^c = s_i^c) \qquad \sum_{i=1}^{I_c} \pi_i^c = 1 \qquad (3)$$

So for each channel the model can be described by the compact notation set of parameters, often used in the literature:

$$\lambda^c = (A^c, B^c, \pi^c) \qquad (4)$$

The final model for general CHMM can be expressed as in Eq.5.

$$\lambda = (\lambda^1, \lambda^2, \ldots, \lambda^C) \qquad (5)$$

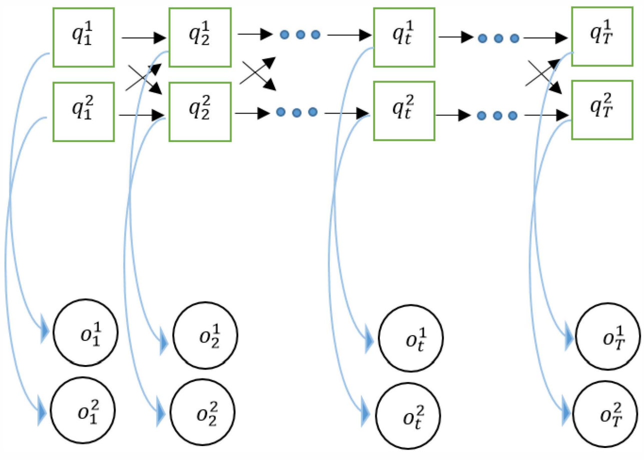

Fig. 1 A general CHMM with 2 channels

According to these characteristics of the CHMM's structure, the following equations can be deduced. One essential probabilistic description of the above system is expressed as

Authorized licensed use limited to: CSIRO Information Technology Services. Downloaded on September 09,2022 at 00:23:12 UTC from IEEE Xplore. Restrictions apply.

$$P(q_{t+1}^1, \dots, q_{t+1}^C | q_t^1, \dots, q_t^C) = \prod_{c=1}^{C} P(q_{t+1}^c | q_t^1, \dots, q_t^C) \quad (6)$$

which declares that the states at time t+1 (q_{t+1}^c,c=1,…,C) given all the states at time t, are independent. We also have the independence of observations at time t (o_t^c, c=1,…,C) when their corresponding states are specified.

$$P(o_t^1, \dots, o_t^C | q_t^1, \dots, q_t^C) = \prod_{c=1}^{C} P(o_t^c | q_t^c) \quad (7)$$

For continuous observation problems, some restrictions have to be forced on the form of the emission probability. One of the most general representation is a finite mixture of Gaussian distributions, and is applied in this work:

$$P_{b_j^c(O_t^c)} = \sum_{m=1}^{M^c} C_{jm}^c \mathfrak{N}[O_t^c, \mu_{jm}^c, \sigma_{jm}^c], \quad 1 \le j \le \mathrm{Ic} \quad (8)$$

where $\mathfrak{N}$ represents the Gaussian distribution and C_{jm}^c stands for its weight coefficient. The number of mixed Gaussian distributions with the mean and variance are noted M^c, μ_{jm}^c and σ_{jm}^c respectively.

Similar to the HMM [14], three basic problems are to be addressed for the CHMM:

(1) Evaluation (also called Classification): Given C observation sequences $\mathbf{o}^c = o_1^c, o_2^c, o_3^c, \dots, o_T^c$ for each of C channels with C set of parameters λ^c, where c=1,.., C, what is the probability of the all C observation sequences with the given model, i.e., $P(\mathbf{o}^1,\mathbf{o}^2,\dots,\mathbf{o}^C|\lambda^1, \lambda^2,\dots, \lambda^C)$?

(2) Decoding (also called Recognition or Inference): Given C observation sequences $\mathbf{o}^c = o_1^c, o_2^c, o_3^c, \dots, o_T^c$ for each of C channels with C set of parameters λ^c, where c=1,.., C, what sequences of hidden states: $\mathbf{q}^c = q_1^c, q_2^c, q_3^c, \dots, q_T^c$, c=1,…,C, will generate these given sequences of observations with maximum probability?

(3) Learning (also called Training): Given C observation sequences $\mathbf{o}^c = o_1^c, o_2^c, o_3^c, \dots, o_T^c$ for each of C channels, how do we adjust the model parameters $\lambda^c = (A^c, B^c, \pi^c)$, c = 1, …, C, to maximize $P(\mathbf{o}^1,\mathbf{o}^2,\dots,\mathbf{o}^C|\lambda^1, \lambda^2,\dots, \lambda^C)$?

Different algorithms have been developed for these problems and are based on the simplification of the CHMM structure. As a comprehensive and precise CHMM method, Rezek et al. [9] framework is employed in this work. They substituted the C channel CHMM with a large single-channel HMM-like structure and each state can be viewed as a Cartesian product of the states in C channels : $\mathbf{s} = [s_{i_1}^1, s_{i_2}^2, s_{i_3}^3, \dots, s_{i_C}^C]$ where $s_{i_c}^c$ is a member of the set S^c for any c=1,…,C.

Therefore, there would be total $N = \prod_{c=1}^{C} I_c$ possible states in this large HMM-like at every time instant. Based on the idea of Rezek et al. [9] an N by N matrix called A is formed, in which each element represents probability of state transition from one C-fold state to another state of the respective structure in a large HMM-like. Strictly speaking, a large HMM is something more general.

In Rezek's CHMM, observation in one time instant can be considered as a C×1 vector $\mathbf{v} = [v_{k_1}^1, v_{k_2}^2, v_{k_3}^3, \dots, v_{k_C}^C]$ where $v_{k_c}^c$ is a member of the set V^c for any c=1,…,C. So there are $M = \prod_{c=1}^{C} K_c$ possible observations at every time instant of the large HMM. An N by M matrix B can be defined to denote the emission probabilities of the CHMM. Thus this large HMM can be described as $\lambda = (A, B, \pi)$.

Practically there is no necessity to estimate the full N×N state transition matrix A , however each element of the transition matrix A can still be obtained as a factor of the elements of the original transition matrices (A^c) of the C-channel CHMM.

Consequently solving the three aforesaid problems for a CHMM is equivalent to solving the same problems in a single-channel HMM context.

The forward–backward procedure[10,11] is used to solve the evaluation problem, whereas for the decoding problems the Viterbi algorithm is applied to find the most likely state sequence given the observation sequence in the maximum a posteriori probability sense by maximizing P(S|O, λ). As for the learning problem, no analytical solution exists in the literature. An iterative procedure such as the Baum–Welch method (or equivalently the Expectation-Maximization algorithm) that locally maximizes P(O|λ) is applied to allow adjustment of model parameters $\lambda = (A, B, \pi)$ [15].

Using Rezek's form, the forward variable $\alpha_t(\mathbf{s})$ is defined as

$$\alpha_t(\boldsymbol{s}) = P(\boldsymbol{o}_{1:t}^1 \boldsymbol{o}_{1:t}^2, \dots, \boldsymbol{o}_{1:t}^C, q_t^1 = s_{i_1}^1, q_t^2 = s_{i_2}^2, \dots, q_t^C = s_{i_C}^C | \lambda) \quad (9)$$

which is employed to calculate the probability of entire O = $\mathbf{o}^1,\mathbf{o}^2,\dots,\mathbf{o}^C$ given the whole model λ. This definition leads to produce the joint likelihood for a CHMM model, i.e., P(O|λ).

Based on work of Rezek et al. [9] there is another forward variable $\boldsymbol{\alpha}_t^c$ defined as

$$\boldsymbol{\alpha}_t^c = P(\boldsymbol{o}_{1:t-1}^1 \boldsymbol{o}_{1:t-1}^2, \dots, \boldsymbol{o}_{1:t}^c, \dots, \boldsymbol{o}_{1:t-1}^C, q_t^c = s_{i_c}^c | \lambda^c) \quad (10)$$

By using this statement which was elicited Eq.7, in evaluation phase there would be one likelihood for each channel. Then in a C channel CHMM the joint probability of $\mathbf{o}^1,\mathbf{o}^2,\dots,\mathbf{o}^C$ given the model λ^c can be the multiplication of all C likelihoods. It can be shown that $\boldsymbol{\alpha}_t^c$ can be obtained as follows:

$$\boldsymbol{\alpha}_t^c = \underbrace{\sum_{i_1} \dots \sum_{i_C}}_{C-1} \frac{\alpha_t(\boldsymbol{s})}{\prod_{\varsigma=1, \varsigma \neq c}^{C} \boldsymbol{b}_{i_\varsigma}^{\varsigma}(\boldsymbol{o}_t^{\varsigma})} \quad (11)$$

In this paper former definition is applied for evaluation process.

Authorized licensed use limited to: CSIRO Information Technology Services. Downloaded on September 09,2022 at 00:23:12 UTC from IEEE Xplore. Restrictions apply.

III. Approaches

Early detection in this work is actually a classification problem as proposed in [5], i.e., for each newly observed data we choose a model among several competing models and the likelihood evaluation from problem (1) allows us to choose the best match based on the observations [14]. The implementation details for these three methods are discussed in following paragraphs.

A. C-channel CHMM

Proposed CHMM based method consists of K models, each representing a certain class. If K equals two, then these classes can be the situations of absence and presence of an event. Decision about attribution of one model to a certain instant t will be made using a sliding window of length T. Every model has C channels, each channel produces a one dimensional observation sequence. Given the observations of a segment of size T, the marginal log likelihood of the kth model can be written as:

$$\mathcal{L}_t^k = logP(O_{t-T+1:t}|\lambda^k)$$
$$k = 1, \dots, K \quad (12)$$

where $O_{t-T+1:t}$ represents the observations of all channels in a segment from t-T+1 to t .

Detection of event α among K incidents is performed by comparing the difference between these log likelihoods to a certain threshold:

$$\mathcal{L}_t^\alpha - \mathcal{L}_t^k > \delta^{\alpha,k}$$
$$\forall k \in \{1,2,..K\} - \{\alpha\} \quad (13)$$

B. HMM/HSMM with C dimensional observation

HMM and HSMM based methods also have K models to present K prenominated possible situations. These models have a sequence of single hidden states. In this paper, multiple observations will be exploited in the HMM/ HSMM algorithm. Similar to what was stated in the previous section about model selection for every segment of observation, log likelihood for each model of HMM or HSMM is defined as in Eq.12. The only difference here is that each of HMM and HSMM models has one channel. This fact reduces the number of states and the complexity of transitions, which therefore reduces the computational load [5, 6]

IV. Evaluation

Inspiring from [6], the analysis have been done over simulated and real observation signals. The evaluation on simulated signals aims at optimizing and validating the proposed methods while experiments on the real signals acquired in NICU, for which apnea-bradycardia episodes are manually annotated, are used for performance evaluation. For event detection we use two models (presence and absence of the event) and for simplicity each model has two states per channel.

A. Simulated data

There is a need to generate two observation signals, to describe an event with potentially different dynamics. Synthetic time series, which are produced by Fitz Hugh-Nagumo model [6] have been used in our work.

$$\frac{dv}{dt} = 3\left(v - \frac{1}{3}v^3 + r + I\right)$$
$$\frac{dr}{dt} = -\frac{1}{3}(v - a + 0.8r) \quad (14)$$

Two groups of these signals, one consisting of rest intervals and the other having perturbation are used to train two models. Each group contains 40 signals of 10 seconds.

A group of 40 signals of 400s consisting both rest and perturbations intervals, are generated for simulation test. Log likelihoods of both models are evaluated from a sliding window of 10s.

B. Real data

RR-interval time series and QRS duration time series, extracted from ECG of 32 preterm infants, are the two-channel observations in the present study and we believe the explicit modelling and estimation of the coupling effect between the two channels might better take advantage of the system dynamics. These observations have been manually divided into two groups. The first group includes 148 segments of 7s in normal situation, and is used to train the normal model (in absence of AB events); the other group includes 30 segments of the same size containing bradycardia events and is used to train the other model (in presence of the AB events).

A set of 40 signals with different length are used as test data in order to evaluate the performance of the algorithms. A 7-second window slides sample by sample on the observation sequence for making a decision about occurrence of the bradycardia event at the sampled time t0.

For the CHMM approach, RR interval and the QRS duration, both extracted from the raw ECG signal are used as the two-channelled observation data. Since every channel utilizes a particular type of observation, its corresponding channel states can be defined as certain amplitude ranges. Typically, the 2-D HMM/HSMM algorithm including RR interval and QRS duration can't interpret certain dynamic characteristics since the observations consist of two incongruous emissions [5, 6].

C. Performance evaluation

The detection results with the annotated data for each of the three algorithms are compared. For this purpose, we define four parameters, i.e. true positive (TP), true negative (TN), false positive (FP) and false negative (FN). True positives is attributed to detections of the bradycardia occurrence within a 20s window, centered at the annotations.

Finally estimating sensitivity (SEN=TP/(TP+FN)) and specificity (SPC=TP/(TN+FP)) for different threshold values, leads to variant quantities of these measures which are represented by means of ROC curve. Time detection delay is also calculated. It is defined as the time elapsed between annotation instant and the detection. This definition produces a

Authorized licensed use limited to: CSIRO Information Technology Services. Downloaded on September 09,2022 at 00:23:12 UTC from IEEE Xplore. Restrictions apply.

negative value when detection situates before the annotated begin of bradycardia. Consequently there would be a number of time detection delays for each threshold. By estimating the average and standard deviations of these time detection delays, dd can be defined (dd= t_{Mean} $\pm t_{SD}$). Optimum performance detection and its respective threshold are evaluated using the criterion introduced as perfect detection (PD) according to Eq.15.

$$PD = max(SEN \times SPC) \tag{15}$$

V. Results

The average results over ten times of executions on simulated data are shown in table 1. Performance measures including sensitivity, specificity and detection delay are calculated for all three methods. Sensitivity and specificity values are acceptable and satisfying for all of them, however CHMM based method has the ability to detect earlier than the labelled onset of the event of interest.

Table 1 Performance measures by evaluating PD for simulated data

Variable	SEN(%)	SPC(%)	dd(seconds)
HMM [16]	98.37%	92.80%	0.81± 0.03
HSMM [17]	97.35%	97.93%	0.30± 0.02
CHMM [9]	97.43%	92.24%	0.69± 1.31

All three methods are also analyzed with a real database of 233 apnea-bradycardia episodes. Achieved results for sensitivity vs specificty are represnetd in ROC curve in Fig.2. The ROC curve of the CHMM proposed method is placed above two other curves which indicates the better performance of this method. The optimum points obtained using Eq.15 are shown with charachter “X” in Fig.2. Superiority of CHMM is also confirmed by PD points.

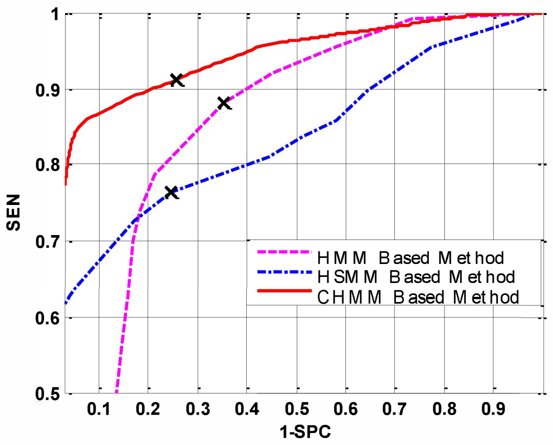

Fig. 2 ROC curves for sensitivity and specificity

Considering the importance of time detection delay in this type of prediction problem, obtained time detection delays versus specificity changes are depicted in Fig.3. Results clearly state that the earlier detection of apnea-bradycardia with a trustable level of accuracy is achieved by CHMM. Optimum points are also represented in this figure.

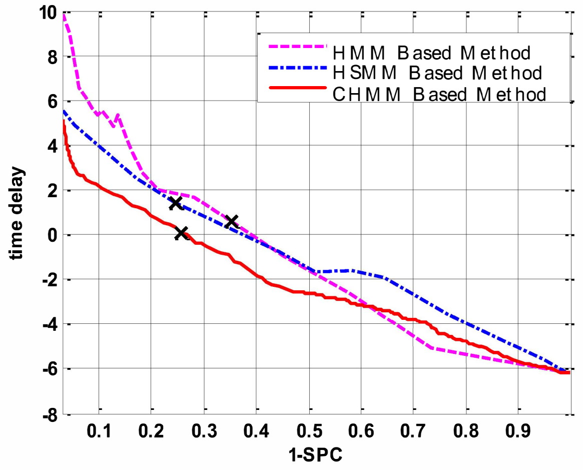

Fig. 3 Specificity and mean detection delay of apnea-bradycardia episodes.

Related values to the PD points, which are obtained for the thresholds of $\delta^{\alpha,k}$ =300, 3900, 27900 respectively, are listed in table.2. All performance measures for CHMM based method are better than two other algorithms. In particular, calculated detection delay and specificity of CHMM has a significant improvement in comparison with other models. It is worthy of noting that same amounts of specificity results in better time detection delay for CHMM as it can be seen in Fig.3.

Table 2 Performance measures by evaluating PD for real data

Variable	SEN(%)	SPC(%)	dd(seconds)
HMM [16]	78.68%	78.64%	1.99±6.7
HSMM [17]	63.79%	94.61%	6.58±10.59
CHMM [9]	84.92%	94.17%	2.32±4.82

VI. Conclusions

Evaluation results show a reduced time detection delay and higher detection performance in Coupled Hidden Markov Model based method rather than conventional detectors based on Hidden Markov Model and Hidden Semi Markov Model. Employing two channels instantaneously, to take into account their reciprocal effects on each other and construct a strong structure. These characteristics of CHMMs provide the capability of reliable performance and make them as strong tools for prediction in medical applications such as apnea-bradycardia detection. On the other hand, the order of calculations in current implementation of CHMM is $O(M^{2C}T)$ where $M = \max(I_1,...,I_C)$ which dramatically increases the calculation load by large number of channels. So our future

Authorized licensed use limited to: CSIRO Information Technology Services. Downloaded on September 09,2022 at 00:23:12 UTC from IEEE Xplore. Restrictions apply.

work will be dedicated to present a new more efficient method for CHMM framework.

ACKNOWLEDGEMENT

This work has been supported by the Center for International Scientific Studies and Collaboration (CISSC) and by Egide-Gundishapour program.

The authors would like to thank Dr. Iead Rezek for his guidance and comments.

REFERENCES

[1] Poets, C. F., Stebbens, V. A., Samuels, M. P., & Southall, D. P. (1993). The relationship between bradycardia, apnea, and hypoxemia in preterm infants. *Pediatric research*, 34(2), 144-147.

[2] Lowe, A., Jones, R. W., & Harrison, M. J. (2001). The graphical presentation of decision support information in an intelligent anaesthesia monitor. *Artificial Intelligence in Medicine*, *22*(2), 173-191.

[3] Pichardo, R., Adam, J. S., Rosow, E., Bronzino, J., & Eisenfeld, L. (2003). Vibrotactile stimulation system to treat apnea of prematurity. *Biomedical instrumentation & technology*, 37(1), 34-40.

[4] Cruz, J., Hernández, A. I., Wong, S., Carrault, G., & Beuchee, A. (2006, September). Algorithm fusion for the early detection of apnea-bradycardia in preterm infants. In *Computers in Cardiology, 2006* (pp. 473-476). IEEE.

[5] Altuve, M., Carrault, G., Beuchee, A., Flamand, C., Pladys, P., & Hernandez, A. I. (2012, September). Comparing hidden Markov model and hidden semi-Markov model based detectors of apnea-bradycardia episodes in preterm infants. In *Computing in Cardiology (CinC), 2012* (pp. 389-392). IEEE.

[6] Altuve, M., Carrault, G., Beuchée, A., Pladys, P., & Hernández, A. I. (2011, August). On-line apnea-bradycardia detection using hidden semi-Markov models. In *Engineering in Medicine and Biology Society, EMBC, 2011 Annual International Conference of the IEEE* (pp. 4374-4377). IEEE.

[7] Bunks, C., McCarthy D., Al-Ani T. (2000). Condition-based maintenance of machines using hidden Markov models. *Mechanical Systems and Signal Processing 14, no. 4, 2000*: (pp. 597-612).

[8] Baruah, P., & Chinnam, R. B. (2005). HMMs for diagnostics and prognostics in machining processes. *International Journal of Production Research*, *43*(6), 1275-1293.

[9] Rezek, I., & Roberts, S. J. (2000). Estimation of coupled hidden Markov models with application to biosignal interaction modelling. In Neural Networks for Signal Processing X, 2000. Proceedings of the 2000 IEEE Signal Processing Society Workshop (Vol. 2, pp. 804-813). IEEE. [source code from : http://www.robots.ox.ac.uk/~irezek/software.html]

[10] Dong, M., & He, D. (2007). A segmental hidden semi-Markov model (HSMM)-based diagnostics and prognostics framework and methodology. *Mechanical Systems and Signal Processing*, *21*(5), 2248-2266.

[11] Ching, W. K., & Ng, M. K. (2006). *Markov chains: Models, algorithms and applications* (Vol. 83). New York: Springer.

[12] Dong, M., & He, D. (2007). Hidden semi-Markov model-based methodology for multi-sensor equipment health diagnosis and prognosis. *European Journal of Operational Research*, *178*(3), 858-878.

[13] Nefian, A. V., Liang, L., Pi, X., Xiaoxiang, L., Mao, C., & Murphy, K. (2002, May). A coupled HMM for audio-visual speech recognition. In *Acoustics, Speech, and Signal Processing (ICASSP), 2002 IEEE International Conference on* (Vol. 2, pp. II-2013). IEEE.

[14] Rabiner, L. R. (1989). A tutorial on hidden Markov models and selected applications in speech recognition. *Proceedings of the IEEE*, *77*(2), 257-286.

[15] Baum, L. E., & Eagon, J. A. (1967). An inequality with applications to statistical estimation for probabilistic functions of Markov processes and to a model for ecology. *Bull. Amer. Math. Soc*, *73*(3), 360-363.

[16] http://www.cs.ubc.ca/~murphyk/Software/HMM/hmm.html

[17] Yu, S. Z. (2010). Hidden semi-Markov models. Artificial Intelligence, 174(2), 215-243.

Authorized licensed use limited to: CSIRO Information Technology Services. Downloaded on September 09,2022 at 00:23:12 UTC from IEEE Xplore. Restrictions apply.
