# Supplementary material for: Exploring Computational Techniques in Preprocessing Neonatal Physiological Signals for Detecting Adverse Outcomes: Scoping Review
Source: Interact J Med Res. 2024 Aug 20;13:e46946. doi: 10.2196/46946 (PMC11372324; doi:10.2196/46946)
Supplement: Multimedia Appendix 3 [file ijmr_v13i1e46946_app3.zip › Included Papers - Final/3810/Stanculescu et al. - A Hierarchical Switching Linear Dynamical System A.pdf]

A Hierarchical Switching Linear Dynamical System Applied to the Detection of Sepsis in Neonatal Condition Monitoring

Ioan Stanculescu
School of Informatics
University of Edinburgh
Edinburgh, UK, EH8 9AB

Christopher K.I. Williams
School of Informatics
University of Edinburgh
Edinburgh, UK, EH8 9AB

Yvonne Freer
Simpson Centre for Reproductive Health
Royal Infirmary of Edinburgh
Edinburgh, UK, EH16 4SA

Abstract

In this paper we develop a Hierarchical Switching Linear Dynamical System (HSLDS) for the detection of sepsis in neonates in an intensive care unit. The Factorial Switching LDS (FSLDS) of Quinn et al. (2009) is able to describe the observed vital signs data in terms of a number of discrete factors, which have either physiological or artifactual origin. In this paper we demonstrate that by adding a higher-level discrete variable with semantics sepsis/non-sepsis we can detect changes in the physiological factors that signal the presence of sepsis. We demonstrate that the performance of our model for the detection of sepsis is not statistically different from the auto-regressive HMM of Stanculescu et al. (2013), despite the fact that their model is given "ground truth" annotations of the physiological factors, while our HSLDS must infer them from the raw vital signs data.

1 INTRODUCTION

In condition monitoring, we are often interested in inferring when a dynamical system "switches" its mode of operation. Inside Neonatal Intensive Care Units (NICUs), one the most important "switches" is associated with the start of late onset neonatal sepsis (LONS). LONS is a bloodstream infection, usually bacterial, which generally occurs after the third day of life. It is a major cause of mortality, lifelong neurodisability and increased health care costs (Modi et al., 2009).

Since early clinical signs are subtle, making the diagnosis of infection is a great challenge. A deterioration of the baby's condition prompts clinicians to take a blood sample for laboratory testing. However, laboratory culture results can take up to a day before becoming available. This delay is known to prevent effective treatment (Griffin et al., 2003). Thus, a dependable early sepsis detector would have a major impact on NICU care. In this work, we discuss a solution which relies exclusively on vital signs monitoring data.

We propose a Hierarchical Switching Linear Dynamical System (HSLDS) to model a dynamical system with complex interactions between modes of operation. The structure of the model is shown in Fig. 1. In the HSLDS, the switch state is represented as a two-level discrete hierarchical structure. The top layer switch variables control the transition matrices used by the lower discrete layer, whose variables are assumed to be conditionally independent given the top layer. Conditioned on the hidden discrete structure, the model is a Linear Dynamical System (LDS), which models continuous hidden state variables and continuous observations. The observations are assumed to come from readings of the monitoring equipment.

The HSLDS can be applied for the real-world task of detecting neonatal sepsis. The discrete top layer determines the state of the infection and the lower-level discrete factors are baby-generated physiological events. The physiological events we monitor for sepsis detection are:

- *bradycardia:* a spontaneous fall in heart rate measurements (Figure 2a), and
- *desaturation:* a drop in the concentration of oxygen in arterial blood (Figure 2b).

The problem of detecting neonatal sepsis from monitoring data has been previously studied. Griffin et al. (2003) and Moorman et al. (2011) have found a positive skew in the inter-beat (RR) interval histograms in the hours before the clinical suspicion of sepsis, and an absence of skew during normal periods. They used this finding to build features subsequently fed to a logistic

regression classifier. However, this work does not use other vital signs apart from the heart rate and also assumes access to the high-frequency RR data. The work of Stanculescu et al. (2013) is probably closest to our approach. They propose using an auto-regressive HMM (AR-HMM) to capture trends of increased physiological event incidence. Unlike the HSLDS, their model uses annotations of physiological events as input, which limits the possibility of model deployment.

The main contributions of this work are: (i) to develop the FSLDS model of Quinn et al. (2009) into a HSLDS in a "deep learning" style by adding a set of higher-level variables to model correlations in the physiological factors in order to detect sepsis, and (ii) to demonstrate that the performance of our model for the detection of sepsis is almost as good as the auto-regressive HMM of Stanculescu et al. (2013), despite the fact that their model is given "ground truth" annotations of the physiological factors, while our HSLDS must infer them from the raw vital signs data.

The structure of the remainder of the paper is as follows: In Section 2 we describe the proposed model, and discuss inference, learning and related work. Section 3 explains how the HSLDS can be used to obtain early predictions about neonatal sepsis and inferences about clinical events. Experimental results are presented in Section 4 and we provide a discussion in Section 5.

2 THE HSLDS

In order to facilitate the introduction of our hierarchical model, we begin with a brief review of the Switching LDS (SLDS). The SLDS is a generative model for sequential data which switches between several different modes of operation. Each mode of operation is modelled as a LDS (Kalman filter), and thus the SLDS can be thought of as a dynamical mixture of LDS models. As the switch settings are hidden, often the main task is to recover them given the observations. Formally, at time t the SLDS has a discrete-continuous hybrid hidden state consisting of a hidden switch variable s_t and a hidden continuous state $\mathbf{x}_t \in \mathbb{R}^{d_x}$. This hybrid state attempts to explain how measurements $\mathbf{y}_t \in \mathbb{R}^{d_y}$ are generated. More precisely, the switch setting s_t determines the set of LDS parameters used at time t:

$$\mathbf{x}_t \sim \mathcal{N}(\mathbf{A}(s_t)\mathbf{x}_{t-1}, \mathbf{Q}(s_t)), \tag{1}$$

$$\mathbf{y}_t \sim \mathcal{N}(\mathbf{C}(s_t)\mathbf{x}_t, \mathbf{R}(s_t)), \tag{2}$$

where $\mathbf{A}(s_t)$ and $\mathbf{Q}(s_t)$ are the dynamics and dynamics noise covariance matrices, and $\mathbf{C}(s_t)$ and $\mathbf{R}(s_t)$ are the observation and observation noise covariance matrices. The switch settings are sampled from a Markov transition matrix $p(s_t|s_{t-1})$.

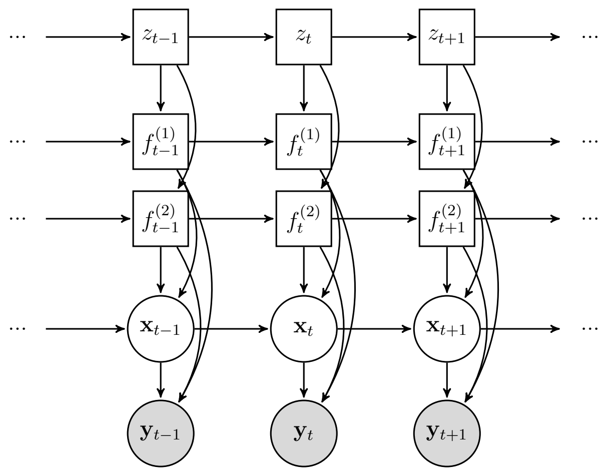

Figure 1: HSLDS with $K = 2$. Squares represent discrete variables and circles represent continuous ones. Shaded nodes are observed variables.

The FSLDS assumes a set of K discrete factors $f_t^{(1)}, f_t^{(2)}, \ldots, f_t^{(K)}$ are collectively affecting the data. The model is obtained by representing the switch variable of the SLDS as the cross product $f_t^{(1)} \otimes f_t^{(2)} \otimes ... \otimes f_t^{(K)}$. An important assumption made by the FSLDS is that the factors are a priori independent:

$$p(s_t|s_{t-1}) = \prod_{k=1}^{K} p(f_t^{(k)}|z_t, f_{t-1}^{(k)})$$

In the HSLDS, we propose relaxing this assumption by introducing a hierarchical structure for the discrete hidden variables. The discrete state is now represented by two layers of variables (see Figure 1). The top layer variable z_t controls the Markovian dynamics $p(f_t^{(\cdot)}|z_t, f_{t-1}^{(\cdot)})$ used by each factor. Conditional on the setting of the top layer switch variable z_t, the model becomes equivalent to an FSLDS. Thus, the HSLDS can be thought of as a dynamical mixture of FSLDS models. If we define a full expansion of the discrete hidden state as $s_t \triangleq z_t \otimes f_t^{(1)} \otimes f_t^{(2)} \otimes ... \otimes f_t^{(K)}$, then the joint distribution of the HSLDS can be written as:

$$p(s_{1:T}, \mathbf{x}_{1:T}, \mathbf{y}_{1:T}) = p(s_1)p(\mathbf{x}_1|s_1)p(\mathbf{y}_1|\mathbf{x}_1, s_1) \prod_{t=2}^{T} p(s_t|s_{t-1})p(\mathbf{x}_t|\mathbf{x}_{t-1}, s_t)p(\mathbf{y}_t|\mathbf{x}_t, s_t), \tag{3}$$

where

$$p(s_1) = p(z_1) \prod_{k=1}^{K} p(f_1^{(k)}|z_1),$$

$$p(s_t|s_{t-1}) = p(z_t|z_{t-1}) \prod_{k=1}^{K} p(f_t^{(k)}|z_t, f_{t-1}^{(k)}),$$

$s_{1:T} \triangleq s_1, s_2, \ldots, s_T$, and $\mathbf{x}_{1:T}$ and $\mathbf{y}_{1:T}$ are similarly defined.

Note that the top hidden layer is conditionally independent of the continuous variables given the factor settings: $\mathbf{x}_{1:T}, \mathbf{y}_{1:T} \perp\!\!\!\perp z_{1:T} | \mathbf{f}_{1:T}$, where we have defined $\mathbf{f}_t \triangleq [f_t^{(1)}, f_t^{(2)}, \ldots, f_t^{(K)}]$. This simplifies both learning and inference.

2.1 RELATED WORK

The basic SLDS model has a long history, see e.g. Shumway and Stoffer (1991), and has been used in many applications. Factorization of the SLDS discrete state gives the Factorial Switching LDS (FSLDS), which has been used for neonatal condition monitoring by Williams et al. (2006) and Quinn et al. (2009), in speech recognition (Deng, 2006) and in music transcription (Cemgil et al., 2006). This mirrors the development of the factorial hidden Markov model (FHMM) of Ghahramani and Jordan (1997) from the standard HMM.

The HMM model has also been elaborated hierarchically by Fine and Singer (1998) to give the hierarchical hidden Markov model (HHMM). A similar construction can be used to create a hierarchical switching LDS (HSLDS). The only previous example of this model we are aware of in the literature is the work of Zoeter and Heskes (2003) which used a HSLDS for visualization of time-series data. Their motivation is to allow a successive refinement of a visualization, starting from projecting onto a single LDS with a two-dimensional (2-d) hidden space. This can be broken down into a SLDS of 2-d LDSes, and then each 2-d LDS can be further independently decomposed into a SLDS. Thus, a set of lower-level states correspond to one higher-level state. Also note that their use case involves interaction from the user to initialise the decomposition.

In contrast, we more naturally think of building our model bottom up, first identifying a set of factors for the FSLDS, and then modelling their correlations with a top-level variable. Notice that in our work the state of the top-level variable affects all of the second-level variables below it.

There are also some similarities between our work and the paper by Taylor et al. (2010). Under their approach, the $\mathbf{x}$ dynamics are modelled by an Implicit Mixture of Conditional Restricted Boltzmann Machines (imCRBM). This is similar to us in that the CRBM part of the model uses a number of discrete latent variables (analogous to our $\mathbf{f}$'s) to affect the $\mathbf{x}$ dynamics. The implicit mixture variable (analogous to our z) switches between different dynamics models. Of course, the details of the model are quite different as it is in part undirected, and that there are no explicit discrete latent variable chains through time, instead these variables "hang off" the $\mathbf{x}$ chain.

2.2 INFERENCE

Since real-time inference is the major concern in physiological condition monitoring, we are mainly interested in marginal filtering distributions. More precisely, we require sepsis predictions of the form $p(z_t|\mathbf{y}_{1:t})$ and clinical event posteriors $p(f_t^{(\cdot)}|\mathbf{y}_{1:t})$. These marginal posteriors can be immediately obtained from the filtering distribution of the fully expanded state $p(s_t|\mathbf{y}_{1:t})$. Thus, running SLDS inference suffices for HSLDS inference. Note that the more general goal of SLDS filtering is inferring $p(\mathbf{x}_{1:t}, s_{1:t}|\mathbf{y}_{1:t})$.

Exact SLDS inference requires computing Gaussian mixtures with a number of components exponential in the length of the sequence. Clearly, this is computationally intractable for most practical purposes (Lerner and Parr, 2001). Several approximate SLDS inference methods have been previously proposed: Gaussian sum approximations (Murphy, 1998; Barber and Mesot, 2007), Rao-Blackwellised Particle Filtering (Murphy and Russell, 2001; de Freitas et al., 2004), variational inference (Ghahramani and Hinton, 2000) or expectation propagation (Zoeter and Heskes, 2003).

Here, we apply the Gaussian Sum approximation described in Murphy (1998). The method ensures tractability by using moment matching to collapse a Gaussian mixture onto a single Gaussian. At any time step, each $p(\mathbf{x}_t|s_t, \mathbf{y}_{1:t})$ is approximated by a single Gaussian, which corresponds to $p(\mathbf{x}_t|\mathbf{y}_{1:t})$ being approximated by a mixture of Gaussians.

When the hidden discrete state is a cross-product of variables, we can speed up inference by allowing at most one variable to change its setting at each time step. This procedure has been previously discussed in Quinn et al. (2009) or Kolter and Jaakkola (2012).

A particular aspect of the baby monitoring application is the presence of several missing data sources. The treatment of this problem will be discussed in detail in Section 3.3.

2.3 LEARNING

HSLDS learning is similar to FSLDS learning to a large extent. Here, we first emphasize the most significant common aspects and then discuss HSLDS learning specifics.

For our application we assume that there are a number of interpretable regimes for which labelled data are available. Labelled data for the HSLDS model are of

the form $\{\mathbf{y}_t, z_t, f_t^{(1)}, f_t^{(2)}, \ldots, f_t^{(K)}\}$.

As in the FSLDS case, the availability of labelled data makes learning equivalent to learning one LDS model for each switch setting. In general, we parameterise LDS dynamics as autoregressive processes and use Expectation Maximisation (EM) for training (Ghahramani and Hinton, 1996). Learning is performed independently for each factor, and then the fitted parameters are carefully combined for each switch setting. This procedure is greatly simplified by considering the interactions between factors. For instance, the activation of one factor might "overwrite" any effect of another factor on certain observation channels. In the neonatal monitoring application, domain knowledge is used to define a factor overwriting ordering, as further discussed in Section 3.2.

For the HSLDS in particular, we use the conditional independence between the continuous variables and the top layer discrete variables to further simplify learning. This means that the (parameters of the) continuous variable distributions (eqs. 1 and 2) do not depend on the setting of z_t.

A straightforward way of learning the Markov transition matrices for individual factors $p(f_t^{(\cdot)}|z_t, f_{t-1}^{(\cdot)})$ would be to make use of the labelled data and maximize the conditional likelihood $p(\mathbf{f}_{1:T}|z_{1:T})$. Estimates of the factor transition probabilities have the form:

$$p(f_t^{(\cdot)} = j | z_t = l, f_{t-1}^{(\cdot)} = i) = \frac{n_{ijl} + n_0}{\sum_{j'}(n_{ij'l} + n_0)}, \quad (4)$$

where n_{ijl} is the number of transitions from state i to state j for factor $f^{(\cdot)}$ under the z-regime l counted over all the training data. The constant count n_0 comes from placing a Dirichlet prior which prevents probabilities from being too close to zero.

However, we have found that an alternative "deep learning" style method can give rise to better results (Section 4.1). Although the $\mathbf{f}$ data is available at training time, at test time these labels must be inferred from the $\mathbf{y}$ data. Hence it makes sense to build a model which looks at the actual inferences of the factors, rather than the ground truth labels.

If $\mathbf{Y}$ is the training set of sequences and the corresponding $\mathbf{F}$ are treated as hidden variables, we could use EM and attempt to optimise $p(\mathbf{Y}|\mathbf{Z})$. The M-step is equivalent to maximizing the expected complete data log likelihood:

$$\mathcal{Q} = \mathbb{E}_{p(\mathbf{X},\mathbf{F}|\mathbf{Y},\mathbf{Z})} \log p(\mathbf{Y}, \mathbf{X}, \mathbf{F}|\mathbf{Z}), \quad (5)$$

where $p(\mathbf{X}, \mathbf{F}|\mathbf{Y}, \mathbf{Z})$ was computed in the preceding E-step using the old parameter settings. Factor transition estimates are of the from:

$$p(f_t^{(\cdot)} = j | z_t = l, f_{t-1}^{(\cdot)} = i) = \frac{\tilde{n}_{ijl} + n_0}{\sum_{j'}(\tilde{n}_{ij'l} + n_0)}, \quad (6)$$

where

$$\tilde{n}_{ijl} = \sum_t p(f_{t-1}^{(\cdot)} = i, f_t^{(\cdot)} = j | \mathbf{Y}, \mathbf{Z}) I(z_t = l)$$

is commonly referred to as a "soft" data count, I is the indicator function, and the sum is taken over all t's in the training data.

Running EM until convergence is likely to be unsatisfactory, as there are no guarantees that the learnt factor transition matrices would produce good factor posteriors. Our solution is to approximate $p(\mathbf{F}|\mathbf{Y}, \mathbf{Z})$, by $p_{FSLDS}(\mathbf{F}|\mathbf{Y})$. Here, the FSLDS model is trained using the standard learning routine of Quinn et al. (2009) and the factor models discussed in Section 3.2, and is thus unaware of the existence of multiple z-regimes. In practice, we found it sufficient to obtain "soft" counts of pairwise filtering marginals $p_{FSLDS}(f_{t-1}^{(\cdot)}, f_t^{(\cdot)}|\mathbf{y}_{1:t})$ for each training sequence. Since FSLDS posteriors do not depend on the learnt HSLDS parameters, the method is non-iterative.

This procedure follows ideas in the "deep learning" literature (Hinton et al., 2006) where layer-wise training of a model is carried out. Similar ideas can also be found e.g. in Karklin and Lewicki (2005) or Farhadi et al. (2009), although in all these cases the models are not for time series.

Finally, estimates of the Markov transition matrix $p(z_t|z_{t-1})$ are learnt from the z-labels. Also note that in the absence of the labelled data, unsupervised learning for the full model would be possible using EM.

3 AN HSLDS FOR NEONATAL CONDITION MONITORING

This section is concerned with applying the HSLDS for condition monitoring in NICUs. We begin with a brief description of baby monitoring, focusing on the early detection of neonatal sepsis. We then explain how the problem can be solved by formulating it as learning and inference in an HSLDS.

3.1 NICU MONITORING AND SEPSIS DETECTION

NICU babies are born several months prematurely and are intrinsically unstable. They are nursed in incubators, and their vital signs are continuously displayed on bedside screens. Clinicians apply their expertise to interpret patterns in the monitoring traces and use

this information in support of their diagnostic inference. The task is challenging for reasons including the amount, dimensionality and frequency of the data, and the need to analyse patient physiology across multiple time scales.

The present application focuses on the early detection of neonatal sepsis based on the information contained in the monitoring data. The hypothesis is that an increased incidence of baby generated physiological events is a symptom of sepsis. In current clinical practice, the laboratory result of a blood culture is taken taken as the "gold standard" for diagnosing neonatal sepsis. Here, we adopted the laboratory result interpretation proposed by Modi et al. (2009) and also discussed by Stanculescu et al. (2013).

The measurement channels used in this work monitor several vital physiological systems. The heart rate measures the cardiovascular system. It is available from two sources: the ECG leads - HR (beats per minute - bpm) and the pulse oximeter - PR (bpm). The core and peripheral temperatures, TC (°C) and TP (°C), monitor the thermoregulatory system. The saturation of oxygen in arterial blood, SO (%), reflects the evolution of the respiratory system. All channels are sampled second-by-second (1Hz).

Our data samples are monitoring windows with a duration of 30 hours, and fall into either a sepsis group or a control group. Sepsis samples have been chosen such that the time the positive blood sample was collected occurs precisely 24 hours after the start of the window. For control samples, there was no suspicion of sepsis in a consecutive 3 day period around the selected windows, and no blood sample had been taken for laboratory testing.

3.2 LEARNING A SEPSIS DETECTION MODEL

We now detail how the baby monitoring HSLDS is trained. We first discuss parameter fitting for the continuous variable distributions and then continue with learning the hidden discrete layers of the HSLDS.

Learning continuous variable distributions

A natural classification of the regimes appearing in the NICU monitoring application is: *stability*, *known factors* and *unknown factors*.

Babies within the NICU are in a stable condition for much of the time, generally being asleep and motionless. We call this regime *stability* and separately fit univariate LDSes to each measurement channel. Thus, the dynamics parameters $\mathbf{A}$ and $\mathbf{Q}$ will have a block diagonal structure (see Quinn et al. (2009) for details).

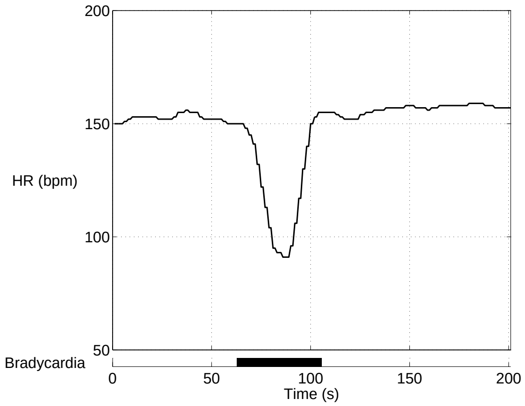

(a) Bradycardia

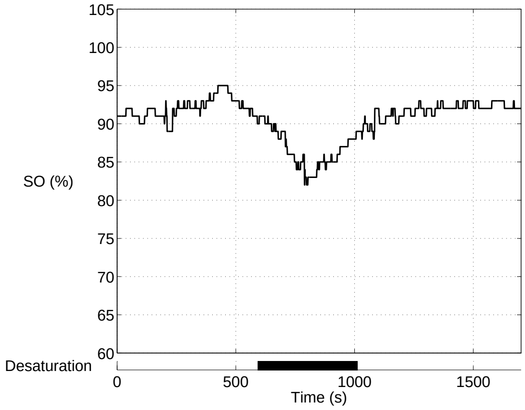

(b) Desaturation

Figure 2: Examples of physiological events. They are notable for the lack of artifact.

When clinical events associated with stereotypical patterns occur on the monitoring traces, the regimes will be referred to as *known factors*. Here, we model two physiological events: braydcardias and desaturations (see Figure 2 for examples). Both are characterised by a drop in the monitored signal (a slowing of the heart rate for bradycardias, and a decrease in the saturation of oxygen in arterial blood for desaturations), after which measurements rise back. We model these factors as two-stage events. The first stage corresponds to measurements dropping and can be explained by an exponential decay, the discrete time equivalent of which is an $AR(1)$ process. To set the mean of the decay process we first compute the empirical distribution F of minimum channel measurements during events. The quantile q^* of F corresponding to $F(q^*) = 0.05$ is chosen to be the decay mean. In the second stage of the event the measurements rise back. This will be referred to as the recovery stage. Recovery dynamics are also modelled as an $AR(1)$ process, where the mean is now the same as the channel's *stability* mean. The

Table 1: Overwriting Ordering of Factors

Channel	Bradycardia	Desaturation	X	Stability
HR	•		•	•
SO		•	•	•

parameters for both decay and recovery models are learnt by running EM, where we chose the dynamics initialisation $\mathbf{A} = \mathbf{0}$.

Finally, certain events cannot be explained by either stability or by any of the known factors. These patterns represent either novel dynamics or their low incidence makes them impractical to model as known factors. Here, we follow the approach of Quinn et al. (2009), where they propose a factor explaining these "known *unknowns*", the X-factor. It shares the same dynamics matrix with stability, but uses an inflated system noise covariance matrix. As the X-factor can claim patterns of both physiology and artifact, we do not use it directly for inferring the presence of sepsis.

Once the factor models have been separately learnt, they are combined using the overwriting order shown in Table 1. For each measurement channel, factors placed towards the left of the table overwrite factors placed towards the right.

Learning discrete variable distributions

In the baby monitoring application the top discrete layer of the HSLDS models the state of the sepsis infection. Here, we assume z_t is a binary variable taking on values $z_t = sepsis$ or $z_t = normal$. We first explain how labels of the form $\{\mathbf{y}_t, z_t\}$ have been defined. We then discuss how these labels are used to train the HSLDS's discrete variable layers.

The task of providing labels for the sepsis indicator variable is non-trivial. For patients in the sepsis group, clinicians only hold records for the exact time of the positive blood test. It is almost certain that the onset of the infection occurred in the hours prior to this time stamp. However, the onset cannot be assumed to be an instantaneous switch. The following labelling scheme has been proposed for samples belonging to the sepsis group; see Stanculescu et al. (2013). First, a period of 6 hours before the time of the positive blood test is labelled as *sepsis*. Second, we introduce a transition period during which the baby progresses from being in the *normal* state to being in the *sepsis* state. The transition period is defined as the 12 hour interval between 18 and 6 hours before the positive test. We do not assign a label for this period and it is not used for either training or testing the discrete layers of the HSLDS. Third, the monitoring data before the transition period (i.e. the first 6 hours of a sample in the sepsis group) is labelled as *normal*. Fourth, we do not assign a label to data after the positive test, as these measurements are likely to be affected by the patient's response to treatment and have less relevance for the task of real-time sepsis detection. Finally, all the data in the control group is assigned the *normal* label.

Table 2: Missing Data Sources Affecting Baby-generated Physiological Events.

	Bradycardia	Desaturation
Handling	•	•
Oximeter error		•
HR dropout	•	
SO dropout		•

Using the sepsis labels, an estimate of $p(z_t|z_{t-1})$ can be directly obtained using data counts. For learning the z-conditioned *known* factors' transition matrices, we apply the procedure explained in Section 2.3; see eq. 5 and the surrounding text . The X-factor's incidence is assumed to be independent of the state of the infection, and thus the factor transition matrix is copied from the previously learnt FSLDS.

3.3 INFERENCE WITH MISSING DATA

We reiterate that this work is centred on the idea of monitoring baby-generated bradycardias and desaturations in order to predict sepsis. However, there are periods of time during which labels for these events cannot be provided even by an expert annotator. We will treat such periods as missing data. There are three distinct sources of missing data: probe dropouts, oximeter errors and patient handling. We first describe these sources and then explain how inference can be performed during such periods.

During probe dropouts measurements are not available due to either malfunctioning or temporary removal of the monitoring devices. They can be readily recognised by the zero values on the recorded channels.

An oximeter error occurs when there is a disagreement between the HR and PR traces. This indicates a temporary unreliability of the SO trace, and thus the impossibility to monitor desaturations. Here, we adopt the approach in Stanculescu et al. (2013), where an automated oximeter error detection algorithm has been applied as a preprocessing step.

Patients are regularly handled by clinical staff (e.g. for changing nappies). During such episodes, we usually see an increased variability in the monitoring channels and often patterns of bradycardia or desaturation. We cannot distinguish whether such instances are caused merely by handling an extremely fragile baby, or they actually reflect the patient's true

Table 3: Population Demographics: Gestation, Birth Weight (BW) and Post Partum Age

Group	Statistic	Gestation	BW	Age
Sepsis	mean	27.2 weeks	873 gr	14.5 days
	std.dev.	1.5 weeks	256 gr	8.5 days
Control	mean	26.7 weeks	837 gr	15.2 days
	std.dev.	1.7 weeks	139 gr	14 days

state of health. Thus, for sepsis detection we analyse only physiological events happening outside handling episodes. Our work still relies on having expert annotations for handling. Quinn et al. (2009) have shown that these episodes can be inferred by monitoring environmental channels such as the incubator's humidity, but such channels have not been available in this work.

Table 2 shows how physiological events are affected by the presence of each missing data source.

For running inference with missing data, we extend the ideas in Quinn et al. (2009). Whenever a missing data source is present, the measurements do not carry information about the true physiology of the patient, and thus should not influence the hidden state estimates. The latter continue to evolve according to the dynamics equations, but without measurement update. Technically, rows of the observation matrix are set to zero whenever there is missing data on the corresponding measurement channel. For these channels the Kalman gain will be zero. Thus, the corresponding hidden continuous state dimensions will be estimated with increasing uncertainty before reaching the stable state of the Kalman filter.

4 EXPERIMENTS

This section describes the experiments we have performed to assess the neonatal condition monitoring model introduced in Section 3. The detection of sepsis is discussed in Section 4.1. Section 4.2 is concerned with the quality of physiological event posteriors.

The dataset we use in this work consists of data collected exclusively from very low birth weight patients (VLBW, birth weight < 1500 grams). It has been previously used by Stanculescu et al. (2013), and contains 36 monitoring samples equally split between the sepsis and the control groups. All sepsis samples come from different patients. In the control group we have two samples from each of 9 different babies. Three patients have samples in both groups, corresponding to a total of 24 different patients. The demographics of the two groups are shown in Table 3.

Expert annotations have been obtained for all the data. A summary of the annotation process is provided in Table 4. The total amount of data for each group is $18 \times 30 = 540$ hours and only baby generated physiological events have been considered. Importantly, the incidence of baby generated bradycardias and desaturations is higher in the sepsis group. As expected, the differences for the X-factor are much smaller. In terms of missing data sources, the amounts of handling and oximeter error are similar between patient groups. Probe dropout statistics are different for each channel, but on average we lack observations for 2% of the time. In addition, a *stability* period of $15-30$ minutes was marked near the start of each sample.

Table 4: Clinical Event Incidence

Event	Group	Incidence	Total	Median
Bradycardia	Sepsis	1718	24 hrs	39 sec
	Control	1133	12 hrs	35 sec
Desaturation	Sepsis	738	32 hrs	101 sec
	Control	231	11 hrs	132 sec
X-factor	Sepsis	226	10 hrs	94 sec
	Control	171	7 hrs	114 sec
Handling	Sepsis	204	44 hrs	530 sec
	Control	210	55 hrs	592 sec
Ox. err.	Sepsis	4051	45 hrs	16 sec
	Control	3395	36 hrs	18 sec

In order to reduce bias, we test our predictions using N-fold cross-validation. Considering the size of our dataset we decided to use $N = 9$ folds. Each fold contains 4 data samples, 2 from each patient group. The 2 control samples are chosen such that they belong to the same patient. Apart from these constraints, the folds have been randomly chosen.

4.1 SEPSIS DETECTION

To gain a better understanding of the HSLDS's effectiveness, we compare its predictions against filtering results obtained with the AR-HMM model of Stanculescu et al. (2013). While the HSLDS infers the posterior distributions of bradycardias and desaturations, the AR-HMM uses expert annotations of these events as input. Note that in the AR-HMM it was possible to run inference exactly and also marginalise over the missing data exactly. For the purposes of this work, the central question is how well the HSLDS inferences match the AR-HMM ones.

In the following we discuss two HSLDS models. The HSLDS learnt as explained in Section 2.3 will be referred to as HSLDSdeep. We will compare it against an HSLDS where the factor transitions for baby-generated events are learnt directly from the expert annotations, HSLDSkf (known factors).

We provide the second-by-second sepsis inferences produced by both the AR-HMM and HSLDSdeep in Fig-

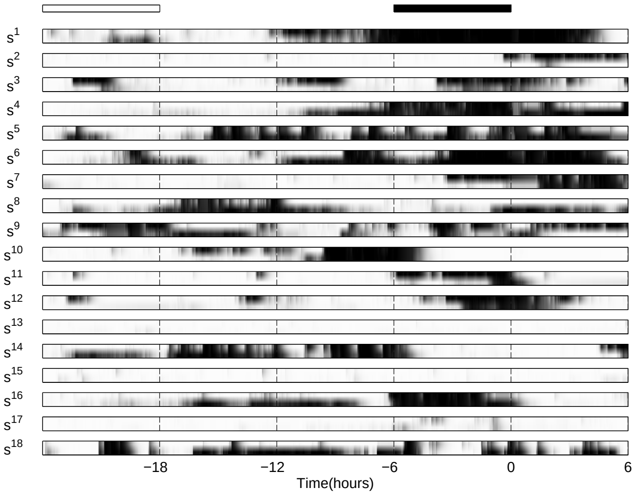

(a) Sepsis group

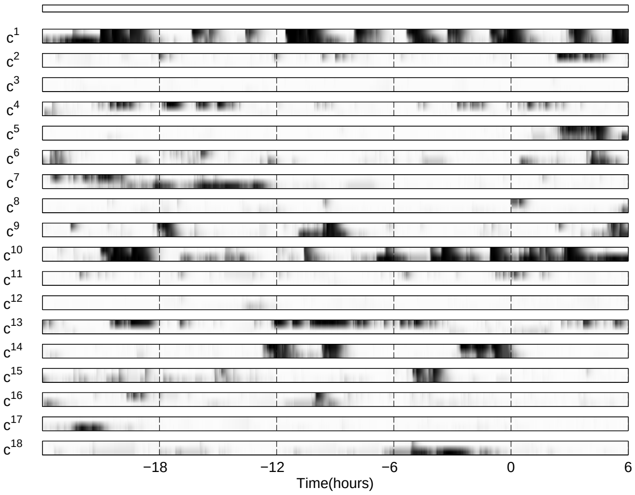

(b) Control group

Figure 3: Sepsis filtering distributions obtained using 9-fold Cross-Validation. On the x-axis, 0 denotes the time the positive blood sample was taken. For each group, the top row represents the sepsis labelling: normal periods are white (probability 0), sepsis periods are black (probability 1); transitioning and treatment periods are not assigned labels. For each data sample the top row corresponds to the AR-HMM model, the bottom row corresponds to HSLDSdeep.

Table 5: Sepsis Inference Summaries Using 9-fold Cross-Validation

	Second-by-second		Episode-based	
Model	AUC	EER	AP	F-score
AR-HMM	0.72	0.34	0.62	0.65
HSLDSdeep	0.69	0.37	0.51	0.54
HSLDSkf	0.62	0.41	0.45	0.47

ure 3. In general, there is strong correlation between the predictions of the two models and we find the inferences of HSLDSdeep to be a good match to the AR-HMM ones. However, in samples s^2, s^7 and s^{11} HSLDSdeep detects sepsis noticeably later than the AR-HMM, and in samples , s^4 and s^6 it does so earlier. In the control group, HSLDSdeep does slightly worse on samples c^7 and c^{18}, but outperforms the AR-HMM on samples c^4 and c^{13}.

For quantifying those results, we project the inferences onto two different metrics. This opens the possibility to reveal different aspects of performance.

Firstly, we are mainly interested in the *second-by-second* inferences produced by our hierarchical models and use the z-labels to draw ROC curves. The AUC (area under the ROC curve) and EER[1] computed by aggregating predictions over folds are shown in Table 5. Compared to HSLDSkf, HSLDSdeep produced results much closer to the AR-HMM benchmark.

We obtained more insight into how the HSLDS predictions compare against the AR-HMM results via an N-fold cross-validated paired t test on the AUC. We found the performance difference between the AR-HMM and our proposed HSLDSdeep model not to be statistically significant ($p = 0.552$). This is a good indication that the HSLDSdeep model can be used instead of the AR-HMM, and thus significantly reduce the need for expert input needed to detect sepsis. At the same time the performance difference between the AR-HMM and the HSLDSkf model is statistically significant ($p = 0.0064$). This suggests HSLDSkf should not be used instead of the AR-HMM.

Secondly, we analyse the inferred *episodes* of infection and draw precision-recall (PR) curves. This analysis has been proposed by Stanculescu et al. (2013), where they argue that it could be more relevant in clinical practice than a second-by-second one. Here we report average precision (AP) and the maximum F-score (see Table 5). Again, the performance of HSLDSdeep is closer to the AR-HMM than the HSLDSkf.

4.2 PHYSIOLOGICAL EVENT POSTERIORS

We can obtain filtering distributions for physiological events by marginalising the sepsis variable from

[1]EER is the error rate computed for the threshold at which the false positive rate (FPR) equals the false negative rate (FNR).

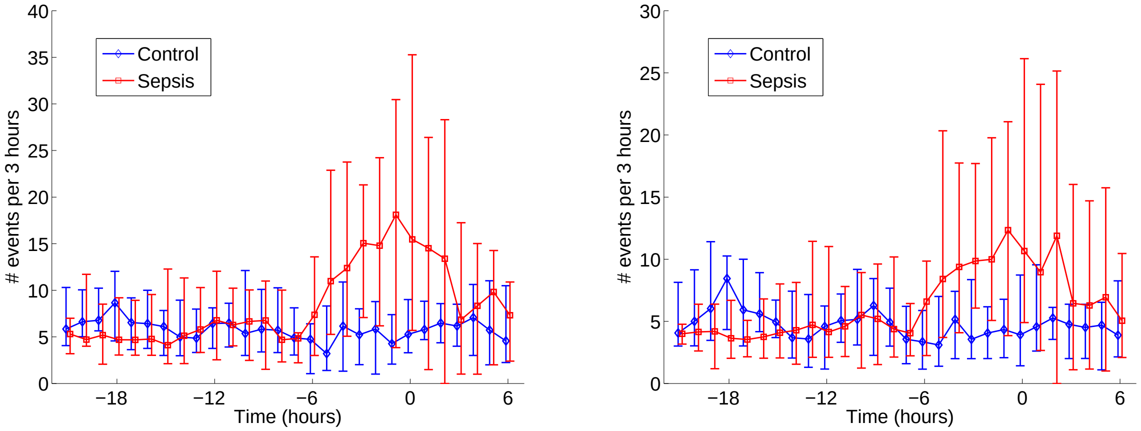

(a) Labelled events

(b) Inferred Events

Figure 4: Median weighted number of true and inferred bradycardias separately computed for each patient group. The counts were computed hourly and summarize the preceding 3 hour period. Error bars mark first and third quartiles. The small offset between the two patient groups was used to improve readability.

Table 6: Factor Inference Summaries Using 9-fold Cross-Validation

		Brady.	Desat.	X
FSLDS	AUC	0.85	0.81	0.63
	EER	0.21	0.28	0.40
HSLDSdeep	AUC	0.86	0.82	0.60
	EER	0.21	0.27	0.42

HSLDS posteriors. As we have labelled data for the predicted factors, we can we compare HSLDS posteriors against FSLDS ones. Summary results computed by aggregating predictions obtained with 9-fold cross-validation are shown in Table 6. Even though the FSLDS has been trained solely for inferring clinical events, there is very little difference between the performance of the two models.

Bradycardia and X-factor inferences obtained using an FSLDS have been previously assessed in (Quinn et al., 2009). The bradycardia results reported here are very similar to that work, but X-factor predictions are worse. Results on oxygen desaturation have not been previously reported.

We also found it interesting to compare the true incidence of baby-generated physiological events against the inferred one. For this purpose we obtained inferred events by binarising factor posteriors. Figure 4 shows a comparative visualisation of the time evolution of annotated and inferred bradycardias. The counts have been weighted in accordance to the amount of missing data in the analysed 3 hour periods. On both plots, there is a clear increase in the incidence of bradycardias in the hours before the sepsis diagnosis.

5 CONCLUSION

In this paper, we have proposed a framework for condition monitoring in situations when the factors that govern the data can be organised in a hierarchy. The structure of our model allows domain knowledge to be naturally incorporated. In addition, we have described a "deep learning" inspired training method.

The effectiveness of our model has been demonstrated for the difficult task of detecting the onset of sepsis in NICU patients. When compared against an AR-HMM model which heavily relies on expert annotations, we found the performance difference not to be statistically significant.

The are several directions in which this work could be extended. It would be interesting to run (H)SLDS smoothing, e.g. as described by Barber and Mesot (2007). This would prove useful both as a retrospective analysis of sepsis detection, and for refining our approach to learning factor transitions. Explicit modelling of event duration could improve the results, as demonstrated by Stanculescu et al. (2013). While we showed that the HSLDS performs similarly to the AR-HMM, sepsis predictions still need improvement. Finally, the X-factor predictions indicate more work could be done on novelty detection.

Acknowledgements

We would like to thank Prof. Neil McIntosh for providing expert insight and supervising with data annotation. Author IS was funded by the Scottish Informatics and Computer Science Alliance and the Engineering and Physical Sciences Research Council.

References

Barber, D. and Mesot, B. (2007). A Novel Gaussian Sum Smoother for Approximate Inference in Switching Linear Dynamical Systems. In Schölkopf, B., Platt, J., and Hoffman, T., editors, *Advances in Neural Information Processing Systems 19*, pages 89–96. MIT Press, Cambridge, MA.

Cemgil, A. T., Kappen, H. J., and Barber, D. (2006). A generative model for music transcription. *IEEE Transactions on Audio, Speech and Language Processing*, 14(2):679–694.

de Freitas, N., Dearden, R., Hutter, F., Morales-Menendez, R., Mutch, J., and Poole, D. (2004). Diagnosis by a waiter and a Mars explorer. *Proceedings of the IEEE*, 92(3):455–468.

Deng, L. (2006). *Dynamic Speech Models: Theory, Algorithms, and Applications*. Synthesis Lectures on Speech and Audio Processing. Morgan & Claypool Publishers.

Farhadi, A., Endres, I., Hoiem, D., and Forsyth, D. (2009). Describing objects by their attributes. In *Proceedings of the IEEE Computer Society Conference on Computer Vision and Pattern Recognition (CVPR)*.

Fine, S. and Singer, Y. (1998). The Hierarchical Hidden Markov Model: Analysis and Applications. In *Machine Learning*, pages 41–62.

Ghahramani, Z. and Hinton, G. E. (1996). Parameter Estimation for Linear Dynamical Systems. Technical report, University of Toronto.

Ghahramani, Z. and Hinton, G. E. (2000). Variational Learning for Switching State-Space Models. *Neural Computation*, 12(4):831–864.

Ghahramani, Z. and Jordan, M. I. (1997). Factorial Hidden Markov Models. *Machine Learning*, 29:245–273.

Griffin, M. P., O'Shea, T. M., Bissonette, E. A., Harrell, F. E., Lake, D. E., and Moorman, J. R. (2003). Abnormal Heart Rate Characteristics Preceding Neonatal Sepsis and Sepsis-Like Illness. *Pediatr Res*, 53(6):920–6.

Hinton, G. E., Osindero, S., and Teh, Y. W. (2006). A fast learning algorithm for deep belief nets. *Neural Computation*, 18:1527–1554.

Karklin, Y. and Lewicki, M. S. (2005). A hierarchical Bayesian model for learning non-linear statistical regularities in non-stationary natural signals. *Neural Computation*, 17(2):397–423.

Kolter, J. Z. and Jaakkola, T. (2012). Approximate Inference in Additive Factorial HMMs with Application to Energy Disaggregation. In Lawrence, N. D. and Girolami, M., editors, *AISTATS*, volume 22 of *JMLR Proceedings*, pages 1472–1482. JMLR.org.

Lerner, U. and Parr, R. (2001). Inference in hybrid networks: Theoretical limits and practical algorithms. In *UAI*, pages 310–318.

Modi, N., Doré, C. J., Saraswatula, A., Richards, M., Bamford, K. B., Coello, R., and Holmes, A. (2009). A case definition for national and international neonatal bloodstream infection surveillance. *Archives of Disease in Childhood - Fetal and Neonatal Edition*, 94(1):F8–F12.

Moorman, J. R., Carlo, W. A., Kattwinkel, J., Schelonka, R. L., Porcelli, P. J., Navarrete, C. T., Bancalari, E., Aschner, J. L., Walker, M. W., Perez, J. A., Palmer, C., Stukenborg, G. J., Lake, D. E., and OShea, T. M. (2011). Mortality Reduction by Heart Rate Characteristic Monitoring in Very Low Birth Weight Neonates: A Randomized Trial. *The Journal of Pediatrics*, 159(6):900 – 906.e1.

Murphy, K. and Russell, S. (2001). Rao-Blackwellised Particle Filtering for Dynamic Bayesian Networks. In A. Doucet, N. d. F. and Gordon, N., editors, *Sequential Monte Carlo in Practice*. Springer-Verlag.

Murphy, K. P. (1998). Switching Kalman Filters. Technical report, U.C. Berkeley.

Quinn, J. A., Williams, C. K. I., and McIntosh, N. (2009). Factorial Switching Linear Dynamical Systems Applied to Physiological Condition Monitoring. *IEEE Trans. Pattern Anal. Mach. Intell.*, 31(9):1537–1551.

Shumway, R. and Stoffer, D. (1991). Dynamic linear models with switching. *J. of the American Statistical Association*, 86:763–769.

Stanculescu, I., Williams, C. K. I., and Freer, Y. (2013). Autoregressive Hidden Markov Models for the Early Detection of Neonatal Sepsis. *Biomedical and Health Informatics, IEEE Journal of*. DOI 10.1109/JBHI.2013.2294692.

Taylor, G. W., Sigal, L., Fleet, D., and Hinton, G. E. (2010). Dynamic binary latent variable models for 3D human pose tracking. In *Proceedings of the IEEE Conference on Computer Vision and Pattern Recognition 2010*.

Williams, C. K. I., Quinn, J. A., and McIntosh, N. (2006). Factorial Switching Kalman Filters for Condition Monitoring in Neonatal Intensive Care. In Weiss, Y., Schölkopf, B., and Platt, J., editors, *Advances in Neural Information Processing Systems 18*. MIT Press.

Zoeter, O. and Heskes, T. (2003). Hierarchical visualization of time-series data using switching linear dynamical systems. *IEEE Transactions on Pattern Analysis and Machine Intelligence*, 25:1201–1214.
