# Supplementary material for: Exploring Computational Techniques in Preprocessing Neonatal Physiological Signals for Detecting Adverse Outcomes: Scoping Review
Source: Interact J Med Res. 2024 Aug 20;13:e46946. doi: 10.2196/46946 (PMC11372324; doi:10.2196/46946)
Supplement: Multimedia Appendix 3 [file ijmr_v13i1e46946_app3.zip › Included Papers - Final/3832/Shirwaikar et al. - 2016 - Machine Learning Techniques for Neonatal Apnea Pre.pdf]

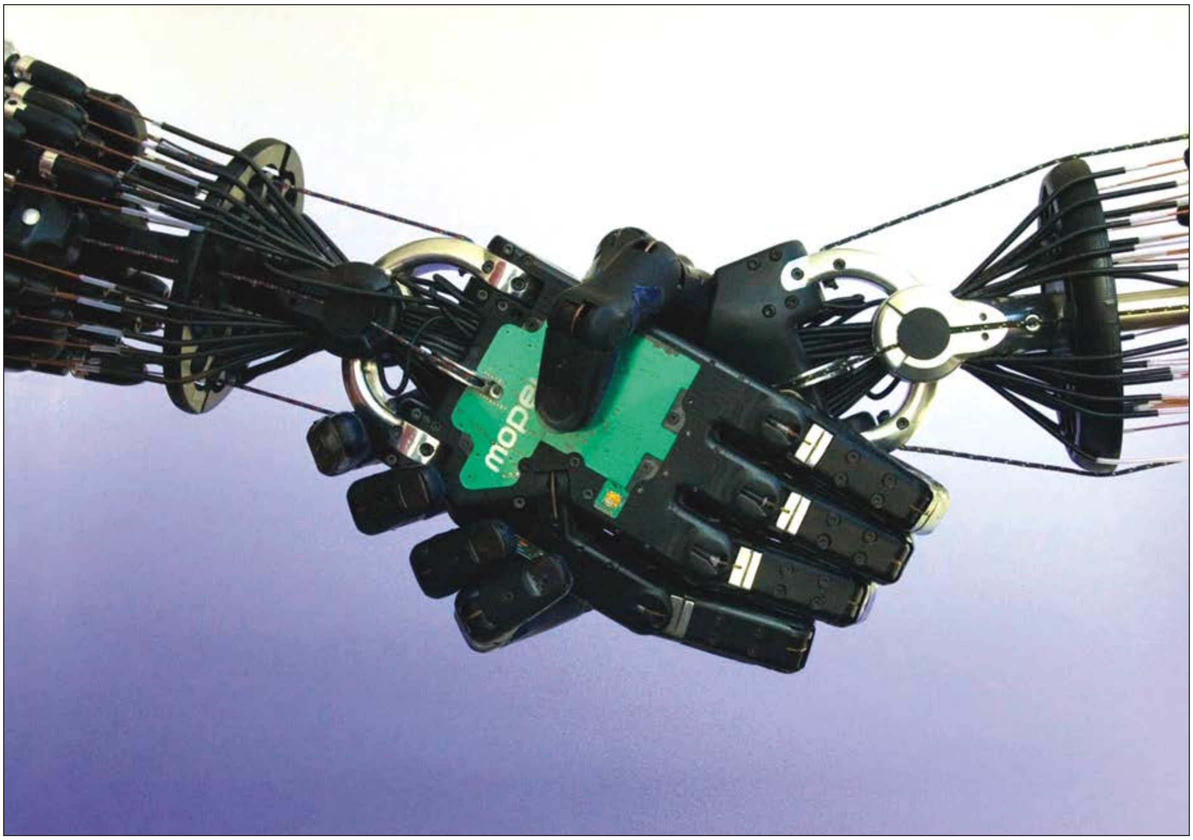

Journal of Artificial Intelligence

ISSN 1994-5450

science alert

ANSInet
an open access publisher
http://ansinet.com

OPEN ACCESS Journal of Artificial Intelligence

ISSN 1994-5450
DOI: 10.3923/jai.2016.33.38

Research Article
Machine Learning Techniques for Neonatal Apnea Prediction

[1]Rudresh D. Shirwaikar, [1]U. Dinesh Acharya, [l]Krishnamoorthi Makkithaya, [2]M. Surulivelrajan and [3]Leslie Edward Simon Lewis

[1]Department of Computer Science and Engg, MIT, Manipal University,
[2]Departmrnt of Pharmacy Practice, MCOPS, Manipal University,
[3]Department of Pediatrics, KH Manipal University, Manipal, India

Abstract

Background and Objective: Machine learning has been widely accepted and applied in different fields to analyze data, but it is still novel in the field of neonatal diseases, especially neonatal apnea prediction. Apnea is a breathing problem associated with pathological changes in heart rate and oxygen saturation and is a common occurrence in neonates especially those who are born preterm. This study is focused on prediction of apnea episodes during the first week of child's birth using machine learning algorithms. **Materials and Methods:** The data consists of 229 examples of neonates admitted to Neonatal Intensive Care Unit (NICU) of Kasturba hospital, Manipal, Karnataka, India. This data is preprocessed and used to develop classification model using machine learning techniques such as decision tree (C5.0), Support Vector Machine (SVM) and ensemble approach, which includes random forest for prediction of apnea episodes. **Results:** The study compares models (decision tree, SVM and ensemble approach such as random forest) for accuracy. Among the results obtained, an accuracy of 0.88 and kappa of 0.72 using random forest algorithm for mtry three is found to be the most accurate model. **Conclusion:** The research work provides an automated machine learning based solution that helps clinicians predict apnea in neonates during the first week of their life. Inclusion of contextual information and preprocessing technique along with heterogeneous ensemble approach may further improve the models performance.

Key words: Machine learning, neonatal apnea, resampling techniques, support vector machine ensemble approach, bagging, boosting, random forest

Received: March 08, 2016 Accepted: April 25, 2016 Published: June 15, 2016

Citation: Rudresh D. Shirwaikar, U. Dinesh Acharya, Krishnamoorthi Makkithaya, M. Surulivelrajan and Leslie Edward Simon Lewis, 2016. Machine learning techniques for neonatal apnea prediction. J. Artif. Intel., 9: 33-38.

Corresponding Author: Rudresh D. Shirwaikar, Department of Computer Sciences and Engineering, AB-5, Manipal Institute of Technology, Manipal-576104, Karnataka, India

Copyright: © 2016 Rudresh D. Shirwaikar *et al*. This is an open access article distributed under the terms of the creative commons attribution License, which permits unrestricted use, distribution and reproduction in any medium, provided the original author and source are credited.

Competing Interest: The authors have declared that no competing interest exists.

Data Availability: All relevant data are within the paper and its supporting information files.

INTRODUCTION

A child in his first hundred days of life is called a neonate. Babies born prior to 37 weeks of gestation are considered premature or preterm[1]. Nearly 80% of neonatal death occurs within first week of birth and as many as 2.9 million children die in the first month of their birth in India every year, according to World Health Organization (WHO) survey[2]. Neonatal Intensive Care Unit (NICU) is a place where neonate's physiological parameters are continuously monitored for knowing their health conditions. Physiological parameters such as heart rate, respiration rate and blood oxygen levels are vital for monitoring neonatal health which needs to be checked at regular intervals.

Apnea, defined as cessation of breathing resulting in pathological changes in heart rate and oxygen saturation, is common occurrence in sick neonates[3]. Apnea in preterm is related to immaturity of the central nervous system and is called Apnea Of Prematurity (AOP). The most widely used definition of AOP specifies a pause in breathing for more than 15-20 sec, or accompanied by oxygen desaturation (blood oxygen saturation (SpO_2) $\leq$80% for $\geq$4 sec) and bradycardia (heart rate <100 per minute for $\geq$4 sec), in infants born less than 37 weeks of gestation[4]. Neonates born less than 34 weeks of gestation should be monitored for the first week of life till the absence of all apneic episodes. Whereas, for the neonates born with more than 34 weeks gestation continuous monitoring is done only if they are found sick.

Machine learning has been widely used in health domain for prediction of hospitalization[5], cancer prognosis with risk assessment after surgery[6], identify the frequent diseases using apriori algorithm[7], predicting breast cancer survivability[8] and neonatal disease prediction and prognosis. Supervised learning techniques such as support vector machines, artificial neural network, decision tree, K-nearest neighbor etc., have been used in neonatal disease diagnosis and prediction of jaundice[9], respiratory distress syndrome[10-12] metabolic disorder and apnea of prematurity[13]. Williamson *et al.*[14] have proposed algorithms for neonatal apnea prediction based on cardio respiratory and movement signals with statistical classifier such as Gaussian mixture model on a limited set of examples.

The goal of this study is to use supervised learning techniques for prediction of apnea episodes at the end of first week of neonatal life with physiological and other investigated variables. This study uses data analysis techniques for exploring, understanding and managing data with correlation analysis and the learning vector quantization (LQR) model for selecting the important features. Decision tree (C5.0) and Support Vector Machine (SVM) are used for classifying neonates with presence or absence of apnea episodes. In order to solve the "Class imbalance" problem discussed in Zeng and Gao[15], the study uses concepts such as under and oversampling of data. Lastly to achieve better diversity and improve accuracy ensemble approach has been used.

MATERIALS AND METHODS

This section will briefly describe the overall methodology, which is divided into four modules as shown in Fig. 1.

Data collection from NICU: Required data is collected from Neonatal Intensive Care Unit (NICU), Kasturba hospital, Manipal, Karnataka, India. Ethical approval has been obtained from Institutional Ethics committee of Manipal University. The data includes 229 examples of neonatal apnea, each with 23 features. The class label is defined with 'yes' as presence of apnea episodes and 'no' as absence. The 22 numeric features consist of (a) Demographic such as gestation age, growth categorization, birth cry, birth weight, (b) Maternal covariates includes delivery mode, surfactant and steroids etc. and (c) Physiological such as heart rate on 24, 48 and 72 h time frame etc.

Data exploration and preprocessing: Data exploration uses a combination of summary statistics such as mean, median, variance, counts and visualization techniques. Problems revealed by data summaries in the apnea data set includes missing values, invalid values or outliers, range and units. In fact visualization and graphical techniques are used to identify these problems in the data. Visualization techniques identify the problems within the data and further examines the distribution of numeric variables. Preprocessing steps include data cleaning, transformation, normalization, feature selection and sampling for modelling and validation.

Machine learning algorithm for prediction: This study has used decision tree (C5.0) and support vector machine algorithm for prediction of apnea episodes. The C5.0 uses information gain as a splitting criteria to build a decision tree and post pruning approach for balancing over-fitting and under-fitting of a tree. It can handle numeric or nominal features, missing data besides using only most important features for classification. Support vector machine with features such as generalizability, nonlinear functionality,

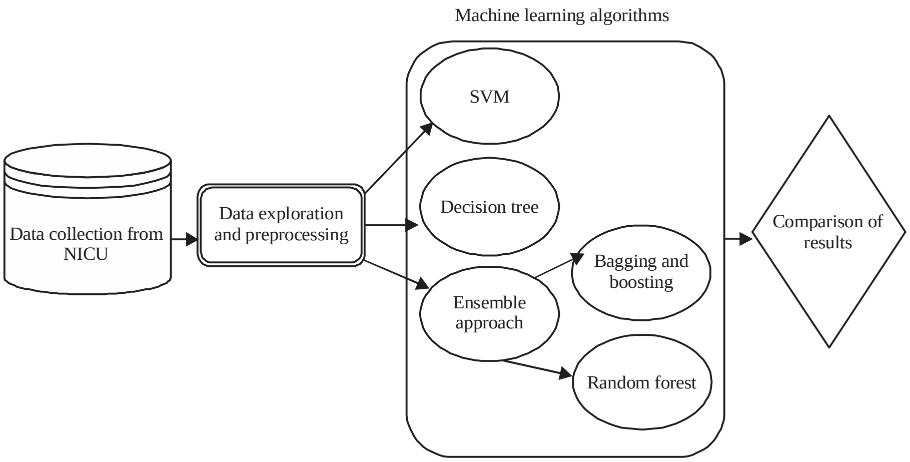

Fig. 1: Overall methodology

efficiency in handling noisy data and with strength in data regularization is also considered to be suitable for the problem.

Improving accuracy using ensemble approach: Imbalanced data set been the major problem identified in the current study which is nothing but presence of rare positive examples and numerous negative ones in the dataset. Techniques which are used to handle the problem include resampling of positive class called over sampling, random elimination of tuples from negative class called under sampling and ensemble techniques. Ensemble techniques used in this study are: (a) Bagged decision tree with default 25 decision trees which uses a voting technique to create an ensemble, (b) Auto tuned boosted C5.0 with additional trial parameters and (c) Random forest with a default ensemble of 500 trees.

RESULTS

For the purpose of this study, all algorithms were developed using R, a statistical computing and data analysis tool. Based on the exploring and preprocessing process described in the previous section, collected data from NICU was preprocessed as per the requirement of the algorithm. As apnea data set consists of features having fewer percentage of missing values, the columns had not been dropped and the missing values were not replaced with mean of the column total. However, for categorical features, a new category with value 0 was added for missing values. For features wherein the relationship between input and output is not linear, discretization techniques were applied for continuous variables. This study had used normalization techniques such as min max normalization and Z score normalization since data distribution is roughly symmetrical. Feature selection was performed by removing redundant features and ranking feature by importance. Linear vector quantization model a "Filter approach" was used to estimate variable importance based on ROC curve. Importance values in Fig. 2 revealed that heart rate on day 1, gestation age and delivery mode attributes were the top three most important attributes in the dataset and the head circumference and echo attributes were the least important ones. Once the feature selection was done and data being preprocessed, it was given as input to the classification model. The data set was divided into two different randomized sets called training set and testing set with 70:30 representative ratio. Next section describes the algorithms used for the prediction of apnea episodes with its comparison and results: (A) C5.0 algorithm and SVM using radial kernel (B) Ensemble approach: Bagging, boosting and random forest.

Decision tree (C5.0) algorithm and SVM using radial kernel: Evaluation was on training data using C5.0 includes 152 cases with 22 predictors. The model correctly classified all but 19 of the 152 training instances for an error rate of 12.5%. Decision trees are known to over fit the model to the training data. For this reason, the error rate reported on the training data may be overly optimistic and it is especially important to evaluate decision trees on the test data set. Table 1 represents the confusion matrix where out of 76 test records, model correctly predicted that 54 have no apnea episodes and 3 have apnea, resulting in an accuracy of 0.75 and error rate of 0.25. Sensitivity of the model was 0.20 which means model could not predict a true positive rate efficiently, i.e. the presence of apnea in neonates. Figure 3 represents visualization of decision tree with relevant features such as gestation age, heart rate, feeding intolerance, echo, birth weight and maternal covariates (surfactant and dexa2).

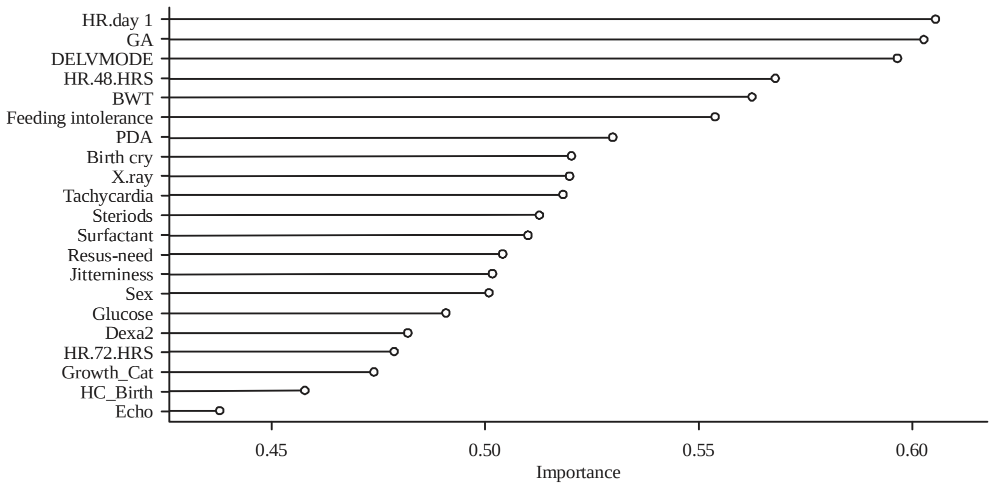

Fig. 2: Selection of features based on importance using ROC (AUC)

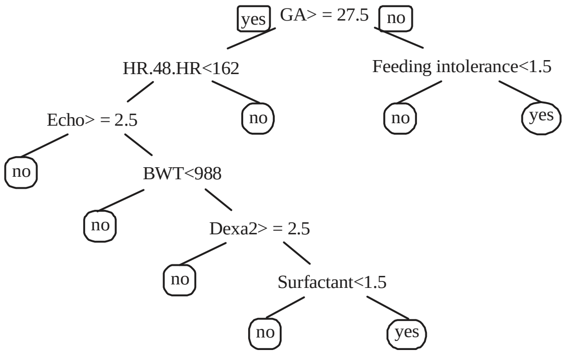

Fig. 3: Representing resulting decision tree

Building a model using SVM deals with right choice of kernel with its best parameter values and C a user predefined cost penalty parameter function. Choice of user defined penalty parameter C has an impact on the accuracy of the classifier. Here we had used radial kernel with 10 fold cross validation method to select the best parameter values. Model was trained with randomized training set using radial kernel with parameter values of gamma (0.01) and cost (10). Based on the confusion matrix represented in Table 2 accuracy of the model found was 0.75, with sensitivity of 0.28 and specificity of 0.72.

To improve the accuracy further, the apnea data set was resampled for effective generation of relatively balanced class distribution. Resampling techniques such as "Under sampling" and "over sampling" methods were used for the comparison of decision trees and SVM as shown in Table 3. The resampled data set consists of: (a) Under sampled method which creates a subset of the original dataset by randomly or selectively deleting some of the samples of the majority class while keeping the original population of the minority class (Dataset of 96 examples with 50 'no' and 48 'yes') and (b) Over sampling methods generate a superset of the original data set by replicating some of the samples of the positive class i.e., Systematic Minority Oversampling Technique (SMOT)[15], which combines informed oversampling of the minority class and random under sampling of the majority class (Dataset of 228 examples with 136 'no' and 92 'yes').

Table 1: Confusion matrix for decision tree (C5.0)

	Predicted		
Actual	No	Yes	Row total
No	54 (TN)	7 (FP)	61
Yes	12 (FN)	3 (TP)	15
Column total	66	10	76

Table 2: Confusion matrix for SVM

	Predicted		
Actual	No	Yes	Row total
No	52 (TN)	6 (FP)	58
Yes	13 (FN)	5 (TP)	18
Column total	65	10	76

Table 3: Decision tree and SVM based models

Algorithm	Data set	Accuracy	Sensitivity	Specificity
Decision tree (C5.0)	Apnea dataset	0.75	0.20	0.88
	Under sampled	0.47	0.42	0.50
	Over sampled	0.69	0.65	0.70
SVM	Apnea dataset	0.75	0.28	0.72
	Under sampled	0.44	0.31	0.64
	Over sampled	0.77	0.61	0.97

Ensemble approach: Bagging, boosting and random forest: Bagged tree is created with a default of 25 decision trees and

Table 4: Results of random forest

mtry	Accuracy	Kappa
3	0.88	0.72
6	0.86	0.69
11	0.86	0.69
22	0.85	0.69

Table 5: Results of boosted C5.0 model

Trials	Accuracy	Kappa
10	0.77	0.49
20	0.77	0.48
30	0.77	0.49
40	0.78	0.50

Table 6: Comparing current work with literature

	Williamson *et al.*[13,14]	Current study
Dataset	6 preterm neonates	229 neonates admitted to NICU
Category	Apnea of prematurity	Neonatal apnea
Features used for prediction	Physiological parameters (heart rate and respiration rate)[13] Physiological parameters and movement features[14]	Demographic, maternal, physiological parameters and other investigated parameters
Classifiers	Statistical classifiers (equal prior quadratic classifier) Statistical classifiers (Gaussian mixture model)	Random forest with mtry = 3
Utility	Accuracy[13]-moderate predictive strength of 50% Accuracy[14]-strong predictive strength of 80%	Accuracy of 88% with Kappa of 0.72

uses a voting technique to create an ensemble. With Kappa statistics of 0.69 and with accuracy of 0.85, the bagged tree model performs better than C5.0 decision tree. The random forest by default creates an ensemble of 500 trees that consider square root of feature at each split. After the ensemble of trees is generated the model uses a voting technique to combine the tree predictions. To get the most accurate comparison of model performance, 10 fold cross validation was used with a tuning grid for the random forest. The only tuning parameter for this model was mtry, which is an integer specifying the number of features randomly selected at each split. The random forest uses square root feature function to select the mtry with a grid values of 3, 6, 11 and 22. Kappa was used to select the optimal model using the largest value. The final value used for the model was mtry = 3 as shown in Table 4. Lastly the results of random forest were compared with boosted C5.0 decision tree with 10, 20, 30, 40 trials shown in Table 5. Accuracy was used to select the optimal model using the largest Kappa value. Among all 8 models with accuracy = 0.88 and kappa = 0.72, random forest with mtry = 3 was found to be the most accurate model.

DISCUSSION

Accuracy of the apnea data set with SVM as well as decision tree is found to be moderate but sensitivity is low as data being highly imbalanced. With under sampled data the accuracy and specificity decreases with slight increase in sensitivity. Further with oversampled data the accuracy, sensitivity and specificity remains to be moderate. From the results it is inferred that a large data set containing a balanced sample improves the utility of the model. Further the result of random forest method with mtry = 3 is found to be the most accurate model on the apnea data set used in this study. The current work is compared with the work of Williamson *et al.*[13,14] as shown in Table 6.

CONCLUSION

Currently in the field of health care, clinicians manually diagnose apnea in neonates and subsequently administer treatment. The study provides an automated machine learning-based solution that helps clinicians “Predict” apnea in neonates during the first week of their life. Furthermore the use of ensemble techniques to improve the prediction accuracy has also been demonstrated. Class imbalance problem is found to be the major issue in the dataset used for predicting apnea. In fact resampling, under sampling, over sampling and ensemble approaches can lead to higher accuracy of the algorithms in the case of class imbalance. Indeed bagging stabilizes decision trees and improves accuracy by reducing variance and further it can reduce generalization errors. Random forest improves decision tree performance by de-correlating individual trees in the bagging ensembles and also with variable importance measure it can help in determining which variables are contributing the most strongly to the model. Future study includes: (a) Applying heterogeneous ensemble approaches on the apnea data set

and subsequently compare with current study results and (b) Reusing the proposed machine learning model to predict other neonatal diseases such as jaundice, respiratory distress syndrome, sepsis etc.

ACKNOWLEDGMENT

The authors are deeply indebted to Kasturba Hospital, Manipal University for providing an opportunity to work on the research problem.

REFERENCES

1. Goodwin, L. and S. Maher, 2000. Data mining for preterm birth prediction. Proceedings of the 2000 ACM Symposium on Applied Computing, Volume 1, March 19-21, 2000, Como, Italy, pp: 46-51.
2. WHO., 2014. Every newborn: An action plan to end preventable deaths. World Health Organization (WHO), Geneva, pp: 2-16.
3. Aggarwal, R.K., A. Singhal, K. Ashok, K. Deorari and K. Pao, 2012. Apnea in the newborn. Indian J. Pediatr., 68: 858-962.
4. Lee, H., C.G. Rusin, D.E. Lake, M.T. Clark and L. Guin *et al*., 2012. A new algorithm for detecting central apnea in neonates. Physiol. Meas., 33: 1-7.
5. Dai, W., T.S. Brisimi, W.G. Adams, T. Mela, V. Saligrama and I.C. Paschalidis, 2015. Prediction of hospitalization due to heart diseases by supervised learning methods. Int. J. Med. Inf., 84: 189-197.
6. Oliveira, T., E. Barbosa, S. Martins, A. Goulart, J. Neves and P. Novais, 2013. A prognosis system for colorectal cancer. Proceedings of the 26th International Symposium on Computer Bases Medical Systems, June 20-22, 2013, Porto, pp: 481-484.
7. Ilayaraja, M. and T. Meyyappan, 2013. Mining medical data to identify frequent diseases using Apriori algorithm. Proceedings of the International Conference on Pattern Recognition, Informatics and Mobile Engineering, February 21-22, 2013, Karaikudi, India, pp: 194-199.
8. Delen, D., G. Walker and A. Kadam, 2005. Predicting breast cancer survivability: A comparison of three data mining methods. Artif. Intell. Med., 34: 113-127.
9. Ferreira, D., A. Oliveira and A. Freitas, 2012. Applying data mining techniques to improve diagnosis in neonatal jaundice. BMC Med. Inform. Decision Making, Vol. 12. 10.1186/1472-6947-12-143
10. Mikhno, A. and C.M. Ennett, 2012. Prediction of extubation failure for neonates with respiratory distress syndrome using the MIMIC-II clinical database. Proceedings of the Annual International Conference of the IEEE Engineering in Medicine and Biology Society, August 28-September 1, 2012, San Diego, CA., pp: 5094-5097.
11. Precup, D., C.A. Robles-Rubio, K.A. Brown, L. Kanbar and J. Kaczmarek *et al*., 2012. Prediction of extubation readiness in extreme preterm infants based on measures of cardiorespiratory variability. Proceedings of the Annual International Conference of the IEEE Engineering in Medicine and Biology Society, August 28-September 1, 2012, San Diego, CA., pp: 5630-5633.
12. Mueller, M., J.S. Almeida, R. Stanislaus and C.L. Wagner, 2013. Can machine learning methods predict extubation outcome in premature infants as well as clinicians? J. Neonatal Biol., Vol. 2. 10.4172/2167-0897.1000118
13. Williamson, J.R., D.W. Browne, P. Indic, E. Bloch-Salisbury and D. Paydarfar, 2013. Individualized apnea prediction in preterm infants using cardio-respiratory and movement signals. Proceedings of the IEEE Conference on Body Sensor Networks, May 6-9, 2013, Cambridge, MA., USA., pp: 1-6.
14. Williamson, J.R., D.W. Bliss, D.W. Browne, P. Indic, E. Bloch-Salisbury and D. Paydarfar, 2011. Using physiological signals to predict apnea in preterm infants. Proceedings of the Conference Record of the 45th Asilomar Conference on Signals, Systems and Computers, November 6-9, 2011, Pacific Grove, CA., pp: 1098-1102.
15. Zeng, Z.Q. and J. Gao, 2009. Improving SVM Classification with Imbalance Data Set. In: Neural Information Processing, Leung, C.S., M. Lee and J.H. Chan (Eds.). Springer Science and Business Media, New York, ISBN: 9783642106767, pp: 389-398.
