# Supplementary material for: Exploring Computational Techniques in Preprocessing Neonatal Physiological Signals for Detecting Adverse Outcomes: Scoping Review
Source: Interact J Med Res. 2024 Aug 20;13:e46946. doi: 10.2196/46946 (PMC11372324; doi:10.2196/46946)
Supplement: Multimedia Appendix 3 [file ijmr_v13i1e46946_app3.zip › Included Papers - Final/3845/Montazeri Ghahjaverestan et al. - 2015 - Switching Kalman filter based methods for apnea br.pdf]

Physiological Measurement

IPEM
Institute of Physics and Engineering in Medicine

PAPER

Switching Kalman filter based methods for apnea bradycardia detection from ECG signals

To cite this article: Nasim Montazeri Ghahjaverestan *et al* 2015 *Physiol. Meas.* **36** 1763

View the article online for updates and enhancements.

You may also like

- Use of high frequency analysis of acoustic emission signals to determine rolling element bearing condition
  A Cockerill, K M Holford, T Bradshaw et al.

- A silicone based piezoelectric and electromagnetic hybrid vibration energy harvester
  Tashfeen Ali and Farid Ullah Khan

- Analysis of electrical motor mechanical failures due to bearings
  R Karakolev and L Dimitrov

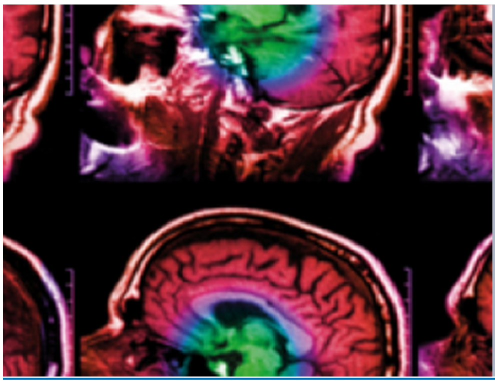

IPEM | IOP
Series in Physics and Engineering in Medicine and Biology

Your publishing choice in medical physics, biomedical engineering and related subjects.

Start exploring the collection–download the first chapter of every title for free.

This content was downloaded from IP address 140.79.77.175 on 09/09/2022 at 06:05

IOP Publishing | Institute of Physics and Engineering in Medicine Physiological Measurement

Physiol. Meas. **36** (2015) 1763–1783 doi:10.1088/0967-3334/36/9/1763

Switching Kalman filter based methods for apnea bradycardia detection from ECG signals

Nasim Montazeri Ghahjaverestan[1,2], Mohammad B Shamsollahi[1], Di Ge[2,3] and Alfredo I Hernández[2,3]

[1] Biomedical Signal and Image Processing Laboratory (BiSIPL), School of Electrical Engineering, Sharif University of Technology, Tehran, Iran
[2] The LTSI, University of Rennes 1, Rennes, F-35000, France
[3] INSERM, UMR 1099, Rennes, F-35000, France

E-mail: montazeri.nasim@gmail.com

Received 3 February 2015, revised 30 April 2015
Accepted for publication 20 May 2015
Published 3 August 2015

Abstract

Apnea bradycardia (AB) is an outcome of apnea occurrence in preterm infants and is an observable phenomenon in cardiovascular signals. Early detection of apnea in infants under monitoring is a critical challenge for the early intervention of nurses. In this paper, we introduce two switching Kalman filter (SKF) based methods for AB detection using electrocardiogram (ECG) signal.

The first SKF model uses McSharry's ECG dynamical model integrated in two Kalman filter (KF) models trained for normal and AB intervals. Whereas the second SKF model is established by using only the *RR* sequence extracted from ECG and two AR models to be fitted in normal and AB intervals. In both SKF approaches, a discrete state variable called a switch is considered that chooses one of the models (corresponding to normal and AB) during the inference phase. According to the probability of each model indicated by this switch, the model with larger probability determines the observation label at each time instant.

It is shown that the method based on ECG dynamical model can be effectively used for AB detection. The detection performance is evaluated by comparing statistical metrics and the amount of time taken to detect AB compared with the annotated onset. The results demonstrate the superiority of this method, with sensitivity and specificity 94.74% and 94.17%, respectively. The presented approaches may therefore serve as an effective algorithm for monitoring neonates suffering from AB.

 © 2015 Institute of Physics and Engineering in Medicine Printed in the UK

Keywords: switching Kalman filter (SKF), Kalman filter (KF), electrocardiography (ECG), ECG modelling, bradycardia, expectation-maximization (EM)

(Some figures may appear in colour only in the online journal)

1. Introduction

Apnea is one of the most common complications of premature birth in infants, which can affect the cardiac rhythm if it continues for more than a few seconds. Apnea bradycardia (AB) is an arrhythmia associated with decreased heart rate (HR), hence it is likely that it can be detected from succeeding ECG signal. Decreased levels of blood oxygen and vagal nerve stimulation (Zhao *et al* 2011) are the main causes of bradycardia. Other symptoms in the morphology of heartbeats, such as reduction of the QRS amplitude and its width prolongation, are also expected to be observed. Recent research has focused on the early detection of apnea from electrocardiogram (ECG) employing strong algorithms based on features extracted from all of its common symptoms (Altuve *et al* 2009, Haskova *et al* 2013). Accordingly, neonatal intensive care units (NICUs) are usually equipped with alarms which employ such detection algorithms. When an apnea event arises, the alarm is intended to alert nurses. There exists a time limit for nursing intervention for preterm infants in need. The more the response time is extended, the more difficult it is to stop the episode of AB leading to significant problems and consequences that may be irreversible. The average response time delay, measured from the activation of the alarm until the application of the therapy, was estimated around 33 s, with an average of 13 s of tactile stimulation for the AB event suppression (Pichardo *et al* 2003). Hence the algorithm that activates the alarm has a crucial role in the early detection of respiratory arrest.

Although a lot of research conducted to detect apnea employs respiration signals (Varady *et al* 2004), the most appropriate sensor for acquiring bio-signals, especially in neonates, is ECG surface electrodes. Since respiration sensors probably interfere with the breathing process, it is more convenient to acquire ECG from weightless chest leads. In the following, we briefly review some of the works where ECG is used for apnea detection. The studies in literature mostly focus on apnea that occurs during sleep in adults (McNames and Fraser 2000, Penzel *et al* 2002, Mendez *et al* 2007). One of them is presented in McNames and Fraser (2000), where the significancy of changes in various features including the time distance between R peaks (RR signal) and amplitudes and width of each QRS complex are studied. They report HR ($\propto 1/RR$) as the most affected feature and the spectrogram of S pulses and the QRS energy also provide useful information about occurrence of apnea. Penzel *et al* (2002) suggested that in comparison to HR temporal characteristics, features extracted in the frequency domain are more informative. In another research, Chazal *et al* (2004) suggested deriving the respiration effect from an ECG which is integrated with HR features. Then a linear discriminant classifier is applied for AB detection. Mendez *et al* (2007) used the beat-by-beat power spectral density of HR and the area under the QRS complex in a supervised learning K-nearest neighbor (KNN) classifier separating obstructive sleep apnea events from normal ones in adults.

The specific type of apnea which we intend to detect is the suspension of breathing which normally results in drops in HR and bradycardia indication in preterm infants. Hence, it requires specific processing and integrating an analysis of the dynamics of HR through the event. The most common approaches for HR characterization and AB detection in this context

are based on applying a fixed or an adaptive threshold (Poets *et al* 1993 and Portet *et al* 2007). Different methods have been proposed according to the characterization of HR of AB episodes using abrupt-change detection methods (Cruz *et al* 2006). While some researchers have used several complementary detectors to increase AB detection accuracy (Cruz *et al* 2006, Portet *et al* 2007), more recent works are proposed based on Bayesian Network (BN) approaches that consider the HR dynamics. An example of such an approach is the work of Travieso *et al* (2013), which classifies the cepstrum of *RR* signals using the hidden Markov model (HMM). Altuve *et al* (2011) trained two hidden semi Markov models (HSMMs) using the *RR* signal of a normal ECG and those with AB. This approach achieves 74.20% sensitivity and 92.77% specificity. The same strategy is also used in Masoudi *et al* (2013) employing a coupled HMM (CHMM) to obtain sensitivity and specificity as 84.92% and 94.17%, respectively. In Altuve *et al* (2015), some improvements in processing of observations, such as quantization are suggested in order to obtain more detection precision (88.66% sensitivity and 92.27% specificity).

As discussed, apnea can greatly affect the activity of the heart. The subtle changes in the ECG patterns can be interpreted as a change happening in the dynamic of the ECG beat generation process. In order to study such systems, one can propose using SKF, which is widely used for modelling systems with changeable dynamics (Ghahramani 1996, Marculescu *et al* 1998). In a simple form of SKF, it is assumed that the model has a linear dynamic at each time instant but it is time variant and switches among several linear subsystems over the time; each linear subsystem can be described by linear dynamical equations of continuous states (x_k) as *state equations* and a linear relation between states and observation (y_k) as an *observation equation*:

$$\begin{aligned} x_k &= Ax_{k-1} + \gamma_k \\ y_k &= Mx_k + r_k \end{aligned} \tag{1}$$

γ_k and r_k are state and measurement noises. A and M are state and observation coefficients respectively. This type of modelling is generally referred to as a state space model (SSM). In SKF, the states are estimated by several KFs with different state or observation equations at each time instant. Meanwhile, a hidden discrete state variable called a *switch* (s) is considered, so according to Markov characteristics, the status of this state changes over the time and indicates to the KF with the best state estimation performance. In the general structure of SKF, both state and observation equations depend on the switch variable as follows:

$$\begin{aligned} x_k &= A(s)x_{k-1} + \gamma_k(s) \\ y_k &= M(s)x_k + r_k(s) \end{aligned} \tag{2}$$

where $\Gamma(s)$ and $R(s)$ are covariance matrices of γ_k and r_k, respectively which are related to the switch s. The inference and training algorithms of SKF are fully described in Murphy (1998). In SKF, an expectation-maximization (EM) approach is applied to estimate the parameters of the linear models. This approach was used previously in other contexts such as tracking meteorological features over time (Manfredi *et al* 2005) and in a computer vision application for event detection and data collection at traffic intersections (Veeraraghavan *et al* 2005, Veeraraghavan *et al* 2006). In Pavlovi *et al* (1999), an approximate Viterbi inference approach is proposed by defining a cost function based on KF parameters. They employ their method for figure motion analysis and the results show that SKF performance is promising and capable of modelling a complex nonlinear system with a set of linear models chosen by a switch at each time instant. This algorithm is also applied in Zheng and Hasegawa-Johnson (2003) for segmentation of vowels, nasal, frication and silence in an acoustic signal. Depending on SKF

application and BN structure, a switch may be set on some of the continuous state variables rather than all of them, thus the independent states to the switch are simply estimated by a standard KF algorithm (Wu *et al* 2003). In some other works where multiple independent state variables have their own dynamics and it is not specified that which of them should be used, the switch is only set on the observation equation (Wu *et al* 2004).

In this paper, in order to model dynamic changes caused by AB occurrence in ECG, we propose two approaches (wave-based and R-based), each of which includes two models corresponding to normal and bradycardia dynamics. Moreover, a switch represents a discrete state that indicates the model with best performance at each time instant. The R-based method inspired by Wu *et al* (2004) is the most apparent way to employ SKF on RR signal for AB detection. Two first-order AR models are expected to model bradycardia pattern and normal RR signal independently. The switch is set on the observation equation and indicates which state should be used in the observation equation. It can be concluded that this approach employs just the inter-beat information since it employs RR signal as the only observation. In a wave-based approach, we try to present a method which gets the benefits of inter-beat as well as intra-beat characteristics. This can be achieved by using a dynamical model for the waveforms of a beat and including ECG signal in addition to observations, which is proportional to $1/RR$. One of the famous dynamical models of ECG beats is McSharry's model, which is used by Sameni *et al* (2007) in an extended Kalman filter (EKF) based framework. Hence, for normal beats and those associated with bradycardia, two dynamic models are considered and the SKF switch indicates the appropriate dynamic model. The algorithm is called the switching extended Kalman filter (SEKF). The details of the proposed approaches are presented in section 2.

The paper is organized as follows. Section 2 provides relevant background on the ECG dynamical model and two algorithms EKF2 (Sameni *et al* 2007) and EKF3 (Akhbari *et al* 2012). This section also includes the details of wave-based SEKF and R-based SKF approaches for AB detection for real data. Section 3 is devoted to the results of the two methods. Finally, a discussion and concluding remarks are provided in section 4.

2. Methods

In this section, in order to be self-contained, a dynamical model of ECG and two EKF-based algorithms which were proposed previously for ECG denoising are presented. Then, the proposed SEKF called wave-based approach is fully described. Finally, the proposed R-based SKF is explained.

2.1. Waveform based SEKF model

Every heartbeat on an ECG record consists of finite number of characteristic waveforms (typically the P wave, QRS complex and T wave), each of which can be easily modelled by the sum of Gaussian kernels. This idea was presented by McSharry *et al* (2003) in order to propose a nonlinear dynamic model for generating synthetic ECG. This model consists of three differential equations in Cartesian coordinates whose solution generates a trajectory in 3D space around a circular limit cycle that upward and downward deviations from the plane of the circle create waves like the waveforms of an ECG beat. The EKF2 algorithm introduced by Sameni *et al* (2007), includes the simplified discrete form of McSharry's model in a cylindrical coordinate system as follows:

$$\varphi_k = (\varphi_{k-1} + \omega_k \delta) \mathrm{mod}(2\pi)$$
$$z_k = z_{k-1} - \sum_{n \in \{P,Q,R,S,T\}} \delta \frac{\alpha_n \omega_k}{b_n^2} \Delta\varphi_n \exp\left(-\frac{\Delta\varphi_n^2}{2b_n^2}\right) + \eta_k \tag{3}$$

where $\Delta\varphi_n = (\varphi_{k-1} - \theta_n)$, φ_k is the phase of ECG signal, ω_k is angular velocity (proportional to the inverse of the RR-interval), z_k is the ECG signal considered as the sum of some Gaussian functions and η_k is a random additive white zero-mean noise that models the baseline wander. δ is the sampling period and α_n, b_n and θ_n are the parameters of the Gaussian kernels (amplitude, angular spread, and location of the Gaussian functions, respectively and assumed to be state noises). A simple interpretation of this model is to map each heartbeat over the time on a unit circle lying on an XY plane in a way that the first and last samples of a beat take phase $-\pi$ and π respectively. Accordingly, the R peak is expected to be located around phase zero. Changing the parameters of the Gaussian kernels results in ECG generation with different morphology. The parameter ω_k tunes the HR of generated ECG and determines the velocity of moving on an unit circle. The aforementioned differential equations can be integrated in the KF model if we consider appropriate observations and relate them to the state variables. Equation (4) shows the observation equations of the EKF2 model:

$$\Phi_k = \varphi_k + r_{1k}$$
$$Z_k = z_k + r_{2k} \tag{4}$$

where $r_k = [r_{1k}, r_{2k}]$ is the measurement noise vector of the model. Φ_k is the phase observation and Z_k represents the measured ECG signal. Since the state equation of this model is nonlinear, EKF is applied for state estimation. The main application of EKF2 is the denoising and compression of ECG signal (Sayadi and Shamsollahi 2008). In a previous algorithm, the angular velocity, ω_k, is one of the state noises. However, it can also be considered as a state variable (Lin *et al* 2011). This is the main object of the EKF3 algorithm proposed by Akhbari *et al* (2012). In this algorithm, ω_k is considered as the third state variable and an appropriate corresponding observation is introduced. Due to the small changes of the distance between different waveforms during several consecutive beats, a simple autoregressive (AR) model is used for angular velocity as $\omega_k = \omega_{k-1} + \beta_k$, where β_k is a white zero-mean Gaussian noise with variance, σ_β^2, and represents the uncertainty in angular velocity estimation. In this model, angular velocity observation, Ω_k, is obtained using RR signal and it is assumed to be almost constant during each beat, but may be contaminated by noise. Hence the corresponding observation equation may be considered as $\Omega_k = \omega_k + r_{3k}$.

In order to detect AB from normal beats in ECG, some modifications can be applied to the EKF3 algorithm to incorporate dynamical variations. In a wave-based approach, we try to employ inter-beat as well as intra-beat characteristics. This can be achieved by using a dynamical model for the waveforms of a beat. Inspired by existing studies, the famous dynamical model of ECG beats suggested by McSharry is used. Hence, for normal beats and those associated with bradycardia, two dynamic models are considered. At this stage, we assumed that bradycardia complications do not change the morphology of the waveforms; hence the properties of parameters of Gaussian kernels (α_i, b_i and θ_i) which are considered as state noise variables during EKF2 and EKF3 algorithms are constant. The only parameter that can be affected by bradycardia is the temporal distance between the peaks of these waveforms. To characterize such phenomena, we take the main idea of the EKF3 algorithm, which gives a continuous state variable to angular velocity (ω_k) and represents the speed of beat generation on the unit circle in McSharry's model. If ω_k is low, the generated beats elongate in time samples and

vice versa. This parameter was mainly applied as a noise with constant properties during beat generation (see Sayadi *et al* 2010). However, by using the EKF3 idea, we relax this parameter to be assigned at each instant of algorithm evolution. The state variable representing ω_k is added to the other state variables in EKF2 to form EKF3 state equations. Consequently, any subtle changes in ω_k can be detected. In our proposed approach, a two-value switch s is set on the state equation corresponding to ω_k, in order to change it whenever the dynamic changes and to judge between normal and bradycardia conditions. This switch indicates the most probable one by which the observation may be generated. s is a discrete state and assumed to have a first-order Markov chain with matrix transition C with $c_{ji} = P(s_k = i|s_{k-1} = j)$. Note that $..._i = ...(s_k = i)$ can be considered for all the parameters of the SKF model (A, M, Γ and R) on which the switch is allocated. The modified EKF3 is given as follows:

$$\begin{aligned} \omega_k &= A(s)\omega_{k-1} + \beta_k(s) \\ \varphi_k &= (\varphi_{k-1} + \omega_{k-1}\delta)\mathrm{mod}(2\pi) \\ z_k &= z_{k-1} - \sum_{n\in\{P,Q,R,S,T\}} \delta\frac{\alpha_n\omega_{k-1}}{b_n^2}\Delta\varphi_n \exp\left(-\frac{\Delta\varphi_n^2}{2b_n^2}\right) + \eta_k \end{aligned} \quad (5)$$

The observation can be related to the states of equation (5) as the following equations:

$$\begin{aligned} \Omega_k &= \omega_k + r_{1k} \\ \Phi_k &= \varphi_k + r_{2k} \\ Z_k &= z_k + r_{3k} \end{aligned} \quad (6)$$

where $r_k = [r_{1k}, r_{2k}, r_{3k}]$ is the measurement noise. Moreover, we define a physiological envelope on the amplitude of the angular velocity corresponding to normal condition (i.e. $\omega_k(s = 1)$) that spans between the upper and lower ranges of Ω_{env} defined as:

$$\Omega_{\mathrm{env}} = \overline{\Omega}_{\mathrm{normal}} \pm 3\sigma_{\Omega_{\mathrm{normal}}} \quad (7)$$

where $\overline{\Omega}_{\mathrm{normal}}$ is the mean of the observation of angular velocity related to normal ECG and its standard deviation is $\sigma_{\Omega_{\mathrm{normal}}}$. Ω_{env} is set by the training data. This constraint is defined since, practically, the value of $\omega_k(s = 1)$ is nearly constant in normal beats. In the case of bradycardia, the value of angular velocity decreased due to beat elongation. So $\omega_k(s = 1)$ is set to the nearest endpoint. Similarly, in normal circumstances, the value of the state of angular velocity related to AB, $\omega_k(s = 2)$, is equal to the mean of Ω_k during the intervals where an AB is observed in training data. As mentioned above, due to the nonlinearity of the third equation in equation (5), our proposed model is called the SEKF. Following the notation of equation (A.1), the state, observation and their corresponding noise vectors are defined as:

$$\begin{aligned} x_k &= [\omega_k\ \varphi_k\ z_k] \\ \gamma_k &= [\alpha_n\ b_n\ \theta_n\ \beta_k\ \eta_k] \quad n \in \{P, Q, R, S, T\} \\ y_k &= [\Omega_k\ \Phi_k\ Z_k] \\ r_k &= [r_{1k}\ r_{2k}\ r_{3k}] \end{aligned} \quad (8)$$

where the covariance matrix of states γ_k is a 17-dimensional matrix, as we have considered three parameters for five Gaussian kernels in addition to two additive noises.

Since the second process equation is nonlinear, a linear approximation of equation (5) near a desired point $(\hat{x}_k, \bar{\gamma}_k)$ is required for estimating the state vector according to the EKF algorithm. Linearization can be performed as follows:

$$x_k = f(x_{k-1}, \gamma_k, k) = f(\hat{x}_{k-1}, \bar{\gamma}_k, k) + G_k(x_{k-1} - \hat{x}_{k-1}) + F_k(\gamma_k - \bar{\gamma}_k) \tag{9}$$

where f represents the nonlinear state evolution function. G_k and F_k are state linearization coefficients given by:

$$\begin{aligned} G_k &= \frac{\partial f(x_{k-1}, \bar{\gamma}_k, k)}{\partial x_{k-1}}\Big|_{x_{k-1}=\hat{x}_{k-1}, s_k=i, s_{k-1}=j} \\ F_k &= \frac{\partial f(\hat{x}_{k-1}, \gamma_k, k)}{\partial \gamma_k}\Big|_{\gamma_k=\bar{\gamma}_k, s_k=i, s_{k-1}=j} \end{aligned} \tag{10}$$

According to equations (5) and (10), we are able to compute G_k and F_k, whose derivations are explained extensively in the appendix A.1. The evolution of the SEKF is changed based on the switch state and consists of three stages summarized in *FilteringSEKF*, *StatesProbability* and *Collapsing* functions (see appendix A.2). In these relations, we define $x_k^{ji} = E\{x_k|y_{1:k}, s_k = i, s_{k-1} = j\}$ and $P_k^{ji} = \mathrm{Cov}\{x_k|y_{1:k}, s_k = i, s_{k-1} = j\}$ that the standard EKF algorithm can be utilized for their calculation in *FilteringSEKF* function. $L_k^{ji} = p(y_k|y_{1:k-1}, s_k = i, s_{k-1} = j)$, the likelihood of observation given states at time k and $k-1$, is also calculated within the execution of this function. Moreover, the *StatesProbability* function calculates some of the probabilistic parameters: $K_k^i = P(s_k = i|y_{1:k}), g_k^{j|i} = P(s_{k-1} = j|y_{1:k}, s_k = i)$ and $K_k^{ji} = K_k^i g_k^{j|i} =$ for $i, j = 1, \cdots, N$ that are used for choosing the EKF with the best performance at each time instant. The *Collapsing* function computes the estimation of x_k^i and P_k^i from the mixture model using an approximation of $p(x_k|y_{1:k}, s_k = i) \simeq \mathcal{N}(x_k^i, P_k^i)$, where $x_k^i = E\{x_k|y_{1:k}, s_k = i\}$ and $P_k^i = \mathrm{Cov}\{x_k|y_{1:k}, s_k = i\}$.

In order to establish our proposed SEKF model, all needed initial values are set similar to Sameni *et al* (2007) and we apply the SKF training approach presented in Murphy (1998). The unknown parameters of switching dynamics (i.e. A_i, Γ_i and C) are achieved during execution of training iteratively. We can write the loglikelihood and optimization criterion respectively as follows:

$$\begin{aligned} L = \log(p\{x_{1:T}, y_{1:T}, s_{1:T}\}) = -\frac{1}{2}\sum_{k=1}^{T} \{\{(x_k^i - A_i x_{k-1}^j)\Gamma_i^{-1}(x_k^i - A_i x_{k-1}^j)^{\mathrm{T}} + \log|\Gamma_i|\}K_k^i \\ +(y_k - Mx_k^i)R^{-1}(y_k - Mx_k^i)^{\mathrm{T}} + \log|R| + \log c_{s_{k-1}s_k}\} + \log(P(s_0)P(x_0|s_0)) \end{aligned} \tag{11}$$

$$\begin{aligned} \arg\max_{A_i,\Gamma_i,c_{ji}} p(\{s_{1:T}, y_{1:T}, x_{1,T}\}) &= \arg\max_{A_i,\Gamma_i,c_{ji}} p(s_{1:T})p(x_{1:T}|s_{1:T})p(y_{1:T}|x_{1:T}) \\ &= \arg\max_{A_i,\Gamma_i,c_{ji}} p(s_{1:T})p(x_{1:T}|s_{1:T}) \end{aligned} \tag{12}$$

where $p(y_{1:T}|x_{1:T})$ is omitted since there is no unknown parameter in this term. Note that the parameter M in the observation equation is known by considering the relation between observation and state variables. R can be set by estimating the covariance of the observation noise vector. The re-estimation repeats until convergence.

We only show the closed-form solution using an approximation of the maximum likelihood (ML) approach for re-estimation as follows:

$$A_i = \frac{\sum_{k=2}^{T} K_k^i x_k^i x_{k-1}^i}{\sum_{k=2}^{T} K_k^i x_{k-1}^i x_{k-1}^i}$$
$$\Gamma_i = \frac{\sum_{k=2}^{T} K_k^i x_k^i x_k^i - A_i \sum_{k=2}^{T} K_k^i x_k^i x_{k-1}^i}{\sum_{k=2}^{T} K_k^i} \tag{13}$$
$$c_{ji} = \frac{\sum_{k=2}^{T} K_k^{ji}}{\sum_{k=1}^{T-1} K_k^i}$$

where it is straightforward to show that:

$$K_k^{ji} = P(s_k = i, s_{k-1} = j | y_{1:k}) = \frac{L_k^{ji} c_{ji} K_{k-1}^j}{\sum_{i=1}^{N} \sum_{j=1}^{N} L_k^{ji} c_{ji} K_{k-1}^j} \tag{14}$$

Obtaining the parameters of each model, the probability of the dynamic i for each time instants $(K_k^i, i = 1, 2)$ is computed using the inference over each test signal. If $K_k^1 > K_k^2$, the observed temporal sample k is likely generated by dynamic 1. The mentioned functions of the SEKF are used in algorithms 1 and 2 illustrating the training and inference procedures.

Physiologically speaking, the elongation of a beat does not occur only at the end of the beat and it is accompanied by an increase of temporal distances between different waveforms. Hence, we try to give an intra-beat interpretation of angular velocity by using a dynamical model to detect the changes not only in *R*–*R* distance but also in temporal distance between the waveforms peaks in a beat. Furthermore, since bradycardia is an event which appears more clearly in terms of beats than in time instants, beats are related conveniently to a dynamic. Therefore, we investigate two approaches in order to compute the local mode on the label of consecutive samples. The first method, mode on window (MOW), is to calculate the mode of labels falling in a window moving through the signal as the SEKF algorithm evolves sample by sample. The length of the moving window is considered equal to the average length of ECG beats in the training data set. In this approach, if more than $\xi\%$ of the temporal samples of an ECG beat are labeled as AB, it is supposed that the AB begins from the first sample of that beat. Alternatively, in the second method, mode on beat (MOB), we can consider the label of samples of a beat and calculate their mode. Note that in the MOB method, the label of the samples in a beat located in the beginning of AB event is determined at the end of the beat. Therefore, although all of its samples are likely to be labeled equally as AB, the detected time onset of AB is set at the end of the beat for time delay of detection. There is an ambiguity in the borders of a beat, so it is necessary to choose a logic for defining the beginning and end points. We have used Sameni's definition in this regard (Sameni *et al* 2007), where the beginning of a beat is considered on a point placed at a equal distance between the corresponding *R* peak and the previous one.

2.2. R-based SKF model

The alternative and simple method that we propose for AB detection consists of an SKF structure where the switch affects just the observation equation. In this structure, the observation includes the *RR* signals obtained from raw ECG. The normal *RR* signals are modelled by an

Algorithm 1. Wave based: training

Inputs: x_0^{ji}, P_0^{ji}, initial values of A_i, Γ_i, M, R and C, i, j=1,2.
Outputs: Trained values of A_i, Γ_i and C

repeat
  for $k = 1, 2, \cdots, T$ **do**
    Compute G_k and F_k from equation (10).
    $[x_k^{ji}, P_k^{ji}, L_k^{ji}] = \text{FilteringSEKF}(x_{k-1}^{j}, P_{k-1}^{j}, A_i, \Gamma_i, M, R, G_k, F_k)$
    $[K_k^{ji}, K_k^{i}, g_k^{j|i}] = \text{StatesProbability}(L_k^{ji}, c_{ji}, K_{k-1}^{j})$
    $[x_k^{i}, P_k^{i}] = \text{Collapsing}(x_k^{ji}, P_k^{ji}, K_k^{i}, g_k^{j|i})$
  end for
  Calculate A_i, Γ_i and C using equation (13).
until Convergence

Algorithm 2. Wave based: inference

Inputs: x_0^{ji}, P_0^{ji}, A_i, Γ_i, M, R and C, i, j=1,2.
Outputs: K_k^i,

for $i = 1, 2, \cdots, M$ **do**
  for $k = 1, 2, \cdots, T$ **do**
    Compute G_k and F_k from equation (10).
    $[x_k^{ji}, P_k^{ji}, L_k^{ji}] = \text{FilteringSEKF}(x_{k-1}^{j}, P_{k-1}^{j}, A_i, \Gamma_i, M, R, G_k, F_k)$
    $[K_k^{ji}, K_k^{i}, g_k^{j|i}] = \text{StatesProbability}(L_k^{ji}, c_{ji}, K_{k-1}^{j})$
    $[x_k^{i}, P_k^{i}] = \text{Collapsing}(x_k^{ji}, P_k^{ji}, K_k^{i}, g_k^{j|i})$
  end for
end for
Applying a threshold on K_k^i

AR process. A bradycardia episode commutes a rise in *RR* signal and can also be independently fitted by an AR model, which differs from the AR model corresponding to normal HR. Normal and bradycardia AR models whose parameters are obtained during the training phase configure the state equation of this SKF. Using higher order AR models would be more accurate, but would be more complex. Therefore, we restrict our study to the first-order AR models. The states of these two models evolve independently and estimate the amplitude of *RR* based on their given dynamic. Hence, x_{1k} and x_{2k} are *RR* signals corresponding to normal and AB, respectively, resulting from two independent AR models. They generate various K_k^1 and K_k^2 which are used to classify the samples of *RR* signal into one of the normal and bradycardia classes. It can be concluded that the approach employs just inter-beat information since it employs *RR* signal as the only observation. The state and observation equations are given as:

$$\begin{aligned} x_k &= [x_{1_k} \quad x_{2_k}]^{\intercal} = A_{2\times 2}[x_{1_{k-1}} \quad x_{2_{k-1}}]^{\intercal} + \gamma_k \\ y_k &= M(s)[x_{1_k} \quad x_{2_k}]^{\intercal} + r_k(s) \end{aligned} \tag{15}$$

where y_k is the calculated *RR* from recorded ECG that can be considered as a noisy version of x_{1k} or x_{2k}. A is a diagonal matrix. In this structure all the model parameters (A, Γ, M_i, R_i and c_{ji})

can be estimated optimally. If we consider the structure of the SKF, the optimization criterion based on the joint probability of states, switch and observation is as equation (16) which is proposed by Wu *et al* (2004):

$$\begin{aligned} &\arg \max_{A,\Gamma,M_i,R_i,c_{ji}} p(\{x_{1:T}, s_{1:T}, y_{1:T}\}) \\ &= \arg\max_{A,\Gamma} p(x_{1:T}) \arg \max_{M_i,R_i,c_{ji}} p(s_{1:T}, y_{1:T}|x_{1:T}) \end{aligned} \tag{16}$$

The first term relates to states which are continuous variables and its maximization is straightforward by using the EM algorithm and gives parameters A and Γ:

$$\begin{aligned} A &= \left(\sum_{k=2}^{T} x_k x_{k-1}^{\intercal}\right)\left(\sum_{k=2}^{T} x_{k-1} x_{k-1}^{\intercal}\right)^{-1} \\ \Gamma &= \frac{1}{T-1}\sum_{k=2}^{T} x_k x_k^{\intercal} - A\sum_{k=2}^{T} x_{k-1}x_k^{\intercal} \end{aligned} \tag{17}$$

By using the second term, we can estimate parameters of the observation equation and the transition matrix of the switch, which is a discrete variable. Therefore, the Baum–Welch algorithm should be applied to estimate the transition matrix of the switch. This algorithm uses expectation of the log likelihood for the discrete parameter estimation. Hence we have the rest of the unknown parameters as follows:

$$\begin{aligned} M_i &= \frac{\sum_{k=2}^{T} K_k^i y_k x_k^{\intercal}}{\sum_{k=2}^{T} K_k^i x_k x_k^{\intercal}} \\ R_i &= \frac{\sum_{k=2}^{T} K_k^i (y_k y_k^{\intercal} - M_i x_k y_k)}{\sum_{k=2}^{T} K_k^i} \\ c_{ji} &= \frac{\sum_{k=2}^{T} K_k^{ji}}{\sum_{k=1}^{T-1} K_k^i} \end{aligned} \tag{18}$$

The training and inference of this method are performed according to algorithms 3 and 4 using *FilteringKF* which is defined in the appendices and includes the standard KF algorithm.

2.3. Data and evaluation metrics

A database including one-lead ECG and respiration signals acquired from 32 preterm infants hospitalized in the NICU at the University Hospital of Rennes, France (Altuve *et al* 2011) is used to study the performance of the proposed wave-based and *R*-based methods. ECG signals were acquired at 400 Hz. This observational study was approved by the ethical committee of the University Hospital of Rennes and written consent was obtained from the parents of each infant. All data were anonymized at acquisition. The data is comprised of 105 segments of ECG with 250 s duration and the total number of AB episodes equal to 233. Each acquired ECG segment was denoised by using a combination of filters for discarding the baseline and the noise of 50 Hz respectively. For baseline cancelation, the output of the 8 Hz low pass filter is subtracted from the main ECG signal. Individual beats for each segment were detected using the *R*-peak detection algorithm proposed in Pan and Tompkins (1985).

Algorithm 3. R-based: training

Inputs: x_0^{ji}, P_0^{ji}, initial values of A, Γ, M_i, R_i and C, i, j=1,2.
Outputs: Trained values of A, Γ, M_i, R_i and C

repeat
  for $k = 1, 2, \cdots, T$ **do**
    $[x_k^{ji}, P_k^{ji}, L_k^{ji}] = \text{FilteringKF}(x_{k-1}^{j}, P_{k-1}^{j}, A, \Gamma, M_i, R_i)$
    $[K_k^{ji}, K_k^{i}, g_k^{j|i}] = \text{StatesProbability}(L_k^{ji}, c_{ji}, K_{k-1}^{j})$
    $[x_k^{i}, P_k^{i}] = \text{Collapsing}(x_k^{ji}, P_k^{ji}, K_k^{i}, g_k^{j|i})$
  end for
  Calculate A and Γ using equation (17) and M_i, R_i and C using equation (18).
until Convergence

Algorithm 4. R-based: inference

Inputs: x_0^{ji}, P_0^{ji}, A, Γ, M_i, R_i and C, i, j=1,2.
Outputs: K_k^{i},

for $i = 1, 2, \cdots, M$ **do**
  for $k = 1, 2, \cdots, T$ **do**
    $[x_k^{ji}, P_k^{ji}, L_k^{ji}] = \text{FilteringKF}(x_{k-1}^{j}, P_{k-1}^{j}, A, \Gamma, M_i, R_i)$
    $[K_k^{ji}, K_k^{i}, g_k^{j|i}] = StatesProbability(L_k^{ji}, c_{ji}, K_{k-1}^{j})$
    $[x_k^{i}, P_k^{i}] = \text{Collapsing}(x_k^{ji}, P_k^{ji}, K_k^{i}, g_k^{j|i})$
  end for
end for
Applying a threshold on K_k^{i}

In this study, ABs were manually annotated by a clinician. In addition, a curve fitting procedure was proposed to locate, with the best reproducibility, the beginning of the AB. A sigmoid function is used to approximate the rise of RR time series around the AB episode. The AB onset is the first point at which the derivation of the sigmoid function is greater than 1 (Altuve *et al* 2015) at a sampling rate of 400 Hz. In order to cross-validate the results, the detection procedure is repeated in 5 rounds, each of which involves a holdout cross-validating and calculation of metrics. In the holdout procedure, we preliminary performed a permutation on the data and then chose 20% of the shuffled data for training and the rest of them for testing. Each segment includes at least one AB episode; therefore, various number of ABs are used for training in each round.

Quantitative results are reported using common metrics: sensitivity (SEN) and specificity (SPC), defined as $TP/(TP+FN)$ and $TN/(TN+FP)$ respectively where TP, FP, TN and FN denote the number of true positives, false positives, true negatives and false negatives, respectively, which are counted over the samples of observations of test data. In R-based method, the extracted RR signal is interpolated to reach 400 Hz sampling rate to be matched with annotations. Then, in order to have AR models with more convenient parameters, it is downsampled to 10 Hz to avoid the algorithm being overtrained, tedious and time-consuming. Although, the number of wave-based and R-based samples are not equal, quantitative comparison between them is possible since the metrics are relative criteria. We define another metric to show the

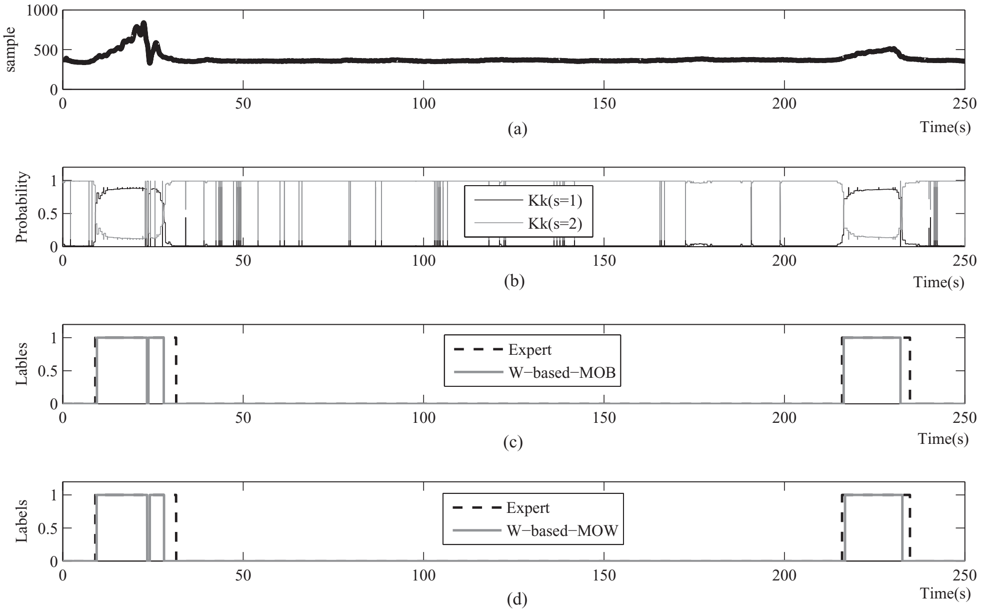

Figure 1. The performance of wave-based frameworks. (a) *RR* signal. (b) K_k^i for i=1,2. (c) and (d) the real annotations and labels determined by the algorithm with MOB and MOW processing, respectively. Labels 0 and 1 indicate normal and bradycardia, respectively.

speed of an algorithm to detect an AB. This metric is time delay (*TD*), calculated as the elapsing time between the annotated onset of occurring AB and the onset, which the algorithm determines. The wave-based method is expected to find the onset by 2.5 ms time resolution while *R*-based method has 0.1 s time resolution according to the sampling rate. The accuracy (*AC*) of detection in terms of events is also computed in each round.

3. Results

Implementation of the wave-based and *R*-based approaches includes a training phase primarily for estimation of unknown parameters of the model and a test phase to evaluate the performance of the proposed procedure in terms of metrics. In this section, we firstly show the method's performance by using an example of an *RR* series in order to visually demonstrate the qualitative results. Then, quantitative results in terms of *SEN*, *SPC*, *TD* and *AC* of detection are presented. Finally, the best proposed method is compared with existing methods in an AB detection context.

3.1. Qualitative results of wave-based performance

Figure 1 depicts an example of the AB detection using the wave-based method in a segment. The *RR* signal of the test data is shown in figure 1(a). Moreover, K_k^i is illustrated in figure 1(b). As depicted in this figure, K_k^i of the wave-based method has many fluctuations. Hence, a mode calculation procedure is employed, which is implemented by using two methods: MOW and MOB. In MOW, first, a mode calculation is employed, hence the local changes in labels,

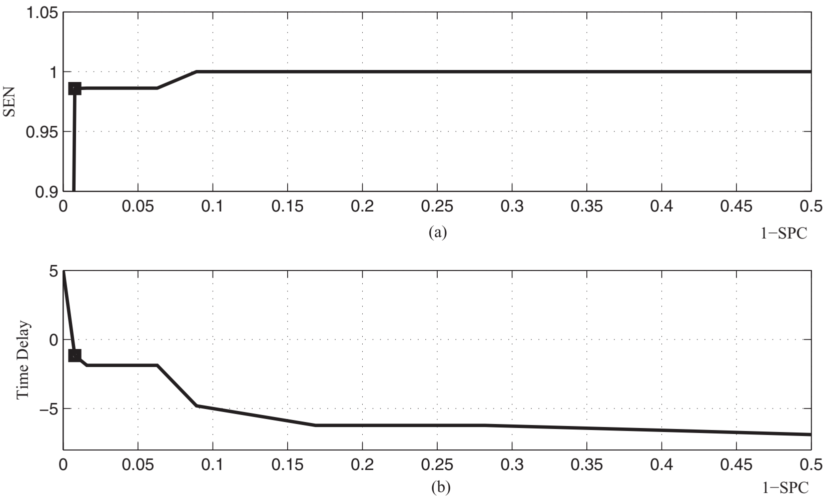

Figure 2. Searching for the best value of ξ, which is the percentage of samples in a beat labeled as AB event by our wave-based MOW method. The results are achieved on training–test data. (a) ROC curve (*SEN* versus 1-*SPC*). (b) Time delay of detection versus 1-*SPC*. PD is marked by an square in each plot.

determined by comparing the K_k^1 with K_k^2, are decreased. Then, the decision on the labels of beat is made using the parameter ξ. The duration of a moving window is 360 samples and the value of ξ is optimized by using the receiver operating characteristic (ROC) curve. Each point on the ROC curve represents a value for sensitivity and specificity achieved by a specific value for ξ. Figure 2(a) shows the ROC curve obtained by altering the value of ξ. The marked point, the perfect detection (PD) point, is introduced by searching according to the following criterion:

$$PD = \arg\max\{SEN \times SPC\} \tag{19}$$

According to figure 2(a), the parameter ξ is chosen equal to 85% for the best detection of MOW using training–test data. The corresponding time delay plot is also depicted in figure 2(b).

In MOB, the result of mode calculation directly leads to the label determination of the corresponding beat.

The annotations determined by an expert and the label decided by the wave-based algorithm by using MOB method are shown in figure 1(c). Similarly, figure 1(d) illustrates the labels decided by this algorithm with the MOW method as well as those determined by an expert.

The estimated state variable corresponding to the angular velocity of the wave-based model is illustrated in figure 3. The physiological envelope (equation (7)) restricts the range of this state variable value (ω_k^i).

3.2. An example of R-based approach performance

The performance of the *R*-based method on the same example segment is shown in figure 4. As can be seen, since each sample of the feature *RR* is extracted from a beat, no fluctuation can be observed in K_k^i (figure 4(b)), thus unlike the wave-based method, mode calculation is

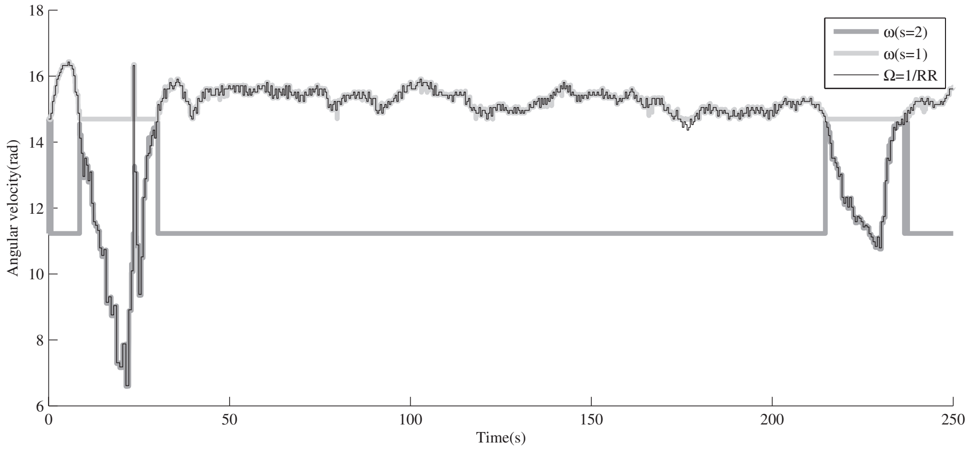

Figure 3. Estimated angular velocity (state variable controlled by switch) and the observation of the angular velocity for the wave-based method.

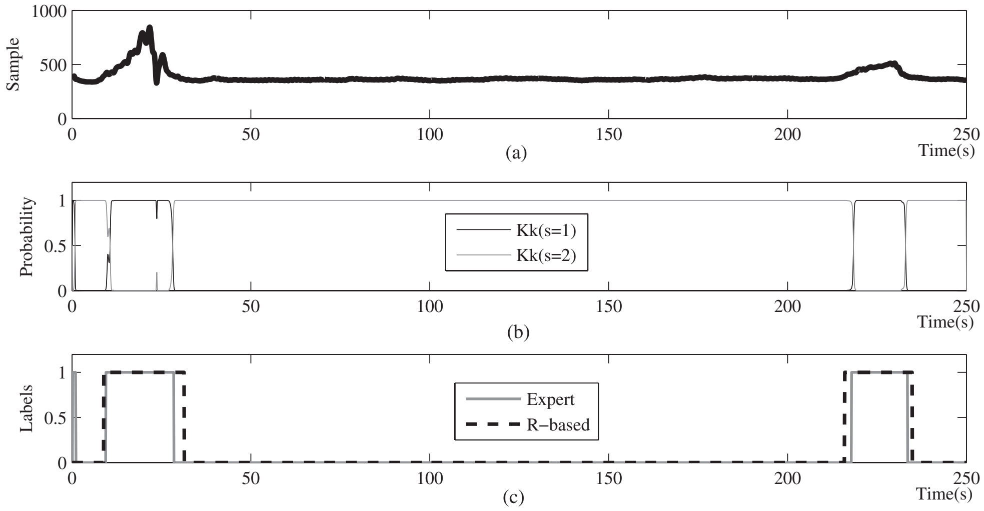

Figure 4. The performance of *R*-based frameworks. (a) *RR* signal. (b) K_k^i for *i*=1,2. (c) The real annotations and labels determined by the algorithm. Labels 0 and 1 indicate normal and bradycardia, respectively.

not required. The labels obtained by *R*-based models and the expert annotations are illustrated in figure 4(c).

3.3. Quantitative results

The metrics for the proposed approaches over test data calculated by cross-validation are reported in table 1. According to this table, MOB comparing with MOW is found to be more reliable for detecting an AB event. This shows that the assumption used for defining the beginning and ending of the beats in ECG is significant. In addition, calculation of mode over beats

mainly reduces the amount of computation compared to MOW in which the mode is computed sample by sample. The results show the ability of wave-based and *R*-based methods to detect the AB occurrence accurately. However, wave-based methods reflect better detection performance, although they are more complicated. Moreover, they are capable of detecting the AB nearly faster than *R*-based method.

The average values of *SEN* and *SPC* using MOB are 94.74% and 94.17%, respectively. The low average time delay in this method, 0.35 ± 2.12, is achieved by searching for AB sample by sample in the SEKF algorithm with MOB postprocessing and introduces this approach as a fast detector. Whereas, in an *R*-based detector, processing of the *RR* signal loses the intra-beat information and has a higher average value for time delay reported as 1.80 ± 1.94.

It can be seen that the wave-based MOB demonstrates the best average performance. MOW performs marginally better than the *R*-based, but still underperforms the MOB approach while the *AC* of all methods are more than 99%.

It should be noted that, in wave-based detectors, the average standard deviation (std) of time delay is larger than its mean, which implies that, in some cases, these detectors predict the occurrence of AB since its onset is reported prior to the annotation determined by the expert. Moreover, the reported value of time delay is the average of all obtained values consisting of the prediction with negative time delays and the detection cases with positive time delays.

3.4. Comparing MOB with other existing methods

In order to compare the proposed methods with other benchmarks, we have studied the previous methods on our database for AB detection in preterm infants. Furthermore, the conventional thresholding has been tested. In this simple method, the value of threshold is determined on training data and the evaluation is performed over test data. The reported results are based on *R* peak detection (Pan and Tompkins 1985) and *RR* signal extraction (the same algorithm used in proposed methods), together with a fixed hard thresholding strategy. For performance evaluation, Altuve *et al* (2011) used 148 *RR* series (series duration = 26.25 ± 11.37 min) with 233 bradycardia episodes. They employed 48 series for training and left the rest for test. We have used the same records; however, to ensure the consistency of the results on various subjects and reducing the number of normal temporal samples, the full length of the records was divided in to 105 segments with 250 s duration with the same total number of AB episodes. The whole procedure was repeated five times over the training data consisting of the 20% of selected records, each time using the same initial parameters.

The average *SEN* and *SPC* of the AB detection for different methods are depicted in table 2. For HSMM and HMM methods, two values for each metric are reported, the first one is the value reported in Altuve *et al* (2015), and the second one (shown by [a] in table 2) is obtained by our implementation of the corresponding algorithm. The minor differences observed between these two results are due to small differences in implementation (for instance in optimum number of states in the models and the cross-validation procedure). The *p*-values of MOB compared to other methods are computed based on the Mann–Whitney u-test. According to the *p*-values, a significant enhancement in most of the metrics can be observed in MOB (assuming $p < 0.05$ as a significant improvement of our algorithm compared to each of the other methods). It can be seen that MOB demonstrates the best average performance in lower ratio of the number of records in training over those in test procedures. Furthermore, its superiority is obvious, especially in terms of average time delay, where it can detect AB far faster than the others. The thresholding method finds the AB episodes based on the amplitude of observation and according to table 2, it has weaknesses in time delay and the

Table 1. Proposed wave-based (MOW and MOB) and *R*-based methods performance evaluation using cross-validation.

Method	*AC*	*SEN*	*SPC*	Mean *TD* (s)	std *TD*(s)
MOW	99.29 ± 0.40	94.51 ± 0.35	92.68 ± 0.30	1.78 ± 0.52	1.77 ± 0.53
MOB	99.11 ± 0.73	94.74 ± 0.69	94.17 ± 0.87	0.35 ± 0.13	2.12 ± 0.29
R-based	99.13 ± 0.02	90.35 ± 5.46	88.59 ± 11.28	1.80 ± 0.89	1.94 ± 0.43

Note: accuracy is computed over the average number of events detected correctly. The rest of the metrics are in terms of samples.

Table 2. Comparing the best results with other methods.

	Method	*SEN*	*SPC*	Mean *TD* (s)	std *TD* (s)
Our work	MOB	94.74 ± 0.69	94.17 ± 0.87	0.35 ± 0.13	2.12 ± 0.29
Altuve *et al* (2015)[a]	HSMM	88.66 ± 1.72	92.87 ± 0.86	1.59 ± 0.24	3.61 ± 0.30
		88.46 ± 1.16	93.57 ± 1.14	2.10 ± 0.66	2.26 ± 0.81
		p=0.0079	p=0.5476	p=0.0079	p=1
Altuve *et al* (2015)[a]	HMM	86.52 ± 3.96	92.27 ± 1.77	1.61 ± 0.43	3.74 ± 0.32
		89.06 ± 1.08	92.28 ± 0.71	1.59 ± 0.36	1.28 ± 0.17
		p=0.0079	p=0.0079	p=0.0079	p=0.0079
Masoudi *et al* (2013)	CHMM	84.92 ± 0.26	94.17 ± 0.51	2.32 ± 0.01	4.82 ± 0.03
		p=0.0079	p=0.6508	p=0.0079	p=0.0079
—	Thresholding	87.98 ± 0.88	84.46 ± 1.42	2.71 ± 0.12	2.07 ± 0.17
		p=0.0079	p=0.0079	p=0.0079	p=0.4206

[a] The results are obtained based on Altuve *et al* (2015) and by our implementation to reconstruct raw results.

precision of detection, while other methods learn the dynamic rather than depending on the amplitude of observation.

Figure 5 is illustrated in order to visually compare the repeatability of the results of table 2. It shows five values for each metric resulted from the cross-validation rounds. The values corresponding to HSMM and HMM are obtained from our implementation. Moreover, lower quartile, median and upper quartile values of the results are included. As depicted, the CHMM method proposed by Masoudi *et al* demonstrates lower std among the other studied methods. In this method, the QRS complex duration is also employed as the second feature beside *RR* signal; hence, it might result in a low variance in metrics. However, it still takes more time to detect AB. While, MOB shows a reasonable std and mostly the best average results.

4. Discussion and conclusions

In this work, the main goal is to use SKF for AB detection. Therefore, we proposed two SKF-based models where in one approach ECG signal is utilized as an observation while, in the other, *RR* series is employed. In the EKF framework previously used in different applications like denoising or compression, McSharry's model is applied in order to model ECG signal as a combination of a finite number of Gaussian kernels and establish a set of state equations of KF. The performance of this model is eligible if the characteristics of ECG has slight alterations. Hence, when an arrhythmia like AB happens, the parameters of the relevant model is totally different from the normal situation. Therefore, if we intend to detect AB, it is convenient to integrate two EKF models for normal and AB in the form of an SKF and monitor the probability of discrete state variable corresponding to the switch (K_k^i) as a detector.

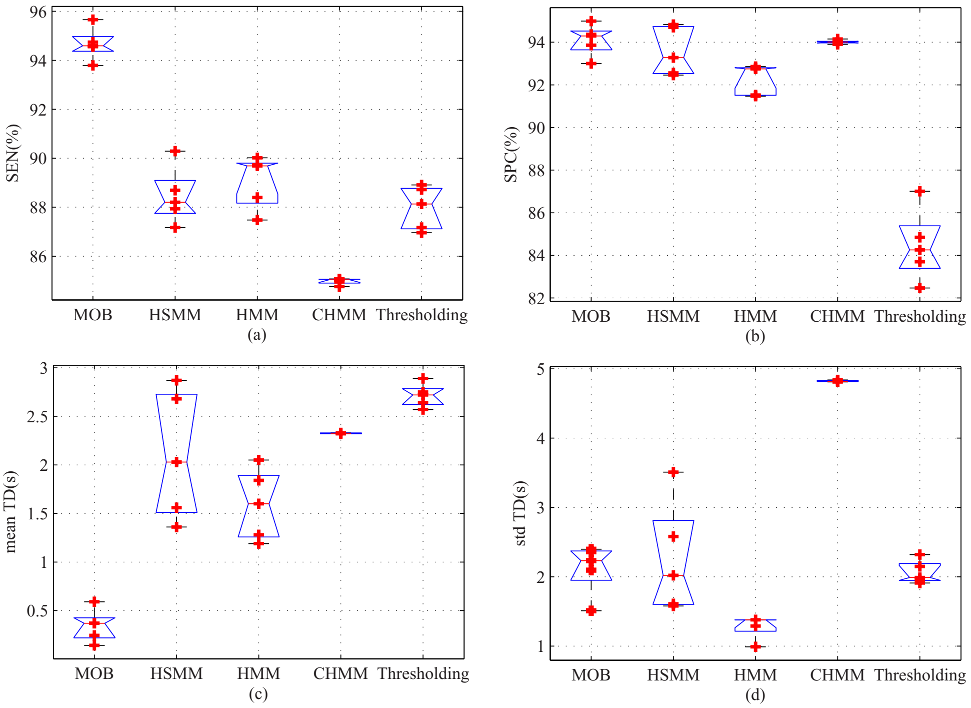

Figure 5. The comparison of wave-based MOB performance with existing methods showing the lower quartile, median and upper quartile values. The raw data are illustrated by red crosses. (a) Sensitivity. (b) Specificity. (c) Mean of time delay. (d) Std of time delay.

On the other hand, we suggest an *R*-based method based on the SKF structure proposed in Wu *et al* (2004), which includes two independent linear AR models. The models provide a state equation whose states are the *RR* signal corresponding to normal and AB. The observation is calculated *RR* which can be considered as a noisy version of state variables. The switch indicates to the state variable which one of the models should be used in the observation equation.

The SKF algorithm is comprised of several KF, each including the model parameters related to a dynamic. Each filter is able to adapt with different morphologies and temporal nonstationarities since the variance of the observation noise defines the degree of reliability of an observation. This determines a bound of how much tracking the observation interferes in estimation of state variables. However, in the case of powerful noises in observation or dynamic alteration, the information that KF obtains from its model is expected to be poor and it decreases the probability of observation to be generated by such model. The SKF quantifies this probability and refers to it as the probability of switch conditions.

The designed filter was applied to real ECG databases comprising AB acquired from preterm infants. Compared to the *R*-based detection scheme, the wave-based model provides a better detection performance, especially in finding bradycardia events without missing them.

The application of HMM-based approaches had been previously reported (Altuve *et al* 2011, Masoudi *et al* 2013, Altuve *et al* 2015). The major drawback of such methods is that each beat is represented by finite number of features, which results in methods using only the inter-beat information, like our *R*-based method. However, the wave-based technique does not depend on the specific features and instead uses a dynamic state space representation

for adaptive signal tracking sample by sample. Moreover, unlike the EKF, which depends on covariance of observation noise in the case of nonstationarities, the wave-based method evaluates the performance of different EKFs and uses the most probable one to match the state variables to the observation. Another point of interest is time delay improvement of the wave-based model that ensures early detection of bradycardia, which is an important factor for the algorithm of a monitoring device in an NICU. A simple thresholding approach takes a couple of seconds detecting a rise in *RR* signal and mainly uses the amplitude, yet the proposed methods are expected to learn the dynamic and to detect the change in observation earlier with better precision. The results show that our proposed wave-based method is capable of detecting AB faster than the other methods such as (Masoudi *et al* 2013, Altuve *et al* 2015).

In summary, the main contribution of this work is summarized as: (1) the introduction of a wave-based state space formulation according to the SEKF for detecting bradycardia, and (2) the derivation of a linear *R*-based model according to the previously proposed SKF for detecting the bradycardia from *RR* signal. Future works include incorporating AR parameter estimation methods instead of the ML approach for the training procedure in both proposed approaches. In the wave-based approach, we can use other dynamical models for ECG waveforms. Other feature signals (like duration and the amplitude of QRS complex) rather than *RR* signal can be extracted and, then, an SKF model similar to the *R*-based approach can be used for processing and AB detection. Furthermore, we can also use multidimensional observation including *RR* signal and other features. Higher orders for AR models may result in better detection performance although they make the algorithm more complex.

Appendix A

A.1. EKF matrices derivation

$$
\begin{aligned}
&G_k(1,1) = A_i \qquad G_k(1,2) = G_k(1,3) = 0\\
&G_k(2,1) = \delta \qquad G_k(2,2) = 1 \qquad G_k(2,3) = 0\\
&G_k(3,1) = -\sum \delta \frac{\alpha_n}{(b_n)^2}\Delta\theta_n \exp\left(-\frac{\Delta(\theta_n)^2}{2(b_n)^2}\right)\\
&G_k(3,2) = -\delta\frac{\alpha_n\omega_{k-1}^j}{b_n^2}\left(1-\frac{(\Delta\theta_n)^2}{b_n^2}\right)\exp\left(-\frac{\Delta\theta_n^2}{2b_n^2}\right) \qquad G_k(3,3) = 1\\
&F_k(1,1) = 1 \qquad F_k(1,2:17) = 0 \qquad F_k(2,1;17) = 0 \qquad F_k(3,1) = 0\\
&F_k(3,2:6) = -\delta\frac{\omega_{k-1}^j\Delta\theta_n}{b_n^2}\exp\left(-\frac{\Delta\theta_n^2}{2b_n^2}\right)\\
&F_k(3,7:11) = 2\delta\frac{\alpha_n\omega_{k-1}^j\Delta\theta_n}{b_n^3}\left(1-\frac{\Delta\theta_n^2}{2b_n^2}\right)\exp\left(-\frac{-\Delta\theta_n^2}{2b_n^2}\right)\\
&F_k(3,12:16) = \delta\frac{\alpha_n\omega_{k-1}^j}{b_n^2}\left(1-\frac{\Delta\theta_n^2}{b_n^2}\right)\exp\left(-\frac{\Delta\theta_n^2}{2b_n^2}\right)\\
&F_k(3,17) = 1 \qquad M = I_3
\end{aligned}
\tag{A.1}
$$

where I_3 is the 3-dimension identity matrix.

A.2. Common functions in SKF and EKF

FilteringKF: evolution of the KF from $k-1$ to k.
Require: x^j_{k-1}, P^j_{k-1}, A_i, Γ_i, M_i and R_i.
Ensure: x^{ji}_k, P^{ji}_k,

$i,j = 1:N$
1: $x^{-ji}_k = A_i x^j_{k-1}$
2: $P^{-ji}_k = A_i P^j_{k-1} A_i^\intercal + \Gamma_i$
3: $e_k = y_k - M x^{-ji}_k$
4: $L^{ji}_k = \mathcal{N}(e_k; 0, MP^{-ji}_k M^\intercal + R)$
5: $B = (P^{-ji}_k M^\intercal)(MP^{-ji}_k M^\intercal + R)^{(-1)}$
6: $P^{ji}_k = (I - BM)P^{-ji}_k$
7: $x^{ji}_k = x^{-ji}_k + B(y_k - M(x^{-ji}_k))$

where $(...)^- \equiv p(...|y_{1:k-1})$. L^{ji}_k has a Gaussian pdf as $\mathcal{N}(Mx^{-ji}_k, MP^{-ji}_k M^\intercal + R)$ which is usually substituted by its zero-mean version, $\mathcal{N}(y_k - Mx^{-ji}_k; 0, MP^{-ji}_k M^\intercal + R)$.

FilteringSEKF: evolution of the EKF from $k-1$ to k.
Require: x^j_{k-1}, P^j_{k-1}, A_i, Γ_i, M,R,G_k,F_k
Ensure: x^{ji}_k, P^{ji}_k,

$i,j = 1:N$
1: $\omega^{-ji}_k = A_i \omega^j_{k-1}$
$\varphi^{-ji}_k = (\varphi^j_{k-1} + \delta\omega^j_{k-1})\mathrm{mod}2\pi$
$$z^{-ji}_k = -\sum_n \delta \frac{\alpha_n \omega^j_{k-1}}{(b_n)^2} \Delta\theta_n \exp\left(-\frac{\Delta(\theta_n)^2}{2(b_n)}\right) + z^j_{k-1}$$
$x^{-ji}_k = [\omega^{-ji}_k \ \varphi^{-ji}_k \ z^{-ji}_k]$
2: $P^{-ji}_k = G_k P^j_{k-1} G_k^\intercal + F_k \Gamma_i F_k^\intercal$
3: $e_k = y_k - Mx^{-ji}_k$
4: $L^{ji}_k = \mathcal{N}(e_k; 0, MP^{-ji}_k M^\intercal + R)$
5: $B = (P^{-ji}_k M^\intercal)(MP^{-ji}_k M^\intercal + R)^{(-1)}$
6: $P^{ji}_k = (I - BM)P^{-ji}_k$
7: $x^{ji}_k = x^{-ji}_k + B(y_k - Mx^{-ji}_k)$

StatesProbability
Require: L^{ji}_k, c_{ji}, K^j_{k-1},
Ensure: K^{ji}_k, K^i_k, $g^{j|i}_k$,

$i,j = 1:N$
1: $K^{ji}_k = \dfrac{L^{ji}_k c_{ji} K^j_{k-1}}{\sum_i \sum_j L^{ji}_k c_{ji} K^j_{k-1}}$
2: $K^i_k = \sum_j K^{ji}_k$
3: $g^{j|i}_k = \dfrac{K^{ji}_k}{K^i_k}$

During the last stage, moment matching is performed for reduction of the mixture components to N Gaussians by the following relations

Collapsing: reducing the number of Gaussian kernels.
Require: x_k^{ji}, P_k^{ji}, $g_k^{j|i}$.
Ensure: x_k^i, P_k^i,

1: $x_k^i = \sum_j x_k^{ji} g_k^{j|i}$

2: $P_k^i = g_k^{j|i}\left(P_k^{ji} + (x_k^{ji} - x_k^i)(x_k^{ji} - x_k^i)^{\mathrm{T}}\right.$

References

Akhbari M, Shamsollahi M B, Jutten C and Coppa B 2012 ECG denoising using angular velocity as a state and an observation in an extended kalman filter framework *Proc. Ann. Int. Conf. IEEE Engineering in Medicine and Biology Society* pp 2897–900

Altuve M, Carrault G, Cruz J, Beuchée A, Pladys P and Hernandez A 2009 Analysis of the QRS complex for apnea-bradycardia characterization in preterm infants *Proc. IEEE Int. Ann. Conf. of the Engineering in Medicine and Biology Society (EMBC 2009)* pp 946–9

Altuve M, Carrault G, Beuchée A, Pladys P and Hernandez A I 2011 On-line apnea-bradycardia detection using hidden semi-Markov models *Proc. 33rd Ann. Int. Conf. IEEE Engineering in Medicine and Biology Society* (*Boston, MA*) vol 1 pp 4374–7

Altuve M, Carrault G, Beuchée A, Pladys P and Hernandez A I 2015 Online apnea–bradycardia detection based on hidden semi-Markov models *Med. Biomed. Eng. Comput.* **53** 1–13

Chazal P, Penzel T and Heneghan C 2004 Automated detection of obstructive sleep apnoea at different time scales using the electrocardiogram *Physiol. Meas.* **25** 967–83

Cruz J, Hernández A, Wong S, Carrault G and Beuchee A 2006 Algorithm fusion for the early detection of apnea-bradycardia in preterm infants *Proc. Computers in Cardiology* (Piscataway, NJ: IEEE) vol 33 pp 473–6

Ghahramani Z and Hinson G 1996 Switching state-space models *Technical Report*

Haskova K, Javorka K, Javorka M, Matasova K and Zibolen M 2013 Apnea in preterm newborns: determinants, pathophysiology, effects on cardiovascular parameters and treatment *Acta Med. Martiniana* **13** 5–17

Lin C, Bugallo M, Mailhes C and Tourneret J Y 2011 ECG denoising using a dynamical model and a marginalized particle filter *Proc. IEEE Asilomar Conf. Signals, Systems and Computers* (*Pacific Grove, CA*) pp 1679–83

Manfredi V, Mahadevan S and Kurose J 2005 Switching kalman filters for prediction and tracking in an adaptive meteorological sensing network *Sensor and Ad Hoc Communications and Networks, 2nd Annual IEEE Communications Society Conf.* pp 197–206

Marculescu R, Marculescu D and Pedram M 1998 Probabilistic modeling of dependencies during switching activity analysis *IEEE Trans. Comput.: Aided Des. Integr. Circuits Syst.* **17** 73–83

Masoudi S, Montazeri N, Shamsollahi M, Beuchée A, Pladys P and Hernandez A I 2013 Early detection of apnea-bradycardia episodes in preterm infants based on coupled hidden Markov model *Proc. IEEE Int. Symp. on Signal Processing and Information Technology* (*Athens, Greece, 12–15 December* 2013) pp 243–8

McNames J and Fraser A 2000 Obstructive sleep apnea classification based on spectrogram patterns in the electrocardiogram *Proc. Computers in Cardiology* (*24–27 September* 2000) pp 749–52

McSharry P E, Clifford G D, Tarassenko L and Smith L A 2003 A dynamic model for generating synthetic electrocardiogram signals *IEEE Trans. Biomed. Eng.* **50** 289–94

Mendez M, Ruini D, Villantieri O, Matteucci M, Penzel T, Cerutti S and Bianchi A 2007 Detection of sleep apnea from surface ECG based on features extracted by an autoregressive model *Proc. 29th Ann. Int. Conf. IEEE Engineering in Medicine and Biology Society* pp 6105–8

Murphy K P 1998 Switching Kalman filters *Technical Report* UC Berkeley

Pan J and Tompkins W J 1985 A real-time QRS detection algorithm *IEEE Trans. Biomed. Eng.* **3** 230–6

Pavlovic V, Rehg J, Cham T and Murphy K 1999 A dynamic Bayesian network approach to figure tracking using learned dynamic models *Proc. 7th IEEE Int. Conf. Computer Vision* (*Corfu, Greece*) vol 1 pp 94–101

Penzel T, McNames J, Chazal P, Raymond B, Murray A and Moody G 2002 Systematic comparison of different algorithms for apnoea detection based on electrocardiogram recordings *Med. Biol. Eng. Comput.* **40** 402–7

Pichardo R, Adam J S, Rosow E, Bronzino J and Eisenfeld L 2003 Vibrotactile stimulation system to treat apnea of prematurity *Biomed. Instrum. Technol.* **37** p 34–40

Poets C, Stebbens V, Samuels M and Southall D 1993 Relationship between bradycardia, apnea, and hypoxemia in preterm infants *Pediatr. Res.* **34** 144–7

Portet F, Gao F, Hunter J and Sripada S 2007 Evaluation of on-line bradycardia boundary detectors from neonatal clinical data *Proc. 29th Ann. Int. Conf. of the IEEE Engineering in Medicine and Biology Society* vol 1 pp 3288–91

Sameni R, Shamsollahi M B, Jutten C and Clifford G 2007 A nonlinear Bayesian filtering framework for ECG denoising *IEEE Trans. Biomed. Eng.* **54** 2172–85

Sayadi O and Shamsollahi M B 2008 ECG denoising and compression using a modified extended Kalman filter structure *IEEE Trans. Biomed. Eng.* **55** 2240–8

Sayadi O, Shamsollahi M B and Clifford G D 2010 Synthetic ECG generation and Bayesian filtering using a gaussian wave-based dynamical model *Physiol. Meas.* **31** 1309–29

Travieso C M, Alonso , J B , del Pozo-Baños M, Ticay-Rivas J R and Lopez-de-Ipiña K 2013 Automatic apnea identification by transformation of the Cepstral domain *Cogn. Comput.* **4** 558–65

Várady P, Micsik T, Benedek S and Benyó Z 2004 A novel method for the detection of apnea and hypopnea events in respiration signals *IEEE Trans. Biomed. Eng.* **49** 936–42

Veeraraghavan H, Schrater P and Papanikolopoulos N 2005 Switching kalman filter-based approach for tracking and event detection at traffic intersections *Proc. IEEE Int. Mediterrean Conf. on Control and Automation Intelligent Control* pp 1167–72

Veeraraghavan H, Papanikolopoulos N and Schrater P 2006 Deterministic sampling-based switching kalman filtering for vehicle tracking *Proc. IEEE Intelligent Transportation Systems Conf.* vol 1 pp 1340–5

Wu Y, Hua G and Yu T 2003 Switching observation models for contour tracking in clutter *Proc. IEEE Computer Society Conf. Computer Vision and Pattern Recognition* vol 1 pp 1–29

Wu W, Black M J, Mumford D, Gao Y, Bienenstock E and Donoghue J P 2004 Modeling and decoding motor cortical activity using a switching Kalman filter *IEEE Trans. Biomed. Eng.* **51** 933–42

Zheng Y and Hasegawa-Johnson M 2003 Acoustic segmentation using switching state Kalman filter *Proc. IEEE Int. Conf. on Acoustics, Speech, and Signal Processing.* vol 1 pp 752–5

Zhao J, Gonzalez F and Mu D 2011 Apnea of prematurity: from cause to treatment *Eur. J. Pediatrics* **170** 1097–105
