# Supplementary material for: Exploring Computational Techniques in Preprocessing Neonatal Physiological Signals for Detecting Adverse Outcomes: Scoping Review
Source: Interact J Med Res. 2024 Aug 20;13:e46946. doi: 10.2196/46946 (PMC11372324; doi:10.2196/46946)
Supplement: Multimedia Appendix 3 [file ijmr_v13i1e46946_app3.zip › Included Papers - Final/3869/Cohen and de Chazal - 2013 - Detection of sleep apnoea in infants using ECG and.pdf]

Detection of Sleep Apnoea in Infants using ECG and Oximetry Signals

Gregory Cohen, Philip de Chazal

MARCS Institute, University of Western Sydney, Sydney, Australia

Abstract

We present a study into the usage of combined night-time electrocardiogram (ECG) and pulse oximetry recordings to automatically detect sleep apnoea in infants.

The study draws upon the polysomnogram recordings found inside the National Collaborative Home Infant Monitoring Evaluation (CHIME) database. Viable ECG data, pulse oximetry data and scored respiratory information was extracted for 288 subjects from this dataset and time-aligned to 30s epochs.

Features were extracted from both the ECG and the pulse-oximetry data and were then used alongside the scored respiratory information to train a classification model based on linear discriminants. Performance of the classifier was evaluated using a leave-one-out cross-validation scheme and an accuracy of 82.6% was achieved, with a specificity of 82.6% and a sensitivity of 58.0%.

1. Introduction

The prevalence of sleep apnoea hypopnea syndrome (SAHS) - a disorder in which the cessation of breathing occurs repeatedly during sleep - has often been estimated to affect approximately 2% to 4% of middle-aged adults [1], but it is also highly prevalent among young children and infants with an estimated 1% to 3% of pre-school children affected by such disorders [2]. Furthermore, it has been shown that young children tend to suffer from more severe episodes of sleep apnoea than adults [3].

Sleep-related breathing disorders in infants has also been shown to have a negative effect on the health and development of infants and young children, and has been linked to conditions such as depression, cognitive impairment, attention-deficit/hyperactivity disorder [4].

It is also a widely under-diagnosed condition; with as many as 80% of cases going undiagnosed [5]. This is in part due to the prohibitively high cost of reliable monitoring and diagnosis of the condition. In-hospital polysomnograms are considered to be the gold standard in the diagnosis of SAHS, but such tests are often prohibitively expensive and even completely unavailable in many countries. The equipment and is also invasive, requiring a multitude of sensors to be attached to the patient. Invasive monitoring of sleep disorders has been shown not to produce good results when used on young children and infants [6].

There is therefore a clear need for a low-cost, minimally invasive means of detecting and diagnosing apnoea events. Building on our previous work in exploring the use of pulse-oximetry data in the automated detection of sleep apnoea in infants [7], we now explore a multimodal approach using both SpO2 data and electrocardiogram (ECG) data.

2. Methodology

The classification system presented in this paper consists of a data processing component, a feature extraction mechanism and a linear discriminants classifier. An overview of the system structure is presented in Figure 1. Details on the specifics of each component are provided below.

2.1. Data

The physiological and annotation data used in this study was obtained from the National Collaborative Home Infant Monitoring Evaluation (CHIME) dataset, which contains overnight physiological recordings from 1079 infants recorded between May 1994 and February 1998 [8].

Infants ranging in age from newborns to 27 weeks were drawn from four groups; children suspected of suffering from apnoea events, healthy term infants, pre-term infants and infants with a history of SIDS in their immediate family.

The CHIME database also contains full laboratory polysomnograms for approximately 700 of the infants in the study. These polysomnograms were recorded using a Healthdyne ALICE3 system comprising a montage of 17 sensors including ECG, EEG and a pulse oximeter.

Authorized licensed use limited to: CSIRO Information Technology Services. Downloaded on September 09,2022 at 05:13:04 UTC from IEEE Xplore. Restrictions apply.

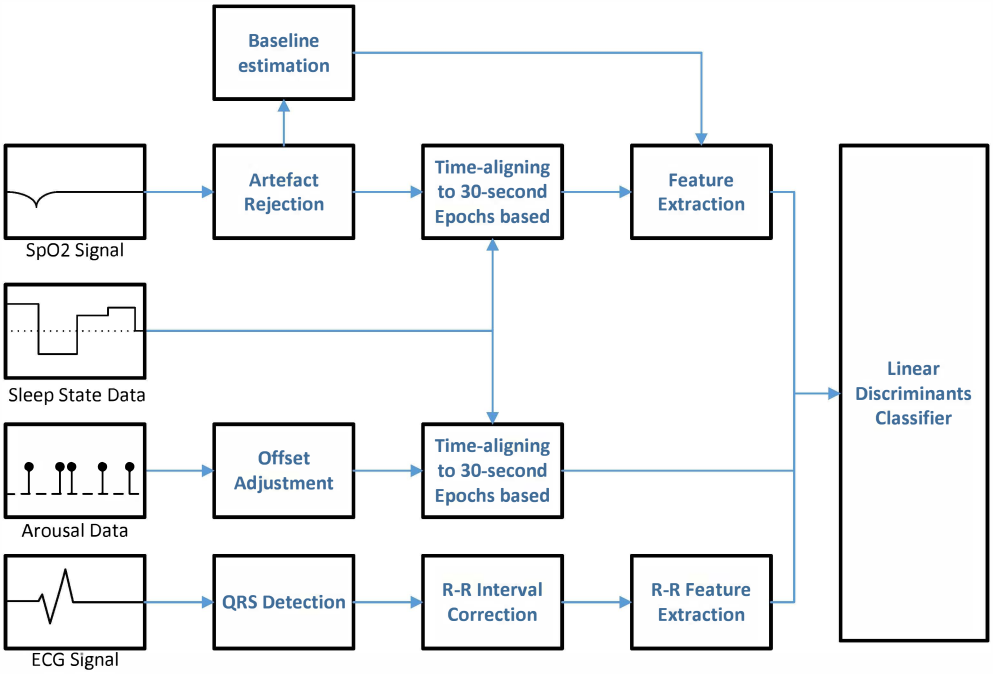

Figure 1. Overview of the SpO2 and ECG Pre-processing and Feature Extraction System.

In addition to the polysomnogram data, the CHIME database also contains extensive recordings taken with the CHIME home monitoring device, which contained six sensors including an Aequitron ECG/Impedance channel and an Ohmeda pulse-oximeter.

Due to a number of factors such as low signal quality, damaged headers and excessively noisy signals, only 288 of the records from the CHIME database could be used to train the classification system. A breakdown of the genders and screening conditions for the subjects used in this study are presented in the Table 1 below:

Screening Criteria	Gender		
	Male	Female	Total
Apnoea of Infancy	32	37	69
Healthy Term	30	28	58
Premature	22	24	46
Sibling of SIDS	54	61	115
**Total**	**138**	**150**	**288**

Table 1: Breakdown of the gender and screening conditions for patients in the study.

Although this study primarily uses the physiological data acquired from the polysomnogram recordings, it is important to note that the CHIME home monitoring device was also used in conjunction with the polysomnogram recording equipment during the laboratory sleep studies. This is significant as the hand-scored respiratory events from the CHIME recordings, and not the polysomnogram recordings, were used to train the classifier in this study.

2.2 Pre-processing

The SpO2 and ECG signals were extracted from each polysomnogram study and time-aligned to 30-second epochs defined by the scoring of the sleep state information extracted for each patient. The sleep state data was also used to identify the first and last periods of active sleep for each subject in order to minimise the introduction of artefacts whilst the subject was awake.

The pulse oximeter used in the ALICE3 system produced a 1Hz oxygen saturation signal representing a rolling three second average. As pulse-oximetry sensors are sensitive to movement and therefore prone to noise, a comprehensive artefact removal step was implemented. Oximetry values below a lower threshold of 65% saturation were discarded, along with any change in saturation exceeding 4% per second. An exclusionary window of ten samples, centred on each artefact was applied to further supress artefact errors.

The ECG data was extracted in the form of a single channel 100Hz signal. A QRS detection algorithm based on the Pan and Tompkins algorithm [9] was used to generate R-R intervals. Due to noise and inaccuracies in the ECG signal and the QRS detector, an R-R interval correction step was performed in which spurious QRS detections were removed and missing QRS intervals approximated using the method outlined in [10]. The filtered R-R intervals were then time-aligned to the same 30-second epochs as used for the SpO2 signal.

Authorized licensed use limited to: CSIRO Information Technology Services. Downloaded on September 09,2022 at 05:13:04 UTC from IEEE Xplore. Restrictions apply.

Respiratory and apnoea annotations were obtained from the scored CHIME monitor recordings which were recorded alongside the polysomnogram data. These annotations were referenced against the internal clock on the CHIME monitor, and not the ALICE3 system used for the polysomnogram recordings and additional calibration data linking the two datasets had to be extracted from the CHIME database and used to correctly adjust the time reference for the annotations before aligning them to the 30-second epoch basis.

2.3. SpO2 feature extraction

A set of seven time-based statistical SpO2 features were chosen for use in this study. The features calculated for each epoch were: as follows:

1. Mean SpO2 Value over the epoch
2. Minimum SpO2 value in the epoch
3. Number of instances below 92% saturation
4. Average absolute rate of change per second in the epoch
5. The 3rd and 57th value in sorted SpO2 values (corresponding to a 5-95% spread)
6. The number of times the baseline value exceeded the SpO2 value by at least 3%
7. The number of times the SpO2 value exceeded the baseline SpO2 by at least 3%

A symmetrical 5-minute rolling-average baseline was calculated for the SpO2 signal to better capture long-term trends within the signal. Two of the selected features utilise the baseline as a proxy for detecting periods of desaturation and re-saturation.

2.4. ECG feature extraction

Four features were calculated from the R-R interval derived from the ECG signal. For each epoch, the following features were calculated:

1. The average R-R interval
2. The standard deviation of the R-R interval
3. The square root of the mean of the sum of the differences between adjacent R-R intervals
4. The Power Spectral Density (PSD) of the R-R intervals (32 features)

The PSD features calculated for each epoch were derived using the method outlined in [9]. For each epoch, the R-R interval values were normalised, padded with zeros to a length of 256 and transformed to the frequency domain by a fast Fourier Transform (FFT).

The coefficients of the magnitude of the FFT were then squared and down-sampled with an averaging filter to 64 values representing the PSD for the epoch. As the PSD is symmetrical, only the first 32 samples of the PSD are required as features.

2.5. Classification

Automatic classification was performed using a linear discriminants classifier. The training data was used to determine the μk-class conditional mean vectors and Σ-common covariance matrix using 'plug-in' maximum likelihood estimates [11].

2.6 Epoch-based performance measures

The linear discriminant classifier was trained to discriminate between normal and any type of sleep disordered breathing (SDB). Each epoch was either labeled ‘Normal’ or ‘SDB’ by the system and the corresponding expert value determined from the respiratory event data.

Each epoch label by the system was compared to the “expert” data derived from the respiratory information and the outcome determined as one of the following:

- True positive (TP): an epoch is labeled as SDB by the respiratory data and labeled as SDB by the system.
- True negative (TN): an epoch is labeled as Normal by the respiratory data and labeled as Normal by the system.
- False negative (FN): an epoch is labeled as Normal by the respiratory data and labeled as SDB by the system.
- False positive (FP): an epoch is labeled as SDB by the respiratory data and labeled as Normal by the system.

The following performance measures were then calculated:

- Specificity = TN/(TN+FP)
- Sensitivity = TP/(TP+FN)
- Accuracy = (TN+TP)/(TP+TN+FN+FP)

A leave-one-out cross-validation scheme was used to assess the ability of the classifier in the face of independent data. In this scheme, one record is left out of the training regime and used to assess the performance of the trained classifier. The record is then cycled with another from the training corpus and the process is repeated until each record has been evaluated in this

Authorized licensed use limited to: CSIRO Information Technology Services. Downloaded on September 09,2022 at 05:13:04 UTC from IEEE Xplore. Restrictions apply.

	Specificity	Sensitivity	Accuracy
7 SpO2 Features	68.5%	55.9%	68.0%
34 ECG Features	76.0%	43.8%	76.0%
41 SpO2 and ECG Features	82.6%	58.0%	82.6%

Table 2. Epoch-based classification results for the oximetry, ECG and combined feature sets

manner. The results are then combined to produce the overall results.

3. Results

The results for the epoch classifier using the SpO2 features, the ECG features and the full montage of features are presented in Table 2.

The results in Table 2 are not AHI-classification results, as the classifier was trained to label epoch as either containing any type of sleep disordered breathing event or as a normal epoch.

It is evident that the full montage of features produced the best result for the classifier and demonstrates that the combination of the two signal paradigms yields a higher accuracy, selectivity and sensitivity. This is due to the complementary diagnostic information of the two sensors – the SpO2 signal captures the desaturations and the ECG captures the heart rate changes associated with the apnoea events.

The epoch classification results found in [12] can be used a point of comparison as it made use of ECG and SpO2 data and extracted similar features. Unlike this study however, the subjects consisted of 125 adult patients. The combined oximetry and ECG epoch classifier yielded a specificity of 94.3%, a sensitivity of 73.4% and an accuracy of 88.8%.

4. Conclusions

It is clear from the results that a multi-modal approach to the detection of sleep apnoea in infants produces a superior result and addresses some of the problems encountered when only a single modality is used.

The physiological differences between adults and infants may also affect the outcome of the classification technique as certain standard assumptions, such as epoch duration and threshold values, may need to be adjusted and tuned for use infant apnoea detection.

References

[1] Young T., Palta M, Dempsey J, Skatrud J, Weber S, Badr, S. The occurrence of sleep-disordered breathing among middle-aged adults. N Engl J Med 328, 1230-1235.

[2] Gislason T, Benediktsdottir B. Snoring apneic episodes, and nocturnal hypoxemia among children 6 months to 6 years old. An epidemiologic study of lower limit prevalence. Chest 107, 963-6.

[3] Brouillette, RT, Fernbach SK, Hunt CE. Obstructive sleep apnea in infants and children. The Journal of pediatrics 1892;100.1: 31-40.

[4] Bonuck K, Freeman K, Chervin RD, Xu L, Bonuck K, Freeman K, Chervin RD, Xu L. Sleep-disordered breathing in a population-based cohort: behavioral out-comes at 4 and 7 years. Pediatrics 2012; 129.4: 857-865.

[5] Vishesh KMD, et al. The medical cost of undiagnosed sleep apnea. Sleep 1999;22.6: 749.

[6] Katz ES, Mitchell RB, D'Ambrosio CM. Obstructive sleep apnea in infants. American journal of respiratory and critical care medicine 2012;185.8: 805-816.

[7] Cohen G, de Chazal P. Automated detection of sleep apnea in infants using minimally invasive sensors. Proceeding of the IEEE EMBS 2013: 1652-1655.

[8] Crowell DH, et al. Infant polysomnography: reliability. Collaborative Home Infant Monitoring Evaluation (CHIME) Steering Committee. Sleep 20.7 (1997): 553.

[9] Pan J, Tompkins WJ. A real-time qrs detection algorithm. IEEE Transactions on Biomedical Engineering 1985; 32:230-236.

[10] de Chazal P, Heneghan C, Sheridan E, Reily R, Nolan P, O'Malley M. Automated processing of the single-lead electrocardiogram for the detection of obstructive sleep apnoea. IEEE Transactions on Biomedical Engineering 2003;50:686-696.

[11] Ripley BD. Pattern recognition and neural networks. Cambridge university press, 2008.

[12] de Chazal P, et al. Home-based assessment of sleep apnea using simultaneous electrocardiogram and oximtery signals. Progress in sleep apnea research 2007: 115-139.

Address for correspondence.

Philip de Chazal
MARCS Institute
University of Western Sydney
Locked Bag 1797, Penrith, NSW, 2751, Australia
p.dechazal@uws.edu.au

Authorized licensed use limited to: CSIRO Information Technology Services. Downloaded on September 09,2022 at 05:13:04 UTC from IEEE Xplore. Restrictions apply.
