# Supplementary material for: Exploring Computational Techniques in Preprocessing Neonatal Physiological Signals for Detecting Adverse Outcomes: Scoping Review
Source: Interact J Med Res. 2024 Aug 20;13:e46946. doi: 10.2196/46946 (PMC11372324; doi:10.2196/46946)
Supplement: Multimedia Appendix 3 [file ijmr_v13i1e46946_app3.zip › Included Papers - Final/3888/Honoré et al. - 2020 - Hidden Markov Models for Sepsis Detection in Prete.pdf]

HIDDEN MARKOV MODELS FOR SEPSIS DETECTION IN PRETERM INFANTS

Antoine Honoré [⋆†], *Dong Liu* [⋆], *David Forsberg* [†], *Karen Coste* [†], *Eric Herlenius* [†],
Saikat Chatterjee [⋆], *Mikael Skoglund* [⋆]

[⋆] Div. Information Science and Engineering, KTH Royal Institute of Technology, Stockholm, Sweden
[†] Dept. Women's and Children's Health, Karolinska Institutet, Stockholm, Sweden

ABSTRACT

We explore the use of traditional and contemporary hidden Markov models (HMMs) for sequential physiological data analysis and sepsis prediction in preterm infants. We investigate the use of classical Gaussian mixture model based HMM, and a recently proposed neural network based HMM. To improve the neural network based HMM, we propose a discriminative training approach. Experimental results show the potential of HMMs over logistic regression, support vector machine and extreme learning machine.

***Index Terms*—** Neonatal Sepsis, Hidden Markov Model

1. INTRODUCTION

Newborn babies who are under care in neonatal intensive care units (NICUs) may rapidly develop infections, including sepsis. It is a standard practice that the diagnosis of a sepsis is aided by the analysis of certain biomarkers from a biological sample such as blood, cerebrospinal fluid or urine. Use of biomarkers has two distinct disadvantages: abnormal levels of biomarkers occur late in the development of the disease, and biomarkers are also known to be unspecific to sepsis. This may lead to delayed treatments, long-term morbidity and death. Our interest is to explore use of physiomarkers for sepsis diagonosis. Physiomarkers are features from physiological signals, for example, respiratory signals.

Infectious diseases alter the heart and breathing patterns in infants [1, 2]. These patterns include clinical events, such as Apnea-Bradycardia-Desaturation (ABD) events [3]. These clinical events are also known to be unspecific as 97% of extremely preterm infants suffer from ABD-events at NICUs [4]. In addition, the developmental age of a preterm infant affects the frequency of such events. NICU at Karolinska University hospital is equipped with bedside monitors performing a continuous recording of vital signs including respiratory frequency (RF), electrocardiogram RR-intervals (RRi) and blood oxygen saturation level (SpO_2). The clinicians receive the data at 1Hz frequency. This is a continuously available sequential data of relevant physiological signals.

In this article we explore the use of HMMs for efficient analysis of the sequential data and prediction of sepsis in preterm infants. We directly use the raw sequential data without any manual feature extraction. To the best of authors' knowledge this line of research has not been done before.

Relevant literature: Heart Rate Observation (HeRO) system [5, 6] is commercially available. HeRO uses features extracted from RRi signal as input to a logistic regression model for sepsis prediction. Extracted features in HeRO are mean, standard deviation and sample asymmetry of RRi signal. RALIS [7] uses age dependent thresholds on RF, RRi, SpO_2, body temperature, desaturation and bradycardia events. Pulse oximetry predictive score (POPS) [8] uses mean, standard deviation, skewness, kurtosis and min-max cross correlation between RR-interval and SpO_2 to compute a risk score. POPS also uses logistic regression.

Use of sequential physiological data for sepsis prediction was recently explored in [9]. The work [9] uses feature extraction from the data and treat them as a static features for further use of machine learning. They do not explore dynamical systems such as HMM. There are two main works [10, 11] where HMM was explored for sepsis prediction. The work [10] uses sequential clinical events such as Bradycardia-Desaturation events and does not use raw physiological signals. On the other hand, the work [11] considers raw physiological data for adults, where HMM state distributions are modelled using kernel density estimators.

Our contributions:

- Motivated by end-to-end learning approaches, we explore direct use of the raw sequential physiological data without any manual feature extraction.
- We explore classical GMM-HMM and recent Flow model based HMM [12]. Flow model is a neural network based distribution modeling method [13].
- For further improvement, we explore use of cross-entropy minimization based discriminative training.
- We compare the performance of HMMs with the performance of logistic regression, support vector machine (SVM) [14] and a popular low complexity neural network called extreme learning machine (ELM) [15].

Notations: We define $\forall n \in \mathbb{N}, [n] = \{1 \cdots n\}$. $E[\cdot]$ denotes the expectation operator. Let $\cdot^{\intercal}$ denote the transpose operation. We denote $\forall I$ interval, $\mathbb{1}_I$ as the indicator function of the interval.

Authorized licensed use limited to: CSIRO Information Technology Services. Downloaded on September 09,2022 at 05:14:37 UTC from IEEE Xplore. Restrictions apply.

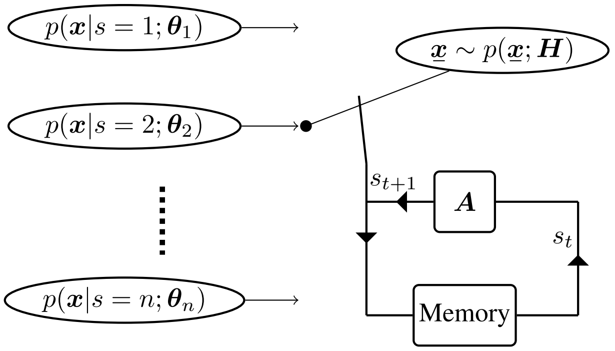

Fig. 1: HMM model illustration [12]

2. HIDDEN MARKOV MODELS - TRADITIONAL AND CONTEMPORARY

HMMs have been shown to be useful in various areas of biology [16], and is widely used for speech recognition tasks [17, 18]. A HMM $\boldsymbol{H}$, is a probabilistic model used to represent time series $\underline{\boldsymbol{x}} = [\boldsymbol{x}_1, \cdots, \boldsymbol{x}_T]^\intercal$, where $\boldsymbol{x}_t \in \mathbb{R}^N$ is the sample at time t and T is the total length. The hypothesis space of HMMs is defined as $\mathcal{H} := \{\boldsymbol{H}|\boldsymbol{H} = \{n, \boldsymbol{q}, \boldsymbol{A}, p(\boldsymbol{x}|i;\boldsymbol{\theta}_i)\}\}$, where

- $n \in \mathbb{N}$ is the number of hidden states in $\boldsymbol{H}$.
- $\boldsymbol{q} = [q_1, q_2, \cdots, q_n]^\intercal$ is the state initialization distribution, i.e. $\forall i \in [n]$, $q_i = p(s_1 = i; \boldsymbol{H})$, where s_1 denotes the state at time 1, i.e. the initial state.
- $\boldsymbol{A} \in R_+^{n\times n}$ is the row stochastic transition matrix, i.e, $\forall i, j \in [n]\times[n]$, $\boldsymbol{A}_{i,j} = p(s_{t+1} = j|s_t = i; \boldsymbol{H})$, where s_t denotes the state at time t.
- $p(\boldsymbol{x}|s;\boldsymbol{\theta}_s)$, $s \in [n]$, is the emission distribution of a sample $\boldsymbol{x} \in \mathbb{R}^N$, where $\boldsymbol{\theta}_s$ is the set of parameters for state s.

At each time instance t, signal $\boldsymbol{x}_t$ is assumed to be sampled from the state emission function $p(\boldsymbol{x}_t|s_t;\boldsymbol{\theta}_{s_t})$, and the sequence of s_t is modeled by a Markov chain. A HMM is depicted in Figure 1.

2.1. Modeling of State Distribution

We use two models for state distribution. In the first case, we use Gaussian mixture model (GMM) as a traditional approach. We do not discuss further GMM based HMM as the model is well known [19, chapter 13.2.1].

In the second case we use neural network based probabilistic model called flow model [13]. The flow model based HMM (Flow-HMM) has recently been explored in [12]. The use of neural network based flow models allows modeling a complex data distribution while being able to compute the likelihood analytically. The exact likelihood computation allows us to train the Flow-HMM in expectation-maximization (EM) approach. We explain in brief the flow model architecture.

For the Flow-HMM, the state distribution $p(\boldsymbol{x}|s;\boldsymbol{\theta}_s)$ is defined as an induced distribution by a generator $\boldsymbol{g}_s : \mathbb{R}^N \to \mathbb{R}^N$, such that $\boldsymbol{x} = \boldsymbol{g}_s(\boldsymbol{z})$, where $\boldsymbol{z}$ is a latent variable following a distribution with density function $q_s(\boldsymbol{z})$. Generator $\boldsymbol{g}_s$ is parameterized by $\boldsymbol{\Phi}_s$. That is, $\boldsymbol{\theta}_s = \{q_s(\boldsymbol{z}), \boldsymbol{\Phi}_s\}$. Assuming $\boldsymbol{g}_s$ is invertible, by change of variable, we have

$$p(\boldsymbol{x}|s;\boldsymbol{\theta}_s) = q_s(\boldsymbol{z}) \left| \det\left(\frac{\partial \boldsymbol{g}_s(\boldsymbol{z})}{\partial \boldsymbol{z}}\right) \right|^{-1}. \quad (1)$$

The parameters of the Flow-HMM are found using expectation-maximization (EM) assisted by gradient search. EM is used in the maximum likelihood sense. The details of the Flow-HMM training algorithm may be found in [12, Algorithm 1].

2.2. Discriminative Flow-HMM (dFlow-HMM)

Certain classes might include a large variety of patterns. Under such conditions, the likelihood of these models w.r.t. incoming input signals will generally be high. To avoid classifying all the signals in these classes, we propose to re-weight using a discriminative step after the Baum-Welch iterations. The implementation of this discriminative training step is intended as a fine tuning step after the initial Flow-HMM training. This discriminative step is performed by maximizing the conditional probability of the correct class given an input training sample. Minimizing the cross-entropy allows us to maximize the quantity:

$$\sum_{(\underline{\boldsymbol{x}},\boldsymbol{y})} \log \frac{p(\underline{\boldsymbol{x}} \mid \boldsymbol{H}_{\boldsymbol{y}})p(\boldsymbol{H}_{\boldsymbol{y}})}{\sum_{j=0}^{1} p(\underline{\boldsymbol{x}} \mid \boldsymbol{H}_j)p(\boldsymbol{H}_j)} \quad (2)$$

where, $\underline{\boldsymbol{x}} = [\boldsymbol{x}_1, \cdots, \boldsymbol{x}_T]^\intercal$ with $\boldsymbol{x}_t \in \mathbb{R}^N$, $t = 1 \ldots T$ is an input training sequence and $\boldsymbol{y} \in \{0, 1\}$ is its class label. The classes prior probabilities $p(\boldsymbol{H}_i)$, $i = 1, 2$ are infered from the training dataset. The probability $p(\underline{\boldsymbol{x}} \mid \boldsymbol{H}_i)$, $i = 1, 2$ of an observed sequence $\underline{\boldsymbol{x}}$ is computed using the forward algorithm.

Computing the denominator of (2) exactly, requires that all training sequences are evaluated by each class HMM. In practice, efficiently computing the denominator is infeasible when the number of classes is too large. In our case, we only have two classes and the computation overhead is manageable. In our implementation, we only update the Flow-models parameters during the maximization of Equation (2). The Markov chain parameters are left unchanged in the discriminative training phase.

We perform the optimization using a stochastic gradient descent approach. The stochastic gradient descent algorithm used here is ADAM [20].

In addition to discriminative training, we use feature extension technique to improve the performance of HMMs. Feature extension was shown to improve the performances of both GMM-HMM and Flow-HMM in practices [12]. Our feature extension consists in concatenating raw inputs with

Authorized licensed use limited to: CSIRO Information Technology Services. Downloaded on September 09,2022 at 05:14:37 UTC from IEEE Xplore. Restrictions apply.

their 1st and 2nd order derivatives. The details about performance comparison with and without feature extension would be shown in the following section.

3. EXPERIMENTAL RESULTS

3.1. Patient Dataset

The bedside monitor signals of 48 premature infants which have been under care at a NICU have been collected. The signals used are the Respiratory Frequency (RF), the beat to beat interval (RRi) and the blood oxygen saturation level (SpO_2). All signals were sampled at 1Hz and segmented into 20 minutes time frames. Time frames containing missing data were discarded. Each time frame was then labelled based on information retrieved from the Electronic Health Records (EHR). Similarly to HeRO and RALIS, we aim at detecting septic events earlier than clinical suspicion of sepsis, defined as the sampling of a blood culture. In our study we use a threshold of 72h prior to blood sample, to label a time frame as "septic". This is in accordance with practices and results from [7] where the RALIS system, was able to trigger sepsis alarms 2.5 days earlier in a subgroup of patients. A time frame was retro-actively labeled 1 if it occurred at most 72h prior to clinical suspicion. A time frame was labeled 0 if it occured during a day when no notes were entered in the infant's EHR. All time frames not labeled either 0 or 1 were discarded.

Our final dataset consists of 22 patients, among which 13 males and 9 females. The birth weight was 1.61 ± 1.10 kg and the gestational age at birth 30.9 ± 6.14. Our dataset consisted in 3501 time frames, among which 1774 with label 0 and 1727 with label 1. All time frames have a constant size of $T = 1200$ samples and are 3-dimensional.

3.2. Baseline Methods

Here we use a baseline model similar to the HeRO model. This model consists in a feature extraction block, followed by a logistic regression. First, the Heart Rate Characteristic index (HRCi) is computed on all time frames. The HRCi computation is summarized by the map

$$h : \underline{\boldsymbol{x}} \mapsto \big(sampen(\underline{\boldsymbol{x}}_{RRi}, r, m), \sigma_{\underline{\boldsymbol{x}}_{RRi}}, sampAs(\underline{\boldsymbol{x}}_{RRi})\big) \in \mathbb{R}^3,$$

where $\underline{\boldsymbol{x}}_{RRi} \in \mathbb{R}^T$ denotes the RRi signal of a time frame $\underline{\boldsymbol{x}}$, $sampen(\underline{\boldsymbol{x}}_{RRi}, r, m)$ denotes the sample entropy of the RRi signal, that is, the probability that $m + 1$ consecutive samples are within a tolerance r from one another, given that m samples are [1]. $sampAs$ is the sample asymmetry, defined as:

$$sampAs : x \mapsto \frac{\sum_{i=1}^{T} \xi_i(x)^2 \mathbb{1}_{\mathbb{R}_+}(\xi_i(x))}{\sum_{i=1}^{T} \xi_i(x)^2 \mathbb{1}_{\mathbb{R}_{*-}}(\xi_i(x))} \in \mathbb{R},$$

where $\forall x \in \mathbb{R}^T \ \xi_i(x) = x_i - \text{median}(x)$. Note that the HeRO system only requires the RRi signal. Here, we do not require sparsity of the results, therefore, we trained a logistic regression model with l_2-regularization. The regularization parameter was learned using a 3-fold cross-validation grid search in the interval $\{10^{-5}, \cdots, 10^5\}$. Additionally we used POPS features [8] defined as: the mean, the standard deviation, the skewness and the kurtosis of the RRi and SpO_2, the min-max cross correlation between the RRi and SpO_2 with a limited lag of 30 seconds. The results associated with these two sets of features are presented in Table 2. We used extreme learning machines (ELM) and support vector machine (SVM) with a gaussian kernel trained on the raw time series data.

3.3. Experiments

We performed binary classification of two types of fixed length input time series in a maximum likelihood framework with GMM, Flow and dFlow -HMMs as probabilistic models. As a baseline, we used the clinically used HRCi index and the more recent POPS features as input to a logistic regression system. We used $m = 4$ and $r = 0.2\sigma_{\underline{\boldsymbol{x}}_{RRi}}$ for the sample entropy computation. We repeated our experiments 3 times and each time a random 30% of the patients was left out for testing. This lead to 2361 ± 353 time series in the training sets and 1140 ± 353 time series in the testing sets. The code was written in Python using the Scikit-learn library for the HeRO system, hmmlearn for GMM-HMM, and PyTorch to implement Flow and dFlow-HMM. The GMM-HMM hyper-parameters were the number of states and the number of Gaussians per state. The number of Gaussians per states was varied between {2,4,6,8,10,12}. For Flow and dFlow-HMMs the hyper-parameters were the number of states, the number of Flow-model per state, the number of chains in each Flow-model, the size of the networks in the coupling layers. Given our limited input dimension, the size of the networks in the coupling layers was fixed to 3. The number of chains in the coupling layer of each Flow-model was varied between 4 and 8. We varied the number of states in our HMMs in $\{3, 6, 9\}$ and show the results in Table 1. For the logistic regression, optimal regularization parameter was found with cross-validation and grid search in the set $\{10^{-5}, \cdots, 10^5\}$. We used a different set of input features to test our models in different conditions. HMMs are trained on raw time series and on raw time series with first and second order derivatives. The logistic regression model is trained on HRCi, 3-dimensional feature, and on POPS, 10-dimensional features.

3.4. Numerical Results

The results for the linear prediction systems are presented in Table 2 and the results for the HMMs are presented in Table 1. The bold fonts corresponds to the maximum performance across HMM models given a number of states.

Authorized licensed use limited to: CSIRO Information Technology Services. Downloaded on September 09,2022 at 05:14:37 UTC from IEEE Xplore. Restrictions apply.

Table 1: Test accuracy of HMMs

Number of states	n=3	n=6	n=9
Raw time series			
GMM-HMM	0.68 ± 0.03	**0.68** ± 0.03	**0.69** ± 0.03
FlowHMM	0.67 ± 0.04	0.61 ± 0.08	0.63 ± 0.08
dFlowHMM	**0.70** ± 0.10	0.67 ± 0.06	0.65 ± 0.04
Raw time series + 1st and 2nd order derivatives			
GMM-HMM	**0.75** ± 0.05	**0.74** ± 0.08	**0.74** ± 0.05
FlowHMM	0.69 ± 0.07	0.66 ± 0.06	0.59 ± 0.08
dFlowHMM	0.71 ± 0.04	0.72 ± 0.10	0.67 ± 0.04

Table 2: Comparison of HMM with other models

Model	Variety	
Using features		
Logistic Regression	POPS	0.54±0.01
	HeRO	0.57±0.04
Using raw sequential data		
SVM		0.60 ± 0.04
ELM		0.60 ± 0.03
dFlowHMM		0.70 ± 0.10

The HeRO (HRCi + logistic regression) reaches 57% of correct classifications. It outperforms the POPS algorithms which reaches only 54% and is the lowest performing algorithm here. As expected, the linear classifiers are outperformed by Gaussian kernel SVM, ELM and our HMMs. SVM and ELM both reach an accuracy of 60% and comparable standard deviations of 4% and 3%. This is lower than dFlow-HMM which reaches 70% accuracy and outperforms both Flow-HMM 67%, and GMM-HMM 68% when the number of states $n = 3$ and for raw-time series. These results are contrasted by the large standard deviation of dFlow-HMM 10%, which is larger than both Flow-HMM 4% and GMM-HMM 3%. When the number of states increases to $n = 6$ and $n = 9$, GMM-HMM reaches 68% and 69% which outperforms Flow-HMM with 61% and 63%, and dFlow-HMM with 67% and 65%. When the input time series is augmented with 1st and 2nd order derivatives, GMM-HMM reaches its highest performance with 75% accuracy at $n = 3$. Flow-HMM and dFlow-HMM also reach their highest performance with 69% at $n = 3$ and 72% at $n = 6$. Here, GMM-HMM outperforms both Flow and dFlow-HMM.

3.5. Discussion

The HeRO model surprisingly under-performs on our dataset. This poor performance may be due to inconsistencies in the sepsis definition. The definition of a sepsis varies between the different studies, here we include culture positive and culture negative which differs from the initial HERO study [6] but is in accordance with the definitions used in the more recent RALIS study [21].

The performance of GMM-HMM is significantly increased when adding 1st and 2nd order derivative as part of the input. This is in accordance with the initial studies performed on speech processing tasks [12]. Our attempt to improve the performance of Flow-HMM using discriminative training was successful for both raw time series and 1st and 2nd order derivatives inputs. This is encouraging, given that our current discriminative training consists of only one epoch. We expect the marginal gain of discriminative training to improve the performance of dFlow-HMM even further with more iterations. However, we note a decrease in performance of Flow-HMM and dFlow-HMM as the number of hidden states is increased. This is due to the fact that there are more parameters to learn as we increase the hidden states, for the same amount of data. Among all the tested systems, GMM-HMM has the smallest standard deviation, which indicates that this model is robust to changes in training dataset. Flow models were originally designed for high-dimensional data distribution modeling. As expected, additional dimensions in Flow-HMM input lead to improvement in performances compared to the 3-dimensional case. We conjecture that Flow-HMM and dFlow-HMM suffer from insufficient training data, considering the fact that more parameters are to be learnt than GMM.

4. CONCLUSION

We studied the performance of Hidden Markov Models compared to state-of-art logistic regression based classification models for neonatal sepsis detection. We showed that, on our dataset consisting of 22 patients, neonatal sepsis detection may be enhanced with the use of Hidden Markov Models. We observed that Gaussian Mixture Models for state emission probability distributions performs well, and with a low standard deviation compared to other models. dFlow-HMM was shown to outperform GMM-HMM in a limited number of scenarios. This may constitute an important building block in the future design of Flow-model based Hidden Markov Model. Adding the derivatives of the signal as an input, lead to improvement of the HMMs. Our study paves the way for further research on Hidden Markov Models topology which may lead to improved neonatal sepsis detection in NICUs.

5. ACKNOWLEDGEMENTS

This project was approved by the regional ethics review board (2011/1891-31/2). Informed consent was provided by all parents/guardians. This study was supported by KTH-SLL collaborative grant HMT (2016-0764 and 20180866), the Swedish National Heart and Lung (20180505) and the Brain Foundation (FO2017-0203). The authors are grateful to Baptiste Cavarec for helpful discussions and detailed comments on an early draft.

Authorized licensed use limited to: CSIRO Information Technology Services. Downloaded on September 09,2022 at 05:14:37 UTC from IEEE Xplore. Restrictions apply.

6. REFERENCES

[1] M Pamela Griffin, T Michael O'Shea, Eric A Bissonette, Frank E Harrell, Douglas E Lake, and J Randall Moorman, "Abnormal Heart Rate Characteristics Preceding Neonatal Sepsis and Sepsis-Like Illness," *Pediatr Res*, vol. 53, no. 6, pp. 920–926, June 2003.

[2] Veronica Siljehav, Annika M. Hofstetter, Kristin Leifsdottir, and Eric Herlenius, "Prostaglandin E2 Mediates Cardiorespiratory Disturbances during Infection in Neonates," *The Journal of Pediatrics*, vol. 167, no. 6, pp. 1207–1213.e3, Dec. 2015.

[3] Karen Fairchild, Mary Mohr, Alix Paget-Brown, Christa Tabacaru, Douglas Lake, John Delos, Joseph Randall Moorman, and John Kattwinkel, "Clinical associations of immature breathing in preterm infants: Part 1—central apnea," *Pediatr Res*, vol. 80, no. 1, pp. 21–27, July 2016.

[4] A. O. Hofstetter, L. Legnevall, E. Herlenius, and M. Katz-Salamon, "Cardiorespiratory development in extremely preterm infants: Vulnerability to infection and persistence of events beyond term-equivalent age," *Acta Paediatrica*, vol. 97, no. 3, pp. 285–292, 2008.

[5] Jamie Fletcher Hicks and Karen Fairchild, "Heart rate observation (HeRO) monitoring was developed for detection of sepsis in preterm infants.[...] The HeRO monitor is now in use in many NICUs in the USA and was approved in 2012 for use in Europe.," p. 5, 2013.

[6] M. P. Griffin, "Heart Rate Characteristics: Novel Physiomarkers to Predict Neonatal Infection and Death," *PEDIATRICS*, vol. 116, no. 5, pp. 1070–1074, Nov. 2005.

[7] Ilan Gur, Gal Markel, Yaron Nave, Igor Vainshtein, Arik Eisenkraft, and Arieh Riskin, "A mathematical algorithm for detection of Late-onset Sepsis in very-low birth weight infants: A preliminary diagnostic test evaluation," *Indian Pediatr*, vol. 51, no. 8, pp. 647–650, Aug. 2014.

[8] B. Sullivan, A. Wallman-Stokes, J. Isler, R. Sahni, J. Moorman, K. Fairchild, and D. Lake, "Early Pulse Oximetry Data Improves Prediction of Death and Adverse Outcomes in a Two-Center Cohort of Very Low Birth Weight Infants," *Amer J Perinatol*, vol. 35, no. 13, pp. 1331–1338, Nov. 2018.

[9] Işıl Güzey and Özlem Uçar, "Machine Learning Based Non-Invasive Vital Signs Analysis for Preterm Sepsis Risk Prediction," SSRN Scholarly Paper ID 3319633, Social Science Research Network, Rochester, NY, Dec. 2018.

[10] Ioan Stanculescu, Christopher K. I. Williams, and Yvonne Freer, "Autoregressive Hidden Markov Models for the Early Detection of Neonatal Sepsis," *IEEE J. Biomed. Health Inform.*, vol. 18, no. 5, pp. 1560–1570, Sept. 2014.

[11] Jacquelyn D. Parente, Knut Möller, Geoffrey M. Shaw, and J. Geoffrey Chase, "Hidden Markov Models for Sepsis Classification," *IFAC-PapersOnLine*, vol. 51, no. 27, pp. 110–115, 2018.

[12] Dong Liu, Antoine Honore, Saikat Chatterjee, and Lars K. Rasmussen, "Powering Hidden Markov Model by Neural Network based Generative Models," *arXiv:1910.05744 [cs, stat]*, Oct. 2019.

[13] Laurent Dinh, David Krueger, and Yoshua Bengio, "NICE: Non-linear Independent Components Estimation," *arXiv:1410.8516 [cs]*, Oct. 2014.

[14] Ingo Steinwart and Andreas Christmann, *Support Vector Machines*, Information Science and Statistics. Springer-Verlag, New York, 2008.

[15] Guang-Bin Huang, Hongming Zhou, Xiaojian Ding, and Rui Zhang, "Extreme Learning Machine for Regression and Multiclass Classification," *IEEE Trans. Syst., Man, Cybern. B*, vol. 42, no. 2, pp. 513–529, Apr. 2012.

[16] Byung-Jun Yoon, "Hidden Markov Models and their Applications in Biological Sequence Analysis," *Curr Genomics*, vol. 10, no. 6, pp. 402–415, Sept. 2009.

[17] Lawrence R Rabiner, "A Tutorial on Hidden Markov Models and Selected Applications in Speech Recognition," *PROCEEDINGS OF THE IEEE*, vol. 77, no. 2, pp. 30, 1989.

[18] S. Chatterjee and W. B. Kleijn, "Auditory Model-Based Design and Optimization of Feature Vectors for Automatic Speech Recognition," *IEEE Transactions on Audio, Speech, and Language Processing*, vol. 19, no. 6, pp. 1813–1825, Aug. 2011.

[19] Christopher M. Bishop, *Pattern Recognition and Machine Learning (Information Science and Statistics)*, Springer-Verlag, Berlin, Heidelberg, 2006.

[20] Diederik P. Kingma and Jimmy Ba, "Adam: A Method for Stochastic Optimization," *arXiv:1412.6980 [cs]*, Dec. 2014.

[21] Leena Bhattacharya Mithal, Ram Yogev, Hannah Palac, Ilan Gur, and Karen K. Mestan, "Computerized vital signs analysis and late onset infections in extremely low gestational age infants," *Journal of Perinatal Medicine*, vol. 44, no. 5, Jan. 2016.

Authorized licensed use limited to: CSIRO Information Technology Services. Downloaded on September 09,2022 at 05:14:37 UTC from IEEE Xplore. Restrictions apply.
