# Supplementary material for: Exploring Computational Techniques in Preprocessing Neonatal Physiological Signals for Detecting Adverse Outcomes: Scoping Review
Source: Interact J Med Res. 2024 Aug 20;13:e46946. doi: 10.2196/46946 (PMC11372324; doi:10.2196/46946)
Supplement: Multimedia Appendix 3 [file ijmr_v13i1e46946_app3.zip › Included Papers - Final/3915/Cohen and de Chazal - 2014 - A multi-modal approach to sleep-wake classificatio.pdf]

A Multi-modal Approach to Sleep-Wake Classification in Infants using Minimally Invasive Sensors

Gregory Cohen, Philip de Chazal

MARCS Institute, University of Western Sydney, Sydney, Australia

Abstract

In this study, we evaluate the potential and efficiency of a low-cost and minimally invasive means of identifying sleep/awake patterns in infants using a combination of pulse-oximetry, electrocardiogram and actigraphy data. Full overnight polysomnogram data from 402 infants from four distinct screening categories was extracted from the National Collaborative Home Infant Monitoring Evaluation (CHIME) database along with hand-scored sleep state annotations and was used to train and validate a classifier model based on linear discriminants.

Results for each screening condition are provided along with the overall results across the entire dataset. The overall classifier achieved an accuracy of 74.1%, a sensitivity of 82.0% and a specificity of 60.9%.

1. Introduction

Sleep/wake identification is an important diagnostic tool in the realm of sleep medicine. It is important in the both the assessment and diagnosis of many sleep-related conditions and is particularly important in infants and newborns, as sleep-wake patterns have been shown to be an indicator of neurobehavioral organisation [1], cognitive status [2] and is used in the detection of sleep-related breathing disorders such as bradycardia and apneas [3].

Sleep-wake identification is traditionally performed using electroencephalography (EEG) signals but despite being the gold-standard method of detecting sleep state, EEG recording equipment is costly, and intrusive, limiting its applicability and efficacy for sensitive groups such as infants [4] and limiting the possibility of in-home monitoring. Therefore, there exists a clear need to explore additional means of determining sleep-state from minimally invasive sensors.

This research focuses on the application of a multi-modal approach combining ECG, pulse oximetry and actigraphy sensors. These sensors were chosen as they were felt to be better tolerated by infants than EEG sensor and would complement and assist in devices for the automated detection of sleep apnea in infants.

2. Methodology

The classification system presented in this paper consists of a data extraction stage, a pre-processing and artefact rejection stage, a feature extraction stage and a linear discriminants classifier. An overview of the system is shown in Figure 1.

2.1. Data Extraction

The physiological data and annotations used in this study were obtained from the National Collaborative Home Infant Monitoring (CHIME) dataset collected between May 1994 and February 1998 [5]. The CHIME study group selected subjects from four screening categories; children suspected of suffering from apnoea events, healthy term infants, pre-term infants and siblings of SIDS. The database contains recordings obtained using a home monitor and full in-laboratory polysomnogram recordings from 1079 infants between 0 to 27 weeks old.

Although the database contains home recordings from 1079 infants, only 660 had full polysomnograms and only 403 contained properly annotated sleep/wake states. One record was discarded to due a damaged header, and the remaining 402 of the records from the CHIME database were used to train the classification system

Electrocardiogram (ECG), pulse oximetry (SpO2) and actigraphy data from the polysomnogram recordings were used as inputs to the classification system. The ECG device used to obtain the data was a Contour S30 1B device sampled with 8-bit resolution at 100Hz, the pulse-oximeter was an Ohmeda SoftProbe 2900-222 with a 1Hz output representing a 3-second averaged reading and the actigraphy data was generated from a 50Hz, 8-bit Healthdyne accelerometer.

The sleep-state scoring performed by the CHIME group was done using the full polysomnograms and was primarily guided by the EEG signals captured during that setup.

2.2. Pre-Processing

The pulse-oximetry data, ECG signal, actigraphy data

Authorized licensed use limited to: CSIRO Information Technology Services. Downloaded on September 09,2022 at 05:16:08 UTC from IEEE Xplore. Restrictions apply.

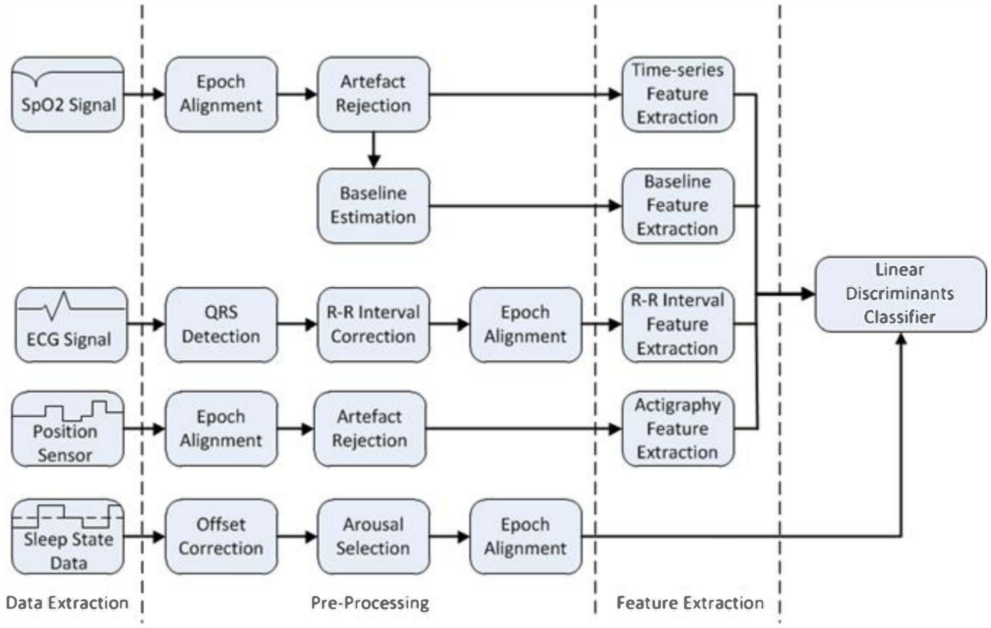

Figure 1: Diagram of the structure of the classification system

and sleep state data were all extracted from the polysomnogram recordings and were time-aligned to the same 30-second epochs.

An artefact rejection step was performed on the epoch-aligned oximetry values in which any values below 65% saturation were automatically excluded, and any values contributing to a change in oxygen saturating exceeding 4% per second were also removed.

A similar artefact rejection step was also performed on the actigraphy data based on the methods outlined in [6].

The ECG data was passed through a QRS detection algorithm to produce R-R intervals and an R-R interval correction step was implemented in which spurious QRS detections were removed and missing QRS intervals were inserted using the method outlined in [7].

2.3. SpO2 Feature Extraction

There is a wide body of existing research on feature generation from pulse-oximetry data for apnoea detection. The majority of methods use time-based statistical properties, which are subject to limitations arising from physiological effects, sensor specificity and sensor location [8]. The following seven well-established time-based features were calculated for each epoch:

1. Mean SpO2 value over the epoch
2. Minimum SpO2 value in the epoch
3. Number of instances below 92% saturation
4. Average absolute rate of change per second of SpO2 values in the epoch
5. The 3rd and 57th value in sorted SpO2 values (corresponding to a 5-95% spread)
6. The number of times the baseline value exceeded the SpO2 value by at least 3%
7. The number of times the SpO2 value exceeded the baseline SpO2 by at least 3%

The baseline utilised in the last two features is a symmetrical 5-minute rolling-average calculated over the SpO2 signal to better capture long-term trends within the signal.

2.4. ECG Feature Extraction

All the features extracted from the ECG data were based around variations and fluctuations in the R-R intervals, which were extracted from the data as part of the pre-processing steps listed above.

Four features were calculated from the R-R intervals derived from the ECG signal. For each epoch, the following features were calculated:

1. The average R-R interval
2. The standard deviation of the R-R interval
3. The square root of the mean of the sum of the differences between adjacent R-R intervals
4. The Power Spectral Density (PSD) of the R-R intervals (32 features)

The PSD features calculated for each epoch were derived using the method outlined in [9]. For each epoch, the R-R interval values were normalised, padded with zeroes to a length of 256 and transformed to the frequency domain by a fast Fourier Transform (FFT).

The coefficients of the magnitude of the FFT were then squared and down-sampled with an averaging filter to 64 values representing the PSD for the epoch. As the PSD is symmetrical, only the first 32 samples of the PSD are

Authorized licensed use limited to: CSIRO Information Technology Services. Downloaded on September 09,2022 at 05:16:08 UTC from IEEE Xplore. Restrictions apply.

Table 1: Classification results by screening condition

	Healthy Term			Apnea of Infancy			Premature			Sibling of SIDS		
	Acc	*Sens*	*Spec*	*Acc*	*Sens*	*Spec*	*Acc*	*Sens*	*Spec*	*Acc*	*Sens*	*Spec*
ECG + SPO2 + ACT	77.74	86.72	61.31	75.87	86.79	57.87	71.49	77.03	63.54	74.70	83.68	67.16
ECG + SPO2	77.51	86.59	60.89	75.68	86.32	58.14	71.08	76.40	62.89	74.67	83.17	66.78
ACT	68.33	91.09	26.88	68.43	93.21	28.26	65.76	92.44	68.36	67.99	90.69	63.74
SPO2	77.58	86.70	60.88	75.58	86.79	58.00	70.87	76.09	62.56	74.87	83.52	67.22
ECG	70.30	86.97	39.93	68.06	85.06	40.49	65.44	80.95	58.90	68.65	85.45	60.90

required as features.

2.5. Actigraphy Feature Extraction

Three features were extracted and calculated from the actigraphy data. These three features include:

1. The mean accelerometer value over the epoch
2. The standard deviation of the accelerometer value over the epoch
3. The Power Spectral Density (PSD) of accelerometer values (32 features)

The accelerometer used in the CHIME study was placed in an unusual position on the infants. It was attached to the infant's diaper as opposed to on an extremity, in order to provide both accelerometer data and position data [10]. This trade-off resulted in a loss of sensitivity to motion and increased artefact noise due to breathing, heartbeat and gastro-intestinal noises.

The DC-level of the accelerometer signal was used to provide a rough estimate of the position of the infant in the original CHIME monitor, and therefore the mean value and the standard deviation of the signal were chosen as features in order to attempt to capture the position of the infant. The power spectral density of the accelerometer data was chosen to capture the dynamics resulting from infant motion and is calculated in a similar fashion to the PSD for the ECG features.

2.6. Linear Discriminants Classifier

A linear discriminant classifier was used for automatic classification. The classifier parameters [11] (class means $\boldsymbol{\mu}_k$ and common covariance $\boldsymbol{\Sigma}$) for linear discriminants can be determined from the training data examples using

$$\boldsymbol{\mu}_k = \sum_{n=1}^{N_k} \mathbf{x}_{kn} \Big/ N_k \ , \boldsymbol{\Sigma} = \sum_{k=1}^{c}\sum_{n=1}^{N_k} \left(\mathbf{x}_{kn} - \boldsymbol{\mu}_k\right)\left(\mathbf{x}_{kn} - \boldsymbol{\mu}_k\right)^T \Big/ N \quad (1)$$

where the number of classes is c (2 in our case), the number of training examples in class k is N_k, the feature vector of the nth training example belonging to class k is denoted $\mathbf{x}_{kn}$. Note that the total number of epochs used for training is $N = \sum_{n=1}^{c} N_k$.

A feature vector x is classified by calculating the estimated posterior probabilities, $P(k \mid \mathbf{x})$ for the kth class using

$$P(k \mid \mathbf{x}) = \exp(y_k) \Big/ \sum_{l=1}^{c} \exp(y_l) \text{, where}$$

$$y_k = -\tfrac{1}{2}\boldsymbol{\mu}_k^T \boldsymbol{\Sigma}^{-1} \boldsymbol{\mu}_k + \boldsymbol{\mu}_k^T \boldsymbol{\Sigma}^{-1} \mathbf{x} \quad (2).$$

The final classification of the system is the class with the highest posterior probability estimate.

2.7. Epoch-based Performance Measures

The linear discriminant classifier was trained to discriminate between awake and any other stage of sleep (including both quiet and active sleep). Each epoch was either labelled 'Awake' or 'Asleep' by the system and the corresponding expert value determined from the sleep stage data.

Each epoch label by the system was compared to the "expert" data extracted from the hand-scored sleep annotations and the outcome determined as one of the following:

- True positive (TP): an epoch labelled asleep by the respiratory data and labelled as asleep by the system.
- True negative (TN): an epoch labelled awake by the respiratory data and labelled as asleep by the system.
- False negative (FN): an epoch labelled awake by the respiratory data and labelled as asleep by the system.
- False positive (FP): an epoch labelled asleep by the respiratory data and labelled as awake by the system.

The following performance measures were then calculated:

Specificity = TN/(TN+FP),
Sensitivity = TP/(TP+FN) and
Accuracy = (TN+TP)/(TP+TN+FN+FP)

A leave-one-out cross-validation scheme was used to assess the ability of the classifier in the face of independent data. The testing results were aggregated across the 402 records and the classification performance values calculated from this aggregated set.

Authorized licensed use limited to: CSIRO Information Technology Services. Downloaded on September 09,2022 at 05:16:08 UTC from IEEE Xplore. Restrictions apply.

3. Results

Various combinations of sensor inputs were tested with the classifier and tested on both the entire collection of viable records and each screening condition. The results for each screening condition are in Table 1. The results from the entire dataset are presented in Table 2.

Table 2: Classification results for the entire dataset

	Accuracy	Sensitivity	Specificity
ECG, SPO2, ACT	74.1 %	82.0 %	60.9 %
ECG, SPO2	74.4 %	82.8 %	60.2 %
ACT	67.7 %	93.8 %	24.6 %
SPO2	73.8 %	81.6 %	60.7 %
ECG	67.0 %	83.0 %	40.6 %

4. Discussion

The results of this study show that a montage of sensors has a clear advantage over any single modality. The combination of all modalities produced the highest accuracy for each condition, with the exception of children who are siblings of SIDS, where the accuracy was marginally higher.

These results can be favorably compared to the results in [6], which explored sleep-wake identification using heart-rate variability and actigraphy data and achieved a prediction accuracy of 89.5% for predicting sleep state, and 56.5% for predicting awake states, yielding an overall accuracy of 78.7% by using a fuzzy clustering algorithm to find features that best separated the data.

A later study focusing on evaluating algorithms for actigraphy can also be used for comparison [12]. Using subsets of recordings from a subset of the CHIME database, they were able to achieve an accuracy of 75.3%, a sensitivity of 81.3% and a specificity of 61.2% on actigraphy alone. Unlike those results, the methods used in this study considered the dataset in its entirely and the results presented show here reflect the true performance of the algorithm and system on all available data.

5. Conclusions

The results of this study clearly demonstrate the potential of a multi-modal approach to the automated identification sleep-awake identification in infants.

The unusual placement of the accelerometer on the infant's diaper may have led to the lower than expected performance of the actigraphy modality in the results. The techniques used in this study may work better on data collected from sensors with higher sensitivity, or with sensors placed on the extremities, where more motion can be expected.

Acknowledgements

This work was supported by ARC grant FT110101098.

References

[1] Freudigman KA, Thoman EB. Infant Sleep During the First Postnatal Day: An Opportunity for Assessment of Vulnerability. Pediatrics. 1993;92(3):373-379.

[2] Gertner S, Greenbaum CW, Sadeh A, Dolfin Z, Sirota L, Ben-Nun Y. Sleep–wake patterns in preterm infants and 6 month's home environment: implications for early cognitive development. Early Hum Dev. 2002;68(2):93-102.

[3] X. Xu, "Prediction of Life-Threatening Events in Infants Using Heart Rate Variability Measurements," West Virginia University, 2002..

[4] Marcus CL. Sleep-disordered breathing in children. Am J Respir Crit Care Med. 2001;164(1):16-30. doi:10.1164/ajrccm.164.1.2008171.

[5] Crowell DH, Brooks LJ, Colton T, et al. Infant polysomnography: reliability. Collaborative Home Infant Monitoring Evaluation (CHIME) Steering Committee. Sleep. 1997;20(7):553-60.

[6] Lewicke, A., Sazonov E, Schuckers, S. Sleep-wake identification in infants: heart rate variability compared to actigraphy. Conf Proc IEEE Eng Med Biol Soc. 2004;1:442-5. doi:10.1109/IEMBS.2004.1403189.

[7] de Chazal P, Heneghan C, Sheridan E, Reilly R, Nolan P, O'Malley M. Automated processing of the single-lead electrocardiogram for the detection of obstructive sleep apnoea. IEEE Trans Biomed Eng. 2003;50(6):686-96. doi:10.1109/TBME.2003.812203.

[8] Morillo DS, Gross N, León A, Crespo LF. Automated frequency domain analysis of oxygen saturation as a screening tool for SAHS. Med Eng Phys. 2012;34(7):946-953.

[9] Shouldice RB, O'Brien LM, O'Brien C, de Chazal P, Gozal D, Heneghan C. Detection of obstructive sleep apnea in pediatric subjects using surface lead electrocardiogram features. Sleep. 2004;27(4):784-92.

[10] Sazonov E, Sazonova N, Schuckers S, Neuman M, Group CS. Activity-based sleep–wake identification in infants. Physiol Meas. 2004;25(5):1291-1304..

[11] Ripley BD. Pattern Recognition and Neural Networks. 2007.

[12] Tilmanne J, Urbain J, Kothare M V, Wouwer A Vande, Kothare S V. Algorithms for sleep-wake identification using actigraphy: a comparative study and new results. J Sleep Res. 2009;18(1):85-98. doi:10.1111/j.1365-2869.2008.00706.x.

Address for correspondence.

Greg Cohen
MARCS Institute
University of Western Sydney
Locked Bag 179,Penrith, NSW, 2751,Australia

Email: g.cohen@uws.edu.au

Authorized licensed use limited to: CSIRO Information Technology Services. Downloaded on September 09,2022 at 05:16:08 UTC from IEEE Xplore. Restrictions apply.
