# Supplementary material for: Exploring Computational Techniques in Preprocessing Neonatal Physiological Signals for Detecting Adverse Outcomes: Scoping Review
Source: Interact J Med Res. 2024 Aug 20;13:e46946. doi: 10.2196/46946 (PMC11372324; doi:10.2196/46946)
Supplement: Multimedia Appendix 3 [file ijmr_v13i1e46946_app3.zip › Included Papers - Final/4372/Peng et al. - 2022 - DeepLOS Deep learning for late-onset sepsis predi.pdf]

Smart Health 26 (2022) 100335

Contents lists available at ScienceDirect

Smart Health

journal homepage: www.elsevier.com/locate/smhl

DeepLOS: Deep learning for late-onset sepsis prediction in preterm infants using heart rate variability

Zheng Peng [a,d,*], Gabriele Varisco [a,d], Rong-Hao Liang [b,c], Deedee Kommers [a,e], Ward Cottaar [a], Peter Andriessen [a,e], Carola van Pul [a,d], Xi Long [c,f,**]

[a] Applied Physics, Eindhoven University of Technology, De Zaale, 5600 MB, Eindhoven, The Netherlands
[b] Industrial Design, Eindhoven University of Technology, De Zaale, 5600 MB, Eindhoven, The Netherlands
[c] Electrical Engineering, Eindhoven University of Technology, De Zaale, 5600 MB, Eindhoven, The Netherlands
[d] Clinical Physics, Máxima Medical Centre, De Run 4600, 5504 DB, Veldhoven, The Netherlands
[e] Neonatology, Máxima Medical Centre, De Run 4600, 5504 DB, Veldhoven, The Netherlands
[f] Philips Research, HTC 34, 5656 AE, Eindhoven, The Netherlands

ARTICLE INFO

Keywords:
Deep learning
Heart rate variability
Late-onset sepsis
Machine learning
Predictive monitoring
Preterm infants

ABSTRACT

Late onset sepsis (LOS) is one of the main causes of death in preterm infants in a neonatal intensive care unit (NICU). LOS can be better treated with early detection, reducing its morbidity and mortality. In this study, an end-to-end deep learning model called DeepLOS was developed to predict LOS in preterm infants in a NICU. The model is based on a residual convolutional neural network (ResNet) with feature map (or channel) attention and uses RR intervals (i.e., interbeat intervals) as input. The model was trained and tested on a dataset composed of 128 preterm infants (60 blood-culture-proven LOS patients and 68 control patients). To minimize the possible age effect on modeling, we also considered an age-matched dataset including 32 LOS and 32 control patients from the full dataset. Prediction was done with a one-hour (non-overlapping) sliding window from 24 h before LOS to onset of LOS. We used 5-fold patient-independent cross validation and F-score to evaluate the model performance. The DeepLOS achieves an F-score of 0.72 for the full dataset and 0.73 for the matched dataset in LOS prediction for all one-hour segments, outperforming the baseline ResNet model without channel attention. F-score is generally higher (>0.75) when coming closer to the onset of LOS. Our study demonstrates the feasibility of deep learning for end-to-end LOS prediction in preterm infants. Furthermore, the model uses readily available RR intervals as input only; and is therefore vendor-processing independent and has the potential to be easily deployed in different NICUs.

1. Introduction

Neonates who are born prematurely (GA < 37 weeks) and particularly the group of very prematurely born infants (GA < 32 weeks) are often admitted and hospitalized in a neonatal intensive care unit (NICU) for optimal treatment and monitoring. Although the advances in neonatal intensive healthcare have led to a steady decline of neonatal mortality over years (Phibbs et al., 2022), the mortality rate of neonatal sepsis, a clinical syndrome caused by invasion of pathogens, can still reach up to 11.3% in preterm infants in NICUs (Al-Matary et al., 2019). Neonatal sepsis can be categorized as early-onset or late-onset sepsis (LOS), depending on

* Corresponding author at: Applied Physics, Eindhoven University of Technology, De Zaale, 5600 MB, Eindhoven, The Netherlands.
** Corresponding author at: Electrical Engineering, Eindhoven University of Technology, De Zaale, 5600 MB, Eindhoven, The Netherlands.
E-mail addresses: z.peng@tue.nl (Z. Peng), x.long@tue.nl (X. Long).

https://doi.org/10.1016/j.smhl.2022.100335
Received 29 September 2022; Accepted 29 September 2022
Available online 5 October 2022
2352-6483/© 2022 Published by Elsevier Inc.

being diagnosed within 72 h after birth, or after 72 h of birth. Although early-onset sepsis is also frequently seen in preterm infants, LOS is even more common because of the immature immune systems of preterm infants and their need for prolonged hospitalization and clinical interventions (Shane et al., 2017).

Although antibiotic treatment can reduce the morbidity and mortality of LOS, inaccurate use of antibiotics can have adverse effects (Alverdy & Krezalek, 2017). Ideally, antibiotic treatment would not be used when not needed. Since the consequences of LOS are so severe, in current clinical practice, clinicians start antibiotic therapy and order blood culture at the moment of clinical symptoms of infection. This moment is often denoted as the CRASH (Cultures, Resuscitation, and Antibiotics Started Here) moment. However, the blood-culture-based diagnosis (current 'gold standard') is time-consuming and not always accurate as it is sensitive to contamination (Klucher et al., 2021). Moreover, as the clinical symptoms are non-specific and subtle, their detection may occur in a late phase of the infection (Shane et al., 2017). These challenges raise the need for early non-culture dependent LOS diagnosis to improve neonatal outcomes in NICUs.

Machine learning models using vital signs have been investigated as a continuously available and non-invasive tool to detect evolving neonatal sepsis, showing that vital sign patterns can be important physiological markers to assist diagnosis of LOS before observed deterioration (Mithal et al., 2017; Peng et al., 2022). In particular, machine learning models with features extracted from heart rate variability (HRV, reflecting beat-to-beat changes in RR intervals) are most often used to early detect LOS in preterm infants as reviewed by Sullivan and Fairchild (2022) and Persad et al. (2021). For instance, the 'HeRo' monitoring system significantly reduced sepsis-related mortality in the NICU by showing a real-time HRV-based risk score for neonatal sepsis (Fairchild et al., 2013; Moorman et al., 2011). This score increases in the 24 h before CRASH. In addition, Leon et al. (2021) developed a machine learning model based on novel HRV features for LOS prediction, achieving an area under the receiver-operating-characteristics curve (AUC) of 0.88 during 6 h preceding CRASH. These promising results indicate the predictive value of HRV signals on LOS prediction. However, these models highly rely on the extracted HRV features based on domain knowledge, with a good model interpretability but at the cost of losing opportunities to uncover unknown but potentially essential information from HRV. Therefore, to better take advantage of all aspects of HRV, an end-to-end approach with intelligent and automated feature extraction is the next step for neonatal LOS prediction. Deep learning is such an approach that has developed fast in terms of performance in a variety of domains such as computer vision, text analytics, and healthcare. It can reveal complex structures in high-dimensional data, playing a significant role in building intelligent and data-driven models for a wide range of applications (Sarker, 2021). With HRV data, often expressed as RR interval (RRI) signals, deep learning models have been successfully applied to detect diseases or biological status such as apnea (Wang et al., 2019), atrial fibrillation (Faust et al., 2020) and (neonatal) sleep states (Werth et al., 2020). However, there are limited studies focusing on LOS prediction using HRV-based deep learning models.

In this study, we aim to predict upcoming LOS in preterm infants using a deep learning model developed based on RRI signals. To the best of our knowledge, this is the first study developing an end-to-end LOS prediction model solely using RRI signals for preterm infants. For this study, we firstly built a deep residual convolutional neural network (ResNet) as the baseline model. Then, we added channel attention on top of ResNet to form DeepLOS. Lastly, we compared the performance of DeepLOS to ResNet for LOS prediction.

2. Datasets

The full dataset used in the present study comprised data from 128 preterm infants born before 32 weeks of gestation and hospitalized in the NICU of the Máxima Medical Center in Veldhoven, the Netherlands, from July 2016 to December 2018. The medical ethical committee of the Máxima Medical Center provided a waiver for this retrospective study in accordance with the Dutch law on medical research with humans.

The LOS patients and their corresponding CRASH moments were defined by a group of neonatologists according to the Vermont Oxford Criteria and C-reactive protein level (Vermont Oxford Network, 2007), Hofer et al. (2012). The LOS patients had clinical symptoms of LOS and culture-proven infection. The control patients had no clinical suspicion and no need to take any blood culture. As RRI signals can change with maturation in preterm infants (Leon et al., 2022), to minimize this impact, we first defined the CRASH moment for each LOS patient and then searched for one or more control patients with a GA within 3 days younger or older than the LOS patient (Peng et al., 2022). Subsequently, we calculated an 'equivalent CRASH' moment for those control patients to determine the 24 h to analyze, based on their postmenstrual age (PMA) close to the LOS patient. This procedure resulted in 60 LOS patients (LOS group) and 68 control patients (control group) with pre-defined CRASH or equivalent CRASH moments, composing the full dataset in this study (Peng et al., 2022). However, even if using this procedure, there was a slightly lower GA for the LOS group and therefore we defined another 'matched' dataset as described in a previous study (Cabrera-Quiros et al., 2021), in which a 'one-to-one' matching of GA and PMA of LOS patients with control patients was performed, resulting in 32 patients in both groups. The matched dataset allowed us to investigate the 'pure' impact of LOS on HRV of preterm infants. Table 1 summarizes the characteristics of the studied patients in the full dataset and the matched dataset. It can be seen that there is a significant difference in maturation between the two groups in the full dataset, but no difference in the matched dataset. We performed all the following deep learning experiments using both datasets separately.

The ECG of all patients was obtained from patient monitors (Philips IntelliVue MX 800, Philips, Hamburg, Germany), with a sampling rate of 250 Hz. The ECG data of 24 h before CRASH or equivalent CRASH moments were used in this study.

3. Methodology

The schematic diagram we proposed for LOS prediction is shown in Fig. 1.

Table 1
Characteristics of the patient population in full and matched datasets—presented as median (interquartile range). The Mann–Whitney U test was used to examine the significance of difference between the two groups. NS: Not Significant ($p > 0.05$).

	Full dataset			Matched dataset		
	LOS group ($n = 60$)	Control group ($n = 68$)		LOS group ($n = 32$)	Control group ($n = 32$)	
Gestational age (weeks) (GA)	27.86 (26.86–29.29)	29.43 (28.71–30.71)	$p < 0.01$	28.71 (27.57–29.36)	28.86 (27.93–29.57)	NS
Postnatal age (days) (PNA)	6.95 (5.04–10.05)	5.17 (3.61–6.77)	$p < 0.01$	5.98 (4.77–9.39)	5.27 (4.46–7.50)	NS
Postmenstrual age (weeks) (PMA)	29.07 (27.96–30.26)	30.12 (29.60–31.57)	$p < 0.01$	35.49 (33.73–37.90)	34.60 (32.90–36.00)	NS
Birth weight (g) (BW)	1075 (870–1285)	1268 (1075–1409)	$p < 0.01$	1150 (895–1320)	1105 (965–1318)	NS

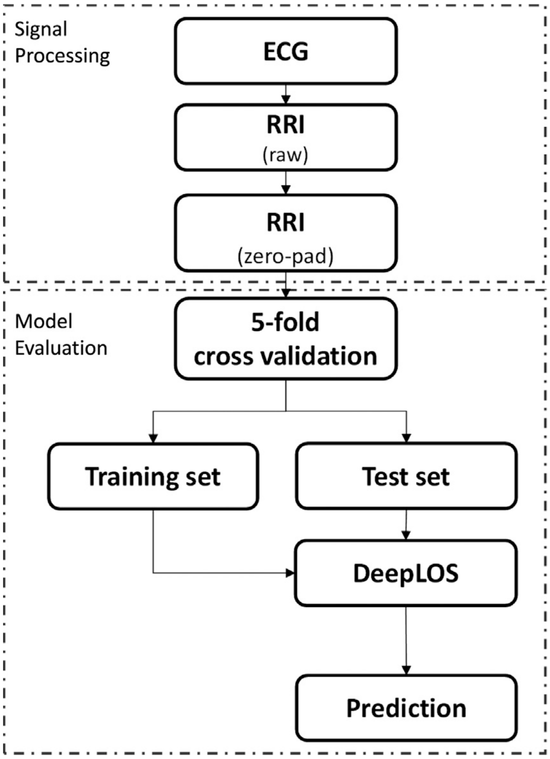

Fig. 1. The schematic diagram for the LOS prediction model. ECG—electrocardiogram, RRI—R-R interval.

3.1. Signal preprocessing and labeling

We first applied a peak detection algorithm to detect R-peaks in ECG waveforms of all patients in 24 h before CRASH or equivalent CRASH (Rooijakkers et al., 2012), followed by the extraction of RRI signals (RRI raw). Each RRI signal (per patient) was divided into non-overlapping one-hour segments. The segments prior to CRASH in the LOS group were labeled as positive samples (labeled as 1). The segments in the control group were labeled as negative samples (labeled as 0). Afterwards, the RRI samples (time points) in each segment were centered, regardless of their original length, within the segment length of 12,000 samples for one hour by filling zeros on two ends for the following model training and evaluation (RRI zero-pad).

3.2. DeepLOS

The neural network architecture of DeepLOS is shown in Fig. 2. It consists of a ResNet and an attention layer with skip connection (ResAtte), ending up with fully connected layers (FC). There are two types of residual blocks (ResConv and ResSkip) in ResNet. The details of each block (ResConv, ResSkip, ResAtte, and FC) are shown in Fig. 3. In ResNet, the convolution-based blocks gradually reduce the size of the temporal domain from 12,000 to 750 and increase the number of feature maps (channels) from 1 to 1024 at the same time to encode information. The ResNet is used to allow training deeper neural networks with less complexity, preventing vanishing gradient problems (He et al., 2016). In each convolutional layer, the neighboring information along the temporal axis is aggregated. Thus, the shallower convolutional layers focus on shorter time scales (seconds) of RRI signals, and the deeper convolutional layers can encode RRI information with larger time scales (minutes). After several convolutional operations

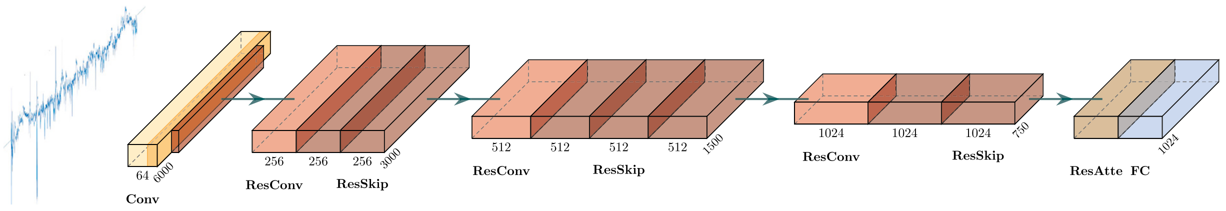

Fig. 2. The neural network architecture in DeepLOS consists of ResNet and attention. The values represent the size of temporal and channel dimensions accordingly.

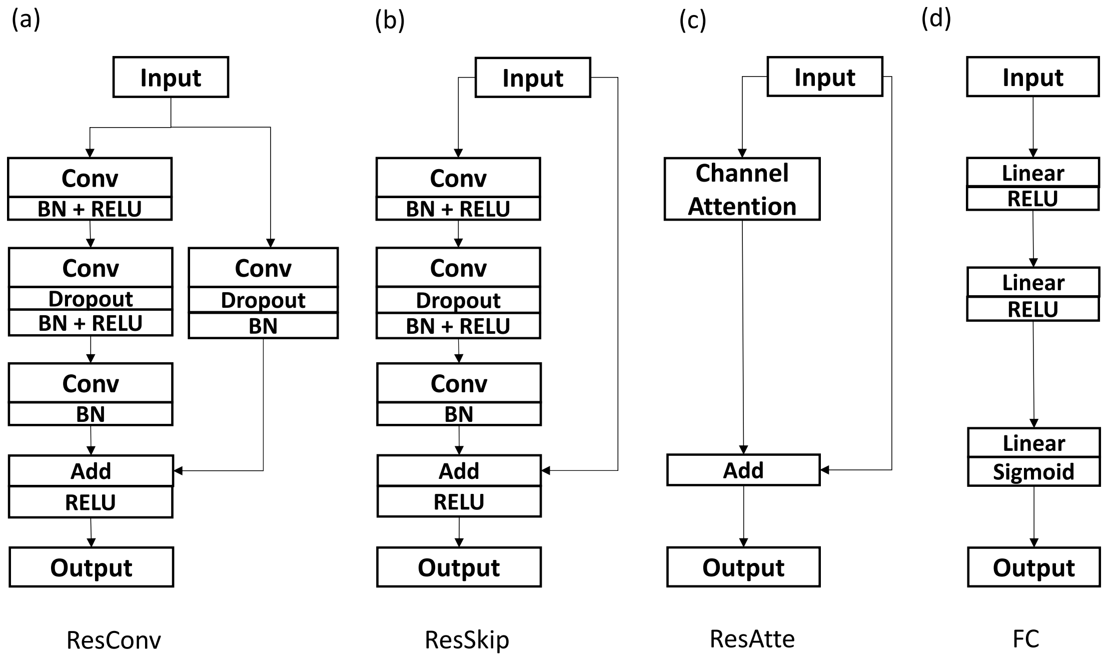

Fig. 3. The building blocks in DeepLOS. (a) The building block of the residual convolutional layer. (b) The building block of residual skip layer. (c) The building block of residual attention layer. (d) The building block of the fully connected layer. Conv—convolution, BN—batch normalization, RELU—rectified linear activation function, ResConv—residual convolutional, ResSkip—residual skip, ResAtte—residual attention, FC—fully connected.

in ResNet, the RRI patterns are aggregated into feature maps (channels) where the "self-attention" layer is applied. Specifically, this self-attention is the scaled dot-product attention proposed in Vaswani et al. (2017). We computed the dot products of the feature maps with their transpose, followed by applying a softmax function to obtain the weights on each feature map (channel). Afterwards, the output of the self-attention is obtained by multiplying these weights with the corresponding feature maps. The attention mechanism is designed to highlight the important RRI patterns associated with LOS since it allows the neural network to learn long-term dependencies (Vaswani et al., 2017), Lin et al. (2017). Finally, the encoded information from attention is fed into the fully connected layers to obtain the probability of LOS for each segment. We set dropout rate of 0.1 for convolutional layers and 0.5 for fully connected layers. The model was optimized using an Adam optimizer with a learning rate of 0.0001. Binary cross entropy was used as the loss function.

3.3. *Prediction evaluation and comparison*

To evaluate the prediction performance of DeepLOS using RRI signals, we applied a 5-fold patient-independent cross validation for training and testing models. Each fold included 25–26 patients for the full dataset or 12–13 patients for the matched dataset. Firstly, the training set (4 folds of data) was further split into inner training (75%) and validation (25%) set. The model was updated for 300 epochs, the hyperparameters and the optimized epoch were determined based on the performance in validation set to avoid overfitting. Then, the model retrained on all training set (4 folds of data) with optimized parameters and epoch was tested on the remaining fold of data during each iteration of the cross validation. Because a previous study showed that the RRI of preterm infants can change 24 h before CRASH [11], we used F-score during 24 h before LOS to optimize parameters during training. Mean

Table 2
Metrics of prediction in both datasets during 24 hours before crash, metrics are presented as mean (SD).

	Model	Sensitivity	Specificity	Positive Predictive Value (PPV)	Negative Predictive Value (NPV)	Accuracy	AUC	F-score
Full dataset (n = 128)	DeepLOS	0.79 (0.07)	0.63 (0.23)	0.68 (0.12)	0.78 (0.02)	0.71 (0.09)	0.77 (0.06)	0.72 (0.06)
	Baseline	0.91 (0.07)	0.22 (0.17)	0.51 (0.04)	0.78 (0.04)	0.55 (0.05)	0.67 (0.02)	0.65 (0.03)
Matched dataset (n = 64)	DeepLOS	0.91 (0.07)	0.43 (0.21)	0.63 (0.11)	0.84 (0.06)	0.66 (0.09)	0.72 (0.05)	0.73 (0.05)
	Baseline	0.93 (0.12)	0.16 (0.26)	0.54 (0.04)	0.74 (0.05)	0.56 (0.06)	0.64 (0.03)	0.68 (0.02)

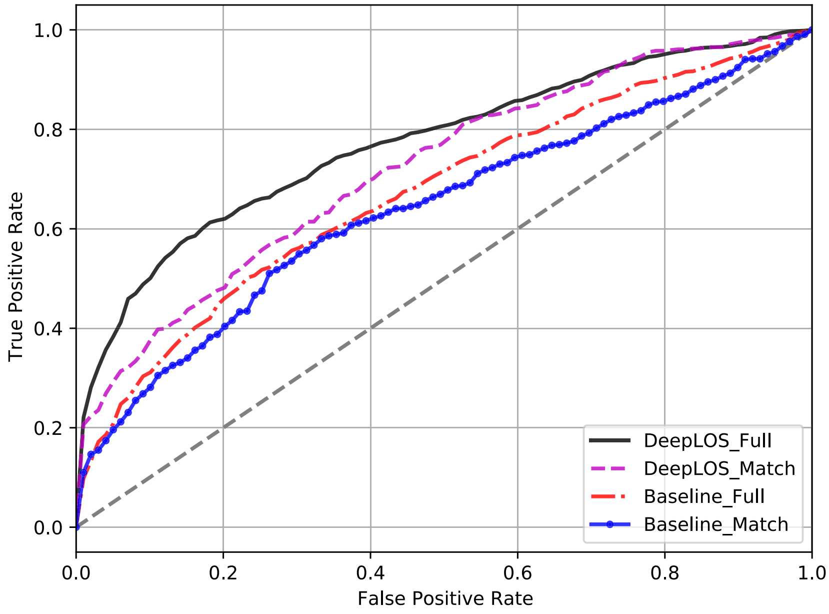

Fig. 4. Receiver operating characteristic (ROC) curve for LOS prediction during 24 h before CRASH using DeepLOS and Baseline in the full dataset and matched dataset.

and standard deviation (SD) of F-score for all one-hour segments (totally 24 h) over folds were used to evaluate the prediction performance.

To evaluate the contribution of channel attention for RRI-based LOS prediction, we also implemented the evaluation scheme to a baseline model using only ResNet. More specifically, the RRI signal of each one-hour segment was fed into ResNet, directly followed by fully connected layers to train the model and obtain the LOS prediction probability for each segment.

In addition to F-score, we calculated other metrics including sensitivity, specificity, positive predictive value (PPV), negative predictive value (NPV), overall accuracy, and AUC. Furthermore, we also used the receiver operating characteristic (ROC) curve for all hours to compare overall LOS prediction performance and used F-score per hour to compare performance over time for the ResNet (Baseline) and DeepLOS.

4. Results

Table 2 presents the prediction performance metrics of the two models (Baseline and DeepLOS) for LOS prediction for all 24 h preceding CRASH in the full dataset and the matched dataset. Using DeepLOS, we achieved a mean (SD) F-score of 0.72 (0.06) on the full dataset and 0.73 (0.05) on the matched dataset, performing clearly better than the Baseline with only ResNet used. This can also be evidenced by comparing the results of almost all other metrics. The ROC curves shown in Fig. 4 indicate the superiority of DeepLOS over Baseline in the entire solution space when using both full and matched datasets. The model performance in the two datasets differs on different metrics. The AUC of DeepLOS in full dataset is higher than that in matched dataset, although the F-score is similar.

To further compare the prediction performance of both models, Fig. 5 illustrates the F-score changes over time (on one-hour basis). It can be observed that DeepLOS outperforms Baseline for almost all the 24 h before CRASH. The performance of DeepLOS increases over time when coming closer to CRASH in both datasets. The performance of DeepLOS in the matched dataset is similar to that in the full dataset.

To monitor the performance of the best-performing model (DeepLOS) that learn incrementally over time (epoch), we plotted the averaged learning curve of DeepLOS in both datasets as shown in Fig. 6. The selected epoch numbers in full dataset and matched

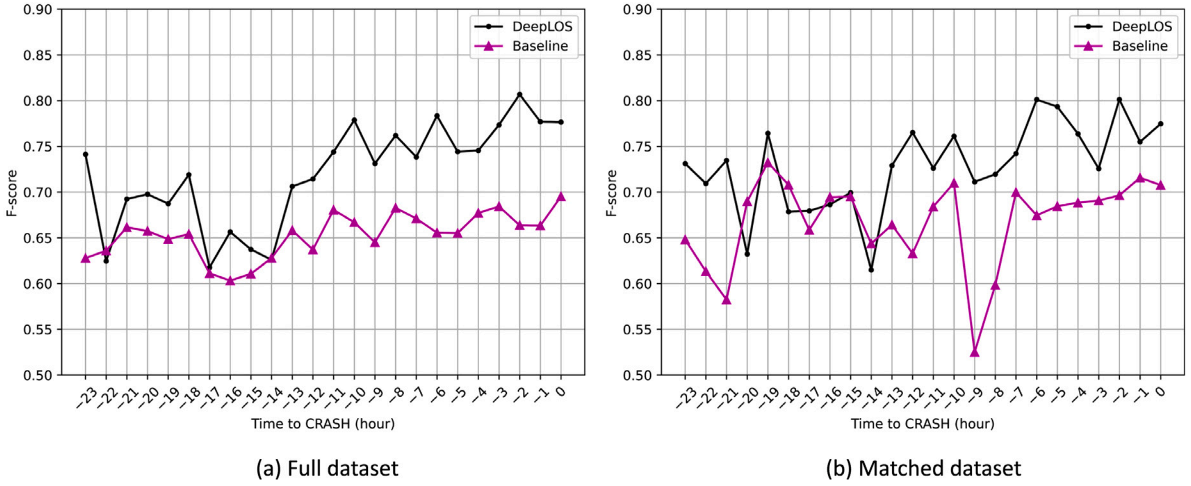

Fig. 5. Prediction performance (F-score) of DeepLOS and Baseline over time before CRASH. (a) In the full dataset. (b) In matched dataset.

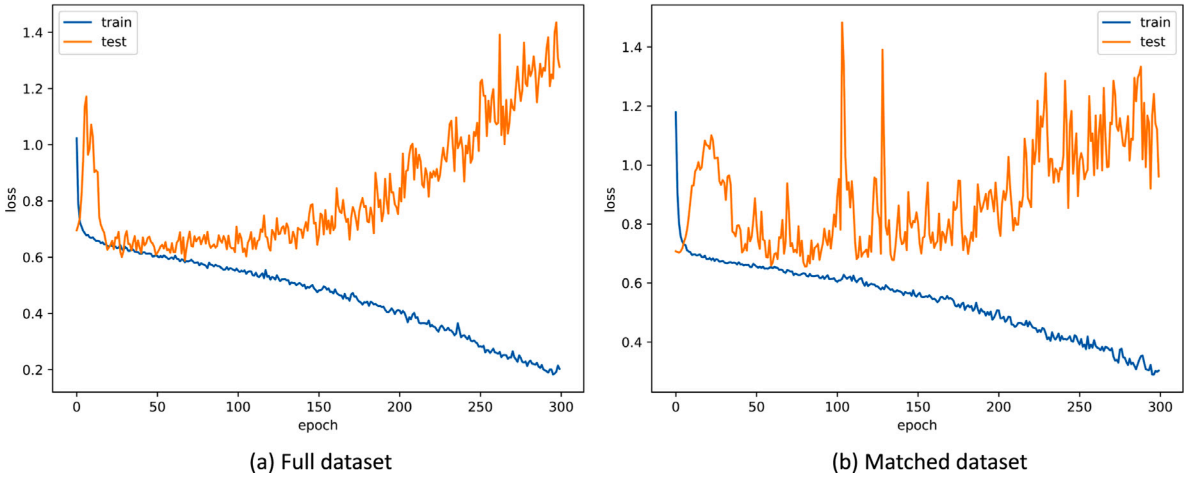

Fig. 6. Learning curve of DeepLOS for LOS prediction during 24 h before CRASH. (a) In the full dataset. (b) In matched dataset.

dataset are within range of 35–67 and 45–97 respectively. It can be seen that the test performance in full dataset is more stable than that in matched dataset. After the epoch of around 80 the level of overfitting keep increasing over epochs.

5. Discussion

The goal of this study is to demonstrate that the upcoming LOS in preterm infants can be predicted several hours before observed deterioration using an end-to-end deep learning model (DeepLOS) on RRI signals. The results of this study also indicate that the use of channel attention can largely improve the prediction performance for LOS in preterm infants.

In addition, we observed that the model (DeepLOS) performed better (higher AUC with similar F-score) in the full dataset than in the matched model. This may be caused by the difference in GA and PMA happening in the full dataset between the LOS and the control group, where the HRV of preterm infants is proven to be associated with the maturation level as measured by age (Patural et al., 2019). However, it is also known that in general LOS patients are younger than control patients because of the inherent higher LOS risk in very preterm infants (Wynn et al., 2010). Besides, although maturation per se is an important risk factor in clinical practice and is associated with LOS in preterm infants (Wynn et al., 2010), the model including maturation based on a small dataset (full dataset) would be difficult to generalize and the performance can be 'overestimated' due to the maturation difference between the two groups. However, the matched dataset we designed might 'underestimate' the performance of the model because the inherent maturation difference was not considered. Therefore, a bigger dataset including more patients with a larger range of maturation should be further investigated to accommodate the use of maturation for a more generalizable model. Interestingly, by comparing the two figures in Fig. 5, it can be observed that the performance of all models in the two datasets stays high and stable from 6 h before CRASH. This may indicate the time moment of the deterioration for LOS patients.

We compared the LOS prediction performance using DeepLOS proposed in this work with several other studies using RRI signals. For instance, Leon et al. (2021) used principal component analysis of HRV features and a logistic regression classifier, obtaining an AUC of 0.88 during 6 h before CRASH. Griffin et al. (2007) achieved an AUC of 0.67 using logistic regression based on HRV features to predict LOS within the next 24 h. Our model outperformed the feature-based model proposed by Griffin et al. (2007) within the similar evaluation metric. To compare with the results by Leon et al. (2021), we also computed the AUC during 6 h before CRASH using DeepLOS and we obtained a lower AUC of 0.85. It is worth mentioning that, comparing results between studies using different datasets is always difficult mainly because of the heterogeneity in the study population, different clinical definitions, and varying evaluation metrics (Persad et al., 2021; Verstraete et al., 2015). Considering this, we also compared with a recent proposed feature-based algorithm (extreme gradient boosting) using the same datasets (Peng et al., 2022). That study analyzed multiple signal modalities including HRV, respiration, and movement for LOS prediction. For a fair comparison, we re-generated the results using only HRV data and obtained an F-score of 0.65 and an AUC of 0.65 for all 24 h of data, which are much lower than those achieved by DeepLOS. Interestingly, combining multiple signal modalities could lead to large performance improvement in neonatal LOS prediction using the feature-based model. This encourages us to verify DeepLOS on multimodal data (adding other signal modalities to HRV) in future work.

This study uses HRV data solely for neonatal LOS prediction. The HRV-based prediction models can be easily deployed in different NICUs because ECG is a standard measurement in a NICU from which RRI can be extracted. The use of RRI is vendor-independent without needing other specific devices. Furthermore, HRV can also be obtained with many advanced unobtrusive monitoring technologies such as a smart mat underneath the infant measuring ballistocardiogram (Cathelain et al., 2020) and a vital sign camera measuring heart rate from the face (Chaichulee et al., 2019). Therefore, the HRV-based algorithm presented in this study can be potentially applied when using an unobtrusive or non-contact measurement device.

One main limitation of this study is that all 24 one-hour segments before CRASH in LOS patients were labeled as positive and fed into the model while some infants may be not infected or the deterioration has not started yet when being far away from CRASH. From the results in the matched dataset, we can see that the deterioration of LOS patients is more predictable when the time was closer to CRASH. Ideally, we would like to predict LOS as early as possible, and in terms of the modeling performance, the positive labels should only include the RRI segments directly around the exact time moment of the deterioration. However, it is impossible to determine exactly when the deterioration starts for each individual. One possible direction could be to take the time window length (number of hours) included in the training set as a hyperparameter to optimize, 'statistically' finding the general time window length for the whole population (Peng et al., 2022). However, the reduced window length also leads to reduced training data size, which can lead to poor performance of deep learning models (Lecun et al., 2015). Besides, the dataset size in this study is limited and the dataset is from a single center. As we can observe in the learning curve (Fig. 6), the generalization error (gas between training and test loss) significantly increases over training after 80 epochs. This may be because the model has more capacity than is required for this application and in turn because of the limited size of the dataset. One option to compensate for this limitation is dataset augmentation, where the new synthetic data is created based on existing data of known labels so that the synthetic data can come from the same generation distribution as existing data (DeVries & Taylor, 2019). This needs to be carefully designed and could be interesting for future research. Also, validation using external datasets is a useful way to enlarge available data. However, this is always challenging due to the different standardization of clinical definitions (Fleuren et al., 2020), Persad et al. (2021) and data. For instance, Das et al. defined sepsis based on culture-proven of blood, urine, or cerebrospinal fluid culture after 7 days of life (Das et al., 2016). In the study of Leon et al. sepsis was defined as antibiotics treatment longer than 4 days after 3 days of life at onset (Leon et al., 2021). The data standardization is also challenging because not all the data are stored with a high temporal resolution, and the collecting sensors in different centers are also not standardized. Although our raw RRI-based model is more vendor-neutral than multimodal feature-based models, cross-center validation is still needed to investigate the generalizability of the model. In future work, the aim is to explore more data (both internal and external) for further model evaluation and to carefully design data augmentation to compensate for the limitation of dataset size (DeVries & Taylor, 2019).

6. Conclusion

In this study, we demonstrate the potential of an end-to-end model (DeepLOS) to predict LOS solely based on the RRI signal which is continuously available through routine patient monitoring in NICUs. The better performance of DeepLOS compared to feature-based models and ResNet suggests further investigations on attention-based end-to-end solutions for LOS prediction in preterm infants.

Declaration of competing interest

The authors declare that they have no known competing financial interests or personal relationships that could have appeared to influence the work reported in this paper.

Data availability

The data that has been used is confidential.

Acknowledgments

This work was supported by the Eindhoven MedTech Innovation Center (e/MTIC), The Netherlands which was a collaboration of the Eindhoven University of Technology, Philips Research, and Máxima Medical Center.

References

Al-Matary, A., Heena, H., AlSarheed, A. S., Ouda, W., AlShahrani, D. A., Wani, T. A., Qaraqei, M., & Abu-Shaheen, A. (2019). Characteristics of neonatal sepsis at a tertiary care hospital in Saudi Arabia. *Journal of Infection and Public Health*, *12*(5), 666–672.

Alverdy, J. C., & Krezalek, M. A. (2017). Collapse of the microbiome, emergence of the pathobiome, and the immunopathology of sepsis. *Critical Care Medicine*, *45*(2), 337–347.

Cabrera-Quiros, L., Kommers, D., Wolvers, M. K., Oosterwijk, L., Arents, N., van der Sluijs-Bens, J., Cottaar, E. J. E., Andriessen, P., & van Pul, C. (2021). Prediction of late-onset sepsis in preterm infants using monitoring signals and machine learning. *Critical Care Explorations*, *3*(1), Article e0302.

Cathelain, G., Rivet, B., Achard, S., Bergounioux, J., & Jouen, F. (2020). U-net neural network for heartbeat detection in ballistocardiography. In *Proceedings of the Annual International Conference of the IEEE Engineering in Medicine and Biology Society* (pp. 465–468).

Chaichulee, S., Villarroel, M., Jorge, J., Arteta, C., McCormick, K., Zisserman, A., & Tarassenko, L. (2019). Cardio-respiratory signal extraction from video camera data for continuous non-contact vital sign monitoring using deep learning. *Physiological Measurement*, *40*(11).

Das, A., Shukla, S., Rahman, N., Gunzler, D., & Abughali, N. (2016). Clinical indicators of late-onset sepsis workup in very low-birth-weight infants in the neonatal intensive care unit. *American Journal of Perinatology*, *33*(9), 856–860.

DeVries, T., & Taylor, G. W. (2019). Dataset augmentation in feature space. In *5th International conference on learning representations* (pp. 1–12).

Fairchild, K. D., Schelonka, R. L., Kaufman, D. A., Carlo, W. A., Kattwinkel, J., Porcelli, P. J., Navarrete, C. T., Bancalari, E., Aschner, J. L., Walker, M. W., Perez, J. A., Palmer, C., Lake, D. E., O'Shea, T. M., & Moorman, J. R. (2013). Septicemia mortality reduction in neonates in a heart rate characteristics monitoring trial. *Pediatric Research*, *74*(5), 570–575.

Faust, O., Shenfield, A., Kareem, M., San, T. R., Fujita, H., & Acharya, U. R. (2020). Automated detection of atrial fibrillation using long short-term memory network with RR interval signals. *Computers in Biology and Medicine*, (102), 327–335.

Fleuren, L. M., Klausch, T. L., Zwager, C. L., Schoonmade, L. J., Guo, T., Roggeveen, L. F., Swart, E. L., Girbes, A. R., Thoral, P., Ercole, A., Hoogendoorn, M., & Elbers, P. W. (2020). Machine learning for the prediction of sepsis: a systematic review and meta-analysis of diagnostic test accuracy. *Intensive Care Medicine*, *46*(3), 383–400.

Griffin, M. P., Lake, D. E., O'Shea, T. M., & Moorman, J. R. (2007). Heart rate characteristics and clinical signs in neonatal sepsis. *Pediatric Research*, *61*(2), 222–227.

He, K., Zhang, X., Ren, S., & Sun, J. (2016). Deep residual learning for image recognition. In *Proceedings of the IEEE Conference on computer vision and pattern recognition* (pp. 770–778). IEEE.

Hofer, N., Zacharias, E., Müller, W., & Resch, B. (2012). An update on the use of C-reactive protein in early-onset neonatal sepsis: Current insights and new tasks. *Neonatology*, *102*(1), 25–36.

Klucher, J. M., Davis, K., Lakkad, M., Painter, J. T., & Dare, R. K. (2021). Risk factors and clinical outcomes associated with blood culture contamination. *Infection Control and Hospital Epidemiology*, *43*(3), 291–297.

Lecun, Y., Bengio, Y., & Hinton, G. (2015). Deep learning. *Nature*, *521*(7553), 436–444.

Leon, C., Cabon, S., Patural, H., Gascoin, G., Flamant, C., Roue, J. M., Favrais, G., Beuchee, A., Pladys, P., & Carrault, G. (2022). Evaluation of maturation in preterm infants through an ensemble machine learning algorithm using physiological signals. *IEEE Journal of Biomedical and Health Informatics*, *26*(1), 400–410.

Leon, C., Carrault, G., Pladys, P., & Beuchee, A. (2021). Early detection of late onset sepsis in premature infants using visibility graph analysis of heart rate variability. *IEEE Journal of Biomedical and Health Informatics*, *25*(4), 1006–1017.

Lin, Z., Feng, M., Santos, C. N. D., Yu, M., Xiang, B., Zhou, B., & Bengio, Y. (2017). A structured self-attentive sentence embedding. In *5th International conference on learning representations* (pp. 1–15).

Mithal, L. B., Yogev, R., Palac, H. L., Kaminsky, D., Gur, I., & Mestan, K. K. (2017). Vital signs analysis algorithm detects inflammatory response in premature infants with late onset sepsis and necrotizing enterocolitis. *Early Hum. Dev.*, *117*, 83–89.

Moorman, J. R., Carlo, W. A., Kattwinkel, J., Schelonka, R. L., Porcelli, P. J., Navarrete, C. T., Bancalari, E., Aschner, J. L., Walker, M. W., Perez, J. A., Palmer, C., Stukenborg, G. J., Lake, D. E., & O'Shea, T. M. (2011). Mortality reduction by heart rate characteristic monitoring in very low birth weight neonates: A randomized trial. *Journal of Pediatrics*, *159*(6), 900–906.

Patural, H., Pichot, V., Flori, S., Giraud, A., Franco, P., Pladys, P., Beuchée, A., Roche, F., & Barthelemy, J. C. (2019). Autonomic maturation from birth to 2 years: normative values. *Heliyon*, *5*(3), Article e01300.

Peng, Z., Varisco, G., Long, X., Liang, R.-h., Kommers, D., Cottaar, W., & Van Pul, C. (2022). A continuous late-onset sepsis prediction algorithm for preterm infants using multimodal physiological signals from a patient monitor. TechRxiv. Preprint.

Persad, E., Jost, K., Honoré, A., Forsberg, D., Coste, K., Olsson, H., Rautiainen, S., & Herlenius, E. (2021). Neonatal sepsis prediction through clinical decision support algorithms: A systematic review. *Acta Paediatrica, International Journal of Paediatrics*, *110*(12), 3201–3226.

Phibbs, C. S., Passarella, M., Schmitt, S. K., Rogowski, J. A., & Lorch, S. A. (2022). Understanding the relative contributions of prematurity and congenital anomalies to neonatal mortality. *Journal of Perinatology*, *42*(5), 569–573.

Rooijakkers, M. J., Rabotti, C., Oei, S. G., & Mischi, M. (2012). Low-complexity R-peak detection for ambulatory fetal monitoring. *Physiological Measurement*, *33*(7), 1135–1150.

Sarker, I. H. (2021). A comprehensive overview on techniques, taxonomy, applications and research directions. *SN Computer Science*, *2*(420).

Shane, A. L., Sánchez, P. J., & Stoll, B. J. (2017). Neonatal sepsis. *The Lancet*, *390*(10104), 1770–1780.

Sullivan, B. A., & Fairchild, K. D. (2022). Vital signs as physiomarkers of neonatal sepsis. *Pediatric Research*, *91*(2), 273–282.

Vaswani, A., Shazeer, N., Parmar, N., Uszkoreit, J., Jones, L., Gomez, A. N., Kaiser, Ł., & Polosukhin, I. (2017). Attention is all you need. In *Advances in neural information processing systems, vol. 30*. Curran Associates, Inc..

Vermont Oxford Network (2007). *Manual of operations: Part 2. data definitions & infant data forms* (Release 22.0).

Verstraete, E. H., Blot, K., Mahieu, L., Vogelaers, D., & Blot, S. (2015). Prediction models for neonatal health care-associated sepsis: A meta-analysis. *Pediatrics*, *135*(4), 1002–1014.

Wang, L., Lin, Y., & Wang, J. (2019). A RR interval based automated apnea detection approach using residual network. *Computer Methods and Programs in Biomedicine*, (176), 93–104.

Werth, J., Radha, M., Andriessen, P., Aarts, R. M., & Long, X. (2020). Deep learning approach for ECG-based automatic sleep state classification in preterm infants. *Biomedical Signal Processing and Control*, *56*(101663).

Wynn, J., Cornell, T. T., Wong, H. R., Shanley, T. P., & Wheeler, D. S. (2010). The host response to sepsis and developmental impact. *Pediatrics*, *125*(5), 1031–1041.
