# Supplementary material for: Exploring Computational Techniques in Preprocessing Neonatal Physiological Signals for Detecting Adverse Outcomes: Scoping Review
Source: Interact J Med Res. 2024 Aug 20;13:e46946. doi: 10.2196/46946 (PMC11372324; doi:10.2196/46946)
Supplement: Multimedia Appendix 3 [file ijmr_v13i1e46946_app3.zip › Included Papers - Final/4376/Varisco et al. - 2022 - Central apnea detection in premature infants using.pdf]

Computer Methods and Programs in Biomedicine 226 (2022) 107155

Contents lists available at ScienceDirect

Computer Methods and Programs in Biomedicine

journal homepage: www.elsevier.com/locate/cmpb

Central apnea detection in premature infants using machine learning

Gabriele Varisco[a,b,*], Zheng Peng[a,b], Deedee Kommers[a,c], Zhuozhao Zhan[d], Ward Cottaar[a], Peter Andriessen[a,c], Xi Long[e,f,*], Carola van Pul[a,b]

[a] Applied Physics, Eindhoven University of Technology, Eindhoven, the Netherlands
[b] Clinical Physics, Máxima Medical Center, Veldhoven, the Netherlands
[c] Pediatrics, Máxima Medical Center, Veldhoven, the Netherlands
[d] Mathematics and Computer Science, Eindhoven University of Technology, Eindhoven, the Netherlands
[e] Philips Research, Eindhoven, the Netherlands
[f] Electrical Engineering, Eindhoven University of Technology, Eindhoven, the Netherlands

ARTICLE INFO

Article history:
Received 1 April 2022
Revised 13 September 2022
Accepted 23 September 2022

Keywords:
Apnea of prematurity
Central apnea
Late-onset sepsis
Machine Learning

ABSTRACT

Background and objective: Apnea of prematurity is one of the most common diagnosis in neonatal intensive care units. Apneas can be classified as central, obstructive or mixed. According to the current international standards, minimal fluctuations or absence of fluctuations in the chest impedance (CI) suggest a central apnea (CA). However, automatic detection of reduced CI fluctuations leads to a high number of central apnea-*suspected* events (CASEs), the majority being false alarms. We aim to improve automatic detection of CAs by using machine learning to optimize detection of CAs among CASEs.
Methods: Using an optimized algorithm for automated detection, all CASEs were detected in a population of 10 premature infants developing late-onset sepsis and 10 age-matched control patients. CASEs were inspected by two clinical experts and annotated as CAs or rejections in two rounds of annotations. A total of 47 features were extracted from the ECG, CI and oxygen saturation signals considering four 30 s-long moving windows, from 30 s before to 15 s after the onset of each CASE, using a moving step size of 5 s. Consecutively, new CA detection models were developed based on logistic regression with elastic net penalty, random forest and support vector machines. Performance was evaluated using both leave-one-patient-out and 10-fold cross-validation considering the mean area under the receiver-operating-characteristic curve (AUROC).
Results: The CA detection model based on logistic regression with elastic net penalty returned the highest mean AUROC when features extracted from all four time windows were included, both using leave-one-patient-out and 10-fold cross-validation (mean AUROC of 0.88 and 0.90, respectively). Feature relevance was found to be the highest for features derived from the CI. A threshold for the false positive rate in the mean receiver-operating-characteristic curve equal to 0.3 led to a high percentage of correct detections for all CAs (78.2%) and even higher for CAs followed by a bradycardia (93.4%) and CAs followed by both a bradycardia and a desaturation (95.2%), which are more critical for the well-being of premature infants.
Conclusions: Models based on machine learning can lead to improved CA detection with fewer false alarms.

© 2022 The Author(s). Published by Elsevier B.V.
This is an open access article under the CC BY license (http://creativecommons.org/licenses/by/4.0/)

1. Introduction

Apnea of prematurity is a condition that is commonly found in premature infants admitted to neonatal intensive care units (NICUs) and is related to the immaturity of their respiratory control system [1]. Patients who develop severe illnesses such as sepsis or necrotizing enterocolitis are particularly prone to recurrent apneas [2–4]. Furthermore, apnea of prematurity is associated with later neurodevelopmental impairment and brain damage [5,6]. Ac-

Abbreviations: NICU, neonatal intensive care unit; CA, central apnea; HR, heart rate; SpO_2, oxygen saturation; CI, chest impedance; CASE, central apnea-*suspected* event; LOS, late-onset sepsis; CRASH-moment, blood Culture collection Resuscitation and Antibiotics Started Here; SII, signal instability index; RRE, ribcage respiratory effort; CRC, cardiorespiratory coupling signal; LR, logistic regression with the elastic net penalty; RF, random forest; SVM, support vector machines; LOPO CV, leave-one-patient-out cross-validation; AUROC, area under the receiver operating characteristic curve; 10-fold CV, 10-fold cross-validation.

* Corresponding authors at: Applied Physics and Electrical Engineering, Eindhoven University of Technology, Eindhoven, the Netherlands.
E-mail addresses: g.varisco@tue.nl (G. Varisco), xi.long@philips.com (X. Long).

https://doi.org/10.1016/j.cmpb.2022.107155
0169-2607/© 2022 The Author(s). Published by Elsevier B.V. This is an open access article under the CC BY license (http://creativecommons.org/licenses/by/4.0/)

curate and timely apnea detection is therefore clinically important to improve outcomes in premature infants.

Apnea of prematurity is most often defined as a cessation of breathing lasting more than 20 s *or* more than 10 s when followed by a bradycardia, desaturation, or both [7,8]. However, some clinical experts disagree on the second part of the definition as they consider it necessary to have at least 15 s of cessation of breathing to define an apnea, even when followed by bradycardia or desaturation [9–11].

Apneas can be categorized as central apnea (CA), characterized by a cessation of respiratory drive with a lack of diaphragmatic activity, obstructive apnea, characterized by an airway obstruction which causes an absence of airflow, and mixed apnea, which includes characteristics of both central and obstructive apnea [1,12–14]. The heart rate (HR) and oxygen saturation (SpO_2) responses vary depending both on the different apnea types as well as their duration [11]. For instance, it was noticed that a fall in HR to less than 100 beats/min is typically found in the case of CAs whereas an initial acceleration in the HR is more often present during obstructive apneas [11]. At the same time, further decreases in SpO_2 were noticed in longer apneas, irrespectively of their type [11,15].

Premature infants are constantly monitored and an alarm is generated by the patient monitor in the event of an apnea. In current clinical practice, patient monitors generate an apnea alarm that is based on temporary reduction in amplitude of the chest impedance (CI), since this reflects a reduction in chest motion and thus indirectly in breathing motion [1,13]. This approach leads to the identification of the so-called central apnea-*suspected* events (CASEs), which may reflect apneic events of central origin. In fact, while among CASEs there are true CAs, several CASEs are also known to be false alarms. Studies have shown the presence of a high number of false apnea associated alarms in the daily NICU practice [9,16–19], indicating that the current clinical apnea detection is deficient.

In the past years, different strategies have been developed with the aim to improve apnea detection, encompassing a variety of signal processing techniques as well as machine learning and deep learning techniques [17,20–25]. Among the different proposed solutions, the algorithm proposed by Lee et al. focuses specifically on CA detection [17–19]. In a previous study, we optimized this algorithm considering annotations from two clinical experts [26]. We observed a very high recall (sensitivity or true positive rate (TPR)) for CAs, but a persistingly low precision (or positive predictive value (PPV)). This indicated that many false CAs were still detected. In order to obtain both a high precision and a high recall, the aim of the current study was to develop an optimized detection model based on machine learning to automatically detect true CAs from all CASEs extracted using the optimized algorithm for CA detection [26].

2. Methods

2.1. Dataset

We used routinely obtained data from premature infants that participated in a previous matched case-control study on sepsis [27]. These were all patients admitted to the NICU of Máxima Medical Center in Veldhoven, the Netherlands, from July 2016 to December 2018. From that existing database we selected premature infants most likely to experience apneas (i.e., all very preterm infants with a gestational age $\leq$ 30 weeks) that developed late-onset sepsis (LOS), and their case-matched controls.

LOS was diagnosed when patients presented the clinical signs of a generalized infection according to the Vermont Oxford Criteria [28], required the use of antibiotics after at least 72 h of life, and pathogens were isolated from their blood culture. For these patients we identified the CRASH-moment (blood Culture collection, Resuscitation, and Antibiotics Started Here), following the definition originally provided by Griffin et and Moorman [29]. We then collected all the data for all 48 h prior to the onset of sepsis, in which apneas are very likely to occur [3,26,30]. For the case-matched controls we calculated an equivalent-in-time CRASH-moment to make sure that the postmenstrual age was matched and we extracted the data from the 48 h prior to the equivalent CRASH-moment. A total of 960 h of data from 20 premature infants (10 LOS patients and 10 matched controls) were included in this study. All patients' characteristics, specified for the LOS and control groups, are presented in Table 1.

Each patient was monitored using Philips IntelliVue MX800 patients' monitors (Philips Medical Systems, Böblingen, Germany) according to the present clinical standard and all data was stored in a data warehouse (PIIC-iX, Data Warehouse Connect; Philips Medical Systems, Andover, MA). The ECG and CI were measured using three ECG leads with a sampling frequency of 250 Hz for the ECG and 62.5 Hz for the CI. The SpO_2 was measured with a photoplethysmogram with a sampling frequency of 1 Hz. All these three signals were then extracted from the data warehouse for further processing.

As this study had a retrospective and non-invasive nature, a waiver was provided by the medical ethical committee in accordance with the Dutch law on medical research with humans (WMO).

2.2. Annotation process

As a first step in this study, we applied the optimized algorithm for CA detection described in our previous paper [26] on our dataset of 20 premature infants. This algorithm was derived from an algorithm originally described by Lee et al. [17,19,18], which allows to filter out the cardiac artifacts found in the ECG from the CI, to compute a CA probability function from the resulting filtered respiration signal and finally to detect CASEs, which include both CAs and false CAs. We previously optimized this algorithm to improve its precision without affecting its high recall [26].

In the current study, the optimized algorithm for CA detection was applied to the patients' signals to detect CASEs longer than 10 s, in a similar way to the previous use of this algorithm [26]. This first step is indicated in Fig. 1, which also includes an overview of all subsequent steps performed in this study. Matlab (The MathWorks, Natick, United States) was used to detect CASEs from our dataset, as well as to perform the annotation process and the signal processing and feature extraction techniques.

A total of 7818 CASEs were extracted from the 20 premature infants. Two clinical experts were asked to provide, independently from each other, annotations for all the extracted CASEs using a user interface developed for this task. Three different options were given for the annotations: 'central apnea' (used for CASEs that clearly presented the characteristics of a CA as pre-defined by the clinical experts, i.e., a trace of flat CI following a previously regular fluctuating CI), 'rejection' (used when a CASE did not present the typical characteristics of a CA, i.e., in case of a flat CI trace mixed with large CI fluctuations), and 'error' (used in case of missing or corrupt signal).

A second (final) round of annotations was performed with the two clinical experts present at the same time to find consensus for all CASEs that presented different annotations after the first round.

2.3. Time windows for CASE detection

We observed a high number of clusters of CASEs, in which CASEs were present at a brief distance from other CASEs. In particular, 2172 CASEs were found to be preceded by another CASE

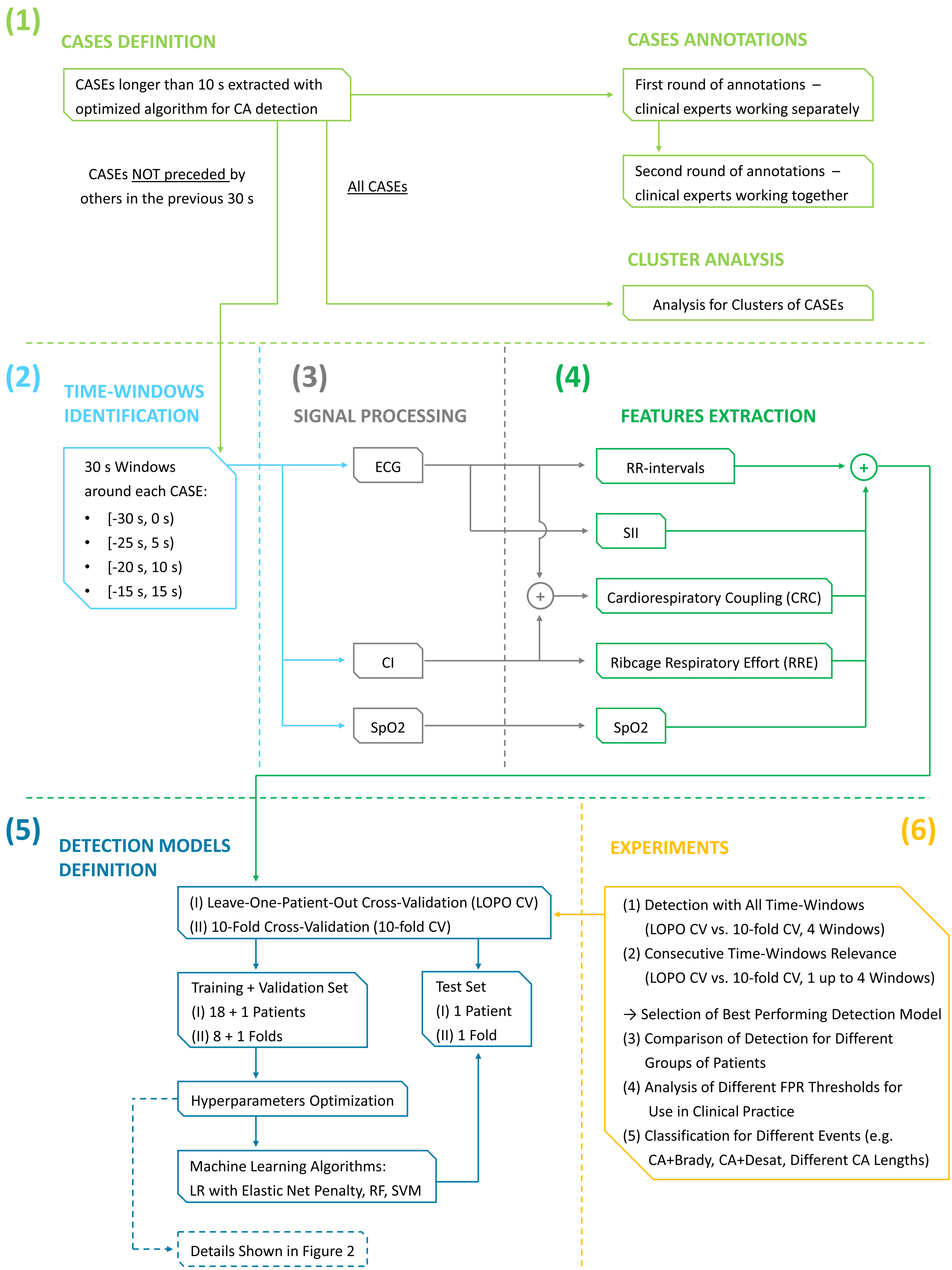

Fig. 1. Overview of the steps performed in this study starting from the extraction of central apnea-*suspected* events (CASEs) and their annotation by clinical experts (1). Next, CASEs cluster analysis was performed and for each CASE not preceded by another CASE in the previous 30 s, four 30 s windows with 5 s overlap ([-30 s, 0 s), [-25 s, 5 s), [-20 s, 10 s) and [-15 s, 15 s)) were identified (2). Signals extracted from the patients were then processed to create five new signals (3). From all these new signals 47 features were extracted (4). Two different cross-validation methods, leave-one-patient-out cross-calidation (LOPO CV (I)) and a 10-fold cross-validation (10-fold CV (II)) and three different machine learning algorithms, logistic regression with elastic net penalty (LR), random forest (RF) and support vector machines (SVM), were defined to create detection models at step (5). Finally, five different machine learning experiments were defined and performed (6).

Table 1
Characteristics of the patients included in the current study, separated considering the Late-Onset Sepsis (LOS) and control groups and all patients together.

Dataset	LOS	Control	All
Count of Patients	10	10	20
Gestational Age (wk) (mean ± std)	27.9 ± 0.9	27.9 ± 1.0	27.9 ± 0.9
Postnatal Days (mean ± std)	10 ± 8	10 ± 9	10 ± 8
Postmenstrual Age (wk) (mean ± std)	29.3 ± 0.9	29.3 ± 0.9	29.3 ± 0.9
Birth Weight (g) (mean ± std)	998 ± 216	1091 ± 156	1044 ± 190
Gender (Female)	50%	50%	50%
Hours per Patient	48	48	48
Count of CASEs (median [IQR])	351.5 [284.3 – 390.5]	231.5 [156.5 – 300.5]	284.5 [187.5 – 367]
Count of CAs (median [IQR])	47.5 [18.7 – 76.5]	17 [4.2 – 44]	26.5 [9.5 – 62.2]

in the previous 30 s (27.8% of the total count of CASEs). We therefore performed a cluster analysis to investigate whether patterns in the transitions for the annotations from the clinical experts were present. We investigated the likelihood of having the same annotation in subsequent CASEs, information that could possibly be relevant in future detection or prediction studies.

Only CASEs which were not preceded by another CASE in the previous 30 s were included to perform machine learning experiments. This clinical choice was motivated by the fact that the decrease in HR or oxygen saturation due to a previous CASE might affect the vital signs in the patient monitor and the derived feature values computed for a subsequent CASE. This could have therefore possibly caused a wrong detection and influenced the learning process of the detection models based on machine learning. Previous studies indicated that the definition of 7.5 min pre-apnea (as well as post-apnea) windows might be an optimal solution to perform a prediction study for apnea of prematurity [20,24,31] but also suggested that reduction of this window length would better suit the needs of the clinical practice. Our choice yielded a total of 5646 CASES to be included in (72.2% of the initial CASEs).

After this process, all the remaining CASEs having an 'error' annotation were discarded from the study. This resulted in a total of 5460 CASEs to be considered for the upcoming steps (69.8% of the initial CASEs).

For all the included CASES, we included information starting from 30 s before the start of a CASE (-30 s) to 15 s after the start of the CASE and we used a moving step size of 5 s. This time window was chosen so that the included time was shorter than the minimum time requested by different apnea definitions to provide an annotation (15 s of cessation of breathing) [9–11]. This criterion led to feature extraction from four 30 s windows: [-30 s, 0 s), [-25 s, 5 s), [-20 s, 10 s) and [-15 s, 15 s), where 0 s was the moment of the start of a CASE.

2.4. Signal processing

RR-intervals were derived from the ECG by applying an algorithm for R-peaks detection and computing the time between consecutive R-peaks, using the same method described in our previous studies [27,32]. From the ECG, the signal instability index (SII) was then calculated with the aim of quantifying patient motion [27,32]. This measure was obtained by applying a band-pass filter (0.001–0.40 Hz) to 10 s-long ECG windows and subsequently by computing a kernel density estimate to return a measure of the patient motion for every second (1 Hz). Low values for this signal are associated with an absence of movement whereas higher values suggest that a movement is performed by the patient.

The CI was processed following the steps described by Redmond et al. to obtain the respiration Ribcage Respiratory Effort (RRE) [33]. This signal was computed by subtracting its mean and by removing the HF noise present above respiratory frequencies by applying a low-pass filter with a tenth-order Butterworth filter with a cutoff equal to 0.8 Hz. The resulting signal obtained for each patient received an amplitude normalization to remove the contribution of the tidal volume, a parameter that is different for each patient. In order to perform the normalization, turning points were first identified and the median peak-to-trough amplitude was found considering all differences between consecutive peaks and troughs. This value was then used to normalize the whole patient signal. The cardiorespiratory coupling signal (CRC) was finally computed by resampling the respiration RRE in correspondence with R-peaks in the ECG. No additional processing was performed on the SpO_2 measured with the photoplethysmogram.

2.5. Feature extraction

The RR-intervals, SII, respiration RRE, CRC and SpO_2 were all used to extract a total of 47 features for 5460 CASEs. Detailed information regarding all features can be found in Appendix A and only features that are less commonly used are more extensively explained in this section, with our motivation to include them in our detection models. Each feature, unless stated differently, was extracted considering 30 s windows following the windows defined in paragraph 2.3.

Features extracted from the RR-intervals using time-domain, frequency-domain and non-linear measures are well-established in previous studies. These include the Mean RR, the standard deviation of the normal RR intervals (SDNN) and the root mean square of successive differences between adjacent normal RR intervals (RMSSD), which can provide an estimate of overall heart-rate variability as well as an estimate of its short-term components [34,35]. We also determined the Sample Asymmetry of the RR-intervals, a measure of symmetry in the histogram of RR-intervals, which in previous studies showed clear differences before different illnesses (e.g., sepsis and systemic inflammatory response syndrome) compared to other instances [36]. Sample entropy, a measure able to quantify similarity for different sequences within a time-series [37], was also applied to the RR-intervals [38]. The Percentage of Decelerations and Standard Deviation of Decelerations were calculated since both proved to be effective in predicting the onset of sepsis [27,39].

The phase-rectified signal averaging (PRSA) [40,41] is capable of quantifying quasi-periodic oscillations in a physiological signal by filtering out artefacts and noise. We identified anchor points considering both increases in the signal (increase events) as well as decreases (decrease events). Windows of length equal to 40 time samples were set around the anchor points (i.e., L=20), a choice motivated by the short time windows (30 s) used in this study. From these PRSA waveforms seven different features were extracted [42]. These included the Immediate Deceleration and Acceleration Response (IDR and IAR) as well as the Average Deceleration and Acceleration Responses (ADR and AAR), with adjusted parameters considering the length of the time windows used in this study. In addition to these features, the Slope of the Decel-

eration and Acceleration Response (SDR and SAR), which are able to quantify the rate of HR response, were also included. Finally, a new feature extracted from the PRSA was developed and included in this study. This was defined by dividing the difference between the count of anchor points from the PRSA with decrease events and with increase events by the count of RR-intervals and then multiplying this value by 100 (Anchor Points Decrease versus Increase).

The Fast Fourier Transform (FFT) was applied on the RR-intervals after resampling this signal at 250 Hz to extract the power spectral densities, allowing to measure the development of the cardio-respiratory coupling and the presence of respiratory sinus arrhythmia (RSA), as suggested in previous studies [43,44]. To achieve this aim, we used the frequency ranges indicated by Indic et al. [43] and defined the Low Frequency (LF: 0.01–0.15 Hz), High Frequency (HF: 0.15–0.45 Hz), Super High Frequency (sHF: 0.45–0.7 Hz) and Ultra High Frequency (uHF: 0.7–1.5 Hz) ranges. The main reason for this choice compared to those provided in other studies [33,44] was the possibility of using the same frequency ranges also for the FFT of the respiration RRE. In addition to these frequency-domain features, we also extracted the LF/HF Ratio and the Mean Respiratory Frequency derived from the RR-intervals, defined as the frequency of maximum power in the frequency range 0.15–0.45 Hz [33].

Four features were extracted from the SII to quantify the movements of premature infants. These were the Mean SII, the Standard Deviation of the SII, Skewness of the SII and Interdecile Range of the SII. These were selected for their ability to identify motion patterns [27,32,45].

From the respiration RRE we extracted the Mean RRE, Standard Deviation of the RRE, Skewness of the RRE and Interdecile Range of the RRE. Sample Entropy of the RRE was computed with the same parameter choices described for the RR-intervals. Other time-domain features extracted from the respiration RRE include the Envelope Power, the Breath-by-Breath Correlation, the Breath Length Variation and the Time Domain Respiratory Frequency, features that proved to be particularly useful in classifying sleep stages. A detailed explanation for these features can be found in the study from Redmond et al. [33]. The Standardized Median of the Peaks and the Standardized Median of the Troughs were then computed as additional features to quantify the amplitude of the respiratory peaks and troughs [46]. With regards to features from the frequency domain, the power spectral densities were computed from the FFT of the respiration RRE by using the same LF, HF, sHF and uHF ranges previously mentioned for the case of the RR-intervals [43]. Similarly to the case of the RR-intervals, the frequency of maximum power in the frequency range 0.15–0.45 Hz was also computed considering the respiration RRE (Mean Respiratory Frequency derived from the respiration RRE) [33].

Cardiorespiratory features, which have proven to be particularly useful to identify sleep stages as well as cardiorespiratory phase-coupling during obstructive sleep apnea in adults, were introduced to verify whether they can be helpful in the context of apnea detection in premature infants [47,48]. For their computation we applied the nonlinear visibility graph (VG), a method which allows for the description of a time-series based on geometric criteria [49], to the CRC, as suggested by Long et al. [47]. This method requires the calculation of the degree δ of all data points in the time-series, also known as nodes, based on their visibility on other data points. The Mean Degree of the nodes and the Degree Variation of the constructed VG network were then introduced as features to quantify the degree of complexity of the time-series. The Assortativity Coefficient was instead introduced to estimate whether nodes were more likely to connect with high-degree or low-degree nodes [47]. Similarly to the study by Long et al., we used 3.5 min windows for the computation of features, in order to capture enough changes in the RR-intervals. However, we set the upper time limit for these extended windows to match exactly the end of each 30 s windows previously defined (i.e., the last 30 s of the 3.5 min windows corresponded to the 30 s windows used to extract other features).

Features extracted from the SpO_2 included its mean and standard deviation. In addition, we also estimated the slope of linear fitting of all data points within a time window. These features were selected to provide an indication about whether a desaturation was present in the current time window or expected in the upcoming ones.

Finally, Z score normalization was applied to the feature matrix which included features extracted for each CASE considering the different time windows. After the feature extraction process, CASEs presenting features with two or more consecutive 'NaN' values were discarded resulting in a total of 5333 CASEs to be considered for the detection models (68.2% of the initial CASEs). As it is reported in Table 1, for each patient we found a median and [IQR] value of 284.5 [187.5 – 367] CASEs and 26.5 [9.5 – 62.25] CAs annotated by clinical experts in the 48 h of data. In 5 patients very few CAs were annotated (count of CAs $\leq$ 5) while a high number of CAs was found in 6 other patients (count of CAs $\geq$ 50).

2.6. Machine learning: detection models

Detection models based on three traditional machine learning algorithms were employed in this study to perform CA detection: logistic regression (LR), random forest (RF) and support vector machines (SVM). LR was selected for its simplicity, which guarantees an easy transfer of knowledge and interpretability of the results. RF and SVM were selected following previous studies which proved that they can be suitable for apnea prediction in premature infants [22,23].

LR was implemented with the elastic net penalty, which linearly combines the L1 and L2 penalty functions of the lasso (least absolute shrinkage and selection operator) and ridge methods. Elastic net was adopted since different studies highlighted that it usually holds a better predictive power compared to the lasso regression while still performing feature selection, a characteristic that was considered relevant for the relatively high number of included features compared to the number of CASEs (records in machine learning terminology) [50]. We performed hyperparameter optimization by means of cross-validation, further discussed in the next paragraph, for the parameter C, which indicates how much LR is penalized and L1 ratio, which influences the algorithm penalty choosing among a mere L1 penalty, a mere L2 penalty or a combination of L1 and L2 penalties.

Three hyperparameters were optimized for the RF: the number of trees, the number of candidate features to consider for the best split at each node, and the maximum depth of the tree.

Finally, three hyperparameters were optimized for the case of the SVM. These included the regularization parameter, the type of kernel to be used and kernel coefficient, suitable for all the different types of kernels used in this study. A list of all the values for each hyperparameter that were investigated is presented in Table 2. All the detection models here described were implemented using the Scikit-learn in Python (Python Software Foundation, Fredericksburg, United States).

2.7. Machine learning: validation and evaluation

Two cross-validation methods were used for each detection model to optimize the hyperparameters, as shown in Fig. 1. The first cross-validation method was a leave-one-patient-out cross-validation (LOPO CV), using for each iteration the CASEs from one

Table 2
List of all machine learning algorithms and parameters values investigated to perform hyperparameter optimization for the different detection models.

Machine Learning Algorithms	Parameters	Parameters Values
Logistic Regression with Elastic Net Penalty (LR)	C	0.001, 0.005, 0.01, 0.05, 0.1, 0.5, 1
	L1 ratio	0, 0.001, 0.005, 0.01, 0.05, 0.1, 0.5, 1
Random Forest (RF)	Number of Trees	1, 2, 5, 10, 20, 50, 100, 200
	Number of Features for the Best Split	1, 2, 5, 10, 'log2' (i.e., $\log_2 num.features$), 'auto' (i.e., $\sqrt{num.\ features}$)
	Maximum Depth of the Tree	1, 2, 5, 10, 'None' (i.e., nodes are expanded until all leaves are pure or until all leaves contain less than 2 samples)
Support Vector Machines (SVM)	Regularization Parameter	0.1, 0.5, 1, 5, 10, 50, 100
	Type of Kernel	'rbf' (i.e., radial basis function), 'poly', 'sigmoid'
	Kernel Coefficient	'scale' (i.e., 1/(num. features • var(input matrix))), 1, 0.1, 0.01, 0.001, 0.0001

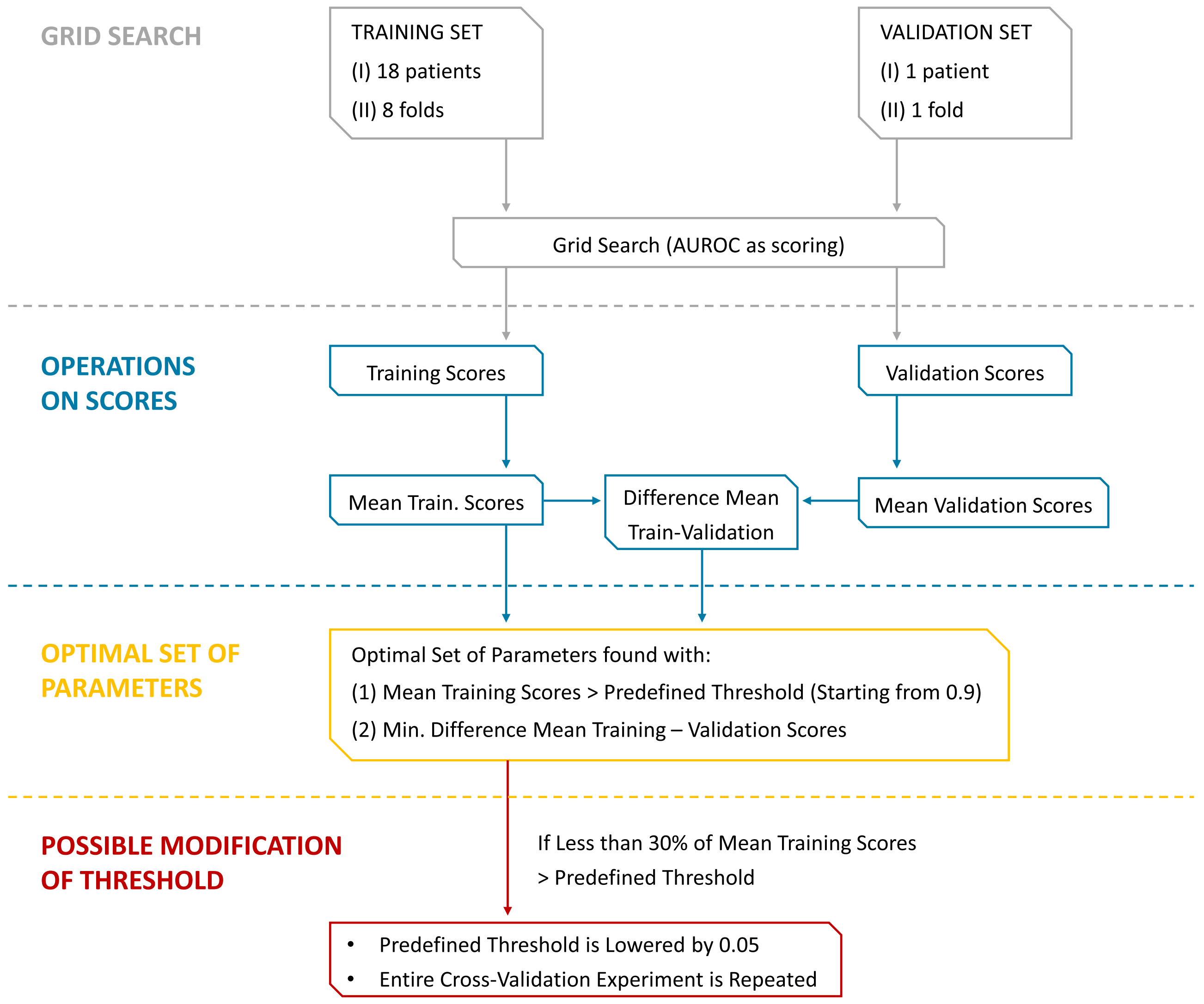

Fig. 2. Steps used in this study to perform hyperparameter optimization for all detection models and for both cross-correlation methods, leave-one-patient-out cross-validation (LOPO CV (I)) and a 10-fold cross-validation (10-fold CV (II)). The optimal parameters found using these criteria were then applied to the patient/fold left as test set.

patient as a test set. LOPO CV was selected as it relates well to clinical practice, where each new patient admitted in a NICU is treated as a test set. CASEs from the remaining 19 patients were also separated using a second LOPO CV, which allowed us to obtain for each iteration 18 patients in the training set and 1 patient in the validation set in order to perform hyperparameter optimization for each detection model. All steps regarding hyperparameter optimization are shown in Fig. 2. A grid search approach was used to determine the training and validation scores for each iteration of the second LOPO CV, computed by using the area under the receiver operating characteristic curve (AUROC) as a scoring function. The mean training scores, mean validation scores and the difference between the mean training and mean validation scores were computed for each iteration of the first LOPO CV. The optimal set of parameters for the detection model to be used on a test set was then selected based on two requirements: a set of parameters leading to (1) a mean training score greater than a predefined threshold (Th_{Class}), solution adopted to prevent underfitting, and to (2) the minimum difference between the mean training and mean validation scores, solution implemented to avoid overfitting. Th_{Class} was

empirically set equal to 0.9. In case less than 30% among the mean training scores computed for all sets of parameters were found to be greater than 0.9, its value was decreased each time by 0.05 and the full experiment repeated until this condition was fully satisfied for all the iterations of the first LOPO CV.

As the CAs were unevenly distributed over the patients, with 5 patients having only a few CAs, we also trained the detection models using a a 10-fold cross-validation (10-fold CV), folded over CASEs rather than patients, as a second cross-validation method. This experiment allowed to distribute CAs more evenly across all the folds, resulting in each fold having around one CAs per 6 CASEs. On the other hand, an important limitation of 10-fold CV is that the inclusion of CASEs from different patients in the training and test sets can introduce overfitting, a problem that is not found with LOPO CV. Nonetheless, this cross-validation method was included in our study also to quantify the extent of the overfitting compared to LOPO CV. Furthermore, as shown in Fig. 2, a second cross-validation consisting of 9 folds, a number selected to keep consistency with the choice applied for the second LOPO CV (where the number of patients was reduced by 1 compared to the first LOPO CV), was used to create the training and validation sets and to perform hyperparameter optimization, following the same steps as described above for LOPO CV.

2.8. Machine learning: experiments

Five different machine learning experiments were performed. These are also all indicated in Fig. 1. All the experiments allowed to evaluate the detection of CAs among CASEs in the test set by using the mean AUROC as metric, unless stated otherwise.

As a first experiment, we evaluated the performance of the different detection models based on machine learning and two cross-validation methods on the detection of CAs using information from all time windows. Features extracted from all time windows [-30 s, 0 s), [-25 s, 5 s), [-20 s, 10 s) and [-15 s, 15 s) were fed to all detection models, for both cross-correlation methods. This allowed us to verify which detection model worked better for CA detection and whether AUROC values were consistent for both cross-correlation methods.

As a second experiment, we investigated the relevance of including features from an increasing number of time windows, starting from window [-30 s, 0 s) only and up to windows [-30s, 0 s), [-25 s, 5 s), [-20 s, 10 s) and [-15 s, 15 s), on the detection. This experiment was also performed for all three detection models and both cross-validation methods.

Before proceeding with the following experiments, we identified the best performing detection model and investigated the feature relevance and the hyperparameters selected per each fold. However, only detection models created by means of LOPO CV were considered since they prevent overfitting (i.e., LOPO CV does not allow to use CASEs extracted from one patient both in the training and test set). In addition to this, the use of LOPO CV might be more reasonable in a clinical context, where an algorithm should work proficiently immediately after the admission of a new patient, where no training can be done with previous events extracted from this same patient. This detection model was then used to perform the remaining experiments.

The third experiment consisted of verifying whether the detection was performed differently for different groups of patients. At first, we compared AUROC values for control and LOS patients and applied the Wilcoxon signed-rank test to compare AUROC values from paired patients belonging to the two groups. We then compared AUROC values for each patient experiencing a low number of CAs (count of annotated CAs $\leq$ 5) and high number of CAs (count of CAs $\geq$ 50). Due to the low number of patients in these two groups, for this comparison we only evaluated the mean, minimum and maximum AUROC for patients in the two groups.

As a fourth experiment we extracted the confusion matrices found after the definition of different thresholds for the false positive rate (FPR) in the mean ROC curve. This allowed to evaluate how different metrics, recall (TPR), precision (PPV), miss rate (or false negative rate (FNR)) and the specificity (or true negative rate (TNR)), varied per each selected FPR. This experiment allowed us to understand the feasibility of using the selected detection model and cross-validation method for performing CA detection in clinical practice.

As a fifth and final experiment, after the identification of an optimal FPR, we investigated how the selected detection model performed in detecting different events (recall). At first, we investigated the percentages of correct detections (recall) for all CAs, CAs followed by a bradycardia (HR $\leq$ 80 bpm), CAs followed by a decrease in HR (80 bpm $<$ HR $\leq$ 100 bpm), CAs followed by a desaturation ($SpO_2 \leq$ 80%), CAs followed by a decrease in SpO_2 (80% $< SpO_2 \leq$ 88%), and CAs followed by both a bradycardia and a desaturation (also often referred as apnea bradycardia desaturation events in literature [17,19]). The definitions for bradycardia and desaturation were selected following the thresholds for alarms used in our NICU [51]. We then evaluated the percentages of correct detection of CAs with different length. For this purpose we separated CAs considering the following lengths L (i.e., 10 s $\leq$ L $<$ 20 s, 20 s $\leq$ L $<$ 30 s, 30 s $\leq$ L $<$ 40 s, 40 s $\leq$ L $<$ 50 s, 50 s $\leq$ L $<$ 60 s, L $\geq$ 60 s) in a similar way to what was proposed by Mohr et al. for an analysis of different apnea lengths [19]. Finally, we investigated the percentages of correct detection for CASEs agreed and disagreed upon by clinical experts during the first round of annotations.

3. Results

Fig. 3 shows results from the cluster analysis of CASEs, in which transitions for the annotations from the clinical experts were investigated. This analysis showed consistency for annotations from clinical experts within clusters, irrespectively of the transition that was considered. Considering the first transition as an example, clusters starting from a CA had a far higher likelihood of being followed by a CA rather than a rejection for the following CASEs (16.7% vs. 3.8%). The same consideration was found for clusters starting from rejection, which were far more ofthen followed by a rejection (24.4%) rather than a CA (1.1%). All the other percentages of transitions indicated instead the end of a cluster.

Our first machine learning experiment consisted of investigating the performance of the different detection models considering features from all time windows. Fig. 4 shows the mean ROC curves for the different detection models. Fig. 4A, computed by using LOPO CV, shows that the highest AUROC was found when using the LR with elastic net penalty (0.88 $\pm$ 0.08). This result was followed closely by the AUROC found using SVM (0.87 $\pm$ 0.09) and RF (0.85 $\pm$ 0.09). Slightly higher results compared to those found with LOPO CV were found by means of 10-fold CV, as shown in Fig. 4B, where the highest AUROC was found with LR with elastic net penalty (0.90 $\pm$ 0.02), result followed closely by the AUROC found with SVM (0.89 $\pm$ 0.02) and RF (0.87 $\pm$ 0.03).

Our second experiment investigated the relevance of including features from an increasing number of time windows on CA detection. AUROC values (mean $\pm$ standard deviation) found by considering features from an increasing number of time windows and including therefore progressively more seconds of CASEs are presented in Table 3. In this table a stepwise increase in the mean AUROC values for all detection models is observed, for both cross-validation methods.

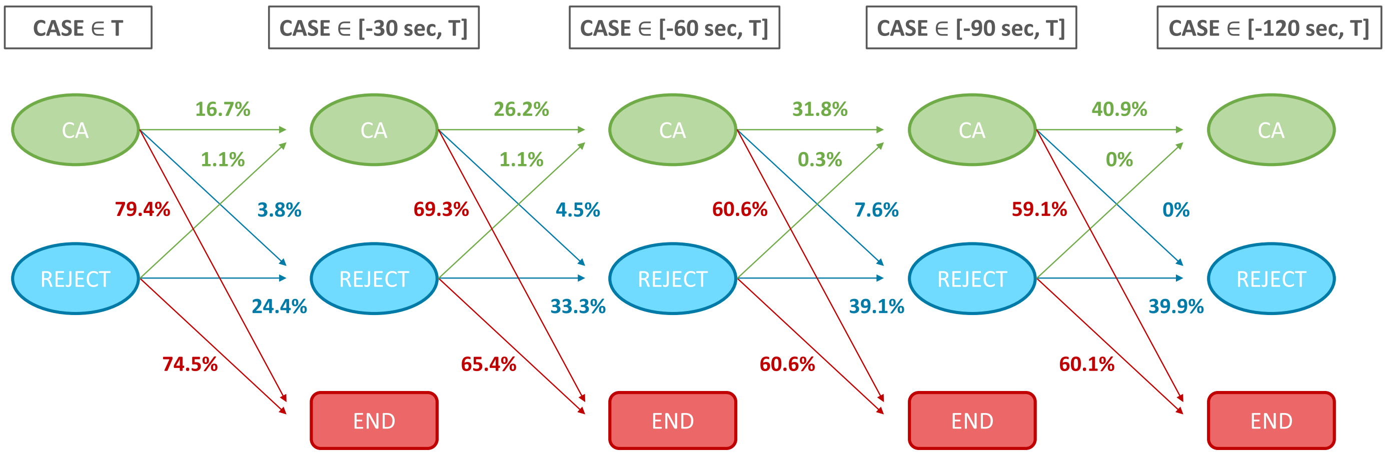

Fig. 3. Representation of the first four stages of transitions between all central apnea-*suspected* events (CASEs) included in this study. Annotations returned by the clinical experts during the second round of annotation are used to create this representation. END is also added to indicate the ending of a cluster. Considering the first transition as an example, clusters starting from a CA were followed by a CA 16.7% of the time, were followed by a rejection 3.8% of the times and were not followed by another CASE in the following 30 s 79.4% of the times. Only the most likely transitions are represented (e.g., transition from CA to error is not represented due to extremely low likelihood of this transition).

Table 3
AUROC values (mean ± standard deviation) found by considering features an increasing number of consecutive time windows for each central apnea-*suspected* event (CASE). Results found for detection models based on logistic regression (LR) with elastic net penalty, random forest (RF) and support vector machines (SVM), as well as both cross-validation methods, leave-one-patient-out cross-validation (LOPO CV) and 10-fold cross-validation (10-fold CV), are shown. The following time windows including progressively more seconds of CASEs were considered to obtain these results: [-30 s, 0 s), [-25 s, 5 s), [-20 s, 10 s) and [-15 s, 15 s).

LOPO CV		LR	RF	SVM
1 Window	AUROC (Mean ± Std)	0.73 ± 0.12	0.70 ± 0.11	0.74 ± 0.12
2 Windows	AUROC (Mean ± Std)	0.80 ± 0.10	0.76 ± 0.11	0.79 ± 0.10
3 Windows	AUROC (Mean ± Std)	0.87 ± 0.08	0.81 ± 0.09	0.86 ± 0.08
4 Windows	AUROC (Mean ± Std)	0.88 ± 0.08	0.85 ± 0.09	0.87 ± 0.09
**10-fold CV**		**LR**	**RF**	**SVM**
1 Window	AUROC (Mean ± Std)	0.80 ± 0.02	0.78 ± 0.02	0.80 ± 0.02
2 Windows	AUROC (Mean ± Std)	0.85 ± 0.03	0.81 ± 0.03	0.84 ± 0.03
3 Windows	AUROC (Mean ± Std)	0.89 ± 0.02	0.86 ± 0.03	0.88 ± 0.03
4 Windows	AUROC (Mean ± Std)	0.90 ± 0.02	0.87 ± 0.03	0.89 ± 0.02

As shown in Fig. 4A, the best performing detection model by means of LOPO CV was identified in LR with elastic net penalty trained using features from all 4 time windows, since it showed the highest mean AUROC. Feature relevance was investigated by extracting the coefficients from the detection model. These are log odds ratios and while positive values indicate increased risk towards CA, negative values indicate increased risk towards rejection. Median log odds ratios computed considering all folds for the twenty most relevant features, ranked considering their absolute values are shown in Fig. 5. In general, relevance for features extracted from the respiration RRE was found to be generally the highest among all features included in this study. Different features extracted from the RR-intervals also proved to be relevant for the detection whereas features extracted from the SII, CRC and SpO_2 showed lower median log odds ratios. The most often selected set of hyperparameters consisted of C = 0.005 and L1 ratio = 1, and was selected for 16 out of 20 folds.

In the third experiment we evaluated whether the detection was performed differently for different groups of patients while using the detection model made using LR with elastic net penalty and LOPO CV. AUROC values for all the patients in the test set are shown in Fig. 6, represented in blue for control patients and in red for LOS patients. No statistical significance while comparing matching LOS and control patients was found (p-value > 0.05). AUROC values for the 5 patients experiencing a low number of CAs (count of annotated CAs ≤ 5) showed high values (mean AUROC = 0.948, min AUROC = 0.895, max AUROC = 1) while a wide variety of AUROC values was found in the six patients with high number of CAs (count of CAs ≥ 50) (mean AUROC = 0.850, min AUROC = 0.659, max AUROC = 0.951).

In our fourth experiment, three different FPR (0.1, 0.2 and 0.3) were defined to extract the confusion matrices for the same detection model based on LR with elastic net penalty and LOPO CV. Confusion matrices as well as other metrics are shown in Table 4. A FPR = 0.3 in particular allowed to detect CAs with a recall (TPR) = 0.78, a precision = 0.42 and a miss rate (FNR) = 0.22. Since we considered as highest priority to avoid missing too many CAs even at the cost of a slight increase of false CA detection, we chose to use this FPR value for the subsequent and final experiment.

In our fifth experiment, percentages of correct detection for different events found with FPR = 0.3 were computed. Results are shown in Fig. 7 together with the total number of events considered for each group. Percentages of correct detections were found to be equal to 78.2% for all CAs and very similar values were also found for CAs followed by a desaturation (79.0%) or decrease in SpO_2 (75.7%). Significantly higher values percentages of correct detections were found for CAs followed by a bradycardia (93.4%), by a decrease in HR (93.9%) and by both a bradycardia and a desaturation (95.2%). In addition, percentages of correct detection were found to be higher for CAs with length 20 s $\leq$ L $<$ 30 s (88.3%) or for CAs with length L $\geq$ 60 s (91.7%). Finally, percentages of correct

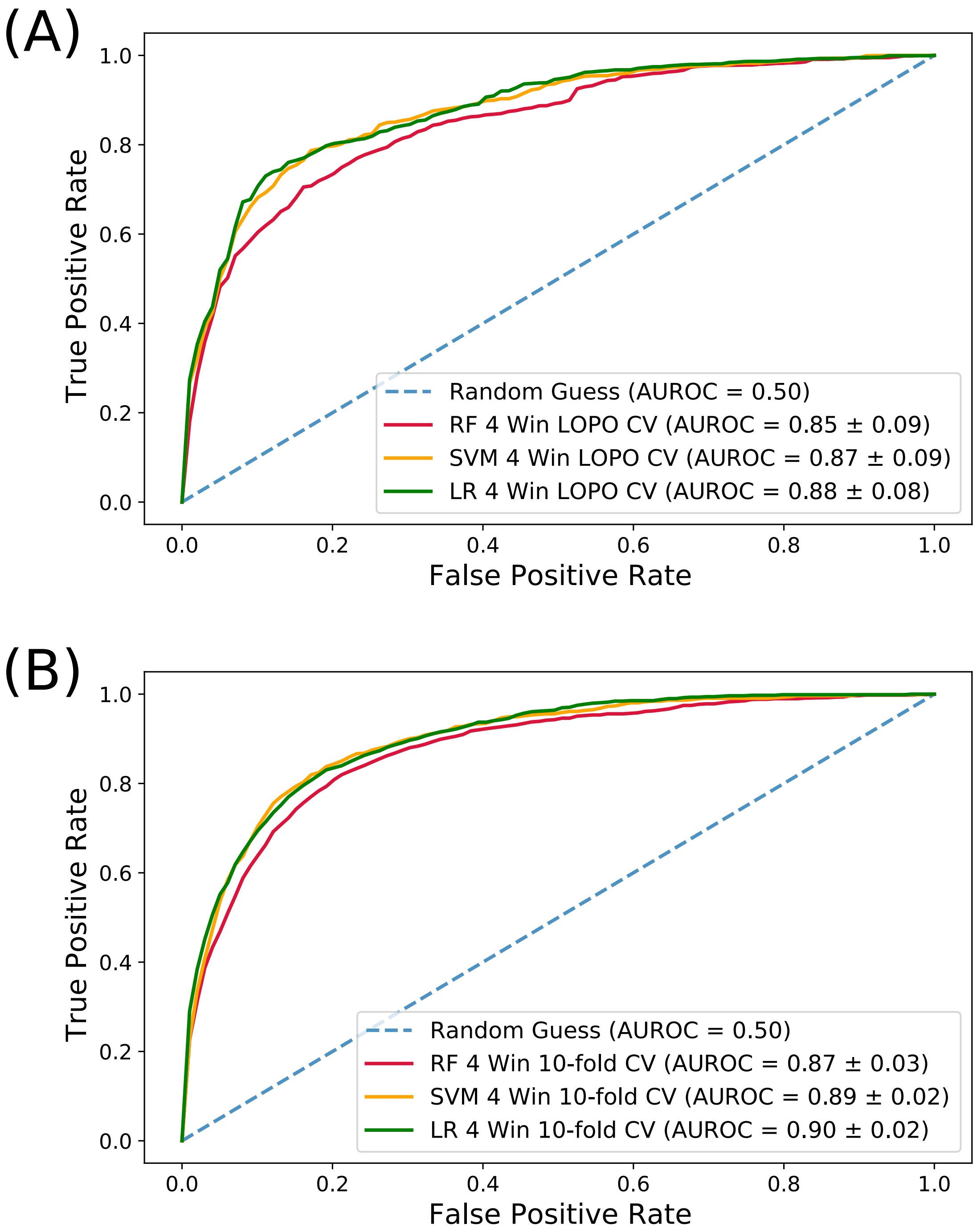

Fig. 4. Mean receiver operating characteristic (ROC) curves computed with all detection models based on random forest (RF), support vector machines (SVM) and logistic regression (LR) with elastic net penalty using features from windows [-30 s, 0 s), [-25 s, 5 s), [-20 s, 10 s) and [-15 s, 15 s) with the leave-one-patient-out cross-validation (LOPO CV) (A) and 10-fold cross-validation (10-fold CV) (B).

detection were found to be higher for CASEs originally agreed upon by clinical experts (82.9%) compared to CASEs for which there was no initial agreement (60.1%).

4. Discussion

Correct identification of apnea of prematurity is important as a correlation has been described between apnea of prematurity and neurodevelopmental impairment as well as other pathologies, such as sepsis, that can represent a major threat to the life of premature infants [2,4–6]. Therefore we developed a detection model based on machine learning which shows that it is feasible to automatically detect true CAs and to reduce the occurrence of high load of false apnea alarms observed in the current clinical practice.

As a result of an investigation of CASEs extracted from our population of premature infants, we found a high likelihood for CASEs to appear in clusters, as 27.8% of the total count of CASEs was found to be preceded by another CASE in the previous 30 s. This result was found to be in agreement with previous literature studies which also indicated that periodic apneas can be found in premature infants [6,14,20]. In addition to this we observed that consistency in annotations can be found for CASEs appearing in a cluster. In particular, we found a far higher likelihood for CAs to be clustered together rather than with other apneic events anno-

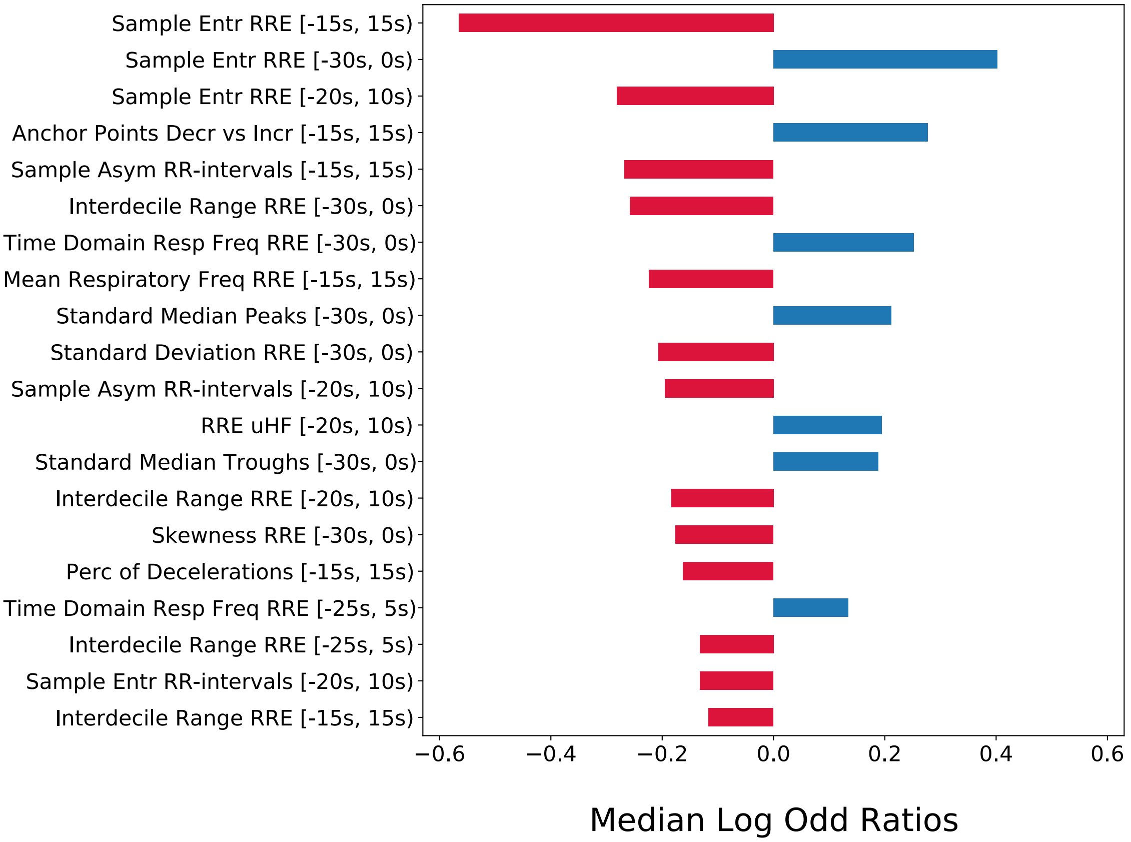

Fig. 5. Feature relevance for the logistic regression (LR) with elastic net penalty fed with features from time windows [-30 s, 0 s), [-25 s, 5 s), [-20 s, 10 s) and [-15 s, 15 s) and trained using leave-one-patient-out cross-validation (LOPO CV). Feature relevance was investigated by extracting the coefficients, indicating the log odds ratios. Median log odds ratios were then computed and were ranked considering their absolute values to represent the twenty most relevant features.

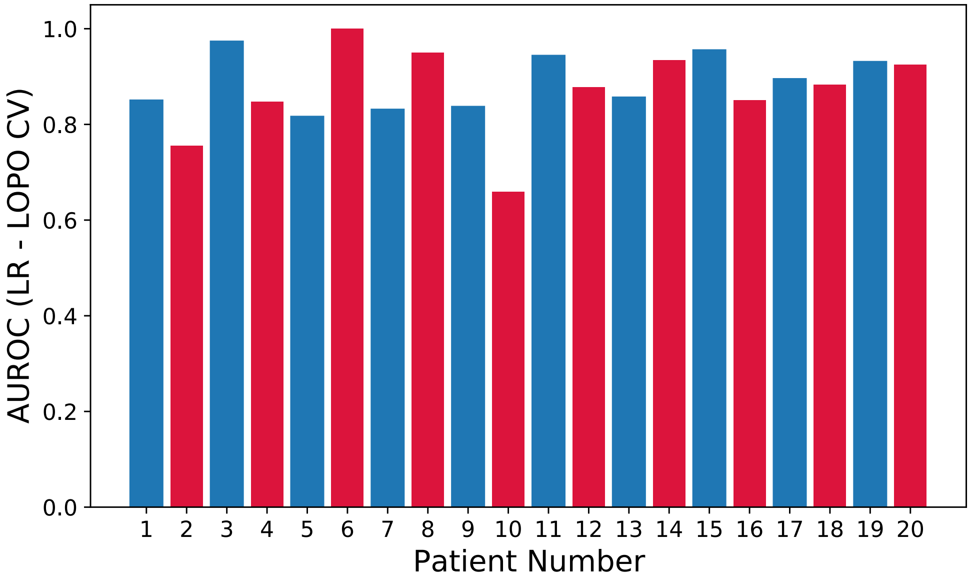

Fig. 6. AUROC values computed for each patient in the test set with the detection model based on logistic regression (LR) with elastic net penalty using features from windows [-30 s, 0 s), [-25 s, 5 s), [-20 s, 10 s) and [-15 s, 15 s) with the leave-one-patient-out cross-validation (LOPO CV). AUROC values are represented in blue for control patients and in red for LOS patients.

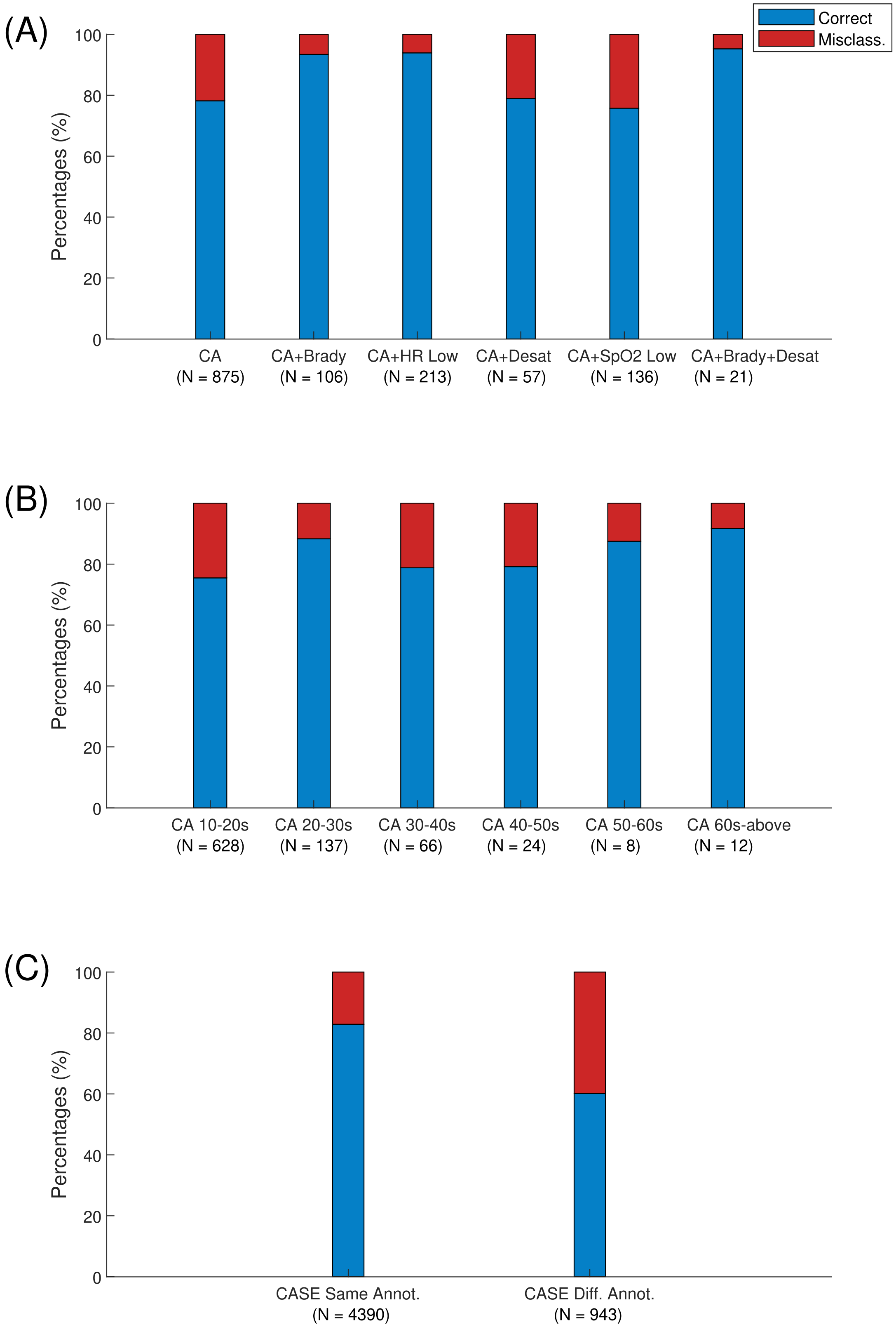

Fig. 7. Percentages of correct detections (recall) performed by logistic regression (LR) with elastic net penalty trained using leave-one-patient-out cross-validation (LOPO CV) on CASEs present in the test set when a FPR = 0.3 was selected. In particular, in (A) all central apneas (CAs), CAs followed by a decrease in heart rate (HR) or oxygen saturation (SpO_2), in (B) CAs with different lengths and in (C) central apnea-*suspected* events (CASEs) agreed and disagreed upon by clinical experts during the first round of annotations are represented.

Table 4
Confusion matrices found considering a False Positive Rate (FPR) equal to (a) 0.1, (b) 0.2 and (c) 0.3 in the mean ROC curve for logistic regression (LR) with elastic net penalty, estimated labels by this detection model using the corresponding threshold and the annotations from the clinical experts as true labels.

(a) FPR = 0.1		Estimated Labels	
		Central Apneas	Rejection
True Labels	Central Apneas	529	346
(Annotations)	Rejections	328	4130
Recall (TPR)	0.60	Precision (PPV)	0.62
Miss Rate (FNR)	0.39	Specificity (TNR)	0.93
(b) FPR = 0.2		Estimated Labels	
		Central Apneas	Rejections
True Labels	Central Apneas	633	242
(Annotations)	Rejections	662	3796
Recall (TPR)	0.72	Precision (PPV)	0.49
Miss Rate (FNR)	0.28	Specificity (TNR)	0.85
(c) FPR = 0.3		Estimated Labels	
		Central Apneas	Rejections
True Labels	Central Apneas	684	191
(Annotations)	Rejections	937	3521
Recall (TPR)	0.78	Precision (PPV)	0.42
Miss Rate (FNR)	0.22	Specificity (TNR)	0.79

tated as rejections. Consistency of annotations within clusters of CASEs might also be considered for the improvement of detection models. Future studies could also provide more precise annotations for rejections (e.g., separating obstructive and mixed apneas) and investigate whether consistency characterizes other clusters as well.

Furthermore, our first machine learning experiment showed very similar AUROC values for the different detections models that were implemented based on LR with elastic net penalty, RF and SVM, considering features extracted from all 4 time windows and both cross-validation methods. AUROC values found with RF and SVM were found to be higher than in the promising previous literature studies which made use of the same machine learning algorithms [22,23]. Our AUROC values found by means of LR with elastic net penalty are instead more difficult to compare to results achieved in the past. This is because, to the best of our knowledge, LR was only used in combination with a Gaussian mixture model by Zuzarte et al. with the aim of performing apnea prediction [6]. Furthermore, this previous model allowed to perform a live apnea prediction, while in our study we considered performing apnea detection based on single CASEs, similarly to what was done in previous studies who used RF and SVM [22,23]. In this study mean AUROC values were also found to be consistent for both cross-validation methods. However, it is important to highlight the higher uncertainty, indicated by the AUROC standard deviation, found with LOPO CV, a result that is related to different count of CASEs identified per patient. On the other hand, methods based on testing the detection model on new patients, like LOPO CV, are more similar to the regular clinical practice, where a detection model based on machine learning should be trained with previously admitted patients and used (i.e tested) on a newly admitted patient. In addition to this, AUROC values found using 10-fold CV, which are due to overfitting resulting from the use of CASEs from the same patient in both the training and test set, are only slightly higher compared to those obtained with LOPO CV, giving further validity for the use of a model based on LOPO CV in the other experiments included in this study.

In the second machine learning experiment we found out that the highest AUROC values were found with features extracted from up to the first 15 s of each CASE, irrespectively of the detection model and the cross-validation method that was used. It must be noted, though, that AUROC values were quite similar by including only features up to the first 10 s of each CASE. This result is particularly significant for the clinical practice, as all definitions present in literature require a minimum of 10 s of a cessation of breathing to define apnea of prematurity [7–11]. Our detection model therefore not only detects apneas based on vital signs but predicts the occurrence of an upcoming true CA alarm.

The investigation of feature relevance performed for LR with elastic net penalty, features from all 4 time windows and LOPO CV showed higher median log odds ratios for features extracted from the respiration RRE compared to all the other features. This result is expected as a CA is likely diagnosed by observations of the CI, with consequences in HR and SpO_2 appearing just at a later stage. Nonetheless, features extracted from the RR-intervals also provided a significant contribution to the detection model whereas features extracted from the CRC and SpO_2 were found to be in general less relevant. In contrast to recent literature [6], in our study the motion signal (calculated in our study as SII) shows only a modest contribution, which may be explained by the fact that we focused our attention on apneas with a central origin, whereas patient movement is associated with apneas of an obstructive origin [52], and by the fact that the SII is extracted from the ECG signal, a signal on which the patient monitor applies different methods to prevent motion artifacts [32]. Future studies could therefore investigate whether wavelet-based algorithms, used for instance by Zuzarte et al. [6], are more suitable than SII to contribute to the detection of apnea of prematurity.

In our third machine learning experiment no difference was found by comparing AUROC values for LOS versus control patients,

indicating the possibility of applying LR with elastic net penalty for CA detection irrespectively of the patient group. We also noticed high AUROC values for 5 patients experiencing a low number of CAs. With an in-depth analysis focused on the interpretation of this result, we found out that CA records for patients with few CAs used as a test set generally presented high probability values, irrespectively of the machine learning model that was used. As soon as high threshold values (low FPR values) were trespassed, an increase in TPR and consequently in the AUROC values became immediately noticeable. We also speculate that this result might have a clinical reason, since we noticed a very regular breathing pattern in this group of patient. Because of this reason, a disruption in this breathing pattern could be easily recognized. Patients with several CAs often showed instead a less regular breathing pattern, which could have led to more rejections being wrongly identified as CAs and at the same time made CAs harder to be detected.

In our fourth machine learning experiment, a FPR = 0.3 was selected as an optimal threshold for our best performing detection model. This choice was performed since in the medical field it is preferred to correctly detect as many true events as possible (i.e., high recall), even at the cost of having a lower precision and triggering therefore more false alarms [9]. Recent studies have indicated a precision equal to 0.35 for the current monitoring systems available in clinical practice (i.e., up to 65% of the apnea alarms currently found in NICUs are false) [17,19,18]. Using our detection model with a FPR = 0.3 we improved the precision to 0.42, reducing the number of false alarms by 66% compared to the application of the optimized algorithm for central apnea detection only, an algorithm derived from the one originally described by Lee et al. which showed a lower number of false positives compared to what is obtained using the current monitoring techniques [17].

Percentages of correct detections (recall) were further examined in the fifth machine learning experiment and were found to be particularly high for CAs followed by a decrease in HR (80 bpm $<$ HR $\leq$ 100 bpm), a bradycardia and both a bradycardia and a desaturation, which are considered the most concerning CAs for the well-being of the infants [2,14]. Our results are in line with the findings of Williamson et al., who showed promising results with an AUROC equal to 0.79 while applying a different machine learning algorithm (i.e., quadratic classifier) [20]. Lower percentages were instead found when CAs were followed by a decrease in SpO_2, a result that can be related to the low relevance found for features extracted from this signal. An explanation for this result was given by Di Fiore et al., who indicated that false desaturation alarms can be reduced by using long averaging time, equal to at least 16 s [2], which is nowadays typically used in most clinical practices. Exceeding the first 15 s of CASE for feature extraction might therefore be one solution to improve detection of CAs followed by a decrease in saturation. On the other hand, since 15 s of cessation of breathing are sufficient to completely define apnea of prematurity [9–11], the inclusion of this requirement would have prevented us from the possibility of performing an actual prediction of CAs and was therefore not considered.

Another solution that can be explored in future studies is the definition of new features capable to estimate possible future decrease in SpO_2, including for instance features computed in the frequency domain which proved to be useful for diagnosing obstructive sleep apnea syndrome in adults [53]. Finally, it is important to note that the percentages of correct detections were lower in case the clinical experts disagreed during the first round of annotations (recall equal to 60.1% instead of 82.9%, found in case of agreement).

This study has some noteworthy limitations. First of all, a limited sample size of patients and CASEs, which was selected considering the time availability for clinicians to provide annotations in different rounds, as this annotation process – even though guided by a GUI – was still a very time-consuming task for the clinical experts. This was also the reason why annotations were made by no more than two clinical experts. Second, in this study detection models were only used retrospectively. Also, the two step approach, with first detection of cessations of breathing and then CAs is slow for implementation. Using a full dataset including all 30 s-long time windows, independent of the presence of a CASE, would be of interest. However, this would result in a highly unbalanced dataset. Third, the inclusion of more refined features from different signals, including the SpO_2, and the inclusion of other features to quantify body motion as well as the gestational age, postnatal days and birth weight could also improve the detection. Fourth, validation on data coming from a different dataset or another hospital was not investigated in this study. Since there is currently no public dataset available that includes annotations for CAs in premature infants as well as all the signals included in this study, future studies could include data from a different dataset to provide a further indication on the validity of the current results. However, we consider our study to be valid as a first feasibility study to verify our methodology before a larger-scale study including more data from more patients and different datasets. Finally, only machine learning models characterized by an easier transfer of knowledge regarding the results and that could cover regression, clustering and tree-based algorithms were included in this study. Future studies, more focused on the technical aspects, could however be considered to expand on the selected models for instance by including deep neural networks.

5. Conclusion

In this study different detection models based on machine learning were applied in order to detect CAs among CASEs. We show that CAs can be automatically detected correctly while reducing the number of false alarms, using a detection model based on well-known machine learning algorithms. A threshold for the FPR in the mean receiver-operating-characteristic curve equal to 0.3 led to a percentage of 78.2% correct detections for all CAs and 93.4% and 95.2% for the most critical and clinically relevant CAs, namely those followed by bradycardia or bradycardia and desaturation.

Declaration of Competing Interest

I declare that I participated in the design, execution and analysis of the paper with the title ‘Central Apnea Detection in Premature Infants using Machine Learning’ together with the all the colleagues listed here, that I have seen and approved the final version and that it has neither been published elsewhere. I also declare that I have no conflict of interest.

Acknowledgment

This study was done within the framework of the Eindhoven MedTech Innovation Center (e/MTIC) which is a collaboration between the Eindhoven University of Technology, Philips Research, and Máxima Medical Center. This study is a result of the ALARM project funded by the Nederlandse Organisatie voor Wetenschappelijk Onderzoek (NWO) grant No. 15345.

Appendix A

List of all the extracted features from the RR-intervals, Signal Instability Index (SII), respiration Ribcage Respiratory Effort (RRE), Cardiorespiratory Coupling (CRC) and SpO_2 signals.

Features extracted from the RR-intervals

#	Feature	Description
1	Mean RR	Mean RR-interval [34]
2	SDNN	Standard deviation of the RR-intervals [34,35]
3	RMSSD	Root mean square of successive differences between adjacent normal RR-intervals [34,35]
4	Sample Asymmetry of the RR-intervals	Measure of the asymmetry in the histogram of RR-intervals [36]
5	Sample Entropy of the RR-Intervals	Sample entropy of the RR-intervals, computed with m=2 and $r = 0.2 \cdot \sigma$ [37,38]
6	Percentage of Decelerations	Percentage of the RR-intervals longer than the mean RR-interval from the current time window [27,39]
7	Standard Deviation of Decelerations	Standard deviation of all RR-intervals that contribute to the Percentage of Decelerations [39]
8	Immediate Deceleration Response (IDR)	Difference between the maximum and minimum values of RR-intervals in the 5 data points after (including the anchor points) and before the anchor point from the PRSA curve computed considering increase events ($T = 1$) [40,42]
9	Immediate Acceleration Response (IAR)	Difference between the maximum and minimum values of RR-intervals in the 5 data points after (including the anchor points) and before the anchor point from the PRSA curve computed considering decrease events ($T = 1$) [40,42]
10	Average Deceleration Response (ADR)	Difference between the mean value of the 10 RR-intervals after (including the anchor point) and the 10 RR-intervals before the anchor point from the PRSA curve computed considering increase events ($T = 1$) [40,42]
11	Average Acceleration Response (AAR)	Difference between the mean value of the 10 RR-intervals after (including the anchor point) and the 10 RR-intervals before the anchor point from the PRSA curve computed considering decrease events ($T = 1$) [40,42]
12	Slope of the Deceleration Response (SDR)	Slope of the line joining the maximum and minimum values of the RR-intervals corresponding to the IDR computed from PRSA curve computed considering increase events ($T = 1$) [40,42]
13	Slope of the Acceleration Response (SAR)	Slope of the line joining the maximum and minimum values of the RR-intervals corresponding to the IAR computed from PRSA curve computed considering decrease events ($T = 1$) [40,42]
14	Anchor Points Decrease versus Increase	Feature computed by dividing the difference between the count of anchor points from the PRSA with decrease events and with increasing events by the count of RR-intervals and then multiplying this value by 100

Features extracted from the RR-intervals (resampled at 250 Hz)

#	Feature	Description
15	RR-intervals LF	PSD computed from the FFT considering the low frequency range (0.01-0.15 Hz) [43]
16	RR-intervals HF	PSD computed from the FFT considering the high frequency range (0.15-0.45 Hz) [43]
17	RR-intervals sHF	PSD computed from the FFT considering the super high frequency range (0.45-0.7 Hz) [43]
18	RR-intervals uHF	PSD computed from the FFT considering the ultra high frequency range (0.7-1.5 Hz) [43]
19	RR-intervals LF/HF Ratio	Ratio between the values for the PSD computed from the FFT in the LF and HF ranges [33,43]
20	Mean Respiratory Frequency RR-intervals	Frequency of maximum power in the range 0.15-0.45 Hz [33]

Features extracted from the Signal Instability Index (SII)

#	Feature	Description
21	Mean SII	Mean value of the SII
22	Standard Deviation of the SII	Standard deviation of the SII [27,32]
23	Skewness of the SII	Skewness of the SII [27,32]
24	Interdecile Range of the SII	Interdecile range of the SII [27,32]

Features extracted from the respiration Ribcage Respiratory Effort (RRE)

#	Feature	Description
25	Mean RRE	Mean value of the respiration RRE
26	Standard Deviation of the RRE	Standard deviation of the respiration RRE
27	Skewness of the RRE	Skewness of the respiration RRE
28	Interdecile Range of the RRE	Interdecile range of the respiration RRE
29	Sample Entropy of the RRE	Sample entropy of the respiration RRE, computed with $m = 2$ and $r = 0.2 \cdot \sigma$ [37,38]
30	Envelope Power	Computed by taking the standard deviation of the peaks for an epoch and of the standard deviation of the troughs. The mean value is computed and divided by the standard deviation of the respiration RRE for the epoch [33]
31	Breath-by-Breath Correlation	Computed by defining as breath cycle the time from the trough of one breath to the trough of the next. After computing the maximum cross-correlation value for adjacent breaths, this value is divided by the maximum of the energy of either breath alone to normalize the maximum cross-correlation value. The maximum cross-correlation values, for all pairs of adjacent breaths, are then averaged [33]
32	Breath Length Variation	Computed considering the standard deviation of the time between peak locations and the standard deviation of the time between trough locations and calculating the mean of these two values [33]
33	Time Domain Respiratory Frequency	Inverse of the mean time between adjacent peaks and between adjacent troughs [33]
34	Standardized Median of the Peaks	Computed by dividing the median of the peaks in the respiration RRE by their interquartile range [46]
35	Standardized Median of the Troughs	Computed by dividing the median of the troughs in the respiration RRE by their interquartile range [46]
36	RRE LF	PSD computed from the FFT considering the low frequency range (0.01-0.15 Hz) [43]
37	RRE HF	PSD computed from the FFT considering the high frequency range (0.15-0.45 Hz) [43]
38	RRE vHF	PSD computed from the FFT considering the super high frequency range (0.45–0.7 Hz) [43]
39	RRE uHF	PSD computed from the FFT considering the ultra high frequency range (0.7–1.5 Hz) [43]
40	RRE LF/HF Ratio	Ratio between the values for the PSD computed from the FFT in the LF and HF ranges [33,43]
41	Mean Respiratory Frequency RRE	Frequency of maximum power in the range 0.15-0.45 Hz [28]

Features extracted from the Cardiorespiratory Coupling (CRC)

#	Feature	Description
42	Mean Degree of the Nodes	Mean node degree computed from the Nonlinear Visibility Graph by averaging the degrees over the nodes with a 3.5 min window, with the last 30 s overlapping with the window used for the detection [47,49]
43	Degree Variation of the Nodes	Standard deviation of the node degrees computed from the Nonlinear Visibility Graph [47,49]
44	Assortativity Coefficient	Computed with the following equation: $\xi = \frac{M^{-1}\sum_i \alpha_i\beta_i - [M^{-1}\sum_i \frac{1}{2}(\alpha_i+\beta_i)]^2}{M^{-1}\sum_i \frac{1}{2}(\alpha_i^2+\beta_i^2) - [M^{-1}\sum_i \frac{1}{2}(\alpha_i+\beta_i)]^2}$ considering a network including a total of M edges, the i-th edge connects two nodes with degree of α_i and β_i at their ends [47,49]

Features extracted from the SpO_2

#	Feature	Description
45	Mean SpO_2	Mean value of the SpO_2
46	Standard Deviation of the SpO_2	Standard deviation of the SpO_2
47	Slope of the SpO_2	Slope of the linear fitting of all the SpO_2 points

References

[1] E.C. Eichenwald, Committee on Fetus and Newborn, "Apnea of prematurity, Pediatrics 137 (1) (2016) 1–7.
[2] J.M. Di Fiore, R.J. Martin, E.B. Gauda, Apnea of prematurity - perfect storm, Respir. Physiol. Neurobiol. 189 (2) (2013) 213–222.
[3] B.A. Sullivan, K.D. Fairchild, Vital signs as physiomarkers of neonatal sepsis, Pediatr. Res. 91 (2) (2022) 273–282.
[4] S.M. Gephart, J.M. McGrath, J.A. Effken, M.D. Halpern, Necrotizing enterocolitis risk, Adv. Neonatal Care 12 (2) (2012) 77–87.
[5] A. Janvier, M. Khairy, A. Kokkotis, C. Cormier, D. Messmer, K.J. Barrington, Apnea is associated with neurodevelopmental impairment in very low birth weight infants, J. Perinatol. 24 (12) (2004) 763–768.
[6] I. Zuzarte, D. Sternad, D. Paydarfar, Predicting apneic events in preterm infants using cardio-respiratory and movement features, Comput. Methods Programs Biomed. 209 (2021) 106321.
[7] N.N. Finer, R. Higgins, J. Kattwinkel, R.J. Martin, Summary proceedings from the apnea-of-prematurity group, Pediatrics 117 (3) (2006) S47–S51 Suppl.
[8] J. Zhao, F. Gonzalez, D. Mu, Apnea of prematurity: From cause to treatment, Eur. J. Pediatr. 170 (9) (2011) 1097–1105.
[9] S.B. Amin, B. Erica, Monitoring apnea of prematurity: validity of nursing documentation and bedside cardiorespiratory monitor, Am. J. Perinatol. 30 (8) (2013) 643–648.
[10] D.E. Elder, A.J. Campbell, D. Galletly, Current definitions for neonatal apnoea: are they evidence based? J. Paediatr. Child Health 49 (9) (2013) E388–E396.
[11] N.N. Finer, K.J. Barrington, B.J. Hayes, A. Hugh, Obstructive, mixed, and central apnea in the neonate: physiologic correlates, J. Pediatr. 121 (6) (1992) 943–950.
[12] S. Picone, R. Aufieri, P. Paolillo, Apnea of prematurity: challenges and solutions, Res. Rep. Neonatol. (2014) 101.
[13] O.P. Mathew, Apnea of prematurity: pathogenesis and management strategies, J. Perinatol. 31 (5) (2011) 302–310.
[14] C.F. Poets, Apnea of prematurity: what can observational studies tell us about pathophysiology? Sleep Med. 11 (7) (2010) 701–707.
[15] T. Carbone, L.C. Marrero, J. Weiss, M. Hiatt, T. Hegyi, Heart rate and oxygen saturation correlates of infant apnea, J. Perinatol. 19 (1) (1999) 44–47.
[16] D.E. Weese-Mayer, R.T. Brouillette, A.S. Morrow, L.P. Conway, L.M. Klemka-Walden, C.E. Hunt, Assessing validity of infant monitor alarms with event recording, J. Pediatr. 115 (5) (1989) 702–708 PART 1.
[17] H. Lee, C.G. Rusin, D.E. Lake, M.T. Clark, L. Guin, T.J. Smoot, A.O. Paget-Brown, B.D. Vergales, J. Kattwinkel, J.R. Moorman, J.B. Delos, A new algorithm for detecting central apnea in neonates, Physiol. Meas. 33 (1) (2012) 1–17.
[18] B.D. Vergales, A.O. Paget-Brown, H. Lee, L.E. Guin, T.J. Smoot, C.G. Rusin, M.T. Clark, J.B. Delos, K.D. Fairchild, D.E. Lake, R. Moorman, J. Kattwinkel, Accurate automated apnea analysis in preterm infants, Am. J. Perinatol. 31 (2) (2014) 157–162.
[19] M.A. Mohr, B.D. Vergales, H. Lee, M.T. Clark, D.E. Lake, A.C. Mennen, J. Kattwinkel, R.A. Sinkin, J.R. Moorman, K.D. Fairchild, J.B. Delos, Very long apnea events in preterm infants, J. Appl. Physiol. 118 (5) (2015) 558–568.
[20] J.R. Williamson, D.W. Bliss, D.W. Browne, P. Indic, E. Bloch-Salisbury, D. Paydarfar, Using physiological signals to predict apnea in preterm infants, Conf. Rec. Asilomar Conf. Signals Syst. Comput. (2011) 1098–1102.
[21] S.Y. Belal, A.J. Emmerson, P.C.W. Beatty, Automatic detection of apnoea of prematurity, Physiol. Meas. 32 (5) (2011) 523–542.
[22] R.D. Shirwaikar, U.D. Acharya, K. Makkithaya, M. Surulivelrajan, L.E.S. Lewis, Machine learning techniques for neonatal apnea prediction, J. Artif. Intell. 9 (1–3) (2016) 33–38.
[23] N. Mago, S. Srivastava, R.D. Shirwaikar, U.D. Acharya, L.E.S. Lewis, M. Shivakumar, Prediction of Apnea of Prematurity in neonates using Support Vector Machines and Random Forests, in: Proceedings of the 2nd International Conference on Contemporary Computing and Informatics, IC3I 2016, 2016, pp. 693–697.
[24] K. Lim, H. Jiang, A.P. Marshall, B. Salmon, T.J. Gale, P.A. Dargaville, Predicting apnoeic events in preterm infants, Front. Pediatr. 8 (r) (2020) 1–7 Septembe.
[25] R.D. Shirwaikar, D. Acharya, K. Makkithaya, S.M.S. Srivastava, L.E.S. Lewis, Optimizing neural networks for medical data sets: a case study on neonatal apnea prediction, Artif. Intell. Med. 98 (July) (2019) 59–76.
[26] G. Varisco, D. Kommers, X. Long, Z. Zhan, M.M. Nano, W. Cottaar, P. Andriessen, C. van Pul, Optimized detection of central apneas preceding late-onset sepsis in premature infants, Annu Int Conf IEEE Eng Med Biol Soc. 2021 (2021) 5463–5468, doi:10.1109/EMBC46164.2021.9629528.
[27] L. Cabrera-Quiros, D. Kommers, M.K. Wolvers, L. Oosterwijk, N. Arents, J. van der Sluijs-Bens, E.J.E. Cottaar, P. Andriessen, C. van Pul, Prediction of late-onset sepsis in preterm infants using monitoring signals and machine learning, Crit. Care Explor. 3 (1) (2021) e0302.
[28] E.M. Edwards, D.E.Y. Ehret, R.F. Soll, J.D. Horbar, Vermont oxford network: a worldwide learning community, Transl. Pediatr. 8 (3) (2019) 182–192.
[29] M.P. Griffin, J.R. Moorman, Toward the early diagnosis of neonatal sepsis and sepsis-like illness using novel heart rate analysis, Pediatrics 107 (1) (2001) 97–104.
[30] K. Fairchild, M. Mohr, A. Paget-Brown, C. Tabacaru, D. Lake, J. Delos, J.R. Moorman, J. Kattwinkel, Clinical associations of immature breathing in preterm infants: part 1-central apnea, Pediatr. Res. 80 (1) (2016) 21–27.
[31] J.R. Williamson, D.W. Bliss, D.W. Browne, P. Indic, E. Bloch-Salisbury, D. Paydarfar, Individualized apnea prediction in preterm infants using cardio-respiratory and movement signals, in: Proceedings of the *IEEE International Conference on Body Sensor Networks, BSN 2013*, 2013, pp. 5–10.
[32] R. Joshi, D. Kommers, L. Oosterwijk, L. Feijs, C. Van Pul, P. Andriessen, Predicting neonatal sepsis using features of heart rate variability, respiratory characteristics, and ECG-derived estimates of infant motion, IEEE J. Biomed. Health Inform. 24 (3) (2020) 681–692.
[33] S.J. Redmond, C. Heneghan, Cardiorespiratory-based sleep staging in subjects with obstructive sleep apnea, IEEE Trans. Biomed. Eng. 53 (3) (2006) 485–496.
[34] Task Force of The European Society of Cardiology and The North American Society of Pacing and Electrophysiology, Heart rate variability Standards of measurement, physiological interpretation, and clinical use, Eur. Heart J. (17) (1996) 354–381.
[35] F. Shaffer, J.P. Ginsberg, An overview of heart rate variability metrics and norms, Front. Public Health 5 (2017) 1–17 no. September.
[36] B.P. Kovatchev, L.S. Farhy, H. Cao, M.P. Griffin, D.E. Lake, J.R. Moorman, Sample asymmetry analysis of heart rate characteristics with application to neonatal sepsis and systemic inflammatory response syndrome, Pediatr. Res. 54 (6) (2003) 892–898.
[37] J.S. Richman, J.R. Moorman, Physiological time-series analysis using approximate entropy and sample entropy maturity in premature infants Physiological time-series analysis using approximate entropy and sample entropy, Am. J. Physiol. Heart Circ. Physiol. 278 (2000) H2039–H2049.
[38] C.C. Mayer, M. Bachler, M. Hörtenhuber, C. Stocker, A. Holzinger, S. Wassertheurer, Selection of entropy-measure parameters for knowledge discovery in heart rate variability data, BMC Bioinform. 15 (6) (2014) 1–11.
[39] D.R. Kommers, R. Joshi, C. van Pul, L. Atallah, L. Feijs, G. Oei, S. Bambang Oetomo, P. Andriessen, Features of heart rate variability capture regulatory changes during kangaroo care in preterm infants, J. Pediatr. 182 (2017) 92–98 e1.
[40] A. Bauer, J.W. Kantelhardt, A. Bunde, P. Barthel, R. Schneider, M. Malik, G. Schmidt, Phase-rectified signal averaging detects quasi-periodicities in non-stationary data, Phys. A Stat. Mech. Appl. 364 (2006) 423–434.
[41] J.W. Kantelhardt, A. Bauer, A.Y. Schumann, P. Barthel, R. Schneider, M. Malik, G. Schmidt, Phase-rectified signal averaging for the detection of quasi-periodicities and the prediction of cardiovascular risk and the prediction of cardiovascular risk, CHAOS 17 (015112) (2007) 1–9.
[42] R. Joshi, D. Kommers, X. Long, L. Feijs, S. Van Huffel, C. van Pul, P. Andriessen, Cardiorespiratory coupling in preterm infants, J. Appl. Physiol. 126 (1) (2019) 202–213.
[43] P. Indic, E.B. Salisbury, D. Paydarfar, E.N. Brown, R. Barbieri, Interaction between heart rate variability and respiration in preterm infants, Comput. Cardiol. 35 (2008) 57–60.
[44] E. Longin, T. Gerstner, T. Schaible, T. Lenz, S. König, Maturation of the autonomic nervous system: differences in heart rate variability in premature vs. term infants, J. Perinat. Med. 34 (4) (2006) 303–308.
[45] Z. Peng, I. Lorato, X. Long, R.H. Liang, D. Kommers, P. Andriessen, W. Cottaar, S. Stuijk, C. van Pul, Body motion detection in neonates based on motion artifacts in physiological signals from a clinical patient monitor, in: Proceedings of the Proceedings of the *43rd Annual International Conference of the IEEE Engineering in Medicine and Biology Society*, Annu Int Conf IEEE Eng Med Biol Soc. 2021, 2021.
[46] X. Long, J. Foussier, P. Fonseca, R. Haakma, R.M. Aarts, Analyzing respiratory effort amplitude for automated sleep stage classification, Biomed. Signal Process. Control 14 (1) (2014) 197–205.
[47] X. Long, P. Fonseca, R.M. Aarts, R. Haakma, J. Foussier, Modeling cardiorespiratory interaction during human sleep with complex networks, Appl. Phys. Lett. 105 (20) (2014).
[48] M.M. Kabir, H. Dimitri, P. Sanders, R. Antic, E. Nalivaiko, D. Abbott, M. Baumert, Cardiorespiratory phase-coupling is reduced in patients with obstructive sleep apnea, PLoS One 5 (5) (2010) 1–12.
[49] L. Lacasa, B. Luque, F. Ballesteros, J. Luque, J.C. Nuño, From time series to complex networks: The visibility graph, Proc. Natl. Acad. Sci. U. S. A. 105 (13) (2008) 4972–4975.
[50] H. Zou, T. Hastie, Regularization and variable selection via the elastic net, J. R. Stat. Soc. B 67 (2) (2005) 301–320.
[51] G. Varisco, H. van de Mortel, L. Cabrera-Quiros, L. Atallah, D. Hueske-Kraus, X. Long, E.J.E. Cottaar, Z. Zhan, P. Andriessen, C. van Pul, Optimisation of clinical workflow and monitor settings safely reduces alarms in the NICU, Acta Paediatr. Int. J. Paediatr. 110 (4) (2021) 1141–1150.
[52] E.S. Katz, R.B. Mitchell, C.M. D'Ambrosio, Obstructive sleep apnea in infants, Am. J. Respir. Crit. Care Med. 185 (8) (2012) 805–816.
[53] D. Álvarez, R. Hornero, J. Víctor Marcos, F. Delcampo, Multivariate analysis of blood oxygen saturation recordings in obstructive sleep apnea diagnosis, IEEE Trans. Biomed. Eng. 57 (12) (2010) 2816–2824.
