# Supplementary material for: Exploring Computational Techniques in Preprocessing Neonatal Physiological Signals for Detecting Adverse Outcomes: Scoping Review
Source: Interact J Med Res. 2024 Aug 20;13:e46946. doi: 10.2196/46946 (PMC11372324; doi:10.2196/46946)
Supplement: Multimedia Appendix 3 [file ijmr_v13i1e46946_app3.zip › Included Papers - Final/4379/Peng et al. - 2022 - A Continuous Late-Onset Sepsis Prediction Algorith.pdf]

This article has been accepted for publication in IEEE Journal of Biomedical and Health Informatics. This is the author's version which has not been fully edited and content may change prior to final publication. Citation information: DOI 10.1109/JBHI.2022.3216055

A Continuous Late-Onset Sepsis Prediction Algorithm for Preterm Infants using Multi-Channel Physiological Signals from a Patient Monitor

Zheng Peng, Gabriele Varisco, Xi Long, Rong-Hao Liang, Deedee Kommers, Ward Cottaar, Peter Andriessen, and Carola van Pul

***Abstract*— The aim of this study is to develop an explainable late-onset sepsis (LOS) prediction algorithm based on continuously measured multi-channel physiological signals that can be applied to a bedside patient monitor for preterm infants in a neonatal intensive care unit (NICU). The study highlights the complementary predictive value of motion information for LOS prediction when combined with cardiorespiratory information. The algorithm uses features that contain information on heart rate variability (HRV), respiration, and motion, based on continuously measured physiological waveforms including electrocardiogram (ECG) and chest impedance (CI). In this study, 127 preterm infants were included, of whom 59 were blood-culture-proven LOS patients and 68 were control patients. Features in 24 hours before the onset of sepsis (for the LOS group), and an age-matched onset time point (for the control group) were extracted and fed into machine learning classifiers together with gestational age (GA) and birth weight. We compared the prediction performance of several well-known classifiers using features extracted from different signal channels (HRV, respiration, and motion) individually as well as their combinations. The prediction performance was evaluated using the area under the receiver-operating-characteristics curve (AUC). The best performance for LOS prediction was achieved by an XGB classifier combining features from all signal channels, with an AUC of 0.88, a positive predictive value of 0.80, and a negative predictive value of 0.83 during the 6 hours preceding LOS onset. This feasibility study demonstrates the complementary predictive value of motion information in addition to cardiorespiratory information for LOS prediction. Furthermore, visualization of how each feature measured in the individual patient impacts the algorithm decision can strengthen its interpretability. In clinical practice, it is important that clinical interventions are motivated and this visualization method can help to support the clinical decision.**

***Index Terms*— Early warning model, heart rate variability, late-onset sepsis, machine learning, motion, multi-channel physiological signal, preterm infants, predictive monitoring, respiration, sepsis prediction**

This research was supported by grant from e/MTIC.(Corresponding authors: Xi Long and Zheng Peng)

Zheng Peng, Gabriele Varisco and Carola van Pul are with the Department of Applied Physics at Eindhoven University of Technology and the Department of Clinical Physics, Maxima Medical Centre, The Netherlands. (z.peng@tue.nl)

Xi Long is with the Philips Research and the Department of Electrical Engineering at Eindhoven University of Technology, The Netherlands. (xi.long@philips.com)

Rong-Hao Liang is with the Department of Industrial Design and Department of Electrical Engineering at Eindhoven University of Technology, The Netherlands.

Deedee Kommers and Peter Andriessen are with the Department of Applied Physics at Eindhoven University of Technology and the Department of Neonatology, Maxima Medical Centre, The Netherlands.

Ward Cottaar is with the Department of Applied Physics at Eindhoven University of Technology, Eindhoven, The Netherlands.

I. Introduction

NEONATAL sepsis is one of the main causes of neonatal mortality, accounting for 15% of total neonatal deaths worldwide [1]. Neonatal sepsis can be categorized into either early-onset sepsis (EOS) or late-onset sepsis (LOS). EOS usually appears within the first 72 hours of life, representing vertical mother-to-infant transmission, whereas LOS represents infection within the hospital environment or the community and appears at least 72 hours after delivery [2]. For preterm infants admitted in a neonatal intensive care unit (NICU), the mortality rate of LOS can reach 11.3% [3]. These infants are especially vulnerable to LOS due to their immature immune systems and prolonged hospital stays [4]. The detailed differences between EOS and LOS can be found in [5].

Timely diagnosis of LOS and starting antibiotic therapy lower the mortality of LOS [6]. However, the current gold standard for diagnosing LOS is based on the results of blood culture analysis which takes more than 24 hours to obtain results [7]. Therefore, in clinical practice, the antibiotic therapy starts at the moment of blood culture ordering (often denoted as a Cultures, Resuscitation, and Antibiotics Started Here (CRASH) moment), based on the clinical signs of infection, even though these clinical signs may be nonspecific and inconspicuous [2]. Therefore many studies focus on the development of data-driven clinical decision support algorithms for the early detection of neonatal sepsis, as recently overviewed by Persad et al. [8] whose systematic review shows that there are two main types of algorithms for LOS prediction. One type is focusing on laboratory data and observations from electronic medical records (EMRs), usually containing data in lower temporal resolution. The second type is based on continuous

This work is licensed under a Creative Commons Attribution 4.0 License. For more information, see https://creativecommons.org/licenses/by/4.0/

This article has been accepted for publication in IEEE Journal of Biomedical and Health Informatics. This is the author's version which has not been fully edited and content may change prior to final publication. Citation information: DOI 10.1109/JBHI.2022.3216055

signals, using higher temporal resolution data. Many models also include demographic data such as gestational age (GA) and birth weight (BW) to the algorithms [8], [9], since low GA corresponds to immature innate leading to an increased risk for LOS [10]; however, it is challenging to determine unbiased datasets that can be used in the development of prediction algorithms [8]. In EMR-based studies, clinical signs are commonly used in the prediction algorithm, for which particularly lethargy is considered an important clinical sign for LOS prediction [9]. The second type with continuously measured vital signs allows for calculating heart rate variability (HRV) features, which are most often used in LOS prediction, as reviewed by Sullivan et al. [4]. The use of changes in HRV preceding LOS has led to the development of an HRV-based risk score that is shown to rise 24 hours before CRASH [11], [12]. Moreover, continuous HRV monitoring in very low birth weight neonates has shown to be associated with a reduction in sepsis-associated mortality [13], [14]. Later studies also show the added value of other vital signs, such as respiration [4], and cross-correlation between different vitals [15] on the prediction performance, though the evaluation of its effectiveness is still needed [4], [8]. Although lethargy is one important clinical sign associated with LOS, only a few studies report using continuous measurement of body motion to automatically detect lethargy preceding the onset of LOS [16], [17] since there is no accepted standard for measuring and quantifying motion patterns continuously.

Despite these promising findings on neonatal LOS prediction, there are limited algorithms that use multiple vital sign data to improve prediction performance [4]. In this feasibility study, we aim to predict upcoming LOS in preterm infants using features derived from the multiple physiological signals of a patient monitor including HRV (obtained from electrocardiogram or ECG), respiration (measured by chest impedance or CI), and motion (derived from ECG and CI waveforms), as well as patients' demographic data including GA and BW. In particular, for clinical practice, it is relevant to understand which features change and impact the prediction of LOS. Therefore we use an explainable method to visualize the real-time feature impact on the LOS prediction.

II. PATIENTS AND DATA ACQUISITION

The present study included a total of 127 hospitalized preterm infants born before 32 weeks of gestation, admitted to the NICU of the Máxima Medical Center in Veldhoven, the Netherlands, from July 2016 to December 2018. The medical ethical committee of the hospital provided a waiver for this retrospective study under the Dutch law on medical research with humans.

Patients with clinical signs of a generalized infection according to the Vermont Oxford Criteria [18], having treatment with antibiotics after at least 72 hours of life and isolated pathogens from blood culture, were included in the LOS group. To prevent introducing contaminated blood cultures, the C-reactive protein (CRP) level in the identified LOS patients should be greater than or equal to 10 mg/L at least once within 5 days after the clinical onset of LOS [19]. Only the first proven episode of LOS was included in this study for each LOS patient. The control group consisted of preterm infants without clinical suspicion of sepsis and no need for taking any blood culture.

As most features derived from physiological signals in premature infants are known to change with maturation [20], we first defined the CRASH moment [21] for each LOS patient as the 'anchor' point and then searched for one or more control patients with a GA within 3 days younger or older than the LOS patient. For the control patients, we subsequently calculated an 'equivalent CRASH moment' to determine which 24-hour period to analyze in control patients, with a postmenstrual age (PMA) close to the LOS patients.

The ECG (250 Hz) and CI (62.5 Hz) waveforms of all patients were obtained from the routine neonatal patient monitors (Philips IntelliVue MX 800, Philips, Hamburg, Germany). Data were stored in a data server (PIIC-iX, Data Warehouse Connect, Philips Medical Systems, Andover, MA) and retrospectively collected from the data warehouse. The waveforms from 24 hours before to 24 hours after the anchor point were used in this study.

Table I summarizes the characteristics of the studied patients in the full dataset. Even though we used a matching procedure, patients in the LOS group were more premature than in the control group. As immaturity (i.e. low GA and BW) is associated not only with LOS but also with changes in physiological signals [10], [20], we defined a variant of the dataset to reduce the influence of different maturation: a matched subdataset containing only 32 patients in the control group that were precisely matched to 32 sepsis patients. In this subdataset, there was no significant difference in maturation (i.e. GA, BW, and PMA) between two groups. Details of characteristics can be found in our previous study [17]. Unless stated differently, we performed all the following machine learning experiments using both the full dataset and the matched subdataset.

III. METHODOLOGY

The schematic diagram to develop the machine learning algorithm for LOS prediction is shown in Fig. 1. This diagram consists of three blocks including physiological signal processing, feature extraction, and prediction evaluation. Similar to our previous studies [16], [17], we applied a peak detection algorithm to detect R-peaks in ECG waveforms of all patients [22], followed by calculating R-R intervals (RRI), resulting in the HRV signal (i.e. RRI time series). The CI (respiration) waveform was first filtered to remove cardiac artifacts, then the peaks and troughs of the waveform were detected [23]. To estimate the body motion of infants, we developed a continuous motion signal by quantifying motion artifacts in both ECG and CI waveforms. This motion quantification method combines the signal instability index (SII) and the low-frequency motion component based on continuous wavelet transform (CWT) [24] in both ECG and CI waveforms. Specifically, we first implemented the CWT to convert ECG and CI waveforms into scalograms. We set a fixed threshold of beat-to-beat interval, 1.5 s for ECG and 0.4 s for CI. A window size of 6 s was utilized for both signals with

This work is licensed under a Creative Commons Attribution 4.0 License. For more information, see https://creativecommons.org/licenses/by/4.0/

This article has been accepted for publication in IEEE Journal of Biomedical and Health Informatics. This is the author's version which has not been fully edited and content may change prior to final publication. Citation information: DOI 10.1109/JBHI.2022.3216055

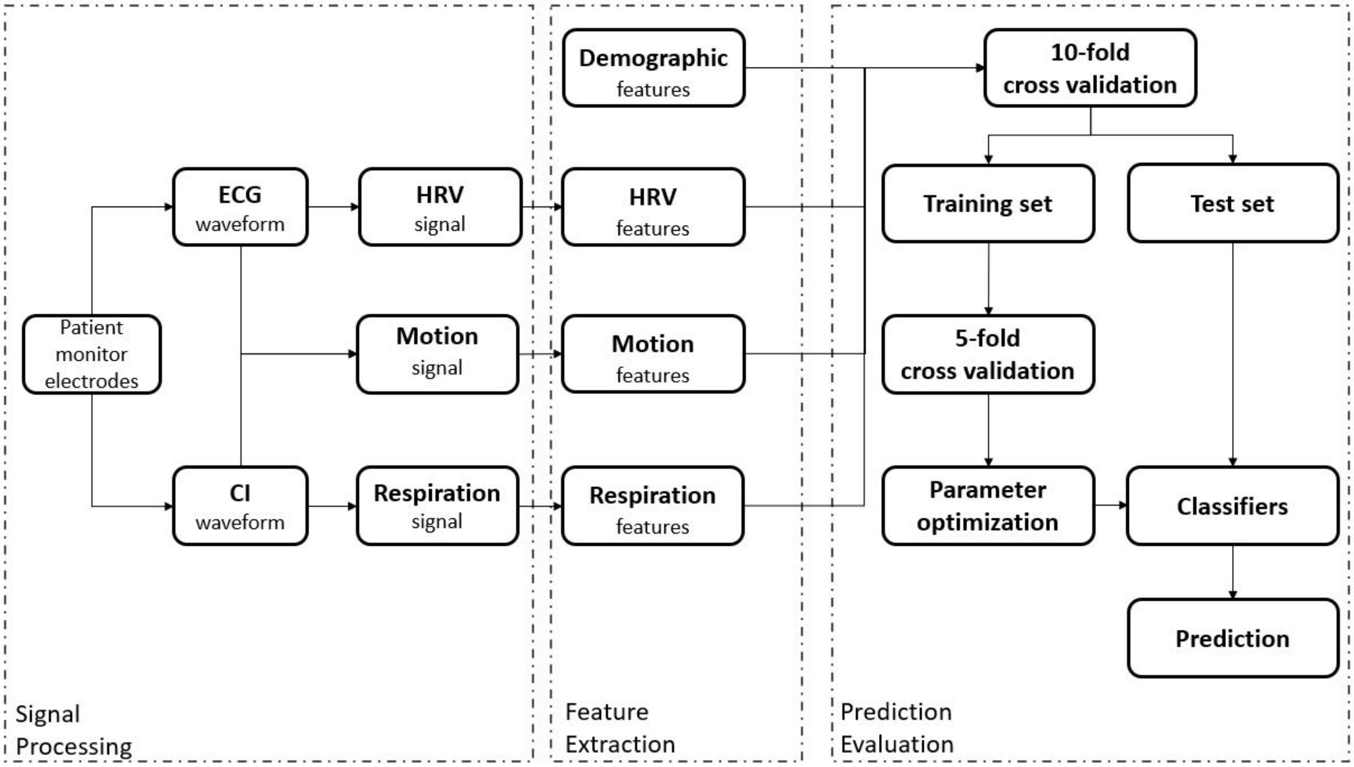

Fig. 1: Schematic diagram of the pipeline for the LOS prediction algorithm development. ECG – electrocardiogram, CI – chest impedance, HRV – heart rate variability

a moving step (sampling period) of 0.4 s. The middle 0.4-second scalogram was averaged along the temporal axis. When the period is greater than the corresponding threshold, the maximum value of the averaged scalogram was taken as the instantaneous motion measure to estimate the intensity of the motion. Then we implemented kernel density estimation which can be interpreted as a smoothed histogram on both waveforms with the same sampling period. The bandwidth of the kernel was extracted as the SII to quantify the motion. Afterwards, the average of both motion measures was taken as our motion signal. This combined motion signal showed better performance than using either single component (SII or CWT) when validating against the annotated videos in our previous study. For details of this method, we refer to our previous work [25].

A. *Feature Extraction*

Each signal was divided into non-overlapping 1-hour segments. The segments prior to CRASH in the LOS group and equivalent CRASH moment in the control group were labeled as LOS (labeled as 1) and control (labeled as 0) segments, respectively. We first calculated features from the entire 1-hour segment. In addition, to characterize physiological changes for a shorter period, we then split each segment into periods of 5 minutes and computed the same features for each 5-minute subsegment. The mean, standard deviation (SD), maximum, and minimum of the features from all the subsegments were computed as 'statistical' features for each 1-hour segment.

We considered four categories of features including HRV features (from HRV signals), respiration features (from respiration signals), motion features (from estimated motion signals), and demographic features. The features in each category are introduced in the following.

1) HRV features: HRV features were extracted from RRI time series. Features in time domain were computed including the mean and SD of the RRIs (RRM and SDNN), the root mean square of successive RRI differences (RMSSD) [26], the percentage of deceleration (pDec), the SD of RRIs that contribute to pDec (SDDec) [27], the sample asymmetry (SampAsy) [28], and the average acceleration and deceleration response (AAR and ADR) [29]. We also extracted frequency-domain features by first resampling RRI time series to 4 Hz and then calculating normalized low frequency power (LFnu) within a frequency range of 0.02-0.2 Hz, normalized high frequency power (HFnu) ranging from 0.2 to 2 Hz, and their ratio (LF/HF). Approximate entropy (ApEn) of RRIs (RRApEn) was also extracted to measure the regularity of heart rate changes [30].

2) Respiration features: The respiratory rate and inspiration to expiration ratio (IER) time series were calculated from the preprocessed respiration signals. We extracted features from respiratory rate including median, interdecile range, skewness, kurtosis, and ApEn (denoted by RespMED, RespIDR, RespSkew, RespKurt, and RespApEn, respectively) [16]. Skewness is a measure of the asymmetry of signal values around the mean in terms of their distribution. For instance, apnea and/or bradypnea can result in a long left tail in respiratory

This work is licensed under a Creative Commons Attribution 4.0 License. For more information, see https://creativecommons.org/licenses/by/4.0/

This article has been accepted for publication in IEEE Journal of Biomedical and Health Informatics. This is the author's version which has not been fully edited and content may change prior to final publication. Citation information: DOI 10.1109/JBHI.2022.3216055

TABLE I: Characteristics of the patient population

	LOS Group ($n = 59$)	Control Group ($n = 68$)	
Gestational age (weeks)	27.86 (26.86-29.29)	29.43 (28.71-30.71)	$p < 0.01$
Postnatal age (days)	6.95 (5.04-10.05)	5.17 (3.61-6.77)	$p < 0.01$
Postmenstrual age (weeks)	29.07 (27.96-30.26)	30.12 (29.60-31.57)	$p < 0.01$
Birth weight (g)	1075 (870-1285)	1268 (1075-1409)	$p < 0.01$
Gender (female)	26 (44%)	39 (57%)	NS
Singleton	42 (71%)	46 (68%)	NS
Intrapartum maternal antibiotic treatment	19 (33%)	21 (31%)	NS
Antenatal corticosteroids	54 (92%)	56 (82%)	NS
Cesarean section	39 (66%)	41 (60%)	NS
Umbilical artery PH	7.28 (7.04-7.33)	7.29 (7.13-7.33)	NS
Apgar score 5 min	7 (6-8)	8 (7-8)	NS
Surfactant replacement therapy	33 (56%)	32 (47%)	NS
Patent ductus arteriosus (ibuprofen)	26 (44%)	9 (13%)	$p < 0.01$
Patients with a central line before CRASH	28 (47%)	6 (9%)	$p < 0.01$
Spontaneous ventilation before CRASH	2 (3%)	0 (0%)	NS
Continuous positive airway pressure before CRASH	41 (69%)	37 (54%)	NS
In-hospital mortality	8 (14%)	2 (3%)	NS

Results are presented as median (interquartile range) or the number of cases (corresponding percentage). The Mann-Whitney U test (for continuous measurements) and the Chi-squared test (for nominal measurements) were used to examine the significance of difference between the two groups. NS: Not Significant ($p > 0.05$)

rate distribution, which can be reflected by a negative skewness value of the distribution. Kurtosis is a measure of the shape distortion compared to a Gaussian distribution of the signal. For instance, a positive kurtosis indicates more respiration rate values located around the mean rather than in the tails of the distribution. Additionally, we also extracted features by computing the mean and SD of the IER (RespIERM and RespIERSD) [31].

3) Motion features: Motion features were developed for this study from the continuous motion signal to capture the motion pattern of preterm infants before LOS. We computed the median, interdecile range, skewness, and kurtosis of the motion signal (denoted by MotMED, MotIDR, MotSkew, and MotKurt, respectively) to characterize body motion. Low values of motion interdecile range (MotIDR) and large positive values of motion skewness (MotSkew) can represent a lack of motion (i.e., lethargy). The ApEn of the motion signal (MotApEn) was also computed to evaluate the repetitive motion pattern of infants. We empirically set the embedded dimension of 3 and the tolerance of 0.3 to calculate this feature. In addition, the cumulative time of body motion (MotDur) and the number of occurrences of body motion (MotFreq) were designed to represent the duration and frequency of body motion of preterm infants. In order not to include small motion from the head or limbs, we empirically set 3 as the threshold for the motion signal to define the presence of body motion, as shown to be an adequate value [25].

4) Demographic features: GA and BW are known to be important risk factors for neonatal sepsis and can represent the physiological maturity at birth of preterm infants [10].

Features based on 1-hour segments and the corresponding statistical features (i.e., mean, SD, maximum, and minimum) from all 5-minute subsegments from the same segment, lead to a total of 60 HRV, 35 respiration, and 35 motion features. All the features and their descriptions are summarized in Table II.

B. LOS Prediction Evaluation

In this study, we first compared several commonly used classifiers with different classification mechanisms, including extreme gradient boosting (XGB), k-nearest neighbors (KNN), logistic regression (LR), and support vector machine (SVM). These classifiers have been used in previous studies for LOS prediction and are generally accepted [8]. XGB is an implementation of gradient boosted decision trees with parallel computation and additional regularization. KNN is a classifier that stores all available samples and classifies new samples based on a similarity measure. LR allows quantifying the relationship between features and predictions. SVM can perform effective non-linear classification by mapping the input features into high-dimensional feature spaces and is known for its high generalization ability and robust performance [32].

Next, to evaluate the predictive value of different signal channels or their combinations, we considered 5 feature sets for comparison, 1) HRV features, 2) respiration features, 3) motion features, 4) the combination of HRV and respiration features, and 5) the combination of all features. Note that the demographic features (i.e., GA and BW) were always included in different feature sets. As shown in Fig.1, we used 10-fold cross validation, which for the full dataset with 127 patients meant leaving 12 or 13 patients out in each iteration of cross validation, to split the feature set into training and test sets. For the matched subdataset of 64 patients, 6 to 7 patients were left in each test set of each iteration. Within each training set, patient-independent 5-fold cross validation was applied to optimize the window length (number of hours before CRASH) and other hyperparameters used for modeling and prediction. Here we used the Bayesian optimization for hyperparameter tuning and used grid search to select the optimal window length, starting from -24 hours to the CRASH moment with a step size of 1 hour, to maximize the performance of LOS prediction in training sets. Afterwards, the classifier trained with the optimized parameters was tested on the test set during each iteration of cross validation. As illustrated in the study by Leon et al [30], the 6 hours prior to CRASH contain the most predictive information of LOS, we also used the AUC of prediction during 6 hours prior to CRASH to determine the best performing classifiers for the following analyses.

Finally, the best performing classifiers (i.e. greatest AUC during 6 hours before CRASH) in the test sets were used to analyze the hourly performance before CRASH. We calculated hourly AUC values for each feature set by combining

This work is licensed under a Creative Commons Attribution 4.0 License. For more information, see https://creativecommons.org/licenses/by/4.0/

This article has been accepted for publication in IEEE Journal of Biomedical and Health Informatics. This is the author's version which has not been fully edited and content may change prior to final publication. Citation information: DOI 10.1109/JBHI.2022.3216055

TABLE II: DESCRIPTION OF FEATURES

1-hour features	5-min statistic features	Description
HRV features		
RRM	RRM_{mean}, RRM_{SD}, RRM_{max}, RRM_{min}	Mean of RRI [26]
SDNN	$SDNN_{mean}$, $SDNN_{SD}$, $SDNN_{max}$, $SDNN_{min}$	Standard deviation of RRI [26]
RMSSD	$RMSSD_{mean}$, $RMSSD_{SD}$, $RMSSD_{max}$, $RMSSD_{min}$	Root mean square of successive RRI differences [26]
pDec	$pDec_{mean}$, $pDec_{SD}$, $pDec_{max}$, $pDec_{min}$	Percentage deceleration of RRI, defined as the percentage of RRI larger than the moving average RRI of the past 5 minutes [27]
SDDec	$SDDec_{mean}$, $SDDec_{SD}$, $SDDec_{max}$, $SDDec_{min}$	Standard deviation of RRI corresponding to pDec [27]
AAR	AAR_{mean}, AAR_{SD}, AAR_{max}, AAR_{min}	Average acceleration response [29]
ADR	ADR_{mean}, ADR_{SD}, ADR_{max}, ADR_{min}	Average deceleration response [29]
SampAsy	$SampAsy_{mean}$, $SampAsy_{SD}$, $SampAsy_{max}$, $SampAsy_{min}$	Sample asymmetry [28]
HFnu	$HFnu_{mean}$, $HFnu_{SD}$, $HFnu_{max}$, $HFnu_{min}$	Normalized high frequency power (0.2-2Hz) [30]
LFnu	$LFnu_{mean}$, $LFnu_{SD}$, $LFnu_{max}$, $LFnu_{min}$	Normalized low frequency power (0.02-0.2Hz) [30]
RRApEn	$RRApEn_{mean}$, $RRApEn_{SD}$, $RRApEn_{max}$, $RRApEn_{min}$	Approximate entropy of RRI [30]
Respiration features		
RespMED	$RespMED_{mean}$, $RespMED_{SD}$, $RespMED_{max}$, $RespMED_{min}$	Median of respiratory rate
RespIDR	$RespIDR_{mean}$, $RespIDR_{SD}$, $RespIDR_{max}$, $RespIDR_{min}$	Interdecile range of respiratory rate, defined as the difference between the 90th and 10th percentile of the respiratory rate [16]
RespSkew	$RespSkew_{mean}$, $RespSkew_{SD}$, $RespSkew_{max}$, $RespSkew_{min}$	Skewness of respiratory rate [16]
RespKurt	$RespKurt_{mean}$, $RespKurt_{SD}$, $RespKurt_{max}$, $RespKurt_{min}$	Kurtosis of respiratory rate
RespApEn	$RespApEn_{mean}$, $RespApEn_{SD}$, $RespApEn_{max}$, $RespApEn_{min}$	Approximate entropy of respiratory rate [16]
RespIERM	$RespIERM_{mean}$, $RespIERM_{SD}$, $RespIERM_{max}$, $RespIERM_{min}$	Mean of inspiration to expiration ratio (IER) [31]
RespIERSD	$RespIERSD_{mean}$, $RespIERSD_{SD}$, $RespIERSD_{max}$, $RespIERSD_{min}$	Standard deviation of IER [31]
Motion features		
MotMED	$MotMED_{mean}$, $MotMED_{SD}$, $MotMED_{max}$, $MotMED_{min}$	Median of motion signal
MotIDR	$MotIDR_{mean}$, $MotIDR_{SD}$, $MotIDR_{max}$, $MotIDR_{min}$	Interdecile range of motion signal, defined as the difference between the 90th and 10th percentile of motion signal [16]
MotSkew	$MotSkew_{mean}$, $MotSkew_{SD}$, $MotSkew_{max}$, $MotSkew_{min}$	Skewness of motion signal [16]
MotKurt	$MotKurt_{mean}$, $MotKurt_{SD}$, $MotKurt_{max}$, $MotKurt_{min}$	Kurtosis of motion signal
MotApEn	$MotApEn_{mean}$, $MotApEn_{SD}$, $MotApEn_{max}$, $MotApEn_{min}$	Approximate entropy of motion signal
MotDur	$MotDur_{mean}$, $MotDur_{SD}$, $MotDur_{max}$, $MotDur_{min}$	Duration of body motion, defined as the cumulative time of motion signal stayed above the threshold
MotFreq	$MotFreq_{mean}$, $MotFreq_{SD}$, $MotFreq_{max}$, $MotFreq_{min}$	Frequency of body motion, defined as the number of occurrences the motion signal up crossed the threshold
Demographic features		
GA		Gestational age
BW		Birth weight

the predictions in test sets for all patients at each hour before CRASH to analyze the 'time-to-CRASH' prediction performance. We then displayed the predicted probability per hour by calculating the average and standard deviation of the predicted probability in test sets for all patients at each hour before CRASH. Visualizing the probability over time can provide clinicians an early warning tool for clinical decision making.

C. Feature importance

It is essential to understand how a model makes an individual prediction in the applications of clinical decision support algorithms since the consequences are for individual patients. Typically, simple models (e.g. linear models) are preferred because of their ease of interpretation; however, there is often a trade-off between the performance and interpretability of a model on the data in clinical practice. Furthermore, the popular feature attribution methods embedded in classifiers (e.g. gain and split count for tree ensembles) are inconsistent [33], making a meaningful comparison of attribution values across features difficult. One of the recent advances for explanation models to estimate feature importance is the Shapley additive explanations (SHAP) [34], a local interpretability technique based on Shapley Value. Several studies have started to take advantage of SHAP to interpret clinical decision support algorithms [35], [36]. SHAP assigns each feature an impact value for an individual prediction, showing the positive or negative relationship for each feature with the prediction. A positive SHAP value or a negative SHAP value indicates the feature pushes prediction more positive (LOS) or negative (control), respectively. A SHAP value of zero represents no impact on the prediction. As an approach to allocating feature importance, SHAP is consistent, accurate, and more in line with human intuition [34], [36]. In this study, we utilized SHAP to estimate the feature importance of the best-performing classifiers to gain more insight into the decision-

This work is licensed under a Creative Commons Attribution 4.0 License. For more information, see https://creativecommons.org/licenses/by/4.0/

This article has been accepted for publication in IEEE Journal of Biomedical and Health Informatics. This is the author's version which has not been fully edited and content may change prior to final publication. Citation information: DOI 10.1109/JBHI.2022.3216055

making process and how the predictions are impacted by each feature of each instance. In addition, we further propose to make the model explainable by visualizing the individualized feature attribution of SHAP. In this visualization, we display the predicted probability and SHAP values of top-10 features evolving over time for the predictions of individual patients as an example for the real-time application in the NICU.

IV. RESULTS

A. Feature Time Series Analysis

To show the changes in features before and after the CRASH moment, we present the time series of 6 features for the 59 LOS and 68 control infants (full dataset) as shown in Fig. 2. Regarding the features of HRV, the pDec decreases significantly in LOS infants in comparison with control infants prior to CRASH. Similarly, HFnu also drops but to a less extent. Regarding respiration features, both RespIDR and RespApEn in the LOS group increase a few hours before CRASH (e.g., -3 to -1 hour). For motion features, higher MotMED values can be observed before CRASH in the LOS group, reflecting more subtle body motion. The MotApEn values in LOS infants are also much higher than those in control infants during almost the entire 24 hours before CRASH, indicating the presence of a more irregular motion pattern in LOS patients.

B. LOS Prediction Performance

Table III presents the prediction performance of compared classifiers using different feature sets in the full dataset and the matched subdataset. XGB does not necessarily perform best when individual feature sets are used, but when using the combination of all features, it stands out in both datasets for predicting LOS during the 6 hours before CRASH, with a mean (SD) AUC of 0.88 (0.09) and 0.85 (0.07) respectively. By including the motion features the performances of most classifiers increase. Using multi-channel features outperforms using single-channel features alone (HRV, respiration, or motion). This indicates that the three physiological signals can be used individually but are also complementary with respect to predictive value for LOS prediction. In general, the performance using all features outperforms that of cardiorespiratory features and each single-channel features. It can be observed that the matched subset, which is smaller but is exactly matched on GA and PMA on a subject-to-subject basis, performs worse than the full dataset (likely due to the smaller number of patients in the training set), but the added value of the motion features is still visible. When using all features, there is an even larger improvement in performance. As mentioned before, from this point on, the best performing classifier (XGB) is used for all following analyses. The hyperparameters we chose to tune in the training process for XGB are the maximum depth of a tree, learning rate, number of estimators, and the minimum sum of instance weight needed in a child.

Table IV reports the XGB performance in LOS prediction in both datasets including sensitivity, specificity, positive predictive value (PPV), negative predictive value (NPV), accuracy, and AUC. The metrics were calculated by the classifier's decision-making threshold of 0.5. It can be seen that the model using features from all physiological signals achieves the highest values in all metrics in each dataset. The metrics in the full dataset outperform those in the matched subdataset.

Fig. 3 shows, for different feature sets in both datasets, how the performance of XGB in LOS prediction changes over time from -24 hours to CRASH. A clear upward trend starting from 12 hours before CRASH can be observed for all feature sets, the trend reaches the peak at -1 hour in the full dataset and at -2 hour in the matched subdataset. In particular, the AUC obtained using only motion features starts increasing consistently from -14 hours, earlier than that using other feature sets, and reaches the maximum value at -6 hours in both datasets. Combining all features leads to the highest AUC of 0.93 at the time of 1 and 2 hours before CRASH in full and matched subdataset, respectively.

Fig. 4 shows the XGB predicted probability of LOS (mean and SD over patients) using all features in the range of -24 hours to CRASH in both datasets. It can be seen in the full dataset that on average the model can separate LOS from the control group, the trend of the prediction probability in both groups is similar for both datasets, but the distinction between two groups is less when the time is far from CRASH in the matched subdataset. As expected, for both datasets, the overlapping area of SDs of the two groups is large when the time is far from CRASH. The predicted probability for control infants does not change over time, whereas that for LOS infants increases after around -12 hours. This change indicates more predictive information of LOS is obtained by the model when the time is closer to CRASH. Additionally, the SD of predicted probability at each hour is large, in most hours the 'lower boundary' (mean - SD) for control patients can even reach zero. Note that the predicted probability is different from the infection probability of LOS but assumed to be highly correlated with it.

C. Feature Importance

To interpret how features impact the predictions of the model in both datasets, the feature importance (top 10) of the XGB model using all features is presented and compared in Fig. 5. It is defined as the average absolute SHAP value (in square bracket) over all predictions, indicating the 'impact on model output' of a feature. The scatter-dense plots show the SHAP value distributions of the features. It can be seen in the full dataset, on average, GA has the largest impact on the prediction. A low GA (blue) pushes the prediction toward LOS while a high GA (red) pushes the prediction toward the control class. As expected, the GA disappears in the top 10 list of the matched subdataset because of the precise matching. MotIDR_{mean} (mean of 5-min motion interdecile range) and MotMED_{min} (minimum of 5-min motion median) stand out for both datasets, showing the low MotIDR_{mean} and high MotMED_{min} lead the predictions toward LOS. In addition, in both datasets, more top-ranked features (7 out of 10) are features extracted from 5-min subsegments. It is also important to note that the list includes features obtained from all signal channels (HRV, respiration, and motion), showing

This work is licensed under a Creative Commons Attribution 4.0 License. For more information, see https://creativecommons.org/licenses/by/4.0/

This article has been accepted for publication in IEEE Journal of Biomedical and Health Informatics. This is the author's version which has not been fully edited and content may change prior to final publication. Citation information: DOI 10.1109/JBHI.2022.3216055

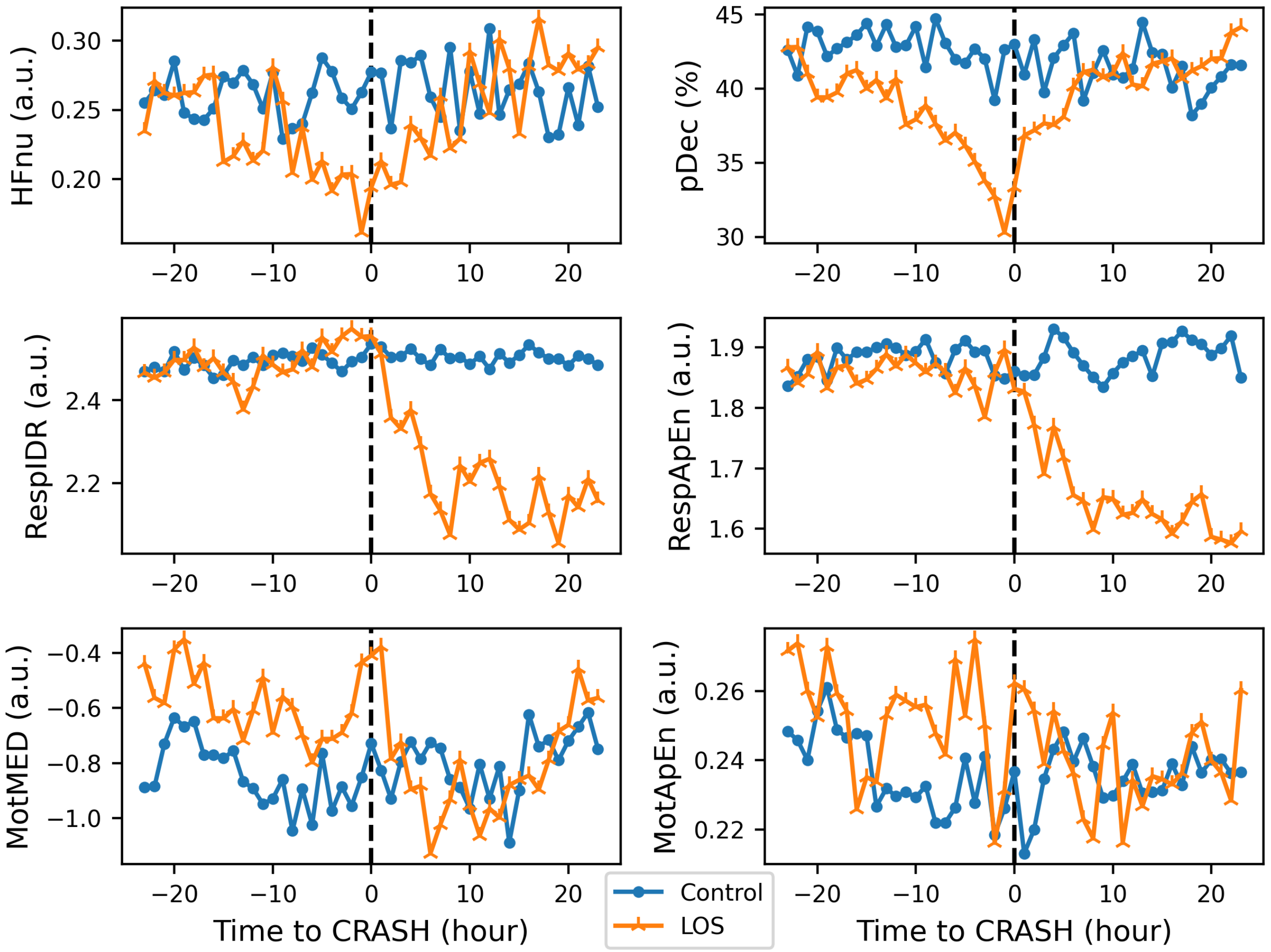

Fig. 2: Feature time series for HRV (top), respiration (middle), and motion (bottom) over 24 hours before and after CRASH. For each hour, the mean value over all patients in each group is presented.

TABLE III: LOS PREDICTION RESULTS (AUC) USING DIFFERENT CLASSIFIERS AND FEATURE SETS IN BOTH DATASETS DURING 6 HOURS BEFORE CRASH

	Classifier	HRV	Respiration	Motion	HRV+Respiration	HRV+Respiration+Motion
Full dataset ($n = 127$)	KNN	0.72 (0.12)	0.79 (0.08)	0.73 (0.09)	0.76 (0.11)	0.77 (0.08)
	LR	0.77 (0.09)	0.74 (0.10)	0.77 (0.12)	0.76 (0.08)	0.80 (0.06)
	SVM	0.79 (0.10)	0.83 (0.09)	0.80 (0.10)	0.84 (0.09)	0.84 (0.09)
	XGB	0.82 (0.09)	0.78 (0.11)	0.81 (0.08)	0.85 (0.11)	**0.88 (0.09)**
Matched subdataset ($n = 64$)	KNN	0.71 (0.12)	0.68 (0.08)	0.73 (0.12)	0.77 (0.11)	0.74 (0.11)
	LR	0.77 (0.08)	0.69 (0.12)	0.68 (0.14)	0.77 (0.07)	0.83 (0.08)
	SVM	0.76 (0.12)	0.74 (0.12)	0.71(0.16)	0.82 (0.10)	0.84 (0.08)
	XGB	0.67 (0.16)	0.71 (0.07)	0.79 (0.12)	0.74 (0.12)	**0.85 (0.07)**

Results are presented as mean (SD). The best results for full and matched dataset are indicated in bold.

that the three physiological signal channels are complementary for LOS prediction. Fig. 6 illustrates the boxplots of SHAP values and the Mann-Whitney U test of feature values for the top 10 features in both datasets. It can be seen the distribution of SHAP values of the top 10 features in both groups can be split well. Most top 10 features are significantly different in both groups.

Fig. 7. displays examples of real-time predictions for two individual patients, one per group in both datasets. It contains the predicted probability and the corresponding explained top features evolving over time, which allows clinicians to better interpret the model. It can be observed that in the matched subdataset, the predicted probability of this particular LOS patient becomes septic from -6 hours as a result of decreased pDec$_{mean}$, increased RespIDR, and decreased MotIDR$_{mean}$. The control patient was wrongly predicted as LOS at -8 hour mainly because of the sharp decrease of MotIDR$_{mean}$ which can represent lethargy.

This work is licensed under a Creative Commons Attribution 4.0 License. For more information, see https://creativecommons.org/licenses/by/4.0/

This article has been accepted for publication in IEEE Journal of Biomedical and Health Informatics. This is the author's version which has not been fully edited and content may change prior to final publication. Citation information: DOI 10.1109/JBHI.2022.3216055

TABLE IV: METRICS OF PREDICTION IN BOTH DATASETS DURING 6 HOURS BEFORE CRASH FOR XGB

	Feature set	Sensitivity	Specificity	Positive Predictive Value(PPV)	Negative Predictive Value(NPV)	Accuracy	AUC
Full dataset ($n = 127$)	HRV	0.70 (0.15)	0.80 (0.14)	0.77 (0.09)	0.77 (0.07)	0.76 (0.06)	0.82 (0.09)
	Respiration	0.68 (0.17)	0.79 (0.13)	0.74 (0.14)	0.75 (0.11)	0.74 (0.11)	0.78 (0.11)
	Motion	0.68 (0.12)	0.75 (0.14)	0.73 (0.13)	0.74 (0.06)	0.72 (0.07)	0.81 (0.08)
	HRV+Respiration	0.75 (0.16)	0.81 (0.13)	0.79 (0.11)	0.80 (0.12)	0.78 (0.11)	0.85 (0.11)
	HRV+Respiration+Motion	**0.79 (0.15)**	**0.82 (0.10)**	**0.80 (0.08)**	**0.83 (0.10)**	**0.81 (0.09)**	**0.88 (0.09)**
Matched subdataset ($n = 64$)	HRV	0.63 (0.24)	0.68 (0.13)	0.67 (0.13)	0.66 (0.16)	0.65 (0.13)	0.67 (0.16)
	Respiration	0.66 (0.13)	0.66 (0.16)	0.69 (0.10)	0.66 (0.08)	0.66 (0.07)	0.71 (0.07)
	Motion	0.70 (0.17)	0.73 (0.10)	0.72 (0.11)	0.72 (0.12)	0.72 (0.11)	0.79 (0.12)
	HRV+Respiration	0.66 (0.17)	0.70 (0.09)	0.69 (0.10)	0.68 (0.12)	0.68 (0.10)	0.74 (0.12)
	HRV+Respiration+Motion	**0.72 (0.19)**	**0.81 (0.09)**	**0.80 (0.07)**	**0.75 (0.12)**	**0.76 (0.08)**	**0.85 (0.07)**

Results are presented as mean (SD). The best results for full and matched dataset are indicated in bold.

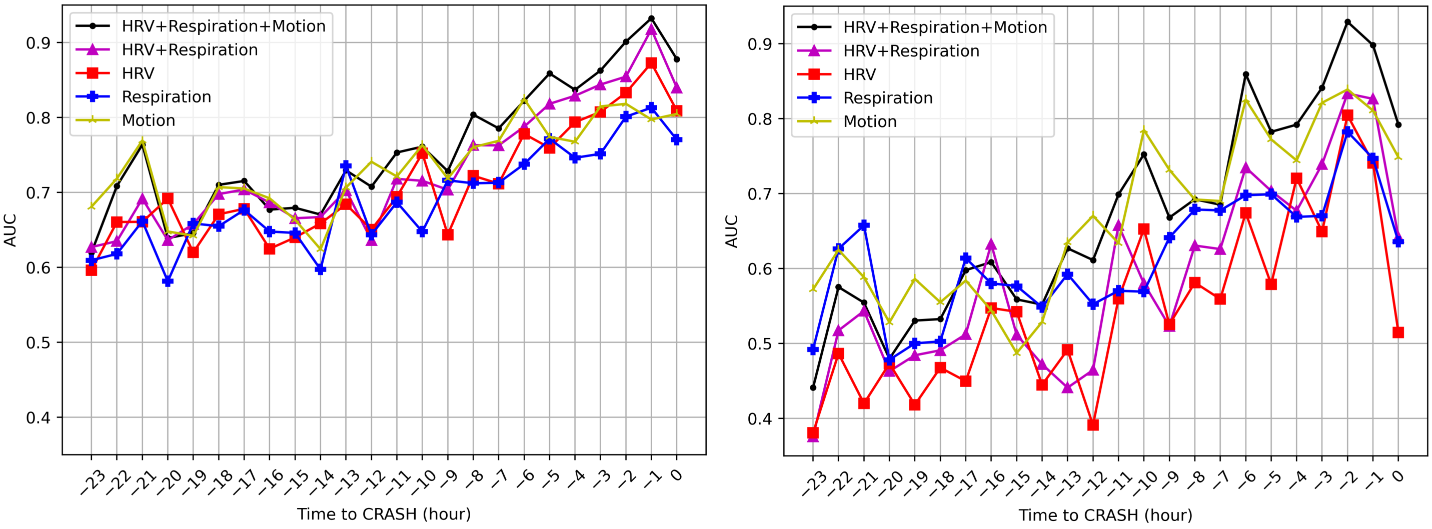

(a) Full dataset

(b) Matched subdataset

Fig. 3: Comparison of LOS prediction performance (AUC) versus time to CRASH using different feature sets extracted from different signal channels or combinations in both datasets

V. DISCUSSION

The results of this study indicate that an upcoming LOS can be predicted earlier than in current clinical routine by machine learning classifiers using multi-channel physiological signals obtained from a patient monitor, potentially providing an extended therapeutic period for clinical intervention for LOS patients. We show that the inclusion of the derived motion features can provide complementary predictive value to cardiorespiratory features for LOS prediction.

By analyzing features extracted from HRV, respiration, and derived motion signals, we confirm previous findings that the features extracted from physiological signals change over time several hours preceding the onset of LOS [16], [30]. In particular, the changes of motion features (e.g. MotMED and MotApEn) start earlier than that of cardiorespiratory features (Fig. 2), improving prediction performance for LOS when time is far from CRASH (Fig. 3). Although several clinical studies have demonstrated that the lack of spontaneous motion (i.e., lethargy) is a valuable clinical sign that can be used to predict LOS, they did neither quantify nor continuously measure body motion [12], [37]. In our previous study [17], the motion was continuously estimated based on the SII of ECG, a feature that changed before the CRASH moment. We now improved the motion estimation method by combining SII and the low-frequency component of ECG and CI, showing that the motion features in themselves can perform similarly to HRV features for LOS prediction. This may be an effect of the extracted low-frequency component of physiological signals, which is not only related to motion but also the information of ECG and CI (e.g. bradycardia and apnea). Hence, independent motion measurement is needed to further analyze the predictive value of continuous motion signals for LOS prediction.

In this study, we observed that motion features, which are available from continuous monitoring, are among the top-ranking features and add complementary information to conventional cardiorespiratory features for LOS prediction. Besides, the top-ranked motion features (Fig. 5) reveal notable findings. For instance, a higher median of the motion signal (MotMED_{min} - minimum of 5-min motion median) and a lower range in this signal (MotIDR_{mean} - mean of 5-min

This work is licensed under a Creative Commons Attribution 4.0 License. For more information, see https://creativecommons.org/licenses/by/4.0/

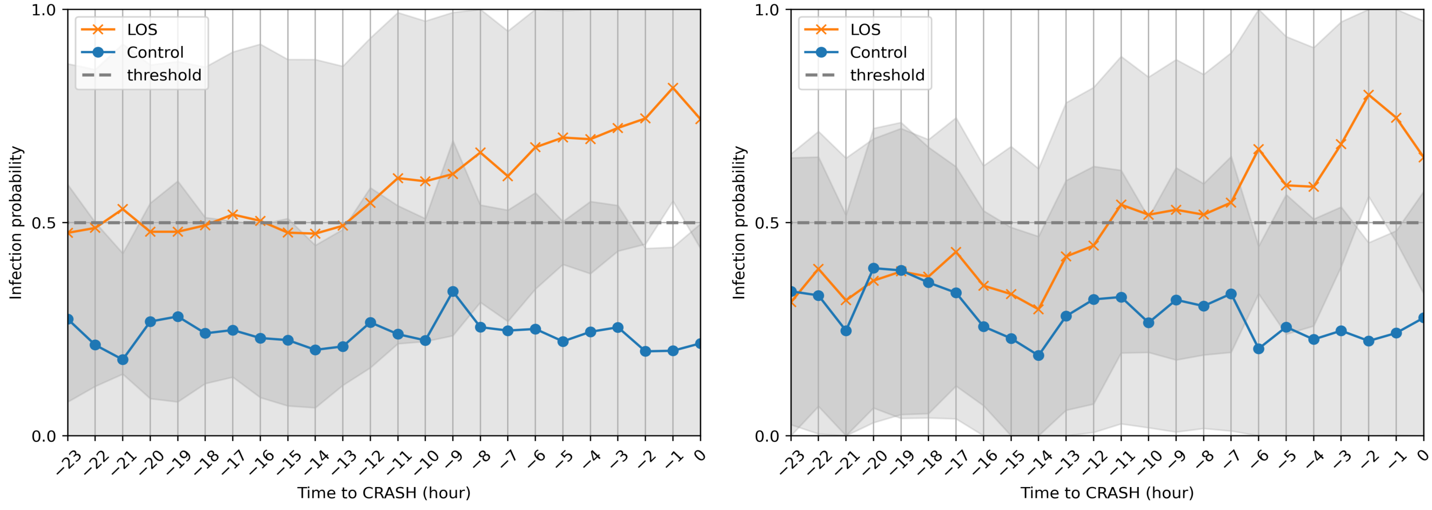

(a) Full dataset

(b) Matched subdataset

Fig. 4: Prediction probability over time before CRASH for both LOS and control groups in both datasets. The values in lines are the average of probabilities, the shadow around lines means the standard deviation of the predicted probabilities. The dashed line represents an example of the decision-making threshold of 0.5.

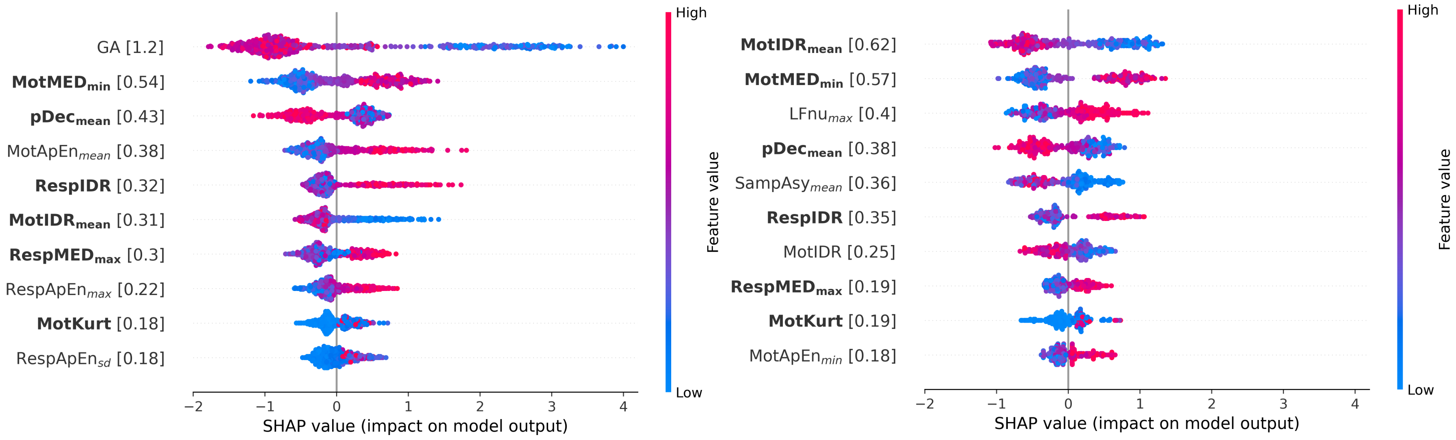

(a) Full dataset

(b) Matched subdataset

Fig. 5: Feature importance (top 10) in both datasets based on SHAP analysis. For each feature, the value in the square bracket represents the mean absolute SHAP value. The color bar represents the feature values. The features that appear in both models are presented in bold

motion interdecile range) may suggest that more subtle motion but less gross motion is indicative of upcoming sepsis in LOS patients. A higher MotApEn$_{mean}$ (mean of 5-min motion approximate entropy) is associated with more irregular changes of motion patterns preceding CRASH in LOS infants. A higher MotKurt (kurtosis of motion) in LOS infants could be due to the presence of sepsis-associated lethargy.

Even though many algorithms show potential for early detection of sepsis, the comparison of prediction performance is still difficult because of the limited standardization [8], [9]. The heterogeneous datasets, varying sepsis definitions, and different reported metrics complicate the performance comparison of LOS prediction algorithms. Nevertheless, we compared our prediction performance to several comparable studies. For instance, a comparison between the results in our full dataset and results from a study by Leon et al. [30], whose dataset also contained significantly different maturation between two groups and who used the same metric (AUC during 6 hours before CRASH) for performance evaluation shows a similar performance (AUC of 0.88). They showed added predictive value of more advanced HRV features, indicating further information can be explored from the physiological signals to improve the prediction performance. We also compared the prediction performance of the refined algorithm to the prediction performance in our previous study when used on the same data, the subdataset of age-matched infants [17]. Our new algorithm outperforms the previous algorithm at every hour shown before CRASH, indicating the improvement of motion signals and the developed pipeline for LOS prediction. Interestingly, we can observe that the performance at the last

This work is licensed under a Creative Commons Attribution 4.0 License. For more information, see https://creativecommons.org/licenses/by/4.0/

This article has been accepted for publication in IEEE Journal of Biomedical and Health Informatics. This is the author's version which has not been fully edited and content may change prior to final publication. Citation information: DOI 10.1109/JBHI.2022.3216055

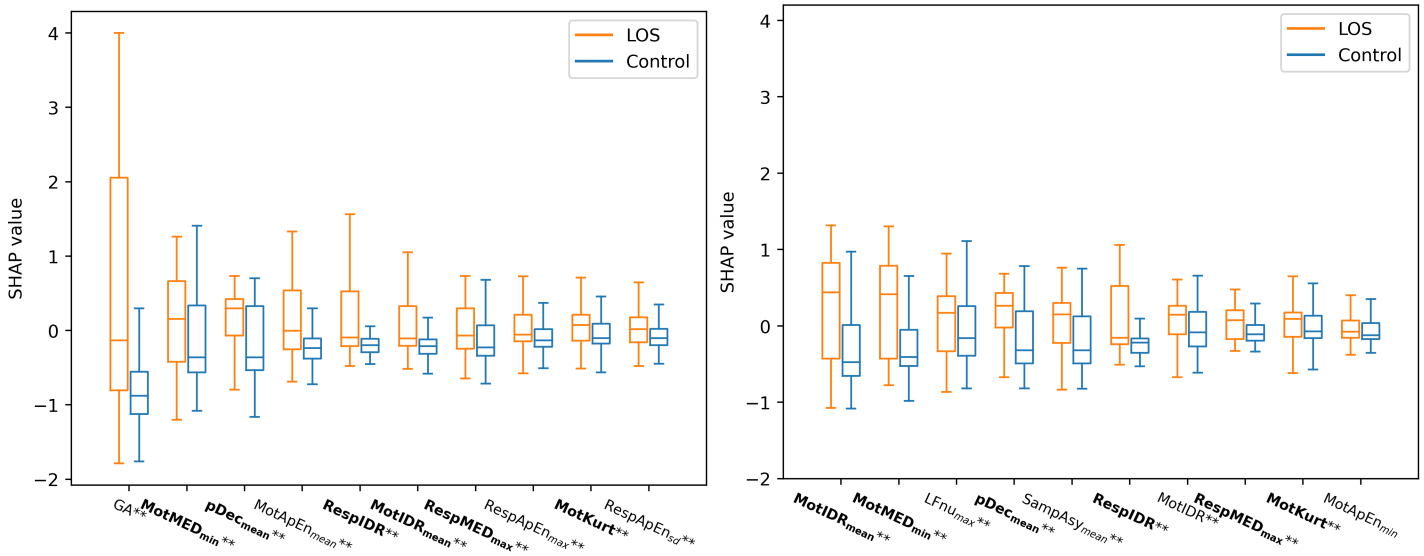

(a) Full dataset

(b) Matched subdataset

Fig. 6: Boxplots of SHAP value for top features in both datasets. * and ** correspond to a significant difference in corresponding feature values between LOS and control groups, with $p < 0.05$ and $p < 0.01$, respectively. The features that appear in both models are presented in bold

hour before CRASH dropped significantly. This might likely be caused by the nurse handling due to the increasing number of critical alarms (e.g. desaturation, bradycardia, and apnea) associated with sepsis. The measured signals therefore became less reliable because of this external disturbance at the last hour before CRASH, which would likely lead to decreased prediction performance.

The method could be deployed for continuous monitoring in a NICU setting, as it uses commonly available vital signs from a routine patient monitor. Moreover, unlike other methods which need an observation period (e.g. first 48 hours) as a baseline reference for each patient [30], our new method can be applied without this observation period. This is particularly useful for newly admitted patients in NICU. Furthermore, the prediction probabilities and explained features evolving over time on an individual patient basis (Fig. 7) allow clinicians to easier understand the model and even ‘correct’ some false predictions based on information from individual features combined with clinical symptoms. For instance, as shown in Fig.7, the predicted probability of the LOS patient keeps increasing from 6 hours before CRASH because of the abnormal changes of pDec$_{mean}$, RespIDR, and MotIDR$_{mean}$, this can trigger the clinicians to earlier check the physiological status of the patient. Additionally, the one-time increase in infection probability demonstrated by the control patient can be ‘corrected’ by a clinician through physical examination which may indicate that in this case the decreased MotIDR$_{mean}$ is caused by for instance normal sleep.

One of the main limitations of our study is the difference in maturation between LOS patients and controls in the full dataset. First, we can observe a difference in GA between LOS and control groups (Table I). This difference in GA is inherent to the higher risk for sepsis in more immature infants. Second, the difference in PMA is related to the procedure of defining equivalent CRASH moment in controls, which is needed to perform our analysis. In our study, we defined an equivalent CRASH moment at a corresponding PMA of the CRASH moment in LOS patients. However, due to the more immature LOS patients, not all LOS patients could be age-matched with controls. As a consequence, a one-week difference in PMA remained between both groups. This difference may influence those features (HRV, respiration, and likely motion) that are known to have maturation dependency [20]. Hence, we defined the matched subdataset to address this limitation. It can be seen that the performance of the subdataset is less than that of the full dataset, indicating that the model performance in the full dataset may be slightly overestimated because of the significant difference in maturation. However, immaturity per se is an important indicator for LOS prediction, and the matched subdataset deliberately excludes this potential benefit for LOS prediction. This indicates that it would be interesting to use the current algorithm on a bigger dataset with a larger range of gestational ages. Besides, even though using methods that inherently include feature selection or penalization to reduce overfitting effects and the use of cross validation was also aimed to prevent model overfitting, the relatively large number of features used in this study might still lead to some overfitting in our current small dataset, which might explain the relatively large standard deviation in prediction performance as shown in Table IV. This encourages us to further validate our algorithm on a bigger dataset. Another limitation is that this is a single-center study. The model needs further external validation with data from other hospitals, since some inevitable issues may be introduced by therapeutic policies within the hospital. As discussed in several reviews [8], [38], a collaboration between institutes to develop multicenter

This work is licensed under a Creative Commons Attribution 4.0 License. For more information, see https://creativecommons.org/licenses/by/4.0/

This article has been accepted for publication in IEEE Journal of Biomedical and Health Informatics. This is the author's version which has not been fully edited and content may change prior to final publication. Citation information: DOI 10.1109/JBHI.2022.3216055

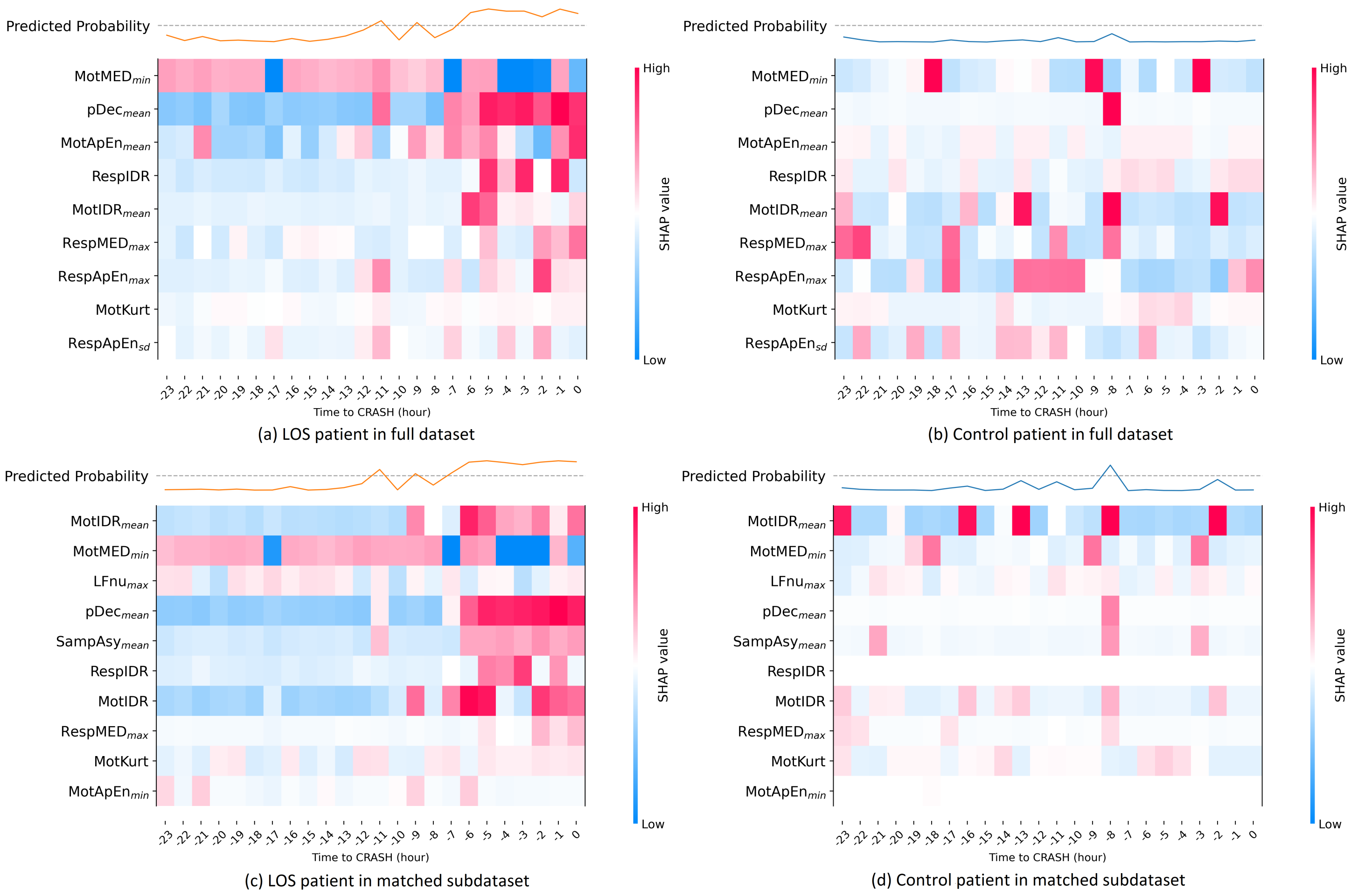

Fig. 7: Real-time prediction for individual patients. The upper line plot represents the predicted probability of each hour before CRASH, with the dashed line representing an example of the decision-making threshold of 0.5. The lower heat map represents top features evolving over time to CRASH for individual patients. (a) for a LOS patient in full dataset. (b) for a control patient in full dataset. (c) for a LOS patient in matched subdataset. (d) for a control patient in matched subdataset

studies should be performed, but this also requires not only the standardization of the clinical definitions and protocols but also the data. One of the main difficulties nowadays is that not all data are stored at a high temporal resolution, not all vital signs are measured for all patients, and different centers use different types of sensors for their clinical patient monitoring, further complicating the collaboration. Moreover, the case-control design in this feasibility study did not represent the true risk or prevalence of LOS in the population of preterm infants. In future work, further validation on the dataset from the full NICU course (random selection of patients) is needed. Lastly, commonly used features were extracted for physiological signals at a fixed segment length in this study. In future work, varying segment length [39], and more advanced features and algorithms such as network-based features or deep neural networks could be investigated for LOS prediction to further improve the LOS-related physiology conveyed in the physiological signals. These may also better capture the temporal information relating to disease development [40]. More fusion of physiological signals such as temperature and blood pressure should be considered in future work because of their clinical implications on sepsis [4]. The end-to-end solutions based on deep neural networks are also planned as future works, since such methods have been successfully applied to several disease predictions such as ventricular fibrillation [41], sepsis in adults [42], and sleep arousal disorder [43]. Besides, the model used segments of 24 hours before CRASH to predict LOS, for a few patients these segments were located in the transitional period, meaning within 72 hours after birth. We did not eliminate this transitional period, because it allows earlier prediction for LOS. Further investigation on the characteristics of the transitional period is required. Moreover, the datasets were built by the segments from each patient which are time dependent, the models focused on binary prediction without attention on time-to-event outcomes, the impact of these repeated measures and the temporal information in LOS development also needs further investigation in future work.

This work is licensed under a Creative Commons Attribution 4.0 License. For more information, see https://creativecommons.org/licenses/by/4.0/

This article has been accepted for publication in IEEE Journal of Biomedical and Health Informatics. This is the author's version which has not been fully edited and content may change prior to final publication. Citation information: DOI 10.1109/JBHI.2022.3216055

VI. CONCLUSION

In this study, we demonstrated the potential for predictive monitoring of LOS based on multiple physiological signals readily available through routine patient monitoring in NICUs. We showed that motion features obtained from cardiorespiratory signal waveforms can add complementary information to electrocardiogram and chest impedance features in predicting LOS in preterm infants. Furthermore, the visualization of how each feature was weighted by the algorithm can strengthen its interpretability and motivate clinical interventions based on a combination of physiological aspects when applying the proposed LOS prediction model in NICUs. In future work, a bigger and cross-center dataset should be used to further validate the performance and generalizability of the model.

REFERENCES

[1] L. Liu et al., "Global, regional, and national causes of under-5 mortality in 2000–15: an updated systematic analysis with implications for the Sustainable Development Goals," Lancet, vol. 388, no. 10063, pp. 3027–3035, 2016.

[2] A. L. Shane, P. J. Sánchez, and B. J. Stoll, "Neonatal sepsis," Lancet, vol. 390, no. 10104, pp. 1770–1780, 2017.

[3] A. Al-Matary et al., "Characteristics of neonatal Sepsis at a tertiary care hospital in Saudi Arabia," J. Infect. Public Health, vol. 12, no. 5, pp. 666–672, 2019.

[4] B. A. Sullivan and K. D. Fairchild, "Vital signs as physiomarkers of neonatal sepsis," Pediatr. Res., vol. 91, no. 2, pp. 273–282, 2022.

[5] E. Giannoni et al., "Neonatal Sepsis of Early Onset, and Hospital-Acquired and Community-Acquired Late Onset: A Prospective Population-Based Cohort Study," J. Pediatr., vol. 201, pp. 106-114, 2018.

[6] R. Singh, L. Sripada, and R. Singh, "Side effects of antibiotics during bacterial infection: Mitochondria, the main target in host cell," Mitochondrion, vol. 16, pp. 50–54, 2014.

[7] J. M. Klucher, K. Davis, M. Lakkad, J. T. Painter, and R. K. Dare, "Risk factors and clinical outcomes associated with blood culture contamination," Infect. Control Hosp. Epidemiol., pp. 1–7, 2021.

[8] E. Persad et al., "Neonatal sepsis prediction through clinical decision support algorithms: A systematic review," Acta Paediatr. Int. J. Paediatr., vol. 110, no. 12, pp. 3201–3226, 2021.

[9] E. H. Verstraete, K. Blot, L. Mahieu, D. Vogelaers, and S. Blot, "Prediction models for neonatal health care-associated sepsis: A meta-analysis," Pediatrics, vol. 135, no. 4, pp. e1002–e1014, 2015.

[10] J. Wynn, T. T. Cornell, H. R. Wong, T. P. Shanley, and D. S. Wheeler, "The host response to sepsis and developmental impact," Pediatrics, vol. 125, no. 5, pp. 1031–1041, 2010.

[11] M. P. Griffin, D. E. Lake, E. A. Bissonette, F. E. Harrell, T. M. O'Shea, and J. R. Moorman, "Heart rate characteristics: Novel physiomarkers to predict neonatal infection and death," Pediatrics, vol. 116, no. 5, pp. 1070–1074, 2005.

[12] M. P. Griffin, D. E. Lake, T. M. O'Shea, and J. R. Moorman, "Heart rate characteristics and clinical signs in neonatal sepsis," Pediatr. Res., vol. 61, no. 2, pp. 222–227, 2007.

[13] K. D. Fairchild et al., "Septicemia mortality reduction in neonates in a heart rate characteristics monitoring trial," Pediatr. Res., vol. 74, no. 5, pp. 570–575, 2013.

[14] J. R. Moorman et al., "Mortality reduction by heart rate characteristic monitoring in very low birth weight neonates: A randomized trial," J. Pediatr., vol. 159, no. 6, pp. 900–906, 2011.

[15] K. D. Fairchild et al., "Vital signs and their cross-correlation in sepsis and NEC: A study of 1,065 very-low-birth-weight infants in two NICUs," Pediatr. Res., vol. 81, no. 2, pp. 315–321, 2017.

[16] R. Joshi, D. Kommers, L. Oosterwijk, L. Feijs, C. Van Pul, and P. Andriessen, "Predicting Neonatal Sepsis Using Features of Heart Rate Variability, Respiratory Characteristics, and ECG-Derived Estimates of Infant Motion," IEEE J. Biomed. Heal. Informatics, vol. 24, no. 3, pp. 681–692, 2020.

[17] L. Cabrera-Quiros et al., "Prediction of Late-Onset Sepsis in Preterm Infants Using Monitoring Signals and Machine Learning," Crit. Care Explor., vol. 3, no. 1, p. e0302, 2021.

[18] "Vermont Oxford Network: Manual of Operations: Part 2. Data Definitions & Infant Data Forms. Release 22.0," 2007.

[19] N. Hofer, E. Zacharias, W. Müller, and B. Resch, "An update on the use of C-reactive protein in early-Onset neonatal sepsis: Current insights and new tasks," Neonatology, vol. 102, no. 1, pp. 25–36, 2012.

[20] C. Leon et al., "Evaluation of Maturation in Preterm Infants Through an Ensemble Machine Learning Algorithm Using Physiological Signals," IEEE J. Biomed. Heal. Informatics, vol. 26, no. 1, pp. 400–410, 2022.

[21] M. P. Griffin, T. M. O'Shea, E. A. Bissonette, F. E. Harrell, D. E. Lake, and J. R. Moorman, "Abnormal heart rate characteristics preceding neonatal sepsis and sepsis-like illness," Pediatr. Res., vol. 53, no. 6, pp. 920–926, 2003.

[22] M. J. Rooijakkers, C. Rabotti, S. G. Oei, and M. Mischi, "Low-complexity R-peak detection for ambulatory fetal monitoring," Physiol. Meas., vol. 33, no. 7, pp. 1135–1150, 2012.

[23] H. Lee et al., "A new algorithm for detecting central apnea in neonates," Physiol. Meas., vol. 33, no. 1, pp. 1–17, 2012.

[24] I. Zuzarte, P. Indic, D. Sternad, and D. Paydarfar, "Quantifying Movement in Preterm Infants Using Photoplethysmography," Ann. Biomed. Eng., vol. 47, no. 2, pp. 646–658, Feb. 2019.

[25] Z. Peng et al., "Body Motion Detection in Neonates Based on Motion Artifacts in Physiological Signals from a Clinical Patient Monitor," in 2021 43rd Annual International Conference of the IEEE Engineering in Medicine & Biology Society (EMBC), Nov. 2021, pp. 416–419.

[26] M. Malik et al., "Heart rate variability: Standards of Measurement, Physiological," Eur. Heart J., vol. 17, pp. 354–381, 1996.

[27] D. R. Kommers et al., "Features of Heart Rate Variability Capture Regulatory Changes During Kangaroo Care in Preterm Infants," J. Pediatr., vol. 182, pp. 92-98.e1, 2017.

[28] B. P. Kovatchev, L. S. Farhy, H. Cao, M. P. Griffin, D. E. Lake, and J. R. Moorman, "Sample Asymmetry Analysis of Heart Rate Characteristics with Application to Neonatal Sepsis and Systemic Inflammatory Response Syndrome," Pediatr. Res., vol. 54, no. 6, pp. 892–898, 2003.

[29] R. Joshi et al., "Cardiorespiratory coupling in preterm infants," J. Appl. Physiol., vol. 126, no. 1, pp. 202–213, 2019.

[30] C. Leon, G. Carrault, P. Pladys, and A. Beuchee, "Early Detection of Late Onset Sepsis in Premature Infants Using Visibility Graph Analysis of Heart Rate Variability," IEEE J. Biomed. Heal. Informatics, vol. 25, no. 4, pp. 1006–1017, 2021.

[31] X. Navarro, F. Porée, A. Beuchée, and G. Carrault, "Respiration signal as a promising diagnostic tool for late onset sepsis in premature newborns," Comput. Cardiol. (2010)., vol. 37, pp. 947–950, 2010.

[32] C. Cortes and V. Vapnik, "Support-vector network," Mach. Learn., vol. 20, pp. 273–297, 1995.

[33] S. M. Lundberg, G. G. Erion, and S.-I. Lee, "Consistent Individualized Feature Attribution for Tree Ensembles," arXiv:180203888, 2018.

[34] S. M. Lundberg and S. I. Lee, "A unified approach to interpreting model predictions," Adv. Neural Inf. Process. Syst., pp. 4766–4775, 2017.

[35] E. Tjoa and C. Guan, "A Survey on Explainable Artificial Intelligence (XAI): Toward Medical XAI," IEEE Trans. Neural Networks Learn. Syst., vol. 32, no. 11, pp. 4793–4813, 2021.

[36] S. M. Lundberg et al., "Explainable machine-learning predictions for the prevention of hypoxaemia during surgery," Nat. Biomed. Eng., vol. 2, no. 10, pp. 749–760, 2018.

[37] J. Bekhof, J. B. Reitsma, J. H. Kok, and I. H. L. M. Van Straaten, "Clinical signs to identify late-onset sepsis in preterm infants," Eur. J. Pediatr., vol. 172, no. 4, pp. 501–508, Apr. 2013.

[38] L. M. Fleuren et al., "Machine learning for the prediction of sepsis: a systematic review and meta-analysis of diagnostic test accuracy," Intensive Care Med., vol. 46, no. 3, pp. 383–400, 2020.

[39] A. Honor, "Classification and feature extraction for neonatal sepsis detection Classification and feature extraction for neonatal sepsis detection," TechRxiv. Prepr., 2022.

[40] J. F. Hsu et al., "Machine learning approaches to predict in-hospital mortality among neonates with clinically suspected sepsis in the neonatal intensive care unit," J. Pers. Med., vol. 11, no. 8, p. 695, 2021.

[41] X. Li and Z. Dong, "Detection and prediction of the onset of human ventricular fibrillation: An approach based on complex network theory," Phys. Rev. E - Stat. Nonlinear, Soft Matter Phys., vol. 84, no. 6, pp. 1–4, 2011.

[42] S. P. Shashikumar, Q. Li, G. D. Clifford, and S. Nemati, "Multiscale network representation of physiological time series for early prediction of sepsis," Physiol. Meas., vol. 38, no. 12, pp. 2235–2248, 2017.

[43] H. Li and Y. Guan, "DeepSleep convolutional neural network allows accurate and fast detection of sleep arousal," Commun. Biol., vol. 4, p. 18, 2021.

This work is licensed under a Creative Commons Attribution 4.0 License. For more information, see https://creativecommons.org/licenses/by/4.0/
