# Supplementary material for: Exploring Computational Techniques in Preprocessing Neonatal Physiological Signals for Detecting Adverse Outcomes: Scoping Review
Source: Interact J Med Res. 2024 Aug 20;13:e46946. doi: 10.2196/46946 (PMC11372324; doi:10.2196/46946)
Supplement: Multimedia Appendix 3 [file ijmr_v13i1e46946_app3.zip › Included Papers - Final/4383/Juraev et al. - 2022 - Multilayer dynamic ensemble model for intensive ca.pdf]

Journal of Biomedical Informatics 135 (2022) 104216

Contents lists available at ScienceDirect

Journal of Biomedical Informatics

journal homepage: www.elsevier.com/locate/yjbin

Original Research

Multilayer dynamic ensemble model for intensive care unit mortality prediction of neonate patients

Firuz Juraev [a], Shaker El-Sappagh [a,b], Eldor Abdukhamidov [a], Farman Ali [c], Tamer Abuhmed [a,*]

[a] College of Computing and Informatics, Sungkyunkwan University, Suwon, South Korea
[b] Faculty of Computer Science and Engineering, Galala University, Suez, Egypt
[c] Department of Software, Sejong University, Seoul, South Korea

ARTICLE INFO

Keywords:
Neonate mortality prediction
Dynamic ensemble classifier
Intensive care unit
Length of stay prediction
Multilayer ensemble
Explainable artificial intelligence

ABSTRACT

Robust and rabid mortality prediction is crucial in intensive care units because it is considered one of the critical steps for treating patients with serious conditions. Combining mortality prediction with the length of stay (LoS) prediction adds another level of importance to these models. No studies in the literature predict such tasks for neonates, especially using time-series data and dynamic ensemble techniques. Dynamic ensembles are novel techniques that dynamically select the base classifiers for each new case. Medically, implementing an accurate machine learning model is insufficient to gain the trust of physicians. The model must be able to justify its decisions. While explainable AI (XAI) techniques can be used to handle this challenge, no studies have been done in this regard for neonate monitoring in the neonatal intensive care unit (NICU). This study utilizes advanced machine learning approaches to predict mortality and LoS through data-driven learning. We propose a multilayer dynamic ensemble-based model to predict mortality as a classification task and LoS as a regression task for neonates admitted to the NICU. The model has been built based on the patient's time-series data of the first 24 h in the NICU. We utilized a cohort of 3,133 infants from the MIMIC-III real dataset to build and optimize the selected algorithms. It has shown that the dynamic ensemble models achieved better results than other classifiers, and static ensemble regressors achieved better results than classical machine learning regressors. The proposed optimized model is supported by three well-known explainability techniques of SHAP, decision tree visualization, and rule-based system. To provide online assistance to physicians in monitoring and managing neonates in the NICU, we implemented a web-based clinical decision support system based on the most accurate models and selected XAI techniques. The code of the proposed models is publicly available at https://github.com/InfoLab-SKKU/neonateMortalityPrediction.

1. Introduction

Patient hospitalization is considered the principal cost of patient care. Admission to an intensive care unit (ICU) is one of the most expensive and complex phases in the hospital stay [1,2]. It accounts for up to one-third of total hospital costs [3]. Hospitals are always under pressure to enhance the ICU's efficiency and reduce costs. ICU mortality and length of stay (LoS) are the most crucial clinical outcomes for patient admission [4]. ICU LoS is a major indicator for the consumption of hospital resources. Its prediction is critical, because clinicians use these predictions to plan ICU capacity, select appropriate treatment options, identify unexpectedly long ICU length of stay, and benchmark ICUs [5]. In addition, this metric is important both for healthcare providers and patients, because it is used as an indicator for other outcomes such as hospital and ICU mortalities, the severity of illness, and healthcare resource utilization. Quantifying ICU LoS outcomes is difficult even for experienced physicians [6]. Verburg et al. [7] reviewed the ICU LoS prediction models for adults and asserted that no model completely satisfied physician requirements. No model produced a prediction with low bias, and R^2 was 0.05–0.28 across patients, and 0.01–0.64 across ICU. This task is complex, because LoS is prone to outliers, there is no standard definition for LoS, and there are no criteria for selecting predictive variables. Different studies have concentrated on different categories of patients. For example, Peres et al. [2] surveyed the most critical risk factors that should be considered in prediction models for prolonged ICU LoS. These factors included severity scores, mechanical

* Corresponding author.
E-mail address: tamer@skku.edu (T. Abuhmed).

https://doi.org/10.1016/j.jbi.2022.104216
Received 24 December 2021; Received in revised form 25 September 2022; Accepted 28 September 2022
Available online 5 October 2022
1532-0464/© 2022 Elsevier Inc. All rights reserved.

ventilation, hypomagnesemia, delirium, malnutrition, infection, trauma, red blood cells, and PaO2:FiO2. Other studies, such as Seaton et al. [8] and Atashi et al. [9], concentrated on the neonatal unit and after coronary artery bypass grafting ICU patients, respectively.

On the other hand, hospitals plan to maximize their ICU statistics by reducing the number of patients dying inside the ICU. There is a strong correlation between LoS and mortality [10,11,12,4], and to improve their prediction, researchers have developed novel machine learning models [4] and scores [19,20]. Awad et al. reviewed the LoS and mortality studies for acute medicine and critical care units and their correlation [13]. Many medical prognostic scores like, SOFA [14], SAPS-II [15], and APACHE II [16], have been utilized to predict outcomes and evaluate the severity of illness [17]. These scoring systems or universal models have several limitations, which include: (1) they led to miscalculation for a specific subgroup of patients, as they applied to patients with significantly different risk factors at admission, (2) models need a periodic update to reflect possible enhancements in medical practice, (3) these scores are based on a small number of baseline characteristics collected from the first 24 h after ICU admission, but do not consider the evolution of the patient's clinical situation during the ICU stay, and (4) they utilized simple models like logistic regression the assume a linear and additive relationship between outcome variable (e.g., mortality) and predictors [4,18,19,20]. Zimmerman et al. [21] used the APACHE score to predict LoS, which showed poor accuracy (R^2= (0.18–0.22)) across individual patients. In fact, APACHE IV developers explicitly stated that "*the APACHE IV model provides clinically useful ICU length of stay predictions for critically ill patient groups, but its accuracy and utility are limited for individual patients*" [21]. Siddiqui et al. [22] compared the pre-ICU admission scores, including the SIRS, EWS, and qSOFA scores, to predict the mortality and ICU LoS for patients admitted with the diagnosis of sepsis. Ding et al. [20] asserted that these scores are neither sufficient nor accurate, because they established global offline models unsuitable for specific patients. Machine learning (ML) and deep learning (DL) models based on big time-series data can be used to improve the prediction of ICU LoS and mortality [1,23]. These models can be easily updated and create customized decisions based on the patient's temporal data collected during the stay. For example, the DeepSOFA model is demonstrated to have better performance than the SOFA score [24]. LoS and mortality predictions have been demonstrated in different domains, such as after cardiac surgeries where short LoS could streamline efficient fast-track pathways, while long LOS could provide the opportunity to consider modifiable risk factors and surgical timing [25]. Neonates are patients aged under 28 days (d). The previous tasks have not been studied for neonates.

After studying the literature, we found the following shortcomings. Many studies have proposed mortality and LoS prediction models for children and adult patients. Such techniques utilized classical machine learning [26,27], ensemble [28,29], and deep learning [30,31,32] algorithms. However, few studies have concentrated on neonate patients using regular machine learning or deep learning models [33,34]. No studies utilized dynamic ensemble models to optimize these tasks (ICU LoS and mortality predictions) for neonates based on raw time-series data. Inspired by these limitations of the literature, in this paper, we propose a two layers model to jointly predict neonates' ICU LoS and mortality. In addition, this is the first work in the literature that jointly explores the ICU LoS and mortality predictions using the MIMIC dataset for neonates. The contributions of the study can be summarized as follows.

1. We propose an explainable multilayer prediction framework of neonates' mortality in ICU based on DES techniques. The proposed model has two layers. The first layer is a classification model to predict the risk of patient mortality in the ICU, and the second layer is a regression model to predict neonates' LoS in the ICU.
2. In the classification layer, we explore the capabilities of different machine learning models to learn ICU time-series data for predicting patient mortality. These models include base classifiers as decision tree, support vector machine, logistic regression, naïve Bayes, k-nearest neighbors, and multilayer perceptron; static ensemble model as random forest, AdaBoost, XGBoost, gradient boosting, CatBoost, LGBM, and majority voting; and DES models that include KNORAE, mETA-DES, DESP, KNORAU, DES-KNN, KNOP, FIRE-KNORA-U, FIRE-KNORA-E, FIRE-mETA-DES, FIRE-DESP, FIRE-DES-KNN, and FIRE-KNOP.
3. In the regression layer, we explore the performances of different classical regression models, static ensemble regression models, and voting regression with homogeneous and heterogeneous base regression models. These models include classical regression models of linear regression, k-nearest neighbors, linear support vector regression, decision tree, ridge, and lasso; static ensemble regression models of random forest, CatBoost, XGBoost, LGBM, gradient boosting, extra trees, and AdaBoost.
4. We propose a novel data preprocessing algorithm to prepare time-series data of 3,133 patients from MIMIC-III dataset with different time intervals and different missing rates.
5. We extend the framework by adding the explainability to help physicians understand model decisions. The selected best machine learning models in the two layers and the explainability features are embedded in a web-based application to provide the physician with a real and complete implementation of the ICU neonates management system.

The study is organized as follows. Section 2 reviews the related work. Section 3 presents the proposed model, while Section 4 provides the performance evaluation criteria, and Section 5 describes the experimental setup. Sections 6 and 7 discuss the classification and regression results, respectively. Section 8 Model addresses explainability, while Section 9 considers system implementation. Section 10 then concludes the paper.

2. Related works

2.1. Mortality prediction

The literature of this task has two branches hospital mortality [35,36], and ICU mortality [37,38,39]. Patient-specific ICU mortality prediction is more complex, time-consuming, and expensive, because it enables timely decisions towards effective patient care and optimized ICU resource management [23]. Many regular ML models have been used to predict ICU mortality. Johnson et al. [38] tested gradient boosting and logistic regression models for ICU mortality prediction using a simple set of features collected from the MIMIC-III dataset [39]. Fika et al. [40] concentrated on adult patients to build their ICU mortality prediction model. The study built a multivariate logistic regression model based on 436 patients collected during the first 24 h of ICU admission from medical/surgical and multidisciplinary ICUs. The model achieved an AUC of 0.85. Sadeghi et al. [41] utilized the vital signs to optimize a decision tree classifier for mortality prediction. Liu et al. [42] proposed an end-to-end learning model based on an SVM classifier, cost-sensitive principal component analysis for feature extraction, and chaos particle swarm optimization for hyperparameter optimization. The model has been tested using 4,000 subjects whose 41 variables were recorded during the first 48 h after admission to ICU. The study concentrated on adult patients admitted to four ICUs (i.e., cardiac, surgical, medical, or trauma). The model achieved an AUC of 0.7718. Ding et al. [20] proposed a two-step model for the personalized mortality prediction of ICU patients. The first step clusters patients, while the second step is for mortality prediction. The model combined just-in-time learning and an extreme learning machine. The experiments have been done using 4000 ICU patients, and the model achieved an AUC of 0.8568. Monteiro [43] proposed a linear SVM model to predict ICU mortality. The model achieved an accuracy of 0.73.

Alam et al. [44] asserted that the current ML literature achieved insufficient accuracy levels, and the literature needs to explore more powerful techniques to boost the performance of these classical ML techniques. There are two main potential approaches to improve this performance. The first approach is to use advanced DL models, while the other approach is to combine more than one classical ML model to form either static or dynamic ensembles. DL offers state-of-the-art capabilities, and research suggests DL could outperform traditional ML techniques [45]. Ge et al. [46] compared an LSTM-based model with a logistic regression model for ICU mortality prediction. Recently, novel DL architectures such as LSTM performed well to predict ICU mortality and LoS (binary classification) [47]. The model achieved a c-statistic of 0.7614, compared to 0.7412 for the logistic regression model. Krishnan et al. [23] proposed an extreme learning machine-based neural network to predict ICU mortality. The authors used a genetic algorithm-based feature selection technique to improve care delivery and cost optimization to use the minimal patient-specific feature set. The study was based on 31,691 adult patients (age > 15) from the MIMIC III dataset. In terms of AUC, the model outperformed the traditional scoring systems by (11–29) % and the state-of-the-art models by up to 14 %. Caicedo-Torres and Gutierrez [48] proposed the ISeeU model. This is a multiscale CNN architecture trained on MIMIC-III for mortality prediction. CNN model can handle both static and dynamic data, which is expected to outperform other hybrid architectures (ANN/RNN). The model attained a testing AUC of (0.8735 $\pm$ 0.0025). Searching for the best neural network architecture is a challenging task that requires intensive resources and a large amount of data. In addition, DL models are black-box models where it is difficult to know why the model has taken a specific decision. If not better, comparable results can be achieved by using easier, faster, and more robust ensemble techniques. Ensemble techniques are discussed in the following subsection.

2.2. Ensemble modeling

Model stability and performance can be improved by building ensemble models, such as boosting [38] and random forests [34]. Ensemble modeling is an active area of research in machine learning and pattern recognition, where ensembles can combine a diverse set of simple base models, using techniques such as bootstrapping, stacking [49], or voting [50]. The base models could be homogeneous or heterogeneous regular ML models or deep learning models [51,52] In recent years, several studies have been published that demonstrated the advantages of ensemble models over base models based on theoretical [53], and empirical [54,55], evaluations. Kefi et al. [33] proposed an ensemble model based on a linear discriminant analysis-based classifier for neonate mortality prediction after the two first hours of admission. The model is based on lab tests, demographics, and vital signs. It achieved an accuracy of 0.947. The study predicted the exact mortality time using a random forest regressor and achieved an F-score of 0.871. Pirracchio [37] tested the performance of the Super Learner algorithm [56] (i.e., an ensemble model) for predicting ICU mortality, and compared its performance with severity scores using the MIMIC-II database. Alam et al. [44] used lab data only to predict ICU mortality based on an ensemble classifier. Awad et al. [57] proposed a random forest ensemble model for early ICU mortality prediction based on the data collected during the first 6 h of admission. The study collected single stays data for 11,722 adult patients (age $\geq$ 16) from MIMIC-II dataset admitted to medical, surgical, or cardiac surgery recovery ICUs. The proposed model achieved an AUC of 0.90 $\pm$ 0.01. El-Rashidy et al. [58] proposed a stacking ensemble model for ICU mortality prediction of adults. The study utilized 10,664 subjects from the MIMIC III dataset. The model achieved an accuracy of 94.4 %. Lin et al. [59] proposed a random forest model to predict ICU mortality for adult patients (age $\geq$ 16) with acute kidney injury. The model achieved an AUC of 0.866 and an accuracy of 0.728. Rowan et al. [60] proposed an ensemble of ANN to investigate the role of 32 demographic factors, medical and surgical histories, and clinical measures, to predict the need for an extended LOS in post-cardiac surgery ICU. They built a binary classifier, i.e., less than or>24 h LOS in the ICU, and achieved an AUC of 0.902, sensitivity of 91 %, and specificity of 78 %. As is apparent, using an ensemble of base classifiers achieved promising results. However, the previous studies are based on the static ensemble techniques, where while building the model, the list of base classifiers is selected only once. Moreover, no studies in the literature used static ensembles to predict neonate mortality in ICU, especially using multimodal and time-series data.

Recently, one of the most promising ensemble techniques has become dynamic ensemble selection (DES), where base classifiers are selected dynamically for each query sample to be classified [50]. For each new case to be classified, the DES techniques firstly estimate the competence level of each classifier from a pool of classifiers. Secondly, an ensemble containing the most competent classifiers is selected to predict the label of this query sample. The main idea of DES is that not every classifier in the pool is an expert in classifying all unknown samples; rather, each base classifier is an expert in a different local region of the feature space. As a result, the key is how to dynamically select the most competent base classifiers for any given query sample. Many ideas have been proposed to solve this challenge, including DES algorithms such as K-nearest oracle elimination (KNORAE), *meta*-learning for dynamic ensemble selection (mETA-DES), dynamic ensemble selection performance (DESP), Dynamic ensemble selection-Clustering (DES-Clustering), K-nearest oracle union (KNORAU), Dynamic ensemble selection-K nearest neighbor (DES-KNN), multiple-classifier behavior (MCB), and Frienemy Indecision Region Dynamic Ensemble Selection (FIRE) frameworks, such as FIRE-KNORA-U and FIRE-KNORA-E [50,61]. DES has been used to solve many real-world problems, including Alzheimer's prediction [62], imbalanced data [63], credit risk assessment [64], and multiclass classification [65]. DES has proven its superiority over simple classifiers, static ensemble classifiers, and dynamic classifier selection techniques. *In the ICU domain, especially neonate mortality prediction, there are no studies that have implemented and tested DES techniques*. Using these techniques to interpret complex ICU data (i.e., multimodal and time-series data) offers promises. In this study, we investigate this possibility.

2.3. Length of stay prediction

LoS is the number of days that a patient stays in ICU or other hospital facilities during a single admission [66]. It is difficult for physicians to predict a patient's LoS periods [19]. Some studies predicted the general hospital LoS at admission based on static features [67]. However, the most critical task is to predict the ICU LoS [68]. Some studies calculated the ICU LoS period by grouping patients based on their conditions. They assumed that every disease, illness, or procedure is associated with a specific LoS [69]. These models are known as diagnosis-related-group systems [70]. However, these systems cannot provide personalized decisions for different patients. LoS calculation is a complex task, because it is affected by other factors, such as demographics, treatment complexity, complications, and lab tests. Almashrafi [68] surveyed the factors that influence the ICU LoS after adult cardiac surgery. Literature studies calculated LoS using different techniques, including arithmetic methods [71], statistical methods [72] and ML methods [32]. Some studies modeled this task as a binary classification task [73] (e.g., LoS is long [>5 d], or short [<5 d]), or a regression task [4]. Arithmetic methods are based on calculating metrics such as the average or median LoS. These simple methods wrongly assumed that LoS is normally distributed [72]. Vasilakis et al. [72] proposed alternative statistical techniques based on survival analysis [74], which studies the role of different features on the patient's survival time. Linear regression and logistic regression are special cases of survival models [71]. Most studies built these models based on patients' diagnoses, procedures, gender, and age [75,76]. Because hospitals are complex stochastic systems, simple deterministic approaches for planning and managing are insufficient to

provide an accurate analysis [77,78,79]. For example, building systems based on simple decision rules extracted from regression trees had low predictive accuracy [80]. Moreover, Grubinger et al. [54] asserted that these systems are unstable, because any minor change in the patient data can lead to a completely different model.

Data-driven approaches used more sophisticated methods, such as deep learning (DL) techniques, ensembles models, and advanced ML techniques, such as support vector machines (SVM), to analyze the patient's big, multivariate, and time-series data. The digitalization of ICU led to the collection of big time-series data at the bedside with different origins and formats. For example, the Epimed Monitor System deployed a tool that predicts ICU LoS [81]. This is a cloud-based ICU management system that collected the data of>2,500,000 ICU admissions in Brazil. Ma et al. [82] proposed an LoS prediction model based on the combination of just-in-time learning and a one-class extreme learning machine. The model was based on 15 time-series physiological indicators; each one is represented by the first 48 h after admission to ICU. The authors extracted 360 statistical features from these indicators and used PCA to reduce the dimensionality of the data. They achieved an area under the curve (AUC), precision, G-mean, accuracy, specificity, and sensitivity of (0.851, 1, 0.7842, 0.82, 1, and 0.615), respectively. Note that the study has modeled LoS prediction as a classification task by checking if LoS will be greater than a threshold. Meyfroidt et al. [25] applied Gaussian processes for ICU LOS prediction. They collected data of 960 patients undergoing cardiac surgery who are monitored in the first 4 h after admission. Houthooft et al. [12] built an SVM model based on data values from the first five days after admission of 14,480 patients to predict ICU LoS and mortality. The study used the SOFA score as a predictor in the model. The model achieved an AUC of 0.77 for predicting mortality, a mean absolute error of 1.79 d, and a median absolute error of 1.22 d for patients who survived a non-prolonged stay. Mansouri et al. [34] evaluated the performance of different ML models to predict LoS of neonates in the neonatal intensive care unit (NICU). The study implemented a random forest regressor model based on the MIMIC III data collected from the first 24 h of admission. This model achieved R^2 of 0.78 by exploiting only patients' diagnoses and demographics data. Li et al. [83] used the LASSO algorithm to build a regression model for ICU LoS prediction. The proposed model achieved the performance of 0.88 ± 0.13 for RMSPE, 0.87 ± 0.07 for MAE, and 0.35 ± 0.09 for R^2. Daghistani et al. [84] proposed a random forest classification model for predicting in-hospital LoS for cardiac patients. The study grouped the patients into three categories based on their LoS: short (<3 d), intermediate (3–5 d), and long (>5 d). However, this could not obtain a quantitative LoS. Dominici et al. [85] proposed a logistic regression model for calculating a new nomogram score and tested it to predict a long ICU LoS among patients undergoing coronary artery bypass graft surgery. The model is based on ten predictors and trained using 7,352 patients. The study achieved a sensitivity of 74 % and a specificity of 60 %. Rouzbahman et al. [86] proposed a clustering step to summarize patients' data before applying the logistic regression analysis. The study has been applied using the MIMIC-II database to predict both ICU LoS and morality. Clustering improved prediction accuracy for both tasks (mortality prediction accuracy of 75.3 % and ICU LoS MAE of 7,300 min).

DL techniques play an important role in analyzing the ICU big time-series data. LaFaro et al. [87] built an artificial neural network (ANN) regression model to predict ICU LoS in the cardiac surgical ICU based on eight factors. The model achieved a training prediction R2 of 0.535 and a cross-validation prediction R2 of 0.410. Tu and Guerriere [88] applied an ANN model to predict ICU LOS following cardiac surgery. They used 15 factors to build a binary classifier, i.e., greater or<48 h of ICU care. They achieved an AUC of 0.7094 ± 0.0224 for training and 0.6960 ± 0.0227 for testing. Staziaki et al. [89] compared SVM and ANN models for ICU LoS prediction after torso trauma. They improved the model's performance by combining image modality with the clinical parameters. The study collected age, sex, vital signs, clinical scores, laboratory values, and CT images from 840 adult patients admitted to a level 1 trauma center after injury to the torso over the course of 1 year. ANN achieved better performance than SVM (AUC of 0.80 ± 0.04 for SVM and 0.81 ± 0.05 for ANN). Purushotham et al. [4] compared DL models with other models, including ensemble models, SAPS II, and SOFA scores for predicting LoS (i.e., regression task) and mortality (i.e., binary classification task). This study was based on the MIMIC-III time-series dataset, where features were extracted from the first 24 h and first 48 h after admission to ICU. The study used the recurrent neural networks (RNN), especially the long short-term memory (LSTM) [37] and gated recurrent unit (GRU) models, to deeply learn the raw time-series data. As a result, these DL models consistently outperform all the other approaches. However, the study concentrated on adult patients (i.e., age > 15 years) only. Rocheteau et al. [90] proposed a temporal pointwise CNN architecture for predicting the remaining LoS of patients in ICU based on electronic health record time-series data. The model combined temporal convolutional layers to capture causal dependencies across time, and pointwise convolutional layers to compute more abstract features from feature interactions. The model achieved (18–51) % improvement over the LSTM model.

Although big data analysis is promising in the medical domain, very limited applications have been used in ICU clinical practice [1,85]. Garg et al. [91] asserted that the current statistical and data-driven methods failed to handle the inherent uncertainty, complexity, and heterogeneity in a healthcare environment. The main reason for these limitations is that the proposed models are simple, and they did not handle the time-series data using more convenient DL models like LSTM or CNN.

About 11 % (440,000 newborns) of all infants are born every year in the USA. This medical care poses a financial burden of about $26 billion every year [34]. However, as noticed, few studies have concentrated on the prediction of ICU LoS for neonate patients. This task is crucial, because it assists healthcare providers in allocating required resources, and this clinical value is considered an indicator of a newborn's health status [92]. More research is needed to determine the important risk factors and build more accurate models for predicting neonates' LoS in the NICU.

2.4. Model explainability

Depending on the decisions of opaque decision systems is not acceptable in the medical domain [93]. Humans do not accept adopting techniques that are not directly interpretable and trustworthy [94]. Recently, XAI has been widely acknowledged as a critical feature for the practical deployment of trustworthy, ethical, and responsible AI models. Arrieta et al. [93] have defined XAI as "*Given an audience, an explainable AI is one that produces details or reasons to make its functioning clear or easy to understand.*" XAI has many roles based on the audience of this feature. For the model's end-users (i.e., physician and patient), this is called *explain to justify*, where the explainer justifies why it took a specific decision. It answers the question of "does it medically make sense to take a specific decision using a specific feature set?" For the model developers, this is called *explain to control*, where model developers can debug the model and discover potential flaws, which facilitate the improvement of the models' accuracy and efficiency. For the regulator, XAI answers the question of "who is responsible for the model decisions?" The European Parliament, in Article 22 of the General Data Protection Regulation (GDPR), sets out the *right of explanation* by giving patients the right to obtain explanations of automated model decisions. XAI features have been implemented in many domains, and for complete literature of the existing methods, readers are guided to the surveys in Refs. [95,93]. Medically, XAI features were implemented to explain decisions for diagnosis and progression detection of many diseases like Alzheimer's disease [96]. In the ICU domain, many studies have been implemented to explain machine learning decisions [97,98,99,100]. Ge et al. [46] proposed an explainable deep learning model for ICU mortality prediction. Comprehensive surveys of XAI in the medical domain

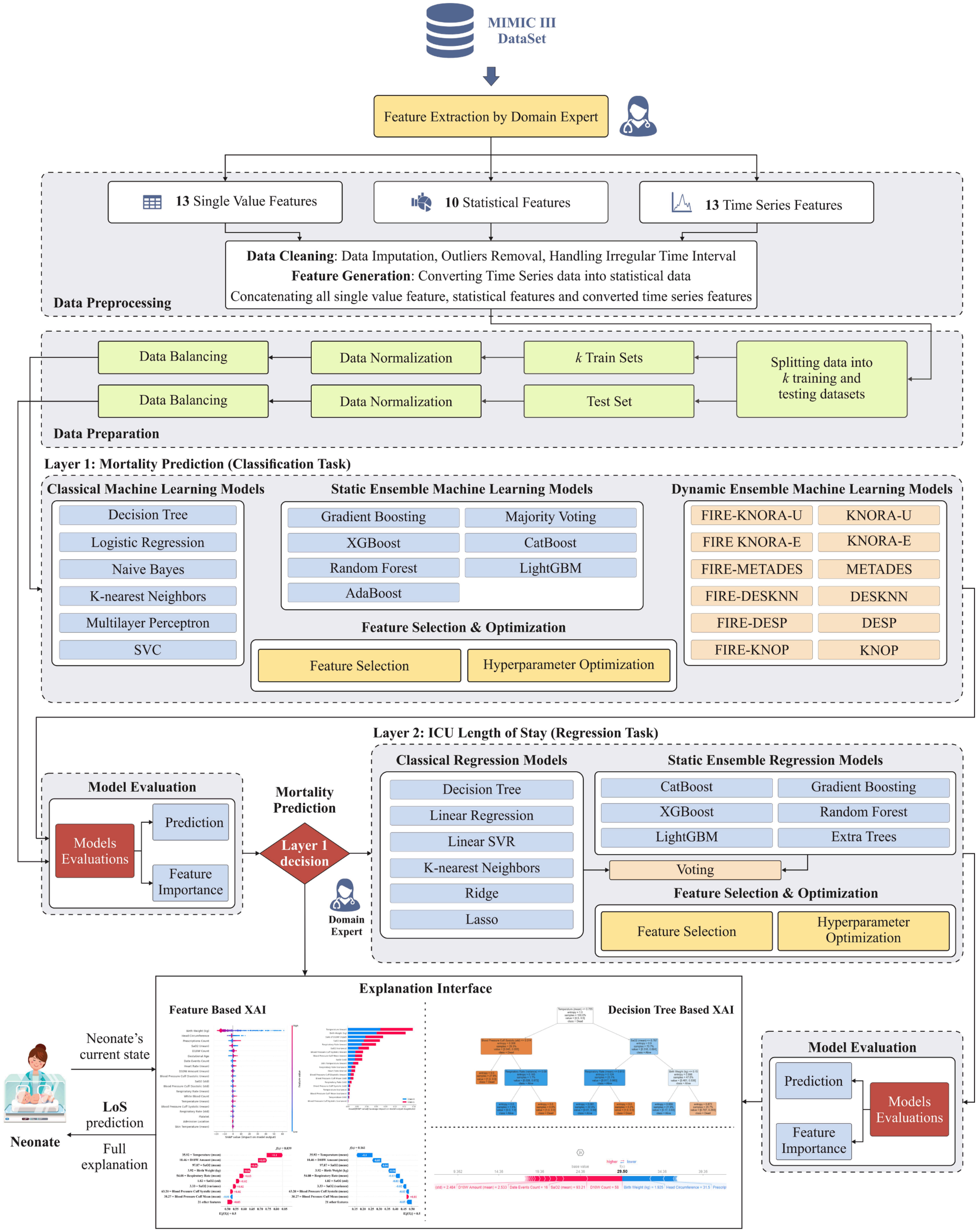

Fig. 1. The architecture of the proposed framework.

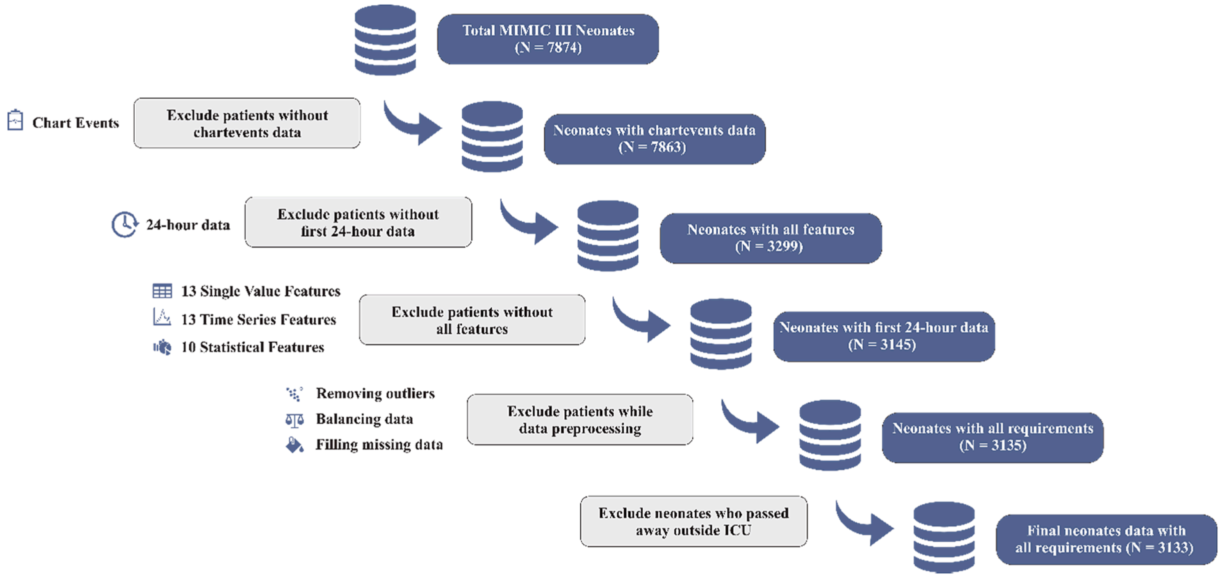

Fig. 2. Inclusion criteria of neonates.

Table 1
Statistics about single value features.

Feature Name	Total	Male/ Female	Minimum (M/F)	Maximum (M/F)	Std (M/F)	UoM
Number of neonates	3133	1748/1385	–	–	–	–
Number of dead neonates	48	30/18	–	–	–	–
Average birth weight	2.14	2.23/2.04	0.44/0.36	6.12/5.06	0.81/0.86	kg
Average birth weight for Alive neonates	2.16	2.24/2.05	0.44/0.49	6.12/5.06	0.85/0.80	kg
Average birth weight for dead neonates	1.17	1.28/0.99	0.57/0.36	3.64/2.53	0.83/0.59	kg
Average head circumference	30.59	30.98/30.1	18.75/19.0	49.0/45.0	3.58/3.56	cm
Average head circumference for Alive neonates	30.67	31.06/30.17	18.75/20.0	49.0/45.0	3.51/3.50	cm
Average head circumference for dead neonates	25.3	25.86/24.36	21.0/19.0	37.0/31.0	3.89/3.85	cm
Average LoS (Length of Stay)	22.0	21.29/22.9	0.07/0.05	150.8/171.7	25.4/26.1	days
Average LoS for Alive neonates	22.21	21.59/22.99	0.08/0.05	150.8/171.7	25.5/26.1	days
Average LoS for dead neonates	8.56	4.21/15.8	0.07/0.40	30.7/117/2	6.5/28.1	days

can be found in [101,102]. However, no studies in the literature discuss XAI capabilities for neonate monitoring in ICUs.

3. Proposed framework

Fig. 1 illustrates the proposed model for ICU mortality prediction for neonates. The main goal of this model is to provide an accurate and explainable prediction of ICU mortality and LoS for neonate patients. The model has two main layers: the classification layer for predicting mortality, and the regression layer for predicting the patient's LoS in the ICU. We optimize a collection of machine learning (ML) models for each layer, including classical ML models, static ensemble models, and dynamic ensemble models. Statistically significant difference among these machine learning models is performed using the most suitable statistical test. The models are compared using default hyperparameters and using optimized hyperparameters. These two options are combined with or without a feature selection optimization step. The best model is selected that achieves the best statistical difference in its performance. The selected model is extended to add XAI capabilities using different techniques. XAI features improve model understandability for both machine learning engineers and medical experts. Machine learning engineers can determine the logic behind the model decision, and whether the model is based on the right feature set or not. As a result, they can check model stability, and predict when the model will make the wrong decisions. On the other hand, understanding why a model takes a specific decision increases the trust of a domain expert in the prediction model's decisions. In the following subsections, we discuss each phase of the proposed model.

3.1. Data collection

This study uses the MIMIC-III database for training and testing the proposed framework. This is an open-source clinical database containing approximately 400,000 cases collected from the ICU at the Beth Israel Deaconess Medical Center in Boston from 2001 to 2012 [103]. The database includes patient's vital signs, hospital records, fluid information, laboratory tests, treatment orders, and free-text medical records. All patient's identification data were anonymized by the dataset owners.

3.1.1. Data inclusion criteria

In the MIMIC-III dataset, there is a total of 7,874 neonates. However, we selected 3133 neonates' data based on the following inclusion criteria. In the first criterion, we excluded all neonates who do not have chart events data. MIMIC-II database contains approximately 460 variables measured as biological signals. In this study, we selected two types of data, i.e., 13 single-valued features and 13 time-series features. This selection is based on the literature studies and domain experts' opinions [33,104]. We built a model to predict the neonates' mortality and length of stay based on the first 24 h data. However, some neonates do not have the first 24 h data. Therefore, in the next criterion, we excluded those neonates who do not have the first 24 h data. In the next criterion, we excluded the neonates who did not have these 26 features. In the fourth criterion, we excluded some neonates' records because of the missing values. Those records have all the features that are required in the

Table 2
Statistics about time-series features.

Feature Name	Mean	Minimum	Maximum	Standard Deviation	p-value	UoM
Heart Rate	142.0	0	300	15.5	<0.001	bpm
Respiratory Rate	52.4	0	150	15.9	<0.001	breath
SaO2	2.14	5	100	3.6	<0.001	%
HR Alarm High	198.4	100	299	12.7	<0.001	bpm
HR Alarm Low	91.4	60	100	9.9	<0.001	bpm
SaO2 Alarm High	98.9	87	100	1.9	<0.001	cmH20
SaO2 Alarm Low	89.4	75	100	2.5	<0.001	cmH20
Temperature	35.8	25	39.2	1.0	<0.001	°C
Skin Temperature	36.2	25.6	39.3	0.4	<0.001	°C
BP Cuff Diastolic	33.2	0	96	8.2	<0.001	cc/min
BP Cuff Mean	42.8	17	98	8.2	<0.001	cc/min
BP Cuff Systolic	59.2	0	113	10.5	<0.001	cc/min
Glucometer	81.4	0	353	32.2	<0.001	gms

aforementioned criteria. However, there are many missing values that are challenging to fill. Therefore, we excluded them from further processing. In the last criteria, we excluded two neonates' records who passed away outside the ICU. We proposed prediction models for mortality prediction at the ICU only. Therefore, neonates who passed away not at the ICU are outliers for our models. In the fourth criterion, we excluded some neonates during the data preprocessing if they have the first 24 h of data, but all these data are missing. Fig. 2 illustrates the sequence of data exclusion steps.

Table 1 contains statistics about the single value features, and Table 2 contains statistics about the time-series features of the neonate patients. MIMIC III database consists of 26 tables. Among them, we used data from nine tables, as shown in Table 3. Our main time-series data are extracted from the CHARTEVENTS table. Table 3 discusses all the relevant tables and columns.

Fig. 3 illustrates the distribution of the time-series features for different classes. The selected twelve features have statistically significantly different values in the two classes (P-value < 0.001). As shown in Fig. 3, these features enable the two classes to be discriminated well. For example, the Dead class has a higher heart rate and lower respiratory rate than the *Alive* class. The resulting distributions of the selected features for both classes are medically intuitive. Moreover, we explored the difference in distributions among features for neonates and not neonates, i.e., adults and children, as shown in Figure S5 in SI. We noticed significant differences between the distributions of neonates and adults.

3.2. Data preprocessing

The first step in the proposed framework is to improve the quality of the collected dataset. In this section, we discuss in detail the steps used to handle the issues in the dataset, including outliers and missing values.

3.2.1. Removing outliers

Most machine learning models are sensitive to outliers. Therefore, our domain expert has defined certain boundaries for outliers; and using those boundaries, we removed the outliers. Patients records that have outliers are not completely removed from the dataset. First, we fill them with null values and consider them as missing values; second, we fill these values using our proposed data imputation technique.

3.2.2. Data imputation

In our dataset, some patient data are missed for some periods. This means that in those periods, there was some failure in the reading system. To fill those missing data, we utilized our proposed customized algorithm. We propose a novel data imputation algorithm (*Algorithm* 1). We used forward and backward filling, and filling with mean. To preserve each patient's data and not mix a patient's data with other patients, we processed every patient's data separately, by splitting them. Other techniques, such as forward and backward fillings, apply their algorithms to whole data, which is not acceptable in the medical field. Applying filling techniques to whole data does not make medical sense, because it may mix different patients' data. For example, suppose the patient's first record was missed, and the applied filling technique is traditional forward filling. In that case, the missing record is filled with the previous patient's last record, which is not acceptable in the medical field. Therefore, in our imputation algorithm, we processed each neonate's data separately. First, the algorithm checks the first record of the individual neonate's data. The algorithm applies backward filling if the first record is missed; otherwise, it applies forward filling. The main reason to use forward and backward filling is that using those techniques, we can fill the missing record with the value of the nearest available record, which makes medical sense. We also applied the filling with the mean, but that does not give a good result as our filling algorithm. The proposed data imputation algorithm is different from all existing techniques. The algorithm fills the missing values with accurate and more intuitive values from the medical point of view.

Algorithm 1: Filling null cells using Forward and Backward filling

Input: Time-series dataset: *D*
Output: filled_dataset *//The dataset with all null values filled using forward and backward techniques*
1. Sort dataset by neonate *ID* and chart time.
2. Make a list of all unique neonates ID: *L*.
3. Add new columns 'row' that indicates row number for each neonate.
4. Fill each neonates' null rows separately:
filled_dataset = []
For *id* in *L* **DO**
single_neonate_records =*D*[*id*]
IF (single_neonate_records['row'] == 1) and (single_neonate_records['value'] == null) **THEN**
single_neonate_records = *BackwardFill*(single_neonate_records) *//Fill the null values using backward filling*
ELSE
single_neonate_records = *ForwardFill*(single_neonate_records) *//Fill the null values using forward filling*
filled_dataset.*append*(single_neonate_records)
END IF
END FOR
5. **RETURN** filled_dataset.

3.2.3. Handling irregular time interval

Most time-series features were entered with irregular time intervals. However, most machine learning techniques cannot handle such kinds of data. We proposed *Algorithm* 2, which introduces a new method to handle the irregular time interval. Note that we only consider the first 24 h of data for every patient. We tried to get 24 measurements for each patient within a specific 24 h. However, some patients have>24 h of measurements, while some have<24 h of data. For those with>24 measurements, we reduced the number of measurements by taking the average values of the nearest records and removing one of them, as shown in Fig. 4. We inserted new records for those with<24 measurements by generating them based on existing records. First, we generated random dates within the given range, and inserted new records with those dates and null values. Then, we filled those null values using the

Table 3
Selected tables and columns with their description.

Table Selected	Column Selected	Content	Extra Details
Patient	Subject-ID	Unique identifier for each patient.	Single value features were extracted from this table: gender, flag for whether the patient survived or not.
	Expire Flag	Boolean flag shows whether the patient has died or not.	
	Gender	Indicates the gender of the patient	
Admissions	HADM-ID	Unique identifier for each admission	Using patients' first admission date, the ages of the patients were found, and patients with ages between 0 and 1 are extracted for our paper. Admission location and type of insurance data were extracted from this table.
	Admission-time	Time when the patient was admitted	
	Discharge-time	Time when the patient was discharged	
ChartEvents	Chart-time	Time when the measurement was recorded	Item IDs for the time-series features: 211, 834, 3,603, 8,518, 3,450, 8,532, 3,609, 8,502, 3,312, 3,313, 3,655, 1,191, 8,537.<br>Item IDs for single value features: 3,,734, 3754, 3,768, 3,771, 3,780, 3,789, 3,451, 3,723, 3,834, 3,446.
	Item-ID	Indicates the type of measurement	
	Value	The value of measurement	
InputEvents	Chart-time	Time when the measurement was recorded	Item IDs: 30,187 (for D10W)<br>How many times the patient took D10W, how much D10W they took were calculated, and the following statistical features were generated: D10W Count, D10W Sum, D10W Mean.
	Item-ID	Indicates the type of measurement	
	Amount	Indicates how much liquid was entered	
OutputEvents	Chart-time	Time when the measurement was recorded	Item IDs: 43,175 (for Urine). How many times the patient's Urine was taken, how much Urine was taken were calculated, and the following statistical features were generated: Urine Count, Urine Sum, Urine Mean.
	Item-ID	Indicates the type of measurement	
	Amount	Indicates the amount of output	
DateTimeEvents	Chart-time	Time when the change was recorded	How many times equipment was changed is calculated as a statistical feature: Date Events Count
MicrobiologyEvents	Chart-time	Time when the test was taken.	How many times the patient took the microbiological test was calculated and the statistical feature was generated: microbiology test count.
	Organism-ID	Indicates the ID of the existing organism. If null, means there is not an organism.	
Prescriptions	Chart-time	Time when the prescription was taken	How many times the patient took prescription was calculated as a statistical feature: Prescriptions Count.
D-items	Label	Name of measurement	This table was used as a dictionary table.

filling algorithms, i.e., *Algorithm* 1. The algorithm analyzes each patient's data separately, so as not to mix their data with other patients' data, which is the main problem of other techniques. In our algorithm, while reducing the number of records, we used the values of removing records as much as possible.

Fig. 4 (a) shows the process of reducing the number of records for patients who have>24 records. First, the algorithm receives the data of a single neonate. Then it calculates the number of extra records and sorts the records by their date. Following that, the algorithm creates a new column called diff (standing for difference) and calculates the difference between the value of the record and the value of the previous record, and stores the value of the difference in the diff column. After calculating the differences between records, it selects N (number of extra records) smallest differences. Next, the algorithm calculates the average value of pair records with the smallest differences, stores the average values to the second record, and removes the first records.

Fig. 4 (b) shows the process of expanding the number of records for patients who have<24 records. First, the algorithm receives the data of a single neonate. Then it calculates how many records it must add, and sorts the records by their date. Following that, the algorithm generates N (shortage) dates within the given time range (between the maximum and minimum dates). After generating N random dates, the algorithm creates N new records (each record consists of neonate ID, chart time, and value). It creates the records with generated dates as chart time, and NULL as the value. After generating N records, the algorithm fills the NULL values of generated records using *Algorithm* 1.

Algorithm 2: Handling Irregular Time Intervals

Input: Dataset containing one time-series feature: *D*
Output: balanced_dataset // Balanced dataset
1. Sort dataset by neonate ID and chart time
2. Add new column 'row' that indicates row number for each neonate
3. Make a list of all unique neonates ID:*L*
4. Balance each neonate's rows separately:
balanced_dataset = []
FOR id **IN** L **DO**
single_neonate_records=*D*[*id*]
count = *length*(single_neonate_records)
IF (count > 24) **THEN**
extra_measurements = count − 24
ELSE
shortage_measurements = 24 - count
IF shortage_measurements > 0 **THEN**
//Create n (shortage_measurements) random dates between first and last chart time
generated_dates = *date_range*(start_date, end_date, periods = shortage_measurements)
FOR k = 0 **TO** shortage_measurements **DO**
// Create new rows for generated dates with null values
single_neonate_records[count + k] = [id, generated_dates[k], null]
END FOR
sort_values(single_neonate_records, by = charttime)
fillByForwardBackward(single_neonate_records) *// Fill null values using filling algorithms*
IF extra_measurements > 0 **THEN**
sort_values(single_neonate_records, by = charttime)
single_neonate_records['diff'] = 0 *//Create new column: diff*
FOR k = 1 **TO** count **DO**

(*continued on next page*)

(*continued*)

Algorithm 2: Handling Irregular Time Intervals

```
// Fill the column diff with the difference between the value of current row with the
   value of previous row
single_neonate_records['diff'] = abs(single_neonate_records['value'][k] -
   single_neonate_records['value'][k − 1])
END FOR
// Make a list of n (extra_measurements) rows with the smallest diffs: drop_list
smallest_diffs_indices = nsmallest(single_neonate_records['diff'], extra_measurements)
drop_list = single_neonate_records[smallest_diffs_indices]
FOR row = 0 TO count DO
IF single_neonate_records[row] IN drop_list THEN
//Calculate an average value of row (in the drop list) with a value of the nearest row and
   assign the average value to the nearest row
single_neonate_records[row + 1]:= avg(single_neonate_records['value'][row],
   single_neonate_records['value'][row + 1])
END FOR
FOR row IN drop_list DO
drop(row) //Drop the row (in the drop list)
END FOR
balanced_dataset.append(single_neonate_records)
RETURN balanced_dataset.
FUNCTION fillByForwardBackward(single_neonate_records):
IF (single_neonate_records['row'] == 1) AND (single_neonate_records['value'] ==
   null) THEN
backwardfilling(single_neonate_records) //Fill the null values using backward filling
ELSE
forwardfilling(single_neonate_records) //Fill the null values using forward filling
END IF
RETURN single_neonate_records.
```

3.2.4. Feature generation

Statistical features can be collected from time-series data to provide a global summary of the knowledge embedded in these time-series [105]. These calculated features can be used to train classical and ensemble ML models. We extracted representative statistical features from 13 time-series features, including Heart Rate, Heart Rate Alarm High, Heart Rate Alarm Low, SaO2, SaO2 Alarm High, SaO2 Alarm Low, Respiratory Rate, Skin Temperature, Temperature, Blood Pressure Cuff Mean, Blood Pressure Cuff Diastolic and Blood Pressure Cuff Systolic. From each time-series feature, we calculated mean, minimum, maximum, variance, and standard deviation.

Along with the aforementioned time-series features, we have extracted relevant futures that the domain expert recommended to include, but we only calculated a smaller number of statistical functions over these futures, namely count, sum, and average. For example, the DATEEVENTS table includes date and time events for some medical activities (e.g., the date of the last dialysis) that we count for the first 24 h. These date/time events are important based on the domain expert recommendation. Also, the PRESCRIPTIONS table had events about a medical prescription that we count for the first 24 h, to know how many times the neonate took the prescription. We have two other important tables that we utilized for creating statistical features (i.e., INPUTEVENTS and OUTPUTEVENTS tables). From the INPUTEVENTS table, we calculated three statistical features (i.e., count, average, and total amount) over the D10W (Dextrose 10 % in Water) time-series for the first 24 h. From the OUTPUTEVENTS table, we found that the urine time series is mostly used as the output type for neonates. Therefore, we calculated the count, average, and total amount of urine for each neonate in the first 24 h. From the MICROBIOLOGYEVENTS table, we calculated two statistical features (i.e., count and count of negative results) for the microbiology test in the first 24 h.

3.3. Data preparation

In this section, we discuss the data splitting, data balancing, and data normalization steps. These are well-known and important steps to implement a robust and accurate ML model.

3.3.1. Data splitting

Our dataset is large, compared to the literature studies. In addition, our models are based on regular machine learning models. However, we utilize an accurate method to divide the dataset into training, validation, and testing. The dataset is divided into 70 % and 30 % ratios for training and testing, respectively. The testing set is separated from the training set very early in the machine learning pipeline, to prevent the information leakage problem. Please note that applying missing values imputation before splitting does not affect the accuracy of the models. The 10-fold cross-validation technique was used with the 70 % partition to train and optimize the machine learning algorithms. The 70 %-30 % division process is repeated 10 times, and the average results with standard deviations are reported.

3.3.2. Data normalization

After data splitting, we apply the MinMax data normalization technique to convert the data into zero mean and one standard deviation, see Eq. (1). The fitted scaler on the training data is used directly to transform the testing data without refitting it on the test data. This step is critical to prevent data leakage problems and over-optimistic results [106].

$$x_{scaled} = \frac{x - x_{min}}{x_{max} - x_{min}} \tag{1}$$

3.3.3. Data balancing

Our dataset is highly imbalanced, with about a 64:1 ratio. We have 3085 records for the first class (i.e., survived) and 48 records for the second class (i.e., dead). Initially, we apply SMOTE, SMOTENC [107], and other balancing techniques that generate fake data, and we use these data to balance our dataset. Although the resulting dataset achieved high performance, generating fake data for data balancing is not recommended in the medical field, because physicians will not trust the results. Another method for data balancing is called under-sampling, where balancing is done by randomly reducing the size of the abundant class and keeping all samples in the rare class. Note that this method wastes a lot of information. As a result, researchers took advantage of the oversampling techniques. Therefore, we apply the oversampling technique [108] separately for training and testing datasets. This technique balances the dataset by increasing the size of rare samples by adding new samples from the rare class (for example, by repetition, bootstrapping, or a synthetic minority).

3.4. Classification layer

In this stage, we compare the performance of many classical machine learning (ML) algorithms (e.g., naive Bayes (NB), logistic regression (LR), k-nearest neighbors (KNN), decision tree (DT), SVM and MLP), static ensemble algorithms (e.g., random forest (RF), XGBoost, voting, AdaBoost, LightGBM, CatBoost, and gradient boosting (GB) classifiers), and the state-of-the-art dynamic ensemble selection (DES) algorithms (e.g., KNORA-E, METADES, DESP, KNORA-U, DESKNN, and KNOP) [50]. Classical ML algorithms have been tested with the MIMIC dataset for solving many ICU problems [109]. Ensemble modeling has better performance than its base classifiers [50]. Creating an ensemble has three main steps, namely generation (to create and train base classifiers), selection (to statically or dynamically select a set of base classifiers to build the ensemble), and integration (to combine the decisions of the base classifiers using techniques like majority voting), as shown in Fig. 5. The selection phase differentiates the static and dynamic ensemble classifiers. Because the ensemble of ML models produces more accurate models, many static ensemble models have been proposed in the literature using the MIMIC dataset to solve different ICU medical problems [57,110,58,111,44,112]. These ensembles are based on the static selection of base classifiers, as shown in Fig. 5. On the other hand, dynamic ensemble selection depends on selecting base classifiers on the fly according to each new sample. This technique estimates the

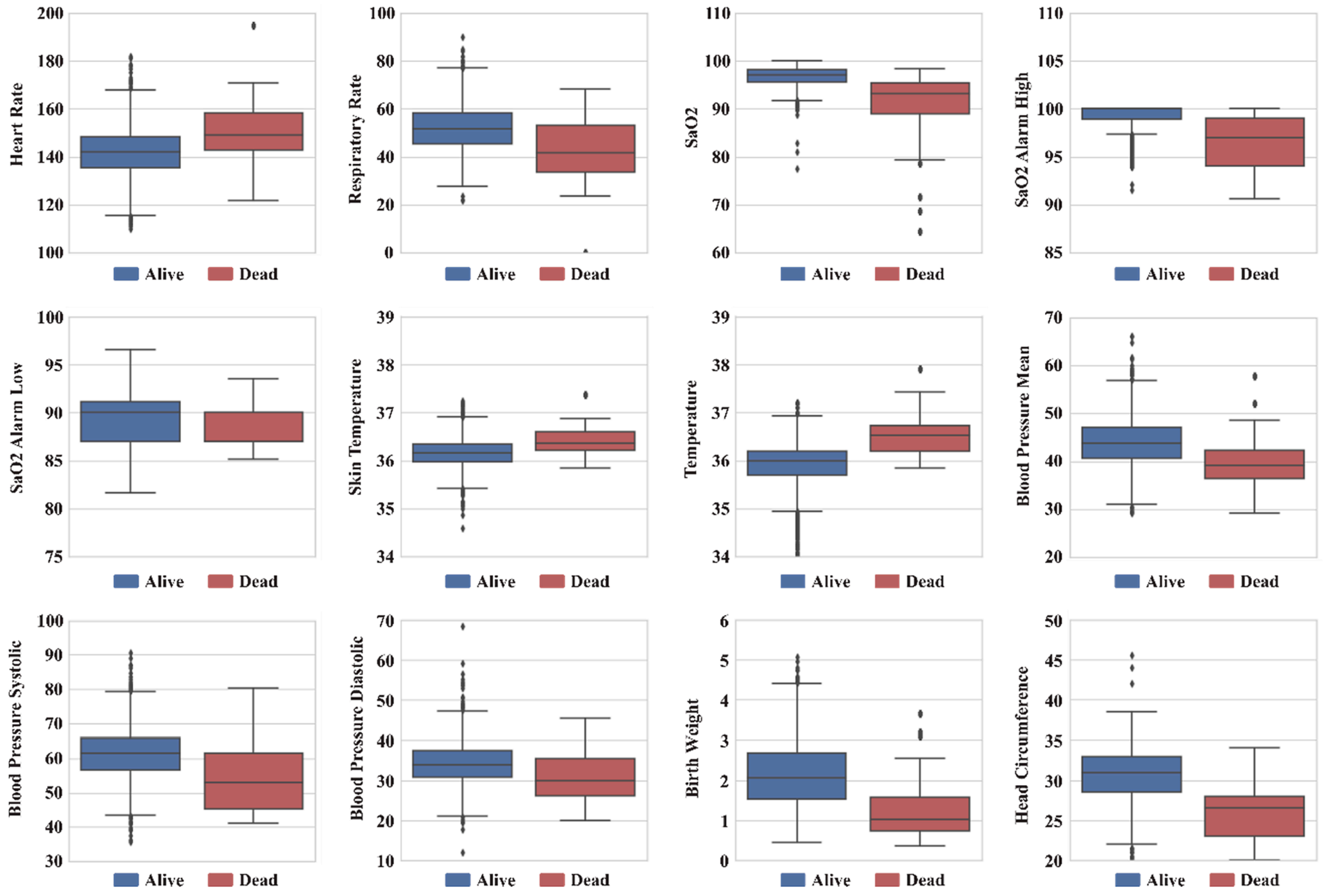

Fig. 3. Data distribution of the time-series data and single value data (birth weight and head circumference).

competence level of each classifier from a pool of classifiers, and the most competent ones are used to predict the label of the test sample, as shown in Fig. 5. Usually, the competence of the base classifiers is measured based on a local region of the feature space where the query sample is located. This region can be defined based on many techniques, such as KNN or clustering, and the competence is based only on samples of this region. This is because each base classifier is an expert in a specific local region of the feature space. Dynamic ensemble modeling has not been sufficiently explored in the ICU domain, especially using the MIMIC-III dataset. Recently, Guo et al. [28] proposed a DES model based on k-means clustering for mortality prediction. They compared the proposed model with other ensembles of base classifiers, and the proposed model achieved the best results. Many proposed DES models in the literature could improve the prediction of critical ICU problems [50]. These models have not been explored in the ICU domain, especially for neonates' mortality prediction. The exploration of these advanced DES algorithms has been done in the Alzheimer's domain by Muhammed and Thiyagarajan [62]. In this study, authors optimized many base classifiers like SVM, static ensembles like random forest, and DES models using grid search algorithm. The DESs achieved significantly better results than other techniques. Our study proposes an accurate and medically relevant data preparation pipeline. The resulting high-quality dataset is used to compare the previously mentioned classifiers. We evaluate the algorithms using default hyperparameters, optimized hyperparameters, without feature selection step, and with feature selection step. The 6 state-of-the-art dynamic selection

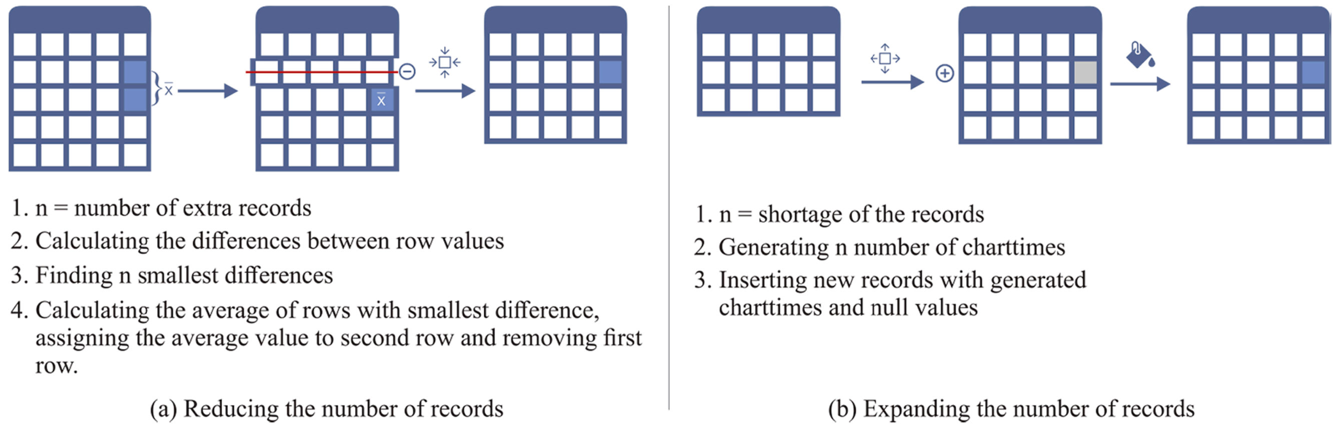

Fig. 4. Handling irregular time intervals for time series data.

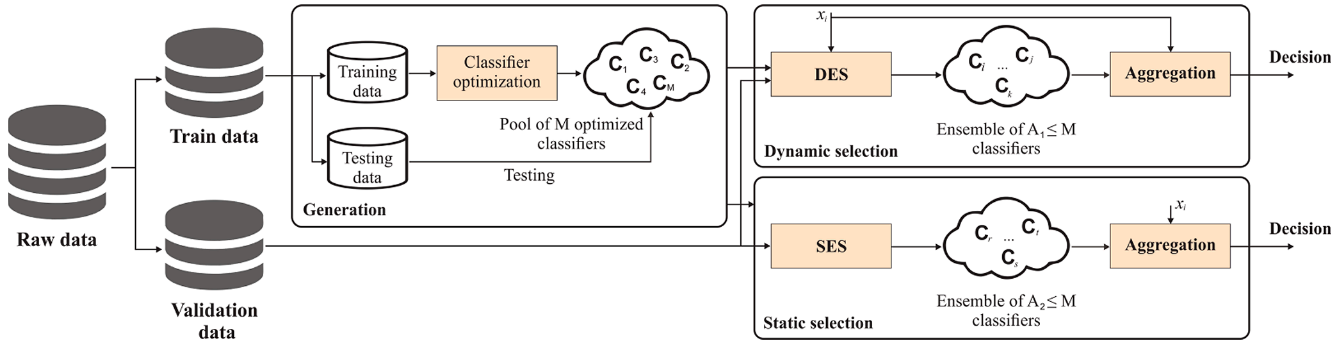

Fig. 5. Ensemble modeling techniques.

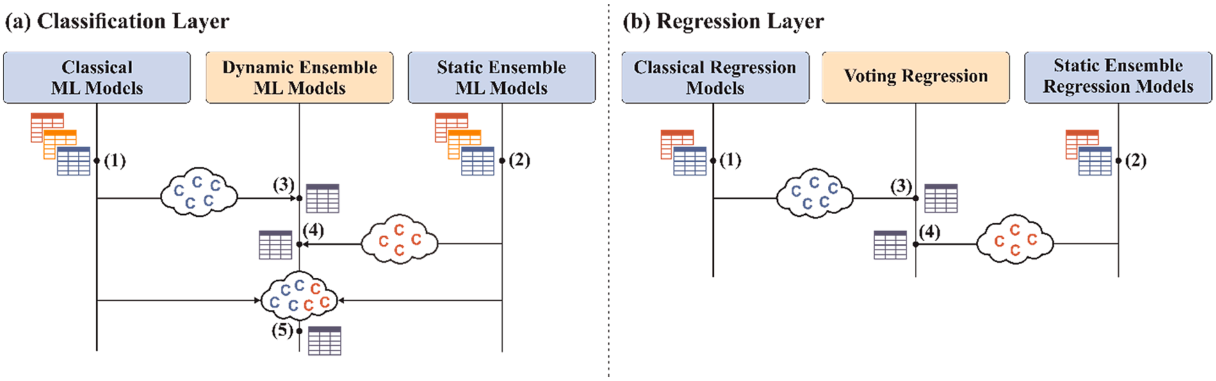

Fig. 6. Road map of experiments.

techniques include KNORA-E, KNORA-U, DESP, METADES, DESKNN, and KNOP (See Appendix A).

All explored ML models are tested using default hyperparameters and optimized hyperparameters based on grid search. In addition, selected machine learning algorithms are optimized based on the whole feature set. However, selecting the best list of more informative features for the classification or regression tasks improves the model performance. There are many feature selection techniques in the literature [113], and there is no rule of thumb for selecting the best one. We apply many different feature selection algorithms like MultiSURF, correlation, and mutual information. Based on our experiments, we found that the mutual information feature selection technique achieves the highest performance. Mutual information from the field of information theory is the application of information gain in feature selection. Mutual information is calculated between two variables and measures the reduction in uncertainty for one variable given the known value of the other variable.

3.5. Regression layer

The second layer in the proposed framework is a regression layer to predict the LoS for neonates at the ICU. In this stage, we compare the performance of different classical ML regression algorithms (e.g., K-nearest neighbors [114], Linear Regression, SVR, Decision Tree, Ridge, and Lasso [115]), static ensemble regression algorithms (e.g., Random Forest [115], CatBoost [116], XGBoost [117], LightGBM [118], Gradient Boosting Regressor, Extra Trees and AdaBoost), and voting of both classical regression models and static ensemble regression models.

4. Performance evaluation

The first stage was a classification task. The performance of the state-of-the-art DES algorithms was compared with other classical and static ensemble classifiers. Four standard metrics were used to evaluate the classification models, i.e., accuracy, precision, recall, and F1-score, which are regularly used in biomedical applications [52,105].

The second stage was a regression task. Three metrics were used to evaluate the regressor models: the mean absolute error (MAE), root mean squared error (RMSE), and R-squared (r^2) [52,105,119].

5. Experimental setup

In Fig. 6, the road map of our conducted experiments is illustrated for the classification and regression layers. We conducted nine experiments (five in the classification layer, and four in the regression layer). These experiments search for the best classifier and regressor for mortality prediction and LoS prediction, respectively. Our strategy is to test the models using default hyperparameters, feature selection optimization, hyperparameters optimization, the best number of based classifiers for both dynamic and static ensemble models, and combinations of the previous options. The selection of models is based mainly on the statistical comparison of the models' performance. In our classification layer in Fig. 6 (a) at left: (1) denotes that the single machine learning classifiers are tested, and three separate results are reported, i.e., one without feature selection and hyperparameter optimization, one with the feature selection step, and one with both the feature selection and hyperparameter optimization steps. In the second step, (2) denotes those experiments are conducted with static ensemble ML classifiers, and the three results are reported as in (1). In the third step, (3) denotes that dynamic ensemble selection is applied with a single machine learning classifiers' pool, and the results are reported. Note that all based classifiers and DES models are optimized. In the fourth step, (4) denotes that DES is applied with a static ensemble machine learning classifiers pool, and the results are collected. In the last step, (5) denotes that DES is used with the mixed pool of single and static ensemble machine learning classifiers, and the results are collected.

In the regression layer of Fig. 6 (b) at right, (1) denotes that the single regression models are tested, and the results are reported. The results are

Table 4
Classical ML classifiers model results with feature selection and hyperparameter optimization.

Models	Accuracy	Precision	Recall	F1-score
NB	0.905 ± 0.001	0.955 ± 0.001	0.849 ± 0.003	0.899 ± 0.002
LR	0.953 ± 0.002	0.913 ± 0.003	1.000 ± 0.000	0.955 ± 0.002
KNN	0.890 ± 0.002	0.979 ± 0.002	0.797 ± 0.004	0.879 ± 0.003
DT	0.931 ± 0.002	0.928 ± 0.003	0.934 ± 0.003	0.931 ± 0.002
**SVC**	**0.958 ± 0.002**	**0.922 ± 0.004**	**1.000 ± 0.000**	**0.959 ± 0.002**
MLP	0.954 ± 0.006	0.916 ± 0.010	1.000 ± 0.000	0.956 ± 0.005

collected before and after applying the feature selection and hyperparameter optimization steps. In the second step, (2) denotes that the same experiments are conducted with the static ensemble regressors. In the third and fourth (b) steps, (3) and (4) denote that the voting regression model is tested with pools of single and static ensemble regression models, respectively, and the results are reported.

6. Results of classification

In this section, we discuss the results of the classification layer. This section has three subsections. First, we discuss the results of the classical ML models. Then, we discuss the results of the static ensemble models and the results of the dynamic ensemble models. We collect and report the testing results for all models. We used the 10-holdout testing technique to obtain more stable results. The results are reported as (*mean* ± *standard deviation*). *Note that we discuss the results using the F1-Score only because it is representative of other metrics.* For more details of the other performance metrics, readers can consult the corresponding tables.

6.1. Classical ML models

We test the classical ML models without using feature selection and hyperparameter optimization *(see Appendix B.1)*, using the feature selection step only *(see Appendix B.2)*, and using both feature selection and hyperparameter optimization. In this section, we test the role of hyperparameter optimization to improve the performance of classical ML models. Note that this step is applied based on the results of the previous experiments, i.e., after the feature selection step. The hyperparameters of every ML model are optimized using grid search techniques. As expected, the performance of most ML models is significantly improved as illustrated in Table 4. Surprisingly, SVC achieved the best results after optimizing its hyperparameters (F1-score of 0.958 ± 0.002). In addition, the model is more stable. The F1-score of NB, LR, DT, SVC, and MLP is improved by (1.6, 3.2, 34.6, 12.3, and 17.1) %, respectively. The performance of KNN is not affected by the hyperparameter optimization step, and the model achieved the same F1 score. Figs. 7 and 8 summarize the results of this stage by illustrating the performance of ML models with and without feature selection and hyperparameter optimization steps. The optimized hyperparameters for these classifiers can be found in Table S1 of the Supplementary Information (SI).

6.2. Static ensemble ML models

In this section, we optimize a set of seven popular static ensembles, namely AdaBoost, XGBoost, RF, GB, CatBoost, LGBM, and majority voting with a pool of five DT classifiers. Each technique is implemented and tested (1) using default hyperparameters and without feature selection *(see Appendix B.3),* (2) after feature selection step *(see Appendix B.4)*, and (3) after feature selection and hyperparameter optimization. Once we add the hyperparameter optimization step to the machine learning pipeline, the performance is enhanced for all static ensemble models, as shown in Table 5. After optimizing its hyperparameters, RF achieved the best results (i.e., F1-score of 0.959 ± 0.002). Note that all

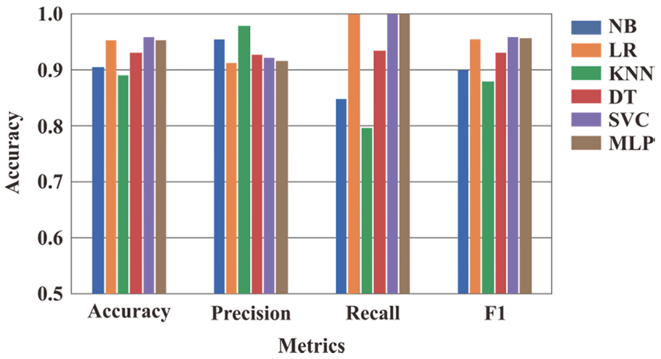

Fig. 8. Comparison between the performances of different classical ML classifiers.

Table 5
Static ensemble ML classifiers model results with feature selection and hyperparameter optimization.

Models	Accuracy	Precision	Recall	F1-score
AdaBoost	0.947 ± 0.003	0.904 ± 0.005	1.000 ± 0.000	0.950 ± 0.003
XGBoost	0.919 ± 0.011	0.916 ± 0.010	0.922 ± 0.026	0.919 ± 0.012
**RF**	**0.957 ± 0.004**	**0.921 ± 0.007**	**1.000 ± 0.000**	**0.959 ± 0.004**
GB	0.910 ± 0.018	0.931 ± 0.009	0.886 ± 0.037	0.908 ± 0.020
CatBoost	0.949 ± 0.003	0.908 ± 0.005	1.000 ± 0.000	0.952 ± 0.003
LGBM	0.940 ± 0.002	0.965 ± 0.003	0.914 ± 0.003	0.939 ± 0.002
Majority Voting	0.933 ± 0.010	0.927 ± 0.003	0.941 ± 0.020	0.934 ± 0.010

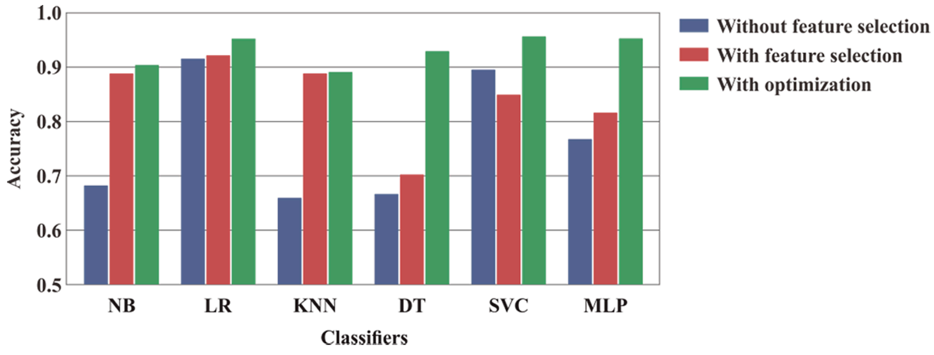

Fig. 7. Performance comparison of classical classifiers: with and without feature selection and optimization.

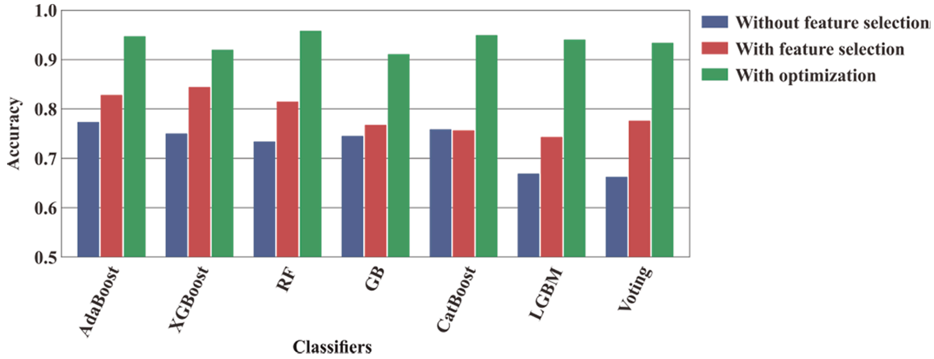

Fig. 9. Performance comparison between the of classifiers (with and without) feature selection and optimization.

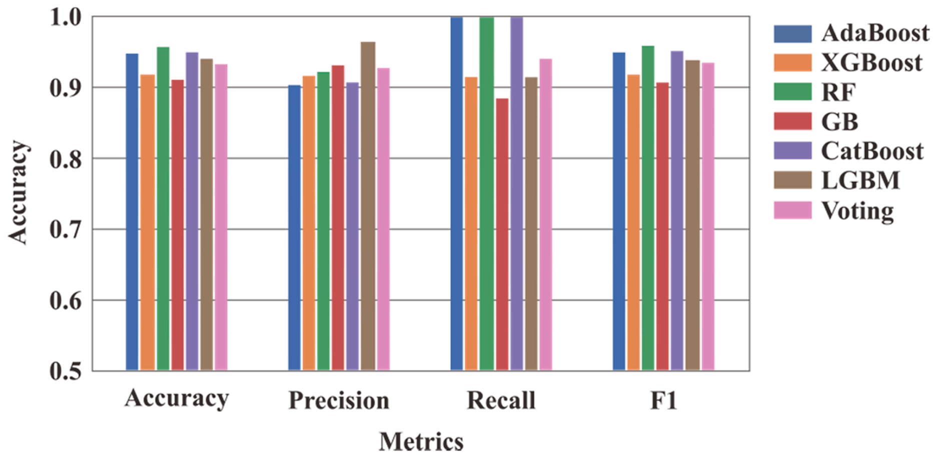

Fig. 10. Comparison between the performances of different classifiers after the optimization.

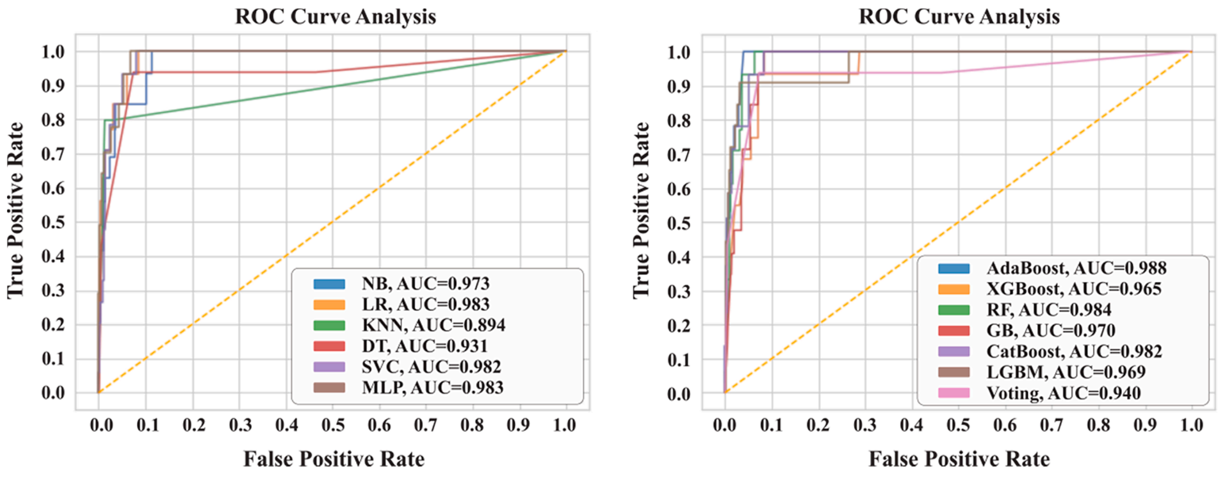

Fig. 11. AUC score for all classifiers with feature selection and hyperparameter optimization.

models become more stable after optimizing their hyperparameters. Fig. 9 compares the results of before and after adding optimization steps. Fig. 10 illustrated the performance metrics of all the optimized models of static ensemble classifiers where RF achieved the best results. Fig. 11 compares the AUC of classical and static ensemble models. Table S2 of the SI shows the optimized hyperparameters for these classifiers.

The feature selection step highlighted the critical roles of some features. Tables S3 and Table S4 in the supplementary materials of the SI show the most important features for different regular ML and static ensemble models, and the selected features by all models. Common features among classifiers are highlighted in bold. These features should have played a critical medical role in the real ICU environment to predict neonate mortality. Statistical comparison among different classifiers is performed based on the Friedman test. The critical diagrams for both classical and static ensemble classifiers are shown in Fig. 12 shows the critical diagrams for both classical and static ensemble classifiers.

Fig. 13 compares the performance of classical and static ensemble classifiers. As seen in the figure, SVC achieves the best results, and KNN achieves the worst results. Fig. 14 shows the statistical comparison between all elements using the Friedman test.

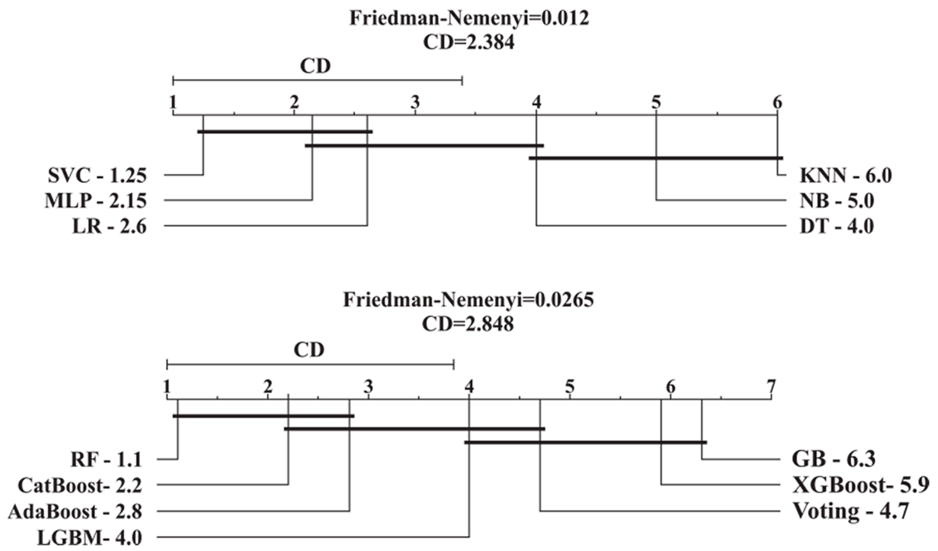

Fig. 12. Comparison of all classifiers based on the Friedman test.

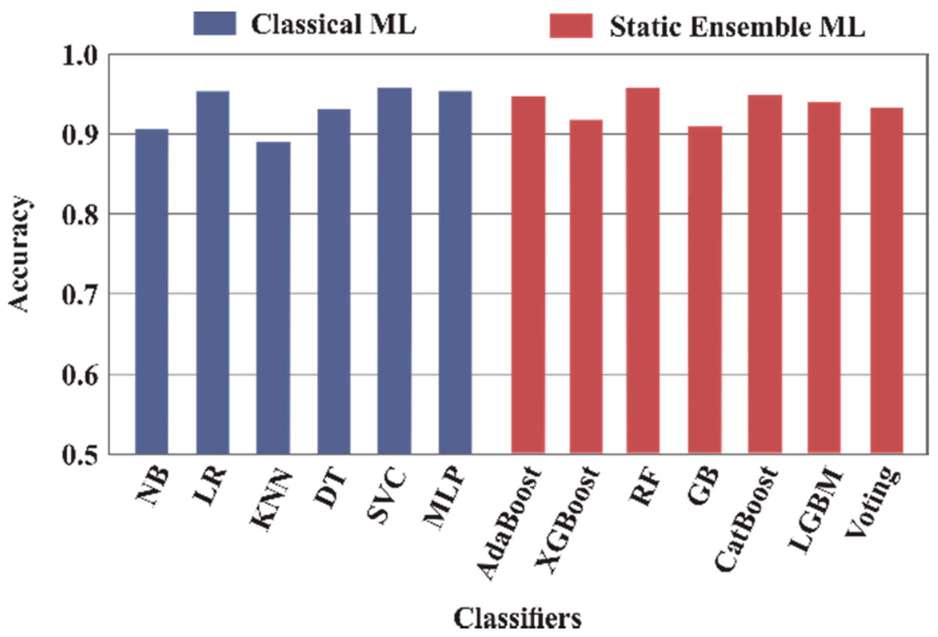

Fig. 13. Comparison of all classical and static ensemble classifiers.

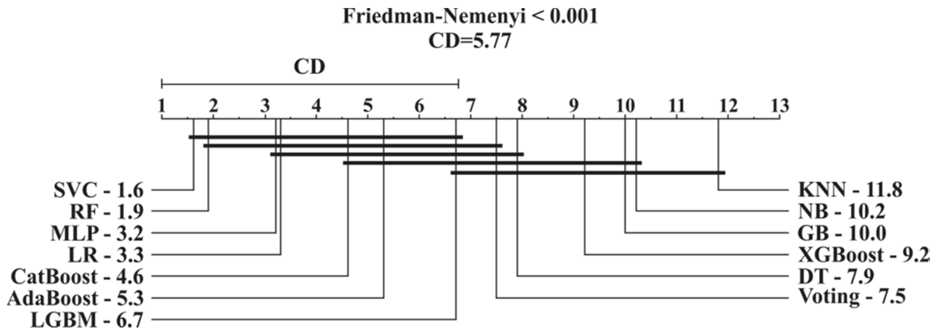

Fig. 14. Comparison of all classical and static ensemble machine learning classifiers based on the Friedman test.

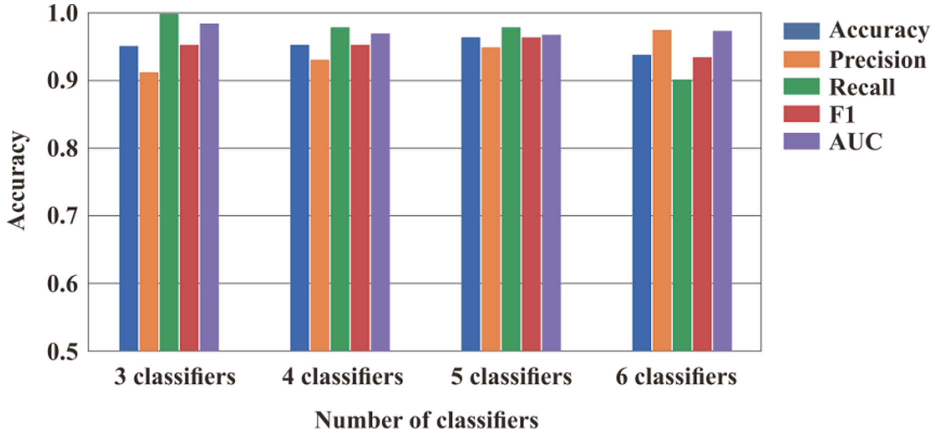

Fig. 15. Comparison between the DES with classical ML classifiers pool with different numbers of classifiers.

Table 6
Dynamic Ensemble Selection model results with classical ML classifiers pool.

Models	Accuracy	Precision	Recall	F1-score
FIRE-KNORA-U	0.971 ± 0.001	0.945 ± 0.002	1.000 ± 0.000	0.972 ± 0.001
KNORA-U	0.967 ± 0.002	0.938 ± 0.003	1.000 ± 0.000	0.968 ± 0.002
FIRE-KNORA-E	0.940 ± 0.000	0.964 ± 0.001	0.914 ± 0.000	0.938 ± 0.000
KNORA-E	0.942 ± 0.000	0.969 ± 0.001	0.914 ± 0.000	0.941 ± 0.000
FIRE-METADES	0.957 ± 0.014	0.931 ± 0.002	0.987 ± 0.028	0.958 ± 0.014
METADES	0.942 ± 0.010	0.942 ± 0.003	0.941 ± 0.021	0.941 ± 0.011
**FIRE-DESKNN**	**0.987 ± 0.001**	**0.975 ± 0.001**	**1.000 ± 0.000**	**0.988 ± 0.001**
DESKNN	0.986 ± 0.001	0.973 ± 0.001	1.000 ± 0.000	0.987 ± 0.000
FIRE-DESP	0.976 ± 0.001	0.954 ± 0.002	1.000 ± 0.000	0.976 ± 0.001
DESP	0.973 ± 0.002	0.949 ± 0.003	1.000 ± 0.000	0.974 ± 0.002
FIRE-KNOP	0.959 ± 0.001	0.924 ± 0.002	1.000 ± 0.000	0.960 ± 0.001
KNOP	0.959 ± 0.018	0.938 ± 0.003	0.983 ± 0.036	0.959 ± 0.019

6.3. *Dynamic ensemble ML models*

In this section, we explore the capabilities of dynamic ensembles in the ICU environment. This is the first study in the literature that implements dynamic ensembles in the ICU environment, especially for neonate mortality prediction.

6.3.1. *Selecting the best number of classical classifiers*

Building dynamic ensembles require selecting the best number and types of base classifiers. Note that the list of base classifiers is the same as that we optimized in Section 6.1. We tested the performance of dynamic ensembles based on the best three, best four, best five, and all six base classifiers. The details of the experiments are included in *Appendix B* Table B5. We noticed that the best results are with the selecting of 5 classifiers as shown in Fig. 15. Using the FIRE DESKNN, the selected base classifiers that achieved the highest DES performance are MLP, LR, SVC, DT, and NB.

6.3.2. *Results of DES with a pool of classical ML models*

In this experiment, we tested the performance of the 12 DES techniques based on the previously selected five base classifiers, see Table 6. As mentioned in the previous experiment, the FIRE-DESKNN achieves the best results (F1-score of 0.988 ± 0.001, P-value < 0.001). The DESKNN algorithm achieves a similar performance (0.987 ± 0.000). The improved performance of the FIRE-DESKNN algorithm is the result of adding the FIRE capability that implements the Dynamic Frienemy Pruning [120] to the DESKNN algorithm. Fig. 16 shows the AUC scores and critical diagram for statistical comparison of the DES models.

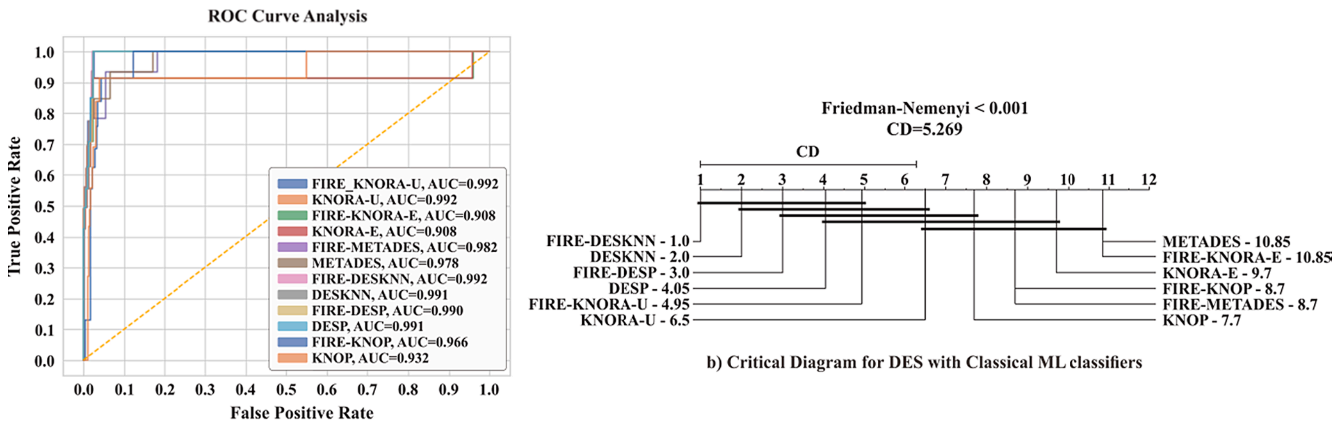

a) ROC Curve for DES with Classical ML classifiers

b) Critical Diagram for DES with Classical ML classifiers

Fig. 16. AUC and statistical comparison of the DES models based on classical ML models.

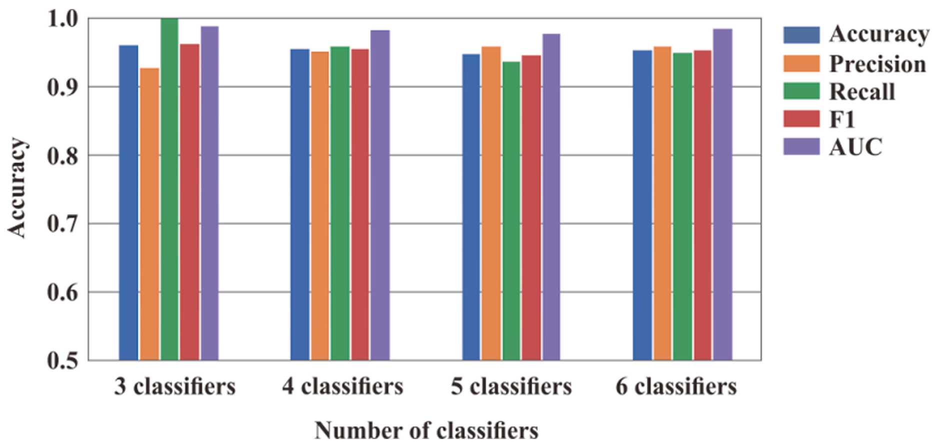

Fig. 17. Comparison of DES models with different pools of static ensemble classifiers.

Table 7
Dynamic DES model results with static ensemble ML classifiers pool.

Models	Accuracy	Precision	Recall	F1-score
FIRE-KNORA-U	0.959 ± 0.003	0.925 ± 0.005	1.000 ± 0.000	0.961 ± 0.003
KNORA-U	0.959 ± 0.003	0.924 ± 0.005	1.000 ± 0.000	0.960 ± 0.003
FIRE-KNORA-E	0.962 ± 0.002	0.929 ± 0.003	1.000 ± 0.000	0.963 ± 0.002
KNORA-E	0.962 ± 0.002	0.930 ± 0.003	1.000 ± 0.000	0.964 ± 0.001
FIRE-METADES	0.965 ± 0.002	0.935 ± 0.004	1.000 ± 0.000	0.966 ± 0.002
**METADES**	**0.965 ± 0.003**	**0.935 ± 0.005**	**1.000 ± 0.000**	**0.967 ± 0.003**
FIRE-DESKNN	0.955 ± 0.005	0.918 ± 0.009	1.000 ± 0.000	0.957 ± 0.005
DESKNN	0.958 ± 0.004	0.922 ± 0.007	1.000 ± 0.000	0.959 ± 0.004
FIRE-DESP	0.961 ± 0.003	0.928 ± 0.005	1.000 ± 0.000	0.963 ± 0.002
DESP	0.961 ± 0.002	0.929 ± 0.004	1.000 ± 0.000	0.963 ± 0.002
FIRE-KNOP	0.960 ± 0.003	0.926 ± 0.005	1.000 ± 0.000	0.962 ± 0.003
KNOP	0.962 ± 0.003	0.929 ± 0.005	1.000 ± 0.000	0.963 ± 0.003

6.3.3. *Selecting the best number of static ensemble classifiers for DES*

We explored the possibility of enhancing the performance of DES algorithms by using static ensemble models as the pool of classifiers of the DES models. We found that FIRE METADES with the three static ensemble models (i.e., *AdaBoost*, *RF*, and *CatBoost*) achieves the best accuracy of 0.965 ± 0.003. The detail of the experiments is included in *Appendix B* Table B6. Fig. 17 shows a comparison of the different DES models based on different pools of classifiers.

6.3.4. *Results of DES with a pool of static ensemble models*

In this experiment, we discuss the detailed results of the 12 DES models that are optimized based on the three selected base static ensemble models. As shown in Table 7, the highest results are achieved by the METADES model (F1-score of 0.967 ± 0.003, P-value < 0.001). There are no significant differences between the 12 DES models. Note that using the classical base models in Section 6.3.2 as the DES pool achieves higher results than using the advanced static ensemble models. As a result, it can be concluded that using simple heterogeneous base classifiers can create a powerful DES model. Fig. 18 shows the AUC scores and the critical diagram for statistical comparison of the DES models.

6.3.5. *Selecting the best number of classifiers for a mixed pool of classical and static ensemble models*

We optimized the performance of DES using a pool of heterogeneous optimized classical and static ensemble models.

We tested different pool sizes from four to ten base classifiers and included the detail of the experiments in *Appendix B* Table B7. The best

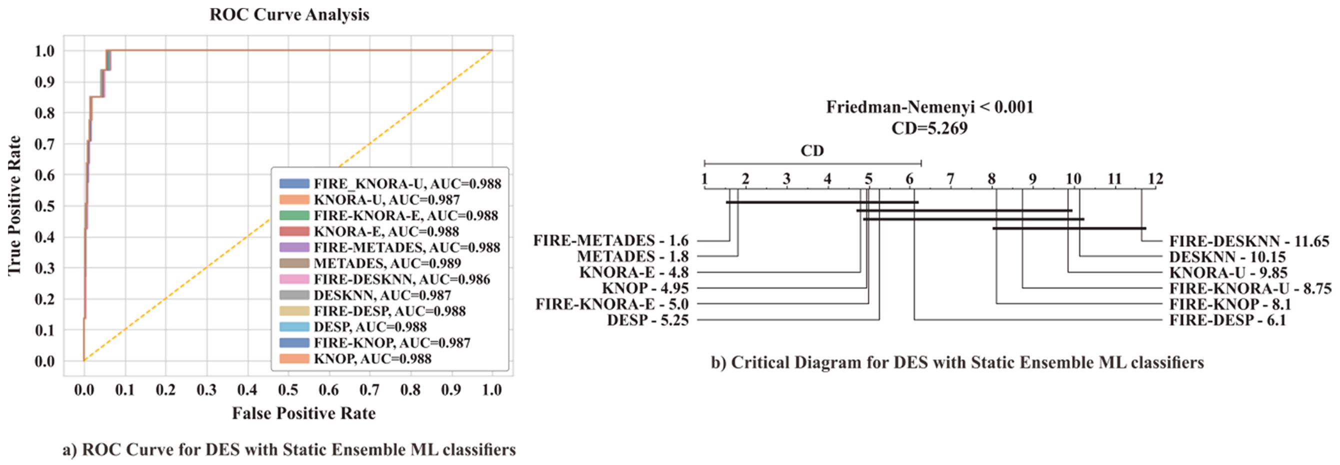

Fig. 18. AUC and statistical comparison of the DES models based on static ensemble models.

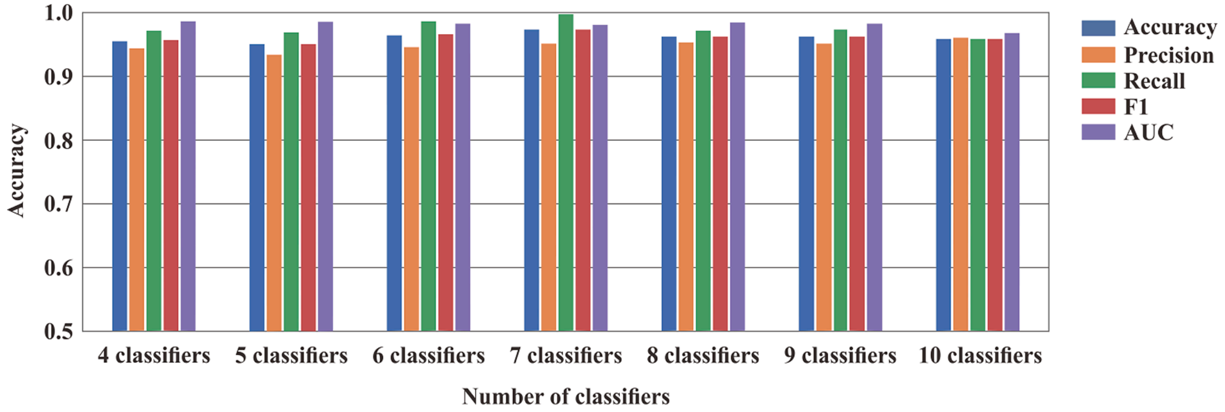

Fig. 19. Comparison of DES models with a pool of mixed classifiers from classical and static ensemble models.

Table 8
DES model results with the mixed pool of classical and static ensemble ML classifiers.

Models	Accuracy	Precision	Recall	F1-Score
FIRE-KNORA-U	0.970 ± 0.002	0.943 ± 0.004	1.000 ± 0.000	0.971 ± 0.002
KNORA-U	0.968 ± 0.002	0.941 ± 0.004	1.000 ± 0.000	0.969 ± 0.002
FIRE-KNORA-E	0.980 ± 0.001	0.962 ± 0.002	1.000 ± 0.000	0.980 ± 0.001
**KNORA-E**	**0.980 ± 0.001**	**0.962 ± 0.002**	**1.000 ± 0.000**	**0.981 ± 0.001**
FIRE-METADES	0.976 ± 0.011	0.960 ± 0.003	0.994 ± 0.021	0.977 ± 0.011
METADES	0.971 ± 0.015	0.957 ± 0.004	0.986 ± 0.030	0.972 ± 0.016
FIRE-DESKNN	0.972 ± 0.004	0.948 ± 0.003	0.999 ± 0.007	0.973 ± 0.004
DESKNN	0.972 ± 0.004	0.947 ± 0.003	0.999 ± 0.007	0.973 ± 0.004
FIRE-DESP	0.972 ± 0.002	0.948 ± 0.004	1.000 ± 0.000	0.973 ± 0.002
DESP	0.971 ± 0.002	0.946 ± 0.004	1.000 ± 0.000	0.972 ± 0.002
FIRE-KNOP	0.972 ± 0.011	0.953 ± 0.003	0.994 ± 0.023	0.973 ± 0.012
KNOP	0.969 ± 0.011	0.948 ± 0.003	0.994 ± 0.023	0.970 ± 0.012

performance is achieved by the KNORA-E DES model (average accuracy of 0.973 ± 0.004 and best accuracy of 0.980), and the model is stable. Fig. 19 illustrates the performance of different combinations of base classifiers.

6.3.6. *Results of DES with a mixed pool of classical and static ensemble models*

Based on the selected numbers and types of base classifiers in Section 6.3.5, which achieved the highest average accuracy, this experiment explores the performance of the 12 DES models. All models have improved performance compared with using classical or static ensemble models. KNORA-E achieved the highest results (F1-score of 0.981 ± 0.001), see Table 8. One main reason for that performance is that KNORA-E is very strict in selecting classifiers, because it selects a classifier if it correctly predicts all neighbors of the given test sample. By adding the FIRE feature to KNORA-E (FIRE-KNORA-E), the resulting model achieves the next highest performance (F1-score of 0.980 ± 0.001). FIRE is used when there are many indecision regions. Indecision regions are regions where there are samples of different classes; however, our dataset has not many indecision regions with high probability, because classical ML models can perform well, such as LR (>95 %) and DT (>93 %). In addition, these models have the lowest standard deviation in their performance, which means they are more robust. Fig. 20 shows the AUC scores for DES models using mixed models.

Table S5 of the SI shows the most critical features that are selected by the best DES models when the pool of classifiers has classical ML models, static ML models, or mixed models. Table S6 of the SI shows the optimized hyperparameters of the selected DES models. As a result, the best

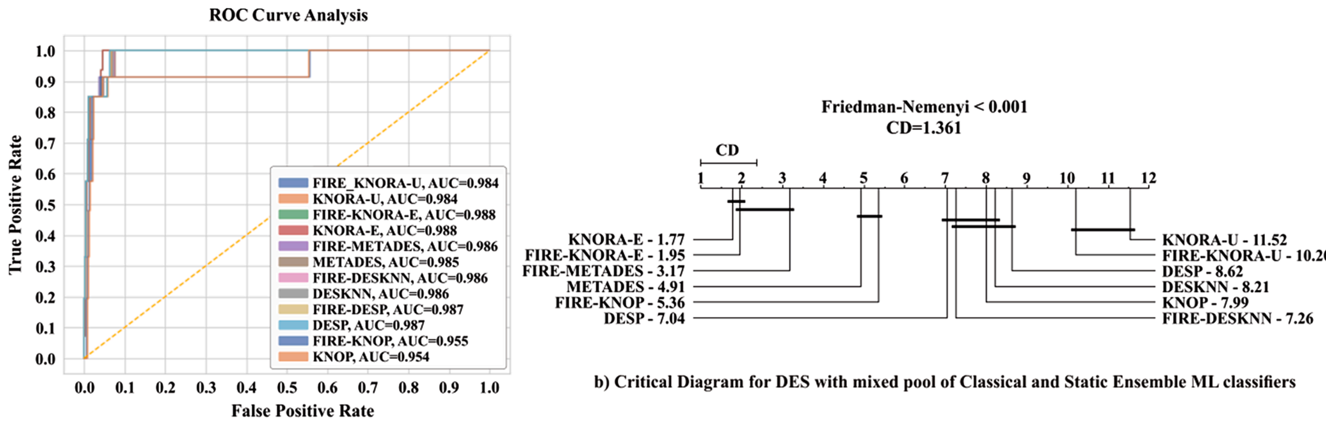

a) ROC Curve for DES with mixed pool of Classical and Static Ensemble ML classifiers

b) Critical Diagram for DES with mixed pool of Classical and Static Ensemble ML classifiers

Fig. 20. AUC and statistical comparison of the DES models based on mixed models.

Table 9
Classical regression models results without feature selection and hyperparameter optimization.

Models	RMSE	MAE	R2
KNNR	24.168 ± 1.452	16.427 ± 0.552	22.566 ± 0.930
LiR	16.180 ± 0.620	11.663 ± 0.405	65.293 ± 0.266
SVR	20.090 ± 31.69	14.308 ± 25.539	45.299 ± 17.712
DTR	20.194 ± 8.076	13.209 ± 4.961	45.861 ± 4.354
Ridge	16.176 ± 0.620	11.657 ± 0.397	65.312 ± 0.266
**Lasso**	**16.019 ± 0.378**	**11.383 ± 0.318**	**65.982 ± 0.161**

Table 10
Classical regression models results with feature selection and hyperparameter optimization.

Models	RMSE	MAE	R2
KNNR	18.155 ± 1.191	11.730 ± 0.593	53.561 ± 0.610
**LiR**	**14.818 ± 0.251**	**10.828 ± 0.210**	**69.064 ± 0.105**
SVR	16.026 ± 0.847	10.782 ± 0.261	63.816 ± 0.383
DTR	15.669 ± 3.934	10.531 ± 1.583	65.391 ± 1.732
Ridge	14.822 ± 0.252	10.828 ± 0.219	69.048 ± 0.105
Lasso	14.852 ± 0.176	10.804 ± 0.200	68.922 ± 0.074

performing model from the classical ML category was SVC while RF was the best static ensemble classifier. FIRE-DESKNN achieved the best results compared to the other DES models. To evaluate the reliability of these best classifiers from different categories, both calibration and precision recall-plots have been used. Figure S1 in SI illustrates a comparison among the top three classifiers for each category, i.e., the classical ML, static ensemble, and dynamic ensemble models. The calibration subplots confirmed our results as they showed that SVC was the best compared to LR and MLP, RF was the best compared to Catboost and Adaboost, and FIRE-DESKNN was the best compared to DESP and FIRE-KNORA-U. Figure S2 compared the best model of each category. As clearly noticed, the FIRE-DESKNN had the best brier score (0.058, 0.099, 0.044 for SVC, RF, FIRE-DESKNN, respectively). Moreover, to measure the performance of the classifiers to detect the minority class (dead neonate), the precision-recall plot has been used to plot the relationship between precision and recall. Figures S3 and S4 in SI compared the performance of the best-selected models. Notice that the DES model (FIRE-DESKNN) achieved the best average precision (AP), i.e., 0.98, 0,98, 0.99 for SVC, RF, FIRE-DESKNN, respectively.

7. Results of regression

After predicting the mortality of neonates in the ICU, it is medically important to determine the LoS for the alive patients. This is critical information to be known by physicians because they can make special decisions based on this information, such as changing the ICU, or providing special care for patients that have a short LoS period. This section discusses the results of the second level of our proposed model (i.e., regression layer). To select the optimum regressor, we conducted five experiments. These experiments compare the performance of classical regressors, static ensemble regressors, and voting regressors. In the following, we discuss the details of these experiments. The performance of these models is evaluated using the RMSE, MAE, and R2 metrics.

7.1. Classical regression models

In this section, we discuss the optimization of classical regressors by feature selection and hyperparameter tuning steps. Six well-known classical regressors are tested, including KNN Regressor (KNNR), linear regression (LiR), linear SVR, DT regressor (DTR), Ridge, and Lasso.

7.1.1. Experiment 12: Regressor results without feature selection and hyperparameter optimization

Table 9 shows the results of the six regressors without adding the feature selection and hyperparameter optimization steps in the ML pipeline. Lasso achieved the best average results (RMSE = 16.019 ± 0.378, MAE = 11.383 ± 0.318, and R2 = 65.982 ± 0.161). Lasso regressor uses the L1 regularization technique, which adds a penalty equal to the absolute value of the magnitude of the coefficient. Larger regularization results in sparse models with few coefficients. KNNR achieved the worst results (RMSE = 24.168 ± 1.452, MAE = 16.427 ± 0.552, and R2 = 22.566 ± 0.930).

7.1.2. Experiment 13: Results after feature selection and hyperparameter optimization

This experiment adds the two optimization steps of feature selection and hyperparameter tuning. It can be noticed that these steps improved the performance of all regressors, see Table 10. LiR achieved the best results after applying these two steps (RMSE = 14.818 ± 0.251, MAE = 10.828 ± 0.210, and R2 = 69.064 ± 0.105, P-value = 0.0219). Its performance is improved by 1.362, 0.835, and 3.771 for RMSE, MAE, and R2, respectively. Lasso performance is improved by 1.167, 0.579, and 2.94 for RMSE, MAE, and R2, respectively. Fig. 21 shows the statistical comparison of the optimized classical regressors. As can be seen, LiR is significantly better than all other models. Table S7 in the supplementary materials shows the selected hyperparameters of the optimized classical techniques.

7.2. Static ensemble regression models

In the following two experiments, we explore the role of static ensemble regressors to improve the performance of LoS prediction. We test the performance of seven static ensemble models, namely CatBoost Regressor (CBR), XGB Regressor (XGBR), LGBM Regressor (LGBMR), GradientBoosting Regressor (GBR), RandomForest Regressor (RFR), ExtraTrees Regressor (ETR), and AdaBoost Regressor (ABR).

7.2.1. Experiment 14: Results without feature selection and hyperparameter optimization

The average performance of the seven static ensemble models is shown in Table 11. The results indicate that CBR is the best model without feature selection and hyperparameter tuning steps. The model achieved a performance of 12.969 ± 0.781, 8.619 ± 0.644, and 74.821 ± 0.303 for RMSE, MAE, and R2, respectively. As it can be seen, LiR is significantly better than all other models.

7.2.2. Experiment 15: Results after feature selection and hyperparameter optimization

After optimizing the models' hyperparameters and feature selection of the seven static ensembles, CBR still achieves the best results of (12.529 ± 0.103, 8.525 ± 0.058, and 78.275 ± 0.356) for RMSE, MAE, and R2, respectively. Table 12 shows that these optimization steps positively affect the performance of all ensemble models. Fig. 22 shows the statistical comparison of the optimized static ensemble regressors, while Fig. 23 analyzes the statistical difference between all optimized classical and ensemble models using the critical diagram. As noted, CBR achieved the best results (P-value 0.05) among the classical and static ensemble models. Table S8 of the SI shows the selected hyperparameters of the optimized static ensemble classifiers.

7.2.3. Experiment 16: Voting regression models

In this experiment, we test the voting regression model by using classical regression models as the pool of the voting model. We explored the voting regression model with a different number of classical regression models. We achieved the best result with the pool of the best three classical regression models (i.e., Linear Regression, Ridge, and Decision Tree Regressor) shown in Table 13. We also explore the voting

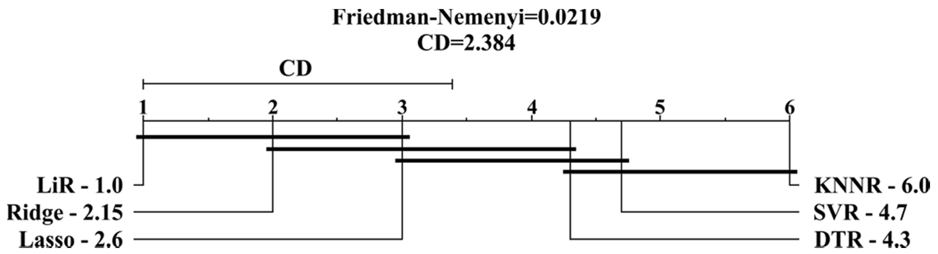

Fig. 21. Comparison of all classical regression models based on the Friedman test.

Table 11
Results of static ensemble regression models without feature selection and hyperparameter optimization.

Models	RMSE	MAE	R2
**CBR**	**12.969 ± 0.781**	**8.619 ± 0.644**	**74.821 ± 0.303**
XGBR	14.098 ± 2.775	9.431 ± 1.725	70.237 ± 1.178
LGBMR	13.247 ± 1.142	8.858 ± 0.867	73.730 ± 0.454
GBR	13.450 ± 0.811	8.933 ± 0.637	72.919 ± 0.327
RFR	13.624 ± 0.897	9.053 ± 0.714	72.212 ± 0.365
ETR	13.328 ± 0.853	8.867 ± 0.406	73.406 ± 0.340
ABR	17.521 ± 3.544	14.956 ± 4.134	54.026 ± 1.864

Table 12
Static ensemble regression models results with feature selection and hyperparameter optimization.

Models	RMSE	MAE	R-square
**CBR**	**12.529 ± 0.103**	**8.525 ± 0.058**	**78.275 ± 0.356**
XGBR	13.015 ± 0.121	8.847 ± 0.074	76.555 ± 0.439
LGBMR	13.052 ± 0.105	8.889 ± 0.051	76.424 ± 0.382
GBR	13.008 ± 0.119	8.853 ± 0.063	76.583 ± 0.430
RFR	13.327 ± 0.091	8.907 ± 0.026	75.420 ± 0.336
ETR	12.921 ± 0.086	8.776 ± 0.046	76.895 ± 0.309
ABR	14.303 ± 0.086	10.178 ± 0.059	71.688 ± 0.342

regression model by using static ensemble regression models as a pool. Table 13 provides the results of the voting regression model with two different numbers of static ensemble regression models. First, we test the voting regression model with the best four static ensemble regression models (i.e., CatBoost, ExtraTrees, Gradient Boosting, and LGBM Regressor), and achieve 78.330 ± 0.258 for R^2. Second, we test the voting regression model with the best three static ensemble regression models (i.e., CatBoost, ExtraTrees, and Gradient Boosting), and achieve 78.345 ± 0.275 for R^2.

Fig. 24 compares the voting regressor with other previously optimized regressors. The voting regressor with classical regressors pool (homogeneous ensemble) performs much better than other classical regression models (P-value < 0.01), i.e., the best classical regression model achieves R^2 of 69.0 and the voting regressor with classical

Table 13
Voting regressor results after feature selection and hyperparameter optimization steps.

Pool	Number of regressors	RMSE	MAE	R2
Classical Regressors	3 regressors	13.935 ± 0.144	9.871 ± 0.045	72.638 ± 0.569
Static Ensemble	4 regressors	12.513 ± 0.075	8.449 ± 0.035	78.330 ± 0.258
**Static Ensemble**	**3 regressors**	**12.509 ± 0.079**	**8.497 ± 0.036**	**78.345 ± 0.275**

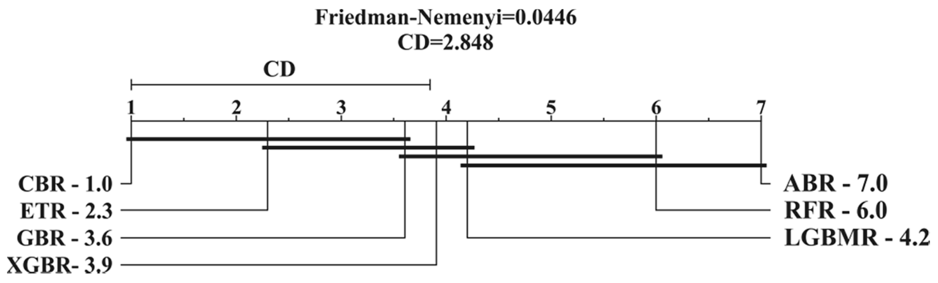

Fig. 22. Comparison of all static ensemble regression models based on the Friedman test.

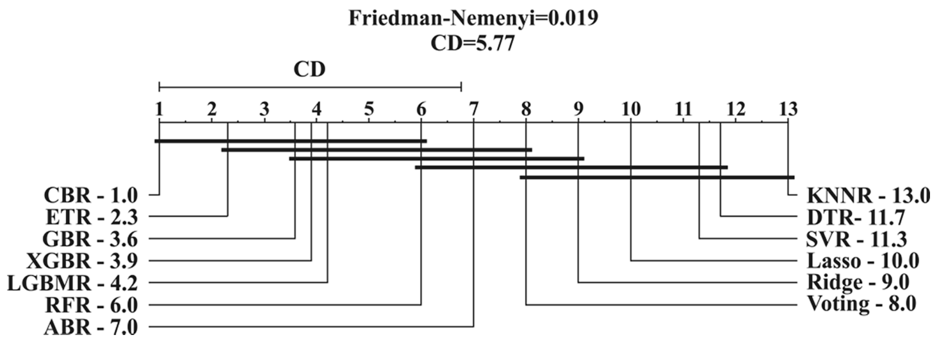

Fig. 23. Comparison of all static ensemble regression models based on the Friedman test.

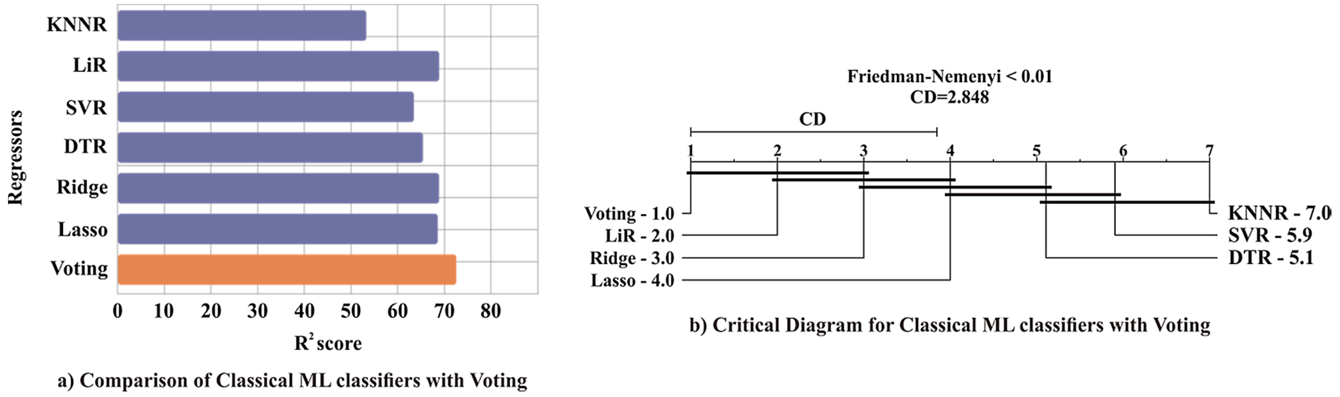

Fig. 24. Comparison of classical and static ensemble regression models with their voting regressor.

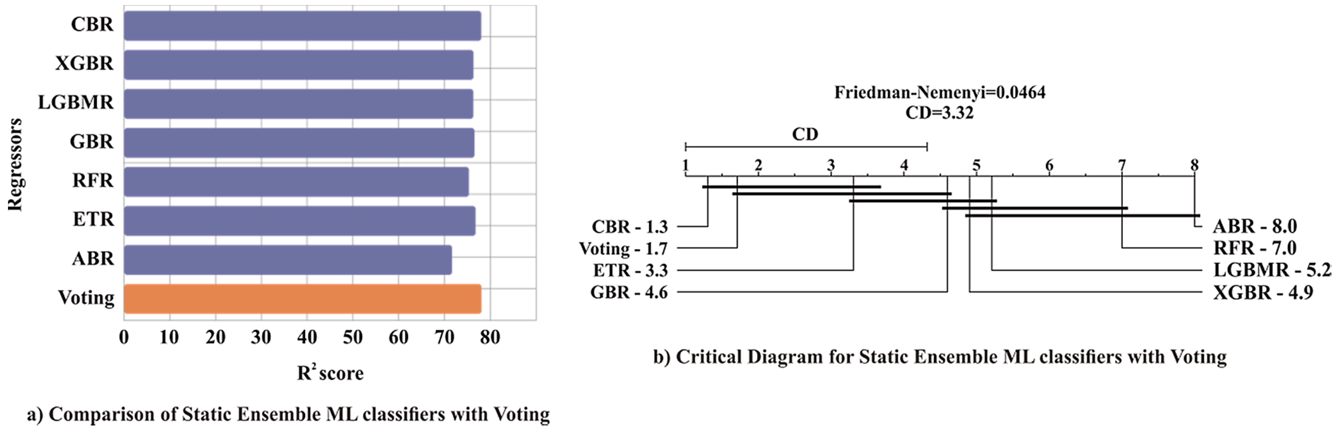

Fig. 25. Comparison of all static ensemble regression models based on the Friedman test.

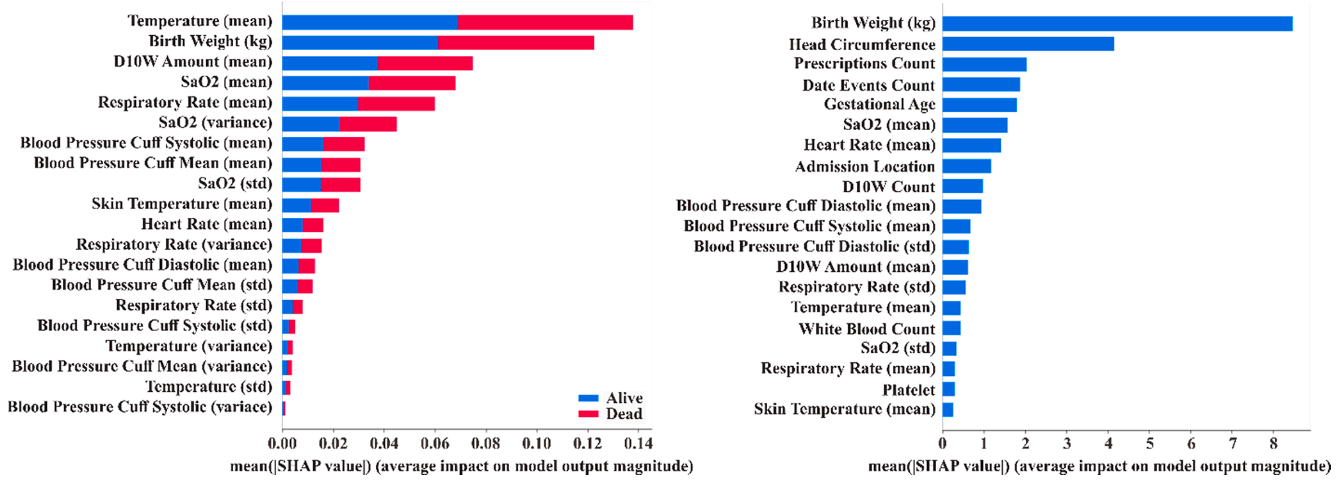

Fig. 26. Feature Importance for the classification layer (left) and the regression layer (right).

regression model achieves R^2 of 72.6 with a difference of 3.6, as shown in Fig. 24. On the other hand, the voting regressor with base classifiers of static ensemble regression models performs better than other static ensemble regression models, as shown in Fig. 25. The main reason for this better performance is possible because, in a classical regression models pool, there are many different regression models with very different techniques. These models are good in different parts of the feature space, which causes the aggregation of decisions to be better than classical decisions. That is why when we combine them, they give better performance. In the case of voting regressors with static ensemble base models, all techniques have a similar approach, and they are good at similar parts of the feature space. Therefore, when we combine them, they do not achieve higher results. The important features table (Table 26) shows that all static ensemble regression models share similar important features.

Table S9 of the SI lists the order sets of features for every classical regressor and their voting, while Table S10 of the SI shows the ordered lists of important features for static ensembles and their voting. The

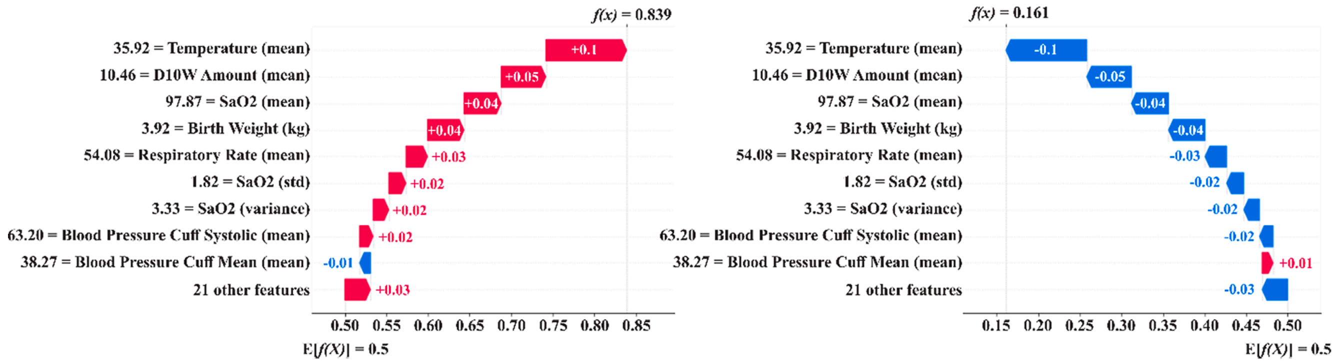

Fig. 27. Waterfall plots for survived neonate (left: probability of Alive class, right: probability of Dead class).

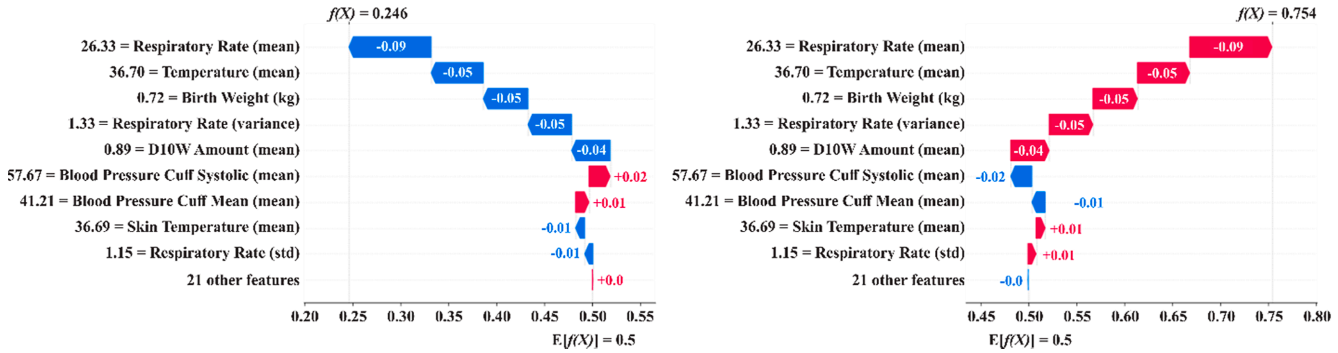

Fig. 28. Waterfall plots for dead neonate (left: probability of class 0-Alive, right: probability of class 1-Dead).

important common features in all classifiers are marked with bold font. Classical regressors highlight the main role of prescriptions count, head circumference, date events count, gestational age, and SaO2. On the other hand, the static ensemble regressors highlight another list of features, including birth weight, head circumference, date events count, prescriptions count, gestational age, SaO2 (mean), and D10W count. It seems interesting that both categorizes highlight the critical role of very similar features, where these features have significant differences between the compared two classes (i.e., Dead vs Alive).

8. Model explainability

From the previous experiments, we select the most accurate and stable classifier and regressor for the first and second layers, respectively. Providing a robust decision only is insufficient in the medical domain. Physicians ask for the reasons behind making specific decisions. They need to know the answer to these questions: *Why has the system taken that decision? Based on what features?* And *are these features medically relevant and sufficient?* In this section, we discuss our implemented XAI features in the current study. The physician will receive XAI capabilities in different formats that include feature importance, decision tree, and rule-based models.

8.1. Feature importance-based explainability

In feature importance-based explainability, it is essential to know the most important features that our classifier and regressor considered to make their final decisions. Fig. 26 shows a sorted list of features for the mortality prediction classifier, and for the LoS prediction regressor. We use the SHAP explainer to calculate the feature importance. Note that the two algorithms ranked the features similarly. In addition, these features are medically important. For example, the patient's temperature is the most important feature for predicting if the neonate will die in the ICU, and this is medically confirmed knowledge [124]. Neonate temperature also has a role in predicting the patient's LoS period, as shown in the right part of Fig. 26. Birth weight is the most important feature to predict the patient's LoS period, followed by heart circumference. The ranking of the features is medically relevant because it includes critical features like SaO2, respiratory rate, blood pressure, and heart rate. The collected statistical features, including means and standard deviations, are the most important features for both the classification and regression tasks. In addition to the statistical features, static features, like birth weight, admission location, D10W, and white blood count, also greatly affect the model decisions.

After analyzing the global feature importance of the used feature set, we need to examine the relevance of features for specific cases (i.e., local feature importance). We give an example of each case (i.e., Alive and Dead), and a case for LoS. Fig. 27 shows the Waterfall plots for a survived neonate case. The final decision for this case is "Alive" with a probability of 83.9 %. The dominating features are the mean temperature (35.92), mean D10w amount (10.46), mean SaO2 (97.87), birth weight (3.92), mean respiratory rate (54.08), the standard deviation of SaO2 (1.82), the variance of SaO2 (3.33), mean blood pressure cuff systolic (63.2), and mean blood pressure cuff (38.27). These values for the features push the decision toward the "Alive" class. Three statistical features are used from SaO2 (i.e., mean, variance, and standard deviation). When the patient has normal ranges of features, such as normal weight (around 4), normal temperature (around 36), etc., the patient is expected to live in the ICU.

On the other hand, Fig. 28 shows a Waterfall plot for a case from the "Dead" class. The dominating features are the mean respiratory rate (26.33), mean temperature (36.70), birth weight (0.72), the variance of respiratory rate (1.33), mean D10w amount (0.89), mean blood pressure cuff systolic (57.67), mean blood pressure cuff (41.21), mean SaO2 (97.87), mean skin temperature (36.69), and standard deviation of respiratory rate (1.15). From Fig. 28, a physician can visually measure the importance of each feature and its value on the final decision. As a result, physicians can determine if the ML model is taking the right

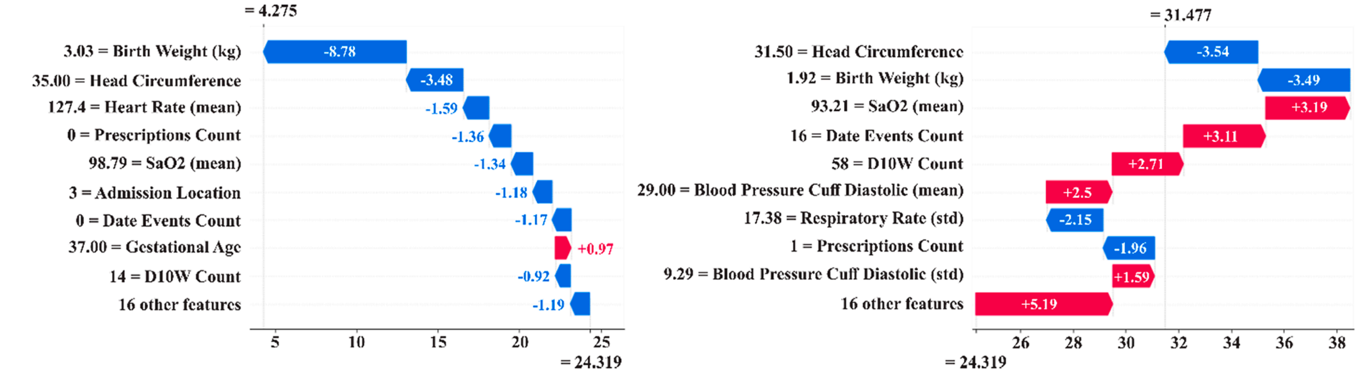

Fig. 29. Waterfall plots for regression (left: short time stay, right: long-time stay).

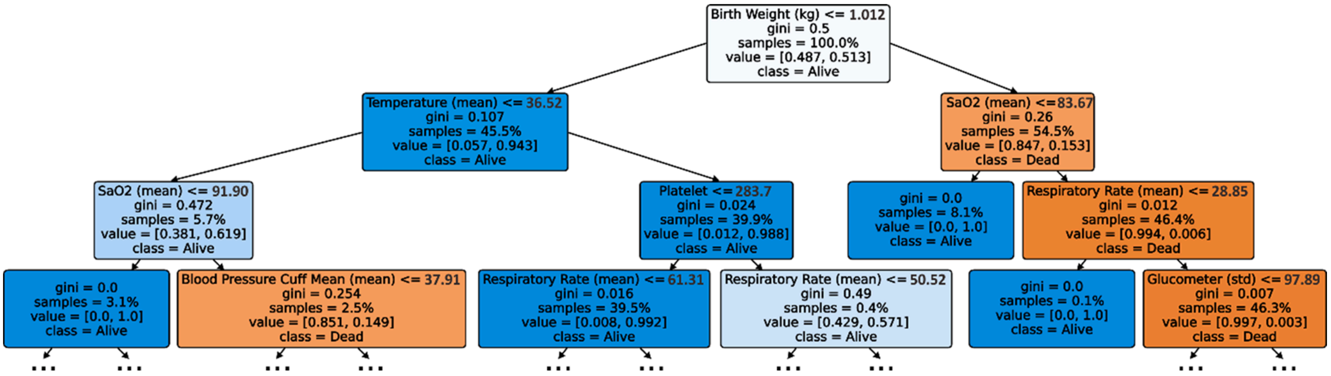

Fig. 30. Decision tree classifier for XAI.

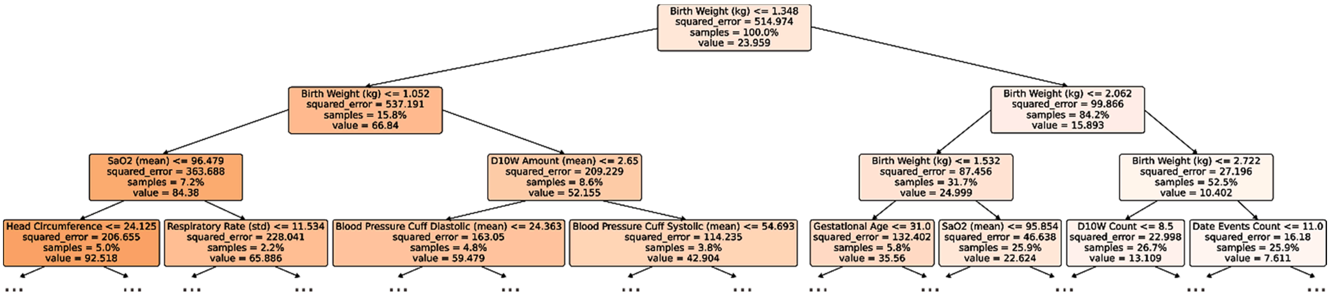

Fig. 31. Decision tree regressor for XAI.

decision or not, which affects their trust. Notice that SaO2 is not very critical to predicting the "Dead" class, but skin temperature appears in the list with a high value of around 37. Low respiratory rate, high temperature, very low birth weight, and very low D10W force the model to predict this case as "Dead". This decision is medically relevant and shows that our model is based on critical features with medically critical values. To investigate the robustness of our model, we explored the incorrectly predicted neonate cases. Out of 1854 test samples, our best model predicts 23 samples wrongly. These 23 neonates are all alive neonates, and our best model predicts them as dead. We explored the main reason for predicting those 23 neonates as dead, and found that they are very close to dead neonate samples, as illustrated in Figure S6 of the SI. Figure S7 in SI shows two examples from these 23 wrongly predicted neonates where the first neonate (left) is predicted as dead with 75.5 % confidence. The respiratory Rate of this neonate is 23.19, but the average respiratory rate for alive neonates is 52.16. The birth weight of this neonate is 1.054 kg. However, the average birth weight for alive neonates is 2.16 kg, and it is 1.17 kg for dead neonates. From the results, it can be clearly seen that this neonate's data is very close to dead neonates' data. Therefore, our best model failed to predict this neonate correctly. The same observation can be seen in the second neonate example (right). To support our observation, we provided data records of the top 5 important features for wrongly predicted neonates' data in Table S11 of the SI.

Fig. 29 shows regression cases for both long and short LoSs. We notice that neonates with normal birth weight have a short LoS compared to neonates with low birth weight. The conditions are for the other features, such as head circumference, heart rate. For space restrictions, we will not discuss the figures in more detail.

Depending only on feature importance to provide model explainability is insufficient for physicians. Physicians have normally based their decisions on a set of rules collected from their experience. The most accurate classifier is the DESKNN with the pool of classical machine learning classifiers, which is a black-box dynamic ensemble classifier where it is difficult to extract its set of rules. The most accurate regressor is the voting regression model with the pool of static ensemble regression models, which is a black-box model. Decision tree classifier can be used as a post-hoc XAI model for the black-box classifier. It is

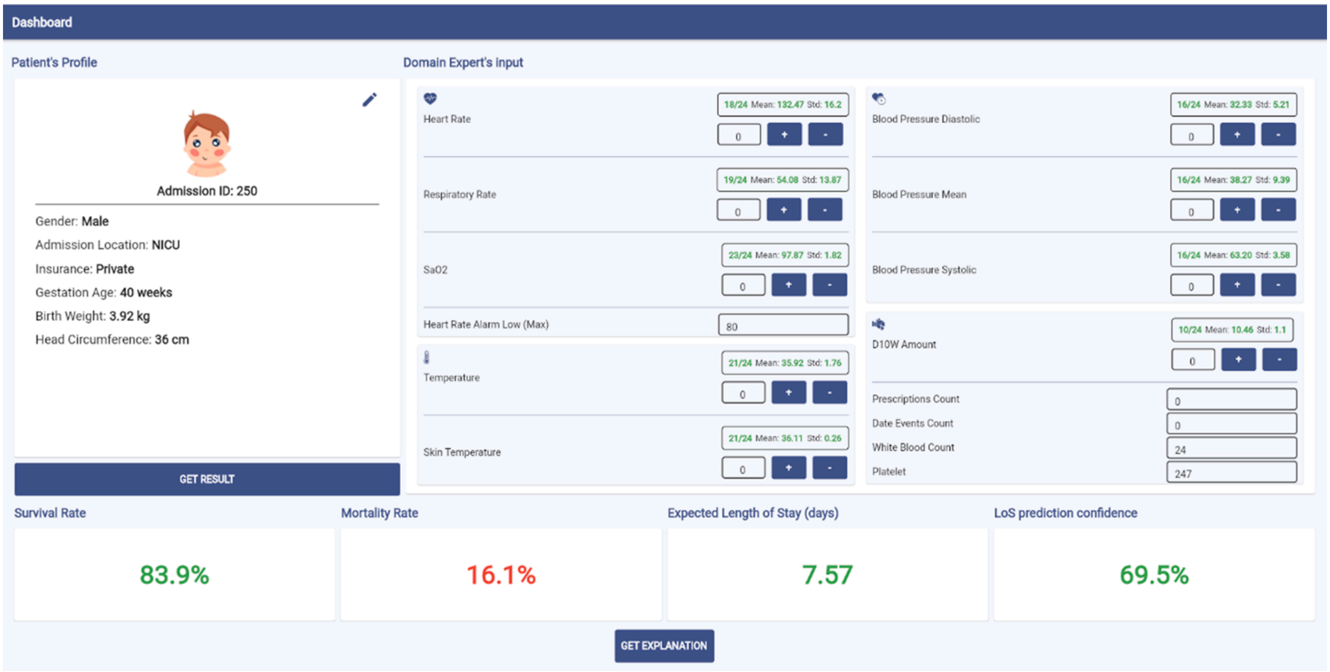

Fig. 32. Mortality and LoS prediction screen.

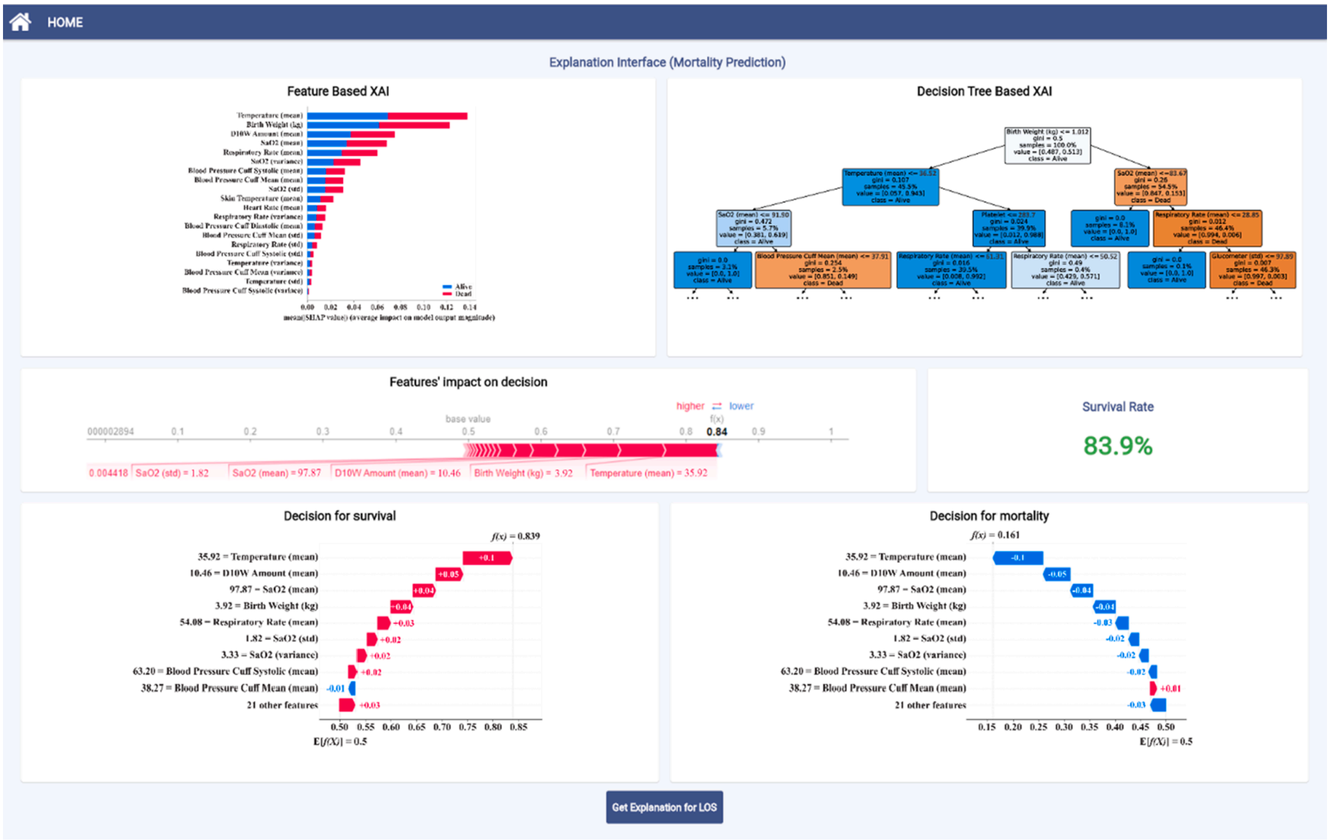

Fig. 33. XAI for mortality prediction decisions.

straightforward to extract a set of rules from any decision tree by following the path from the root of the tree to every leaf. Figs. 30 and 31 provide examples for the decision tree classifier and the decision tree regressor, respectivly. From these trees, the physician is able to collect the exact set of rules. These rules can assist domain experts confirm their decisions, and enable junior physicians to enrich their knowledge.

9. System implementation

The best performing models from the previously optimized

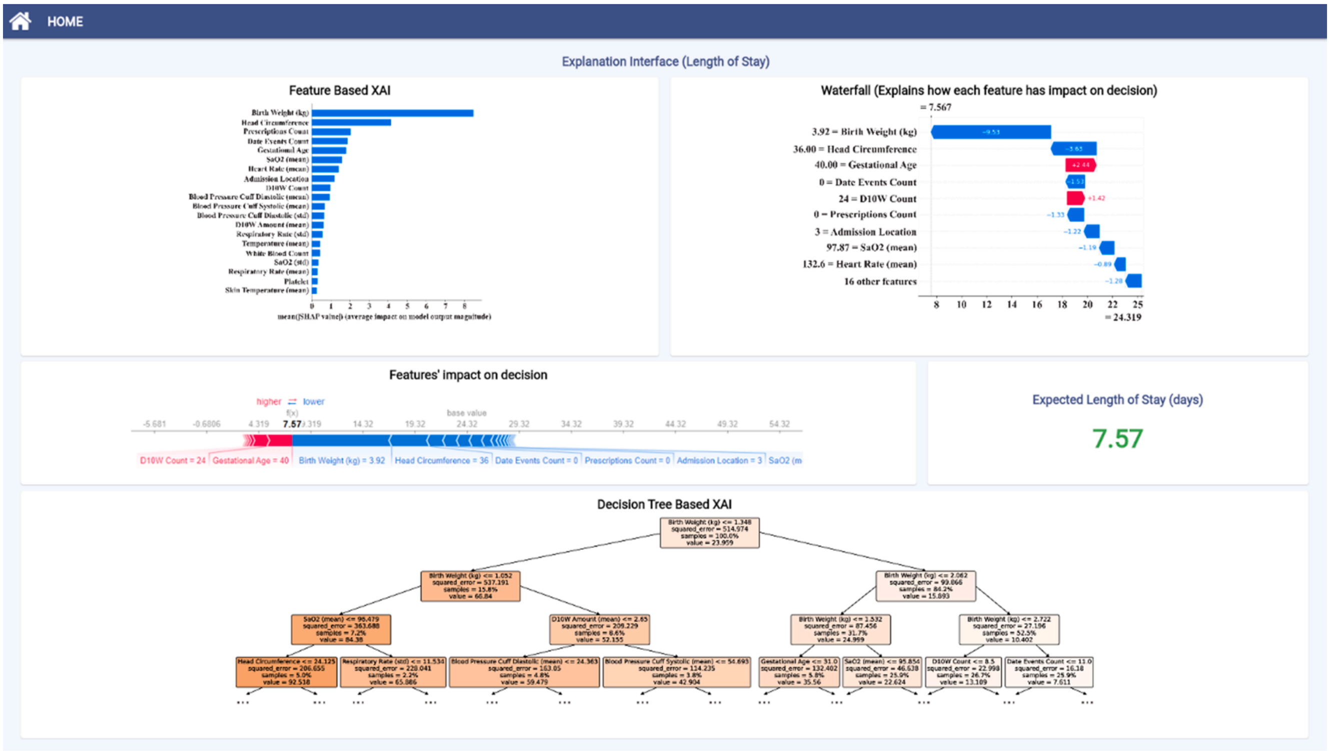

Fig. 34. XAI for LoS prediction decisions.

classification and regression models are used to implement a web-based system for neonate monitoring in ICU. For the classification backend, we use the DESKNN classifier, while for the regression backend, we use the CatBoost regressor. In addition, the proposed system can explain its decisions using many XAI capabilities. To the best of our knowledge, this is the first system for neonate management in ICU based on dynamic ensemble and XAI. As shown in the video illustration attached with the paper, the system works as follows:

1. The physician logs in to access the patient profile in the hospital information system. Once authorized, he/she sees the neonate's profile, as shown in Fig. 32.
2. Some of the patient data are collected automatically based on the patient ID from his/her electronic health record (EHR) at the admission time, such as gender, age, insurance, etc.
3. In this screen, a physician can insert the patient's vital signs. These data are time-series data where the physician can enter as much data as possible. Physicians are expected to enter the recorded data of the previous 24 h. Physicians can import these data directly from the patient EHR and can add or remove data manually if needed by clicking the plus and minus buttons. Mean and standard deviation derived features of these time series data are calculated and will be updated dynamically based on the newly added values by the domain expert.
4. After collecting the needed data from physicians and the EHR, the system provides the domain expert with the capability to accurately predict the survival rate, mortality rate, expected length of stay, and length of stay confidence using the previously optimized ML models. These decisions are calculated by clicking the "Get Results" button.
5. One of the most critical capabilities of the proposed system is the data preparation features. If the domain expert does not enter sufficient time-series data (i.e., the 24 h data after admission), the system will use our proposed Algorithms 1 and 2 to fill in the missing data.
6. As previously asserted, physicians do not believe ML decisions without knowing why the model has taken a specific decision. In addition, providing explainability features using multiple methods improves the confidence of the domain expert in the model decisions. The proposed system implements many XAI capabilities ranging from feature importance to decision tree rules. XAI features can be accessed by clicking the "Get Explanation." Button, which opens the screen shown in Fig. 33.

The proposed system provides two types of XAI, one for mortality prediction and the other for the length of stay prediction. In the first dashboard shown in Fig. 33, we provide three types of XAI, of feature importance, decision tree, and rules. The system provides both global XAI to describe the classifier's logic in general and local XAI to explain decisions of specific cases. The system decisions are explained by using a list of decision rules and decision trees as well. The implemented XAI features show consistency regarding the feature importance.

The second dashboard provides XAI features for the LoS prediction regression task. The system implements the feature importance and DT-based XAI to show the features that impact LoS prediction decisions, as shown in Fig. 34.

This paper explored the dynamic ensemble modeling techniques to predict neonate mortality and static ensemble modeling techniques to predict the patient's LoS. We explored the capabilities of these models using different combinations of base classifiers, using feature selection step, and hyperparameter optimization step. The study has been extended to include XAI features for both layers of the proposed model. Finally, and most importantly, we have implemented the proposed techniques in a web-based system to provide online assistance to physicians in ICU units to monitor neonate patients accurately and robustly. Our system provides accurate decisions and explanations for every decision and whole model logic. As a result, using this system can solve the problems of unavailability of qualified experienced physician and of less experienced physician.

Our study has some limitations that will be handled in future studies.

First, our model is based on a large set of features that could be expensive to collect in ICU. In future studies, we will explore the role of every feature on the model performance using accurate statistical analysis tests and select the least number of features. We will try to implement the most accurate and cost-effective models. *Second*, we concentrated here on the XAI of our model to build a medically acceptable clinical decision support system that requires XAI. The system must be trustworthy, which means robust, fair, and interpretable. In a future study, we will explore and implement the other two dimensions of robustness and fairness and explore other methods of explainability. *Third*, our models are based on the extracted features from time-series data to build accurate classifiers based on classical ML models. Even though these models are faster than the deep learning model; it is expected that using the raw time-series data with deep learning models like RNN and LSTM could improve the model performance. As noticed, there is no study for deep learning in neonate management. In addition, few studies have been conducted using advanced DL techniques such as multilayered LSTM, Bidirectional LSTM, CNN, ConvLSTM, and hybrid CNN-LSTM [121,122,123,105,119] These techniques are expert in extracting dynamical patterns within univariate time-series and interactions among heterogeneous or multivariate time-series. These deep features support the creation of decision support tools that provide personalized decisions [124]. We will explore the capabilities of DL models to learn time-series data in a separate study.

10. Conclusion

In this work, we proposed a two-layers machine learning model for the ICU mortality prediction of neonates. In the first layer, we investigated the role of dynamic ensemble modeling to predict mortality for neonate patients in ICU. In the second layer and for the survived patients, we predicted their LoS. The study was based on the time-series data of the patients for the previous 24 h. We proposed intelligent and medically acceptable algorithms for preparing these time-series data. To search for the optimum model, we implemented and tested different categories of machine learning models, including classical ML models, static ensemble models, and dynamic ensemble models. These models have been tested after using the default hyperparameters, optimized hyperparameters, and feature selection steps. Results were collected after optimizing these models using the MIMIC-III real-world dataset. We used a cohort of 3133 patients. For mortality prediction classification task, the most performing model was DESKNN with the pool of classical machine learning classifiers (accuracy = 0.987 ± 0.001, precision = 0.975 ± 0.001, recall = 1.000 ± 0.000, and F-score = 0.988 ± 0.001). The best model for the LoS prediction regression task was the Voting regression model with the pool of static ensemble regression models (RMSE = 12.509 ± 0.079, R^2 = 78.345 ± 0.275). We extended these optimized models to provide XAI capabilities using different techniques, including feature importance based on SHAP, decision trees, and rule-base. Our resulting model was robust because it achieved high performance, and it was explainable by adding the XAI features. As a result, this model was medically more intuitive and trustworthy than other literature studies. Note that this is the first study that predicts ICU mortality and LoS for neonates based on time-series data and dynamic ensembles. In addition, this is the first study that provides XAI for this task. To complete the task, we utilized the optimized classifier and regressors with their explainability power to implement a website for neonate monitoring. The system can assist physicians in predicting patient mortality and LoS, and at the same time, provide explanations for why the model takes a specific decision. In future studies, we will handle the limitations of this study as discussed in the manuscript.

Availability of data and material.

The dataset can be requested via the MIMIC III website (https://mimic.mit.edu/).

Code availability.

We have made our source codes and models publicly available at https://github.com/InfoLab-SKKU/neonateMortalityPrediction.

Funding.

This research was supported by the MSIT (Ministry of Science and ICT), Korea, under the ICT Creative Consilience Program (IITP-2021–2020-0–01821) supervised by the IITP(Institute for Information & communications Technology Planning & Evaluation), and the National Research Foundation of Korea(NRF) grant funded by the Korea government(MSIT) (No. 2021R1A2C1011198).

CRediT authorship contribution statement

Firuz Juraev: Conceptualization, Methodology, Software. **Shaker El-Sappagh:** Data curation, Writing – original draft, Conceptualization. **Eldor Abdukhamidov:** Software, Visualization, Investigation. **Farman Ali:** Conceptualization, Methodology, Validation. **Tamer Abuhmed:** Supervision, Methodology, Validation, Formal analysis, Funding acquisition, Project administration, Writing – review & editing.

Declaration of Competing Interest

The authors declare that they have no known competing financial interests or personal relationships that could have appeared to influence the work reported in this paper.

Acknowledgments

This research was supported by the MSIT (Ministry of Science and ICT), Korea, under the ICT Creative Consilience Program (IITP-2021–2020-0–01821) supervised by the IITP (Institute for Information & communications Technology Planning & Evaluation), and the National Research Foundation of Korea(NRF) grant funded by the Korea government (MSIT) (No. 2021R1A2C1011198). We would like to thank Dr. Khudoykulova Hafiza Egamkulovna for her assistance in identifying the most predictive indicators for predicting the neonate mortality and for formulating the medical problem. Since 2012, Dr. Khudoykulova Hafiza Egamkulovna has been the head neonatologist at the Samarkand Regional Perinatal Center, Samarkand, Uzbekistan. Since 2013, she also has been working as a trainer on Neonates' Resuscitation. We would also like to thank Dr. Usanova Nigora Ishmanovna for her assistance in identifying the most predictive indicators for predicting the neonate's length of stay and for her contribution to the evaluation of the framework visualizations and explainability. Dr. Usanova Nigora Ishmanovna is a doctor of 1st category, and she has been working at Samarkand Prenatal Center, Samarkand, Uzbekistan as a neontologist since 2012.

Appendix A. . Used dynamic ensemble classifier algorithms

KNORA-E [125] algorithm searches for local Oracle classifiers from the pool of classifiers that correctly classify all the K nearest neighbors for a given test data in the training set. This classifier is a base classifier that correctly classifies all samples belonging to the region of competence of the test sample. The list of classifiers that have a perfect performance in the region of competence defines the local Oracles. If no classifier achieves perfect accuracy, the algorithm keeps reducing the size of the competence region by removing the farthest neighbors, until finding at least one classifier that classifies all the training examples in the neighborhood of the test set correctly. The ensemble of the chosen classifiers is assigned and used for voting to classify the test data, i.e., decisions of all local oracles are combined using the majority voting scheme.

DESP [126] algorithm selects all base classifiers from the pool of classifiers that achieve a higher performance in the region of competence than the random classifier. Random classifier performance is $1/M$ for M being the number of classes in the dataset. Hence, the competence level is calculated as $P(c_i|\theta_j) - \frac{1}{M}$, for a region of competence θ_j. A classifier with a positive value for that value is selected. If no base classifier achieves that performance, the whole pool is used for classification. The dynamic selection is made for every test case by comparing the performance of all classifiers with the random classifier in the defined neighborhood of the test data.

METADES [127] algorithm models the DES as a *meta*-problem that uses different criteria to evaluate the behavior of a base classifier c_i to decide if the classifier is competent enough to classify a sample. There are two main steps in solving this *meta*-problem: (1) METADES has a *meta*-training stage of extracting a list of *meta*-features (*MFs*) from each example in training and the dynamic selection dataset for all classifiers in the pool. Four types of *MFs* could be collected. (a) posterior probability for each label, (b) the overall local accuracy of c_i in the region of competence, (c) neighbors' hard classification (vector $v \in \mathbb{R}^n$ created for each classifier c_i, where, n is the number of samples in the region of competence; if classifier c_i, correctly classifies a sample x_i, $v[i] = 1$, otherwise $v[i] = 0$), and (d) classifier's confidence (the perpendicular distance between the input sample and the decision boundary of the classifier). (2) These extracted *MFs* are used to train a *meta*-classifier λ to predict whether a base classifier c_i is competent enough to classify a given sample. To classify an unknown sample x, *first*, the *MFs* for each base classifier *ci* in relation to $\times$ are calculated and presented to λ. Second, λ estimates the competence level of each base classifier *ci* for the classification of x. All base classifiers with competence level higher than a pre-defined threshold are selected. If all base classifiers have lower competence level than the threshold, the whole pool is used for classification.

KNORA-U [125] algorithm selects all classifiers from the pool of classifiers that correctly classifies any sample of the K nearest neighbors of a case in the region of competence. These classifiers are combined to form an ensemble for the given test data. Majority voting is used for KNORAU prediction. The number of votes for each of the selected classifiers equals the number of correctly classified training set samples in the K neighborhood by that classifier. The votes obtained by all base classifiers are aggregated to obtain the final ensemble decision.

DESKNN [128] algorithm selects all classifiers from the pool of classifiers based on accuracy and diversity. The first step is to define the region of competence θ_j. After that, DESKNN ranks the base classifiers based on the accuracy in decreasing order, and it ranks classifiers in increasing order based on their diversity. The Double Fault measure [118] was utilized to measure the diversity of the base classifiers. Next, DESKNN selects N classifiers from the pool based on their accuracy. Then, it selects J classifiers from the N most accurate classifiers based on their diversity for creating Ensemble of Classifiers (EoC). ($J \leq N$) must be defined.

KNOP [127] algorithm selects all classifiers from the pool of classifiers that correctly classifies at least one sample of the K nearest neighbors of a case in the region of competence θ_j. To define the region of competence, samples that are close to the given query instance are selected. The similarity between the query and samples in the validation set is calculated using decision space, not feature space. Then each selected classifier predicts the label of the given query, and the predictions are aggregated to obtain the final decision.

Appendix B. . Preliminary results of Classical, static Ensemble, dynamic ensemble mortality prediction models

1. Classical ML Models Results without feature selection and hyperparameter optimization.

In this experiment, we implement six of the most popular ML classifiers. We test the models using the default hyperparameters and with all features. As shown in Table B1**,** Logistic regression model achieved the best results (F1-score of 0.918 ± 0.012). However, support vector classifier (SVC) is the most stable model because it has the lowest standard deviation. KNN classifier achieved the worst results (F1-score of 0.499 ± 0.028).

2. Classical ML Models Result after feature selection step only.

In this experiment, we study the role of the feature selection step to train ML models with default hyperparameters. The selected six models are test-based, and the results are collected. After applying the feature selection step, most classifiers (i.e., NB, LR, KNN, DT, and MLP) show improved results, see Table B2. The performance of NB is improved by 12.3 %, the performance of LR is improved by 0.5 %, the performance of KNN is improved by 37.9 %, the performance of DT is improved by 6.4 %, and the performance of MLP is improved by 8.5 %. LR achieved the best results (F1-score of 0.923 ± 0.003), and the model became more stable. SVC performed 88.9 % without feature selection, but after applying the feature selection step, its performance decreased by 5.3 %.

3. Static Ensemble Models Results without feature selection and optimization.

Table B1
Classical ML classifiers model results without feature selection and hyperparameter optimization.

Models	Accuracy	Precision	Recall	F1-score
NB	0.682 ± 0.005	0.611 ± 0.004	1.000 ± 0.000	0.759 ± 0.003
**LR**	**0.918 ± 0.010**	**0.922 ± 0.004**	**0.914 ± 0.021**	**0.918 ± 0.012**
KNN	0.659 ± 0.011	0.938 ± 0.011	0.340 ± 0.021	0.499 ± 0.028
DT	0.667 ± 0.047	0.900 ± 0.032	0.371 ± 0.092	0.521 ± 0.099
SVC	0.895 ± 0.002	0.946 ± 0.003	0.838 ± 0.003	0.889 ± 0.002
MLP	0.768 ± 0.040	0.978 ± 0.006	0.549 ± 0.082	0.700 ± 0.069

Table B2
Classical ML classifiers model results with feature selection.

Models	Accuracy	Precision	Recall	F1-score
NB	0.888 ± 0.002	0.920 ± 0.004	0.849 ± 0.003	0.883 ± 0.002
**LR**	**0.922 ± 0.003**	**0.912 ± 0.004**	**0.935 ± 0.003**	**0.923 ± 0.003**
KNN	0.889 ± 0.002	0.976 ± 0.002	0.797 ± 0.004	0.878 ± 0.002
DT	0.703 ± 0.037	0.955 ± 0.007	0.426 ± 0.075	0.585 ± 0.082
SVC	0.848 ± 0.011	0.908 ± 0.007	0.776 ± 0.024	0.836 ± 0.013
MLP	0.817 ± 0.012	0.948 ± 0.008	0.671 ± 0.031	0.785 ± 0.019

Table B3 shows the results of this experiment. As noticed, AdaBoost achieved the best results (F1-score of 0.706 ± 0.081). Although LGBM is a strong ensemble model, testing it using the default hyperparameters achieved the worst results.

4. Static Ensemble Result after feature selection step.

Table B4 reports the results of the static ensemble classifiers after applying the feature optimization step. As expected, the feature optimization step enhances the performance of all classifiers. XGBoost achieved the best results (F1-score of 0.814 ± 0.032). The other boosting algorithm (i.e., AdaBoost) achieved comparable results (F1-score of 0.795 ± 0.065), but AdaBoost is less stable than XGBoost.

5. Selecting the best number of classical classifiers for the dynamic ensembles pool.

Table B5 shows four experiments that explore the role of a different number of base classifiers on the performance of dynamic ensemble models. We tested the performance of dynamic ensembles based on the best three, best four, best five, and all six base classifiers. When we used a pool of six classifiers, this behavior is changed, when we used a pool of six classifiers because the model performance is decreased. As a result, we used the best five classifiers to build the pool of base classifiers for the DES. The selected base classifiers that result in the highest DES performance are MLP, LR, SVC, DT, and NB. We report the average accuracy over all DES algorithms, including namely FIRE-KNORA-U, KNORA-U, FIRE-KNORA-E, KNORA-E, FIRE-METADES, METADES, FIRE-DESKNN, DESKNN, FIRE-DESP, DESP, FIRE-KNOP, and KNOP. Using the pool of five classifiers, the FIRE DESKNN achieved an average accuracy of 0.963 ± ±0.016. This ensemble is also the most robust, because it has the best confidence interval for model performance [0.987, 0.940].

6. Selecting the best number of static ensemble classifiers for dynamic ensembles pool.

As shown in Table B6, we tested the best optimized three static ensemble models (i.e., *AdaBoost*, *RF*, and *CatBoost*), and the average accuracy of the 12 DES models is 0.961 ± 0.003. The best DES model is FIRE METADES, which achieves an accuracy of 0.965. We notice that adding more static ensemble models (e.g., *LightGBM*, *Gradient Boosting*, or *Majority Voting*) to the pool decreases the performance of the DES model. As a result, we retain stick this number to build the pool of best-performing base classifiers.

7. Selecting the best number of classifiers for a mixed pool of classical and static ensemble models.

We explored the possible role of building a pool of heterogeneous classifiers by mixing both classical and static ensemble base classifiers. We tested different pool sizes from four to ten base classifiers and explored the effect of changing the number of base classifiers from different categories.

Table B3
Static ensemble ML classifiers model results without feature selection and hyperparameter optimization.

Models	Accuracy	Precision	Recall	F1-score
**AdaBoost**	**0.772 ± 0.046**	**0.969 ± 0.008**	**0.561 ± 0.093**	**0.706 ± 0.081**
XGBoost	0.749 ± 0.049	0.991 ± 0.006	0.503 ± 0.096	0.661 ± 0.090
RF	0.733 ± 0.032	0.952 ± 0.007	0.490 ± 0.065	0.644 ± 0.057
GB	0.745 ± 0.019	0.978 ± 0.005	0.501 ± 0.038	0.662 ± 0.033
CatBoost	0.759 ± 0.028	0.987 ± 0.004	0.525 ± 0.057	0.683 ± 0.050
LGBM	0.668 ± 0.022	0.994 ± 0.003	0.338 ± 0.044	0.504 ± 0.054
Majority Voting	0.663 ± 0.037	0.910 ± 0.018	0.359 ± 0.074	0.511 ± 0.081

Table B4
Static ensemble ML classifiers model results with feature selection.

Models	Accuracy	Precision	Recall	F1-score
**AdaBoost**	0.829 ± 0.045	0.970 ± 0.005	0.679 ± 0.092	0.795 ± 0.065
XGBoost	**0.843 ± 0.024**	**0.989 ± 0.003**	**0.693 ± 0.047**	**0.814 ± 0.032**
RF	0.815 ± 0.054	0.961 ± 0.008	0.656 ± 0.107	0.775 ± 0.077
GB	0.767 ± 0.032	0.960 ± 0.004	0.557 ± 0.065	0.703 ± 0.053
CatBoost	0.756 ± 0.026	0.986 ± 0.003	0.520 ± 0.052	0.679 ± 0.043
LGBM	0.743 ± 0.032	0.991 ± 0.002	0.491 ± 0.065	0.654 ± 0.057
Majority Voting	0.776 ± 0.018	0.946 ± 0.004	0.586 ± 0.036	0.723 ± 0.029

Table B5
Selecting the best number of classifiers for DES with classical ML classifiers.

Experiment	Average Accuracy	Best Model	Best Model Accuracy	Worst Model	Worst Model Accuracy
3 classifiers	0.952 **(0.007)**	DESKNN, KNORA-E	0.960	FIRE KNOP	0.939
4 classifiers	0.953 (0.022)	DESKNN	0.972	FIRE KNOP	0.920
**5 classifiers (selected)**	**0.963 (0.016)**	**FIRE DESKNN**	**0.987**	**FIRE KNORA-E**	**0.940**
6 classifiers	0.939 (0.029)	FIRE KNORA-U	0.981	FIRE KNOP	0.890

Table B6
Selecting the best number of classifiers for DES with static ensemble ML classifiers.

Experiment	Average Accuracy	Best Model	Best Model Accuracy	Worst Mode	Worst Model Accuracy
**3 classifiers (selected)**	**0.961 (0.003)**	**FIRE METADES**	**0.965**	**FIRE DESKNN**	**0.955**
4 classifiers	0.955 (0.018)	KNORA-E	0.975	KNOP	0.932
5 classifiers	0.947 (0.026)	KNORA-E	0.976	METADES	0.906
6 classifiers	0.954 (0.021)	KNORA-E	0.980	KNOP	0.928

Table B7
Selecting the best number of classifiers for DES with a mixed pool of classical and static ensemble classifiers.

Experiment	Classic/Static	Average Accuracy	Best Model	Best Model Accuracy	Worst Model	Worst Model Accuracy
4 classifiers	2 / 2	0.940 (0.005)	FIRE DESKNN	0.945	KNORA-E	0.932
4 classifiers	3 / 1	0.955 (0.008)	DESKNN	0.967	FIRE KNOP	0.941
4 classifiers	1 / 3	0.956 (0.013)	FIRE KNORA-E	0.971	KNOP	0.940
5 classifiers	3 / 2	0.944 (0.006)	FIRE METADES	0.955	KNORA-U	0.936
5 classifiers	2 / 3	0.951 (0.011)	FIRE KNORA-E	0.965	METADES	0.937
6 classifiers	3 / 3	0.962 (0.009)	FIRE METADES	0.969	FIRE DESKNN	0.943
6 classifiers	4 / 2	0.965 (0.014)	DESKNN	0.979	KNOP	0.933
**7 classifiers***	**3 / 4**	**0.973 (0.004)**	**KNORA-E**	**0.980**	**KNORA-U**	**0.968**
7 classifiers	4 / 3	0.968 (0.011)	FIRE KNORA-E	0.980	KNOP	0.943
8 classifiers	4 / 4	0.962 (0.019)	KNORA-E	0.979	KNOP	0.916
9 classifiers	5 / 4	0.958 (0.017)	FIRE DESP	0.974	KNOP	0.927
9 classifiers	4 / 5	0.962 (0.019)	FIRE KNORA-E	0.977	KNOP	0.908
10 classifiers	5 / 5	0.959 (0.018)	DESKNN	0.981	FIRE KNOP	0.932

Table B7 shows all experiments, and highlights that using seven base classifiers (the three best classical [MLP, LR, and SVC] and four best static ensembles [AdaBoost, RF, CatBoost, and LightGBM]) achieves the best results with the KNORA-E DES model (average accuracy of 0.973 ± 0.004 and best accuracy of 0.980), and the model is more stable. Note that mixing classical and static ensemble classifiers achieved the highest average accuracy. We also noticed that increasing the number of classifiers gradually improves the DES model performance. This behavior reached the optimum results when the pool had seven base classifiers. However, when the number of base classifiers increased to more than seven, the performance started to decrease. As a result, it can be concluded that increasing the number of base classifiers causes the ensemble to become more complex, and overfits the data. In addition, we note that the static ensemble has a more positive effect on the performance of the DES model than the simple classical models.

Appendix C. Supplementary data

Supplementary data to this article can be found online at https://doi.org/10.1016/j.jbi.2022.104216.

References

[1] G. Carra, J.I.F. Salluh, F.J. da Silva Ramos, G. Meyfroidt, Data-driven ICU management: Using Big Data and algorithms to improve outcomes, J. Crit. Care 60 (2020) 300–304, https://doi.org/10.1016/j.jcrc.2020.09.002.
[2] I.T. Peres, S. Hamacher, F.L.C. Oliveira, A.M.T. Thomé, F.A. Bozza, What factors predict length of stay in the intensive care unit? Systematic review and meta-analysis, J. Crit. Care 60 (2020) 183–194.
[3] A.M. Mclaughlin, J. Hardt, J. Canavan, M. Donnelly, Determining the economic cost of ICU treatment: A prospective 'micro-costing' study, Intensive Care Med. 35 (2009) 2135–2140, https://doi.org/10.1007/s00134-009-1622-1.
[4] S. Purushotham, C. Meng, Z. Che, Y. Liu, Benchmarking deep learning models on large healthcare datasets, J Biomed Inform 83 (2018) 112–134, https://doi.org/10.1016/j.jbi.2018.04.007.
[5] L.D. Straney, A.A. Udy, A. Burrell, C. Bergmeir, S. Huckson, D.J. Cooper, D. V. Pilcher, S. Brakenridge, Modelling risk-adjusted variation in length of stay among Australian and New Zealand ICUs, PLoS ONE 12 (5) (2017) e0176570.
[6] F. Vicente, F. Lomar, C. Mélot, J.-L. Vincent, Can the experienced ICU physician predict ICU length of stay and outcome better than less experienced colleagues? Intensive Care Med. 30 (2004) 655–659, https://doi.org/10.1007/s00134-003-2139-7.
[7] I.W.M. Verburg, A. Atashi, S. Eslami, R. Holman, A. Abu-Hanna, E. de Jonge, N. Peek, N.F. de Keizer, Which Models Can I Use to Predict Adult ICU Length of Stay? A Systematic Review, Crit. Care Med. 45 (2) (2017) e222–e231.
[8] S.E. Seaton, L. Barker, D. Jenkins, E.S. Draper, K.R. Abrams, B.N. Manktelow, What factors predict length of stay in a neonatal unit: A systematic review, BMJ Open 6 (10) (2016) e010466.
[9] A. Atashi, I.W. Verburg, H. Karim, M. Miri, A. Abu-Hanna, E. de Jonge, N.F. de Keizer, S. Eslami, Models to predict length of stay in the Intensive Care Unit after coronary artery bypass grafting: a systematic review, J. Cardiovasc. Surg. (Torino) 59 (3) (2018).
[10] J.-L. Vincent, M. Singer, Critical care: advances and future perspectives, Lancet 376 (9749) (2010) 1354–1361, https://doi.org/10.1016/S0140-6736(10)60575-2.
[11] J. L. Vincent, "Is the current management of severe sepsis and septic shock really evidence based?," *PLoS Medicine*, vol. 3, no. 9. Public Library of Science, pp. 1488–1491, 2006. doi: 10.1371/journal.pmed.0030346.
[12] R. Houthooft, J. Ruyssinck, J. van der Herten, S. Stijven, I. Couckuyt, B. Gadeyne, F. Ongenae, K. Colpaert, J. Decruyenaere, T. Dhaene, F. De Turck, Predictive modelling of survival and length of stay incritically ill patients using sequential organ failure scores, Artif Intell Med 63 (3) (2015) 191–207.
[13] A. Awad, M. Bader–El–Den, J. McNicholas, Patient length of stay and mortality prediction: A survey, Health Serv Manage Res 30 (2) (2017) 105–120.
[14] J.-L. Vincent, R. Moreno, J. Takala, S. Willatts, A. De Mendonça, H. Bruining, C. K. Reinhart, P.M. Suter, L.G. Thijs, The SOFA (Sepsis.related Organ Failure Assessment) score to describe organ dysfunction/failure, Intensive Care Med 22 (7) (1996) 707–710.
[15] J.R. le Gall, S. Lemeshow, F. Saulnier, A new Simplified Acute Physiology Score (SAPS II) based on a European/North American multicenter study, JAMA 270 (24) (1993) 2957–2963, https://doi.org/10.1001/jama.270.24.2957.
[16] W. Knaus, E.A. Draper, D.P. Wagner, J.E. Zimmerman, APACHE II: a severity of disease classification system, Crit Care Med 13 (Nov. 1985) 818–829, https://doi.org/10.1097/00003465-198603000-00013.
[17] J.I.F. Salluh, M. Soares, ICU severity of illness scores, Curr Opin Crit Care 20 (5) (2014) 557–565.
[18] R.O. Deliberato, S. Ko, M. Komorowski, M.A. Armengol de La Hoz, M. P. Frushicheva, J.D. Raffa, A.E.W. Johnson, L.A. Celi, D.J. Stone, Severity of Illness Scores May Misclassify Critically Ill Obese Patients, Crit Care Med 46 (3) (2018) 394–400.
[19] A.P. Nassar, P. Caruso, ICU physicians are unable to accurately predict length of stay at admission: a prospective study, Int J Qual Health Care 28 (1) (2016) 99–103.
[20] Y. Ding, Y. Wang, D. Zhou, Mortality prediction for ICU patients combining just-in-time learning and extreme learning machine, Neurocomputing 281 (2018) 12–19, https://doi.org/10.1016/j.neucom.2017.10.044.
[21] J. Zimmerman, A. Kramer, D. Mcnair, F. Malila, V. Shaffer, Intensive care unit length of stay: Benchmarking based on Acute Physiology and Chronic Health Evaluation (APACHE) IV, Crit Care Med 34 (Nov. 2006) 2517–2529, https://doi.org/10.1097/01.CCM.0000240233.01711.D9.
[22] S. Siddiqui, M. Chua, V. Kumaresh, R. Choo, A comparison of pre ICU admission SIRS, EWS and q SOFA scores for predicting mortality and length of stay in ICU, J Crit Care 41 (2017) 191–193.
[23] G. S. Krishnan and S. K. S., "A novel GA-ELM model for patient-specific mortality prediction over large-scale lab event data," *Appl Soft Comput*, vol. 80, pp. 525–533, 2019, doi: https://doi.org/10.1016/j.asoc.2019.04.019.
[24] B. Shickel, T.J. Loftus, L. Adhikari, T. Ozrazgat-Baslanti, A. Bihorac, P. Rashidi, DeepSOFA: A Continuous Acuity Score for Critically Ill Patients using Clinically Interpretable Deep Learning, Sci Rep 9 (1) (2019) 1879, https://doi.org/10.1038/s41598-019-38491-0.
[25] G. Meyfroidt, F. Güiza, D. Cottem, W. De Becker, K. Van Loon, J.-M. Aerts, D. Berckmans, J. Ramon, M. Bruynooghe, G. Van den Berghe, Computerized prediction of intensive care unit discharge after cardiac surgery: development and validation of a Gaussian processes model, BMC Med Inform Decis Mak 11 (1) (2011), https://doi.org/10.1186/1472-6947-11-64.
[26] N. Hou, M. Li, L.u. He, B. Xie, L. Wang, R. Zhang, Y. Yu, X. Sun, Z. Pan, K. Wang, Predicting 30-days mortality for MIMIC-III patients with sepsis-3: a machine learning approach using XGboost, J Transl Med 18 (1) (2020), https://doi.org/10.1186/s12967-020-02620-5.
[27] H.R. Darabi, D. Tsinis, K. Zecchini, W.F. Whitcomb, A. Liss, Forecasting Mortality Risk for Patients Admitted to Intensive Care Units Using Machine Learning, Procedia Comput Sci 140 (2018) 306–313, https://doi.org/10.1016/j.procs.2018.10.313.
[28] C. Guo, M. Liu, M. Lu, A Dynamic Ensemble Learning Algorithm based on K-means for ICU mortality prediction, Appl Soft Comput 103 (2021), 107166, https://doi.org/10.1016/j.asoc.2021.107166.

[29] J. Wu, Y. Lin, K. Lin, Y. Hu, and G. Kong, “Predicting length of stay in intensive care unit using ensemble learning methods,” Apr. 2020, pp. 841–848. doi: 10.1142/9789811223334_0101.
[30] R. Davoodi, M.H. Moradi, Mortality prediction in intensive care units (ICUs) using a deep rule-based fuzzy classifier, J Biomed Inform 79 (2018) 48–59, https://doi.org/10.1016/j.jbi.2018.02.008.
[31] M. Hashir, R. Sawhney, Towards unstructured mortality prediction with free-text clinical notes, J Biomed Inform 108 (2020), 103489, https://doi.org/10.1016/j.jbi.2020.103489.
[32] G. Harerimana, J.W. Kim, B. Jang, A deep attention model to forecast the Length Of Stay and the in-hospital mortality right on admission from ICD codes and demographic data, J Biomed Inform 118 (2021), 103778, https://doi.org/10.1016/j.jbi.2021.103778.
[33] K. Zaineb, K. Aloui, N. Saber, New Approach based on Machine Learning for Short-Term Mortality Prediction in Neonatal Intensive Care Unit, International Journal of Advanced Computer Science and Applications 10 (Nov. 2019), https://doi.org/10.14569/IJACSA.2019.0100778.
[34] A. Mansouri, M. Noei, and M. S. Abadeh, “Predicting Hospital Length of Stay of Neonates Admitted to the NICU Using Data Mining Techniques,” in *2020 10th International Conference on Computer and Knowledge Engineering (ICCKE)*, Oct. 2020, pp. 629–635. doi: 10.1109/ICCKE50421.2020.9303666.
[35] I.M. Ball, S.M. Bagshaw, K.E.A. Burns, D.J. Cook, A.G. Day, P.M. Dodek, D. J. Kutsogiannis, S. Mehta, J.G. Muscedere, H.T. Stelfox, A.F. Turgeon, G.A. Wells, I.G. Stiell, A Clinical Prediction Tool for Hospital Mortality in Critically Ill Elderly Patients, J Crit Care 35 (2016) 206–212.
[36] O.-P. Rinta-Koski, S. Särkkä, J. Hollmén, M. Leskinen, S. Andersson, Gaussian process classification for prediction of in-hospital mortality among preterm infants, Neurocomputing 298 (2018) 134–141, https://doi.org/10.1016/j.neucom.2017.12.064.
[37] R. Pirracchio, “Mortality prediction in the ICU based on MIMIC-II results from the Super ICU Learner Algorithm (SICULA) project,” in *Secondary Analysis of Electronic Health Records*, 2016, pp. 295–313. doi: 10.1007/978-3-319-43742-2_20.
[38] A. E. W. Johnson, T. J. Pollard, and R. Mark, “Reproducibility in critical care: a mortality prediction case study,” 2017.
[39] A.E.W. Johnson, T.J. Pollard, L.u. Shen, L.-W. Lehman, M. Feng, M. Ghassemi, B. Moody, P. Szolovits, L. Anthony Celi, R.G. Mark, MIMIC-III, a freely accessible critical care database, Sci Data 3 (1) (2016), 160035, https://doi.org/10.1038/sdata.2016.35.
[40] S. Fika, S. Nanas, G. Baltopoulos, E. Charitidou, P. Myrianthefs, A novel mortality prediction model for the current population in an adult intensive care unit, Heart and Lung: Journal of Acute and Critical Care 47 (1) (Jan. 2018) 10–15, https://doi.org/10.1016/j.hrtlng.2017.10.009.
[41] R. Sadeghi, T. Banerjee, W. Romine, Early hospital mortality prediction using vital signals, Smart Health 9–10 (2018) 265–274, https://doi.org/10.1016/j.smhl.2018.07.001.
[42] J. Liu, X.X. Chen, L. Fang, J.X. Li, T. Yang, Q. Zhan, K. Tong, Z. Fang, Mortality prediction based on imbalanced high-dimensional ICU big data, Comput Ind 98 (2018) 218–225.
[43] F. Monteiro, F. Meloni, J.A. Baranauskas, A.A. Macedo, Prediction of mortality in Intensive Care Units: a multivariate feature selection, J Biomed Inform 107 (2020), 103456, https://doi.org/10.1016/j.jbi.2020.103456.
[44] M.Z. Alam, M.M. Masud, M.S. Rahman, M. Cheratta, M.A. Nayeem, M.S. Rahman, Feature-ranking-based ensemble classifiers for survivability prediction of intensive care unit patients using lab test data, Inform Med Unlocked 22 (2021), 100495, https://doi.org/10.1016/j.imu.2020.100495.
[45] H. Harutyunyan, H. Khachatrian, D.C. Kale, G. Ver Steeg, A. Galstyan, Multitask Learning and Benchmarking with Clinical Time Series Data, Sci Data 6 (1) (2019), https://doi.org/10.1038/s41597-019-0103-9.
[46] W. Ge, J.-W. Huh, Y. R. Park, J.-H. Lee, Y.-H. Kim, and A. Turchin, “An Interpretable ICU Mortality Prediction Model Based on Logistic Regression and Recurrent Neural Networks with LSTM units,” *AMIA Annu Symp Proc*, vol. 2018, pp. 460–469, Dec. 2018, [Online]. Available: https://pubmed.ncbi.nlm.nih.gov/30815086.
[47] A. Rajkomar, E. Oren, K. Chen, A.M. Dai, N. Hajaj, M. Hardt, P.J. Liu, X. Liu, J. Marcus, M. Sun, P. Sundberg, H. Yee, K. Zhang, Y.i. Zhang, G. Flores, G. E. Duggan, J. Irvine, Q. Le, K. Litsch, A. Mossin, J. Tansuwan, D.e. Wang, J. Wexler, J. Wilson, D. Ludwig, S.L. Volchenboum, K. Chou, M. Pearson, S. Madabushi, N.H. Shah, A.J. Butte, M.D. Howell, C. Cui, G.S. Corrado, J. Dean, Scalable and accurate deep learning with electronic health records, NPJ Digit Med 1 (1) (2018), https://doi.org/10.1038/s41746-018-0029-1.
[48] W. Caicedo-Torres, J. Gutierrez, ISeeU: Visually interpretable deep learning for mortality prediction inside the ICU, J Biomed Inform 98 (2019), 103269, https://doi.org/10.1016/j.jbi.2019.103269.
[49] N. El-Rashidy, T. Abuhmed, L. Alarabi, H.M. El-Bakry, S. Abdelrazek, F. Ali, S. El-Sappagh, Sepsis prediction in intensive care unit based on genetic feature optimization and stacked deep ensemble learning, Neural Comput Appl 34 (5) (2022) 3603–3632.
[50] R.M.O. Cruz, R. Sabourin, G.D.C. Cavalcanti, Dynamic classifier selection: Recent advances and perspectives, Information Fusion 41 (2018) 195–216, https://doi.org/10.1016/j.inffus.2017.09.010.
[51] M.P. Sesmero, J.A. Iglesias, E. Magán, A. Ledezma, A. Sanchis, Impact of the learners diversity and combination method on the generation of heterogeneous classifier ensembles, Appl Soft Comput 111 (2021), 107689, https://doi.org/10.1016/j.asoc.2021.107689.
[52] S. El-Sappagh, H. Saleh, F. Ali, E. Amer, T. Abuhmed, Two-stage deep learning model for Alzheimer's disease detection and prediction of the mild cognitive impairment time, Neural Comput Appl 34 (17) (2022) 14487–14509.
[53] L.I. Kuncheva, A theoretical study on six classifier fusion strategies, IEEE Trans Pattern Anal Mach Intell 24 (2) (2002) 281–286, https://doi.org/10.1109/34.982906.
[54] M. Zounemat-Kermani, O. Batelaan, M. Fadaee, R. Hinkelmann, Ensemble machine learning paradigms in hydrology: A review, J Hydrol (Amst) 598 (2021), 126266, https://doi.org/10.1016/j.jhydrol.2021.126266.
[55] R. Polikar, Ensemble based systems in decision making, IEEE Circuits Syst. Mag. 6 (3) (2006) 21–45, https://doi.org/10.1109/MCAS.2006.1688199.
[56] U. C. Berkeley, E. C. Polley, and M. J. van der Laan, “Super Learner In Prediction.” [Online]. Available: http://biostats.bepress.com/ucbbiostat/paper266.
[57] A. Awad, M. Bader-El-Den, J. McNicholas, J. Briggs, Early Hospital Mortality Prediction of Intensive Care Unit Patients Using an Ensemble Learning Approach, Int J Med Inform 108 (2017) 185–195.
[58] N. El-Rashidy, S. El-Sappagh, T. Abuhmed, S. Abdelrazek, H.M. El-Bakry, Intensive Care Unit Mortality Prediction: An Improved Patient-Specific Stacking Ensemble Model, IEEE Access 8 (2020) 133541–133564, https://doi.org/10.1109/ACCESS.2020.3010556.
[59] K.e. Lin, Y. Hu, G. Kong, Predicting In-hospital Mortality of Patients with Acute Kidney Injury in the ICU Using Random Forest Model, Int J Med Inform 125 (2019) 55–61.
[60] M. Rowan, T. Ryan, F. Hegarty, N. O'Hare, The use of artificial neural networks to stratify the length of stay of cardiac patients based on preoperative and initial postoperative factors, Artif Intell Med 40 (3) (2007) 211–221, https://doi.org/10.1016/j.artmed.2007.04.005.
[61] A.S. Britto, R. Sabourin, L.E.S. Oliveira, Dynamic selection of classifiers—A comprehensive review, Pattern Recognit 47 (11) (2014) 3665–3680, https://doi.org/10.1016/j.patcog.2014.05.003.
[62] M.N. K. p., T. P., Alzheimer's classification using dynamic ensemble of classifiers selection algorithms: A performance analysis, Biomed Signal Process Control 68 (2021) 102729.
[63] P. Zyblewski, R. Sabourin, M. Woźniak, Preprocessed dynamic classifier ensemble selection for highly imbalanced drifted data streams, Information Fusion 66 (2021) 138–154, https://doi.org/10.1016/j.inffus.2020.09.004.
[64] W. Hou, X. Wang, H. Zhang, J. Wang, L. Li, A novel dynamic ensemble selection classifier for an imbalanced data set: An application for credit risk assessment, Knowl Based Syst 208 (2020), 106462, https://doi.org/10.1016/j.knosys.2020.106462.
[65] B. Krawczyk, M. Galar, M. Woźniak, H. Bustince, F. Herrera, Dynamic ensemble selection for multi-class classification with one-class classifiers, Pattern Recognit 83 (2018) 34–51, https://doi.org/10.1016/j.patcog.2018.05.015.
[66] D. Huntley, D.W. Cho, J. Christman, J.G. Csernansky, Predicting length of stay in an acute psychiatric hospital, Psychiatric services 49 (8) (1998) 1049–1053.
[67] L. Turgeman, J.H. May, R. Sciulli, Insights from a machine learning model for predicting the hospital Length of Stay (LOS) at the time of admission, Expert Syst Appl 78 (2017) 376–385.
[68] A. Almashrafi, M. Elmontsri, P. Aylin, Systematic review of factors influencing length of stay in ICU after adult cardiac surgery, BMC Health Serv Res 16 (Nov. 2016), https://doi.org/10.1186/s12913-016-1591-3.
[69] S. Shea, R.V. Sideli, W. DuMouchel, G. Pulver, R.R. Arons, P.D. Clayton, Computer-generated informational messages directed to physicians: effect on length of hospital stay, J Am Med Inform Assoc 2 (1) (1995) 58–64.
[70] T. Grubinger, C. Kobel, K.-P. Pfeiffer, Regression tree construction by bootstrap: Model search for DRG-systems applied to Austrian health-data, BMC Med Inform Decis Mak 10 (Nov. 2010) 9, https://doi.org/10.1186/1472-6947-10-9.
[71] M. Guzman Castillo, “Modelling patient length of stay in public hospitals in Mexico,” 2012.
[72] A. Marshall, C. Vasilakis, E. El-Darzi, Length of Stay-Based Patient Flow Models: Recent Developments and Future Directions, Health Care Manag Sci 8 (3) (2005) 213–220, https://doi.org/10.1007/s10729-005-2012-z.
[73] T. Gentimis, A. Alnaser, A. Durante, K. Cook, and R. Steele, “Predicting Hospital Length of Stay Using Neural Networks on MIMIC III Data,” Nov. 2017, pp. 1194–1201. doi: 10.1109/DASC-PICom-DataCom-CyberSciTec.2017.191.
[74] G. Enderlein, Cox, D. R.; Oakes, D.: Analysis of Survival Data. Chapman and Hall, London – New York 1984, 201 S., £ 12,–, Biom. J. 29 (1) (1987) 114.
[75] L. Garg, S. McClean, B. Meenan, P. Millard, “Phase-Type Survival Trees and Mixed Distribution Survival Trees for Clustering Patients' Hospital Length of Stay”, Informatica Lith. Acad. Sci. 22 (Nov. 2011) 57–72, https://doi.org/10.15388/Informatica.2011.314.
[76] A. Freitas, T. Silva-Costa, F. Lopes, I. Garcia-Lema, A. Teixeira-Pinto, P. Brazdil, A. Costa-Pereira, Factors influencing hospital high length of stay outliers, BMC Health Serv Res 12 (1) (2012), https://doi.org/10.1186/1472-6963-12-265.
[77] C.A. Bain, Myths of ideal hospital occupancy, Med J Aust 193 (5) (2010) 311.
[78] S. McCarthy, Hospital capacity: What is the measure and what is the goal? Med J Aust 193 (Nov. 2010) 252–253, https://doi.org/10.5694/j.1326-5377.2010.tb03898.x.
[79] R. Jones, Myths of ideal hospital size, Med J Aust 193 (Nov. 2010) 298–300, https://doi.org/10.5694/j.1326-5377.2010.tb03912.x.
[80] L. Garg, S. McClean, B. Meenan, P. Millard, A non-homogeneous discrete time Markov model for admission scheduling and resource planning in a cost or capacity constrained healthcare system, Health Care Manag Sci 13 (Nov. 2010) 155–169, https://doi.org/10.1007/s10729-009-9120-0.
[81] F. Zampieri, M. Soares, L. Borges, J. Salluh, O. Ranzani, The Epimed Monitor ICU Database®: A cloud-based national registry for adult intensive care unit patients

in Brazil, Rev Bras Ter Intensiva 29 (Nov. 2017), https://doi.org/10.5935/0103-507X.20170062.
[82] X. Ma, Y. Si, Z. Wang, Y. Wang, Length of stay prediction for ICU patients using individualized single classification algorithm, Comput Methods Programs Biomed 186 (Nov. 2019), 105224, https://doi.org/10.1016/j.cmpb.2019.105224.
[83] C. Li, L. Chen, J. Feng, D. Wu, Z. Wang, J. Liu, W. Xu, Prediction of Length of Stay on the Intensive Care Unit Based on Least Absolute Shrinkage and Selection Operator, IEEE Access 7 (2019) 110710–110721.
[84] T. Daghistani, R. Elshawi, S. Sakr, A.M. Ahmed, A. Al-Thwayee, M.H. Al-Mallah, Predictors of in-hospital length of stay among cardiac patients: A machine learning approach, Int J Cardiol 288 (2019) 140–147.
[85] C. Dominici, A. Salsano, A. Nenna, C. Spadaccio, R. Barbato, G. Mariscalco, F. Santini, F. Biancari, M. Chello, A Nomogram for Predicting Long Length of Stay in The Intensive Care Unit in Patients Undergoing CABG: Results From the Multicenter E-CABG Registry, J Cardiothorac Vasc Anesth 34 (11) (2020) 2951–2961.
[86] M. Rouzbahman, A. Jovicic, M. Chignell, Can Cluster-Boosted Regression Improve Prediction: Death and Length of Stay in the ICU? IEEE J Biomed Health Inform 21 (Nov. 2016) 1, https://doi.org/10.1109/JBHI.2016.2525731.
[87] R.J. LaFaro, S. Pothula, K.P. Kubal, M.E. Inchiosa, V.M. Pothula, S.C. Yuan, D. A. Maerz, L. Montes, S.M. Oleszkiewicz, A. Yusupov, R. Perline, M.A. Inchiosa, Z. Deng, Neural Network Prediction of ICU Length of Stay Following Cardiac Surgery Based on Pre-Incision Variables, PLoS ONE 10 (12) (Nov. 2015) e0145395.
[88] J.V. Tu, M.R.J. Guerriere, Use of a Neural Network as a Predictive Instrument for Length of Stay in the Intensive Care Unit Following Cardiac Surgery, Comput. Biomed. Res. 26 (3) (1993) 220–229.
[89] P.V. Staziaki, D.i. Wu, J.C. Rayan, I.D.d.O. Santo, F. Nan, A. Maybury, N. Gangasani, I. Benador, V. Saligrama, J. Scalera, S.W. Anderson, Machine learning combining CT findings and clinical parameters improves prediction of length of stay and ICU admission in torso trauma, Eur Radiol 31 (7) (2021) 5434–5441.
[90] E. Rocheteau, P. Lio, and S. Hyland, "Predicting Length of Stay in the Intensive Care Unit with Temporal Pointwise Convolutional Networks." Nov. 2020.
[91] L. Garg, S. McClean, M. Barton, B. Meenan, K. Fullerton, Intelligent Patient Management and Resource Planning for Complex, Heterogeneous, and Stochastic Healthcare Systems, Systems, Man and Cybernetics, Part A: Systems and Humans, IEEE Transactions on 42 (Nov. 2012) 1332–1345, https://doi.org/10.1109/TSMCA.2012.2210211.
[92] B.N. Manktelow, S.E. Seaton, D.J. Field, E.S. Draper, Population-Based Estimates of In-Unit Survival for Very Preterm Infants, Pediatrics 131 (2) (2013) e425–e432.
[93] A. Barredo Arrieta, N. Díaz-Rodríguez, J. Del Ser, A. Bennetot, S. Tabik, A. Barbado, S. Garcia, S. Gil-Lopez, D. Molina, R. Benjamins, R. Chatila, F. Herrera, Explainable Artificial Intelligence (XAI): Concepts, taxonomies, opportunities and challenges toward responsible AI, Information Fusion 58 (2020) 82–115.
[94] J. Zhu, A. Liapis, S. Risi, R. Bidarra, G.M. Youngblood, "Explainable AI for Designers: A Human-Centered Perspective on Mixed-Initiative Co-Creation", in, IEEE Conference on Computational Intelligence and Games (CIG) 2018 (2018) 1–8, https://doi.org/10.1109/CIG.2018.8490433.
[95] G. Vilone, L. Longo, Notions of explainability and evaluation approaches for explainable artificial intelligence, Information Fusion 76 (2021) 89–106.
[96] S. El-Sappagh, J.M. Alonso, S.M.R. Islam, A.M. Sultan, K.S. Kwak, A multilayer multimodal detection and prediction model based on explainable artificial intelligence for Alzheimer's disease, Sci Rep 11 (1) (2021) 2660, https://doi.org/10.1038/s41598-021-82098-3.
[97] C. Panigutti, D. Pedreschi, and A. Perotti, "Doctor XAI: an ontology-based approach to black-box sequential data classification explanations," Nov. 2020, doi: 10.1145/3351095.3372855.
[98] D. Wang, Q. Yang, A. Abdul, and B. Lim, "Designing Theory-Driven User-Centric Explainable AI," Nov. 2019. doi: 10.1145/3290605.3300831.
[99] E. Parimbelli, G. Nicora, S. Wilk, W. Michalowski, and R. Bellazzi, "Tree-based local explanations of machine learning model predictions, AraucanaXAI." Nov. 2021.
[100] I. Gandin, A. Scagnetto, S. Romani, G. Barbati, Interpretability of time-series deep learning models: A study in cardiovascular patients admitted to Intensive care unit, J Biomed Inform 121 (2021), 103876, https://doi.org/10.1016/j.jbi.2021.103876.
[101] S.N. Payrovnaziri, et al., Explainable artificial intelligence models using real-world electronic health record data: A systematic scoping review, J Am Med Inform Assoc 27 (Nov. 2020), https://doi.org/10.1093/jamia/ocaa053.
[102] E. Tjoa, C. Guan, A Survey on Explainable Artificial Intelligence (XAI): Toward Medical XAI, IEEE Trans Neural Netw Learn SystPP 32 (11) (2021) 4793–4813.
[103] A.E.W. Johnson, T.J. Pollard, L.u. Shen, L.-W. Lehman, M. Feng, M. Ghassemi, B. Moody, P. Szolovits, L. Anthony Celi, R.G. Mark, Data Descriptor: MIMIC-III, a freely accessible critical care database, Thromb Haemost 3 (1) (2016), https://doi.org/10.1038/sdata.2016.35.
[104] X. Huang, Z. Liang, T. Li, Y. Lingna, W. Zhu, H. Li, A nomogram to predict in-hospital mortality of neonates admitted to the intensive care unit, Int Health 13 (6) (Nov. 2021) 633–639, https://doi.org/10.1093/inthealth/ihab012.
[105] S. El-Sappagh, T. Abuhmed, S.M. Riazul Islam, K.S. Kwak, Multimodal multitask deep learning model for Alzheimer's disease progression detection based on time series data, Neurocomputing 412 (2020) 197–215, https://doi.org/10.1016/j.neucom.2020.05.087.
[106] J. Wen, E. Thibeau-Sutre, M. Diaz-Melo, J. Samper-González, A. Routier, S. Bottani, D. Dormont, S. Durrleman, N. Burgos, O. Colliot, Convolutional neural networks for classification of Alzheimer's disease: Overview and reproducible evaluation, Med Image Anal 63 (2020) 101694.
[107] N.V. Chawla, K.W. Bowyer, L.O. Hall, W.P. Kegelmeyer, SMOTE: Synthetic Minority over-Sampling Technique, J. Artif. Int. Res., Jun. 16 (2002) 321–357.
[108] N. Japkowicz, "The Class Imbalance Problem, Significance and Strategies" (2000).
[109] S. Kim, W. Kim, R.W. Park, A Comparison of Intensive Care Unit Mortality Prediction Models through the Use of Data Mining Techniques, Healthc Inform Res 17 (4) (2011) 232.
[110] R. Viegas, C.M. Salgado, S. Curto, J.P. Carvalho, S.M. Vieira, S.N. Finkelstein, Daily prediction of ICU readmissions using feature engineering and ensemble fuzzy modeling, Expert Syst Appl 79 (2017) 244–253, https://doi.org/10.1016/j.eswa.2017.02.036.
[111] M.N.M. García, J.C.B. Herráez, M.S. Barba, F.S. Hernández, "Random Forest Based Ensemble Classifiers for Predicting Healthcare-Associated Infections in Intensive Care Units BT - Distributed Computing and Artificial Intelligence, 13th International Conference" (2016) 303–311.
[112] M. Zabihi, S. Kiranyaz, and M. Gabbouj, "Sepsis Prediction in Intensive Care Unit Using Ensemble of XGboost Models," in *2019 Computing in Cardiology (CinC)*, 2019, p. Page 1-Page 4. doi: 10.23919/CinC49843.2019.9005564.
[113] G. Chandrashekar, F. Sahin, A survey on feature selection methods, Comput. Electr. Eng. 40 (1) (2014) 16–28.
[114] O. Kramer, "K-Nearest Neighbors", in *Dimensionality Reduction with Unsupervised Nearest Neighbors*, Berlin, Heidelberg: Springer, Berlin Heidelberg (2013) 13–23, https://doi.org/10.1007/978-3-642-38652-7_2.
[115] B. Minasny, The Elements of Statistical Learning, Trevor Hastie, Robert Tishirani, Jerome Friedman, (2009), Springer Series in Statistics, ISBN 0172-7397, 745 pp, Elsevier, 2009.
[116] A. V. Dorogush, V. Ershov, and A. Gulin, "CatBoost: gradient boosting with categorical features support," *ArXiv*, vol. abs/1810.11363, 2018.
[117] T. Chen and C. Guestrin, "XGBoost: A Scalable Tree Boosting System," in *Proceedings of the 22nd ACM SIGKDD International Conference on Knowledge Discovery and Data Mining*, 2016, pp. 785–794. doi: 10.1145/2939672.2939785.
[118] G. Ke, et al., "LightGBM: A Highly Efficient Gradient Boosting Decision Tree", in *Advances in Neural Information* [Online] Available: Processing Systems 30 (2017) https://proceedings.neurips.cc/paper/2017/file/6449f44a102fde848669bdd9eb6b76fa-Paper.pdf.
[119] T. Abuhmed, S. El-sappagh, J.M. Alonso, Robust hybrid deep learning models for Alzheimer's progression detection, Knowl Based Syst 213 (2021), 106688, https://doi.org/10.1016/j.knosys.2020.106688.
[120] D.V.R. Oliveira, G.D.C. Cavalcanti, R. Sabourin, Online pruning of base classifiers for Dynamic Ensemble Selection, Pattern Recognit 72 (2017) 44–58, https://doi.org/10.1016/j.patcog.2017.06.030.
[121] M. Canizo, I. Triguero, A. Conde, E. Onieva, Multi-head CNN–RNN for multi-time series anomaly detection: An industrial case study, Neurocomputing 363 (2019) 246–260, https://doi.org/10.1016/j.neucom.2019.07.034.
[122] K. Wang, K. Li, L. Zhou, Y. Hu, Z. Cheng, J. Liu, C. Chen, Multiple convolutional neural networks for multivariate time series prediction, Neurocomputing 360 (2019) 107–119.
[123] C.-L. Liu, W.-H. Hsaio, Y.-C. Tu, Time Series Classification With Multivariate Convolutional Neural Network, IEEE Trans. Ind. Electron. 66 (6) (2019) 4788–4797.
[124] H. Ismail Fawaz, G. Forestier, J. Weber, L. Idoumghar, P.-A. Muller, Deep learning for time series classification: a review, Data Min Knowl Discov 33 (4) (2019) 917–963, https://doi.org/10.1007/s10618-019-00619-1.
[125] A.H.R. Ko, R. Sabourin, Jr. Britto Alceu Souza, From dynamic classifier selection to dynamic ensemble selection, Pattern Recognit 41 (5) (2008) 1718–1731, https://doi.org/10.1016/j.patcog.2007.10.015.
[126] T. Woloszynski, M. Kurzynski, P. Podsiadlo, G.W. Stachowiak, A measure of competence based on random classification for dynamic ensemble selection, Information Fusion 13 (3) (2012) 207–213, https://doi.org/10.1016/j.inffus.2011.03.007.
[127] R.M.O. Cruz, R. Sabourin, G.D.C. Cavalcanti, T. Ing Ren, META-DES: A dynamic ensemble selection framework using meta-learning, Pattern Recognit 48 (5) (2015) 1925–1935, https://doi.org/10.1016/j.patcog.2014.12.003.
[128] R. G. F. Soares, A. Santana, A. M. P. Canuto, and M. C. P. de Souto, "Using Accuracy and Diversity to Select Classifiers to Build Ensembles," in:The 2006 IEEE International Joint Conference on Neural Network Proceedings, pp. 1310–1316, 2006.
