# Supplementary material for: Exploring Computational Techniques in Preprocessing Neonatal Physiological Signals for Detecting Adverse Outcomes: Scoping Review
Source: Interact J Med Res. 2024 Aug 20;13:e46946. doi: 10.2196/46946 (PMC11372324; doi:10.2196/46946)
Supplement: Multimedia Appendix 3 [file ijmr_v13i1e46946_app3.zip › Included Papers - Final/4386/Honoré et al. - Vital sign-based detection of sepsis in neonates u.pdf]

Herlenius Eric (Orcid ID: 0000-0002-6859-0620)

Accepted Article

Vital sign-based detection of sepsis in neonates using machine learning

Antoine Honoré[1, 2, 3#]; David Forsberg[1, 2#]; Katja Adolphson[1, 2]; Saikat Chatterjee[3]; Kerstin Jost[1, 2]; Eric Herlenius*[1, 2]

1: Department of Women's and Children's Health, Karolinska Institutet, Stockholm, Sweden

2: Astrid Lindgren Children's Hospital, Karolinska University Hospital, Stockholm, Sweden

3: Division of Information Science and Engineering, Royal Institute of Technology - KTH, Stockholm, Sweden

Short Title: Newborn non-invasive sepsis prediction.

Corresponding author*

Eric Herlenius, Department of Women's and Children's Health Karolinska Institutet | Karolinska University Hospital, CMM Center for Molecular Medicine L8:01, 171 76, Solna, Stockholm, Sweden.

Email: eric.herlenius@ki.se tel: 0046/ 851775042

# Equal contribution

Category of study: Clinical

This article has been accepted for publication and undergone full peer review but has not been through the copyediting, typesetting, pagination and proofreading process which may lead to differences between this version and the Version of Record. Please cite this article as doi: 10.1111/apa.16660

This article is protected by copyright. All rights reserved.

16512227, ja, Downloaded from https://onlinelibrary.wiley.com/doi/10.1111/apa.16660 by CSIRO Library Services Information Support, Wiley Online Library on [08/01/2023]. See the Terms and Conditions (https://onlinelibrary.wiley.com/terms-and-conditions) on Wiley Online Library for rules of use; OA articles are governed by the applicable Creative Commons License

Key words; Artificial intelligence, Clinical Decision Support system, Naïve-Bayes classifier, prediction, physiological monitoring, respiration-related.

Abbreviations: NICU, Neonatal Intensive Care Unit; HR, heart rate; RR, respiratory rate; SpO_2, peripheral oxygen saturation; EHR, electronic health records; VLBW, very low birth weight; GDPR, General Data Protection Regulation; LOS, late onset sepsis; LR, likelihood radio; PPV, positive predictive value; NPV, negative predictive value; IQR, interquartile range; nSOFA, neonatal Sequential Organ Failure Assessment.

Accepted Article

16512227, ja, Downloaded from https://onlinelibrary.wiley.com/doi/10.1111/apa.16660 by CSIRO Library Services Information Support, Wiley Online Library on [08/01/2023]. See the Terms and Conditions (https://onlinelibrary.wiley.com/terms-and-conditions) on Wiley Online Library for rules of use; OA articles are governed by the applicable Creative Commons License

Aim: Sepsis is a leading cause of morbidity and mortality in neonates. Early diagnosis is key but difficult due to non-specific signs. We investigate the predictive value of machine learning-assisted analysis of non-invasive, high frequency monitoring data and demographic factors to detect neonatal sepsis.

Methods: Single center study, including a representative cohort of 325 infants (2866 hospitalization days). Personalized event timelines including interventions and clinical findings were generated. Time-domain features from heart rate, respiratory rate and oxygen saturation values were calculated and demographic factors included. Sepsis prediction was performed using Naïve-Bayes algorithm in a maximum a posteriori framework up to 24 hours before clinical sepsis suspicion.

Results: Twenty sepsis cases were identified. Combining multiple vital signs improved algorithm performance compared to heart rate characteristics alone. This enabled a prediction of sepsis with an area under the receiver operating characteristics curve of 0.82, up to 24 hours before clinical sepsis suspicion. Moreover, 10 hours prior to clinical suspicion, the risk of sepsis increased 150-fold.

Conclusion: The present algorithm using non-invasive patient data provides useful predictive value for neonatal sepsis detection. Machine learning-assisted algorithms are promising novel methods that could help individualize patient care and reduce morbidity and mortality.

Accepted Article

16512227, ja, Downloaded from https://onlinelibrary.wiley.com/doi/10.1111/apa.16660 by CSIRO Library Services Information Support, Wiley Online Library on [08/01/2023]. See the Terms and Conditions (https://onlinelibrary.wiley.com/terms-and-conditions) on Wiley Online Library for rules of use; OA articles are governed by the applicable Creative Commons License

Key Notes

- We present a machine learning-based algorithm using vital signs and demographic factors for sepsis detection in neonates up to 24 hours before first clinical suspicion.
- Adding respiratory-related vital signs improved performance compared to heartrate characteristics alone.
- Notably, sepsis detection achieved a median area under the receiver operating characteristics curve of 0.82, in a representative cohort including preterm and term newborns, with possible betterment of personalized medicine for all neonatal infants.

Introduction

Newborns are fragile and many born both term and preterm, experience life-threatening events during their first weeks of life each year.(1) Despite hospitalization in a neonatal intensive care unit (NICU) and vigilant clinical assessment, diagnosis of infection-related events such as sepsis is a difficult task. Therefore, sepsis remains a leading cause of death in neonates with a mortality rate reported between 11 and 19%.(2) Early symptoms of systemic infections in neonates are often sparse. Additionally, although sepsis most commonly is defined as the identification of a bacterial pathogen in the blood, the lack of consensus on the definition of neonatal sepsis has made comparison between different studies and hence advances in daily clinical practice almost impossible.(3-5)

The standard monitoring systems in NICUs usually record vital signs such as heart rate (HR), respiratory rate (RR), and peripheral oxygen saturation (SpO_2) continuously. Notably, this contrasts with electronic health records (EHRs) that are manually updated several times a day by the medical staff. This results in an enormous amount of data that may contain important in-

Accepted Article

16512227, ja, Downloaded from https://onlinelibrary.wiley.com/doi/10.1111/apa.16660 by CSIRO Library Services Information Support, Wiley Online Library on [08/01/2023]. See the Terms and Conditions (https://onlinelibrary.wiley.com/terms-and-conditions) on Wiley Online Library for rules of use; OA articles are governed by the applicable Creative Commons License

formation about impending clinical deterioration.(6, 7) Several clinical decisions support systems based on vital sign patterns have been developed and seem promising, but often lack generalizability and are developed for subgroups within the NICU population.(7-11) In a pilot study, consisting of 22 infants at the Karolinska NICU, we showed that Hidden Markov models outperform logistic regression-based classification models for neonatal sepsis detection.(12) In the present study, we investigate the performance of a sepsis detection algorithm using timely aligned high frequency physiomarkers from standard monitoring systems and EHR annotations representing the whole duration of the NICU stay for each individual patient.

We aim to develop a machine learning-based algorithm for sepsis detection that utilizes heart rate characteristics respiratory features and unbiased inclusion of a whole NICU population. This in contrast to most algorithms and clinical decision support systems, developed and evaluated on a subgroup of the NICU patients. Classical deep learning architectures are capable of modeling complex data distributions but often require large cohorts to be trained. Since the size of our cohort is limited, we choose a Naïve-Bayes model, in a maximum a posteriori framework, which learns the data distribution with limited training data, without overfitting.(13)

We specifically: i) evaluate heart rate characteristics (HRC)-based prediction algorithms on the overall, representative NICU population; ii) compare the efficiency of HRC in combination with other high frequency vital signs, namely RR and SpO_2; and iii) test the performance in a subgroup analysis on the very low birth weight (VLBW, <1500g) infant population for comparison with previously published algorithms within this subgroup.

Material and Methods

Study design and population

Accepted Article

16512227, ja, Downloaded from https://onlinelibrary.wiley.com/doi/10.1111/apa.16660 by CSIRO Library Services Information Support, Wiley Online Library on [08/01/2023]. See the Terms and Conditions (https://onlinelibrary.wiley.com/terms-and-conditions) on Wiley Online Library for rules of use; OA articles are governed by the applicable Creative Commons License

Waveform monitoring data was stored prospectively from infants hospitalized in the NICUs of Karolinska University Hospital Solna and Huddinge, Stockholm, Sweden, between February 2016 and April 2020. Intermittent monitoring data availability, due to networking issues, allowed access to a total of 23 months (2016; 1 month, 2017; 5 months, 2018; 3 months, 2019; 10 months, 2020; 4 months). EHR data was collected retrospectively after discharge of the patients. No features or models were available to the care team, and all patients received standard of care. All data was stored and utilized in a General Data Protection Regulation (GDPR)-compliant IT infrastructure. Inclusion criteria was admission to the NICU and exclusion criteria was monitor/EHR data missing completely or mismatched (acquired for different time periods). The Swedish ethical review board approved this study with a waiver of consent (#2020-02487, granted in May 2020, including all data of patients that were hospitalized and already discharged since February 2016). A requirement of consent would risk the bias of excluding infants with severe illness, multiple complications and those who died, i.e. those who potentially would benefit most from the study results. Additionally, it would increase the risk of systematically excluding non-English and non-Swedish speaking parents.

Data collection

Vital sign (inter-beat-interval derived from electrocardiography, RR derived from chest impedance and SpO_2 from pulse oximetry) data was obtained from standard patient monitors used in daily clinical care (Philips IntelliVue MX800 Patient Monitor, Philips Healthcare, Amsterdam, the Netherlands). Raw, high frequency waveforms (1-500 Hz) were automatically transferred, along with lower frequency inter-beat-interval, RR and SpO_2 parameter data, to Karolinska central data servers and subsequently to servers at Karolinska Institutet, Stockholm, Sweden. The pre-computed parameter data were all resampled at a frequency of 1 Hz to synchronize the

Accepted Article

16512227, ja, Downloaded from https://onlinelibrary.wiley.com/doi/10.1111/apa.16660 by CSIRO Library Services Information Support, Wiley Online Library on [08/01/2023]. See the Terms and Conditions (https://onlinelibrary.wiley.com/terms-and-conditions) on Wiley Online Library for rules of use; OA articles are governed by the applicable Creative Commons License

samples from all three signals. Missing segments of at most 15 seconds were linearly interpolated. The inter-beat-interval, RR, and SpO_2 signals were filtered with a moving mean filter of width 3 to smooth the signals and the interpolated regions.

The inter-beat-interval signal was further filtered due to the use of the more sensitive sample entropy feature. To remove ectopic beats and strong nonlinearities we used a composition of a moving median filter of width 3 samples and a passband Butterworth filter of order 6 with low cut frequency of 0.0021 Hz and high cut frequency 0.43 Hz.

For each individual patient, factors from pregnancy, delivery, and postnatal period were collected and a personal event timeline based on EHR annotations was generated (see example in supplemental Figure 1). EHRs (TakeCare, CompuGroup Medical Sweden AB, Stockholm, Sweden and Obstetrix, Cerner Sverige AB, Stockholm, Sweden) were used to identify patient characteristics and events covering the entire hospitalization based on a nomenclature that was built together with a team of neonatologists for this study. The nomenclature included events such as sepsis (e.g. late onset sepsis (LOS), early onset sepsis (EOS), bacterial culture positive or negative sepsis), other infections as well as other complications and co-morbidities. For every event, the time-stamp of the clinical note or specification in the written text was used. Each timeline was generated by a trained team member and reviewed for accuracy by another. Discrepancies were solved by discussion or inclusion of a third examiner. In the present study, only the late onset sepsis events (>72h postnatal age) were included in the algorithm development.

Sepsis definition

Accepted Article

16512227, ja, Downloaded from https://onlinelibrary.wiley.com/doi/10.1111/apa.16660 by CSIRO Library Services Information Support, Wiley Online Library on [08/01/2023]. See the Terms and Conditions (https://onlinelibrary.wiley.com/terms-and-conditions) on Wiley Online Library for rules of use; OA articles are governed by the applicable Creative Commons License

Sepsis was defined as a neonatologist's documented assessment of the patient as having sepsis with antibiotic treatment for at least five days, culture positive or negative, similar to other studies.(3, 11) Time of sepsis onset was defined as clinical suspicion of sepsis, marked by the time the first blood culture was drawn. When two or more sepsis episodes occurred within 14 days they were noted as the same septic episode, with the first blood culture used as time of suspicion, so that the recovery of the first sepsis episode doesn't interfere with detection of the second in the training of the algorithm.

Data analysis

The clinical event timeline was reduced to binary labels, "1" indicating sepsis diagnosis, "0" otherwise. Simultaneously acquired continuous data on inter-beat-interval, RR and SpO_2 was divided into frames of 45 minutes. This resulted in state-of-art algorithm performance, as previously presented.(13) Patients were included when at least one such frame was available. No minimum number of frames prior to an event were required. A frame was labeled "1" if the start of the frame was between 24 h before and 4 h after a sepsis diagnosis. We computed features on sliding time frames with 50% overlap, i.e. a prediction is made every 22.5 minutes. In clinical practice, the frame overlap can be tuned and predictions can be obtained arbitrarily frequently. The features used for all three signals (inter-beat-interval, RR and SpO_2) were minimum, maximum, mean, standard deviation (SD), skewness, and kurtosis. Additionally, the sample entropy and the sample asymmetry of the inter-beat-interval signal was calculated to include the features used in the commercially available HeRO system.(14) The SD, sample asymmetry, and the sample entropy of the inter-beat-interval signal are denoted as HRC features in the rest of the text.

Accepted Article

16512227, ja, Downloaded from https://onlinelibrary.wiley.com/doi/10.1111/apa.16660 by CSIRO Library Services Information Support, Wiley Online Library on [08/01/2023]. See the Terms and Conditions (https://onlinelibrary.wiley.com/terms-and-conditions) on Wiley Online Library for rules of use; OA articles are governed by the applicable Creative Commons License

The sepsis detection algorithm consists of two competing statistical models: one model to detect signs of sepsis in the sepsis time frames, and one to detect non-sepsis time frames. The time frames are assumed independent. The models were trained with the objective to give high probabilities to the training time frames of their respective class. The two statistical models chosen were multivariate Gaussian distributions with diagonal covariance matrices.(15) This corresponds to the Gaussian Naïve-Bayes binary classifier used in a case control study by Joshi et al. (8)To train the sepsis detection algorithm, infants were randomly split into a training and a validation set using a leave patients out cross-validation scheme. This scheme was used independently for patients in the negative and positive population. We ensured that (1) two positive and 20% of the negative patients were used for validation, (2) training and validation were always performed with different patients and (3) all the patients from the positive population were used at least once as validation patients. This scheme was repeated three times, leading to 24 combinations of training and validation patients in total Python (Python programming language, version 3.7.4, Python Software Foundation, Wilmington, Delaware) with package scikit-learn (Scikit-learn, version 0.21.1, Cournapeau D, http://www.scikit-learn.org) was used to implement the model.

Various data processing and statistical models were evaluated and presented in a preprint.(13) Based on that discussion we selected the parameters used in this study. Please see Appendix S1 and Honoré *et al.*(13) for extended description of the data analysis.

Outcome assessment

The primary outcome was the predictive ability of the described features from standard monitoring vital sign time-series on sepsis onset. Two combinations of specific vital sign characteristics assessed for each window of 45 minutes were applied separately (feature sets); 1; HRC

Accepted Article
16512227, ja, Downloaded from https://onlinelibrary.wiley.com/doi/10.1111/apa.16660 by CSIRO Library Services Information Support, Wiley Online Library on [08/01/2023]. See the Terms and Conditions (https://onlinelibrary.wiley.com/terms-and-conditions) on Wiley Online Library for rules of use; OA articles are governed by the applicable Creative Commons License

alone, 2; HRC combined with minimal-maximal, mean, SD, kurtosis and skewness of the RR and SpO_2 signals. Thus, feature set 2 added respiratory features to the cardiac (HRC) features. Moreover, addition of demographic factors (postnatal age, birth weight and sex) to the model was assessed. The algorithm was evaluated in the whole study cohort and in a subgroup of VLBW infants.

Statistical analysis

Normal distribution for the numeric continuous measures was assessed using density plots and tested with Shapiro-Wilks normality test. Comparisons of birth weight, gestational age (GA) and length of hospitalization data were assessed using Kruskal-Wallis-ANOVA. Categorical measures (delivery mode, sex and prematurity classification) were analyzed using Pearson's Chi-squared test. The predictive algorithms were calibrated using the isotonic regression method on the training sets. The ability of our algorithm to discriminate between septic and non-septic time frames was measured with several performance metrics. We presented specificity and sensitivity, area under receiver operating characteristics (AUROC) curve, positive predictive value (PPV), negative predictive value (NPV), positive likelihood ratio (LR+), and negative likelihood radio (LR-) on the validation sets and compared using ANOVA. Based on the average LR+ and LR-, a Fagan's normogram was calculated for the overall population and the VLBW subgroup, respectively (http://araw.mede.uic.edu/cgi-bin/testcalc.pl?DT=39&Dt=6&dT=2691&dt=10717&2x2=Compute) and http://araw.mede.uic.edu/cgi-bin/testcalc.pl?DT=73&Dt=4&dT=1410&dt=7028&2x2=Compute).

Additionally, we show the predicted risk score (*i.e.* the probability of a frame being classified as "1"), versus time to sepsis diagnosis, both as individual risk scores and a smoothened curve

Accepted Article

16512227, ja, Downloaded from https://onlinelibrary.wiley.com/doi/10.1111/apa.16660 by CSIRO Library Services Information Support, Wiley Online Library on [08/01/2023]. See the Terms and Conditions (https://onlinelibrary.wiley.com/terms-and-conditions) on Wiley Online Library for rules of use; OA articles are governed by the applicable Creative Commons License

over time. We also present the population distribution of the predicted risk scores for positive patients over time. The risk score prediction timelines of different patients were aggregated by grouping the predictions in 4h bins. The distributions over time are shown in terms of their density and third quartile. Post hoc analysis was performed using Tukey's HSD (honestly significant difference) test and utilizing a Bonferroni correction due to the high number of individual comparisons. A p-value <0.05 was considered statistically significant. All descriptive statistics were performed using OriginPro 2020 (v9.7, OriginLabs, Northampton, MA).

Results

Patient characteristics

During 23 months, spanning a 4-year period (February 2016 until April 2020), a population of 489 infants (8311 monitoring days) were identified for this study cohort (for patient characteristics see Table 1). For 164 of these, data from both modalities (EHR and data of all three vital signs of interest) did not overlap for included time period and were thus excluded. The non-overlapping data sets mainly resulted from temporary technical issues in data transfer from monitors, patients temporarily being disconnected from the monitors or that not all vital signs were monitored simultaneously. Data from a total of 325 infants (2866 monitoring days) was utilized for algorithm development and subsequent analysis. Patient characteristics are summarized in Table 1. The median birth weight was 2524 g (IQR 2184 g), with 29 % having a VLBW (<1500 g). The median GA was 36 weeks (IQR 10 weeks) with half (53 %) born preterm (GA <37 weeks). The study cohort did not differ from the identified NICU population

Accepted Article

16512227, ja, Downloaded from https://onlinelibrary.wiley.com/doi/10.1111/apa.16660 by CSIRO Library Services Information Support, Wiley Online Library on [08/01/2023]. See the Terms and Conditions (https://onlinelibrary.wiley.com/terms-and-conditions) on Wiley Online Library for rules of use; OA articles are governed by the applicable Creative Commons License

with respect to birth weight and GA (p>0.05). The overall NICU population had fewer cases of extreme prematurity compared to the included study cohort and VLBW subgroup (p<0.05). The sex distribution did not differ between the groups. The frequency of vaginal birth was higher in the overall population compared to the study cohort (p<0.05) and the VLBW group had a lower frequency of vaginal birth and elective cesarean section but a higher frequency of acute cesarean section (p<0.05). One infant (VLBW), not experiencing a sepsis episode, died.

In total, 20 late onset sepsis cases were identified in 18 unique patients, among which 16 patients were VLBW. The sepsis was first suspected clinically at median postnatal age of 15 days (IQR 17 days). In eight of the sepsis patients full continuous monitoring data were not available during the sepsis event. Infants experiencing sepsis had a median birth weight of 636 g (IQR 377 g) and GA of 24 weeks (IQR 3 weeks). This was lower (p<0.05), when compared to the infants without sepsis. Information about culture findings is summarized in Table S1.

Diagnostic accuracy

During the hours leading up to the time of clinical sepsis suspicion, an increased risk of sepsis based on the vital sign analysis was evident. Averaging the risk for the whole population showed a more than 150-fold increase in risk of sepsis between 10 to 6 hours prior to clinical suspicion. This compared to a reference median risk calculated 48 hours away from clinical suspicion (**Figure 1a**). Among the patients later developing sepsis, we observed an increased proportion of patients with a high risk while the proportion of patients presenting a low sepsis risk decreased from 20 hours prior to clinical suspicion (**Figure 1b**). The dynamics of the sepsis risk was evident in risk over time for individual patients (**Figure 1c**). Since the analysis is based on multiple iterations with varying validation and training splitting of the cohort, a more detailed individualized risk assessment has not been feasible in the current study setup.

Accepted Article

16512227, ja, Downloaded from https://onlinelibrary.wiley.com/doi/10.1111/apa.16660 by CSIRO Library Services Information Support, Wiley Online Library on [08/01/2023]. See the Terms and Conditions (https://onlinelibrary.wiley.com/terms-and-conditions) on Wiley Online Library for rules of use; OA articles are governed by the applicable Creative Commons License

Within the entire NICU study cohort the sepsis detection algorithm generated median AUROCs between 0.69 and 0.81, depending on the input variables. Adding demographic factors increased the prognostic ability (**Figure 2a**, $p<0.001$). The prognostic ability did not improve, measured as AUROC, when including respiratory features over cardiac (HRC) features alone, even if there was a trend (**Figure 2b** and **c**, and Table S2, p=0.12). However, positive likelihood ratios varied between 1.7 and 3.5 and the negative likelihood ratio between 0.2 and 0.5. Notably, the positive likelihood ratio was significantly higher when including respiratory features compared to HRC alone (**Figure 2d**, $p<0.001$). The pre-test probability of sepsis in our cohort was 0.3 %. The positive post-test probability, calculated from the likelihood ratio, more than quadrupled using the algorithm (1,4%) while the negative post-test probability was lowered to 0.1% (or 1 in 1000 negative frames would be identified incorrectly) (**Figure 2d**).

In the VLBW subpopulation median AUROCs were between 0.69 and 0.82, positive likelihood ratios between 1.7 and 3.3 and negative likelihood ratios between 0.2 and 0.5. The fold risk of sepsis increased to 50 times higher risk scores 10 hours prior to clinical suspicion (**Figure 3a** and b). As in the whole cohort, including demographic factors increased the prognostic ability (**Figure 3c**, $p<0.001$) while adding respiratory features to cardiac features indicated higher but did not attain significantly better predictive value (**Figure 3d**, p=0.17).

The pre-test probability of sepsis in the VLBW cohort was 0.9 %. The positive post-test probability, calculated from the likelihood ratio, increased to 5% using the algorithm (or 1 in 20 positive frames would be identified correctly), while the negative post-test probability was lowered to 0.1% (or 1 in 1000 negative frames would be identified incorrectly) (**Figure 3e**).

The specificity for all feature combinations was high, while sensitivity was lower (see Table S2). Consequently, the sepsis detection algorithm provides a high NPV and low PPV.

Discussion

Accepted Article

16512227, ja, Downloaded from https://onlinelibrary.wiley.com/doi/10.1111/apa.16660 by CSIRO Library Services Information Support, Wiley Online Library on [08/01/2023]. See the Terms and Conditions (https://onlinelibrary.wiley.com/terms-and-conditions) on Wiley Online Library for rules of use; OA articles are governed by the applicable Creative Commons License

Here we applied a new machine learning algorithm to evaluate the predictive ability of non-invasive, high frequency monitoring data for sepsis detection in a representative NICU population. Our algorithm that includes cardiac (HRC) and respiratory (RR and SpO_2) features as well as basic demographic factors result in a high specificity and negative predictive value up to 24 hours before clinical suspicion of sepsis. Of special interest for smooth implementation into daily clinical practice is that our results were generated from the standard monitoring data and in a non-selective patient group without allocation into matched case-control datasets.

Combination of several vital sign features and demographic factors improve algorithm performance

Early diagnosis of sepsis is important but difficult due to subtle initial symptoms. Biomarkers are often assessed but are slow and of limited value due to low specificity.(16) Blood culture is considered the gold standard for the diagnosis of neonatal sepsis,(16) but is remarkably insensitive and time to detection can be up to 72 hours.(17) This results in delayed treatment, long-term morbidity and potentially fatal outcome.(18)

A growing body of research suggests that analysis of subtle changes in vital sign patterns can help clinicians diagnose diseases such as sepsis and necrotizing enterocolitis earlier in newborns.(10, 19, 20) Recently the semi-manually calculated, low frequency neonatal Sequential Organ Failure Assessment (nSOFA) score that evaluates respiratory support, SpO_2, lab values and need for medication, provided an AUROC for mortality of 0.91.(21) The usefulness of a clinical decision support system based on HRC was shown in a large randomized clinical trial including about 3000 VLBW infants.(7, 22) A significant reduction in septicaemia-related mortality was associated with displaying an HRC-based warning score reaching AUROCs between 0.67 and 0.78.(7, 22) The present model, developed from a significantly smaller cohort, reached

Accepted Article
16512227, ja, Downloaded from https://onlinelibrary.wiley.com/doi/10.1111/apa.16660 by CSIRO Library Services Information Support, Wiley Online Library on [08/01/2023]. See the Terms and Conditions (https://onlinelibrary.wiley.com/terms-and-conditions) on Wiley Online Library for rules of use; OA articles are governed by the applicable Creative Commons License

AUROCs of 0.81 when using the most advanced vital sign input features and adding information about postnatal age and demographic factors. Moreover, here we calculated and show the likelihood ratio. This is used to assess how much a test, in our setting the vital sign analysis algorithm, adds to the likelihood of correctly diagnosing a disease, here neonatal sepsis.(23) This has unfortunately not been done in previous algorithm evaluations. Notably, we show that our algorithm increases the probability of being correct in categorizing time frames as both positive and negative, respectively.

In addition to changes in heart rate, the frequency of apnea with associated desaturation and altered respiratory patterns can indicate an emerging instability and sepsis in newborns.(24-28) Therefore, the analysis of several vital signs depicting more closely the multi-organ genesis of a systemic infection has the potential of adding precision in sepsis detection. Notably, combining multiple features improved the ability of our algorithm to recognize early signs of sepsis. This is in agreement with recent suggestions from two case control studies(29, 30) and expected based on our previous findings.(24, 25) Including respiratory features into the present algorithm resulted in an additive predictive value for sepsis detection with an increase in positive likelihood ratio (1.6, 62%). Furthermore, birth weight and postnatal age influence autonomic control and thus affect vital signs and sex is an important determinant factor for morbidity risk in preterm infants. We therefore included them as input variables in the algorithm. This improved the performances and thus allowed our algorithm to function in a broader NICU patient population than previously investigated.(7, 29, 30) Notably, despite the majority of sepsis patients being VLBW, sepsis episodes could be identified within the overall NICU population equally good as within the VLBW subgroup. The current method does not include treatment strategies such as drugs or information about ventilation status, therefore this correlation cannot

Accepted Article

16512227, ja, Downloaded from https://onlinelibrary.wiley.com/doi/10.1111/apa.16660 by CSIRO Library Services Information Support, Wiley Online Library on [08/01/2023]. See the Terms and Conditions (https://onlinelibrary.wiley.com/terms-and-conditions) on Wiley Online Library for rules of use; OA articles are governed by the applicable Creative Commons License

be evaluated here. However, despite of not including eventual clinical interventions, our algorithm exhibited a high AUROC, comparable with other studies testing similar decision support systems exclusively in VLBW populations.(8, 10, 22, 29-31)

Thus, without the potential bias of a case control study and in a smaller cohort, the combination of several continuous vital signs and demographic data renders a risk score that could be implied in a clinical decision support system using already available monitoring and EHR data.

Our algorithm exhibits a high NPV (0.997-0.999) but overall a low PPV (0.009-0.022) for sepsis detection. This suggest that both our non-invasive semiautomatic algorithm and the manual RALIS system, that is based on retrospective EHR data analysis, are mainly useful to confirm the absence of sepsis rather than diagnosing the condition.(30) The low PPV is inherently due to the low prevalence of sepsis in the current cohort as well as the risk of other adverse events exhibiting similar patterns of vital sign alterations.(32) However, the high NPV, is in line with the current process in clinical practice limiting unnecessary antibiotic treatment that is associated with increased morbidity and mortality and instead wait and monitor.(33) The risk score might support faster stop of prophylactic antibiotic treatment. Reduction of antibiotic use is also of utmost importance to decelerate development of antimicrobial resistance, one of the global major health threats.

Implication for daily clinical care and future research

An earlier and more reliable diagnosis of sepsis in infants is important, enabling more individualized patient care that finally will save lives. Our findings add valuable information about characteristic changes of different vital sign patterns in the hours preceding clinical suspicion of sepsis in a representative NICU cohort. In particular, the success of our Naïve-Bayes model

Accepted Article

16512227, ja, Downloaded from https://onlinelibrary.wiley.com/doi/10.1111/apa.16660 by CSIRO Library Services Information Support, Wiley Online Library on [08/01/2023]. See the Terms and Conditions (https://onlinelibrary.wiley.com/terms-and-conditions) on Wiley Online Library for rules of use; OA articles are governed by the applicable Creative Commons License

shows that adding more individual patient information to algorithms for early sepsis detection improves their performance. Similarly, nSOFA has recently been reported to provide a longitudinal evaluation for risk of serious adverse events.(21)

In the present study, the predictability of sepsis was assessed retrospectively. To date only one large prospective randomized controlled trial assessing safety and usability of clinical decision support algorithms to improve early neonatal sepsis diagnosis in daily clinical practice is available.(7) The HRC-based, commercially available HeRO algorithm showed promising results when applied in VLBW infants from different US NICUs. However, when it was tested in a NICU following different treatment strategies and on a broader population, the results could not be reproduced.(34) We therefore suggest data sharing between different institutes for external validation of algorithm performance in different settings and populations.(5, 11, 35)

Strengths and limitations

This study included a smaller patient number when compared to some other studies assessing usability of vital sign characteristics to detect sepsis. This is of special importance given the low incidence of sepsis cases that might lead to underpowered and therefore falsely negative findings, given the sparse training ability for the used machine learning algorithm.(14, 36, 37) Clinical decision support systems have the potential to add supplementary information, especially to the culture-negative sepsis cases, and motivate withdrawal of antibiotics, if sepsis can be ruled out.

One of the strengths in the present study is the automated and continuous high frequency collection of vital sign data. This is less time consuming and less prone to data entry error, when compared to studies relying on manually collected vital signs.(8, 31, 38) In the present study, we used SpO_2 but did not adjust for applied respiratory support levels or administered oxygen

Accepted Article

16512227, ja, Downloaded from https://onlinelibrary.wiley.com/doi/10.1111/apa.16660 by CSIRO Library Services Information Support, Wiley Online Library on [08/01/2023]. See the Terms and Conditions (https://onlinelibrary.wiley.com/terms-and-conditions) on Wiley Online Library for rules of use; OA articles are governed by the applicable Creative Commons License

fraction. Other complications causing systemic inflammation might also lead to an increase in vital sign-based risk score and therefore alter algorithm evaluation. We speculate that including this information might lead to even better performance of the system and help to develop better clinical decision systems.

Notably, we here describe a representative NICU population during their daily clinical routine. This is in contrast to the majority of studies that highlight a specific subgroup in terms of gestational or postnatal age(6, 7, 21, 28) under well-defined, standardized research conditions(31) or with a case-control design that is far from being clinically applicable.(8, 29) Furthermore, we did not exclude infants that died during their NICU stay. This in contrast to some previous studies.(6, 30) We recognize that this is a population that potentially could benefit most from more individualized diagnosis, earlier therapeutic interventions and personalized care.

Our approach, being clinically relevant, however limits the number of positive cases and affects the distribution of the cases among the potential subgroups (VLBW, culture negative/culture positive). Thus, it renders a comparison of the subgroups unreliable in the current dataset. Notably, a clear majority of the infants experiencing sepsis were VLBW (16/20) which limits the training of the algorithm. Yet, the prognostic ability is shown also in the non-VLBW infants. Further investigation in larger cohorts, where subgroup analysis is possible is highly warranted.

Conclusions

The present machine learning-based algorithm, using combined high frequency vital signs and demographic factors, has a high predictive ability to detect sepsis in a cohort of representative NICU patients up to 24 hours before first clinical suspicion. Consensus on neonatal sepsis definition and prospective studies are needed to further fine-tune machine learning algorithms. Clinical decision support systems, using a combination of multiple features, could finally lead

Accepted Article

16512227, ja, Downloaded from https://onlinelibrary.wiley.com/doi/10.1111/apa.16660 by CSIRO Library Services Information Support, Wiley Online Library on [08/01/2023]. See the Terms and Conditions (https://onlinelibrary.wiley.com/terms-and-conditions) on Wiley Online Library for rules of use; OA articles are governed by the applicable Creative Commons License

to more individualized patient care, help improve healthcare resource allocation and reduce morbidity and mortality for all NICU infants.

Accepted Article

16512227, ja, Downloaded from https://onlinelibrary.wiley.com/doi/10.1111/apa.16660 by CSIRO Library Services Information Support, Wiley Online Library on [08/01/2023]. See the Terms and Conditions (https://onlinelibrary.wiley.com/terms-and-conditions) on Wiley Online Library for rules of use; OA articles are governed by the applicable Creative Commons License

Data Availability

The datasets generated and/or analyzed during the current study are available, in GDPR-compatible format, from the corresponding author on reasonable request.

Accepted Article

16512227, ja, Downloaded from https://onlinelibrary.wiley.com/doi/10.1111/apa.16660 by CSIRO Library Services Information Support, Wiley Online Library on [08/01/2023]. See the Terms and Conditions (https://onlinelibrary.wiley.com/terms-and-conditions) on Wiley Online Library for rules of use; OA articles are governed by the applicable Creative Commons License

References

1. Herlenius E, Kuhn P. Sudden unexpected postnatal collapse of newborn infants: a review of cases, definitions, risks, and preventive measures. *Transl Stroke Res* 2013; 4 2:236-47.
2. Fleischmann-Struzek C, Goldfarb DM, Schlattmann P, Schlapbach LJ, Reinhart K, Kissoon N. The global burden of paediatric and neonatal sepsis: a systematic review. *Lancet Respir Med* 2018; 6 3:223-30.
3. Hayes R, Hartnett J, Semova G, Murray C, Murphy K, Carroll L, et al. Neonatal sepsis definitions from randomised clinical trials. *Pediatr Res* 2021.
4. Molloy EJ, Bearer CF. Paediatric and neonatal sepsis and inflammation. *Pediatr Res* 2022; 91 2:267-9.
5. Molloy EJ, Wynn JL, Bliss J, Koenig JM, Keij FM, McGovern M, et al. Neonatal sepsis: need for consensus definition, collaboration and core outcomes. *Pediatr Res* 2020; 88 1:2-4.
6. Mithal LB, Yogev R, Palac H, Gur I, Mestan KK. Computerized vital signs analysis and late onset infections in extremely low gestational age infants. *J Perinat Med* 2016; 44 5:491-7.
7. Moorman JR, Carlo WA, Kattwinkel J, Schelonka RL, Porcelli PJ, Navarrete CT, et al. Mortality reduction by heart rate characteristic monitoring in very low birth weight neonates: a randomized trial. *J Pediatr* 2011; 159 6:900-6 e1.
8. Joshi R, Kommers D, Oosterwijk L, Feijs L, van Pul C, Andriessen P. Predicting Neonatal Sepsis Using Features of Heart Rate Variability, Respiratory Characteristics, and ECG-Derived Estimates of Infant Motion. *IEEE J Biomed Health Inform* 2020; 24 3:681-92.
9. Gur I, Markel G, Nave Y, Vainshtein I, Eisenkraft A, Riskin A. A mathematical algorithm for detection of late-onset sepsis in very-low birth weight infants: a preliminary diagnostic test evaluation. *Indian Pediatr* 2014; 51 8:647-50.
10. Sullivan BA, Wallman-Stokes A, Isler J, Sahni R, Moorman JR, Fairchild KD, et al. Early Pulse Oximetry Data Improves Prediction of Death and Adverse Outcomes in a Two-Center Cohort of Very Low Birth Weight Infants. *Am J Perinatol* 2018; 35 13:1331-8.
11. Persad E, Jost K, Honore A, Forsberg D, Coste K, Olsson H, et al. Neonatal sepsis prediction through clinical decision support algorithms: A systematic review. *Acta Paediatr* 2021; 110 12:3201-26.
12. Honoré A, Liu D, Forsberg D, Coste K, Herlenius E, Chatterje S, et al. Hidden Markov Models for Sepsis Detection in Preterm Infants. *IEEE International Conference on Acoustics, Speech and Signal Processing (ICASSP)* 2020; https://doi.org/10.1109/ICASSP40776.2020.9054635:2020:1130-4.
13. Honore A, Forsberg D, Jost K, Adolphson K, Stålhammar A, Herlenius E, et al. Classification and feature extraction for neonatal sepsis detection. *TechRxiv powered by IEEE* 2022; https://doi.org/10.36227/techrxiv.19290257.v1.
14. Griffin MP, O'Shea TM, Bissonette EA, Harrell FE, Jr., Lake DE, Moorman JR. Abnormal heart rate characteristics preceding neonatal sepsis and sepsis-like illness. *Pediatr Res* 2003; 53 6:920-6.
15. Bishop C. Pattern Recognition and Machine Learning. Singapore: *Springer*, 2006.
16. Iroh Tam PY, Bendel CM. Diagnostics for neonatal sepsis: current approaches and future directions. *Pediatr Res* 2017; 82 4:574-83.

Accepted Article

16512227, ja, Downloaded from https://onlinelibrary.wiley.com/doi/10.1111/apa.16660 by CSIRO Library Services Information Support, Wiley Online Library on [08/01/2023]. See the Terms and Conditions (https://onlinelibrary.wiley.com/terms-and-conditions) on Wiley Online Library for rules of use; OA articles are governed by the applicable Creative Commons License

17. Huber S, Hetzer B, Crazzolara R, Orth-Holler D. The correct blood volume for paediatric blood cultures: a conundrum? *Clin Microbiol Infect* 2020; 26 2:168-73.
18. Rivers E, Nguyen B, Havstad S, Ressler J, Muzzin A, Knoblich B, et al. Early goal-directed therapy in the treatment of severe sepsis and septic shock. *N Engl J Med* 2001; 345 19:1368-77.
19. Fairchild KD, O'Shea TM. Heart rate characteristics: physiomarkers for detection of late-onset neonatal sepsis. *Clin Perinatol* 2010; 37 3:581-98.
20. Griffin MP, Lake DE, Bissonette EA, Harrell FE, Jr., O'Shea TM, Moorman JR. Heart rate characteristics: novel physiomarkers to predict neonatal infection and death. *Pediatrics* 2005; 116 5:1070-4.
21. Lavilla OC, Aziz KB, Lure AC, Gipson D, de la Cruz D, Wynn JL. Hourly Kinetics of Critical Organ Dysfunction in Extremely Preterm Infants. *Am J Respir Crit Care Med* 2022; 205 1:75-87.
22. Fairchild KD, Schelonka RL, Kaufman DA, Carlo WA, Kattwinkel J, Porcelli PJ, et al. Septicemia mortality reduction in neonates in a heart rate characteristics monitoring trial. *Pediatr Res* 2013; 74 5:570-5.
23. Akobeng AK. Understanding diagnostic tests 2: likelihood ratios, pre- and post-test probabilities and their use in clinical practice. *Acta Paediatr* 2007; 96 4:487-91.
24. Siljehav V, Hofstetter AM, Leifsdottir K, Herlenius E. Prostaglandin E2 Mediates Cardiorespiratory Disturbances during Infection in Neonates. *J Pediatr* 2015; 167 6:1207-13 e3.
25. Hofstetter AO, Saha S, Siljehav V, Jakobsson PJ, Herlenius E. The induced prostaglandin E2 pathway is a key regulator of the respiratory response to infection and hypoxia in neonates. *Proc Natl Acad Sci U S A* 2007; 104 23:9894-9.
26. Das A, Shukla S, Rahman N, Gunzler D, Abughali N. Clinical Indicators of Late-Onset Sepsis Workup in Very Low-Birth-Weight Infants in the Neonatal Intensive Care Unit. *Am J Perinatol* 2016; 33 9:856-60.
27. Patel M, Mohr M, Lake D, Delos J, Moorman JR, Sinkin RA, et al. Clinical associations with immature breathing in preterm infants: part 2-periodic breathing. *Pediatr Res* 2016; 80 1:28-34.
28. Honore A, Siljehav V, Chatterje S, Herlenius E. Large Neural Network Based Detection of Apnea, Bradycardia and Desaturation Events. *Neural Information Processing System (NIPS), Workshop on Machine Learning for Health* 2017.
29. Cabrera-Quiros L, Kommers D, Wolvers MK, Oosterwijk L, Arents N, van der Sluijs-Bens J, et al. Prediction of Late-Onset Sepsis in Preterm Infants Using Monitoring Signals and Machine Learning. *Crit Care Explor* 2021; 3 1:e0302.
30. Mithal LB, Yogev R, Palac HL, Kaminsky D, Gur I, Mestan KK. Vital signs analysis algorithm detects inflammatory response in premature infants with late onset sepsis and necrotizing enterocolitis. *Early Hum Dev* 2018; 117:83-9.
31. Gur I, Riskin A, Markel G, Bader D, Nave Y, Barzilay B, et al. Pilot study of a new mathematical algorithm for early detection of late-onset sepsis in very low-birth-weight infants. *Am J Perinatol* 2015; 32 4:321-30.
32. Groves AM, Edwards AD. Heart rate characteristic monitoring-HeRO or villain? *J Pediatr* 2011; 159 6:885-6.
33. Ting JY, Synnes A, Roberts A, Deshpandey A, Dow K, Yoon EW, et al. Association Between Antibiotic Use and Neonatal Mortality and Morbidities in Very Low-Birth-Weight Infants Without Culture-Proven Sepsis or Necrotizing Enterocolitis. *JAMA Pediatr* 2016; 170 12:1181-7.

Accepted Article

16512227, ja, Downloaded from https://onlinelibrary.wiley.com/doi/10.1111/apa.16660 by CSIRO Library Services Information Support, Wiley Online Library on [08/01/2023]. See the Terms and Conditions (https://onlinelibrary.wiley.com/terms-and-conditions) on Wiley Online Library for rules of use; OA articles are governed by the applicable Creative Commons License

34. Coggins SA, Weitkamp JH, Grunwald L, Stark AR, Reese J, Walsh W, et al. Heart rate characteristic index monitoring for bloodstream infection in an NICU: a 3-year experience. *Arch Dis Child Fetal Neonatal Ed* 2016; 101 4:F329-32.
35. Loftus T, Tinghe P, Ozragat-Baslanti T, Davis J, Ruppert M, Ren Y, et al. Ideal algorithms in healthcare: Explainable, dynamic, precise, autonomous, fair, and reproducible. *PLOS Digital Health* 2022; 1.
36. Masino AJ, Harris MC, Forsyth D, Ostapenko S, Srinivasan L, Bonafide CP, et al. Machine learning models for early sepsis recognition in the neonatal intensive care unit using readily available electronic health record data. *PLoS One* 2019; 14 2:e0212665.
37. Zimmet AM, Sullivan BA, Moorman JR, Lake DE, Ratcliffe SJ. Trajectories of the heart rate characteristics index, a physiomarker of sepsis in premature infants, predict Neonatal ICU mortality. *JRSM Cardiovasc Dis* 2020; 9:2048004020945142.
38. Nguyen N, Vandenbroucke L, Hernandez A, Pham T, Beuchee A, Pladys P. Early-onset neonatal sepsis is associated with a high heart rate during automatically selected stationary periods. *Acta Paediatr* 2017; 106 5:749-54.
39. Ng A, Jordan M. On Discriminative vs. Generative Classifiers: A comparison of logistic regression and naive Bayes. Advances in Neural Information Processing Systems (NIPS)2002

Accepted Article

16512227, ja, Downloaded from https://onlinelibrary.wiley.com/doi/10.1111/apa.16660 by CSIRO Library Services Information Support, Wiley Online Library on [08/01/2023]. See the Terms and Conditions (https://onlinelibrary.wiley.com/terms-and-conditions) on Wiley Online Library for rules of use; OA articles are governed by the applicable Creative Commons License

Competing interests The authors have no conflicts of interest relevant to this article to disclose.

Accepted Article

16512227, ja, Downloaded from https://onlinelibrary.wiley.com/doi/10.1111/apa.16660 by CSIRO Library Services Information Support, Wiley Online Library on [08/01/2023]. See the Terms and Conditions (https://onlinelibrary.wiley.com/terms-and-conditions) on Wiley Online Library for rules of use; OA articles are governed by the applicable Creative Commons License

Figure Legends

Figure 1. Machine learning-based risk score increases prior to clinical sepsis suspicion.

Hours prior to clinical suspicion of sepsis (time of blood culture sampling indicated by red line) the vital sign-based risk score increased. For the whole population, the third quartile of the risk scores aggregated in 4 hours bins peaks around 6 hours prior to clinical suspicion, and starts to rise from 10 hours (A). Density plot (B), depict risk score intervals of 4 hours on the y-axis, time until sepsis on the x-axis and the density, i.e. number of data points, is graded from blue, few data points, to red, high amount of data points. Here the amount of high-risk time frames increases from 20 hours prior to clinical suspicion in patients with sepsis (B). Simultaneously the proportion of low-risk frames (bottom row) decreases, indicating the increasing probability of being categorized as sepsis by the vital sign analysis within the hours leading up to sepsis. Additionally, the risk increase is rapid, as the proportion of intermediate risk frames (middle portion of y-axis) is continuously low. An example of a single patient with sepsis shows that the risk score could alert for sepsis up to 24 hours earlier than current clinical praxis (C). Dots correspond to the predictions performed on individual time frames. The line corresponds to a smoothing of these individual predictions with a median filter of length 4h. Underneath, the raw trace of heart rate (BtB HF), oxygen saturation (SpO_2) and respiratory rate (RF) shows what is displayed at the bedside monitor. No changes, apart from this patient being intubated ~10 hours after sepsis, decreasing heart rate variability, could be identified through visual in-spection.

Figure 2. Combining multiple features improves algorithm performances.

Area under median receiver operating characteristics (AUROC) curves exhibit good algorithm performances and individualized data analysis through inclusion of demographic data birth

Accepted Article

16512227, ja, Downloaded from https://onlinelibrary.wiley.com/doi/10.1111/apa.16660 by CSIRO Library Services Information Support, Wiley Online Library on [08/01/2023]. See the Terms and Conditions (https://onlinelibrary.wiley.com/terms-and-conditions) on Wiley Online Library for rules of use; OA articles are governed by the applicable Creative Commons License

weight, sex, and postnatal age (PNA) improves overall performance (A). Combining heart rate-based features with respiration-based features further improved performance (B-C). Utilizing the algorithm analysis improved the probability of correctly classifying frames as sepsis (positive likelihood ratio, blue line) or non-sepsis (negative likelihood ratio, red line) depicted in a Fagan's Normogram (D).

The violin plots display the distribution of the AUROC/likelihood scores over 24 cross validation runs. The boxes show the median and interquartile range. The violin shape shows the empirical distribution. Feature comparison (HRC vs HRC combined with respiratory (resp.) features) evaluated including demographic features. *p<0.05; LR(+); positive likelihood ration, LR(-) negative likelihood ratio.

Figure 3. Sepsis prediction in VLBW infants.

In the very low birth weight (VLBW) subpopulation, the vital sign-based risk (aggregated third quartile) increases from 40 hours prior to sepsis (A, indicated by red line). This is also recognized in the density plot, where the proportion of high-risk frames increases from 24 hours prior to clinical suspicion (B). Adding respiratory features to cardiac-based features (HRC) improved the ability to detect sepsis in the VLBW subgroup compared to HRC alone (C-E). Utilizing the algorithm improved the probability of correctly classifying frames as sepsis (positive likelihood ratio, blue line) or non-sepsis (negative likelihood ratio, red line) depicted in a Fagan's Normogram (E). LR(+); positive likelihood ration, LR(-) negative likelihood ratio.

Accepted Article
16512227, ja, Downloaded from https://onlinelibrary.wiley.com/doi/10.1111/apa.16660 by CSIRO Library Services Information Support, Wiley Online Library on [08/01/2023]. See the Terms and Conditions (https://onlinelibrary.wiley.com/terms-and-conditions) on Wiley Online Library for rules of use; OA articles are governed by the applicable Creative Commons License

Accepted Article

Funding/Support

This study was supported by grants to E.H. from the Swedish Research Council (2019-01157), the Stockholm County Council (2019-0400, 2019-0974 and FoUI-966449), the Swedish Brain (FO2019-0087 and FO2021-0313), the Swedish National Heart and Lung (20180505 and 20210579), the Axel Tielmans, and the Freemasons Children's House foundations. K.J. was supported by a Postdoc Mobility Fellowship from the Swiss National Science Foundation (P400PM_194474). The funders did not participate in the design or conduct of the study.

Acknowledgements

We would like to thank K. Coste, L. van Breugel, E. and H. Olsson for their initial help in building up the study database and data annotation.

Consent statement: Patient consent was waived in May 2020 for both future patients and those previously discharged.

Author Contributions

Antoine Honoré did substantially contribute to conception and design, acquisition, analysis, and interpretation of data. He did draft the article and gave final approval of the version to be published.

Dr. David Forsberg did substantially contribute to conception and design, acquisition, analysis, and interpretation of data. He did draft and revise the article critically for important intellectual content and gave final approval of the version to be published.

Dr. Katja Adolphson did substantially contribute to acquisition, and interpretation of data. She did draft the article and gave final approval of the version to be published.

16512227, ja, Downloaded from https://onlinelibrary.wiley.com/doi/10.1111/apa.16660 by CSIRO Library Services Information Support, Wiley Online Library on [08/01/2023]. See the Terms and Conditions (https://onlinelibrary.wiley.com/terms-and-conditions) on Wiley Online Library for rules of use; OA articles are governed by the applicable Creative Commons License

Dr. Saikat Chatterjee did revise the article critically for important intellectual content and gave final approval of the version to be published.

Dr. Kerstin Jost did substantially contribute to the analysis, and interpretation of data. She did revise the article critically for important intellectual content and gave final approval of the version to be published.

Prof. Eric Herlenius did substantially contribute to conception and design, acquisition, analysis, and interpretation of data. He did revise the article critically for important intellectual content and gave final approval of the version to be published.

Conflict of interest

The authors declare that the research was conducted in the absence of any commercial or financial relationships that could be construed as a potential conflict of interest.

Accepted Article

16512227, ja, Downloaded from https://onlinelibrary.wiley.com/doi/10.1111/apa.16660 by CSIRO Library Services Information Support, Wiley Online Library on [08/01/2023]. See the Terms and Conditions (https://onlinelibrary.wiley.com/terms-and-conditions) on Wiley Online Library for rules of use; OA articles are governed by the applicable Creative Commons License

Accepted Article

16512227, ja, Downloaded from https://onlinelibrary.wiley.com/doi/10.1111/apa.16660 by CSIRO Library Services Information Support, Wiley Online Library on [08/01/2023]. See the Terms and Conditions (https://onlinelibrary.wiley.com/terms-and-conditions) on Wiley Online Library for rules of use; OA articles are governed by the applicable Creative Commons License

Table 1. Demographics of included infants used for algorithm development and analysis, the very low birth weight subgroup and the whole population

		**Study cohort (n=325)**	**VLBW (n=93)**	**Population (n=489)**
**Gestational age (w)**		34+6 (4+4)*<br>35+6 (10+1)	27+6 (3+0)*<br>27+5 (3+6)	35+2 (5+2)*<br>36+6 (8+0)
	Full term	153 (47 %)	3 (3 %)*	234 (48 %)
	Moderately preterm	67 (21 %)	4 (4 %)*	126 (26 %)
	Very preterm	57 (17 %)	38 (41 %)*	71 (14 %)
	Extremely preterm	48 (15 %)	48 (52 %)*	58 (12 %)*
**Birth weight (g)**		2468 (1215)*<br>2524 (2183)	974 (287)*<br>994 (417)	2555 (1138)*<br>2732 (1913)
**Sex male/ female**		176 (54 %)/149 (46 %)	42 (45 %)/51 (55 %)	270 (55 %)/219 (45 %)
**Delivery mode**	Partus normalis	151 (47 %)	26 (28 %)*	251 (51 %)*
	Acute cesarean section	144 (44 %)	62 (67 %)*	179 (37 %)
	Elective cesarean section	28 (9 %)	5 (5 %)*	55 (12 %)
**Hospitalization days**		2866	1847	8311
**Sepsis cases**		20	16	31

Gestational age and birth weight are presented as mean (standard deviation) (above) and median (interquartile range) (below) and all others as n (% of group). Explanation: extremely preterm <28 w of gestation, LBW: low birth weight (1500-2000 g), moderately preterm 33-37 w of gestation, very preterm 28-32 w of gestation, VLBW: very low birth weight (<1500 g), w: weeks. *significant (p<0.05) intercolumn difference.

Accepted Article

Appendix S1

Supplemental information regarding data analysis

In parallel with this study, we have investigated the technical aspects and based on those results we have selected the approach used in the present study. Here follows a summary of the comparisons made. For results and discussion please see the preprint available at ORCID: 0000 -0003 -0166 -1356 (Honore, A. et al. Classification and Feature Extraction for Neonatal Sepsis Detection. TechRxiv powered by IEEE; 2022). Two main aspects have been evaluated; algorithm classes and time factors in the data.

Algorithm classes

Five algorithm classes have been compared: logistic regression (LR), Extreme learning machines (ELM), Gaussian Mixture model (GMM; full covariance GMM and Diagonal GMM), Multilayer Perceptron (MLP), Naïve Bayes (NB) and Normalizing flow (NF) models. Discriminative training was used for LR, ELM and MLP while NB, GMM and NF are generative model -based methods and maximum -likelihood training was utilized. Discriminative training was evaluated for NF as well.

Time Factors

For the multiple algorithm classes, we evaluated different time window lengths, ranging from 5 minutes to 155 minutes. During the evaluation the sampling period interval was fixed to 1 sec. For selection of the optimal time period, in the present study selected as 45 minutes, the results from the evaluation were considered together with clinical relevance and expected practical usefulness. Too short time window lengths could potentially limit real -time analysis while too long time window lengths could overshoot clinical events. To find the optimal conditions

16512227, ja, Downloaded from https://onlinelibrary.wiley.com/doi/10.1111/apa.16660 by CSIRO Library Services Information Support, Wiley Online Library on [08/01/2023]. See the Terms and Conditions (https://onlinelibrary.wiley.com/terms-and-conditions) on Wiley Online Library for rules of use; OA articles are governed by the applicable Creative Commons License

we also evaluated different sampling period intervals, from 1 second to 60 seconds, and assessed the multiple combinations between time window lengths and sampling period intervals. Finally, we weighted multiple features and ranked them hierarchically to identify the most prominent.

Based on this parallel evaluation, we selected the Naïve Bayes model using a time window length of 45 minutes and a sampling period of 1 second. We included minimum, maximum, mean, standard deviation (SD), skewness, and kurtosis features as well as heart rate characteristics in the present study.

In the present manuscript, we used a Naïve-Bayes generative machine learning algorithm to classify time frames as either "sepsis" or "non-sepsis". Generative classifiers reach their maximum performance with a limited amount of training data. This is in contrast with discriminative classifiers, such as logistic regression, which reach a higher performance when more training data is available.(39) Moreover, generative algorithms such as Naïve-Bayes or other Gaussian Mixture-based classifiers take the class imbalance explicitly into account in the statistical model, whereas the class imbalance is modeled indirectly in the training procedure of discriminative classifiers.(15) This makes generative classifiers more suited for neonatal sepsis detection tasks, where both the number of similar sepsis cases is small and the prevalence of sepsis among the frames is low.

Algorithm design and training

The repeated measures of vital signs features on consecutive time frames are assumed independent. Our detection performances are thus valid, regardless of the monitoring history of a patient. This is useful since collecting long periods of uninterrupted monitoring data is challenging in NICUs, in particular when patients are transferred or undergo surgeries. Moreover,

Accepted Article

16512227, ja, Downloaded from https://onlinelibrary.wiley.com/doi/10.1111/apa.16660 by CSIRO Library Services Information Support, Wiley Online Library on [08/01/2023]. See the Terms and Conditions (https://onlinelibrary.wiley.com/terms-and-conditions) on Wiley Online Library for rules of use; OA articles are governed by the applicable Creative Commons License

taking into account time correlation increases the number of parameters to learn. The models were trained with the objective to give high probabilities to the training time frames of their respective class.

Performance scores were calculated on posterior probabilities calibrated with isotonic regression. Time frames were assigned label “1”, when the calibrated posterior probability of class “1” was above a fixed threshold. The threshold was chosen to maximize the specificity/sensitivity performances on the training set. Since the calibration step redistributes the posterior probabilities, the threshold adaptation should not impact the classification results as much as without calibration, and thus not lead to overfitting.

The calibration step changes the baseline of the posterior probability depending on the distribution of the dependent variable (the label) in the training data set. To enable comparison across patients and across multiple training/validation splits, the individual risk versus time figures are plotted without calibration.

To evaluate algorithm performance (posterior probability assessment) we modeled the dependencies between the features extracted for each time frame and label of the time frame (denoted from the clinical event timeline). Thus, a prediction of sepsis/no sepsis is possible for every new 45 minutes time frame. This classification process is learned through training on a training dataset and then validated on a validation data set. The data was split into a training and validation data set on a patient level. As the patient cohort is heterogeneous and limited in size, we did not split beyond the patient level to perform a cross-validation. Instead, we performed 20 random splits into training/validation (70%/30%) at the patient level to better evaluate the algorithm performance. Training features were normalized so that each feature has zero mean and unit variance. The normalizing coefficients of the training set was used for the validation set.

Accepted Article

16512227, ja, Downloaded from https://onlinelibrary.wiley.com/doi/10.1111/apa.16660 by CSIRO Library Services Information Support, Wiley Online Library on [08/01/2023]. See the Terms and Conditions (https://onlinelibrary.wiley.com/terms-and-conditions) on Wiley Online Library for rules of use; OA articles are governed by the applicable Creative Commons License

LOS					
20					
**>1500g**		**<1500g**			
2		18			
S. Aureus	*Culture negative*	*S. Aureus*	*Enterobacteriales*	*CONS*	*Culture negative*
1	1	4	1	8	5

Table S1. Neonatal Sepsis patients with LOS: Late onset sepsis, CONS: Coagulase -negative staphylococcus, S. Aureus: Staphylococcus Aureus, culture negative: blood culture negative.

Accepted Article

16512227, ja, Downloaded from https://onlinelibrary.wiley.com/doi/10.1111/apa.16660 by CSIRO Library Services Information Support, Wiley Online Library on [08/01/2023]. See the Terms and Conditions (https://onlinelibrary.wiley.com/terms-and-conditions) on Wiley Online Library for rules of use; OA articles are governed by the applicable Creative Commons License

Accepted Article

	Features	Demographics		AUROC	Specificity	Sensitivity	PPV	NPV
All population	HRC	PNA	BW & Sex	0.72 (0.65-0.83)	0.66 (0.54-0.70)	**0.89** (0.83-0.92)	0.010 (0.008-0.015)	0.999 (0.999-0.999)
			None	0.76 (0.70-0.81)	0.66 (0.69-0.72)	0.83 (0.77-0.89)	0.010 (0.007-0.017)	0.999 (0.999-0.999)
		no PNA	BW & Sex	0.69 (0.61-0.77)	0.61 (0.53-0.66)	0.82 (0.73-0.85)	0.009 (0.007-0.014)	0.998 (0.997-0.999)
			None	0.69 (0.69-0.73)	0.62 (0.56-0.70)	0.72 (0.62-0.78)	0.009 (0.006-0.014)	0.998 (0.997-0.999)
	HRC + Resp	PNA	BW & Sex	**0.81** (0.70-0.88)	0.72 (0.64-0.78)	0.81 (0.60-0.88)	0.015 (0.009-0.02)	0.997 (0.999-0.999)
			None	0.81 (0.67-0.83)	0.76 (0.69-0.83)	0.73 (0.57-0.80)	0.015 (0.009-0.022)	0.998 (0.997-0.999)
		no PNA	BW & Sex	0.76 (0.64-0.85)	**0.77** (0.61-0.83)	0.67 (0.53-0.79)	0.014 (0.01-0.023)	0.998 (0.997-0.999)
			None	0.76 (0.60-0.82)	0.74 (0.65-0.80)	0.73 (0.544-0.80)	0.013 (0.007-0.018)	0.998 (0.996-0.999)
Birth weight < 1500g	HRC	PNA	BW & Sex	0.80 (0.71-0.86)	0.71 (0.66-0.79	**0.88** (0.71-0.92)	0.020 (0.011-0.031)	0.999 (0.999-0.999)
			None	0.74 (0.69-0.80)	0.66 (0.57-0.74)	0.82 (0.71-0.86)	0.012 (0.01-0.017)	0.998 (0.997-0.999)
		no PNA	BW & Sex	0.70 (0.61-0.80)	0.70 (0.60-0.77)	0.74 (0.58-0.79)	0.016 (0.011-0.019)	0.997 (0.996-0.998)
			None	0.66 (0.60-0.73)	0.61 (0.55-0.71)	0.70 (0.63-0.75)	0.010 (0.007-0.016)	0.997 (0.996-0.998)
	HRC + Resp	PNA	BW & Sex	**0.82** (0.59-0.87)	**0.72** (0.63-0.81)	0.83 (0.60-0.88)	0.022 (0.005-0.03)	0.999 (0.996-0.999)
			None	0.80 (0.62-0.87)	0.71 (0.59-0.77)	0.80 (0.60-0.85)	0.020 (0.004-0.032)	0.998 (0.996-0.999)
		no PNA	BW & Sex	0.77 (0.52-0.83)	0.72 (0.61-0.79)	0.74 (0.60-0.76)	0.018 (0.005-0.026)	0.998 (0.995-0.999)
			None	0.74 (0.55-0.81)	0.72 (0.62-0.76)	0.74 (0.60-0.81)	0.016 (0.004-0.024)	0.997 (0.995-0.999)

16512227, ja, Downloaded from https://onlinelibrary.wiley.com/doi/10.1111/apa.16660 by CSIRO Library Services Information Support, Wiley Online Library on [08/01/2023]. See the Terms and Conditions (https://onlinelibrary.wiley.com/terms-and-conditions) on Wiley Online Library for rules of use; OA articles are governed by the applicable Creative Commons License

Accepted Article

Table S2. Overview for different models applied to overall cohort and very low birth weight subgroup for sepsis detection 24 hours prior to clinical suspicion. Feature set 1; HRC (heart rate characteristics), Feature set 2; HRC combined with respiratory rate and oxygen saturation characteristics (Resp). Values displayed as median (interquartile range (IQR)). Abbreviations: AUROC: area under receiver operating characteristics, BW: birth weight, HRC: heart rate characteristics, NPV: negative predictive value, PNA: Post Natal Age, PPV: positive predictive value, Resp: respiratory rate and O2 saturation characteristics

16512227, ja, Downloaded from https://onlinelibrary.wiley.com/doi/10.1111/apa.16660 by CSIRO Library Services Information Support, Wiley Online Library on [08/01/2023]. See the Terms and Conditions (https://onlinelibrary.wiley.com/terms-and-conditions) on Wiley Online Library for rules of use; OA articles are governed by the applicable Creative Commons License

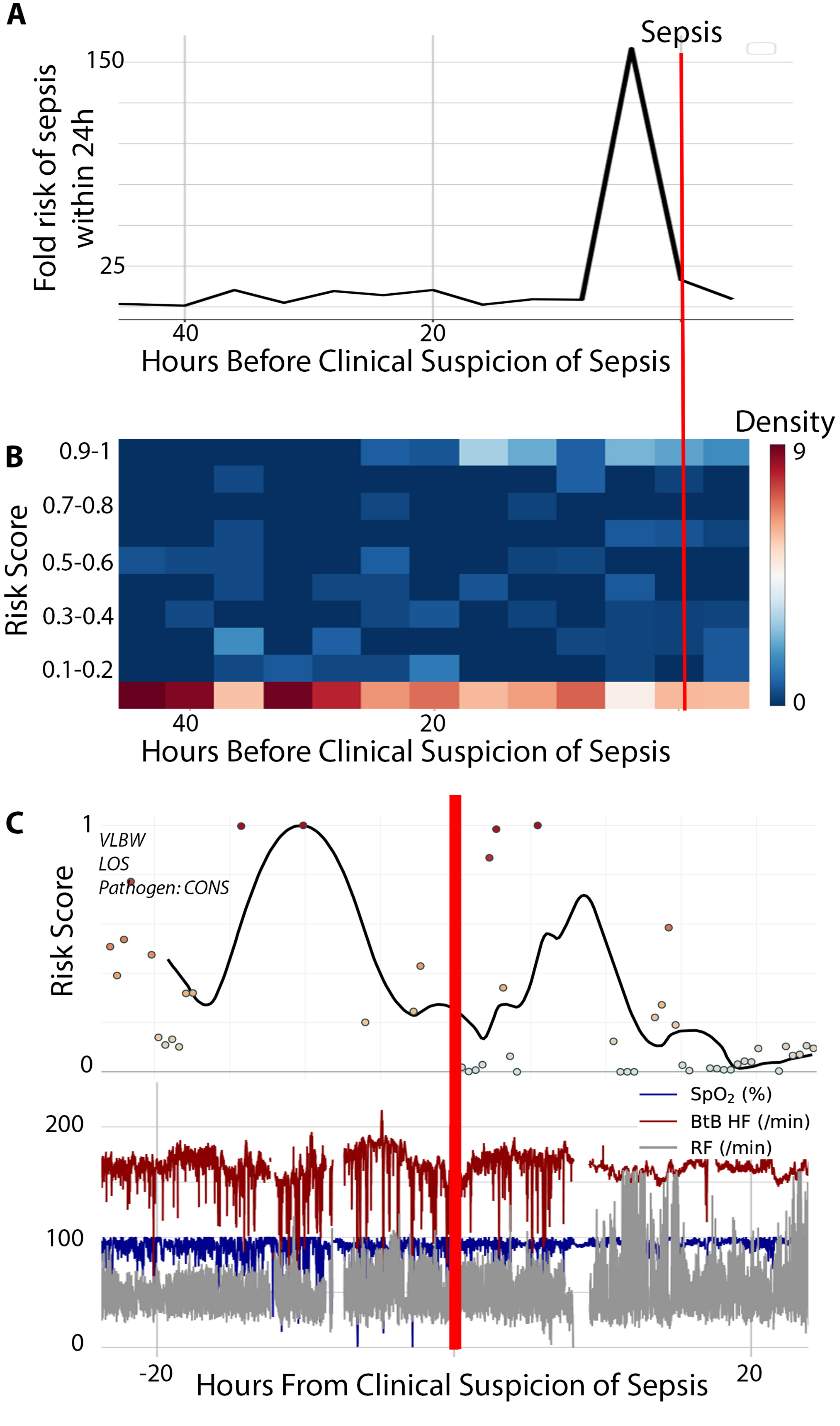

16512227, ja, Downloaded from https://onlinelibrary.wiley.com/doi/10.1111/apa.16660 by CSIRO Library Services Information Support, Wiley Online Library on [08/01/2023]. See the Terms and Conditions (https://onlinelibrary.wiley.com/terms-and-conditions) on Wiley Online Library for rules of use; OA articles are governed by the applicable Creative Commons License

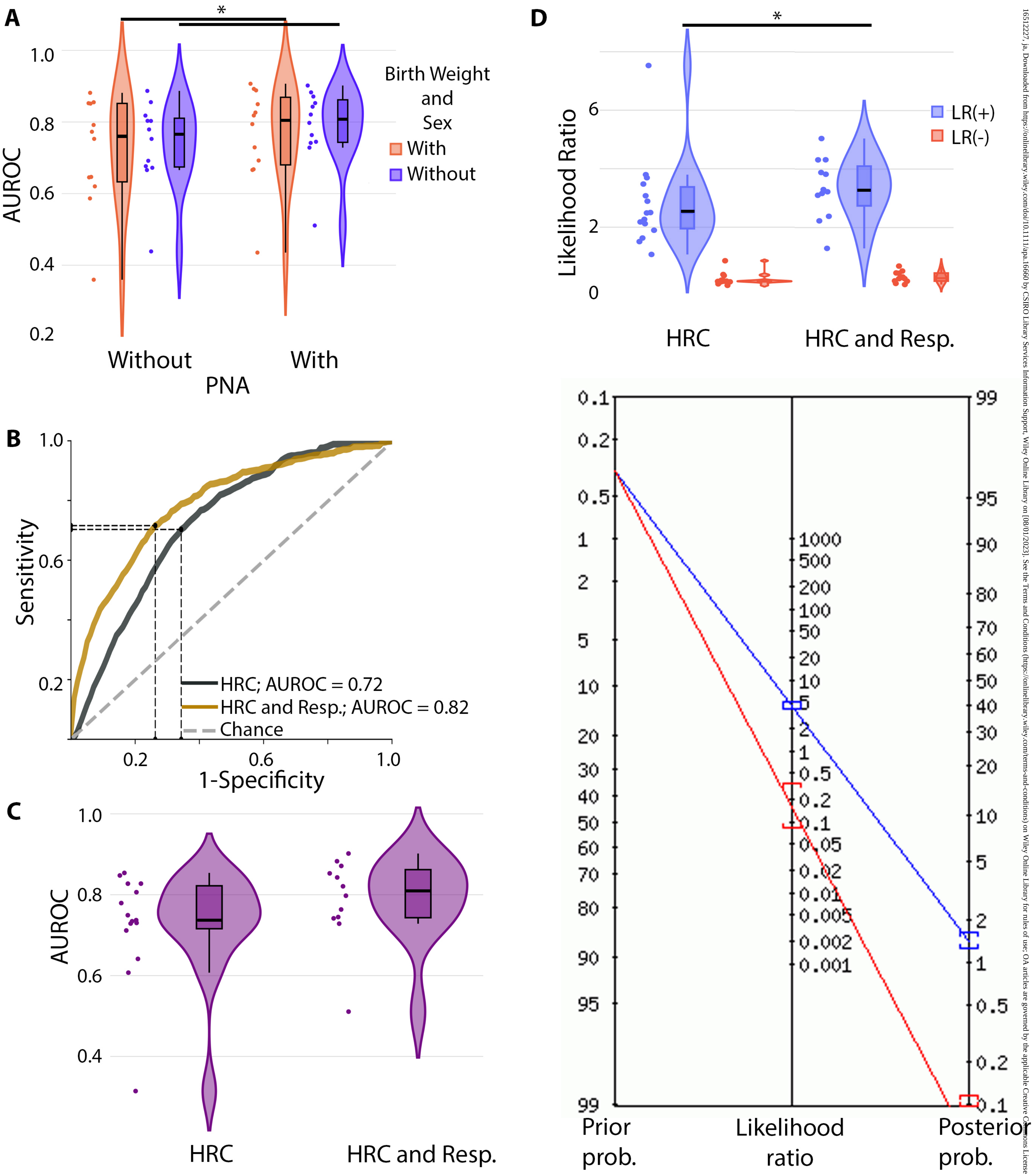

16512227, ja, Downloaded from https://onlinelibrary.wiley.com/doi/10.1111/apa.16660 by CSIRO Library Services Information Support, Wiley Online Library on [08/01/2023]. See the Terms and Conditions (https://onlinelibrary.wiley.com/terms-and-conditions) on Wiley Online Library for rules of use; OA articles are governed by the applicable Creative Commons License

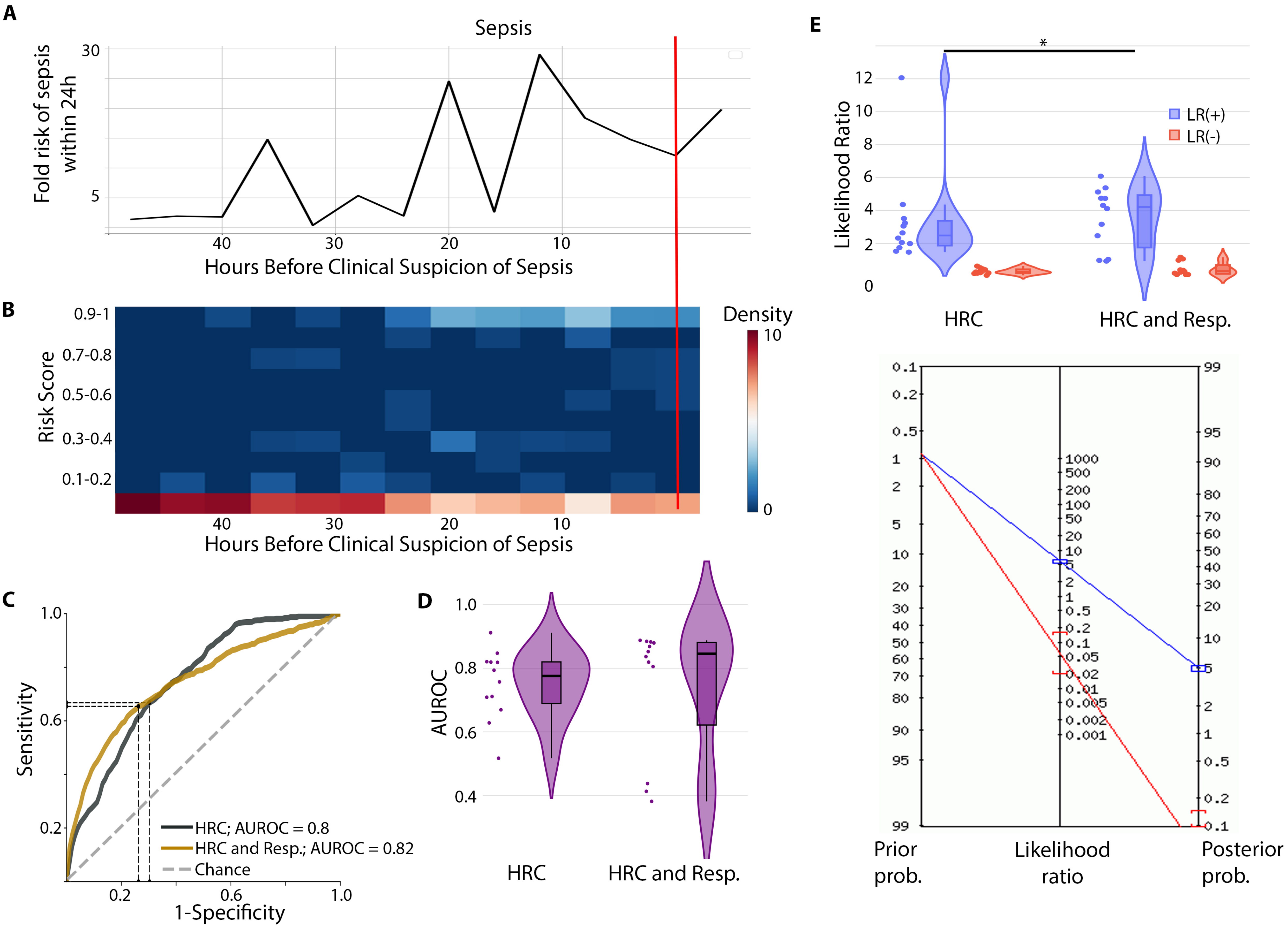

16512227, ja, Downloaded from https://onlinelibrary.wiley.com/doi/10.1111/apa.16660 by CSIRO Library Services Information Support, Wiley Online Library on [08/01/2023]. See the Terms and Conditions (https://onlinelibrary.wiley.com/terms-and-conditions) on Wiley Online Library for rules of use; OA articles are governed by the applicable Creative Commons License
