# Supplementary material for: Exploring Computational Techniques in Preprocessing Neonatal Physiological Signals for Detecting Adverse Outcomes: Scoping Review
Source: Interact J Med Res. 2024 Aug 20;13:e46946. doi: 10.2196/46946 (PMC11372324; doi:10.2196/46946)
Supplement: Multimedia Appendix 3 [file ijmr_v13i1e46946_app3.zip › Included Papers - Final/4389/Kausch et al. - 2023 - Cardiorespiratory signature of neonatal sepsis de.pdf]

www.nature.com/pr

CLINICAL RESEARCH ARTICLE

Cardiorespiratory signature of neonatal sepsis: development and validation of prediction models in 3 NICUs

Sherry L. Kausch[1]✉, Jackson G. Brandberg[2], Jiaxing Qiu[2], Aneesha Panda[3], Alexandra Binai[3], Joseph Isler[4], Rakesh Sahni[4], Zachary A. Vesoulis[3], J. Randall Moorman[2], Karen D. Fairchild[1], Douglas E. Lake[2] and Brynne A. Sullivan[1]

© The Author(s), under exclusive licence to the International Pediatric Research Foundation, Inc 2022

BACKGROUND: Heart rate characteristics aid early detection of late-onset sepsis (LOS), but respiratory data contain additional signatures of illness due to infection. Predictive models using cardiorespiratory data may improve early sepsis detection. We hypothesized that heart rate (HR) and oxygenation (SpO_2) data contain signatures that improve sepsis risk prediction over HR or demographics alone.
METHODS: We analyzed cardiorespiratory data from very low birth weight (VLBW, <1500 g) infants admitted to three NICUs. We developed and externally validated four machine learning models to predict LOS using features calculated every 10 m: mean, standard deviation, skewness, kurtosis of HR and SpO_2, and cross-correlation. We compared feature importance, discrimination, calibration, and dynamic prediction across models and cohorts. We built models of demographics and HR or SpO_2 features alone for comparison with HR-SpO_2 models.
RESULTS: Performance, feature importance, and calibration were similar among modeling methods. All models had favorable external validation performance. The HR-SpO_2 model performed better than models using either HR or SpO_2 alone. Demographics improved the discrimination of all physiologic data models but dampened dynamic performance.
CONCLUSIONS: Cardiorespiratory signatures detect LOS in VLBW infants at 3 NICUs. Demographics risk-stratify, but predictive modeling with both HR and SpO_2 features provides the best dynamic risk prediction.

Pediatric Research; https://doi.org/10.1038/s41390-022-02444-7

IMPACT:

- Heart rate characteristics aid early detection of late-onset sepsis, but respiratory data contain signatures of illness due to infection.
- Predictive models using both heart rate and respiratory data may improve early sepsis detection.
- A cardiorespiratory early warning score, analyzing heart rate from electrocardiogram or pulse oximetry with SpO_2, predicts late-onset sepsis within 24 h across multiple NICUs and detects sepsis better than heart rate characteristics or demographics alone.
- Demographics risk-stratify, but predictive modeling with both HR and SpO_2 features provides the best dynamic risk prediction.
- The results increase understanding of physiologic signatures of neonatal sepsis.

INTRODUCTION

Early detection of late-onset sepsis (LOS, sepsis beyond 3 days of age) reduces mortality and improves outcomes for survivors.[1,2] Although many infants with LOS exhibit clinical instability, signs and symptoms are nonspecific and occur late in the course of illness. The challenge with subtle and overlapping clinical signs of sepsis lies in the balance between early appropriate treatment and over treatment with unnecessary antibiotics. Early, prolonged antibiotic exposure disrupts the developing microbiome, which has been associated with adverse short and long-term outcomes for premature infants.[3–6] Late recognition of sepsis, when overt clinical signs represent organ dysfunction due to systemic inflammation, can lead to fatal or long-lasting organ damage. Therefore, precision medicine tools using predictive monitoring aim to address this challenge by guiding clinical decisions to right-time antibiotics.[7]

Signatures of illness are present in physiologic time series data derived from heart rate (HR) and oxygen saturation (SpO_2) monitoring in the early stages of sepsis in premature infants.[8–10] We developed and validated algorithms to detect abnormal patterns in continuous HR[9] and SpO_2[11] data and in their cross-correlation.[8] The heart rate characteristics (HRC) index estimates the risk of imminent sepsis using the standard deviation of RR intervals, sample asymmetry,[12] and sample entropy[13] to detect decreased HR variability with transient decelerations.[9,14] A multicenter randomized clinical trial showed HRC display reduced mortality for premature infants.[1,15]

[1]Department of Pediatrics, University of Virginia School of Medicine, Charlottesville, VA, USA. [2]Department of Medicine, University of Virginia School of Medicine, Charlottesville, VA, USA. [3]Department of Pediatrics, Washington University in St. Louis School of Medicine, St. Louis, MO, USA. [4]Department of Pediatrics, Columbia University Vagelos College of Physicians and Surgeons, New York, NY, USA. ✉email: slk7s@uvahealth.org

Received: 17 August 2022 Revised: 21 November 2022 Accepted: 12 December 2022
Published online: 02 January 2023

We have found additional physiological signatures of neonatal illness and explored new analyses and modeling methods.[8,10,11] Respiratory deterioration prompts many sepsis evaluations[10] in premature infants, and apnea increases during sepsis[16] as inflammation affects central control of breathing.[17] Thus, we hypothesize that abnormal patterns in pulse oximetry might add to HR characteristics in early detection of sepsis. While studies in small cohorts have explored this hypothesis,[18–20] none have analyzed the added value of continuous SpO_2 data for sepsis prediction in large data sets with external validation. Here, we used data from three tertiary NICUs to develop and validate statistical models combining HR and SpO_2 analytics for sepsis detection in very low birth weight (VLBW, <1500 g) infants. We refer to models of this kind as POWS (Pulse Oximetry Warning System). As this acronym implies, we hypothesized that model performance would not change when using heart rate derived from pulse oximetry (pulse rate) instead of electrocardiogram monitoring, which would allow the algorithm to run on data from a single device. We aimed to evaluate multiple cardiorespiratory features, modeling methods, and performance metrics to test the hypothesis that respiratory data contain signatures of illness caused by sepsis and add information to HR characteristics and demographic variables for risk prediction.

METHODS

Patients

We studied VLBW infants admitted to 3 NICUs: University of Virginia Children's Hospital (NICU 1, 2012-2021), Morgan Stanley Children's Hospital of New York, Columbia University (NICU 2, 2012–2019), and St. Louis Children's Hospital, Washington University School of Medicine (NICU 3, 2016-2021). We report the results of this study in accordance with the Transparent Reporting of a multivariable prediction model for Individual Prognosis Or Diagnosis (TRIPOD) guidelines.[21,22]

The study was approved by the Institutional Review Board at each center (University of Virginia, Columbia University, and Washington University) with waiver of informed consent. We excluded infants with major chromosomal or congenital anomalies and those with no vital sign data collected. We collected demographic and clinical variables from the electronic health record and unit databases.

Sepsis definition

Late-onset sepsis (LOS) is the primary outcome of our modeling. Clinicians at each site reviewed blood cultures obtained after three days of age for infants who met inclusion criteria. We recorded the event as LOS if the blood culture was positive, the infant was treated with at least five days of antibiotics, and the culture was preceded by at least two days with no antibiotics. We excluded negative blood cultures, positive blood cultures obtained within seven days of a prior positive blood culture, and positive blood cultures treated as contaminants (defined as <5 days of antibiotics).

HR and SpO_2 data collection and preprocessing

Continuous HR and SpO_2 data were collected from standard NICU bedside monitors (GE, Philips) using the BedMaster system (Hillrom's Medical Device Integration Solution, Chicago, IL). Electrocardiogram-derived HR and pulse oximeter-derived SpO_2 and pulse rate were collected at 0.5 Hz at NICU 1 and 2. NICU 3 collected data at 1 Hz and was down-sampled to 0.5 Hz to match the other sites. SpO_2 was measured with the default averaging time of 8 s Masimo at NICU 1 and 2 and Nellcor Oximax at NICU 3. HR and SpO_2 underwent single-step preprocessing where values representing incontrovertible artifact (zeros) were removed.

Candidate predictors: HR, SpO_2, demographics

In 10-min non-overlapping windows, we calculated the mean, standard deviation (SD), skewness, kurtosis of both HR and SpO_2, and their minimum and maximum cross-correlation.[8,10] We examined the empirical univariate risk of each feature to evaluate individual predictor-risk relationships. We noticed differences in distributions of mean HR between the three sites. Additionally, prior work suggests mean HR does not predict imminent LOS.[23] Therefore, we excluded mean HR as a candidate predictor. Furthermore, including mean HR reduced performance across sites.

Model development

Figure 1 provides a schematic overview of the methods, including model development, validation, comparisons, and secondary analyses. Every 10-min window of raw data was labeled as "control," "LOS," or censored. Windows in the 24-h period preceding the time of positive blood culture were labeled LOS, those falling in the seven days following LOS were censored, and all other windows falling between 72 h after birth and NICU discharge were labeled as control. Windows with more than 50% data missing were excluded. All 10-min windows labeled as LOS and a sub-sample of those labeled as control (one 10-min window per hour) were used for training. We trained models using data from infants at NICU 1 and tested the models on NICU 2 and 3. Modeling was performed in R (R Foundation for Statistical Computing, Vienna, Austria) and Python (Python Software Foundation, https://www.python.org/).

Each model generates the estimated probability of LOS, updated every 10-min. To translate model outputs into the fold-increased risk of LOS in the next 24 h, we divided the estimated probability by the average probability of LOS. We evaluated different smoothing windows that averaged the 10-min model outputs over different time periods, ranging from the preceding 1–12 h. We determined an optimum smoothing window by balancing the rise in area under the receiver operating characteristic (AUC) against the decrease in the average risk score for LOS. After smoothing, model outputs were sampled hourly. Finally, hourly, smoothed model predictions were recalibrated using the sigmoid function.

To determine importance, features were permuted to calculate the loss in AUC and ranked from highest to lowest. We added ranks across models to estimate the overall feature importance.

Modeling methods

Four modeling strategies were evaluated, including logistic regression, a neural network, an extreme gradient boosting classifier or XGBoost, and random forest (Fig. 1).

For model #1, we built an 18-dimensional logistic regression model with restricted cubic spline transformations using three knots of nonlinearity on each of the nine features.[24,25] Modeling was performed using the rms package and knots were selected by default based on quantiles of the data.[26] We adjusted for repeated measures using the Huber-White method.[27] We removed minimum cross-correlation, SD SpO_2, and non-linear effects with p-values >0.05. The result was an 11-dimensional model that included seven features and four nonlinear terms.

For model #2, we developed a neural network model with four hidden layers. These layers had 512, 256, 128, and 64 hidden neurons, respectively. Dropout layers at a rate of 0.3 were added as a regularization measure between each hidden layer.[28] Leaky ReLU was used as the activation function in the hidden layers and a sigmoid activation function was used in the last layer.[29] The model was trained using binary cross-entropy as the loss function and Adam as the optimizer with a learning rate of 0.001. An early stopping callback was used to avoid overfitting which monitored the loss with a patience of 10.

Model #3 was an Extreme Gradient Boosting (XGBoost) classifier.[30] We trained multiple models on a parameter grid to identify optimal hyperparameters for learning rate, alpha (l1 regularization), lambda (l2 regularization), and max depth based on the highest cross-validatedAUC on NICU 1. The model used binary logistic as its objective function.

Model #4 was a random forest model with 800 classification trees and the square root of the number of features were sampled as candidates at each split.[31] The output of the model was the fraction of trees that classified an outcome as an event.

Model validation

We validated models on unseen data from two independent sites. We trained models on NICU 1, the largest and most complete data set, and externally validated on data from the other sites. All subsequent analyses of model validation and performance used the models trained on data from NICU 1. We assessed model discrimination by AUC with confidence intervals based on 200 bootstrap runs.

We evaluated model calibration by plotting the predicted vs. observed relative risk of LOS. We assessed model performance in the critical period near the time of LOS diagnosis by examining the time course of the average relative risk. We identified a rise in risk by performing a sign-rank test with the null hypothesis that hourly risk estimates are equal to risk estimates from the same patient 24 h prior with a statistical significance threshold of $p < 0.05$.

Sensitivity analyses

We assessed the dynamic change in the model output near the time of other "sepsis-like" events, including negative blood cultures diagnosed as

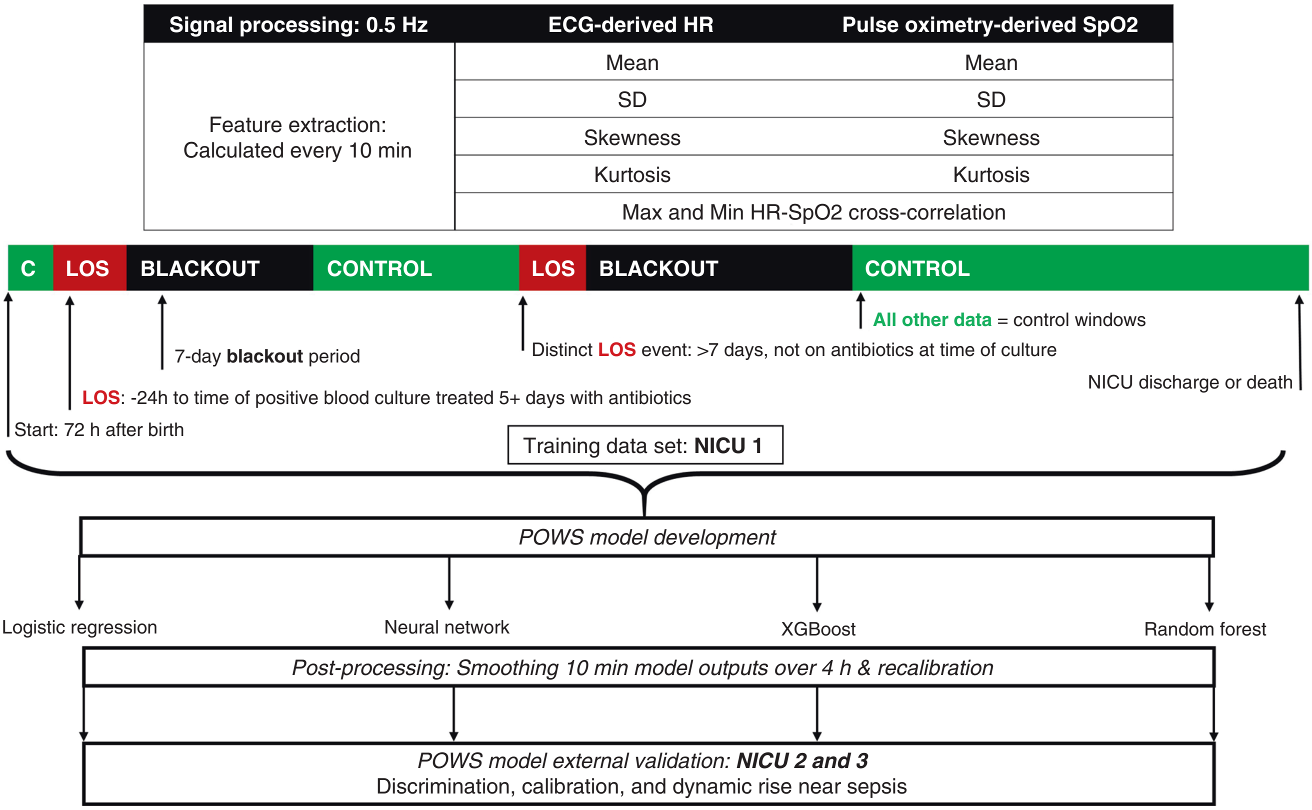

Fig. 1 Schematic overview of methods. From top to bottom, we processed the raw signals, sampled at 0.5 Hz, by calculating HR and SpO_2 features every 10 min. Each 10-min window, from 72 h after birth until NICU discharge or death, was labeled as late-onset sepsis (LOS), control, or removed as a blackout period window. Data from NICU 1 were used to train four machine learning models. Before external validation on data at NICU 2 & 3, post-processing steps included smoothing the 10-min model outputs over 4 h and recalibrating. Metrics used for external validation included discrimination by AUC, calibration, and plotting the average risk over the 48 h preceding sepsis to look for a dynamic rise from baseline near the time of diagnosis by blood culture.

clinical sepsis or necrotizing enterocolitis (NEC) without bacteremia. Because mechanical ventilation can alter HR and SpO_2 patterns,[8,32,33] we examined differences in models' predicted probability of sepsis based on ventilator status. Given the potential confounding of coagulase-negative staphylococcus (CONS),[34,35] we conducted a subgroup analysis to assess model performance in CONS versus non-CONS positive blood cultures.

While ECG and pulse oximetry monitoring are standard in the ICU setting, resource-poor settings might benefit from an algorithm that operates from an oximeter alone. Therefore, we tested model performance using features derived from pulse rate rather than the features derived from ECG HR.

Model comparisons

We hypothesized that HR, SpO_2, and static demographic variables are independent predictors of LOS. To quantify the added value, we built three additional models. First, we trained a demographics model that included birth weight, sex, and chronological age as baseline risk factors.[9,23,36,37] Second, we created a HR model that contained SD, skewness, and kurtosis. Third, we developed an SpO_2 model that contained mean, SD, skewness, and kurtosis. For comparison to a validated measure, we analyzed HRC index data at NICU 1 where HRC monitoring is used for all infants.[1,9]

At a range of thresholds, we calculated the number of alerts per patient day, where a hypothetical alarm is triggered and remains on until the infant has had 24 h with no threshold crossings. An alarm on at any point in the 72 h preceding sepsis was considered a true positive. We excluded alerts in the seven days following a sepsis event.

RESULTS

Participants

We studied 3151 VLBW infants at the three sites. Vital sign data were available for 2494/3151 (79%) infants and 302 of 390 episodes of LOS (77%). LOS events were excluded when vital sign data were missing from the event window. At NICU 1 and 2, the data were missing at random, while at NICU 3 the data retrieval process favored the smaller, sicker infants (less likely to change rooms, interrupting the flow of data). Infants were excluded when they could not be mapped to a vital sign file, which happened most often in the earlier years of the study at NICU 3 due to the data retrieval process. Table 1 shows the characteristics of the study population. The median age of LOS was 16 days (IQR: 8–32). As expected, infants with LOS had lower gestational age and birth weight.

Model development

We trained models on 923 infants from NICU 1. The nine candidate predictors and their empirical relationship with the outcome of sepsis are shown in S1 Fig. The retained features used in the logistic regression model were mean SpO_2, SD HR, skewness of HR, skewness of SpO_2, kurtosis of HR, kurtosis of SpO_2, and maximum positive value of the HR-SpO_2 cross-correlation. Table S1 presents model coefficients. To ensure we had arrived at an optimal local model, we performed a sensitivity analysis using recursive feature elimination using cross-validation AUC as the measure of model performance (Table S2).

After models were trained, we engaged in three post-processing steps. First, we evaluated different smoothing windows that averaged the 10-min model outputs over different time periods. The optimal smoothing window was 4 h (S2 Fig). After smoothing all model outputs over 4 h, we sampled the model outputs hourly (rather than every 10-min) and recalibrated the model outputs.

SPRINGER NATURE

Table 1. Comparison of clinical characteristics and outcomes among patients at all sites.

Characteristic[a]	NICU 1	NICU 2	NICU 3	Overall
Infants analyzed	923	886	685	2494
Birthweight (grams)	1014 ± 296	1039 ± 306	969 ± 286	1011 ± 298
Gestational Age (weeks)	27.8 ± 3.0	28.5 ± 2.9	27 ± 2.5	27.8 ± 2.9
Sex				
Male	475 (51%)	425 (48%)	348 (51%)	1248 (50%)
Female	448 (49%)	461 (52%)	337 (49%)	1246 (50%)
Race				
Black	219 (24%)	401(45%)	275 (40%)	895 (36%)
White	644 (70%)	412(46%)	375 (55%)	1481 (57%)
Other	60 (7%)	92 (10%)	22 (3%)	174 (7%)
Inborn	679 (73%)	751 (85%)	467 (68%)	1897 (76%)
Cesarean Delivery	657 (71%)	707(75%)	498 (73%)	1862 (75%)
Infants with LOS event(s) analyzed	146 (16%)	76 (9%)	56 (8%)	278 (11%)
LOS events	162	82	58	302
Age at sepsis (days)	18 (8–37)	19 (9–39)	8 (5–15)	16 (8–32)
Died	41 (4%)	49 (5%)	83 (12%)	173 (7%)

LOS late onset sepsis, *SD* standard deviation.
[a]Data are given as number ± SD for each group, unless otherwise specified.

Table 2. Performance at NICU 1, 2, and 3 for predicting sepsis within 24 h for various machine learning models.

	AUC		
Model	NICU 1 (train)	NICU 2 (test)	NICU 3 (test)
Logistic Regression	0.820 (0.813–0.827)	0. 791(0.780–0.808)	0.793 (0.782–0.804)
Random Forest	0.825 (0.818–0.830)	0.792 (0.780–0.801)	0.802 (0.789–0.814)
Neural Network	0.820 (0.814–0.826)	0.783 (0.773 - 0.792)	0.792 (0.781–0.803)
XG Boost	0.834 (0.828–0.840)	0.792 (0.780–0.802)	0.807 (0.795–0.818)

Data are given as AUC (95% CI).
AUC area under the receiver operating characteristic curve, *CI* confidence interval.

Model performance

All modeling methods discriminated sepsis windows from control windows with good performance on the training date (AUCs >0.82, Table 2), with only small performance diminishment at the two external validation sites (AUCs >0.78, Table 2). Additionally, model performance was similar whether using ECG-derived HR or pulse rates from oximetry (AUC loss −0.009, −0.005, −0.002 at NICU 1, 2, and 3, respectively; Table 3). There was high and consistent calibration (Fig. 2). At the lowest risk of sepsis, when the risk is less than the population average risk, models slightly under-predict risk at NICU 2 and over-predict at NICU 3.

Figure 3 plots the average fold-increase in each risk model as a function of time to LOS at each NICU. A fold-increase of 1 indicates no greater risk for sepsis than the baseline (0.26%); a fold-increase of 2 would indicate twice the average daily risk (0.52%). The relative risk predicted by the logistic regression model increased by 92% in the 24 h before sepsis (2.5-fold to 4.8-fold), and the predicted risk calculated in the XGBoost model increased by 150%, from 3.2-fold to 8.0-fold. Predicted risk values deviated significantly from baseline from 23 to 24 h before the blood culture (Fig. 3).

Feature importance

Figure 4 shows feature importance in the logistic regression (A), neural net (B), XGBoost (C), and random forest (D) sepsis prediction models. Features are ordered by decrease in AUC introduced by permuting each feature. Of note, seven features were included in the logistic regression model, while nine features were used in the remaining models. The features of greatest importance, identified by adding the ranks of the features across models, were skewness of HR, SD of HR, kurtosis of SpO_2, and maximum cross-correlation. The random forest model identified kurtosis of SpO_2 as the feature of greatest importance, while the remaining models ranked it as less important than skewness of HR and SD of HR.

Table 3. Performance at NICU 1, 2, and 3 using features derived from pulse oximetry for Predicting Sepsis within 24 h.

	AUC		
Model	NICU 1 (train)	NICU 2 (test)	NICU 3 (test)
POWS	0.811 (0.804–0.817)	0.786 (0.774–0.798)	0.781 (0.766–0.791)

Data are given as AUC (95% CI).
AUC area under the receiver operating characteristic curve, *CI* confidence interval.

Do HR-SpO2 models add information to HR and demographics?

Having established that there is a cardiorespiratory signature of neonatal sepsis, we evaluated the potential clinical utility of the

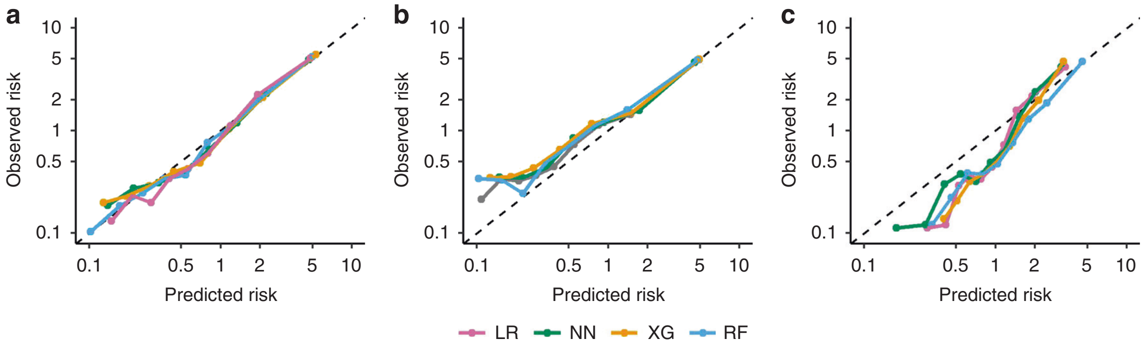

Fig. 2 Calibration plots. Calibration of each POWS model for (**a**) NICU 1, (**b**) NICU 2, and (**c**) NICU 3. Predicted risk relative to average is on the abscissa and observed risk relative to average is on the ordinate. Each point represents one decile of predicted risk. The line of identity is shown as a dashed line. LR logistic regression, NN neural network, XG XGBoost, RF random forest.

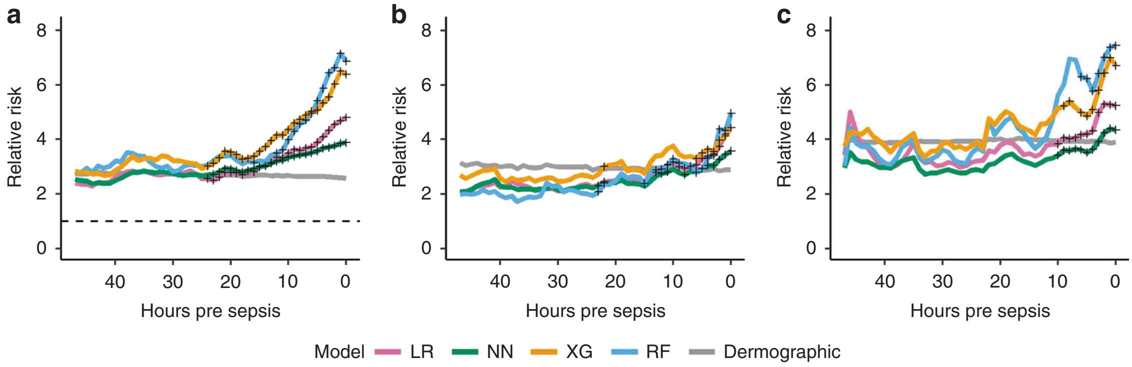

Fig. 3 The average risk of sepsis. The average relative risk of sepsis as predicted by each model as a function of the time to event in hours. Panels show the results of each model at (**a**) NICU 1, (**b**) NICU 2, and (**c**) NICU 3. Results are shown for the four POWS models and the demographic-only model (in gray). Black crosses indicate times where the model outputs are significantly higher ($p < 0.05$) than outputs from the same patient 24 h prior. LR logistic regression, NN neural network, XG XGBoost, RF random forest.

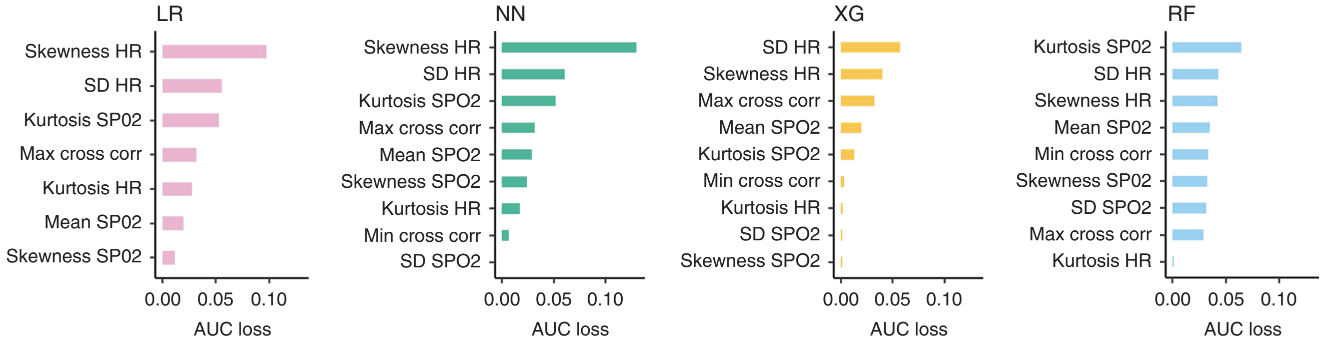

Fig. 4 Variable importance plots. Variable importance plots for components of the logistic regression (LR), neural net (NN), XG Boost (XG), and random forest (RF) models. Features are ordered by decreasing AUC loss introduced by permuting the values of each feature.

statistical models. We chose to use the logistic regression model for the remaining analyses. We chose to call this model POWS, for Pulse Oximetry Warning System, because we aimed to determine the added value of SpO_2 from pulse oximetry and validate pulse-oximetry-derived pulse rate as an equivalent data source to ECG-derived heart rate.

We compared POWS to models with features derived from only HR data, only SpO_2 data, from only the demographics variables of chronological age, sex, and birth weight, or from a combination (i.e., HR data plus demographics, POWS plus demographics). The parameters for each of these models are shown in supplementary tables (Tables S3–S5). We compared model discrimination for LOS using AUCs with bootstrapped 95% confidence intervals (Table 4). Using this criteria, we conclude that a HR-SpO_2 model adds to a HR only model. The POWS improved over the models using only HR-derived (AUC, 0.82 vs 0.80 NICU 1; 0.75 vs 0.79 NICU 2) or only SpO_2-derived features (AUC, 0.82 vs 0.71, NICU 1; 0.79 vs 0.79 NICU 2) or only demographics (AUC 0.82 vs 0.78 NICU 1; 0.80 vs 0.79 NICU 1). This does not hold at NICU 3. Adding demographics improved the POWS model discrimination. However, the demographics model provided only near-static information (Fig. 3).

To further understand how combining HR and SpO_2 data features in prediction models adds to HR characteristics alone, we compared POWS to the HRC index, a logistic regression model that uses only features derived from HR data.[9] Although POWS and HRC both utilize HR-derived features, POWS uses every

SPRINGER NATURE

Table 4. Performance at NICU 1, 2, and 3 for predicting sepsis within 24 h for logistic regression models with varying features.

	AUC		
**Model**	**NICU 1 (train)**	**NICU 2 (test)**	**NICU 3 (test)**
Demographics	0.781 (0.774–0.788)	0.800 (0.790–0.808)	0.836 (0.827–0.844)
HR only	0.797 (0.790–0.804)	0.745 (0.734–0.756)	0.788 (0.775–0.799)
SpO_2 only	0.706 (0.702–0.718)	0.703 (0.692–0.715)	0.683 (0.670–0.698)
POWS (HR + SpO_2)	0.820 (0.813–0.827)	0. 791(0.780–0.808)	0.793 (0.782–0.804)
HR + Demographics	0.819 (0.818–0.831)	0.821 (0.811–0.831)	0.852 (0.842–0.861)
SpO_2 + Demographics	0.786 (0.784–0.799)	0.808 (0.797–0.816)	0.839 (0.828–0.851)
POWS (HR + SpO_2) + Demographics	0.831 (0.824–0.837)	0.830 (0.820–0.839)	0.853 (0.845–0.861)

Data are given as AUC (95% CI).
AUC area under the receiver operating characteristic curve, *CI* confidence interval.

two-second HR data to calculate HR SD, skewness, and kurtosis, while the HRC index uses inter-beat-intervals to calculate HR SD, sample asymmetry, and sample entropy. POWS performed better than the HRC index in the NICU 1 cohort (POWS AUC 0.820, CI 0.813–0.827; HRC index AUC 0.795, CI 0.798–0.803). Data were not available to compare POWS and HRC index performance at NICU 2 and 3.

Sensitivity and alarm rates
We devised a hypothetical alert strategy for the physiologic-based models. An alarm is triggered based on a range of thresholds and the alarm remains on until 24 consecutive hours with no threshold crossings have elapsed. For the demographic model, we defined alerts as daily threshold crossings. Examining the sensitivity of the models and the number of alarms in a 50-bed unit demonstrates that POWS has a perceptibly higher sensitivity than the models using HR or SpO_2 features alone (Fig. 5).

The low sensitivity of the demographics model again speaks to the limitations of static variables for continuous risk prediction. Adding demographic features to POWS decreases the sensitivity across the range of alert rates and therefore limits its utility as an early warning score.

We examined lift charts as a way to visually compare the ability of each model to detect LOS events (Fig S4). Lift is the ratio of the percent of events captured at a given percentile of data to the random expectation of the percent of events captured. POWS had a higher lift than HR-only models, and both had a greater lift than the SpO_2-only or demographics-only models. We performed this analysis to demonstrate the added value of combined cardiorespiratory modeling to detect sepsis within the highest risk segments of data.

Model performance in other clinical contexts
We trained our models on blood-culture positive sepsis, but assessed model performance around the time of clinical deterioration with other diagnoses and stratified by factors that denote illness severity. First, we examined the dynamic risk estimates of POWS near the time of blood cultures for clinical sepsis (negative blood culture treated with antibiotics for at least five days due to clinical illness) and the time of blood cultures for confirmed NEC without bacteremia. Figure S3 shows a steep rise in risk in the hours preceding cultures for clinical sepsis and NEC, with a slower decrease in risk in the 48 h following cultures.

Figure S5 shows model performance near LOS events, stratified by ventilator status at the time of sepsis and by causative organism, grouped as CONS vs. non-CONS. On average, LOS episodes on a ventilator and those caused by non-CONS bacteremia had higher risk scores before, during, and after diagnosis of LOS.

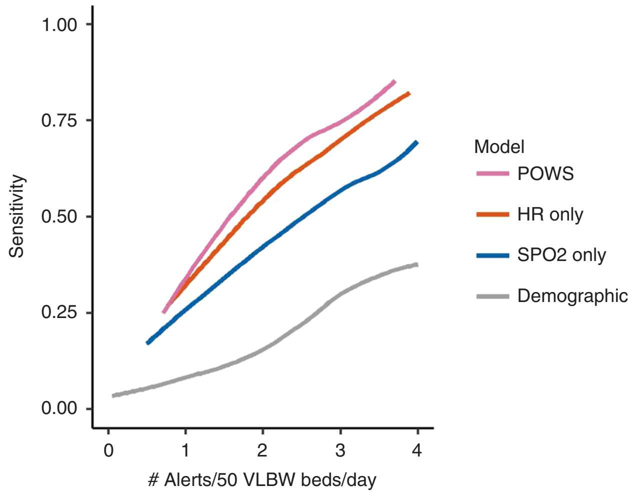

Fig. 5 Evaluating the model sensitivity across a range of thresholds. Using an alert strategy for physiologic- based models where an alarm is initially triggered based on a threshold crossing and the alarm remains on until 24 consecutive hours where there have been no threshold crossings, we selected a range of thresholds and then calculated the number of alerts per day and required an alert display in the 3 days preceding the clinical diagnosis of sepsis. We defined alerts as daily threshold crossings for the demographic model. The y-axis displays the percent of sepsis events detected when allowing for different numbers of alerts per day.

DISCUSSION
Cardiorespiratory signatures provide important information for early warning of LOS in VLBW infants. Prior work identified abnormal heart rate characteristics and increased HR-SpO_2 cross-correlation as physiomarkers of illness due to sepsis.[8,10,38] Here, we report that more information exists in the data. We tested the hypothesis that HR and SpO_2 data contain patterns that contribute independent, additive information for LOS detection. In doing so, we developed and validated cardiorespiratory models that predict an increased risk of sepsis before clinical diagnosis, up to 24 h before the time of blood culture. External validation at two NICUs confirmed that these signatures may be generalized for sepsis detection at centers with variable patient populations, monitoring equipment, and practice patterns. Additionally, we found that static demographic variables help to risk-stratify infants at baseline but dampen the dynamic risk prediction.

A cardiorespiratory signature of neonatal sepsis
Previously, we showed that a signature of sepsis exists in the HR signal.[14,39] Decreased HR variability with transient HR decelerations was recognized as similar to patterns of fetal distress that

also served as a physiologic biomarker of sepsis in premature infants.[40] A decade of prior work translated abnormal HR patterns into mathematical algorithms,[12,13,41] produced a validated predictive model for LOS in VLBW infants,[41] known as the HRC index or HeRO score, and demonstrated reduced mortality in a multicenter randomized trial.[1] The past work exemplified a pathway for the translation of ideas about signatures of illness in continuous cardiorespiratory monitoring data into bedside tools for clinicians and patients.[42]

More recently, our group studied control of ventilation in premature infants and recognized that the HR and SpO_2 signals correlate when episodes of apnea or periodic breathing lead to decreases in both HR (bradycardia) and SpO_2 (desaturation).[8,43] Addition of the HR-SpO_2 cross-correlation coefficient improved the performance of statistical models to detect sepsis over HRC monitoring alone in a two-NICU cohort. We found HR-SpO_2 cross-correlation to be the best individual feature to discriminate LOS vs. sepsis-ruled out events.[10] We also compared vital signs across our three collaborating sites and found clinically trivial though statistically significant differences in HR and SpO_2.[44]

Respiratory deterioration prompts a majority of the sepsis evaluations in VLBW infants[10] and autonomic dysfunction due to sepsis impairs control of breathing as well as heart rate. Features in the POWS model quantify changes in the HR and SpO_2 signals and their interaction, thereby capturing reduced variability, decelerations, desaturations, and other more subtle components of a signature of cardiorespiratory deterioration due to sepsis.

These findings support the idea that analysis of respiratory data, in addition to heart rate data, can enhance early detection of subacute but potentially catastrophic illnesses such as sepsis.

Machine learning methods yield similar results

Interestingly, the choice of machine learning methods did not significantly impact performance. Logistic regression performed comparably to more complex methods. Unlike our prior work,[8,10] here we used cubic splines to account for non-linear relationships of predictors. We hypothesize that this resulted in similar performance of logistic regression compared with other machine learning methods that allow for non-linear relationships. Given equal performance but better explainability, we used the logistic regression approach for the remainder of the analysis. Our finding is matched by others in the literature; previous analyses of multiple machine learning methods for detecting LOS have also found similar performance across modeling techniques.[45,46]

Comparison with other work

Other groups have used machine learning methods and high-resolution cardiorespiratory data to develop models predicting LOS. Researchers have developed novel algorithms to detect reduced infant motion[18,47] and demonstrated improved prediction when combined with features measuring heart rate, SpO_2, and respiratory rate.[19] We also find that combining physiological features improves LOS prediction.

Studies have repeatedly shown clinical variables and laboratory values to add to vital sign data for sepsis prediction.[10,45,48–50] We found that demographic variables improve model AUCs, but dampen the dynamic performance of a continuous sepsis risk model. Physiologic data is continuous, objective, and contains the earliest signs of the inflammatory response to infection via the autonomic nervous system.[51] An algorithm that runs on continuously patient-generated data allows for automated detection of the infant who is getting sick, rather than relying on clinicians with mixed skill levels to recognize early, subtle changes in a busy unit.[52]

Limitations

Some infants and events were excluded due to missing data. Prospective analysis with comprehensive data capture is needed to test models and risk trajectories over time and episodes in a multicenter cohort of premature infants.

Definitions of neonatal sepsis in premature infants vary among published studies.[53–55] We chose to model events of culture-proven sepsis, knowing that this definition, while unequivocal, also excludes clinically important events associated with a negative blood culture. The heterogeneity of non-culture proven events likely impacts model development[48] and, therefore, performance on new data. Nevertheless, our models trained exclusively on culture-positive events displayed a rise in predicted risk near the time of negative blood cultures diagnosed as clinical sepsis or NEC, indicating utility for detecting deterioration associated with sepsis-like events.

Other co-morbidities of prematurityaffect cardiorespiratory function and may alter the features incorporated in POWS. We analyzed the impact of mechanical ventilation in this study because of prior work and the ability to accurately determine the timing of this variable in retrospective data. The influence of co-morbidities with less discrete timing of onset warrants further study in future prospective analysis in multicenter cohorts.

Clinical implications and future directions

The goal of this work is to optimize a physiology-based sepsis early warning system to improve outcomes by bringing the clinician to the bedside at the right time, even when resources are limited to pulse oximetry. The ability to use a single device, the pulse oximeter, to run the algorithm may simplify the technical implementation of POWS monitoring. All too often, LOS diagnosis occurs after the infection progresses to an advanced phase of systemic inflammation, organ dysfunction, and shock. However, antibiotic overuse is an urgent concern in most NICUs. Using predictive monitoring to balance early sepsis detection and antibiotic overuse will require careful attention to the implementation and adoption of the technology. Model-generated alerts and clinical decision guidelines may help standardize the response to changes in sepsis risk and allow quantitative analysis of its impact.

Cardiorespiratory predictive monitoring can detect a subclinical prodrome in HR and SpO_2 data with superior discrimination and sensitivity compared to analytics from either signal alone or demographic risk factors. External validation of POWS in two geographically distinct cohorts indicates that the signatures of sepsis may be general and are not greatly impacted by center-specific practice patterns or equipment. The ultimate test for validating our models and findings will be a multicenter clinical trial to measure the impact on clinical care and outcomes.

CONCLUSION

A cardiorespiratory early warning score, analyzing heart rate from electrocardiogram or pulse oximetry together with SpO_2, predicts late-onset sepsis diagnosis within 24 h across multiple NICUs and detects sepsis better than heart rate characteristics or demographics alone.

DATA AVAILABILITY

The datasets generated during and/or analyzed during the current study are available from the corresponding author on reasonable request.

REFERENCES

1. Moorman, J. R. et al. Mortality reduction by heart rate characteristic monitoring in very low birth weight neonates: a randomized trial. *J. Pediatr.* **159**, 900–906 (2011). e1.
2. King, W. E., Carlo, W. A., O'Shea, T. M. & Schelonka, R. L., HRC neurodevelopmental follow-up investigators. Heart rate characteristics monitoring and reduction in mortality or neurodevelopmental impairment in extremely low birthweight infants with sepsis. *Early Hum. Dev.* **159**, 105419 (2021).

3. Greenberg, R. G. et al. Prolonged duration of early antibiotic therapy in extremely premature infants. *Pediatr. Res* **85**, 994–1000 (2019).
4. Lu, J. & Claud, E. C. Connection between gut microbiome and brain development in preterm infants. *Dev. Psychobiol.* **61**, 739–751 (2019).
5. Ting, et al. Duration of initial empirical antibiotic therapy and outcomes in very low birth weight infants. *Pediatrics*. **143**, e20182286 (2019).
6. Dardas, M. et al. The impact of postnatal antibiotics on the preterm intestinal microbiome. *Pediatr. Res* **76**, 150–158 (2014).
7. Sullivan B. A., Kausch S. L., Fairchild K. D. Artificial and human intelligence for early identification of neonatal sepsis. *Pediatr Res*. 2022. https://doi.org/10.1038/s41390-022-02274-7 Epub ahead of print.
8. Fairchild, K. D. et al. Vital signs and their cross-correlation in sepsis and NEC: a study of 1,065 very-low-birth-weight infants in two NICUs. *Pediatr. Res* **81**, 315–321 (2017).
9. Griffin, M. P. et al. Abnormal heart rate characteristics preceding neonatal sepsis and sepsis-like illness. *Pediatr. Res* **53**, 920–926 (2003).
10. Sullivan, B. A. et al. Clinical and vital sign changes associated with late-onset sepsis in very low birth weight infants at 3 NICUs. *J. Neonatal Perinat. Med* **14**, 553–561 (2021).
11. Sullivan, B. A. et al. Early pulse oximetry data improves prediction of death and adverse outcomes in a two-center cohort of very low birth weight infants. *Am. J. Perinatol.* **35**, 1331–1338 (2018).
12. Kovatchev, B. P. et al. Sample asymmetry analysis of heart rate characteristics with application to neonatal sepsis and systemic inflammatory response syndrome. *Pediatr. Res* **54**, 892–898 (2003).
13. Lake, D. E., Richman, J. S., Griffin, M. P. & Moorman, J. R. Sample entropy analysis of neonatal heart rate variability. *Am. J. Physiol. Regul. Integr. Comp. Physiol.* **283**, R789–R797 (2002).
14. Griffin, M. P. et al. Heart rate characteristics: novel physiomarkers to predict neonatal infection and death. *Pediatrics* **116**, 1070–1074 (2005).
15. Fairchild, K. D. et al. Septicemia mortality reduction in neonates in a heart rate characteristics monitoring trial. *Pediatr. Res.* **74**, 570–575 (2013).
16. Fairchild, K. et al. Clinical associations of immature breathing in preterm infants: part 1-central apnea. *Pediatr. Res* **80**, 21–27 (2016).
17. Herlenius, E. An inflammatory pathway to apnea and autonomic dysregulation. *Respir. Physiol. Neurobiol.* **178**, 449–457 (2011).
18. Joshi, R. et al. Predicting neonatal sepsis using features of heart rate variability, respiratory characteristics, and ECG-derived estimates of infant motion. *IEEE J. Biomed. Health Inf.* **24**, 681–692 (2020).
19. Cabrera-Quiros, L. et al. Prediction of late-onset sepsis in preterm infants using monitoring signals and machine learning. *Crit. Care Explor* **3**, e0302 (2021).
20. Peng Z., et al. A continuous late-onset sepsis prediction algorithm for preterm infants using multi-channel physiological signals from a patient monitor. *IEEE J Biomed Health Inform*. 2022. https://doi.org/10.1109/JBHI.2022.3216055 Epub ahead of print.
21. Collins, G. S., Reitsma, J. B., Altman, D. G. & Moons, K. G. M. Transparent reporting of a multivariable prediction model for individual prognosis or diagnosis (TRIPOD): the TRIPOD statement. *Ann. Intern Med* **162**, 55–63 (2015).
22. Moons, K. G. M. et al. Transparent reporting of a multivariable prediction model for Individual Prognosis or Diagnosis (TRIPOD): explanation and elaboration. *Ann. Intern. Med*. **162**, W1–W73 (2015).
23. Lake, D. E., Fairchild, K. D., Kattwinkel, J. & Moorman, J. R. Reply to: Heart rate predicts sepsis. *J. Pediatr.* **161**, 770–771 (2012).
24. CRAN - Package rms [Internet]. [cited 2022 Mar 15]. Available from: https://cran.r-project.org/web/packages/rms/index.html
25. Harrell F. E. rms: Regression Modeling Strategies. 2015;4.3-0.
26. Harrell F. E. rms: Regression Modeling Strategies. R package version 5.1-0.1. [Internet]. R package version 5.1-0.1. 2018 [cited 2022 Oct 17]. Available from: https://CRAN.R-project.org/package=rms
27. Harrell, F. E. Regression Modeling Strategies: With Applications to Linear Models, Logistic and Ordinal Regression, and Survival Analysis. Cham: Springer International Publishing; 2015.
28. Srivastava N., Hinton G., Krizhevsky A. Dropout: a simple way to prevent neural networks from overfitting. *J. Mach.* 2014;15:1929–58.
29. LeCun, Y., Bengio, Y. & Hinton, G. Deep learning. *Nature* **521**, 436–444 (2015).
30. Chen T., Guestrin C. XGBoost: A Scalable Tree Boosting System. Proceedings of the 22nd ACM SIGKDD International Conference on Knowledge Discovery and Data Mining - KDD' ' '16. New York, New York, USA: ACM Press; 2016. p. 785–794.
31. Breiman L. Random Forests. Springer Science and Business Media LLC. 2001;
32. van Ravenswaaij-Arts, C. M., Hopman, J. C., Kollée, L. A., Stoelinga, G. B. & van Geijn, H. P. The influence of artificial ventilation on heart rate variability in very preterm infants. *Pediatr. Res* **37**, 124–130 (1995).
33. Di Fiore, J. M., Poets, C. F., Gauda, E., Martin, R. J. & MacFarlane, P. Cardiorespiratory events in preterm infants: etiology and monitoring technologies. *J. Perinatol.* **36**, 165–171 (2016).
34. Jean-Baptiste, N. et al. Coagulase-negative staphylococcal infections in the neonatal intensive care unit. *Infect. Control Hosp. Epidemiol.* **32**, 679–686 (2011).
35. Cantey, J. B., Anderson, K. R., Kalagiri, R. R. & Mallett, L. H. Morbidity and mortality of coagulase-negative staphylococcal sepsis in very-low-birth-weight infants. *World J. Pediatr.* **14**, 269–273 (2018).
36. Downey, L. C., Smith, P. B. & Benjamin, D. K. Risk factors and prevention of late-onset sepsis in premature infants. *Early Hum. Dev.* **86**, 7–12 (2010). Suppl 1.
37. Dong, Y. & Speer, C. P. Late-onset neonatal sepsis: recent developments. *Arch. Dis. Child Fetal Neonatal Ed.* **100**, F257–F263 (2015).
38. Sullivan, B. A. & Fairchild, K. D. Predictive monitoring for sepsis and necrotizing enterocolitis to prevent shock. *Semin Fetal Neonatal Med* **20**, 255–261 (2015).
39. Lake, D. E., Fairchild, K. D. & Moorman, J. R. Complex signals bioinformatics: evaluation of heart rate characteristics monitoring as a novel risk marker for neonatal sepsis. *J. Clin. Monit. Comput* **28**, 329–339 (2014).
40. Griffin, M. P. & Moorman, J. R. Toward the early diagnosis of neonatal sepsis and sepsis-like illness using novel heart rate analysis. *Pediatrics* **107**, 97–104 (2001).
41. Lake, D. E., Griffin, M. P. & Moorman, J. R. New mathematical thinking about fetal heart rate characteristics. *Pediatr. Res* **53**, 889–890 (2003).
42. Moorman, J. R. The principles of whole-hospital predictive analytics monitoring for clinical medicine originated in the neonatal ICU. *npj Digital Med.* **5**, 41 (2022).
43. Fairchild, K. D. & Lake, D. E. Cross-correlation of heart rate and oxygen saturation in very low birthweight infants: association with apnea and adverse events. *Am. J. Perinatol.* **35**, 463–469 (2018).
44. Zimmet A. M. et al. Vital sign metrics of VLBW infants in three NICUs: implications for predictive algorithms. *Pediatr Res*. **90**, 125–130 (2021).
45. Song, W. et al. A predictive model based on machine learning for the early detection of late-onset neonatal sepsis: development and observational study. *JMIR Med. Inf.* **8**, e15965 (2020).
46. Masino, A. J. et al. Machine learning models for early sepsis recognition in the neonatal intensive care unit using readily available electronic health record data. *PLoS One* **14**, e0212665 (2019).
47. Joshi, R. et al. A ballistographic approach for continuous and non-obtrusive monitoring of movement in neonates. *IEEE J. Transl. Eng. Health Med* **6**, 2700809 (2018).
48. Mani, S. et al. Medical decision support using machine learning for early detection of late-onset neonatal sepsis. *J. Am. Med Inf. Assoc.* **21**, 326–336 (2014).
49. Griffin, M. P., Lake, D. E. & Moorman, J. R. Heart rate characteristics and laboratory tests in neonatal sepsis. *Pediatrics* **115**, 937–941 (2005).
50. Griffin, M. P., Lake, D. E., O'Shea, T. M. & Moorman, J. R. Heart rate characteristics and clinical signs in neonatal sepsis. *Pediatr. Res.* **61**, 222–227 (2007).
51. Sullivan, B. A. & Fairchild, K. D. Vital signs as physiomarkers of neonatal sepsis. *Pediatr. Res.* **91**, 273–282 (2022).
52. Monfredi O. J. et al. Continuous ECG monitoring should be the heart of bedside AI-based predictive analytics monitoring for early detection of clinical deterioration. *J Electrocardiol.* **76**, 35–38 (2022).
53. Henry, C. J. et al. Neonatal sepsis: a systematic review of core outcomes from randomised clinical trials. *Pediatr. Res.* **91**, 735–742 (2022).
54. Hayes R. et al. Neonatal sepsis definitions from randomised clinical trials. *Pediatr Res*. 2021. https://doi.org/10.1038/s41390-021-01749-3 Epub ahead of print.
55. McGovern, M. et al. Challenges in developing a consensus definition of neonatal sepsis. *Pediatr. Res* **88**, 14–26 (2020).

AUTHOR CONTRIBUTIONS

S.L.K., B.S., K.F., D.L., R.S., Z.V., and J.R.M. have made substantial contributions to the conception or design of the work; S.L.K., J.Q., J.B., A.P., A.B., and J.I. made substantial contributions to the acquisition, analysis, or interpretation of data; SK and BS drafted the work and all other authors have substantively revised it. All authors have approved the submitted version. All authors have agreed both to be personally accountable for the author's own contributions and to ensure that questions related to the accuracy or integrity of any part of the work, even ones in which the author was not personally involved, are appropriately investigated, resolved, and the resolution documented in the literature.

FUNDING

We acknowledge the following grants for funding the work presented in this manuscript: K23 HD097254 [PI: B Sullivan]; R01 HD092071 [Co-PIs KD Fairchild & JR Moorman, Co-I DE Lake] K23NS111086 [PI: Z Vesoulis].

COMPETING INTERESTS

Some authors have financial conflicts of interest. J.R.M. and D.E.L. own stock in Medical Prediction Sciences Corporation. J.R.M. is a consultant for Nihon Kohden Digital Health

Solutions. Z.A.V. is a consultant for Medtronic. All other authors have no financial conflicts to disclose. No authors have any non-financial conflicts of interest to disclose.

CONSENT STATEMENT

This study was approved by the IRB at each site with waiver of consent.

ADDITIONAL INFORMATION

Supplementary information The online version contains supplementary material available at https://doi.org/10.1038/s41390-022-02444-7.

Correspondence and requests for materials should be addressed to Sherry L. Kausch.

Reprints and permission information is available at http://www.nature.com/reprints

Publisher's note Springer Nature remains neutral with regard to jurisdictional claims in published maps and institutional affiliations.

Springer Nature or its licensor (e.g. a society or other partner) holds exclusive rights to this article under a publishing agreement with the author(s) or other rightsholder(s); author self-archiving of the accepted manuscript version of this article is solely governed by the terms of such publishing agreement and applicable law.
